# The Golden Age of Thermally Activated Delayed Fluorescence Materials: Design and Exploitation

**DOI:** 10.1021/acs.chemrev.3c00755

**Published:** 2024-12-12

**Authors:** John Marques Dos Santos, David Hall, Biju Basumatary, Megan Bryden, Dongyang Chen, Praveen Choudhary, Thomas Comerford, Ettore Crovini, Andrew Danos, Joydip De, Stefan Diesing, Mahni Fatahi, Máire Griffin, Abhishek Kumar Gupta, Hassan Hafeez, Lea Hämmerling, Emily Hanover, Janine Haug, Tabea Heil, Durai Karthik, Shiv Kumar, Oliver Lee, Haoyang Li, Fabien Lucas, Campbell Frank Ross Mackenzie, Aminata Mariko, Tomas Matulaitis, Francis Millward, Yoann Olivier, Quan Qi, Ifor D. W. Samuel, Nidhi Sharma, Changfeng Si, Leander Spierling, Pagidi Sudhakar, Dianming Sun, Eglė Tankelevičiu̅tė, Michele Duarte Tonet, Jingxiang Wang, Tao Wang, Sen Wu, Yan Xu, Le Zhang, Eli Zysman-Colman

**Affiliations:** 1 Organic Semiconductor Centre, EaStCHEM School of Chemistry, 7486 University of St Andrews, St Andrews, Fife KY169ST, UK; 2 Organic Semiconductor Centre, SUPA School of Physics and Astronomy, University of St Andrews, St Andrews, Fife KY169SS, UK; 3 Institute of Organic Chemistry (IOC), 98929 Karlsruhe Institute of Technology (KIT), Fritz-Haber-Weg 6, 76131 Karlsruhe, Germany; 4 Laboratory for Computational Modeling of Functional Materials, Namur Institute of Structured Matter, Université de Namur, Rue de Bruxelles, 61, 5000 Namur, Belgium; 5 Department of Physics, 151527 Durham University, Durham DH1 3LE, UK; 6 Department of Chemistry, University of Delhi, Delhi 110007, India; 7 EaStCHEM School of Chemistry, 151018 The University of Edinburgh, Edinburgh, EH9 3FJ, UK

## Abstract

Since the seminal report by Adachi and co-workers in 2012, there has been a veritable explosion of interest in the design of thermally activated delayed fluorescence (TADF) compounds, particularly as emitters for organic light-emitting diodes (OLEDs). With rapid advancements and innovation in materials design, the efficiencies of TADF OLEDs for each of the primary color points as well as for white devices now rival those of state-of-the-art phosphorescent emitters. Beyond electroluminescent devices, TADF compounds have also found increasing utility and applications in numerous related fields, from photocatalysis, to sensing, to imaging and beyond. Following from our previous review in 2017 ( Adv. Mater. 2017, 1605444 ), we here comprehensively document subsequent advances made in TADF materials design and their uses from 2017–2022. Correlations highlighted between structure and properties as well as detailed comparisons and analyses should assist future TADF materials development. The necessarily broadened breadth and scope of this review attests to the bustling activity in this field. We note that the rapidly expanding and accelerating research activity in TADF material development is indicative of a field that has reached adolescence, with an exciting maturity still yet to come.

## Graphical abstract

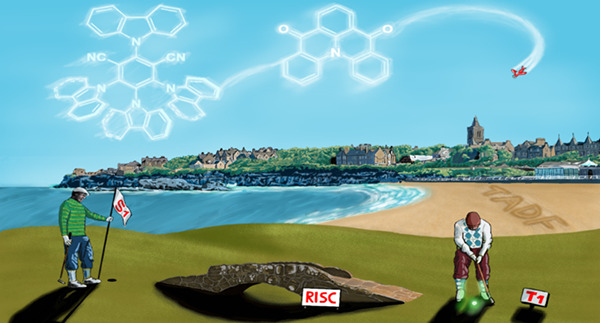

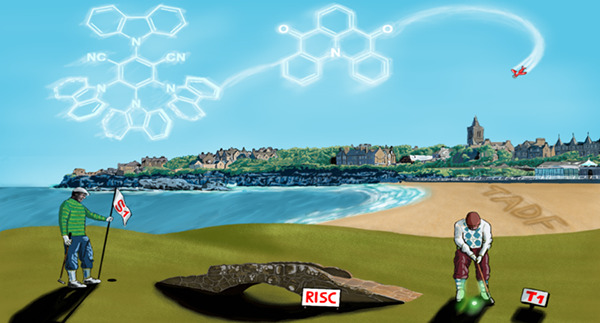

## 1 Introduction

Being able to control the evolution, energy and spin of excitons in advanced materials underpins technologies ranging from organic light-emitting diodes (OLEDs), to solar cells, to optical sensing and imaging, to photocatalysis and wider technological applications. Many of these applications rely on efficient radiative decay of the generated exciton, that is, the generation of light. Light is not only generated as a result of photoexcitation (photoluminescence) but can be produced following electrical excitation (electroluminescence), chemical reaction (chemiluminescence), biochemical reaction (bioluminescence), application of mechanical force (mechanoluminescence), changes in crystallographic structure (crystalloluminescence), external sound (sonoluminescence), or high-energy ionized particle bombardment (cathodoluminescence, radioluminescence). In particular, the use of OLEDs (applied electroluminescent devices) has exploded over the last decade due to their superior performance in displays and promise for solid-state lighting (SSL) over preceding technologies such as liquid crystalline displays (LCDs), plasma display panels (PDPs), and inorganic light-emitting diodes (LEDs). Unlike now-ubiquitous LCDs, OLED display pixels are self-illuminating and individually addressable, and so do not require a uniform backlight pane. This allows pure black to be produced, resulting in a simpler and more energy-efficient display architecture with deeper achievable visual contrast. Unlike LCD or inorganic LED displays, OLED displays can also be fabricated on a wide range of substrates, offering ultrathin, foldable, flexible and even transparent displays supporting innovative technological applications. Primarily because of their superior picture quality and color gamut (supported by the endless tunability of photophysical properties of the organic materials) OLED displays are now used in the majority of high-end smartphone^1^ and smartwatch^2^ screens, and are being increasingly adopted in the large-area television,^3^ monitor, and automotive markets.^4^

**Figure 1:**
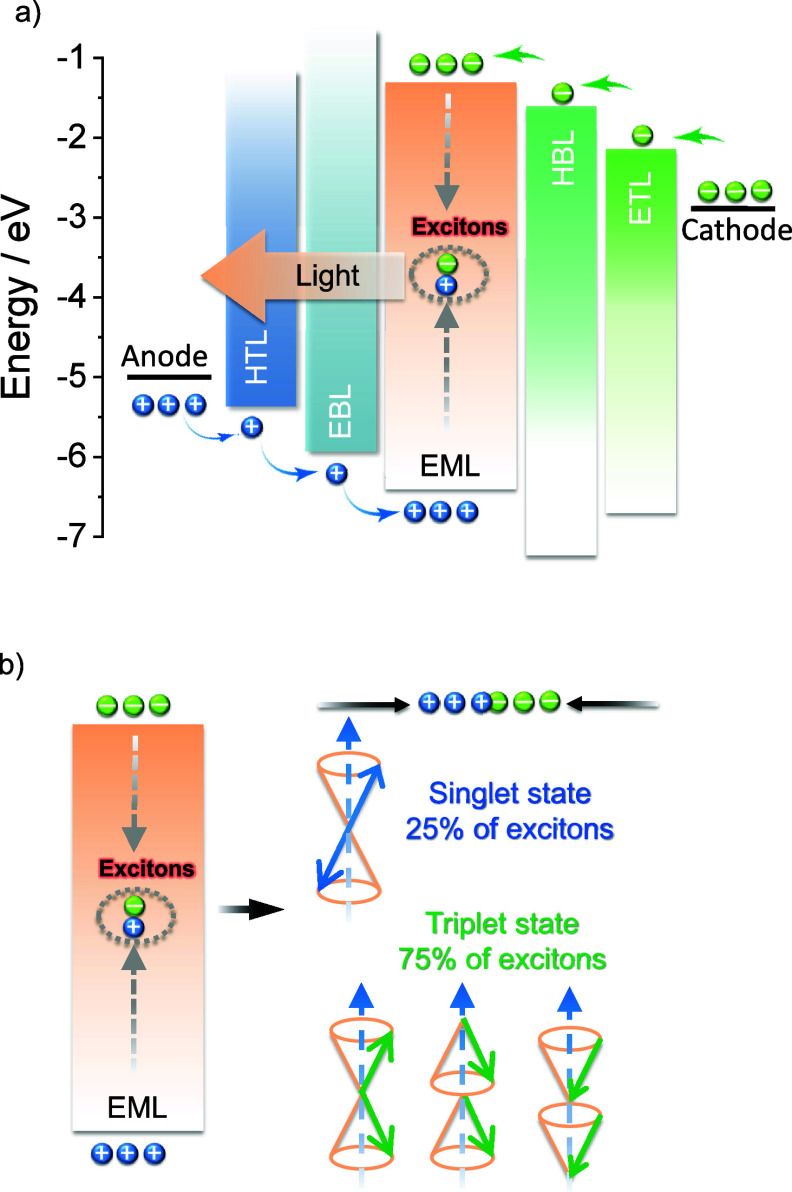
(a) Structure and operational mechanism of an OLED. (b) Fermionic spin statistics of exciton generation in the OLED, showing that they are formed in a 3:1 ratio of triplets to singlets.

OLEDs consist of a multilayer stack of organic semiconductor materials that are sandwiched between the cathode and anode. These devices produce light upon the application of a voltage, which leads to the injection of charges (holes from the anode and electrons from the cathode) that migrate through the layers of the device, ultimately recombining within the emissive layer (EML) to form excitons (bound electron-hole pairs, Figure 1a). As both holes and electrons – which correspond directly to molecular radical cations and anions – possess spin 1/2, random recombination and Fermionic spin statistics dictate that the excitons formed will exist in a 1:3 ratio of singlet:​triplet excited states (Figure 1b).^5^ Subsequent radiative decay from the excited states to the ground state produces light emission.

### 1.1 OLED Performance Metrics

OLED performance is assessed primarily in terms of color, operational stability, and efficiency, the latter of which is quantified in terms of its external quantum efficiency (EQE). The EQE, *η*_E_ *_QE_*, of the OLED is defined as the ratio of the number of photons exiting the device to the number of injected charges, and is dependent on the product of four terms according to equation 1:^6^

$$\eta_{EQE}=\gamma \cdot \beta \cdot \Phi_{PL}\cdot \chi_{out}$$ 1

In this expression *γ* is the Langevin recombination factor of the electron and holes, which is taken to be unity in efficient OLEDs but can be substantially less when charge recombination is not confined to the emissive layer. *Φ*_PL_ is the photoluminescence quantum yield of the emissive layer or emissive dopant contained therein, which is the ratio of photons emitted to photons absorbed and quantifies the efficiency of light produced upon photoexcitation. *β* is the fraction of electrically produced excitons that can decay radiatively, which is typically 1 for singlet excitons, 0 for triplet excitons emanating from most organic compounds, and hence 0.25 for a simple 1:3 mixture of singlets and triplets (Figure 1b). The ability of advanced emitters to harvest otherwise non-emissive triplet excitons can in practice restore *β* to 1, overcoming this fundamental limit imposed by charge recombination. The combination of these three terms (*γβΦ*_PL_) is termed the internal quantum efficiency (IQE) and represents the ratio of photons generated within the OLED compared to charges injected.^7^ The final term, *χ*_out_, is the light outcoupling efficiency, which is the fraction of light that escapes the device through a transparent electrode. This term is discussed in greater detail in the context of the orientation of the transition dipole moment (TDM) of the emitter below, although assuming isotropic TDM orientation of the emitter molecules, *χ*_out_ is around 20–30% for devices fabricated on a flat glass substrate.

In the academic literature, the overall performance of an OLED is frequently judged simply on the maximum achieved value of external quantum efficiency, EQE_max_. It is important to note that the EQE_max_ value typically occurs at very low luminance values, as OLEDs frequently operate most efficiently under minimal current density and corresponding low electrical stress. EQE_max_ values are consequently often reported at <1 cd m^–2^, corresponding to an impractically large 1 m^2^ OLED at this brightness giving off the same total light as a single candle. For applications in displays and lighting, much higher brightnesses on the order of hundreds or thousands cd m^–2^, respectively, are typically required, and so EQE_max_ is not a sufficient metric to judge the suitability of the device for most applications.^8^ We therefore quote not only EQE_max_, but also EQE at 100 cd m^–2^ (EQE_100_) and at 1,000 cd m^–2^ (EQE_1000_) wherever possible in this review, and encourage this practice in research articles.

For display applications, the color coordinate of the OLEDs, as defined by the Commission Internationale de l’Éclairage (CIE), is another key parameter which is directly linked to the spectral profile of the electroluminescence, Figure 2. A subset of all human-visible colors can be demarcated by the standard red-blue-green colur space (sRBG), which assigns ‘pure’ red, blue, and green as (0.64, 0.33), (0.15, 0.06), and (0.30, 0.60), respectively.^9^ All other color points contained within a triangle of the connecting points (Figure 2, circles and solid white line) can then be generated from mixtures of the red, green, and blue primary colors. Reflecting consumer demand for more vibrant color displays (with access to a wider color gamut), the current industry standard for ultra HD-TVs advancing towards Rec. 2020, redefines the primary colors as (0.13, 0.05), (0.17, 0.80), and (0.71, 0.29) for blue, green and red, respectively (Figure 2, squares and dotted white line).^10^ Achieving these more deeply saturated color coordinates remains an ongoing challenge for the OLED research community.

**Figure 2:**
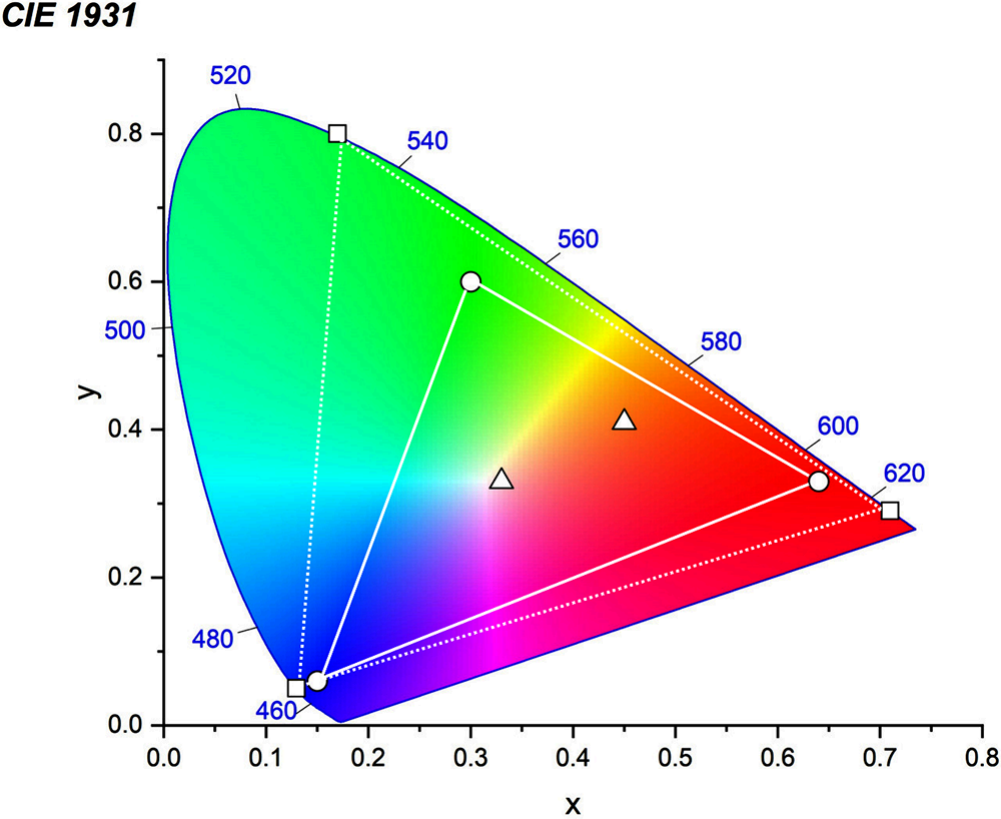
CIE diagram displaying two different color gamuts, where the sRGB color points are highlighted as circles (connected by solid white lines) and the Rec. 2020 as squares (connected by dotted white lines). Pure (0.33, 0.33) and warm (0.45, 0.41) white coordinates are identified by triangles.

In contrast to multicolor displays, for ‘white’ lighting applications there are two key relevant CIE values. Pure white is defined as having CIE coordinates of (0.33, 0.33) and is similar to outdoor daylight, while warm white has orange-tinted CIE coordinates of (0.45, 0.41).^11^ Warm white is the color associated with an incandescent light bulb, and is the most comfortable for human eyes. Ultimately, the CIE coordinates of an emitter are dependent on its emission peak, but also the spectral width of emission often quoted as the emission full width at half of the emission intensity maximum (FWHM). ‘Narrowband’ emission with small FWHM, frequently discussed in terms of color purity, is particularly prized as it allows emitters to more easily attain the saturated extreme ‘corner’ CIE coordinates required by evolving color standards. Broader emission spectra instead correspond to CIE values closer to white (0.33,0.33), as they contain a larger fraction of the entire visible spectrum. OLED pixels with these broad emission profiles therefore produce color displays limited to unappealing ‘white-ish’ desaturated color.

Beyond the efficiency and color of the device, its operational stability is also central to its performance and commercial applicability. The decrease in EQE with increasing driving voltage and luminance (efficiency roll-off) provides a useful insight into the stability of the OLED, where a stable device shows only a minimal efficiency roll-off and retains high EQE even at high luminances.^12^ Another important and related metric to assess the stability of an OLED is the operational lifetime of the device (LT_*n*_), which is defined as the time taken for the device performance under constant driving current to degrade to a certain percentage of its initial brightness (subscript *n*). There is to date no universally agreed starting luminescence value nor a target percentage decrease used to report device lifetimes in the literature; however, LT_90_ and LT_50_ using an initial luminance of 1,000 cd m^–2^ are the most frequently reported device lifetime metrics. Short operational lifetimes are deeply unappealing for consumer applications of OLEDs, as the brightness of the panel will reduce noticeably through normal use. Different operational lifetimes of the differently colored display subpixels can also lead to a color-shift of the display, as the different colors reduce in achievable brightnesses at different rates, and so industry is most interested in LT_90_ or LT_95_.

Device stability is directly associated with the photochemical and electrochemical degradation of OLED materials. Under electrical excitation, high-energy species can form through undesired competing bi-excitonic processes such as triplet-triplet annihilation (TTA), singlet-triplet annihilation (STA), singlet-polaron annihilation (SPA), and triplet-polaron annihilation (TPA), which then initiate unwanted chemical transformations and degradation of OLED materials.^12^ The frequency of these bi-excitonic processes is dependent on the density of the excitons, and become more prevalent at high exciton concentration and higher driving currents. Consequently, device lifetimes are not linear with driving current or starting luminance, as higher driving currents will cause the OLED to operate at lower EQE, with this lower emission efficiency permitting faster degradation within the device and a shorter lifetime. Therefore, the longer-lived triplet excitons in devices are often considered the primary driver of degradation. Rapidly harvesting these triplet excited states to efficiently produce light (or even just quenching them to the inert ground state) is viewed as the key to improving both the device efficiency and stability.

### 1.2 Exciton Harvesting in OLEDs

The first-generation of OLEDs contained simple fluorescent emitters, and thus light was only produced from the radiative decay of the singlet excitons, as radiative triplet exciton decay is a spin-forbidden process and thus in these devices these excitons only decayed non-radiatively (Figure 3).^5^ As a result, the *β* of these devices was 0.25 and the maximum IQE (IQE_max_) of early fluorescent OLEDs was capped at 25%. In 1987, Tang and VanSlyke at Kodak were the first to report a functional fluorescent OLED that could operate at modest electric potential, employing **Alq3** as the emitter with an EQE_max_ of ∼1%.^13^ Despite the exploration of a wide range of fluorescent emitters in OLEDs, the limit of 25% IQE_max_ along with typical outcoupling capped the overall EQE_max_ to no greater than around 5% for these first-generation OLEDs.

**Figure 3:**
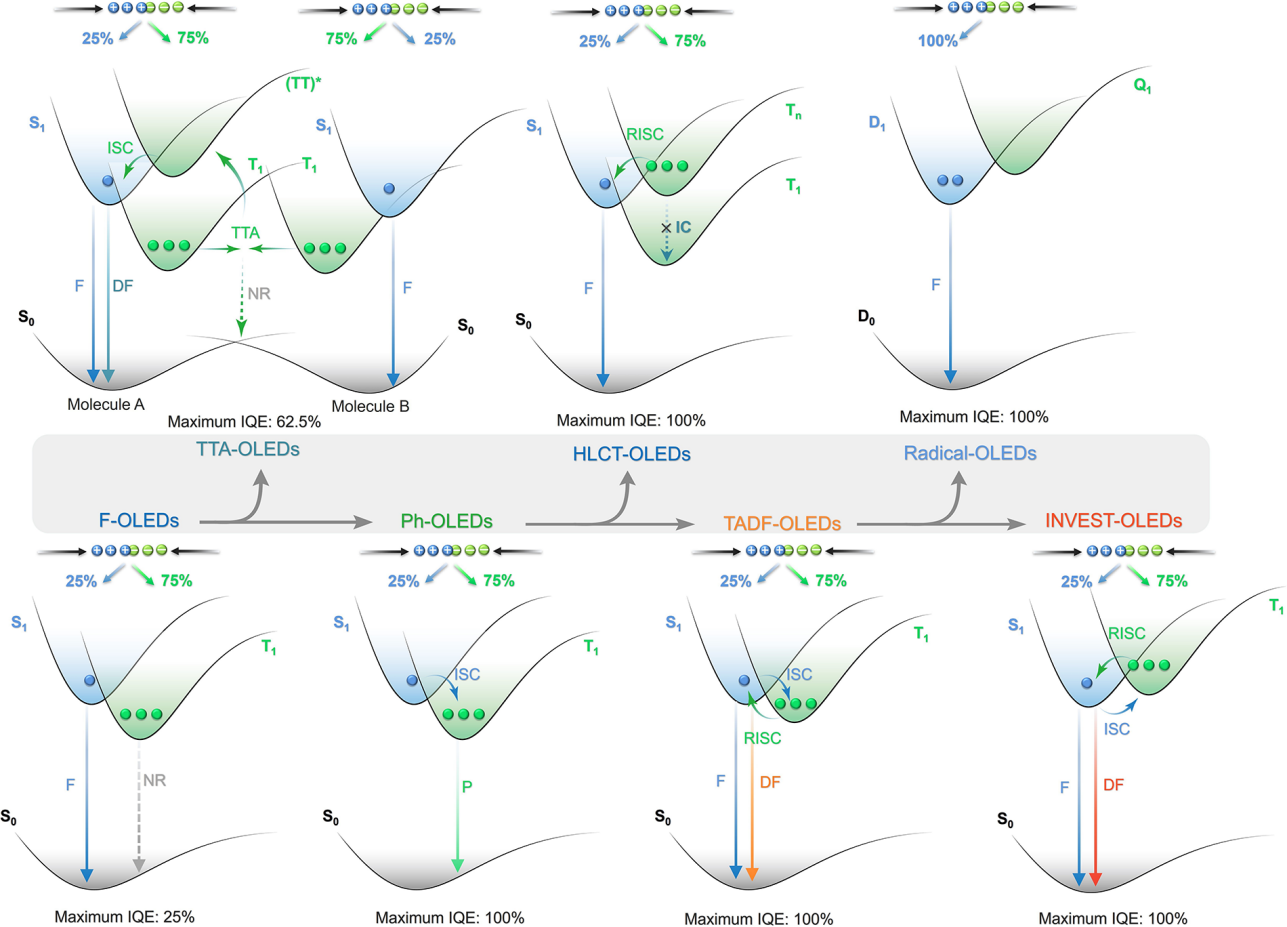
Exciton formation mechanisms in different classes of OLEDs and associated maximum internal quantum efficiency in the device, from fluorescent OLEDs (F-OLEDs) to inverted singlet-triplet gap OLEDs (INVEST-OLEDs).

A step-change in efficiency was realized in 1998 when Baldo *et al*.^14^ produced devices that exceeded the 5% EQE_max_ limit using phosphorescent emitters materials, developing so-called PhOLEDs. Organometallic phosphorescent emitters can harvest both singlet and triplet excitons to produce light because of the strong spin-orbit coupling (SOC) mediated by a central heavy transition metal ion (e.g., Pt(II), Ir(III)) within the material. The large SOC mediates singlet and triplet spin mixing that enables both intersystem crossing (ISC) of singlet excitons to become triplets, and radiative decay from the triplet excited state in the form of phosphorescence (Figure 3). Thus, PhOLEDs can achieve up to 100% IQE_max_.^15^ This exciton harvesting strategy has now been widely adopted by industry and in commercialized OLEDs, with both the green and red subpixels of OLED displays typically employing phosphorescent emitters.^16^

However, blue phosphorescent emitters have so far failed to – and may be fundamentally incapable of – delivering the required stability demanded by industry, and so blue subpixels typically contain a fluorescent TTA material.^17^ These TTA or ‘triplet fusion’ materials are highly stable and can still harvest triplet excitons, but require two triplet excitons to generate one singlet, and so have a limiting *β* of ∼0.63 and maximum achievable IQE of ∼63% (Figure 3). There thus remains a search for new emitter materials that (1) address the color and stability deficiencies of blue phosphorescent complexes and (2) can be produced more cheaply than those containing noble metals.^18^ This context also explains the keen focus of the OLED community specifically on new blue emitters (as well as UV and NIR OLEDs),^19,20^ with other visible colors largely considered ‘solved’ problems,^21^ with mature, commercialized products.

Beyond phosphorescence, a number of exciton harvesting mechanisms exist that can convert both singlet and triplet excitons into light. These include TTA discussed above, dynamics of excited states with hybridized local and intramolecular charge transfer (HLCT) character, materials with inverted singlet-triplet gap (INVEST), doublet organic radical emitters, and thermally activated delayed fluorescence (TADF). A ‘hot exciton’ or HLCT strategy^22–24^ involves the conversion of higher-energy triplet states (T_n>1_) into singlets via reverse intersystem crossing (RISC), followed by radiative decay from the singlet manifold (Figure 3).^23^ Despite an IQE_max_ of up to 100%, such a RISC process from T_n_ must compete with typically rapid internal conversion to T_1_, and the device must also efficiently produce the higher-energy T_n_ triplet excitons in the device. This T_n_ recombination process remains poorly understood, and there are thus relatively few reports of devices using this mechanism to date.

Reports of molecules emitting via an INVEST mechanism have recently garnered much excitement in the organic semiconductor community, as this mechanism offers a tantalizingly simple mechanism for converting long-lived triplet excitons into light. Computational studies^25–27^ have provided a preliminary framework for materials design, and the first report of an INVEST OLED has recently been published.^28^ The INVEST mechanism involves a fundamental violation of Hund’s rule, where the S_1_ state is lower in energy than the T_1_ state, rendering RISC a formally exothermic process that should thus be accelerated (Figure 3). The core challenge for INVEST research is to thus fully understand and apply design rules that can deliver materials with this ‘impossible’ ordering of excited states.

Beyond the singlet-triplet picture of excited states, recent work from Ai *et al*. has highlighted that organic radicals can be used as emitters in OLEDs.^29^ As open shell systems, the excited states have spin multiplicity, as such there are no non-radiative triplets^30^ yet IQE_max_ can still reach 100% (Figure 3). Despite this promise, the chemical space is narrowly explored, based only on donor-decorated tris(trichlorophenyl) radicals, and emission is limited to the red region.^30^

Now an established research theme globally, TADF involves the endothermic upconversion of triplet excitons into singlets followed by radiative decay, ensuring 100% IQE_max_ is possible (Figure 3).^31^ The research and development of TADF-based materials has progressed rapidly since the first report of a TADF material used in an OLED in 2009.^32^ As well as driving progress in state-of-the-art device efficiency, the use of TADF materials has also branched out to include other uses in OLEDs such as host materials,^33^ exciton harvesting materials in hyperfluorescent OLEDs,^34,35^ in other electroluminescent devices such as light-emitting electrochemical cells (LECs), as photocatalysts,^36^ bioimaging reagents,^37^ optical components in sensors,^38^ and as materials in photovoltaics and lasing.^39^

Since our last comprehensive review of TADF materials in 2017,^40^ several other reviews have been published, focusing on various facets of TADF materials design and their applications.^36,37,41–56^ Readers are recommended to these reviews to gain an appreciation of the evolution of our understanding of TADF and the materials that operate via this mechanism. In this review we focus on the use of TADF in OLEDs as well as emphasising their wider applications.^40^ We document the diversity of material categories that show TADF, moving beyond organic twisted donor-acceptor (D-A) systems and covering multi-resonant TADF (MR-TADF) materials, exciplexes, macromolecules such as polymers and dendrimers, and metal complexes. We discuss how TADF materials can also exhibit other interesting and valuable photophysical properties such as circularly polarized luminescence (CPL), aggregation induced emission (AIE), mechanochromism, and excited-state intramolecular proton transfer (ESIPT). Beyond their use as emitters in OLEDs, we also discuss examples where TADF materials have been employed as hosts, and as both terminal emitters and as exciton harvesters in hyperfluorescent OLEDs (HF-OLEDs). Finally, we cover their use in applications such as bioimaging, sensors, photocatalysis, supramolecular chemistry, and lasers.

### 1.3 Early History of Thermally Activated Delayed Fluorescence (TADF)

While fluorescence is typically a fast (ns timescale) process, the recognition of ‘slow’ microsecond-to-millisecond TADF is not new, and there are reports of this photophysical process dating back to 1929 (Figure 4). Delayed emission was first reported by Perrin while studying **Eosin Y**,^57^ where it was referred to as “fluorescence with long duration”, distinct from phosphorescence, which was termed “true phosphorescence” in this work.^58^ Subsequent studies by Boudin in 1930, again studying the long-lived emission observed in a solution of **Eosin Y**, miscategorized the delayed emission as room-temperature phosphorescence (RTP).^59^ Subsequent reports expanded on this initial incorrect assignment (vide infra).^60^ TADF was described qualitatively to occur in **fluorescein** (Figure 4) by Lewis *et al*. in the 1940s, with measurements made in boric acid glass showing distinct phosphorescence and fluorescence bands.^58^ A temperature-dependent delayed fluorescence was reported as a “thermally activated” process, disappearing below −35 °C and with an approximate activation energy of 8 ± 1 kcal/mol. The putative mechanism was presented in the form of a Jablonski diagram, mimicking the TADF picture that is widely reproduced today, where TADF was called the “alpha process” to distinguish it from phosphorescence, termed the “beta process”. In 1961, studies of **Eosin Y** in solution undertaken by Parker and Hatchard demonstrated conclusively that the detected photoluminescence (PL) resulted from TADF,^60^ work that directly led from the earlier observations of Boudin.^59^ The measurements performed by Parker and Hatchard were in ethanol and glycol solutions, with the researchers firstly noting a low intensity red-shifted emission peak, missed by Boudin, which they ascribed to phosphorescence, while the main peak was assigned to TADF.^60^ Emission intensity differences as a function of temperature between the two peaks helped confirm the TADF mechanism analogous to the earlier observations of Lewis *et al*. An in-depth kinetics study revealed an approximate rate constant for ISC (*k*_ISC_ ∼ 4 × 10^7^ s^–1^) and one for the “reverse process” (5 × 10^7^ s^–1^), which we now know as RISC. They concluded that the activation energy should be equal to the energetic difference between singlet and triplet excited states, which we now know to be a crude approximation of the activation energy for RISC (*vide infra*). The changes in *k*_RISC_ between ethanol and glycol were ascribed to their differing viscosities, with the greater viscosity of glycol translated to faster *k*_RISC_. Subsequent work in the 1960s sought to distinguish the delayed emission in TADF from the newly identified TTA mechanism, with TADF now referred as E-type fluorescence, distinct from P-type fluorescence (TTA), where the E and P monickers referring to Eosin-type and Pyrene-type emission, respectively, the molecules wherein these phenomena were observed.^61,62^

**Figure 4:**
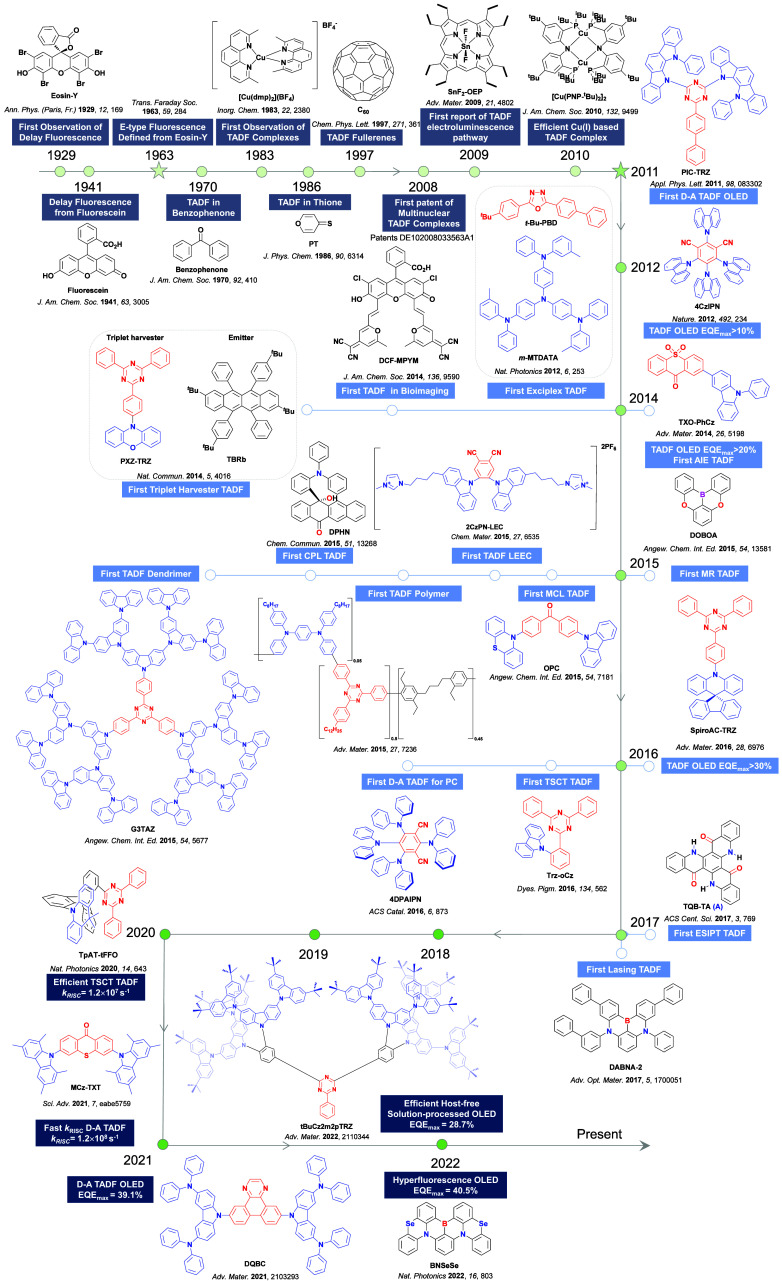
Timeline of key milestones and structures of TADF materials (the blue color signifies donor moieties, while the red color signifies acceptor moieties).

In the 1970s the origin of the delayed emission of benzophenone was probed independently by several groups (Figure 4), with TADF initially proposed as the emission mechanism by Saltiel *et al*.^63^ They observed a high-energy shoulder in the benzophenone emission spectra in carbon tetrachloride, assigned to fluorescence, and noted that the intensity of this band increased with temperature. Time-resolved PL studies by Parks, Brown and Singer, corroborated this assignment where they observed that fluorescence band persisted even after 10 ns in benzene solution and assigned this longer-lived emission as a delayed fluorescence distinct from prompt fluorescence.^64^ Subsequent in-depth analysis by the same group using benzophenone and several derivatives^65^ demonstrated that the decay mechanism of benzophenone type materials is complex, with contributions to the PL from prompt fluorescence, TADF, TTA, and RTP. They calculated the triplet to singlet activation energy to be 3.9–5.1 kcal/mol across their series. Work on structurally related thiones undertaken initially by Maciejewski *et al*.^66^ revealed similar behavior. They studied four structurally distinct thiones, each showing the same phenomenon of a high-energy shoulder of the PL spectra in deoxygenated non-polar solvents. Due to its long PL lifetime, the origin of this shoulder was ascribed to TADF. At temperatures below 220 K this spectral feature disappeared, indicating its appearance to originate from an endothermic process, while both the intensity of the TADF and phosphorescence bands showed a sensitivity to oxygen. Across the series of thiones, as Δ*E*_ST_ decreased, the amount of TADF increased, with **PT** (Figure 4) having the smallest Δ*E*_ST_ of the series.

Observation of TADF was also documented in the late 1990s in C_60_ and C_70_ by Berberan-Santos and co-workers.^67,68^ It was first noted in C_70_, where the usually weak fluorescence observed was enhanced by two orders of magnitude with increasing temperature in liquid paraffin under deoxygenated conditions thanks to the TADF.^67^ The Δ*E*_ST_ was measured to be 26 kJ mol^–1^ (0.26 eV). The study of C_60_ followed shortly thereafter, with a somewhat larger measured Δ*E*_ST_ of 35 kJ mol^–1^ in USP light oil solution.

TADF has also been observed in transition metal complexes, first noted in Cu(I) complexes in the 1980s, though this assignment was initially in dispute.^69–71^ McMillin and co-workers first reported TADF in three mononuclear Cu(I) complexes containing different nitrogen heterocyclic ligands, with **[Cu(dmp)_2_]BF_4_** investigated in detail (Figure 4). In degassed DCM solutions a decreased emission intensity was observed with decreasing temperature, which the authors assigned to TADF. A thermal equilibrium between the triplet and singlet excited states was posited to occur due to the modest calculated Δ*E*_ST_ of 1,800 cm^–1^ (0.22 eV). This two-state TADF mechanism was disputed by Parker and Crosby, who ascribe the emission decay to occur exclusively from the triplet state in this class of material.^69^ Subsequent temperature-dependent measurements by McMillin and co-worker confirmed the original TADF mechanism.^71^ The first example of a patent protecting the IP surrounding TADF metal complexes was authored by Yersin and Monkowius and had a priority filing in 2008 (published in 2010).^72^ The patent disclosed the use of di- and trinuclear metal complexes that possessed small Δ*E*_ST_ (50–2,000 cm^–1^/0.006–0.25 eV) to achieve triplet harvesting following thermal activation. Metals disclosed in the patent included mainly 2^nd^ and 3^rd^ transition row elements. This patent has now been withdrawn.

In 2009, the first example of a non-transition metal TADF emitter for OLEDs was used in terms of a tin(IV) porphyrin-based complex.^32^ Six emitters were investigated photophysically, with an enhancement in emission intensity with increasing temperature confirming their TADF character. Of the family of six emitters studied, all of which were demonstrated to emit TADF from temperature-dependent PL studies, **SnF_2_-OEP** was probed in the greatest detail as a 2 wt% doped film in PVCz. Streak camera images showed TADF until 200 K, while overall Φ_PL_ increased from 1.2% at this temperature to approximately 3.0% at 400 K, again consistent with TADF. The Δ*E*_ST_ extracted from an Arrhenius analysis was 0.24 eV, this moderate gap resulted in inefficient TADF associated with a *k*_RISC_ of 5 × 10^1^ s^–1^ at 300 K. Devices were fabricated though no EQE_max_ was reported, expected to be small given the low Φ_PL_ and inefficient *k*_RISC_. Although TADF was not conclusively demonstrated as the electroluminescent pathway given the poor device efficiency, the streak camera showed an enhancement in the electroluminescence intensity at elevated temperatures that is consistent with TADF as the emission mechanism. EQE_max_ values far surpassing the 5% fluorescence limit were first reported in 2010 ^73^ in devices with the copper(I) complex, **[Cu(PNP-^t^Bu)_2_]_2_** where the EQE_max_ of a green-emitting device was 16.1% (Figure 4). Though not explicitly discussed, it is likely that earlier examples of copper(I) based OLEDs likely emit by TADF.^74,75^ See Section 9 for more details surrounding TADF metal complexes.

The first all-organic TADF OLEDs were reported in 2011 by Adachi and co-workers,^76^ who developed the D-A emitter **PIC-TRZ** (Figure 4); it is likely that other organic compounds have been miss-reported as fluorescent or TTA emitters prior to this report. In doped films and solution **PIC-TRZ** showed an oxygen sensitive delayed emission that is consistent with TADF. Devices showed an EQE_max_ reaching only 5.3%, this due to the low Φ_PL_ of this compound. Streak camera and time-resolved EL studies confirmed that TADF was operational in the devices; the calculated IQE was 34%, surpassing the theoretical limit imposed on fluorescent systems. The following year the same group disclosed a new family of D-A compounds based on carbazolyl dicyanobenzenes (CDCBs).^31^ In this seminal report, the authors reported sky-blue to red emitters and their use in state-of-the art OLEDs using all-organic emitters. The green-emitting OLED using **4CzIPN** performed exceptionally well, with a EQE_max_ of 19.1%. This work demonstrated conclusively that high EQE_max_ devices could be fabricated using purely organic compounds as emitters. Since then, thousands of materials based on their initial D-A design have been reported. Since then, TADF emitters have been the subject of numerous studies and applications as represented in Figure 5.

**Figure 5:**
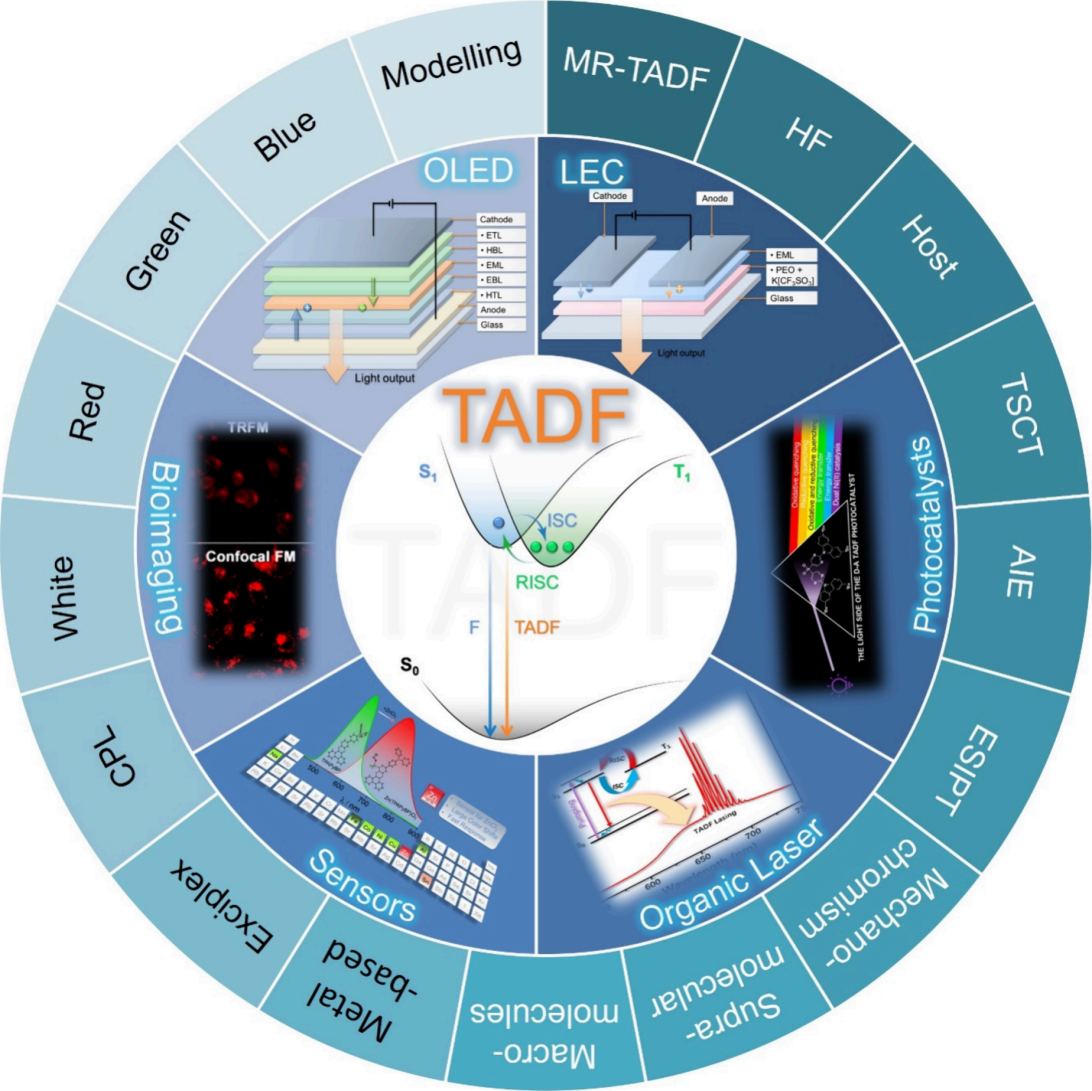
Overview of categories of TADF materials and the applications that benefit from their use.

### 1.4 A Deep Dive into the TADF Mechanism

TADF involves the upconversion of T_1_ excitons to S_1_ excitons via a RISC process, evidenced by a biexponential decay profile in the transient PL.^77^ When a TADF compound is excited by light (photoexcitation), singlet excited states are first populated. These singlet excitons typically relax to S_1_ by rapid internal conversion (IC) and vibrational relaxation (VR) processes, typically following Kasha’s rule.^78^ The generated S_1_ excitons can either decay radiatively or non-radiatively to the ground state, or be converted to T_1_ or T_n_ triplet excitons by ISC owing to the non-trivial SOC and the small singlet-triplet energy gap, Δ*E*_ST_, whereby these all rapidly populate T_1_ by IC and VR processes. The radiative decay from the S_1_ state is experimentally detected as prompt fluorescence with emission lifetimes, t_p_, on the order of 10^–9^ – 10^–7^ s. The triplet excitons can also decay radiatively as phosphorescence or non-radiatively. In TADF molecules, however, thermal upconversion to the singlet state via RISC can occur. The emission from S_1_ that results from the eventual radiative decay following RISC (or potentially several ISC/RISC cycles) is observed as delayed fluorescence, with the same emission spectrum associated with a distinct delayed emission lifetime, τ_d_, of 10^8^–10^2^ s^–1^.^77^ RISC is formally a spin-forbidden process based on the spin selection rules; however, RISC becomes possible once state mixing occurs. As RISC is an endothermic process, an increase in the temperature will result in a faster RISC rate.^79^ This is manifested in an observed increase in the intensity and an acceleration in the decay rate of the delayed fluorescence with temperature, which partially distinguishes this mechanism from TTA.^77^ Under electrical excitation singlet and triplet excitons are formed in a ratio of 1:3, resulting in a significantly larger initial triplet exciton population. The emission in the device results from fluorescence from singlet states, populated simultaneously by direct formation of singlet excitons and by RISC acting on triplet excitons (Figure 6). In this process, the RISC is typically the rate-limiting step to delayed emission and a key determinant of OLED performance. Therefore, a deep understanding of the mechanism of RISC, methods to reliably quantify it, and an appreciation of the ratio of ISC:RISC that affects the relative population of singlet and triplet excitons are required to push TADF materials design further.

**Figure 6:**
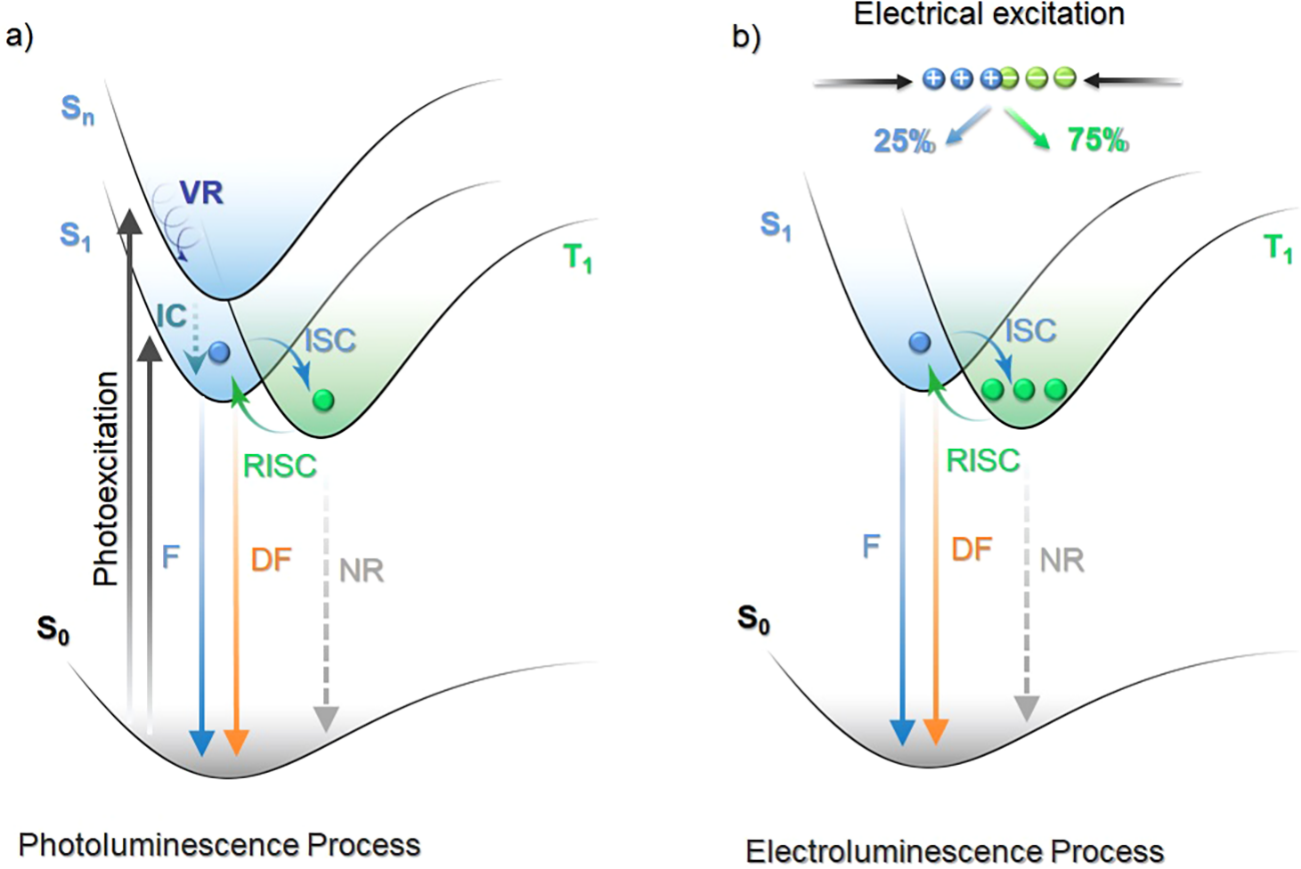
Diagram illustrating the TADF mechanism following a) photoexcitation and b) electrical excitation. S_0_ and S_1_ are the ground and the excited singlet states, respectively; T_1_ is the triplet state; S_n_ refers to the higher-lying singlet state; ISC is the intersystem crossing and RISC the reverse intersystem crossing processes; F and DF are the prompt and delayed fluorescence, respectively; NR is the nonradiative transition process; and VR refers to vibrational relaxation.

#### 1.4.1 First-Order State Mixing

The strength of the first-order mixing between singlet and triplet excited states wavefunctions is governed by the first-order mixing coefficient, *λ*, (equation 2);^31^

2 $$\lambda \propto \frac{H_{\mathrm{SOC}}}{\Delta E_{\mathrm{ST}}}$$

where *H*_SOC_ is the SOC between the relevant singlet and triplet states, and Δ*E*_ST_ is the energy difference between these states. Thus, λ is directly proportional to the magnitude of the SOC and inversely proportional to Δ*E*_ST_. The magnitude of the SOC is affected by the nature of the excited states and orbital types as described empirically by El-Sayed,^80^ as well as the atomic mass of the atoms involved in the transitions to these states, known as the heavy atom effect. El-Sayed’s rule effectively states that ISC/RISC become less forbidden (partially allowed) when accompanied by a change in orbital angular momentum, as this ensures that the total angular momentum is conserved.^81,82^ In the original paper, this was exemplified by ^1^ππ* → ^3^ππ* and ^1^nπ* → ^3^nπ* transitions having negligible SOC and small transition rates while ^1^ππ* → ^3^nπ* and ^1^nπ* → ^3^ππ* transitions have much larger SOC and rates.^82^ In TADF materials, most of the singlet and triplet excited states involves electronic transitions between π orbitals so that El-Sayed’s rule has to be revised in terms of the spatial localization of the molecular orbitals (MOs) involved in the excited state description. We distinguish cases whether the excited states are locally-excited (LE) and charge-transfer (CT) states (Figure 7).^83^

**Figure 7:**
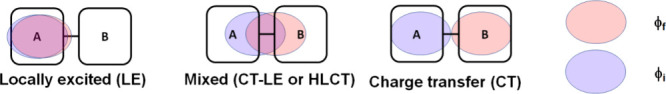
Schematic representation of different classifications of excitons based on MO overlap between initial (blue) and final (red) molecular orbitals in a hypothetical molecule with two different moieties, A and B, connected covalently to each other.

For an LE state the molecular orbitals (MOs) involved in the transition from the ground state are localised on the same part (or evenly throughout) the molecule (Figure 7) leading to a strong MO spatial overlap, large Δ*E*_ST_ and oscillator strength. There is thus a minimal electronic reorganisation upon the transition to the LE excited state, resulting in a very similar transition dipole in both the ground and in the LE excited state.

By contrast, a CT state is described by a transition from an occupied MO to an unoccupied MO that are relatively spatially segregated, and so there is a small exchange integral. Therefore, a large electronic density reorganisation upon transition to the CT excited state is observed, resulting in a large increase of the transition dipole in comparison to the ground state (Figure 7).^84^ Intermediate cases, termed mixed CT-LE states (sometimes also referred to as hybrid locally charge transfer – HLCT – states), can also exist where partial overlap between the occupied and the unoccupied MOs exists.^85^ In this picture, there is a relatively larger SOC between a triplet state of LE character and a singlet state of CT character, while the SOC is much smaller when both the singlet and triplet states have CT character.^83^ Accordingly, RISC occurring between a ^3^LE state and ^1^CT state would be allowed, while the direct upconversion from a ^3^CT to ^1^CT would be formally forbidden, assuming the transition between these excited states involves the exact same MOs. Since the majority of TADF emitters have S_1_ and T_1_ states carrying a strong CT character, SOC between these states remains very small and to thus ISC/RISC between these states is inefficient.^83^

It is clear from equation 2 that for efficient RISC to occur Δ*E*_ST_ must be minimized. The threshold value of Δ*E*_ST_ where non-negligible RISC is reported is often presented as <0.2 eV, with thermal energy at room temperature able to overcome the energy barrier between excited states.^86^ When approximating that both T_1_ and S_1_ originate from HOMO to LUMO transitions, Δ*E*_ST_ as well as the energies of the two states (*E*_T_ and *E*_S_) can be framed within Hartree-Fock theory (equation 3):

3 $$\Delta E_{\mathrm{ST}}=\underset{E_{S}}{\underset{︸}{\left(E+J+K\right)}}-\underset{E_{T}}{\underset{︸}{\left(E+J-K\right)}}=2K$$

Since the HOMO-LUMO orbital energy difference, *E*, as well as the Coulomb repulsion energy, *J*, are the same for T_1_ and S_1_, Δ*E*_ST_ can be expressed solely in terms of the exchange integral, *K*. The exchange integral quantifies the interaction between the unpaired electrons in S_1_ or T_1_ and S_0_, where the distribution can be approximated to the LUMO and HOMO, respectively (equation 4):

4 $$K=\frac{e^{2}}{4\pi \varepsilon_{0}}\int \int \phi_{\mathrm{HOMO}}^{*}\left(r_{1}\right)\phi_{\mathrm{LUMO}}^{*}\left(r_{2}\right)\left(\frac{1}{r_{1}-r_{2}}\right)\phi_{\mathrm{HOMO}}\left(r_{2}\right)\times \phi_{\mathrm{LUMO}}\left(r_{1}\right)dr_{1}dr_{2}$$

where *ϕ*_HOMO_ and *ϕ*_LUMO_ are the spatial wavefunctions of the HOMO and LUMO with the respective complex conjugates *ϕ*_HOMO_^*^ and *ϕ*_LUMO_^*^, *e* is the electronic charge, *ε*_0_ is the vacuum permittivity, and *r*_1_ and *r*_2_ are the positions of electron 1 and electron 2, respectively. Based on equation 4, the simplest strategy to reduce the magnitude of *K* and thus also Δ*E*_ST_ is to minimize the overlap of the electron density in the HOMO and LUMO. From a molecular design point of view, the principal manner to localize the HOMO and LUMO on different parts of the emitter is to adopt a twisted D-A architecture (*vide infra*) to induce a charge transfer character in the S_1_ and T_1_ excited states. A negative consequence of segregating the HOMO and LUMO onto different parts of the molecule results in a decrease in the radiative rate constant, *k*_r_, owing to reduced wavefunction overlap with the ground state that is quantified in terms of the oscillator strength, *f*, of the transition.^31,77^ The optimal emitter design therefore must carefully balance reducing Δ*E*_ST_ (to improve the RISC efficiency) while preserving an adequately large *f* and fast *k*_r_, which both contribute to Φ_PL_.^87^

The value of Δ*E*_ST_ can be obtained spectroscopically from the measured fluorescence and phosphorescence spectra at low temperature. Either spectral onsets or peak values of these spectra have be used to estimate the energies of their corresponding states (*E*_S_ and *E*_T_), with Δ*E*_ST_ = *E*_S_ – *E*_T_. Additionally, as the rate of RISC (*k*_RISC_) is temperature-dependent it can be approximated using an Arrhenius analysis (equation 5):

5 $$k_{\mathrm{RISC}}\propto \exp\left(-\frac{\Delta E_{a}}{k_{B}T}\right)$$

where Δ*E*_a_ is the activation energy, *k*_B_ is the Boltzmann constant and *T* is temperature. If RISC were solely dependent on energetics, a direct correlation from *E*_a_ and Δ*E*_ST_ to *k*_RISC_ would be expected.^88^ Indeed, while there is a strong trend of smaller Δ*E*_ST_ producing faster RISC, this relationship is not always linear, with numerous anomalous examples in the literature where the emitter possesses a relatively large Δ*E*_ST_ yet unexpectedly fast *k*_RISC_ as inferred from photophysical data.^89^ Therefore, TADF efficiency cannot be explained only in terms of the first-order mixing of states; spin-vibronic coupling of states may also be important, which implies second-order mixing.

#### 1.4.2 Second-Order State Mixing

Indeed, in recent years it has become widely accepted that the three-state model (S_1_, T_1_, and S_0_, that invokes only first-order mixing between S_1_ and T_1_) is too simple to account for the observed photophysics in many organic TADF emitters. In a second-order state mixing picture the Born–Oppenheimer approximation is broken,^90^ and interactions of electronic and vibrational degrees of freedom must also be considered. In this mechanism, upconversion from T_1_ to S_1_ occurs through the involvement of higher-lying triplet states (T_n>1_), which are accessible via reverse internal conversion (RIC) due to strong vibrational coupling between T_1_ and T_n>1_.^83^ If one of these higher-lying triplet states is of a different orbital nature than that of S_1_ (which is typically CT), then according to El-Sayed’s rule the SOC will be significantly enhanced, and RISC can then proceed much more readily. The vibrational coupling between T_1_ and T_n>1_ is maximized when the T_1_ and T_n>1_ states are sufficiently close in energy to enable efficient RIC and state-mixing to occur.^83,91^ Such a mechanism is frequently invoked to account for an efficient TADF process and the seemingly required involvement of both CT and LE states; however, evidence to support such a mechanism is most usually inferred using computational approaches (*vide infra*).^90–93^

A clear example of second-order mixing was reported by Noda *et al.*,^91^ who studied a series of structurally related emitters for which *k*_RISC_ correlates very well with the evolution of the ^3^CT and ^3^LE energy gaps (Δ*E*_TT_), Figure 8. Based on the parent emitter, **5CzBN**, the energy difference between ^3^CT and ^3^LE was calculated to be 0.32 eV (Δ*E*_ST_ measured to be 0.17 eV) and the corresponding *k*_RISC_ was 2.2 × 10^5^ s^–1^. Replacing two of the carbazole donor groups for phenyl-substituted carbazoles introduced ^3^LE states closer to the lowest-lying ^3^CT, reducing the calculated Δ*E*_TT_ to 0.16 eV for **3Cz2DPhCzBN**, which translated into a faster *k*_RISC_ of 7.2 × 10^5^ s^–1^ (Figure 8) while the measured Δ*E*_ST_ remained similar at 0.15 eV. OLEDs using these two emitters showed strongly contrasting stability, where the LT_97_ device lifetime improved from 3 hours for the OLED with **5CzBN** to 110 hours for the device with **3Cz2DPhCzBN**.

**Figure 8:**
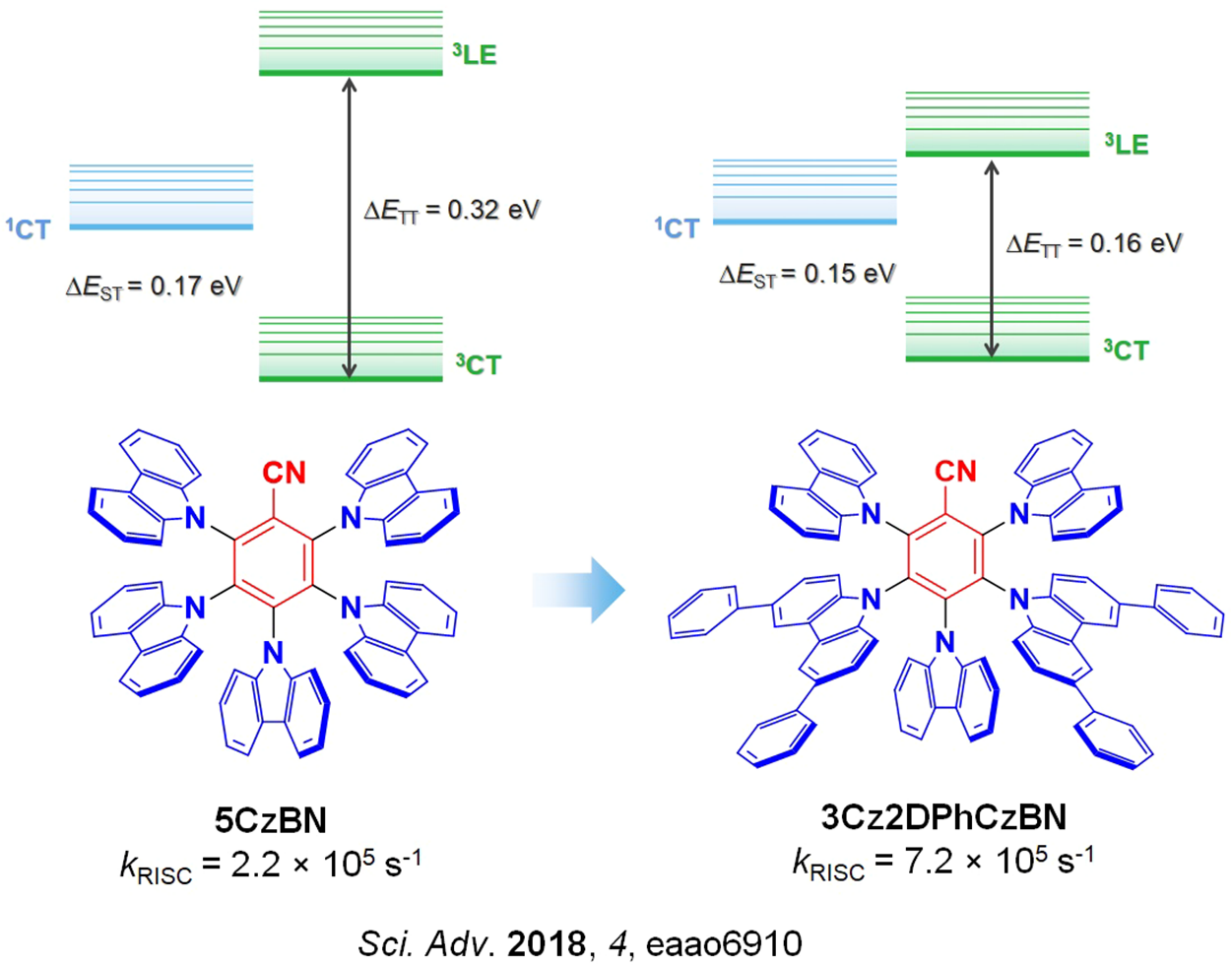
An example of modulation of the ^3^CT and ^3^LE energy levels, achieved by replacing two carbazole donor groups with 3,6-diphenylcarbazole groups, for faster *k*_RISC_ (the blue color signifies donor moieties, while the red color signifies acceptor moieties).

**Figure 9:**
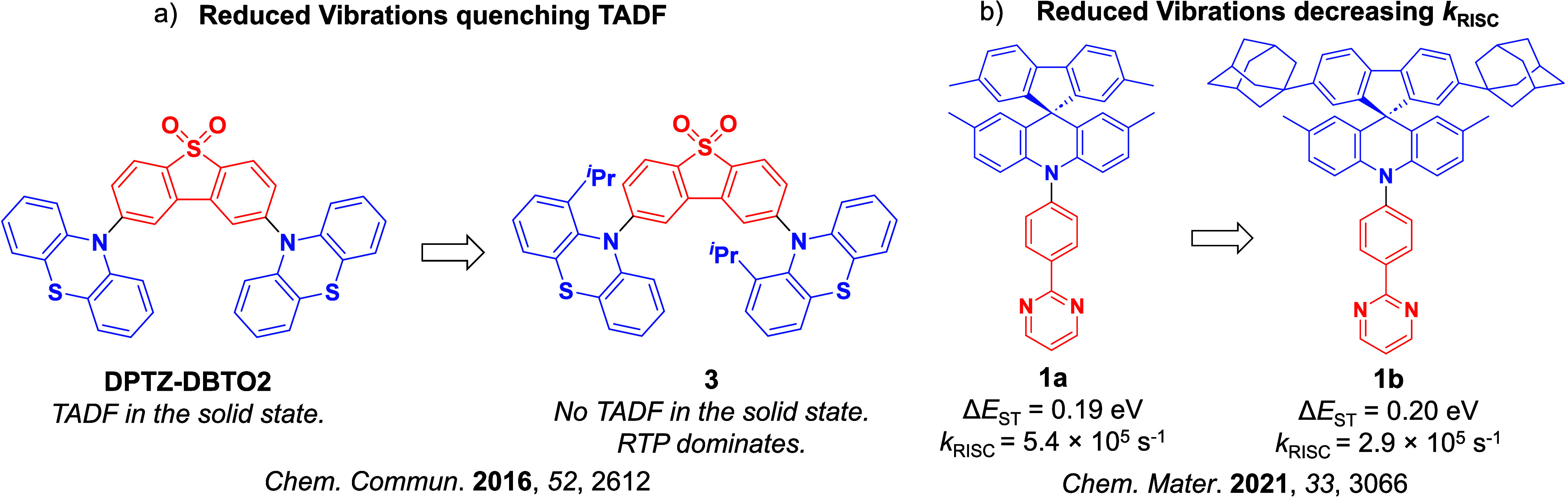
Two examples where reducing the conformational flexibility of the emitter results in a change in the photophysics. Here, a) highlights the shutdown of TADF with the addition of ^i^Pr groups and b) *k*_RISC_ is slowed upon substitution of methyl groups for adamantyl groups within the donor (the blue color signifies donor moieties, while the red color signifies acceptor moieties).

Due to large-scale dipole rearrangement and relaxation, the energy of CT states is dependent on the polarity of the medium surrounding the emitter, while the energy of LE states is largely insensitive to the surroundings. Thus, as the S_1_ state of TADF emitters is almost always CT in character, altering the polarity of the medium will also affect Δ*E*_ST_ and *k*_RISC_.^83^ External fine-tuning of the energy of the ^1^CT and ^3^CT states to closely align with the static ^3^LE levels was found to be possible with **DPTZ-DBTO2** (Figure 9), where the fastest *k*_RISC_ was observed in hosts and solvents that simultaneously minimized the ^1^CT–^3^LE and ^3^CT–^3^LE gaps. The conformational flexibility inherent in the emitter can also affect the RISC rate.^94,95^ The rigidity of the compound can hinder the necessary vibrational motion that is required for coupling to occur between triplet states, ultimately suppressing RISC. Two studies are summarized here to exemplify this effect (Figure 9). In the first, Ward *et al*. incorporated bulky substituents onto a phenothiazine (PTZ) donor of the parent emitter, **DPTZ-DBTO2** (Figure 9a).^94^ Clear TADF was observed for this compound in doped films; however, upon addition of *iso*propyl groups at the 1-position that reduces conformational flexibility as a result of increased sterics, TADF in material **3** was no longer observable, and instead RTP was the dominant emission mechanism. The second illustrative example was reported by Hempe *et al*., who showed that despite essentially unchanged singlet and triplet energy levels and similar Δ*E*_ST_ of 0.19 eV and 0.20 eV for compounds **1a** and **1b** (Figure 9b), the addition of the bulky adamantly groups led to a decreased *k*_RISC_ from 5.4 × 10^5^ s^–1^ to 2.9 × 10^5^ s^–1^ in *ortho*-dichlorobenzene, presumably due to their inertial impact on dampening D-A dihedral vibrations.^95^

### 1.5 TADF Kinetics

Control of the various decay processes for the excited states in TADF compounds is crucial to both understand and account for the efficiency of the TADF molecules.^89^ By analysing the transient PL of the compounds, many of the rate constants shown in equations 6–11 can be extracted. However, extracting every rate constant remains challenging, and in the case of *k*_RISC_ is contentious, with several methods suggested across the literature, each using a different set of assumptions to simplify the mathematics. Most of these methods rely on fitting the emission decay to a pair of mono-exponential lifetimes in the prompt (*k*_PF_^–1^) and in the delayed (*k*_DF_^–1^) florescence regime.

Adachi and co-workers first calculated *k*_RISC_ by assuming that there is no non-radiative decay from the singlet state (*k*_nr_^S^ ≈ 0) and no phosphorescence (*k*_r_^T^ ≈ 0) as equation 6:

6 $$k_{\mathrm{RISC}}=\frac{k_{\mathrm{PF}}k_{\mathrm{DF}}}{k_{\mathrm{ISC}}}\frac{\Phi_{\mathrm{DF}}}{\Phi_{\mathrm{PF}}}=k_{\mathrm{DF}}\frac{\Phi_{\mathrm{DF}}}{\Phi_{\mathrm{PF}}\Phi_{\mathrm{ISC}}}$$

where Φ_PF_ is the photoluminescence quantum yield due to only the prompt fluorescence, and Φ_DF_ is photoluminescence quantum yield from the delayed emission that is enabled by RISC.^96^ These components of Φ_PL_ are typically approximated from measurements in the presence (Φ_PF_) or absence (Φ_PF_ + Φ_DF_) of atmospheric oxygen, although this has been demonstrated to introduce its own set of issues.^97^

For TADF emitters that show significant delayed fluorescence (Φ_DF_Φ_PF_^–1^ > 4) in the transient PL, Dias *et al*. proposed that *k*_RISC_ could be approximated by equation 7,^77^ where the authors assumed that there is no non-radiative decay from the triplet state nor any phosphorescence (i.e., Φ_RISC_ ≈ 1):

7 $$k_{\mathrm{RISC}}=\frac{k_{\mathrm{DF}}}{1-\Phi_{\mathrm{ISC}}}=k_{\mathrm{DF}}\frac{\Phi_{\mathrm{PF}}+\Phi_{\mathrm{DF}}}{\Phi_{\mathrm{PF}}}$$

This model has been further refined by Kaji and co-workers to allow for the extraction of rate constants from samples that do not show a strong DF contribution in the transient PL (equation 8):^98^

8 $$k_{\mathrm{RISC}}=\frac{k_{\mathrm{DF}}+k_{\mathrm{PF}}}{2}-\sqrt{{\left(\frac{k_{\mathrm{DF}}+k_{\mathrm{PF}}}{2}\right)}^{2}-k_{\mathrm{DF}}k_{\mathrm{PF}}\left(1+\frac{\Phi_{\mathrm{DF}}}{\Phi_{\mathrm{PF}}}\right)}$$

To avoid the somewhat subjective and artificial nature of manually identifying and fitting exponential lifetimes to the prompt and delayed emission regimes, Monkman and co-workers have advanced a strategy that relies on simultaneous fitting of the entire transient PL to a three-level kinetic model using equation 9, under the assumption that the intensity of the PL is proportional to the singlet population:^99^

9 $$\frac{d}{dt}\left(\begin{array}{l} \left[S_{1}\right]\\ \left[T_{1}\right] \end{array}\right)=\left(\begin{array}{ll} -\left(k_{r}^{S}+k_{\mathrm{ISC}}\right) & k_{\mathrm{RISC}}\\ k_{\mathrm{ISC}} & -k_{\mathrm{RISC}} \end{array}\right)\left(\begin{array}{l} \left[S_{1}\right]\\ \left[T_{1}\right] \end{array}\right)$$

To simplify the fit parameters, any non-radiative decay as well as phosphorescence were assumed to contribute negligibly (*i.e.*, Φ_PL_ ≈ 1). Transient absorption spectroscopy was used to independently assess the applicability of the fitting, which simultaneously generates a decay trace of triplet population [T_1_]. Similar to this approach, kinetics modelling of the transient electroluminescence has also been employed in a device context.^100^ This approach can also be extended with additional kinetics terms, for example with the inclusion of Φ_PL_ measurements to quantify nonradiative rates.^101,102^

Nguyen *et al*. developed a method to determine *k*_RISC_ from the transient PL in the presence of an exciton quencher using a Stern–Volmer quenching experiments.^103^ The prompt and delayed fluorescence rate constants are extracted for different quencher concentrations, where the prompt and delayed fluorescence rate constants for the pristine film (*k*_PF,0_ and *k*_DF,0_, respectively) are extrapolated by a fit. The fit of the delayed emission yields *k*_ISC_ and the RISC rate is calculated according to equation 10, assuming no exciton decay from the triplet state. A similar approach was also recently reported for measuring energy transfer rates in hyperfluorescence (HF) blends, although this revealed that distributions of emitter-quencher distances in these films results in time-dependant quenching rates, which can lead to initially misleading trends.^104^

10 $$k_{\mathrm{RISC}}=\frac{{k_{\mathrm{DF},0}}^{2}-k_{\mathrm{PF},0}k_{\mathrm{PF},0}}{k_{\mathrm{ISC}}+k_{\mathrm{DF},0}-k_{\mathrm{PF},0}}$$

Recently, Tsuchiya *et al*. presented a full analysis of the three-level system, which does not require any assumptions to be made and permits the extraction of all kinetics parameters from the photophysical experiments.^102^ The RISC rate constant is calculated according to equation 11:

11 $$k_{\mathrm{RISC}}=\frac{\left(k_{\mathrm{PF}}-k^{S}\right)\left(k^{S}-k_{\mathrm{DF}}\right)}{k_{\mathrm{ISC}}}$$

where *k* ^S^ = *k*_r_^S^ + *k*_nr_^S^ + *k*_ISC_. To calculate *k*_ISC_, the ratio of the delayed emission originating from S_1_ (*i.e.*, fluorescence) to the delayed emission originating from T_1_ (phosphorescence) must be determined from an analysis of the spectral shift of the delayed emission over time.

As mentioned previously, most methods for determining *k*_RISC_ rely on different sets of assumptions. Therefore, analysing the same material and photophysics using different models can lead to a range of values for *k*_RISC_.^99,102,103^ For example, by assuming no non-radiative decay from the singlet state, the value of *k*_RISC_ is underestimated for materials with a Φ_PL_ of less than 80%.^102^ Recognizing this limitation, Tsuchiya *et al*. introduced an evaluation of the rate constants obtained with equations 6–8, revealing a range of *k*_RISC_ due to over- and underestimations when assuming negligible non-radiative decay from either the singlet or triplet states. Despite this diversity of calculation methods, it is widely recognized that new D-A TADF materials require RISC rates of ∼10^6^ or faster in order to achieve leading device performance.

### 1.6 OLED Fabrication

While relatively quick and convenient photophysical measurements can guide the design of TADF materials, once high-performance candidates are identified their electroluminescence performance must be directly confirmed. Generally, the fabrication of OLEDs occurs using one of two approaches: thermal evaporation under vacuum, which is restricted to low molecular weight-based materials (typically <1000 dalton),^105,106^ and solution-processing techniques such as spin-coating, inkjet printing, or doctor blading (which require minimum levels of solubility).^107–109^ Solution-processed OLEDs therefore are the only option for high molecular weight materials such as polymers^110^ and dendrimers,^111^ which cannot be thermally evaporated; solution-processed OLEDs using low molecular weight emitter is also possible, assuming their solubility and film-forming properties allow formation of homogenous amorphous films. OLEDs based on TADF emitters usually employ a multilayer architecture. Careful choice of the materials in these emissive and transport/injection layers permits optimal charge injection, transport, and exciton recombination kinetics that support high-efficiency devices (Figure 3 and Figure 10).

**Figure 10:**
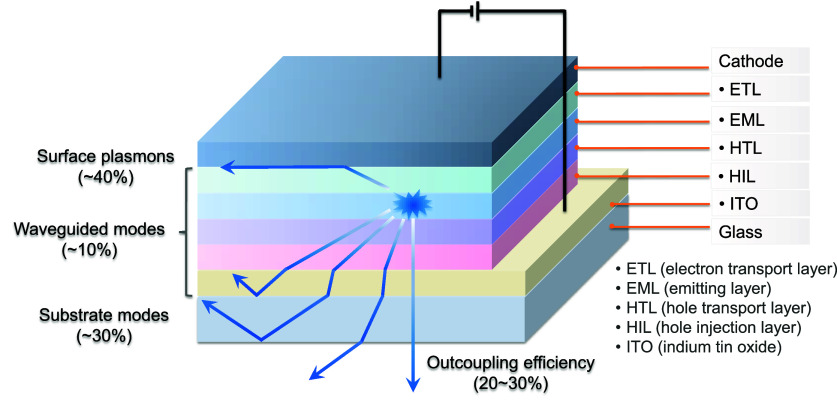
Illustration showing different trajectories of the generated photons following exciton formation within the emitting layer of an OLED.

### 1.7 Outcoupling

The photons generated inside the emissive layer of an OLED have a number of pathways by which their energy can dissipate, although only those that can escape the device are useful. These pathways include waveguided modes, substrate modes, surface plasmon polaritons (SPPs), (re)absorption, and the aforementioned direct emission (Figure 10), which are described in detail in the literature.^112–115^ The proportion of light energy that is lost to each of these pathways depends on a number of properties of the device itself, including the thickness and refractive indices of each of the constituent layers, the wavelength of the emitted light and any cavity effects related to the metallic electrode(s), the surface morphology of the glass-air interface, and, most importantly, the angle relative to the surface of the device at which the light is emitted.^113^ At shallow angles, waveguided modes in either the glass substrate or in the organic layers themselves are greatly favoured, as these pathways trap light by total internal reflection at either the organic layer interfaces or the glass/air interface. Likewise, SPPs require very shallow emission angles as they are dependent on near-field coupling of the generated light to the metallic cathode surface. All of these aforementioned modes, which are unproductive and lead to lower efficiency devices, are, however, avoided at high emission angles (i.e., emission normal to the substrate plane), where instead photons can more easily escape the device layers and thus be used productively in the outside world.

#### 1.7.1 Outcoupling Efficiency and Emitter Orientation

The outcoupling efficiency, *χ*_out_ of a device is the ratio of internally generated photons to externally emitted photons, ideally 100%. *χ*_out_ is one of the four crucial parameters of an OLED that constitutes its EQE (equation 1) and is reduced by photons that are coupled into waveguided or SPP modes, and thus is dependent on the angle of emission of the photons from within the device. The angle at which light is emitted from an excited molecule is itself not random, but rather is dependent on the orientation of the TDM of the emissive electronic transition. The majority of light emitted will be perpendicular to the TDM vector, as this is the direction in which the interaction between the oscillating molecular electric dipole and propagating light wave is strongest and thus emission is most likely to occur.^112^ Therefore, if a majority of the emitting molecular TDMs are orientated parallel to the plane of the OLED, then a greater proportion of light will leave the device *via* direct emission, resulting in greater overall efficiency. Unfortunately most molecules are deposited randomly to form isotropic films, and with no preferential orientation of the ensemble of TDMs in the EML, light outcoupling efficiency is typically limited to only 20–30%, meaning that as much as 80% of the generated photons remain trapped within the layers of the OLED. This is the origin of the 20–30% EQE_max_ limit experienced by many devices.

The overall *χ*_out_ of a device is influenced not only by any TDM orientation of the EML, but also by other properties of the device such as the emission wavelength and the thickness and refractive indices of the device layers. However, optical simulations have demonstrated that the *χ*_out_ of a device can be increased by at least as much as 50% by preferentially orientating the TDMs, compared to the isotropic case. In a device with an EQE_max_ of 20% and with an isotropic arrangement of TDMs, an equivalent device where all TDMs are preferentially orientated would therefore achieve an EQE_max_ of 30%, and reports have demonstrated that even this limit can be surpassed with further device engineering.^113^ This headroom for significantly improved OLED efficiency has attracted significant attention from the community to design emitters that possess a preferentially (horizontally) orientated TDM in the as-deposited film state. Indeed, as the IQE of modern devices has effectively reached 100% with triplet harvesting strategies, light outcoupling remains as a key factor for advancing higher efficiency devices.

#### 1.7.2 Controlling Orientation

A number of molecular properties are now known to influence the TDM alignment of emitters in a film.^112^ Of these, the most well documented are the length and/or width of the emitter (and/or the aspect ratio of the emitter), the glass-transition temperature of the host, the temperature of the substrate during deposition and, perhaps most importantly, the deposition mechanism by which the film is made. No single one of these parameters can be used in isolation to control or predict the TDM alignment of the emitters in a film, but TDM alignment is typically achieved by using emitters with greater molecular length and/or width (*i.e.*, larger aspect ratio), hosts with a higher glass-transition temperature, substrates with a lower temperature and films synthesised by vacuum deposition (as opposed to solution-processing such as spin-coating). In particular any preferential horizontal alignment of the TDMs exhibited in a vacuum-deposited film is typically lost in an equivalent solution-processed film, as the molecules deposit to form a film all-at-once from a randomised solution state, rather than gradually building up from a molecular beam on a surface.

Despite these advancements, a unifying theory by which complete horizontal TDM alignment can be reliably achieved remains elusive, as the interplay between the different effects influencing TDM alignment is poorly understood.^112^ In addition, new parameters that impact TDM orientation are still being reported, and it is more than likely that yet more await discovery. We recently reviewed this topic in-depth,^112^ and concluded that the following are all parameters that can induce horizontal TDM orientation in TADF emitters: high molecular weight of the emitter; high linearity of the emitter; high molecular weight of the host; small thickness of the emitter; greater length of the emitter relative to the host; and high glass transition temperature of the host. It was additionally found that the relative importance of each of these parameters depends on the exact system under study. For example, for low molecular weight emitters (MW < 600 g mol^–1^) the most influential parameter is the glass transition temperature of the host, while for heavier emitters the degree of horizontal orientation is better correlated to the molecular weight of the emitter itself. Finally, in the literature, many authors have used arguments relating to the high aspect ratio of the emitter to explain preferential horizontal orientation and the resulting high EQE_max_. These arguments are supported by the extensive work by Yokoyama *et al*.^116^ in demonstrating that molecules with higher aspect ratios tend to preferentially orient horizontally in thin films, thus also aligning the TDM horizontally so long as the TDM is aligned along the plane of the molecule itself. However, it is rare for the aspect ratio of a molecule to be quantified in the literature, making the true strength of this relationship hard to ascertain.^112^ Further, it is unclear whether the aspect ratio of a molecule is a meaningful predictor of TDM orientation in its own right. Instead, it may merely be a proxy for other parameters, such as molecular length and weight, as molecules with higher aspect ratios tend to be longer and therefore heavier. Thus, the challenge of controlling the TDM orientation of the emitters within the EML remains unsolved, and further research is required in order to construct a set of comprehensive design paradigms by which perfectly horizontal TDM orientation can be reliably enforced.

### 1.8 Outlook

Although the mechanism for TADF is much more complex than simple thermal upconversion of T_1_ to S_1_ states, in practice the magnitude of Δ*E*_ST_ largely dictates the feasibility of the process and reducing Δ*E*_ST_ is almost always a desirable strategy for the design of new TADF materials. According to equation 3, reducing the electron density overlap between HOMO and LUMO can effectively reduce the Δ*E*_ST_, provided the transition is predominantly HOMO to LUMO. This has been achieved in D-A systems, which can be in the form of twisted intramolecular D-A compounds or molecules that possess pseudo co-planar D and A groups that possess through-space charge transfer (TSCT) states, or in exciplexes where distinct donor and acceptor molecules interact weakly intermolecularly via π-π bonding. In this context a donor is an electron-rich group while an acceptor is an electron-deficient moiety, where the HOMO is situated on the donor and the LUMO on the acceptor.^86^

For twisted D-A compounds, minimization of the exchange integral and thus Δ*E*_ST_ can be achieved through (1) the use of substituents close to the D-A bond such as addition of methyl groups to confer a highly twisted conformation,^117^ or (2) the inclusion of multiple donors or acceptors, which forces large torsions to mitigate steric congestion between these moieties.^31^ Donors like 9,9-di­meth­yl-9,10-di­hy­dro­acri­dine (DMAC), phen­oxa­zine (PXZ), and pheno­thia­zine (PTZ) that are linked to acceptors via the nitrogen atom adopt highly twisted conformations owing to their bulky nature.^117^ Although thousands of D-A based TADF emitters have been reported, they are ultimately composed of a relatively limited diversity of D or A units (Figure 11).^40,42,87^ Color tuning in D-A TADF systems is possible by altering the strength of the donor and acceptor groups, which affects both the band gap, Δ*E*, as well as the energy of the excited states. Increasing the donor strength destabilizes the HOMO, while increasing the acceptor strength stabilizes the LUMO, both of which decrease the energy of the excited states. The emission spectrum in D-A TADF compounds is generally broad, which is due to a large geometric reorganization in the CT excited state, characterized by a large FWHM.^118^ To improve the emission purity of the molecule, incorporation of substituents that not only suppress vibrations but also increase rigidity are needed. Beyond adjusting the structures of the donors and acceptors, the properties are also dependent on their relative regiochemistry.^119–121^ Intramolecular interactions can also influence both the emission color and the TADF efficiencies.^122,123^ As well, the photophysical properties of compounds are also affected by their environment and intermolecular interactions.^124–126^

**Figure 11:**
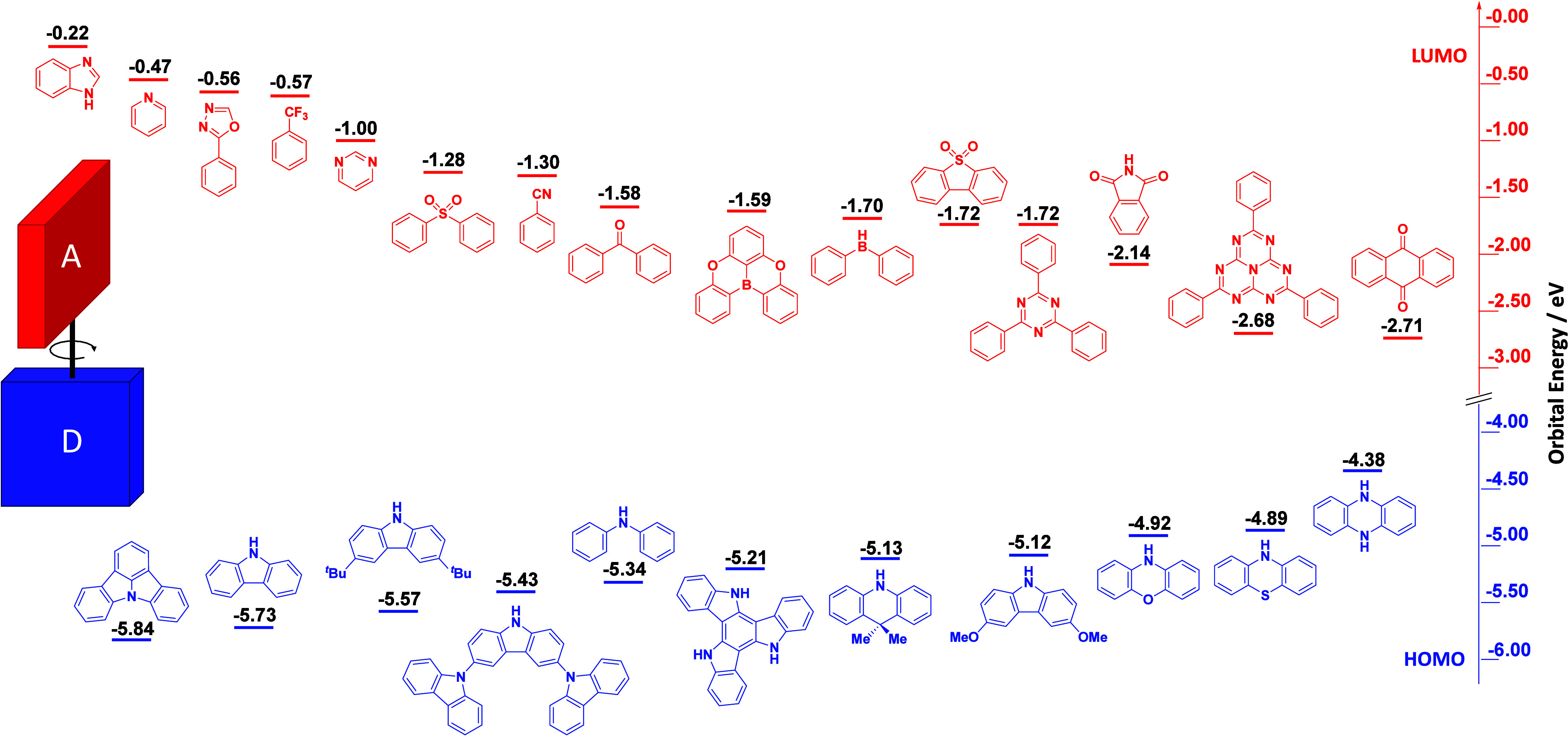
Schematic of a D-A TADF emitter design, with examples of widely used donors and acceptors and their respective HOMO and LUMO values calculated in the gas phase using DFT (PBE0/6-31G(d,p)). The blue color signifies donor moieties, while the red color signifies acceptor moieties.

**Figure 12:**
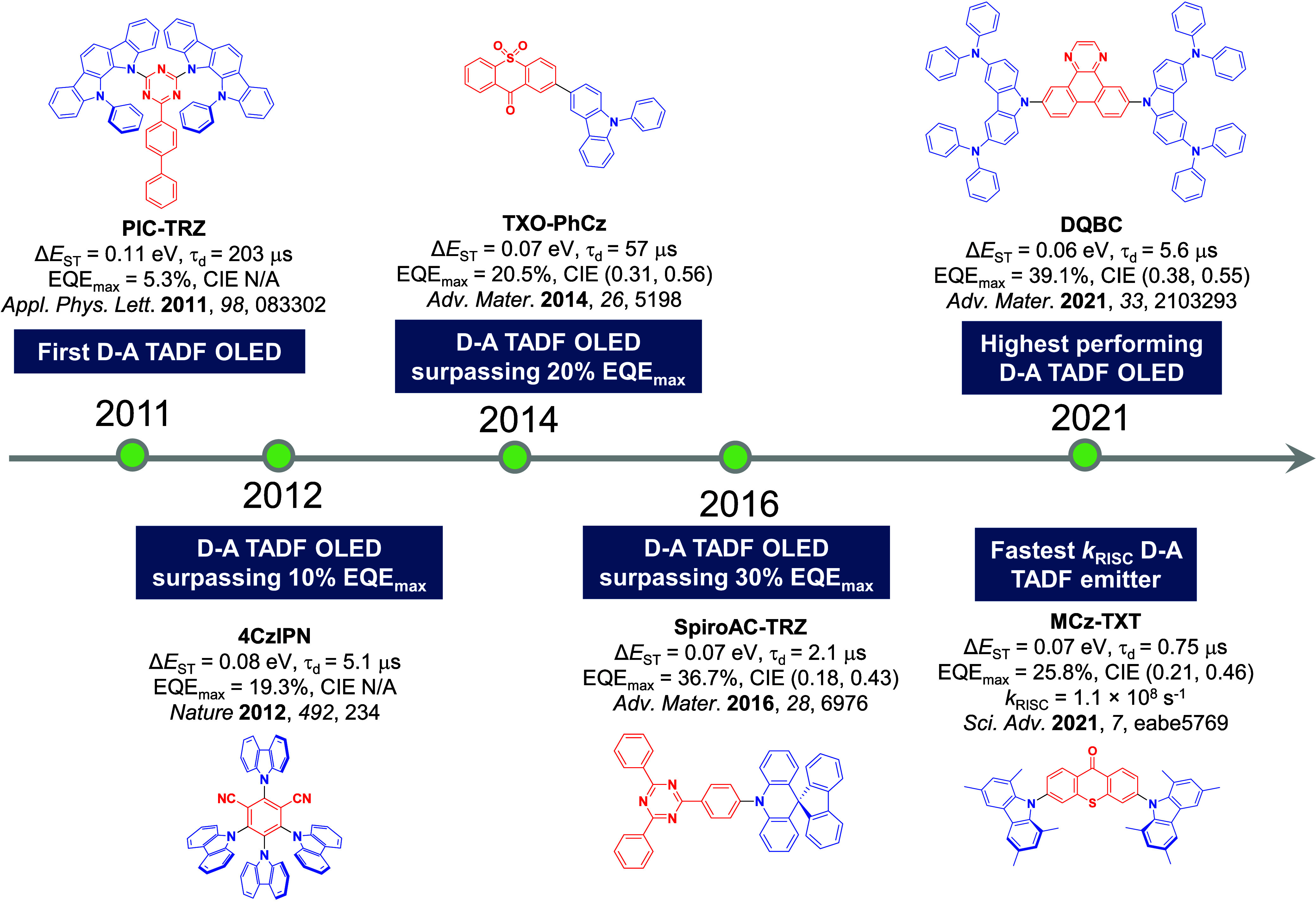
Timeline, structures, properties, and device data of key milestones in D-A TADF emitter development from 2011 to 2022 (the blue color signifies donor moieties, while the red color signifies acceptor moieties).

This simple D-A design paradigm is nonetheless the most commonly adopted by the community and has led to an explosion of examples since 2011,^76^ aided by the predictive power of density functional theory calculations (Figure 12). A steady increase in overall EQE_max_ has been driven by a combination of improved emitter and OLED design. It is now much more common, for instance, to witness reports of OLED efficiencies surpassing 30%. Blue, green, and red donor-acceptor designs are surveyed in Sections 3, 4, and 5, respectively. Each of these sections focuses on trends in properties as a function of common structural motifs. Design rules for other classes of TADF compounds such as TSCT emitters (Section 12), exciplexes (Section 8), metal complexes (Section 9), and MR-TADF materials (Section 11) will be covered separately. Regardless of structure, the impact, interest, and pace of exploration of TADF and the materials that emit via this mechanism have clearly captured the interest and imagination of chemists, physicists, and materials scientists globally.

## 2 Molecular Modelling

### 2.1 Introduction

Computational chemistry is now routinely used in the literature as a valuable predictive tool to design and understand new TADF materials. Concurrently, the TADF field has inspired computational chemists and physicists to develop and refine new methodologies to accurately describe the nature, energies, and transition rates between the excited states of existing emitters. These methodologies are essentially divided in two categories: time-dependent density functional theory (TD-DFT) and wave-function-based approaches.^127^ An accurate description of the excited states is key to gaining insight into the mechanistic aspects behind the TADF process, especially when modelling the excited state dynamics.^128–133^ This requires identification of key intrinsic features associated with the excited states of TADF materials as well as their interactions, each of which must be accurately modelled. Specifically, an ideal computational protocol must be able to accurately model the orbitals and energies of singlet and triplet excited states, which for organic compounds are either LE, CT, or mixed CT-LE (HLCT).^134^ Compounding this challenge, the effect of the solvent or host environment can play a significant role, even wholly reshuffling the relative energies of both singlet and triplet excited states. Indeed, this new ordering of excited states can be significantly different than in gas-phase modelling which affects both the TADF mechanism and its efficiency.^135^ Furthermore, since electron-phonon coupling is usually large in organic π-conjugated materials, and since molecular vibrations are fundamental to the electronic processes governing TADF, the dynamic nature of the excited-state landscape makes TADF particularly challenge to accurately model. Beyond detailed investigation of excited states for single compounds, large scale high-throughput computational screening protocols have also been implemented to assist in materials design and identification. Here, we will briefly discuss these different computational approaches in view of the recent literature.

### 2.2 Excited-State Energy Level Calculations and the Prediction of Their Nature

#### 2.2.1 Predicting Δ*E*_ST_

Although the mechanism behind TADF is frequently more complex than direct RISC from T_1_ to S_1_,^83^ Δ*E*_ST_ remains the key guiding parameter that both experimentalists and theoreticians use to identify emitters as promising targets.^136^ The community has largely used TD-DFT, which is well-adapted for organic D-A (see for instance Sections 3–5) and carbene metal amide (CMA) (see Section 9) TADF emitters. However, the features of the excited states of MR-TADF emitters (Section 11) makes them incompatible with TD-DFT approaches, as we have recently demonstrated (*vide infra*).^137,138^

In the literature, vertical excitation calculations based on the optimized ground-state geometry are most frequently reported, and the vertical Δ*E*_ST_ is computed from the difference in vertical excitation energies to S_1_ and T_1_ using TD-DFT methods (Figure 13).^139^ These calculations are particularly cost-effective as they do not require excited-state geometry optimisations, and essentially mimic an absorption process; however, they are often misguidedly used to then interpret the emission properties of TADF materials. When investigating emission properties, it is instead recommended to commit to optimization of the geometry in the excited states, with S_1_ and T_1_ optimizations used to model fluorescence phosphorescence spectra, respectively, since molecular relaxation occurs at much faster timescales (ps and faster) than these radiative processes and thus originating from the relaxed geometry of the excited state. However, this is a more computationally costly approach as each excited state of interest must be reoptimized separately, and as such, this approach is less frequently employed.

**Figure 13:**
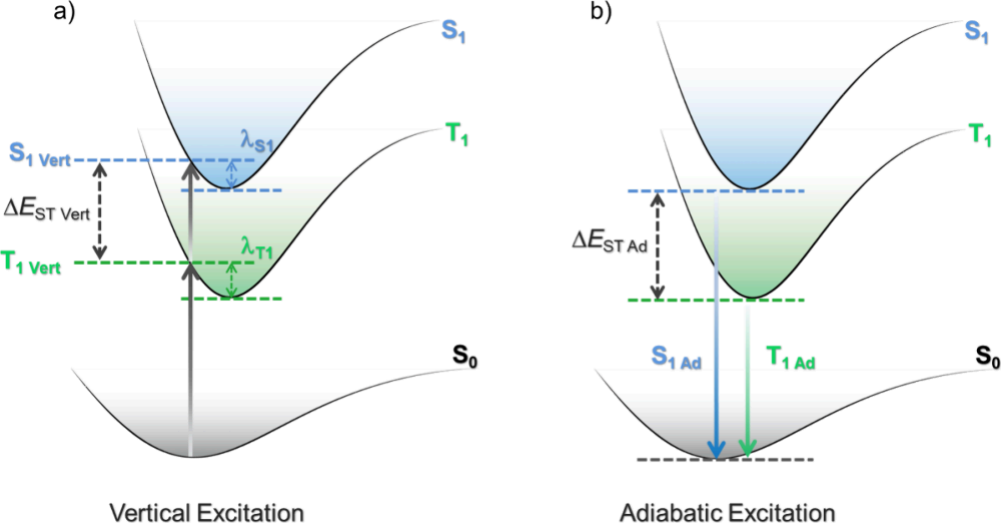
Simplified representation of the calculations of a) vertical and b) adiabatic Δ*E*_ST_, where λ_S1_ and λ_T1_ are the S_1_ and T_1_ relaxation energies, respectively.

However, even this more careful approach can fail when excited states are close in energy, and start to acquire a multiconfigurational character which cannot be captured by TD-DFT. In these cases, one must rely instead on appropriate wavefunction-based methodologies such as the complete active space self-consistent field (CASSCF).^140^ The adiabatic excitation energy corresponds to the difference in energy between the optimized (relaxed) ground and excited states (Figure 13).^139^ Thus, the adiabatic Δ*E*_ST_ is determined from the difference in energy between the adiabatic S_1_ and T_1_ excitation energies. Although the adiabatic Δ*E*_ST_ is more closely related to the measured Δ*E*_ST_, it has been highlighted that the vertical Δ*E*_ST_ and the adiabatic Δ*E*_ST_ can provide similar results when both S_1_ and T_1_ share a similar electronic configuration, (*i.e.* the same nature) resulting in similar relaxation energies.^139^ This is often the case for D-A TADF compounds which frequently possess S_1_ and T_1_ excited states with a strong CT character, with the modest accuracy and lower cost of vertical Δ*E*_ST_ explaining its persistent use.

Faced with a plurality of potential computational methods, the preferred choice for characterizing TADF emitters is almost entirely dependent on whether the D-A TADF or MR-TADF D-A TADF materials are capably described using TD-DFT, especially within the Tamm–Dancoff approximation (TDA) that relaxes the triplet instability issue, which results in an over-stabilized T_1_ with respect to S_1_.^141,142^ The modelling of MR-TADF compounds requires the use of wavefunction-based methods through either single-reference couple-cluster methods or multi-reference protocols such as CASSCF/CASPT2 (Complete active space 2^nd^ order perturbation theory) or CASSCF/NEVTP2 (n-Electron Valence 2^nd^-order Perturbation Theory) to improve the description of the S_1_ state with a proper inclusion of (dynamic) electron correlation (which is not as important for T_1_).

#### 2.2.2 Characterizing the Nature of the Excited States

As highlighted in the introduction, alongside energies, the nature of the excited states and the resulting spin-orbit coupling between them (governed by El-Sayed’s rule) are critical in order to infer the mechanism of a particular TADF process.^83^ Although the nature of these states is still often discussed in terms of the electron density distribution of the HOMO and LUMO (Figure 14), this can be misleading, as excited states can display contributions from more than the simple one-electron transition.^143^ More compact pictures of excited states such as natural transition orbitals (NTOs), attachment/detachment frameworks, or the difference in electron density between the ground and excited state are becoming increasingly popular in order to more accurately characterize the nature of the excited states (Figure 14).^127^ These methods each portray the spatial distribution or changes of the hole and electron densities for the singlet and triplet excited states, thus accounting for contributions of all relevant orbitals.^134^

**Figure 14:**
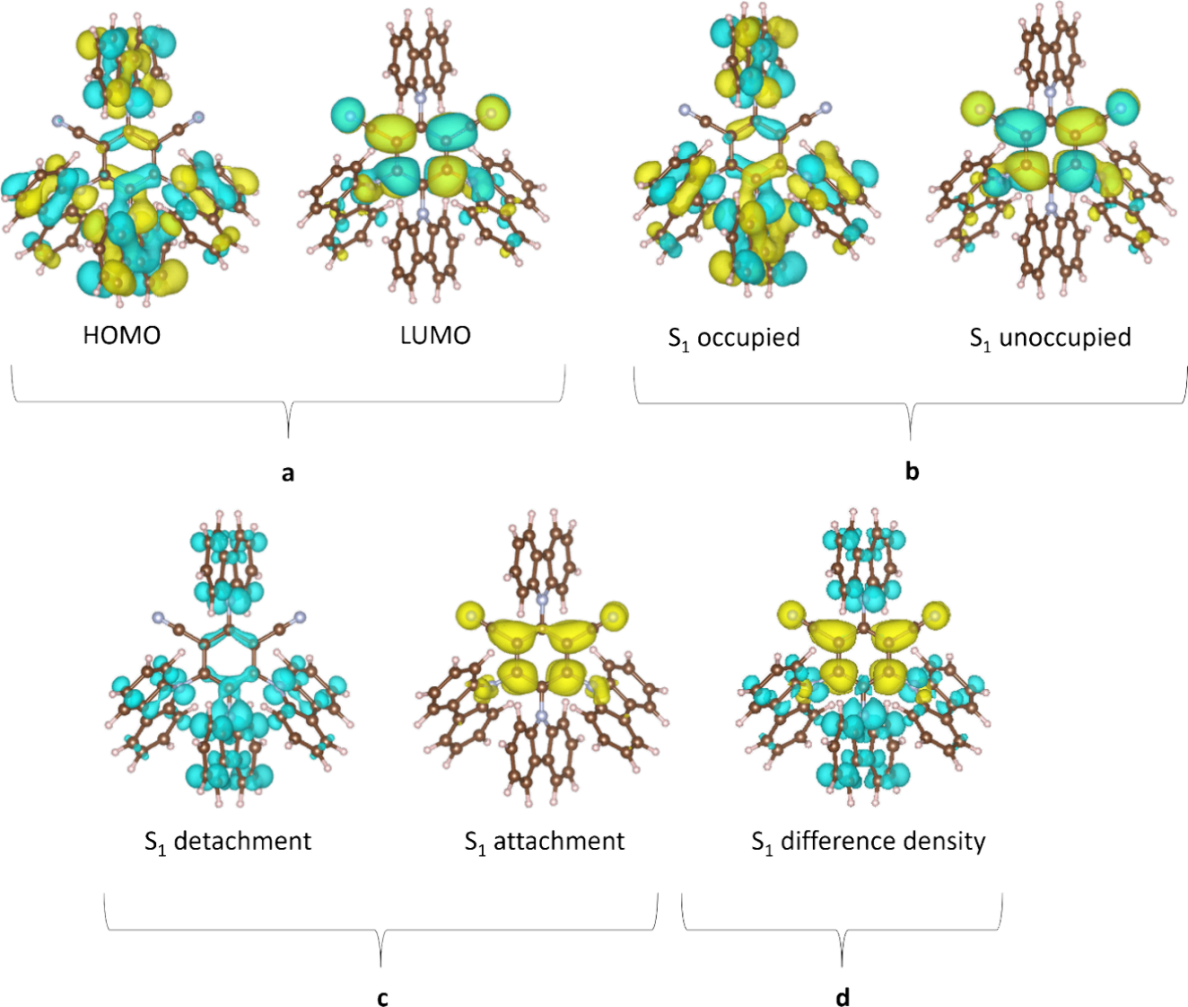
DFT calculations of **4CzIPN**: a) HOMO-LUMO, b) NTO corresponding to the S_1_-S_0_ transition, c) attachment/detachment densities associated with S_1_, and d) difference density S_1_-S_0_ plots at the TDA-PBE0/6-31G(d,p) level. Isovalue = 0.02 for a and b, 0.001 for c and d. The nature of the emissive S_1_ state is long-range charge transfer (LRCT).

The nature of the excited states can in some cases be qualitatively inferred by inspection of orbital visualisations but more quantitatively by computing metrics relying on the difference in distance between the hole and electron density barycenters, such as D_CT_.^144^ Alternatively, overlap indices include Λ, which describes the overlap between pairs of (NTO) orbitals,^145,146^ or Φ_S_, which is a measure of the overlap between the hole and the electron densities computed within the attachment/detachment formalism. Typically, CT (or LE) excited states exhibit large (or small) D_CT_ beyond (below) 1.6 Å or small (large) Λ/Φ_S_ of around 0 (1).^85,147^ Several studies have highlighted clearly that the nature of the excited state in D-A TADF emitters is never purely LE or CT, but a mixture of both.^85^ In a recent effort, we identified that for D-A TADFs the nature of each of S_1_, T_1_, and T_2_ is well-reproduced when TDA-CAM-B3LYP or TDA-M06-2X functionals are used, when compared to Spin Component Scaling Second order Couple Cluster (SCS-CC2) calculations.^148,149^ However, irrespective of the functional, the nature of both T_1_ and T_2_ computed at the TDA level does not match as accurately with the SCS-CC2 prediction as it does for the S_1_ state.

In contrast, for MR-TADF compounds, the lower-lying singlet and triplet excited states consistently exhibit a short-range charge transfer-like (SRCT) character with a difference density pattern showing excess hole and electron densities on adjacent atomic sites (Figure 15 with **DABNA-1**).^137,138^ While this does not exclude the possibility of higher-lying excited states with LE or a long-range CT-like (LRCT) character (for example triggered by the presence of peripheral substituents possessing a significant electron-donating or -withdrawing character)^150^ as mentioned above these SRCT require alternate computational treatment to account for an accurate account of electron correlation. For MR-TADF emitters, difference density plots provide the clearest picture of the alternating hole-electron density pattern present within their excited states.^138^

**Figure 15:**
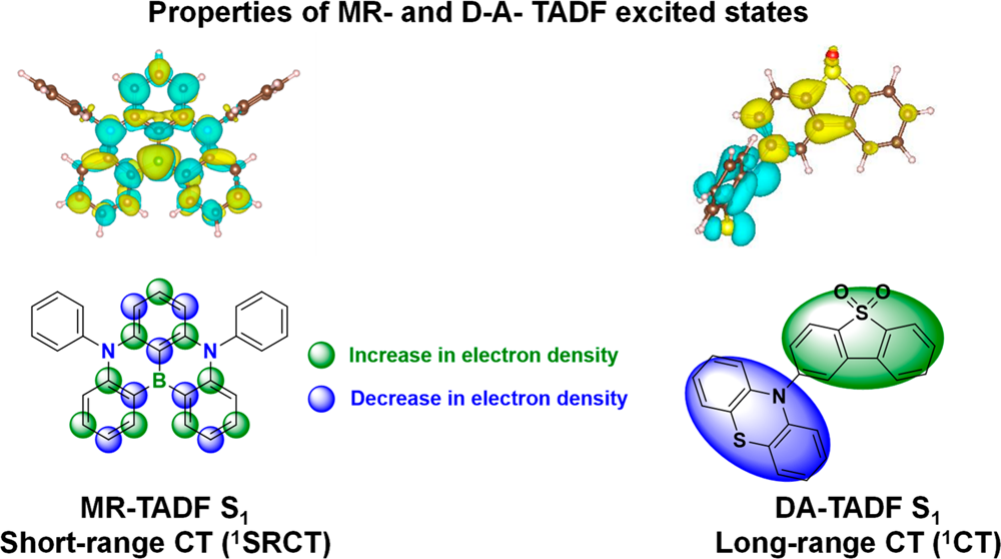
Excited-state difference density plot of a MR-TADF (**DABNA-1**) emitter (left) and D-A TADF (**PTZ-DBTO2**) emitter (right). Taken and adapted with permission from ref 138. Copyright [2022/Journal of Chemical Theory and Computation] American Chemical Society.

The relationship between the extent of CT character of the lower-lying singlet and triplet excited states and Δ*E*_ST_ has been probed using several metrics. Work by Moral *et al*.^151^ demonstrated, considering six molecules (three hosts and three emitters), that a larger CT character of both S_1_ and T_1_ states results in a reduced Δ*E*_ST_, in line with equations 3 and 4 (see Section **1.4.1**). Their analysis was based on the calculation of the Δr metric, namely the distance between the hole and electron density barycenters as computed from NTOs to quantify the extent of CT in the compounds. Lee and Kim^152^ investigated the influence of donor substitution and showed that the inclusions of additional donors increase the CT content of T_1_ while having a minimal effect on S_1_, which in turn reduce Δ*E*_ST_. Along this line, Olivier *et al*. showed in a series of D-A and D-A-D emitters that the biggest challenge to decreasing Δ*E*_ST_ consists of increasing the CT character of the T_1_.^153^ This is exemplified when computing the Δ*E*_ST_, the Φ_S_ metric for S_1_ and T_1_ and oscillator strength along the torsional profiles of the D-A single bond. Typically, we observed a faster decrease (increase) of the T_1_ as compared to S_1_ CT (LE) character. It arises because the T_1_ state is subject to the exchange interaction (while not the S_1_ state), which induces a localization of the T_1_ wavefunction on either the D or A units and significantly increases the LE character of that state. Inducing a larger CT character in T_1_ results in a larger energy difference between the ^3^LE and ^3^CT as compared to the energy difference between the ^1^LE and ^1^CT for the corresponding S_1_ state. A similar study, undertaken by us,^123^ where we performed a torsional screen of four emitters, supports this finding. Here, Δ*E*_ST_ is smallest when the CT content of S_1_ and T_1_ is greatest. By observing the difference in CT character between S_1_ and T_1_ Φ_S_, (ΔΦ_S_), Olivier *et al*. reported a decreasing Δ*E*_ST_ when ΔΦ_S_ is the smallest, namely when the CT character in both states is the largest.^85^ These studies show that it is easier to induce a larger CT character in S_1_ state than in T_1_. However, reaching a large CT character in both states is key to minimizing Δ*E*_ST_ and it has been the most popular design strategy thus far. In rare exceptions, Δ*E*_ST_ was made rather small (below 0.1 eV) even though T_1_ and S_1_ bore, respectively, an LE and a large CT character, but this requires a careful engineering of the energies of the ^3^LE and ^3^CT states.^154^

### 2.3 Benchmarking Δ*E*_ST_

#### 2.3.1 D-A TADF Emitters

The variety of DFT functionals are essentially distinguished by the way the exchange-correlation potential is defined, and this also introduces a significant disparity between the excited-state pictures associated with D-A TADF emitters. The most popular functionals employed to model TADF emitters are usually global hybrids such as B3LYP and PBE0, meta-GGA (Generalized Gradient Approximation) such as M06-2X, and long-range corrected functionals such as CAM-B3LYP and LC-ωPBE. Basis set effects can also lead to a further variation of the absolute energies of the excited states. However, no dramatic variation in relative energy has been observed going from Pople-based basis sets such as 6-31G(d,p) to Karlsruhe basis sets such as def2-TZVP.^153^

In the computational chemistry literature, consensus on different methodologies is often approached (although rarely fully achieved) by identifying the most accurate methodologies across a group of known compounds.^139,142,155,156^ These benchmark studies involve comparison between a calculated property of interest at a level of theory under assessment (*y*_*i*_^calc^) with the corresponding experimental or ‘trusted’ higher-level method property (*y*_*i*_^ref^). The mean average difference (MAD), the root mean square deviation (RMSD) and the standard deviation (σ) each allow for the determination of the most appropriate methodology based on a statistical analysis.

12 $$\mathrm{MAD}=\frac{1}{n}\sum_{a=1}^{n}\left|x_{i}\right|$$

13 $$\mathrm{RMSD}=\sqrt{\frac{1}{n}\sum_{i=1}^{n}{\left|x_{i}\right|}^{2}}$$

14 $$\sigma =\sqrt{(\frac{1}{n}\sum_{i=1}^{n}{\left|x_{i}\right|}^{2})-{\left(\frac{1}{n}\sum_{i=1}^{n}\left|x_{i}\right|\right)}^{2}}$$

15 $$x=\left|y_{i}^{calc}-y_{i}^{ref}\right|$$

The accuracy of the selected method(s) with respect to a test data set is assessed by these three metrics, with smaller values corresponding to better performance, although with no consideration for different computational costs.

Moral *et al*. compared several TD-DFT approaches against experimental data and highlighted that using TDA-DFT compared to TD-DFT produced a more accurate Δ*E*_ST_ prediction, essentially because the triplet instability issue was better handled.^142^ A study using a larger data set of 17 emitters was undertaken by Sun *et al.*,^139^ where M06-2X and ω-tuned LC-ωPBE showed excellent agreement between calculated and experimental Δ*E*_ST_ for both vertical and adiabatic excitations. In ω-tuned LC-ωPBE, the electron repulsion operator is divided into a short-range description at the DFT level and a long-range domain described at the Hartree-Fock level.^133^ The range separation, ω, delimits the two domains and is often optimized to tune the HOMO and LUMO energies to the ionization potential and the electron affinity, respectively. However, this parameter must be optimized for every compound and potentially for each different starting geometry.^157^ Using a similar ω optimisation procedure, other long-range corrected functionals such as LC-B3LYP^158^ or LC-ωHPBE have been employed within the literature, differing only in their DFT exchange-correlation potential.

Moving away from TD-DFT, Kunze *et al*. employed spin-unrestricted (UKS) and restricted open-shell Kohn-Sham (ROKS) SCF calculations to investigate 32 emitters, covering a range of structures.^155^ Their study showed a remarkably small MAD for predicted Δ*E*_ST_ of 0.025 eV. This impressive accuracy was assigned to an improved CT description owing to the inclusion of orbital relaxation, which other computational schemes based on TD-DFT do not include. However, excited state transition properties are not accessible with ROKS, and TD-DFT should be invoked to access them.

The community primarily still uses hybrid functionals like B3LYP and PBE0, despite the conclusions from these benchmark studies that have highlighted that both produce excessive stabilization of CT states due to their low Hartree-Fock exchange content.^139,155^

#### 2.3.2 MR-TADF Emitters

Unlike the modelling of the excited states of D-A TADF compounds, TD-DFT struggles to accurately predict the excited states of MR-TADF emitters,^137,138^ where there is a consistent overestimation of the Δ*E*_ST_. Despite documenting the inaccurate prediction of the excited-state energies, the community continues to employ TD-DFT methods to model MR-TADF emitters. Recently, we highlighted that coupled cluster calculations can accurately predict the nature and energies of the excited states of MR-TADF compounds as these calculations include a double excitation contribution that current implementation of linear response TD-DFT neglects in the adiabatic approximation.^25,137,138^ We anticipate that the use of TD-DFT will be rapidly superseded by wavefunction-based methods as new MR-TADF materials continue to be reported at an accelerating pace.

This was initially demonstrated for two emitters for **DABNA-1** and **TABNA**, where excellent agreement was obtained between experimental and calculated Δ*E*_ST_ values (Figure 16).^137^ Using this method, a series of linearly extended systems were also designed, and it was demonstrated that increasing the length produced an increase in *f* and a reduced Δ*E*_ST_. However, this came at the price of a predicted red-shift of the emission. However, suitable substitutions with nitrogen and/or boron atoms enables either a blue- or a red-shift of the emission energy. Ultimately, increased charge transfer character and a reduced CT distance ensured a reduced exchange energy in these compounds, resulting in smaller Δ*E*_ST_, and stronger polarizability, leading to a larger *f*.

**Figure 16:**
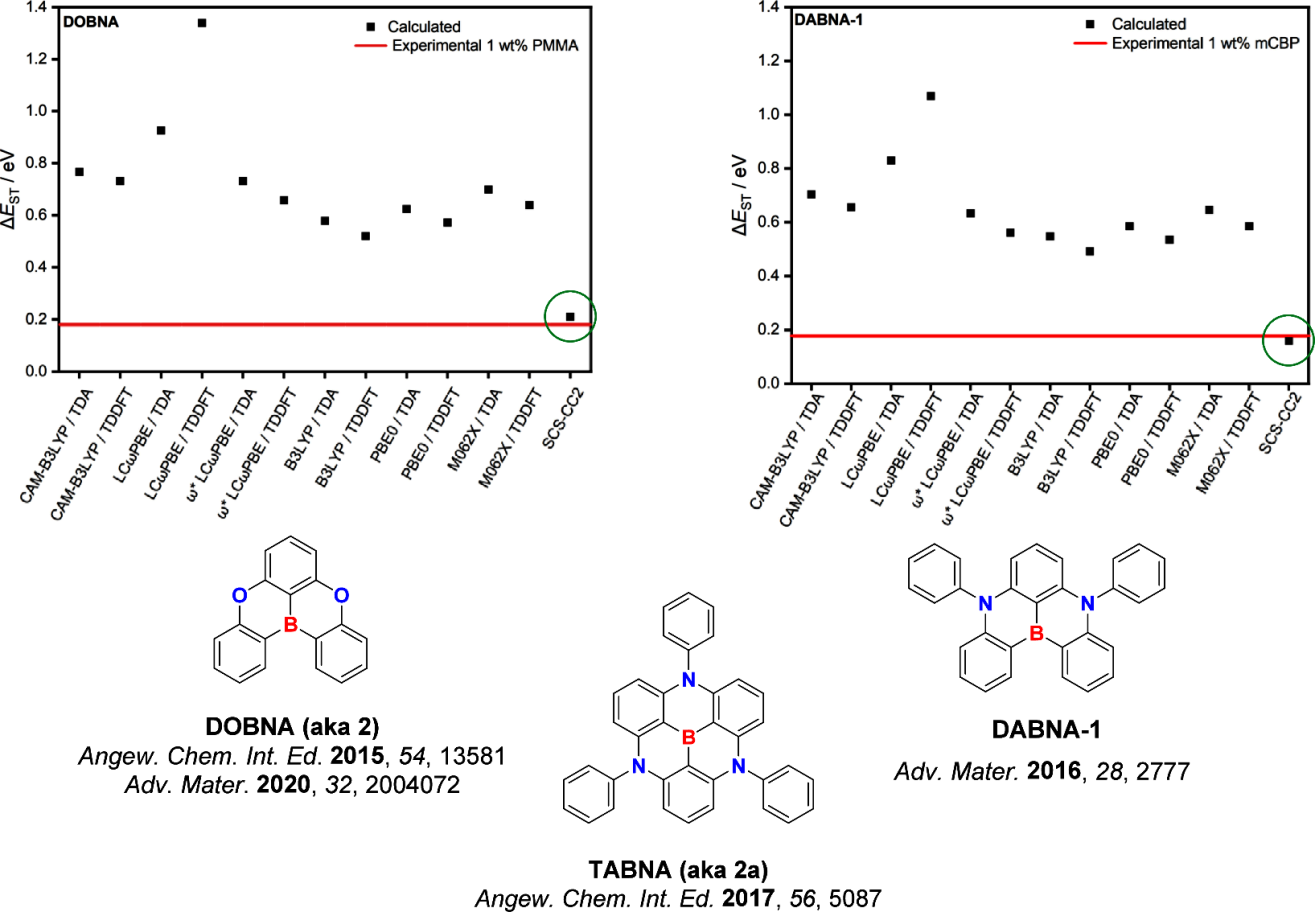
Calculated vs experimental Δ*E*_ST_ data of **DOBNA** (left) and **DABNA-1** (right). The Δ*E*_ST_ values from the coupled cluster calculations (SCS-CCS) are circled in green. The structure of **TABNA**, a model MR-TADF compound used in this study, is also displayed. Figure taken and adapted with permission from ref 45. Copyright [2020/Advanced Functional Materials] John Wiley & Sons under Creative Commons Attribution 4.0 International License https://creativecommons.org/licenses/by/4.0/.

We also recently compared the experimentally and calculated Δ*E*_ST_ values across 35 literature MR-TADF emitters using SCS-CC2/cc-pVDZ as well as a range of DFT functionals (B3LYP, PBE0, M062X, LC-ωPBE, CAM-B3LYP and ω-tuned LC-ωPBE).^138^ TD(A)-DFT calculations consistently overestimated Δ*E*_ST_, with MAD values ranging between 0.29 eV and 0.98 eV. When employing SCS-CC2/cc-pVDZ, the MAD significantly decreases to only 0.04 eV, highlighting the ability of this method to accurately predict the Δ*E*_ST_ (see Figure 16). When considering exclusively boron-acceptor MR-TADF emitters, a strong correlation becomes apparent between SCS-CC2 and the experimental T_1_ and S_1_ energies. However, the correlation between calculated and experimental Δ*E*_ST_ of ketone-acceptor MR-TADF derivatives is not as strong, Figure 17. This was attributed both to the fact that vertical Δ*E*_ST_ were considered, thus neglecting excited-state relaxation, and to solvent interactions with the lone pair of the carbonyl functionalities that result in a stabilization of the excited states of this class of MR-TADF emitters. Since the original report, this methodology has enjoyed wide and growing implementation in materials design.^55,150,159–180^

**Figure 17:**
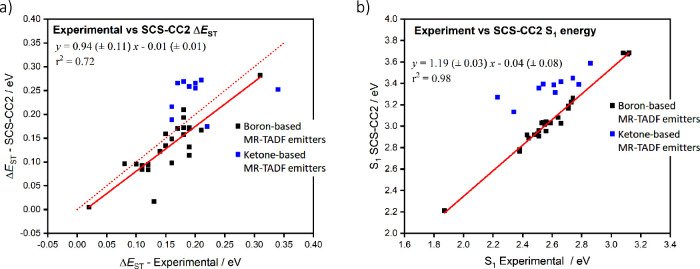
Plots of experimental vs calculated a) Δ*E*_ST_ and b) S_1_ energies of modelled MR-TADF emitters at the SCS-CC2/cc-pVDZ level. Taken and adapted with permission from ref 138. Copyright [2022/Journal of Chemical Theory and Computation] American Chemical Society.

Recently, Sotoyama reported an alternative strategy for the accurate prediction of the Δ*E*_ST_ of MR-TADF emitters using ΔSCF.^181^ ΔSCF calculations involved two SCF calculations; a first one where an electron with either a spin-up or spin-down is promoted from the occupied to the virtual orbitals. This results in a state halfway between a singlet and a triplet spin configuration, leading to some spin contamination from the triplet state configuration. A second SCF calculation is then performed on the triplet state configuration. The energy of the singlet state is thus obtained as the difference between twice the energy of the first calculation (emulating the singlet state, and doubled to account for the degeneracy of the spin up and spin down electronic configuration) and the energy of the triplet state. The correlation between ΔSCF-calculated versus experimental Δ*E*_ST_ was investigated across 13 MR-TADF emitters and compared to conventional the results from TD-DFT methodologies. Here, MADs of 0.04 eV using both the B3LYP and the PBE0 functionals using ΔSCF were reported, performing similarly to SCS-CC2 calculations^138^ although at notably reduced computational cost.^181^ The author attributed the accurate prediction of Δ*E*_ST_ to the orbital relaxation rather than the inclusion of double excitation.

Recently, a benchmark study of both D-A TADF and MR-TADF emitters was performed using the hole-hole (h-h) TDA-DFT method.^182^ This method includes static electronic correlation in a similar fashion to CASSCF, considering electronic transitions within an active space including only single and double excitations while dynamic correlation is introduced through the exchange-correlation functional. Interestingly, these calculations revealed a very good agreement with SCS-CC2 calculations yet at a much cheaper computational cost, with a MAD of 0.04 eV for Δ*E*_ST_ predictions using the h-h TDA-B3LYP method.

### 2.4 Higher-Lying Triplet States

Understanding the role of higher-lying triplet states is becoming increasingly crucial in explanation otherwise anomalously fast *k*_RISC_ observed in some TADF materials. Spectroscopically, these states are difficult to observe since they are either a ‘dark state’ or internal conversion to lower-lying excited states outcompetes any radiative decay. Their existence can be detected indirectly using transient absorption spectroscopy (TAS) methods, where their own photoinduced absorption (PIA) features emerge either though T_1_-T_n_ or T_n_-T_m_ transitions. However, TAS often requires extensive excited-state calculations to model the triplet absorption spectrum from the optimized T_1_ or higher T_n_ states. Therefore, in many of the examples where fast *k*_RISC_ is reported experimentally, the role of an intermediate upper triplet state has been either asserted speculatively or inferred solely from calculations (Figure 18).^92,98,183^ As an example, among D-A TADF emitters, the fast *k*_RISC_ of **TAT-3DBTO2** was rationalized using TDA-DFT calculations that predicted the existence of 12 higher-lying triplet states within 0.2 eV of T_1_. This high density of triplet states within a small energy window was proposed to favor fast RISC.^92^ A similar conclusion was obtained also in the study of **MCz-TXT**, which has one of the fastest reported RISC to date.^183^ TD-DFT was also used to explain the very fast *k*_RISC_ in **TpAT-tFFO_,_** where a subtle alignment of ^3^CT and ^1^CT as well as a higher-lying ^3^LE state was proposed to explain the efficient TADF in this compound.^98^ This is further confirmed by constructing linearly interpolated potential energy profiles (LIPs) between target singlet and triplet states minimum showing potential multiple conical intersections between closely-lying singlet and triplet excited states.^184^ Closely-lying singlet and triplet excited states has often been used to identify compounds emitting from a Hot Exciton mechanism in the device, where close alignment of a higher-lying T_n+1_ with either S_1_ or S_2_ is implicated to explain the origins of the RISC.^185^

**Figure 18:**
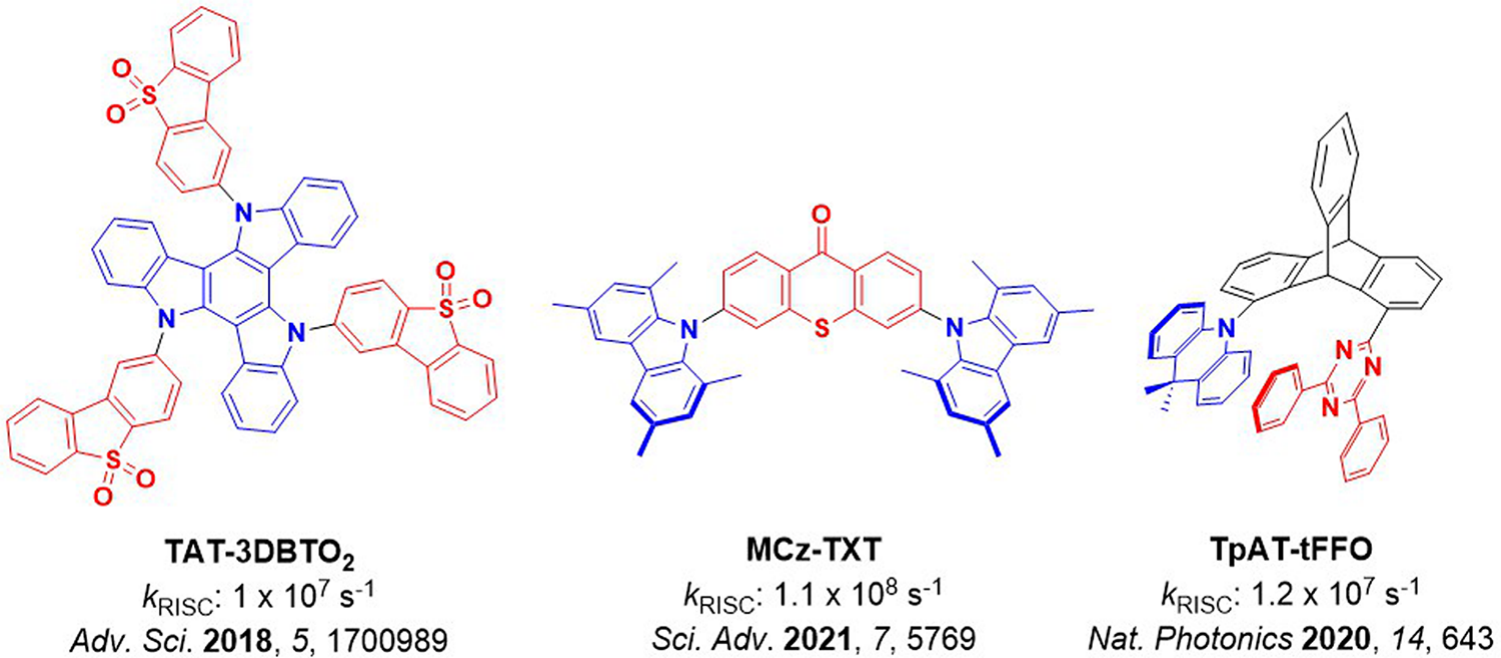
Structures of three D-A TADF emitters with fast *k*_RISC_ involving higher-lying T_n_ states (the blue color signifies donor moieties, while the red color signifies acceptor moieties).

**Figure 19:**
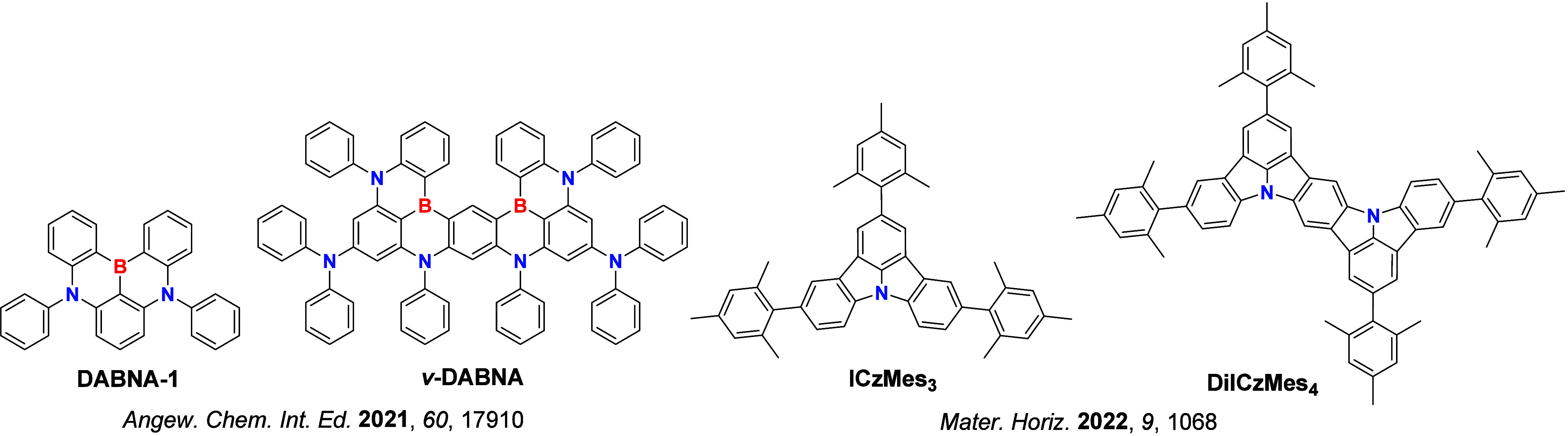
Structures of MR-TADF compounds, where the structure of the emitters **DABNA-1** and **ICzMes_3_** have been expanded in the emitters **v-DABNA** and **DiICzMes_4_** containing a decreased T_1_-T_2_ gap (the blue color signifies donor atoms, while the red color signifies acceptor atoms).

Further establishing useful methods to investigate higher-lying triplet states, a benchmark computational study of 10 different D-A TADF emitters was undertaken by Cardeynaels *et al.*,^156^ which compared DFT functionals and higher-level CC2 calculations. The study revealed that of the DFT functionals investigated, M06-2X provided the smallest MAD for the absolute energy of T_2_ with respect to the CC2 calculations. Our recent investigation of 14 chemically diverse D-A TADF emitters supports these previous results,^147^ demonstrating that both M06-2X and CAM-B3LYP perform as well as ω-tuned functionals LCωPBE and LCωHPBE, all relative to SCS-CC2 calculations.

Focussing on MR-TADF emitters, single triangulene core structures usually possess a large energy gap between T_1_ and T_2_ as exemplified in **DABNA-1** and **DiKTa**, which appears to hinder upconversion from T_1_ to T_2_.^138^ As discussed in the section on **MR-TADF Emitters**, Section 11, expansion of the size of the MR-TADF skeleton is seen as a promising strategy to simultaneously improve *f* while decreasing Δ*E*_ST_. This was confirmed experimentally when comparing **DABNA-1** and **ν-DABNA** (Figure 19). In addition, there is a shrinking of the T_1_-T_2_ gap from 0.75 eV to 0.12 eV from **DABNA-1** to **ν-DABNA**, computed at SCS-CC2 level based on the ground-state optimized geometry, leading to a significant boost in *k*_RISC_ from 10^3^ s^–1^ to 10^5^ s^–1^.^173^ This behavior is also observed to a lesser extent when comparing **ICzMes_3_** and **DiICzMes_4_**, Figure 19, where the T_1_-T_2_ gap decreases from 0.46 eV to 0.33 eV using the same method.^161^

### 2.5 Spin-Orbit Coupling and RISC

SOC can be considered as the dominant source for spin mixing, driving the triplet-singlet interconversion in D-A TADF and MR-TADF materials and thus crucial for computing *k*_RISC_. When considering RISC as an intramolecular process, SOC is often computed to be on the order of a few tenths of meV, while hyperfine interaction (HFI) and spin-spin dipolar coupling are calculated to be much smaller, on the order of a few μeV, and hence can be neglected.^90^ The magnitude of the computed SOC is also very important, as El-Sayed’s rule only qualitatively establishes that SOC vanishes between singlet and triplet excited states of the same nature, while remaining sizeable between excited states of different character.^81,82^ The SOC is never exactly zero though, as triplets rarely exhibit perfectly identical nature as their corresponding singlets, as the electronic exchange interaction acts more strongly on triplets than singlets, conferring a higher degree of LE character.

This is not to say that SOC is exclusively the dominant factor in RISC. In bimolecular TADF exciplexes, it is often hypothesized that RISC must be driven by hyperfine interactions as the singlet and triplet excited states are intermolecular states possess a strong CT character between electron-donating and accepting moieties that are not covalently linked and with vanishing hole and electron densities overlap.^186^ Recently, a combination of TAS, transient electron paramagnetic (TrEPR) measurements, and TD-DFT calculations showed that **BF2** (Figure 20), a curcuminoid derivative, possesses delocalized intermolecular CT states from which intermolecular RISC is driven by a hyperfine intermolecular process.^187^ While this intermolecular RISC mechanism is not the most common spin interconversion mechanism, it should not be neglected outright, especially when devices exhibit high EQE despite the large Δ*E*_ST_ of the emitter.

**Figure 20:**
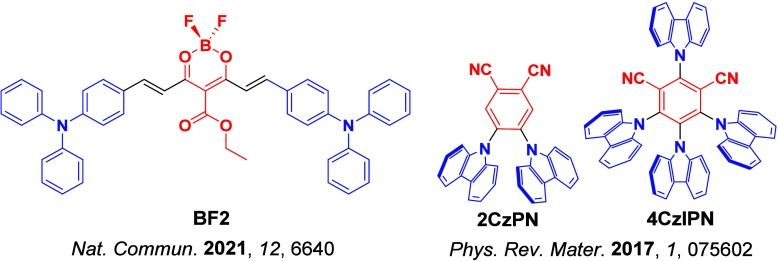
Structures of three D-A TADF systems where *k*_RISC_ and SOC were investigated computationally (the blue color signifies donor moieties, while the red color signifies acceptor moieties).

**Figure 21:**
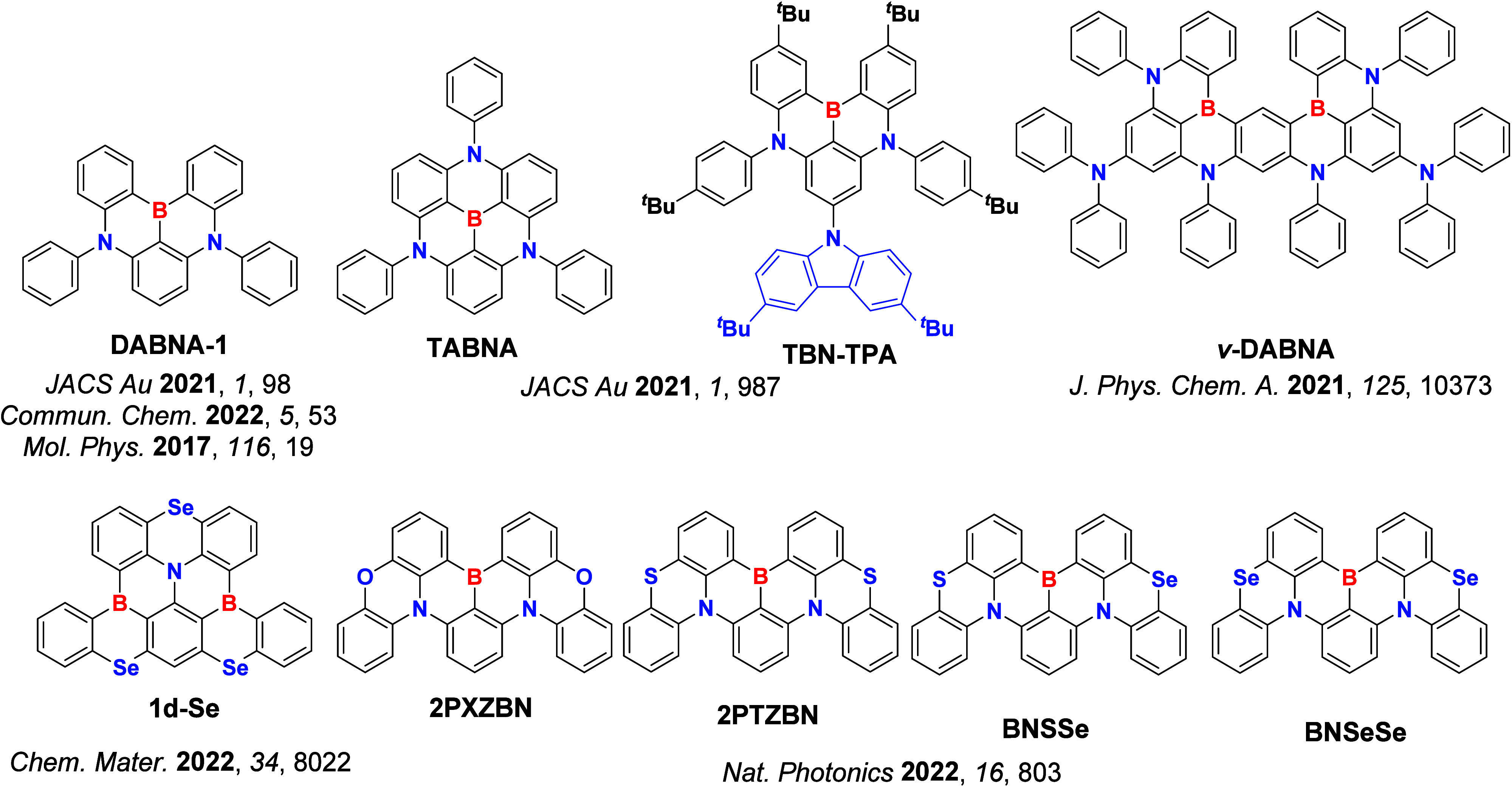
MR-TADF emitters for which SOC and *k*_RISC_ have been investigated computationally (the blue color signifies donor atoms/functional groups, while the red color signifies acceptor atoms/functional groups).

Returning to the intramolecular RISC mechanisms as operant in D-A TADF emitters, computational modelling of **2CzPN** and **4CzIPN** (Figure 20) revealed that the increase of SOC between S_1_ and T_1_ occurs at the expense of an increase of Δ*E*_ST_.^85^ In line with El-Sayed’s rule, the SOC between S_1_ and T_1_ in these compounds increases as the natures of S_1_ and T_1_ diverge, and is strictly zero when S_1_ and T_1_ are pure CT. The mixed CT-LE nature of S_1_ and T_1_ in both **2CzPN** and **4CzIPN** is dynamically modulated by fluctuations of the torsion angle between the donor and the acceptor, as probed along a molecular dynamics simulation, which impacts the D-A electronic interaction and instantaneous SOC value. This finding was further confirmed by a study of 15 D-A TADF emitters in which Marcus-type rate expressions for *k*_RISC_ indicated a careful balance is needed between Δ*E*_ST_ and SOC to ensure fast *k*_RISC_.^154^ When the nature of S_1_ and T_1_ are significantly different, Δ*E*_ST_ is very large and RISC is slow regardless of the value of SOC because Δ*E*_ST_ appears in the exponential factor of the Marcus rate expression. However, while Δ*E*_ST_ decreases as S_1_ and T_1_ take on increasingly similar natures, the reduced contribution from SOC becomes increasingly prominent in the overall *k*_RISC_. Although identifying candidate emitters by finding an optimum balance of SOC and Δ*E*_ST_ seems promising, large variations in both SOC and Δ*E*_ST_ appear when changing functionals, hence comparison between calculations and experiments must be made with caution.

While the RISC rates for leading D-A TADFs now often exceed 10^6^ s^–1^, the experimental *k*_RISC_ values of MR-TADF emitters are significantly slower, on the order of 10^3^–10^4^ s^–1^ (see Section 11 for more detail). The direct SOC between T_1_ and S_1_ in MR-TADFs is often quite small due to the very similar nature and electronic configuration of both SRCT states. Instead, a recent computational study carried out on **DABNA-1**, **TABNA**, and **TBN-TPA** (Figure 21) revealed that a superexchange mechanism should drive RISC involving primarily the much higher-lying T_3_ states.^188^ Shizu *et al*. instead reported that RISC in **DABNA-1** is actually a two-step process entailing RIC from T_1_ to T_2_ followed by efficient RISC from T_2_ to S_1_.^189,190^ Lin *et al*. also tried to rationalize the **DABNA-1** excited-state dynamics by computing the RISC rate between the first three triplets and S_1_, and concluded instead that the T_1_-S_1_ channel is the most likely route.^190^ These three independent studies, involved three different computational protocols and resulting in very different pictures of the detailed RISC mechanism of the same molecule, certainly needs further clarification.

An extended analogue of **DABNA-1**, the *k*_RISC_ mechanism was simulated for ***v*-DABNA** (Figure 21), achieving good agreement between calculated and experimental values when considering a direct T_1_ to S_1_ conversion.^181^ Despite the small SOC between these states, a very small Δ*E*_ST_ was believed to be sufficient to account for the efficient *k*_RISC_ reported, while the computed *k*_RISC_ assuming a T_2_-mediated RISC mechanism leads to a difference in magnitude compared with experimental findings.

The role of heavy atom effects in modulating *k*_RISC_ in MR-TADF emitters was recently discussed by Pratik *et al*. In their two studies, they investigated the role of embedding heavy atoms into a MR-TADF structure using the DLPNOCCSD/def2-TZVP method.^178,191^ They focused on chalcogen elements of group 8, probing oxygen, sulfur, and selenium derivatives and the resulting changes in SOC and *k*_RISC_. A range of emitters was presented, with SOC and in turn *k*_RISC_ increasing significantly in selenium-containing materials, compared to oxygen and sulfur congeners; **1d-Se** was proposed as a particularly promising emitter wherein *k*_RISC_ was predicted to outcompete *k*_r_ (Figure 21). Simply considering SOC, Hu *et al*. have computationally investigated changing *k*_RISC_ for a similar series of compounds comparing their calculated SOC with the experimental *k*_RISC_.^180^ They observed a steady increase in experimental *k*_RISC_ from 0.04 × 10^6^ s^–1^ (**2PXZBN**) and 0.19 × 10^6^ s^–1^ (**2PTZBN**) to 0.60 × 10^6^ s^–1^ (**BNSSe**) and 2.0 × 10^6^ s^–1^ (**BNSeSe**), that is broadly in-line with their calculated T_1_ – S_1_ SOC values (Figure 21). **BNSeSe** was calculated to have the most active RISC, which they attributed to a larger SOC; two orders of magnitude larger for S_1_-T_1_ in **BNSeSe** than for **2PXZBN** and **2PTZBN**. The S_1_ – T_2_ SOC was calculated to be largest for **BNSeSe**, which explains the faster *k*_RISC_ than for **BNSSe**, which actually has a larger S_1_ – T_1_ SOC. In accordance with these computational studies, and as with D-A TADF systems, introduction of the heavy atom has proved an attractive strategy to increase *k*_RISC_ in MR-TADF emitters in experimental studies.^103^

### 2.6 Conformational and Vibronic Effects

It is important to note that the approaches mentioned so far primarily involve isolated molecules in either their ground or excited-state optimized geometry. In the OLED, this is clearly not an accurate representation of the emitting layer (EML). The amorphous nature of the EML means that different conformers of the TADF emitters will be present, while simultaneously intramolecular vibrational modes will modulate their geometries over time.

In 2017, Olivier *et al.* ^153^ identified the critical role of torsional vibrational modes around the donor-acceptor single bond in D-A and linear D-A-D TADF emitters, impacting both the energies of the S_1_ and T_1_ excited states (and so Δ*E*_ST_) and the oscillator strength of the S_0_-S_1_ transition. This rather simple approach helped to resolve the apparent contradiction of how a TADF emitter with seemingly negligible S_0_-S_1_ oscillator strength (because of the large CT character of the S_1_ state) may nonetheless possess decent radiative lifetime and so a high Φ_PL_. The role of vibrations within TADF systems is indeed becoming recognized as ever more important as our understanding develops. In 2016, Marian discussed a mechanism involving an intramolecular vibrational mode of the C=O bond of the xanthen-9-one donor in **ACRXTN** to justify the promising RISC rate of the emitter (Figure 22).^192^ Olivier *et al*. showed that direct T_1_-S_1_ *k*_RISC_ was significantly boosted due to non-Condon effects on the SOC – i.e. the impact of geometry distortions associated with active vibrational modes on the SOC.^85^ Brédas and co-workers investigated carbazole-based TADF emitters and highlighted that the rotation of the carbazole group in between the two cyano groups in **4CzIPN** (Figure 22) triggered the RIC from T_1_ to T_2_ from which RISC was computed to take place.^193^

**Figure 22:**
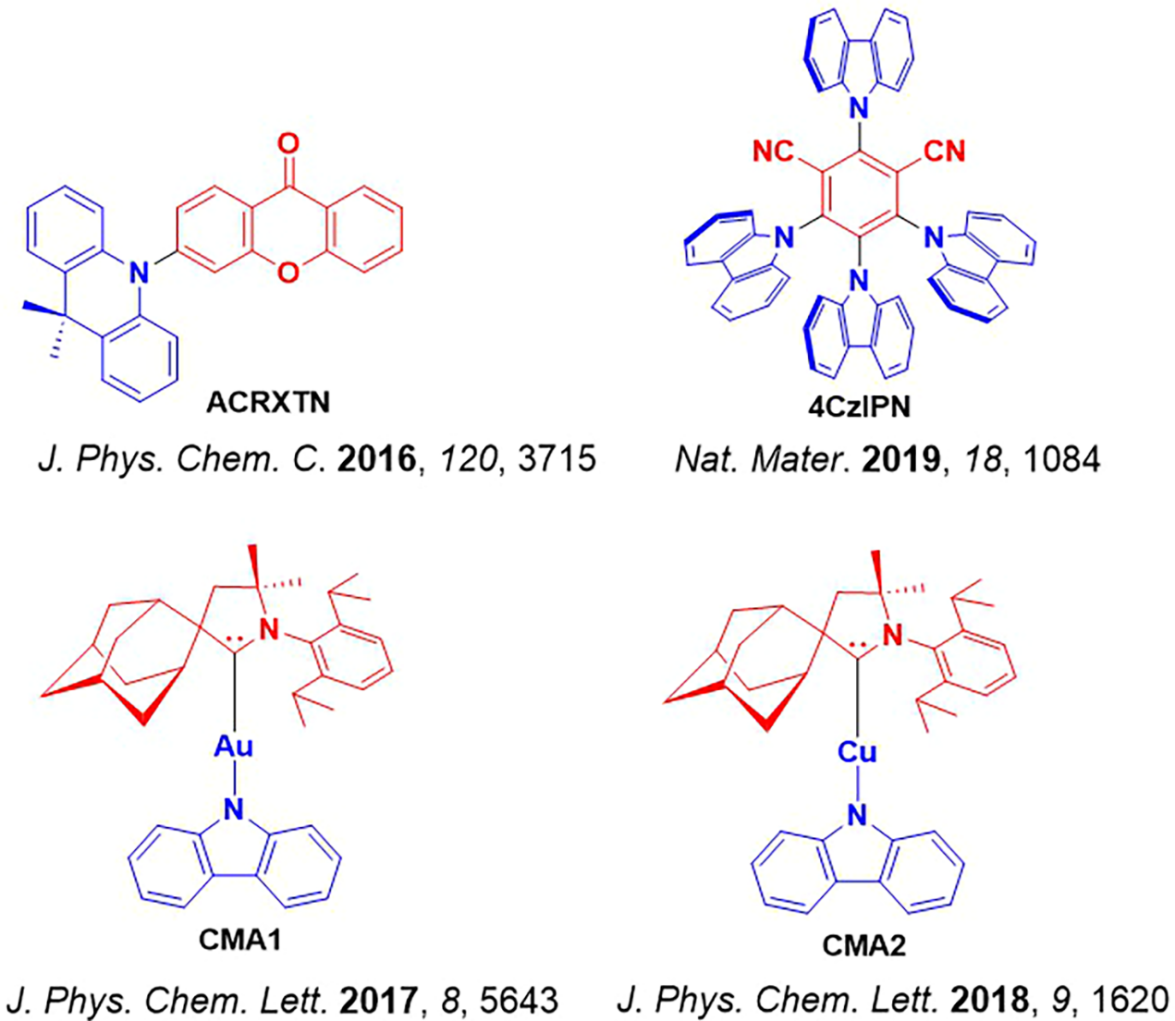
Examples of four emitters where the impacts of conformational and vibronic effects on *k*_RISC_ have been probed (the blue color signifies donor moieties, while the red color signifies acceptor moieties).

Computational chemistry was recently employed to rationalize the exciton harvesting mechanism present in carbene metal amide (CMA) TADF emitters,^194^ notably so as Di *et al.* had originally invoked an unusual RISC mechanism implicating a negative Δ*E*_ST_. Torsional freedom of the carbazolate bonded directly to the coinage metal was believed to be the driving force, but studies by Foller *et al*. and Taffet *et al*. contradicted this putative mechanism.^195,196^ Initially, it was shown that rotation around the N_Cz_-Metal bond in fact did reduce Δ*E*_ST_, but not to the extent that an inverted gap was calculated. The reduced Δ*E*_ST_ comes at a cost of reduced *k*_f_, hence this cannot be responsible for the extremely efficient emitters presented in the original study such as **CMA1** (Figure 22).^195,196^ In a subsequent study, Taffet *et al*. again highlighted the problems associated with the rotating ligand model, showing that SOC decreases along with Δ*E*_ST_, suggesting RISC will be less efficient as in the case of **CMA2** (Figure 22).^196^ Instead they proposed that the metal-carbene C-N bond deformation and the resulting bond length changes from a bending mode increase both *f* and SOC, enabling efficient RISC. It was hypothesised in both works that the coplanar geometry is responsible for the TADF observed in these two emitters.^195,196^

### 2.7 Role of Solvent and Solid-State Host Matrix: Polarization Effects

Excited states carrying a significant CT character are extremely sensitive to their environment, stabilizing their energy thanks to both electronic (and nuclear polarization) in host/guest systems in the solution state. When modelling emitters in solution, the polarizable continuum model (PCM) is often applied to address this behavior, partly because of its widespread availability within computation software packages and low computational cost. The PCM model approximates the solvent as a continuous medium of fixed dielectric, and therefore also considers that the solvent reorganization around the solute is slow (adiabatic approximation) so that the solute problem is solved for a fixed ‘configuration’ of the solvent molecules. This approach remains largely valid for highly polar solutions for which solvent molecules explore their available (electronic and nuclear) degrees of freedom much more slowly than that of the solute. The SRCT excited states nature typical of MR-TADF emitters are much less sensitive to host environment though, and so these considerations are typically restricted to D-A TADF emitters.

Recently, Painelli and co-workers developed in the frame of Onsager model (point dipole approximation), an anti-adiabatic approach where the electronic degrees of freedom of the solvent are considered to rearrange instantaneously.^197^ This approach is particularly relevant for solvents of low polarity. Interestingly for **DPTZ-DBTO2** (Figure 23), while Corrected Linear Response-PCM and State Specific-PCM approaches wrongly predict a negative Δ*E*_ST_, the anti-adiabatic approach predicts a positive Δ*E*_ST_ using a dielectric constant corresponding to toluene. The complex photophysics of **DMAC-TRZ**, Figure 23, have been modelled by Huu *et al*. who used a model including four electronic states (S_0_, one zwitterionic singlet and one zwitterionic triplet state, one LE state) that are coupled to a high frequency and a torsion vibrational modes.^131^ Solvent effects are also included within the frame of the Onsager model considering an anti-adiabatic approach for the electronic degrees of freedom of the solvent while the orientational ones is treated adiabatically. Their model nicely reproduced the absorption (emission) weak (strong) solvatochromism observed experimentally because of the very similar (different) relaxation of the electronic (orientational) part of the solvent response. Moving towards organic matrices, their model attributed the non-exponential behavior in the time-evolution of the S_1_ population to conformational static disorder and the large spectral shift of the delayed fluorescence (red- and blue-shift) to dielectric disorder.

**Figure 23:**
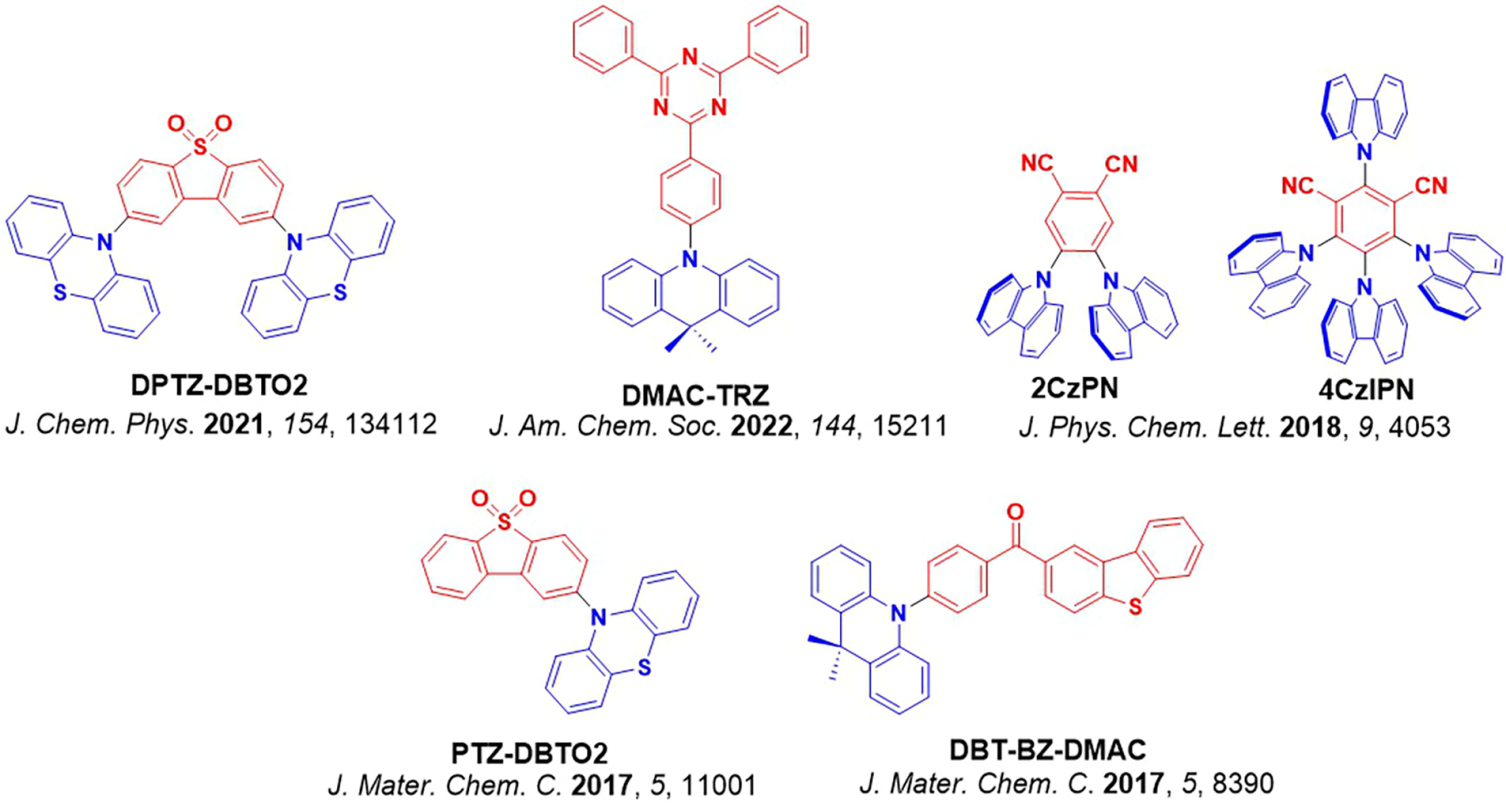
Structures of D-A TADF emitters whose properties have been computed in the condensed state and for which the solid-state polarization effects have been explicitly taken into account (the blue color signifies donor moieties, while the red color signifies acceptor moieties).

Along the same line, Gillet *et al*. performed QM/MM adiabatic molecular dynamics along the S_1_ and T_1_ potential energy surfaces of the **TXO-TPA** D-A TADF emitters in a toluene solvent.^135^ The S_1_ dynamics revealed that **TXO-TPA** evolves from a conformation where the D-A torsion is around 40 degrees in the ground state to a completely orthogonal one. The S_1_ state acquires a stronger CT character resulting in a drastic decrease of ΔE_ST_ from 0.3 eV to a nearly vanishing value. The highly twisted conformation is stabilized by the reorganization of the orientation of the toluene solvent molecules around the TADF emitter occurring within few picoseconds which further stabilize the highly CT S_1_ state. The T_1_ dynamics shows that in some instances T_1_, T_2_ and S_1_ are close to each other such as RISC becomes active. As a result, when going to vacuum to toluene, the RISC rate is boosted by three orders of magnitude due to the significant decrease of the RISC activation energy arising from both conformational changes and solvent reorganization.

Moving toward atomistic models for sold films, molecular dynamics (MD) calculations were used by Olivier *et al*. to simulate the organization of **2CzPN** and **4CzIPN** as neat films, Figure 23.^198^ In this study, they performed atomistic microelectrostatic calculations to understand solid-state polarization effects. Upon introducing this polarization treatment both the S_1_ and the T_1_ states, which are mixed CT-LE states, are each stabilized by 0.2 to 0.3 eV. This shift in energy is much smaller than the solid-state stabilization observed for charge carriers (holes and electrons), which is on the order of 1 eV for crystalline oligoacenes.^199,200^ This contrasting behavior is readily explained by the dipolar character of the CT-like excitation, as opposed to monopolar electrical excitations that are relevant in the case of the studies on oligoacenes. In the case of **2CzPN**, solid-state polarization leads to a decrease of Δ*E*_ST_, acting through the larger CT character computed of the S_1_ state compared with the T_1_ state. However, for **4CzIPN**, Δ*E*_ST_ is almost unaffected since both the S_1_ and the T_1_ states have nearly the same nature.

Investigating the role of host-guest interactions over time was performed on the emitter **PTZ-DBTO2** (Figure 23), considering DPEPO, PY2D and CBP as hosts.^201^ Using a combination of MD and TD-DFT calculations, it was shown that a blue-shift of the delayed emission of D-A TADF emitters in films at the longest time scale does not result from host reorganization, (and thus on specific host-guest interactions) but rather from a distribution of CT states each with different emission energies. The prompt fluorescence is essentially governed by a subset of higher-energy CT states that exhibit the largest hole–electron wave function overlap and therefore have the lowest CT content and thus the larger oscillator strength. As for the delayed fluorescence, the early part of the signal appears to be red-shifted in comparison to the prompt fluorescence because RISC occurs most rapidly in the subset of molecules with lower-energy CT states that have the smallest Δ*E*_ST_. The late delayed fluorescence component then occurs through higher-lying but more emissive CT states with slower RISC, thereby rationalizing the subtle transient blue-shift of the emission spectrum. Similarly, as a consequence of distributions of molecular geometries in experimental films, QM/MM simulations, where the QM calculations were performed at the TD-DFT level, revealed that the AIE of **DBT-BZ-DMAC**, Figure 23, and the resulting increase in Φ_PL_,^202^ was a consequence of restricted low energy (< 200 cm^–1^) torsional motion in the solid state, suppressing non-radiative decay to the ground state.

### 2.8 Emission Spectra Prediction

Prediction of the emission spectrum of TADF emitters is key to identifying whether or not the emission of a candidate material fulfils desired criteria in terms of color coordinate and purity. In this context, both the energy of the emission peak and the FWHM of the emission spectrum are relevant parameters to predict the color coordinate. At the molecular scale, calculations can also provide information as to which vibrational modes can contribute to the broadness of the emission spectrum, potentially driving molecular design rules to dampen their impact and reduce FWHM.^203^

Prediction of the emission spectrum of D-A emitters is quite a complex task because of the flexibility around the single bond connecting the donor and acceptor moieties. This broadness is usually emulated by reasonably assuming a Gaussian broadening, centred around the computed emission energy at the optimized geometry of the excited state.^131^ Another approach involves TD(A)-DFT calculations carried out on selected configurations extracted from a simulated MD trajectory. The broadening of the spectrum naturally arises from the fluctuations in the S_1_ excitation energy due to changes in conformation along the trajectory, and corresponds more directly to the experimental scenario.^85^ The difficulty with computational treatment of flexible vibrational modes primarily arises from their high anharmonicity, which requires treatment with more advanced methodologies such as adiabatic molecular dynamics. We refer the reader to a recent review for further information on these methods.^204^

Prediction of the emission spectrum of MR-TADF compounds has also been carried out, taking into consideration appropriate vibronic models.^173,205,206^ Due to the rigidity of the compounds, the most popular approaches reported in the literature assume that the potential energy surfaces of the ground and the excited states are described within an harmonic approximation. The transition dipole moment is approximated as a Taylor expansion up to the first order. The 0^th^ order term is the transition dipole moment at the S_1_ equilibrium geometry and corresponds to the Franck-Condon contribution, while the 1^st^ order term is the Herzberg-–eller contribution and accounts for the variation of the transition dipole moment along the 3N-6 ground-state normal modes of a given compound. The emission cross-section is obtained by thermal averaging over the vibrational manifold, usually performed in the time-domain. This approach allows for the determination of the temperature dependence of the population of the different vibrational levels, and thus of the emission spectrum. The undistorted harmonic oscillator approach is also commonly employed. This approximation assumes that the normal modes of vibration and their frequencies are identical between the ground and the excited state, and that the wavefunction of the vibrational modes are in their ground state (i.e. no thermal excitation of the vibrational modes).^207,208^ In most MR-TADF simulations the Herzberg–Teller contribution is neglected and only the Franck-Condon contribution is retained, because of the usually high 0^th^ order component of the transition dipole moment.

Demonstrating the utility of these approaches, a recent study of four **DiKTa** derivatives, **QA-PF**, **QA-PCN**, **QA-PMO**, and **QA-PCZ** (Figure 24), presented a normal mode analysis that identified the broadening of the emission spectra as arising due to two specific low-frequency vibrational modes below 130 cm^–1^. A higher-frequency mode was associated with the twisting of the **DiKTa** core, and a lower-frequency torsional mode involved the phenyl ring substituents.^203^ The addition of the phenyl substituents slightly reduces the Huang–Rhys factor of the higher-energy mode that primarily contributes to the width of the emission in **DiKTa**, resulting in comparably narrow emission spectra for these modified derivatives. Pei *et al*. studied two molecules, ***m*-Cz-BNCz** and ***p*-Cz-BNCz** (Figure 24), displaying significantly different emission spectra. The *meta*-substituted compound has a broad emission spectrum that is red-shifted with respect to the parent **BNCz** compound, while the *para*-substituted compound has a very similar and narrow emission spectrum to the parent compound. The difference in emission is attributed to the differing nature of the excited states where ***p*-Cz-BNCz** exhibits a typical SRCT-like emission spectrum while ***m*-Cz-BNCz** possesses an excited state with stronger LRCT character.^209^ TD-DFT calculations using a ω-tuned LRC-ωPBE/6-311G(d,p) method simulated the emission spectrum within the Franck-Condon approximation. The red-shifted emission in ***m*-Cz-BNCz** compared to ***p*-Cz-BNCz** was properly predicted, attributed to antibonding mixing of the HOMOs of the Cz and BNCz units, which allows for the spread of the HOMO electron density of ***m*-Cz-BNCz** onto the Cz substituent but not for for ***p*-Cz-BNCz**. Due to this anti-bonding interaction, the orbital localization of the HOMO is highly sensitive to variation in the torsion between the MR-TADF core and the Cz substituent, resulting in a modulation of the CT character upon variation of the torsion angle. This results in a broadened simulated spectrum in this MR-TADF material akin to those of D-A TADF emitters, which was also observed experimentally.^210^

**Figure 24:**
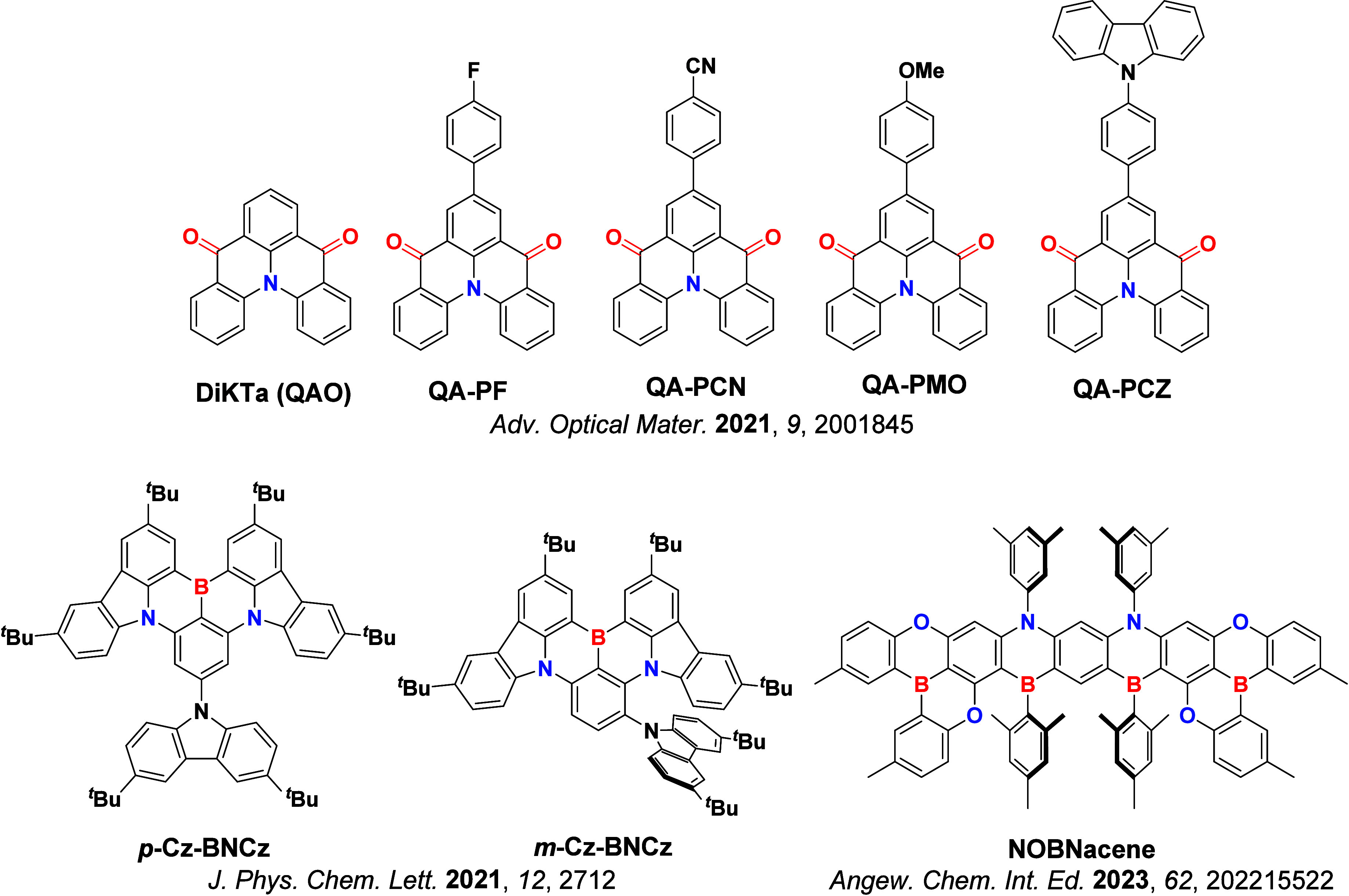
Examples of MR-TADF compounds where the emission spectra and vibrational contributions have been simulated (the blue color signifies donor atoms/functional groups, while the red color signifies acceptor atoms/functional groups).

**Figure 25:**
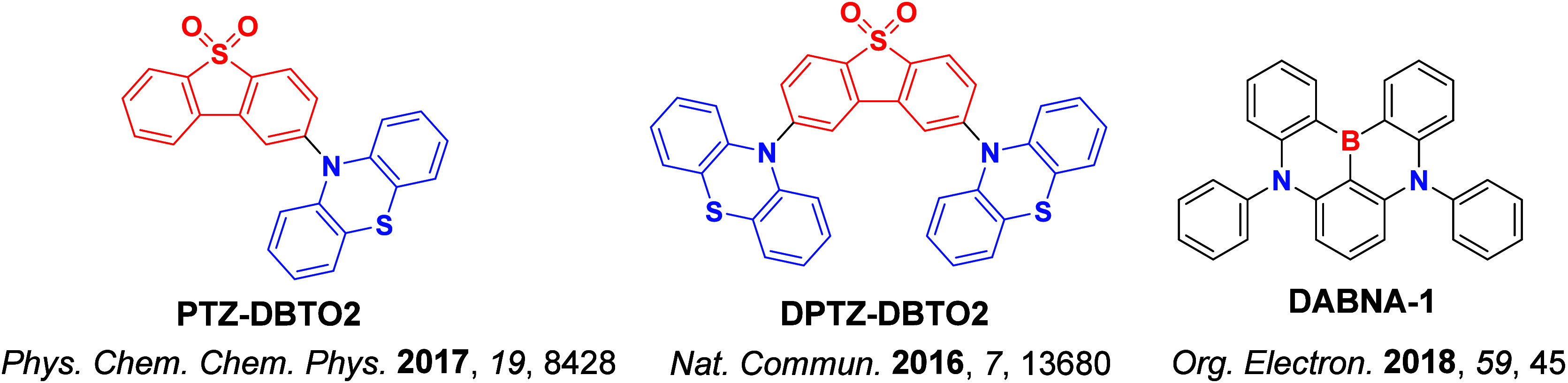
Examples of emitters where their excited-state dynamics have been investigated computationally (the blue color signifies donor moieties, while the red color signifies acceptor moieties).

Again highlighting the importance of considering of vibronic effects, we recently performed distorted harmonic oscillator modelling of the emission spectrum of a deep-blue nonacene emitter, **NOBNacene** (Figure 24).^211^ These simulations revealed that the main peak is broadened due to two lower-frequency vibrational modes at around 180 and 650 cm^–1^ involving out-of-plane distortion of the conjugated core, while the side band is dominated by a high-frequency stretching mode at around 1600 cm^–1^. These findings support the narrowband emission reported for this compound, with FHWM of 40 nm (0.29 eV) in THF.

### 2.9 Excited-State Dynamics

The excited-state dynamics of TADF emitters are typically investigated using either a rate approach or quantum dynamics simulations. *k*_RISC_ and radiative *k*_r_ rates as well as non-radiative processes such as *k*_IC_ and *k*_ISC_ have been computed both in a fully quantum Fermi Golden rule treatment, or using the derived semi-classical Marcus rate expression^189^ that was largely invoked in Section 4.^189,190^ Of these, quantum dynamics has the advantage of accounting equally for nuclear and electronic degrees of freedom, allowing for the study of the excited-state dynamics without making any assumptions about the interconversion mechanism.^81^ Following this strategy, Gibson *et al*. highlighted early on the importance of vibronic coupling in the upconversion of triplet excitons into singlets.^83,212^ Specifically, investigating **PTZ-DBTO2** and **DPTZ-DPTO2** (Figure 25), they showed using Multi Configuration Time-Dependent Hartree quantum dynamics simulations that RISC takes place through an intermediate LE triplet state and that RISC is strongly coupled to torsional vibrational modes. They also concluded that Δ*E*_ST_ is not the sole consideration when discussing RISC, but also identified the importance of the magnitude of the S_1_-T_2_ gap. Using a similar method, Northey et *al*. later showed that the accurate prediction of the size of the S_1_-T_2_ gap was also crucial to reproduce the timescale of the RISC process in **DABNA-1** (Figure 25).^213^

### 2.10 Machine Learning Screening of TADF Emitters

Considering the large volume of new TADF materials reported annually and the vast potential chemical space for their design, it is unsurprising that machine learning (ML) and the high-throughput computational screening of molecules have emerged as strategies to identify and assess promising candidate materials. The first study in 2015 used a tree-based genetic algorithm looking for compounds exhibiting a balance between small Δ*E*_ST_ and a large S_1_ transition dipole moment.^214^ A sea of 1.26 × 10^6^ fragments were used as the building blocks and 4000 potential targets were identified using the genetic algorithm, although none of the proposed emitters were synthesised in this study. Aspuru–Guzik and co-workers screened approximately a million candidate molecules using a combination of DFT ground-state optimization and TD-DFT excitation energies. ML techniques allowed selection of compounds offering the best balance between a small Δ*E*_ST,_ a large *f* and a fast *k*_TADF_.^136^ Within this study and following these calculations, experts (mostly synthetic organic chemists) rated the promising emitters (over a thousand) based on their predicted properties, their novelty, and their synthetic accessibility – identifying a set of four compounds to be synthesized and incorporated into OLEDs. A promising EQE_max_ of 22% was achieved for a device using one of the compounds, **J1** (Figure 26).

**Figure 26:**
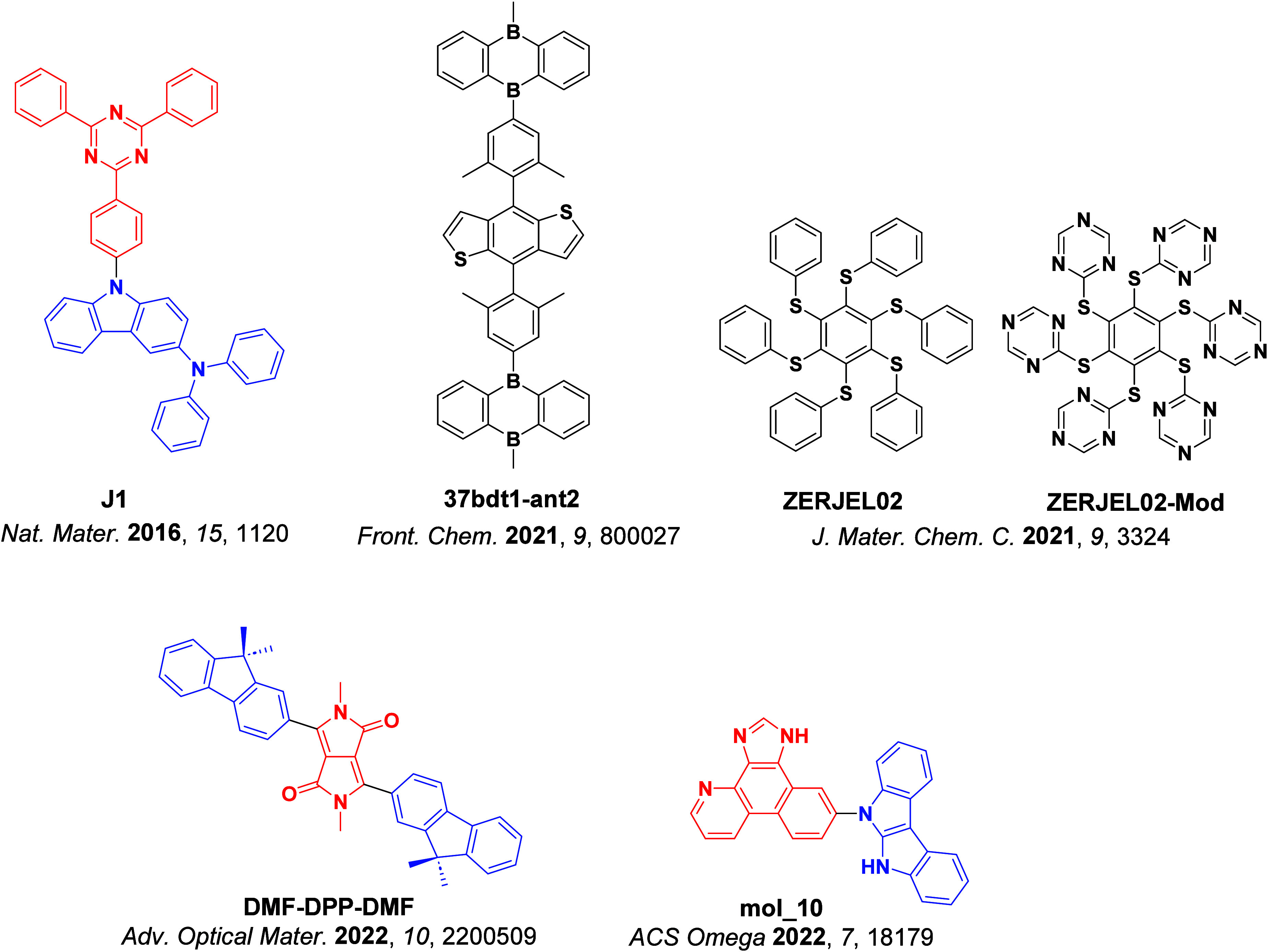
Examples of emitters generated from ML models and high-throughput screening techniques (the blue color signifies donor moieties, while the red color signifies acceptor moieties).

Along the same lines of computer-aided design, a study was carried out to identify the best combination of host materials and green TADF emitters.^215^ This was achieved by training an ML model that considered the Φ_PL_ of the guest materials in a host matrix, the frontier orbital energy differences between the host and the guest materials, and the Δ*E*_ST_ obtained experimentally in order to predict the EQE_max_ of the OLED device. This model appears to be quite reliable and allows identification of the best combination of host and green TADF emitters without the need for preparing the OLED. A similar study was carried out by Shi *et al*. to predict the EQE of TADF OLEDs based on the properties of the TADF emitters and the host in the EML, the transport layers, and the interfaces.^216^ Among the four algorithms employed, the neural network provides the greatest accuracy in predicting the EQE of the TADF OLEDs. Andrienko and co-workers reported a virtual screening study of TADF emitters for single-layer OLEDs.^217,218^ The requirements of the proposed emitters included a small Δ*E*_ST_, ambipolar transport (guaranteed by an ionization potential larger than 6.5 eV and an electron affinity below −2.5 eV), and small energetic disorder (supported by a small electric dipole). These three criteria were used to identify devices with a high expected EQE, supported by efficient triplet upconversion and charge recombination occurring far from the electrodes to avoid exciton quenching. After selection of potential candidates, amorphous phase molecular dynamics simulations were carried out to characterize the width of the energetic disorder associated with hole σ_h_ and electron transport σ_e_. Using this approach, **37bdt1-ant2** (Figure 26) was identified as a promising candidate due to its small σ_h_ and σ_e_.

Troisi and co-workers performed a high-throughput screening study on a series of compounds found in the Cambridge Structural Database without imposing a D-A type of structure.^219^ Interestingly, they found a category of compound not based on the typical D-A scaffold and subsequently designed new compounds based on these hits to optimize Δ*E*_ST_ together with the oscillator strength. A promising candidate, **ZERJEL02** (Figure 26), represents how compounds far removed from conventional D-A design rules can be identified using ML models, and shows reasonable calculated Δ*E*_ST_ of 0.35 eV and *f* of 0.06. The authors then refined the structure to improve TADF performance, with **ZERJEL02-Mod** (Figure 26) having a smaller calculated Δ*E*_ST_ of 0.19 eV and a comparable *f* of 0.03.

Zhu *et al*. reported the high-throughput screening of D–A–D triads designed to emit via a “Hot Exciton” (HCLT) mechanism.^220^ The strategy consisted of first establishing the threshold for large triplet–triplet splitting and a small singlet–triplet gap with the higher-lying triplet, then filtering combinations through rate comparison of competitive crossing pathways, and finally confirming RISC predictions with a more expensive evaluation of the magnitude of the spin-orbit coupling. Based on a dataset of 234 compounds, this protocol identified 31 candidate “hot exciton” emitters, four of which were indeed reported in the literature (**DMF-DPP-DMF** is one of the promising candidates, Figure 26). Remarkably, while most of the promising systems show prominent HLCT character, several candidates did not fulfil this condition, indicating that unidentified design principles exist to afford efficient OLED materials.

Tan *et al.* trained an ML model based on set of D-A TADF emitters that was used in combination with an adversarial autoencoder to generate new chemical structures of emitters.^221^ Among the large set of compounds generated, the ones with a Δ*E*_ST_ smaller than 0.4 eV and a *f* value larger than 0.02 were taken on for subsequent vertical excitation TD-DFT calculations. The set of compounds was further refined by computing Δ*E*_ST_ and *f* for the relaxed excited states geometry. Besides some known electron-donating and electron-accepting groups, the authors uncovered some new electro-active groups (such as **mol_10**, Figure 26). In the end, they identified 19 compounds with Δ*E*_ST_ smaller than 0.2 eV and S_1_-T_1_ SOC of tenths of meV.

### 2.11 Outlook

As summarised in this section, computational chemistry has proven to be an essential tool for generating mechanistic insight, and increasingly in recent years for actively guiding TADF emitter design. Computational chemistry is now arguably one of the key drivers of new TADF emitter development, allowing for quick and accurate screening of candidate structures. Supporting this utility, over the past 10 years there has been significant refinement in the computational methodologies used to calculate Δ*E*_ST_ to a high degree of accuracy; however, holistic prediction of efficient emitters beyond this parameter remains challenging. Aside from Δ*E*_ST_ the oscillator strength is a crucial indicator of Φ_PL_, but computing accurately the relevant rates of non-radiative processes remains non-trivial. Studies aiming at determining computationally the emission FWHM and *k*_RISC_ are also becoming more common, which while computationally demanding are welcome additional lenses for assessing TADF materials designs. For MR-TADF compounds specifically, although we can now accurately predict their Δ*E*_ST_ using wavefunction-based approaches, large-scale accurate screening has not yet been demonstrated due to their increased computational cost beyond the DFT approaches suitable for D-A TADF emitters. For both categories of TADF emitters finding a workable balance between computational cost and accuracy remains elusive, which is a key prerequisite before chemical space can be reliably explored and rapidly charted purely *in silico*.

In this context we note that while computational approaches towards assessing individual molecules are now reasonably mature (commonly performed in the gas phase or employing polarizable continuum solvent approximations), for yet deeper understanding and predictive power future efforts must increasingly focus on large multimolecular systems. The ability to simulate a molecule (or group of molecules) in an environment closely corresponding to real-world applications – namely by modelling atomistic solid-state host-guest morphologies – will give the most direct insight into real-world systems. These morphologies are what should then be used for subsequent excited-state calculations, rather than the more accessible relaxed geometries of isolated molecules typically used at present. Such calculations should also account for host-guest polarization effects, which are experimentally known to influence the ordering and spacings of excited states within the singlet and triplet manifolds, and therefore radically change the mechanistic picture of the whole TADF process. With the aforementioned effects already representing a challenging scale of simulation, for supreme accuracy these systems must also incorporate dynamic effects, as vibrational motion is also known experimentally to play a significant role in both the radiative and non-radiative processes governing TADF. Further, the involvement of higher-lying triplet states in RISC is already experimentally established, and computational approaches are uniquely suitable for probing these upper-state interactions towards building a more complete understanding of the structural features that support efficient TADF.

Aside from direct computations for specific molecular structures, recently we have also witnessed an increase in large-scale molecule screenings using machine learning tools. These strategies are welcome, likely speeding emitter design and pointing towards molecular structures far outside typical human intuition or imagination. However, the true impact of this approach is yet to be realised, with the optimal predicted emitters often being either synthetically very challenging (a difficult feature to quantify for model training), or not particularly novel (likely reflective of a limited training dataset of structures). We note that the utility of any machine learning model is intimately tied to the quality and size of its training data as well as identifying the desirable molecular features to be optimized. Moreover, the current academic research/cultural practices deprive the field of knowledge of TADF materials that do not reach “publishable” thresholds of performance or novelty. It is unclear how the research community’s decentralised and nebulous understanding of what doesn’t work – equally precious to the data scientist as what does work – could be made more accessible to support these data-driven efforts in materials design.

## 3 Blue TADF Emitters λ_EL_ < 490 nm

### 3.1 Introduction

Blue electroluminescence (EL) is uniquely challenging because blue photons are the highest in energy needed for human color vision (for a discussion of green and red EL see Sections 4 and 5, respectively). To produce these high photon energies large band gap emitters are required, with S_1_ energies typically > 2.7 eV. As most OLEDs contain an EML with an emitter doped into a host, these hosts must also be stable towards charge carriers and excitons of such high energy, significantly limiting the choice of usable chemical groups. Furthermore, in order to support high device efficiencies unlocked by triplet-harvesting in the emissive dopants, the host must also possess a higher triplet energy than that of the emitter. While these considerations are also applicable to red and green OLEDs, at the high energies associated with blue emission these requirements become especially limiting, with energies coming close or even exceeding bond dissociation energies of some of the organic materials. The relatively weak metal-ligand bonds associated with phosphorescent complexes are thought to be a main reason why blue PhOLEDs have not developed as rapidly as other colors, and these material stability issues are also a main factor that contributes to the severe efficiency roll-off in blue OLEDs.

To obtain blue emission, the bandgap of the emitter can be increased by stabilizing the HOMO and/or destabilizing the LUMO energy levels. In D-A TADF compounds this is typically achieved by combining weak electron donors (stabilized HOMO) with weak electron acceptors (destabilized LUMO), or by connecting multiple weak donors to a moderate acceptor. However, the energy of the emissive CT state is sensitive to the polarity of the environment, which leads to undesired emission red-shifting and broadening in the solid state. Together with the large FWHM typical of emissive CT states, this makes it very challenging to obtain deep blue emission that meets the Rec. 2020 standard.^222^ The emergence of MR-TADF emitters has helped to address the color purity of blue TADF OLEDs (see Section 11); however, their RISC rates are often far lower and to preserve this blue color most MR-TADF emitters must be doped into a host at a very low doping concentrations to avoid aggregation. These restrictions in applications lead to poorer exciton harvesting within the EML and a sub-optimally situated recombination zone. D-A TADF emitters, on the other hand, can be doped at higher concentrations or even used neat, and in many cases also contribute to charge balance in the devices.

In this context there has therefore been a particularly focused effort to develop efficient blue TADF emitters across the years 2017–2022.^40,223–228^ Many design strategies have been reported, almost always with the aims of achieving deep-blue emission while maintaining rapid RISC and high Φ_PL_. Some investigated facets of molecular design include dihedral angle control (increasing the D-A dihedral angle, and restricting D-A rotation), positional tuning between the donor and acceptor, heteroatom or heavy atom doping, rigidification of the emitter structure, and varying the number of donors and acceptors to optimize the D-A interaction. In this section we compare and analyze these blue D-A TADF emitters in terms of their photophysical properties and OLED performance, considering those having λ_EL_ < 490 nm and where the device showed an EQE_max_ > 10%, while the criterion for deep-blue emitters included the OLEDs having a CIE_y_ coordinate < 0.10. For the sake of clarity, the summarized examples are divided into subsections according to the electron-acceptor and their properties are summarized in Table S1.

### 3.2 History and Context

In their seminal 2012 paper Uoyama *et al.* reported **2CzPN** (Figure 27), a blue TADF emitter based on a phthalonitrile acceptor and carbazole donors.^31^ The design uses a moderate electron acceptor phthalonitrile coupled with multiple (in this case two) weak carbazole donors to give blue emission. The two donors are *ortho* to each other, which also helps to restrict the D-A rotation. This compound hence became one of the early benchmark emitters, with sky-blue emission (λ_PL_ = 473 nm, Φ_PL_ = 47%) and a delayed lifetime, τ_d_, of 166 μs in toluene (no Δ*E*_ST_ was measured). OLEDs with 5 wt% **2CzPN** in PPT host showed an EQE_max_ of 8%. Subsequent reports documented optimization of **2CzPN**-based OLEDs and 5% of **2CzPN** in a mixed cohost system of mCP:PPT (named PO15 in that work) showed the highest EQE_max_ of 21.8% at λ_EL_ = 480 nm [CIE coordinates of (0.17, 0.27)]. However, the efficiency roll-off remained severe with an EQE_1000_ of only 2.8%.^229^ Such a large drop in efficiency was attributed to the relatively slow RISC rate in **2CzPN**.

**Figure 27:**
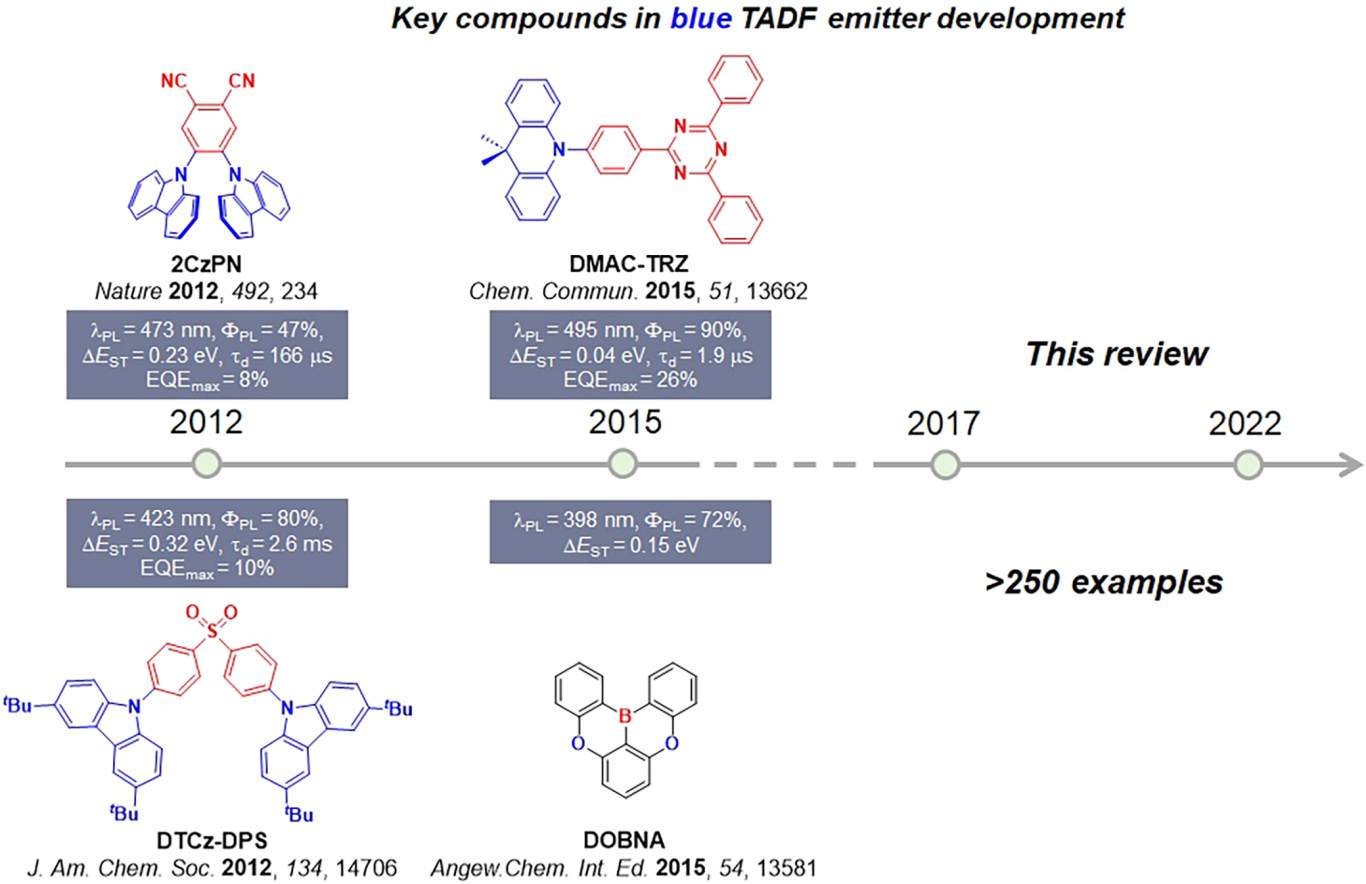
Timeline and structures of key blue TADF emitters preceding this review (the blue color signifies donor moieties, while the red color signifies acceptor moieties).

Zhang *et al*. reported the first deep-blue TADF OLED containing an emitter based on carbazole and diphenylsulfone, **DTCz-DPS** (originally named **3** in that work, Figure 27).^230^ Diphenylsulfone is a weak acceptor and two *tert*-butylcarbazoles served as moderate donors that yielded a deep-blue TADF emitter (λ_PL_ = 423 nm and Φ_PL_ = 80% in 10 wt% doped films in DPEPO), albeit with a long τ_d_ of 2.6 ms and a large Δ*E*_ST_ of 0.32 eV. OLEDs with **DTCz-DPS** showed an EQE_max_ of 9.9% at λ_EL_ = 423 nm [CIE coordinates of (0.15, 0.07)], but the efficiency roll-off was expectedly high considering the long τ_d_. Since this first report, the sulfone acceptor has been employed widely within blue TADF emitters.

Another benchmark TADF emitter, **DMAC-TRZ** (Figure 27),^231^ despite being a sky-blue emitter, provided a good starting point for further fine-tuning and enhancing of emission properties. Many subsequent blue TADF emitters are based on similar structures, and the 9,10-di­hy­dro-9,9-di­meth­yl­acri­dine (DMAC) donor has become extremely popular for its moderate donating strength and near orthogonal conformation adopted when it is N-bound to an acceptor (or bridge). The acceptor in **DMAC-TRZ** is 2,4,6-tri­phen­yl-1,3,5-tri­azine (TRZ), which has also become popular in both blue and green TADF materials. **DMAC-TRZ** shows efficient sky-blue emission (λ_PL_ = 495 nm and Φ_PL_ = 90% in 8 wt% doped mCPCN films) with a fast τ_d_ of 1.9 μs (Table S1). Such fast TADF is a consequence of the very small Δ*E*_ST_ of 0.046 eV. Efficient triplet harvesting was evident in the device, with EQE_max_ of 26.5% at λ_EL_ of 495 nm, and showing a negligible efficiency roll-off with EQE_100_ of 25.1%.

While in this section we do not review the multi-resonant TADF (MR-TADF) emitters (see Section 11), MR-TADF compounds have infiltrated the D-A world too; most notably employing MR-TADF moieties as acceptor, exemplified by **DOBNA** (Figure 27).^232^ Its intrinsically high triplet energy of 2.97 eV, its high Φ_PL_ of 72% and the ease of chemical functionalization of this molecule has made **DOBNA** a very component of blue D-A TADF emitters since its first report in 2015.

### 3.3 Triazine-Containing Emitters

The TRZ moiety is one of the most widely employed acceptors used in blue TADF emitter design, and is the subject of a detailed review previously published by our group.^51^ The popularity of TRZ stems from a reasonably shallow calculated LUMO energy of −1.72 eV suitable for blue emission, thermal stability and rigidity, and the ease of chemical substitution at the 2,4,6-carbon atoms. The chemical structures of recent TRZ-based blue TADF emitters summarized here are shown in Figure 28–Figure 32. One of the simplest recent examples of a blue D-A emitter containing TRZ is **Cz-Ph-TRZ** ^233^ (also reported as ***p*CzTPTZ** or **CzTRZ**,^234,235^ Figure 28). Doped at 10 wt% in DPEPO, this compound emits at λ_PL_ of 438 nm and has a Φ_PL_ of 71%, however the large Δ*E*_ST_ of 0.36 eV prohibits TADF and **Cz-Ph-TRZ** is classed as a purely fluorescent blue emitter – often used as a reference or control material in the development of other new TADF emitters. OLEDs with **Cz-Ph-TRZ** showed no indications of triplet harvesting, with the EQE_max_ reached only 4.1% (or 5.8% in a separate report^236^) at λ_EL_ = 446 nm [CIE coordinates of (0.14, 0.12)].

**Figure 28:**
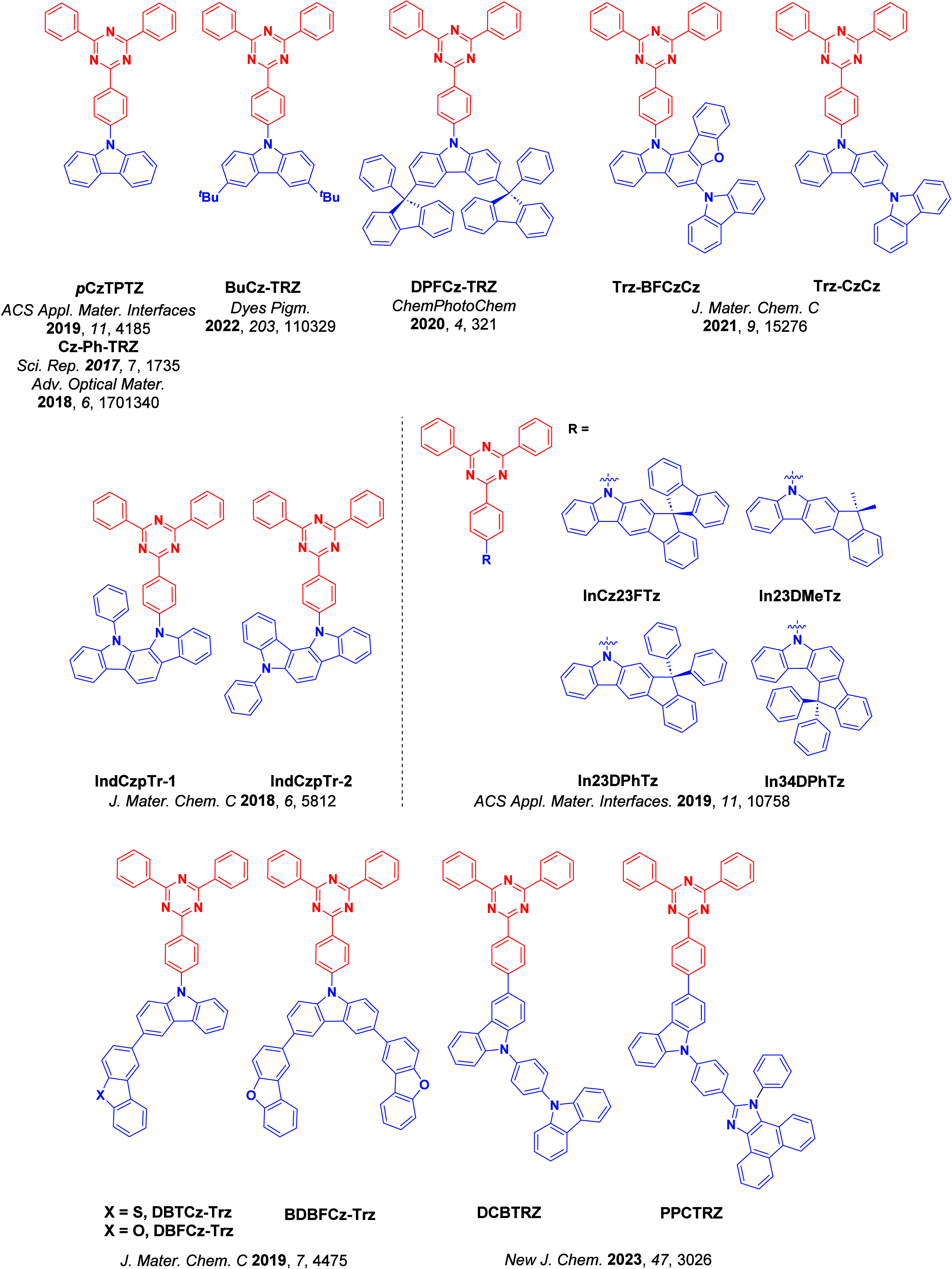
Structures of blue TADF emitters featuring TRZ as the acceptor moiety and single donor groups (the blue color signifies donor moieties, while the red color signifies acceptor moieties).

In tandem with TRZ, carbazole is one of the most commonly used donors for the design of TADF emitters, with its rigid structure and relatively weak calculated electron-donating strength (HOMO = −5.73 eV) supporting blue and deep-blue emission. However, unlike other larger donors such as DMAC, PXZ, or PTZ, carbazole has a smaller five-membered central ring which leads to it adopting less twisted conformations when linked to an acceptor (or bridge) via the nitrogen atom. This increased planarity can lead to a larger HOMO/LUMO overlap that translates to larger Δ*E*_ST_ and poor TADF performance, for example in the aforementioned **Cz-Ph-TRZ**. The Δ*E*_ST_ can, however, be reduced by increasing the donor strength of the carbazole by adding electron-donating substituents. The simplest example of such a modification was reported by Liu *et al.*, who introduced *tert*-butyl substituents at the 3- and 6-positions of carbazole in the compound **BuCz-TRZ** (Figure 28).^237^ The increased donor strength was enough to turn on TADF in this emitter, and **BuCz-TRZ** doped at 6 wt% in DPEPO film emits at λ_PL_ = 439 nm, has a Φ_PL_ of 83%, and a τ_d_ of 68.1 μs. The presence of TADF can be rationalized by the smaller Δ*E*_ST_ to 0.29 eV, compared to 0.36 eV in **Cz-Ph-TRZ**. The OLED with **BuCz-TRZ** showed an EQE_max_ of 9.3% at λ_EL_ of 459 nm [CIE coordinates (0.15, 0.15)], although severe efficiency roll-off of 69% at 100 cd m^–2^ was reported (Table S1). Employing the same strategy, bulkier analogue **DPF­Cz-TRZ** emits at λ_PL_ = 429 nm, has a Φ_PL_ of 88%, and a surprisingly short τ_d_ of 0.64 μs in toluene despite its moderately large Δ*E*_ST_ of 0.21 eV.^238^ The devices with 40 wt% **DPF­Cz-TRZ** doped in DPEPO showed an EQE_max_ of 15.5% and deep-blue emission at λ_EL_ = 445 nm [CIE coordinates of (0.15, 0.10)], although again with severe efficiency roll-off of 47% at 1000 cd m^–2^.

The electronics of carbazole as a donor can also be modified by fusing additional rings to it, with these extended donors also impacting the conformation of the emitter. Two isomeric hybrids based on indolocarbazole (ICz), **Ind­Czp­Tr-1** and **Ind­Czp­Tr-2** (Figure 28), exemplify this strategy.^239^ Both isomers showed comparable photophysics in neat films (λ_PL_ = 492 and 510 nm, Φ_PL_ = 75 and 71%, τ_d_ = 35 and 34 μs, and Δ*E*_ST_ = 0.13 and 0.11 eV, all respectively), with the less sterically crowded **Ind­Czp­Tr-2** also having preferential horizontal dipole orientation (Table S1). This resulted in a twofold increase of EQE_max_ in the OLED with **Ind­Czp­Tr-2** (30%) compared to that with **Ind­Czp­Tr-1** (14.5%). However, the indolo[3,2-b]carbazole of **Ind­Czp­Tr-2** is a stronger electron donor than indo­lo[2,3-a]car­ba­zole of **Ind­Czp­Tr-1**, which led to a red-shift in the emission of the former as was also observed in film photoluminescence. Similarly, fused carbazolyl donors incorporating spiro-fluor­enyl fragments are another well-studied category of extended donors. A family of four donors featuring differently substituted fluorenyl groups with a spiro­fluor­ene (**In­Cz23­FTz**, λ_PL_ = 470 nm), diphenyl groups (**In­Cz23­DPhTz** with λ_PL_ = 471 nm, and **In­Cz34­DPh­Trz** with λ_PL_ = 475 nm) and a dimethyl analogue (**In­Cz23­DMeTz**, λ_PL_ = 488 nm) were reported.^240^ Each of the emitters displayed high Φ_PL_ values between 86 and 98% in 10 wt% doped films in DPEPO; however, in each case the delayed emission contribution was low (between 11 and 17%), suggesting inefficient triplet harvesting. Long τ_d_ between 70 and 98 ms support this conclusion, which was surprising given the relatively small Δ*E*_ST_ values between 0.11 to 0.19 eV. The devices with **In­Cz23­FTz** (λ_EL_ = 468 nm), **In­Cz23­DPhTz** (λ_EL_ = 472 nm), **In23­DMeTz** (λ_EL_ = 480 nm), and **InCz34­PhTz** (λ_EL_ = 472 nm) showed EQE_max_ of 17.2, 17.9, 22.8, and 25.9%, respectively, although with significant efficiency roll-off of 63, 55, 43, and 45% at 100 cd m^–2^, and 82, 83, 75, and 80% at 1000 cd m^–2^.

Fusing a carbazole donor with a benzofuran group and attachment of an additional secondary carbazole unit gave the D-A emitter **Trz-BFCzCz** (Figure 28).^241^ The compound doped at 20 wt% in DPEPO emits at λ_PL_ of 460 nm, has a Φ_PL_ of 75%, a τ_d_ of 37 μs, and a moderate Δ*E*_ST_ of 0.13 eV in frozen toluene. Compared to reference emitter **Trz-CzCz** containing only bicarbazole and no fused furan group, the Δ*E*_ST_ was decreased by 100 meV and the τ_d_ was shortened by 70 μs with only a minor sacrifice in Φ_PL_ (Φ_PL_ of **Trz-CzCz** is 89%). The sky-blue OLED [CIE coordinates of (0.18, 0.32)] with **Trz-BFCzCz** showed an EQE_max_ of 23.3%, but the EQE_1000_ dropped considerably to 13.1%. Despite the efficiency roll-off being high, the **Trz-BFCzCz** OLED showed an improvement of four percentage points in the EQE_1000_ compared to device with **Trz-CzCz** (EQE_max_ = 23.8%, EQE_1000_ = 8.9%).

The electron-donating strength of carbazole derivatives can be further tuned by incorporating heteroaromatic substituents at the 3- and 6-positions. Examples include the use of diben­zo­thio­phene (**DBT­Cz-Trz**) and diben­zo­furan (**DBF­Cz-Trz** and **BDBF­Cz-Trz**, Figure 28).^242^ These compounds showed moderate Δ*E*_ST_ values of 0.20–0.23 eV and high Φ_PL_ of >89% in 15 wt% doped films in DPEPO. The OLEDs with **DBT­Cz-Trz**, **DBF­Cz-Trz**, and **BDBF­Cz-Trz** showed EQE_max_ of 21.7, 21.6, and 21.5% at λ_EL_ of 472, 472, and 488 nm, all respectively (Table S1). However, the efficiency roll-off was very high (88, 85, and 80%, respectively at 1000 cd m^–2^), which was attributed to singlet polaron quenching resulting from charge imbalance when using DPEPO host. Wang *et al*. reported a similar tria­zine-car­ba­zole hybrid named **PPC­TRZ** that featured phen­an­thro­imi­da­zole substitution on the carbazole and showed deep-blue emission, with a λ_PL_ of 411 nm and Φ_PL_ of 38% in 10 wt% doped films in CBP.^243^ The reference compound **DCB­TRZ** contained a second carbazole instead of the phen­an­thro­imi­da­zole, and also showed similar photophysics with λ_PL_ of 435 nm and Φ_PL_ of 39% in doped films. Both compounds have large Δ*E*_ST_ of 0.39 and 0.38 eV for **DCB­TRZ** and **PPC­TRZ**, respectively, and transient PL decay measurements of 10 wt% doped CBP films showed multiexponential decay kinetics with only short nanosecond lifetimes. Analysis of the variable-temperature data did not reveal any notable TADF behavior, despite the claims by the authors that the compounds are TADF-active. Deep-blue OLEDs with **DCB­TRZ** and **PPC­TRZ** in CBP host showed λ_EL_ of 440 and 442 nm (CIE_y_ of 0.059 and 0.063), which was very red-shifted compared to the film PL yet exhibited rather lower EQE_max_ values of 6.6 and 6.5% – likely illustrating the lack of triplet harvesting in the devices.

Frequently unmodified carbazole in D-A compounds adopts a relatively less twisted conformation with the acceptor, which can lead to large HOMO/LUMO overlap that is detrimental to overall TADF performance. Significant efforts have therefore been devoted to modifying carbazole to increase the steric congestion close to the nitrogen atom and tune the D-A dihedral angles. This is often achieved by either introducing substituents on a π-bridge connecting D-A moieties or attaching directly on the donor itself at the 1- and 8-positions. For example, cyano groups *ortho*-disposed to the donor act not only as a functional steric control units but can also tune the electronics of the resulting compound.^119,120^ This strategy was demonstrated in **Trz­CN­BFCz** and **Trz2­CN­BFCz** containing one or two CN groups, which were compared to reference material **Trz­BF­Cz** without such modification (Figure 29).^244^ **Trz­CN­BFCz** and **Trz2­CN­BFCz** in THF at 77 K have smaller Δ*E*_ST_ of 0.13 and 0.10 eV (respectively) compared to 0.27 eV for **Trz­BF­Cz**. As well as providing steric control, the CN groups also act to stabilize both the HOMO and LUMO levels of the emitters as determined by CV, where the HOMO of **Trz­CN­BF­Cz** was stabilized by ca. 100 meV and the LUMO by 200 meV in comparison to **Trz­BF­Cz**. In **Trz2­CN­BF­Cz** the influence of the CN groups on the orbital energies is even more dramatic, with the HOMO stabilized by 200 meV and LUMO by 670 meV. Consequently, the reduced HOMO-LUMO gap in **Trz2­CN­BF­Cz** resulted in a red-shifted emission (λ_PL_ = 432 nm) while the λ_PL_ is 407 nm for **Trz­CN­BF­Cz**. **Trz­CN­BF­Cz** has 100% Φ_PL_ in 10 wt% doped films in DPEPO, while the Φ_PL_ of **Trz2­CN­BF­Cz** is lower at 62% and short τ_d_ were registered for both compounds (τ_d_ = 9.4 μs for **Trz­CN­BF­Cz** and 3.1 μs for **Trz2­CN­BF­Cz**) (Table S1). Deep-blue OLEDs with **Trz­BF­Cz** [CIE coordinates of (0.15, 0.10)] showed an EQE_max_ of 18% which decreased by 55% at 1000 cd m^–2^. The OLED with **Trz­CN­BF­Cz** showed sky-blue emission [CIE coordinates of (0.17, 0.31)], an improved EQE_max_ of 20.9%, and a reduced efficiency roll-off of 37% at 1000 cd m^–2^. The OLED with **Trz2­CN­BF­Cz** showed CIE coordinates of (0.27, 0.52) and EQE_max_ of 15%, a consequence of the considerably lower Φ_PL_.

**Figure 29:**
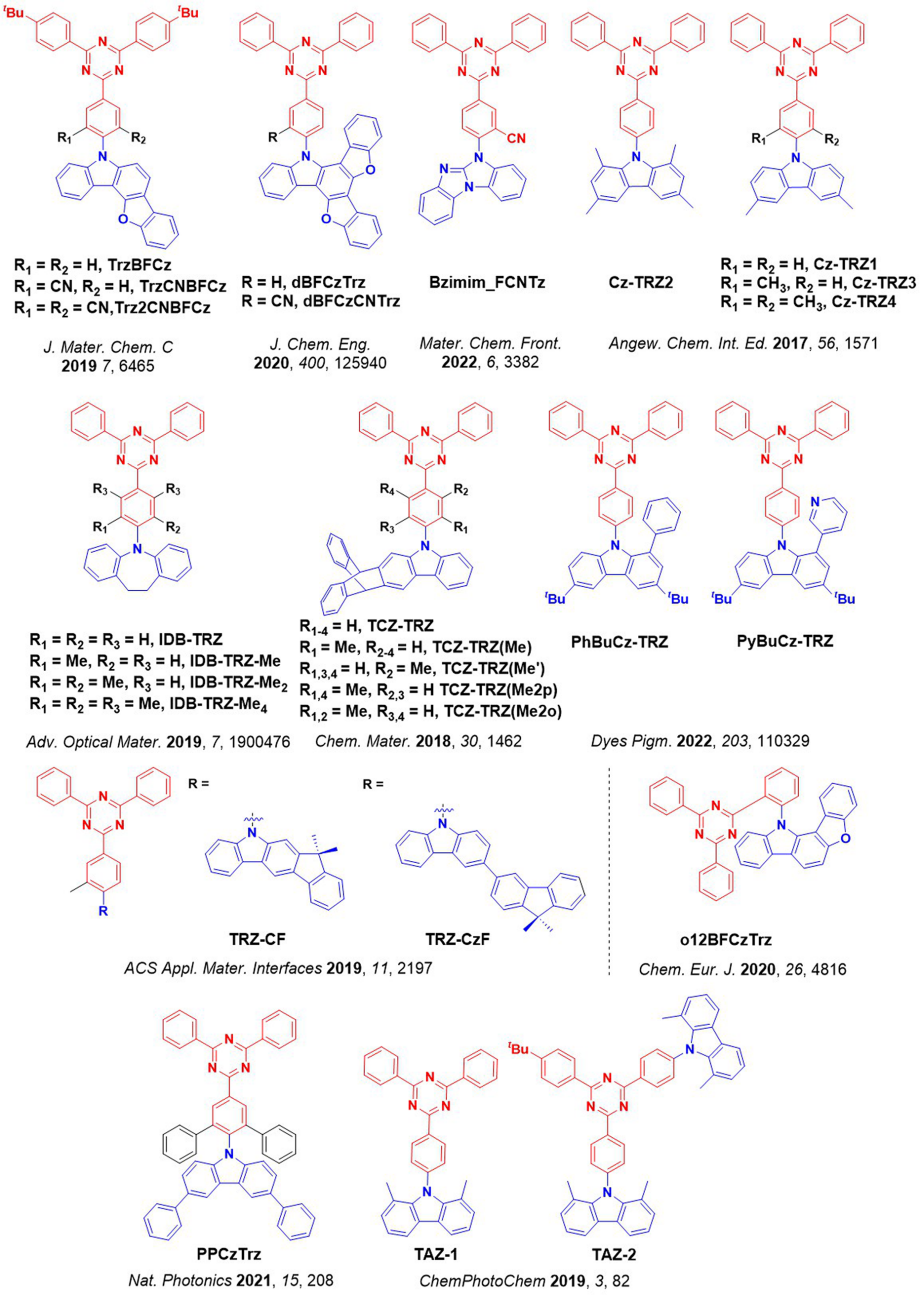
Structures of blue TADF emitters featuring TRZ as the acceptor moiety with sterically restricted donor groups resulting from substitution either on the bridging aryl moiety or on the donors themselves (the blue color signifies donor moieties, while the red color signifies acceptor moieties).

Demonstrating a similar impact of CN substitution on the π-bridge connecting D-A emitters, **dBF­Cz­Trz** and **dBF­Cz­CN­Trz** containing a dioxoazatruxene type donor were developed (Figure 29).^245^ As expected the bulky donor, both compounds adopt a strongly twisted geometry, with triplet energies found to be 2.88 and 3.02 eV for **dBF­Cz­CN­Trz** and **dBF­Cz­Trz**, respectively. While both molecules have near-unity Φ_PL_, **dBF­Cz­Trz** has a longer τ_d_ of 30 μs compared to 4.9 μs for **dBF­Cz­CN­Trz**, each at 20 wt% in DPEPO (Table S1). The OLED with **dBF­Cz­Trz** is bluer [CIE coordinates (0.16, 0.27)] although showed a lower EQE_max_ of 22.6% (efficiency roll-off of 46% at 1000 cd m^–2^) compared to its counterpart with **dBF­Cz­CN­Trz** ([CIE coordinates (0.22, 0.47)] and EQE_max_ of 27.5%, and efficiency roll-off of 12% at 1000 cd m^–2^). The same research group also investigated the effect of changing the donor position in **dBF­Cz­Trz**, where three ben­zo­furo­car­ba­zole isomers were *ortho*-connected to the triazine moiety.^246^ While all three of the newly reported compounds showed similar Φ_PL_ (82–88%), τ_d_ (3–4 μs), and triplet energy values (2.9–3.1 eV), the device with **o12­BF­Cz­Trz** showed the bluest emission with λ_EL_ = 478 nm [CIE coordinates of (0.16, 0.29)], an EQE_max_ of 19.2%, and a surprisingly low efficiency roll-off of 11% at 1000 cd m^–2^.

A fused imidazole-carbazole based donor in conjunction with a CN-substituted π-bridge was similarly explored for blue TADF emission in **Bzimim_​FCN­Tz** (Figure 29).^247^ **Bzimim_​FCN­Tz** emits at λ_PL_ of 461 nm with moderate Δ*E*_ST_ of 0.17 eV in toluene, and has unity Φ_PL_ with short τ_d_ of 7.9 μs in 20 wt% doped films in DPEPO. OLEDs with 20 wt% **Bzimim_​FCN­Tz** in DPEPO demonstrated the best performance with an EQE_max_ of 22.6% [CIE coordinates of (0.17, 0.27)], and a low efficiency roll-off of only 11% at 100 cd m^–2^ (Table S1).

Replacing electron-withdrawing cyano groups on phenylene bridges with donating methyl groups should achieve similar steric control while also supporting larger HOMO-LUMO gaps and blue-shifted emission. Cui *et al*. reported a series of carbazole-triazine compounds featuring such methyl substituents, used to fine-tune the blue emission color and TADF performance (Figure 29).^248^ DFT calculations showed that the dihedral angle between the donor and acceptor planes was tuned from 49.8° in reference emitter **Cz-TRZ1** (no methyl group between D-π-A) to 71.3° in **Cz-TRZ3** (1 methyl group on the π-bridge) to 86.7° in **Cz-TRZ2** (2 methyl groups on the donor) and 82.3° in **Cz-TRZ4** (2 methyl groups on the π-bridge). Incorporation of methyl groups on the π-bridge resulted in a modest red-shift in the emission in **Cz-TRZ3** and **Cz-TRZ4**, with λ_PL_ of 435 and 432 nm respectively. **Cz-TRZ2** bears two additional methyl groups on the donor, which increases the electron-donating strength and leads to a larger red-shift of the emission to a λ_PL_ of 465 nm in toluene. As with previous examples, the steric control of the dihedral angle between donor and acceptor has a significant impact on Δ*E*_ST_. **Cz-TRZ1** has a Δ*E*_ST_ of 0.43 eV in toluene, while introduction of methyl groups decreases Δ*E*_ST_ to 0.07, 0.17, and 0.15 eV in **Cz-TRZ2**, **Cz-TRZ3**, and **Cz-TRZ4**, respectively. This steric control has a negative impact on Φ_PL_ though, which falls from 72% for **Cz-TRZ1** to 35% for **Cz-TRZ4** in toluene. Notably, in the 6 wt% doped films in DPEPO the Φ_PL_ for **Cz-TRZ1–4** increases from 87 to 98, 92, and 85%, respectively. The impacts of steric control on the emitter conformation is also reflected in τ_d_, with unsubstituted **Cz-TRZ1** having the longest τ_d_ of 29 μs, while the τ_d_ of **Cz-TRZ2–4** are 3.5, 13, and 10 μs, respectively. Deep-blue OLEDs with **Cz-TRZ3** and **Cz-TRZ4** both showed CIE coordinates of (0.15, 0.10) and respective EQE_max_ of 19.2 and 18.3%, as well as efficiency roll-off of approximately 19 and 23% at 100 cd m^–2^. The OLED with **Cz-TRZ2** showed an EQE_max_ of 22.0% and sky-blue electro­lumin­es­cence due to its stronger donor, with CIE coordinates (0.16, 0.24).

Triptycene can also direct steric interactions to enhance frontier molecular orbital spatial separation, which was shown in a series of triptycene-modified carbazole-triazine materials. The presence of triptycene fused to carbazole enables TADF in **TCZ-TRZ** (λ_PL_ = 432 nm, Φ_PL_ = 77%, Δ*E*_ST_ = 0.27 eV, τ_d_ = 38 μs in toluene) while the reference carbazole-triazine compound **Cz-Ph-TRZ** is only fluorescent.^249^ Introduction of a methyl moiety *ortho* to the carbazole in **TCZ-TRZ­(Me)** or *ortho* to the TRZ in **TCZ-TRZ­(Me′)** results in further reduction in Δ*E*_ST_ for both methylated derivatives, while the specific position of the methyl group significantly affects the Φ_PL_. **TCZ-TRZ­(Me)** and **TCZ-TRZ­(Me′)** emit at λ_PL_ = 431 and 429 nm, have Φ_PL_ of 60 and 80%, Δ*E*_ST_ of 0.16 and 0.12 eV, and τ_d_ of 51 and 58 μs in toluene, all respectively. Additional emitters with two methyl groups decorating the bridging moiety have reduced Φ_PL_ although an improvement in RISC efficiency, as seen in **TCZ-TRZ­(Me2p)** and **TCZ-TRZ­(Me2o)** with λ_PL_ for both at 427 nm, Φ_PL_ of 46 and 47%, Δ*E*_ST_ of 0.14 and 0.18 eV, and τ_d_ of 39 and 37 μs in toluene, all respectively (Table S1). The devices with **TCZ-TRZ** and **TCZ-TRZ­(Me)** showed EQE_max_ of 10.4% [CIE coordinates of (0.16, 0.14)] and 11.1% [CIE coordinates of (0.17, 0.18)], respectively. Unfortunately, the devices suffered from a severe efficiency roll-off with EQE_50_ being only 3.4 and 2%, which was attributed to the long excited state lifetimes of the emitters.

The impact of methyl substitution on the linking phenylene bridge was also studied in a family of emitters containing an imino­di­benzyl donor. The reported molecules were **IDB-TRZ** (unsubstituted π-bridge), **IDB-TRZ-Me** (1 methyl group adjacent to the donor), **IDB-TRZ-Me_2_** (2 methyl groups adjacent to the donor), and **IDB-TRZ-Me_4_** (4 methyl groups, Figure 29).^250^ While the donor itself is quite flexible due to the ethyl bridge, it became locked in a highly twisted geometry in **IDB-TRZ-Me_2_** which resulted in decreased non-radiative decay, leading to a high Φ_PL_ of 98% in 20 wt% doped films in PPF. The steric control of the donor conformation also impacted the Δ*E*_ST_, which decreased progressively from 0.182 eV in **IDB-TRZ** to 0.093 eV in **IDB-TRZ-Me**, 0.077 eV in **IDB-TRZ-Me_2_**, and ∼0 eV in **IDB-TRZ-Me_4_**. The negligible Δ*E*_ST_ in **IDB-TRZ-Me_4_** supported a two-order magnitude accelerated *k*_RISC_ of 120 × 10^4^ s^–1^ compared to the other materials in the family (*k*_RISC_ = 1.7, 4.3, and 6.4 × 10^4^ s^–1^ for **IDB-TRZ**, **IDB-TRZ-Me**, and **IDB-TRZ-Me_2_**, respectively). However, this faster RISC came at a cost of a relatively low Φ_PL_ of 37%. The devices employing the **IDB-TRZ** derivatives showed sky-blue emission with λ_EL_ ranging from 474 to 496 nm, and with device efficiency reflecting the underlying photophysics of the emitter. The device with **IDB-TRZ-Me_2_** showed the highest EQE_max_ at 28.3%, followed by **IDB-TRZ-Me_4_** (16.4%), **IDB-TRZ-Me** (12.3%), and **IDB-TRZ** (6.8%). A relatively low efficiency roll-off of ∼14% at 100 cd m^–2^ was additionally reported for the device with **IDB-TRZ-Me_2_**.

Using a previously discussed fused carbazole-fluorene donor, emitter **TRZ-CF** (Figure 29) also contained a methyl group on the phenylene bridge adjacent to the donor. Comparator emitter **TRZ-CzF** contained the same modified acceptor with a carbazole donor instead featuring a pendant (rather than fused) fluorenyl group at the 2-position.^251^ **TRZ-CF** and **TRZCzF** have long τ_d_ of 7.3 and 11 ms in 20 wt% DPEPO films, with moderately large Δ*E*_ST_ of 0.22 and 0.31 eV in 2-MeTHF glass, all respectively. **TRZ-CF** with more conjugated fused donor emits at λ_PL_ = 474 nm, which is red-shifted compared to **TRZCzF** (λ_PL_ = 458 nm). Consistent with the respective Φ_PL_ values of 86 and 69%, the devices with **TRZ-CF** and **TRZ-CzF** showed EQE_max_ of 20 and 13.3% at λ_EL_ of 476 and 460 nm (Table S1). The magnitudes of the efficiency roll-off at 1000 cd m^–2^ were 43 and 72% respectively, which correlated with the magnitude of the delayed lifetime.

Bulky electron-withdrawing groups positioned at the C-1 position of carbazole were introduced to increase the torsion between triazine and the donor.^237^ The properties of five molecules containing phenyl, (**Ph­Bu­Cz-TRZ**), pyridinyl (**Py­Bu­Cz-TRZ**, **Py­Bu­Cz-Me­TRZ**), and cyano groups (**CN­Bu­Cz-TRZ**, Figure 29) were compared to reference compound **BuCz-TRZ** (Figure 28). The compounds showed near-UV to deep-blue emission with λ_PL_ ranging from 398–440 nm in toluene and high Φ_PL_ ranging from 77–87% in 6 wt.% doped films in DPEPO. Introduction of bulky group on the donor positively affected the delayed lifetimes, with the τ_d_ of 68.1 μs for the unsubstituted **BuCz-TRZ** dropping to 44.1 μs for phenyl-substituted **Ph­Bu­Cz-TRZ**, and then to 35.8 μs for pyridinyl-substituted **Py­Bu­Cz-TRZ**, and to 30.1 and 23.6 μs for methyl-substituted **Bu­Cz-Me­TRZ** and **Py­Bu­Cz-Me­TRZ**, respectively. Surprisingly, donor modifications had only a minor effect on the excited state energies with Δ*E*_ST_ ranging narrowly between 0.27–0.30 eV for **BuCz-TRZ**, **Ph­Bu­Cz-TRZ**, and **Py­Bu­Cz-TRZ**. Introduction of the methyl group, however, resulted in a further reduction of the Δ*E*_ST_ to 0.25 eV and 0.24 eV for **Bu­Cz-Me­TRZ** and **Py­Bu­Cz-Me­TRZ**, respectively. The reference OLED with **BuCz-TRZ** showed an EQE_max_ of 9.3% with λ_EL_ of 459 nm [CIE coordinates (0.15, 0.15)], but the devices with **Ph­Bu­Cz-TRZ** and **Py­Bu­Cz-TRZ** showed higher EQE_max_ of 12.1 and 15.3%, respectively (Table S1). These OLEDs were also bluer, with respective λ_EL_ of 458 and 455 nm [CIE coordinates of (0.15, 0.16) and (0.15, 0.13)]. On the other hand, OLEDs containing methyl and cyano-substituted derivatives showed significantly different performance metrics. The OLEDs with **Bu­Cz-Me­TRZ** and **Py­Bu­Cz-Me­TRZ** showed triplet harvesting with EQE_max_ of 15.5 and 11.7%, respectively, while the EQE_max_ of **CN­Bu­Cz-TRZ** OLED was only 4.1%. The devices suffered from severe efficiency roll-off though, with EQE dropping to 50% of the maximum values at 100 cd m^–2^ and no data provided at 1000 cd m^–2^.

The conformation of carbazole donors can also be twisted through the introduction of methyl substituents at both the 1- and 8-positions, as in **TAZ-1** and **TAZ-2** (Figure 29).^252^ Steric locking of carbazole not only yielded smaller Δ*E*_ST_ values (0.15 and 0.10 eV for **TAZ-1** and **TAZ-2**, respectively), but also improved Φ_PL_ of 88 and 100% at λ_PL_ = 468 or 476 nm in 20 wt% doped films in PPF, respectively (Table S1). The OLEDs with **TAZ-1** and **TAZ-2** showed EQE_max_ of 17.7 and 21.2%, and emitted at λ_EL_ of 478 and 479 nm [CIE coordinates of (0.16, 0.25) and (0.16, 0.27)], all respectively. Both OLEDs showed an efficiency roll-off of ∼40% at 1000 cd m^–2^. Yeon *et al*. instead explored the use of phenyl substituents on the phenylene bridged, *ortho* to the donor in **PPCzTrz**.^253^ The compound emits at λ_PL_ of 444 nm, has Φ_PL_ of 93%, τ_d_ of 25 μs, and Δ*E*_ST_ of 0.16 eV in 20 wt% doped films in DPEPO (Table S1). The OLEDs showed high EQE_max_ of 34% [CIE coordinates of (0.13, 0.20)] and a moderate efficiency roll-off of 24% at 1000 cd m^–2^. When a mixed co-host system of *o*CBP:​CN*m*CBPCN was used the EQE_max_ dropped to 10%, but the device lifetime (LT_50_) was improved significantly from 1 to 24 h running at 1000 cd m^–2^.

**DMAC-TRZ** (Figure 30) was designed relatively early in the current boom of TADF research, and has become a popular reference compound due to its high solid-state Φ_PL_ (90%), remarkably short τ_d_ of 1.9 μs, and negligible Δ*E*_ST_ of just 46 meV in 8 wt% doped mCPCN films.^231^ However, the compound is a sky-blue-emitter with λ_PL_ of 495 nm, and often green-emissive in other solvents and hosts. Significant effort has been devoted to derivatizing this model structure in order to retain the efficient TADF properties and tune the emission color deeper into the blue. For example, the methyl moieties in DMAC were substituted for an adamantyl group in ***a*-DMAc-TRZ**.^254^ Dual fluorescence was observed as a result of quasi-equatorial (QEC) and quasi-axial (QAC) excited state conformers. At 1 wt% doping in DPEPO the λ_PL_ of 430 nm was attributed to locally-excited fluorescence from QAC, exhibiting only a prompt lifetime of 15.45 ns and a large Δ*E*_ST_ of 0.31 eV (Table S1). Increasing the doping concentration to 20 wt% caused a red-shift in the emission to λ_PL_ = 490 nm along with activating efficient TADF with τ_d_ = 4.1 μs, Φ_PL_ = 86%, and a reduced Δ*E*_ST_ of 0.20 eV attributed to dominant QEC (Table S1). The OLED showed a high EQE_max_ of 28.9% at CIE coordinates of (0.18, 0.35), however the device showed a rather severe efficiency roll-off at 100 cd m^–2^ of 56%.

**Figure 30:**
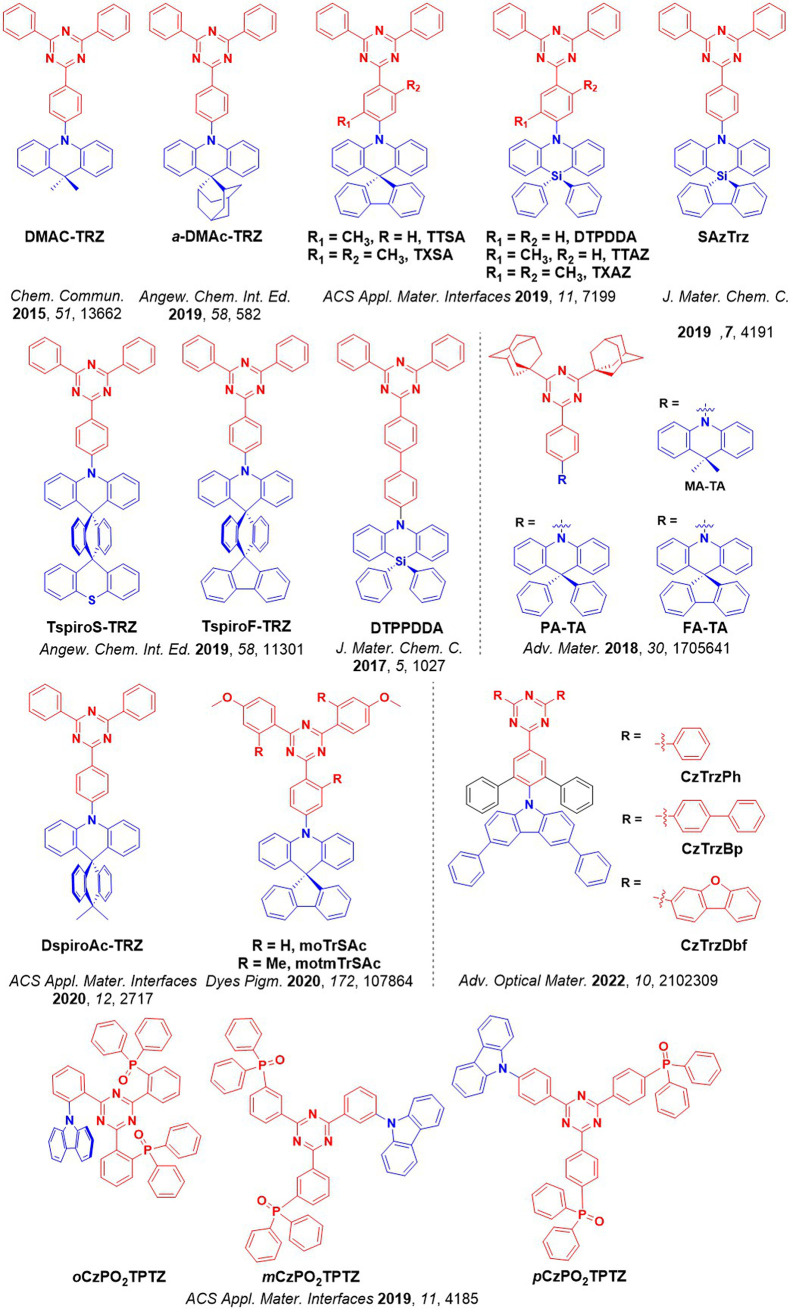
Structures of blue TADF emitters featuring modified acridine donor or TRZ acceptor groups (the blue color signifies donor moieties, while the red color signifies acceptor moieties).

Similar to the carbazole-containing examples discussed further above, methyl groups can be installed into the linking phenylene bridge to influence the acridine torsional angle.^255^ **TTSA** (λ_PL_ = 481 nm in 10 wt% mCP:​TSPO1) and **TTAZ** (λ_PL_ = 465 nm) feature only one methyl group *ortho* to the donor (Figure 30). **TXSA** (λ_PL_ = 475 nm in 10 wt% DPEPO) and **TXAZ** (λ_PL_ = 458 nm) have two methyl groups to further twist the Ph-acceptor torsion, resulting in a blue-shifted emission due to the reduced conjugation. The increased twisting of the bridging phenylene had only a minor effect on Φ_PL_ and Δ*E*_ST_, with **TTSA**, **TXSA** showing near-unity Φ_PL_ values and Δ*E*_ST_ as small as 60 meV. Phenazasiline derivatives **TTAZ** and **TXAZ** showed much lower Φ_PL_ of 68 and 50% and much larger Δ*E*_ST_ of 0.16 and 0.18 eV, respectively (Table S1). These values were found to be comparable to the ones of the previously reported unsubstituted phenazasiline-triazine hybrid, **DTP­DDA** (Φ_PL_ = 74% and Δ*E*_ST_ of 0.14 eV in 16 wt% doped films in mCP:​TSPO1).^256^ The EQE_max_ for the devices with **TTSA** and **TXSA** were 27.9 and 20.7% at λ_EL_ of 480 and 476 nm respectively, with reasonable efficiency roll-offs of 19 and 22% at 100 cd m^–2^. The devices with phenazasiline emitters **TTAZ** and **TXAZ** showed EQE_max_ values of 23.7 and 16.0% at λ_EL_ = 464 and 456 nm, with efficiency roll-off of 39 and 47% at 100 cd m^–2^.

The use of a phenazasiline donor coupled to an extended triazine yields deep-blue TADF emitter **DTPP­DDA** (Figure 30).^257^ When doped at 8 wt% in a cohost of mCP:​TSPO1, **DTPP­DDA** emits at λ_PL_ of 439 nm and has a Φ_PL_ of 38%. Despite the small Δ*E*_ST_ of 0.04 eV, no delayed emission was reported (Table S1). Deep-blue OLEDs with CIE coordinates (0.15, 0.09) nonetheless showed a moderate EQE_max_ of 4.7%, exceeding the theoretical limiting EQE_max_ of 4.4% for fluorescence and demonstrating at least some triplet harvesting activity.^257^ The absence of effective triplet harvesting resulted in a large efficiency roll-off of 50% at 100 cd m^–2^ and a low maximum luminance of 281 cd m^–2^. Similar silicon-containing analogues **DTP­DDA** and **SAzTrz**, were also reported.^258^ **SAzTrz** doped at 10 wt% in mCP:​TSPO1 co-host emits at λ_PL_ of 465 nm and has a Φ_PL_ of 65%, however a moderately large Δ*E*_ST_ of 0.25 eV resulted in slow TADF with τ_d_ of 173 μs (Table S1). The device employing the same co-host system emitted at CIE coordinates of (0.15, 0.18) and showed an EQE_max_ of 20.6%, but again severe efficiency roll-off of 64% at 100 cd m^–2^ was reported with a maximum luminance of 440 cd m^–2^.

A so-called tri-spiro donor strategy was shown to be effective in reducing ACQ, as well as in increasing the horizontal orientation of the TDMs in **TspiroS-TRZ** and **TspiroF-TRZ** (Figure 30).^259^ Perpendicular chromophore orientation ensured sufficient frontier orbital separation, which resulted in Δ*E*_ST_ values as small as 0.05 and 0.08 eV for **TspiroS-TRZ** and **TspiroF-TRZ** respectively. Both materials showed sky-blue emission in 30 wt% doped films in DPEPO (λ_PL_ = 470 and 479 nm) with τ_d_ of 3.0 and 4.5 μs, and Φ_PL_ of 75 and 82%, all respectively (Table S1). Such outstanding photophysics was reflected in the device performance, with EQE_max_ values of 33.3 and 28.1% for the OLEDs with **TspiroS-TRZ** and **TspiroF-TRZ** at λ_EL_ = 481 and 493 nm. These devices also displayed moderate efficiency roll-off of 29 and 18% at 100 cd m^–2^. Remarkably, a non-doped device containing **TspiroS-TRZ** demonstrated an EQE_max_ of 20%, which at the time was one of the most efficient sky-blue non-doped OLEDs. The same authors subsequently reported a slightly modified emitter structure, **Dspiro­Ac-TRZ**.^260^ Studying intermolecular interactions in the crystalline state, the authors discovered that the intermolecular distances were sufficiently long to decrease the HOMO-LUMO interactions of dimers while still allowing for horizontal orientation of their TDM. This afforded high Φ_PL_ in crystalline and amorphous non-doped films of 78.5% at λ_PL_ = 496 nm and 83.7% at λ_PL_ = 482 nm, respectively. The Δ*E*_ST_, determined in frozen toluene, was 0.04 eV and the τ_d_ of the neat film was τ_d_ = 3.2 μs. The non-doped sky-blue device consequently outperformed the parent device with **TspiroS-TRZ**, with an EQE_max_ of 25.7% and an efficiency roll-off of 36% at 1000 cd m^–2^.

In addition to donor modification, modulating of the degree of conjugation in TRZ has been probed as a strategy to blue-shift the emission. Compounds **moTrSAc** and **motmTr­SAc** (Figure 30)^261^ contain a spiro-DMAC donor linked to a modified TRZ acceptor. Twisting of the TRZ phenylenes by means of *ortho*-methyl groups resulted in destabilization of both the singlet and triplet energies by ca. 100 meV in **motmTr­SAc** compared to the unsubstituted parent **moTr­SAc**, with very low Δ*E*_ST_ of 0.01 eV for both compounds. Short τ_d_ of 3.4 and 3.0 μs and Φ_PL_ of 70% at λ_PL_ = 482 nm or 51% at λ_PL_ = 469 nm were reported for **moTr­SAc** and **motmTr­SAc** in respective 10 wt% doped films in DPEPO (Table S1). The bluest device incorporating **motmTr­SAc** showed an EQE_max_ of 19.5% at CIE coordinates of (0.16, 0.22).

Starting from the basis of the previously discussed **PPCz­Trz** (Figure 29), Kang *et al*. modified the TRZ acceptor moiety in the hope of localizing the triplet excitons far from the weak D-A C-N bond.^262^ A series of triazine-carbazole compounds was designed with a focus on expanding conjugation in the TRZ moiety through introduction of biphenyl (**Cz­Trz­Bp**) or dibenzofuranyl fragments (**Cz­Trz­Dbf**). SCS-ADC(2) calculations revealed migration of the ^3^LE state, from localization on the π-spacer with slight extension into the donor in reference molecule **Cz­Trz­Ph**, to localization mainly on the distal arms of the acceptor in **Cz­Trz­Bp** and **Cz­Trz­Dbf**, thereby reducing excited state electron density near the vulnerable C-N bond. All three compounds showed deep-blue emission with λ_PL_ ranging from 444–451 nm in toluene and have moderate Φ_PL_ of 34, 35, and 49% for **CzTrzPh**, **CzTrzBp**, and **CzTrzDbf** respectively in 15 wt% doped films in CNmCBPCN. The extension of the π-conjugation on the acceptor however increased the Δ*E*_ST_ values from 0.16 to 0.26 and 0.31 eV, which translated into lengthening of the τ_d_ from 15.3 to 26.3 and 30.3 μs for **CzTrzPh**, **Cz­Trz­Dbf**, and **Cz­TrzBp**, all respectively. Blue OLEDs with **Cz­Trz­Dbf** and **Cz­Trz­Bp** showed EQE_max_ of 12.4 and 9.2% at CIE coordinates of (0.16, 0.19) and (0.16, 0.14), respectively. In terms of initial performance, the **CzTrzPh** containing OLED showed almost identical values to the device with **Cz­Trz­Dbf**; however, the LT_80_ of the former was only 17.2 hours compared to 40.3 hours for the latter, both running at an initial 500 cd m^–2^. The **CzTrzBp** OLED also showed an improved device lifetime with LT_80_ of 30.5 hours.

Replacing the peripheral rings of TRZ with adamantyl groups not only improved solubility, allowing solution-processed devices to be fabricated, but also resulted in a destabilized LUMO and a blue-shifted emission.^263^ Wada *et al*. employed this acceptor in combination with DMAC donors in three blue TADF emitters, **MA-TA**, **FA-TA**, and **PA-TA** (Figure 30).^263^ **MA-TA**, **FA-TA**, and **PA-TA** doped at 10 wt% in CzSi have Φ_PL_ of 83% [CIE coordinates of (0.15, 0.19)], 76% [CIE coordinates of (0.15, 0.13)], and 70% [CIE coordinates of (0.15, 0.10)], respectively. The solution-processed devices showed respective EQE_max_ of 22.1, 11.2, and 6.7% at the same CIE coordinates as the photoluminescence, with efficiency roll-off of 37% at 100 cd m^–2^ noted for the device with **MA-TA**. Luminance of 100 cd m^–2^ could not be reached for the devices using the other two emitters.

Using a carbazole donor and a TRZ acceptor functionalized with phosphine oxide groups, excellent TADF efficiency was observed in ***o*CzPO2­TPTZ**, ***m*CzPO2­TPTZ**, and ***p*CzPO2­TPTZ** (Figure 30).^264^ These compounds emit at λ_PL_ of 470–485 nm and showed a clear trend in Φ_PL_ across the *o*/*m*/*p* isomers of 25, 53 and 75%, respectively. The trends in Φ_PL_ were then reflected in the OLED performance: an EQE_max_ of 20.9% was reported for the device with ***p*CzPO2­TPTZ**, which decreased to 11.6% for the device with ***m*CzPO2­TPTZ** and to 6.7% for the device with ***o*CzPO2­TPTZ** (Table S1). Compared to the non-phosphine oxide parent, ***p*Cz­TPTZ** (Figure 28), the presence of the secondary acceptor led to a smaller Δ*E*_ST_, higher Φ_PL_, and faster *k*_RISC_ (Δ*E*_ST_ = 0.01 and 0.17 eV, Φ_PL_ = 73 and 11%, and *k*_RISC_ = 7.1 and 0.6 × 10^4^ s^–1^ for **pCzPO2­TPTZ** and ***p*Cz­TPTZ**, respectively).

Blue TADF can alternatively be enabled by incorporating multiple weak donors about a central triazine acceptor. Oh *et al*. explored the impact on blue TADF devices of changing the relative position of a pair of carbazole groups about the same phenylene bridge attached to a triazine acceptor.^265^ A blue-shift was observed in moving from *ortho-meta* substitution [**23CT**, CIE coordinates of (0.17, 0.33)] to *ortho-para* [**24CT**, CIE coordinates of (0.15, 0.26)] and finally to *meta-para* substitution [**34CT**, CIE coordinates of (0.15, 0., 0.17)], which is in line with the decreasing D-A dihedral angles that result in the later compounds having excited states with more of LE character (Figure 31). This blue-shift is accompanied by a negative impact on the TADF properties though, with Δ*E*_ST_ = ∼0, 0.11, and 0.29 eV for **23CT**, **24CT**, and **34CT**, respectively (Table S1). The devices showed EQE_max_ of 21.8% [CIE coordinates of (0.17, 0.33)], 22.4% [CIE coordinates of (0.15, 0.26)], and 13.3% [CIE coordinates of (0.15, 0.17)], respectively, with extraordinary efficiency roll-off of only 5% at 1000 cd m^–2^ for the device with **23CT**. The efficiency roll-off for the devices with **24CT** (32%) and **34CT** (58%) was considerably larger. The improved device performance for **23CT** was attributed to efficient RISC as a result of its well-aligned ^1^CT and ^3^LE states. The same authors also explored the benefit of having three carbazole donors similarly substituted about the same phenylene at different positions.^266^ While the greenest compound from the series **234Cz­Trz** has the shortest τ_d_ of 4.1 μs and a Φ_PL_ of 90%, the other two compounds, **235Cz­Trz** and **245Cz­Trz**, have longer τ_d_ of 8.4 and 9.7 μs respectively and also almost unity Φ_PL_ values in 30 wt% doped films in DPEPO. The bluest device using **245Cz­Trz** [CIE coordinates of (0.17, 0.39)] showed an EQE_max_ of 22% as well as an efficiency roll-off of 37% at 1000 cd m^–2^. Comparing the device performance of emitters with 2 *vs* 3 carbazoles, *i.e.*, OLEDs with **245Cz­Trz** *vs* **23CT**, the latter outperformed the former in terms of color purity and efficiency roll-off despite having almost identical EQE_max_. In separate report, excellent efficiency roll-off of 5% at 1000 cd m^–2^ with CIE coordinates of (0.15, 0.22) was achieved using **trisCz-TRZ**, an emitter containing three carbazoles symmetrically *ortho*-substituted to a triphenyltriazine core.^267^ This performance was supported by its short τ_d_ of 5.0 μs and small Δ*E*_ST_ of 0.03 eV, although the EQE_max_ was only 16.5%.

**Figure 31:**
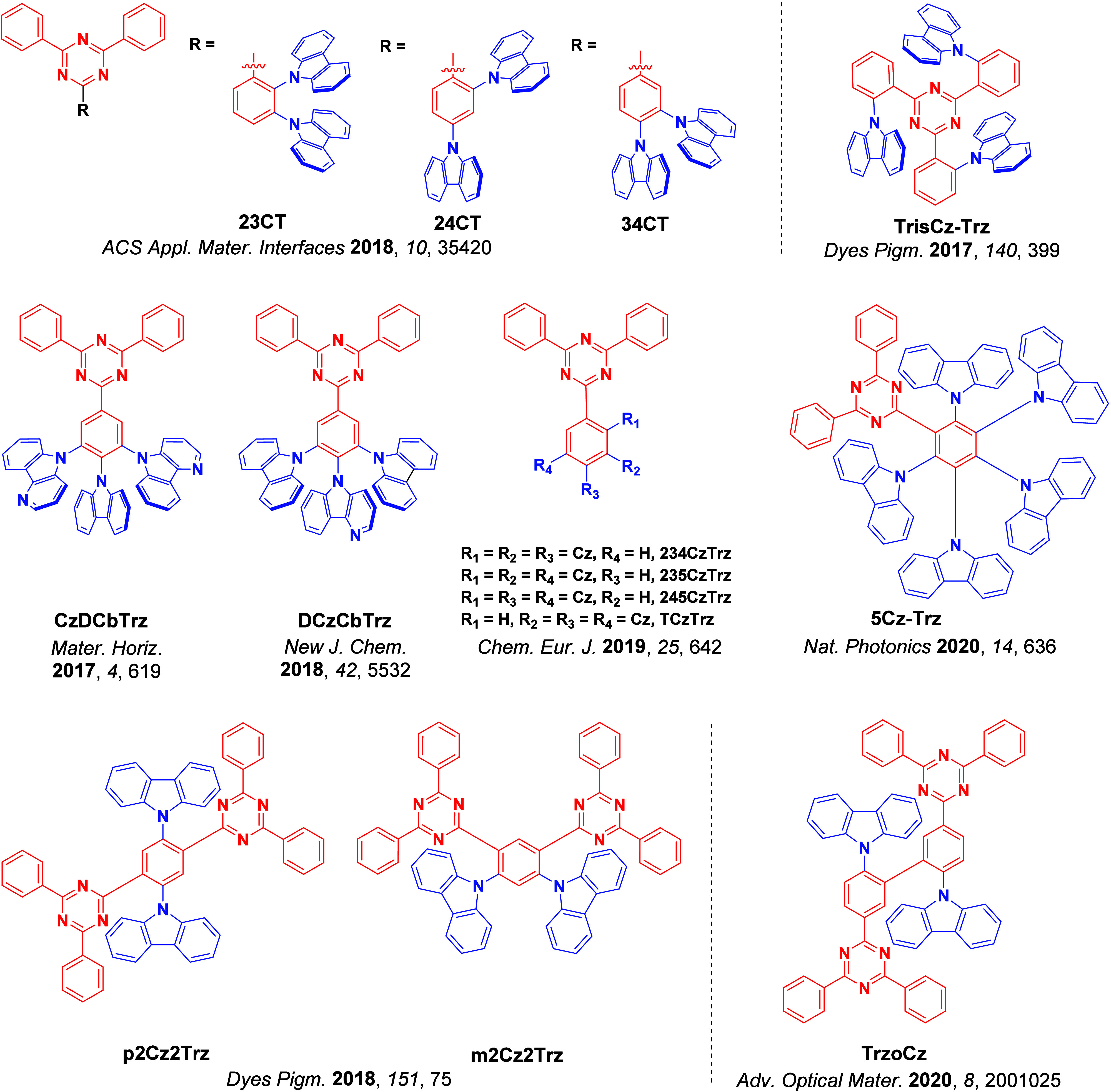
Structures of blue TADF emitters featuring TRZ acceptor moieties attached to multiple donors (the blue color signifies donor moieties, while the red color signifies acceptor moieties).

Another multi-donor-substituted example is **CzDCbTrz**, whereby two δ-carbolines are attached at *meta* positions to TRZ with a carbazole at the *para* position (Figure 31). The EQE_max_ of the device with **CzDCbTrz** was 22.0%, which is an improvement from the 19.0% reported for tris-carbazole containing reference emitter **TCzTrz**.^268^ A shorter τ_d_ of 7.5 μs (compared to 9.2 μs **TCzTrz**) likely contributes to the improved device performance while a slight blue-shift was also reported for the **CzDCbTrz** λ_EL_, shifting from 476 to 471 nm on the inclusion of carbolines. Similar to **CzDCbTrz**, **DCzCbTrz** instead contains one *para*-connected δ-carboline and two *meta*-substituted carbazole donors around the bridging phenylene.^269^ The OLED with **DCzCbTrz** showed a similar EQE_max_ of 22.1% and a similar efficiency roll-off at 1000 cd m^–2^ (54% for the device with **DCzCbTrz** and 57% for the device with **CzDCbTrz**), which was again an improvement from carboline-free **TCzTrz**. Other emitters featuring α- or δ- carbolines paired with benzonitrile acceptors are discussed further below.

Increasing further the number of carbazole donors, compound **5Cz-Trz** (Figure 31) is an example of how multiple donor units can form “charge-resonance-type hybrid triplet states” leading to large spin–orbit coupling and a dense manifold of triplet states energetically close to the singlets.^93^ **5Cz-Trz** emits at λ_PL_ of 486 nm and has almost unity Φ_PL_, a short τ_d_ of 2.1 μs, and a negligible Δ*E*_ST_ of 0.02 eV (Table S1). The device with **5Cz-Trz** showed very high EQE_max_ of 29.3% at λ_EL_ of 486 nm, along with negligible efficiency roll-off. Moreover, the sky-blue device showed very high operational stability, with LT_90_ at 1,000 cd m^–2^ of ca. 600 h.

Multiple carbazoles can also be combined with multiple triazines to produce blue TADF emitters. A pair of rigid bistriazine and biscarbazole isomers, **p2Cz2Trz** and **m2Cz2Trz**, were studied by Lee *et al.* (Figure 31).^270^ Each of the two TRZ acceptors and two carbazoles are disposed *para* to each other in **p2Cz2Trz**, such that each donor is *ortho* to one acceptor and *meta* to the other. In **m2Cz2Trz** each donor is arranged in *para* and *ortho* dispositions to the two acceptors, resulting in different orbital separation among the D-A pairs. Both molecules have deep-blue emission in 1 wt% doped films in PMMA, with the singlet state energies estimated at S_1_ = 2.88 and 3.02 eV, with Φ_PL_ values of 82 and 91% in air, and τ_d_ of 16.6 and 12.2 μs for **p2Cz2Trz** and **m2Cz2Trz**, all respectively (Table S1). The device with **p2Cz2Trz** displayed green emission and showed an EQE_max_ of 12.5% [CIE coordinates of (0.39, 0.58)], while that with **m2Cz2Trz** remained sky-blue [CIE coordinates of (0.20, 0.47)] with an EQE_max_ of 18.5% and low efficiency roll-off of 12% at 1000 cd m^–2^. In another similar structure, substituents *ortho*- to the carbazole moiety restrain molecular motion as well as increase the D-A dihedral angle in **TrzoCz** (Figure 31). This has the potential of boosting the solid-state Φ_PL_ while yielding a smaller Δ*E*_ST_, and indeed **TrzoCz** doped at 10 wt% in DPEPO emits at λ_PL_ = 450 nm, has close-to-unity Φ_PL_, and a τ_d_ of 20 μs.^271^ The **TrzoCz** devices showed an EQE_max_ of 28% at λ_EL_ of 484 nm [(CIE of (0.15, 0.32)] although suffered from a severe efficiency roll-off of 60% at 1000 cd m^–2^.

An unconventional macrocyclic triphenylamine donor in conjunction with TRZ was recently explored for the construction of blue TADF emitters. Lin *et al.* designed **c-NN-TRZ** and **c-NN-MeTrz** either with or without methyl groups *ortho* to the donor to help to maintain the strongly twisted conformation, with **DPA-MeTRZ** serving as an uncyclized reference compound (Figure 32).^272^ **DPA-MeTrz**, **c-NN-TRZ**, and **c-NN-MeTrz** emit at λ_PL_ of 466, 476, and 467 nm respectively, and each have unity Φ_PL_. Moderately large Δ*E*_ST_ values of between 0.24–0.32 eV led to τ_d_ on the millisecond timescale though. OLEDs with 12 wt% of **c-NN-TRZ** or **DPA-MeTRZ** doped in mCPCN film showed EQE_max_ of 26.3 and 19.1%, respectively (Table S1), while the device incorporating **c-NN-MeTRZ** showed the highest EQE_max_ of 32.2% which was attributed to the more horizontally oriented TDMs. However, all the devices suffered from severe efficiency roll-off of between 59–70% at 500 cd m^–2^, likely a consequence of the long τ_d_.

**Figure 32:**
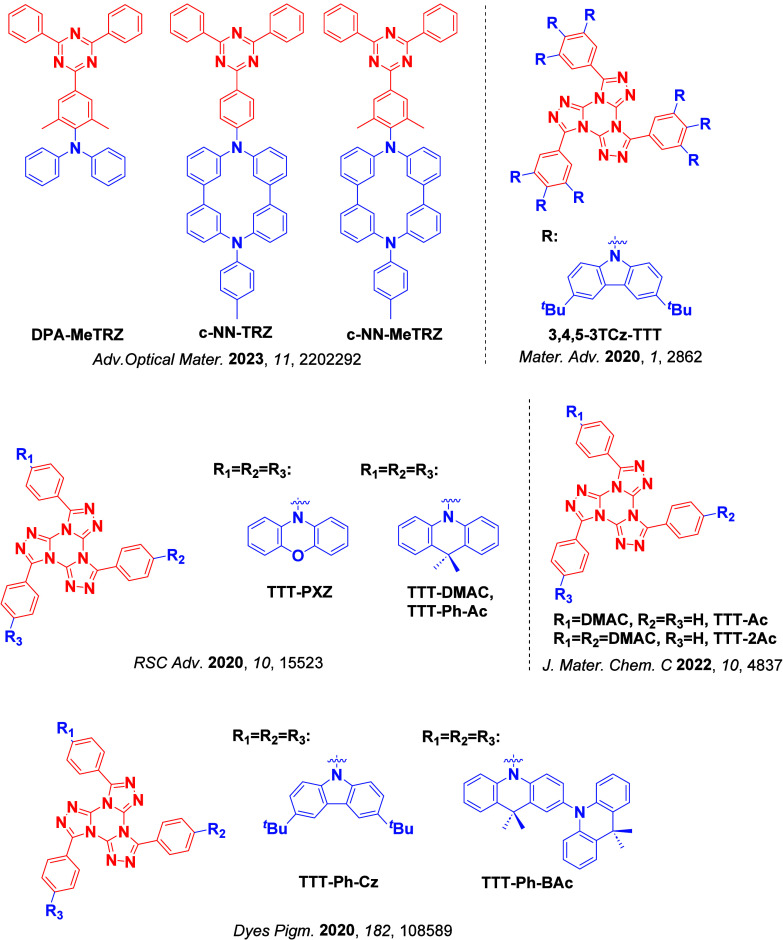
Structures of blue TADF emitters featuring donor macrocycle-substituted TRZ acceptor moieties or tristriazolotriazines as acceptors (the blue color signifies donor moieties, while the red color signifies acceptor moieties).

In 2020 three research groups simultaneously reported tristriazolotriazine (TTT) TADF derivatives, combined with various donors.^273–275^ TTT is an extended and planar 1,3,5-triazine derivative, and hence was originally used for the design of discotic liquid crystals.^276^ The first reported TTT-based TADF compounds were triply substituted with either carbazole (**TTT-Ph-Cz**), DMAC (**TTT-DMAC**), PXZ (**TTT-PXZ**), or biacridine (**TTT-Ph-Bac**) moieties, or instead substituted with 9 carbazoles (**3,4,5-3TCz-TTT**) (Figure 32).^273,275^ The highest solid-state Φ_PL_ were reported for **TTT-DMAC** and **3,4,5-3TCz-TTT**, reaching values of 79 and 80% respectively, while phenoxazine-based green **TTT-PXZ** has a more moderate Φ_PL_ of 39% and the triply substituted carbazole derivative **TTT-Ph-Cz** has a Φ_PL_ of only 42%. **TTT-DMAC** in 5 wt% doped films in CzSi films has a τ_d_ of 4.6 μs (Δ*E*_ST_ = 0.20 eV), while in 3 wt% DPEPO it was separately reported to have much longer τ_d_ of 142 μs (Δ*E*_ST_ = 0.24 eV), and even longer in 15 wt% CzSi at 4.7 ms (Δ*E*_ST_ = 0.27 eV) (Table S1). The inclusion of additional Cz donors in **3,4,5-3TCz-TTT** resulted in a much smaller Δ*E*_ST_ of 0.21 eV, compared to 0.43 eV for **TTT-Ph-Cz**, which did not show any delayed fluorescence in its transient PL. **3,4,5-3TCz-TTT** instead showed a τ_d_ of 3.1 ms in 15 wt% doped film in CzSi. **TTT-Ph-Bac** demonstrated only a moderate Φ_PL_ at 32%, but also the smallest Δ*E*_ST_ of 0.09 eV among the TTT derivatives reported in 2020. Solution-processed OLEDs with green **TTT-PXZ** showed a moderate EQE_max_ of 6.2%, while the device with **TTT-DMAC** achieved only an EQE_max_ of 1.9% at λ_EL_ of 480 nm. The **TTT-DMAC** device was substantially improved by adding a PVK layer, which possibly helped to better confine the excitons and raised the EQE_max_ to 11%. The device with **3,4,5-3TCz-TTT** showed an EQE_max_ of 5.8%, while the device with purely fluorescent derivative **TTT-Ph-Cz** showed an even lower EQE_max_ of 3.3%.

Recently Fang *et al*. reported asymmetrical singly or double substituted TTT derivatives, **TTT-Ac** and **TTT-2Ac** (Figure 32).^277^ The two compounds emit at λ_PL_ of 468 and 471 nm and have moderate Φ_PL_ of 63 and 47%, respectively. Introduction of the extra donor in **TTT-2Ac** results in a much smaller Δ*E*_ST_ (0.19 eV) compared to **TTT-Ac** (0.35 eV), with the smaller Δ*E*_ST_ translating to a shorter τ_d_ of 20 μs compared to **TTT-Ac** (27 μs) (Table S1). Solution-processed OLEDs with **TTT-Ac** and **TTT-2Ac** emitting at λ_EL_ of 470 and 474 nm [CIE coordinates of (0.16, 0.21) and (0.17, 0.26)] showed EQE_max_ of 9.2 and 8.1%, respectively.

In summary, the nature of the donor can have a strong influence on the photophysical and device properties of triazine-based TADF emitters, as is evident in the comparison of previously discussed **Tspiro­S-TRZ** and **Tris-Cz-TRZ** (Figure 33). Rigid and sterically twisted D-A structures lead to small Δ*E*_ST_ and efficient TADF with short excited-state lifetimes, that often translate into high-efficiency OLEDs. However, OLEDs employing these emitters are frequently sky-blue at best, with CIE_y_ coordinates far from Rec. 2020 standard for blue. A handful of examples do show deep-blue emission with CIE_y_ coordinates of < 0.1, however OLEDs utilizing such emitters as **DCB­TRZ** struggle to achieve efficiencies exceeding 10%, and the efficiency roll-off remains high.

**Figure 33:**
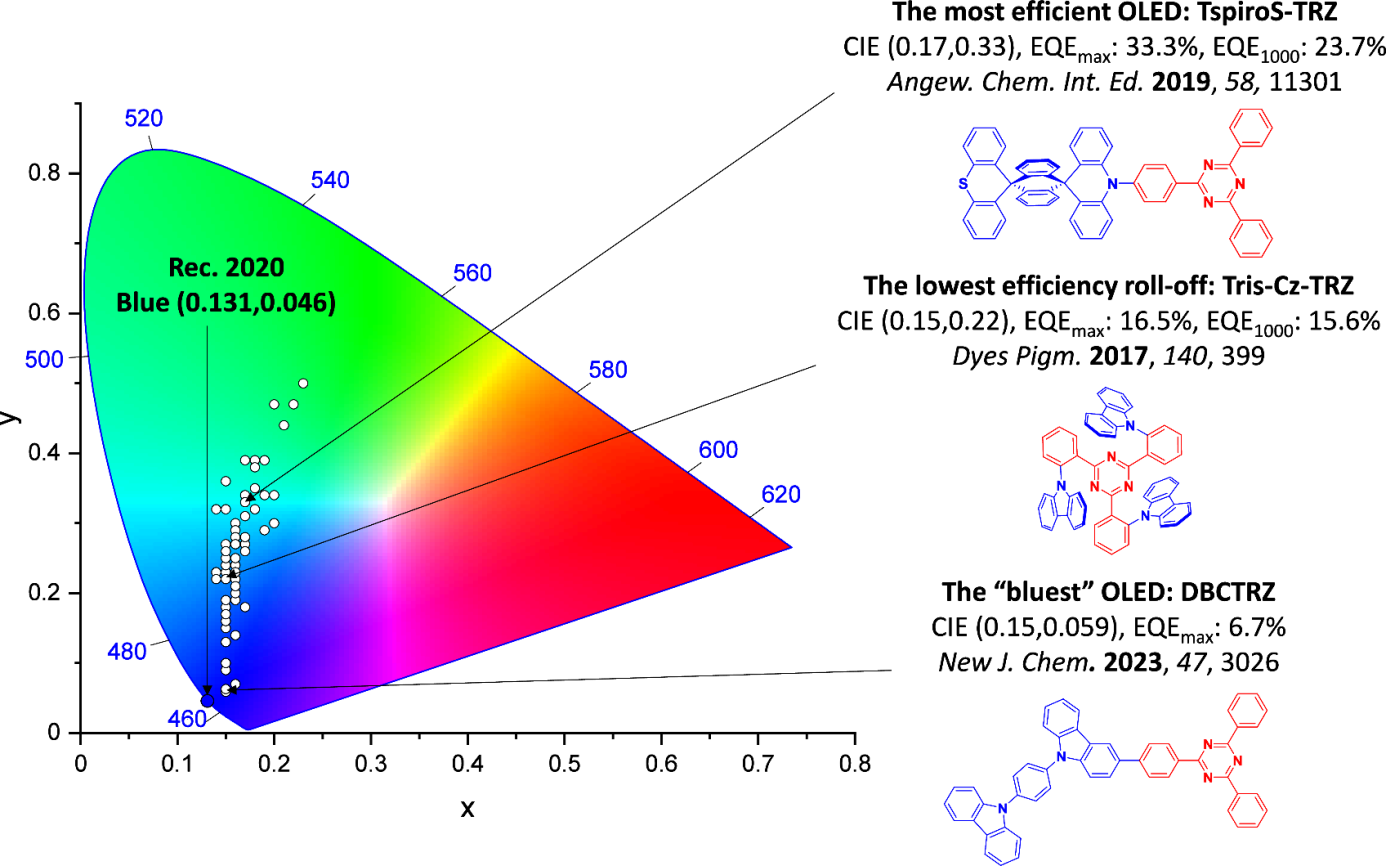
CIE color coordinates of blue D-A TADF emitters containing triazine acceptors. The white circles illustrate the spread of the emission color of the device. Selected devices and their associated CIE coordinates are highlighted illustrating the structure of the emitter of the “bluest” device, the structure of the emitter used in the device showing the highest EQE_max_ and the structure of the emitter associated with the device showing the lowest efficiency roll-off. Only TADF OLEDs where the λ_EL_ *<* 490 nm are included. The device with the CIE coordinates closest to the Rec. 2020 defined coordinates for blue, (0.131, 0.046), is defined as the “bluest”. The most efficient device is quantified by the highest EQE_max_. The efficiency roll-off is quantified as the change in efficiency between EQE_max_ and EQE_1000_. In the chemical structures, the blue color signifies donor moieties, while the red color signifies acceptor moieties.

### 3.4 Other Nitrogen Heterocycles: Pyrazine- and Pyrimidine-Containing Emitters

Similar to TRZ, a variety of blue TADF emitters have been designed using pyrimidine and pyrazine as acceptor moieties. These possess shallower LUMOs than TRZ, hence are weaker electron acceptors and thus compatible with a wider range of (stronger) donors while maintaining blue emission.^278^ The 2,4,6-positions of the pyrimidine ring and 2,3,5,6-positions of the pyrazine ring are also easily functionalized, which adds to the attractiveness of these heterocycles in the construction of blue TADF emitters. The pyrimidine and pyrazine-based blue TADF emitters discussed here are shown in Figure 34.

**Figure 34:**
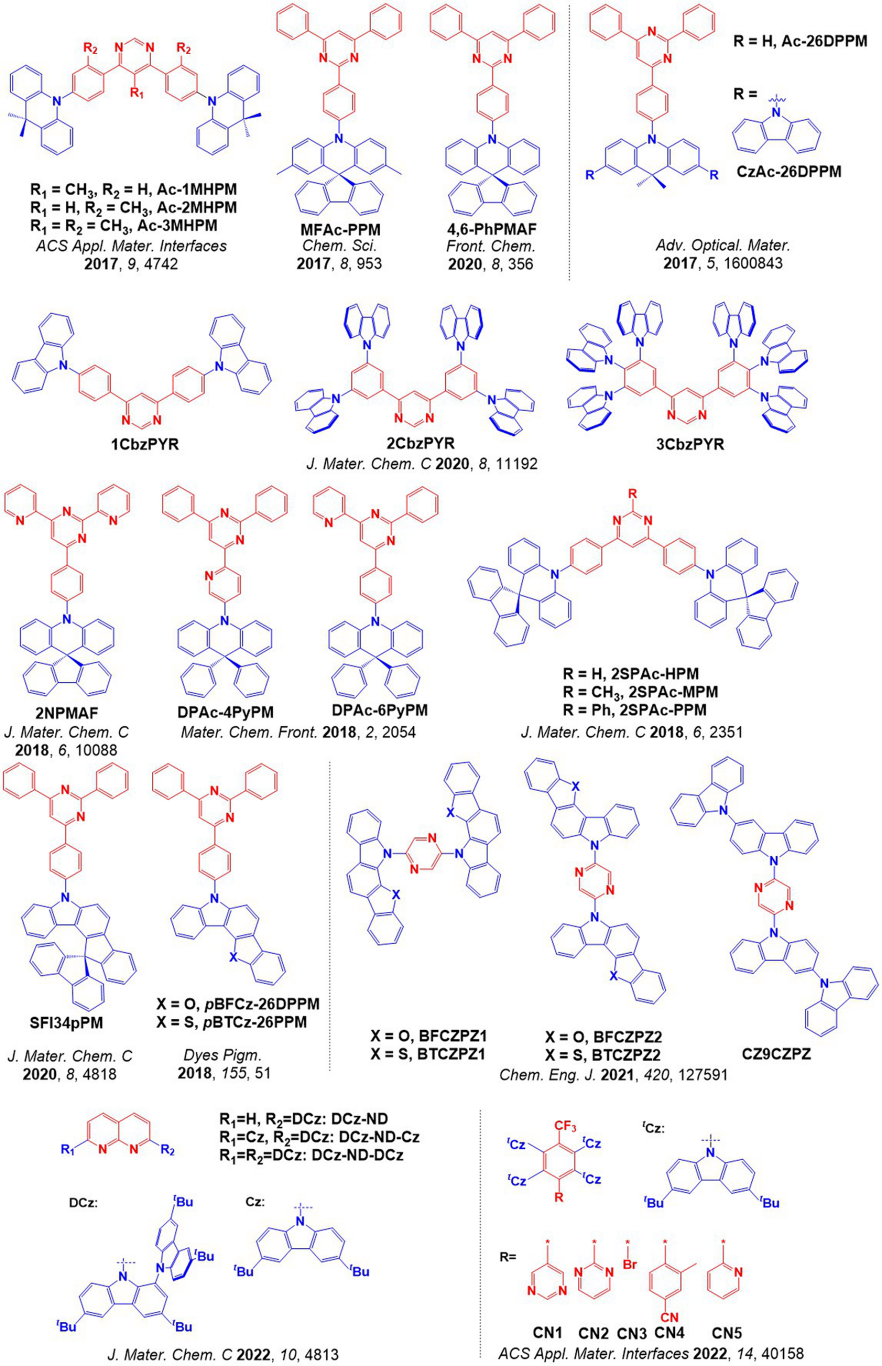
Molecular structures of pyrimidine- and pyrazine-based blue TADF emitters (the blue color signifies donor moieties, while the red color signifies acceptor moieties).

The introduction of methyl groups around a pyrimidine acceptor unit leads to high torsions between the pyrimidine and adjacent phenylenes, causing an increase in the excited-state energies and producing deep-blue emission in combination with DMAC donors.^279^ Moving from one central (**Ac-1MHPM**) to two symmetric methyl groups (**Ac-2MHPM**, Figure 34) resulted in limited changes to the photophysics (λ_PL_ = 481 and 477 nm, with Φ_PL_ = 75 and 71%, respectively). The conjugation was dramatically reduced with the introduction of three methyl groups though (**Ac-3MHPM**), affording a more twisted structure with the λ_PL_ blue-shifted to 454 nm and the Φ_PL_ decreased to 47% (Table S1). However, the number of methyl groups had surprisingly little impact on the TADF properties, with τ_d_ ranging between 44 and 50 ms and Δ*E*_ST_ between 0.22–0.24 eV for the three compounds. The OLEDs with **Ac-1MHPM**, **Ac-2MHPM**, and **Ac-3MHPM** showed EQE_max_ values of 24.0, 19.8, and 17.8%, respectively, and the EL reflected in the λ_PL_ values with the **Ac-3MHPM** device having CIE coordinates of (0.15, 0.16) compared to (0.15, 0.27) and (0.15, 0.28) for the devices with **Ac-1MHPM** and **Ac-2MHPM**, respectively. A large efficiency roll-off was observed for each of the devices, with the bluest device using **Ac-3MHPM** unable to reach 1000 cd m^–2^.

When using the less sterically bulky donor carbazole, both the position and number of donors must be carefully optimized to achieve efficient TADF. Serevičius *et al*. employed a symmetric bis(phenyl)pyrimidine acceptor in this way, and when carbazole groups were substituted at the *para* positions the resulting **1Cbz­PYR** (Figure 34) possessed a large Δ*E*_ST_ of 0.48 eV and showed no TADF in 1 wt% doped films in PMMA.^280^ When carbazole groups were instead substituted at the *meta* positions as in **2Cbz­PYR**, a smaller Δ*E*_ST_ of 0.27 eV was achieved but with a low Φ_PL_ of 22% (Table S1). Compound **3Cbz­PYR** featuring a full set of *meta* and *para* substituted carbazoles has a much higher Φ_PL_ of 81%, benefitting from both reduced non-radiative decay and enhanced radiative decay. The OLEDs with **3Cbz­PYR** emitted at λ_EL_ of 464 nm [CIE coordinates of (0.16, 0.23)] and showed EQE_max_ of 19.7%. Although the maximum luminance approached 10,000 cd m^–2^, severe efficiency roll-off of 55% at 100 cd m^–2^ was observed due to the large Δ*E*_ST_ of 0.32 eV.

Li *et al*. reported three D-A-D structures using various pyrimidine acceptors coupled to spiro-acridine donors, which exhibited good performance in OLED devices.^281^ The pyrimidine acceptors were either unsubstituted (**2SPAc-HPM**), methyl substituted (**2SAPAc-MPM**), phenyl substituted (**2SPAc-PPM**, Figure 34), and showed moderate Δ*E*_ST_ between 0.15 and 0.19 eV with high Φ_PL_ ranging from 82–97%. The devices consequently showed high EQE_max_ values of 25.6, 24.3, and 31.5%, respectively; however, severe efficiency roll-off of 26, 34, and 43% was reported at 100 cd m^–2^ arising from the long τ_d_ (52–57 ms). Another spiro-acridine donor with methyl groups at the 2,7-positions was instead coupled between the heteroatoms of diphenyl pyrimidine to give **MFAc-PPM**. This emitter has a moderately high Δ*E*_ST_ of 0.25 eV and of τ_d_ of 78 μs in 18 wt% doped films in PPF, yet was nonetheless able to produce sky blue OLEDs with CIE coordinates of (0.16, 0.23), an EQE_max_ of 20.4%, and reasonable efficiency roll-off of 24% at 100 cd m^–2^ (Table S1).^282^ A corresponding analogue without methyl groups on the donor, **4,6-PhPMAF**, gave deep-blue emission in a device using 22 wt% doping in DPEPO, with λ_EL_ of 458 nm and CIE coordinates of (0.15, 0.11).^283^ The device however showed an EQE_max_ of only 3% due to the low Φ_PL_ of 17%, while the efficiency roll-off associated with the relatively large Δ*E*_ST_ of 0.27 eV and long τ_d_ of 0.3 ms was so severe that even 200 cd m^–2^ was not achieved. **Ac-26DPPM** and **CzAc-26DPPM** feature asymmetric substitution of the pyrimidine acceptor with acridine-based donors, and in 10 wt% doped films in DPEPO exhibited sky-blue emission with λ_PL_ of 476 and 496 nm, respectively. **Ac-26DPPM** and **CzAc-26DPPM** both have Φ_PL_ of 81%, and τ_d_ of 87 and 55 μs in the same DPEPO. The OLEDs with **Ac-26DPPM** and **CzAc-26DPPM** showed sky-blue emission at CIE of (0.18, 0.32) and (0.21, 0.37), EQE_max_ of 19.3 and 23.7%, and efficiency roll-offs of 67 and 60% at 1000 cd m^–2^, all respectively.^284^

Decorating pyrimidine with pyridines in conjunction with spiro-acridine donors afforded efficient sky-blue TADF emitters **2NPMAF**, **DPAc-4PyPM**, and **DPAc-6PyPM** (Figure 34). Higher Φ_PL_ (>80%) and faster *k*_RISC_ (∼10^5^ s^–1^) were observed for **DPAc-4PyPM** and **DPAc-6PyPM** in comparison to reference emitter **DPAc-TPPM** (Φ_PL_ = 70%, *k*_RISC_ = 6.9 × 10^4^ s^–1^) without pyridines.^285^ Intramolecular H-bonding between the pyridine units and the pyrimidine core was suggested as responsible, however its significance in relation to the TADF mechanism was not apparent. The devices with **2NPMAF**, **DPAc-4PyPM**, and **DPAc-6PyPM** showed EQE_max_ of 23.6, 24.3, and 22.4% at λ_EL_ of 481, 484, and 472 nm, respectively (Table S1).^286^

Again, featuring pyrimidine, **SFI34­pPM** (Figure 34) features a sterically hindered spiro-fluorene-fused carbazole derivative as the electron donor, leading to internal rigidity, excellent thermal stability, and a Φ_PL_ of 74% in 10 wt% doped films in DPEPO. A deep-blue device with **SFI34p­­PM** showed CIE_y_ of 0.09 and an EQE_max_ of 8.2%.^287^ Another family of emitters containing asymmetric pyrimidine acceptors coupled to functionalized carbazoles also produced deep-blue OLEDs.^288^ Benzo­furo- and ben­zo­thieno- carbazoles were used as donors in ***p*BFcz-2,6DPPM** and ***p*BTCz-2,6DPPM**, which emit similarly at λ_PL_ = 437 and 435 nm with Φ_PL_ of 71 and 75%, respectively. However, long τ_d_ of 200 and 383 μs were measured due to large Δ*E*_ST_ of 0.27 and 0.34 eV, also respectively (Table S1). The OLEDs using 10 wt% doping in DPEPO host retained identical deep-blue emission with CIE coordinates of (0.15, 0.05), and EQE_max_ of 6.2 and 5.4% respectively. The efficiency roll-off was severe though, with neither emitter able to achieve 1000 cd m^–2^ and only **pBFCz-2,6DPPM** able to reach 100 cd m^–2^ (with efficiency roll-off of ∼29% at that brightness). Non-doped devices with **pBFcz-2,6DPPM** and **pBTCz-2,6DPPM** showed a slight red-shift in the emission (CIE_y_ shifting to 0.07), with EQE_max_ of 5.8 and 5.4% and both achieving brightness of over 3000 cd m^–2^.

The effect of heteroatoms on spin-orbit coupling (SOC) between S_1_ and T_1_ states was investigated within a series of pyrazine-based TADF emitters bearing donors of benzofuran fused carbazole (**BF­CZ­PZ1** and **BF­CZ­PZ2**), benzothiophene carbazole (**BT­CZ­PZ1** and **BT­CZ­PZ2**), or a 9-bicarbazole (**CZ9­CZ­PZ**, Figure 34).^289^ **BT­CZ­PZ1** possesses the smallest Δ*E*_ST_ (0.24 eV), while the others have Δ*E*_ST_ values range between 0.31–0.37 eV (Table S1). The Φ_PL_ of **BF­CZ­PZ1** is 68%, while for the other emitters it is above 91%, all in 7 wt% doped films in PPF. TD-DFT calculation showed that in emitters with shorter distances between the donor heteroatoms and the acceptor moiety the SOC between the S_1_ and T_1_ states was enhanced. Accordingly, the spin-orbital coupling matrix elements between S_1_ and T_1_ of **BF­CZ­PZ1** and **BT­CZ­PZ1** were 0.311 and 0.980 cm^–1^, compared to 0.122, 0.149, and 0.252 cm^–1^ for **BF­CZ­PZ2**, **BT­CZ­PZ2**, and **CZ9­CZ­PZ**, all respectively. As a result, **BT­CZ­PZ1** showed the shortest τ_d_ of 90 μs and fastest k_RISC_ of 8.5 × 10^4^ s^–1^ of this series. The device with **BF­CZ­PZ1** showed the bluest emission with λ_EL_ of 436 nm and CIE coordinates of (0.15, 0.06), while the λ_EL_ for the devices with **BF­CZ­PZ2**, **BT­CZ­PZ1**, **BT­CZ­PZ2**, and **CZ9­CZ­PZ**, were shifted to 464, 472, 468, and 468 nm, respectively. The EQE_max_ of the device with **BF­CZ­PZ1** was 6.5% due to the lower Φ_PL_, while the EQE_max_ for **BF­CZ­PZ2**, **BT­CZ­PZ1**, **BT­CZ­PZ2**, and **CZ9­CZ­PZ**, were much improved at 21.3, 21.1, 19.7, and 20.0%, respectively. The devices with **BF­CZ­PZ1** and **BT­CZ­PZ1** showed the best efficiency roll-off of 20 and 26% at 100 cd m^–2^, while for the other three devices the efficiency roll-off was around 50% at the same brightness level.

Banevičius *et al*. reported a series of naphthyridine-carbazole hybrids with particular focus on asymmetric derivative **DCz-ND-Cz** (Figure 34). This emitter which showed a shortened τ_d_ (4.4 μs), faster *k*_RISC,_ and a high Φ_PL_ of 74% compared to the singly substituted analogue **DCz-ND** and symmetric congener **DCz-ND-DCz** (τ_d_ of 6 and 7μs, Φ_PL_ of 46 and 72%, respectively), all in 20 wt% doped films in DPEPO (Table S1).^290^ The OLEDs showed comparable performance with EQE_max_ ranging between 18.1–20.8% at λ_EL_ of 464–469 nm, however the device with **DCz-ND-Cz** demonstrated the smallest efficiency roll-off of 53% at 1000 cd m^–2^ compared to 67% for **DCz-ND-DCz** and 70% for **DCz-ND**.

Mahmoudi *et al*. reported a series of multi­car­ba­zole derivatives in the structural template of **4CzTPN** featuring various electron-acceptor moieties and a common trifluoromethyl substitutent.^291^ The bluest compounds were **CN1** and **CN4** (Figure 34), which in neat films emitted sky-blue at λ_PL_ of 482 and 490 nm, and have Φ_PL_ 76 and 27%, respectively. Short τ_d_ were reported for these emitters (2.4 and 1.8 μs), accompanied by small Δ*E*_ST_ values of 0.03 and 0.04 eV. Non-doped devices achieved sky-blue emission with λ_EL_ of 481 and 476 nm [CIE coordinates (0.16, 0.27) and (0.17, 0.24)] and EQE_max_ of 8.4 and 5.5% respectively. These EQEs nearly doubled when a doped device architecture (20 wt% in mCBP) was used.

Of these examples, the best non-triazine nitrogen-heterocycle blue TADF emitters all feature pyrimidine (Figure 35). Sky-blue-emitting OLEDs achieved efficiencies as high as 31% with **2SPAc-PPM**, while the OLEDs with the lowest efficiency roll-off employed **2NPMAF** featuring the same spiro-donor. In terms of color, the OLED with **pBFCz-2,6DPPM** most closely approaches the target CIE coordinates of Rec. 2020 standard, however poor efficiency is still unavoidable in this color region.

**Figure 35:**
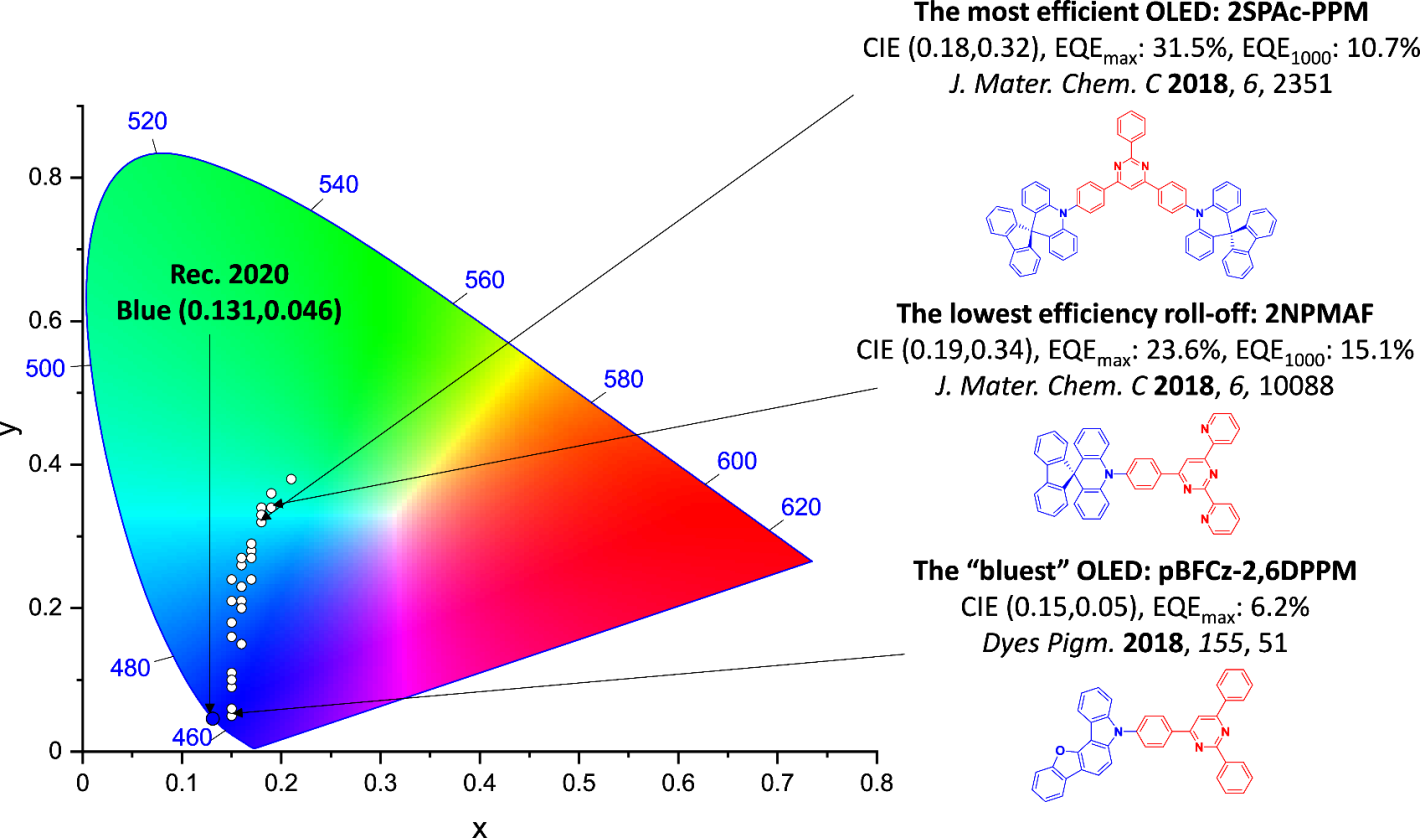
CIE color coordinates of blue D-A TADF emitters containing nitrogen heterocycle acceptors. The white circles illustrate the spread of the emission color of the device. Selected devices and their associated CIE coordinates are highlighted, illustrating the structure of the emitter of the “bluest” device, the structure of the emitter used in the device showing the highest EQE_max_ and the structure of the emitter associated with the device showing the lowest efficiency roll-off. Only TADF OLEDs where the λ_EL_ *<* 490 nm are included. The device with the CIE coordinates closest to the Rec. 2020 defined coordinates for blue, (0.131, 0.046), is defined as the “bluest”. The most efficient device is quantified by the highest EQE_max_. The efficiency roll-off is quantified as the change in efficiency between EQE_max_ and EQE_1000_. In the chemical structures, the blue color signifies donor moieties, while the red color signifies acceptor moieties.

### 3.5 Boron-Containing Emitters

The use of boron as an acceptor has been widely reported in the literature.^226,292^ Generally tri- or tetra- substituted boron acceptors are decorated with donors to form D-A TADF emitters, although recent boron-containing MR-TADF emitters^118,293^ are discussed separately in Section 11. The configuration of tri-substituted boron acceptors can be classified as either fully fused (a boron atom directly attached to three contiguous aryl units) or unfused (a boron atom directly connected to at least one isolated aryl group). Examples of tetra-substituted boron acceptors are typically composed of a BF_2_ group linked to aryl units.

As an early and structurally simple example, the *ortho* regiochemistry in **Cz*o*B** (Figure 36) resulted in the Cz donor adopting a twisted conformation that produces a significantly smaller Δ*E*_ST_ than the equivalent *para*-congener, with Δ*E*_ST_ of 0.15 and 0.39 eV for **Cz*o*B** and **Cz*p*B**, respectively, in toluene. **Cz*o*B** emits at λ_PL_ of 463 nm in toluene and has Φ_PL_ of 84% in 20 wt% doped films in DPEPO. The corresponding OLED showed an EQE_max_ of 22.6% with CIE coordinates of (0.14, 0.15), although the long τ_d_ of 56.3 μs resulted in large efficiency roll-off of 19 and 77% at 100 and 1000 cd m^–2^.^294^

**Figure 36:**
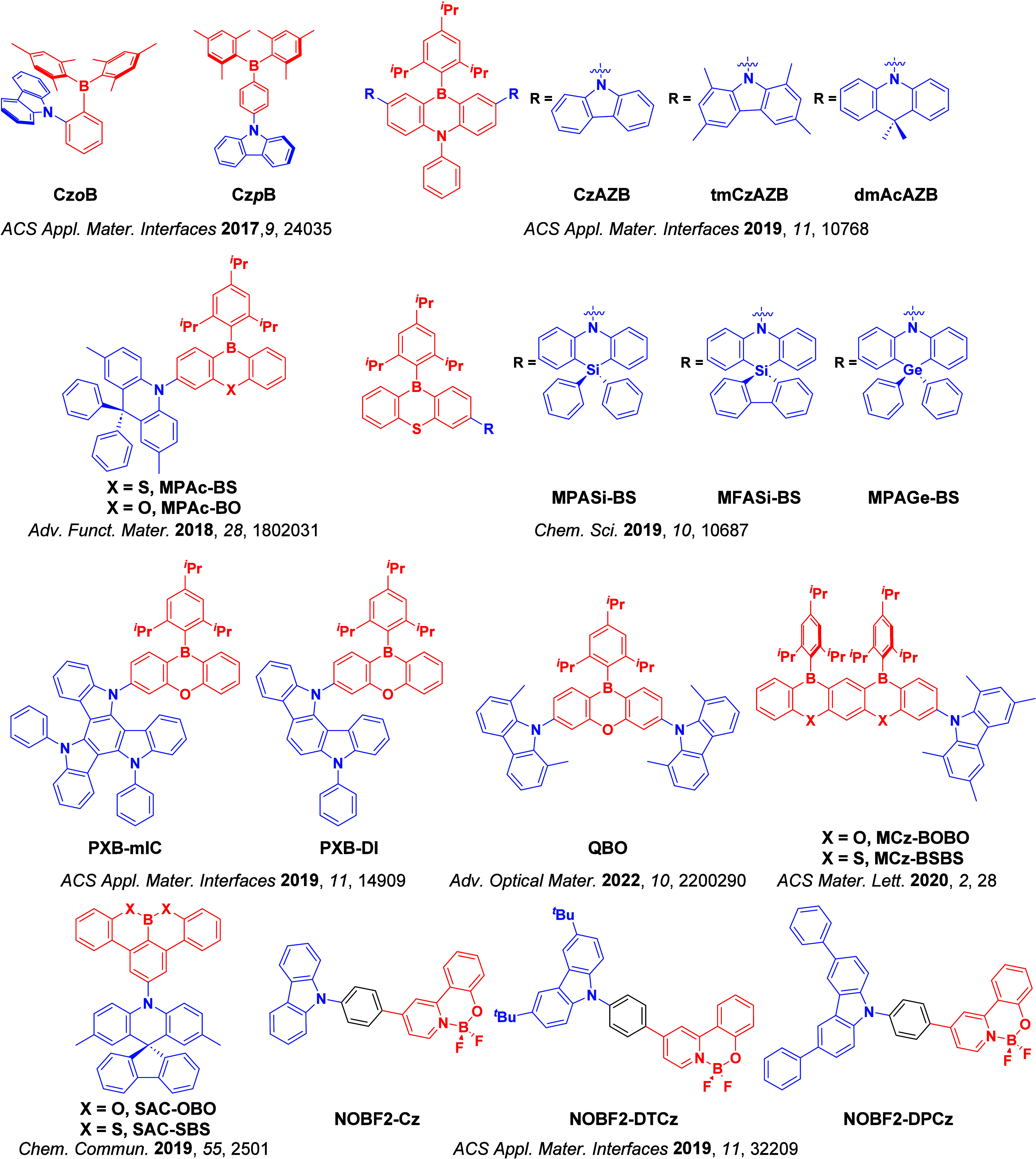
Structures of blue TADF emitters featuring unfused boron acceptors (the blue color signifies donor moieties, while the red color signifies acceptor moieties).

Using unfused boron acceptor dibenzo-1,4-azaborine, a series of three emitters were designed bearing DMAC (**dmAcAZB**), tetra­meth­yl­carba­zole (**tmCzAZB**), and carbazole donors (**CzAZB**, Figure 36).^117^ Forcing a near-orthogonal D-A conformation was key to promoting RISC and TADF, and indeed **CzAZB** displayed no delayed fluorescence due to its more planarized structure and large Δ*E*_ST_ of 0.31 eV compared to 0.26 and 0.11 eV for **tmCzAZB** and **dmAcAZB**, respectively. **CzAZB**, **tmCzAZB**, and **dmAcAB** emit at λ_PL_ of 452, 451, and 469 nm, and although **CzAZB** exhibited an excellent Φ_PL_ of 99% its lack of TADF resulted in poor device EQE_max_ of 5.5%. **tmCzAZB** and **dmAcAB** have τ_d_ of over 150 μs, but were nonetheless able to achieve EQE_max_ of 12.4 and 20.8% in 10 wt% doped mCP host. The emission of these latter two was at λ_EL_ of 464 and 469 nm with CIE coordinates of (0.14, 0.15) and (0.14, 0.19), but significant efficiency roll-off at 100 cd m^–2^ of 56 and 38% was reported, all respectively.

**MPAc-BS** and **MPAc-BO** (Figure 36) contain bulky diben­zo­hetera­borin acceptors containing either sulfur or oxygen atoms, connected to a dimeth­yl­di­phen­yl­acri­dine (MPAc) donor.^295^ **MPAc-BS** and **MPAc-BO** emit at λ_PL_ 481 and 466 nm and have high Φ_PL_ values of 99 and 98% respectively, as neat films. Along with suppression of ACQ to support these Φ_PL_, the neat films also have short τ_d_ of 1.7 and 2.4 μs and fast *k*_RISC_ of 3.5 and 1.0 × 10^6^ s^–1^, all respectively. The sulfur atom in **MPAc-BS** was proposed to enhance the SOC, resulting in faster *k*_RISC_ than in **MPAc-BO**. Non-doped devices with **MPAc-BS** and **MPAc-BO** showed EQE_max_ of 22.8 and 21.3%, emitting at λ_EL_ of 487 and 474 nm [CIE coordinates of (0.15, 0.36) and (0.14, 0.23)], respectively. Additionally, the devices showed low efficiency roll-off of 1 and 14% at 100 cd m^–2^, respectively. The rigid nature of these sterically crowded emitters helps to explain both the high Φ_PL_, resistance to ACQ, and narrow emission FWHM of 63 and 59 nm.

Matsuo *et al.* have also explored incorporating heavy atoms in an effort to boost SOC and thus *k*_RISC_. A family of blue emitters containing pheno­thia­borin (BS) as the acceptor was developed using acridan-analogue donors, whereby the bridging carbon atom of the donor is substituted by silicon or germanium atoms. Compounds **MPASi-BS**, **MFASi-BS**, and **MPAGe-BS** (Figure 36) emit at λ_PL_ of 479, 483, and 468 nm, respectively, in 50 wt% doped films in PPF.^296^ The closely lying ^1^CT, ^3^CT, and ^3^LE states along with heavy atom effects combined to improve the SOC between the singlet and triplet states, and thus accelerate RISC. The emitters involving a donor heavy atom (**MPASi-BS**, **MFASi-BS**, and **MPAGe-BS**) all exhibited *k*_RISC_ above 1 × 10^7^ s^–1^ in doped PPF film, much faster than the green-emissive reference material **MPAc-BS** ^297^ (3.5 × 10^6^ s^–1^). In addition to the fast *k*_RISC_, the high Φ_PL_ (close to 100%), and moderate Δ*E*_ST_ (<0.11 eV) of **MPASi-BS**, **MFASi-BS**, and **MPAGe-BS** allowed them to support strong performance in OLEDs. The devices with **MPASi-BS** and **MFASi-BS** showed sky blue emission with λ_EL_ of 478 and 484 nm, CIE coordinates of (0.14, 0.26) and (0.14, 0.32), and EQE_max_ of 27.6 and 23.9% with only 5 and 8% efficiency roll-off at 1000 cd m^–2^, all respectively. Interestingly, for **MPAGe-BS** the excitons were initially generated on high energy quasi-axial (QA) conformers, requiring subsequent energy transfer to the lower energy emissive quasi-equatorial (QE) conformer. As a result, the device EQE_max_ was diminished to 15.7% (16% efficiency roll-off at 1000 cd m^–2^).

Combining rigid diindolocarbazole or indolocarbazole donors with a dibenzooxaborin acceptor afforded the efficient blue TADF emitters **PXB-DI** and **PXB-mIC** (Figure 36).^298^ As 20 wt% doped films in PPBI, **PXB-DI** has a higher Φ_PL_ of 79%, faster *k*_RISC_ of 1.17 × 10^6^ s^–1^ and a red-shifted emission of λ_PL_ = 470 nm compared to **PXB-mIC** (Φ_PL_: 51%, *k*_RISC_: 5.22 × 10^5^ s^–1^, and λ_PL_ = 425 nm). The Δ*E*_ST_ values are 0.09 and 0.19 eV for **PXB-DI** and **PXB-mIC**, respectively. The enhanced TADF properties of **PXB-DI** than **PXB-mIC** were attributed by the authors to the stronger donor strength and extended rigid structure of diindolocarbazole. The OLEDs with **PXB-mIC** showed an EQE_max_ of 12.5% at CIE coordinates of (0.15, 0.08), although the efficiency roll-off (58% at 1000 cd m^–2^) was rather severe. The devices with **PXB-DI** instead showed sky-blue emission at CIE coordinates of (0.16, 0.34) and very high EQE_max_ of 37.4%, with only a 15% roll-off of the efficiency at 1000 cd m^–2^.

Quadrupolar D-A-D blue TADF emitter **QBO** (Figure 36) was designed using the same phenoxaborin acceptor and 1,8-dimethylcarbazole donors.^299^ The key to this design strategy was to generate doubly degenerate CT excited states associated with the two separate donor units, which would enhance the density of excited states and SOC – and indeed a high SOCME of 0.41 cm^–1^ was calculated for **QBO**. The λ_PL_, Δ*E*_ST_, Φ_PL_, *τ*_d_, and *k*_RISC_ values are 455 nm, 0.01 eV, 83%, 0.65 μs, and 19×10^5^ s^–1^ in 20 wt% doped films in PPF. The resulting OLEDs emitted at λ_EL_ at 460 nm with CIE coordinates of (0.14, 0.12) and EQE_max_/EQE_1000_ of 20.5 and 17.7%. Similar derivatives **MCz-BOBO** and **MCz-BSBS** featured an acceptor extended with additional boron-oxygen/boron-sulfur moieties.^300^ These blue emitters, employing a ladder-shaped heteraborin acceptor and tetramethyl carbazole as the donor, achieved sufficiently separated HOMO/LUMO for small Δ*E*_ST_. Indeed, **MCz-BSBS** and **MCz-BOBO** emit at λ_PL_ of 483 and 476 nm and have Δ*E*_ST_ of 0.17 and 0.01 eV, along with Φ_PL_ of 93 and 100% and τ_d_ of 2.7 and 0.78 μs in 20 wt% doped films in PPF, all respectively. TD-DFT calculations revealed similar excited states topologies for the two emitters, with the closely lying S_1_ and T_1_ states showing similar CT character while the slightly higher T_2_ states exhibited LE character. The SOCME value between El-Sayeed-allowed T_2_ and S_1_ in the sulfur-containing **MCz-BSBS** (2.93 cm^–1^) was more than 30 times higher than the value for oxygen-containing **MCz-BOBO** (0.09 cm^–1^), demonstrating the impact of heavy-atom effects in this context. These calculations aligned well with the faster experimental *k*_RISC_ of **MCz-BSBS** (8.8 and 2.5 × 10^6^ s^–1^). The OLEDs with **MCz-BSBS** and **MCz-BOBO** emitted with CIE coordinates of (0.14, 0.33) and (0.13, 0.20), and showed EQE_max_ of 25.9 and 20.1% with low efficiency roll-offs of 25 and 12% at 1000 cd m^–2^, all respectively.

An unusual boron-containing acceptor with thioether linking groups produced efficient sky-blue emitter **SAC-SBS** (Figure 36).^301^ In 20 wt% doped films in PPF **SAC-SBS** showed promising photophysical properties with λ_PL_ of 491 nm, Φ_PL_ of 81%, Δ*E*_ST_ of 0.12 eV, τ_d_ of 22 μs, and *k*_RISC_ of 32×10^5^ s^–1^ compared to its ether-linked analogue **SAC-OBO** (λ_PL_ of 470 nm, Φ_PL_ of 28%, Δ*E*_ST_ of 0.30 eV, τ_d_ of 140 μs, and *k*_RISC_ of 3.5 × 10^5^ s^–1^) (Table S1). Devices with **SAC-SBS** emitted at λ_EL_ of 489 nm [CIE coordinates of (0.17, 0.39)] and showed EQE_max_ of 20.9%. The efficiency roll-off was moderate, at 16% at 100 cd m^–2^. **SAC-OBO** was found to deactivate the boron center too much, producing a worse triplet harvester albeit accompanied by a blue-shift in the emission. The OLED with **SAC-OBO** consequently showed an EQE_max_ of only 5.2% at λ_EL_ of 471 nm and CIE coordinates of (0.16, 0.22).

Contrasting to the previous examples, a tetra-coordinated boron acceptor was used in conjunction with carbazole-based donors in **NOBF2-Cz**, **NOBF2-DTCz**, and **NOBF2-DPCz** (Figure 36).^302^ These emitters use boron difluoride (BF_2_) in the chelating acceptor 4-phenylpyridin-2-yl)phenol (PPyPOH) moiety, which increased the overall acceptor strength enough to enable TADF in these materials. Compounds **NOBF2-Cz**, **NOBF2-DTCz**, and **NOBF2-DPCz** emit at respective λ_PL_ of 449, 473, and 471 nm in toluene. In 10 wt% doped films in DPEPO they show high Φ_PL_ values (70 to 99%), moderately large Δ*E*_ST_ values (0.20 to 0.22 eV) and long *τ*_d_ (110 to 132 μs). The blue OLEDs with **NOBF2-Cz**, **NOBF2-DTCz**, and **NOBF2-DPCz** nonetheless showed EQE_max_ values of 11.0, 12.7, and 15.8% at CIE coordinates of (0.14, 0.16), (0.14, 0.21), and (0.14, 0.28), all respectively. This study hence demonstrated the utility of tetra-coordinated boron in the acceptors of D-A TADF emitters.

The fully fused triaryl-boron acceptor 5,9-dioxa-13b-boranaphtho[3,2,1-de]anthracene (DBA) has a triangulene shape, with Hirai *et al*. first developing the material for use in OLEDs.^303^ Later, a *tert*-butyl modified DBA as acceptor (TDBA) was joined with DMAC or diindolocarbazole as donors, giving blue-emitting materials **TDBA-Ac** and **TDBA-DI** (Figure 37).^304^ These compounds emit at λ_PL_ of 458 and 456 nm, have Δ*E*_ST_ of 0.06 and 0.11 eV in toluene, and high Φ_PL_ of 93 and 99% in 20 wt% doped films in DBFPA, all respectively. The device with **TDBA-Ac** in PPBI showed an EQE_max_ of 21.5% at CIE coordinates of (0.15, 0.06), with the rigid structure conferring a reasonably narrow emission FWHM of 48 nm. In contrast, the devices with **TDBA-DI** in either PPBI or DBFPO emitted with CIE coordinates of (0.14, 0.15) and (0.15, 0.28), achieving very high EQE_max_ values of 32.2 and 38.2% and small efficiency roll-off of 17 and 10% at 1000 cd m^–2^, respectively. The observed spectral shift to sky-blue in DBFPO host was attributed to its more polar nature compared to PPBI. The outstanding EL performance of **TDBA-DI** is supported by its almost unity Φ_PL_, fast *k*_RISC_ (1.1 × 10^6^ s^–1^ in DBFPO) and high horizontal TDM orientation (89% in DBFPO).^304^ Subsequently reported **DBA-DI** is a close analogue of **TDBA-DI** without *tert*-butyl groups on the DBA moiety. This compound showed improved electrochemical stability with higher bond dissociation energies, an important trait for device stability.^305^ **DBA-DI** emits at λ_PL_ of 467 nm in toluene (456 nm for **TDBA-DI**) and maintains a high Φ_PL_ (95.3% in mCBP-CN), small Δ*E*_ST_ (0.03 eV in toluene), short τ_d_ (1.25 μs in mCBP-CN), and fast *k*_RISC_ of 6.2 × 10^6^ s^–1^ (in mCBP-CN). The device with **DBA-DI** in mCBP-CN exhibited sky-blue emission with CIE coordinates of (0.16, 0.39). The device also achieved a high EQE_max_ of 28.1%, with only 1% of efficiency roll-off at 1000 cd m^–2^ and a maximum luminance as high as 126 200 cd m^–2^. The device lifetime (LT_50_) also reached 329 hours, running at an initial 1000 cd m^–2^. Longer device lifetime (540 h) was achieved when a mixed host of mCBP-CN:​DDBFT was adopted, however the emission color was slightly red-shifted with CIE coordinates of (0.17, 0.40).

**Figure 37:**
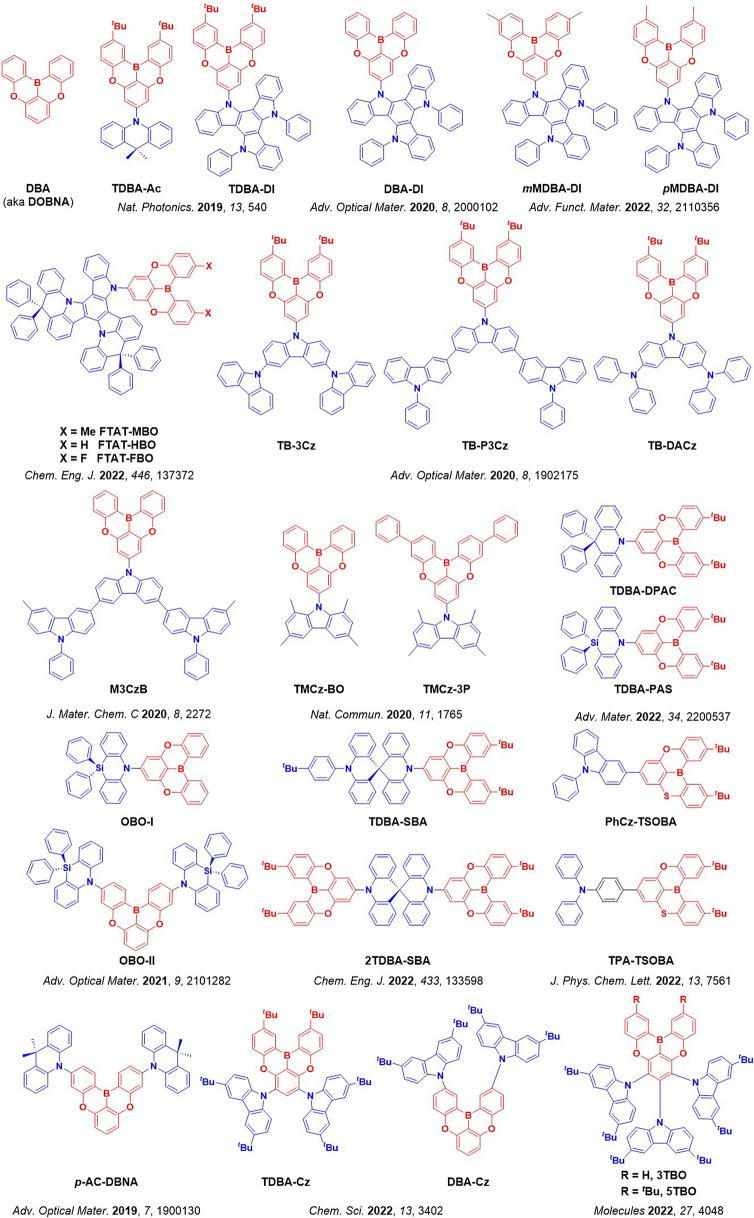
Structures of blue TADF emitters featuring fully fused boron acceptors (the blue color signifies donor moieties, while the red color signifies acceptor moieties).

In a subsequent study, the acceptor strength and CT character of **DBA-DI** ^305^ was manipulated by incorporating methyl groups *para* or *meta* to the oxygen atoms to afford ***p*MDBA-DI** and ***m*MDBA-DI** (Figure 37).^306^ This design strategy resulted in blue-shifted emission with λ_PL_ = 451 nm for ***m*MDBA-DI** and 460 nm for ***p*MDBA-DI**, while retaining moderately small Δ*E*_ST_ and short τ_d_ at 0.12 eV and 1.90 μs for ***m*MDBA-DI** and 0.07 eV and 1.60 μs for ***p*MDBA-DI** in toluene. Furthermore, near unity Φ_PL_ of 97.3 and 97.8% were recorded, respectively, for ***m*MDBA-DI** and ***p*MDBA-DI** in 30 wt% doped DBFPO films. The respective OLEDs with ***p*MDBA-DI** and ***m*MDBA-DI** showed EQE_max_ of 33.1 and 32.8% at λ_EL_ of 483 and 474 nm [CIE coordinates of (0.15, 0.31) and (0.14, 0.23)], with low efficiency roll-off of 2.4 and 13.4% at 1000 cd m^–2^. Additional blue TADF emitters **FTAT-MBO**, **FTAT-HBO**, and **FTAT-FBO** containing an intramolecular-locked triazatruxene (FTAT) donor moiety were designed, featuring DBA acceptors substituted with either methyl, hydrogen, or fluorine.^307^ The Δ*E*_ST_ values are 0.24, 0.21, 0.09 eV in toluene, with associated τ_d_ of 3.6, 3.5, 1.8 μs and Φ_PL_ of 55, 66, 90% in 20 wt% doped films in mCP, all respectively. The solution-processed OLEDs with **FTAT-FBO** emitted at λ_EL_ = 473 nm and CIE coordinates of (0.15, 0.25), and showed the best performance with an EQE_max_ of 17.5% which decreased only slightly to 17.3% at 1000 cd m^–2^.

Employing peripheral carbazole or diphenylamine substituents on the main carbazole donor leads to deep-blue to sky-blue solution-processable emitters **TB-3Cz**, **TB-P3Cz**, and **TB-DACz** (Figure 37).^308^ The emission of **TB-DACz** (λ_PL_ of 493 nm) is red-shifted compared to **TB-3Cz** (413 nm) and **TB-P3Cz** (433 nm) in toluene, which was attributed to the diphenylamine axillary donor strengthening the D-A interactions. The sufficiently separated HOMO/LUMO nonetheless enables small Δ*E*_ST_ values of 0.06, 0.11, and 0.07 eV respectively for **TB-3Cz**, **TB-P3Cz**, and **TB-DACz**. All the three emitters showed AIE behavior and high Φ_PL_ (>90%) in non-doped films, while the τ_d_ values were <10 μs. Solution-processed devices with **TB-3Cz** and **TB-P3Cz** showed deep-blue emission at λ_EL_ of 424 and 428 nm and [CIE coordinates of (0.17, 0.07) and (0.15, 0.08)], while the λ_EL_ of the **TB-DACz**-based device was red-shifted to 492 nm. The EQE_max_ for the solution-processed devices with **TB-3Cz** and **TB-3PCz** were as high as 9.9 and 6.1% respectively, but unfortunately the devices suffered from severe efficiency roll-off of ∼93 and 43% at 1000 cd m^–2^. Vacuum-deposited devices with **TB-P3Cz** showed a much higher EQE_max_ of 29.1% albeit still with 54% efficiency roll-off at 1000 cd m^–2^, along with LT_50_ of 60 h and blue emission at CIE coordinates (0.14, 0.19). By removing the *tert*-butyl groups from the acceptor unit of **TB-P3Cz** and adding methyl groups to the same donor moiety, an optimized structure **M3CzB** was developed and separately reported. The device with **M3CzB** in DBFPO showed an even higher EQE_max_ of 30.7% and with a lower efficiency roll-off of 30% at 1000 cd m^–2^, albeit with red-shifted emission [λ_EL_ = 470 nm, CIE coordinates (0.14, 0.26)]. A device fabricated with 20 wt% **M3CzB** in mCBP-CN as the EML for improved stability exhibited an LT_50_ of 81 h at an initial 400 cd m^–2^.^309^

Kim *et al*. used a tetramethylcarbazole donor with the DBA acceptor in blue TADF emitters **TMCz-BO** and **TMCz-3P** (Figure 37). **TMCz-BO** and **TMCz-3P** emit at λ_PL_ of 446 and 455 nm respectively in toluene, with **TMCz-BO** having degenerate ^1^CT, ^3^CT, and ^3^LE states which resulted in small Δ*E*_ST_ of 0.02 eV, short τ_d_ of 0.75 μs, and fast *k*_RISC_ of 1.9 × 10^6^ s^–1^ in 30 wt% doped films in PPF. In **TMCz-3P** the geometry and orbital character of the ^3^LE and ^3^CT states deviate more from ^1^CT, resulting in a larger Δ*E*_ST_ of 0.13 eV, longer τ_d_ of 14.5 μs, and reduced *k*_RISC_ of 0.03 × 10^6^ s^–1^. As a result the device with **TMCz-BO** showed an EQE_max_ of 20.7% and efficiency roll-off of 16% at 1000 cd m^–2^ [λ_EL_ of 471 nm and CIE coordinates of (0.14, 0.18)], outperforming the OLED with **TMCz-3P** with a similar EQE_max_ of 20.4% but more severe efficiency roll-off of 37% at 1000 cd m^–2^, and with a somewhat red-shifted λ_EL_ of 479 nm at CIE coordinates of (0.14, 0.26).^310^

The emitters **TDBA-PAS** and **TDBA-DPAC** contain phenazasiline and diphenylacridine donor moieties coupled to the DBA acceptor (Figure 37), in which the larger Si center weakens the electron donating strength.^311^ As a result the emission of the former in toluene is blue-shifted (λ_PL_= 427 nm) compared to the latter (λ_PL_ = 444 nm), and the emission of **TDBA-DPAC** is itself blue-shifted compared to previously discussed **TDBA-Ac** (λ_PL_ = 458 nm).^304^ **TDBA-PAS** also shows dual emission at low dopant concentrations, which was attributed to the presence of both quasi-axial (QA) and quasi-equatorial (QE) conformers associated with the higher and lower energy emission respectively, and enabled by the increased flexibility of the Si linking center. After increasing the dopant concentration from 10 to 30 wt% in DPEPO film to minimize the impact of the QA conformer, the emission of **TDBA-PAS** narrowed to a FWHM of 54 nm and the Φ_PL_ was enhanced from 80.7 to 92.1%. In contrast, for **TDBA-DPAC** the FWHM increased from 57 to 66 nm and the Φ_PL_ decreased from 85.3 to 76.8%. The increase in Φ_PL_ for **TDBA-PAS** was attributed to improved energy transfer from the high energy QA conformer to low energy QE conformer, while the decrease of Φ_PL_ in **TDBA-DPAC** was attributed to concentration quenching. Both emitters nonetheless showed small Δ*E*_ST_ < 0.06 eV, fast *k*_RISC_ > 12.3×10^5^ s^–1^, and short τ_d_ < 3.14 μs. The OLEDs with **TDBA-PAS** and **TDBA-DPAC** showed respective EQE_max_ of 22.4 and 24.6% at CIE coordinates of (0.16, 0.04) and (0.15, 0.09). Further enhanced TADF properties were achieved by removal of the *tert*-butyl group from the DBA acceptor in **OBO-I**, which was reported alongside a symmetric D-A-D emitter **OBO-II**.^312^ These structural changes afforded emitters with significantly smaller Δ*E*_ST_ of 0.007 and 0.013 eV in 20 wt% doped films in PPF, resulting in much shorter τ_d_ of 1.60 and 1.70 μs, higher Φ_PL_ of 81 and 98%, and faster *k*_RISC_ of 11 and 9.2 × 10^–5^ s^–1^ for **OBO-I** and **OBO-II**, respectively. The OLEDs with **OBO-I** and **OBO-II** showed EQE_max_ of 21.7 and 31.7% at CIE coordinates of (0.14, 0.10) and (0.14, 0.13), and non-doped devices showed EQE_max_ of 8.7 and 23.1% at CIE coordinates of (0.15, 0.17) and (0.15, 0.20), all respectively. The non-doped devices with **OBO-I** and **OBO-II** also showed reduced efficiency roll-off with EQE decreasing from maximum values by 6.9/48.3% and 7.8/43.7% at 100/1000 cd m^–2^, respectively.

Another strategy to increase the efficiency of OLEDs is to enhance the emitter molecular anisotropy to boost optical outcoupling efficiency. Linearly shaped emitters **TDBA-SBA** and **2TDBA-SBA** (Figure 37) containing spiro-bisacridine donors exhibited this desired preferential horizontal orientation of their TDMs, reported at 86 and 88% respectively in 20 wt% doped flims in DBFPO.^313^ Both compounds have small Δ*E*_ST_ of 0.01 eV leading to similar τ_d_ of 1.47 and 1.38 μs alongside Φ_PL_ of 89 and 87%. The blue OLEDs with **TDBA-SBA** and **2TDBA-SBA** showed EQE_max_ values (with CIE coordinates) of 29.3% (0.13, 0.15) and 18.3% (0.13, 0.21), respectively, supported in part by the horizontal TDMs. The lower efficiency of **2TDBA-SBA** was attributed to its higher *k*_nr_ and slower *k*_RISC_ compared to **TDBA-SBA**.

The effect of donor position has also been investigated by incorporating DMAC moieties in *meta*-, *para*-, or *meta′*-positions relative to the boron atom in the DBA acceptor. The *para*-substituted compound (***p*-AC-DBNA**, Figure 37) exhibited sky-blue emission with λ_PL_ of 496 nm, higher Φ_PL_ of 96%, smaller Δ*E*_ST_ of 0.09 eV, shorter τ_d_ of 1.5 μs, and faster *k*_RISC_ of 1.1×10^6^ s^–1^ in 5 wt% doped BCPO films. The OLEDs with **p-AC-DBNA** emitted at λ_EL_ of 488 nm, and showed an EQE_max_ of 20.5% and low efficiency roll-off of 8% at 100 cd m^–2^ and 20% at 1000 cd m^–2^ (Table S1).^314^ Similarly, two isomeric carbazole-substituted emitters with DBA acceptors, **TDBA-Cz** and **DBA-Cz** (Figure 37), exhibited narrowband emission at λ_PL_ of 461 and 447 nm (FWHM of 43 and 38 nm) and Δ*E*_ST_ of 0.14 and 0.03 eV, all respectively, in toluene.^315^ The Φ_PL_ in 10 wt% doped films in DPEPO are unity and 90%, while the Φ_PL_ remained relatively high at 88 and 52% in neat films. The devices with **TDBA-Cz** and **DBA-Cz** hence showed high respective EQE_max_ of 31.1 and 30.3% at CIE coordinates of (0.13, 0.13) and (0.14, 0.15), while the non-doped device with **TDBA-Cz** showed an EQE_max_ of 21.4% at CIE of (0.14, 0.16). Incorporation of an additional carbazole unit and subsequent removal of the acceptor *tert*-butyl groups afforded new TADF emitters **5TBO** and **3TBO**.^316^ **5TBO** showed a blue-shifted emission (λ_PL_ = 457 nm) while retaining a FWHM of 44 nm, while **3BTO** showed a slightly red-shifted emission (λ_PL_ = 462 nm) and a narrower FWHM of 39 nm. **3BTO** and **5BTO** have similar Δ*E*_ST_ values of ∼0.15 eV, yet differ significantly in their Φ_PL_ of 78.4 and 96.7% in respective 30 wt% doped mCBP films. These differences were attributed to smaller conformational freedom in **5BTO**, resulting in slower *k*_nr_ and the larger Φ_PL_. The device with **5TBO** consequently showed a higher EQE_max_ of 26.2% than the device with **3BTO** (17.3%), with both embittering at λ_EL_ of 484 nm with similar CIE coordinates of (0.13, 0.28) and (0.12, 0.29), respectively.

Compounds **PhCz-TSOBA** and **TPA-TSOBA** replace an oxygen atom in the DBA core with a sulfur atom (Figure 37).^317^ **PhCz-TSOBA** and **TPA-TSOBA** emit at 444 and 447 nm, respectively, in toluene and show narrowband emission (FWHM of 32 and 34 nm). However, their Δ*E*_ST_ are large at 0.23 and 0.36 eV in toluene, their Φ_PL_ are moderate at 60.8 and 61.8%, and their *k*_RISC_ are slow at 4.08 and 1.91×10^–4^ s^–1^ in 10 wt% doped films in 2,6-DczPPy, all respectively. The OLEDs with **PhCz-TSOBA** and **TPA-TSOBA** showed the same EQE_max_ of 16.7% at λ_EL_ of 456 nm, with FWHM of 57 and 55 nm [CIE coordinates of (0.14, 0.15) and (0.14, 0.12)], respectively.

Summarizing these reported results using boron-containing acceptors, the fully fused DBA-based materials frequently exhibit superior TADF properties and device performances than unfused boron. The more rigid and bulky nature of the DBA acceptor provides an optimal dihedral angle with many types of donor to produce emitters that have well separated frontier orbitals, enhanced Φ_PL_, and often also horizontally oriented TDM. The most efficient OLED with a boron acceptor (**TDBA-DI**) showed an EQE_max_ of 38.2%, and this class of emitters is able to reach rather deep into the blue region of CIE space (Figure 38).^304^ Despite these high achievable EQE, common to all current blue TADF OLEDs, these devices with boron-based acceptors still frequently suffer from poor device roll-off and lifetime.

**Figure 38:**
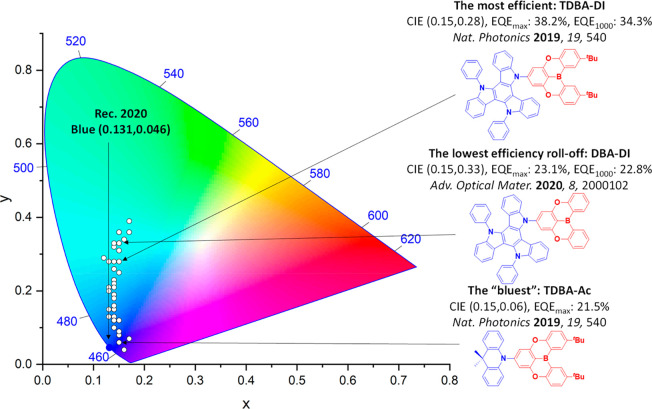
CIE color coordinates of blue TADF emitters containing boron acceptors. The white circles illustrate the spread of the emission color of the device. Selected devices and their associated CIE coordinates are highlighted, illustrating the structure of the emitter of the “bluest” device, the structure of the emitter used in the device showing the highest EQE_max_ and the structure of the emitter associated with the device showing the lowest efficiency roll-off. Only TADF OLEDs where the λ_EL_ < 490 nm are included. The device with the CIE coordinates closest to the Rec. 2020 defined coordinates for blue, (0.131, 0.046), is defined as the “bluest”. The most efficient device is quantified by the highest EQE_max_. The efficiency roll-off is quantified as the change in efficiency between EQE_max_ and EQE_1000_. In the chemical structures, the blue color signifies donor moieties, while the red color signifies acceptor moieties.

### 3.6 Nitrile-Containing Emitters

Likely due to its compact size and simple chemical structure, many of the early reported organic TADF materials contained nitrile acceptors. This includes the first sky-blue emitter **2CzPN**, and materials development using this acceptor has only accelerated in the intervening years. For example, the replacement of Cz in **2CzPN** ^31^ with a δ-carboline led to the formation of sky-blue emitter **δ-2CbPN** (Figure 39). In toluene this compound has a smaller Δ*E*_ST_ of 0.13 eV, higher Φ_PL_ of 93%, shorter τ_d_ of 180 μs, and blue-shifted λ_PL_ of 453 nm compared to **2CzPN** (Δ*E*_ST_ of 0.21 eV, Φ_PL_ of 89%, τ_d_ of 270 μs, and λ_PL_ of 473 nm). Thus, the corresponding OLED with **δ-2CbPN** showed an improved EQE_max_ of 22.5% and blue-shifted λ_EL_= 486 nm compared to 19.2% and 491 nm for **2CzPN**, both using the same device structure. The device containing **α-2CzPN** instead showed a very low EQE_max_ of 4.2% at λ_EL_ of 473 nm, owing to the moderate Φ_PL_ of 37%. Furthermore, the Δ*E*_ST_ of **α-2CzPN** in 20 wt% doped mCP films (0.28 eV) is more than double that of **δ-2CzPN** (0.13 eV). As a result, there is less efficient RISC in **α-2CzPN**, with the weaker donating properties of the α-carboline directly leading to lower charge-transfer exciton character and thus an increased Δ*E*_ST_.

**Figure 39:**
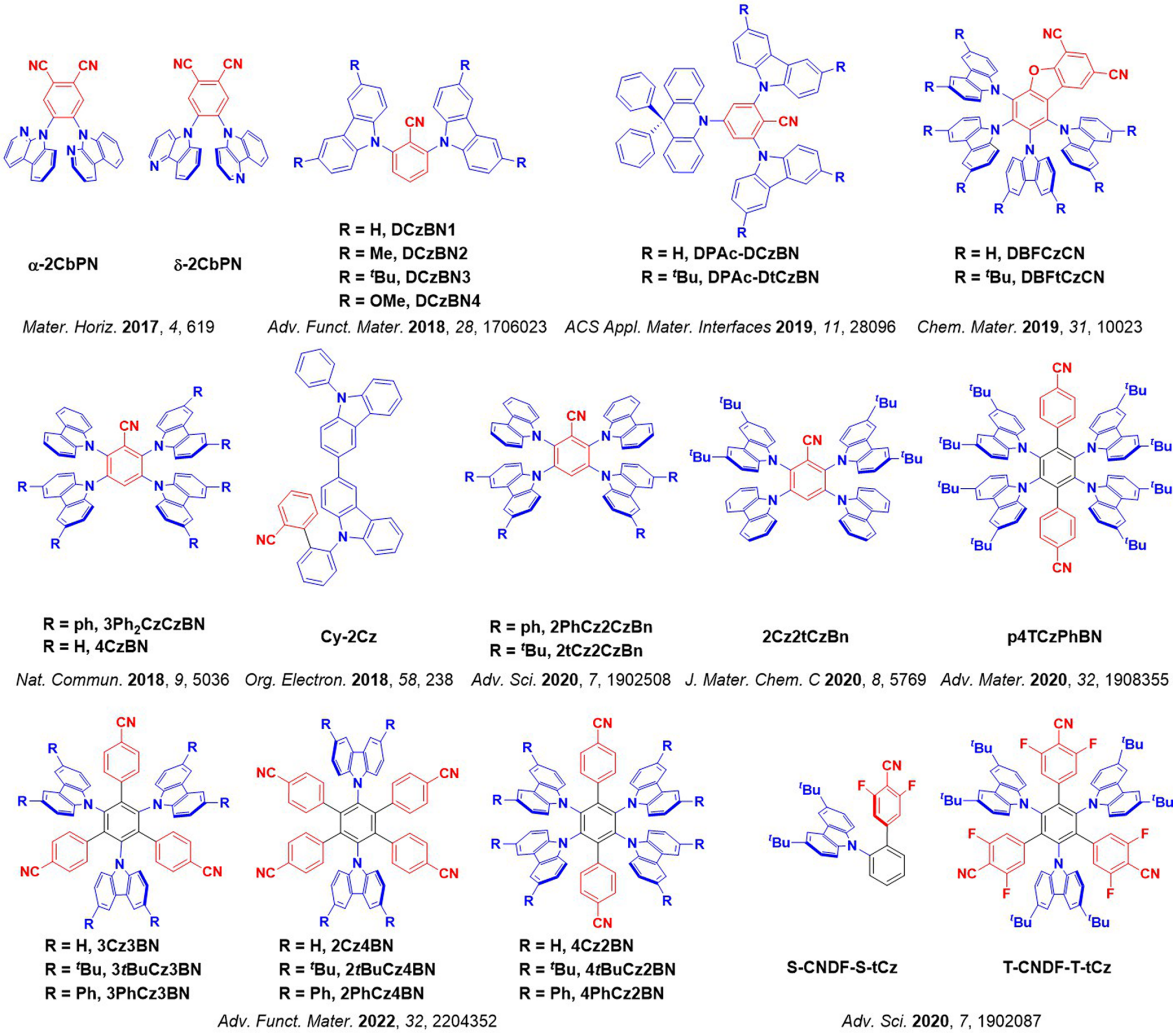
Molecular structures of nitrile-based blue TADF emitters discussed here (the blue color signifies donor moieties, while the red color signifies acceptor moieties).

A benzonitrile acceptor was functionalized with pairs of *ortho* carbazole donor derivatives to produce deep-blue TADF emitters that showed CIE_y_ coordinates < 0.08 in devices: **DCzBN1** (0.15, 0.05), **DCzBN2** (0.15, 0.07), and **DCzBN3** (0.16, 0.06) (Figure 39).^318^ The devices showed EQE_max_ values of 2.5, 7.7, and 10.3%, respectively, with the latter representing one of the highest performing deep-blue TADF OLEDs at that time. An EQE_max_ of 18.0% was achieved for the device with related structure **DCzBN4**; however, a red-shift in the EL was observed with CIE coordinates of (0.16, 0.23). Unfortunately, all the devices displayed severe efficiency roll-off, with brightness of 100 cd m^–2^ not achieved for **DCzBN1–3**, while the efficiency roll-off for **DCzBN4** was 42% at 100 cd m^–2^. The longer τ_d_ for **DCzBN1–3** (11.2 to 18.0 μs) compared to **DCzBN4** (5.5 μs) is consistent with the difference in efficiency roll-off behavior. Additionally, the high T_1_ energies (∼3.0 eV) of all the emitters and the use of mCP (T_1_ ≈ 2.9 eV) as a blocking layer may have contributed to inefficient exciton confinement. Introducing a spiro-acridan donor at the *para* position of benzonitrile in the **DCzBN** framework resulted in improved device efficiency, although this was also accompanied by a red-shift in the emission. The stronger ICT between the spiro-acridine and the benzonitrile moieties in **DPAc-DCzBN** and **DPAc-DtCzBN** led to higher Φ_PL_ (71 and 64%, respectively) compared to **DCzBN1–3** all with a Φ_PL_ below 35%, but broader and red-shifted emission in the doped DPEPO films. The devices with **DPAc-DCzBN** and **DPAc-DtCzBN** showed EQE_max_ of 23% for both devices yet suffered around 50% efficiency roll-off at 1000 cd m^–2^; the CIE coordinates were (0.17, 0.25) and (0.16, 0.15), respectively.^319^

Substituting a dibenzofuran core with four carbazole donors and two nitrile acceptors was highly effective in achieving efficient TADF in the emitters **DBFCzCN** and **DBFtCzCN** (Figure 39).^320^ **DBFCzCN** and **DBFtCzCN** emit with λ_PL_, Δ*E*_ST_, and τ_d_ of 451 and 489 nm, 0.28 and 0.18 eV, and 89.0 and 29.1 μs respectively in THF. They also have high Φ_PL_ of 100 and 92% in 20 wt% doped films in DPEPO. The device with **DBFCzCN** showed an EQE_max_ of 25.2% with CIE coordinates of (0.15, 0.29), although with a large roll-off (93%) at 1000 cd m^–2^. The device with **DBFtCzCN** showed an EQE_max_ and CIE of 17.4% and (0.19, 0.49), with the green EL arising from the relatively stronger *t*BuCz donors.

Decoration of carbazole donors with peripheral phenyl groups in **3Ph_2_CzCzBN** (Figure 39) led to improved TADF behavior and blue emission in toluene, with λ_PL_, Δ*E*_ST_, Φ_PL_, and τ_d_ of 464 nm, 0.19 eV, 95%, and 10 μs. These properties compared favorably to **4CzBN** (443 nm, 0.23 eV, 63%, and 50 μs), which contains unsubstituted Cz donors. The device with **3Ph_2_CzCzBN** (15 wt% in mCBP) showed EQE_max_ and CIE coordinates of 15.9% and (0.17, 0.37), albeit with a red-shifted emission at 482 nm compared to the reference device with **4CzBN** (EQE_max_ of 9.7% and CIE coordinates (0.19, 0.32) at λ_EL_ of 471 nm). Further, the device performance of **3Ph_2_CzCzBN** was further improved in an EML consisting of 20 wt% emitter in mCBP, with EQE_max_, CIE, and λ_EL_ of 17.9%, (0.18, 0.39) and 486 nm, and reduced efficiency roll-off of 1.7% at 1000 cd m^–2^.^321^

Zou *et al*. employed a mixture of substituted and unsubstituted carbazole donors in **2tCz2CzBn** and **2PhCz2CzBn** (Figure 39),^322^ both of which showed similar λ_PL_ of 455 and 458 nm in toluene, Δ*E*_ST_ values of 0.15 and 0.17 eV in 2Me-THF, and Φ_PL_ of 87 and 86% in doped mCBP films (20 and 30 wt%), all respectively. The OLED with **2tCz2CzBn** showed an EQE_max_ of 23.8% at λ_EL_ of 464 nm with CIE coordinates of (0.15, 0.19), while the OLED based on **2PhCz2CzBn** showed an improved EQE_max_ of 26.6% with an almost identical EL spectrum. In another report, non-doped solution-processed devices with **2tCz2CzBn** also exhibited high efficiency, with the EQE_max_ reaching 24.5% albeit with a red-shifted EL [λ_EL_ = 472 nm and CIE coordinates (0.16, 0.24)] compared to an EQE_max_ of 25.8% [λ_EL_ = 488 nm and CIE coordinates (0.21, 0.42)] for the device with isomer **2Cz2tCzBn**. The high EQE_max_ of the devices with **2tCz2CzBn** and **2Cz2tCzBn** are supported by the AIE properties of these materials as neat films, however both devices showed severe efficiency roll-off of more than 75% at 1000 cd m^–2^.^323^

To further blue-shift the emission of multi-carbazole emitters, Zhang *et al*. adopted a weaker cyanophenyl acceptor in lieu of the stronger (directly attached) cyano acceptor. In toluene **p4TCzPhBN** (Figure 39) has λ_PL_, Δ*E*_ST_, Φ_PL_, τ_d_, and *k*_RISC_ of 452 nm, 0.1 eV, 93.4%, 6.3 μs, and 2.36 × 10^6^ s^–1^, respectively. The OLED showed an EQE_max_ of 22.8% at λ_EL_ of 456 nm and CIE coordinates of (0.15, 0.10).^324^ Later, Madama *et al*. developed a series of TADF emitters using the cyanophenyl group as a weak acceptor and different numbers and types of carbazole derivatives (Cz, *t*BuCz, or PhCz) in the structural template **2-4(D)(2-4)BN** (Figure 39).^325^ Their preliminary photophysical results revealed that emitters containing carbazole as the donor showed either no (**3Cz3BN** and **2Cz4BN**) or weak (**4Cz2BN**) TADF character. Likewise, **3*t*BuCz3BN**, **2*t*BuCz4BN**, **3PhCz3BN**, and **2PhCz4BN** also showed weak TADF character with Δ*E*_ST_ > 0.18 eV and low Φ_PL_ < 19% in toluene. Only compounds **4*t*BuCz2BN** and **4PhCz2BN** exhibited efficient TADF with Δ*E*_ST_ of 0.07 and 0.03 eV, τ_d_ of 29.0 and 5.0 μs, and Φ_PL_ of 45 and 87%, respectively. OLEDs were fabricated with **4PhCz2BN** and the optimized devices showed an EQE_max_ of 18.2% at CIE coordinates of (0.16, 0.28). Non-doped devices also performed well with EQE_max_ of 16.4% and efficiency roll-off of only 13% at 1000 cd m^–2^, albeit with a red-shifted λ_EL_ = 498 nm and CIE coordinates (0.21, 0.45).

**Figure 40:**
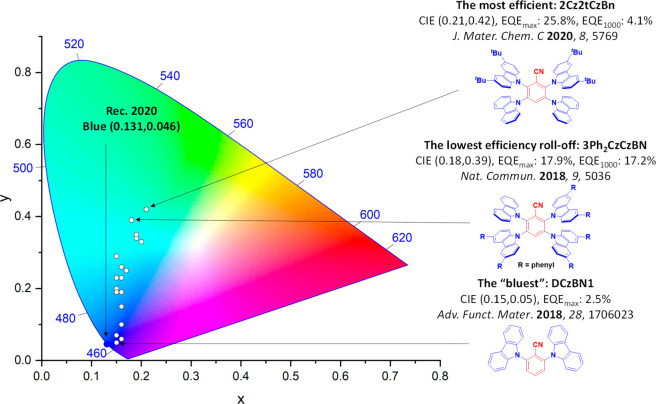
CIE color coordinates of blue D-A TADF emitters containing nitrile acceptors. The white circles illustrate the spread of the emission color of the device. Selected devices and their associated CIE coordinates are highlighted, illustrating the structure of the emitter of the bluest device, the structure of the emitter used in the device showing the highest EQE_max_ and the structure of the emitter associated with the device showing the lowest efficiency roll-off. Only TADF OLEDs where the λ_EL_ *<* 490 nm are included. The device with the CIE coordinates closest to the Rec. 2020 defined coordinates for blue, (0.131, 0.046), is defined as the “bluest”. The most efficient device is quantified by the highest EQE_max_. The efficiency roll-off is quantified as the change in efficiency between EQE_max_ and EQE_1000_. In the chemical structures, the blue color signifies donor moieties, while the red color signifies acceptor moieties.

The deep-blue emitter **Cy-2Cz** contains a benzonitrile acceptor with an *ortho*-substituted bisN-phenylcarbazole donor (Figure 39), and has Δ*E*_ST_ of 0.18 eV in toluene. The OLED with **Cy-2Cz** showed deep blue emission with CIE coordinates of (0.16, 0.10) and an EQE_max_ of 11.9%. The relatively low efficiency roll-off of ∼21% at 1000 cd m^–2^ results from the short τ_d_ of 9.4 μs, indicated the promise of this emitter design strategy.^326^ Through-space charge transfer (TSCT) and through-bond charge transfer (TBCT) contributions to the excited state character were simultaneously incorporated into an emitter with three alternating difluorocyanobenzene acceptor units and three di-*tert*-butyl carbazole donor groups, connected to a central benzene ring. TD-DFT calculation showed that **T-CNDF-T-tCz** (Figure 39) has multiple degenerate excited singlet and triplet states, leading to a low energy barrier for RISC. **T-CNDF-T-tCz** emits at λ_PL_ of 477 nm, has a small Δ*E*_ST_ of 0.03 eV, a high Φ_PL_ of 76%, and a τ_d_ of 7.79 μs as a neat film (Table S1). The non-doped solution-processed OLED exhibited sky-blue emission with λ_EL_ of 484 nm at CIE coordinates of (0.19, 0.35), and showed an EQE_max_ of 21.0% – nine times higher than the device with reference mono D-A emitter **S-CNDF-S-tCz** (EQE_max_ of 2.6%).^327^

The most efficient devices containing benzonitrile-based blue TADF emitters are highlighted in Figure 40. These devices largely struggle to achieve high efficiency due to their low Φ_PL_. Further, these devices generally show suboptimal blue color coordinates due to their broad emission. Thus, this molecular design seems to be less promising than those based on, for instance, triazine or boron-containing acceptors.

### 3.7 Oxadiazole-Containing Emitters

The inherently electronegative nature of the heteroatoms in oxadiazole has long been deployed in the design of electron-transporting materials for OLEDs, such as PBD and OXD-7.^328–330^ The very shallow LUMO level (−0.55 eV) of oxadiazole^331^ also makes 1,3,4-oxadiazole and its derivatives potentially attractive as acceptors in the design of deep-blue TADF emitters. The oxadiazole functionality can be easily obtained from an existing nitrile precursor, and thus a wide variety of donor-acceptor compounds can be readily accessed. Selected oxadiazole based emitters discussed here are shown in Figure 41. For example, replacement of the nitrile groups in **2CzPN** with weaker electron-accepting oxadiazoles resulted in blue-shifted emission, which could be fine-tuned with the distal aryl groups. The respective λ_PL_ of oxadiazole-based **2CzdOXDME**, **2CzdOXDPh**, and **2CzdOXD4MeOPh** (Figure 41) are 453, 466, and 459 nm, whereas **2CzdOXD4CF_3_Ph** has a red-shifted λ_PL_ of 487 nm due to the auxiliary trifluoromethyl acceptor unit. Further, the oxadiazole-based emitters **2CzdOXDME**, **2CzdOXDPh**, **2CzdOXD4CF_3_Ph**, and **2CzdOXD4MeOPh** have high Φ_PL_ of 28.7, 38.3, 39.1, and 47% respectively, superior to **2CzPN** (28.1%).^332^ However, **2CzdOXDME**, **2CzdOXDPh**, **2CzdOXD4CF_3_Ph**, and **2CzdOXD4MeOPh** have long τ_d_ of 24.6, 58.6, 31.5, and 64.9 ms in 10 wt% doped films in PMMA and large Δ*E*_ST_ of 0.31, 0.31, 0.32, and 0.44 eV in 10 wt% doped films in DPEPO, all respectively (Table S1).^333^ The OLEDs with **2CzdOXDMe**, **2CzdOXDPh**, **2CzdOXD4CF_3_Ph**, and **2CzdOXD4MeOPh** showed EQE_max_ of 11.8, 7.0, 12.3, and 4.8% with λ_EL_ of 452, 460, 472, and 452 nm at CIE of (0.17, 0.17), (0.15, 0.16), (0.18, 0.28), and (0.17, 0.17), all respectively. Unfortunately, the large Δ*E*_ST_ and longer τ_d_ led to severe efficiency roll-off (89, 81, 79, and 73% respectively at 100 cd m^–2^). Therefore, although blue-shifted emission had been achieved, it came at the cost of efficiency and overall performance.^332^

**Figure 41:**
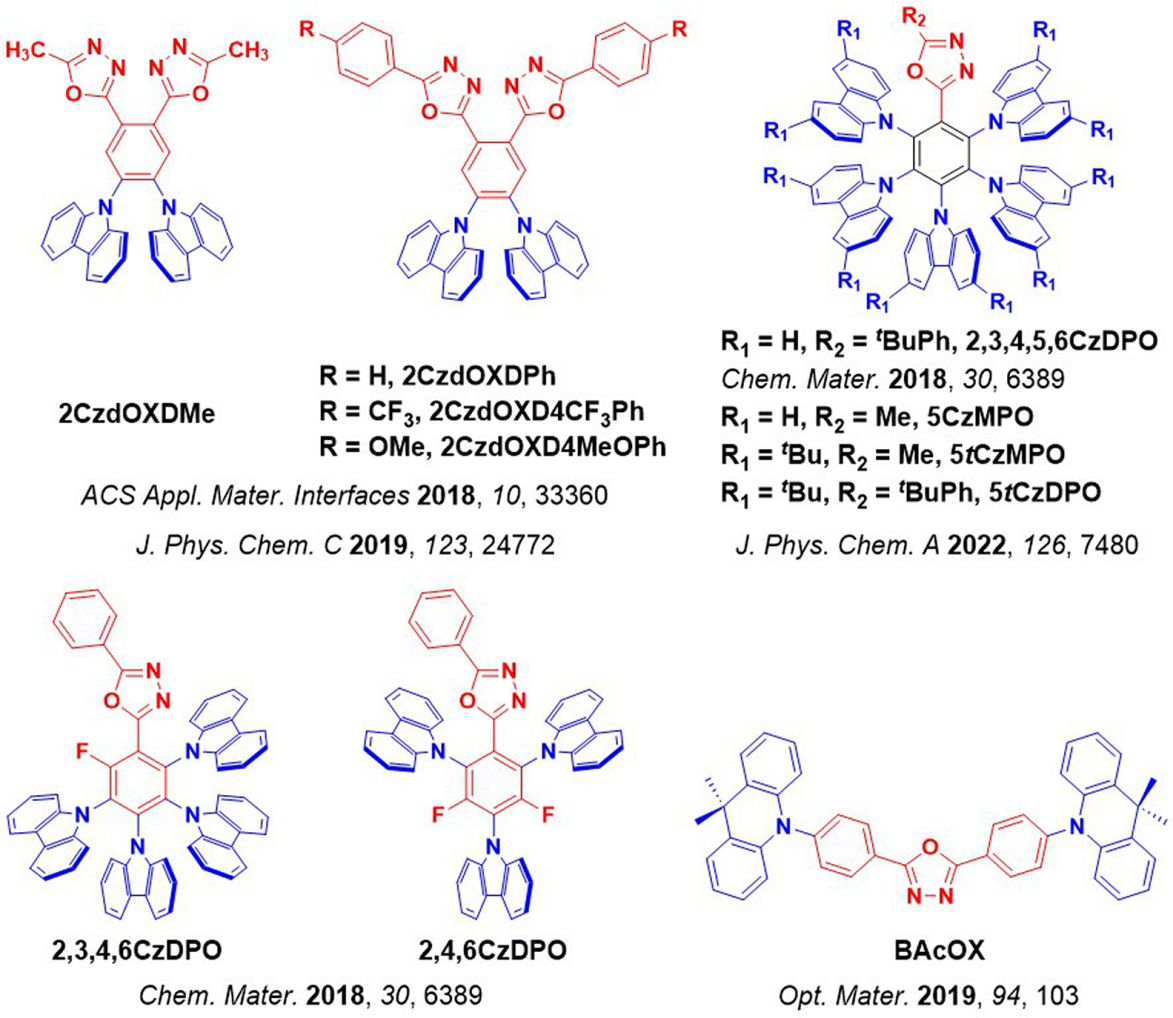
Molecular structures of oxadiazole-based blue TADF emitters discussed here (the blue color signifies donor moieties, while the red color signifies acceptor moieties).

Using a similar oxadiazole acceptor the effect of changing the number of carbazole donors was investigated along with the use of secondary fluorine acceptor moieties connected to the central benzene.^334^ Although six emitters were investigated, of these only **2,4,6CzDPO**, **2,3,4,6CzDPO**, and **2,3,4,5,6CzDPO** (Figure 41) displayed TADF, emitting at λ_PL_ of 490, 483, and 482 nm respectively in toluene. Increasing the number of donors altered the S_1_ energy, with substitution at the 3- and 5-positions particularly destabilizing the S_1_ energy relative to **2,4,6CzDPO**. The devices with **2,4,6CzDPO**, **2,3,4,6CzDPO**, and **2,3,4,5,6CzDPO** showed respective EQE_max_ values of 6.1, 17.8, and 24.4% at CIE coordinates of (0.17, 0.30), (0.18, 0.36) and (0.16, 0.29), far from target blue color coordinates. Relatively high efficiencies roll-off ranging from 23 to 54% at 100 cd m^–2^ also occurred, due to long τ_d_ of between 75 and 308 and 75 μs. Later the same group studied the effect of adding methyl and phenyl substituents on the oxadiazole acceptor of the most promising emitter previously identified (**2,3,4,5,6CzDPO**) and designed additional compounds **5*t*CzDPO**, **5CzMPO**, and **5*t*CzMPO**. The methyl-modified **5CzMPO** showed a blue-shifted emission (λ_PL_ = 466 nm) while the emission of **5*t*CzDPO** is red-shifted (λ_PL_ = 496 nm) compared to **2,3,4,5,6CzDPO** (λ_PL_ = 482 nm), all in toluene. These three emitters showed smaller Δ*E*_ST_ in the range of 0.01 to 0.12 eV and higher Φ_PL_ (20 to 30%) compared to **2,3,4,5,6CzDPO** (Δ*E*_ST_ = 0.12 eV and Φ_PL_ = 13%), attributed to the increased donor strength of the *t*Cz donor. The OLED with **5*t*CzDPO** showed a higher EQE_max_ of 29% but red-shifted CIE coordinates of (0.18, 0.36) compared to the reference device with **2,3,4,5,6CzDPO** [EQE_max_ = 24.4%, CIE coordinates of (0.16, 0.29)] (Table S1).^335^

A simple D-A-D design using DMAC as the donor produced highly efficient sky-blue TADF emitter **BAcOX** (Figure 41).^336^ **BAcOX** emits at λ_EL_ of 461 nm and has a Δ*E*_ST_ of 0.26 eV in toluene, while the Φ_PL_ and τ_d_ are 93% and 84.1 μs in 10 wt% doped films in DPEPO. The OLED showed an EQE_max_ of 22.3% at λ_EL_ of 475 nm and CIE coordinates of (0.16, 0.24), however the device suffered from severe efficiency roll-off and a low maximum luminance of 200 cd m^–2^ was attributed to TTA and STA quenching permitted by the relatively long τ_d_ of 84 μs. This quenching was further exacerbated by unbalanced transport of carriers and inferior exciton confinement in the emission layer. Using DPPOC as the host instead of DPEPO improved the efficiency roll-off, but at a cost to the EQE_max_ which reached only 16.7%.

To date, only a few oxadiazole-based blue TADF materials have been developed. The best performing devices in terms of efficiency, roll-off, and color are shown in Figure 42. Though the devices with oxadiazole-based emitters showed high EQE, they exhibit higher CIE_y_ (> 0.05) coordinates compared to the Rec. 2020 blue standard. This is because of the too strong electron-withdrawing nature of the oxadiazole. This results in red-shifted and broadened emission. The generally poor thermal and photochemical stability of this moiety and the sub-optimal performance of these emitters in OLEDs are contributing factors to the lack of popularity of oxadiazole based TADF materials.

**Figure 42:**
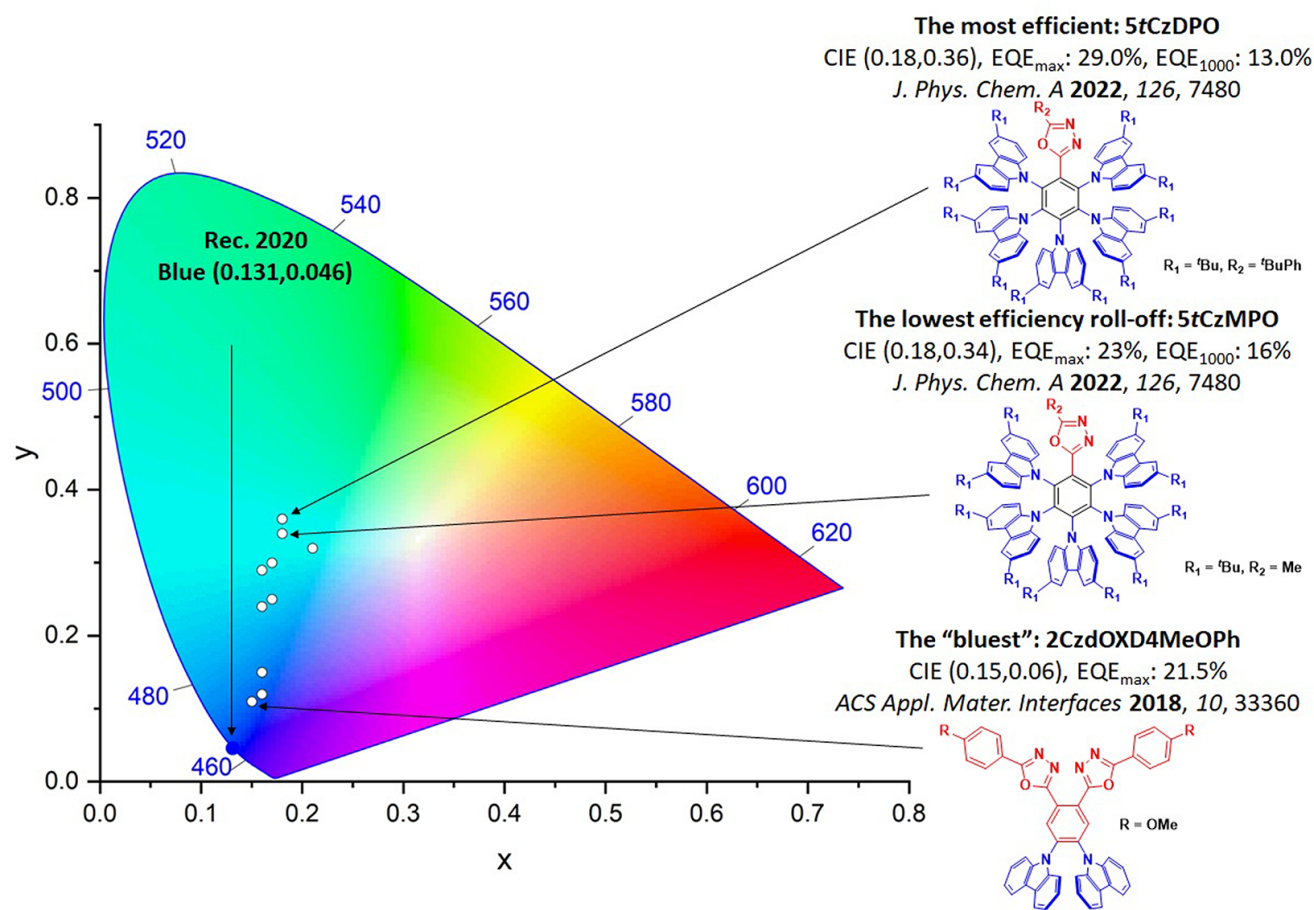
CIE color coordinates of blue D-A TADF emitters containing oxadiazole acceptors. The white circles illustrate the spread of the emission color of the device. Selected devices and their associated CIE coordinates are highlighted, illustrating the structure of the emitter of the bluest device, the structure of the emitter used in the device showing the highest EQE_max_ and the structure of the emitter associated with the device showing the lowest efficiency roll-off. Only TADF OLEDs where the λ_EL_ *<* 490 nm are included. The device with the CIE coordinates closest to the Rec. 2020 defined coordinates for blue, (0.131, 0.046), is defined as the “bluest”. The most efficient device is quantified by the highest EQE_max_. The efficiency roll-off is quantified as the change in efficiency between EQE_max_ and EQE_1000_. The blue color signifies donor moieties, while the red color signifies acceptor moieties.

### 3.8 Sulfone-Containing Emitters

Diphenylsulfone (DPS) is a versatile electron-accepting group, with moderate electron-withdrawing ability (LUMO = −1.81 eV)^337^ making it a suitable acceptor for blue TADF emitter design. The very first deep-blue TADF emitter **tCz-DPS** featured a D-A-D structure with tCz donors and was reported by Adachi and co-workers in 2012.^230^ **tCz-DPS** exhibited deep blue emission with λ_PL_ of 423 nm and Φ_PL_ of 80% in 10 wt% doped films in DPEPO.^230^ However, the Δ*E*_ST_ is large at 0.32 eV, accompanied by a long τ_d_ of 8.2 ms. The OLEDs showed deep-blue emission with λ_EL_ of 420 nm and an EQE_max_ of 9.9% but with very severe efficiency roll-off.^230^ The same group soon after reported **DMAC-DPS** (Figure 43), where the tCz donors were replaced with stronger and bulkier DMAC donors. **DMAC-DPS** exhibited blue emission with λ_PL_ of 464 nm (Φ_PL_ = 80%), and more importantly the Δ*E*_ST_ decreased to 0.08 eV with τ_d_ shortened to 3.1 μs in 10 wt% doped films in mCP.^338^ The device with **DMAC-DPS** emitted at λ_EL_ = 465 nm [CIE coordinates of (0.16, 0.20)], and showed what was a record-high EQE_max_ of 19.5%. These two benchmark TADF emitters illustrate the potential of DPS in achieving highly efficient blue TADF and have inspired a large number of related emitter designs that contain the sulfone motif in the subsequent years.

**Figure 43:**
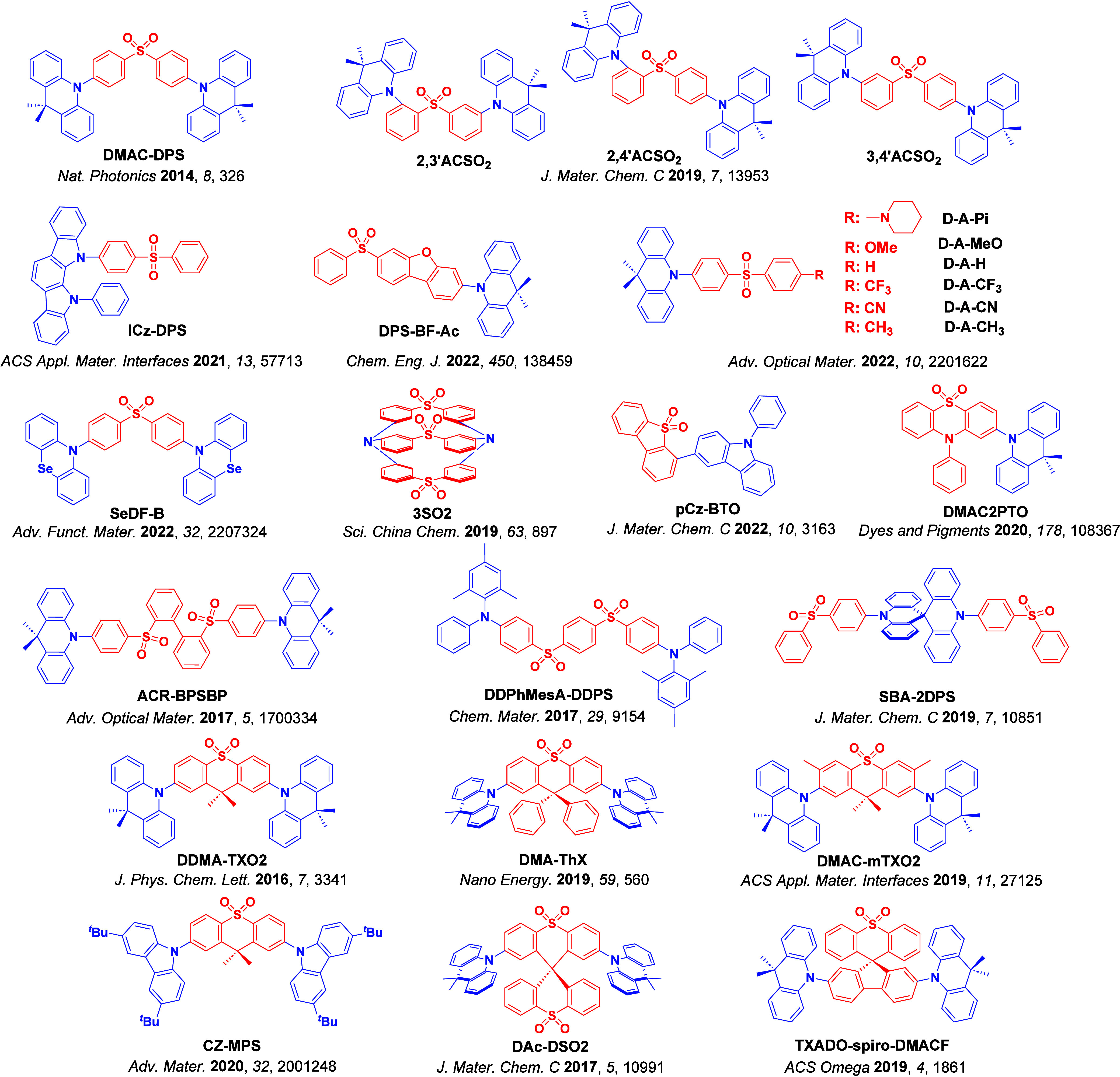
Molecular structures of sulfone-based blue TADF emitters discussed here (the blue color signifies donor moieties, while the red color signifies acceptor moieties).

Three isomers of **DMAC-DPS** were designed to investigate the effect of the donor’s position on the photophysical properties of the emitters.^339^ The molecules **2,3′ACSO2** and **2,4′ACSO2** (Figure 43) both have one *ortho*-substituted acridine donor, with the second acridine placed either in the *meta* or *para* position on the opposite side of the central DPS. These two compounds exhibited a red-shift in the emission (λ_PL_ of 502 and 499 nm, respectively) and a decrease in their Φ_PL_ (59 and 66%) in 10 wt% doped films in DPEPO compared to **DMAC-DPS** (λ_PL_ of 464 nm and Φ_PL_ of 90% in 10 wt% doped films in mCP). The OLEDs of the two emitters in 10 wt% DPEPO emitted in the green and showed only moderate EQE_max_ of around 12%. Compound **3,4′ACSO2**, containing one *meta* and one *para* acridine group, instead emits in the sky-blue at λ_PL_ of 476 nm, has a Φ_PL_ of 77%, and τ_d_ of 5.4 μs in 10 wt% doped films in DPEPO. The OLED with **3,4′ACSO2** doped in 10 wt% DPEPO displayed sky-blue emission with CIE coordinates (0.17, 0.29) and showed an EQE_max_ of 20.5% with efficiency roll-off of around 35% at 1000 cd m^–2^.^339^

To investigate the role of higher-lying LE triplet states (^3^LE) in the TADF mechanism, Ryoo *et al*. replaced one of the donors of **DMAC-DPS** with weakly conjugated electron-donating groups of piperidine (**D-A-Pi**), -OMe (**D-A-MeO**), -CH_3_ (**D-A-CH_3_**), or -H (**D-A-H**), or with electron-withdrawing groups of -CF_3_ (**D-A-CF_3_**) or -CN (**D-A-CN**, Figure 43).^340^ These six emitters exhibited blue emission in 10 wt% doped films in DPEPO, with λ_PL_ ranging from 448 to 488 nm and Φ_PL_ all above 80% apart from **D-A-Pi** (62%). All six emitters possess S_1_ and T_1_ states of CT character, with Δ*E*_ST_ values all smaller than 0.10 eV. The gaps between the LE T_2_ state (3.19 eV, T_1_ from DMAC) the S_1_ state were calculated to be −0.04, 0.02, 0.05, 0.10, 0.23, and 0.28 eV for **D-A-Pi**, **D-A-MeO**, **D-A-CH_3_**, **D-A-H**, **D-A-CF_3_**, and **D-A-CN**, respectively. **D-A-CF_3_** and **D-A-CN** possessing degenerate S_1_ and T_1_ states have shorter τ_d_ of 3.6 and 2.1 μs and faster *k*_RISC_ of 1.9 and 2.1×10^6^ s ^–1^ respectively, compared to τ_d_ > 5 μs and *k*_RISC_ < 1.5 ×10^6^ s ^–1^ for **D-A-Pi**, **D-A-MeO**, **D-A-CH_3_** each containing electron-donating groups, and also **D-A-H**. Devices with each of the six emitters exhibited EQE_max_ values between 17.2 and 23.9% at λ_EL_ between 447 and 489 nm. Among these devices those with **D-A-CF_3_** and **D-A-CN** showed small respective efficiency roll-off of 9.0% (EQE_max_ = 21.3%) and 14.2% (EQE_max_ = 20.5%) at a luminance of 100 cd m^–2^. Zhu *et al*. also explored D-A molecular design and extended the connecting DPS phenyl ring into a bulky benzofuran group, affording **DPS-BF-Ac**. This linking unit adopts an almost perpendicular geometry between the donor and acceptor moieties, leading to a small Δ*E*_ST_ of 0.03 eV in 10 wt% doped films in PMMA. These films emit at λ_PL_ of 477 nm, having a Φ_PL_ of 87% and a τ_d_ of 14.5 μs.^341^ The solution-processed device using **DPS-BF-Ac** showed an EQE_max_ of 24.7%, and sky-blue emission with λ_EL_ of 482 nm.

Xi *et al*. used a bulky and weakly electron-donating syn-indolocarbazole in **ICz-DPS** (Figure 43), which emits at 438 nm and has a Φ_PL_ of 72% with a small Δ*E*_ST_ of 0.03 eV in 10 wt% doped films in DPEPO.^342^ **ICz-DPS** exhibited a very short τ_d_ of 0.6 μs and thus a fast *k*_RISC_ of 3.3 × 10^6^ s^–1^. The device with **ICz-DPS** showed deep-blue emission with an λ_EL_ of 435 nm at CIE coordinates of (0.15, 0.08), and the EQE_max_ reached 11.6% with an efficiency roll-off of only 6% at 1000 cd m^–2^.

Sharif *et al*. explored the effect of enhancing SOC and minimizing Δ*E*_ST_ by incorporating the heavy atom selenium within the donor in **SeDF-B** (Figure 43).^343^ Theoretical calculations predicted two low-energy conformers, axial and equatorial, with only the latter showing the potential to be TADF-active. **SeDF-B** emits at λ_PL_ of 490 nm but has a very low Φ_PL_ of 3% in 10 wt% doped solution-processed mCBP films. Despite the moderately long τ_d_ of 18.5 μs, the *k*_RISC_ reached 0.6×10^6^ s ^–1^, and the vacuum-processed device with **SeDF-B** showed an EQE_max_ of 25.6% at CIE coordinates of (0.17, 0.14) and very small efficiency roll-off (∼10%) below 1000 cd m^–2^. The operational lifetime (LT_80_) of the OLED also reached 29 hours. The abnormally high EQE_max_ considering the low Φ_PL_ was ascribed to the different populations of axial/equatorial conformers during the thermal evaporation process, with the more efficient equatorial conformer being the dominant species in the evaporated films.

A D-A-A-D structure using two sulfone groups coupled with DMAC donors produced the blue emitter **ACR-BPSBP** (Figure 43), with λ_PL_ = 460 nm, Φ_PL_ = 82%, and τ_d_ = 5.0 μs.^344^ The OLED showed an EQE_max_ of 24.6% at CIE coordinates of (0.16, 0.21), however the efficiency roll-off at 200 cd m^–2^ was high (∼47%), and a luminance of 1000 cd m^–2^ could not be reached. This was attributed to slow RISC resulting from poor alignment of the LE/CT excited states, with long exciton lifetimes allowing increased TTA and SPA in the device.^344^ A deep-blue solution-processed device with an EQE_max_ of 8.5% and low efficiency roll-off of ∼9% at 1000 cd m^–2^ at CIE coordinates of (0.16, 0.08) was produced using a similar emitter **DDPhMesA-DDPS** (named **3b** in that work) containing mesityl-diphenylamine donors with the same bis-DPS acceptor.^345^

Sky-blue emitter **DAc-DSO2** (Figure 43) contains a central acceptor comprised of two spiro-linked DPS groups with two acridine donors, and emits at λ_PL_ of ∼465 nm, with a small Δ*E*_ST_ of 0.01 eV. The device with **DAc-DSO2** showed sky-blue emission with CIE coordinates of (0.18, 0.33) and an EQE_max_ of 25.4%, with very low efficiency roll-off of 13% at 1000 cd m^–2^ supported by the tailored device structure and small Δ*E*_ST_.^346^ A similar DPS-spiro-fluorene acceptor was coupled to acridines affording the blue emitter **TXADO-spiro-DMACF**. The corresponding non-doped device showed an EQE_max_ of 5.3% with a CIE_y_ coordinate of 0.09, but suffered from severe efficiency roll-off of 43% at 200 cd m^–2^ associated with its long τ_d_ of 101 μs.^347^ The elongated emitter **SBA-2DPS** instead contains a central spiro-bis-acridine donor, decorated on each side with terminal DPS groups and resulting in a small Δ*E*_ST_ of 0.09 eV and fast τ_d_ of 4.3 μs.^348^ Owing to its molecular weight and linear shape **SBA-2DPS** shows preferential horizontal TDM orientation (87%), and the device with **SBA-2DPS** showed EQE_max_ of 25.5% linked to its improved light-outcoupling efficiency. Emitting at λ_EL_ of 467 nm and CIE coordinates of (0.15, 0.20), the device exhibited modest efficiency roll-off of 10 and 39% at 100 and 1000 cd m^–2^, respectively.

Linking the phenyl rings of DPS gives the structurally related acceptor dimeth­yl­thio­xan­thene-S,S-di­oxide (TXO2), which has also been used in blue TADF emitters as exemplified in **DDMA-TXO2** (Figure 43).^349^ Like **DMAC-DPS**, **DDMA-TXO2** has a high Φ_PL_ of 95% and shows blue emission (λ_PL_ ∼ 460 nm) with a small ΔE_ST_ of 0.01 eV, and τ_d_ of 44 μs in 13 wt% doped films in DPEPO. The device showed an EQE_max_ of 22.4% at λ_EL_ of 465 nm and CIE coordinates of (0.16, 0.24).^349^ Replacing the methyl groups on the acceptor with phenyl groups in **DMA-ThX** produced a device with blue-shifted CIE coordinates of (0.14, 0.10) despite the similar λ_EL_ of 459 nm, which may be more a function of the change of host to mCBP rather than the intrinsic photophysics of the emitter. Indeed, the balance between color point and efficiency was brought into stark relief when comparing performance for the device in mCBP (EQE_max_ of 2.9% and Lum_max_ of 848 cd m^–2^) with that in DPEPO [EQE_max_ up to 18.4%, λ_EL_ of 462 nm, CIE coordinates of (0.14, 0.15), and Lum_max_ of 1460 cd m^–2^].^350^ Adding additional methyl groups *meta* to the sulfone in **DDMA-TXO2** decreases the acceptor strength and forces the D–A torsion angle to be more twisted, leading to a blue-shift the emission as reported in **DMAC-mTXO2**.^351^ **DMAC-mTXO2** exhibited blue emission with λ_PL_ of around 450 nm and Φ_PL_ of 88% in 35 wt% doped DPEPO films. Moreover, the twisted structure resulted in a very small Δ*E*_ST_ of 0.05 eV and a short τ_d_ of 3 μs. The device with **DMAC-mTXO2** displayed blue-shifted emission with CIE coordinates of (0.15, 0.18), compared to (0.16, 0.25) for **DDMA-TXO2** using the same device stack. The EQE_max_ of **DMAC-mTXO2** device was slightly improved to 22.6% compared to 20.0% for the device with **DDMA-TXO2**, tracking with the higher Φ_PL_ (88 and 80%, respectively). The device with **DMAC-mTXO2** also showed a very small efficiency roll-off of 0.5 and 12% at 100 cd m^–2^ and 1000 cd m^–2^ respectively, which were attributed to the fast *k*_RISC_ of 2.8 × 10^6^ s^–1^.

Replacing the acridine groups in **DDMA-TXO2** with weaker dtCz donors afforded the deep-blue emitter **CZ-MPS** (Figure 43).^352^ The Δ*E*_ST_ of **CZ-MPS** in toluene is large at 0.49 eV, which seems to be too large to support effectively RISC. TD-DFT calculations, however, predicted the presence of intermediate T_2_ and T_3_ triplet states, with LE character and with large SOCME values of 0.16 and 0.19 cm^–1^ respectively, that could alternatively contribute to RISC. **CZ-MPS** emits in the ultraviolet with λ_PL_ of 384 nm, Φ_PL_ of 47%, and long τ_d_ of 4.8 ms in 10 wt% doped films in PMMA. The device with **CZ-MPS** in tCzSi showed ultraviolet emission with an λ_EL_ of 389 nm, CIE_y_ coordinate of 0.06, and an EQE_max_ of 9.3% that was the highest reported for a UV-emitting OLED at that time. Considering the Φ_PL_ of the 10 wt% **CZ-MPS** doped tCzSi film is only 46%, such a high EQE_max_ indicated that triplet harvesting nonetheless occurs, most likely through TADF.

The TADF emitter **DMAC2PTO** (Figure 43) employs a phenylamine-linked DPS as a stronger acceptor in conjunction with a DMAC donor. The Δ*E*_ST_ for this compound is small at 0.03 eV in 2-MeTHF glass.^353^ **DMAC2PTO** emits at λ_PL_ of 448 nm with Φ_PL_ of 62% and short τ_d_ of 4.2 μs in 15 wt% doped films in DPEPO. The optimized device with **DMAC2PTO** emitted at λ_EL_ of 448 nm with CIE coordinates of (0.15, 0.11) and showed an EQE_max_ value of 15.2%. The severe efficiency roll-off documented is likely due in part to the use of the notably unstable DPEPO host, along with TTA and STA quenching.^353^

Wang *et al*. used dibenzo[b,d]thiophene-5,5-dioxide as the acceptor unit and 9-phenyl-9*H*-carbazole (pCz) as the donor in the AIE compound **pCz-BTO** (Figure 43).^354^ This compound emits at λ_PL_ of 438 nm, has a Φ_PL_ of 59%, and a Δ*E*_ST_ of 0.18 eV. The EQE_max_ of the non-doped device reached 7.1% with CIE coordinates of (0.15, 0.10), while the device with 10 wt% emitter in DPEPO had EQE_max_ slightly higher at 9.5% with almost identical CIE coordinates of (0.15, 0.09).

A creatively designed and highly soluble organic cage consisting of three DPS units connected by two donating nitrogen bridges (**3SO2**, Figure 43) shows promising TADF properties.^355^ Due to the cage structure intramolecular conformational flexibility was restricted, and **3SO3** showed narrowed deep blue emission with λ_PL_ of 414 nm and FWHM of 34 nm, along with Δ*E*_ST_ of 0.18 eV, all in toluene. **3SO3** has a low Φ_PL_ of 14% and τ_d_ of 8.6 μs in 5 wt% doped films in 26DCzPPY, and the OLED showed narrowband emission at λ_EL_ of 413 nm (FWHM of 35 nm) with CIE coordinates of (0.15, 0.04). However, the EQE_max_ was only 2.6% which was attributed to the low Φ_PL_, also to challenges selecting an appropriate device structure due to the high S_1_/T_1_ energies and shallow LUMO of **3SO2**.

Thanks to its relatively weak electron-withdrawing ability, the DPS moiety and its related cyclic structures have become popular in the design of blue TADF emitters, particularly when paired with acridine donors (Figure 44). The deepest blue OLED is based on **3SO2** and emits at λ_EL_ of 413 nm and has CIE coordinates of (0.14, 0.04). **SeDF-B**, where DPS moiety is used as acceptor and phenoselenazine is used as donor, exhibited the highest EQE_max_ of 25.6% among the blue sulfone-based emitters and also exhibited a small efficiency roll-off with EQE_1000_ of 23.0%. The lowest efficiency roll-off was achieved in the device with **ICz-DPS** where the EQE_max_ of 11.6% decreased only to 10.9% at 1000 cd m^–2^ at CIE coordinates of (0.15, 0.08). Overall, the examples of sulfone-based blue TADF emitters demonstrate a capacity to approach the Rec. 2020 blue emission CIE coordinates; however, the devices based on these materials suffer from severe efficiency roll-off and poor device stability, which are likely unfortunately due to the intrinsically poor photochemical stability of the sulfone group.

**Figure 44:**
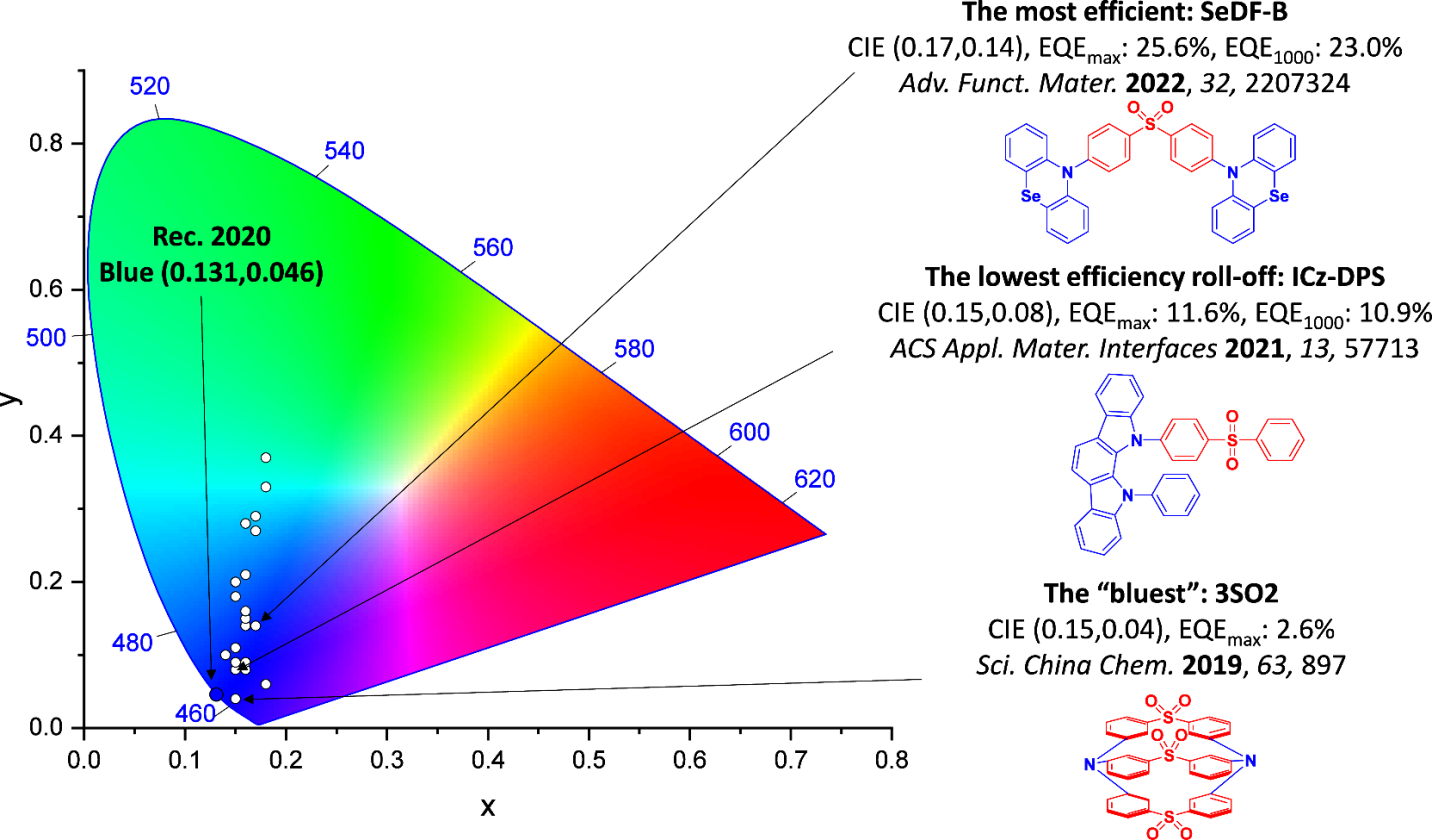
CIE color coordinates of blue D-A TADF emitters containing sulfone acceptors. The white circles illustrate the spread of the emission color of the device. Selected devices and their associated CIE coordinates are highlighted, illustrating the structure of the emitter of the bluest device, the structure of the emitter used in the device showing the highest EQE_max_ and the structure of the emitter associated with the device showing the lowest efficiency roll-off. Only TADF OLEDs where the λ_EL_ < 490 nm are included. The device with the CIE coordinates closest to the Rec. 2020 defined coordinates for blue, (0.131, 0.046), is defined as the “bluest”. The most efficient device is quantified by the highest EQE_max_. The efficiency roll-off is quantified as the change in efficiency between EQE_max_ and EQE_1000_. In the chemical structures, the blue color signifies donor moieties, while the red color signifies acceptor moieties.

### 3.9 Ketone-Containing Emitters

The ketone moiety was first introduced in TADF emitter design in the form of the benzophenone acceptor in the compound **Cz2BP**, where the device emitted at λ_EL_ of 446 nm [CIE coordinates of (0.16, 0.14)] and showed an EQE_max_ of 8.1% (Figure 45).^356^ More recently, a series of D-A emitters constructed with isobenzofurine (**MXAc-BF**) or chromone (**MXAc-CM** and **XAc-CM**) coupled to a xanthene-spiro-acridine donor unit showed sky-blue to blue TADF emission.^357^ The three compounds emit with λ_PL_ ranging from 461–482 nm, with small ΔE_ST_ (0.08–0.11 eV) and short delayed lifetimes (τ_d_ = 2.8–4.3 μs) in 50 wt% doped films in PPF. Although their EQE_max_ were moderate (16.2, 15.0, and 12.1% at λ_EL_ 478, 478, and 462 nm, respectively), the efficiency roll-off at 100 cd m^–2^ was very low at only 3–4%. Moreover, the devices with **MXAc-BF** and **MXAc-CM** exhibited low efficiency roll-off of 26% at 1000 cd m^–2^, attributed to their relatively fast *k*_RISC_ of 4.9 and 7.5 × 10^5^ s^–1^ respectively.

**Figure 45:**
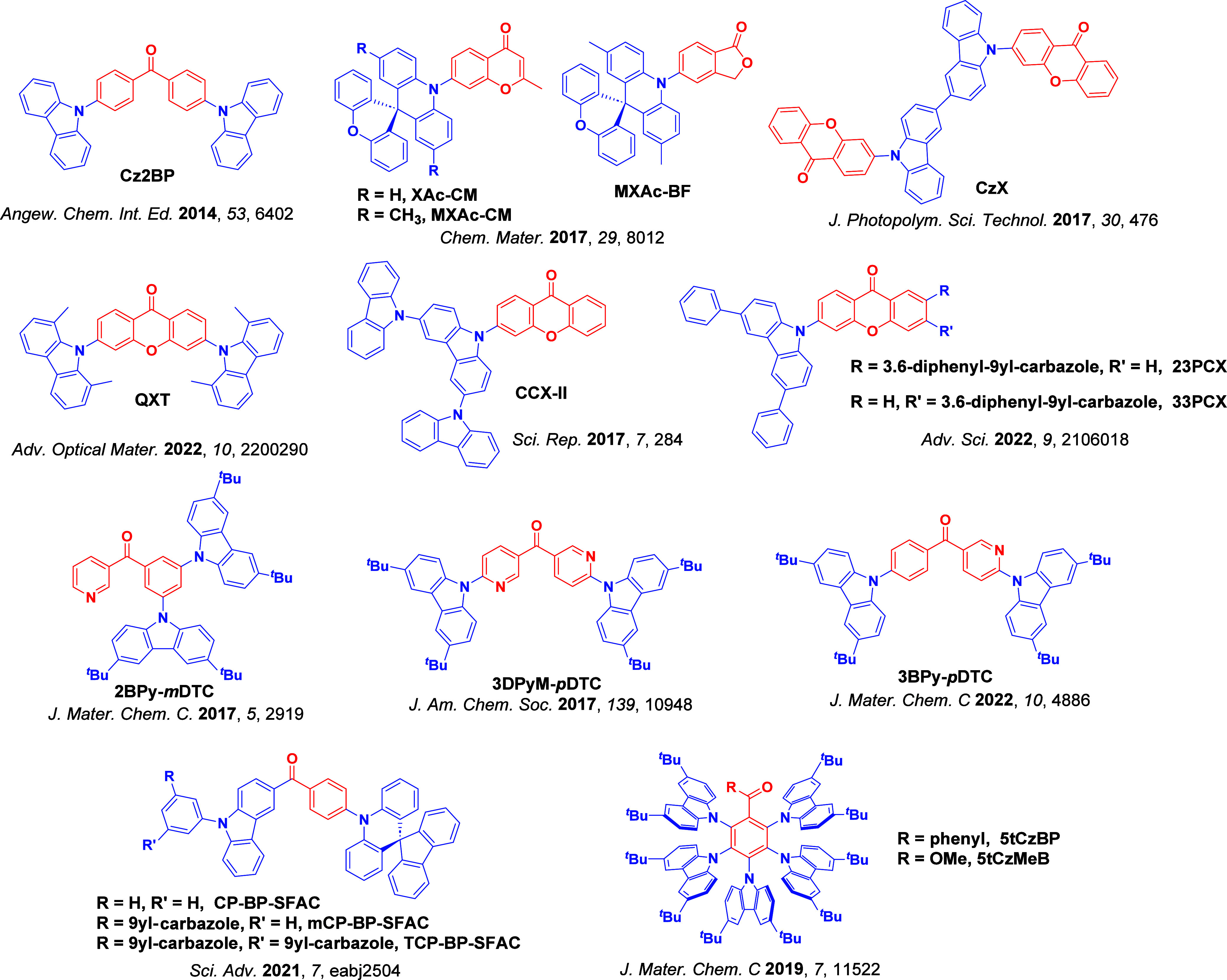
Molecular structures of ketone-based blue TADF emitters discussed here (the blue color signifies donor moieties, while the red color signifies acceptor moieties).

**CzX** (Figure 45) possesses an A-D-A design consisting of a central dicarbazole donor coupled to two terminal xanthone acceptors.^358^ This emitter produced a blue emitting device with λ_EL_ 482 nm and EQE_max_ 19.9%, along with a reasonable efficiency roll-off of ∼25% at 100 cd m^–2^. No lifetime or Δ*E*_ST_ were reported, although a modest Φ_PL_ of 54% in toluene suggests very efficient triplet conversion must have occurred. Another xanthone acceptor coupled to a tercarbazole donor dendron yielded the blue emitter **CCX-II**, which was demonstrated to have a high Φ_PL_ of 97% along with preferentially horizontally orientated TDM in 6 wt% doped films in PPF.^359^ The impressive EQE_max_ of 25.9% at CIE coordinates of (0.15, 0.22) was further enhanced to 33.3% with the use of an external outcoupling sheet. An extremely small Δ*E*_ST_ of 0.03 eV helped to support the efficient RISC that led to small efficiency roll-off values of 13 and 34% at 100 and 1000 cd m^–2^, respectively.

Min *et al*. designed symmetric D-A-D material **QXT** (Figure 45), featuring 1,8-dimethylcarbazole donors and a xanthone acceptor.^360^ The compound showed fast *k*_RISC_ of 2.4 ×10^6^ s ^–1^ in 20 wt% doped PPF films, and the OLED emitted at λ_EL_ of 480 nm with an EQE_max_ of 24.9%, along with a small efficiency roll-off of ∼13% at 1000 cd m^–2^. Zhang *et al*. instead used 3,6-diphenylcarbazole as the donors and connected them at different positions on a xanthone acceptor in **23PCX** and **33PCX**.^361^ **23PCX** and **33PCX** in 20 wt% doped films in PPF emit at λ_PL_ of 485 and 472 nm, with respective Φ_PL_ of 88 and 92%, and Δ*E*_ST_ below 0.05 eV for both, compared to λ_PL_ of 489 nm, Φ_PL_ of 96% and Δ*E*_ST_ of 0.02 eV for **QXT** in the same medium.^360^ The OLEDs with **23PCX** and **33PCX** emitted at λ_EL_ of 484 and 469 nm with CIE coordinates of (0.17, 0.36), and (0.16, 0.25) and EQE_max_ of 25.5 and 27.5%, all respectively. The efficiency roll-off of at 1000 cd m^–2^ was moderately large at 38 and 33% respectively.

A dipyridyl-ketone acceptor with *tert*-butylcarbazole donors (**3DPyM-*p*DTC**, Figure 45) exhibited blue emission with λ_PL_ of 464 nm, a high Φ_PL_ of 98%, a small Δ*E*_ST_ of 0.02 eV, and a short τ_d_ of 10 μs in 7 wt% doped mCBP films.^362^ Moreover, the TDM of **3DPyM-*p*DTC** in films adopts near-perfect horizontal orientation, leading to enhanced light outcoupling and supporting an EQE_max_ of 31.9%. The device CIE coordinates were (0.14, 0.18), but it also showed moderate efficiency roll-off of 18 and 49% at 100 and 1000 cd m^–2^. Replacing the one of the pyridines with a phenyl ring in **3BPy-*p*DTC** leads to blue-shifted emission at λ_PL_ of 453 nm in 7 wt% doped films in mCBP, compared to 475 nm for **3DPyM-*p*DTC**. This comes but at the cost of Δ*E*_ST_ increasing from 0.02 eV (for **3DPyM-*p*DTC**) to 0.19 eV though.^363^ The OLED with **3BPy-*p*DTC** showed an EQE_max_ of 25% at λ_EL_ of 458 nm with CIE coordinates of (0.14, 0.13) (Table S1). **2BPy-*m*DTC** is a related compound containing a similar pyridyl ketone acceptor coupled to *tert*-butylcarbazole donors.^364^ This compound emits at λ_PL_ of 476 nm, has a high Φ_PL_ of 92%, a small Δ*E*_ST_ of 0.05 eV, and τ_d_ of 8.3 μs in 7 wt% doped mCBP films. The OLEDs with **2BPy-*m*DTC** showed an EQE_max_ of 24.6% at CIE coordinates of (0.15, 0.28), along with modest efficiency roll-off at 100 cd m^–2^ of ∼13%.

Converting the phenyl-ketone in **5tCzBP** (Figure 45) to a methyl ester in **5tCzMeB** blue-shifts the emission, but also suppresses triplet non-radiative decay (3 × 10^6^ s^–1^ in **5tCzBP** to 0.3 × 10^6^ s^–1^ of **5tCzMeB**) while conserving *k*_RISC_ at around 4 × 10^6^ s^–1^ in toluene.^365^ The 20 wt% in DPEPO doped and non-doped OLEDs with **5tCzMeB** showed divergent performance at similar emission color, with EQE_max_ of 24.6% [λ_EL_ of 481 nm and CIE coordinates of (0.19, 0.32)] and 13.4% [λ_EL_ of 488 nm and CIE coordinates of (0.20, 0.36)], and both shared high efficiency roll-off of 33 and 44% at 100 cd m^–2^, all respectively. By contrast, the emission of the OLEDs with **5tCzBP** were shifted to the green, with λ_EL_ of 497 nm and a moderate EQE_max_ ≈ 10%.

Fu *et al*. constructed D-A emitters **CP-BP-SFAC**, **mCP-BP-SFAC**, and **TCP-BP-SFAC** (Figure 45) employing spiro[acridine-9,9′-fluorene] (SFAC) as electron donor and incorporated increasing numbers of terminal carbazoles in structures typically associated with host materials on the opposite side of a benzophenone acceptor.^366^ All three compounds showed AIE with Φ_PL_ above 80% in neat films, and generally similar photophysical properties with λ_PL_ at around 480 nm and Δ*E*_ST_ around 0.10 eV in either 20 wt% doped DPEPO or non-doped films. The non-doped devices with each of the three emitters exhibited sky-blue emission with λ_EL_ at around 489 nm and EQE_max_ values ranging from 22.5 to 26.1%, while for the 20 wt% doped DPEPO devices the EQE_max_ of each reached above 36.6% at λ_EL_ of around 480 nm. The extremely high EQE_max_ was ascribed to the high Φ_PL_ (∼100%) and preferential horizontal TDM orientation (Θ_//_ > 72%) in the DPEPO films.

The summary of ketone-containing blue TADF emitters is shown in Figure 46. Compound **3BPy-pDTC** exhibited the “bluest” emission with CIE coordinates of (0.14, 0.13), λ_EL_ of 458 nm, and EQE_max_ of 25.3%. The highest EQE_max_ of 38.6% was achieved in the device with **TCP-BP-SFAC**, which has CIE coordinates of (0.16, 0.28), whereas the device with **QXT** exhibited the smallest efficiency roll-off with EQE_max_ of 24.9% and EQE_1000_ of 21.7 at CIE coordinates of (0.16, 0.30). A large portion of the emitters based on ketone are classified as sky-blue. Thus, future research should be devoted to modulating the acceptor strength to promote a blue-shift of the emission.

**Figure 46:**
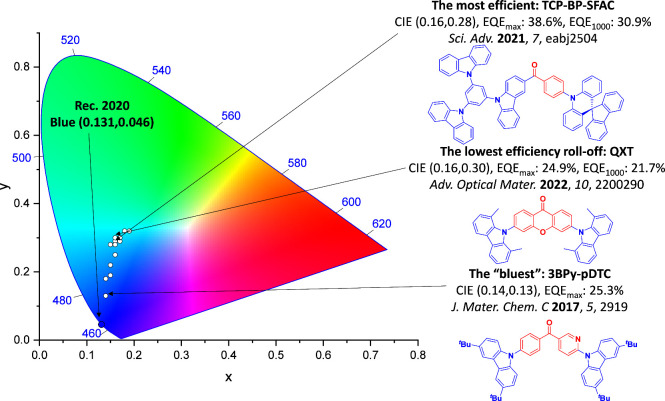
CIE color coordinates of blue D-A TADF emitters containing ketone acceptors. The white circles illustrate the spread of the emission color of the device. Selected devices and their associated CIE coordinates are highlighted, illustrating the structure of the emitter of the bluest device, the structure of the emitter used in the device showing the highest EQE_max_ and the structure of the emitter associated with the device showing the lowest efficiency roll-off. Only TADF OLEDs where the λ_EL_ < 490 nm are included. The device with the CIE coordinates closest to the Rec. 2020 defined coordinates for blue, (0.131, 0.046), is defined as the “bluest”. The most efficient device is quantified by the highest EQE_max_. The efficiency roll-off is quantified as the change in efficiency between EQE_max_ and EQE_1000_. In the chemical structures, the blue color signifies donor moieties, while the red color signifies acceptor moieties.

### 3.10 Other Emitters

Beyond the commonly used acceptor groups described above, there are a number of emitters that contain alternative or less frequently used acceptor groups. For example, OLED host materials featuring phosphine oxide groups are also sources of potential inspiration as weak acceptor units in the design of blue TADF emitters. The electron-withdrawing effect of the phosphine oxide (PO) groups adjusts excitonic ICT character for blue emission,^367^ while the sp^3^-hybridized phosphorus atom enhances molecular distortion of the frontier molecular orbitals, separating them to establish small Δ*E*_ST_.^368^

An acceptor with two PO groups at *meta* positions relative to carbazole donors around a central phenyl linker gave blue emitter ***m*2tBCzPO** (Figure 47). The corresponding device showed an EQE_max_ of 21.0% at CIE coordinates (0.16, 0.17) and good efficiency roll-off at 100 and 1000 cd m^–2^ of 7 and 26%, respectively. The higher efficiency of the device with ***m*2tBCzPO** compared to other derivatives where the PO groups are located at the *ortho* or *para* positions was ascribed to the better balance between electronic and steric effects that contributed to efficient RISC.^369^ A follow-up study of D–A–D variant **4tBCzDPDPO2A** utilized a phosphine oxide homo-conjugated acceptor to bridge four di-*tert*-butyl-carbazolyl groups.^370^ In comparison to **4tBCzPPOPO** and **4tBCzPPODPO**, which both adopted a non-conjugated D-A-A-D structure, the through-space conjugation effect in **4tBCzDPDPO2A** leads simultaneously to small Δ*E*_ST_ and improved oscillator strength, evidenced by doubling of Φ_PL_ and a quadrupling of *k*_RISC_. The device with **4tBCzDPDPO2A** showed an EQE_max_ of 23.7% with only 6 and 22% efficiency roll-off at 100 and 1000 cd m^–2^, respectively, with sky-blue emission at λ_EL_ of 470 nm and CIE coordinates of (0.18, 0.30). The low efficiency roll-off was attributed to the high RISC efficiency (94%) and fast radiative decay of 3.2 ×10^7^ s^–1^ of this emitter. The devices with **4tBCzPPOPO** and **4tBCzPPODPO** showed blue-shifted emission with λ_EL_ = 460 nm for both and CIE coordinates of (0.18, 0.23) and (0.19, 0.25), respectively. However, the low Φ_PL_ (∼30%) led to poor device efficiency with EQE_max_ reaching only 3.6 and 4.0%.

**Figure 47:**
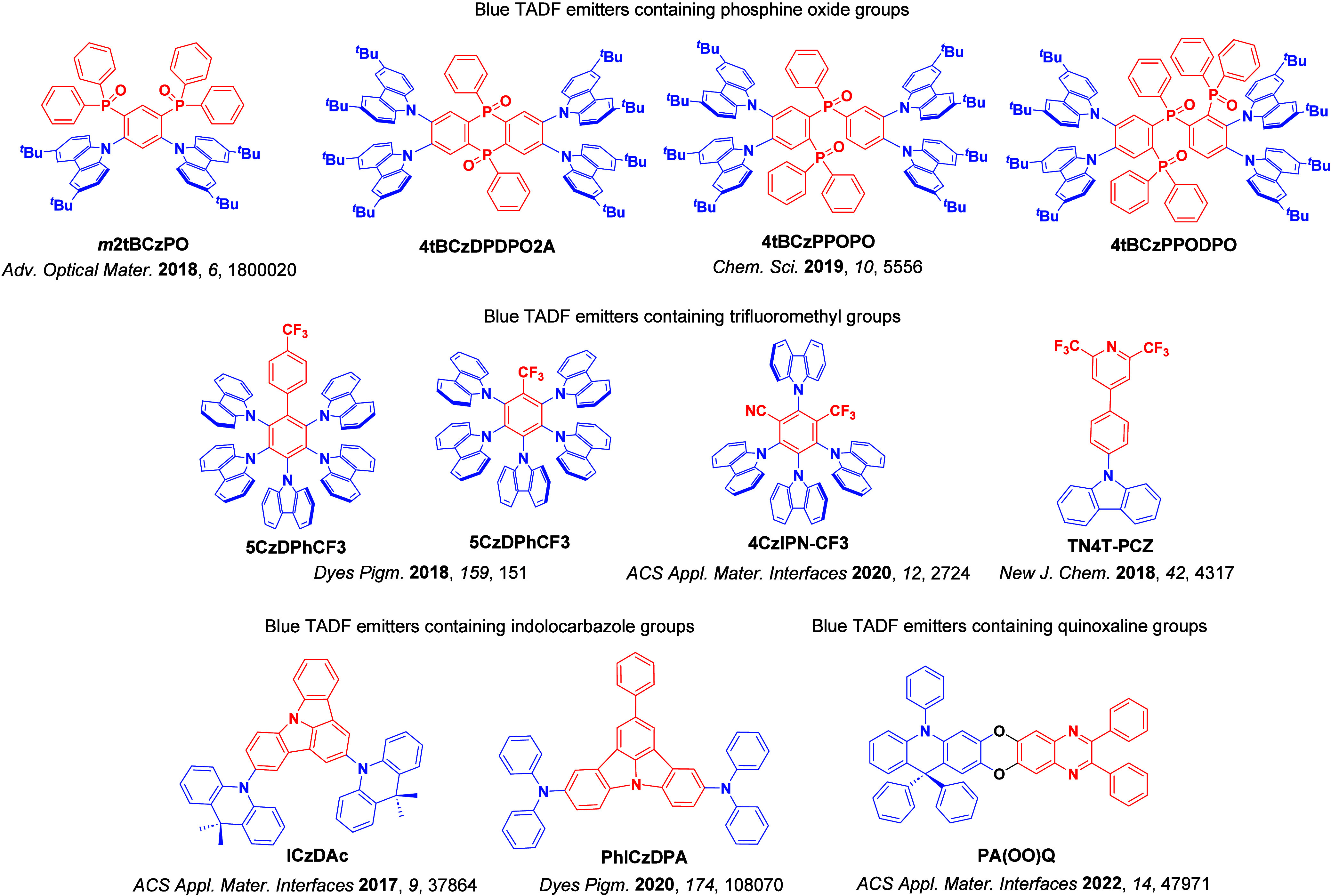
Molecular structures of blue emitters with one of phosphine oxide, trifluoromethyl, indolocarbazole, or quinoxaline as the acceptor moiety (the blue color signifies donor moieties, while the red color signifies acceptor moieties).

Trifluoromethyl (CF_3_) is of potential interest for producing deep-blue emission due to its weak electron-withdrawing ability [LUMO for (trifluoromethyl)benzene: −1.71 eV].^371^ CIE coordinates of (0.16, 0.07) were achieved in a device with **5CzDPhCF_3_**, which pairs this acceptor with carbazoles (Figure 47).^372^ A poor EQE_max_ of 2.0% and high efficiency roll-off of 75% at 100 cd m^–2^ were noted though, likely due to the low Φ_PL_ of 27% and the long τ_d_ of 0.14 ms (Table S1). The additional phenylene spacer in **5CzDPhCF_3_** proved essential for deep-blue emission, as the CIE coordinates shifted to (0.18, 0.33) for the device with **5CzDPhCF_3_**. Similarly, replacing one of the cyano groups in **4CzIPN** for CF_3_ in **4CzIPN-CF3** tuned the emission from green to sky-blue.^373^ A device with **4CzIPN-CF3** exhibited sky-blue emission at λ_EL_ of 487 nm, benefiting from the high emitter Φ_PL_ (77%) and an exciplex host system to acheive the EQE_max_ of 23.1% with only 10% efficiency roll-off at 1000 cd m^–2^. Compound **TN4T-PCZ** is another carbazole-containing blue emitter coupled with a trifluoromethyl-substituted pyridine as the acceptor.^374^ **TN4T-PCZ** emits at λ_PL_ of 411 nm, has a Φ_PL_ of 87%, and a small Δ*E*_ST_ of just 100 meV. The device with **TN4T-PCZ** showed deep-blue emission with λ_EL_ of 415 nm, CIE coordinates of (0.16, 0.03), and an EQE_max_ of 20.4%, making it one of the most efficient deep-blue D-A TADF OLEDs to date.

Coupling iCz with acridine donors produced the blue TADF emitter **ICzDAc** (Figure 47) with Δ*E*_ST_ of 0.17 eV and 98% Φ_PL_ in 10 wt% doped DPEPO films.^375^ The device with **ICzDAc** showed an EQE_max_ of 19.7% although with large 50% efficiency roll-off at 1000 cd m^–2^, with blue emission at CIE coordinates of (0.15, 0.16). A follow-up study instead used diphenylamines as donor groups in **PhICzDPA**, which maintained the high Φ_PL_ of 94% and a reduced Δ*E*_ST_ (0.12 eV) in 10 wt% doped DPEPO films. The device with **PhICzDPA** exhibited a high EQE_max_ of 30.4% and sky-blue emission with CIE coordinates of (0.13, 0.32).^376^ However, this device also showed severe efficiency roll-off (43% at 100 cd m^–2^) and failed to reach 1000 cd m^–2^. This behavior can be rationalized by the long-lived excitons (τ_d_ = 249 μs) that are prone to quenching by multi-excitonic non-radiative decay processes under electrical excitation.

Wang *et al*. designed unique D–A emitter **PA(OO)Q** (Figure 47), containing a fused ring structure where coplanar acridine donor and quinoxaline acceptor were connected by two oxygen-bridges within a six-membered ring.^377^ Although DFT calculations predicted a small HOMO/LUMO overlap, the emitter with a more planar geometry still has a large Δ*E*_ST_ of 0.35 eV and a long τ_d_ of 1.5 ms in 5 wt% doped films in mCBP. The OLED exhibited sky-blue emission with λ_EL_ of 488 nm, CIE coordinates of (0.19, 0.37), and EQE_max_ of 19.5%. However, the device showed severe efficiency roll-off of 73% at 100 cd m^–2^ due to the slow RISC process (*k*_RISC_ = 1.1×10^3^ s ^–1^), likely resulting from its unusual structure.

**Figure 48:**
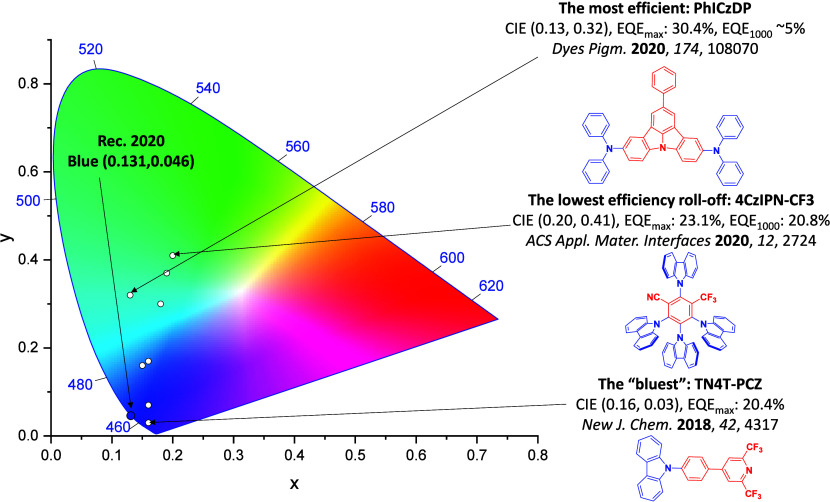
CIE color coordinates of blue D-A TADF emitters containing acceptors illustrated in Figure 47. The white circles illustrate the spread of the emission color of the device. Selected devices and their associated CIE coordinates are highlighted, illustrating the structure of the emitter of the bluest device, the structure of the emitter used in the device showing the highest EQE_max_ and the structure of the emitter associated with the device showing the lowest efficiency roll-off. Only TADF OLEDs where the λ_EL_ < 490 nm are included. The device with the CIE coordinates closest to the Rec. 2020 defined coordinates for blue, (0.131, 0.046), is defined as the “bluest”. The most efficient device is quantified by the highest EQE_max_. The efficiency roll-off is quantified as the change in efficiency between EQE_max_ and EQE_1000_. In the chemical structures, the blue color signifies donor moieties, while the red color signifies acceptor moieties.

Although the acceptors discussed in this section are under-explored compared to other classes of acceptors discussed above, these studies nonetheless illustrate their great potentials in terms of approaching the standard blue CIE coordinates and exhibiting high device performance (Figure 48). Thus, many of these “exotic” acceptors deserve greater attention in the design of blue TADF emitters.

### 3.11 Outlook

The period between 2017–2022 has witnessed an intense search for an ideal blue emitter, which to this day remains elusive. Tremendous efforts have translated into numerous examples of blue TADF OLEDs achieving EQE_max_ greater than 20%, yet only a handful of examples achieved the desired deep-blue emission (CIE_y_ < 0.1) while maintaining this high efficiency (Figure 49). Most of these deep-blue devices also suffer from unacceptable efficiency roll-off at practical brightnesses, and there also remains a lack of concerted effort to quantify device lifetimes necessary to correlate emitter structure to device stability.

**Figure 49:**
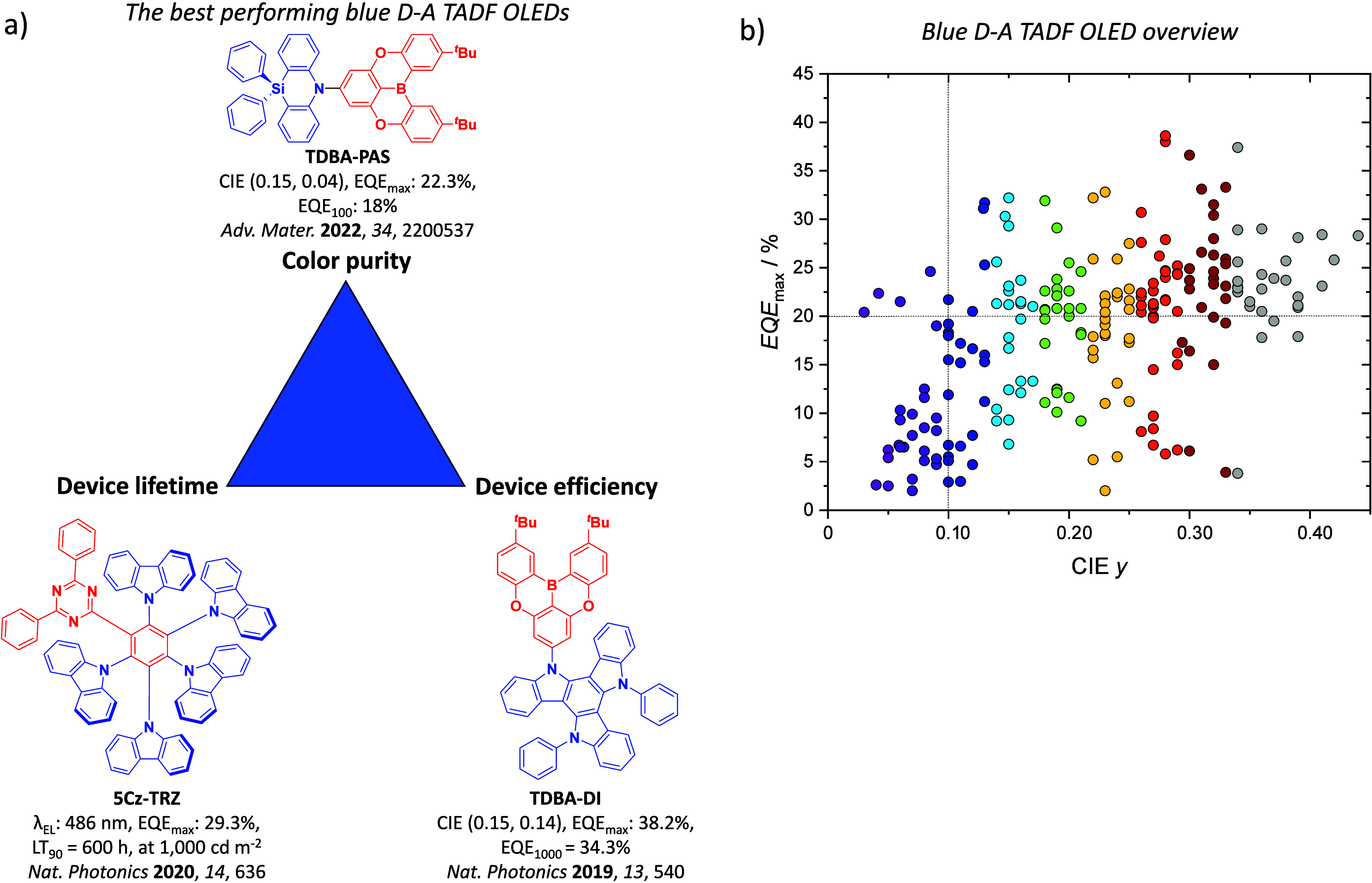
a) Selected structures of the emitters of the best performing blue OLEDs summarized in this section, with respect to color purity, device lifetime, and maximum efficiency (the blue color signifies donor moieties, while the red color signifies acceptor moieties). b) EQE_max_ vs CIE_y_ coordinate of all the blue OLEDs reviewed in this section. Different colors act as a visual guide (non-literal) of the device emission color.

Triazine by far remains the most popular acceptor in the design of blue emitters. Deep blue emission can be achieved when triazine is combined with weak donors (carbazoles, carbolines, imidazoles), as exemplified by **DCBTRZ** (EQE_max_ of 6.6%) where the device achieves CIE coordinates of (0.15, 0.056).^243^ There are a large number of highly efficient devices with reported EQE_max_ exceeding 30%, most notably for **TspiroS-TRZ**, which achieved EQE_max_ of 33.3%.^259^ The molecular shape of **TspiroS-TRZ** helps to promote horizontal emitting dipole orientation, which supports this impressive device performance.

While device lifetime is clearly a central concern for commercial applications, only some studies report device lifetime information. For example, the device with **PPCzTrz** demonstrated LT_50_ of 24 h at an initial 1000 cd m^–2^,^253^ which was later improved to an LT_80_ of 30.5 hours using a phenylated derivative **CzTrzBp**.^262^ By far the longest reported blue OLED lifetime belongs to **5Cz-Trz**, with a LT_90_ of ca. 600 h at inital 1,000 cd m^–2^,^93^ although this device with λ_EL_ of 486 nm, actually is sky-blue.

Boron-containing D-A emitters have also emerged as a subclass with generally attractive photophysical properties for blue emitters. Examples of OLEDs based on rigid DBA emitters frequently surpass EQE_max_ >30%. Of these, the bluest OLED incorporated **TDBA-Ac** (CIE coordinates of 0.15, 0.05)^304^ and the most efficient and stable OLEDs were fabricated using emitters with a rigid triazatruxene donor: **TDBA-DI** (EQE_max_ = 38.2%)^304^ and **DBA-DI** (EQE_max_ = 28.1%, with efficiency roll-off of 1% at 1000 cd m^–2^).^305^ The device with the longest lifetime was one employing **DBA-DI**, with LT_50_ of 329 h at an initial 1000 cd m^–2^, albeit again with sky blue emission (λ_EL_ ∼ 470 nm). As Figure 49 depicts, two out of three the best-performing emitters summarized here feature the DBA acceptor. However, device lifetime studies remain limited and the most stable devices to date are still based on early triazine-carbazole hybrids.

Aside from boron or triazine-based acceptors, while deep blue emission is readily achieved using pyrimidine-based emitters, these OLEDs typically struggle to achieve CIE_y_ < 0.1. Relatively stronger electron-accepting nitrile, oxadiazole, and ketone acceptors are less preferred chromophores for the design of deep-blue emitters, but feature heavily in green TADF emitter design (Section 4). The same color-tuning considerations also apply to derivatives that contain multiple donors. Numerous examples of OLEDs with sulfone-containing emitters achieve CIE_y_ < 0.1; however, these devices also typically show a significant efficiency roll-off. This may be due to suspected photochemical instability of the diphenylsulfone-type acceptor, although equally may be due to similar suspected instability of DPEPO and other phosphine oxide materials that are currently the only suitable hosts for such high T_1_ emitters.

Evidently, the triazine and boron acceptor-based emitters are the most promising designs for highly efficient deep-blue D-A TADF emitters with CIE_y_ < 0.10. In particular, the devices with boron acceptor-based emitters showed excellent color purity in the deep-blue region and had the highest device efficiency but possessed poor device stability, whereas the triazine acceptor-based emitters showed excellent device stability. In analyzing and aggregating the optoelectronic and device data presented in this section there are some important trends that inform the design of efficient deep-blue D-A TADF emitters; 1) a large dihedral angle between appropriately chosen donor and acceptor is essential for spatial HOMO and LUMO separation to attain sufficiently small Δ*E*_ST_ while maintaining the oscillator strength to the S_1_ state, which is a very challenging task; 2) a rigid molecular structure is desirable to help avoiding non-radiative decay and to maintain high Φ_PL_, although this is often counteracted by larger dihedral angles; 3) a large planar (e.g., diindolocarbazole) or linear difunctionalized donor (e.g., spiro-acridine) moiety seems to facilitate the horizontal alignment of the TDM in the emissive layer, leading to enhanced light outcoupling efficiencies. Thus, though much progress has been made and many highly efficient, deeply blue emissive, or highly stable emitters have been designed or discovered, the search for a single material simultaneously possessing all these traits continues as a central research focus of the global organic electronics research community.

## 4 Green TADF Emitters λ_EL_ 490–580 nm

### 4.1 Introduction

Green emitters, which we define as those having λ_EL_ between 490 and 580 nm, have emerged as the largest class of TADF emitters, and ones that lead to OLEDs with some of the highest reported efficiencies. The ground-breaking paper by Adachi and co-workers indeed featured a green device using **4CzIPN** (Figure 50), with efficiencies nearing 20% that were unprecedented for an OLED using an organic emitter.^378^ Unburdened by molecular instability and restricted choice of hosts faced by blue emitters (Section 3), while still being sufficiently high in energy to avoid the energy gap law that hinders the efficiency of red emitters (Section 5), the reported efficiencies of green TADF devices and the number of reported green-emissive TADF compounds have steadily increased year on year.

**Figure 50:**
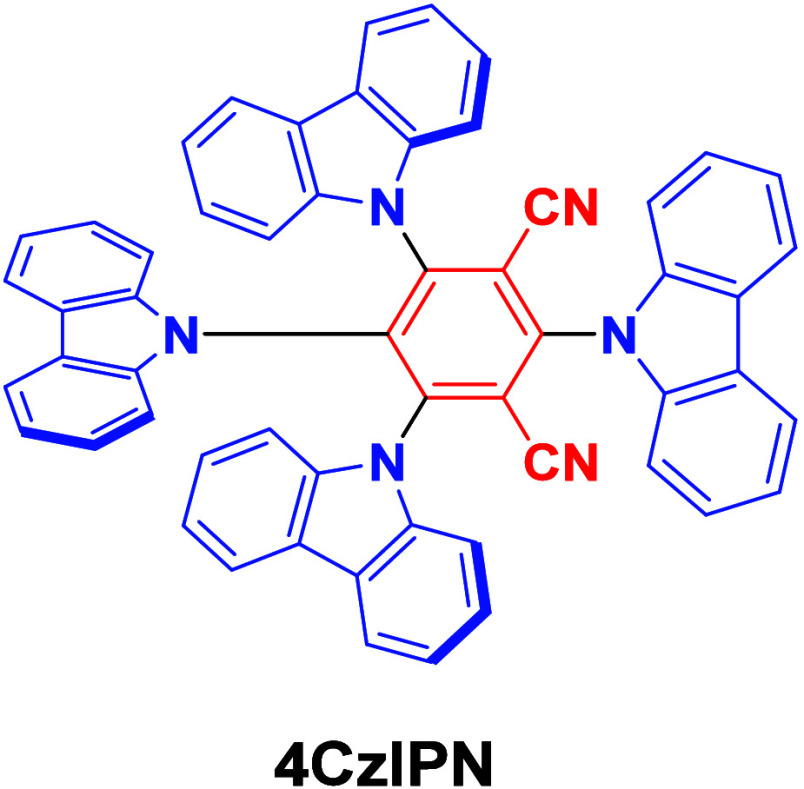
Molecular structure of **4CzIPN**, one of the first notable green TADF emitters. Note the twisted donor carbazole groups with respect to the isophthalonitrile acceptor. The blue color signifies donor moieties, while the red color signifies acceptor moieties.

Due to the expansiveness of the green TADF emitter literature, here we restrict our scope to purely organic D-A emitters reported since 2017 where the OLED showed an EQE_max_ > 20% and/or exhibited notably low efficiency roll-off and high brightness. Similar to the organization in Section 3, the emitters in Section 4 are classified in subsections according to the acceptors and their key photophysical and device properties are summarized in Table S2. Green TADF emitters with other molecular designs (through-space TADF (Section 12), MR-TADF (Section 11), and metal-based TADF (Section 9)) or green emitters designed with alternate applications in mind (chiral TADF, assistant dopants, and others) are summarized in other relevant sections of this review.

### 4.2 Nitrile-Based Acceptors

As a relatively simple and synthetically accessible withdrawing group, nitrile acceptors featured heavily in the seminal Nature paper authored by Adachi and co-workers describing TADF from a series of carbozolyl dicyanobenzene compounds.^378^ Many TADF emitters have since been reported using one or more cyano groups within the acceptor moiety, with various donor groups either directly connected to the same aryl ring as the cyano group, or *via* a bridging aryl group. The number, type, and positions of these substituents impact both the emission energy and the efficiency of the TADF process.^119–121,379^ For example, by altering the positions of additional benzonitrile substituents in phenoxazine-cyanobenzene compounds from the *meta* (**mPTBC**) to the *ortho* (**oPTBC**) position, different emission colors were observed (λ_PL_ of 518 and 561 nm, respectively, in toluene).^380^ These two compounds have comparable Φ_PL_ and Δ*E*_ST_ with 58.4% and 0.006 eV for **mPTBC** and 57.6% and 0.007 eV for **oPTBC** (Figure 51). This in turn resulted in similar device performance with EQE_max_ of 18.1% (λ_EL_ = 516 nm) and 17.8% (λ_EL_ = 540 nm) (Table S2). Both devices showed good efficiency roll-off with efficiency at 1000 cd m^–2^ declining by only 13 and 18% for **mPTBC** and **oPTBC**, respectively.

**Figure 51:**
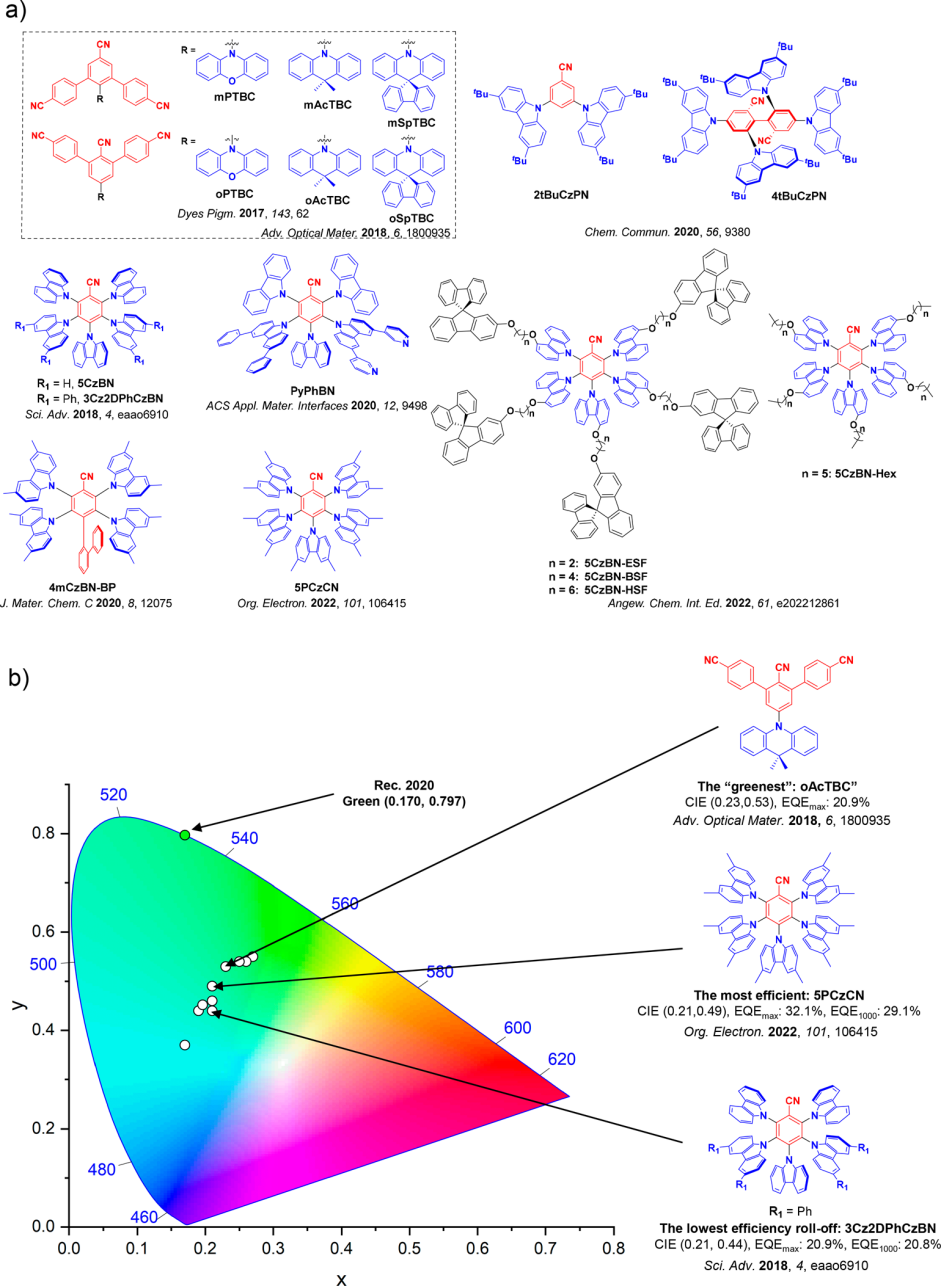
a) Molecular structures of green D-A TADF emitters containing nitrile acceptors and b) CIE color coordinates of green D-A TADF emitters containing nitrile acceptors. The white circles illustrate the spread of the emission color of the device. Selected devices and their associated CIE coordinates are highlighted, illustrating the structure of the emitter of the “greenest” device and the structure of the emitter used in the device showing the highest efficiency and the lowest efficiency roll-off. Only D-A TADF OLEDs where the λ_EL_ = 490–580 nm that show EQE_max_ > 20% or have minimal efficiency roll-off are included. The most efficient device is quantified by the highest EQE_max_. The device with the CIE coordinates closest to the Rec. 2020 defined coordinates for green, (0.170, 0.797), is defined as the “greenest”. In the chemical structures the blue color signifies donor moieties, while the red color signifies acceptor moieties.

The same group subsequently investigated the impact of restricting molecular motions upon photophysical properties in related structures **oAcTBC** and **mAcTBC**, employing an acridine donor instead of phenoxazine (Figure 51).^381^ Steric restriction was increased by once again altering the positions of the auxiliary benzonitrile substituents. Replacing DMAC for a spirofluorene derivative afforded **oSpTBC** and **mSpTBC**. The emitters **oAcTBC**, **oSpTBC**, **mAcTBC**, and **mSpTBC** have Φ_PL_ values of 84, 93, 77, and 65% respectively, doped at between 10–27 wt% in mCP. The increase in Φ_PL_ from **oAcTBC** to **oSpTBC** was expected since the more rigid *spiro*-based donor suppresses *k*_nr_. A decrease in Φ_PL_ was observed in the *meta* derivatives, which may be due to the larger dihedral angle between donor and acceptors. The four compounds all show small Δ*E*_ST_ of between 0.01 and 0.03 eV, along with similar τ_d_ between 13.3 and 17.4 μs. EQE_max_ of 20.9 (λ_EL_ = 512 nm), 26.8 (λ_EL_ = 508 nm), 19.2 (λ_EL_ = 496 nm), and 18.9% (λ_EL_ = 492 nm) were obtained for the devices with **oAcTBC** (10 wt% in mCP), **oSpTBC** (16 wt% in mCP), **mAcTBC** (27 wt% in mCP), and **mSpTBC** (24 wt% in mCP), all respectively, with the device efficiencies correlating with the respective Φ_PL_ (Table S2). Reduced efficiency roll-off at 1000 cd m^–2^ was observed for the device with **oAcTBC** (17%) compared to **oSpTBC** (29%), which was attributed to the higher prompt fluorescence contribution (34% compared to 25%). Efficiency roll-off of 37 and 26% was noted for the devices with **mAcTBC** and **mSpTBC**, respectively.

Wang *et al*. reported the emitter **4tBuCzPN** (Figure 51) that contains two *ortho*-bonded benzonitrile groups as the acceptor.^382^ Developing from the simpler D-A-D structure of **2tBuCzPN**, the dual-core and axially chiral **4tBuCzPN** is more conformationally rigid and also adopts a more twisted structure. The Φ_PL_ of **4tBuCzPN** (74%) is much higher than in **2tBuCzPN** (29%) while the t_d_ decreases from 14.1 to 4.0 μs (Table S2). The resulting OLED performance improve dramatically, with EQE_max_ of 5.3 and 20.8% for the devices with **2tBuCzPN** and **4tBuCzPN**, respectively. The chiroptical properties of **4tBuCzPN** are discussed in Section 7.

A strategy to control the relative energies of ^3^CT and ^3^LE states was proposed by Noda *et al.*, whereby the ^3^LE level was brought close to the ^3^CT state by the addition of a second type of donor unit.^91^ The structure of the original **5CzBN** emitter was altered to phenyl-substitute two of the carbazoles in **3Cz2DPhCzBN** (Figure 51). The compound emits at λ_PL_ of 495 nm and has a Φ_PL_ of 80%, compared to 24% at 520 nm for **5CzBN**, both in 20 wt% doped mCBP films. Additionally, **3Cz2DPhCzBN** has an improved *k*_RISC_ of 9.9 × 10^5^ s^–1^ compared to 3.6 × 10^5^ s^–1^ for the parent emitter (Table S2). The devices with **5CzBN** and **3Cz2DPhCzBN** showed similar EQE_max_ of 18.0 and 20.9%, respectively, and thanks to faster RISC the device with **3Cz2DPhCzBN** showed markedly reduced efficiency roll-off (11% at 5000 cd m^–2^, compared to 23% for **5CzBN**). Furthermore, better operational stability was demonstrated for the device with **3Cz2DPhCzBN** with an LT_97_ of 110 hours at 1000 cd m^–2^, compared to just 3 hours for the device with **5CzBN**.

Similar structural modification of **5CzBN** was reported by Balijapalli *et al.*, in which phenyl, pyridyl, and trifluoromethyl groups were substituted onto the carbazole donors of **5CzBN**.^383^ Of the family of compounds, the one with the most attractive set of emission properties was **PyPhBN** (Figure 51), which possesses three unsubstituted carbazole donors, one carbazole extended with two phenyl units, and another decorated with two pyridine units. These modifications led to a Φ_PL_ of 92% and a Δ*E*_ST_ of 0.13 eV, which translated into a device with improved EQE_max_ of 20.6% at 501 nm. Woo *et al.* also reported a modified version of **5CzBN** in compound **4mCzBN-BP**, containing four dimethylcarbazole donors about a benzonitrile acceptor core as well as an *ortho*-biphenyl substituent *para* to the nitrile.^384^ The *ortho*-biphenyl enforces a large steric hindrance and larger D-A dihedral angles between the carbazoles and the acceptor core, while the ^3^LE of the biphenyl group can couple with ^3^CT of **4mCzBN-BP** to accelerate RISC compared to the parent molecule. **4mCzBN-BP** emits at λ_PL_ of 491 nm, has a Φ_PL_ 95%, and a *k*_RISC_ of 2.28 × 10^6^ s^–1^ in 10 wt% doped mCP films (Table S2). The device showed an EQE_max_ of 23.1% at λ_EL_ of 496 and CIE coordinates of (0.20, 0.45), and showed moderate efficiency roll-off of 26% at 400 cd m^–2^. Zhang *et al.* reported the emitter **5PCzCN**, which has five dimethylcarbazole donors and emits at λ_PL_ of 489 nm with a high Φ_PL_ of 96.5% and a small Δ*E*_ST_ of 0.028 eV, in 10% doped mCP films.^385^ The OLED with **5PCzCN** showed green emission at 504 nm [CIE coordinates of (0.21, 0.49)] with an excellent EQE_max_ of 32.1% and efficiency roll-off of 9.3% at 1000 cd m^–2^. This increase in device performance compared to **5CzBN** highlights the crucial importance of balancing donor strength to achieve efficient RISC, while the peripheral methyl substituents likely also help to suppress concentration quenching. The OLED also displayed high stability with LT_50_ of 95.5 h at 1000 cd m^–2^.

Unlike vacuum-deposited OLEDs, which can show enhanced light-outcoupling when the TDM of the emitters are preferentially aligned, solution-processed devices with the same emitter typically exhibit no improved light-outcoupling as the processing technique results in isotropic orientation of the TDMs. However, Zhao *et al*. demonstrated that by attaching flexible alkyl chains terminated with spirobifluorene groups to **5CzBN**, these groups helped not only to improve the solubility for solution-processed OLEDs, aided carrier mobility, and likely assisted in preventing aggregation quenching, but crucially also supported spontaneous horizontal orientation of the emitter TDM. Measurements of Θ// gave values of 72–73% for **5CzBN**, compared to 67% (i.e., isotropic alignment) for **5CzBN-Hex** in solution-processed neat films.^386^ **5CzBN-ESF**, containing the shortest alkyl chain of the series, emits at λ_PL_ of 480 nm and achieved the highest Φ_PL_ of 80%, with a small Δ*E*_ST_ of 0.06 eV and a short τ_d_ of 1.82 μs in toluene (Table S2). OLEDs with **5CzBN-ESF** showed high EQE_max_ of 30.6% and λ_EL_ of 508 nm [CIE coordinates of (0.27, 0.55)], with efficiency roll-off of 33% at 1000 cd m^–2^.

### 4.3 Boron-Containing Acceptors

Many acceptors have been developed using the inherent electron-withdrawing ability of the lowest-lying vacant p-orbital of boron. Two emitters, **ACBM** and **SACBM** (Figure 52), containing a simple N-borylated acceptor unit coupled to various acridine-based donors were reported.^387^ **ACBM** and **SACBM** emit at λ_PL_ of 527 and 518 nm and have Φ_PL_ of 76 and 99%, respectively, in 8 wt% and 4 wt% doped films in 2,6-DCzppy (Table S2). Moderate Δ*E*_ST_ of 0.11 eV for both **ACBM** and **SACBM** along with short τ_d_ of 3.0 and 2.6 μs, respectively, resulted in low efficiency roll-off in the devices. The OLEDs with **ACBM** and **SACBM** showed EQE_max_ of 11.2 and 19.1% at CIE coordinates of (0.33, 0.56) and (0.22, 0.59), and efficiency roll-offs of 9 and 2% at 100 cd m^–2^, all respectively. Significant research effort has since followed in the use of boron as part of acceptor systems for D-A TADF materials.

**Figure 52:**
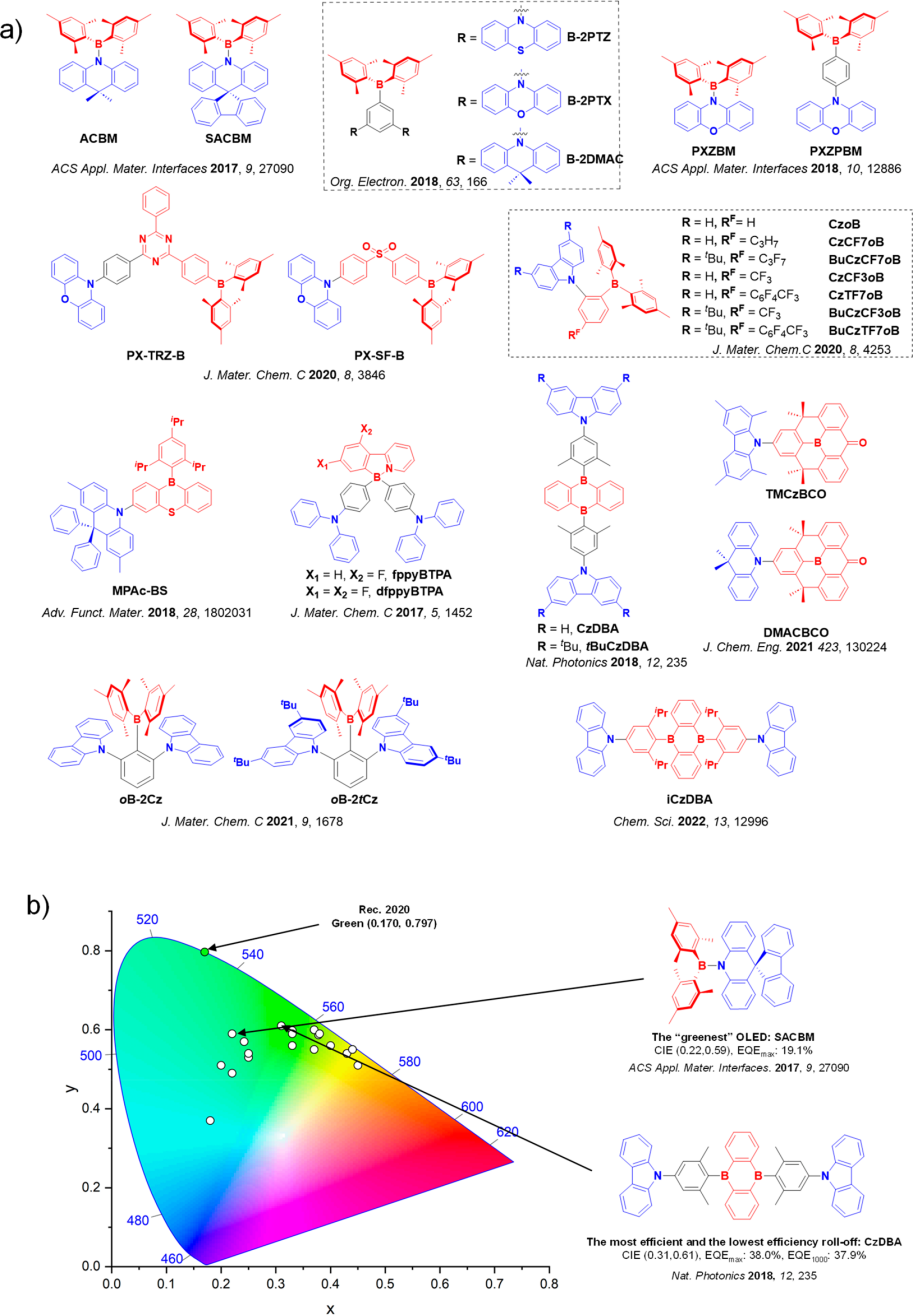
a) Molecular structures of green D-A TADF emitters containing boron acceptors and b) CIE color coordinates of green D-A TADF emitters containing boron acceptors. The white circles illustrate the spread of the emission color of the device. Selected devices and their associated CIE coordinates are highlighted, illustrating the structure of the emitter of the “greenest” device and the structure of the emitter used in the device showing the highest efficiency and the lowest efficiency roll-off. Only D-A TADF OLEDs where the λ_EL_ = 490–580 nm that show EQE_max_ > 20% or have minimal efficiency roll-off are included. The most efficient device is quantified by the highest EQE_max_. The device with the CIE coordinates closest to the Rec. 2020 defined coordinates for green, (0.170, 0.797), is defined as the “greenest”. In the chemical structures, the blue color signifies donor moieties, while the red color signifies acceptor moieties.

In another report, D-A-D derivatives using the same acceptor and similar donors attached *meta* to the acceptor were designed. **B-2DMAC** (Figure 52, λ_PL_ of 505 nm, Δ*E*_ST_ of 0.03 eV, Φ_PL_ of 46.8%, τ_d_ of 3.4 μs, and *k*_RISC_ of 0.8 × 10^5^ s^–1^) gave the highest performance green device, with λ_EL_ = 507 nm, EQE_max_ = 19.3% [CIE coordinates of (0.25, 0.53)], and efficiency roll-off of 6% at 100 cd m^–2^ and 21% at 1000 cd m^–2^.^388^ Devices with the phenothiazine (**B-2PTZ**) and phenoxazine (**B-2PXZ**) analogues emitted at λ_EL_ of 556 and 544 nm [CIE coordinates of (0.43, 0.54) and (0.40, 0.56)] with similar efficiency roll-off but lower EQE_max_ (7.6 and 10.1%) resulting from the lower Φ_PL_ (18 and 26%). The same acceptor was again used in two similar emitters that have only a single donor group, either with (**PXZPBM**) or without a phenylene linker (**PXZBM**).^389^ The steric hindrance between the phenoxazine and dimesitylboryl in **PXZBM** caused a puckered conformation of the phenoxazine in the ground state, leading to elongation of the N-B bond and reduced *π*-conjugation in the excited state. The introduction of the spacer in **PXZPBM** permitted the phenoxazine to adopt a planar conformation (itself perpendicular to the acceptor) and emit from a charge transfer state. The presence of the spacer also improved Φ_PL_ from 36 to 80%, reduced Δ*E*_ST_ from 0.13 to 0.08 eV, and a shortened τ_d_ from 3.6 to 2.2 μs, all respectively in toluene (Table S2). In turn, contrasting device performances were observed with EQE_max_ of 10.9% compared to 22.6%, at λ_EL_ = 567 [CIE coordinates of (0.45, 0.51)] and 505 nm [CIE coordinates of (0.25, 0.54)] for the OLEDs with **PXZBM** and **PXZPBM** respectively. Both compounds showed low efficiency roll-off with the EQE_100_ decreasing by either 10 or 1% compared to their respective maximum values. The EQE_1000_ remained as high as 20.8% for the device with **PXZPBM**, representing an 8% efficiency roll-off; by contrast, due to the larger Δ*E*_ST_ and longer τ_d_ of **PXZBM** there was a significantly larger efficiency roll-off of 63% at 1000 cd m^–2^ for this device.

The same dimesitylborane acceptor was again used in another report by Qu *et al*. to produce D-A-A’ emitters **PX-TRZ-B** and **PX-SF-B** (Figure 52).^390^ Small Δ*E*_ST_ values 0.037 and 0.013 eV for **PX-TRZ-B** and **PX-SF-B** in toluene, respectively, are achieved due to large HOMO-LUMO spatial separation, caused by the expanded LUMO distribution over the tandem acceptor. **PX-SF-B** has the higher Φ_PL_ of 84% (compared to 65%) in 5 wt% doped CBP films. This translated into devices with higher EQE_max_ of 24.8% (10 wt% in CBP) at λ_EL_ of 535 nm [CIE coordinates of (0.37, 0.55)] for the device with **PX-SF-B**, compared to **PX-TRZ-B** with an EQE_max_ of 18.6% (10 wt% in CBP) at λ_EL_ of 557 nm [CIE coordinates of (0.43, 0.54)]; a small increase in EQE_max_ (19.2%) was observed at lower device loading (5 wt% in CBP). The efficiency roll-off for both materials was impressive with almost no loss in performance up to 1000 cd m^–2^, likely supported by the additional charge transporting properties of either the triazine or diphenylsulfone groups.

The addition of perfluoroalkyl (CF_3_ and C_3_F_7_) and perfluoroaryl (4-CF_3_C_6_F_4_) units to a reference boron-based emitter, **Cz*o*B** (Figure 52), allowed Kumar *et al*. to develop a series of compounds that reached Φ_PL_ of up to 100% in toluene.^391^ Amongst this family of emitters, **CzCF3*o*B**, **BuCzCF3*o*B**, and **BuCzTF7*o*B** were identified as the most promising. The **CzCF3*o*B** device showed an EQE_max_ of 22.9% at λ_EL_ of 517 nm [CIE coordinates of (0.24, 0.57)] and with an efficiency roll-off of 2 and 23% at 100 and 1000 cd m^–2^ (Table S2). The device with **BuCzTF7*o*B** showed an EQE_max_ of 21.9% with almost no efficiency roll-off at 100 cd m^–2^ and a 16% decrease at 1000 cd m^–2^. This device also showed the most red-shifted emission [λ_EL_ = 550 nm, CIE coordinates of (0.33, 0.60)], demonstrating the color tuning utility of these perfluorinated substituents.

A series of emitters using a dibenzo[b,e][1,4]heteraborin acceptor with an acridine donor were reported by Park *et al*.^297^ Of these, **MPAc-BS** (Figure 52) has the most noteworthy properties and emits at λ_PL_ of 497 nm, has Φ_PL_ of 100%, a small Δ*E*_ST_ of 0.023 eV, and a τ_d_ of 1.3 μs in 50 wt% doped films in PPF. The OLED with **MPAc-BS** showed an EQE_max_ of 25.3% at λ_EL_ of 503 nm [CIE coordinates of (0.20, 0.51)] and showed a very mild efficiency roll-off of only 1.2 and 6.3% at 100 and 1000 cd m^–2^, respectively. **CzDBA** and **tBuCzDBA** contain a similar diboroanthracene acceptor unit in combination carbazole donors and have similarly small Δ*E*_ST_ of 0.03 and 0.02 eV in 10% doped film in CBP, along with very fast τ_d_ of 3.2 and 2.1 μs, and high Φ_PL_ of 100 and 86%, all respectively (Table S2).^392^ The devices showed EQE_max_ of 37.8 and 32.4% at λ_EL_ of 528 [CIE coordinates of (0.31, 0.61)] and 542 nm [CIE coordinates of (0.37, 0.60)], respectively, as well as excellent efficiency roll-off at 1000 cd m^–2^ of 0.3 and 3%, representing some of the highest-performance green-emissive devices to date. The high EQE_max_ was attributed to both the high Φ_PL_ and the preferentially horizontally oriented TDMs arising from the rod-like molecular design.

Ouyang *et al*. reported a derivative of **Cz*o*B** that contains an additional carbazole donor at the *ortho* position of triarylborane, resulting in D-A-D materials **oB-2Cz** and **oB-2tCz** (Figure 52).^393^ The large D-A dihedral angles enforced by the double *ortho* substitution and the highly rigid structure gave rise to small Δ*E*_ST_ values of 0.06 and 0.03 eV, high Φ_PL_ of 93 and 96%, and fast *k*_RISC_ of 5.17 and 17.06 ×10^5^ s^–1^ for **oB-2Cz** and **oB-2tCz**, all respectively (Table S2). The devices with **oB-2Cz** and **oB-2tCz** showed EQE_max_ of 28.1% (efficiency roll-off of 51% at 1000 cd m^–2^) and 27.5% (efficiency roll-off of 44% at 1000 cd m^–2^) at λ_EL_ of 486 [CIE coordinates of (0.18, 0.37)] and 498 nm [CIE coordinates of (0.22, 0.49)], again respectively.

A rigid and planar hybrid boron-carbonyl group was used by Lee *et al*. as an acceptor in **TMCzBCO** and **DMACBCO** (Figure 52).^394^ **TMCzBCO** and **DMACBCO** are efficient green emitters, emitting at λ_PL_ of 526 and 520 nm and with Φ_PL_ of 94 and 93%, and have small Δ*E*_ST_ of 0.007 and 0.011 eV and fast *k*_RISC_ of 5.38 and 6.42 × 10^6^ s^–1^, all respectively (Table S2). Devices with these two emitters showed EQE_max_ of 24.7 and 28.4% at λ_EL_ of 532 [CIE coordinates of (0.33, 0.59)] and 556 nm [CIE coordinates of (0.43, 0.54)], respectively. Remarkably, the efficiency remains as high as 20.3 and 21.5% respectively at 5000 cd m^–2^.

To explore the influence of different bulky groups on the horizontal dipole alignment in films, Wu *et al*. reported a new emitter **iCzDBA** ^395^ and compared it with the previously reported emitters **CzDBA** and **tBuCzDBA** ^392^ (Figure 52). The 10 wt% **iCzDBA** doped film in CBP showed a small Δ*E*_ST_ of 0.03 eV. Due to increased steric hindrance of the *tert*-butyl or isopropyl groups, aggregation induced quenching was alleviated in the films of **tBuCzDBA** and **iCzDBA**, evidenced by the high Φ_PL_ of 84 and 88% and short delayed lifetimes of 1.2 and 1.4 μs, all respectively (Table S2). The neat film of **tBuCzDBA** exhibits a higher Θ// of 92% compared to **iCzDBA** (Θ// 77%), ascribed to the bulky groups on the terminal ends of **tBuCzDBA** extending the long axis of the compound. The OLEDs with **tBuCzDBA** and **iCzDBA** showed better performance with EQE_max_ of 26.9% [CIE coordinates of (0.44, 0.55), λ_EL_ 558 nm] and 18.7% [CIE coordinates of (0.38, 0.59), λ_EL_ 540 nm], respectively, while the device with unsubstituted **CzDBA** showed an EQE_max_ of 13.5% [CIE coordinates of (0.44, 0.55, λ_EL_ 557 nm]. In addition to this, efficiency remain high with roll-off of 1.1 and 0.5% at 1000 cd m^–2^ for the devices with **tBuCzDBA** and **iCzDBA**, respectively.

All boron-containing acceptors summarized so far have featured 3-coordinate boron centers. A smaller additional class of boron-containing materials also feature 4-coordinate boron. Separating donors and acceptors with a 4-coordinate boron bridge isolates the HOMO and LUMO from one another, resulting in small Δ*E*_ST_ and allowing TADF to occur. Shiu *et al*. reported the two such compounds **fppyBTPA** and **dfppyBTPA** (Figure 52), which emit, respectively, at λ_PL_ of 494 and 508 nm, and have Φ_PL_, Δ*E*_ST_, and τ_d_ of 72%, ≈ 0 eV, and 2.0 μs (for **fppyBTPA** in 8 wt% doped film in mCPCN) and 100%, ≈ 0 eV and 2.4 μs (for **dfppyBTPA** in 25 wt% doped film in mCPCN) (Table S2).^396^ The devices with **fppyBTPA** and **dfppyBTPA** showed EQE_max_ of 20.2 and 26.6% at CIE coordinates of (0.27, 0.54) and (0.26, 0.58), however the OLED with **fppyBTPA** suffered from large efficiency roll-off of 23% at 100 cd m^–2^, while the device with **dfppyBTPA** showed a much smaller efficiency roll-off of just 5% at 100 cd m^–2^.

### 4.4 Sulfone-Containing Acceptors

Sulfones have been thoroughly explored as acceptors in D-A TADF emitter design and are almost as popular and established as cyano-based acceptors in the context of blue and green emitters (Figure 53). In a recent example, a diphenylsulfone acceptor coupled to two acridine-based donor dendrons containing peripheral diphenylamines gave the emitter **DDA-DP**.^397^ This compound emits at 549 nm as a neat film and has a Φ_PL_ of 12.4% in toluene (Table S2). The Φ_PL_ increases to 45% in 15 wt% doped films, in mCP and with a Δ*E*_ST_ of 0.04 eV. The solution-processed OLED showed an EQE_max_ of 8.1% at λ_EL_ of 550 nm [CIE coordinates of (0.36, 0.56)]. Notably, the efficiency roll-off at 1000 cd m^–2^ was only 1%, which was attributed to the very fast τ_d_ of 0.45 μs, limiting the accumulation of triplet excitons and associated triplet quenching processes in the device.

Wang *et al*. employed a related thianthrene tetraoxide acceptor in combination with carbazole donors in the emitters **DCz-TTR** and **Cz-TTR** (Figure 53).^398^ With only one carbazole, **Cz-TTR** emits at 487 nm with a higher Φ_PL_ of 56.5%, although also a larger Δ*E*_ST_ of 0.10 eV in 6.5 wt% doped films (Table S2). in mCP. The dicarbazole congener **DCz-TTR** in contrast has λ_PL_ of 502 nm, Φ_PL_ of 47.1%, and Δ*E*_ST_ of 0.03 eV. OLEDs with **DCz-TTR** showed an EQE_max_ of 20.1% at λ_EL_ of 512 nm (efficiency roll-off of 40% at 1000 cd m^–2^), while the device with **Cz-TTR** performed worse, showing an EQE_max_ of 14.4% (λ_EL_ of 492 nm), which decreased by ca. 83% at 1000 cd m^–2^). Using the same thianthrene tetraoxide acceptor three additional D-A emitters were reported using acridine donor derivatives: **DMAC-TTR**, **DMAC-PTR**, and **SADF-TTR**.^399^ Similar to the aforementioned carbazole analogues, these compounds emit at 555, 572, and 530 nm, have small Δ*E*_ST_ (0.01, 0.02, and 0.03 eV), fast τ_d_ (5.2, 3.4, and 5.2 μs) and moderate Φ_PL_ (43, 59, and 52%) all in 10 wt% doped films in mCP. The OLEDs with **DMAC-TTR**, **DMAC-PTR**, and **SADF-TTR** showed EQE_max_ values of 13.9, 18.2, and 20.2% at CIE coordinates of (0.33, 0.50), (0.40, 0.56), and (0.35, 0.57), respectively. The device efficiency roll-off was low, reflective in the EQE_100_ of ∼12, ∼14, and ∼17%, respectively. The thianthrene tetraoxide acceptor was later also combined with a acridine-decorated carbazole donor dendron in the D′-D-A compound **DMAC-CZ-TTR**.^400^ The acridines act as secondary electron donating group (D′) to fortify the donating strength of the primary carbazole donor. The doped film of 10 wt% **DMAC-CZ-TTR** in CBP emits at 550 nm and has a Φ_PL_ of 69.5%, a small Δ*E*_ST_ of 0.066 eV, a τ_d_ of 14.7 ms, and *k*_RISC_ of 7.67×10^5^ s^–1^. The solution-processed and vacuum-deposited devices showed almost the same EQE_max_ of 20.6% [λ_EL_ 568 nm, CIE coordinates of (0.45, 0.51)] and 21.2% [λ_EL_ 550 nm, CIE coordinates of (0.40, 0.54)], respectively.

A related acceptor was generated by replacing one of the SO_2_ groups in the thianthrene with a ketone.^401^ Coupling this acceptor to N-phenyl carbazole donors resulted in highly efficient emitters in 5 wt% doped films in CBP: **2,3-TXO-PhCz** (λ_PL_ = 540 nm; Φ_PL_ = 62%), **2,6-TXO-PhCz** (λ_PL_ = 526 nm; Φ_PL_ = 84%), **2,7-TXO-PhCz** (λ_PL_ = 530 nm; Φ_PL_ = 89%), and **3,6-TXO-PhCz** (λ_PL_ = 544 nm; Φ_PL_ = 85%) (Figure 53). The OLEDs prepared with **2,6-TXO-PhCz**, **2,7-TXO-PhCz**, and **3,6-TXO-PhCz** showed EQE_max_ of 23.2, 24.4, and 18.1% respectively, although the τ_d_ of 77, 63, and 74 μs proved detrimental to device performance with efficiency roll-off at 100 cd m^–2^ of ∼59, ∼51, and ∼45% (Table S2). Considerably worse device performance was exhibited for the OLED with **2,3-TXO-PhCz** (EQE_max_ of 11.9%), which was qualitatively in trend with the lower Φ_PL_ of 62.1% and larger Δ*E*_ST_ of 0.24 eV of this emitter.

Employing a triazatruxene donor coupled to three dibenzothiophene-5,5-dioxide acceptors, dos Santos *et al*. demonstrated that the D-A_3_ compound **TAT-3DBTO_2_** (Figure 53) showed very efficient TADF owing to the large density of triplet states resulting from the multiple conformers present.^92^ This was reflected in the multiple fitted τ_d_ components, which were ascribed by the authors to the different conformers. An average τ_d_ of 11.7 μs and very fast *k*_RISC_ of 1.5×10^7^ s^–1^ was reported for the fastest delayed emission component (τ_1_ = 103.9 ns, supported by Δ*E*_ST_ of 0.03 eV).^83^ The green OLEDs showed very high EQE_max_ of 30.9% at CIE coordinates of (0.26, 0.46). The efficient TADF was also reflected in the efficiency roll-off, where the EQE_100_ was maintained at 29%, although the EQE_1000_ dropped to 16.5%.

**Figure 53:**
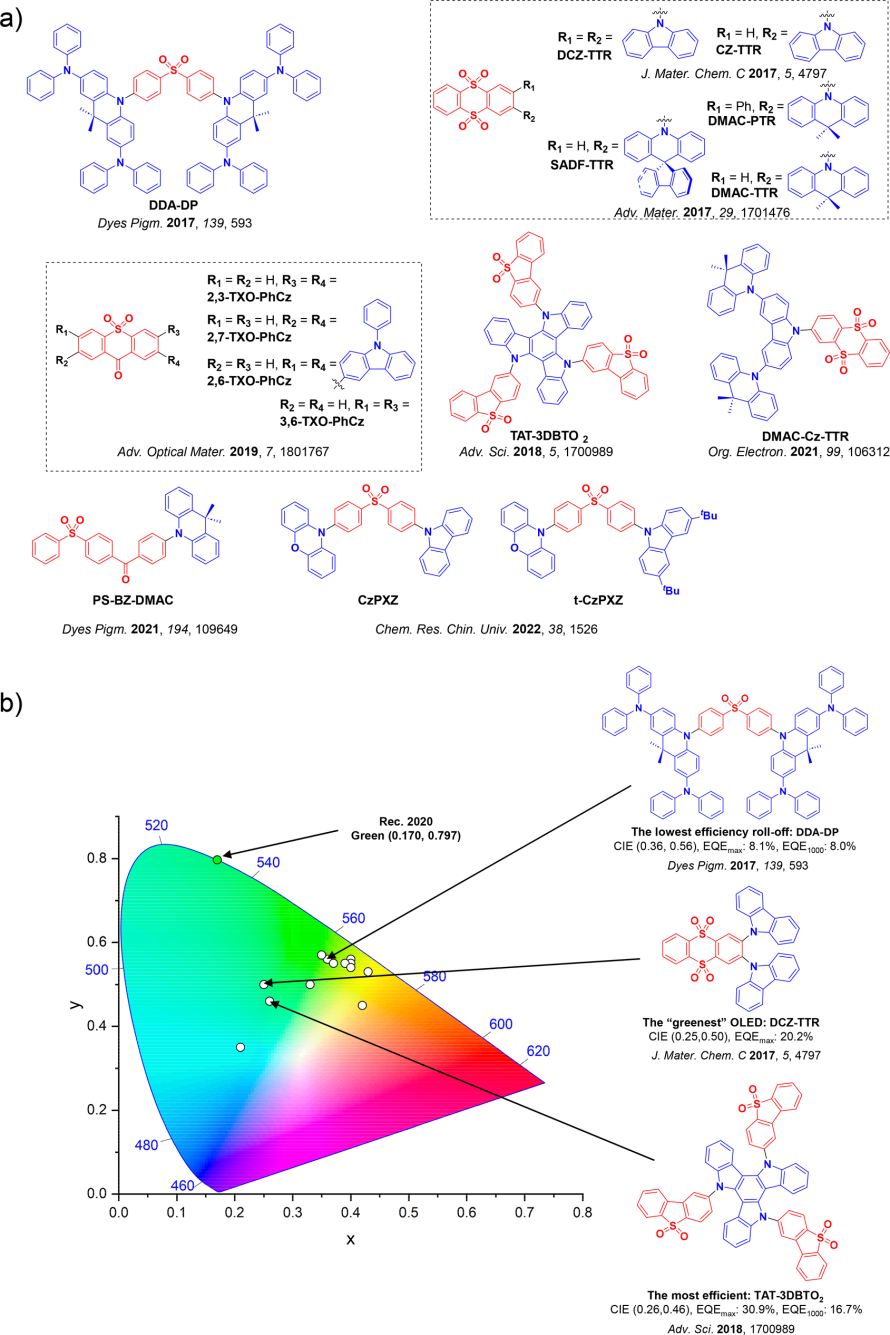
a) Molecular structures of green D-A TADF emitters containing sulfone acceptors and b) CIE color coordinates of green D-A TADF emitters containing sulfone acceptors. The white circles illustrate the spread of the emission color of the device. Selected devices and their associated CIE coordinates are highlighted, illustrating the structure of the emitter of the “greenest” device and the structure of the emitter used in the device showing the highest efficiency and the lowest efficiency roll-off. Only D-A TADF OLEDs where the λ_E_ = 490–580 nm that show EQE_max_ > 20% or have minimal efficiency roll-off are included. The most efficient device is quantified by the highest EQE_max_. The device with the CIE coordinates closest to the Rec. 2020 defined coordinates for green, (0.170, 0.797), is defined as the “greenest”. In the chemical structures, the blue color signifies donor moieties, while the red color signifies acceptor moieties.

Exploring asymmetric D-A-D emitters, **PS-BZ-DMAC** (Figure 53) incorporates an unusual D-A-A’ structure with a sulfone terminal acceptor bridged to the acridine donor by a benzophenone group.^402^ This compound emits at 574 nm, has a Φ_PL_ of 76%, and a τ_d_ of 2.83 μs in 5 wt% doped films in CBP. The OLEDs with **PS-BZ-DMAC** emitted at λ_EL_ of 537 nm [CIE coordinates of (0.37, 0.55)] and showed an EQE_max_ of 20.6%. Gao *et al*. reported asymmetric TADF materials **CzPXZ** and **t-CzPXZ** that also showed AIE (Figure 53).^403^ The neat films of **CzPXZ** and **t-CzPXZ** emit at 533 and 528 nm, have τ_d_ of 3.6 and 1.4 μs, small Δ*E*_ST_ of 0.03 and 0.04 eV, and Φ_PL_ of 79 and 77% in respective neat films. Non-doped OLEDs showed EQE_max_ of 21.8% (λ_EL_ of 520 nm) and 17.4% (λ_EL_ of 514 nm), respectively.

### 4.5 Triazine-Containing Acceptors

Due to its moderate electron-withdrawing ability, triazine has become a widely used acceptor in TADF emitter design. A prototypical green-emissive compound **DMAC-TRZ** (Figure 54)^231^ (λ_PL_ of 495 nm, Φ_PL_ of 90%, Δ*E*_ST_ of 0.046 eV, and τ_d_ of 1.9 μs in 8 wt% doped film in mCPCN) has since inspired many derivative molecular designs, much like how **4CzIPN** has been the starting point for derivatization of benzonitrile-based TADF materials. For example, Gan *et al*. used a spiro-acridine-based donor coupled to triazine in the two derivatives **TRZ-*p*-ACRSA** and **TRZ-*m*-ACRSA** (Figure 54).^404^ Both intramolecular and through-space CT interactions were proposed to occur between the donor and acceptor, which also allowed various intermediate ^3^LE states to be close in energy to ^1^CT and mediate efficient RISC, reflected in the τ_d_ of 4.7 and 5.7 μs, respectively. The two compounds emit at around λ_PL_ of 500 nm and have Φ_PL_ of 97 and 70% in 20 wt% doped films in DPEPO. The devices with **TRZ-p-ACRSA** and **TRZ-*m*-ACRSA** showed EQE_max_ of 28.0% [CIE coordinates of (0.19, 0.42)] and 17.7% [CIE coordinates of (0.22, 0.45)], respectively. At 100 cd m^–2^ the efficiency roll-off was as low as 1% for the device with **TRZ-p-ACRSA** and 3% for the device with **TRZ-*m*-ACRSA**, while at 1000 cd m^–2^, the efficiency roll-off was 21 and 24%, respectively.

**Figure 54:**
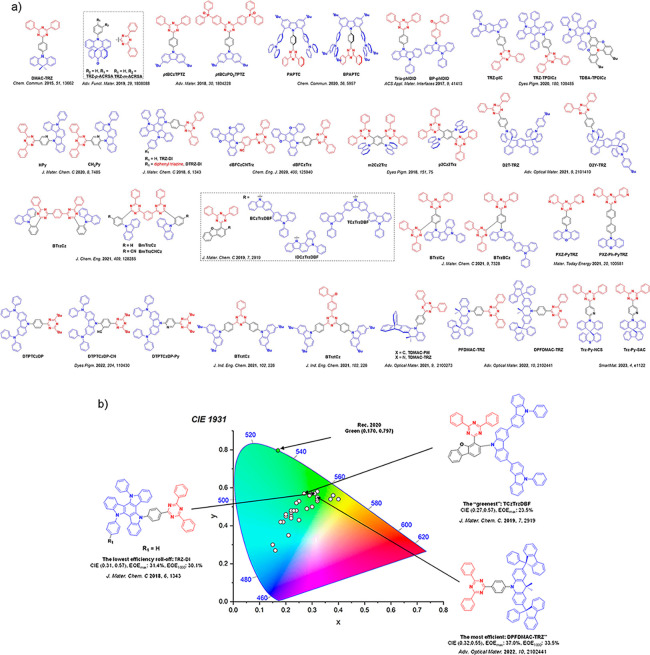
a) Molecular structures of green D-A TADF emitters containing triazine acceptors and b) CIE color coordinates of green D-A TADF emitters containing triazine acceptors. The white circles illustrate the spread of the emission color of the device. Selected devices and their associated CIE coordinates are highlighted, illustrating the structure of the emitter of the “greenest” device and the structure of the emitter used in the device showing the highest efficiency and the lowest efficiency roll-off. Only D-A TADF OLEDs where the λ_EL_ = 490–580 nm that show EQE_max_ > 20% or have minimal efficiency roll-off are included. The most efficient device is quantified by the highest EQE_max_. The device with the CIE coordinates closest to the Rec. 2020 defined coordinates for green, (0.170, 0.797), is defined as the “greenest”. In the chemical structures, the blue color signifies donor moieties, while the red color signifies acceptor moieties.

Coupling phosphine oxide auxiliary acceptors to an existing D-A structure produced a high performing green/blue emitter, ***p*tBCzPO_2_TPTZ** (Figure 54). Compared to the control material ***p*tBCzTPTZ** (λ_PL_ of 446 nm, Φ_PL_ of 25%, Δ*E*_ST_ of 0.24 eV), this compound has a very high Φ_PL_ of 96%, small Δ*E*_ST_ of 0.01 eV, and good charge balance in 10 wt% doped film in DPEPO, supporting exciton utilization efficiency of 96%. The OLED performance was thus vastly improved, with an EQE_max_ of 28.9% (λ_EL_ 492 nm), increased from just 4.4% for the device with ***p*tBCzTPTZ**.^405^ The efficiency roll-off of the device with ***p*tBCzPO_2_TPTZ** at 100 cd m^–2^ was only 10%, however the performance degraded considerably beyond this point with an efficiency roll-off of 43% at 1000 cd m^–2^.

Constraining the conformational landscape and balancing of through-space and through-bond CT interactions were used by Li *et al*. within the design of **PAPTC** and **BPAPTC** (Figure 54).^406^ The reference emitter **TC** consists only of triazine and ^*t*^Bu-carbazole that are directly coupled to have a TBCT state. **PAPTC** and **BPAPTC** instead feature suppressed rotation of the triazine acceptor about the 9-position of the ^*t*^Bu-carbazole donor resulting from the donor-acceptor-donor “sandwich” structure, which also enabled through-space charge transfer interactions (see Section 12). **PAPTC** and **BPAPTC** have much smaller Δ*E*_ST_ in toluene of 0.07 and 0.06 eV, respectively, compared to 0.28 eV for **TC**. These two compounds emit at λ_PL_ of 509 and 519 nm and have Φ_PL_ of 78 and 90%. The solution-processed devices with **PAPTC** and **BPAPTC** both emitted at λ_EL_ of 520 nm, showed EQE_max_ of 17.4 and 24.3%, and efficiency roll-off of 17 and 7% at 1000 cd m^–2^, all respectively.

Ryoo *et al*. introduced a novel fused bicarbazole donor that was coupled with either benzophenone in **BP-phIDID**, or triazine in **Tria-phIDID** (Figure 54).^407^ The two compounds in toluene emit at λ_PL_ of 520 and 526 nm and have Δ*E*_ST_ of 0.20 and 0.12 eV. In 6 wt% doped films in PMMA the τ_d_ are 22.8 and 18.4 μs, and in 8 wt% doped films in CBP the Φ_PL_ are 56.9 and 69.8%, all respectively. Other acceptors were also screened using this emitter design, (benzonitrile, benzosulfone, and nitrobenzene) though none of the resulting emitters showed TADF. Devices with **BP-phIDID** and **Tria-phIDID** showed EQE_max_ of 13.9 and 20.8% at λ_EL_ of 497 nm [CIE coordinates of (0.25, 0.43)] and 504 nm [CIE coordinates of (0.28, 0.49)], but suffered from severe efficiency roll-off of 56 and 49% at 100 cd m^–2^, all respectively.

Maeng *et al.* investigated the impact of phenyl substitution on indolocarbazole donors in emitters **TRZ-TPDICz** and **TDBA-TPDICz** (Figure 54).^408^ These two emitters were compared with the reference emitter **TRZ-pIC**, with unsubstituted indolocarbazole. **TRZ-TPDICz** has nearly the same λ_PL_ and Δ*E*_ST_ in toluene (λ_PL_ of 479 nm, Δ*E*_ST_ of 0.26 eV) as **TRZ-pIC** (λ_PL_ of 478 nm, Δ*E*_ST_ of 0.29 eV), and possesses the same Φ_PL_ of 86% in 20 wt% doped films in DBFPO (Table S2). **TDBA-TPDICz** (λ_PL_ of 447 nm, Δ*E*_ST_ of 0.41 eV in toluene) has a higher Φ_PL_ of 96% in the same films than the reference emitter, but at the expense of a much larger Δ*E*_ST_. **TDBA-TPDICz** is bluer due to the weaker DOBNA-based TDBA acceptor. Phenyl-substituted TPDICz turns out to be a stronger donor than pIC evidenced by the shallower HOMO level (-5.37 eV for **TRZ-TPDICz** vs −5.66 eV for **TRZ-pIC**), while the steric impact of the modified donor helped to maintain an orthogonal conformation between the donor and acceptor segments. The device with **TRZ-TPDICz** showed a superior EQE_max_ of 30.3% at λ_EL_ of 509 nm and CIE coordinates of (0.25, 0.53). The device with the reference emitter **TRZ-pIC** showed an EQE_max_ of 26.8% [λ_EL_ of 507 nm, CIE coordinates of (0.27, 0.53)], while the device with **TDBA-TPDICz** showed comparatively poorer device performance of the series with EQE_max_ of 16.9% at λ_EL_ of 462 nm and CIE coordinates of (0.14, 0.14) due to its large Δ*E*_ST_.

Yoon *et al*. modified **TRZ-pIC** by replacing the phenylene bridge between the triazine unit and a different indolocarbazole with pyridine, or by additionally incorporating a methyl group on the pyridyl bridge in **HPy** and **CH3Py** (Figure 54).^409^ **HPy** and **CH3Py** both emit at around 500 nm, and the 10% doped films of these emitters in DPEPO have high Φ_PL_ of 94% (Δ*E*_ST_ of 0.22 eV, τ_d_ of 5.08 ms) and 88.7% (Δ*E*_ST_ of 0.10 eV, τ_d_ of 3.42 ms), respectively (Table S2). The devices with **HPy** and **CH3Py** showed EQE_max_ of 23.6% [CIE coordinates of (0.22, 0.44)], and 24.6% [CIE coordinates of (0.22, 0.44)], respectively. The device with **HPy** showed high efficiency roll-off of 63% at 1000 cd m^–2^, whereas the equivalent efficiency roll-off of the device with **CH3Py** was smaller at only 28%.

Kim *et al*. explored a similar design strategy to that of **TAT-3DBTO_2_**_,_ ^92^ using a triazatruxene donor but with triazines as the acceptor^410^ in D-A (**TRZ-DI**) and D-A_2_ (**DTRZ-DI**) structures (Figure 54). **TRZ-DI** and **DTRZ-DI** both emit at λ_PL_ = 521 nm, have high Φ_PL_ of 87 and 83%, small Δ*E*_ST_ of 0.02 and 0.03 eV, and τ_d_ of 1.32 and 1.47 μs, all respectively. This translated to excellent devices with EQE_max_ of 31.4 and 26.2% reported using **TRZ-DI** (λ_EL_ = 526 nm) and **DTRZ-DI** (λ_EL_ = 526 nm), while the efficiency roll-off at 10, 000 cd m^–2^ was found to be 19 and 26% respectively, representing two of the best performing green TADF devices to date. The minor difference in device performance was attributed to the differing Φ_PL_, although it is remarkable that different levels of acceptor functionalization occurred without any change in the emission spectrum itself. A furan modified triazatruxene donor was incorporated within emitters **dBFCzCNTrz** and **dBFCzTrz** (Figure 54).^245^ The insertion of a nitrile group *ortho* to the donor in **dBFCzCNTrz** (λ_PL_ of ca. 490 nm) produced a stronger acceptor leading to a red-shifted emission compared to **dBFCzTrz** (λ_PL_ of ca. 455 nm). **dBFCzCNTrz** and **dBFCzTrz** have high Φ_PL_ of 80.7 and 89.4%, Δ*E*_ST_ of 0.09 and 0.13 eV, and τ_d_ of 4.9 and 30.4 μs in 20 wt% doped films in DPEPO, all respectively (Table S2). The device with **dBFCzCNTrz** emitted at λ_EL_ of 497 nm with CIE coordinates of (0.22, 0.47), and showed an EQE_max_ of 27.5% which decreased to 24.3% at 1000 cd m^–2^. The device with **dBFCzTrz** emitted at λ_EL_ of 470 nm with CIE coordinates of (0.15, 0.18) and showed a lower EQE_max_ of 22.6% that also suffered more severe efficiency roll-off (EQE_1000_ of 12.3%).

A pair of isomeric emitters incorporating two triazine acceptors and two carbazoles donors, **m2Cz2TRZ** and **p2Cz2TRZ** (Figure 54), emit at 465 and 502 nm, have Δ*E*_ST_ of 0.09 and 0.18 eV (1 wt% doped films in PMMA) and have Φ_PL_ of 96 and 86% with τ_d_ of 12.2 and 16.6 μs (10 wt% doped films in DPEPO), all respectively (Table S2).^270^ Due to the higher Φ_PL_ of the emitter, the device with **m2Cz2TRZ** showed a higher EQE_max_ of 18.5% (λ_EL_ = 493 nm) compared to 12.5% (λ_EL_ = 534 nm) for the device with **p2Cz2TRZ**, and also had lower efficiency roll-off of 13% compared to 26% at 1000 cd m^–2^, all respectively.

Rather than a typical phenylene linker, **BCzTrzDBF**, **TCzTrzDBF**, and **IDCzTrzDBF** each instead contained a benzofuran unit (Figure 54).^411^ The computed ground- and excited-state energies suggested little change arising from the replacement of the phenylene with a benzofuran linker. **BCzTrzDBF**, **TCzTrzDBF**, and **IDCzTrzDBF** have Φ_PL_ of 82, 86, and 85% in 5 wt% doped films in mCBPTrz, respectively. Their small Δ*E*_ST_ (0.06, 0.01, and 0.05 eV) and short τ_d_ (5.4, 4.4, and 2.8 μs) ensured large k_RISC_ of 3.9, 6.0, and 8.1 × 10^5^ s^–1^, all respectively (Table S2). The OLEDs with **BCzTrzDBF**, **TCzTrzDBF**, and **IDCzTrzDBF** showed EQE_max_ of 20.1% [λ_EL_ = 503 nm, CIE coordinates of (0.24, 0.52)], 23.5% [λ_EL_ = 511 nm, CIE coordinates of (0.27, 0.57)] and 12.2% [λ_EL_ = 500 nm, CIE coordinates of (0.22, 0.48)]. The devices with **BCzTrzDBF**, **TCzTrzDBF**, and **IDCzTrzDBF** showed efficiency roll-offs of 33, 24, and 13%, respectively, at 3000 cd m^–2^, which were inversely proportional to the k_RISC_ of the emitters. The TDMs of **BCzTrzDBF** and **TCzTrzDBF** were also found to be preferentially horizontally aligned, resulting in enhanced light outcoupling in these devices.

The effect of doping concentration was studied by Liu *et al*. in emitters **D2T-TRZ** and **D2Y-TRZ**, possessing similar donor and acceptor subunits but contrasting molecular shapes (Figure 54).^412^ **D2T-TRZ** and **D2Y-TRZ** in 10 wt% doped films in DPEPO emit at 489 and 491 nm, have Φ_PL_ of 97 and 71%, and Δ*E*_ST_ of 0.10 and 0.41 eV respectively (Table S2). The small Δ*E*_ST_ in **D2T-TRZ** translated to excellent electroluminescence properties at doping levels below 70 wt%. The highest EQE_max_ of 27.1% [CIE coordinates of (0.20, 0.45)] was observed for the device with an EML comprising 20 wt% **D2T-TRZ** in DPEPO. An equivalent device with 30 wt% **D2Y-TRZ** in DPEPO showed an EQE_max_ of 16.4% [CIE coordinates of (0.22, 0.47)]. The lower EQE_max_ in the device with **D2Y-TRZ** was correlated to both the lower Φ_PL_ and slower k_RISC_ of that emitter. Similar to the device with **D2T-TRZ**, the EQE_max_ of the device with **D2Y-TRZ** was significantly negatively impacted when the doping concentration increased beyond 40 wt%.

Dual-emissive **BmTrzCz** and **BmTrzCNCz** (Figure 54) are based on the previously reported dual-emissive TADF material **BTrzCz**.^413,414^ The extended conjugation between the *para*-linked triazines in **BTrzCz** resulted in a larger Δ*E*_ST_ of 0.14 eV compared to **BmTrzCz** (Δ*E*_ST_ of 0.07 eV), where the two triazine units are *meta*-disposed to each other. A secondary cyano acceptor present in **BmTrzCNCz** further reduced the Δ*E*_ST_ to 0.05 eV (Table S2). **BmTrzCz** and **BmTrzCNCZ** have comparable Φ_PL_ of 85 and 84%, and *k*_RISC_ of 4.95 and 3.70 × 10^5^ s^–1^, respectively. The devices with **BmTrzCz** and **BmTrzCNCz** showed higher EQE_max_ of 20.3 and 21.9% compared to the OLED with the parent **BTrzCz** at only 13.5%. The two devices also showed low efficiency roll-off of 18.0 and 19.3% at 1000 cd m^–2^, respectively. Structurally related compounds **BTrzICz** and **BTrzBCz** also have two triazine acceptor units connected *meta* to each other through a central phenyl linker, with one donor carbazole that is connected *ortho* to one of the triazine acceptors and *para* to the other.^415^ Films of **BTrzICz** and **BTrzBCz** (5 wt% in CzTrz) emit at 490 and 500 nm, have Φ_PL_ of 97 and 92%, Δ*E*_ST_ of 0.00 and 0.04 eV, and τ_d_ of 3.7 and 7.2 μs, all respectively. In line with their τ_d_ the *k*_RISC_ of **BTrzICz** is 8.07×10^5^ s^–1^, 4 times faster than **BTrzBCz** (*k*_RISC_ of 2.12 × 10^5^ s^–1^). The devices with **BTrzICz** and **BTrzBCz** showed EQE_max_ of 20.7 and 20.5% at CIE coordinates of (0.29, 0.56) and (0.30, 0.57), with low efficiency roll-off corresponding to EQE_3000_ of 20.1 and 18.7%, all respectively.

Zhang *et al*. replaced two of the peripheral phenyl groups of triazine with electron-withdrawing pyridine in **PXZ-PyTRZ**, and also obtained **PXZ-Ph-PyTRZ** by inserting an additional phenylene spacer between the donor and acceptor moieties (Figure 54).^416^ **PXZ-PyTRZ** and **PXZ-Ph-PyTRZ** emit at λ_PL_ of 572 and 538 nm, have Δ*E*_ST_ of 0.01 and 0.09 eV, and Φ_PL_ of 65 and 76% in 10 wt% doped films in CBP (Table S2). The EQE_max_ increased from 18.5% at λ_EL_ 560 nm and CIE coordinates of (0.44, 0.54) in the device with **PXZ-PyTRZ**, to 22.2% at λ_EL_ 540 nm, CIE coordinates of (0.38, 0.56) in the device with **PXZ-Ph-PyTRZ**. The efficiency roll-off was milder in the device with **PXZ-PyTRZ** (6.5% at 1000 cd m^–2^) than the device with **PXZ-Ph-PyTRZ** (18.9% at 1000 cd m^–2^).

**BTrztCz** (Figure 54) contains a benzoylphenyltriazine acceptor and carbazole donors. It has a Φ_PL_ of 70% and a *k*_RISC_ of 8.70×10^4^ s^–1^ in 10 wt% doped films in DPEPO (Table S2).^417^ The additional benzoyl unit of **BTrztCz** compared to **TrztCz** (Figure 54) strengthened the acceptor and produced longer wavelength emission (red-shifted from 466 to 496 nm) in 10 wt% doped films in DPEPO. The OLEDs with **BTrztCz** showed an EQE_max_ of 21.4% at λ_EL_ of 496 nm.

**TDMAC-TRZ** and **TDMAC-PM** (Figure 54) were developed by Zhan *et al*. using a triptycene-fused acridine donor with triazine or pyrimidine acceptors.^418^ **TDMAC-TRZ** and **TDMAC-PM** emit at λ_PL_ of 525 and 505 nm, have Δ*E*_ST_ of 0.045 and 0.048 eV, Φ_PL_ of 82 and 77%, and τ_d_ of 1.7 and 5.0 μs in 10 wt% doped films in DPEPO (Table S2). OLEDs with **TDMAC-TRZ** and **TDMAC-PM** both displayed EQE_max_ of 24.2% but with diverging respective efficiency roll-off of 45.5 and 78.5% at 1000 cd m^–2^. These devices emitted at λ_EL_ of 525 and 505 nm and CIE coordinates of (0.32, 0.54) and (0.32, 0.53), respectively. The performance of non-doped devices remained high with EQE_max_ of 23% at λ_EL_ 529 nm [CIE coordinates of (0.35, 0.56)] and 18% at λ_EL_ 503 nm [CIE coordinates of (0.34, 0.54)], respectively. Both non-doped devices showed severe efficiency roll-off though, of 48.2 and 48.9%, respectively at 1000 cd m^–2^.

Shi *et al*. reported three 2,4-di-*tert*-butyl-1,3,5-triazine based emitters, **DTPTCzDP**, **DTPTCzDP-CN**, and **DTPTCzDP-Py** (Figure 54) where substitution of phenyl groups in the typical triphenyltriazine for *tert*-butyl groups effectively weakened the acceptor.^419^ The π-bridge between the donors and acceptors was also varied from phenylene to pyridyl or benzonitrile to modulate the conformation of the emitter and to further tune the acceptor strength. **DTPTCzDP** and **DTPTCzDP-Py** emit at λ_PL_ of 508 and 532 nm and have similar Φ_PL_ of 68 and 70%, Δ*E*_ST_ of 0.14 and 0.08 eV, and τ_d_ of 2.78 and 1.76 μs in 7 wt% doped films in DPEPO, all respectively (Table S2). **DTPTCzDP-CN** instead emits at 545 nm in 7 wt% doped CBP films with a Φ_PL_ of 62%, a somewhat smaller Δ*E*_ST_ of 0.03 eV, and shorter τ_d_ of 1.47 μs. The OLEDs with each of the three emitters at 10 wt% in CBP showed greater than 16% EQE_max_: the device with **DTPTCzDP** emitted at λ_EL_ of 510 nm with CIE coordinates of (0.30, 0.50), and had the leading EQE_max_ of 20.1%. The devices with **DTPTCzDP-CN** and **DTPTCzDP-Py** emitted at λ_EL_ of 548 and 538 nm with CIE coordinates of (0.40, 0.54) and (0.37, 0.54), and showed EQE_max_ of 17.8 and 16.9%, all respectively.

**PFDMAC-TRZ** and **DPFDMAC-TRZ** (Figure 54), inspired by **DMAC-TRZ** but featuring spirofluorene-substituted acridine donors, were reported by Feng *et al*.^420^ **PFDMAC-TRZ** and **DPFDMAC-TRZ** showed high Φ_PL_ of 93 and 97%, the same Δ*E*_ST_ of 0.16 eV, and short τ_d_ of 1.6 and 1.3 μs in 30 wt% doped films in DPEPO, all respectively (Table S2). These emitters also showed comparably horizontal oriented TDMs of Θ// from 78 and 81%. The device with **DPFDMAC-TRZ** showed a very high EQE_max_ of 37.0% [CIE coordinates of (0.32, 0.55)] and excellent efficiency roll-off (EQE_1000_ of 33.5%) at λ_EL_ of 524 nm. The device with **PFDMAC-TRZ** showed a similar EQE_max_ of 35.1% [CIE coordinates of (0.32, 0.55)], but with comparatively poorer efficiency roll-off (EQE_1000_ of 24.9%) at λ_EL_ of 521 nm.

In a bid to enhance SOC, Fan *et al*. reported the compounds **Trz-Py-NCS** and **Trz-Py-SAC** (Figure 54), which contain a heavy sulfur atom within the spiro-linked acridine donor in the former and pyridine bridges in both.^421^ However, the sulfur atom in **Trz-Py-SAC** (λ_PL_ of ca. 510 nm) imparted no significant changes in the photophysical properties compared to **Trz-Py-NCS** (λ_PL_ of ca. 505 nm). Remarkably, the Φ_PL_ is 100% in the neat films of both emitters, with additional small Δ*E*_ST_ values of 0.059 and 0.058 eV giving short τ_d_ of 1.2 and 1.3 μs and high *k*_RISC_ of 1.8 and 1.6 × 10^6^ s^–1^, all respectively (Table S2). The non-doped OLEDs with **Trz-Py-NCS** and **Trz-Py-SAC** showed EQE_max_ of 30.8 and 30.3% and impressive EQE_1000_ of 29.1 and 28.1%, also respectively (λ_EL_ = 520 and 524 nm).

### 4.6 Pyrimidine-Based Acceptors

Beyond triazine, other N-heterocycles have also been used as acceptors or linking groups in green-emissive TADF materials.^122^ Exploiting the heavy-atom effect, Xiang *et al*. modified the structure of **PXZPM** with halogens to improve the Φ_PL_ and shorten the τ_d_.^422^ **ClPPM** and **BrPPM** (Figure 55) have higher Φ_PL_ of 93 and 91% compared to 88% for **PXZPM**, shorter τ_d_ of 1.4 and 1.3 μs (2.6 ms for **PXZPM**), and smaller Δ*E*_ST_ of 0.06 and 0.07 eV (0.08 eV for **PXZPM**) in 1.5 wt% doped films in CBP, all respectively (Table S2). *k*_RISC_ thus improved from 2.71×10^5^ to ∼10^6^ s^–1^ for both of the halogenated analogues. The more efficient TADF in these two compounds translated into higher performing devices, with EQE_max_ of 25.3 and 23.6% (19.9% for the device with **PXZPM**). Moreover, the EQE_1000_ of the devices with **ClPPM** and **BrPPM** remain as high as 22.2 and 19.8%, respectively as compared to 14.2% for the device with **PXZPM**. To achieve improved horizontal orientation of the emitter TDM, the same group also elongated the acceptor of **PXZPM** in **PXZPyPM** and **PXZTAZPM** in 6.0 wt% doped films in mCPCN.^423^ These emitters also have 100% Φ_PL_ for **PXZPM** and **PXZPyPM**, and 93% for **PXZTAZPM**. The Δ*E*_ST_ of all the materials are also very small at 0.04, 0.07 and 0.05 eV respectively. These excellent optical properties then translated into devices which showed respective EQE_max_ of 29.5, 33.9, and 30.1%, all at λ_EL_ of 528 nm.

**Figure 55:**
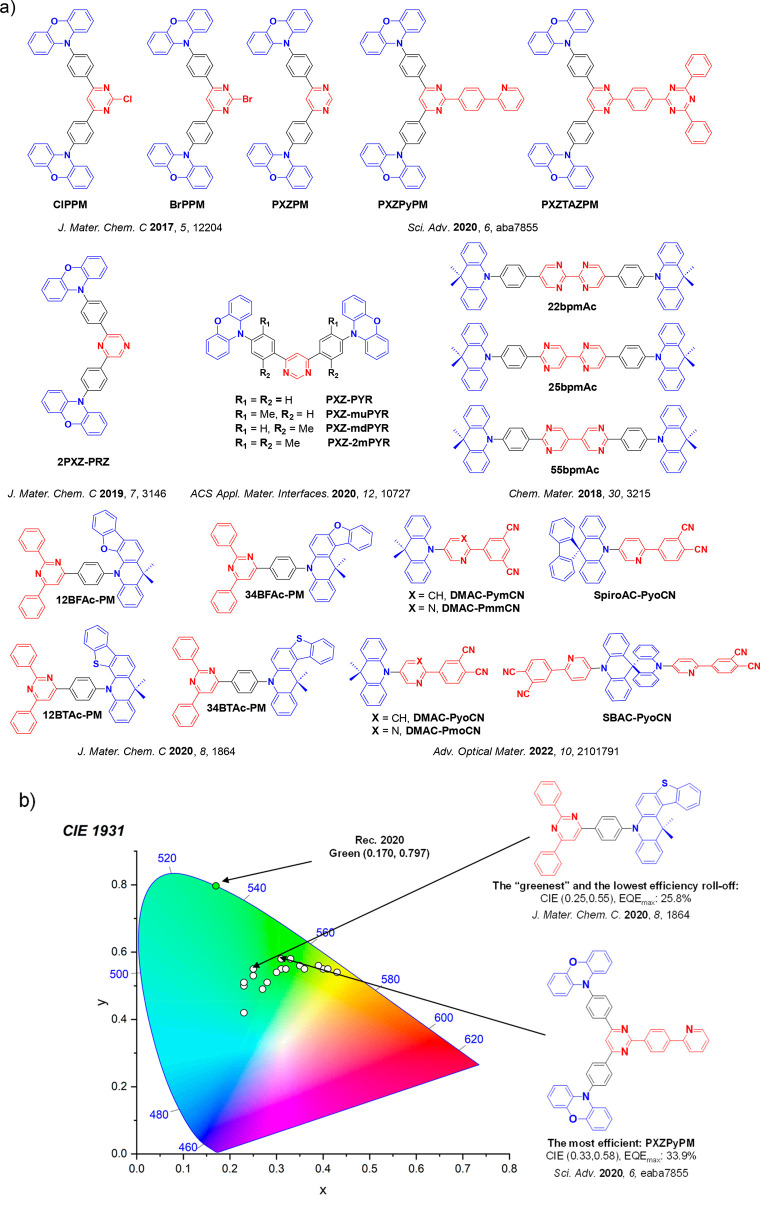
a) Molecular structures of green D-A TADF emitters containing diazine acceptors and b) CIE color coordinates of green D-A TADF emitters containing pyrimidine or pyridine acceptors. The white circles illustrate the spread of the emission color of the device. Selected devices and their associated CIE coordinates are highlighted, illustrating the structure of the emitter of the “greenest” device, the structure of the emitter used in the device showing the highest efficiency and the structure of the emitter with the lowest efficiency roll-off. Only D-A TADF OLEDs where the λ_EL_ = 490–580 nm which show EQE_max_ > 20% or have minimal roll-off are included. The most efficient device is quantified by the highest EQE_max_. The device with the CIE coordinates closest to the Rec. 2020 defined coordinates for green, (0.170, 0.797), is defined as the “greenest”. In the chemical structures, the blue color signifies donor moieties, while the red color signifies acceptor moieties.

Kato *et al*. introduced the use of a pyrazine acceptor in an otherwise identical structure to **PZXPM**, producing the emitter **2PXZ-PRZ** (Figure 55).^424^ Despite the promising Φ_PL_ of 65%, a relatively long τ_d_ of 54 μs and quite a large Δ*E*_ST_ of 0.21 eV suggested the devices would suffer from significant efficiency roll-off (Table S2). The devices showed an EQE_max_ of 21.4% at CIE coordinates of (0.31, 0.55), but indeed the device efficiency at 100 and 1000 cd m^–2^ dropped by 19 and 59%. These differences in device performance compared to the previous examples highlight how a modest structural change, in this case pyrimidine to pyrazine as the acceptor, can lead to significant differences in the optoelectronic properties and device performance.

Phenoxazine was used alongside a central pyrimidine acceptor by Serevičius *et al.* in the **PYR** series of emitters.^425^ The reference material **PXZ-PYR** (identical to the aforementioned **PXZPM**) emits at λ_PL_ of 543 nm, has a Φ_PL_ of 42%, and a τ_d_ of 1.6 μs in toluene (Table S2). The device showed an EQE_max_ of 27.9% at λ_EL_ of 536 nm and CIE coordinates of (0.35, 0.56). This reference structure was then modified through the addition of methyl groups at different positions relative to the donors to give **PXZ-muPYR**, **PXZ-mdPYR**, and **PXZ-2dPYR** (Figure 55), impacting both electronic and conformational properties. The λ_PL_ of **PXZ-muPYR**, **PXZ-mdPYR**, and **PXZ-2mPYR** are all blue-shifted relative to **PXZ-PYR** (λ_PL_ of 530, 528, and 519 nm), have Φ_PL_ of 52, 38, and 53%, τ_d_ of 4.2, 1.7, and 4.3 μs in toluene, and Δ*E*_ST_ of 0.07, 0.15, and 0.13 eV in 1 wt% doped films in PMMA, all respectively. The corresponding devices showed EQE_max_ of 29.1, 27.5, and 26.3% at λ_EL_ of 529, 514, 502 nm with CIE coordinates of (0.32, 0.55), (0.27, 0.49), and (0.23, 0.42), all respectively.

Benzofuran and benzothiophene were fused to acridine donors in different geometries to generate four emitters, each with a pyrimidine acceptor: **12BFAc-PM**, **12BTAc-PM**, **34BFAc-PM**, and **34BTAc-PM** (Figure 55).^426^ The 30 wt% doped films in DPEPO of **12BFAc-PM** emits at λ_PL_ of 475 nm while **12BTAc-PM**, **34BFAc-PM**, and **34BTAc-PM** emit in the green region at λ_PL_ of 509, 519, and 521 nm. The device with **12BFAc-PM** showed a relatively low EQE_max_ of 12.9% at 482 nm [CIE coordinates of (0.16, 0, 0.29)], due to its large Δ*E*_ST_ of 0.37 eV and moderate Φ_PL_ of 69%. The related structure **12BTAc-PM** has a much smaller Δ*E*_ST_ of 0.17 eV with high Φ_PL_ of 87%, and the device with this emitter consequently performed better, emitting at λ_EL_ of 503 nm and having an EQE_max_ of 25.6% [CIE coordinates of (0.23, 0, 0.50)]. Compounds **34BFAc-PM** and **34BTAc-PM** have much smaller Δ*E*_ST_ of 0.08 eV and much higher Φ_PL_ of 95 and 92%, leading to devices with EQE_max_ of 27.7 and 25.8% at λ_EL_ of 503 and 509 nm [CIE coordinates of (0.25, 0.55) and (0.23, 0.51)], all respectively. The efficiency roll-off of the devices with **12BTAc-PM**, **34BFAc-PM**, and **34BTAc-PM** were also relatively small, with EQE_1000_ of 22.0, 24.6, and 25.3%, respectively.

The role of intramolecular hydrogen bonding was investigated by Park *et al*. in a series of emitters containing bi(pyrimidine) acceptors.^427^ Two compounds, **25bpmAc** and **55bpmAC** (Figure 55), adopt a planarized acceptor conformation, while the hydrogen bonding is absent in **22bpmAc** which adopts a more twisted conformation. The differing conjugation resulting from this change in conformation is manifested in Φ_PL_ of 75, 98, and 99% for **22bpmAc** (λ_PL_ of 471 nm), **25bpmAc** (λ_PL_ of 472 nm), and **55bpmAc** (λ_PL_ of 466 nm), all respectively in 1 wt% doped films in PS. The Δ*E*_ST_ of these emitters range narrowly between 0.24 to 0.29 eV and their τ_d_ range from 17.1 to 37.5 μs (Table S2). **25bpmAc** and **55bpmAc** also have narrower emission spectra, with FWHM of 87 nm (**25bpmAc**) and 82 nm (**55bpmAc**), compared to 96 nm for **22bpmAc**. The corresponding devices showed EQE_max_ of 20.5, 24.9, and 15.7% for **25bpmAc** (λ_EL_ = 524 nm), **55bpmAc** (λ_EL_ = 512 nm), and **22bpmAc** (λ_EL_ = 517 nm), all respectively. Significant efficiency roll-off was observed at 100 cd m^–2^ and 1000 cd m^–2^ though, ranging between 24–49% and 63–83%.

**DMAC-PymCN**, **DMAC-PmmCN**, **DMAC-PyoCN** and **DMAC-PmoCN** (Figure 55) feature combinations of pyridine/pyrimidine and phthalonitrile acceptors.^428^ All four emitters bear highly twisted geometries, giving rise to small Δ*E*_ST_ values of 0.20, 0.14, 0.13, and 0.11 eV. Among the four emitters **DMAC-PyoCN** has the highest Φ_PL_ of 91% (8 wt% doped in mCPCN) and slowest *k*_nr_ of 2.8 × 10^6^ s^–1^, despite having a moderate Δ*E*_ST_ of 0.13 eV (Table S2). The device with **DMAC-PyoCN** showed a EQE_max_ of 25.9% [CIE coordinates of (0.41, 0.55)], with the devices of the other three emitters having EQE_max_ no higher than 22.3%. PyoCN was therefore demonstrated to be the best choice of acceptor amongst those studied, and to optimize the emitter design the authors also employed spiro-acridine and spiro-bisacridine donors in **SpiroAC-PyoCN** (λ_PL_ of 518 nm) and **SBAC-PyoCN** (λ_PL_ of 525 nm), both of which have Φ_PL_ of 100% in 8 wt% doped films in mCPCN. The devices with **SpiroAC-PyoCN** and **SBAC-PyoCN** showed excellent EQE_max_ of 33.7% [CIE coordinates of (0.31, 0.58)] and 36.1% [CIE coordinates of (0.31, 0.58)], with moderate efficiency roll-offs of 15.7 and 13.1% at 1000 cd m^–2^ due to an unremarkable *k*_RISC_ of 8.3 and 7.7 × 10^4^ s^–1^, all respectively.

### 4.7 Other N-Heterocycle Acceptors

Extending from small N-heterocycles like pyridine, pyrazine, and pyrimidine, larger or more elaborate π-systems have also been explored in green-emissive D-A TADF emitter design. For example, two pyridine units were fused together to create a napthylpyridine acceptor and coupled with phenoxazine or phenothiazine donors in **NyDPO** and **NyDPt** (Figure 56).^429^ The most interesting feature of these two linear emitters is the high degree of horizontal TDM orientation they exhibit in 5 wt% doped films in mCP, with Θ// of 81 and 84% respectively. **NyDPO** has a Φ_PL_ of 79%, which combined with the preferential horizontal dipole orientation led to an EQE_max_ of 29.9% (Table S2). **NyDPt** on the other hand has a much lower Φ_PL_ of 45% due to the presence of a non-TADF quasi-axial conformer. The impact of the two conformers could be seen from the Δ*E*_ST_ measurements, where the Δ*E*_ST_ **NyDPO** is small at 0.09 eV, while there are two Δ*E*_ST_ values for **NyDPT** from the quasi-axial and quasi-equatorial conformers, at 0.59 eV (too large for TADF to occur) and 0.016 eV, respectively. The OLEDs with **NyDPT** nonetheless showed an EQE_max_ of 25.8%. Both materials unfortunately presented long τ_d_ which led to severe efficiency roll-off, and especially for the devices with **NyDPt** where the EQE_1000_ was only 6.5%. The same group later reported the related structure **NyDPAc**, composed of the same napthylpyridine acceptor but coupled to DMAC.^430^ **NyDPAc** emits at λ_PL_ of 510 nm, has a Φ_PL_ of 57%, τ_d_ of 451 μs, and a large ΔE_ST_ of 0.29 eV in 10 wt% doped films in DPEPO. The device with **NyDPAc** showed an EQE_max_ of 20.9% [λ_EL_ = 516 nm, CIE coordinates of (0.28, 0.53)], which was lower than those with **NyDPO** and **NyDPt**, though the preferentially horizontally oriented TDM of **NyDPAc** compensated somewhat for its lower Φ_PL_.

**Figure 56:**
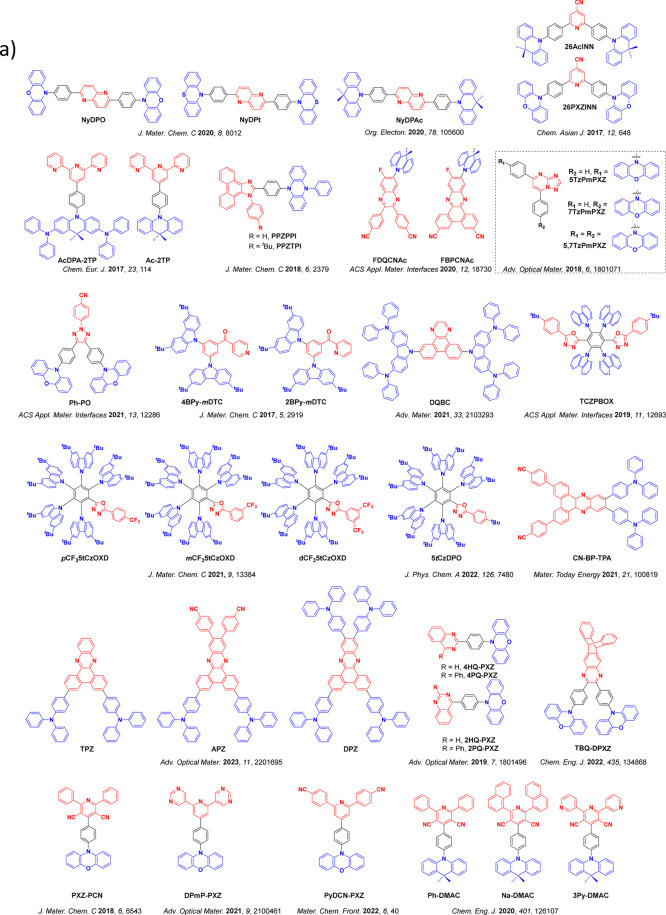

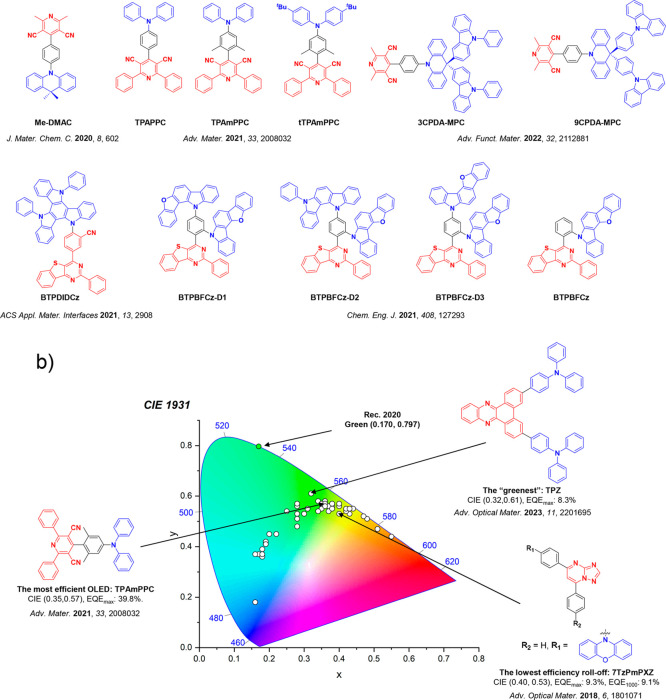
a) Molecular structures of green D-A TADF emitters containing other N-heterocycle acceptors and b) CIE color coordinates of green D-A TADF emitters containing other N-heterocycle acceptors. The white circles illustrate the spread of the emission color of the device. Selected devices and their associated CIE coordinates are highlighted, illustrating the structure of the emitter of the “greenest” device, the structure of the emitter used in the device showing the highest efficiency and the structure of the emitter with the lowest efficiency roll-off. Only D-A TADF OLEDs where the λ_EL_ = 490–580 nm which show EQE_max_ > 20% or have minimal roll-off are included. The most efficient device is quantified by the highest EQE_max_. The device with the CIE coordinates closest to the Rec. 2020 defined coordinates for green, (0.170, 0.797), is defined as the “greenest”. In the chemical structures, the blue color signifies donor moieties, while the red color signifies acceptor moieties.

Two D-A-D emitters with a 4-cyanopyridine acceptor, **26AcINN** and **26PXZINN** (Figure 56), were developed by Sasabe *et al*.^431^ **26AcINN** and **26PXZINN** emit at λ_PL_ of 495 and 522 nm, have the same Φ_PL_ of 79%, but divergent τ_d_ of 117 and 27 μs, all respectively, in 10 wt% doped films in CBP (Table S2). The devices with **26AcINN** and **26PXZINN** showed EQE_max_ of 21.6% [λ_EL_ = 501 nm, CIE coordinates of (0.22, 0.45)] and 22.7% [λ_EL_ = 527 nm, CIE coordinates of (0.34, 0.58)]. The device with **26AcINN** showed considerable efficiency roll-off, with efficiency dropping by 36% at 100 cd m^–2^ and by 66% at 1000 cd m^–2^, while the device **26PXZINN** showed a much smaller efficiency roll-off of 2% at 100 cd m^–2^ and 26% at 1000 cd m^–2^. Because of the use of the stronger PXZ donor a smaller Δ*E*_ST_ of 0.06 eV and faster τ_d_ of 27 μs was achieved for **26PXZINN** compared to **26AcINN** (Δ*E*_ST_ = 0.28 eV; τ_d_ = 117 μs), which explains the starkly contrasting device efficiency roll-off behavior. The same group also reported a family of emitters containing a new terpyridine acceptor.^432^ An acridine donor either with or without peripheral diphenylamine units was coupled to this terpyridine to give emitters **AcDPA-2TP** and **Ac-2TP**. By employing a donor dendron, **AcDPA-2TP** has a higher Φ_PL_ of 62% compared to **Ac-2TP** (Φ_PL_ of 53%) while the Δ*E*_ST_ was reduced from 0.38 eV in **Ac-2TP** to 0.03 eV in **AcDPA-2TP**, consequently improving the TADF characteristics as reflected in the much shorter τ_d_ of 15 versus 319 ms. The devices with **AcDPA-2TP** showed a much-improved EQE_max_ of 23.7% and superior efficiency roll-off (EQE_1000_ remaining at 21.9%) compared to the devices with **Ac-2TP** (EQE_max_ of 9.2% and EQE_1000_ of 1.1%).

Huang *et al*. used the strong donor phenazine coupled to imidazole-based acceptors to produce emissive TADF compounds **PPZTPI** and **PPZPPI** (Figure 56).^433^ With Φ_PL_ of 73% for **PPZTPI** (λ_PL_ of 527 nm) and 99% for **PPZPPI** (λ_PL_ of 533 nm), both emitters also showed comparable Δ*E*_ST_ of 0.11 and 0.12 eV along with fairly long τ_d_ of 127 and 118 μs, all respectively (Table S2). The devices with **PPZTPI** (λ_EL_ of 528 nm) and **PPZPPI** (λ_EL_ of 528 nm) showed EQE_max_ of 20.5 and 21.1%, however, the long-lived excitons proved damaging to the efficiency roll-off (∼33 and ∼21% at 100 cd m^–2^, all respectively). Kothavale *et al*. also employed phenazine as the donor to give **FDQCNAc** (λ_PL_ of 549 nm) and also employed a fused phenanthrene to give red emitter **FBPCNAc** (λ_PL_ of 607 nm) (Figure 56).^434^ **FDQCNAc** and **FBPCNAc** have Φ_PL_ of 87 and 79% with τ_d_ of 24.0 and 11.1 μs, respectively. Introduction of a fluorine atom in the emitter helped to achieve small Δ*E*_ST_ of 0.08 and 0.04 eV in 1 wt% doped PS films at 300 K. These modifications led to highly efficient devices using 1 wt% emitter doped in PBICT, having EQE_max_ of 27.6% [λ_EL_ of 554 nm, CIE coordinates of (0.42, 0.55),] for the device with **FDQCNAc** and 23.8% [λ_EL_ of 597 nm, CIE coordinates of (0.55, 0.44)] for the device with **FBPCNAc**.

Singly substituted D-A systems **5TzPmPXZ** (λ_PL_ = 527 nm) and **7TzPmPXZ** (λ_PL_ = 532 nm, Figure 56) containing an unusual [1,2,4]triazolo[1,5-a]pyrimidine (**TzPm**) have moderate Φ_PL_ of 64 and 49% and small Δ*E*_ST_ of 0.10 and 0.07 eV along with fast τ_d_ of 2.9 and 2.8 μs in 0.7 wt% doped films in CBP, all respectively (Table S2).^435^ The corresponding D-A-D emitter, **5,7TxPmPXZ** (λ_PL_ = 543 nm), showed slightly improved TADF behavior and slightly red-shifted emission, with similar Φ_PL_ (66%), Δ*E*_ST_ (0.06 eV), and τ_d_ (2.6 μs). Solution-processed devices showed EQE_max_ of 9.3% with both D-A emitters (λ_EL_ 542 and 552 nm for the devices with **5TzPmPXZ** and **7TzPmPXZ**, respectively), and 14.3% for the OLED with **5,7TxPmPXZ**. Low efficiency roll-off of just 2% at 1000 cd m^–2^ for the device with **7TzPmPXZ** and 13% for the device with **5,7TzPmPXZ** were noted while the efficiency dropped by 22% for the device with **5TzPmPXZ**.

1,2,4-Triazole was also recently used as an acceptor by Kim *et al*. in a series of six emitters.^436^ The best performing OLED used the phenoxazine derivative **Ph-PO** (Figure 56), which showed an EQE_max_ of 20.8% at λ_EL_ of 524 nm – this being strongly red-shifted compared to the devices with the other emitters due the use of stronger electron-donating phenoxazine. The high device EQE_max_ is the result of a confluence of a Φ_PL_ of 78% (λ_PL_ of 525 nm), a small Δ*E*_ST_ 0.14 eV, and a τ_d_ of 5.36 μs in 20 wt% doped films in DPEPO (Table S2), which matches the composition of the EML.

Two blue-green OLEDs with λ_EL_ at 490 nm were prepared using pyridyl-ketone acceptors coupled to *tert*-butylcarbazole donors in **4BPy-mDTC** and **2Bpy-mDTC** (Figure 56).^364^ The devices with **4BPy-mDTC** and **2Bpy-mDTC** showed EQE_max_ of 28.1 and 28.0% with CIE coordinates of (0.17, 0.37) and (0.16, 0.37), although the efficiency roll-off was significant at ∼58 and ∼53% at 1000 cd m^–2^, all respectively (Table S2). The very high efficiencies were due to the near unity Φ_PL_ of 97 and 96%, resulting from the pyridyl nitrogen atom restricting conformational changes in the excited state, along with small ΔE_ST_ of 0.01 and 0.02 eV.

Chen *et al.*, reported one of the highest EQE_max_ for green TADF OLEDs using the emitter **DQBC** (Figure 56). **DQBC** emits at λ_PL_ of 551 nm, and the D-A-D structure having an extended π-conjugation helped to achieve a small Δ*E*_ST_ of 0.06 eV as well as a high Φ_PL_ of 95% with fast k_RISC_ of 1.16 × 10^6^ s^–1^ and τ_d_ of 5.5 μs (Table S2).^437^ Furthermore, the TDM of **DQBC** is strongly horizontally oriented and this coupled with its high Φ_PL_ explain the outstanding EQE_max_ of 39.1% (λ_EL_ of 534 nm), with an efficiency roll-off of 25.6% at 1000 cd m^–2^ when doped at 20 wt% in the mCPBC host. When increasing the concentration from 10 wt% (EQE_max_ 29.3%) to 30 wt% (EQE_max_ 32.2%), a red-shift of the emission from 528 to 538 nm was observed. The low EQE at other doping ratios was attributed to poor charge transport at low doping and severe aggregation-caused quenching at high doping.

**TCZPBOX** (Figure 56) is an emitter composed of oxadiazole acceptors coupled with carbazole donors.^438^ Only a slight decrease in Φ_PL_ was observed moving from the 40 wt% doped PYD2 films (89%, λ_PL_ = 527 nm) to neat films (71%, λ_PL_ = 546 nm), with the red-shift suggests some aggregation in the neat film. Both doped and neat films showed TADF characteristics with Δ*E*_ST_ 0.03 eV for both. A shorter biexponential decay with τ_d_ of 4 and 30 μs was reported for the 40% doped PYD2 films, which is nonetheless very similar to the τ_d_ of 4 and 26 μs of neat films. The doped and non-doped devices showed EQE_max_ of 27.9 and 20.2%, respectively, and the efficiency decreased by only 5% at 100 cd m^–2^ for both and by either 13 or 14% at 1000 cd m^–2^. This report therefore contained some of the first high-performance non-doped TADF OLEDs. Peripheral substituents in similar carbazole/oxadiazole-based TADF emitters was studied by Hu *et al*.^439^ In ***p*CF_3_5tCzOXD**, **mCF_3_5tCzOXD**, and **dCF_3_5tCzOXD**, *tert*-butyl and CF_3_ groups were attached to the periphery of both the carbazole and oxadiazole. The purpose of the *tert*-butyl group was to decrease intermolecular interactions, while electronic tuning was mediated by the electron-withdrawing CF_3_ groups. ***p*CF_3_5tCzOXD** (λ_PL_ = 535 nm in CH_2_Cl_2_) and **mCF_3_5tCzOXD** (λ_PL_ = 532 nm in CH_2_Cl_2_) have Φ_PL_ of 66.7 and 66.2%, small Δ*E*_ST_ values of 0.12 and 0.005 eV, τ_d_ of 2.16 and 1.90 μs, and k_RISC_ values of 2.3 and 2.1 × 10^6^ s^–1^ in 10 wt% doped films in o-CzOXD, all respectively (Table S2). Devices with ***p*CF_3_5tCzOXD** and ***m*CF_3_5tCzOXD** doped in 26DCzPPy showed EQE_max_ of 20.3% (λ_EL_ = 494 nm) and 22.1% (λ_EL_ = 494 nm). The device with **dCF_3_5tCzOXD**, which has two CF_3_ substituents, showed an increased EQE_max_ of 23.3% (λ_EL_ = 496 nm) due to its superior Φ_PL_ of 87.8% and k_RISC_ of 4.6 × 10^6^ s^–1^. Cooper *et al*. similarly used oxadiazole as the acceptor in the emitter **5tCzDPO**, which contained five *tert*-butylcarbazole donors.^335^ **5tCzDPO** emits at λ_PL_ of 496 nm in toluene while the emission is blue-shifted to 474 nm in the 12.5 wt% doped films in DPEPO. In DPEPO the Φ_PL_ is 79%, the Δ*E*_ST_ is 0.01 eV, and the τ_d_ is 6.8 μs. The OLED with **5tCzDPO** emitted at around λ_EL_ of 490 nm and exhibited an EQE_max_ of 29.0% at CIE coordinates of (0.18, 0.36), however the efficiency roll-off was significant at 55.2% at 1000 cd m^–2^.

Zhou *et al*. used dibenzo[a,c]phenazine as an electron acceptor in **CN-BP-TPA** (Figure 56). This compound emits at λ_PL_ of 578 nm, has a Φ_PL_ of almost 100% in 10 wt% doped film in CBP and a moderate Δ*E*_ST_ of 0.19 eV (Table S2).^440^ The device showed an EQE_max_ of 26.0% at λ_EL_ of 580 nm and CIE coordinates of (0.51, 0.47). Strong π-π interactions between the π-conjugated phenazine acceptors in neighboring molecules had an adverse effect on the neat film Φ_PL_, reflected in the much lower EQE_max_ of the non-doped device of 5.0% and the strongly red-shifted emission at 607 nm. Liu *et al*. used similar acceptors with the electron donors introduced at different positions in **DPZ**, **TPZ**, and **APZ**.^441^ These three compounds showed yellow-green emission at λ_PL_ = 539, 564, and 577 nm with Φ_PL_ of 54, 67, and 86%, respectively in 10 wt% doped films in CBP. Due to their large Δ*E*_ST_ values of 0.29, 0.34, and 0.20 eV, the emitters have long τ_d_ of 284, 240 and 298 μs, respectively. However, the horizontally oriented TDMs in the 10 wt% doped films were measured to be 84, 92 and 88%, respectively. Thus, the best-performing device in the study with **APZ** achieved an EQE_max_ of 27.5% at λ_EL_ of 562 nm and CIE coordinates of (0.44, 0.55), although the efficiency roll-off was very poor with the EQE decreasing by 86% at 1000 cd m^–2^.

Another nitrogen-rich acceptor quinazoline was coupled to phenoxazine to produce a series of emitters **4HQ-PXZ**, **4PQ-PXZ**, **2HQ-PXZ**, and **2PQ-PXZ** (Figure 56).^442^ Smaller Δ*E*_ST_ of 0.10 and 0.09 eV were reported for **2HQ-PXZ** and **2PQ-PXZ** compared to 0.19 and 0.22 eV for **4HQ-PXZ** and **4PQ-PXZ**, all in 6 wt% doped films in CBP. This contrast was due to more twisted geometries adopted by **2HQ-PXZ** and **2PQ-PXZ**, associated with substitution of the donor at the 4-position of the quinazoline. This also resulted in faster τ_d_ of 35.9 and 28.3 μs along with improved Φ_PL_ of 81.0 and 73.9% for **2HQ-PXZ** and **2PQ-PXZ**, compared to 48.3 and 40.1 μs and 66.9 and 67.5% for **4HQ-PXZ** and **4PQ-PXZ**, all respectively. Devices with **2HQ-PXZ** and **2PQ-PXZ** showed EQE_max_ of 16.0% [λ_EL_ = 538 nm, CIE coordinates of (0.36, 0.57)] and 17.1% [λ_EL_ = 538 nm, CIE coordinates of (0.36, 0.56)], while those with **4HQ-PXZ** and **4PQ-PXZ** showed higher EQE_max_ of 20.2% [λ_EL_ = 511 nm, CIE coordinates of (0.25, 0.54)] and 20.5% [λ_EL_ = 518 nm, CIE coordinates of (0.28, 0.57)] (Table S2). The trend in Φ_PL_ is opposite to that of the EQE_max_, which was ascribed to different populations of conformers present in the devices for the different materials, resulting from the two PXZ units (crooked and planar form). The OLEDs with **4HQ-PXZ**, **4PQ-PXZ**, **2HQ-PXZ**, and **2PQ-PXZ** all showed similar efficiency roll-off at 100 cd m^–2^ of 22, 37, 14, and 27%, respectively.

Ji *et al*. reported emitters **TBP-DPXZ** (Figure 56) (a red emitter) and **TBQ-DPXZ** (Figure 56) consisting of phen­oxa­zine donors attached to either locked (trip­ty­cene-fused) diben­zo­phena­zine or unlocked (trip­ty­cene-fused) 2,3-di­phen­yl­quin­oxa­line acceptors.^443^ Due to the weaker electron-withdrawing ability of the unlocked acceptor and the small HOMO-LUMO overlap, **TBQ-DPXZ** emits at 537 nm with a Φ_PL_ of 91%, Δ*E*_ST_ of 0.07 eV, and τ_d_ of 3.6 μs in 20 wt% doped films in BCPO (Table S2). **TBP-DPXZ** with the locked phenanthrene acceptor emits at 586 nm, has a Φ_PL_ of 50.3%, a Δ*E*_ST_ of 0.03 eV, and a τ_d_ of 4.8 μs. The green OLED with **TBQ-DPXZ** showed an EQE_max_ of 25.1% at λ_EL_ of 533 nm and CIE coordinates of (0.35, 0.55), but the efficiency roll-off was strong with the EQE dropping by 53% at 1000 cd m^–2^].

Chen *et al*. reported green emitter **PXZ-PCN** that contains a PXZ donor and a dicy­ano­pyri­dine acceptor (Figure 56).^444^ This compound emits at λ_PL_ of 565 nm and has a Φ_PL_ of 57%, a small Δ*E*_ST_ of 0.01 eV, and a τ_d_ of 1.58 μs in 10 wt% doped films in CBP. The device showed an EQE_max_ of 15.1% [λ_EL_ = 568 nm, CIE coordinates of (0.48, 0.51)], and efficiency roll-off of 21% at 1000 cd m^–2^. A similar structure with 2,6-di(pyri­mi­din-5-yl) pyridine as the acceptor (**DPmP-PXZ**) was used by Shi *et al*. to develop a highly efficient non-doped OLED.^445^ A putative hydrogen-bonding network present in the neat films of **DPmP-PXZ** controls the conformation of the emitter and the orientation of the TDM, suppressing exciton annihilation and improving charge mobility in the non-doped device. The non-doped device showed an EQE_max_ of 21.8% (λ_EL_ = 560 nm), while a 60 wt% doped device in mCP host showed a modestly higher EQE_max_ of 23.6% (Table S2). **PyDCN-PXZ** is another emitter with a similar acceptor and a phenoxazine donor that emits at λ_PL_ of 532 nm in toluene, has a Φ_PL_ of 89.6%, and a small Δ*E*_ST_ of 0.06 eV in 15 wt% doped CBP film. The devices showed an EQE_max_ of 26.9% at λ_EL_ of 519 nm.^446^

Liu *et al*. reported three emitters based on differently substituted dicyanopyridine, **Ph-DMAC**, **Na-DMAC**, and **3Py-DMAC** (Figure 56).^447^ The 10 wt% doped films in CBP of these emit at λ_PL_ of 532, 531 and 538 nm, respectively. **Ph-DMAC** has a higher Φ_PL_ of 89% (Δ*E*_ST_ of 0.06 eV) compared to those of **Na-DMAC** and **3Py-DMAC** with Φ_PL_ of 56 (Δ*E*_ST_ of 0.15 eV) and 60% (Δ*E*_ST_ of 0.04 eV). The devices with the best performance employed **Ph-DMAC** as the emitter and showed an EQE_max_ of 29.1% at 539 nm, while the OLEDs with **Na-DMAC** and **3Py-DMAC** with stronger acceptors showed red-shifted emission at 554 and 567 nm and lower EQE_max_ of 21.2 and 21.5%. OLEDs with the three emitters presented quite different efficiency roll-off behavior: the devices with **Ph-DMAC** and **3Py-DMAC** showed EQE_100_ and EQE_1000_ of 21.7/18.5% and 18.9/17.3% respectively (Table S2), with the smaller efficiency roll-off of the latter correlated to its shorter τ_d_ (2.5 μs for **Ph-DMAC** and 1.5 μs for **3Py-DMAC**) and faster RISC (k_RISC_ of 0.96 × 10^6^ s^–1^ for **Ph-DMAC** and 1.83 × 10^6^ s^–1^ for **3Py-DMAC**). By contrast, the device with **Na-DMAC** (τ_d_ of 0.68 μs and k_RISC_ of 1.6 × 10^6^ s^–1^) showed the most severe efficiency roll-off, with the EQE_1000_ dropping significantly to 8.3%. **Me-DMAC** is an emitter that contains a similar dimeth­yl­di­cyan­o­pyri­dine acceptor.^448^ Its twisted geometry ensured separation of the HOMO and LUMO, leading to a ΔE_ST_ of 0.12 eV, and emitting at λ_PL_ of 542 nm in toluene. Photophysical investigations of 10 wt% doped CBP film showed a high Φ_PL_ of 96%, a short τ_d_ of 2.7 μs, and a fast k_RISC_ of 1.7 × 10^6^ s^–1^. The OLED showed an EQE_max_ of 25.8% [CIE coordinates of (0.28, 0.51)], which decreased to 18.2% at 1000 cd m^–2^. **TPAPPC**, **TPAmPPC**, and **tTPAmPPC** are three additional emitters that also employ pyridine-carbonitrile acceptors.^449^ Due to the presence of the 3,5-dicyano groups on the pyridine ring alongside 2,6-dimethyl groups on the linking phenyl ring these emitters adopt a strongly twisted confirmation, leading to small Δ*E*_ST_ of 0.027 and 0.020 eV in **TPAmPPC** and **tTPAmPPC**, yet retaining high Φ_PL_ of 100 and 79%, all respectively. The dihedral angle between the D/A planes of **TPAPPC**, which does not have the methyl groups, is much smaller (38.2°) resulting in comparatively large Δ*E*_ST_ of 0.21 eV, yet a Φ_PL_ of 100%. The OLEDs with **TPAmPPC** showed a record-breaking EQE_max_ of 39.8% at λ_EL_ of 537 nm with CIE coordinates of (0.35, 0.57). The device with **TPAPPC** (λ_EL_ = 520 nm) showed an EQE_max_ of 37.5% [CIE coordinates of (0.28, 0.56)], while the device with **tTPAmPPC** (λ_EL_ = 556 nm) showed a relatively lower EQE_max_ of 29.8% [CIE coordinates of (0.42, 0.55)] due to its lower Φ_PL_ (79%). A non-doped device was also fabricated using **TPAmPPC**, showing an EQE_max_ of 22.2%. Xie *et al*. reported the emitters **3CPDA-MPC** and **9CPDA-MPC**, which use a similar dicy­ano­pyri­dine-based acceptor linked to carbazolyl acridine donor dendrons.^450^ Neat films of **3CPDA-MPC** and **9CPDA-MPC** emit at λ_PL_ of 522 and 509 nm, have Φ_PL_ of 89 and 92%, ΔE_ST_ of 0.11 and 0.13 eV, and τ_d_ of 1.65 and 2.05 μs as well as showing preferential horizontal TDM orientation (Θ// of 73 and 78%) in neat films, all respectively. Due to the higher Φ_PL_, enhanced horizontal orientation ratio, and charge carrier mobility, the non-doped OLED with **9CPDA-MPC** demonstrated better performance at 510 nm with EQE_max_ of 29.6%.

The compound **BTPDIDCz** (Figure 56) containing a benzo­thi­eno­pyri­mi­dine acceptor with triazatruxene as the donor moiety was reported by Lee *et al*.^451^ It emits at λ_PL_ of 520 nm and has a Φ_PL_ of 83%, a small Δ*E*_ST_ of 0.01 eV, and a τ_d_ of 4.1 μs. The device with **BTPDIDCz** showed an EQE_max_ of 24.5% at CIE coordinates of (0.38, 0.57) and had a very low efficiency roll-off, with EQE_3000_ of 23.2%. The same group also coupled a 5H-benzofuro[3,2-c]carbazole donor ortho to the benzo­thi­eno­pyri­mi­dine acceptor in **BTPBFCz**. Three derivatives of this reference emitter, **BTPBFCz-D1**, **BTPBFCz-D2** and **BTPBFCz-D3**, contain one of the three secondary donors, 5H-benzofuro[3,2-c]carbazole, 12H-ben­zo­furo­[3,2-a]car­ba­zole, or 5-phen­yl-5,12-di­hy­dro­in­dolo­[3,2-a]car­ba­zole, all emit around 480 nm in toluene. These structural modifications result in increased Φ_PL_ of 84, 85, and 92% in 20 wt% doped films in DPEPO, respectively, compared to 74% for **BTPBFCz** (Table S2).^452^ While the Δ*E*_ST_ of **BTPBFCz** is 0.09 eV, those of **BTPBFCz-D1**, **BTPBFCz-D2**, and **BTPBFCz-D3** are larger at 0.10, 0.23, and 0.12 eV, respectively. The τ_d_ of all of the emitters range narrowly from 15.6 to 22.0 μs. The devices with **BTPBFCz-D1**, **BTPBFCz-D2**, **BTPBFCz-D3** showed comparable EQE_max_ of 20.7, 20.0, and 22.7% at λ_EL_ ranging from 491 to 497 nm and with CIE coordinates of (0.18, 0.39), (0.18, 0.37), and (0.19, 0.41), all respectively. This represents an improvement of more than 40% over the EQE_max_ of the device with **BTPBFCz** [EQE_max_ of 15.8%, CIE coordinates of (0.20, 0.43)].

### 4.8 Carbonyl Containing Acceptors

Similar to sulfones, ketones and other carbonyl-based acceptors are popular in TADF materials design, with the low-lying n-π* transition of the carbonyl able to facilitate ISC/RISC.^453^ For example, an imide acceptor was coupled to two carbazole donors to give two bright emitters **AI-Cz** and **AI-TBCz** (Figure 57).^454^ They emit at λ_PL_ of 510 and 545 nm, have Φ_PL_ of 84 and 72%, Δ*E*_ST_ of 0.09 and 0.08 eV, and rather long τ_d_ of 81 and 64 μs, all respectively (Table S2). OLEDs with **AI-Cz** and **AI-TBCz** showed EQE_max_ of 23.2% (λ_EL_ = 510 nm) and 21.1% (λ_EL_ = 540 nm) but showed significant efficiency roll-off (EQE_100_ of 15.2 and 11.5% and EQE_1000_ of 7 and 5.5%, all respectively).

**Figure 57:**
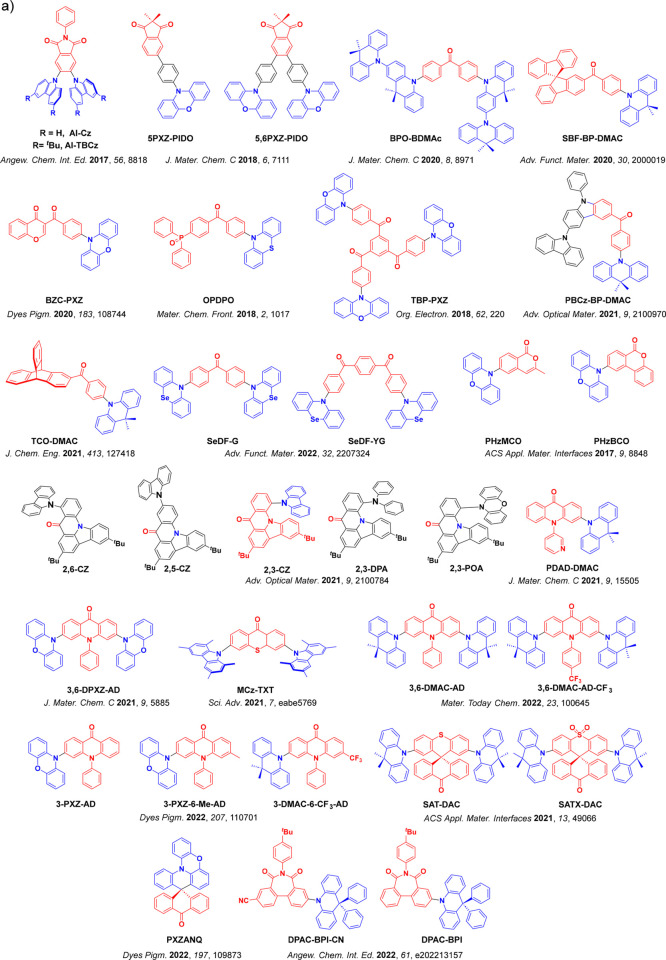

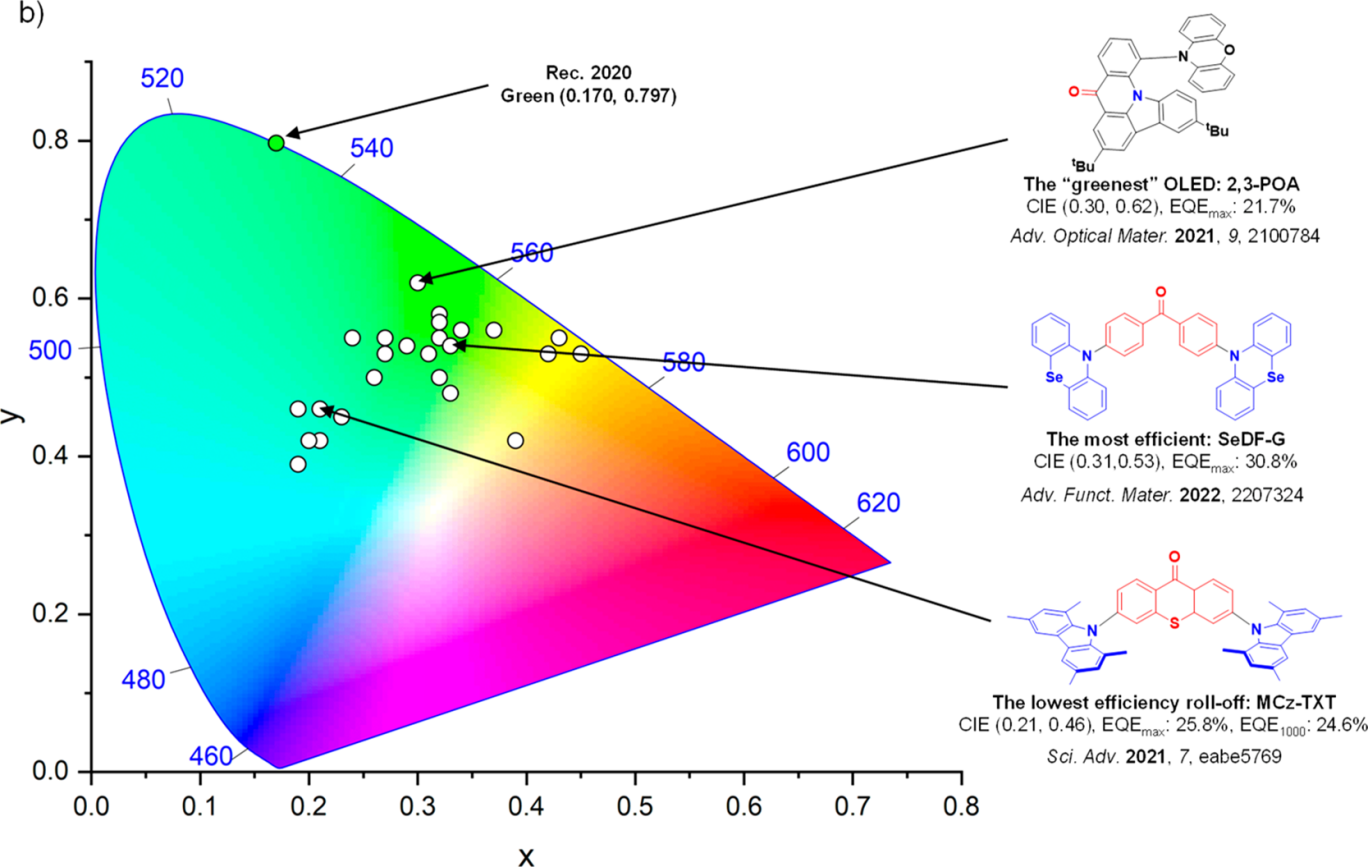
a) Molecular structures of green D-A TADF emitters containing carbonyl acceptors and b) CIE color coordinates of green D-A TADF emitters containing carbonyl acceptors. The white circles illustrate the spread of the emission color of the device. Selected devices and their associated CIE coordinates are highlighted, illustrating the structure of the emitter of the “greenest” device, the structure of the emitter used in the device showing the highest efficiency quantified by the EQE_max_ and the structure of the emitter with the lowest efficiency roll-off. Only D-A TADF OLEDs where the λ_EL_ = 490–580 nm which show EQE_max_ > 20% or have minimal roll-off are included. The most efficient device is quantified by the highest EQE_max_. The device with the CIE coordinates closest to the Rec. 2020 defined coordinates for green, (0.170, 0.797), is defined as the “greenest”. In the chemical structures, the blue color signifies donor moieties, while the red color signifies acceptor moieties.

Xiang *et al*. introduced D-A (**5PXZ-PIDO**) and D-A-D (**5,6PXZ-PIDO**) emitters (Figure 57) containing a diketone acceptor coupled to PXZ donors.^455^ The two compounds emit at λ_PL_ of 535 and 544 nm and have Φ_PL_ of 72 and 76%, respectively, in 1.5 wt% doped films in CBP (Table S2). As a result of the small Δ*E*_ST_ of 0.11 and 0.06 eV and short τ_d_ of 2.37 and 1.98 ms, **5PXZ-PIDO** and **5,6PXZ-PIDO** have fast *k*_RISC_ of 4.06 and 5.89 × 10^5^ s^–1^. Although the OLEDs with **5PXZ-PIDO** and **5,6PXZ-PIDO** showed only modest EQE_max_ of 14.4% [CIE coordinates of (0.39, 0.54)] and 16.9% [CIE coordinates of (0.42, 0.53)], the fast RISC ensured relatively low efficiency roll-off of just 25 and 16% at 1000 cd m^–2^, all respectively.

Liu *et al*. combined benzophenone with the donor dendron BDMAc (9,9,9′,9′-tetra­meth­yl-9,9′,10,10′-tetra­hy­dro-2,10′-bi­acri­dine) to give **BPO-BDMAc** (Figure 57).^456^ **BPO-BDMAc** emits at λ_PL_ of 516 nm, has a Φ_PL_ of 89.1%, a Δ*E*_ST_ of 0.03 eV, and a τ_d_ of 3 μs in 25 wt% doped films in mCPCN. Solution-processed OLEDs emitted at λ_EL_ of 522 nm and showed an EQE_max_ of 22.5% (Table S2).

Benzophenones with ancillary functional substituents alongside the donors are a recurring theme in emitter design. A ketone-based emitter featuring an acridine donor and a spirobifluorene, **SBF-BP-DMAC** (Figure 57), was developed by Zheng *et al*.^457^ With a high Φ_PL_ of 72.1% as a neat film this compound was employed in both non-doped and doped devices, giving EQE_max_ of 20.1 and 24.5%, respectively (Table S2). Wang *et al*. developed emitter **BZC-PXZ** which similarly features a ketone acceptor unit with a chromone moiety and a phenoxazine donor unit, which emits at λ_PL_ of 561 nm, has a small Δ*E*_ST_ of 0.02 eV, and a high Φ_PL_ of 93% in 5 wt% doped films in mCP.^458^ The OLEDs with **BZC-PXZ** emitted at λ_EL_ of 544 nm and showed an EQE_max_ of 22.0% with a small efficiency roll off of 7.3% at 1000 cd m^–2^. In a similar manner asymmetric phosphine oxide-substituted benzophenone-based emitter **OPDPO** was reported by Chen *et al*.^459^ The compound emits at λ_PL_ of 589 nm and has a small Δ*E*_ST_ of 0.02 eV as a neat film. OLEDs with **OPDPO** doped in CBP (10 wt%) showed an EQE_max_ of 26.7% at λ_EL_ 552 nm, as well as a low efficiency roll-off of 18% at 1000 cd m^–2^. The same emitter was used to prepare a non-doped device that displayed red-shifted emission with λ_EL_ of 588 nm, a lower EQE_max_ of 16.6%, and a higher efficiency roll-off of 29% at 1000 cd m^–2^. The efficiency roll-off could be improved to 9% by increasing the emitter layer thickness from 7 to 10 nm, however the EQE_max_ decreased to 12.8%.

Bai *et al*. introduced an emitter composed of a triketone acceptor coupled to phenoxazine donors, **TBP-PXZ** (Figure 57).^460^ The 10 wt% doped CBP film emits at λ_PL_ of 592 nm and has a Φ_PL_ of 68%, a τ_d_ of 11.9 μs, and a Δ*E*_ST_ of 0.02 eV. The relatively short τ_d_ was postulated to be due to the presence of multiple conformers, some of which facilitate efficient RISC. The OLEDs with **TBP-PXZ** emitted at λ_EL_ of 564 nm and showed an EQE_max_ of 17.7% [CIE coordinates of (0.45, 0.53)], which decreased only slightly to 16.0% at 1000 cd m^–2^.

A self-hosting AIE-based TADF material, **PBCz-BP-DMAC** (Figure 57), was reported by Dong *et al*.^461^ PBCz, a common host moiety, was coupled to BP-DMAC resulting in an enhancement of the charge transporting properties of the emitter. The 10 wt% doped film of **PBCz-BP-DMAC** in PPF emits at λ_PL_ of 488 nm, has a high Φ_PL_ of 92.3%, a Δ*E*_ST_ of 0.02 eV, and a τ_d_ of 9.2 μs (Table S2). Both doped (10 wt% in PPF, λ_EL_ = 492 nm) and non-doped **PBCz-BP-DMAC** devices (λ_EL_ = 494 nm) emitted effectively and give comparable EQE_max_ of 27.5% [CIE coordinates of (0.21, 0.42)] and 23% [CIE coordinates of (0.21, 0.43)], respectively. Very low efficiency roll-off was also noted for both the doped and non-doped devices, falling by just 8 or 6% at 1000 cd m^–2^.

Jing *et al*. designed **TCO-DMAC** (Figure 57), where a triptycene-fused benzophenone serves as an acceptor and is coupled to a dimethylacridine as the donor.^462^ **TCO-DMAC** emits at λ_PL_ of 499 nm and has a high Φ_PL_ of 92%, a small Δ*E*_ST_ of 0.04 eV, and fast *k*_RISC_ of 1.33 × 10^6^ s^–1^ in 20 wt% doped films in BCPO (Table S2). The OLEDs showed an EQE_max_ of 21.2% at λ_EL_ of 499 nm and CIE coordinates of (0.23, 0.45). The efficiency roll-off was also low at 4% at 100 cd m^–2^ and 17% at 1000 cd m^–2^. The non-doped OLEDs showed a somewhat lower EQE_max_ of 15.6% [λ_EL_ 501 nm, CIE coordinates of (0.25, 0.48)] but had comparable efficiency roll-off of 4% at 100 cd m^–2^ and 13% at 1000 cd m^–2^.

Sharif *et al.* employed phenoselenazine as the donor, which was coupled to benzophenone or 1,4-phen­ylene­bis­(phen­yl­meth­an­one) acceptors in **SeDF-G** and **SeDF-YG** (Figure 57).^343^ Due to the strong heavy atom effect of the selenium, enhanced spin-orbit couplings (H_so_) of 110 and 52 cm^–1^ between S_1_ and T_1_ and very fast *k*_RISC_ ≈ 10^12^ s^–1^ were calculated using DFT for the quasi-equatorial conformers of **SeDF-G** and **SeDF-YG**. Experimentally the Δ*E*_ST_ are 0.15 eV for both compounds, the τ_d_ are 3.9 and 4.6 μs, and the *k*_RISC_ are 5.7 and 10.6 × 10^6^ s^–1^ in 10 wt% doped films in mCBP, all respectively (Table S2). OLEDs with **SeDF-G** and **SeDF-YG** showed respective EQE_max_ of 30.8 and 18.8% at CIE coordinates of (0.31, 0.53) and (0.33, 0.48). However, very low Φ_PL_ of 7.6 and 8.5% were measured in the corresponding solution-processed 10 wt% doped films in mCBP, which was suggested to arise from the evaporated films having a different distribution of axial/equatorial conformers compared to solution-processed films. The higher Φ_PL_ and narrower emitting conformer was postulated to be dominant in the evaporated films relevant to the OLEDs.

Two emitters with phenoxazine coupled to coumarin-based acceptors, **PHzMCO** and **PHzBCO**, were reported by Chen *et al*. (Figure 57).^463^ Similar emission properties with λ_PL_ = 510 and 524 nm, Φ_PL_ = 47 and 52%, and τ_d_ = 17.9 and 9.3 μs were observed, all respectively, in 8 wt% doped films in mCP. The very small Δ*E*_ST_ of 0.018 eV for **PHzMCO** and 0.006 eV for **PHzBCO** ensured efficient RISC, and OLEDs with **PHzMCO** and **PHzBCO** showed relatively high EQE_max_ of 17.8% [CIE coordinates of (0.26, 0.50)] and 19.6% [CIE coordinates of (0.32, 0.50)] – surprisingly high considering the low Φ_PL_ of the emitters. The OLEDs also exhibited low efficiency roll-off, with EQE_1000_ of 15.3 and 17% and EQE_10000_ of 10.3 and 12.9%, respectively.

A series of five emitters **2,6-CZ**, **2,5-CZ**, **2,3-CZ**, **2,3-DPA**, and **2,3-POA** contained the same fused carbonyl-carbazole acceptor coupled with different donors featuring differing regiochemistry (Figure 57).^464^ Only **2,3-POA** showed CT emission due to the use of the strongly electron-donating phenoxazine, with the others being classified as MR-TADF (See Section 11). **2,3-POA** emits at λ_PL_ of 547 nm (toluene) and has a Φ_PL_ of 82.5%, a Δ*E*_ST_ of 0.01 eV, and a τ_d_ of 6.2 ms in 3.5 wt% doped films in mCBP. The OLEDs with **2,3-POA** showed an EQE_max_ of 21.7% at λ_EL_ of 528 nm, with CIE coordinates of (0.30, 0.62).

**PDAD-DMAC** (Figure 57) is an AIE TADF emitter with pyridine-substituted acridone acceptor and an acridine donor.^465^ The 20 wt% doped PPF film of this emitter has a high Φ_PL_ of 94%, a small Δ*E*_ST_ of 0.029 eV, and short τ_d_ of 4.8 μs at λ_PL_ of 502 nm. The device showed an EQE_max_ of 24.1% at a λ_EL_ of 492 nm. Using very similar acceptor but with two PXZ donors, Mei *et al*. reported **3,6-DPXZ-AD**, which emits at λ_PL_ of 563 nm in toluene. In 7 wt% doped films in CBP, the emitter has a high Φ_PL_ of 94.9% and a *k*_RISC_ of 1.1 ×10^6^ s^–1^, arising from the quasi-equatorial conformation of the molecule.^466^ The OLEDs with **3,6-DPXZ-AD** emitted at λ_EL_ of 552 nm, and showed a high EQE_max_ of 30.6% and low efficiency roll-off (6% at 100 cd m^–2^ and 27% at 1000 cd m^–2^). Mei *et al*. also incorporated methyl or trifluoromethyl groups at the 6-position of the acridone to tune the energy levels of the ^1^CT, ^3^CT, and ^3^LE states.^467^ **3-DMAC-6-CF_3_-AD**, **3-PXZ-AD**, and **3-PXZ-6-Me-AD** have Δ*E*_ST_ of near 0 eV in 2-MeTHF, leading to short τ_d_ of 3.5 μs for **3-DMAC-6-CF_3_-AD** in 7 wt% doped films in DPEPO, and 2.2 and 2.3 μs for **3-PXZ-AD** and **3-PXZ-6-Me-AD** in 7 wt% doped films in CBP, respectively. **3-DMAC-6-CF_3_-AD**, **3-PXZ-AD**, and **3-PXZ-6-Me-AD** emit at λ_PL_ of 514, 555, and 533 nm and have high Φ_PL_ of 85% (7 wt% **3-DMAC-6-CF_3_-AD** in DPEPO), 86%, and 91% (7 wt% in CBP) in the doped films, all respectively. The OLEDs with **3-DMAC-6-CF_3_-AD**, **3-PXZ-AD**, and **3-PXZ-6-Me-AD** emitted at λ_EL_ of 512, 519, and 505 nm with CIE coordinates of (0.27, 0.55), (0.32, 0.57), and (0.27, 0.53), and showed comparable EQE_max_ of 21.7, 21.1, and 23.3%, all respectively (Table S2). The same research group also reported analogous D-A-D emitters **3,6-DMAC-AD** and **3,6-DMAC-AD-CF_3_**, each containing two DMAC donors.^468^ Almost isoenergetic ^3^LE-^3^CT states and small Δ*E*_ST_ (^3^LE-^1^CT and ^3^CT-^1^CT) in 2-MeTHF resulted in strong SOC, short τ_d_ of 3.4 and 2.2 μs, and fast RISC (*k*_RISC_ = 2.6 and 4.2 × 10^6^ s^–1^), along with Φ_PL_ of 81.1 and 74.4%, all respectively in 7 wt% doped films in DPEPO. OLEDs with **3,6-DMAC-AD** and **3,6-DMAC-AD-CF_3_** showed EQE_max_ of 23.2 and 21.6% and had low efficiency roll-off of 20 and 5% at 1000 cd m^–2^.

Aizawa *et al.* employed a thioxanthone acceptor in the emitter **MCz-TXT** (Figure 57).^183^ The 10 wt% doped film in mCBP emits at λ_PL_ of 490 nm and has a high Φ_PL_ of 92%. The sulfur atom serves to enhance the SOC between S_1_ and T_2_, thus accelerating RISC which is reflected in the very short τ_d_ of 750 ns and outstanding *k*_RISC_ of 1.1×10^8^ s^–1^. The OLED with **MCz-TXT** showed an EQE_max_ of 25.8% and excellent efficiency roll-off (5% at 100 cd m^–2^ and 16% at 1000 cd m^–2^) (Table S2). Inspired by this field-leading RISC rate, other groups have since studied the use of heavy-atoms in similar acceptors, extending even to polonium derivatives, albeit only computationally.^469^

Wang *et al*. designed two TADF emitters containing spiro-linked dual acceptors, **SAT-DAC** and **SATX-DAC** (Figure 57).^470^ These two compounds emit at λ_PL_ of 510 and 517 nm, have Δ*E*_ST_ of 0 and 0.05 eV as neat films, and have Φ_PL_ of 76.8 and 68.1% in 30 wt% doped films in DPEPO, all respectively. OLEDs with **SAT-DAC** and **SATX-DAC** emitted at λ_EL_ of 520 and 524 nm, and showed EQE_max_ of 22.6 and 20.9%. The spiro-D-σ-A architecture was proposed to enhance through-space charge transfer and reduce efficiency roll-off (21 and 19% at 1000 cd m^–2^_,_ respectively). The added bulk of the spiro design in this emitter design also likely helped alleviate concentration quenching. A similarly spiro-linked TADF emitter with anthracenone acceptor, **PXZANQ**, was reported by Yang *et al*.^471^ The rigidly orthogonal arrangement between the donor and acceptor fragments led to a Φ_PL_ of 71%, a small Δ*E*_ST_ of 0.03 eV, and a τ_d_ of 10.2 μs in 10 wt% doped films in DPEPO (Table S2), similar to the previously studied **ACRSA**.^453,472–475^ The OLED with **PXZANQ** emitted at 528 nm with CIE coordinates of (0.33, 0.54), and showed an EQE_max_ of 22.1% – much higher than the ∼16% EQE_max_ previously reported for the device with **ACRSA**.

Huang *et al*. used an electron-deficient heptagonal diimide to conformationally lock a biphenyl-based acceptor in emitters **DPAC-BPI-CN** and **DPAC-BPI** (Figure 57).^476^ The stronger electron-withdrawing ability of the cyano-substituted acceptor endowed **DPAC-BPI-CN** with a smaller Δ*E*_ST_ of 0.07 eV in toluene (0.15 eV for **DPAC-BPI**) and a red-shifted emission at 525 nm in neat film (472 nm for **DPAC-BPI**). The modestly flexible heptagonal geometry suppressed intermolecular interactions, reflected in the high Φ_PL_ of 90.1% of the neat film. The **DPAC-BPI-CN** neat films also showed a high Θ// of 83%, resulting in device EQE_max_ of 26.2% at 531 nm.

### 4.9 Other Acceptors

While trifluoromethyl groups have been used as auxiliary electron-withdrawing groups^477^ and investigated as acceptors computationally,^478^ one of the few experimental examples using it directly as an acceptor group is **7CzFDCF_3_DPh** (Figure 58).^479^ This emitter is composed of two phenyl rings; one substituted by four carbazoles and a trifluoromethyl group *para* to the other phenyl, and the other decorated with three carbazoles, one trifluoromethyl at the *para* position, and a fluorine at the *ortho* position. This design ensured a strongly twisted conformation between the two halves of the emitter, resulting in a small Δ*E*_ST_ of 0.05 eV. **7CzFDCF_3_DPh** emits at 555 nm, has a Φ_PL_ of 55%, and a *k*_RISC_ of 9.5 × 10^5^ s^–1^ as a neat film. The corresponding OLEDs emitted at CIE coordinates of (0.36, 0.56) and showed an EQE_max_ of 20.8%, with mild efficiency roll-off (EQE_100_ and EQE_1000_ of 18.5 and 16.8% respectively).

**Figure 58:**
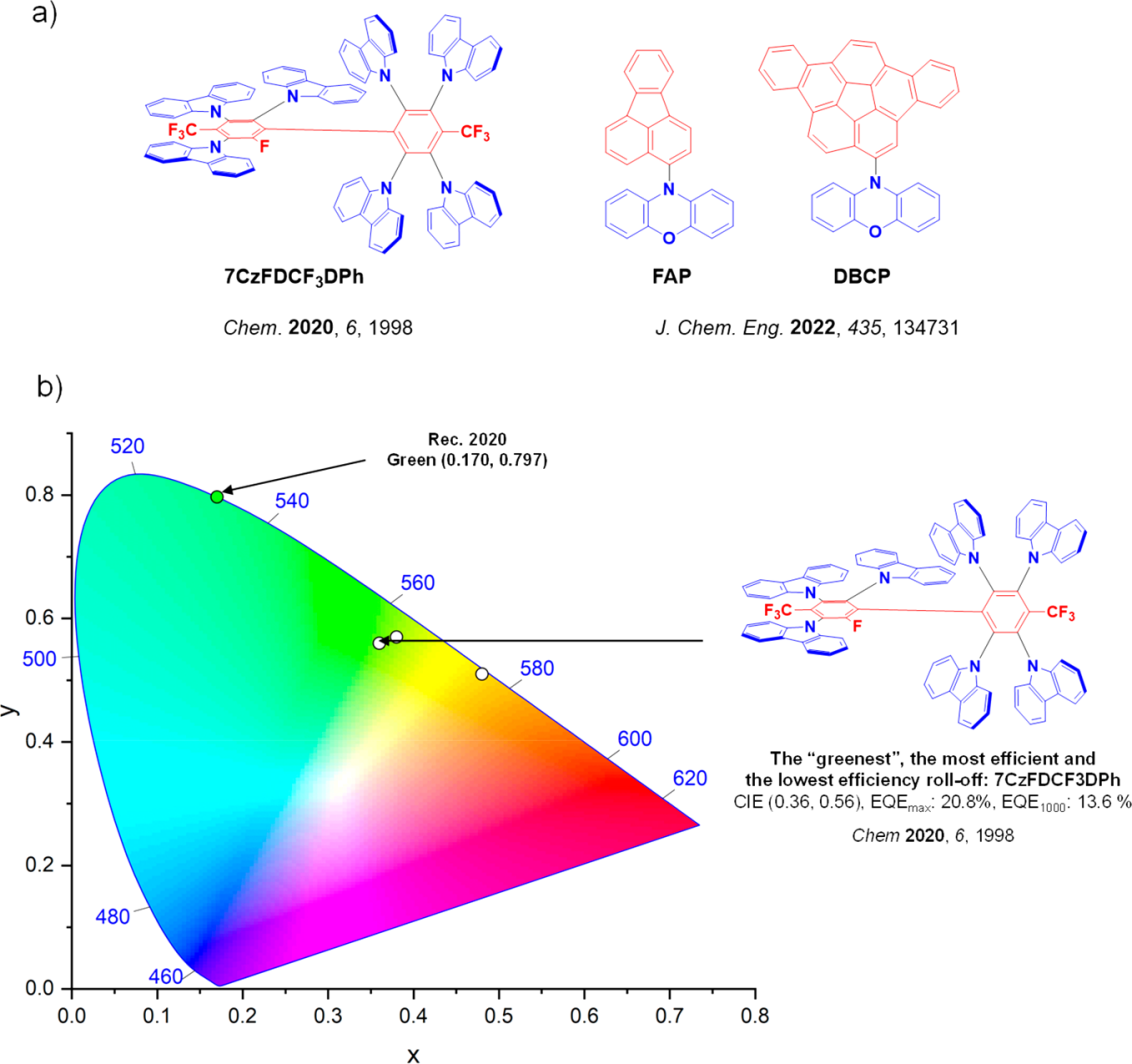
a) Molecular structures of green D-A TADF emitters containing other acceptors and b) CIE color coordinates of green D-A TADF emitters containing other acceptors. The white circles illustrate the spread of the emission color of the device. Selected devices and their associated CIE coordinates are highlighted, illustrating the structure of the emitter of the “greenest” device, the structure of the emitter used in the device showing the highest efficiency and the structure of the emitter with the lowest efficiency roll-off. Only D-A TADF OLEDs where the λ_EL_ = 490–580 nm which show EQE_max_ > 20% or have minimal roll-off are included. The most efficient device is quantified by the highest EQE_max_. The device with the CIE coordinates closest to the Rec. 2020 defined coordinates for green, (0.170, 0.797), is defined as the “greenest”. In the chemical structures, the blue color signifies donor moieties, while the red color signifies acceptor moieties.

Chen *et al.* reported the emitters **DBCP** and **FAP** that used heteroatom-free polyaromatic hydrocarbons as acceptors (Figure 58).^480^ The planar geometry of the fluoranthene showed more π-delocalization than the bowl-like dibenzocorannulene, which lowered the energy of the lowest-lying ^3^LE state in **FAP** (5% doped films in mCP) and thus increased the Δ*E*_ST_ to 0.32 eV compared to 0.10 eV for **DBCP**. **DBCP** has a higher Φ_PL_ of 89% and much shorter τ_d_ of 30 μs compared to **FAP** (Φ_PL_ of 54% and τ_d_ of 489.4 μs). The OLEDs with **DBCP** and **FAP** showed EQE_max_ of 20.2 and 12.8% at λ_EL_ of 544 and 568 nm, respectively.

### 4.10 Outlook

This section provides a detailed overview of green-emitting TADF materials. Numerous green TADF emitters have been reported between 2017 and 2022. These emitters have icorporated a range of acceptor types, including nitrile, boron, sulfone, N-heterocycles, and carbonyl-based acceptors, that permit the fine tuning, and even the enhancement, of the performance of green D-A TADF emitters. This substantial progress has led to the achievement of remarkable efficiencies in green TADF OLEDs, with EQE_max_ values now routinely exceeding 30%. This level of efficiency was unattainable at the beginning of this period.

By leveraging a preferential horizontal TDM alignment of the emitter, the performance of numerous devices has stood out by achieving EQE_max_ values approaching 40% in single-stack configurations. Among these, the OLED featuring **TPAmPPC**, an emitter containing a pyridine-carbonitrile as the acceptor, reached the pinnacle with an EQE_max_ of 39.8% and CIE coordinates of (0.35, 0.57). **CzDBA**, with diboroanthracene as the acceptor, is another exemplar emitter used in high-efficiency green-emitting devices. This OLED not only showed a remarkable EQE_max_ of 38%, but demonstrated a negligible efficiency roll-off of 0.3% at 1000 cd m^–2^ at CIE coordinates of (0.31, 0.61), in close proximity to the BT.709 green coordinates of (0.300, 0.600).

However, it remains a challenge for D-A TADF devices to meet the demanding Rec. 2020 green coordinates of (0.170, 0.797) due to their generally too broad and unstructured emission spectra that is a consequence of the CT nature of the excited state and inherent conformational flexibility of the emitters. Narrowband MR-TADF emitters (see Section 11) hold promise as candidates to address this design flaw. Furthermore, most reported green-emitting D-A and MR-TADF OLEDs still suffer from a too severe efficiency roll-off. Therefore, ongoing efforts are thus still necessary towards simultaneously reducing the efficiency roll-off while maintaining high device efficiencies, which will eventually pave the way for highly stable and efficient green-emitting OLEDs.

Emitters with smaller Δ*E*_ST_ and strong SOC are indispensable for facilitating a rapid RISC rate needed to alleviate TTA and STA processes that occur in the device. New OLED fabrication strategies are also promising. For example, hyperfluorescence OLEDs (see Section 17) decouple exciton harvesting from emission by employing separate materials. Rapid FRET from the assistant dopant to the terminal emitter in the device effectively reduces the triplet exciton population and thus minimizes the chance of multiexcitonic quenching. Beyond advancements in the design of the green emitters themselves, we also suggest that much of these future gains will be achieved through the development of new transporting materials and host materials for better charge balance and optimal pairing with TADF emitter.

## 5 Red and NIR TADF Emitters λ_EL_ > 580 nm

Compared to blue (Section 3) and green (Section 4) emitters, red emitters represent an underdeveloped area of TADF research owing to fundamental difficulties in engineering high Φ_PL_ in the red color region. This is primarily a consequence of the energy gap law, which states that as the energy gap (E_g_) decreases between the excited and ground states, the density of vibronic states in both the ground and excited states will increase.^481^ Such an increased density of states (and smaller energy gaps between the S_1_ and S_0_ sublevels) leads to increased internal conversion rates for S_1_ to S_0_, and accelerated non-radiative decay. Furthermore, the rate of radiative decay is proportional to the cube of the frequency of the transition, with a decreased S_1_–S_0_ energy gap therefore leading to a decrease in *k*_r_. Thus it becomes fundamentally more difficult to engineer high Φ_PL_ in materials that emit at longer wavelengths, and particularly so for deep red (DR) and near-infrared (NIR) emitting materials. As a separate but additional factor, the low-energy S_1_ states associated with red emission typically require significantly expanded π-conjugation systems, making π–π stacking interactions more likely and resulting in significant aggregation-caused quenching (ACQ) for red emitters.^482,483^ As a result, efforts to design efficient red TADF materials (and red emitters in general) have not progressed as rapidly as for blue and green counterparts.

Following the design rules discussed in previous sections, red TADF emitters typically incorporate strong electron donors (D) and acceptors (A) linked in a strongly twisted D-A geometry. This choice of molecular fragments affords a shallow HOMO for D and a deep LUMO for A, which together induces a narrow bandgap and therefore a low ^1^CT emission energy.^484^ Examples of chemical structures used as acceptors sorted by their acceptor strength (informed by experimentally inferred LUMO energies) and by the extent of π-conjugation are shown in Figure 59. To help suppress non-radiative decay pathways, rigid and/or planar fused donors or acceptors are favored, resulting in simultaneously higher Φ_PL_ and a narrowing of the emission spectrum.^485^ This can in turn increase the Φ_PL_ of these emitters and ultimately device EQE_max_, reaching above 30% for some vacuum-deposited OLEDs despite intrinsic challenges for this color.^486^ However, increased the planarity of the emitter often increases the likelihood of π–π stacking, worsening aggregation and potentially leading to increased ACQ. Therefore, rationally controlling molecular packing with appropriate intra- and inter-molecular interactions is important for the control of TADF-activity, Φ_PL_, and effective carrier transport.^487^

**Figure 59:**
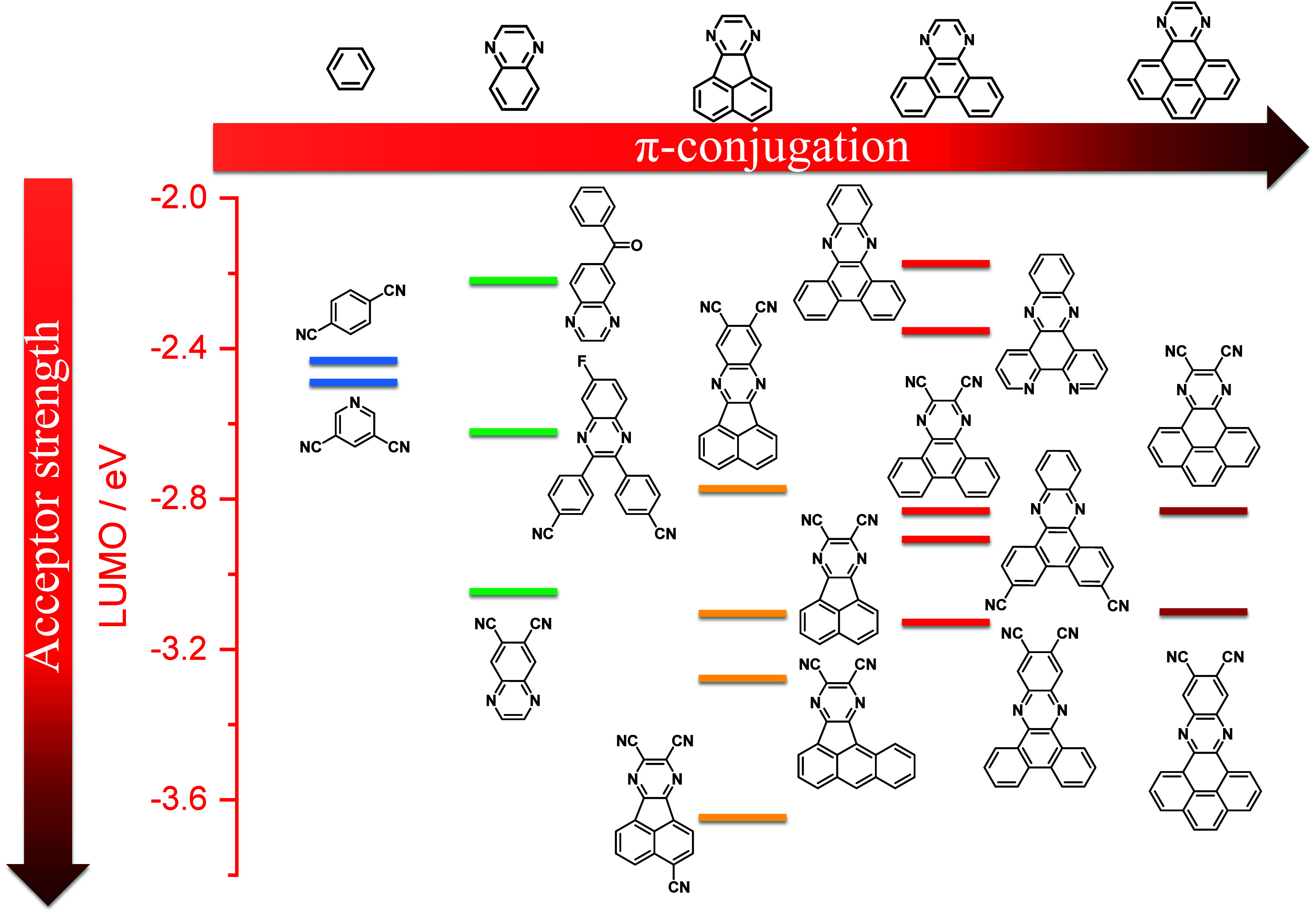
Acceptor motifs commonly used in the design of red and NIR TADF emitters, ordered according to the acceptor strength and π-conjugation length. The LUMO values were calculated at the PBE0/6-31G(d,p) level in the gas phase.

Here we discuss some of the best red and NIR emitters reported between 2017–2022, and summarize their reported photophysical and device performances in Table S3. Despite fewer reports than for the other colors, substantial progress has been made to increase the red OLED efficiencies.^488,489^ Red OLEDs using MR-TADF emitters can also reach very high EQE_max_ and are covered in Section 11. For the purpose of this review, we focus only on red/NIR emitters where the device emits at λ_EL_ > 580 nm and/or has an EQE_max_ greater than 9%.

### 5.1 Pyridine-3,5-dicarbonitrile Acceptors

Prior to the timeline of this review, the first red TADF emitter, 1,4-dicy­ano-2,3,5,6-tetra­kis­(3,6-di­phen­yl­carba­zol-9-yl)­ben­zene (**4CzTPN-Ph**), was reported by Adachi and co-workers group in 2012 (Figure 60).^31^ Composed of a strongly electron-deficient terphthalonitrile acceptor unit and four carbazole derivative donors, **4CzTPN-Ph** has a small τ_d_ of 1.1 μs and a Φ_PL_ of 26.3% in toluene, emitting at λ_PL_ = 577 nm. The device showed an EQE_max_ of 11.2% at λ_EL_ ≈ 580 nm, corresponding to CIE coordinates of (0.52, 0.45) and had efficiency roll-off of ∼20% at 100 cd m^–2^ and ∼70% at 1000 cd m^–2^.

**Figure 60:**
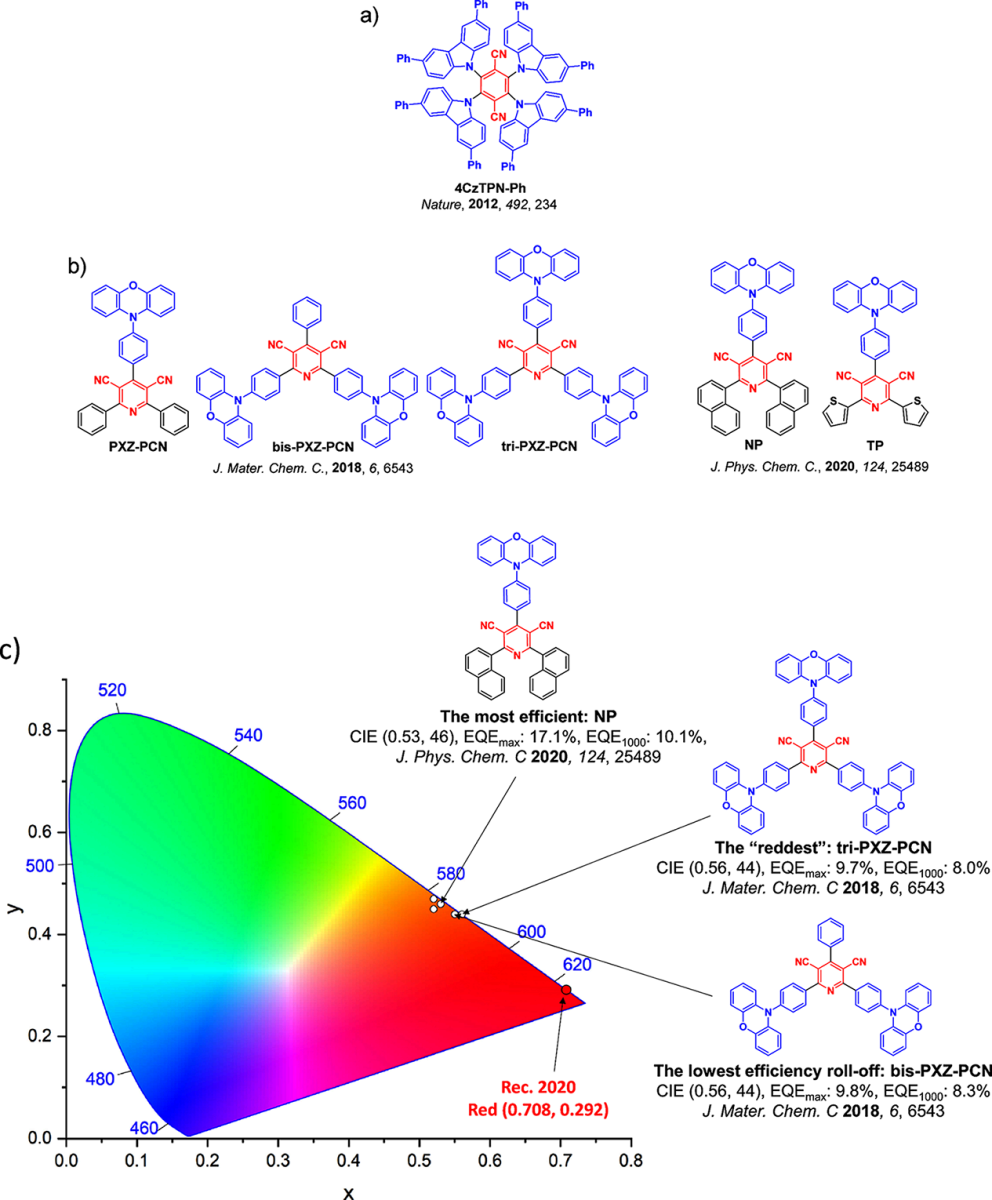
a) Molecular structures of red D-A TADF emitters containing terephthalonitrile acceptors, b) molecular structures of red D-A TADF emitters containing pyridine-3,5-dicarbonitrile acceptors, and c) CIE color coordinate of the most efficient emitter based on a pyridine-3,5-dicarbonitrile acceptor. The white circles illustrate the spread of the emission color of the device. Selected devices and their associated CIE coordinates are highlighted, illustrating the structure of the emitter of the “reddest” device and the structure of the emitter used in the device showing the highest efficiency and the lowest efficiency roll-off. Only D-A TADF OLEDs where the λ_EL_ > 580 nm that are high performing are included. The most efficient device is quantified by the highest EQE_max_. The device with the CIE coordinates closest to the Rec. 2020 defined coordinates for red, (0.708, 0.292), is defined as the “reddest”. In the chemical structures, the blue color signifies donor moieties, while the red color signifies acceptor moieties.

In 2018, Chen *et al*. reported three emitters **PXZ-PCN**, **bis-PXZ-PCN**, and **tri-PXZ-PCN** (Figure 60) that contain one to three PXZ donors with a pyridine-3,5-dicarbonitrile (PCN) acceptor.^444^ The emission of **bis**- and **tri-PXZ-PCN** peaks narrowly between λ_PL_ of 601 and 606 nm in 10 wt% doped CBP films while **PXZ-PCN** emits at 565 nm. **Bis-PXZ-PCN** and **tri-PXZ-PCN** have low Φ_PL_ of 36 and 34%, yet short τ_d_ of 1.40 and 1.48 μs and fast *k*_RISC_ of 9.8 and 8.8 × 10^5^ s^–1^, all respectively (Table S3). Whilst the devices with **bis-PXZ-PCN** and **tri-PXZ-PCN** showed EQE_max_ of only 9.8 (λ_EL_ = 600 nm) and 9.7% (λ_EL_ = 608 nm), their EQE_1000_ remained at 8.3 and 8.0%, representing a low efficiency roll-off. Liu *et al.* subsequently reported red-emitters **NP** and **TP** (Figure 60), with the same PCN acceptor substituted with either naphthyl or thienyl donor groups.^490^ **NP** and **TP** emit at 622 and 619 nm in toluene, and at 560 and 555 nm in 10 wt% doped CBP films. They have Φ_PL_ of 50 and 40% and Δ*E*_ST_ of 0.14 and 0.15 eV, and very short τ_d_ of 0.65 and 0.80 μs in the same films, all respectively. The devices with **TP** showed an EQE_max_ of 12.4% with λ_EL_ at 591 nm while **NP** showed an EQE_max_ of 17.1% with λ_EL_ at 590 nm.

### 5.2 Quinoxaline Acceptors

Quinoxalines are another example of strong electron-acceptor that have been used in red TADF emitter design. Li *et al*. reported an asymmetric D-A emitter, **TPA-QCN** (Figure 61)^491^ for which varying the concentration in doped **TPA-QCN**:​TPBi films from 15 to 30 wt% shifted the λ_PL_ from 649 to 700 nm, with the neat film emitting at λ_PL_ = 733 nm. The Φ_PL_ remained high in the doped films (47–70%), although dropped considerably in the neat film (Φ_PL_ = 21%). **TPA-QCN** has a rather large Δ*E*_ST_ of 0.23 eV in toluene at 77 K and a long τ_d_ of 943 μs. The OLEDs showed an EQE_max_ of 14.5% at 644 nm (15 wt% doped in TPBi), however this was accompanied by a large efficiency roll-off of ∼72% at 100 cd m^–2^ (Table S3). A much lower EQE_max_ of 3.9% was obtained for a non-doped device with NIR emission (λ_EL_ = 728 nm).

**Figure 61:**
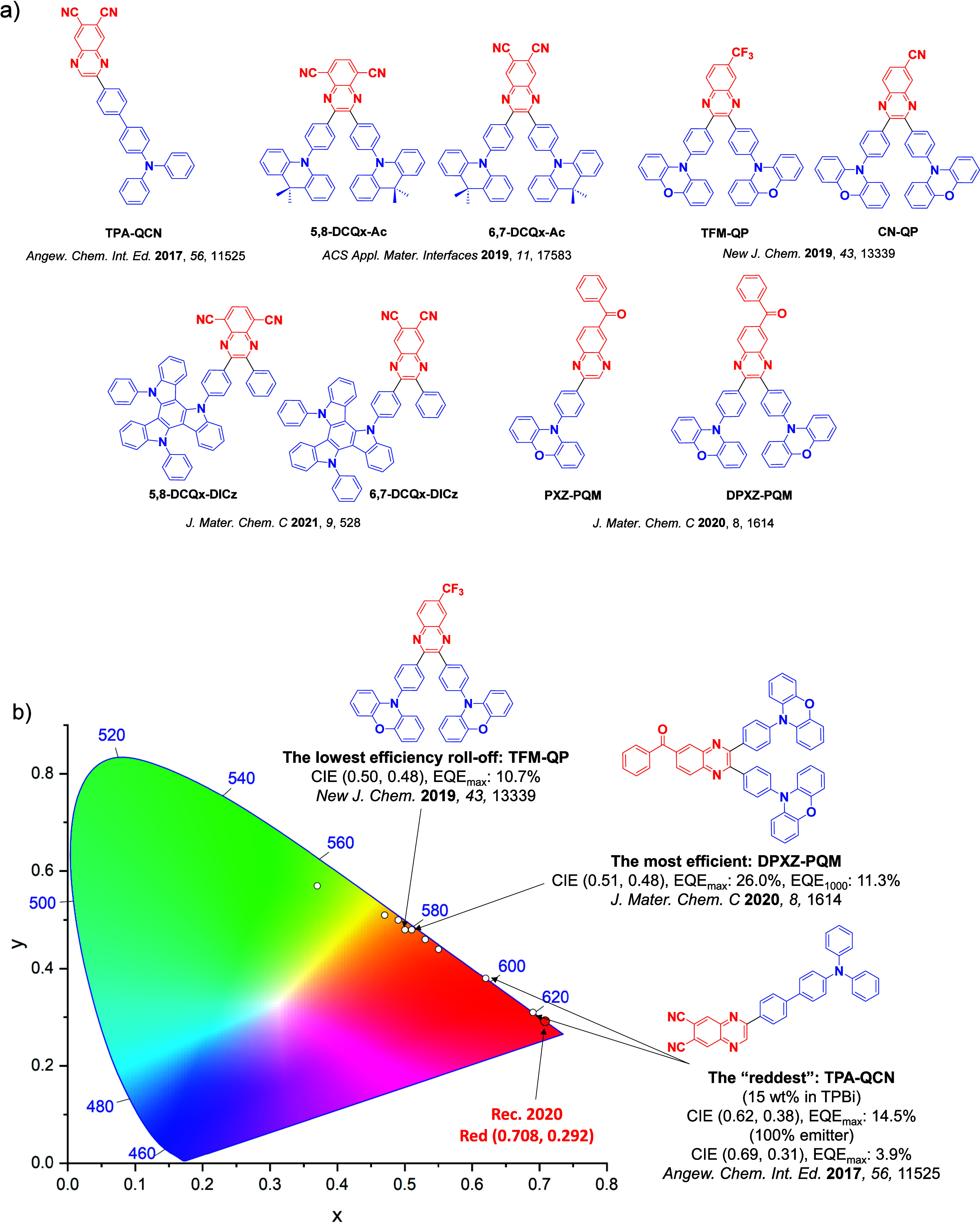
a) Molecular structures of red D-A TADF emitters containing quinoxaline acceptors and b) CIE color coordinates of red D-A TADF emitters containing quinoxaline acceptors. The white circles illustrate the spread of the emission color of the device. Selected devices and their associated CIE coordinates are highlighted, illustrating the structure of the emitter of the “reddest” device and the structure of the emitter used in the device showing the highest efficiency and the lowest efficiency roll-off. Only D-A TADF OLEDs where the λ_EL_ > 580 nm that are high performing are included. The most efficient device is quantified by the highest EQE_max_. The device with the CIE coordinates closest to the Rec. 2020 defined coordinates for red, (0.708, 0.292), is defined as the “reddest”. In the chemical structures, the blue color signifies donor moieties, while the red color signifies acceptor moieties.

Using a bis-cyano substituted quinoxaline substituted with two DMAC donors, Kothavale *et al*. reported the emitters **5,8-DCQx-Ac** and **6,7-DCQx-Ac** (Figure 61).^492^ **5,8-DCQx-Ac** displayed a much deeper LUMO, leading to a red-shift in emission from 620 nm (**6,7-DCQx-Ac**) to 663 nm (**5,8-DCQx-Ac**) in toluene. **5,8-DCQx-Ac** has a moderate Φ_PL_ of 72%, a Δ*E*_ST_ of 0.11 eV, and short τ_d_ of 3.12 μs (Table S3). The OLEDs with this emitter doped in bipolar host (1 wt% in mCP-PFP) showed an EQE_max_ of 16.4% at λ_EL_ of 602 nm and CIE coordinates of (0.55, 0.44). Despite the **6,7-DCQx-Ac** device showing an EQE_max_ of 21.1%, the λ_EL_ was 578 nm. In a subsequent paper by the same group, analogous emitters **6,7-DCQx-DICz** and **5,8-DCQx-DICz** (Figure 61) were reported with a triazatruxene donor in lieu of an acridan.^493^ Changing the position of the two cyano groups from the *ortho* to the *meta* positions with respect to the pyrazine ring resulted in a significant increase in Φ_PL_ from 40 (**5,8-DCQx-DICz**) to 73% (**6,7-DCNQx-DICz**). However, this improvement is accompanied by a blue-shift of almost 50 nm in the emission λ_PL_, from 651 (**5,8-DCQx-DICz**) to 603 nm (**6,7,DCQx-DICz**). The device with **6,7-DCQx-DICz** (1 wt% doped in PBICT) showed a higher EQE_max_ of 23.9% at λ_EL_ of 578 nm than the device with **6,7-DCQx-Ac** at 21.1% at λ_EL_ of 578 nm. The **5,8-DCQx-DICz** device, on the other hand, showed an EQE_max_ of 12.5% at λ_EL_ of 603 nm.

The strong donor phenoxazine was combined with similar acceptors 6-(trifluoromethyl)quinoxaline or 6-(cyano)quinoxaline to form compounds **TFM-QP** and **CN-QP** (Figure 61).^494^ **TFM-QP** and **CN-QP** emit at λ_PL_ of 613 and 611 nm in toluene, and both have Φ_PL_ of 61% in 5 wt% doped CBP films (Table S3). The compounds showed delayed fluorescence with rather long τ_d_ of 5.0 ms (**TFM-QP**) and 1.6 ms (**CN-QP**) despite their small Δ*E*_ST_ of 0.04 and 0.03 eV, all respectively. The yellow OLEDs fabricated with **TFM-QP** and **CN-QP** exhibited EQE_max_ of 14.1 and 9.7%, both with λ_EL_ of 584 nm, illustrating that the red emission achieved in solution measurements is not always straightforward to translate into devices.

**PXZ-PQM** and **DPXZ-PQM** (Figure 61) similarly combine benzoyl and quinoxaline units, with differing numbers of phenoxazine donor units.^495^ The PL spectra of **PXZ-PQM** (Δ*E*_ST_ of 0.03 eV) and **DPXZ-PQM** (Δ*E*_ST_ of 0.02 eV) in 5 wt% doped films in DCzDPy gave broad orange-to-red emission at λ_PL_ of 588 and 586 nm, demonstrating little impact of the number of donor groups in this case. The device with **DPXZ-PQM** exhibited the best EL performance with an EQE_max_ of 26.0%, and orange-red emission at λ_EL_ of 590 nm corresponding to CIE coordinates of (0.51, 0.48) (Table S3). This performance was attributed to the high Φ_PL_ (88%), relatively small Δ*E*_ST_ (0.02 eV), and fast RISC rate (*k*_RISC_ = 2.05 × 10^5^ s^–1^). The OLED with **PXZ-PQM** showed a somewhat lower EQE_max_ of 20.4%, attributed to the Φ_PL_ of 70% of the emitter. The efficiency roll-off at 100 and 1000 cd m^–2^ were 14 and 45% for the **PXZ-PQM**-based device, and 23 and 47% for the **DPXZ-PQM**-based device.

### 5.3 Acenaphtho[1,2-b]pyrazine Acceptors

Acenaphtho[1,2-b]pyrazine-8,9-dicarbonitrile (APDC), a stronger acceptor than quinoxaline, was used in conjunction with two TPA donors to produce the deep-red emitter **APDC-DTPA** (Figure 62).^496^ Doped at 10 wt% in TPBi, **APDC-DTPA** emits at λ_PL_ of 687 nm with a Φ_PL_ of 63% and a Δ*E*_ST_ of 0.14 eV (Table S3). As neat films the emission is red-shifted to λ_PL_ = 756 nm, which is accompanied by a drop in Φ_PL_ to 17% due to ACQ. The OLEDs with **APDC-DTPA** showed an EQE_max_ of 10.2% at λ_EL_ of 693 nm. Non-doped OLEDs produced NIR λ_EL_ of 777 nm although with a lower EQE_max_ of 2.2%. While both devices represent some of the deepest red TADF OLEDs reported to date, they also suffer from severe efficiency roll-off, with the EQE_100_ for the doped device being ∼0.8%, while the non-doped device did not reach this level of luminance. This low efficiency was attributed to triplet–triplet or singlet–triplet annihilation arising from the relatively long triplet lifetime, along with low Φ_PL_ in the solid state.^497^

**Figure 62:**
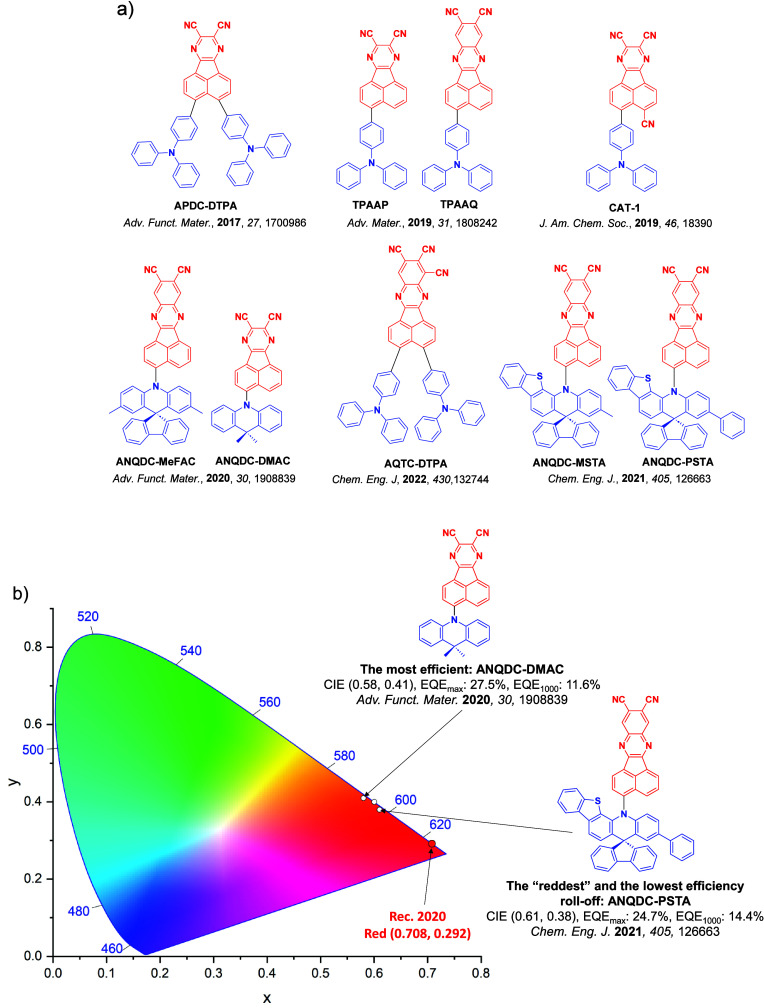
a) Molecular structures of red D-A TADF emitters containing acenaphtho[1,2-b]pyrazine acceptors and b) CIE color coordinates of red D-A TADF emitters containing acenaphtho[1,2-b]pyrazine acceptors. The white circles illustrate the spread of the emission color of the device. Selected devices and their associated CIE coordinates are highlighted, illustrating the structure of the emitter of the “reddest” device and the structure of the emitter used in the device showing the highest efficiency and the lowest efficiency roll-off. Only D-A TADF OLEDs where the λ_EL_ > 580 nm that are high performing are included. The most efficient device is quantified by the highest EQE_max_. The device with the CIE coordinates closest to the Rec. 2020 defined coordinates for red, (0.708, 0.292), is defined as the “reddest”. In the chemical structures, the blue color signifies donor moieties, while the red color signifies acceptor moieties.

Xue *et al*. reported two D-A TADF emitters **TPAAP** and **TPAAQ** (Figure 62), containing the strong electron-drawing acceptors ace­naph­tho­[1,2-b]py­ra­zine-8,9-di­car­bo­nitrile (AP) and ace­naph­tho­[1,2-b]quin­oxa­line-8,9-di­car­bo­nitrile (AQ).^498^ Compared to **APDC-DTPA** both compounds contain only one TPA moiety. In toluene **TPAAP** emits at λ_PL_ of 609 nm, has a high Φ_PL_ of 97% and a Δ*E*_ST_ of 0.19 eV, while **TPAAQ** has Φ_PL_ of 93% but with a larger Δ*E*_ST_ of 0.33 eV (Table S3). Notably, both molecules exhibited significantly red-shifted PL spectra in their aggregated forms, falling into the NIR region with λ_PL_ at 777 nm for **TPAAP** and 716 nm for **TPAAQ** due to the formation of J-aggregates that possess strong intermolecular CT excited states. OLEDs with 5 or 10 wt% **TPAAP** in TPBi showed EQE_max_ of 15.8 and 14.1% with λ_EL_ of 630 and 657 nm, all respectively. The non-doped devices with **TPAAQ** (Φ_PL_ = 16.3%) and **TPAAP** (Φ_PL_ = 20.3%) exhibited NIR emission with EQE_max_ of 3.5% at 711 nm and 5.1% at 765 nm, respectively.

Congrave *et al*. reported a NIR TADF emitter, **CAT-1** (3-tri­phen­yl­amine-4-cy­ano-ace­naph­tho­[1,2-b]-py­ra­zine-8,9-di­car­bo­nitrile), which is structurally similar to **TPAAP**. **CAT-1** incorporates triphenylamine as the donor and ace­naph­tho­[1,2-b]-py­ra­zine as the acceptor, enabling NIR TADF emission (Figure 62).^20^ Compared to **APDC-DTPA** (neat, λ_PL_ = 756 nm), **CAT-1** in 10 wt% doped CBP films has a red-shifted emission (λ_PL_ = 763 nm) at modest Φ_PL_ of 8.8% and a rather long *τ*_d_ of 80 μs considering its Δ*E*_ST_ of ca. 0.04 eV (Table S3). Increasing the doping ratio of **CAT-1** in the evaporated films led to significant red-shift of the emission and a corresponding decrease in Φ_PL_; for example, the 40 wt% doped CBP film emission was recorded at λ_PL_ = 820 nm with low Φ_PL_ of 2%. Evaporated neat films of **CAT-1** emit at λ_PL_ of 887 nm, while neat films dropcasted from chlorobenzene solution were further red-shifted to λ_PL_ = 950 nm. The non-doped OLEDs showed an EQE_max_ of 0.019% at an λ_EL_ at 904 nm. Computational studies of this material later revealed the potential for intramolecular hydrogen bonding (CH-CN) between the TPA donor and CN acceptor group as assisting in the overall performance compared to **TPAAP**.^499^

Gong *et al.* reported the emitters **ANQDC-DMAC** and **ANQDC-MeFAC** (Figure 62), using the same acceptor as **TPAAQ** but coupled with either DMAC or MeFAC as the donor unit.^500^ This combination of donor and acceptor resulted in λ_PL_ at 596 and 604 nm in 1.5 wt% CBP:​TPBi co-host and high Φ_PL_ of 95 and 77%, along with a small Δ*E*_ST_ values of 0.06 and 0.05 eV, all respectively (Table S3). Both compounds displayed preferential horizontal TD alignments of ∼80%, attributed to the linear and planar acceptor motif and rod-like molecular configuration. The OLEDs with **ANQDC-DMAC** and **ANQDC-MeFAC** achieved EQE_max_ of 27.5 and 26.3% at λ_EL_ at 615 and 614 nm respectively, corresponding to CIE coordinates of (0.58, 0.41) and (0.60, 0.40).

Cheng *et al*. reported a NIR TADF emitter containing an auxiliary electron-withdrawing group attached to the AQ acceptor, **AQTC-DTPA** (Figure 62).^501^ **AQTC-DTPA** in 10 wt% doped films in CBP emits at λ_PL_ of 718 nm and has a Φ_PL_ of 19.1%, while in neat films this compound emits at λ_PL_ of 878 nm and has a Φ_PL_ of 1.1%. A large red-shift (82 nm) was observed in the emission spectrum of **AQTC-DTPA** in the 10 wt% doped film relative in toluene (636 nm). The EL spectra also showed a significant bathochromic shift from 694 to 894 nm when the doping ratio increased in CBP film from 10 to 100 wt% (neat). The red-shifting of the emission was attributed to strong intermolecular interactions in the emissive layer, growing in strength as the doping concentration increased. The large and coplanar AQ unit in **AQTC-DTPA** favoured π-π stacking interactions in thin films, which was supported by single-crystal X-ray analysis. The solid-state structure of amorphous **AQTC-DTPA** obtained by cluster analysis indeed showed a tight packing pattern, aggregated in a head-to-head mode with π-π distances below 3.6 Å. A device with **AQTC-DTPA** (10 wt% doped in CBP) showed an EQE_max_ of 9.3% at λ_EL_ of 694 nm, with the EQE_max_ decreasing significantly to 0.51/0.41/0.30/0.23% as the doping concentration increased from 60/70/80/100 wt% at λ_EL_ of 810/828/852/894 nm, all respectively (Table S3).

Recently, Gong *et al*. reported red emitters **ANQDC-MSTA** and **ANQDC-PSTA** (Figure 62) using rigid, linear, and planar ANQDC as the acceptor coupled with thienocarbazole-fused acridine donors MSTA and PSTA.^502^ The compounds emit at λ_PL_ of 623 and 618 nm and have Φ_PL_ of 65 and 72% respectively, in 1.5 wt% doped films in a 1:1 CBP:​TPBi co-host (Table S3). The **ANQDC-MSTA**-based OLED (1.5 wt% emitter doped in CBP:​TPBi co-host) exhibited an EQE_max_ of 21.8%, while the **ANQDC-PSTA**-based OLEDs displaying slightly higher EQE_max_ of 24.7%. Both OLEDs displayed λ_EL_ of 622 nm and CIE coordinates of (0.61, 0.38).

### 5.4 Pyrazino- or Quinoxalino-Expanded Phenanthrene Acceptors

Pyrazine-fused phenanthrene is a large and rigid π-conjugated structure that has been widely used as an acceptor in red TADF emitters. For example, Wang *et al*. employed dicyano-substituted pyrazino-phen­an­threne (DCPP) as the acceptor and DPA or DMAC as the donor in a series red and deep-red TADF emitters.^503^ DMAC derivatives **DMAC-DCPP** and **DMAC-Ph-DCPP** (Figure 63) emit at 618 and 594 nm, have Φ_PL_ of 33 and 65%, small Δ*E*_ST_ of 0.08 and 0.05 eV, and short τ_d_ of 2.4 and 3.2 μs, all respectively (Table S3). The OLEDs with **DMAC-DCPP** and **DMAC-Ph-DCPP** showed EQE_max_ of 10.1 and 16.5%, with CIE coordinates of (0.60, 0.40) and (0.53, 0.46), respectively. However, large efficiency roll-off were observed with EQE_500_ of just 4.2% for the device with **DMAC-DCPP** and 6.3% for the device with **DMAC-Ph-DCPP**. Replacing DMAC with DPA resulted in red-shifted emission at λ_PL_ = 606 and 628 nm for **DPA-DCPP** and **DPA-Ph-DCPP**, respectively, along with Φ_PL_ of 64 and 65%. The devices with these two emitters also experienced severe efficiency roll-off (EQE_max_ of 10.4 and 15.1%, EQE_500_ of 0.9 and 1.6%), due to their larger Δ*E*_ST_ (0.28 and 0.10 eV) and much longer delayed lifetimes (τ_d_ = 579 and 82 μs).

**Figure 63:**
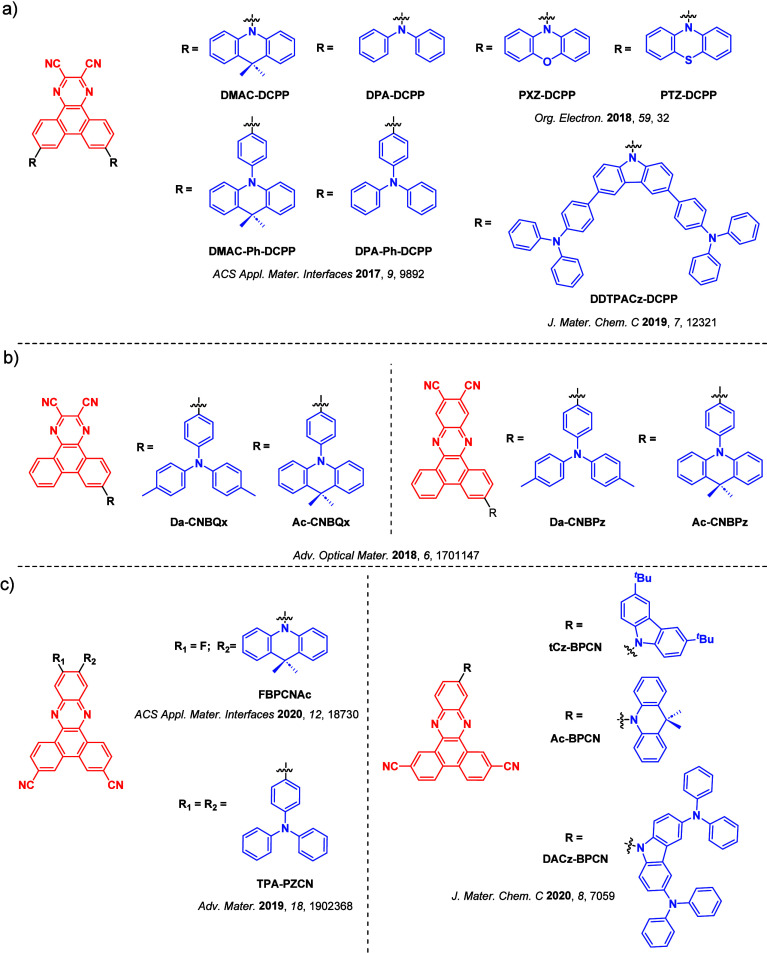
Structures of red TADF emitters using a) di­ben­zo[f,h]quin­oxa­line-2,3-di­car­bo­nitrile acceptor with two donor groups, b) di­ben­zo[f,h]quin­oxa­line-2,3-di­car­bo­nitrile acceptor with one donor group, and c) di­ben­zo[a,c]phen­azine-3,6-di­car­bo­nitrile acceptor (the blue color signifies donor moieties, while the red color signifies acceptor moieties).

The same DCPP acceptor unit was also reported by Wang *et al.* coupled with stronger phenoxazine and phenothiazine donors (Figure 63).^504^ In toluene, **PXZ-DCPP** and **PTZ-DCPP** emit at 564 and 580 nm, have Φ_PL_ of 11.9 and 17.4% and Δ*E*_ST_ of 0.09 and 0.18 eV, respectively (Table S3). The devices with **PXZ-DCPP** and **PTZ-DCPP** showed EQE_max_ of 17.4 and 12.3% at λ_EL_ of 608 and 640 nm with associated CIE coordinates of (0.56, 0.43) and (0.62, 0.36), all respectively. Importantly, these devices exhibited only modest efficiency roll-off with the EQE_1000_ of 12.9% and 6.1%. A similar red emitter with carbazole donors that are additionally decorated with two TPA units was also reported by Wang *et al*..^505^ **DDTPACz-DCPP** (Figure 63) has a Φ_PL_ of 53% and emits at λ_PL_ of 663 nm in 10 wt% doped films in CBP, along with having a Δ*E*_ST_ of 0.16 eV and a τ_d_ of 9.7 μs. The solution-processed devices with **DDTPACz-DCPP** showed an EQE_max_ of 13.6% at λ_EL_ of 646 nm and CIE coordinates of (0.63, 0.37).

Furue *et al*. reported two asymmetric D−π–A emitters **Da-CNBPz** and **Ac-CNBPz** (Figure 63), consisting of 11,12-dicyanodibenzo[a,c]phenazine (CNBPz) as a strong acceptor unit.^506^ These were compared with D−π–A TADF analogues **Da-CNBQx** and **Ac-CNBQx**, containing 2,3-di­cy­ano­di­ben­zo­[f,h]quin­oxa­line (CNBQx) as a less π-conjugated and weaker acceptor unit (Figure 63). Comparing **Da-CNBQx** and **Da-CNBPz**, the emission red-shifted from 633 to 688 nm and there is a modest decrease in Φ_PL_ from 85 to 72% (Table S3). The same behavior was observed for **Ac-CNBQx** and **Ac-CNBPz**, with λ_PL_ red-shifted from 561 to 615 nm and Φ_PL_ decreasing from 75 to 67%, respectively. Calculated non-radiative rate constants (*k*_nr_) for the emitters showed an increase in non-radiative decay upon extending the π-conjugation of the acceptor, with values of 1.6 × 10^7^ s^–1^ for **Da-CNBQx** and 2.4 × 10^7^ s^–1^ for **Da-CNBPz**; the same trends were observed for **Ac-CNBQx** and **Ac-CNBPz**, where an increase of non-radiative decay from 0.16 to 0.25 × 10^7^ s^–1^ was seen, in line with the energy gap law. Devices with **Da-CNBQx** and **Da-CNBPz** showed EQE_max_ of 15.0% at λ_EL_ of 670 nm and 20.0% at λ_EL_ of 617 nm, respectively, representing some of the highest efficiency red TADF OLEDs to date. However, both of these devices suffered severe efficiency roll-off with EQE_100_ dropping to 3.8 and 7.5%, respectively. Although the devices with **Ac-CNBQx** and **Ac-CNBPz** show lower EQE_max_ (and blue-shifted emission) of 16.2% (λ_EL_ = 630 nm) and 14.0% (λ_EL_ of 685 nm), their EQE_100_ were superior at 14.5 and 13.9%, all respectively. This change is due to smaller Δ*E*_ST_ values when using Ac as the donor of 0.03 and 0.10 eV for **Ac-CNBPz** and **Ac-CNBQx** compared to 0.11 and 0.18 eV for **Da-CNBPz** and **Da-CNBQx**, respectively, all in 6 wt% doped films in CBP.

In a similar approach to the previous example, cyano groups were added to the 3- and 6- positions of a phenazine core to increase the electron-accepting strength, while two TPA groups were employed as the donors.^507^ **TPA–PZCN** (Figure 63) emits at 610 nm and has a very high Φ_PL_ of 97%, a Δ*E*_ST_ of 0.13 eV, and a τ_d_ of 133 μs. The devices with **TPA–PZCN** showed an EQE_max_ of 27.4% at λ_EL_ at 628 nm and CIE coordinates of (0.65, 0.35), which represents the best result with a peak wavelength longer than 600 nm among the reported red TADF devices. In a subsequent report, Kothavale *et al*. functionalized the same acceptor with a fluorine atom and used DMAC as the donor in **FBPCNAc** (Figure 63).^434^ The fluorine substituent was attached *ortho* to the DMAC, which strengthened the electron-acceptor. **FBPCNAc** emits at 607 nm, has a Φ_PL_ of 79%, a small Δ*E*_ST_ of 0.05 eV, and a τ_d_ of 11.1 μs. The OLEDs with **FBPCNAc** realized an EQE_max_ of 23.8% at λ_EL_ of 597 nm and CIE coordinates of (0.55, 0.44). This blue-shift of the emission compared to the previous examples is likely due to there being only one donor unit in this emitter design compared to two for the others. Kothavale *et al*. also reported two related emitters, **Ac-BPCN** and **DACz-BPCN** (Figure 63), which differ in the substitution position of the CN group on the BPCN acceptor unit.^508^ **Ac-BPCN** and **DACz-BPCN** emit at 618 and 654 nm, have Φ_PL_ of 66 and 47%, Δ*E*_ST_ of 0.13 and 0.07 eV, and τ_d_ of 11.1 and 7.2 μs, all respectively. The OLEDs with **Ac-BPCN** and **DACz-BPCN** in the bipolar host PBICT showed EQE_max_ of 20.7% (λ_EL_ = 597 nm) and 11% (λ_EL_ = 631 nm) at CIE coordinates of (0.54, 0.45) and (0.60, 0.39), respectively.

Moving away from CN-substituted π-conjugated acceptors, Xie *et al*. developed three TADF molecules **xDMAC-BP** (**x** = 1, 2, 3) containing a rigid planar phenazine acceptor core and different numbers of DMAC donors at the 3-/6-/11-positions (Figure 64).^509^ The emission color of the **xDMAC-BP** series could be tuned from green to orange-red by changing the number of the DMAC units. The reddest emitting analogue **3DMAC-BP** emits at λ_PL_ of 590 nm, has a high Φ_PL_ of 89%, a small Δ*E*_ST_ of 0.05 eV, and a short τ_d_ of 2.9 μs in 20 wt% doped films in mCBP. The OLEDs with **3DMAC-BP** showed an EQE_max_ of 22.0% at λ_EL_ of 606 nm. Crucially, the EQE_100_ of the **3DMAC-BP**-based device remained as high as 17.5%. The same molecular design was also employed using PXZ as the donor.^510^ Expectedly, increasing the number of PXZ units red-shifted the emission from 602 to 682 nm in toluene. The Δ*E*_ST_ and Φ_PL_ values for **1PXZ-BP** are 0.25 eV and 73%, for **2PXZ-BP** are 0.10 eV and 63%, and for **3PXZ-BP** are 0.03 eV and Φ_PL_ = 22%. Thus, as the number of PXZ groups increases the Φ_PL_ decreases as does Δ*E*_ST_ and also τ_d_ from 4.8 to 4.3 μs and 2.0 μs, respectively. The orange-red OLEDs with **1PXZ-BP**, **2PXZ-BP**, and **3PXZ-BP** showed EQE_max_ of 26.3% (λ_EL_ of 590 nm), 19.2% (λ_EL_ of 606 nm) and 7.1% (λ_EL_ of 634 nm). However, compared to **xDMAC**–**BP** the efficiency roll-off of the **xPXZ-BP** series are all higher, which the authors speculated may be due to the inferior charge balance of the devices.

**Figure 64:**
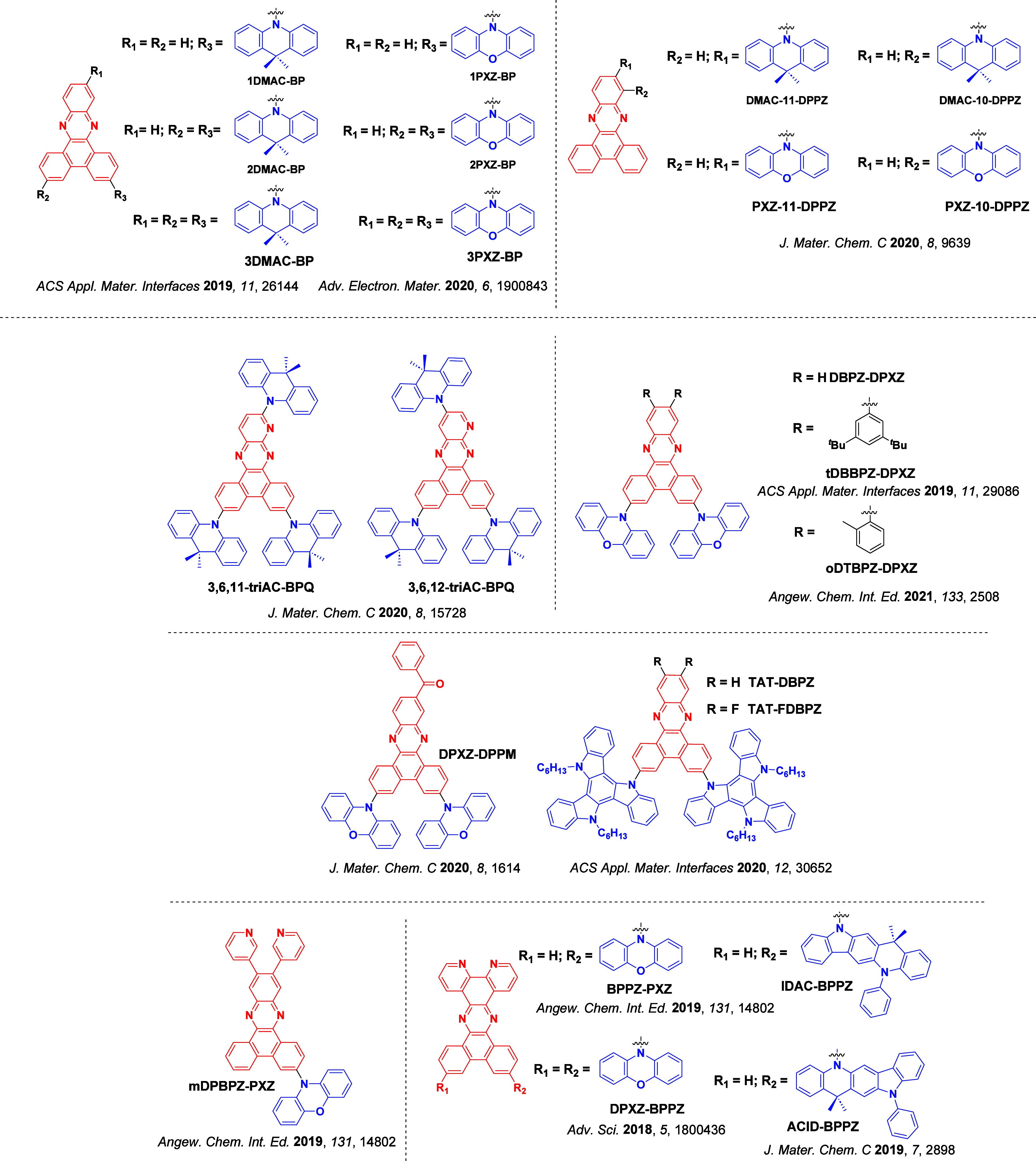
Molecular structures of red TADF emitters featuring pyrazino-phenanthrene acceptors. The blue color signifies donor moieties, while the red color signifies acceptor moieties.

Xie *et al*. used a similar dibenzo[*f,h*]pyrido[*2,3*-b]quinoxaline (BPQ) acceptor coupled to three DMAC donors at either the 3-,6-,11-positions or 3-,6-,12-positions in **3,6,11-triAC-BPQ** and **3,6,12-triAC-BPQ** (Figure 64).^511^ In 15 wt% doped films in mCBP **3,6,11-triAC-BPQ** and **3,6,12-triAC-BPQ** emit at λ_PL_ of 516 and 611 nm in toluene, have Φ_PL_ of 75 and 53%, and τ_d_ of 2.50 and 2.25 μs, all respectively. **3,6,11-triAC-BPQ** was claimed to have HLCT character due to intramolecular hydrogen bonding between the isolated donor and the adjacent pyridine nitrogen, whilst the **3,6,12-triAC-BPQ** displayed typical CT character. **3,6,11-triAC-BPQ** and **3,6,12-triAC-BPQ** have Δ*E*_ST_ of 0.10 and 0.03 eV, and the corresponding devices showed EQE_max_ of 22.0% [λ_EL_ of 581 nm, CIE coordinates of (0.51, 0.48)] and 16.5% [λ_EL_ of 616 nm, CIE coordinates of (0.58, 0.39)], all respectively (Table S3).

Zhou *et al.* developed two pairs of emitters **DMAC-11-DPPZ** and **DMAC-10-DPPZ**, and **PXZ-11-DPPZ** and **PXZ-10-DPPZ**, differing only in the nature of the donor (DMAC or PXZ) connected through 10- or 11- positions on the acceptor BP moiety (Figure 64).^512^ The compounds substituted at the 11-position achieved much higher Φ_PL_ (57.4 and 40.9%) than those substituted at the 10-position (28.6 and 5.3%), owing to suppressed non-radiative vibrational modes. The PL spectrum of **DMAC-11-DPPZ** in toluene has two emission peaks, at λ_PL_ of 567 and 490 nm, attributed to the coexistence of quasi-equatorial (QE) and quasi-axial (QA) conformers. **DMAC-10-DPPZ** emits at 620 nm, which is significantly red-shifted owing to the stronger CT state. The emission of **PXZ-11-DPPZ** shows a red emission peak at λ_PL_ of 630 nm, while by contrast is significantly red-shifted compared to **PXZ-10-DPPZ**, which unexpectedly exhibits a blue-shifted emission peak at λ_PL_ of 573 nm in toluene. The τ_d_ for **DMAC-11-DPPZ**, **DMAC-10-DPPZ**, **PXZ-11-DPPZ**, and **PXZ-10-DPPZ** are 1.53, 0.83, 0.72, and 0.51 μs, respectively. These values are in good agreement with the trend in the corresponding Δ*E*_ST_ values of 0.112, 0.075, 0.062, and 0.057 eV, respectively. The **DMAC-11-DPPZ** based device showed orange emission at λ_EL_ of 588 nm [CIE coordinates of (0.53, 0.46)] with an EQE_max_ of 23.8%, while the device with **DMAC-10-DPPZ** showed a much lower EQE_max_ of 8.3%, albeit with a red-shifted λ_EL_ of 624 nm [CIE coordinates of (0.61, 0.38)]. Similarly, the **PXZ-10-DPPZ** device showed a red-shifted emission at λ_EL_ of 655 nm [CIE coordinates of (0.63, 0.37)] yet with a higher EQE_max_ of 8.7% compared to the device with **PXZ-11-DPPZ** at λ_EL_ of 627 nm [CIE coordinates of (0.65, 0.35)] and an EQE_max_ of only 0.8%.

Introduction of two 3,5-di-*tert*-butylphenyl groups in **tDBBPZ-DPXZ** improved the solubility compared to **2PXZ-BP** (Figure 64).^513^ **tDBBPZ-DPXZ** emits at λ_PL_ of 617 nm, has a Φ_PL_ of 83%, and a small Δ*E*_ST_ of 0.03 eV in 10 wt% doped films in CBP. A solution-processed **tDBBPZ-DPXZ** OLED emitted at λ_EL_ of 620 nm and CIE coordinates of (0.62, 0.37), and showed an EQE_max_ of 10.1%. **tDBBPZ-DPXZ** has nearly the same photophysical properties as **2PXZ-BP** (**DBBPZ-DPXZ** in that work). Vacuum-deposited devices with **tDBBPZ-DPXZ** and **DBPZ-DPX** both emitted at λ_EL_ 608 nm, corresponding to CIE coordinates of (0.58,0.42) and (0.57,0.43), and showed EQE_max_ of 17.0 and 17.8%, all respectively. In a subsequent report from the same group, a similar compound **oDTBPZ-DPXZ** containing *o*-tolyl groups instead of *tert*-butylphenyl groups shows comparable photophysics.^514^ **oDTBPZ-DPXZ** emits at 622 nm, has a high Φ_PL_ of 87%, and a small Δ*E*_ST_ of 0.04 eV (Table S3). Solution-processed OLEDs with **oDTBPZ-DPXZ** achieved an EQE_max_ of 18.5% at λ_EL_ of 612 nm and CIE coordinates of (0.60, 0.40).

Liang *et al*. used a weakly electron-withdrawing benzoyl group attached to **2PXZ-BP** to construct the emitter **DPXZ-DPPM** (Figure 64).^495^ **DPXZ-DPPM** doped in 5,5′-bis(carbazol-9-yl)-3,3′-bipyridine (DCzDPy) films emits at λ_PL_ of 630 nm, has a Φ_PL_ of 61%, a ΔE_ST_ of 0.05 eV, and a τ_d_ of 3.53 μs. Compared to **2PXZ-BP** (λ_EL_ of 606 nm), **DPXZ-DPPM**-based devices display a much redder emission at λ_EL_ of 630 nm corresponding to CIE coordinates of (0.61, 0.38), and showed an EQE_max_ of 11.5%. Fan *et al*. reported two similar TADF emitters, **mDPBPZ-PXZ and BPPZ-PXZ**, which have either substituted or annulated pyridyl groups on the acceptor.^515^ In 14 wt% doped CBP films **mDPBPZ-PXZ** emits at λ_PL_ of 638 nm, has a small Δ*E*_ST_ of 0.04 eV, and a high Φ_PL_ of 95%, whereas the neat film has only a moderate Φ_PL_ of 33% with a λ_PL_ of 607 nm (Table S3). This suggests the introduction of pyridine moieties somewhat relieves concentration-induced quenching. The OLEDs with **mDPBPZ-PXZ** in mCP showed an EQE of 21.7% at λ_EL_ of 624 nm and CIE coordinates of (0.62, 0.38). Non-doped devices showed a much lower EQE of 5.2% at λ_EL_ of 680 nm with CIE coordinates of (0.68, 0.32). The fused analogue **BPPZ-PXZ** emits at 607 nm and has a high Φ_PL_ of 100%, Δ*E*_ST_ of 0.03 eV, and a τ_d_ of 3.6 μs.^516^ The OLED doped with **BPPZ-PXZ** showed an EQE_max_ of 25.2% at λ_EL_ at 604 nm, whereas the non-doped device showed a much lower EQE_max_ of only 2.5% at λ_EL_ at 656 nm. This contrast was attributed to more serious concentration quenching due to close molecular packing of this more planar emitter (compared to **mDPBPZ-PZX** with conformationally flexible pyridyl substituents). A disubstituted analogue **DPXZ-BPPZ** was also reported by Chen *et al.* and has similar optoelectronic properties.^517^ The **DPXZ-BPPZ** OLED emitted at λ_EL_ of 612 nm and showed an EQE_max_ of 20.1%, and EQE_100_/EQE_1000_ that remained at ∼19.7/16.7% – an efficiency roll-off that was superior to the device with single-donor material **BPPZ-PXZ**. The superior performance of the devices was in part due to the excellent Φ_PL_ of 97%, the reasonably fast *k*_RISC_ of 2.24 × 10^5^ s^–1^ and suppressed *k*_nr_ of 0.5 × 10^4^ s^–1^, the latter of which was attributed to the rigid nature of the molecule.

Chen *et al*. used the same acceptor in combination with fused donors in **IDAC-BPPZ** and **ACID-BPPZ** (Figure 64).^518^ Similar emission properties were observed for both compounds with λ_PL_ of 583 and 596 nm and Φ_PL_ of 84 and 75%, respectively. **IDAC-BPPZ** and **ACID-BPPZ** have Δ*E*_ST_ of 0.06 and 0.01 eV, and similar τ_d_ of 14 and 12 μs (Table S3). The OLEDs with **IDAC-BPPZ** showed EQE_max_ of 18.3% at λ_EL_ = 580 nm, as compared to only 14.7% for the device with **ACID-BPPZ** at λ_EL_ = 588 nm. A greater efficiency roll-off was observed for the device with **IDAC-BPPZ**, decreasing from maximum values by ∼39 and ∼68% at 1000 cd m^–2^ for the OLEDs with **ACID-BPPZ** and **IDAC-BPPZ**, respectively. This difference was ascribed to the faster τ_d_ alleviating triplet accumulation and quenching processes in **ACID-BPPZ**.

Liu *et al*. developed two red TADF emitters by incorporating triazatruxene (TAT) as the electron donor (Figure 64).^519^ Fluorine-substituted **TAT-FDBPZ** displayed a red-shifted emission (λ_PL_ = 601 nm) compared to that of **TAT-DBPZ** (λ_PL_ = 572 nm) as a result of the electron-withdrawing nature of the two fluorine atoms. The large steric hindrance between TAT and DBPZ was suggested to be responsible for a reduced Δ*E*_ST_ value of 0.16 eV and suppressed ACQ, enabling AIE and high Φ_PL_ in the 20 wt% doped films in CBP of these emitters (Φ_PL_ of 76% for **TAT-DBPZ** and 62% for **TAT-FDBPZ**). **TAT-DBPZ** and **TAT-FDBPZ** indeed have small Δ*E*_ST_ of 0.16 and 0.10 eV and short τ_d_ of 2.30 and 1.51 μs, respectively. Solution-processed OLEDs with **TAT-DBPZ** showed an EQE_max_ of 15.4% at λ_EL_ of 604 nm, while the **TAT-FDBPZ** based OLEDs showed a red-shifted at λ_EL_ of 611 nm and a smaller EQE_max_ of 9.2%. These values were accompanied by very low efficiency roll-off of only 1.0% at 100 cd m^–2^ and 19% at 1000 cd m^–2^.

Rather than installing fused pyridine groups onto phenanthrene, Xu *et al*. developed phenanthroline-based D-A red TADF emitters ***o*TPA-DPPZ** and ***p*TPA-DPPZ** (Figure 65a and 65b).^520^ In a 30 wt% doped DBFDPO (4,6-bis­(di­phen­yl­phos­phor­yl)-di­ben­zo­furan) film, ***o*TPA-DPPZ** emits at λ_PL_ of 605 nm, has a Φ_PL_ of 75%, a Δ*E*_ST_ of 0.07 eV, and a τ_d_ of 12 μs (Table S3). OLEDs with ***o*TPA-DPPZ** showed an EQE_max_ of 18.5% at λ_EL_ of 600 nm. Through adjusting the position of the donor groups, the T-shaped ***p*TPA-DPPZ** emits to the red at λ_PL_ of 644 nm in neat film. The spatial arrangement of D and A groups in ***p*TPA-DPPZ** dramatically accelerated the rate of singlet emission without an increase in non-radiative decay, resulting in an increased Φ_PL_ of 87% in the neat film. This change in optical properties was accompanied by remarkably improved carrier transport in the neat film. As a result, a high-efficiency bilayer non-doped OLED was demonstrated, displaying deep-red emission at λ_EL_ = 652 nm and CIE coordinates of (0.67, 0.33,) and showing an EQE_max_ of 12.3% with EQE_1000_ of 10.4%.

**Figure 65a:**
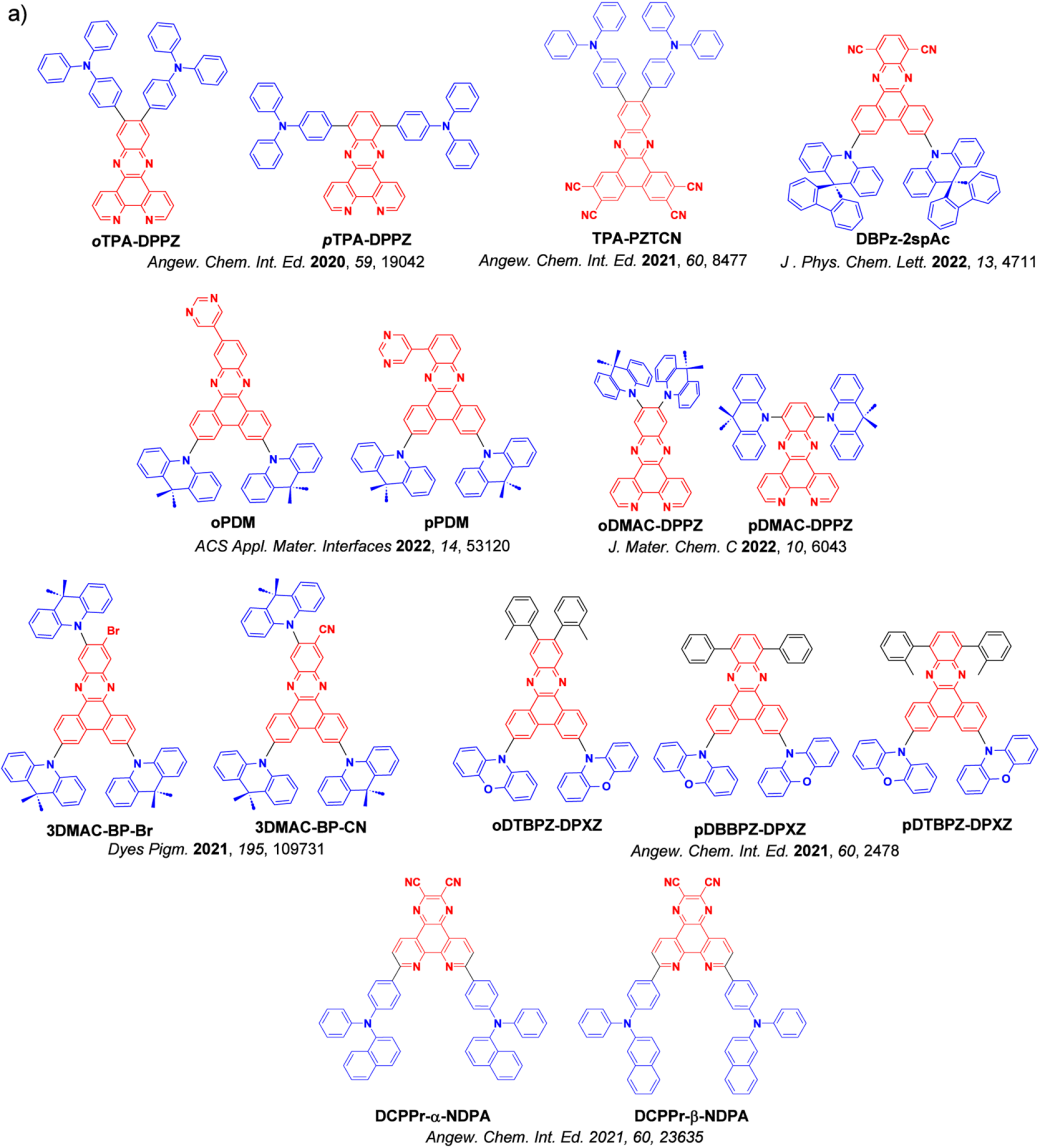
Molecular structures of red D-A TADF emitters containing either pyra­zin­yl­phen­an­threne or pyra­zin­yl­phen­an­thro­line acceptors. In the molecular structures, the blue color signifies donor moieties, while the red color signifies acceptor moieties.

**Figure 65b:**
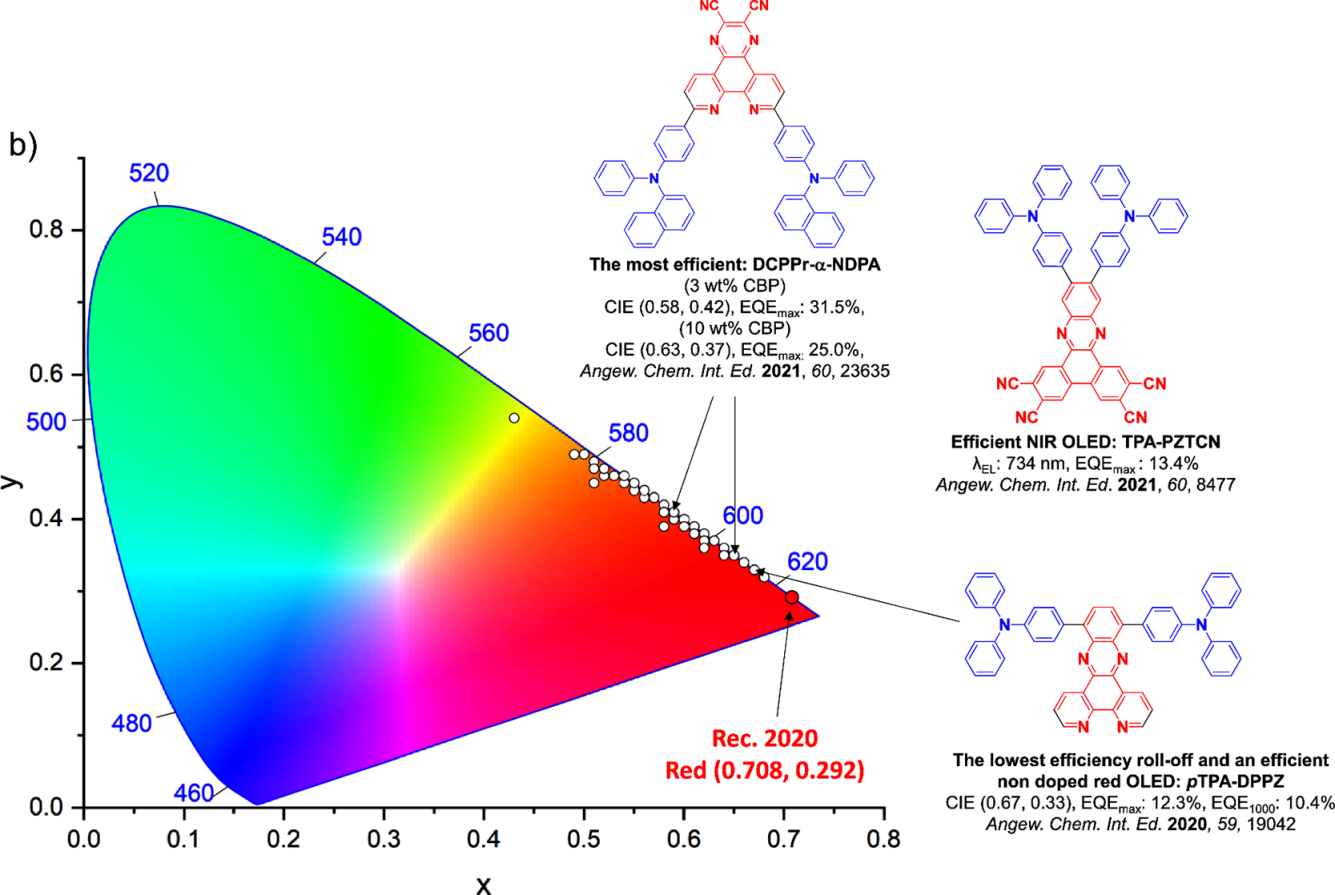
CIE color coordinates of red D-A TADF emitters containing phenanthrene acceptors. The white circles illustrate the spread of the emission color of the device. Selected devices and their associated CIE coordinates are highlighted, illustrating the structure of the emitter used in the device showing a high efficiency which is quantified by the EQE_max_, the structure of a near-IR emitter (λ_EL_ ∼ 780 nm) used in a device that showed a high efficiency which is quantified by the EQE_max_ and the structure of the emitter with the lowest efficiency roll-off, which was accomplished in a non-doped device with high efficiency. Only D-A TADF OLEDs where the λ_EL_ > 580 nm which are high performing are included. In the molecular structures, the blue color signifies donor moieties, while the red color signifies acceptor moieties.

Zhang *et al*. reported the red TADF emitter **DBPz-2spAc**, (Figure 65a) based on an 8b,14a-di­hy­dro­di­ben­zo[a,c]phen­azine-10,13-di­car­bo­nitrile acceptor and containing two spiro-acridines as donors.^521^ **DBPz-2spAc** has an Φ_PL_ of 27% (λ_PL_ = 632 nm) in toluene and 65% (λ_PL_ = 632 nm) in 1 wt% doped films in CBP. OLEDs at 1 wt% doping ratio showed high EQE_max_ of 13.3%, with the λ_EL_ at 630 nm. However, the devices suffer from severe efficiency roll-off, were the EQE_100_ drops to about 1%, attributed to triplet–triplet annihilation (TTA).

Tan *et al*. reported two isomeric orange-red TADF emitters, ***o*PDM** and ***p*PDM** (Figure 65a), with the same basic donor-acceptor backbone but with pyrimidine (Pm) attached at different positions.^522^ ***o*PDM** and ***p*PDM** emit at λ_PL_ of 582 and 573 nm, have moderate Δ*E*_ST_ of 0.11 and 0.15 eV, and high Φ_PL_ of ca. 100 and 88% in respective 8 wt% doped films in CBP (Table S3). OLEDs with ***o*PDM** or ***p*PDM** exhibited orange-red EL emission at λ_EL_ of 596 and 582 nm and CIE coordinates of (0.56, 0.44) and (0.52, 0.47), respectively. Despite similar PL properties, a significant difference in efficiency was seen in the two devices, with the OLEDs with ***o*PDM** or ***p*PDM** showing EQE_max_ of 28.2 and 11.8%, respectively. The difference was attributed to the differing molecular packing of the emitters in the aggregated state, resulting in very different charge transport performance.

Kothavale *et al*. reported red TADF emitters, ***o*DMAC-DPPZ** and ***p*DMAC-DPPZ** (Figure 65a), whose structures differ in the regiochemistry of the DMAC donor.^523^ The Φ_PL_ in toluene of the more red-shifted compound, ***p*DMAC-DPPZ** (λ_PL_ = 669 nm, Φ_PL_ = 15%) is lower than that of ***o*DMAC-DPPZ** (λ_PL_ = 652 nm, Φ_PL_ = 63%). In the bipolar host 2-phen­yl-4,6-bis­(12-phen­yl­in­do­lo[2,3-a]car­ba­zole-11-yl)-1,3,5-tri­azine (PBICT, 1 wt%) the emission of ***o*DMAC-DPPZ** is blue-shifted to λ_PL_ = 614 nm with CIE coordinates (0.59, 0.40), while ***p*DMAC-DPPZ** emits at λ_PL_ of 638 nm with CIE coordinates (0.64, 0.35). Aligning with their Φ_PL_, the device with ***o*DMAC-DPPZ** showed a higher EQE_max_ of 13.4% at λ_EL_ of 614 nm, while the device with ***p*DMAC-DPPZ** displayed a lower EQE_max_ of 4.0%.

Huang *et al*. synthesized two orange-red TADF emitters with D_3_-A structures, and modified by either an inductively electron-withdrawing bromine atom (**DMAC-BP-Br**) or a cyano-group (3**DMAC-BP-CN**, Figure 65a).^524^ 3**DMAC-BP-Br** and 3**DMAC-BP-CN** emit at λ_PL_ of 612 and 617 nm in toluene, have Φ_PL_ of 83 and 92%, Δ*E*_ST_ of only 0.04 and 0.02 eV, and τ_d_ of 3.8 and 4.6 μs, all respectively in 15 wt% doped films in CBP. The OLEDs with 3**DMAC-BP-Br** and 3**DMAC-BP-CN** showed EQE_max_ of 18.9 and 22.4% at λ_EL_ of 596 and 586 nm, respectively (Table S3).

Balijapalli *et al*. reported a D-A deep-red/NIR emitting compound, **TPA-PZTCN**, featuring multiple cyano groups about the acceptor (Figure 65a and 65b).^525^ **TPA-PZTCN** emits at λ_PL_ of 674 nm and has a Φ_PL_ of 77% in toluene. Doped at 1 wt% in mCBP, the emission of **TPA-PZTCN** at 672 nm maintains a high Φ_PL_ of 78%, while at 10 wt% loading the Φ_PL_ decreases to 40% accompanied by a much red-shifted λ_PL_ at 729 nm. OLEDs with 1, 3, or 6 wt% **TPA-PZTCN** showed EQE_max_ of 19.3% (λ_EL_ = 651 nm), 17.7% (λ_EL_ = 671 nm), and 15.8% (λ_EL_ = 712 nm), respectively (Table S3).

Chen *et al*. reported red TADF emitters **pDBBPZ-DPXZ**, **pDTBPZ-DPXZ**, and **oDTBPZ-DPXZ** (Figure 65a) using phenoxazine as donors and differently functionalised acceptors.^514^ **pDBBPZ-DPXZ**, **pDTBPZ-DPXZ**, and **oDTBPZ-DPXZ** have distinctive Φ_PL_ of 49, 66, and 87% in respective 8 wt% doped CBP films. Despite the differing Φ_PL_, the photophysical properties of the three compounds are quite similar, (**pDBBPZ-DPXZ** with λ_PL_ = 622 nm, Δ*E*_ST_ = 0.23 eV, and τ_d_ = 53.3 μs, **pDTBPZ-DPXZ** with λ_PL_ = 621 nm, Δ*E*_ST_ = 0.10 eV, and τ_d_ = 5.6 μs, and **oDTBPZ-DPXZ** with λ_PL_= 621, Δ*E*_ST_ = 0.04, and τ_d_ = 3.3 μs), except for the differences in the energies of the T_1_ states. From **pDBBPZ-DPXZ** to **pDTBPZ-DPXZ** and **oDTBPZ-DPXZ**, the ^3^LE_A_ energy levels gradually approach the CT states, from being deeply stabilized in **pDBBPZ-DPXZ** to near-isoenegetic with the ^3^CT state in **oDTBPZ-DPXZ** and leading to faster *k*_RISC_. The OLEDs with **pDBBPZ-DPXZ**, **pDTBPZ-DPXZ**, and **oDTBPZ-DPXZ** showed very similar red emission spectra, with λ_EL_ ∼ 604 nm and CIE coordinates of (0.59, 0.40), (0.58, 0.41), and (0.59, 0.41), respectively (Table S3). Following the ordering of Φ_PL_, the device with **oDTBPZ-DPXZ** showed the highest EQE_max_ of 20.1%, compared to the devices with **pDTBPZ-DPXZ** and **pDBBPZ-DPXZ** showing EQE_max_ of 16.0 and 8.0%, respectively.

A series of outstanding red emitters were reported by Cai *et al.*, which were designed to contain varying electron-donating tri­ar­yl­amine moieties attached to a pyra­zin­yl­phen­an­thro­line acceptor.^526^ In this series, nitrogen atoms within the pyridine rings were conjectured to engage in hydrogen bonding with the hydrogen atoms found in phenyl rings from the donating moieties. This interaction produces a more planar confirmation and rigid molecule. Out of the series, the compounds **DCPPr-α-NDPA** and **DCPPr-β-NDPA**, Figure 65a and 65b, which contain *N*,*N*-di­phen­yl­naph­tha­len-1-amine and *N*,*N*-di­phen­yl­naph­tha­len-2-amine, respectively, as the donor groups showed the most interesting photophysics. **DCPPr-α-NDPA** and **DCPPr-β-NDPA** emit at similar λ_PL_ of 598 and 612, respectively, in toluene, whereas in the neat film the λ_PL_ were considerably red-shifted at 692 and 710 nm, respectively. **DCPPr-α-NDPA** has a superior Φ_PL_ of 82%, compared to 74% for **DCPPr-β-NDPA**, which was attributed to the fact that the naphthalene is connected to the nitrogen atom via its α-position, which led to a suppressed molecular packing. On the other hand, a longer delayed lifetime of 42.7 μs was observed for **DCPPr-α-NDPA**, compared to 28.2 μs of **DCPPr-β-NDPA**, while the Δ*E*_ST_ are similar at 0.07 and 0.08 eV, respectively in 3 wt% doped film in mCP. Importantly, the hydrogen bonding was asserted to be responsible to aid in the preferential horizontal orientation of the compounds in the vacuum-deposited films, which led to high-efficiency red OLEDs with CIE coordinates of (0.58, 0.42) for the device with **DCPPr-α-NDPA** and (0.59, 0.40) for the device with **DCPPr-β-NDPA**. Devices were fabricated using 3 wt% emitters in mCP, achieving an outstanding EQE_max_ of 31.5% (λ_EL_ = 606 nm) with **DCPPr-α-NDPA**, whereas the device with **DCPPr-β-NDPA** resulted in an EQE_max_ of 27.1% (λ_EL_ = 616 nm). No efficiency roll-off data were reported.

### 5.5 Phenanthro[4,5-abc]phenazine-11,12-dicarbonitrile or Phenanthro[4,5-fgh]quinoxaline Acceptors

The Phenanthro[4,5-abc]phenazine-11,12-dicarbonitrile (PPDCN) acceptor was combined with a TPA donor to form the D–A NIR TADF emitter **TPA-PPDCN** (Figure 66).^527^ By replacing the phenanthrene in the acceptor core of the previous examples with pyrene, the π-conjugation of the acceptor is increased. This substitution results in a significantly deeper LUMO energy and a red-shifted emission. The Δ*E*_ST_ of **TPA-PPDCN** is 0.23 eV in toluene and the neat film emits at 725 nm, has a Φ_PL_ of 21%, and a τ_d_ of 1.96 μs. The PL spectra of the doped films gradually red-shift from 650 to 687 nm with increasing doping concentration (5 to 20 wt% in CBP), indicating a shift from monomolecular emission to emission from aggregates. The highest Φ_PL_ is 87% in the 10 wt% doped film in CBP, with λ_PL_ of 663 nm although the Φ_PL_ is maintained at 77% when the doping concentration is as high as 20 wt%. The OLEDs with **TPA-PPDCN** (10 and 20% doped in CBP) showed deep red and NIR emission with respective λ_EL_ of 664 nm [CIE coordinates of (0.68, 0.32)] and 692 nm [CIE of (0.70, 0.30)], at EQE_max_ of 20.2 and 16.4%, all respectively (Table S3). However, all devices exhibited large efficiency roll-off, with EQE_100_ decreasing to 4.7 and 3.7%, also respectively.

**Figure 66:**
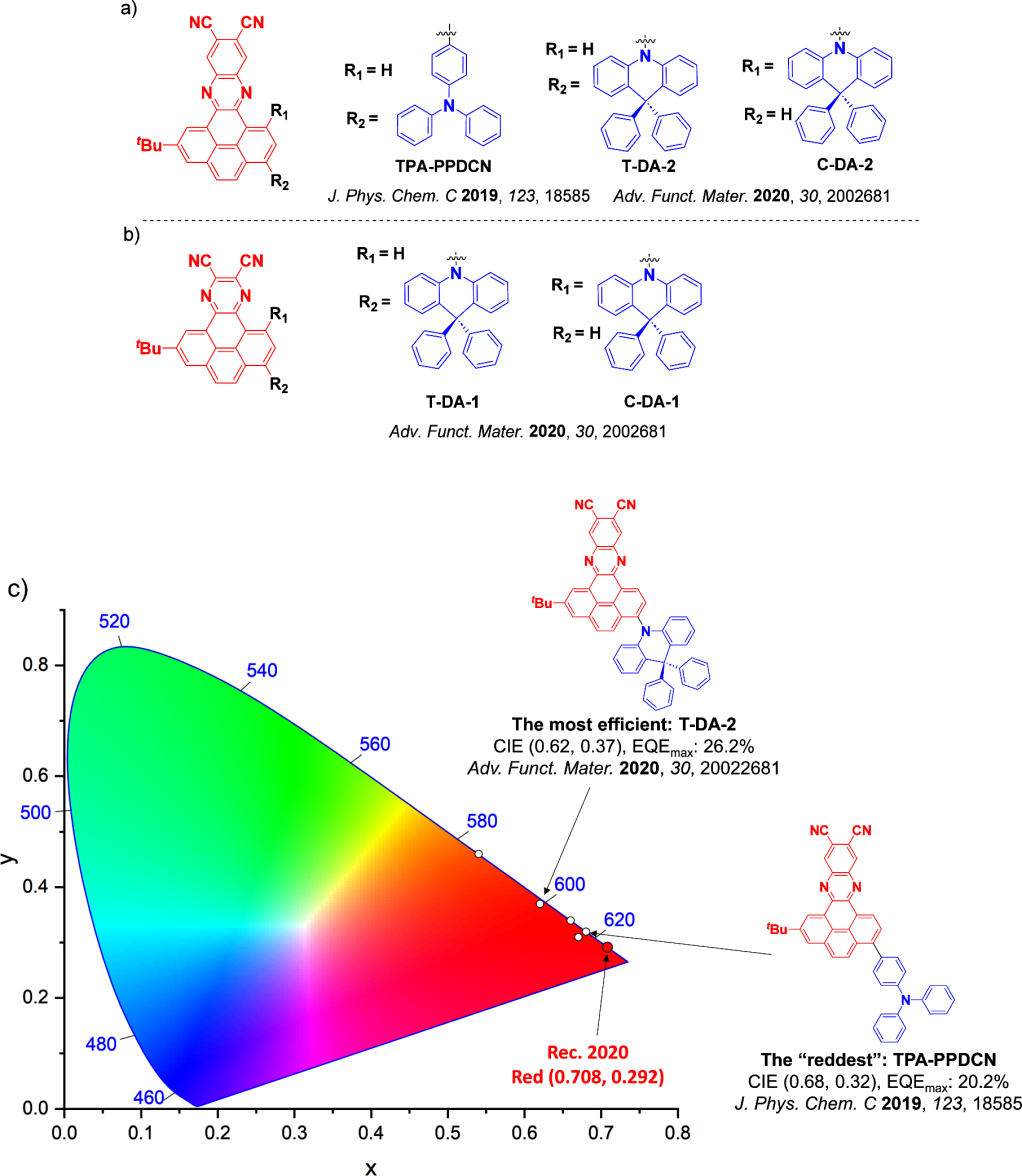
a) Molecular structures of red D-A TADF emitters containing phen­an­thro­[4,5-abc]phen­azine-11,12-di­car­bo­nitrile acceptors, b) molecular structures of red D-A TADF emitters containing phen­an­thro­[4,5-fgh]quin­oxa­line-10,11-di­car­bo­nitrile acceptors, and c) CIE color coordinates of red D-A TADF emitters containing phen­an­thro­[4,5-abc]phen­azine-11,12-di­car­bo­nitrile and phen­an­thro­[4,5-fgh]quin­oxa­line-10,11-di­car­bo­nitrile acceptors. The white circles illustrate the spread of the emission color of the device. Selected devices and their associated CIE coordinates are highlighted, illustrating the structure of the emitter of the “reddest” device and the structure of the emitter used in the device showing the highest efficiency. Only D-A TADF OLEDs where the λ_EL_ > 580 nm which are high performing are included. The most efficient device is quantified by the highest EQE_max_. The device with the CIE coordinates closest to the Rec. 2020 defined coordinates for red, (0.708, 0.292), is defined as the “reddest”. In the molecular structures, the blue color signifies donor moieties, while the red color signifies acceptor moieties.

The same group reported two pairs of isomers employing either the same PPDCN as the acceptor or shortened analogue PDCN acceptor in combination with acridine donors attached at two different locations (**T/C-DA-1/2**, Figure 66).^528^ In 10 wt% doped films in mCBP, **T-DA-1**, **T-DA-2**, **C-DA-1**, and **C-DA-2** emit at λ_PL_ of 601, 640, 640, and 689 nm, respectively. The Φ_PL_ of the *trans*-isomers (**T-DA-1** and **T-DA-2**, 78 and 89% respectively) are significantly higher than those of their corresponding *cis*-isomers (**C-DA-1** and **C-DA-2**, 12 and 14%). The Δ*E*_ST_ values are 0.16, 0.05, 0.02, and 0.02 eV for **T-DA-1**, **T-DA-2**, **C-DA-1**, and **C-DA-2** respectively, in toluene at 77 K. The OLEDs with **T-DA-1**, **T-DA-2**, **C-DA-1**, and **C-DA-2** doped at 10 wt% in mCBP showed orange-red to deep-red emission at λ_EL_ of 596, 640, 648, and 684 nm and CIE coordinates of (0.54, 0.46), (0.62, 0.37), (0.66, 0.34), and (0.67, 0.31), all respectively. Due to their low Φ_PL_, the devices with **C-DA-1** (EQE_max_ of 3.5%) and **C-DA-2** (EQE_max_ of 3.1%) showed much poorer efficiencies compared to the devices with **T-DA-1** and **T-DA-2**, which instead showed EQE_max_ of 22.6 and 26.3% respectively (Table S3). Crucially, the EQE_100_ of the **T-DA-2** based device still remained as high as 24%, corresponding to efficiency roll-off of just 8.8%.

### 5.6 1,8-Naphthalimide Acceptors

In addition to N-doped PAH acceptors discussed in the previous subsections, naphthalimide is another planar and strong acceptor with a deep LUMO. Incorporating a naphthalimide acceptor coupled to acridine donors, Zeng *et al*. reported efficient red emitters **NAI-DMAC** and **NAI-DPAC** (Figure 67).^529^ **NAI-DMAC** and **NAI-DPAC** emit at λ_PL_ of 582 and 570 nm, and have Φ_PL_ of 59 and 71% respectively in 1.5 wt% doped films in mCPCN. Increasing this doping ratio to 6 wt% resulted in ACQ with Φ_PL_ decreasing to 45% for **NAI-DMAC**. The concentration quenching effects were not observed for **NAI-DPAC** though, which retained a Φ_PL_ of 72% even after increasing the doping concentration to 24 wt%. Both emitters showed preferential horizontal orientation of their TDMs, assisting the optical outcoupling to support high device EQE_max_ of 23.4 and 29.2% from 1.5 wt% **NAI-DMAC** and 6 wt% **NAI-DPAC** in mCPCN, at λ_EL_ of 597 and 584 nm, all respectively (Table S3). Although these compounds are not as deep red as some of the previously discussed examples, they represent some of the most efficient red TADF OLEDs to date. However, there was a large efficiency roll-off, with the EQE_100_ dropping to 13.6 and 13.0% for the devices with **NAI-DMAC** and **NAI-DPAC**, respectively, while the EQE dropped by 80 and 92% at 1000 cd m^–2^. The same group later reported two orange/red emitters, **BFDMAc-NAI** and **BTDMAc-NAI** (Figure 67), by coupling fused heterocyclic DMAC donors to the NAI acceptor.^530^ Both compounds showed red-shifted emission compared to those of **NAI-DMAC** and **NAI-DPAC** at 600 and 650 nm, respectively. **BTDMAc-NAI** in 1.5 wt% doped films in mCPCN has a lower Φ_PL_ of 39%, while for **BFDMAc-NAI** the Φ_PL_ is higher at 73%, similar to that of its parent compound **NAI-DMAC**. The lower Φ_PL_ of **BTDMAc-NAI** was attributed to the introduction of the sulfur atom, which by virtue of the heavy atom effect increases both *k*_RISC_ but also the competing phosphorescence rate constant. This compound also has a smaller Δ*E*_ST_ of 0.07 eV compared to 0.16 eV for **BFDMAc-NAI**. OLEDs with **BTDMAc-NAI** emitted at λ_EL_ of 641 nm [CIE coordinates of (0.62, 0.38)], while the device with **BFDMAc-NAI** emitted at λ_EL_ of 590 nm [CIE coordinates of (0.54, 0.45)]. The redder **BTDMAc-NAI**-based device achieved an EQE_max_ of 9.2% (EQE_100_ dropping to 6.3%), while the orange **BFDMAc-NAI**-based device showed an EQE_max_ of 20.3%, but a large efficiency roll-off (EQE_100_ dropping to 10.6%).

**Figure 67:**
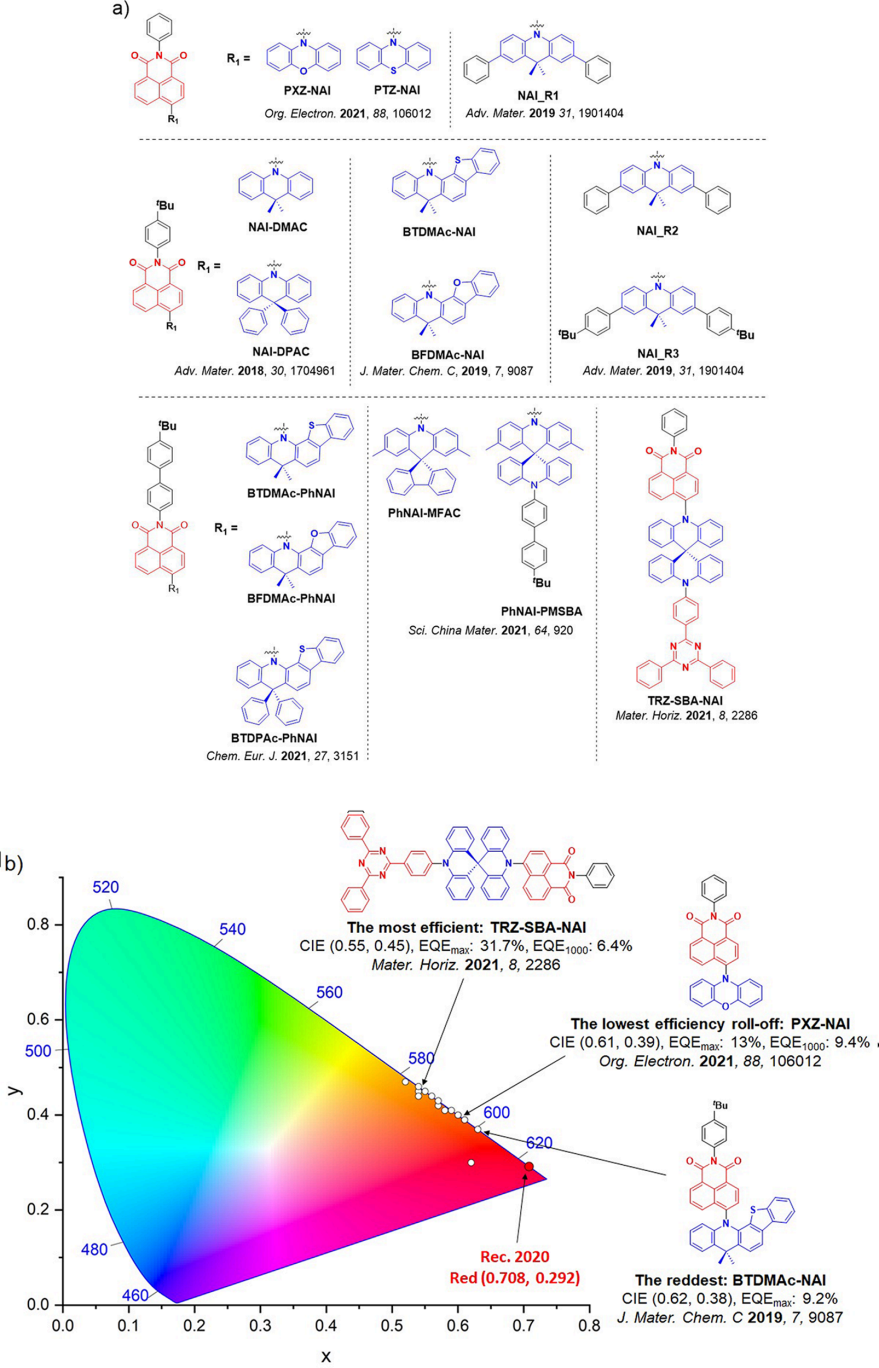
a) Molecular structures of red D-A TADF emitters containing 1,8-naphthalimide acceptors and b) CIE color coordinates of red D-A TADF emitters containing 1,8-naphthalimide acceptors. The white circles illustrate the spread of the emission color of the device. Selected devices and their associated CIE coordinates are highlighted, illustrating the structure of the emitter of the “reddest” device, the structure of the emitter used in the device showing the highest efficiency and the lowest efficiency roll-off. Only D-A TADF OLEDs where the λ_EL_ > 580 nm which are high performing are included. The most efficient device is quantified by the highest EQE_max_. The device with the CIE coordinates closest to the Rec. 2020 defined coordinates for red, (0.708, 0.292), is defined as the “reddest”. In the molecular structures, the blue color signifies donor moieties, while the red color signifies acceptor moieties.

High-efficiency solution-processed OLEDs with NAI-based red emitters have been developed by Zeng *et al*..^531^ **NAI_R1**, **NAI_R2**, and **NAI_R3** contain phenyl disubstituted DMAC donors, and *tert*-butyl substitution on the 1,8-naphthalimide acceptor (Figure 67). The phenyl extending units bestowed a stronger electron-donating ability and shallower HOMO level to these donors compared to DMAC, while also partially sterically protecting the emitter. The *tert*-butyl units were also attached at the para-position of the outer phenyl units of the donor and acceptor moieties, with the aim to both improve the solubility of the emitters and fine-tune their excited state energies. The emitters containing donors with *tert*-butyl substitution have significantly red-shifted emission (**NAI_R3** at λ_PL_ of 639 nm compared to **NAI_R1** and **NAI_R2** both with λ_PL_ of 627 nm in toluene). **NAI_R1**, **NAI_R2**, and **NAI_R3** have small Δ*E*_ST_ of 90, 92, and 58 meV, and relatively high Φ_PL_ of 63, 65, and 66%, respectively (Table S3). Solution-processed OLEDs were fabricated and displayed red λ_EL_ ranging from 610 to 622 nm, with the device with **NAI_R3** exhibiting the reddest CIE coordinates of (0.60, 0.40) and also the highest EQE_max_ of 22.5%.

Wang *et al*. incorporated PXZ or PTZ donors onto an NAI acceptor, leading to λ_PL_ of 605 and 617 nm for **PXZ-NAI** and **PTZ-NAI** respectively (Figure 67).^532^ **PXZ-NAI** and **PTZ-NAI** have Δ*E*_ST_ of 0.10 and 0.11 eV and short τ_d_ of 1.2 and 1.6 μs (Table S3). The OLEDs with **PXZ-NAI** and **PTZ-NAI** showed EQE_max_ of 13.0 and 11.4%, with λ_EL_ at 624 and 632 nm and CIE coordinates of (0.61, 0.39) and (0.63, 0.37), all respectively. Both devices exhibited relatively small efficiency roll-offs, with the EQE_1000_ values decreasing to 9.4 and 6.0%.

Zeng *et al*. reported a linear TADF molecule **PhNAI-PMSBA** (Figure 67) bearing an NAI acceptor attached to a large spiro-acridan PMSBA donor, and employing a design strategy to control the orientation of the TDM of the emitter.^533^ The properties and device performance were compared with a shortened reference emitter **PhNAI-MFAC**. **PhNAI-PMSBA** and **PhNAI-MFAC** emit at λ_PL_ of 606 and 603 nm, have Φ_PL_ of 61 and 55%, Δ*E*_ST_ of 0.06 and 0.05 eV, and short τ_d_ of 2.9 and 2.7 μs, in respective 1.5 wt% doped films (Table S3). The horizontal dipole ratio (Θ_||_) of **PhNAI-PMSBA** is 95%, enhanced compared to that of **PhNAI-MFAC** (Θ_||_ = 88%) and validating the emitter design strategy. The devices with **PhNAI-MFAC** and **PhNAI-PMSBA** emitted at λ_EL_ of 610 and 615 nm with corresponding CIE coordinates of (0.59, 0.41) and (0.60, 0.40). The reference device with **PhNAI-MFAC** showed an EQE_max_ of 22.5%, and despite its lower Φ_PL_ the device with **PhNAI-PMSBA** achieved an EQE_max_ of 22.3%, supported by its higher outcoupling efficiency of 43.2%. Both devices exhibited severe efficiency roll-offs though, with EQE_1000_ values reducing to 7.6 and 5.7%, respectively. The same group also reported three orange–red TADF emitters, **BFDMAc-PhNAI**, **BTDPAc-PhNAI**, and **BTDMAc-PhNAI** (λ_PL_ of 600, 610, and 650 nm in toluene, Figure 67), based on the same elongated acceptor coupled to three different fused heterocyclic donors.^534^ All three emitters have Δ*E*_ST_ < 0.16 eV and τ_d_ of around 45 μs, with Φ_PL_ of 77, 63, and 42% in respective 1.5 wt% doped films in mCPCN. OLEDs with **BTDPAc-PhNAI** showed EQE_max_ of 18.7% with λ_EL_ at 601 nm, compared to 19.8% for the device with **BFDMAc-PhNAI** (λ_EL_ = 590 nm) and 10.1% for **BTDMAc-PhNAI** (λ_EL_ = 642 nm).

Zeng *et al*. reported an asymmetric linear A–D-A’ type TADF emitter, **TRZ-SBA-NAI** (Figure 67), which contained a spiro-bisacridine donor core coupled to both an NAI and a triazine acceptor.^535^ Due to the coexistence of two distinct charge-transfer excited states, dual emission was observed in toluene comprising a dominant orange-red emission and a sky-blue emission shoulder. In 3 wt% doped films in mCBPCN, **TRZ-SBA-NAI** has a single emission band at λ_PL_ of 577 nm, a high Φ_PL_ of 87 %, Δ*E*_ST_ of 0.16 eV, and a long τ_d_ of 398 μs. Similar in molecular design to **PhNAI-PMSBA**, **TRZ–SBA–NAI** has a Θ_||_ of 88% in the same films. The OLED with **TRZ-SBA-NAI** consequently demonstrated an outstanding EQE_max_ of 31.7% at λ_EL_ at 593 nm with CIE coordinates of (0.55, 0.45). The OLEDs suffered from severe efficiency roll-off though, with the EQE values reducing by 79.8% at a luminance of 1000 cd m^–2^.

In a more advanced molecular design, Hua *et al*. reported a series of emitters based on a trinaphtho[3,3,3]propellane (TNP) core that is derivatized with **NAI-DMAC** (Figure 68).^536^ The unique TNP hexagonal stacking architecture allows the D-A TADF units to be encapsulated in cavities between two adjacent TNPs, reducing quenching via aggregation and/or annihilation of long-lived triplet excitons on the active chromophore. In this series of emitters, **tBu-S-mCP** possesses the best photophysical properties, emitting at λ_PL_ of 604 nm with Φ_PL_ of 70.9 % and a very small Δ*E*_ST_ of 7 meV, which corresponds to a surprisingly long τ_d_ of 6.41 μs (Table S3). The solution-processed OLEDs with **tBu-S-mCP** showed an EQE_max_ of 24.7% at λ_EL_ of 594 nm, however, the devices exhibited a large efficiency roll-off, with EQE_100_ of 14.9%.

**Figure 68:**
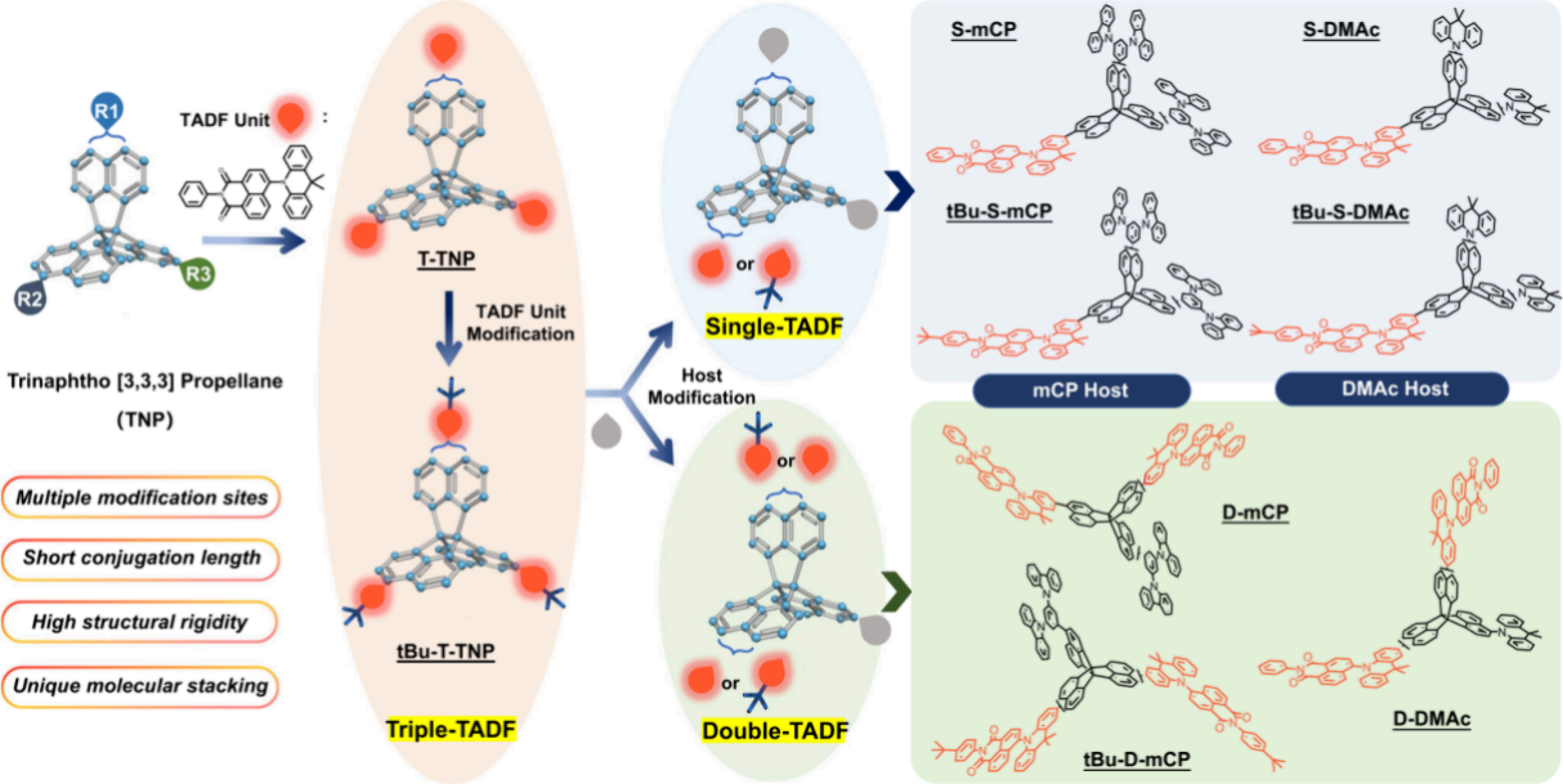
Chemical structure of trinaphtho[3,3,3]propellane (TNP) and molecular engineering pathway. Taken and adapted with permission from ref 536. Copyright [2022/Nature Communications] Springer Nature under Creative Commons Attribution 4.0 International License https://creativecommons.org/licenses/by/4.0/.

### 5.7 Other Miscellaneous Examples

The near-infinite scope of innovative and diverse design strategies associated with the development of red TADF emitters extends far beyond what this review can reasonably summarize. Apart from molecules using the previously discussed acceptor units, we also highlight here a collection of other notable design strategies. Kim *et al*. reported a highly efficient near-infrared TADF emitter, **2TPA-BF2** (Figure 69) constructed from a boron difluoride curcuminoid acceptor and TPA donors.^485^ By increasing the doping concentration from 2 to 60 wt% in CBP, the λ_PL_ shifted from 706 to 782 nm while the Φ_PL_ decreased from 59 to 7.5%. The neat film also emits at 782 nm and has a Φ_PL_ of 3.5% (Table S3). The highest Φ_PL_ of 70% is for the 6 wt% doped film in CBP (λ_PL_ of 721 nm), which translated into a superior solution-processed device that showed outstanding NIR EQE_max_ of 10% at λ_EL_ of 721 nm. Quantum-chemical calculations revealed that the TADF mechanism was assisted by vibrational and spin–orbit coupling alongside a large oscillator strength, which was illustrated by the overlap of electron and hole wave functions together with a non-adiabatic coupling effect.

**Figure 69:**
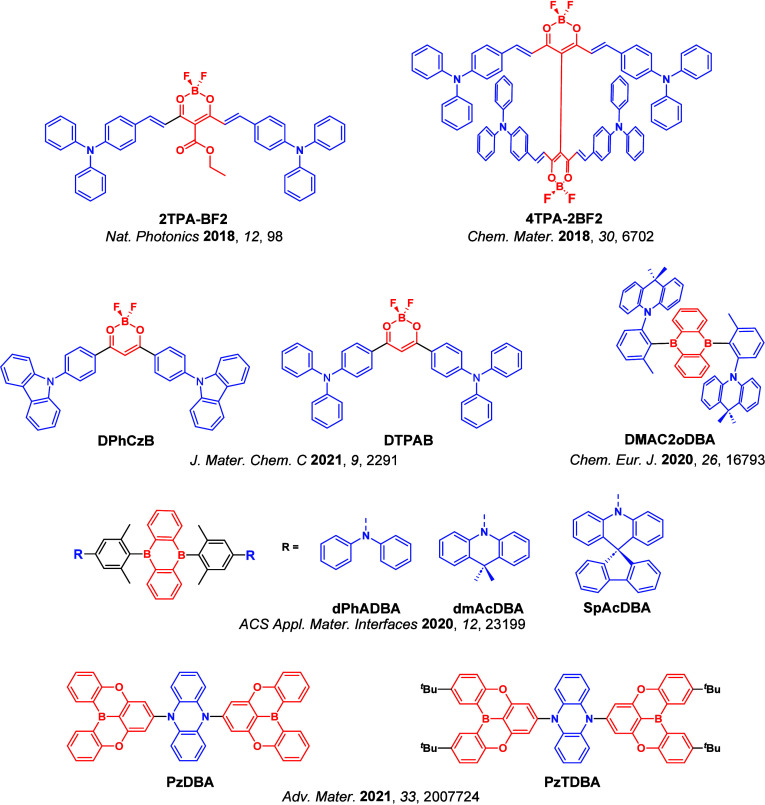
Chemical structures of red TADF emitters using other miscellaneous boron-containing acceptor moieties. The blue color signifies donor moieties, while the red color signifies acceptor moieties.

To further red-shift the electroluminescence, Ye *et al*. reported the dimeric bisborondifluoride curcuminoid dye **4TPA-2BF2** (Figure 69) that emits from 760 to 801 nm, and has decreasing Φ_PL_ (from 45.2 to 4.1%) as the doping concentration increases (from 2 to 40 wt%) in CBP.^537^ From DFT calculations the Δ*E*_ST_ is 0.3 eV and SOC between the S_1_ and T_1_ states is 0.13 cm^–1^. Solution-processed devices showed an EQE_max_ of 5.1% at λ_EL_ of 758 nm, supported by its high Φ_PL_ of 45.2%. These advances in NIR OLEDs, though unsuitable for displays and lighting, can unlock new technological applications in sensing, LIDAR/optical wireless networking, and biological imaging through the tissue transmission window.^538^ Utilizing the same difluoride curcuminoid acceptor with carbazole or DPA donors, Jin *et al*. reported the red TADF emitters **DPhCzB** and **DTPAB**.^539^ Neat films of **DPhCzB** and **DTPAB** emit at λ_PL_ of 637 and 650 nm and have Φ_PL_ of 54 and 56%, all respectively (Table S3). In 5 wt% doped films in mCP, the Φ_PL_ increased to 87 and 97%, although the emission blue-shifted to λ_PL_ of 587 and 605 nm. Solution-processed OLEDs with **DPhCzB** and **DTPAB** showed EQE_max_ of 6.7 and 8.2% at λ_EL_ 587 and 605 nm, all respectively. Interestingly, both devices showed low efficiency roll-offs of 3.7 and 9.0% at 100 and 1000 cd m^–2^ which was attributed by the authors to the respective τ_d_ of the emitters (19.5 and 55.7 μs).

Kumar *et al*. reported the doubly boron-doped emitter **DMAC2*o*DBA** (Figure 69), based on a 9,10-diboraanthracene (DBA) acceptor decorated with *ortho*-substituted acridine donors.^540^ This compound emits at λ_PL_ of 602 nm and has a moderate Φ_PL_ of 44% in 20 wt% doped film in CBP. The low Δ*E*_ST_ of 54 meV was attributed to the very strongly twisted conformation, supported by the additional *ortho*-methyl substitution of the phenylene linkers. However, due to its Φ_PL_, the EQE_max_ was limited to 10.1% at λ_EL_ 615 nm. Utilizing the same DBA acceptor, Hsieh *et al*. reported orange-red emitters, **dPhADBA**, **dmAcDBA**, and **SpAcDBA** by attaching either DPA, DMAC, or spiro-acridine, respectively.^488^ In contrast to *ortho* substitution, these *para*-substituted compounds showed increased Φ_PL_ ranging from 53 to 85% and λ_PL_ between 570 to 614 nm in 12 wt% doped films in CBP (Table S3). These compounds have fast *k*_RISC_ (1.3–2.4 ×10^5^ s^–1^) resulting from the small Δ*E*_ST_ values, ranging from 0.04 to 0.08 eV. The fast RISC and highly horizontal TDM orientation ratio of between 84–86% translated into devices with EQE_max_ ranging from 11.1 to 30.0%, tracking with the Φ_PL_ of the emitters, at λ_EL_ from 567 to 613 nm.

Karthik *et al*. reported red TADF emitters **PzTDBA** and **PzDBA** (Figure 69), constructed from rigid oxygen-bridged boron acceptors (DOBNA, see Section MR-TADF) and a central dihydrophenazine donor.^489^ **PzTDBA** and **PzDBA** emit at λ_PL_ of 599 and 610 nm, have high Φ_PL_ of 99.8 and 85.4%, small Δ*E*_ST_ of 0.06 and 0.05 eV, short τ_d_ of 2.63 and 2.00 μs, and fast *k*_RISC_ of 1.19 and 0.84 ×10^6^ s^–1^, all respectively in 5 wt% doped films of TCTA/Bepp2 (1:1) mixed ambipolar host (Table S3). The devices with **PzTDBA** and **PzDBA** showed EQE_max_ of 30.3 and 21.8% and extremely low efficiency roll-off (reducing by 3.6 and 3.2% of maximum values at 1000 cd m^–2^) at λ_EL_ of 576 and 595 nm, respectively. Impressively, the devices with **PzTDBA** and **PzDBA** showed operating device lifetimes (LT_50_) of 159 and 193 h at 1000 cd m^–2^, also respectively.

Kumsampao *et al*. reported the NIR TADF D-A-D emitter **TPACNBz** (Figure 70) based on strongly electron-deficient 5,6-di­cy­ano­[2,1,3]ben­zo­thia­dia­zole (CNBz) acceptor and TPA donors.^541^ **TPACNBz** emits at λ_PL_ of 750 nm and has a Φ_PL_ of 21% as a neat film (Table S3). The emission blue-shifts to 710 nm and the Φ_PL_ increases to 52% in 30 wt% doped films in CBP, and the OLEDs showed an EQE_max_ of 6.6% at λ_EL_ of 712 nm. These results clearly demonstrate that this acceptor, commonly used in OPV dyes, is an excellent building block for creating low-band-gap emitters.

**Figure 70:**
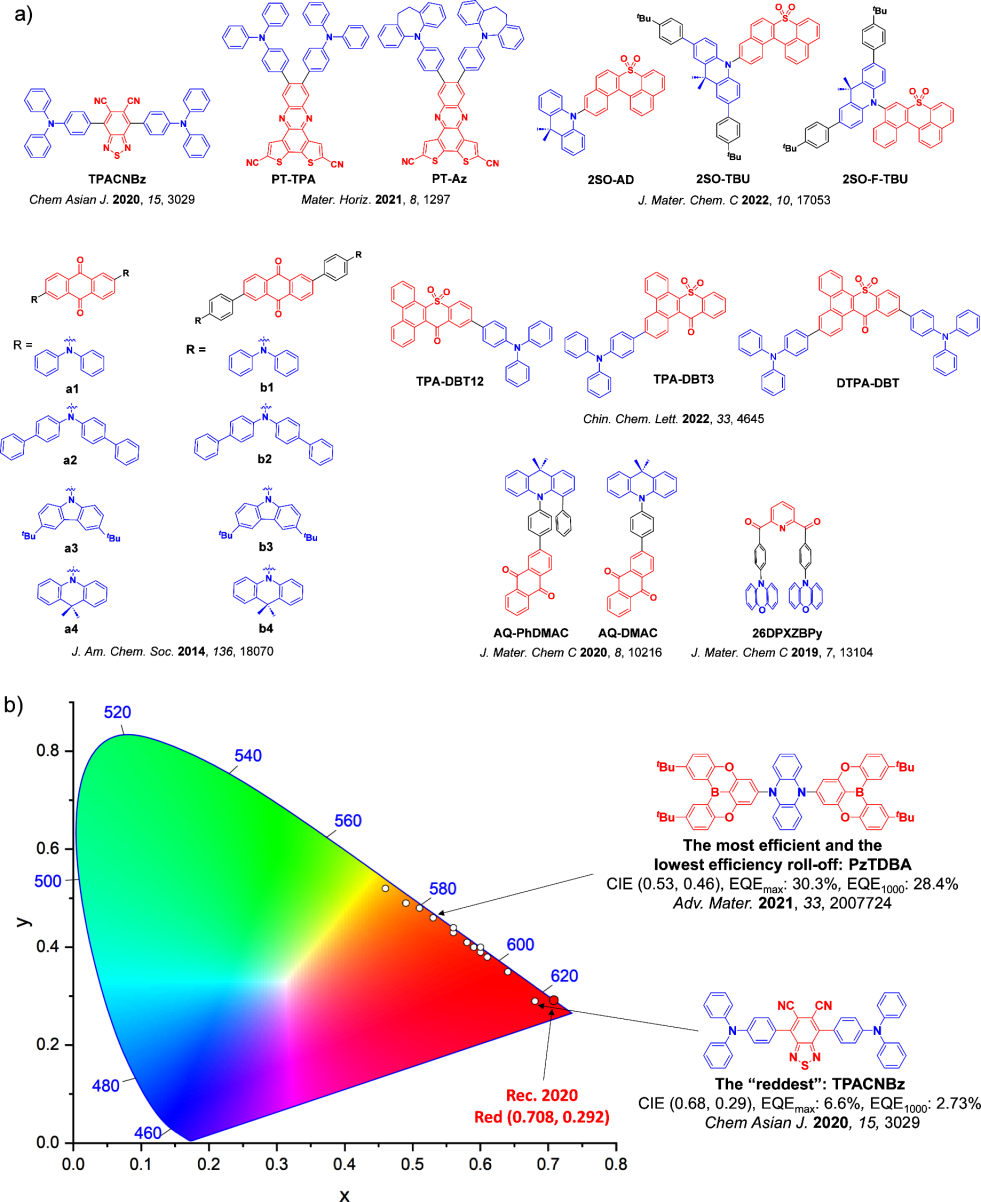
a) Molecular structures of red D-A TADF emitters containing other acceptors and b) CIE color coordinates of red D-A TADF emitters containing other acceptors. The white circles illustrate the spread of the emission color of the device. Selected devices and their associated CIE coordinates are highlighted, illustrating the structure of the emitter of the “reddest” device and the structure of the emitter used in the device showing the highest efficiency and the lowest efficiency roll-off. Only D-A TADF OLEDs where the λ_EL_ > 580 nm which are high performing are included. The most efficient device is quantified by the highest EQE_max_. The device with the CIE coordinates closest to the Rec. 2020 defined coordinates for red, (0.708, 0.292), is defined as the “reddest”. In the chemical structures, the blue color signifies donor moieties, while the red color signifies acceptor moieties.

Wang *et al*. nicely demonstrated the impact of chromophore rigidity and/or flexibility on photophysics and the corresponding device performance.^542^ Red emitters **PT-TPA** and **PT-Az** (Figure 70) containing dithi­eno­[3,2-a:2′,3′-c]phen­azine acceptor and either a flexible TPA donor in **PT-TPA** or a relatively rigid Az donor in **PT-Az** were investigated. This structural variation in the donors of **PT-TPA** and **PT-Az** did not alter the energy levels of the S_1_ and T_1_ states to any appreciable extent, and in toluene both compounds have similar respective Δ*E*_ST_ of 0.26 and 0.28 eV and Φ_PL_ of 66.5 and 56.3% (Table S3). In **PT-Az** the rotation of terminal phenyl groups is constrained by an ethylene linker, leading to its inferior Φ_PL_. In contrast, **PT-TPA** with freely rotating phenyl groups has a low reorganization energy and a larger transition dipole moment for the S_1_–S_0_ transition, which resulted in a high *k*_r_ of 2.31 × 10^7^ s^–1^ (**PT-Az** *k*_r_ = 2.33 × 10^6^ s^–1^). In 15 wt% doped films in CBP **PT-TPA** has a near unity Φ_PL_ of 99.7% (τ_d_ = 57.79 μs) while it is much lower at 52.7% (τ_d_ = 27.68 μs) for **PT-Az**, attributed to out-of-plane wagging vibration modes associated with the restricted Az units of the emitter contributing to increased non-radiative decay. OLEDs showed EQE_max_ of 29.7% (λ_EL_ = 632 nm) for **PT-TPA** and 14.1% (λ_EL_ = 612 nm,) for **PT-Az**.

Hu *et al*. reported the use of a rigid diben­zo­thio­xan­thone (DBT) acceptor that possesses a low-lying localized triplet excited state to facilitate effective RISC.^543^ Isomeric D-A emitters **TPA-DBT12**, **TPA-DBT3**, and D-A-D **DTPA-DBT** (Figure 70) emit at 597, 616, and 632 nm and have Φ_PL_ of 44, 55, and 42%, in respective 5 wt% doped 35DCzPPY films (Table S3). Red OLEDs showed EQE_max_ of 14.5, 15.0, and 11.8% at λ_EL_ of 608, 612 and 628 nm, for the same emitters, respectively. Gao *et al*. employed a similar approach using a modified diben­zo­thio­xan­thene acceptor that had a low-lying localized triplet excited state.^544^ **2SO-AD**, **2SO-TBU**, and **2SO-F-TBU** additionally contained bulky acridine donors to suppress ACQ (Figure 70). The three compounds doped in 10 wt% 35DCzPPY films emit at 581, 615, and 591 nm and have Φ_PL_ of 25, 58, and 53%, respectively. **2SO-AD**, **2SO-TBU**, and **2SO-F-TBU** have Δ*E*_ST_ values of 0.27, 0.14, and 0.20 eV and long τ_d_ of 553.0, 272.1, and 577.5 μs, all respectively. Red OLEDs with **2SO-AD**, **2SO-TBU**, **2SO-F-TBU** showed EQE_max_ of 3.2, 16.3, and 14.5% with λ_EL_ of 599, 608, and 612 nm, also respectively.

Anthraquinone (AQ) has also been exploited as an acceptor unit in the design of red emitters owing to its deep LUMO (-2.80 eV). The first AQ-based TADF red emitter was reported by Zhang *et al*. where they synthesized four D-π-A-π-D type emitters (**b1**, **b2**, **b3**, and **b4**) with an AQ acceptor (Figure 70).^545^ They incorporated various donors such as DPA, BBPA, DTC, and DMAC, and employed phenyl (Ph) rings as π-bridges, respectively. The synthesized emitters were also compared to the corresponding D-A-D type emitters (**a1**, **a2**, **a3**, and **a4**). For all of these emitters the measured Δ*E*_ST_ values are relatively small and showed a gradual decrease in magnitude with increasing donor strength from DPA to DMAC. For molecules **a1–4**, the Δ*E*_ST_ values range from 0.08 to 0.29 eV, while for molecules **b1–4**, the values range from 0.07 to 0.24 eV, all in 1 wt% doped films in CBP. However, neither the λ_PL_ nor Φ_PL_ strictly correlated with the donor strength. For molecules **a1–4**, the λ_PL_ (Φ_PL_) are 593 (0.5%), 603 (0.4%), 575 (0.5%), and 600 nm (0.1%) in the same respective films. By comparison, for molecules **b1–4**, the equivalent λ_PL_ (Φ_PL_) are 594 (0.8%), 601 (0.8%), 550 (0.7%), and 564 nm (0.5%). The **b1**- and **b2**-based OLEDs in 10 wt% CBP host emitted at λ_EL_ of 624 and 637 nm with corresponding CIE coordinates of (0.61, 0.39) and (0.63, 0.37), while **b3** and **b4** OLEDs emitted at λ_EL_ of 574 nm and 584 nm, all respectively. The devices with **b1–4** showed EQE_max_ of 12.5, 9.0, 9.0, and 6.9%, but the devices with **b1** and **b2** exhibited severe efficiency roll-off, decreasing to 8.1 and 5.7% at a luminance of 100 cd m^–2^, and to 2.3 and 1.7% at a luminance of 1000 cd m^–2^, all respectively. This was attributed to the long τ_d_ of 416 and 185 μs observed for **b1** and **b2** in respective 1 wt% doped films in CBP. Material **b4** having a much shorter τ_d_ of 6.5 μs translated in devices with much reduced efficiency roll-off of 6% at a luminance of 1000 cd m^–2^. Emitters **b3** exists as a mixture of rotamers in the doped CBP films, with some having a short τ_d_ of 16 μs comparable to **b4**, while others having a long τ_d_ of 156 μs comparable to **b2**. Consequently, the efficiency roll-off of the OLEDs with **b3**-based fell between those of the devices with **b2** and **b4**.

Hao *et al*. reported the emitter **AQ-PhDMAC** (Figure 70, containing a phenyl-substituted DMAC) and compared it to **AQ-DMAC**.^546^ Owing to the steric effect of the α-phenyl ring, **AQ-PhDMAC** emits at λ_PL_ of 586 nm, has a Δ*E*_ST_ of 0.22 eV, a τ_d_ of 63.6 μs, and a high Φ_PL_ of 89%, trading off TADF performance for higher Φ_PL_ compared with **AQ-DMAC** (λ_PL_ = 580 nm, Δ*E*_ST_ = 0.02 eV, τ_d_ = 21.2 μs, and Φ_PL_ = 63%) (Table S3). The orange-red OLED with **AQ-PhDMAC** showed an EQE_max_ of 18.1% which was higher than the device with **AQ-DMAC** (EQE_max_ of 13.9%). Both devices emitted similarly at λ_EL_ of 580 nm and with CIE coordinates of (0.49, 0.49); however, the **AQ-PhDMAC**-based device exhibited more serious efficiency roll-off arising from its longer τ_d_.

Pandidurai *et al*. reported yellow-orange TADF emitter **26DPXZBPy** (Figure 70), containing dibenzoyl pyridine as the acceptor and PXZ as the donors.^547^ **26DPXZBPy** emits at around 600 nm, has a Φ_PL_ of 76%, a small Δ*E*_ST_ of 0.04 eV, and a τ_d_ of 1 μs in 10 wt% doped films in mCBP (Table S3). Devices with **26DPXZBPy** gave orange emission at λ_EL_ of 590 nm at CIE coordinates of (0.49, 0.49), and showed an EQE_max_ of 13.7%.

### 5.8 Outlook

TADF emitters based on N-doped PAH acceptors have drawn significant attention towards the development of efficient red OLEDs. There are now examples of devices with reported EQE_max_ exceeding 30%. As a representative example, OLEDs with **DCPPr-α-NDPA** achieved an EQE_max_ of 31.5% at λ_EL_= 606 nm (Figure 71).^526^ To achieve deeper-red emission though, acceptors with a small degree of π-conjugation such as pyridine, quinoxaline, and acenaphtho[1,2-b]pyrazine require fortification with strong electron-withdrawing units like nitrile or fluorine to stabilize the CT singlet states. For example, **pCNQ–TPA** contains an acceptor decorated with two nitrile units, and the corresponding devices demonstrated deep red electroluminescence at λ_EL_ of 660 nm and an outstanding EQE_max_ of 30.3%.^525^

**Figure 71:**
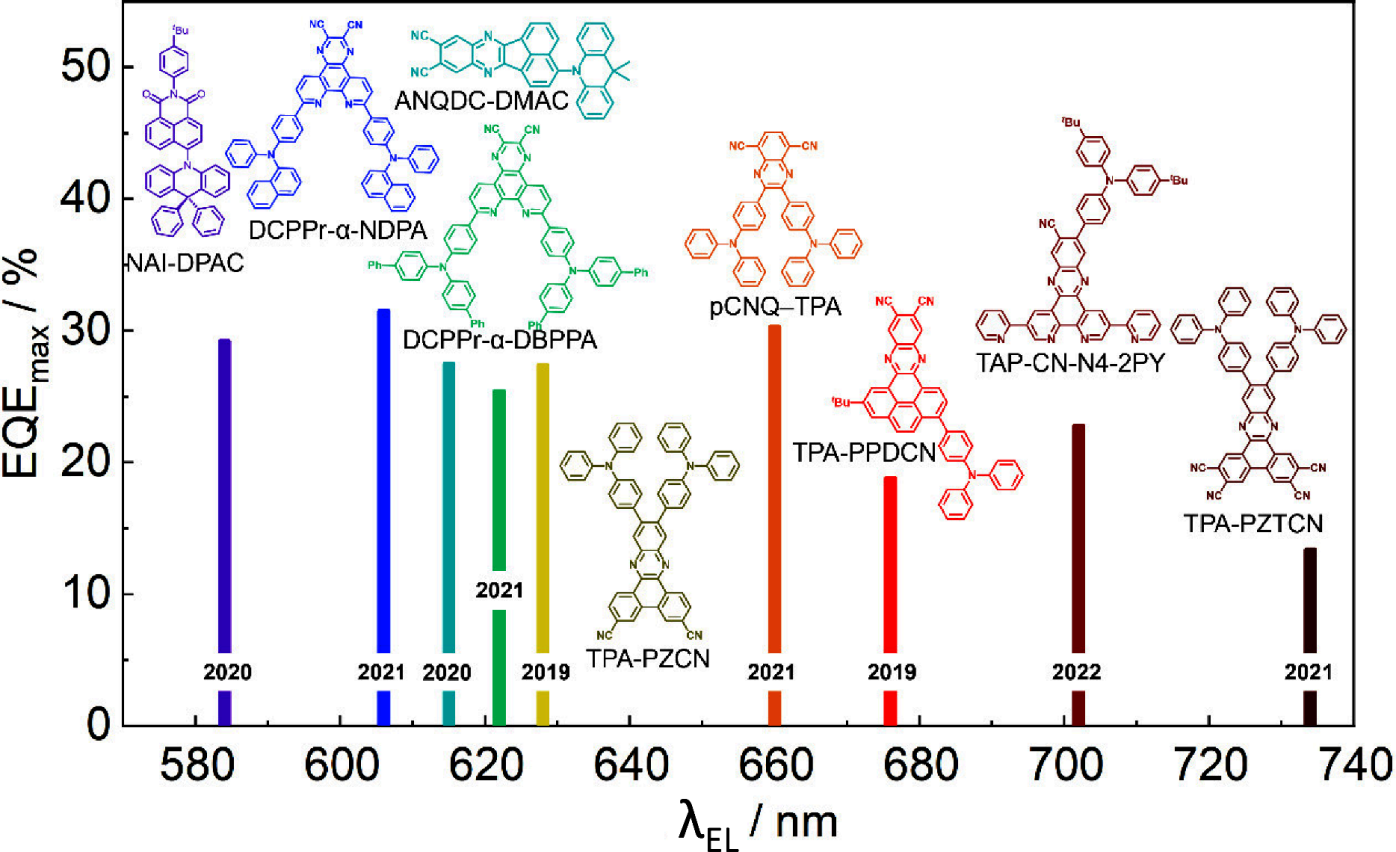
EQE_max_ vs λ_EL_ for selected emitter structures from high-performance red TADF OLEDs reviewed in this section.

Extending past the visible spectrum, OLEDs with **TPA-PZTCN**, containing a π-expanded acceptor, exhibited intense NIR EL (EQE_max_ = 13.4%) at a λ_EL_ of 734 nm, which is particularly impressive in this wavelength region where the energy-gap law typically limits emission efficiency. This strategy of π-expansion can have drawbacks though, as acceptors with too large a π-conjugation like phenanthro[4,5-fgh]quinoxaline can result in D-A compounds with larger Δ*E*_ST_ due to the presence of a too-stabilized acceptor LE state. Illustrating this balance, although the device with **TPA-PPDCN** showed an EQE_max_ of 18.8% at a λ_EL_ of 676 nm, there was also serious efficiency roll-off (EQE_100_ = 3.7%)^527^ resulting from the moderate Δ*E*_ST_ of 0.23 eV. Indeed, many of the examples in this section demonstrate the challenge of obtaining the desired deep-red emission while also preserving high EQE and low efficiency roll-off. There is certainly still pressing need for new emitter designs that address these deficiencies in device performance.

Aside from N-PAH acceptors, the recent use boron­di­fluoride cur­cu­min­oids, dibora­an­thra­cene, di­ben­zo­thiox­an­thone, and 5,6-di­cy­ano­[2,1,3]ben­zo­thia­di­azole acceptors have also shown promise in delivering deep-red and NIR emission. Additionally, while strong intermolecular interactions usually have a negative impact on device efficiency and stability, they are not always detrimental to performance (Section 13), and can offer a route to red-shifted emission spectra. Since red TADF emitters with low T_1_ energies can take advantage of the full range of available OLED host materials (in contrast to high T_1_ blue emitters), we expect that development of red emitters in the coming years will follow in tandem with the development of similarly unrestricted green TADF emitters – although a few years behind in terms of raw performance metrics as more challenging energy-gap law and ACQ considerations are navigated by the research community. Red emission, lowest in energy in the visible wavelength range, can also take the fullest advantage of hyperfluorescence approaches (Section 17), and thus benefit directly from performance advancements in materials of other colors. With multiple promising strategies clearly identifiable, the pace of progress and achievement in often-neglected red TADF OLEDs is hence likely to rise to match that of other colors in the near future.

## 6 White OLEDs Using TADF Materials

### 6.1 Introduction

White OLEDs (WOLEDs) show great potential for use in efficient and low power, flexible, large-area displays and lighting.^548,549^ Considering the now-established successes of high-performance red (R, Section 5), green (G, Section 4), and blue (B, Section 3) TADF OLEDs, it is natural and promising to develop high-performance WOLEDs using TADF materials.^42^ In this section we first summarize the distinct performance metrics and engineering challenges associated with WOLEDs (in comparison to single color devices), and then highlight the range of molecular and device design strategies that use TADF toward this goal.

Although WOLEDs understandably share many similarities with single color OLEDs, their performance is evaluated based on subtly different criteria, which include power efficiency, color quality, and color and operational stability. For WOLEDs, the power efficiency (PE, lm/W) is a more important parameter than EQE alone, which is closely related to the overall power consumption.^11^ Whereas the EQE values of both single color and white TADF OLEDs have achieved the theoretical limits, it is not easy to optimize the PE values, especially for the intrinsically complicated WOLEDs. For both OLED categories, high PE value is often achieved by tuning the types and thicknesses of functional layers including transport layer and host, in order to make charge injection barriers between layers (and therefore the required driving voltages) as low as possible while also maintaining the EQE. To be competitive with commercial lighting luminaires such as those based on fluorescent tubes, a PE of greater than 90 lm/W, and ideally greater than 120 lm/W is expected. Furthermore, for lighting applications brightnesses of thousands of cd m^–2^ are typically required, with corresponding higher exciton densities and larger demand for triplet harvesting presenting a challenge for current TADF materials.

The second of these assessment criteria is the quality of the white light. As previously mentioned, there are two main kinds of white light: cool white and warm white. Cool white light has CIE coordinates of (0.33, 0.33), with a correlated color temperature (CCT, equating color to that of blackbody radiation at a set temperature) of around 5000 K. Warm white light has CIE coordinates of (0.448, 0.408) and a CCT corresponding to a lower temperature of 2856 K.^550^ While ‘cool’ white corresponding to a hotter CCT appears at first contradictory, it is more readily understood when considered in the colloquial sense of ‘white-hot’ and less extreme ‘red-hot’ thermal emission. Warm white light therefore contains a smaller contribution from blue emission and is typically used in domestic lighting, whereas cool white light luminaires are more frequently found in commercial and industrial settings. Other types of white light with variable associated CCT exist both between and beyond these extremes, although these two have become de facto standards in research and industry.

An important associated parameter for the quality of the light is the color rendering index (CRI), ranging between 0 and 100. This index describes the degree to which the light source can resemble a ‘natural’ light source with a continuous blackbody emission spectrum, such as sunlight. Distinct from incandescence, WOLEDs can instead exhibit emission spectra that have some visible wavelengths overexaggerated, and others completely absent. Although such emission spectra may still be physiologically averaged to produce a perceived white CIE coordinate, illumination with such an OLED (with low CRI) will produce perceptible color changes in any illuminated objects. This is because some wavelengths that contribute to the normal reflectance and perceived color of the object are absent from the illumination source, and so balanced emission intensity at all visible wavelengths is required to achieve high CRI. For typical luminaires a CRI value of 80 is required, whereas for specialized applications such as art displays, in hospitals, and the textile industry, CRI values of over 90 are expected.

Finally, as with single-color OLEDs the stability of the WOLED is vital for commercialization. WOLEDs for luminaires must, however, show both color stability and device stability under continuous operation. As WOLEDs typically employ multiple emitter species, each with their own triplet harvesting performance and overall stability, the amount of emission from each and therefore also the overall color and CIE coordinates of the device can change significantly both at different brightnesses and over time. This can be assessed by CIE variation (both at different driving currents, and over time) and device lifetime, whereby smaller CIE variation and longer device lifetime are desired. For lighting applications, device stability is typically quantified in terms of LT_50_, which indicates the time at which the overall EL intensity is at 50% of its initial value (usually taken at 1000 cd m^–2^) under constant current driven conditions.

To fulfil all the above criteria, both high-performance emitters and rational designs for device structure are needed. Although all-phosphor based WOLEDs with maximum PE (PE_max_) of over 100 lm/W have been reported, the poor stability of blue phosphorescent emitters renders them unsuitable for commercial applications.^551^ Instead, a hybrid device structure currently enjoys widespread commercialization in which blue and the complementary colors (green, G, yellow/orange, Y/O and red, R) are generated from fluorescent and phosphorescent dyes, respectively.^552^ In these vertically stacked multilayer devices, careful exciton management is crucial to excite the different layers in the correct ratios, harvest all the excitons, suppress unintentional exciton energy transfer, and ensure device operational stability. These simultaneous considerations result in a complicated device structure and doping scheme.^553,554^

The arrival of high-efficiency TADF materials has stimulated new strategies to manipulate excitons, optimize device structure, and ultimately improve WOLED device performance. There are several potential advantages and ways of using TADF materials in WOLEDs; they could serve as emitters, as hosts, as sensitizers, or combinations of these functions. Efficient exciton harvesting is certainly achievable, a prerequisite evidenced in single color TADF OLED devices. Indeed using TADF molecules, WOLEDs with EQE_max_ of 30% have already been achieved and surpassed, indicating that further advancement is limited now only by the light out-coupling efficiency.^555–557^ Furthermore, by using an exciplex-type TADF host, WOLEDs with PE_max_ of over 80 lm/W have been reported.^558^ Ultimately, the typical donor-acceptor molecular structure reported for TADF emitters and the associated capacity to fine tune the photophysical properties of the emitters provides a large freedom in materials design to generate white light systems. Dual-emission properties associated with this kind of D-A structure have also enabled a small number of examples of single molecule white TADF emitters, that have been explored in WOLEDs.^557^ These emission properties – unrelated to TADF-activity but exceedingly rare for simple fluorescent molecules – provide an avenue to fabricate WOLEDs with considerably simpler device structures and therefore lower fabrication cost. Furthermore, contrary to the requirement for high color purity in displays, the use of CT emitters showing broad emission (FWHM: 70–120 nm) is desirable in WOLEDs to achieve a high CRI.

From all of these potential advances of using TADF materials in WOLEDs, there emerge two main design approaches to obtain white light emission: two-color and three-color systems. In two-color systems, the blue light originates in most cases from a TADF emitter, while the yellow or orange component comes from a separate phosphorescent, fluorescent, TADF exciplex or TADF emitter. This approach benefits from simpler device design, but without a dedicated green emitter often struggles to achieve high CRI. In three-color systems, TADF emitters have been used as one or more of the separate red, green, and blue components. For WOLEDs based on a three-color system, a CRI above 80 has been reported, which is still rare for most two-color systems.^559,560^

Using TADF components, the complexity of device structure and exciton management in WOLEDs can be somewhat mitigated as well. Examples of an emitting layer (EML) containing only TADF molecules as both the emitter and host, non-doped TADF EMLs, single EML TADF WOLEDs, and single molecule white TADF emitters have all been reported.^561–564^ The simplified device structure eases the device fabrication and reduces the number of associated optimization parameters, reducing costs for both research and development as well as for commercial production. Although device stability studies are quite limited, especially in terms of identifying the degradation mechanism, recent reports show encouraging evidence of stable TADF WOLEDs. The LT_50_ of hybrid TADF WOLEDs can now exceed 10^4^ hours, while the LT_80_ of all-fluorescent TADF WOLEDs have reached over 8200 hours.^565,566^ Additional systematic studies are needed to thoroughly understand the degradation mechanisms in TADF WOLEDs. While such studies are both fundamentally and practically challenging to perform, the resulting insights will ultimately inform the design of materials leading to improved device performance.

Separating from their classification as two- or three-color devices, WOLEDs can be divided into three categories depending on the photophysical properties of the individual color components as shown in Figure 72. Hybrid TADF WOLEDs contain both phosphorescent and TADF emitters, all-fluorescent TADF WOLEDs contain either a combination of fluorescent and TADF emitters or TADF emitters only, and lastly single molecule TADF WOLEDs have also been demonstrated. In view of their importance and potential in industry, only vacuum-evaporated small molecule WOLEDs are considered in this section. Other related topics such as solution-processed WOLEDs, polymer-based WOLEDs, and out-coupling enhancement techniques are summarized in other sections and elsewhere.^550,567,568^ Unless indicated, the device characterization is performed in the forward-viewing mode and without the aid of a light out-coupling structures.

**Figure 72:**
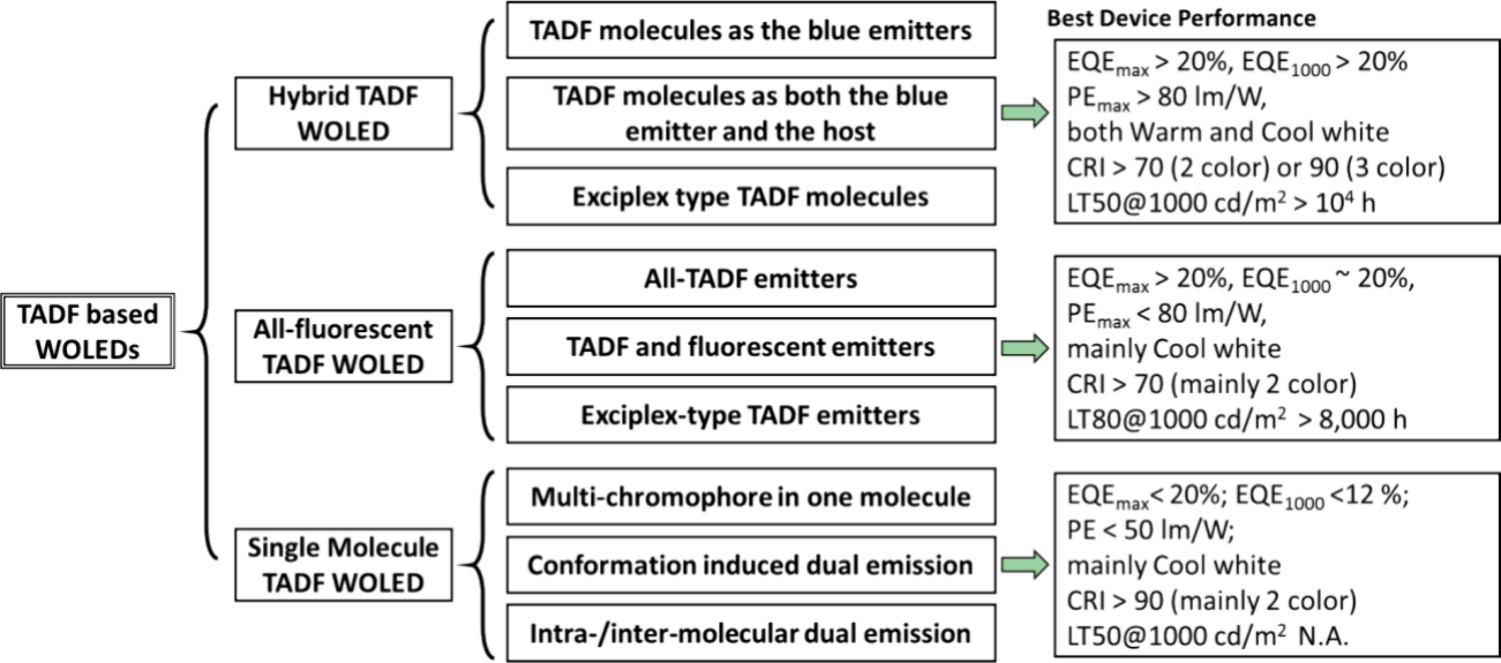
A summary of strategies to achieve TADF-based WOLEDs and their associated performance metrics for the best-forming examples.

### 6.2 Hybrid TADF WOLEDs

The lack of available high-efficiency and stable blue emitters (and stable high triplet energy hosts) remains a bottleneck for high performance WOLEDs and displays.^21^ Even today a hybrid strategy is adopted in industry, in which a stable blue fluorescent or TTA emitter and high-efficiency phosphorescent emitters of complementary colors are deposited inside the EML(s). To harvest all the excitons, the blue fluorescent emitter should have a higher triplet energy level than the phosphorescent emitters to avoid triplet energy trapping and subsequent triplet exciton quenching on the non-harvesting blue emitter. By careful control of the doping concentration, layer thickness, interlayer distance and EML architecture, the singlet excitons of the blue emitter can decay radiatively while its triplet excitons can diffuse to nearby phosphorescent emitters where they are harvested and radiatively decay efficiently. FRET transfer from the blue emitter to other color emitters can also occur, meaning that the balance of emission and overall color are extremely sensitive to dopant concentrations. Despite the desirable performance metrics of hybrid WOLEDs, the complicated device structure and delicate exciton management produce challenges for device fabrication and quality control. In addition, there are a limited number of blue fluorescent emitters that have sufficiently high triplet energy levels to be used within this device architecture, while the use of low-triplet TTA blue emitters only partially alleviated the issue of triplet quenching due to the fundamentally lower IQE limits of the TTA triplet harvesting channel.^569^

High-efficiency blue TADF emitters can not only address the triplet harvesting issue – boosting the EQE of WOLEDs up to or even beyond 20% – but also enable new exciton manipulation strategies and device architectures serving as emitters, sensitizers, and/or hosts. Hybrid TADF WOLEDs with phosphorescent emitters and TADF components already show impressive device performance with reported EQE_1000_ greater than 20%, PE_max_ over 80 lm/W, and the CRI greater than 70 (two-color) or 90 (three-color). The device stability can also be promising despite widespread stability issues for blue TADF emitters and associated high-triplet hosts, with LT_50_ longer than 10^4^ h demonstrated (though for commercial applications typically 20,000 h is required).^565,570,571^ Depending on the specific role of the TADF components in hybrid TADF WOLEDs, devices can be further subcategorised into those with a TADF blue emitter, those with TADF molecules as both the blue emitters and the host, and those with exciplex-type TADF emitters or hosts. The typical molecular structures are shown in Figure 73.

**Figure 73:**
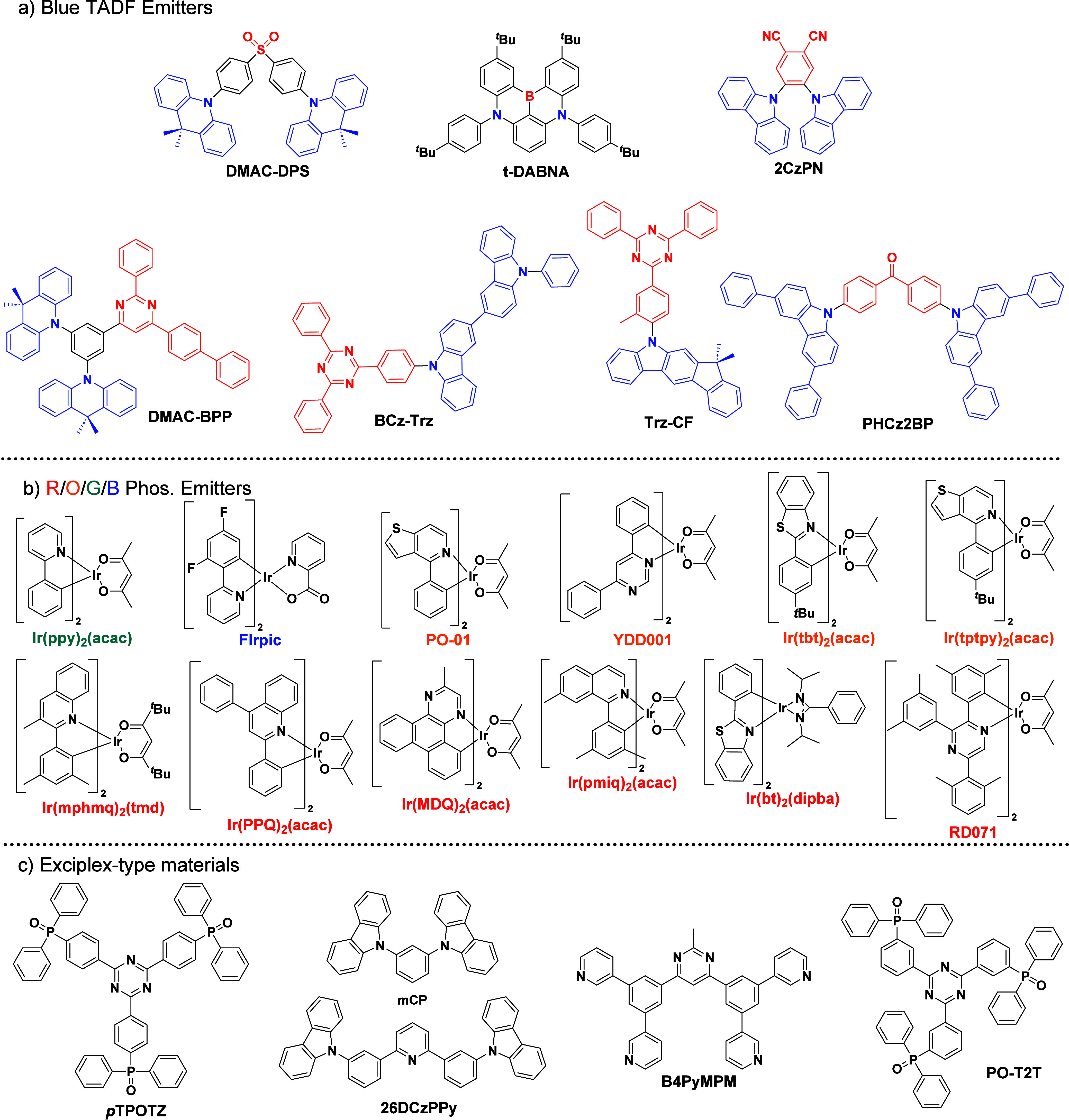
Molecular structures of components used in hybrid TADF WOLEDs: a) blue TADF emitters, b) R/O/G/B phosphorescence emitters, c) exciplex-type materials. In a, the blue color signifies donor moieties/atoms, while the red color signifies acceptor moieties/atoms.

### 6.3 TADF Molecules as Blue Emitters

With high efficiency blue TADF emitters such as **DMAC-DPS**, **2CzPN**, and ***t*-DABNA** (Figure 73), singlet excitons formed by direct charge recombination can either radiatively decay to generate blue prompt fluorescence (PF), or transfer to lower energy phosphorescent emitters by FRET. Meanwhile, triplet excitons either undergo RISC to produce either blue delayed fluorescence (DF) or can diffuse to nearby phosphorescent emitters by a Dexter energy transfer process. In this manner, all the generated singlet and triplet excitons can be harvested, leading to IQEs of up to 100%. Typical phosphorescent emitters used in conjunction with blue TADF emitters in hybrid WOLEDs include red **Ir(MDQ)_2_(acac)**, orange emitter **PO-01**, and green **Ir(ppy)_2_(acac)**. To improve the device performance, much effort has been devoted to designing and optimizing the EML structure for efficient exciton energy transfer, confinement, and distribution; however, studying the energy transfer pathways directly remains challenging due to the number and complexity of processes involved.^104^

#### 6.3.1 Doped Single or Multiple EML

The most direct strategy for white light generation is to dope all the emitters (commonly blue and orange emitters) within a suitable host matrix into one single EML, i.e., S-EML. Through careful control of the doping concentration of each emitter, the extent of energy transfer and thus the ratio of blue and orange emission can be tuned, resulting in white light emission and high EQE. For an efficient exciton harvesting scheme, singlets should be confined to the TADF emitters, or transferred partly from the TADF emitters to the phosphorescent emitters via FRET. Triplet excitons formed on the TADF emitter are either up-converted to singlets by efficient RISC or diffuse to phosphorescent emitters by Dexter energy transfer. Therefore, as previously mentioned, the emission spectrum is exquisitely sensitive to the doping concentrations, which is usually kept lower than 0.5 wt% for the orange emitter to give balanced or warm white emission. As an illustrative example, using this strategy ***t*-DABNA:​PO-01** and **DMAC-DPS:​PO-01** co-doped S-EML WOLEDs were fabricated, showing efficient warm and cool white emission with CIE coordinates of (0.41, 0.47) and (0.33, 0.37), and high EQE_max_ (EQE_1000_) of 19.2% (15%), and 22.4% (18.3%), respectively.^572,573,556^ However, this all-in-one EML strategy with very low doping concentration of one emitter leaves little room for further device optimization.

Multiple emitting layer (M-EML) structures, including directly adjacent doped EML stacks or those separated by interlayers, provide more freedom and control to tune the emission spectrum, confine the excitons, and maintain efficiency and device lifetime. An example of such a WOLED used the TADF emitter **DMAC-DPS** as a sensitizer for the fluorescent blue emitter **TBPe** in a blue ‘hyperfluorescence’ EML, and a yellow emitter **YDD001** in a yellow EML. This WOLED produced PE_max_ approaching 70 lm/W, EQE_max_ (EQE_1000_) of 20% (11.3%), and a device lifetime LT_50_ of over 1500 h at 1000 cd/m^2^.^574^ Although the interlayer connecting the two EMLs was carefully tuned for better carrier balance, the best device still had a poor CRI of only 44. To improve the color quality, a three-color system was explored in another study. This time, WOLEDs comprising one TADF doped blue EML (B) of **DMAC-DPS** and one phosphorescent co-doped EML of **Ir(PPQ)_2_(acac)** (R) and **Ir(ppy)_2_(acac)** (G), demonstrated an EQE_max_ (EQE_1000_) of 23% (17.5%), and a CRI as high as 89.^559^ Due to the well-confined excitons, all M-EML devices showed good color stability at high brightness.

#### 6.3.2 Non-doped Multiple EML

An ultrathin non-doped M-EML structure can alleviate many limitations arising from host material selection, co-evaporation process, and dopant distribution. However, it requires the emitters to show negligible ACQ, and a careful control of the EML thickness. For example, using a **DMAC-DPS** (7 nm)/**PO-01** (0.08 nm)/**DMAC-DPS** (7 nm) M-EML, warm white light devices with CIE coordinates of (0.44, 0.48) were generated with EQE_max_ (EQE_1000_) of 9.1% (7.1%)^575^ Further increasing the number of EML, a seven-layer non-doped M-EML warm WOLED consisting of alternating **DMAC-DPS** (2.5 nm, B), **Ir(MDQ)_2_(acac)** (0.03 nm, R), and **Ir(ppy)_2_(acac)** (0.09 nm, G) layers, was fabricated with CIE coordinates of (0.42, 0.42), EQE_max_ (EQE_1000_) of 19.1% (17.3%), and a high CRI value of 83.^576^ Similarly, a cool white light device with CIE coordinates of (0.26, 0.36) was generated using an ultrathin non-doped phosphorescent layer, **Ir(tbt)_2_(acac)** (0.1 nm, Y), sandwiched between two doped TADF layers, **DPEPO:​DMAC-DPS** (9 nm, B). This device possessed an EQE_max_ of 15.7%, decreasing to 12.1% for EQE_1000_, and a stable EL spectrum at up to 10^4^ cd/m^2^.^577^

### 6.4 TADF Molecules Acting as Both the Blue Emitter and the Host

Blue TADF molecules resistant to ACQ effects (and so maintain high Φ_PL_ in neat films) can serve as both the blue emitter and as a host for phosphorescent emitters in hybrid TADF WOLEDs. Not only does this simplify the EML structure, but it also facilitates direct exciton energy transfer between emitters, enabling improved device efficiency and stability. However, due to the rapid exciton energy transfer of both singlets and triplets from the blue TADF host to the phosphorescent emitters, the EL spectrum of the device once again depends sensitively on the doping concentration of the phosphorescent emitters, which is usually kept below 3 wt%.

Representative of this approach, with a low doping concentration of the orange **PO-01** phosphorescent emitter in the blue TADF molecule **Trz-CF** (0.8 wt%), two-color S-EML WOLEDs showed CIE coordinates of (0.38, 0.45), low efficiency roll-off with EQE_max_ (EQE_1000_) of 20.3% (20.1%), and LT_50_ of over 1,000 h, which was attributed to the balanced bipolar carrier transport and efficient exciton harvesting of **Trz-CF**.^251^ However, the dominant emission at around 560 nm from **PO-01** results in an EL spectrum that deviates from a standard white light source, which can be improved by replacing **PO-01** with another emitter or using a three-color system. With the red phosphorescent emitter, **Ir2** (0.2 wt%) doped in a highly efficient blue TADF molecule, **D-tCz-D-BP**, S-EML WOLEDs showed slightly reduced EQE_max_ of 18.8%, but similar CIE coordinates of (0.41, 0.42), and CRI of around 80.^578^

Iterating this same strategy, a M-EML WOLED was fabricated using red [**Ir(pmiq)_2_(acac)**] and yellow (**PO-01**) phosphorescent emitters doped separately into the blue TADF emitter **DMAC-BPP**. This device showed CIE coordinates of (0.50, 0.42), with EQE_max_ of 15.6% (EQE_1000_ of 14%), and a CRI of 86.^579^ Similar results were reported by using a new blue bipolar TADF molecule **PHCz2BP** as the host for green [**Ir(ppy)_2_(acac)**] and red [**Ir(bt)_2_(dipba)**] phosphorescent emitters. The M-EML warm-white WOLEDs showed CIE coordinates of (0.41, 0.46), high EQE with low efficiency roll-off, i.e., EQE_max_ (EQE_1000_) of 25.6% (25.1%), and CRI of 85.^570^ To simplify the EML structure, co-doping of green [**Ir(ppy)_2_(acac)**] and red [**Ir(mphmq)_2_tmd**] phosphorescent emitters together in the blue TADF molecule **DMAC-DPS** was proposed. S-EML WOLEDs generated efficient cool white light with EQE_max_ (EQE_1000_) of 20.2% (19.4%), CIE coordinates of (0.36, 0.39), and CRI of 85.^580^

### 6.5 Exciplex Type TADF Molecules

Exciplex blends consisting of donor and acceptor molecules are ambipolar by nature, facilitating the transport of both holes and electrons, which is helpful for reducing the carrier injection barrier and balancing bipolar carrier transport in devices. These valuable transport properties – rarely possessed by individual TADF molecules or hosts – can improve device performance, especially in terms of power efficiency and device lifetime. This concept is covered thoroughly in Section 8. Exciplexes can be formed either through the mixing of donor and acceptor molecules (bulk exciplex), or by depositing layers of donor and acceptor molecules on top of each other (interfacial exciplex). By carefully matching the energy levels, balancing ambipolar carrier transport, and optimizing doping concentration (in the bulk exciplex), low turn-on voltage, high PE, and long device lifetime can be achieved in hybrid TADF WOLEDs. Because of the extreme decoupling of CT excitons that can form between exciplex D-A pairs, intrinsically low Δ*E*_ST_ for these materials also often bestows them with TADF and triplet harvesting properties alongside any molecular TADF or phosphorescent dopants.

Wu *et al*. developed a co-doped **mCP:​B4PyMPM** (Figure 73) system, which by itself showed efficient bulk exciplex emission with a high triplet energy and TADF behavior.^571^ S-EML WOLEDs with blue (**FIrpic**, 15 wt%) and orange (**PO-01**, 0.2 wt%) phosphorescent emitters co-doped into the **mCP:​B4PyMPM** host were fabricated, showing PE_max_ as high as 105 lm/W, EQE_max_ (EQE_1000_) of 28.1% (21.5%), and CIE coordinates of (0.40, 0.48). However, the degradation of the warm white color into cool white was observed upon increasing the brightness, indicating an exciton-density dependant bottleneck in energy transfer to the orange emitter.

Besides serving as an efficient ambipolar host, some exciplex-type TADF hosts can directly provide blue emission, which further simplifies the EML structure. The bulk exciplex consisting of a co-doped **mCP:​*p*TPOTZ** layer shows both blue PL and EL emission.^581^ When doping **PO-01** into a **mCP:​*p*TPOTZ** layer, a warm white light was produced with CIE coordinates of (0.43, 0.49), the devices showing EQE_max_ (EQE_1000_) of 24.6% (22%), CRI of 71, and high PE_max_ of 90 lm/W. The EL spectrum was quite stable with increasing brightness.

The use of interfacial exciplexes has also been explored, for example using the **PO-T2T** and **26DCzPPy** double layers.^582^ The interfacial exciplex shows TADF behavior at λ_EL_ of 470 nm. By sandwiching non-doped ultrathin phosphorescent emitters (<0.5 nm) between **26DCzPPy** and **PO-T2T** layers, high efficiency WOLEDs were fabricated. For a 2-color system, **FIrpic** (B) and **Ir(tptpy)_2_acac** (O) emitters separated by 3 nm thick **26DCzPPy** were used to produce a white-emitting device, which has CIE coordinates of (0.46, 0.46), high PE_max_ of 83.2 lm/W, and EQE_max_ (EQE_1000_) of 19.6% (16.5%). In a 3-color system, **FIrpic** (B), **Ir(ppy)_2_acac** (G), and **RD071** (R) were used, which enhanced the CRI from below 60 up to 86.

As the emission of exciplex-based devices alongside their dopants can support improved CRI, this approach was further investigated using a deep-blue emitter **OCT** as an excellent electron acceptor in combination with **TAPC** and **m-MTDATA** as electron donors.^583^ Initially, single color green (λ_EL_ = 524 nm) devices using a **TAPC:​OCT** exciplex and single color orange-red (λ_EL_ = 596 nm) devices using a **m-MTDATA:​OCT** exciplex were fabricated. Due to the small Δ*E*_ST_ of 0.03 eV efficient RISC was achieved, with the **TAPC:​OCT** exciplex-based green devices exhibiting an adequate EQE_max_ of 10.6% suitable for use as a component in WOLEDs. An M-EML system with different exciplex pairs was employed, with **TAPC:​OCT**, **OCT**, **m-MTDATA:​OCT**, and **m-MTDATA** giving green, blue, red, and orange emissions, respectively. Although the resulting WOLEDs possessed a poor EQE_max_ of 1.7%, an impressive CRI of 97 was achieved in these devices.

Another encouraging result was the development of a 3-color tandem WOLED that included two sub-units. One sub-unit incorporated the blue TADF emitter **BCz-Trz** and the red [**Ir(mphmq)_2_tmd**] phosphorescent emitter co-doped into mCP as the host, and the other one employed the yellow (**PO-01**) and red [**Ir(mphmq)_2_tmd**] phosphorescent emitters co-doped into an exciplex host. Without optical extraction structure, this warm-white WOLED showed CIE coordinates of (0.47, 0.45), PE_max_ of 66.3 lm/W, and EQE_max_ (EQE_1000_) of 44.3% (42.3%). With an optical extraction structure, the optical outcoupling and device performance increased significantly, with PE_max_ of 162.9 lm/W, EQE_max_ (EQE_1000_) of 128.1% (126.2%), and CRI of 78. More impressively, a long device lifetime (LT_50_) of 12,600 h was achieved.^565^

In summary, hybrid TADF WOLEDs successfully combine the advantages of both TADF and phosphorescent emitters, showing high performance in terms of efficiency, color quality, and stability. Nevertheless, the scarce and toxic heavy metal component remains an intrinsic shortcoming, which can be addressed by using metal-free all-fluorescent emitters.

### 6.6 All-Fluorescent TADF WOLEDs

The successes of high-efficiency primary color TADF molecules provides an avenue to fabricate high performance WOLEDs without the use of heavy metal complexes, i.e. all-fluorescent TADF WOLEDs. At present, most of the reported examples are simpler two-color systems, consisting of blue and yellow/orange emitters. Depending on the photophysical class of each emitter, all-fluorescent TADF WOLEDs can be subdivided into either all-TADF emitters, or TADF and fluorescent emitters, or exciplex-type TADF emitters. The typical molecular structures are shown in Figure 74, with some fluorescent structures also able to perform TTA in some cases (e.g., rubrene). Strategies used in the hybrid TADF WOLEDs to improve the device performance are also applicable here, such as S-EML, non-doped M-EML, and exciplex-type host. With the availability of an ever-increasing number of TADF emitters, we may soon see high-performance all-fluorescent TADF WOLEDs competitive with phosphorescent ones. The EQE_max_ of fluorescent TADF WOLEDs has indeed already reached and even surpassed the theoretical limit of 20%, with devices that show low efficiency roll-off and maintain EQE_1000_ at around 20%.^584^ However, compared with the hybrid TADF WOLEDs, the efficiency roll-off of all-fluorescent TADF WOLEDs is typically more severe and their larger exciton energies (requiring higher driving voltages) means that reported PE_max_ remains low (below 70 lm W^–1^). In addition, due to the relatively strong blue emission in these two-color systems, the CIE_x_ value is usually below 0.4, implying the generation of a cooler white light. Although long lifetime devices (LT_80_) of over 8,000 h have been reported, more studies are needed to assess and improve the stability of these fully organic all-fluorescent TADF WOLEDs.^566^

**Figure 74:**
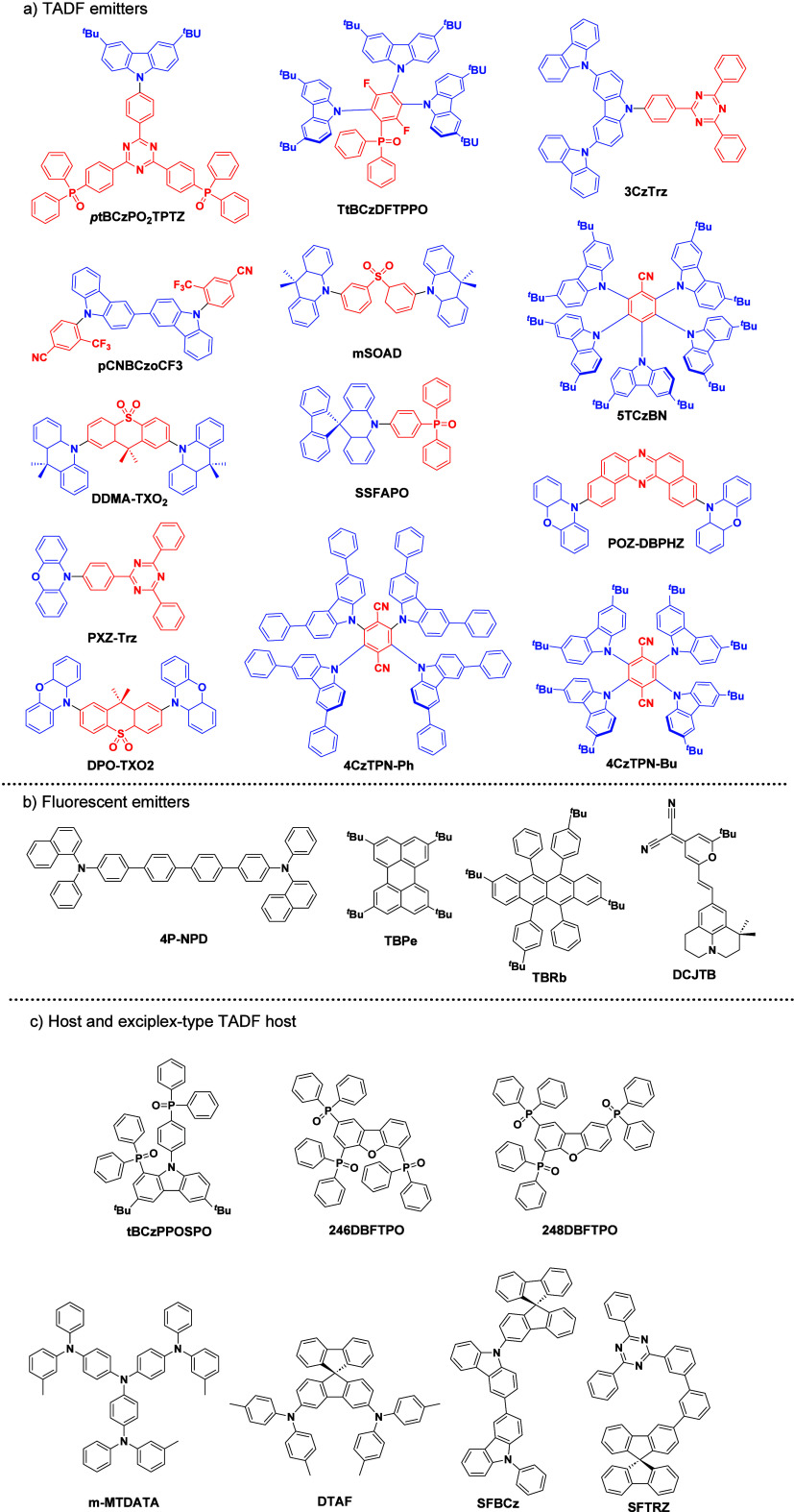
Molecular structures of components used in TADF WOLEDs: a) TADF emitters, b) fluorescent emitters, c) host and exciplex-type TADF hosts. The blue color signifies donor moieties, while the red color signifies acceptor moieties.

### 6.7 All-TADF Emitters

High-efficiency blue and yellow/orange TADF emitters play a key role in all-fluorescent TADF WOLEDs. Here, instead of enumerating all the new molecules and their photophysical properties, which have been discussed in other sections of this review, attention is devoted to the EML structure and the impact of the choice of host material. Although some of these WOLEDs show EQE_max_ of greater than 20%, the severe efficiency roll-off due to the long triplet exciton lifetime remains an issue common with single-color TADF OLEDs. In addition, most of these WOLEDs produce cool white light with low PE_max_ (< 70 lm W^–1^).

S-EML WOLEDs, using **DMAC-DPS** as both a blue emitter and as a host for the orange TADF molecule **4CzTPN-Ph**, were fabricated. With a careful control of the doping concentration of **4CzTPN-Ph** (0.8 wt%), the device showed cool white emission with CIE coordinates of (0.29, 0.39) and reasonable EQE_max_ (EQE_1000_) of 13.4% (9.4%).^585^ With the same emitters but using a doped M-EML structure, i.e. B (DPEPO:​**DMAC-DPS**)/Y (**DMAC-DPS:​4CzTPN-Ph**)/B (DPEPO:​**DMAC-DPS**), the emission spectrum was tuned to pure cool white with CIE coordinates of (0.33, 0.33), EQE_max_ of 12%, and CRI value of 82.^586^ However, the orange emission became stronger with increasing brightness, and in both cases the EQE was low compared to optimised **DMAC-DPS** single-color devices (EQE_max_ ∼ 20%).

Replacing the phenyl groups in the 3- and 6-positions of carbazole in **4CzTPN-Ph** with more sterically demanding *tert*-butyl groups gave **4CzTPN-Bu**, with suppressed intermolecular interactions and improved device performance. In **4CzTPN-Bu:​DMAC-DPS** co-doped S-EML WOLEDs, the effects of phosphine oxide (PO)-based hosts were systematically studied. By carefully modifying the number, position, and symmetry of the PO-group, the triplet energy level and carrier transport properties were tuned, resulting in an improved ambipolar carrier transport, suppressed intermolecular interaction, and enhanced exciton confinement. The EQE_max_ (EQE_1000_) of the WOLEDs employing these PO-based hosts, i.e., **248DBFTPO**, **246DBFTPO**, and ***t*BCzPPOSPO** were 22.2% (19.8%), 21.9% (19.8%), and 21.1% (17.5%), respectively.^587–589^ These devices also showed PE_max_ ranging from 63 to 77 lm W^–1^. Efficient exciton confinement and transfer resulted in controlled white light emission with CIE coordinates of (0.30, 0.40), (0.39, 0.48), and (0.36, 0.44), respectively.

The electron-withdrawing PO group was also explored as the acceptor in blue D-A TADF compounds, such as ***p*tBCzPO_2_TPTZ**, **TtBCzDFTPPO**, and **xSFAPO**, all of which were used giving excellent device performance.^584^ S-EML WOLEDs were fabricated by doping **4CzTPN-Bu** directly into ***p*tBCzPO_2_TPTZ**, and by varying the doping concentration of **4CzTPN-Bu** (*x* wt%), cool white (*x* = 1.5%) and warm white (*x* = 2.0%) devices were fabricated with CIE coordinates of (0.34, 0.36) and (0.41, 0.42), EQE_max_ (EQE_1000_) of 23.6% (20.7%) and 20.3% (15.7%), and CRI of 87 and 73, respectively. Further, co-doping of **4CzTPN-Bu** (1 wt%) with **TtBCzDFTPPO** (80 wt%) – or **4CzTPN-Bu** (0.5 wt%) and **SSFAPO** (30 wt%) – into a DBFDPO host resulted in high-performance S-EML WOLEDs.^590,591^ These devices showed CIE coordinates of (0.28, 0.40) and (0.42, 0.50), EQE_max_ (EQE_1000_) of 22.3% (18%) and 25.1% (20.3%), and PE_max_ of 61.4 and 82.6 lm W^–1^, respectively.

To improve the PE of the WOLEDs, an orange-yellow TADF emitter **DPPZ-DMAC** was designed with DMAC as a donor unit and DPPZ as a strong acceptor unit.^592^ The careful design of the molecule was claimed to suppress sensitizer-sensitizer interactions (SSI), improved the charge transport, and resulted in an efficient up-conversion of triplets transferred from the host. The monochrome OLEDs fabricated with 6 wt% of **DPPZ-DMAC** doped in CBP showed an EQE_max_ of 27.8%. However, the efficiency roll-off was large, especially in devices where the concentration of this dopant was higher. **DPPZ-DMAC** was combined with the blue TADF emitter **2tCz2CzBn** (used as a co-host with mCBP) to produce WOLEDs. The devices showed an enhanced PE_max_ of > 80 lm W^–1^, an impressive EQE_max_ of ∼30%, and warm white emission with CIE coordinates of (0.40, 0.41). Nonetheless, the efficiency roll-off of these WOLEDs was severe, with a drop of the EQE_1000_ to 4.6%.

An attempt was made to reduce the efficiency roll-off of TADF WOLEDs by using compounds with fast *k*_r_ and *k*_RISC_, thus reducing the triplet exciton population. The proposed pyridine-based emitters **PyDCN-DMAC** and **PyDCN-PXZ** have *k*_r_ on the order of 10^7^ s^–1^, and emit in the blue (λ_PL_ = 480 nm), and green (λ_PL_ = 532 nm), respectively.^446^ The Φ_PL_ for the 10 wt% doped film of **PyDCN-DMAC** in PPF is 82.8%, while the 15 wt% doped film of **PyDCN-PXZ** in CBP has a Φ_PL_ of 89.6%. WOLEDs with a CIE of (0.39, 0.44) and CRI of 69 were fabricated using **PyDCN-DMAC** as a blue host and with an orange TADF molecule (**PP-PXZ**) as an emitter. Despite having a low CRI and a PE of 49 lm W^–1^, these WOLEDs showed an EQE_max_ of 18.5% and a L_max_ of 9000 cd m^–2^. The efficiency roll-off was also reduced, where an EQE_1000_ of 12.6% was maintained by minimizing the Dexter energy transfer from **PyDCN-DMAC** to **PP-PXZ** due to an efficient *k*_RISC_ in the host material.

Another approach for reducing the triplet loss via DET in S-EML WOLEDs involves the use of molecules with peripheral methyl substituents that weaken intermolecular interactions and increase intermolecular distances.^385^ The sky-blue TADF emitter **5PCzCN** was designed for this purpose, having a Φ_PL_ of 96.5% and a high RISC efficiency of 99.3%. All-TADF WOLEDs were fabricated using 8 wt% of **5PCzCN** with 0.7 wt% of the orange emitter **4CzTPN-Ph** in a mCP host. The resulting devices showed an EQE_max_ (EQE_1000_) of 20.2% (16.9%), a lifetime LT_50_ of 10,010 h at a luminance of 100 cd m^–2^, but a low PE of 45.8 lm W^–1^. The CIE coordinates of the WOLEDs were found to be very stable with varying luminescence, with Δ(x, y) of only (0.01, 0.01) when increasing from 100 cd m^–2^ (0.31, 0.45) to 10,000 cd m^–2^ (0.30, 0.44). This was attributed to balanced exciton distributions throughout the emission layer.

In addition to two-color systems, three-color (R-G-B or Y-G-B) all-fluorescent TADF WOLEDs have been reported as well. With a doped M-EML structure based on **4CzTPNPh** (O), **4CzPN** (G), and **3CzTRZ** (B) or **POZ-DBPHZ** (Y), **DPO-TXO2** (G), and **DDMA-TXO2** (B), M-EML WOLEDs were fabricated showing CIE coordinates of (0.30, 0.38) and (0.30, 0.40), EQE_max_ (EQE_1000_) of 17.1% (8.1%) and 16.1% (11%), respectively.^593,594^

To enhance the efficiency of carbazole-based TADF emitters, the number of carbazole groups on a molecule can be increased, in some cases leading to efficient RISC, enhanced excited-state mixing, and a delocalized HOMO across the carbazoles. This, however, can simultaneously lead to a randomised (more isotropic) orientation of molecules in the film and thus a lower light outcoupling efficiency. An alternative approach was proposed where a series of **CzBN**-based molecules with only two donors and a π-extended acceptor were designed to maintain a strongly horizontal orientation of the TDM.^595^ Amongst the emitters in the study, **2PCzBN-FPh** possessed the highest Φ_PL_ of >90% and most strongly aligned horizontal TDM. As a result, blue OLEDs showed a EQE_max_ (EQE_1000_) of 35.7% (24.3%) at λ_EL_ of 469 nm. Due to these exceptional properties, **2PCzBN-FPh** was used as a host in M-EML WOLEDs (two-color and three-color devices). The two-color devices used an orange MR-TADF emitter **CNCz-BNCz** and showed strong EQE_max_ of 29.3%, but poor CRI of 65 in this case hindered by the narrowband MR-TADF emission.^486^ To improve the CRI, a three-color system with **2PCzBN-FPh** as a blue TADF host, a green-yellow TADF emitter **4CzTPN-tBu** along with a fluorescent red emitter **RD** were used. The CRI improved to 83 with CIE coordinates of (0.39, 0.41), although a lower EQE_max_ of 21.1% was obtained.

Red/yellow emitting TADF compounds containing more than one acceptor (A-D-A), such as **DTXO-PhCz2**, **DTXO-PhCz4**, **DTXO-TPA2** and **DTXO-TPA4**, have been used as components in WOLEDs.^596^ Among these emitters, the device with **DTXO-TPA2** showed the best performance, with EQE_max_ (EQE_1000_) of 25.0% (10.06%), PE_max_ of 77.7 lm W^–1^ and a LT_50_ of 1392 hrs at 1000 cd m^–2^. The high efficiency of this device was attributed to the Φ_PL_ of 70% of the emitter, good charge balance within the EML, and most importantly a strongly horizontally oriented TDM of **DTXO-TPA2**. The WOLEDs were made by combining **DTXO-TPA2** with the blue TADF emitter **2SPAc-MPM**, and the devices showed an EQE_max_ of 18.0% at CIE coordinates of (0.31, 0.31) with a CRI of 85.

### 6.8 TADF and Fluorescent Emitters

Despite the reduced IQE_max_ of around 25%, OLEDs using fluorescent emitters show high chemical/electrical stability and high brightness, owing in part to their chemical structures, low triplet energies, fast singlet radiative rates, and high Φ_PL_. These fluorescent emitters can be used in combinations with the TADF emitters to form M-EML WOLEDs with high stability and low efficiency roll-off. However, the low triplet energy states of the fluorescent materials can result in quenching of the triplet excitons of the TADF material. One of the strategies to solve the triplets and energy transfer losses is by the addition of an interlayer between the fluorescent and TADF emitters so that the excitons can be harvested adequately in their respective channels.^597^ The interlayers of mCBP doped with different concentrations of Bepp2 were investigated where 30 wt% of Bepp2 presented the best results. A M-EML WOLED with two-color system but with double yellow EML was fabricated to better manage the exciton and charge distribution. For the first yellow EML, a fluorescent emitter 0.4 wt% **TBRb** with 6 wt% **4CzPN** as a TADF assistant host doped in mCBP was used, while the second yellow EML contained 0.8 wt% **TBRb**:​10 wt% **4CzPN** in mCBP. For blue emission a fluorescent emitter DSA-Ph with 5 wt% in MADN host was used. The WOLEDs showed the highest EQE_max_ (EQE_1000_) of 15.1% (12.1) among all devices with CIE coordinates (0.35, 0.49) however, due to the absence of a red emitter, the CRI value of the WOLEDs was low (49). Hence, a three-color system was adopted where the first yellow EML was replaced with a red fluorescent emitter 0.4 wt% **DBP**:​6 wt% **CzPN** in mCBP host. A moderate CRI of 68 with an EQE_max_ (EQE_1000)_ of 14.7% (10.8%) was achieved.

Another approach involves a careful co-doping of TADF and fluorescent emitters into the EML, triplet excitons can be efficiently harvested on the TADF, resulting in enhanced device efficiency whilst maintaining good device stability. Long device lifetime WOLEDs have been achieved using this ‘hyperfluorescence’ strategy by balancing the completeness of FRET transfer from for example a blue TADF emitter to an orange or red fluorescent dye. However, as the lower energy dopant may not have any triplet harvesting properties, DET to this species as well as direct recombination must be avoided, enforcing low co-doping ratios.M-EML WOLEDs containing one co-doped EML with a fluorescent yellow emitter, **TBRb**, and a green TADF molecule, **PXZ-TRZ** in **SF4-TPE** as the host, alongside another doped EML of a fluorescent blue emitter, **4P-NPD** in **SF4-TPE** as the host, showed CIE coordinates of (0.39, 0.39) and EQE_max_ (EQE_1000_) of 17.7% (15.5%). The CIE coordinates varied little between 300 to 13,000 cd m^–2^ [Δ(0.001, 0.012) for one of the systems], implying good color stability.^598^ With the same fluorescent yellow emitter, **TBRb** and co-doped with the TADF blue emitter, **5TCzBN** in an exciplex-type TADF host (**SFBCz:​SFTRZ**), two-color S-EML WOLEDs showed CIE coordinates of (0.40, 0.51), EQE_max_ (EQE_1000_) of 21.7% (21.4%), PE_max_ of 78 lm W^–1^, and a long lifetime (LT_80_) of over 8200 h.^566^ The long device lifetime was attributed to the advantages of both the exciplex-type host (bipolar carrier transport, TADF-type triplet harvesting) and the chosen emitters (inherent stability of fluorescent emitter, efficient triplet exciton harvesting of the TADF emitter).

In contrast to the low doping concentrations approach, the use of an ultrathin (< 1 nm) host-free blue fluorescent layer of **TBPe** and a TADF sensitizer assisted yellow fluorescent layer of **TBRb** with high concentration (3 wt%) was investigated.^599^ The proposed system supported an efficient exciton harvesting by avoiding the dexter energy transfer to the blue emitter from the TADF host due to spatial separation while to the yellow emitter due to the large triplet gap. Two molecules **DCzSPOTz** and **PhCzSPOTz** were synthesised to be used as the hosts for the quasi-bilayer HF EML system. The resulting M-EML WOLEDs with **PhCzSPOTz** host showed an EQE_max_ (EQE_1000_) of 20.9% (17.7%), a high PE_max_ (PE_1000_) of 78.3 lmW^–1^ (38.0 lmW^–1^) with a CIE of (0.40, 0.52) and CCT of 4000K. Despite using an efficient approach, the devices failed to achieve an EQE higher than 20% which indicated that the triplet diffusion was still occurring in the system.^600^ Hence, a HF system with very low yellow dopant concentrations was readopted for making efficient WOLEDs and was termed as a triplet-free exciton allocation system. Three TADF emitters ***p*tBCzPO_2_TPTZ**, **2CzPN**, and **DMAC-DPS** were used for blue emission as well as sensitizers with a commonly used yellow fluorescent emitter **TBRb**. The WOLEDs with DBFDPO as a host and 40% ***p*tBCzPO_2_TPTZ** and 0.1% **TBRb** showed an impressive EQE_max_ (EQE_1000_) of 30.7% (27%), PE_max_ (PE_1000_) of over 100 lmW^–1^ (65 lmW^–1^) at CIE of (0.31, 0.37).

In a separate strategy that is already well-proven for white inorganic LEDs in industry and commercial applications, orange or green fluorescent emitters entirely external to the OLED can be used as partial down-conversion layers to produce white light from otherwise unaltered blue OLEDs. This approach was demonstrated for a blue TADF emitter, **DMAC-TXO2** in DPEPO host, with layers of a polymer doped with green or orange perylene diimides spin coated directly atop the device.^601^ The overall color could be controlled by the number of layer depositions, although with some complexity due to the radiative rather than FRET energy transfer between the OLED and external dyes. As the perylene dyes were external to the device, they completely avoid any formation of triplet excitons, with only the TADF emitter electrically excited. The balanced white WOLED itself maintained the good performance of the underlying blue OLED, with EQE_max_ of 17% and PE_max_ of 24.3 lm W^–1^, while also exhibiting perfect color stability at different driving voltages and CRI of 80.

### 6.9 Exciplex-Type TADF Emitters

Exciplex-type TADF emitters not only have low carrier injection barriers and balanced carrier transport but can also show efficient light emission properties. By carefully matching the energy levels of donor and acceptor molecules, exciplex-type TADF emitters can generate emission in the whole visible light range.^186^ As previously mentioned, both bulk and interfacial exciplexes have been investigated to fabricate high performance WOLEDs. Though many other exciplex-type TADF emitters have been reported, the device performance using exciplex TADF emitters lags far behind other types of WOLEDs. In addition, due to the high exciton energy, high-efficiency blue exciplex-type TADF emitters are quite limited. Nonetheless, exciplex-type TADF emitters can be used along with fluorescent emitters or TADF emitters as documented above.

Doped layers of **mCP:​PO-T2T** and **DTAF:​PO-T2T** show exciplex-type TADF behavior with blue and orange emission, respectively. With a tandem device structure, WOLEDs were fabricated with CIE coordinates of (0.29, 0.35) and EQE_max_ (EQE_1000_) of 11.6% (10.5%).^554^ It has also been demonstrated that some blue TADF emitters can form interfacial exciplexes with the adjacent organic layer, resulting in orange light emission and simplified device structure. Both **mSOAD** and ***p*CNBCzoCF3** are efficient blue TADF emitters. When their non-doped layers are in contact with **PO-T2T** or **m-MTDATA** layers, respectively, orange interfacial exciplex-type emission is observed. **mSOAD**-based WOLEDs showed CIE coordinates of (0.49, 0.47) and EQE_max_ (EQE_1000_) of 11.6% (9.6%),^602^ while ***p*CNBCzoCF3**-based WOLEDs showed CIE coordinates of (0.40, 0.44) and EQE_max_ (EQE_1000_) of 18.8% (17%).^603^

Summarising the previous categories and examples, all-fluorescent TADF WOLEDs not only have a simpler EML structure, do not contain heavy-metal emitters, but are also showing improved device performance with examples of devices with EQE_max_ higher than 20%, PE_max_ approaching 80 lm/W, and LT_80_ of over 8200 h. However, due to the complicated exciton dynamics and long triplet excitons persisting in the EML, attention and progress is still required to further improve the efficiency roll-off, power efficiency, CRI, and color stability.

### 6.10 Single Molecule TADF WOLEDs

For even more simplicity in device design, it is desirable to achieve white light from single molecules.^557,556^ The most direct approach to achieve white emission is to integrate multiple chromophore units into one polymer chain. For individual small molecules this white emission property is typically rare, and at least dual-emission of blue and yellow/orange is needed. Nonetheless this can still be achieved by three main approaches: multiple chromophores within in one molecule; conformation induced dual-emission; and intra-/inter- molecular dual-emission. Examples of such molecules are shown in Figure 75.

**Figure 75:**
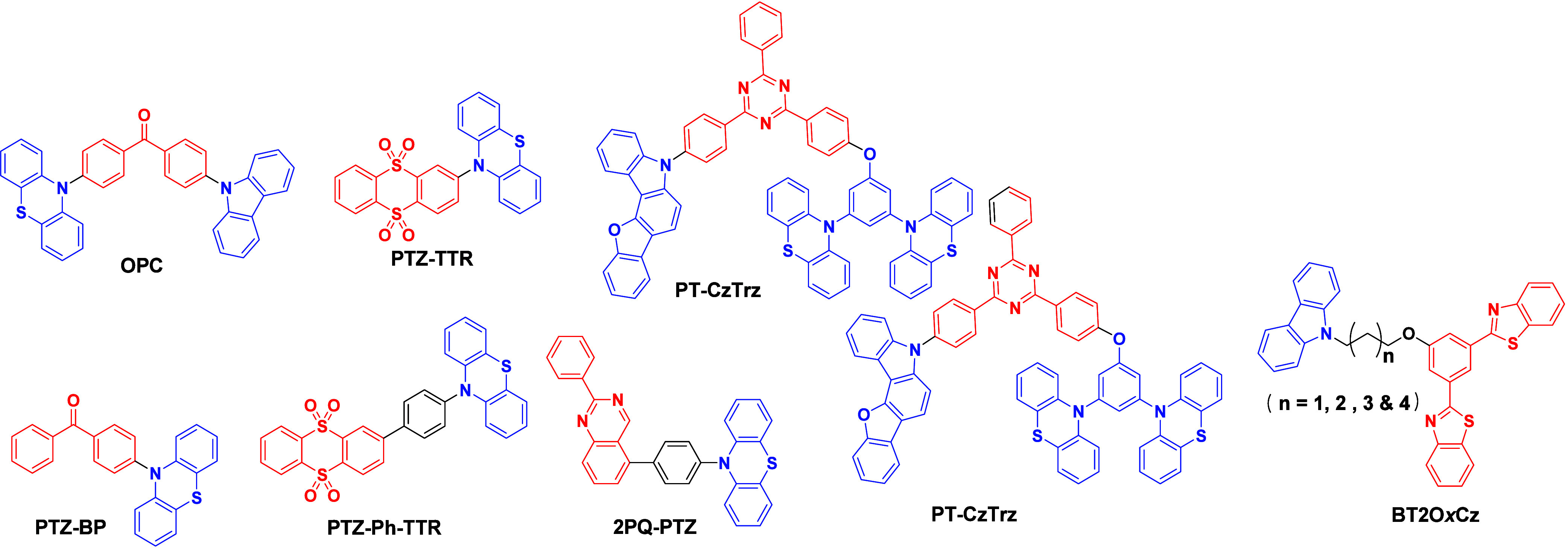
Molecular structures of emitters used in single emitter material TADF WOLEDs (the blue color signifies donor moieties, while the red color signifies acceptor moieties).

With an asymmetric D-A-D’ molecular design, a butterfly-shaped dual-emission white light emitter **OPC** was designed and synthesized.^604^ When the Cz and PTZ donors are connected to a common benzophenone acceptor (BP), different CT states are formed, giving simultaneous blue and yellow emission from the bulk material. Though the blue component is fluorescent, delayed fluorescence was observed from the yellow emission. Under optical excitation, cool white emission with CIE coordinates of (0.35, 0.35) was observed from **OPC**, although no devices were fabricated in the report.

The strongly electron-donating PTZ can adopt two different conformations, quasi-axial or quasi-equatorial, which results in emission from different states that can be used to generate dual-emission. The **PTZ-TTR** molecule adopts both planar and orthogonal conformations, generating fluorescent blue and TADF-type yellow emissions, respectively.^564^ By doping **PTZ-TTR** into CBP as the host, S-EML WOLEDs were fabricated with pure cool white emission with CIE coordinates of (0.33, 0.33) and a high CRI value of 92; however, EQE_max_ of the device was less than 3% and the EL spectrum was unstable. With a phenyl linker inserted between the PTZ and TTR moieties, **PTZ-Ph-TTR** preferentially adopts the orthogonal conformation, leading to greater TADF-type yellow emission that resulted in warm white light with CIE coordinates of (0.41, 0.48). The EQE_max_ of the device was significantly increased to 16.3% (EQE_1000_ = 11%), though the CRI value was lower at 64. Ultimately, this example reveals that the simplicity of single-molecule WOLEDs is also somewhat offset by a lack of control over their color.^564^

**PTZ-BP** likewise shows dual-emission consisting of blue fluorescence and yellow TADF-type emission from LE and ICT states, respectively. Doping **PTZ-BP** into a DCzDPy host, S-EML WOLEDs showed CIE coordinates of (0.34, 0.46) and EQE_max_ (EQE_1000_) of 6.2% (2.8%).^605^ With a similar design using a quinazoline (PQ) acceptor, the emitter **2PQ-PTZ** shows white light emission (blue fluorescence and orange TADF emission emanating from quasi-axial and quasi-equatorial conformations, respectively).^606^ Doping of **2PQ-PTZ** into mCP, the S-EML WOLEDs produced cool white light with CIE coordinates of (0.32, 0.34), a CRI of 89, and an EQE_max_ of 10.1%.

Reiterating, despite their promise it is often difficult to tune the emission spectra of single molecule white light emitters. One solution is to simultaneously exploit both intramolecular and intermolecular CT emissions. An example of a molecule that does this is **PT-CzTrz**, which contains a twisted donor-acceptor moiety (**pBFCz-Trz**) responsible for blue emission and an electronically decoupled stronger donor moiety (**mPTZ**) that interacts intermolecularly with a second molecule of **PT-CzTrz** to produce a yellow-emitting exciplex (Figure 75)^607^ By varying the doping concentration, the relative contributions of the blue and yellow components was tailored effectively. However, the device performance was still poor with CIE coordinates of (0.25, 0.31) and EQE_max_ of less than 2%.

In another approach, a series of emission-tunable molecules (**BT2O*x*Cz**, *x* = 3, 4, 5, 6, where *x* refers to the number of aliphatic carbons) were designed with higher lying singlet and triplet states (Figure 75).^556^ The molecules contained a Cz donor and a BT2 acceptor connected through non-conjugated alkyl chains, and the emission color could be tuned by altering the length of the connecting alkyl chains. Here, the **BT2O6Cz** molecule is of the most interest as it provides a combination of TADF, room temperature phosphorescence and J/H-aggregates that emit in the blue, green, and red, respectively. The CIE coordinates for all the emitters are near pure white light emission (0.33, 0.33); however, the devices were not fabricated.

While still a developing area, dual-emissive single molecule white light emitters have shown great progress in recent years. This progress can be attributed in most cases to novel molecular design of different intra-/intermolecular CT states, conformation states, exciplex, and aggregate states. However, the overall device performance is still far behind other WOLED strategies, for the minority of examples where devices are demonstrated. Nonetheless, the appeal of massively simplified device design makes this an area of both practical and fundamental interest.

### 6.11 Outlook

In summary of this section, the device performance of WOLEDs using TADF materials as emitters, host, or sensitizers has significantly improved in efficiency, color quality and stability since their first reports in 2004.^593,608,556,554^ The hybrid TADF WOLEDs that show the best performance in terms of efficiency (up to ∼40% EQE_max_), device lifetime, and CRI frequently rely on phosphorescent molecules doped in exciplex TADF hosts. All-fluorescent TADF WOLEDs show promise to have long device lifetime and are more environmentally friendly than hybrid TADF WOLEDs, though their power efficiencies and color quality still must improve to challenge phosphorescent devices. Single molecule WOLEDs are attractive as their device structures are significantly simpler; however, their efficiencies are the poorest of the WOLEDs that employ a TADF component in the EML, and are difficult to optimize from a given material structure. To further improve device performance, especially if organometallic phosphorescent co-dopants are to be avoided, judicious molecular design for high-performance TADF emitters as well as efficient exciton management are clearly still needed. Additionally, more insight and understanding of the degradation processes within WOLEDs to clarify the underlying mechanisms will help to improve the device lifetime towards industry requirements.

However, considering the technological underpinnings of WOLED use in displays and lighting, we predict that there will be a considerable decline in dedicated WOLED research in the coming years. This is because as the performance of monochromatic blue OLEDs continues to improve, WOLEDs will directly benefit in parallel. In the display industry this follows as a result of the ‘blue backplane’ concept,^21^ using emissive color filters to achieve other colors from exclusively blue subpixel excitation. In lighting applications only blue and orange emission are required, which is once again most simply achieved through the use of external color downconversion filters,^601^ which are already highly efficient. In both cases it therefore follows that the most impressive gains for WOLEDs can be achieved by exclusively focussing research on the underlying blue emitter, allowing simpler and longer-lived device architectures to be used inside the display or luminaire, and relying on photonic materials to generate other colors. Indeed, this is the currently dominant paradigm for now-widespread inorganic LED lighting, which has significant advantages over OLED in terms of efficiency, lifetime, and production cost. Apart from displays, which require small subpixels, and niche applications like aeronautical engineering, where weight is a critical concern, it seems unlikely that WOLEDs will be able to displace this now well-established technology.

## 7 Circularly Polarized Luminescence in TADF Emitters

### 7.1 Introduction

With the primary goal of increasing light output from the OLED, researchers have been focused not only on optimizing the intrinsic photophysics of the emitters but also devoting efforts to sidestep losses arising from external anti-glare polarising filters that are necessary in many display applications. Once such strategy is to employ materials that emit preferentially right- or left-circularly polarized emission. Indeed, circularly polarized luminescence (CPL) is the manifestation of preferential right- or left-circularly polarized emission emanating from materials that are either chiral or are influenced by their chiral environment. Chiral molecules emitting CPL have been widely investigated for their potential integration in optical data storage^609^ and optical spintronics applications.^610^ This class of emitters has generated significant interest for their use in electroluminescent displays such as circularly polarized OLEDs (CP-OLEDs) with the promise of mitigating the significant efficiency losses associated with the presence of ‘anti-glare’ filters.^611^ Many display technologies employ circular polarizing filters (a linear polarizer and a quarter-wave plate) to trap and attenuate reflections of surrounding unpolarized (randomly polarized) light sources (e.g. sunlight) that can otherwise cause glare.^612^ This, however, also unavoidably blocks 50% of the unpolarized electroluminescence from exiting the display. CPL though can pass through such filters without loss, potentially doubling the external quantum efficiency and achievable brightness of these OLEDs while still preventing glare.^47,613,614^

The extent of CPL from a chiral emitter is quantified by the luminescence dissymmetry factor, g_lum_ or g_PL_, which is defined in equation 16:

16 $$g_{\mathrm{PL}}=2\left(\frac{I_{L}-I_{R}}{I_{L}+I_{R}}\right)$$

where *I_L_* and *I_R_* are the intensities of left- and right-handed light, respectively. Thus, g_PL_ values can range from −2 to +2 for perfectly right- or left-CP emission, respectively, and 0 for unpolarized or linearly polarized light. For CP-OLEDs the equivalent electroluminescence dissymmetry factor (g_EL_) is used, which is defined analogously to g_PL_.

The molecular origin of the emission dissymmetry is related to the relative orientation of the electric and magnetic transition dipole moments for the emissive transition, as defined in equation 17:

17 $$g_{PL}=\frac{4|\mu ||m|}{{\left|\mu \right|}^{2}+{\left|m\right|}^{2}}\cos\;\theta$$

where ***μ*** and ***m*** are the respective electric and magnetic transition dipole moments between the excited and ground states (usually S_1_ and S_0_) and *θ* is the angle between the vectors of these TDMs. In closed shell systems like organic TADF emitters the electric transition dipole moment is typically large while the magnetic transition dipole moment is usually ∼100-fold smaller, and so CPL-active small organic chiral molecules often show low g_PL_ values typically less than 10^–2^, limiting their practical applications. Much effort has been devoted to rationally design materials to tune the magnitude of ***μ*** and ***m*** to improve g_PL_ at the molecular level.^615–618^

In the context of CP-OLEDs, not only should the device show high g_EL_ but the intrinsic EQE must also remain competitively high. Consequently, chiral compounds that can also support triplet harvesting through TADF are an especially appealing class of emitters.^619,620^ We identify two key strategies used to construct CP-TADF molecules: (1) the design of molecules with an intrinsically chiral TADF skeleton (using point, axial, or planar chirality), or (2) the design of compounds that couple chiral groups to achiral TADF moieties (chiral perturbation). A number of recent reviews focusing on CP-TADF molecules have been published,^47,613,614^ and so here we highlight recent developments in CP-TADF emitter design. Key photophysical data of these chiral emitters are summarized in Table S4.

### 7.2 CP-TADF Emitters Containing Stereogenic Centers

The first example of a small molecule TADF CPL emitter, **DPHN** (Figure 76), was developed by Imagawa, Hirata, *et al*. in 2015.^621^ This compound contains a stereogenic carbon center linking the donor and the acceptor moieties. This molecule emits at λ_PL_ of 513 nm and has a moderate Δ*E*_ST_ of 0.26 eV, a g_PL_ of 1.1 × 10^–3^, and has a low Φ_PL_ of only 4% and a τ_PL_ of 13.9 ns in toluene. **DPHN** also has a small Φ_PL_ of 26% and a moderate Δ*E*_ST_ of 0.19 eV in 9 wt% doped mCP films. Understandably from these low Φ_PL_, no CP-OLEDs were reported.

**Figure 76:**
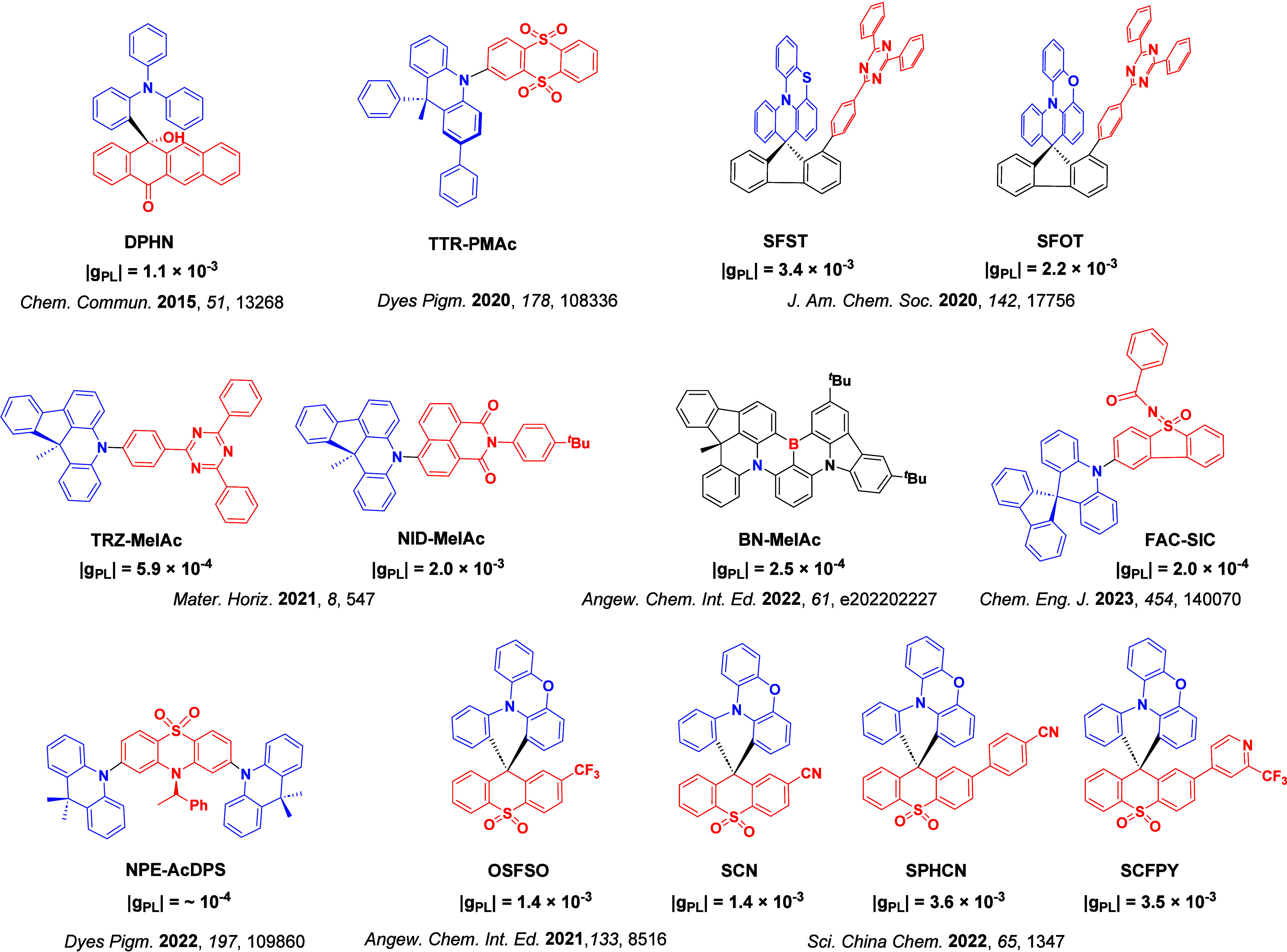
Structures of CP-TADF emitters containing stereogenic centers and their respective |g_PL_| (the blue color signifies donor moieties, while the red color signifies acceptor moieties).

Using a similar strategy Hao *et al*. reported emitters **(*R*)**- and **(*S*)-TTR-PMAc** (Figure 76) containing chiral donor units, (*R*)- and (*S*)-9-meth­yl-2,9-di­phen­yl-9,10-di­hy­dro­acri­dine (PMAc) linked to achiral acceptor thi­an­threne 5,5,10,10-tetra­oxide.^622^ This emitter exists in two distinct conformations, one that is near-planar and the other near-orthogonal, with associated calculated dihedral angles between the TTR and either (*R*)- or (*S*)-PMAc units of 173.46° and 85.57° respectively. Interestingly, it was demonstrated that in both enantiomer the CPL signals from the near-planar and near-orthogonal conformations showed dissymmetry factors of opposite sign. Both enantiomers display two broad and structureless emission bands at λ_PL_ of ∼430 and 577 nm. **(*R*)-TTR-PMAc** and **(*S*)-TTR-PMAc** have similar large Δ*E*_ST_ of 0.36 and 0.39 eV, respectively for their near-planar conformers in 2-MeTHF. In contrast, only the orthogonal conformers are observed in neat films, which have much smaller associated Δ*E*_ST_ of 0.02 and 0.05 eV, respectively.

Ni *et al*. later introduced a chiral rigid donor MeIAC, which was coupled to a triazine acceptor to give the sky-blue emitter **TRZ-MeIAc** (Figure 76). This material emits at λ_PL_ of 473 nm with a Φ_PL_ of 89%, Δ*E*_ST_ of 0.19 eV, and a τ_d_ of 82.3 μs in 12 wt% doped films in mCPCN.^623^ The same chiral MeIAc unit was also coupled to a naphthalimide acceptor in the orange emitter **NID-MeIc**. This compound emits at λ_PL_ of 565 nm, has a Φ_PL_ of 86%, a Δ*E*_ST_ of 0.22 eV, and thus a longer τ_d_ of 235.4 μs in 6 wt% doped films in mCPCN. The high Φ_PL_ of these two emitters was attributed to the rigid molecular structure of the donor. CP-OLEDs with **TRZ-MeIAc** showed an EQE_max_ of 20.3%, while the device with **NID-MeIc** showed an EQE_max_ of 23.7%. The CP-OLED based on **(*R*)-TRZ-MeIAc** showed definite CPL although with a low g_EL_ of 6.4 × 10^–4^, while the device based on **(*S*)**-**NID-MeIc** displayed a fourfold larger g_EL_ of −2.4 × 10^–3^. It is fascinating, although entirely unclear, how the same chiral donor group can lead to significantly different CPL dissymmetry for the different emitters.

Subsequently, Yang *et al*. integrated the same MeIAc block into a B/N-doped aromatic skeleton to develop a pair of chiral green emitters **(*R*)-BN-MeIAc** and **(*S*)-BN-MeIAc** (Figure 76), which featured an MR-TADF design strategy where the CPL properties originate from the chiral carbon centre.^624^ The sp^3^-hybridized carbon atom in the structure not only serves as a configurationally stable stereocenter to induce CPL, but also locks the molecular geometry to guarantee high conformational stability. In addition, the fluorenyl unit within **MeIAc** extends the π-conjugation of the MR-TADF skeleton, which contributes to the simultaneous enhancement of the oscillator strength and the horizontal transition dipole orientation of the emitter in the devices. As a result of this rational design, **BN-MeIAc** displayed narrowband green emission with λ_PL_ of 497 nm, FWHM of 30 nm, g_PL_ of +2.5 × 10^–4^ for **(*R*)-BN-MeIAc** and −2.5 × 10^–4^ for **(*S*)-BN-MeIAc**, and a small Δ*E*_ST_ of 0.11 eV for both, all in toluene. These desirable photophysical properties also included a high Φ_PL_ of 96%, a moderate τ_d_ of 28.1 μs, and a highly horizontal orientation of the TDM of 90% in 1 wt% doped films in DMIC-TRZ. The corresponding OLEDs showed EQE_max_ values up to 37.2%, although still with modest g_EL_ of +2.7 × 10^–4^ for **(*R*)-BN-MeIAc** and −2.9 × 10^–4^ for **(*S*)-BN-MeIAc**, presumably limited by the intrinsic g_PL_ of the emitters. This work expanded the application of the chiral acridan-derived building block used in chiral MR-TADF emitters, and although it also represents the highest device efficiency for all reported CP-OLEDs to date, it also highlights the need for greatly improved intrinsic molecular CPL properties to support higher g_EL_.

Yang *et al*. reported the first examples of through-space charge transfer (TSCT) CP-TADF emitters, **SFST** and **SFOT** (Figure 76), containing either a PTZ or a PXZ donor attached alongside a triazine acceptor on a spiro-fluorene scaffold.^625^ Both compounds showed a small Δ*E*_ST_ of 0.05 eV and emit at λ_PL_ of 512 nm in toluene. The subtle difference in the structure of the donor brought about considerable changes in the secondary photophysical properties of the molecules though. A higher Φ_PL_ of 89% and a much faster *k*_RISC_ of 1.17 × 10^5^ s^–1^ was observed for **SFOT** in 30 wt% doped films in mCBP, which led to devices with an EQE_max_ of 23.1% and EQE_1000_ of 21.3% (λ_EL_ at 508 nm). The larger sulfur atom in **SFST** instead distorted the molecular backbone of PTZ and altered the donor-acceptor distance with negative consequences on the TSCT interaction. This substitution resulted in a lower Φ_PL_ of 53% and slower *k*_RISC_ of 9.93 × 10^4^ s^–1^ in 30 wt% doped films in mCBP, which translated into a device with a lower EQE_max_ of 12.5% (λ_EL_ at 508 nm). Both enantiomers of **SFST** presented higher |g_PL_| values than those of **SFOT**, up to 4.0 × 10^–3^ in toluene; in fact, they are almost double those of **(*S*)-SFOT/(*R*)-SFOT** (|g_PL_| up to 2.2 × 10^–3^). The increased CPL character was attributed to the large atomic radius of sulfur and consequently the more distorted and asymmetric structure of **SFST**. The CP-OLEDs based on **(*S*)-SFST** and **(*S*)-SFOT** showed g_EL_ of 1.30 × 10^–3^ and 1.0 × 10^–3^, respectively.

Zhang *et al*. reported a similar example of a CP-TADF emitter containing a rigid spiro structure, **(*R*)/(*S*)-OSFSO** (Figure 76).^626^ The molecule possesses a similar PXZ-based donor motif as **SFOT**, while the acceptor thioxanthene moiety was linked directly to the donor across a spiro-center bridging atom. **(*Rac*)-OSFSO** has a small Δ*E*_ST_ of 0.022 eV leading to a τ_d_ of 4.7 μs, emits at λ_PL_ of 470 nm, and has a Φ_PL_ of 81% in 25 wt% doped films in DPEPO. The CP-OLEDs fabricated with both enantiomers showed not only an EQE_max_ of 20.0% (λ_EL_ of 472 nm), but also featured a remarkably low efficiency roll-off with an EQE_1000_ of 19%. The device g_EL_ was 3.1 × 10^–3^, again small relative to application-relevant values but also somehow double the reported g_PL_ (1.4 × 10^–3^ in toluene).

Hao *et al.* reported the first CP-TADF emitters containing heteroatomic stereocentres.^627^ By combining sulfoximine-based acceptors and acridan-based donors within a highly twisted structure, a pair of chiral enantiomers [**(*R*)-FAC-SIC** and **(*S*)-FAC-SIC**, Figure 76] were synthesized with the asymmetric sulfur atom serving as the stereocenter. The strongly twisted geometry facilitates a small Δ*E*_ST_ and TADF, while intramolecular hydrogen bonding in the SIC acceptor helps to reduce non-radiative decay pathways by rigidifying the *N*-substituent. As a result, **FAC-SIC** emits at λ_PL_ of 507 nm and has a small Δ*E*_ST_ of 0.075 eV in toluene, and a high Φ_PL_ of 99% and a short τ_d_ of 5.8 μs in 10 wt% doped films in DBFPO, as well as g_PL_ of +2.4 × 10^–4^ for **(*R*)-FAC-SIC** and −2.0 × 10^–4^ for **(*S*)-FAC-SIC** in toluene, respectively. The corresponding OLEDs with **(*R*)-FAC-SIC** showed an EQE_max_ of 28.5%, although the CPL signal was too weak to be detected.

Similar to having sulfur as the stereocenter, Huang *et al*. reported a pair of enantiomers, **(*S*)-NPE-AcDPS** and **(*R*)-NPE-AcDPS** (Figure 76), that contained the commercially available chiral (*S*)-/(*R*)-1-phenylethylamine linked to the previously reported TADF emitter **DMAC-DPS**.^628^ **(*S*)-NPE-AcDPS** emits at λ_PL_ of 451 nm, has a small Δ*E*_ST_ of 0.05 eV, a τ_d_ of 3.4 μs, and a Φ_PL_ of 86% in 12 wt% doped films in DBFPO, while the chirality conferred by the presence of the asymmetric nitrogen atom resulted in a g_PL_ on the order of 10^–4^. The corresponding OLEDs with **(*S*)-NPE-AcDPS** showed an EQE_max_ of 18.5%, although again no obvious CPL signal was detected.

Finally, Zheng *et al*. developed three pairs of spiro-type TADF enantiomers with carbon stereocenters, similar to their previously reported **OSFSO** but with differently substituted acceptors: **(*R*/*S*)-SCN**, **(*R*/*S*)-SPHCN**, and **(*R*/*S*)-SCFPY** (Figure 76).^629^ SCN possesses a cyano group as the acceptor, SPHCN contains a benzonitrile as an elongated acceptor, and SCFPY uses 2-(trifluoromethyl)pyridine as a stronger acceptor. All three materials show green emission at λ_PL_ of 522 nm for **(*R*/*S*)-SCN**, 505 nm for **(*R*/*S*)-SPHCN**, and 526 nm for **(*R*/*S*)-SCFPY**. These compounds all have relatively small Δ*E*_ST_ (in toluene) and high Φ_PL_ (in 25 wt% doped films in 26DCzPPy): 0.01 eV and 89% for **(*R*/*S*)-SCN**, 0.16 eV and 67% for **(*R*/*S*)-SPHCN**, and 0.04 eV and 89% for **(*R*/*S*)-SCFPY**. The impact of the molecular structures on the CPL properties were then studied by comparing their chiroptical properties and device performances. **(*R*/*S*)-SCN** showed a |g_PL_| of 1.4 × 10^–3^ in toluene and the device showed an EQE_max_ of 23.0% with g_EL_ of −1.4/1.8 × 10^–3^. For **(*R*/*S*)-SPHCN** with a longer acceptor, although the EQE_max_ decreased to 15.4% there is a larger |g_PL_| of 3.6 × 10^–3^ and |g_EL_| of −3.6 × 10^–3^. **(*R*/*S*)-SCFPY**, possessing an acceptor of similar size to **(*R*/*S*)-SPHCN**, has a similar |g_PL_| of 3.5×10^–3^ but the device showed a higher EQE_max_ of 23.3% (g_EL_ of −3.7/3.6 × 10^–3^), which represents the highest efficiency spiro-type TADF material-based OLED to date. The authors therefore report that g_PL_ and g_EL_ can be enhanced by extending the length of the acceptor, which in this study caused a better alignment between ***μ*** and ***m*** (smaller *θ*), as confirmed by their calculations.

### 7.3 CP-TADF Emitters with Axial Chirality

The first examples of intrinsic axially chiral TADF emitters, **(*R/S*)-1** and **(*R/S*)-2** (Figure 77), were developed by Wang *et al.* in 2019 ^630^ and contained a stereogenic binaphthol (BINOL) unit. **(*R/S*)-1** and **(*R/S*)-2** show yellow or green emission at λ_PL_ of 568 and 530 nm and have Φ_PL_ and Δ*E*_ST_ of 18.5 and 15.7% and 0.059 and 0.076 eV, all respectively. **(*R*)-1** has similar g_PL_ of 1.6 × 10^–3^ in toluene, 8.2 × 10^–4^ in 15 wt% doped films in TCTA, and 9.2 × 10^–4^ as a neat film. Interestingly, **(*R/S*)-2** did not show CPL, likely due to the rotatable benzophenone structure that limits the chirality transfer process from the binaphthyl to the peripheral D-A TADF chromophore. OLEDs with ***S*-1** exhibited orange emission (λ_EL_ at 580 nm) with an EQE_max_ of 1.8% and g_EL_ of +1.0 × 10^–3^.

**Figure 77:**
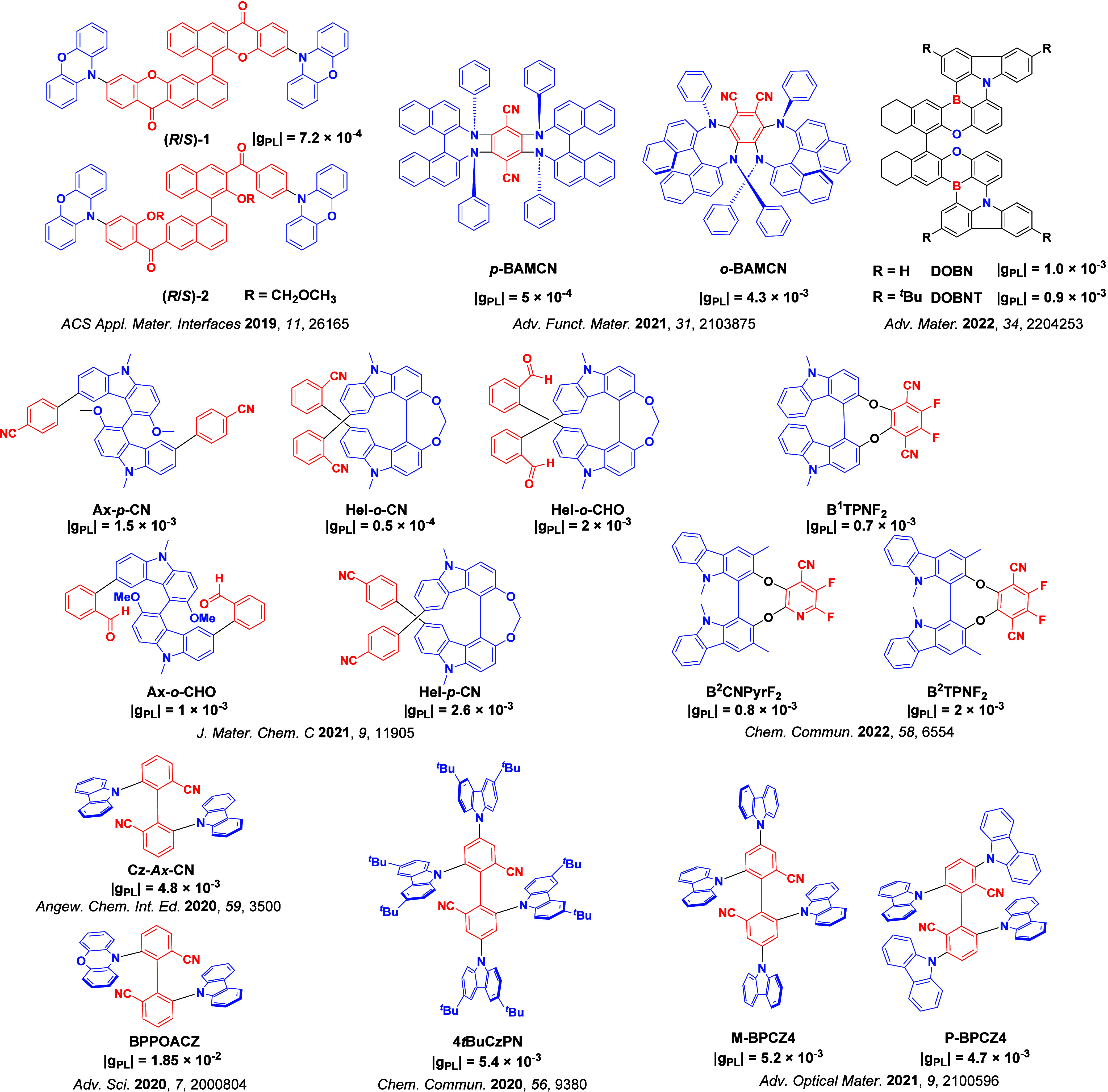
Structures of CP-TADF emitters possessing axial chirality and their respective |g_PL_| (the blue color signifies donor moieties, while the red color signifies acceptor moieties).

In 2021 Yan *et al*. designed two new chiral TADF materials, ***p*-BAMCN** and ***o*-BAMCN** (Figure 77), containing modified chiral BINOL peripheral groups acting as axially chiral donors around either *para* or *ortho* substituted dicyanobenzene as the acceptor.^631^ Both emitters showed narrowband green emission (FWHM of 61 nm for both), with λ_PL_ at 537 and 503 nm and Φ_PL_ of 86 and 77% in either 8 wt% doped films in TCTA or in 26DCzPPy for ***p*-BAMCN** and ***o*-BAMCN**, all respectively. The Δ*E*_ST_ were also similar for the pair of emitters at 0.18 eV for ***p*-BAMCN** and 0.15 eV for ***o*-BAMCN**. **(*S*)-*o*-BAMCN** showed higher but similar g_PL_ in both toluene (5.3 × 10^–3^) and in the doped film (4.3 × 10^–3^) when compared to **(*S*)-*p*-BAMCN** (0.3 and 0.5 × 10^–3^), which was rationalized in terms of the different DFT-predicted angles between ***μ*** and ***m*** in the *para*- and *ortho*-derivatives. The OLED with **(*R*)-*p*-BAMCN** showed a high EQE_max_ of 27.6% although the CPEL of the device was too weak to be obtained. OLEDs with **(*R*)-*o*-BAMCN** showed an EQE_max_ of 20.5%. Semi-transparent devices were also fabricated to reduce the reflection of metallic cathodes and improve CPL performance, with **(*S*)-*o*-BAMCN** showing a g_EL_ of 4.6 × 10^–3^ in line with its g_PL_.

To achieve narrower CPL emission, the same authors combined axial chirality with an MR-TADF design, leading to **DOBN** and **DOBNT** (Figure 77).^632^ **DOBN** and **DOBNT** emit at λ_PL_ of 453 and 459 nm, and both have FWHMs of 21 nm in toluene. Although both emitters exhibited g_PL_ values lower than 0.2 × 10^–4^ in toluene, they showed high Φ_PL_ of 91 and 96% and moderate g_PL_ of 1.0 and 0.9 × 10^–3^ in 5 wt% doped films in 26DCzPPy. The CP-OLEDs with **(*R*)-DOBN** and **(*R*)-DOBNT** displayed narrowband blue emission at λ_EL_ of 459 and 464 nm with CIE coordinates of (0.14, 0.10) and (0.13, 0.12), and showed EQE_max_ of 23.9 and 25.6% with g_EL_ of −0.9 and −1.0 × 10^–3^.

The axially chiral TADF emitter **Cz-*Ax*-CN** (Figure 77), reported by Li *et al.*, contains two coupled D-A 3-(9H-carbazol-9-yl)benzonitrile fragments.^633^ Both enantiomers of **Cz-*Ax*-CN** exhibited dual TADF and AIE, emitting at 460 nm and have a small Δ*E*_ST_ of 0.029 eV, a short τ_d_ of 12.6 ms, and a Φ_PL_ of 68% in 15 wt% doped films in DPEPO. The g_PL_ of **(−)-(*S*)-Cz-*Ax*-CN** in the film reached −4.8 × 10^–3^. The CP-OLED with **(−)-(*S*)-Cz-*Ax*-CN** showed blue electroluminescence at λ_EL_ of 468 nm, with an EQE_max_ of 12.5% and a g_EL_ value of −1.2 × 10^–2^, which is larger than the g_EL_ values of most other reported CP-TADF OLEDs.

In a separate report, the same group modified the nature and number of the donor moieties to further enhance CPL activity. **4tBuCzPN** (Figure 77) emits at λ_PL_ of 476 nm, has a Φ_PL_ of 74%, and g_PL_ values of 5.4 × 10^–3^ in toluene and a small Δ*E*_ST_ of 0.05 eV, a short τ_d_ of 4 ms, and a g_PL_ value of 5.2 × 10^–3^ in 25 wt% doped films in DPEPO.^382^ The OLED fabricated with racemic **4tBuCzPN** showed a significantly improved EQE_max_ of 20.8% compared to the device with **Cz-*Ax*-CN** (12.5%), and emitted at λ_EL_ of 500 nm. The authors did not however report g_EL_ values for the devices since racemization of the enantiomers was discovered during the vacuum evaporation. Tu *et al*. employed the same design, substituting one carbazole for a phenoxazine to construct the emitter **BPPOACZ** (Figure 77).^634^ *rac*-BPPOACZ exhibited two emission bands peaking at 384 and 543 nm in toluene. Both enantiomers showed high Φ_PL_ of 86%, |g_PL_| of 9.7 × 10^–3^, Δ*E*_ST_ of 0.04 eV, and short τ_d_ of 1.1 ms in toluene, while only one emission peak at 527 nm appeared in 20 wt% doped films in 26DCzPPy at 527 nm, and the g_PL_ in this host was higher at 1.85 × 10^–2^. The CP-OLED based on **(*S*)-BPPOACZ** displayed green electroluminescence (λ_EL_ = 537 nm) and showed an EQE_max_ of 17.8% and a low efficiency roll-off with EQE_1000_ of 15.2% and EQE_10000_ of 12.6%. However, the g_EL_ was only 4.5 × 10^–3^ for reasons that remain unclear.

This same group also reported another two similar blue emitters, **M-BPCZ4** and **P-BPCZ4** (Figure 77), which contain additional carbazole moieties but have different donor connectivity.^635^ **M-BPCZ4** and **P-BPCZ4** both emit at 470 nm and show Δ*E*_ST_ of 0.09 and 0.05 eV respectively in toluene. The emission is slightly red-shifted to λ_PL_ at 485 nm, and the Φ_PL_ and τ_d_ are 64 and 76%, and 6.4 and 7.0 ms in 25 wt% doped films in DPEPO, respectively. **(*R*)-M-BPCZ4** and **(*R*)-P-BPCZ4** have high g_PL_ of −5.0 and −4.7 × 10^–3^ in toluene. The blue CP-OLEDs with **(*R*)-P-BPCZ4** showed a higher EQE_max_ of 18.3% (λ_EL_ = 480 nm) and a lower efficiency roll-off with EQE_1000_ of 17.2%, compared with **(*R*)-M-BPCZ4** which showed an EQE_max_ of 16.7% and EQE_1000_ of 15.7%. This team also found that the position of the carbazole units affected the racemization temperatures and corresponding CPL properties of the enantiomer OLEDs greatly. The presence of a crowded set of carbazole donors in **(*R/S*)-P-BPCZ4** results in a centralization of both the ***μ*** and ***m***. Additionally, the steric congestion present in (***R/S*)-P-BPCZ4** prevents undesired racemization during vacuum deposition for device fabrication. Consequently, the device with **(*R/S*)-P-BPCZ4** possesses a higher g_EL_ value (-5.5 × 10^–3^) than that with **(*R/S*)-M-BPCZ4** (-3.8 × 10^–3^).

Sumsalee *et al.* designed five chiral emitters **Ax-*p*-CN**, **Ax-*o*-CHO**, **Hel-*o*-CN**, **Hel-*p*-CN**, and **Hel-*o*-CHO** (Figure 77) that each contain carbonyl-based acceptors with both axially or helically chiral bicarbazole electron donors.^636^ **Ax-*o*-CHO** and **Hel-*o*-CHO** displayed TADF and emit at λ_PL_ of 460 and 439 nm in toluene and have τ_d_ of 1.04 and 0.80 μs in doped DPEPO films respectively; however, their Φ_PL_ in toluene are very low at 3 and 2%, which also remained low at 10 and 5% along with Δ*E*_ST_ of 0.19 and 0.37 eV in the DPEPO films, respectively. Interestingly the CPL properties changed with increasing solvent polarity, with **(+)-Ax-*o*-CHO** having a higher g_PL_ of 1.0 × 10^–3^ in toluene than in chloroform (0.7 × 10^–3^) or DMF (0.5 × 10^–3^), while **(+)-Hel-*o*-CHO** showed a higher g_PL_ of 2.0 × 10^–3^ in chloroform than in DMF (1.6 × 10^–3^) or toluene (∼0). This sensitivity of g_PL_ to solvent polarity was rationalized as due to subtle reorganization of the intramolecular charge-transfer excited and ground states in the different solvents. These results also suggested that higher CPL intensity can be achieved with helical emitters, although these did also display lower TADF efficiency.

Subsequently, Poulard *et al.* reported TADF emitters **B^1^TPNF_2_**, **B^2^TPNF_2_**, and **B^2^CNPyrF_2_**, containing axially chiral bicarbazole donors (Figure 77).^637^ All three showed green emission with λ_PL_ of 529, 530, and 492 nm, and Φ_PL_ of 11, 29, and 23%, respectively. Their g_PL_ values were determined to be 0.7, 2.0, and 0.8 × 10^–3^ in toluene. The higher g_PL_ value for **B^2^TPNF_2_** was attributed to a more favorable orientation between ***μ*** and ***m***, likely a result of its helical structure.

### 7.4 CP-TADF Emitters with Planar Chirality

[2,2]Paracyclophane (PCP) and its derivatives have emerged as useful planar-chiral skeletons in the construction of CP-TADF emitters. Zhang *et al*. first introduced an electron-donating -NMe_2_ group and electron-withdrawing -Bmes_2_ group onto the two separate benzene rings of the PCP in ***g*-BNMe_2_-Cp** and ***m*-BNMe_2_-Cp** (Figure 78).^638^ These emit at λ_PL_ of 531 and 521 nm in toluene, respectively. The HOMOs and LUMOs were efficiently separated in these two compounds, resulting in Δ*E*_ST_ of 0.17 and 0.12 eV in 2-MeTHF glass at 77 K. The powder Φ_PL_ were moderate at 53 and 33% for ***g*-BNMe_2_-Cp** and ***m*-BNMe_2_-Cp**, respectively. The g_PL_ value for ***g*-BNMe_2_-Cp** reached 4.24 × 10^–3^. The g_PL_ for ***m*-BNMe_2_-Cp** was not mentioned and low energy barriers to racemization limited their application, with CP-OLEDs not explored.

**Figure 78:**
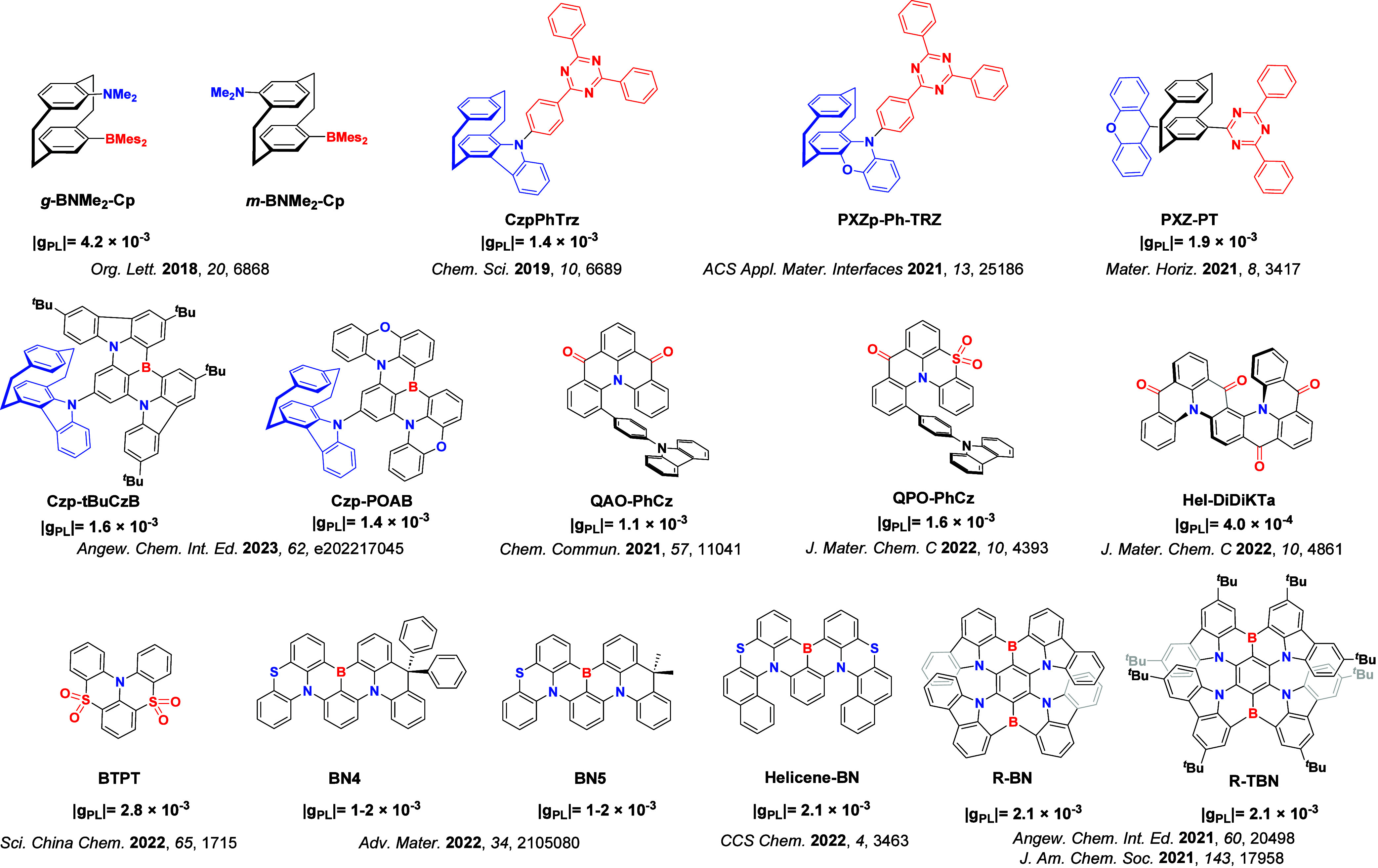
Structures of CP-TADF emitters with planar chirality and their respective |g_PL_| (the blue color signifies donor moieties/atoms/functional groups, while the red color signifies acceptor moieties/atoms/functional groups).

Sharma *et al*. soon after reported the first example of a carbazolophane (Czp) containing TADF emitter, **CzpPhTrz** (Figure 78).^236^ The increased steric bulk of the Czp unit induced an increased torsion angle between the donor and the phenylene bridge compared to the unsubstituted carbazole-containing analogue. This more twisted geometry coupled with a stronger electron-donor in the Czp compared to Cz resulted in a Δ*E*_ST_ of 0.16 eV in 10 wt% doped film in DPEPO. **(*R*)-CzpPhTrz** emits at λ_PL_ of 470 nm and has a g_PL_ value of 1.3 × 10^–3^ in toluene, while ***Rac*-CzpPhTrz** showed a Φ_PL_ of 69% in 10 wt% doped DPEPO film. The OLEDs showed EQE_max_ of 17% at λ_EL_ of 480 nm, but CP-OLEDs were not pursued in this study. Liao *et al*. subsequently reported a structurally related CP-TADF molecule, **PXZp-Ph-TRZ** (Figure 78), using a phenoxazine-based analogue to Czp.^639^ The yellow emitter (λ_PL_ = 527 nm) has a much smaller Δ*E*_ST_ of 0.03 eV compared to **CzpPhTrz** owing to the stronger donor, and has a Φ_PL_ of 60% in 10 wt% doped films in CBP. The solution-processed CP-OLEDs displayed a g_EL_ of 4.6 × 10^–3^ and showed an EQE_max_ of 7.8%.

Zhang *et al*. reported a pair of D-(chiral *π*)-A TADF emitters, **(*R*/*S*)-PXZ-PT** (Figure 78), with a PCP skeleton attached to the central phenylene linker (but not to either the donor or acceptor).^640^ This design strategy not only suppressed the racemization between the two enantiomers, making it possible to fabricate CP-OLEDs by vacuum-deposition, but also reduced non-radiative transitions that led to higher Φ_PL_. **(*R*/*S*)-PXZ-PT** emits at λ_PL_ of 565 nm and has a Δ*E*_ST_ of 0.19 eV and a high Φ_PL_ of 78% in 10 wt% doped films in CBP, while the g_PL_ is ±1.9 × 10^–3^. The vacuum-deposited CP-OLEDs exhibited yellow emission [λ_EL_ of 557 nm, CIE coordinates of (0.44, 0.55)] and showed a higher EQE_max_ of 20.1% than those of the earlier reported devices with **CzpPhTrz** and **PXZp-Ph-TRZ**; the g_EL_ was 1.5 × 10^–3^.

Liao *et al*. reported two pairs of Czp-substituted MR-TADF materials. **Czp-tBuCzB** and **Czp-POAB** (Figure 78).^641^ **(*R*/*S*)-Czp-tBuCzB** and **(*R*/*S*)-Czp-POAB** emit at 478 and 497 nm with narrow FWHMs of 23 and 36 nm, have Δ*E*_ST_ of 0.09 and 0.13 eV and g_PL_ of 0.54/-0.51 × 10^–3^ and 0.48/-0.46 ×10^–3^ in toluene. Both emitters have near unity Φ_PL_ of 98 and 96%, and τ_d_ of 41.8 and 62.4 ms in doped films (5 wt% and 8 wt% doped films in 2,6DCzPPy), all respectively. The sky-blue CP-OLEDs with **(*R*)-Czp-tBuCzB** (λ_EL_ of 479 nm) showed a high EQE_max_ of 32.1%, EQE_100_ of 29.2%, EQE_1000_ of 30.9%, and the narrowest FWHM of 24 nm among reported CP-OLEDs alongside g_EL_ of +1.54 × 10^–3^. Devices with **(*R*)-Czp-POAB** displayed near-pure green CP electroluminescence [CIE coordinates of (0.23, 0.65)] with EQE_max_ of 28.7%, EQE_100_ of 28.1%, EQE_1000_ of 20.4%, and g_EL_ of +1.30 × 10^–3^. These studies demonstrate that the PCP unit can be used towards the construction of CPL-active D-A TADF and MR-TADF emitters, both showing modest g_PL_.

Helicenes are a class of fused polycyclic aromatic frameworks that possess a helical chirality. In helicenes larger than five rings the overlap between the opposite ends of the fused system renders the enantiomers kinetically stable towards racemization. Helicenes have attracted significant research interest due in part to their promising applications in CPL and CP-OLEDs. Yang *et al*. reported a blue CP-TADF emitter, **QAO-PhCz**, possessing a rigid hetero-helicene structure (Figure 78).^642^ The synergistic effects of the sterically hindered donor linkage and the rigid emissive core generated narrowband emission at λ_PL_ of 460 nm with FWHM of 29 nm. **(*P*)-QAO-PhCz** has a Δ*E*_ST_ of 0.11 eV, τ_d_ of 40.36 ms, and a moderate Φ_PL_ of 46.6% in 5 wt% doped films in mCBP. The corresponding CP-OLED showed a narrow FWHM of 36 nm (λ_EL_ of 467 nm) and an EQE_max_ of 14%. The enantiomers of **QAO-PhCz** displayed similar |g_PL_| and |g_EL_| of 1.1 and 1.5 × 10^–3^, respectively. Following this concept, the same group reported another pair of chiral hetero-helicene molecules **(*P*/*M*)-QPO-PhCz** (Figure 78), this time with a carbonyl-/sulfone-bridged triarylamine structure.^643^ Compared to **QAO-PhCz**, **QPO-PhCz** showed similar photophysical properties, emitting at 446 nm, having a Δ*E*_ST_ of 0.23 eV, and a |g_PL_| of 1.2 × 10^–3^ in toluene. The compound has a long τ_d_ of 536 ms and a Φ_PL_ of 51% in 18 wt% doped films in DPEPO. The CP-OLEDs with **(*M*)-QPO-PhCz** showed sky-blue emission (λ_EL_ of 488 nm) with EQE_max_ of 10.6%, and g_EL_ of +1.6 × 10^–3^.

Extending the concept of helically chiral emitters further, Marques dos Santos *et al*. reported extended helical structure **Hel-DiDiKTa** (Figure 78), which is an S-shaped double [4]helicene based on a pair of fused **QAO** (or equivalently **DiKTa**) cores.^644^ The CPL-active MR-TADF molecule **(*P*)-Hel-DiDiKTa** emits in the sky-blue emission (λ_PL_ at 473 nm) and has a small Δ*E*_ST_ of 0.15 eV, and τ_d_ of 5.4 ms in 1 wt% doped films in mCP. However, the g_PL_ is only 4.0 × 10^–4^ and the Φ_PL_ is low at 6.2% in 1 wt% doped PMMA film, which precluded devices from being investigated. Compared to previously reported **DiKTa**-based emitters, the molecular distortions present in this helical compound are thought to result in severe emission quenching.

Ning *et al*. reported a strikingly simple polycyclic aromatic heterocycle **BTPT** (Figure 78) that contains sulfone groups at the two *ortho*-positions of a triphenylamine core and is helically chiral.^645^ **(*P*)-BTPT** emits in the ultraviolet (λ_PL_ = 368 nm) with a narrow FWHM of 33 nm in toluene. In 1 wt% doped films in PMMA, **(+)-(*P*)-BTPT** has a Δ*E*_ST_ of 0.14 eV, a τ_d_ of 109 ms, yet a Φ_PL_ of only 9%. The enantiomeric crystals of **BTPT** not only displayed CPL with a g_PL_ on the order of 10^–3^, but also showed room temperature phosphorescence.

Wu *et al.* developed another type of MR-TADF emitter with helical chirality, exemplified in **BN4** and **BN5** (Figure 78).^646^ These two compounds contain an asymmetrical peripheral lock to the well-known MR-TADF molecule **DABNA-1**, enhancing the helical nature of the B/N doped nanographene. Sulfur was chosen as the bridging atom of the rigid locked ring, and both compounds emit at λ_PL_ of 500 and 497 nm and have the same Δ*E*_ST_ of 0.14 eV in toluene, alongside high Φ_PL_ of 96 and 92% for **BN4** and **BN5** in 3 or 1 wt% doped films in mCPCN. **BN4** and **BN5** thus have similar *k*_RISC_ of 3.7 and 3.3 × 10^4^ s^–1^, all respectively. **(*R*)/(*S*)-BN4** and **(*R*)/(*S*)**-**BN5** in doped mCPCN films displayed g_PL_ of +1.1/-1.0 and +1.3 /-1.0 × 10^–3^, respectively. The CP-OLEDs with **BN4** and **BN5** achieved narrowband emission at λ_EL_ of 510 and 506 nm (FWHM of 49 and 48 nm) and showed EQE_max_ of 20.6 and 26.5% with g_EL_ of +3.7/-3.1 and +1.9/-1.6 × 10^–3^, respectively.

Yang *et al*. developed a pair of helicene-based enantiomers, **(*P*)-helicene-BN** and **(*M*)-helicene-BN** (Figure 78), which merged helical chirality and the B/N/S doped polycyclic aromatic framework to concurrently exhibit CPL and MR-TADF behavior.^647^ **Helicene-BN** emits at λ_PL_ of 525 nm, has a Δ*E*_ST_ of 0.15 eV and a Φ_PL_ of 100% in 1 wt% doped films in DMIC-TRZ. In toluene, the g_PL_ values are +2.0 × 10^–3^ for **(*P*)-helicene-BN** and −2.1 × 10^–3^ for **(*M*)-helicene-BN**, while in the 1 wt% doped films in DMIC-TRZ, the g_lum_ values are +1.3 × 10^–3^ for **(*P*)-helicene-BN** and −2.0 × 10^–3^ for **(*M*)-helicene-BN**. CP-OLEDs with **(*P*)-helicene-BN** and **(*M*)-helicene-BN** showed EQE_max_ of 31.5% at CIE coordinates of (0.26, 0.66). The devices also exhibited g_EL_ of +1.2 × 2.2 × 10^–3^, respectively.

Zhang *et al.* developed two similar helical deep-red MR-TADF emitters **R-BN** and **R-TBN** (Figure 78) that emit at λ_PL_ of 662 and 692 nm and have Φ_PL_ of 100%, Δ*E*_ST_ of 0.18 and 0.16 eV, and τ_d_ of 16.6 and 46.4 ms in toluene, all respectively.^177^ In 3 wt% doped films in CBP, they emits at 672 and 698 nm, and have τ_d_ of 0.31 and 0.71 ms, respectively. The OLEDs with **R-BN** and **R-TBN** showed EQE_max_ of 28.1 and 27.6%. Li, Wang *et al.* then explored the chiroptical properties of these two emitters, which have g_PL_ of 2 × 10^–3^ in dichloromethane.^648^ These examples illustrate state-of-art strategies to fabricate CP-TADF emitters with narrow emission based on a helical skeleton, but again illustrate the difficulties in discovering or designing molecules with g_EL_ or g_PL_ greater than 10^–2^.

### 7.5 CP-TADF Emitters Featuring Chiral Perturbation

The chiral perturbation strategy to construct CPL-active TADF compounds involves the introduction of a chiral peripheral group to an otherwise achiral TADF structure. The chiral unit does not directly participate in the emissive process. This strategy is now widely used because of the ease of the synthesis and enantiomer separation processes. The reported emitters using this strategy can maintain efficient TADF inherited from previously validated designs, while also exhibiting promising CPL behavior bestowed by the perturbing group.

Feuillastre *et al*. reported the first chiral perturbation TADF materials, **(*R*)-1** and **(*S*)-1** (Figure 79), incorporating a BINOL unit to confer axial chirality to the molecule.^649^ **(*R*)-1** emits at 486 nm in cyclohexane with a g_PL_ of 1.3 × 10^–3^, while the compound has a Φ_PL_ of 53% and τ_d_ of 2.9 ms in toluene; however, the OLEDs based on **(*S*)-1** displayed only a modest EQE_max_ of 9.1% with λ_EL_ at 535 nm. Song *et al*. used a similar design strategy to combine TADF, AIE, and CPL properties in the emitters **BN-CF**, **BN-CCB**, **BN-DCB**, and **BN-AF** (Figure 79).^650^ Compound **(*S*)-BN-CF** showed the highest g_PL_ of 1.2 × 10^–3^ with λ_PL_ of 495 nm and Φ_PL_ of 32% in toluene. It also showed the same Φ_PL_ of 32% and τ_d_ of 24.33 ms in 10 wt% doped film in mCP. Surprisingly the g_PL_ values of the neat films were amplified significantly, especially for **(*S*)-BN-CF** which achieved a very high g_PL_ of 4.1 × 10^–2^. The CP-OLEDs using 10 wt% doped films in mCP as emitting layers showed an EQE_max_ of 9.3% and g_EL_ of 2.6 × 10^–2^. The non-doped **(*S*)-BN-CF** OLED exhibited a further amplified g_EL_ of 6 × 10^–2^. The higher g_PL_ values of **(*S*)-BN-CF** than previous reported **(*R/S*)-1** were attributed to the AIE properties. Huang *et al*. later also reported two BINOL-based chiral emitters, **CPDCz** and **CPDCB** (Figure 79).^651^ **(*S*)-CPDCz** and **(*S*)-CPDCB** emit at λ_PL_ of 511 and 533 nm, with Δ*E*_ST_ of 0.08 and 0.04 eV in respective 10 wt% doped films in mCP. They also have Φ_PL_ of 20 and 55%, τ_d_ of 18 and 10 ms, and g_PL_ values of −3.3 and −4.0 × 10^–4^, respectively. The solution-processed CP-OLEDs with **(*S*)-CPDCB** showed an EQE_max_ of 10.6% and g_EL_ of −3.9 × 10^–3^ compared to the device with **(*S*)-CPDCz**, which showed an EQE_max_ of 10.1% and g_EL_ of −3.7 × 10^–3^.

**Figure 79:**
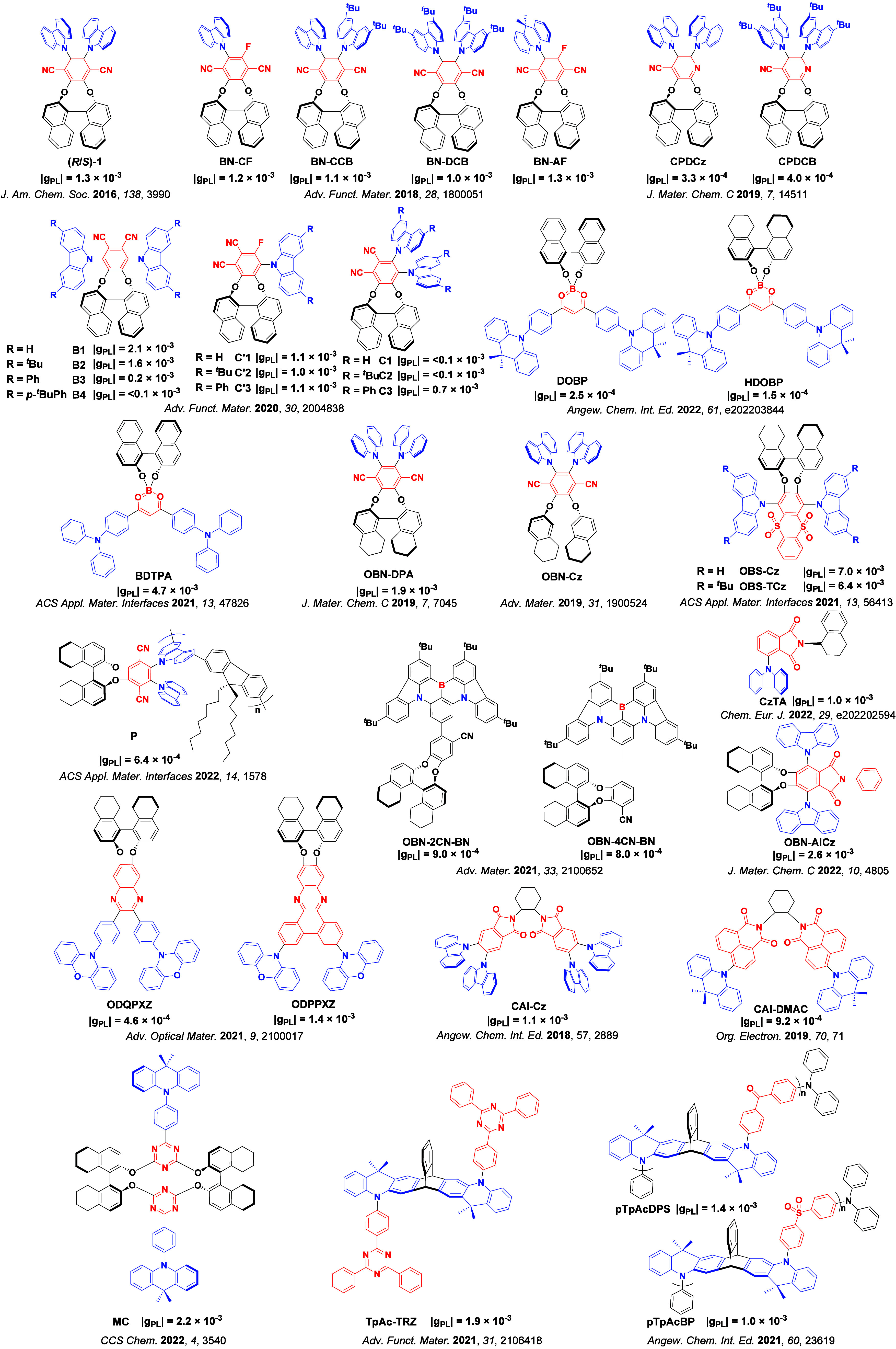
Structures of CP-TADF emitters featuring chiral perturbation and their respective |g_PL_| (the blue color signifies donor moieties, while the red color signifies acceptor moieties).

Pieters *et al*. reported three families of BINOL-based chiral TADF emitters (**B**, **C**, and **C′**, Figure 79) with different numbers of donors at different positions and with different distances between the chromophore and the chiral perturbing unit.^652^ For the **B** series, the molecule emits at λ_PL_ of 469–516 nm, having Φ_PL_ of 7–30%, τ_d_ of 10–45–ms, and Δ*E*_ST_ of 0.1–0.31 eV. For the **C** series, they emit at λ_PL_ of 493–519 nm, having Φ_PL_ of 25–47%, τ_d_ of 18–40 ms, and Δ*E*_ST_ of 0.1–0.22 eV. For the **C′** series, they emit at λ_PL_ of 481–510 nm, having Φ_PL_ of 29–42%, τ_d_ of 6–19 ms, and Δ*E*_ST_ of 0.11–0.28 eV. The Φ_PL_ are obtained in doped PMMA films and Δ*E*_ST_ are estimated from spectra in 2-MeTHF, with other data are obtained in toluene. The **B** series has the smallest distance between two carbazole donors and the stereogenic unit, and showed improved CPL performance compared to the **C** and **C′** families as predicted by the higher ***m*** and smaller *θ* from DFT calculations. Compound **B1** exhibited the highest g_PL_ of 2.1 × 10^–3^ (in toluene) of all the compounds in the study. As **C’3** shows the best compromise between optical and chiroptical properties, it was used as the emitter in top emitting CP-OLED. The device showed green emission centered at λ_EL_ of 510 nm, a low EQE of only 0.8%, and g_EL_ of 1.0 × 10^–3^.

Zhou *et al.* reported two pairs of enantiomers [**(*R*/*S*)-DOBP** and **(*R*/*S*)-HDOBP**, Figure 79] that contain tetracoordinate boron atoms. These two compounds displayed concomitantly AIE, CPL, mechanochromism, and piezochromism.^653^ **(*R*/*S*)-DOBP** and **(*R*/*S*)-HDOBP** emit at 536 and 534 nm and have large Δ*E*_ST_ values of 0.28 and 0.23 eV in dilute toluene. In neat films the Δ*E*_ST_ values decrease to 0.14 and 0.08 eV for **(*R*)-DOBP** and **(*R*)-HDOBP**, although the Φ_PL_ are only 1 and 2%, all respectively. The g_PL_ values are ±2.5 and ±1.5 × 10^–4^ for **(*R*/*S*)-DOBP** and **(*R*/*S*)-HDOBP** in 1,4-dioxane, respectively. The non-doped solution-processed OLEDs with **(*R*)-DOBP** showed NIR emission (λ_PL_ = 716 nm) and an EQE_max_ of 1.9%.

Xue *et al.* reported the emitter **BDTPA** that has a similar structure as the previous example **(*R*/*S*)-DOBP**, but with the DMAC donor replaced by a triphenylamine.^654^ **(*R*)-BDTPA** emits at λ_PL_ of 560 nm and has a Δ*E*_ST_ of 0.14 eV, a τ_d_ of 53.5 ms, and a significantly improved g_PL_ of −1.7 × 10^–3^ in toluene. It is at present not clear why the g_PL_ of **(*R*)-BDTPA** is so much higher than those of **(*R*)-DOBP** and **(*R*)-HDOBP**. **(*R*)-BDTPA** emits at 600 nm and has a Φ_PL_ of 15.8% in 10 wt% doped mCP film. The solution-processed CP-OLED with **(*R*)-BDTPA** showed an EQE_max_ of 2.0% and a g_EL_ value of −1.6 × 10^–3^ (λ_EL_ of 598 nm).

Wu *et al*. reported an analogue of **(*R*)-1** that instead contained an octa­hy­dro­bi­naph­thol unit, **OBNCz** (Figure 79).^655^ **(*R*)-OBNCz** has a g_PL_ value of −1.55 × 10^–3^, emits with λ_PL_ of 504 nm, and has a Φ_PL_ of 92% and a small Δ*E*_ST_ of 0.037 eV in 10 wt% doped films in 26DCzPPy. CP-OLEDs with **(*R*)-OBNCz** showed an EQE_max_ of 32.6%, with very low efficiency roll-off (EQE_1000_ of 31.7% and EQE_5000_ of 30.6%) and g_EL_ of 1.94 × 10^–3^ (λ_EL_ of 526 nm), making this example the best performing CP-OLED to date in terms of efficiency. Wu *et al*. also reported a pair of similar enantiomers, **OBN-DPA**, which replaced the carbazole in **OBNCz** with a diphenylamine moiety (Figure 79).^656^ **(*R*)-OBN-DPA** exhibited green emission peaking at 538 nm, a small Δ*E*_ST_ of 0.09 eV, and a g_PL_ of 1.88 × 10^–3^ in toluene. The 10 wt% doped film of **(*R*)-OBN-DPA** in 26DCzPPy has a Φ_PL_ of 84.7% and a short τ_d_ of 13.5 ms along with a |g_PL_| value of 2.9 × 10^–3^. The doped and non-doped CP-OLEDs showed lower EQE_max_ of 12.3 and 6.6% compared to the device with **OBNCz**, though with somewhat higher g_EL_ values of 2.9 and 2.3 × 10^–3^, respectively.

Liu *et al*. reported the compound **(*R*)/(*S*)-OBS-TCz** and the analogue **(*R*)/(*S*)-OBS-Cz**, both containing a 5,5,10,10-tetraoxide acceptor and the **(*R*)/(*S*)-OBS** group as the chiral perturbing unit (Figure 79).^657^ The enantiomers **(*R*)/(*S*)-OBS-Cz** and **(*R*)/(*S*)-OBS-TCz** emit at λ_PL_ of 504 and 520 nm in toluene, have small Δ*E*_ST_ of 0.04 and 0.05 eV, short τ_d_ of 3.2 and 2.7 ms, Φ_PL_ of 73 and 87%, and g_PL_ of 8.7 and 6.4×10^–4^ in 15 wt% doped films in mCP, all respectively. CP-OLEDs with **(*R*)/(*S*)-OBS-TCz** showed higher EQE_max_ of 20.3% and EQE_1000_ of 20.1%, but smaller g_EL_ values of +0.80/–1.00 × 10^–3^ than the devices with **(*R*)/(*S*)-OBS-Cz** (EQE_max_ of 15%, EQE_1000_ of 14.5%; g_EL_ +5.00/–4.00 × 10^–4^).

Li *et al*. synthesized the first highly efficient green CP-MR-TADF molecules.^658^ They introduced chiral (*R*)/(*S*)-octa­hy­dro-bi­naph­thol ((*R*)/(*S*)-OBN) units onto the previously reported blue-green MR-TADF emitter (**DtBuCzB**) to induce CPL. The enantiomers **(*R*)/(*S*)-OBN-2CN-BN** and **(*R*)/(*S*)-OBN-4CN-BN** (Figure 79) exhibit narrowband emission at 493 and 500 nm, with FWHM of 22 and 24 nm, and small Δ*E*_ST_ of 0.12 and 0.13 eV in toluene, respectively. Both compounds have high Φ_PL_ of 95 and 90%, and τ_d_ of 95.3 and 97.4 ms in 3 wt% doped films in PhCzBCz. Unfortunately, the g_PL_ values are all rather low; +9.0 × 10^–4^ for **(*R*)-OBN-2CN-BN**, −9.1 × 10^–4^ for **(*S*)-OBN-2CN-BN**, +8.0 × 10^–4^ for **(*R*)**-**OBN-4CN-BN**, and −10.4 × 10^–4^ for **(*S*)**-**OBN-4CN-BN**. CP-OLEDs with **(*R*)/(*S*)-OBN-2CN-BN** and **(*R*)/(*S*)-OBN-4CN-BN** emitted at λ_EL_ of 496 and 508 nm and had small FWHMs of 30 and 33 nm, leading to CIE coordinates of (0.11, 0.52) and (0.14, 0.64), and EQE_max_ of 29.4 and 24.5% with g_EL_ values of +1.43/-1.27 × 10^–3^ and +4.60/-4.76 × 10^–4^, all respectively. This report was the first example of a highly efficient narrowband-emitting CP-MR-TADF OLED. Despite these advances, the low g_PL_ factors still indicate that there is significant space to design new molecules that show higher dissymmetry factors.

Teng *et al*. reported conjugated polymers **(*R*)-P** and **(*S*)-P** (Figure 79) using the same strategy of chiral perturbation exemplified with the axial chiral binaphthyl units,^659^ wherein the chirality is transferred from the stereogenic moiety to the D-A TADF monomers. As a result, the ***R*** and ***S*** polymers exhibited excellent TADF properties with small Δ*E*_ST_ of 0.045 and 0.061 eV measured in 2-MeTHF glass, emit at λ_PL_ of 549 and 547 nm, and have similar Φ_PL_ of 72 and 76% and short τ_d_ of 1.6 and 2.3 ms in 10 wt% doped films in mCP, all respectively. The *k*_RISC_ are 6.28 and 6.31×10^–5^ s^–1^ based on their neat films. The polymers have g_PL_ values of up to 1.9×10^–3^ obtained from the annealed doped films. The corresponding solution-processed CP-OLEDs with **(*R*)-P** and **(*S*)-P** emitted at λ_EL_ of 546 and 544 nm and showed EQE_max_ of 14.9 and 15.8% with g_EL_ of −1.5 and +1.6×10^–3^, respectively. This work expanded the strategy of chiral perturbation with binaphthyl units to TADF polymers.

Xie *et al*. reported contrasting pairs of enantiomers, flexible **(*R*/*S*)-ODQPXZ** and rigid **(*R*/*S*)-ODPPXZ**, each containing (*R*/*S*)-octahydro-binaphthol as the stereogenic unit (Figure 79).^660^ **(*R*)-ODQPXZ** and **(*R*)-ODPPXZ** emit at λ_PL_ of 589 and 630 nm and have Δ*E*_ST_ values of 0.16 and 0.07 eV in toluene, respectively. They also have high Φ_PL_ of 92 and 89%, and short τ_d_ of 3.6 and 3.7 ms in 15 wt% doped films in CBP. **(*R/S*)-ODPPXZ** showed higher g_PL_ values of 1.4/1.9×10^–3^ compared to **(*R/S*)-ODQPXZ** (g_PL_ = −4.6/4.0×10^–4^). The yellow-emitting CP-OLED (λ_EL_ of 548 nm) with **(*R*)-ODQPXZ** showed higher EQE_max_ of 28.3%, EQE_100_ of 20.6%, and smaller g_EL_ of 6.0×10^–4^, compared to **(*R*)-ODPPXZ** which showed EQE_max_ of 20.3% and EQE_100_ of 17.2%, with λ_EL_ of 600 nm and g_EL_ of 2.4×10^–3^. The authors ascribed the more intense CPL in **(*R*)-ODPPXZ** to its more rigid structure wherein the phenyl groups are fused into a larger phenanthrene unit in the acceptor.

Zhao *et al*. applied a similar strategy in the design of CP-TADF macrocyclic enantiomers **(+)-(*R***, ***R*)-MC** and **(−)-(*S***, ***S*)-MC**, which combine two TADF skeletons with similar octahydro-binaphthol moieties (Figure 79).^661^ Macrocycle **(+)-(*R***, ***R*)-MC** emits at λ_PL_ of 505 nm, has a very small Δ*E*_ST_ of 0.069 eV, Φ_PL_ of 78%, and short τ_d_ of 1.76 ms as neat film. The g_PL_ value was measured to be 2.2 × 10^–3^ in toluene. The solution-processed CP-OLED with **(+)-(*R***, ***R*)-MC** emitted at λ_EL_ of 522 nm and showed EQE_max_ of 17.1%, EQE_1000_ of 16.5%, and a g_EL_ value of 1.5 × 10^–3^. This work documents the first example of a CP-TADF macrocycle. The same group also reported a pair of aromatic-imide-based TADF enantiomers, **(*R*/*S*)-OBN-AICz**, which contain (*R*/*S*)-octa­hy­dro­bi­naph­thol attached to a D-A skeleton (Figure 79).^662^ **(*R*)-OBN-AICz** emits at λ_PL_ of 509 nm, has a Φ_PL_ of 81%, and τ_d_ of 4.0 ms in 13 wt% doped film in mCBP. It also has Δ*E*_ST_ of 0.08 eV as neat film. Clear mirror-image CPL with |g_PL_| values of up to 2.6 × 10^–3^ were reported in toluene. The CP-TADF OLEDs with **(*R*)-OBN-AICz** emitted at 514 nm, showed EQE_max_ of 19%, and had g_EL_ of 4.7 × 10^–4^.

Instead of binaphthyl derivatives as the stereogenic unit, a separate strategy involved the use of chiral *trans*-1,2-di­amino­cy­clo­hex­ane to link two imide-based D-A TADF emitters.^663^ **(+)-(*S,S*)-CAI-Cz** (Figure 79) has a |g_lum_| value of 1.1 × 10^–3^ and emits at λ_PL_ of 528 nm with Φ_PL_ of 98%, and has a small Δ*E*_ST_ value of 0.06 eV, yet a rather long τ_d_ of 130 ms in 15 wt% doped films in mCBP. The CP-OLEDs showed an EQE_max_ of 19.8% at λ_EL_ of 520 nm and have g_EL_ values of −1.7 and 2.3 × 10^–3^ for **(+)-(*S,S*)-CAI-Cz** and **(−)-(*R,R*)-CAI-Cz**, respectively. Using the same design but replacing the carbazole donor with DMAC, the same group reported another pair of enantiomers, **CAI-DMAC** (Figure 79).^664^ **(−)-(*R***, ***R*)-CAI-DMAC** emits at 583 nm in toluene and has a Δ*E*_ST_ value of 0.07 eV, a τ_d_ of 37.4 ms, a low Φ_PL_ of 39.9%, and g_PL_ value of 9.2 × 10^–4^ in 6 wt% doped film in CBP. The OLEDs emitted at λ_EL_ of 592 nm and showed EQE_max_ of 12.4%, EQE_100_ of 9.7%, and EQE_1000_ of 4.1%; no g_EL_ was reported for these devices. 1,2,3,4-Tetra­hy­dro-1-naph­thyl­amine is another stereogenic unit that has been used in CP-TADF emitter design, exemplified in **(*R/S*)-CzTA** (Figure 79).^665^ In the crystalline state **(*R*)-CzTA** and **(*S*)-CzTA** emit at λ_PL_ at 465 nm and have Φ_PL_ of 48.7 and 45.3% and delayed lifetimes of 3.37 and 3.40 ms, respectively. The compounds both have Δ*E*_ST_ of 0.13 eV, and the enantiomers have g_PL_ of −1.03 × 10^–3^ for **(*S*)-CzTA** and +0.84 × 10^–3^ for **(*R*)-CzTA** in toluene. No CP-OLEDs were prepared.

Wang *et al*. developed a series CP-TADF emitters containing a chiral triptycene scaffold, exemplified by **(*S***, ***S*)-/(*R***, ***R*)-TpAc-TRZ** (Figure 79).^666^ The enantiomers emit at λ_PL_ of 541 nm and have a small Δ*E*_ST_ of 0.03 eV as neat films. The chiral triptycene scaffold mitigates intermolecular π–π stacking, which led to a Φ_PL_ of 85% and short τ_d_ of 1.1 ms of the neat film. Obvious mirror-image CPL signals were also observed with g_PL_ values of +1.9 and −1.8 × 10^–3^ for **(*S***, ***S*)-(+)-TpAc-TRZ** and **(*R***, ***R*)-(−)-TpAc-TRZ** as neat films, respectively. The solution-processed non-doped CP-OLEDs with **(*S***, ***S*)-(+)-TpAc-TRZ** showed EQE_max_ of 25.5%, EQE_100_ of 16.8%, and EQE_1000_ of 1.6% with g_EL_ of +1.5 × 10^–3^. Using a similar triptycene scaffold the same group reported two pairs of chiral non-conjugated TADF polymers, **(*R***, ***R*)-/(*S***, ***S*)-pTpAcDPS** and **(*R***, ***R*)-/(*S***, ***S*)-pTpAcBP** (Figure 79). The chiral triptycene donor subunit was introduced into the backbone of the polymers, and the well separated FMOs of the monomers produced a material that emits at λ_PL_ of 532 nm with a small Δ*E*_ST_ of 0.01 eV, a high Φ_PL_ of 92%, and a g_PL_ value of −1.0 × 10^–3^ in 10 wt% doped films in mCP.^667^ Solution-processed CP-OLED device with (***R***, ***R*)-pTpAcBP** showed an EQE_max_ of 22.1% and g_EL_ of −1.0 × 10^–3^. This is the first report of CP-OLEDs based on a ‘main-chain’ chiral TADF polymer.

### 7.6 Other Strategies for Designing Chiral TADF Systems

TADF exciplexes are formed from a blend of hole and electron transporting materials, where the HOMO and LUMO are located on the two different molecules (See Section 8). The completely separated FMOs produce a small ***μ*** while maintaining the same magnitude of ***m***, which can be exploited for achieving higher g_PL_ in chiral exciplexes. Favereau *et al*. reported exciplex emitters involving chiral bicarbazole donor **1** and achiral acceptor 5-fluoroisophthalonitrile **A** (Figure 80).^668^ The **1**:**A** (1:2 ratio) blend emits at λ_PL_ of ≈520 nm with a Δ*E*_ST_ of 0.16 eV and a Φ_PL_ of 19%. Importantly, the g_PL_ of 7 × 10^–3^ is ten times higher than the g_PL_ of the chiral donor **1** alone (7 × 10^–4^).

**Figure 80:**
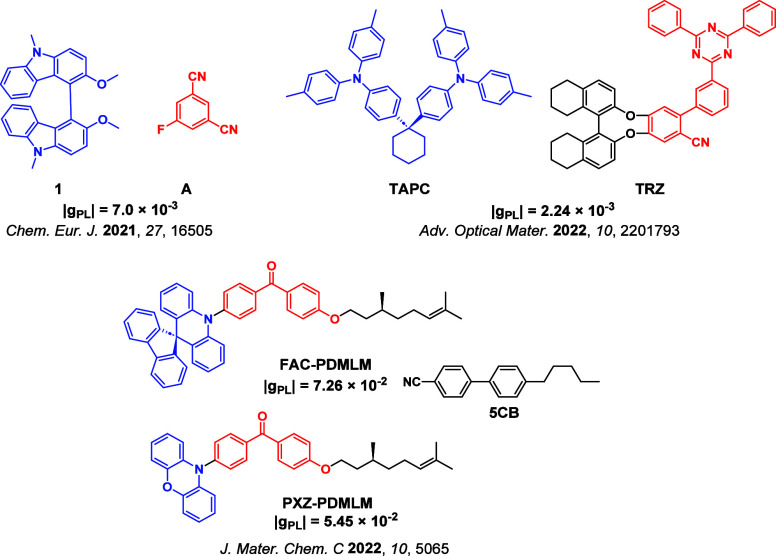
Structures of chiral TADF exciplexes and LC emitters and their respective |g_PL_| (the blue color signifies donor moieties, while the red color signifies acceptor moieties).

Gu *et al*. designed a pair of chiral acceptors **(*R/S*)-TRZ**, and used the hole transporting material 4,4′-cyclo­hex­yli­dene­bis­[N,N-bis­(4-meth­yl­phen­yl)]­aniline (**TAPC**) as the donor to form another CPL-active TADF exciplex (Figure 80).^669^ The **(*R/S*)-TRZ:​TAPC** blended film at the ratio of 1:1 emits at λ_PL_ of 520 nm, and has a very small Δ*E*_ST_ of 0.012 eV with Φ_PL_ of 39.5%. The |g_lum_| of **(*R*)-TRZ:​TAPC** increased from 2.07 to 2.73 × 10^–3^ when the ratio of the donor and chiral acceptor was changed from 2:1 to 1:2. The CP-OLEDs with **(*R*)-TRZ:​TAPC** (1:1) film showed an EQE_max_ of 12.7%, and **(*S*)-TRZ:​TAPC** (1:1) film showed an EQE_max_ of 9.3%. The **(*S*)-TRZ:​TAPC** based device showed the higher g_EL_ of −9.89 × 10^–3^ compared to **(*R*)-TRZ:​TAPC** (g_EL_ = 7.25 × 10^–3^).

Doping chiral emitters and introducing chiral groups into nematic liquid crystals have also been identified as good strategies to realize and amplify CPL properties. Yang *et al*. designed two green and yellow chiral TADF emitters, **FAC-PDMLM** and **PXZ-PDMLM** (Figure 80), which have Φ_PL_ of 18 and 13% and common Δ*E*_ST_ of 0.02 eV in toluene.^670^ Both emitters were virtually CPL-silent between 300 and 500 nm though, as the alkyl chains were unable to transfer chiroptical properties to the TADF moieties. However, when doping **FAC-PDMLM** and **PXZ-PDMLM** into the achiral liquid crystal **5CB**, the co-assembly led to the formation of a chiral nematic liquid crystal phase with very high g_PL_ values of 7.26 × 10^–2^ and 5.45 × 10^–2^, respectively.

### 7.7 Outlook

In this section we have systematically summarized the recent evolutions in the design of chiral TADF emitters, with CPL typically induced via intrinsically chiral emitter skeletons or via chiral perturbation. Significant progress has been made since the first report of chiral TADF emitters showing CPL, and OLEDs employing chiral emitters have seen improvements in g_EL_ values from 10^–4^ to nearly 10^–2^. However, further improvements of these dissymmetry values are required for CP TADF OLEDs to be useful in chiroptical devices. The spread of g_EL_ and EQE_max_ (for devices with EQE_max_ > 10%) of reported CP TADF OLEDs is plotted in Figure 81, with the current trend suggesting that it is extremely challenging to achieve both high EQE_max_ and significant g_EL_ values simultaneously. Of the myriad structures presented herein, we highlight in particular **(*S*)-Cz-Ax-CN**, which showed a g_PL_ of −4.8 × 10^–3^ in 15 wt% doped films in DPEPO, and with CP-OLEDs showing an EQE_max_ of 12.5% and g_EL_ value of −1.2 × 10^–2^; the largest amongst CP TADF OLEDs reported to the end of 2022. Neat films of **(*S*)-BN-CF**, **(*S*)-BN-CCB**, **(*S*)-BN-DCB** and **(*S*)-BN-AF** also have high g_PL_ of 4.1, 3.8, 3, and 2 × 10^–2^, respectively. CP TADF OLEDs with these emitters also exhibited very high g_EL_ values, yet gave low EQE_max_ (6.0 × 10^–2^ and 3.5%; 5.4 × 10^–2^ and 2.3%; 6.7 × 10^–2^ and 2.9%; and 8.4 × 10^–2^ and 0.6%, respectively). Indeed, we note that most of the best performing CP-TADF emitters still show small g_PL_ values of around 10^–3^, and there is currently no example of a CP-TADF OLED that shows both high EQE_max_ (> 20%) and g_EL_ (> 0.1). The trend of decreasing g_EL_ values with increasing EQE_max_ values may be fundamentally rationalized by equation 17, with a large electric transition dipole moment ***μ*** supporting a high Φ_PL_ and thus EQE_max_, but simultaneously limiting g_EL_.

**Figure 81:**
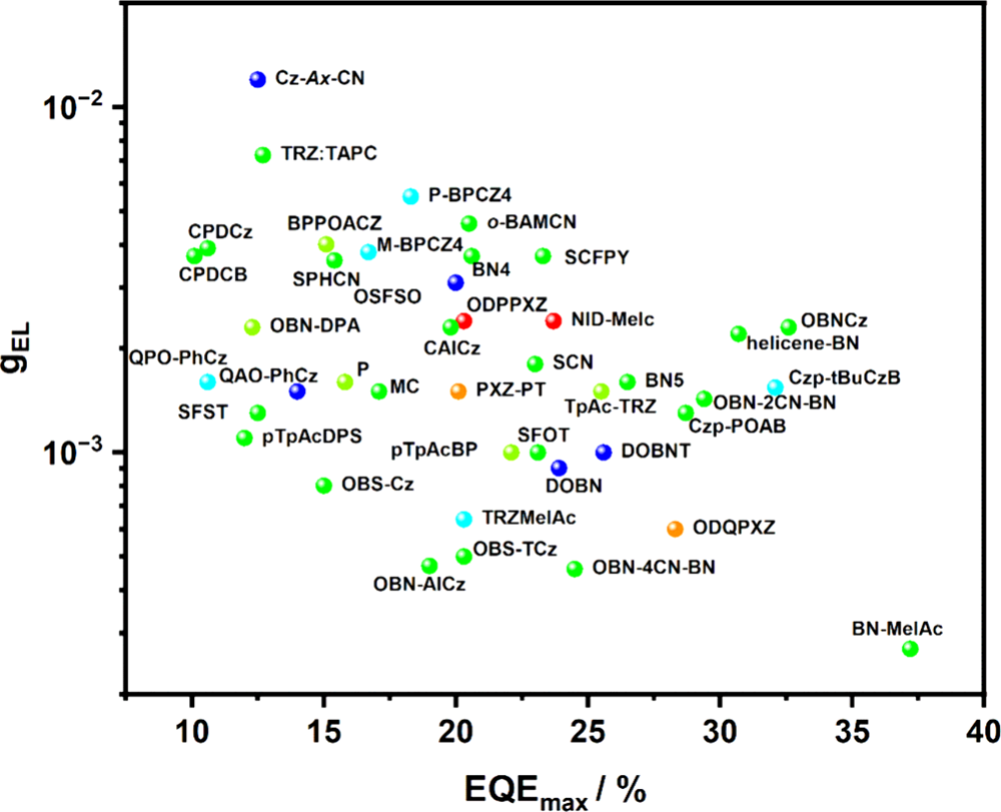
g_EL_ vs EQE_max_ comparison for reported CP TADF OLEDs reviewed in this section (the color points represent the emission color of the devices).

To overcome this apparent design limitation, we predict that strategies involving independent modulation of the electric transition dipole moment and magnetic transition dipole moment on separately optimized compounds (or systems) will become increasingly prominent in the coming years. As an example of the power of this approach, blend films comprising achiral polymers and chiral small molecule additives have resulted in the most robust chiroptical systems reported to date. Notably, g_PL_ values exceeding 0.15 have been reported, mediated by CP-FRET between the conjugated polymer matrix and the chiral small molecule additives, although so far this has been demonstrated for fluorescent systems. Moreover, the integration of chiral macrocycles such as pillar[n]arenes and [n]cycloparaphenylenes with emissive subunits represents another unexplored potential strategy for forming highly luminescent chiroptical systems. This integration allows for the transfer of chiral information from the macrocycles to the emitter, without compromising the photophysical properties of the luminescent component. Integration of TADF emitters within macrocycles in this way could support simultaneous efficient triplet harvesting in OLEDs and high g_PL_, in a manner analogous to current hyperfluorescence approaches (Section 17). Towards these outcomes, a more robust understanding of the design of chiral emitters (with or without TADF properties), chirality-preserving energy transfer, and optimized preparation processes for high-performance CP-TADF OLEDs will be essential to unlocking further performance and utility in these intriguing materials.

## 8 TADF Exciplex Emitters

### 8.1 Introduction

Sections 3–5 showcased emitter designs based on twisted donor-acceptor compounds where electronic communication is mediated through bond across the π-network. Another strategy is to weakly couple donor and acceptor motifs through space by engineering π-stacking interactions. Similar to intramolecular CT states in covalently linked TADF molecules (see Section 12), TADF can also arise from intermolecular CT states created by photo/electrical excitation of mixtures of distinct electron-donor and electron-acceptor molecules. The intermolecular CT state is formed by the transition of an electron from the LUMO of the excited-state donor to the LUMO of the acceptor, forming an exciton with the hole on the donor HOMO and electron on the acceptor LUMO. Since there is no interaction in the ground state, the emissive species is termed an excited-state complex (exciplex).^87^ The mechanism behind exciplex TADF is then analogous to conventional TADF molecules, where a sufficiently small Δ*E*_ST_ allows RISC to occur at ambient temperatures. This outcome is effectively an intermolecular analogue to the TSCT excited states that form when pseudo co-facially oriented donor and acceptor motifs, attached to a common scaffold, are electronically coupled (Figure 82).^671^ By modulating the energies of the HOMO of the donor and the LUMO of the acceptor and the distance between the two molecules, it is then possible to manipulate the exciplex emission wavelength in a straightforward, though perhaps less controllable, manner compared to TBCT or TSCT compounds. The T_1_ energy of the exciplex CT state is also typically lower than the LE triplet energies of the (donor) D or acceptor (A) molecules, assisting RISC by enforcing strong exciton confinement on the exciplex pair, although this confinement has been shown in some cases to be not complete and some diffusion can occur.^672^

**Figure 82:**
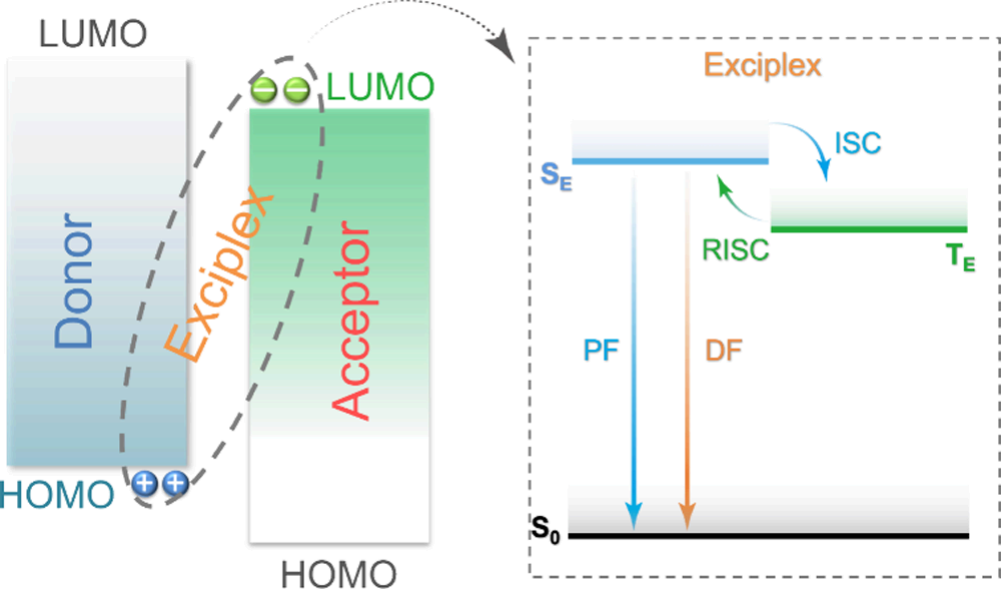
Simplified energy level diagram of donor and acceptor molecules and their interaction in a bimolecular exciplex. On the left is a representation of exciton formation, and on the right is a Jablonski diagram showing the relative energies of the singlet, S_E_, and the triplet, T_E_, states of the exciplex.

There are two key routes to prepare exciplex emitter films: bulk heterojunctions, and bilayer structures, also known as interfacial exciplexes. In the former, the donor and the acceptor materials are blended and/or co-deposited at a specific weight/volume ratio that is typically 1:1, allowing for the formation of interpenetrating networks of the materials with large contact surface area, facilitating the interaction between donor and acceptor compounds. For interfacial exciplexes, separate layers of the electron donor and acceptor materials are sequentially deposited. Interaction and exciplex formation can only occur at the interface between the two layers, although with the benefit of a considerably simpler fabrication.^671^ In this review we denote bulk exciplexes by [Donor:​Acceptor], with interfacial exciplexes represented by [Donor/Acceptor]. In both scenarios, as the electron-donating and accepting materials popular for exciplexes usually also possess excellent individual electron or hole transporting properties, the same materials are often used as charge transport layers, leading to simplified device architectures. Figure 91, at the end of this section, showcases the most efficient exciplex OLEDs in terms of EQE_max_ at specific color points that will be covered in this section.

### 8.2 Materials Development

Not long after the pioneering studies of organic TADF OLEDs published by Adachi and co-workers in 2011 and 2012,^76,673^ the first exciplex TADF OLED was reported by the same group in a series of two reports.^674,675^ In these articles three bulk heterojunction devices were fabricated using the same donor ***m*-MTDATA** (Figure 83), blended with one of three different acceptors: **3TPYMB**, ***t*-Bu-PBD**, and **PPT** (Figure 84) in 1:1 doping ratios. The device with ***m*-MTDATA:​*t*-Bu-PBD** showed an EQE_max_ of 2.0%, while for the device with ***m*-MTDATA:​3TPYMB** the EQE_max_ was higher at 5.4%, and for the device with ***m*-MTDATA:​PPT** the EQE_max_ was the highest at 10%. While these values are low by current standards, the devices were considered promising at the time especially given the low film Φ_PL_ of 20, 26, and 29%, respectively, indicating strong triplet harvesting ability of the exciplexes. The poor OLED performance was also partially explained by the energy levels of the exciplex allowing for exciton migration out of the emissive layer and into the ***m*-MTDATA** hole-transporting layer.

**Figure 83:**
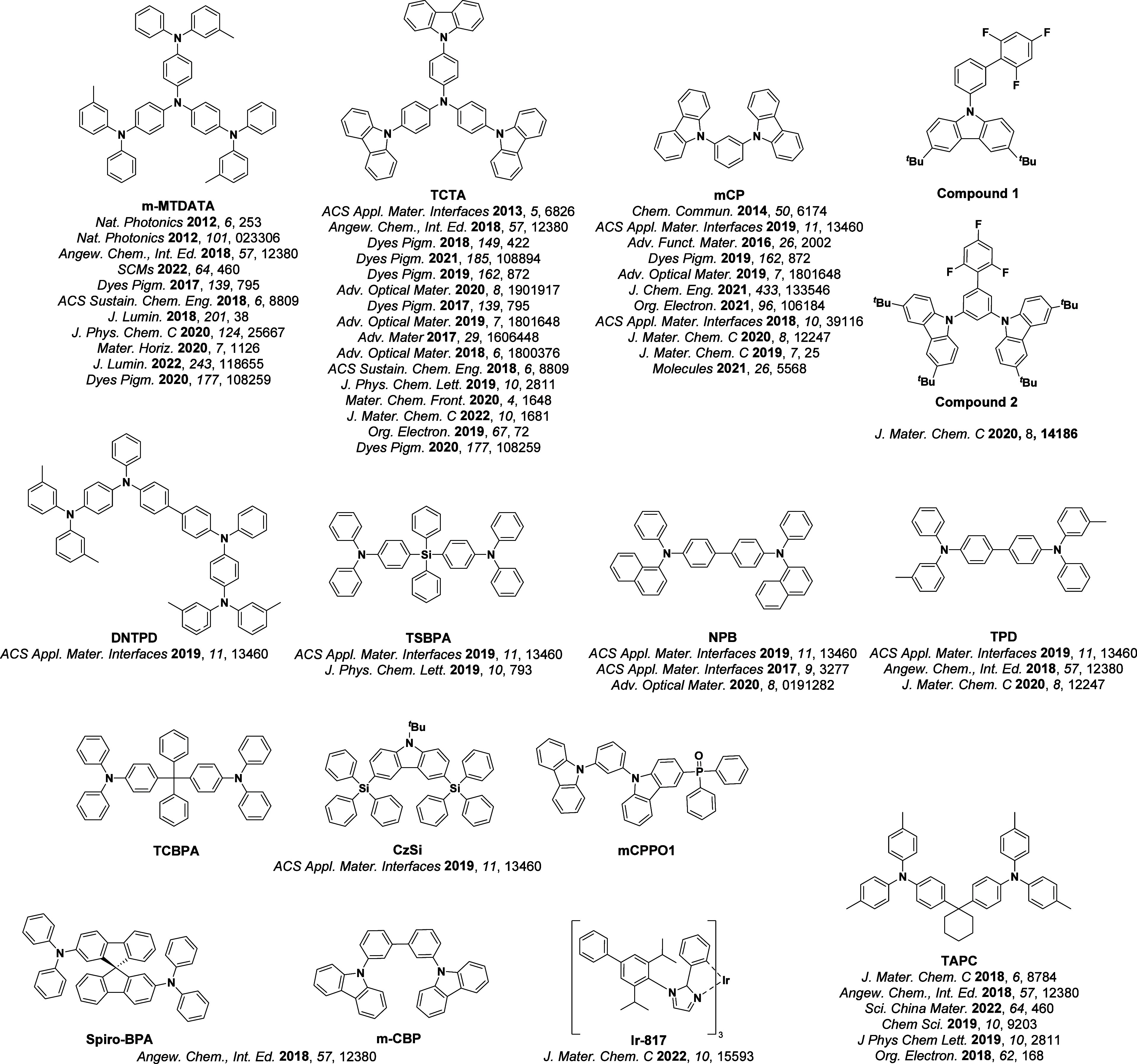
Chemical structures of electron donor molecules used in TADF exciplex systems.

**Figure 84:**
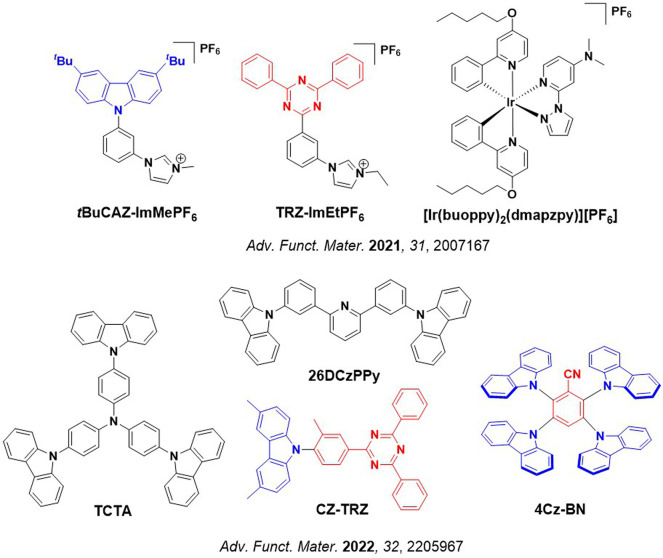
Chemical structures of electron acceptor molecules used in TADF exciplex systems.

In 2013, Hung *et al*. published a study comparing the performance of TADF exciplex OLEDs with either interfacial or bulk heterojunction structures (1:1 ratio). The devices were produced with hole-transporting **TCTA** (Figure 83) as the donor and electron-transporting **3P-T2T** (Figure 84) as the acceptor. The bilayer interface device showed yellow emission at λ_EL_ of 544 nm, an EQE_max_ of 7.7%, and very low efficiency roll-off with EQE_1000_ of 6.0%. The bulk heterojunction device displayed slightly higher EQE_max/1000_ values of 7.8 and 7.7%, respectively. The greater surface area of the interpenetrating networks of donor and acceptor molecules in the bulk heterojunction device therefore resulted in a more efficient emitting layer when compared with the bilayer device.^676^ Regardless, both types of devices showed comparable and promising performance, supported by the balanced hole- and electron-transporting properties of the donor and acceptor molecules of the exciplex.

The first bulk heterojunction exciplex TADF devices with different ratios of D and A molecules were reported by Li *et al*. in 2014. In this study the acceptor **HAP-3MF** (Figure 84) was used in various doping percentages (8, 25, and 50 wt%, see Table S5) with the remainder made up by donor **mCP** (Figure 83). The best results were obtained for the device with 92:8% **mCP:​HAP-3MF**, which emitted at λ_EL_ of 538 nm and showed a EQE_max_ of 11.3%, supported by a Φ_PL_ of 66.1% and τ_d_ of 1.7 and 5.7 μs (Δ*E*_ST_ and CIE coordinates were not provided). The authors contended that by increasing the concentration of **HAP-3MF** beyond 8 wt%, deleterious concentration quenching would occur resulting in the lower EQE. These changes in composition likely also tune the charge transport within the emissive layer, and hence the size and position of the recombination zone.^677^

Due to their typically lower EQEs compared to D-A TADF OLEDs, interest in exciplexes subsequently waned, reflected in the relatively small numbers of publications in the mid-to-late 2010s. However, in 2019 Chapran *et al*. published a study comparing a series of exciplex blends (1:1 wt% ratio) using the now-popular **PO-T2T** as the acceptor with different donors (Table S5). Aside from reporting an impressive EQE_max_ of 20% for the green-emitting **TSBPA:​PO-T2T** devices (λ_EL_ = 528 nm, Figure 83), the devices showed a highest reported maximum current efficiency of 60.9 cd A^–1^ and maximum power efficiency of 71 lm W^–1^.^186^ The study also demonstrated that by changing the donor in the exciplex blend, the emission wavelength could be systematically modulated. The devices with **NPB:​PO-T2T** and **TPD:​PO-T2T** (Figure 83) both showed orange-yellow emission (λ_EL_ = 585 nm) and EQE_max_ of 1.7 and 2.4% respectively. According to the authors, these low EQE_max_ values are related to the high rate of internal conversion, and therefore a low TADF contribution to the emission of the blends despite their near 0 Δ*E*_ST_.^186^ The devices with **TSPBA:​PO-T2T** and **TCBPA:​PO-T2T** emitted at λ_EL_ = 528 and 542 nm, and had CIE color coordinates of (0.33, 0.57) and (0.38, 0.56), respectively. Interestingly, while these exciplex systems both showed excellent Φ_PL_ of 100 and 93%, respectively, only the device using **TSPBA:​PO-T2T** showed a relatively high EQE_max_ of 20.0% (EQE_max_ of 12.8% was reported for the **TCBPA-PO-T2T** devices). According to the authors, this difference in OLED performance is due to the formation of electron traps in the **TCBPA:​PO-T2T** devices, which is detrimental to their performance. In the same study, the authors reported a sky-blue exciplex TADF OLED with **mCP:​PO-T2T**, which showed CIE coordinates of (0.16, 0.28) and improved EQE_max_ of 16.0%, supported by the short τ_d_ ∼of 2 μs and Δ*E*_ST_ < 0.01 eV reported for this blend. An exciplex TADF OLED showing a deeper blue emission [CIE coordinates of (0.16, 0.21)] was achieved using **CzSi:​PO-T2T** (Figure 83), though the EQE_max_ was only 6.1%. This exciplex blend has a Δ*E*_ST_ of 0.10 eV leading to a τ_d_ of 6.3 μs. According to the authors, at the time of their report this device represented one of best known blue exciplex TADF OLEDs.^186^ Blends with **mCPPO1:​PO-T2T** and **DNTPD:​PO-T2T** (Figure 83) both emitted at λ_PL_ of 480 nm; however, only the former was TADF-active, which was surprising given the near-zero Δ*E*_ST_ of both. Devices with **mCPPO1:​PO-T2T** showed sky-blue emission with CIE coordinates of (0.16, 0.29) and an EQE_max_ of 6.5%.^186^

Keruckiene *et al*. also employed **PO-T2T** to fabricate TADF exciplex systems with novel donors **Compound 1** and **Compound 2** (Figure 83), bearing trifluorophenyl and carbazole moieties *meta* disposed to each other, through a phenylene spacer. These two exciplexes emit at λ_PL_ of 489 and 470 nm, and are TADF-active with τ_d_ of 865 and 879 ns (Table S5), all respectively. Even though the reported Φ_PL_ for the blends are only 4 and 2% in air, the devices showed relatively high EQEs of 6.5 and 7.8%, values that the authors themselves noted do not easily correlate with each other. The device with an extra carbazole donor (**Compound 2**) presented the best overall efficiency of 7.8%, showing maximum CE of 24.8 cd A^–1^ and PE of 12.2 lm W^–1^.^678^ As with much of the TADF exciplex research, it is difficult to disentangle whether the improved device performance arises from intrinsically superior exciplex photophysics, or from the tuning of charge transport properties inextricably linked to the use of the different donor materials.

Wu *et al*. reported exciplex blends between **TAPC** (Figure 83) as the donor and two new acceptor molecules **CbPyCN** and **CzPyCN** (Figure 84) that act both as the emitter and as host materials for PhOLEDs. The devices with **TAPC**:​**CzPyCN** and **TAPC**:​**CbPyCN** emitted at λ_EL_ of 530 and 520 nm and showed EQE_max_ of 7.4 and 9.1%, respectively. The higher device efficiency was ascribed to the more electron-deficient character of **CbPyCN** compared to **CzPyCN**, which led to better charge balance and thus less-active loss channels for excitons located in the middle of the emitting layer.^679^

Mamada *et al*. outlined the importance of closely aligned LE and CT states in exciplex systems, using boron-based electron acceptors **BFPPy-DPE** (**BFPD**) and **BPPy-DPE (BPD)** (Figure 84) in combination with ***m*-MTDATA**, **TPD**, **TAPC**, **TCTA**, **Spiro-BPA**, and ***m*-CBP** donors (Figure 83). Not unexpectedly, the highest devices efficiencies (Table S5) were achieved when charge transfer (^1^CT and ^3^CT) and ^3^LE are closely aligned, allowing ^3^LE to be involved in the RISC process. The concentration ratio of the blend films also plays an important role, since it can modulate the energies of the CT states. The closest alignment of the ^1^CT, ^3^CT, and ^3^LE states was found in the **BFPD:​TAPC** (1:1) exciplex system, which emits at λ_PL_ = 501 nm, has a Φ_PL_ of 50.2%, and a Δ*E*_ST_ of 0.04 eV. The device with this blend showed an EQE_max_ of 10.5% at λ_EL_ of 518 nm. However, severe efficiency roll-off was observed, with an achieved maximum luminance of only 1700 cd m^–2^.^680^

Cao *et al*. reported cyano-substituted spiro­[fluor­ine-9,9′-xan­thene] (SFX) acceptors 2-carbo­ni­trile-spiro­[fluor­ene-9,9′-xan­thene] and 2,7-di­carbo­nitrile-spiro­[fluor­ene-9,9′-xan­thene] (**CNSFX** and **DCNSFX**, Figure 84) that were combined with **TCTA** to form bulk heterojunction exciplexes. Only the **TCTA:​DCNSFX** blend showed TADF emission, with a Δ*E*_ST_ of 0.05 eV and a τ_d_ of 4.67 μs. The blend emits at λ_PL_ of 520 nm and has a Φ_PL_ of 31%. The optimized device emitted at λ_EL_ of 520 nm with CIE coordinates of (0.33, 0.52), and showed a relatively low EQE_max_ of 3.0%. The **TCTA:​CNSFX** exciplex emits at λ_PL_ of 448 nm, has a Φ_PL_ of 15%, a much larger Δ*E*_ST_ of 0.32 eV, and a τ_PL_ of 65.9 ns.^681^ Cao *et al*. later used similar SFX acceptors decorated with triazines (**TRZSFX** and **DTRZSFX**, Figure 84) in combination with **TCTA** as the donor (Table S5). The exciplex **TCTA:​TRZSFX** emits at λ_PL_ of 510 nm, has a Δ*E*_ST_ of 0.03 eV, a very short τ_d_ of 0.18 μs, and high Φ_PL_ of 81%, while the blend **TCTA:​DTRZSFX** emits at λ_PL_ of 539 nm, has a Δ*E*_ST_ of 0.06 eV, a comparably short τ_d_ of 0.28 μs, but a much lower Φ_PL_ of 41%. Devices with **TCTA:​TRZSFX** exhibited higher overall efficiency, with EQE_max_, CE_max_, and PE_max_, of 22.5%, 79.6 cd A^–1^, and 78.1 lm W^–1^, respectively, at CIE coordinates of (0.35, 0.60). Not surprisingly considering its Φ_PL_, the devices with **TCTA:​DTRZSFX** showed a much lower EQE_max_ of 9.7% at CIE coordinates of (0.44, 0.51).^682^

Chapran *et al*. reported the use of a variety of electron-rich materials acting as donors, such as **mCP** and **TCTA**, together with phthalimide derivatives as acceptors to form a range of TADF exciplex systems. By varying both donor and acceptor components, a series of 20 exciplex OLEDs were studied (see Table S5). The highest efficiency device employed **mCP**:​**4-BpPht** (Figure 84), which emitted at CIE coordinates of (0.24, 0.41) (λ_PL_ of 497 nm) and showed an EQE_max_ of 2.9%. Despite this blend having a small Δ*E*_ST_ of 0.06 eV and short of τ_d_ of 0.42 μs, its low Φ_PL_ of 26% explains the low EQE_max_ of the device. Despite the low EQE, comparing to the Φ_PL_ the authors nonetheless concluded that there must be active harvesting of triplet excitons in the device.^683^

Zhang *et al*. reported the use of a sky-blue phosphorescent complex **Ir-817** (Figure 83) as donor motif in combination with the acceptor compounds **B2PyMPM**, **B3PyMPM**, and **B4PyMPM** (Figure 84) to produce a series of TADF exciplex OLEDs showing deep-red-to-NIR emission. The decrease in non-radiative decay and higher emission efficiency observed in these blends was attributed to the strong spin-orbit coupling generated by the iridium atom, which contributes to faster *k*_RISC_ as well as in promoting phosphorescent decay from T_1_ to S_0_. The blends with **Ir-817:​B2PyMPM**, **Ir-817:​B3PyMPM**, and **Ir-817:​B4PyMPM** emit at λ_PL_ of 606, 632, and 642 nm, have Φ_PL_ of 13.7, 7.2, and 4.6%, τ_d_ of 11.8, 9.6, and 9.1 μs, and Δ*E*_ST_ of 0.01, 0.02, and 0.02 eV, all respectively. The OLEDs showed EQE_max_ of 3.1, 1.5, and 1.0%, emitting at λ_EL_ of 620, 640, and 672 nm [CIE coordinates of (0.58, 0.42), (0.62, 0.37), and (0.66, 0.33)]. By further increasing the strength of the acceptors in the use of **TRZ-1SO_2_**, **TRZ-2SO_2_**, and **TRZ-3SO_2_** (Figure 84), a stronger red-shift in the blends was observed with emission at λ_PL_ of 647, 666, and 698 nm, respectively and Δ*E*_ST_ of 0.02 eV for all three blends. The NIR devices emitted at λ_EL_ of 658, 700, and 746 nm, and showed EQE_max_ of 0.26, 0.22, and 0.20%, respectively.^684^

### 8.3 TADF Compounds Applied as Either Donors or Acceptors in Exciplex Systems

Beyond mixing separate fluorescent D and A molecules to form TADF exciplexes, D-A TADF materials can themselves be used, acting as either the donor or the acceptor in the blend. Lui *et al*. reported such an exciplex consisting of **PO-T2T** as the acceptor and **MAC** as a D-A TADF donor (Figure 85). As an emitter in its own right, **MAC**, which is a composed of a DMAC donor and a 3-meth­yl-1*H*-iso­chro­men-1-one acceptor, has a small Δ*E*_ST_ of 0.02 eV. The study compared the EQE_max_ of a reference exciplex device using a blend of 1:1 **mCP:​PO-T2T**, and a device with 1:1 **MAC:​PO-T2T**. The former emits at λ_PL_ of 472 nm and has Δ*E*_ST_ of 0.01 eV, while **MAC:​PO-T2T** has a similar Δ*E*_ST_ of 0.014 eV, but emitting at λ_PL_ of 514 nm. Even though the Φ_PL_ of the blends are effectively the same under air (7.3 and 8.0%, respectively) there was a significant improvement in the device EQE_max_, increasing from 8.6% for the device with **mCP:​PO-T2T** [λ_EL_ of 476 nm and CIE coordinates (0.17, 0.26)] to 13.1% for the device with **MAC:​PO-T2T** [λ_EL_ = 516 nm and CIE coordinates (0.31, 0.55)]. This improvement in EQE_max_ could be increased further to 17.8% by modifying the blend ratio to 7:3 wt% **MAC:​PO-T2T**, which at the time of publication was one of the highest reported efficiencies for TADF exciplex OLEDs. This outstanding result was attributed to the parallel RISC processes in the TADF donor molecule and in the exciplex pair, resulting in higher triplet exciton harvesting efficiencies.^685^

**Figure 85:**
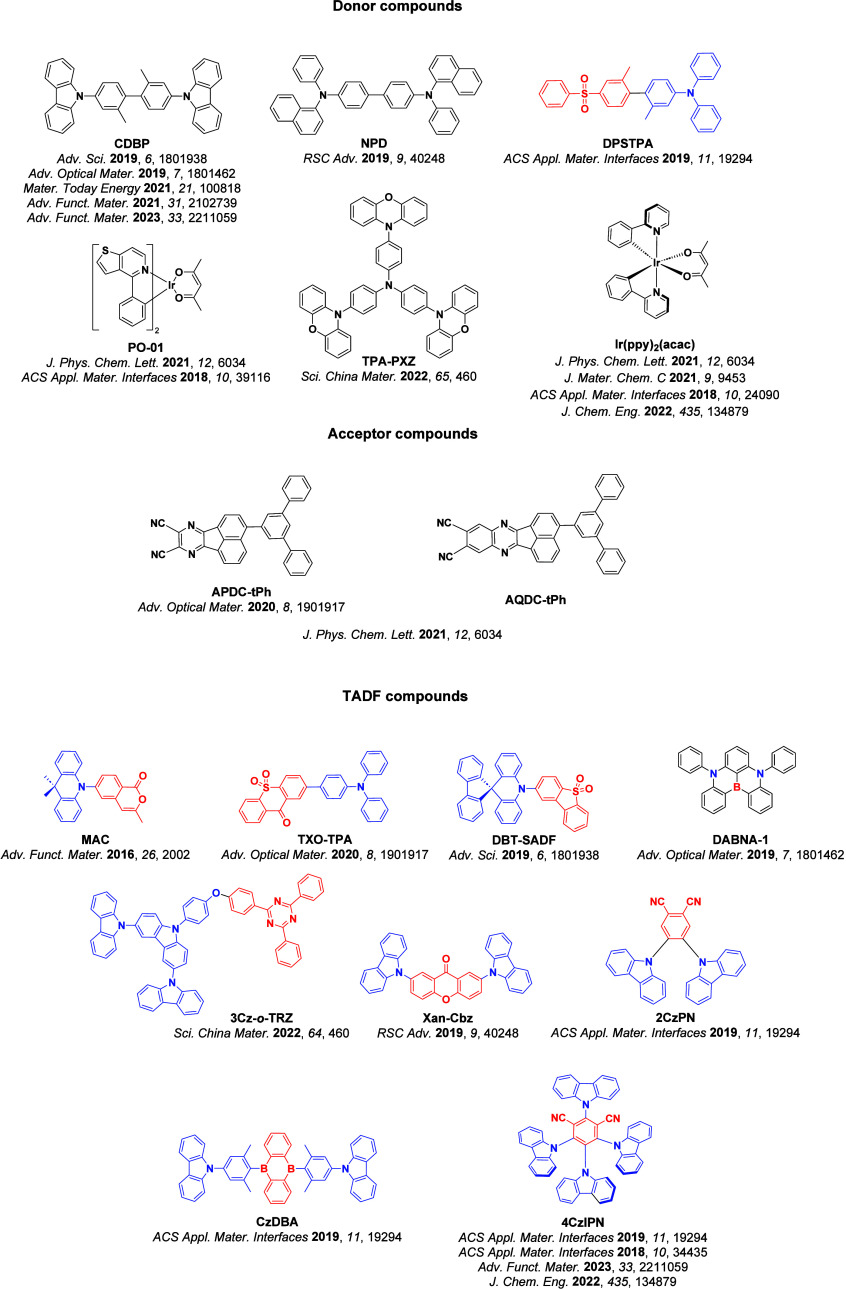
Chemical structures of donor and acceptor molecules employed in exciplexes with a TADF molecule as a component of the exciplex (the blue color signifies donor moieties, while the red color signifies acceptor moieties).

Zhang *et al*. proposed a similar strategy to improve exciton utilization in TADF exciplex emitters. A three-component exciplex featuring **CDBP** (Figure 85) and the TADF compound **DBT-SADF** (Figure 85) as the donors along with **PO-T2T** as the acceptor was investigated. Three separate RISC channels operating in **DBT-SADF**, **DBT-SADF:​PO-T2T**, and **CDBP:​PO-T2T** were posited to exist, evidenced by an increase of the Φ_PL_ from 38% for **DBT-SADF:​PO-T2T** to 61% (or the three-component exciplex. Additionally, a relatively fast combined *k*_RISC_ of 14.2 × 10^5^ s^–1^ was reported. Devices fabricated using the ternary mixture showed a low turn-on voltage of 2.4 V, EQE_max_ of 20.5%, CE_max_ of 60.0 cd A^–1^, and PE_max_ of 69.7 lm W^–1^.^686^ A ternary TADF exciplex system was also reported by Jeon *et al*.^687^ who employed a mixture of **CDBP** donor, **PO-T2T** acceptor, and different ratios of the MR-TADF emitter **DABNA-1** as an additional donor (Figure 85). The 1:1 blends of **CDBP:​PO-T2T** and **PO-T2T:​DABNA-1** emit at λ_PL_ of 494 and 550 nm, have Φ_PL_ of 53 and 46%, and τ_d_ of 17.2 and 14.7 μs respectively, and the ternary blends of **CDBP:​PO-T2T:​DABNA-1** (Table S6) display similar photophysical properties. Ternary blends with ratios of 47.5:47.5:5, 45:45:10 and 40:40:20 all emit at λ_PL_ of 550 nm, have the same τ_d_ of 15.1 μs, and have Φ_PL_ of 67, 69, and 50%, respectively (no Δ*E*_ST_ values were reported). Devices with 47.5:47.5:5.0 **CDBP:​PO-T2T:​DABNA-1** showed the highest EQE_max_ of the series at 17.5% [CIE coordinates of (0.31, 0.58)], while devices with 45:45:10 and 40:40:20 **CDBP:​PO-T2T:​DABNA-1** achieved EQE_max_ of 16.9 and 13.4% [CIE coordinates of (0.34, 0.60) and (0.37, 0.60)], respectively. These efficiencies were partly attributed to energy transfer from the high energy exciplex (**CDBP:​PO-T2T**, acting as a host), to the low-energy exciplex **DABNA-1:​PO-T2T** acting as emissive dopant.

Siddiqui *et al*. (2019) demonstrated that the color emitted by an exciplex OLED could be modulated by simply changing the applied voltage. A carbazole-xanthone-based D-A-D TADF material (**Xan-Cbz**, Figure 85), emitting at λ_PL_ of 470 nm and having a Δ*E*_ST_ of 0.32 eV with a τ_d_ of 3.8 μs, was used as an acceptor alongside donor **NPD** (Figure 85). The device fabricated using a bilayer structure of **NPD/Xan-Cbz** showed dual emission with λ_EL_ at 465 nm, associated with the TADF acceptor, as well as at 525 nm corresponding to the exciplex emission. By increasing the voltage, and hence changing the ratio of molecular/exciplex excitons across the interface, the ratio of two peaks changed along with the color of the device. Additional photophysical data for this interfacial exciplex was not provided by the authors, although similar voltage-dependant color changes in an interfacial exciplex using simpler materials were studied in detail previously.^688,689^

Wu *et al*. demonstrated the use of TADF compounds as electron acceptors in a study that showed how the intermolecular distancing between donor and acceptor molecules affects the overall efficiency of the exciplex OLEDs. Ambipolar **DPSTPA** (Δ*E*_ST_ = 0.27 eV), was used as the donor alongside each of three TADF acceptors: **2CzPN**, **CzDBA**, and **4CzIPN** (Figure 85). Devices with 3:1 wt% of **DPSTPA:​2CzPN** or **DPSTPA:​CzDBA** emitted at λ_EL_ of 544 and 592 nm, with EQE_max_ of 19.0 and 14.6%, CE_max_ of 59.9 and 29.6 cd A^–1^, and PE_max_ of 62.7 and 31.0 lm W^–1^, all respectively. However, devices with **DPSTPA:​4CzIPN** (3:1 wt%) achieved a much lower EQE_max_ of 3.8%. This poor performance was attributed to the larger distances between **DPSTPA** and **4CzIPN** molecules caused by the steric bulk of the carbazole (Cz) groups of **4CzIPN**, which hinders the exciplex-forming interaction of the nitrile groups of **4CzIPN** and the TPA donor of **DPSTPA**.^690^

Hu *et al*. fabricated NIR exciplex OLEDs by combining **APDC-tPh** (Figure 85) as the acceptor along with donors such as **TCTA** and TADF **TXO-TPA** (Figure 85) in different weight percentages (Table S6). Due to the additional RISC pathway associated with the TADF acceptor, the 1:1 ratio device with **TXO-TPA:​APDC-tPh** showed an EQE_max_ of 1.27% at λ_PL_ = 704 nm (a respectable efficiency at this NIR wavelength), contrasting with an EQE_max_ of 0.09% at λ_EL_ = 730 nm in the device with a 1:1 ratio of **TCTA:​APDC-tPh**.^691^ The same authors also explored different ratios of an exciplex blend consisting of the phosphorescent complexes **Ir(ppy)_2_acac** (Figure 85) and **PO-01** as the donors, while **APDC-tPh** and **AQDC-tPh** (Figure 85) were employed as the acceptors (Table S6). The optimized device with 15:85 **PO-01:​AQDC-tPh** emitted at λ_EL_ of 750 nm and showed an EQE_max_ of 0.23%, withthe triplet excitons being harvested by both the exciplex and the phosphor donor.^692^

Yang *et al*. reported TADF material **3Cz-o-TRz** containing a D-o-A structure (Figure 85), which was employed as both a donor or acceptor in different exciplex blends (Table S6). A total of six blends were fabricated using the acceptors **B3PyMPM**, **B4PyMPM**, and **PO-T2T**, and with donor compounds **TAPC**, **TPA-PXZ**, and ***m*-MTDATA** (Figure 85). The best performing devices had EML compositions of **3Cz-*o*-TRz:​PO-T2T** or **TAPC:​3Cz-*o*-TRz**, both of which showed TADF at λ_PL_ of 510 nm and similar Φ_PL_ and τ_d_ of 66 and 68%, and of 1.5 and 1.8 μs respectively. The respectively devices emitted at λ_EL_ of 516 and 520 nm and showed EQE_max_ of 11.8 and 12.1%. These results prompted fabrication of tandem OLEDs with the mixed heterojunction/interfacial structure **TAPC:​3Cz-*o*-TRz|3Cz-*o*-TRz|3Cz-*o*-TRz:​PO-T2T**, which emitted at λ_EL_ of 516 nm, had low turn-on voltage of 2.4 V, and showed an EQE_max_ of 14.1% with CE_max_ of 43.8 cd A^–1^.^693^

### 8.4 Understanding and Improving Exciplex Efficiency

Hung *et al*. documented an interesting strategy to improve the Φ_PL_ and thus the efficiency of exciplex TADF OLEDs.^694^ The authors claimed that an enhancement in the performance can be achieved by introducing steric bulk onto the donors, thereby weakening the electronic coupling between donor and acceptor molecules. To demonstrate this, two reference blends (1:1 wt% ratio) using **DTAF** or **CPF** (Figure 86) as donor molecules in combination with electron-acceptors **3N-T2T** (Figure 87) and **PO-T2T**, respectively, were fabricated. Their performance (Table S7) was compared to blends using bulkier congener donors **DSDTAF** and **CPTBF** (Figure 86), containing either extra triphenylsilyl (SiPh_3_) groups or *tert*-butyl substituents. The **DSDTAF:​3N-T2T** blend emits at λ_PL_ of 535 nm and has a Φ_PL_ of 59%, with a τ_d_ of 2.54 μs and the corresponding device showing an EQE_max_ of 13.2%. The reference blend **DTAF:​3N-T2T** emits at the same wavelength, has a modestly lower Φ_PL_ of 51%, and has device EQE_max_ also slightly lower at 11.6%. Similarly, **CPTBF:​PO-T2T** emits at λ_PL_ of 480 nm and has an Φ_PL_ of 44% with τ_d_ of 5.86 μs, translating into a device EQE_max_ of 12.5%. The corresponding reference exciplex blend **CPF:​PO-T2T** also emits at 480 nm, has similar Φ_PL_ of 41% and τ_d_ of 2.8 μs, and yet the device showed a considerably reduced EQE_max_ of 9.5%. Although it is thought that the emission of the exciplex can be tuned as result changing the intermolecular distance of donor and acceptor, this work reported no shift in in the λ_PL_.^695^ These results, however, suggested that bulky ^*t*^Bu or SiPh_3_ substituents can improve the Φ_PL_ of exciplex blends and their performance in OLEDs.

**Figure 86:**
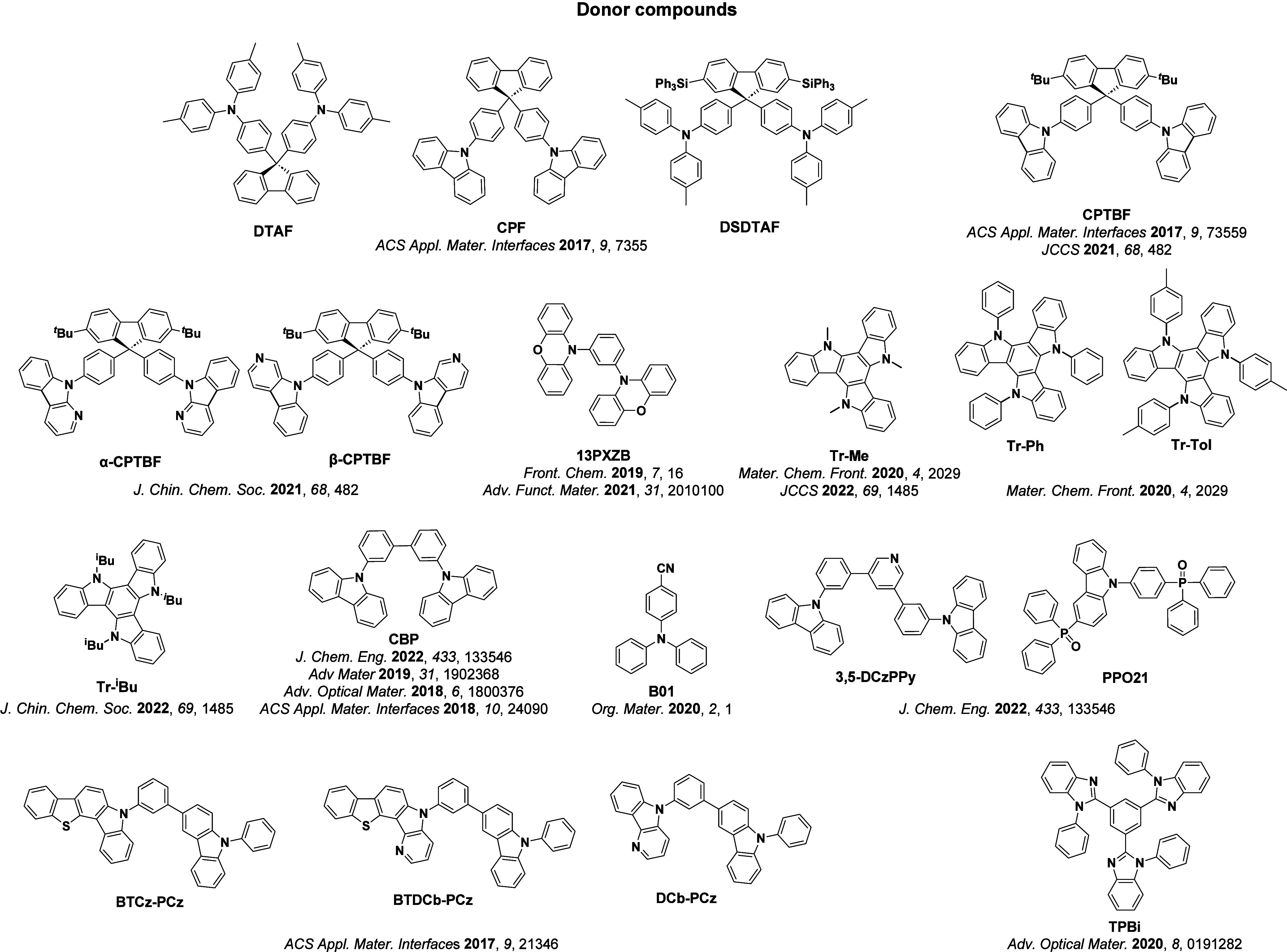
Structures of donor materials used to understand and improve the performance of exciplex systems discussed in this section.

**Figure 87:**
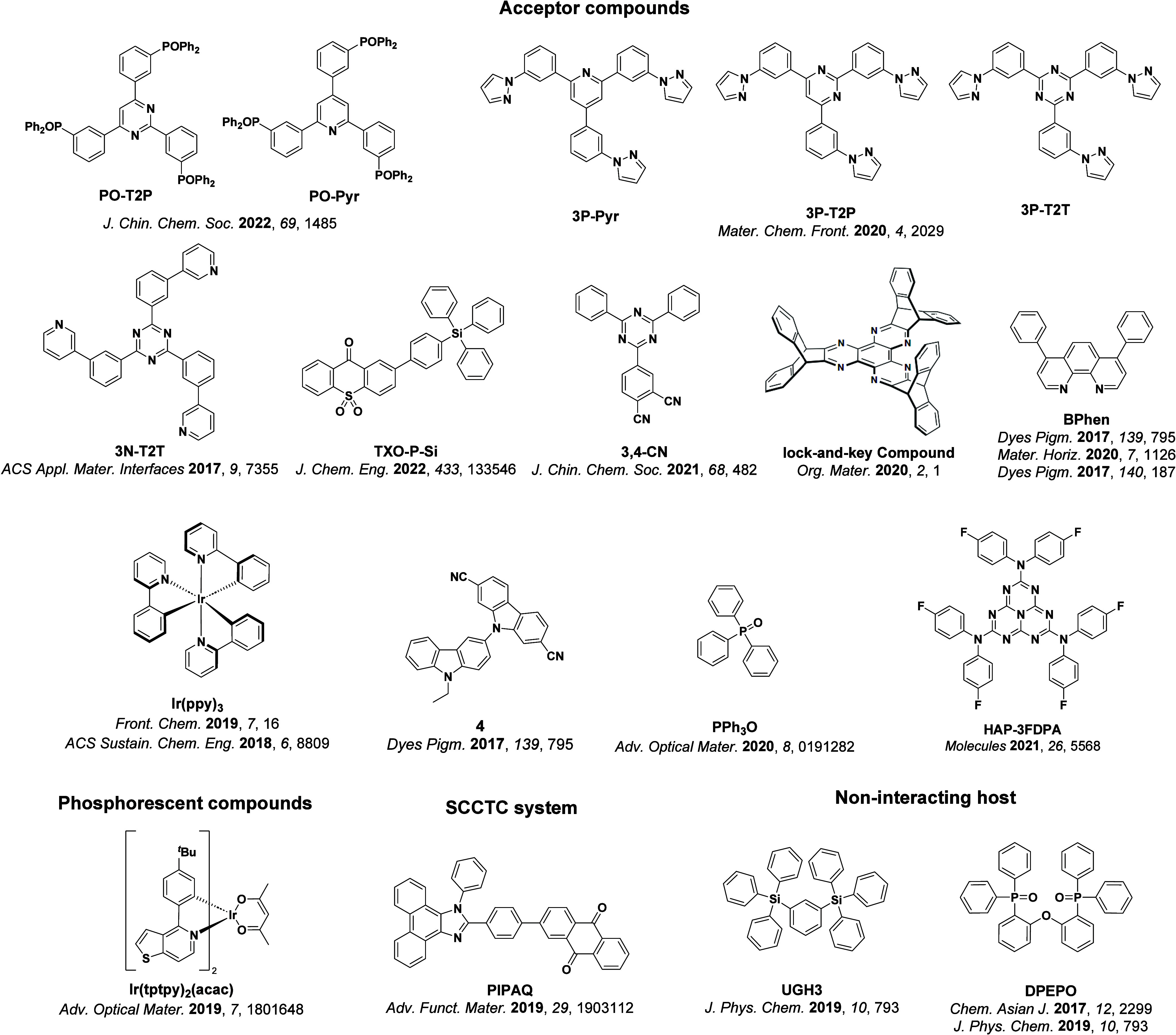
Structures of acceptor materials used to understand and improve the performance of exciplex systems. The structure of **Ir(tptpy)_2_(acac)** was misdrawn in the original publication.^695^

Skuodis *et al*. developed a new approach for the fabrication of TADF exciplex OLEDs in a device featuring both bilayer and bulk structures. A new carbazol-9-yl-substituted 9-ethylcarbazole derivative containing nitrile groups (material **4**, Figure 87) was employed as the acceptor along with standard donor materials. The exciplexes **TCTA:4** and ***m*-MTDATA:4** (1:1 ratio) emit at λ_PL_ of 490 and 584 nm, have Φ_PL_ of 43.8 and 3.8%, and very short τ_d_ of 0.31 and 0.19 μs, respectively. The devices fabricated using the hybrid bulk/interfacial approach, **TCTA:​4/4/Bphen** and ***m*-MTDATA:​4/4/Bphen**, emitted at λ_EL_ of 490 and 600 nm, and showed comparable respective EQE_max_ of 4.2 and 3.2%.^696^ By contrast, a device of **4/Bphen** emitted at λ_EL_ of 475 nm and showed a lower EQE_max_ of 2.0%, demonstrating the contribution of the exciplex to the overall device performance.

Colella *et al*. demonstrated how to simultaneously induce a blue-shift in the emission of an exciplex TADF systems and also improve the Φ_PL_, leading to higher device EQE. This occurs due to weakened electronic coupling between donor and acceptor molecules in the exciplex blend as a third component is added, which reduces the Coulomb binding term of the exciton’s electron and hole and therefore increases the total energy of the emissive CT state. Previously reported blend **TSPBA:​PO-T2T** (1:1 ratio) was diluted with different concentrations of a third non-interacting host, either UGH-3 or DPEPO (Figure 87). By adding 90 vol% of UGH-3, the photoluminescence onset changed from 2.67 (for 1:1 exciplex films) to 2.85 eV. An increase in the Φ_PL_ from 58% in undiluted exciplex films to 80% in a film with 50 vol% UGH-3 was also reported, although the source of this Φ_PL_ enhancement remains a mystery and was later shown to not be universally translatable to improved OLED performance.^697^ For concentrations higher than 50 vol% of the inert host material, the Φ_PL_ began to decrease as the concentration of the exciplex-forming materials decreased. The highest device performance was achieved using 50 vol% of UGH-3, where the device showed an EQE_max_ of 19.2% in comparison with 14.8% for the undiluted exciplex.^698^

Yuan *et al*. also explored the acceptor **PO-T2T** in order to show the importance of spatial distancing between donor and acceptor molecules in exciplex TADF systems.^695^ According to the authors, the potential energy surfaces of the excited states have a strong dependence on this distance. By manipulating the separation between D and A compounds in a blend of **TCTA:​PO-T2T** (1:1 weight ratio) using different weight concentrations **mCP** as a spacer within the bulk exciplex, an enhancement of up to 105% in the EQE_max_ was observed (Table S7). The best results were reported for the blend with a weight ratio of 1:1:3 **TCTA:​PO-T2T:​mCP**, where an EQE_max_ of 8.0% was achieved in comparison with only 3.9% for the device with **TCTA:​PO-T2T**. The addition of the host also affected the Δ*E*_ST,_ decreasing from 0.06 (without **mCP**) to 0.02 eV. As a result of the smaller Δ*E*_ST_ there was an enhancement from 78 to 92% of the fraction of delayed fluorescence contributing to the total PL, and also an increase of the Φ_PL_ from 13 to 37% for films with weight ratio of 1:1:0 and 1:1:3, respectively. It remains unclear though what role competing exciplex formation between **PO-T2T**:​**mCP** plays in this performance enhancement. The authors then used the **TCTA:​PO-T2T**:​**mCP** blend as a host for orange phosphorescent emitter **Ir(tptpy)_2_(acac)** (Figure 87), where the devices emitted at λ_EL_ of 555 nm and showed an EQE_max_ of 21.7%.^695^

In a similar study, Pu *et al*. controlled the distance between donor and acceptor molecules of a TADF exciplex by incorporating an inert spacer layer of up to 70 nm between the layers of an interfacial exciplex system. **TAPC** and **DCA** were used as the respective donor and acceptor for the interfacial exciplex, while **DMA** and **CBP** (Figure 86) were applied as the inert spacers (Table S7). The highest device efficiencies were achieved for the OLEDs with **TAPC**/**DMA** (70 nm)/**DCA** and **TAPC**/**CBP** (20 nm)/**DCA**, with EQE_max_ of 0.86 and 3.0% and emitting at λ_EL_ of 445 and 550 nm respectively. Exciplex formation at both the donor/spacer interface and at the spacer/acceptor interface resulted in long-distance spacer-mediated coupling between the donor and the acceptor.^699^

Zhang *et al*. reported efficient red TADF exciplex devices using a phosphorescent complex as the acceptor moiety. Blends with **PO-T2T** acting unusually here as the donor and with ***fac***-**Ir(ppy)_3_** (Figure 87) as the acceptor have small Δ*E*_ST_ of 0.026 eV, τ_d_ of 2.8 μs, and Φ_PL_ of 23.3%. The devices with **PO-T2T:​Ir(ppy)_3_** (92:8) emitted at λ_EL_ at 604 nm [CIE coordinates of (0.55, 0.44)] and showed an EQE_max_ of 5.0%, CE_max_ of 9.3 cd A^–1^, and PE_max_ of 11.6 lm W^–1^. By contrast, the use of **13PXZB** (Figure 87) as the donor, together with **PO-T2T** as the acceptor (60:40 **13PXZB:​PO-T2T)** as the exciplex emitter resulted in a device that emitted at λ_EL_ at 592 nm [CIE coordinates of (0.52, 0.47)] and showed a lower EQE_max_ of 1.9%. The authors claimed that there is enhanced SOC within the **PO-T2T:​Ir(ppy)_3_** exciplex, associated with the iridium center, which is responsible for faster ISC and RISC processes, which leads to the higher Φ_PL_ of the **PO-T2T:​Ir(ppy)_3_** blend (23.3%, compared to 8.6% **13PXZB:​PO-T2T**) and, hence, also an enhancement in the overall OLED efficiency.^700^

Chen *et al*. published a study claiming to report the first example of a single-component charge transfer complex (SCCTC), showing deep-red-to-NIR TADF. A SCCTC is a molecule that has donor and acceptor moieties that only electronically couple to the respective acceptor and donor groups of neighboring molecules, essentially corresponding to a single-component bulk exciplex material. **PIPAQ** (Figure 87) where the phenanthro[9,10-dimidazole (PI) and anthraquinone (AQ) are the respective donor and acceptor moieties is purported to be one such SCCTC compound. The isolated molecule has a moderate Δ*E*_ST_ of 0.13 eV, yet forms co-facial head-to-tail dimers in neat films which result in exciplex emission at λ_PL_ of 650 nm, having a τ_d_ of 40.5 μs and a Φ_PL_ of 12.3%.^701^ Devices with **PIPAQ** showed an EQE_max_ of 2.1% at CIE coordinates of (0.64, 0.36).^702^

Hu *et al*. showed that aggregation of the donor material can strongly affect device performance due to substantial residual emission from the aggregate.^703^ In their study triazatruxene-based molecules **Tr-Me**, **Tr-Ph**, and **Tr-Tol** (Figure 86) were used as donor materials alongside the acceptors **3P-T2T** and its pyrimidine (**3P-T2P**) and pyridine (**3P-Pyr**) derivatives (Figure 87). The blends using **Tr-Me** showed significant donor aggregation which prevented exciplex formation; however, the other donors blends were promising (Table S7), with the exciplex systems formed using **3P-T2P** as the acceptor showing the best results. Blends of **Tr-Ph:​3P-T2P** and **Tr-Tol:​3P-T2P** showed similar photophysical properties, emitting at λ_PL_ of 526 and 525 nm, having τ_d_ of 1.77 and 2.39 μs, Φ_PL_ of 41 and 40%, and Δ*E*_ST_ of 0.18 and 0.10 eV, all respectively. The devices with **Tr-Ph:​3P-T2P** and **Tr-Tol:​3P-T2P** emitted in the green at CIE coordinates of (0.33, 0.54) and (0.35, 0.54) and showed EQE_max_ of 10.4 and 12.8%.^703^ In a subsequent study, the same group analysed a series of exciplex blends with the goal to suppress donor aggregation. A total of six blends (Table S7) were fabricated using either **Tr-Me** or a triazatruxene-based analogue donor with larger alkyl substituents (**Tr-iBu**, Figure 86) in combination with **PO-T2T**, **PO-T2P**, or **PO-Pyr** (Figure 87) as acceptors. The blends using **Tr-iBu** showed suppressed donor aggregation and blue-shifted emission compared to those using **Tr-Me**, attributed to an increased intermolecular distance between donor and acceptor molecules leading to a destabilized charge transfer states. The highest performing OLEDs were obtained with **Tr-iBu:​PO-T2P** (1:2 ratio), showing λ_EL_ at 560 nm [CIE coordinates of (0.43, 0.54)] and EQE_max_ of 8.3%. Devices with **Tr-iBu:​PO-Pyr** (1:2 ratio), emitted at λ_EL_ of 516 nm [CIE coordinates of (0.27, 0.50)] and showed similar EQE_max_ of 7.5%. **Tr-iBu:​PO-Pyr** was also used as an exciplex host for the emitter **DPy2CN** (Figure 89). The highest efficiency device contained3 wt% **DPy2CN**, and showed an EQE_max_ of 6.3% at CIE coordinates of (0.63, 0.35).^704^ Introducing silyl groups to similarly address molecular aggregation, Wei *et al*. reported a family of green-emissive TADF exciplexes consisting of **TXO-P-Si** (Figure 87) as the acceptor and varying the donor compounds through **mCP**, **CBP**, **3,5-DCzPPy**, and **PPO21** (Figure 86). The highest performance devices included **mCP:​TXO-P-Si** (1:4) and **3,5-DCzPPy:​TXO-P-Si** (1:1), which showed EQE_max_ of 16.9 and 16.1% respectively.^705^ This is due to their relatively higher respective Φ_PL_ of 55.4 and 47.7% and small Δ*E*_ST_ of 0.02 and 0.06 eV.

Chen *et al*. reported weak donor compounds **α-CPTBF** and **β-CPTBF** (Figure 86), where the carbazole moiety of the model donor molecule **CPTBF** was replaced by either an α- or β-carboline. Used in conjunction with **3,4-CN** (Figure 87) as the acceptor, the 1:1 exciplex blends **CPTBF:​3.4-CN**, **α-CPTBF:​3.4-CN**, and **β-CPTBF:​3.4-CN** each showed TADF emission with τ_d_ of 0.12, 0.10, and 0.10 μs, and Φ_PL_ of 18.0, 20.0, and 21.0%, all respectively. The blends **α-CPTBF:​3.4-CN** and **β-CPTBF:​3.4-CN** both emit at λ_PL_ of 504 nm, which is blue-shifted in comparison with the reference blend **CPTBF:​3.4-CN** (λ_PL_= 522 nm). Such a blue-shift is not surprising, since the weaker carboline leads to a deeper HOMO of the donor molecules. The devices with **α-CPTBF:​3,4-CN** emitted at CIE coordinates at (0.30, 0.56) and showed the highest EQE_max_ of the series at 7.6%, with CE_max_ of 25.2 cd A^–1^ and PE_max_ of 25.9 lm W^–1^. The superior performance was mainly attributed to the higher Φ_PL_ and the faster RISC, reflected in the greater contribution of delayed fluorescence to the total emission of the device.^706^

Zhang *et al*. demonstrated the value of introducing intermolecular hydrogen bonds between the donor and acceptor compounds, which were hypothesized to reduce inter- and intra-molecular vibrational relaxation and thus increase Φ_PL_.^707^ Three exciplex systems composed of the donor **13PXZB** (Figure 86) and each of the acceptors **B4PyMPM**, **B3PyMPM**, and **B2PyMPM** were investigated. These were expected to have different numbers of hydrogen bonds between donor and acceptor groups: **13PXZB:​B4PyMPM** having the most intermolecular hydrogen bonds followed by **13PXZB:​B3PyMPM**, while **13PXZB:​B2PyMPM** does not have any hydrogen bonding between D and A. Correlated with this trend, the device with **13PXZB:​B4PyMPM** emitted at λ_EL_ of 560 nm [CIE coordinates of (0.41, 0.55)] and showed the highest EQE_max_ of 14.6% (CE_max_ of 43.1 cd A^–1^, PE_max_ of 48.3 lm W^–1^).

Voll *et al*. explored interlocking molecular donor-acceptor designs using a lock-and-key approach, where acceptor “key” and donor “lock” molecules were tailored to fit each other by supramolecular self-assembly. The acceptor contained a hexa­aza­tri­phen­ylene core flanked by three triptycene moieties, and was partnered with donors featuring triarylamines, tri­aryl­ben­zenes, and tri­aryl­ben­zo­tri­thio­phenes (**lock and key compound**, Figure 87). Only one device was reported, fabricated using triarylamine donor **B01** (Figure 86) in a 1:1 weight ratio D:A blend. This device showed an EQE_max_ of 5.4% and emitted at λ_EL_ = 536 nm, which was significantly red-shifted compared to the film λ_PL_ of 461 nm (τ_d_ of 45.1 μs).^708^

Towards developing elusive blue OLED emission, Wang *et al*. reported δ-carboline derivatives **BTCz-PCz**, **BTDCb-PCz**, and **DCb-PCz** as donors in both bulk and interfacial exciplexes with acceptor **TmPyPB** (Figure 87).^709^ The highest efficiency OLED used **BTDCb-PCz:​TmPyPB** (99:1 wt% ratio), emitting at λ_EL_ of 468 [CIE coordinates (0.16, 0.21)] with Δ*E*_ST_ of 0.05 eV, and with an EQE_max_ of 2.4%, CE_max_ of 4.64 cd A^–1^, and PE_max_ of 2.91 lm W^–1^. On the other hand, the interfacial exciplex device **BTDCb-PCz/TmPyPB** showed an EQE_max_ of only 1.1% at CIE coordinates (0.20, 0.31). The authors attributed the reduced efficiency of the interfacial exciplex device to the recombination zone being very close to the ETL. Guzauskas *et al*. documented that a device with interfacial exciplex **mCP/PO-T2T** emitted at λ_EL_ of 497 nm and showed an EQE_max_ of 8.2%. By thermally annealing the emitting layers after deposition, a red-shift to a λ_EL_ of 570 nm was observed while the EQE_max_ remained the same (Table S7).^710^ Hippola *et al*. demonstrated that with an appropriate device structure, deep blue exciplex OLEDs could be fabricated with **TPBi:​PPh_3_O** (Figure 86 and Figure 87). The OLED emitted at λ_EL_ of 435 nm and showed an EQE_max_ of 4.0%. According to the authors, the EL spectrum arose from the interfacial exciplex between **NPB** and the 5:1 **TPBi:​PPh_3_O** blend.^711^ Li *et al*. fabricated deep-blue devices with 92:8 wt% **mCP:​HAP-3FDPA** (Figure 87), which emitted at λ_PL_ of 433 nm, had Φ_PL_ of 53.2%, and a ΔE_ST_ of 0.09 eV. The devices showed an EQE_max_ of 10.2% at CIE coordinates of (0.16, 0.12), making it the bluest exciplex OLED reported to date.^712^

### 8.5 TADF Exciplex as Hosts

Using a sensitization approach that mirrors hyperfluorescence (see Section 17), many studies now use TADF exciplexes as co-hosts and triplet harvesters for separate terminal emitters. This design allows harvesting of triplet excitons by the exciplex (and sometimes also TADF-active terminal emitters), while avoiding some of the undesirable photophysical properties of exciplexes such as broad CT emission spectra, slow radiative rates, and low Φ_PL_. This approach is enabled by Förster resonance energy transfer (FRET) from the exciplex to the emissive guest,^713,714^ and can be particularly effective in the design of NIR OLEDs. For instance, Huang *et al*. reported an OLED with EQE_max_ of 6.6% at λ_EL_ of 710 nm using **NOz-t-TPA** (Figure 89) doped in the **Tris-PCz:​CN-T2T** exciplex (Figure 88),^713^ while Chen *et al*. reported a device EQE_max_ of 5.3% at λ_EL_ of 774 with an EML of 7 wt% **TTDSF** (Figure 89) doped in **DPSF:​CN-T2T** (Figure 88).^714^ Zhang *et al*. similarly demonstrated the differences in device performance using a ‘passive’ conventional host (**CBP**) compared to a TADF-active exciplex host (**CBP:​PO-T2T**) doped with the same red TADF emitter (**TPA-PZCN**, Figure 89). With **CBP:​PO-T2T** the device EQE_max_ was slightly improved (28.1%) along with a red-shifted electroluminescence indicating more complete energy transfer to the terminal emitter (λ_EL_ of 648 nm, Table S8) comparing favorably to the device with **CBP** as the host (λ_EL_ = 628 nm and EQE_max_ of 27.4%). No other photophysical data were provided.^507^

**Figure 88:**
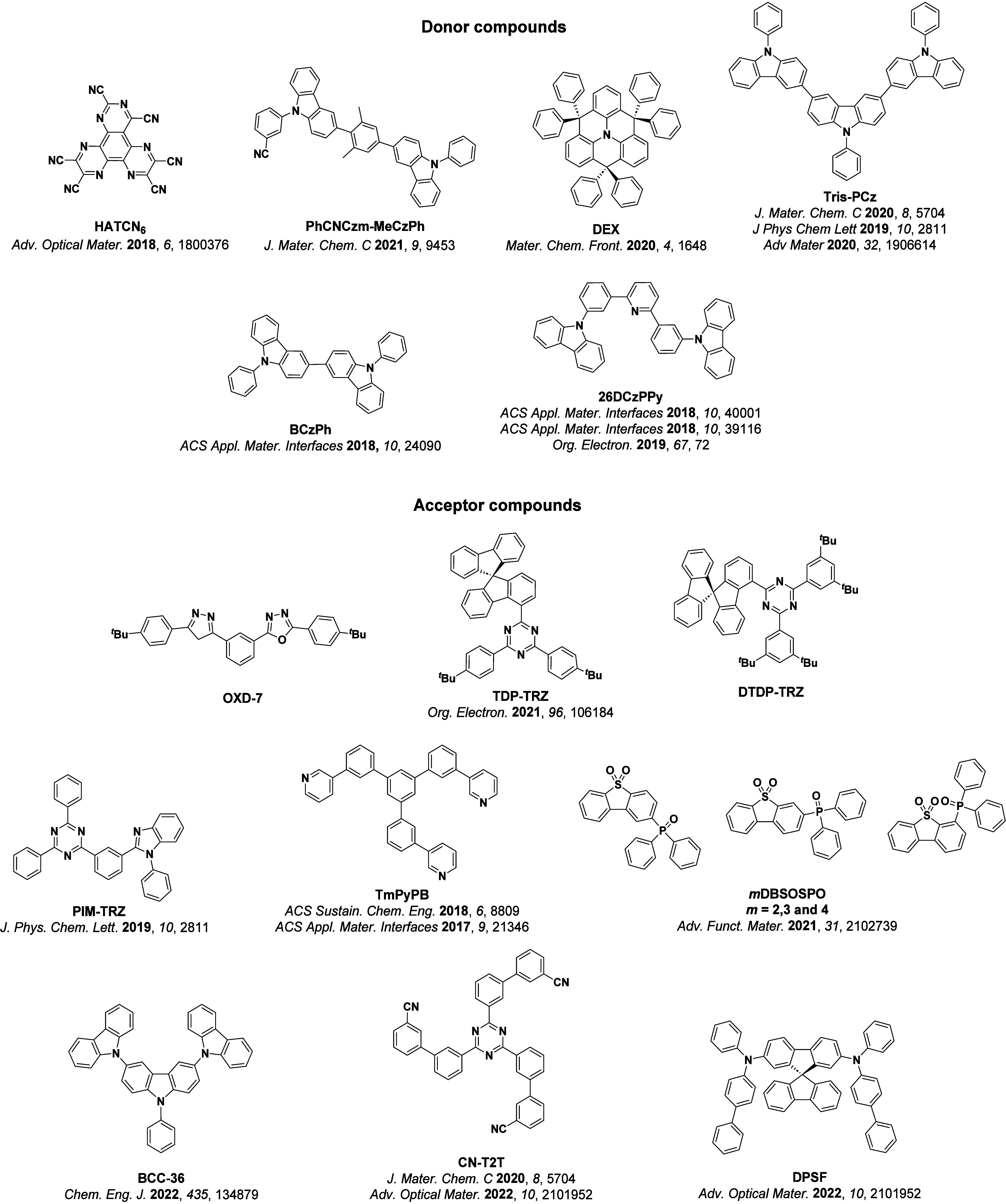
Structures of donor and acceptor materials used in either exciplex hosts or solution-processed TADF exciplex systems.

**Figure 89:**
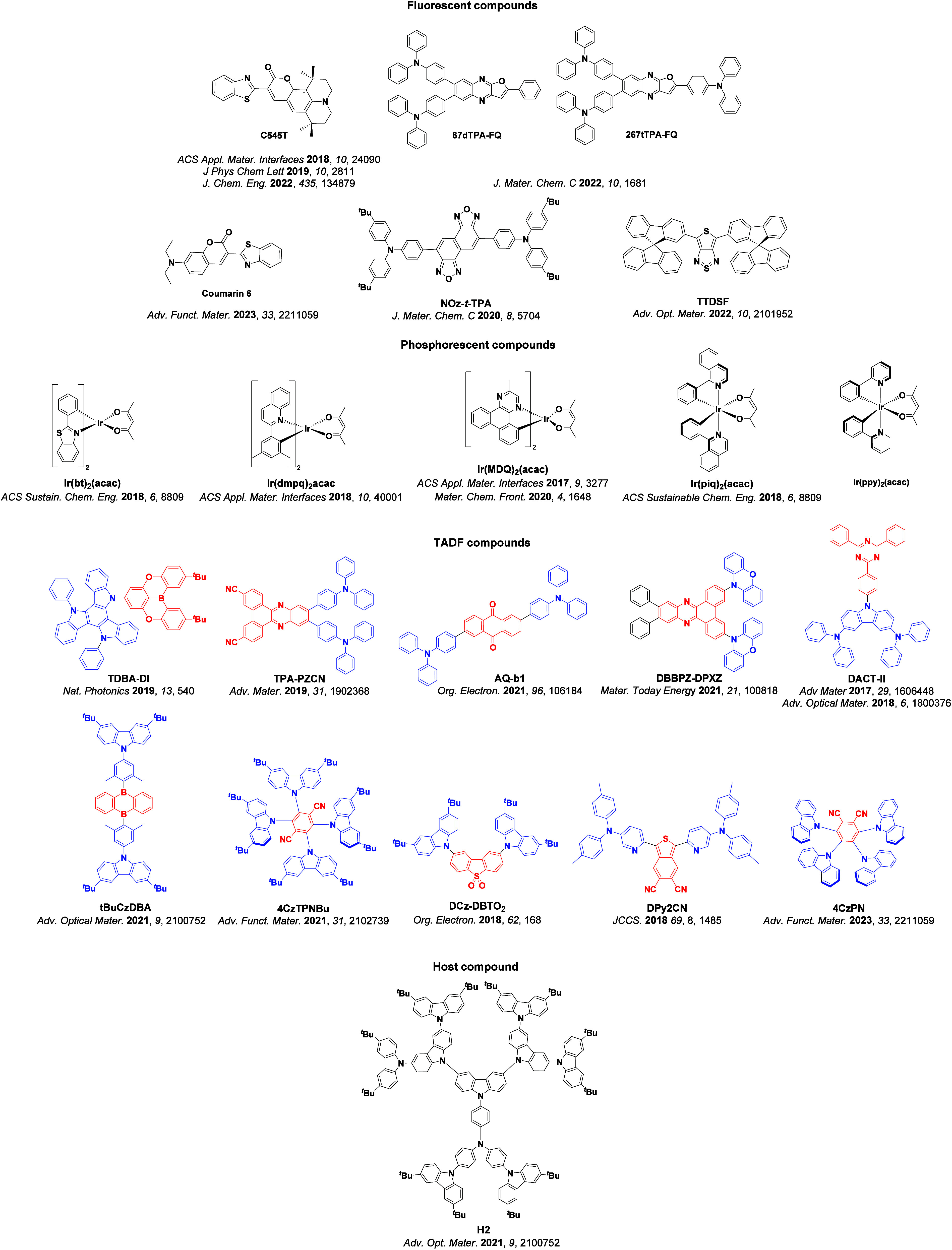
Luminescent, phosphorescent, TADF, and host materials used as dopants in either exciplex hosts or solution-processed TADF exciplex systems. The blue color signifies donor moieties, while the red color signifies acceptor moieties for TADF compounds.

Examples of white, blue, and green TADF OLEDs using exciplex hosts have been demonstrated with high efficiencies.^673^ For example, Chen *et al*. used different doping concentrations of the red TADF emitter **DBBPZ-DPXZ** (Figure 89) in a blue emissive exciplex matrix (**CDBP:​PO-T2T**) to achieve red and white emission. Using 0.2 wt% of red **DBBPZ-DPXZ**, a warm white OLED with CIE coordinates of (0.40, 0.38) and EQE_max_ of 20.7% was obtained. When the concentration of **DBBPZ-DPXZ** was increased to 6 wt% the energy transfer from host to dopant was more complete, resulting in a red OLED emitting at λ_EL_ of 628 nm and showing an EQE_max_ of 20.8%.^715^ Similarly, Moon *et al*. reported green OLEDs using **TCTA:​B3PyMPM** as the exciplex host and **DACT-II** (Figure 89) as the TADF dopant. The blend emits at λ_PL_ ∼ 525 nm and has a near unity Φ_PL_ of 96%. The devices showed an EQE_max_ of 34.2%, CE_max_ of 114 cd A^–1^ and a PE_max_ of 121.3 lm W^–1^.^716^

The electron transport materials **B4PyMPM** and **B3PyMPM** (Figure 84) are often employed as acceptors in TADF exciplex OLEDs. Sasabe *et al*. reported several interfacial devices using **B3PyMPM**, **B4PyMPM**, and **B4PyPPM** as the electron transport layer and **CBP** or **TCTA** as donors. **DACT-II** was employed as the emitter, doped at concentrations ranging from 4 to 20 wt% in the EML. The 20 wt% **DACT-II:​CBP/B4PyPPM** device with structure ITO (100 nm) / HAT-CN6 (1 nm) / TAPC (65 nm) / TCTA (5 nm) / 20 wt% DACT-II-doped CBP (10 nm) / **B4PyPPM** (50 nm) / Liq (1 nm) / Al (80 nm)] emitted at 534 nm [CIE coordinates (0.38, 0.58)], and showed an EQE_max_ of 26.8% with PE_max_ of 122.2 lm W^–1^. However, the highest efficiency device was achieved with 9 wt% DACT-II-doped into the exciplex blend **CBP:​B4PyMPM** as the EML. The device was fabricated with structure ITO (100 nm) / HAT-CN6 (1 nm) / TAPC (65 nm) / TCTA (5 nm) / 9 wt% DACT-II-doped CBP:​B4PyMPM (10nm) / B4PyPPM (50 nm) / Liq (1 nm) / Al (80 nm), emitting at λ_EL_ of 534 nm [CIE coordinates (0.37, 0.58)] and showing an EQE_max_ of 29.2% with PE_max_ of 133.2 lm W^–1^.^717^

A series of phenylcarbazole-based donors were used in conjunction with the acceptor **B3PyMPM** to form exciplex hosts for the phosphorescent green emitter **Ir(ppy)_2_(acac)** (Figure 89). The devices with the highest efficiencies (Table S8) were obtained using **PhCNCzm-MeCzPh** (Figure 88) as the exciplex donor, which adopts a twisted conformation with a relatively high triplet energy amongst the donors studied. The blend **PhCNCzm-MeCzPh:​B3PyMPM** has Δ*E*_ST_ of 0.32 eV and yet a very short τ_d_ of 0.15 μs. The devices showed an EQE_max_ of 31.5%, CE_max_ of 113.06 cd A^–1^, and PE_max_ of 99.41 lm W^–1^ at CIE coordinates of (0.32, 0.64).^718^

Jia *et al*. fabricated a blue-emissive TADF exciplex using a 7:3 wt% ratio of **m-MTDATA:​TmPyPB** (Figure 88), which emits at λ_PL_ of 479 nm, has a modest Φ_PL_ of 12.3%, and a small Δ*E*_ST_ of 0.04 eV. According to the authors the suitably high triplet energy level of this exciplex made it a good candidate as a host for green, yellow, and red phosphorescent emitters (Table S8). With the yellow emitter **Ir(bt)_2_(acac)** (Figure 89), the OLED showed EQE_max_, CE_max_, and PE_max_, of 18.5%, 50.7 cd A^–1^, and 57.6 m W^–1^ respectively, at CIE coordinates of (0.51, 0.49). The green-emitting device with 2 wt% of ***fac*-Ir(ppy)_3_** showed an EQE_max_ of 10.0% at CIE coordinates at (0.32, 0.61). When using the red phosphorescent complex **Ir(piq)_2_(acac)** (Figure 89) the OLED showed an EQE_max_ of 10.0% at CIE (0.67, 0.33).^719^

Shih *et al*. reported a device with the exciplex **BCzPh:​3P-T2T** (2:1), (Figure 88). which emits at λ_PL_ of 536 nm and has a Φ_PL_ of 68%. The exciplex devices showed an EQE_max_ of 13.5%, and this exciplex was also used as a host for both fluorescent **C545T** (Figure 89) and phosphorescent **Ir(ppy)_2_(acac)** green emitters. The exciplex film with 1 wt% of **C545T** emits at λ_PL_ of 516 nm and has a Φ_PL_ of 97%, with the device achieving an EQE_max_ of 15.5%. The blend with 8 wt% of **Ir(ppy)_2_(acac)** emits at λ_PL_ of 523 nm and has Φ_PL_ of 85%, with that device achieving an EQE_max_ of 29.7%.^720^ This work demonstrates how the exciton harvesting capacity of the dopant can contribute significantly the efficiency, e.g. in the phosphorescent device. Furthermore, the very high EQE_max_ was also suggestive of some preferential horizontal orientation of the TDM of the dopant in the exciplex host. Liang *et al*. later reported an even higher efficiency device using **C545T** as a dopant in an exciplex blend host, consisting of **TAPC** as the donor and a bespoke acceptor containing benzimidazole and triazine units (**PIM-TRZ**, Figure 88). The OLED with 0.6 wt% **C545T** in **TAPC:​PIM-TRZ** showed an EQE_max_ of 20.2%, CE_max_ of 68.3 cd A^–1^, and PE_max_ of 86.4 lm W^–1^ at CIE coordinates of (0.29, 0.62). In comparison, the non-doped exciplex **TAPC:​PIM-TRZ** device showed an EQE_max_ of 21.7%, CE_max_ of 71.2 cd A^–1^, and PEmax of 97.3 lm W^–1^ at CIE coordinates of (0.35, 0.58).^721^

Colella *et al*. studied the energy-transfer from **26DCzPPy/PO-T2T** interfacial exciplex (Figure 88) to phosphorescent guest **Ir(dmpq)_2_acac** (Figure 89), included in different ratios varying from 1 to 10 wt%. According to the study, both DET and FRET are operational between the exciplex and the dopant, with the former being the dominant energy transfer mechanism. The devices of **26DCzPPy:​4 wt% Ir(dmpq)_2_aca/PO-T2T** showed the highest efficiency with an EQE_max_ of 28.6%, and emitting at λ_EL_ of 630 nm.^722^

Tian *et al*. reported an exciplex with **DEX** (Figure 88), a bulky triphenyl amine donor of similar structure to HMAT (hexamethylazatriangulene, see Figure 246 in Section 21), with **PO-T2T** acceptor (Table S8). The device with **DEX:​PO-T2T** emitted at λ_EL_ of 520 nm, and showed an EQE_max_ of 11.2%, CE_max_ of 36.0 cd A^–1^, and PE_max_ of 44.6 lm W^–1^. The PhOLED using **DEX:​PO-T2T** as a host with 5 wt% **Ir(MDQ)_2_(acac)** (Figure 89) as the dopant emitted at λ_EL_ of around 600 nm, and showed an EQE_max_ of 21.7%. A co-host system was formed once an extra layer of **PO-T2T** (15 nm) was introduced [device structure [ITO / HAT-CN6 (10 nm) / TAPC (30 nm) / DEX (10 nm) / **DEX:​​PO-T2T**:5 wt % **Ir(MDQ)2(acac)** (5 wt %, 20 nm) / **PO-T2T** (15 nm) / **Bphen**:0.1 wt% LiH / Al (120 nm)], leading to devices having the same λ_EL_ as above but showing improved EQE_max_ of 24.5%, CE_max_ of 36.0 cd A^–1^, and PE_max_ of 146.1 lm W^–1^.^723^

Duan *et al*. reported an exciplex matrix fabricated using the hole-transporting molecule **CDBP** and varying the choice of phosphine-oxide-based acceptors (**mDBSOSPO**, m = 2, 3, and 4, Figure 88). The exciplex blend doped with the yellow TADF emitter **4CzTPNBu** (Figure 89) emits at λ_PL_ of 570 nm, has a near unity Φ_PL_ of 97%, a small Δ*E*_ST_ of 0.02 eV, and a τ_d_ of 6.8 μs; the exciplex by itself emits at λ_PL_ of 471 nm, has a much lower Φ_PL_ of only 26%, and a τ_d_ of 4.3 μs. A family of devices with different exciplex blends similarly doped with 3 wt% of **4CzTPNBu** were fabricated (Table S7). Of these, the device with the highest efficiency consisted of **CDBP:​2DBSOSPO:​4CzTPNBu**, showing an EQE_max_ of 30.3%, a PE_max_ of 114.9 lm W^–1^, and emitting at CIE coordinates of (0.48, 0.49). By contrast, the device without the TADF dopant (**CDBP:​2DBSOSPO**) showed an EQE_max_ of only 0.82% at CIE coordinates of (0.17, 0.23).^724^

Zhou *et al*. investigated the changes in device performance of three OLEDs containing different green-emitting dopants with the same interfacial exciplex host **CDBP/B4PyPPM**. Devices with 5 wt% of the fluorescent material **Coumarin 6** (Figure 89), the phosphorescent complex **Ir(ppy)_2_acac**, or the TADF emitter **4CzIPN** (Figure 89) were fabricated. The highest efficiency device employed the TADF dopant and showed an EQE_max_ of 20% at λ_EL_ of 536 nm, supported by a near unity Φ_PL_ of 98.9% of the dopant in this matrix as well as near 100% exciton utilization efficiency. Devices with the fluorescent or phosphorescent dopants showed EQE_max_ of just 4.0 and 7.9%, respectively. The low efficiency of the PhOLED is surprising considering that the Φ_PL_ of the dopant is 93.0%. The enhanced performance of the device with **4CzIPN** was attributed by the authors to the large electric dipole of this dopant molecule which assisted FRET, and to the short exciton lifetimes of **4CzPN** which mitigated the build-up of triplet excitons and associated losses at a high current density.^725^

Wang *et al*. reported fluorescent molecules **67dTPA-FQ** and **267TTPA-FQ** (Figure 89), emitting at λ_PL_ of 532 and 526 nm with Φ_PL_ of 91 and 100% (in toluene), and having T_1_ levels of 2.19 and 2.32 eV all respectively. These compounds were then used as dopants (1 wt%) in the bulk exciplex system **TCTA:​PO-T2T** (8:2 wt% ratio); the blend itself emits at λ_PL_ of 538 nm and has a T_1_ level of 2.35 eV. Devices with just **TCTA:​PO-T2T** emitted at λ_EL_ of 556 nm and showed EQE_max_ of 7.4%, while the devices with **67dTPA-FQ** in **TCTA:​PO-T2T** performed similarly, emitting at λ_EL_ of 552 nm and showing EQE_max_ of 8.4%. However, the device with **267TTPA-FQ** in **TCTA:​PO-T2T** emitted at λ_EL_ of 524 nm and showed a modest improvement in EQE_max_ to 9.6%. The improvement in the performance of the latter device was attributed partially to Förster energy transfer between the dopant and exciplex host, which was improved in the system **(TCTA:​PO-T2T):​267TTPA-FQ**.^726^

### 8.6 Solution-Processed TADF Exciplexes

While the vast majority of reported exciplex OLEDs use thermal evaporation for the control over film composition and morphology that this method offers, solution-processing of exciplex emitters and hosts is also growing in prominence and necessary for molecules above a certain molecular weight. Chen *et al*. showed that small variations in the structure of isomeric acceptors significantly affected the energies of the CT excited states and device efficiencies using solution-processed interfacial exciplex host systems.^727^ Oligocarbazole **H2** (Figure 89), was doped with TADF emitted ***t*BuCzDBA**, and used as donor in an interfacial exciplex with **B3PyMPM** or **B4PyMPM** as the acceptors (Table S7). The highest efficiency OLED consisted of **H2:​*t*BuCzDBA** (10 wt%)**/B3PyMPM**, which emitted at λ_EL_ ∼ 550 nm [CIE coordinates of (0.42, 0.55)], with an EQE_max_ of 26.4% and a PE_max_ of 95.0 lm W^–1^. When the acceptor was switched to the isomeric **B4PyMPM** the emission wavelength did not change, but the performance of the devices decreased to an EQE_max_ of 20.0% and PE_max_ of 69.9 lm W^–1^. The lower efficiency of the latter device was attributed to the poorer hole-electron recombination ratio in **H2:​*t*BuCzDBA/B4PyMPM**.^727^

Xu *et al*. employed the red TADF emitter **AQ-b1** (Figure 89) as a dopant in a series of binary (1:1) and ternary (1:1:1) exciplex systems. **mCP** and **OXD-7** (Figure 88) were used as the respective exciplex donor and acceptor, while two molecules showing high electron mobility and containing spirofluorene and s-triazine moieties (**TDP-TRZ** and **DTDP-TRZ**, Figure 88) were used as additional acceptors in the ternary blends. Solution-processed OLEDs with the binary (**mCP:​DTDP-TRZ**) and ternary exciplex systems (**mCP:​OXD-7:​DTDP-TRZ**) doped with 10 wt% **AQ-b1** showed EQE_max_ of 2.5 and 1.6% at CIE coordinates at (0.59, 0.39) and (0.60, 0.39), respectively. According to the study, multiple exciplex pairs in the ternary co-host contributed to improving the exciton harvesting efficiency and also provided balanced injection of charge carriers.^728^

Colella *et al*. demonstrated that solution-processable TADF exciplex OLEDs can show similar efficiencies to vacuum-deposited devices, using a bulk exciplex consisting of **TAPC** as the donor and the D-A-D TADF molecule **DCz-DBTO2** as the acceptor (Figure 89) (70:30 wt% ratio). The authors used different solvents and spin-coating parameters to vary the thickness of the emissive exciplex layer (Table S7). The optimized device was fabricated using a 5:95 vol% solvent blend of chloro­ben­zene:​chloro­form, which produced an emissive layer thickness of 60 nm. The solution-processed device emitted at λ_EL_ of 550 nm, and showed EQE_max_ of 8.9%, a CE_max_ of 27.5 cd A^–1^, and a PE_max_ of 15 lm W^–1^.^729^ The vacuum-deposited device was previously published by Jankus *et al*.^730^, emitting at λ_pL_ of 540 nm and showing comparable EQE of 10.3%, CE_max_ of 32.3 cd/A, and PE_max_ of 26.7 lm W^–1^.

Kesavan *et al*. fabricated a solution-processed exciplex OLED that showed an EQE_max_ of 20% and CE_max_ of 41 cd A^–1^ at CIE coordinates of (0.29, 0.52). At the time of publication this was the highest-performing solution-processed exciplex OLED without the use of an additional emissive dopant. This exciplex consisted of carbazole-based donor **BCC-36** (Figure 88) with **PO-T2T** in a 5:1 ratio, which emits at λ_PL_ of 490 nm, has a Φ_PL_ of 90%, a Δ*E*_ST_ of 0.04 eV, and a τ_d_ of 1.1 μs. This exciplex was also used as a host for fluorescent (**C545T**), phosphorescent (**Ir(ppy)_2_(acac)**), and TADF (**4CzIPN**) compounds (Table S7). Devices using 1 wt% **C545T** showed EQE_max_ of 12.5% [CIE coordinates at (0.24, 0.57)], while devices with 7.5 wt% of **4CzIPN** showed EQE_max_ of 26.5% [CIE coordinates at (0.26, 0.56)]. The devices doped with 12.5 wt% **Ir(ppy)_2_(acac)** showed the highest EQE_max_ of 32.5% [CIE coordinates at (0.31, 0.64)]. According to the authors, the strong spin-orbital coupling associated with the heavy metal in the phosphorescent material leads to an increased rate of ISC, increasing the energy transfer process from the host to the emitter, which contributes to highest device efficiency obtained for the device doped with the phosphorescent compound.^731^

### 8.7 Fundamental Studies of TADF Exciplex Systems

As well as pursuing the highest performing devices, many studies have focused on exploring the fundamental mechanisms and decay pathways in TADF exciplex systems. For example, Huang *et al*. used transient photoluminescence and electroluminescence measurements to study the exciton dynamics in a 1:1 wt% blend of **m-MTDATA:​3TPYMB**. According to the authors, exciplex excitons can stretch while remaining bound, and the recombination rate is determined by a local process involving the lateral motion of carriers that is related to the electron-hole separation.^732^ A similar work published by Lin *et al*. measured steady-state and time-resolved IR spectroscopy and grazing incident X-ray diffraction (GIWAX) to gain in-depth insight into the structure and emission mechanisms associated with the TADF exciplex **CN-Cz2:​PO-T2T** (Figure 90 and Table S9). The devices using a 1:1 ratio showed the highest EQE_max_ of 16%, CE_max_ of 37.8 cd A^–1^, and PE_max_ of 47.5 lm W^–1^ at coordinates of CIE (0.20,0.40). The study reported the formation of polaron pairs in the exciplex blend, which could recombine to give charge-transfer emission or dissociate back to polarons. When dissociation occurs, positive and negative polarons would be created and their recombination for light generation would be prohibited, leading to losses.^733^

**Figure 90:**
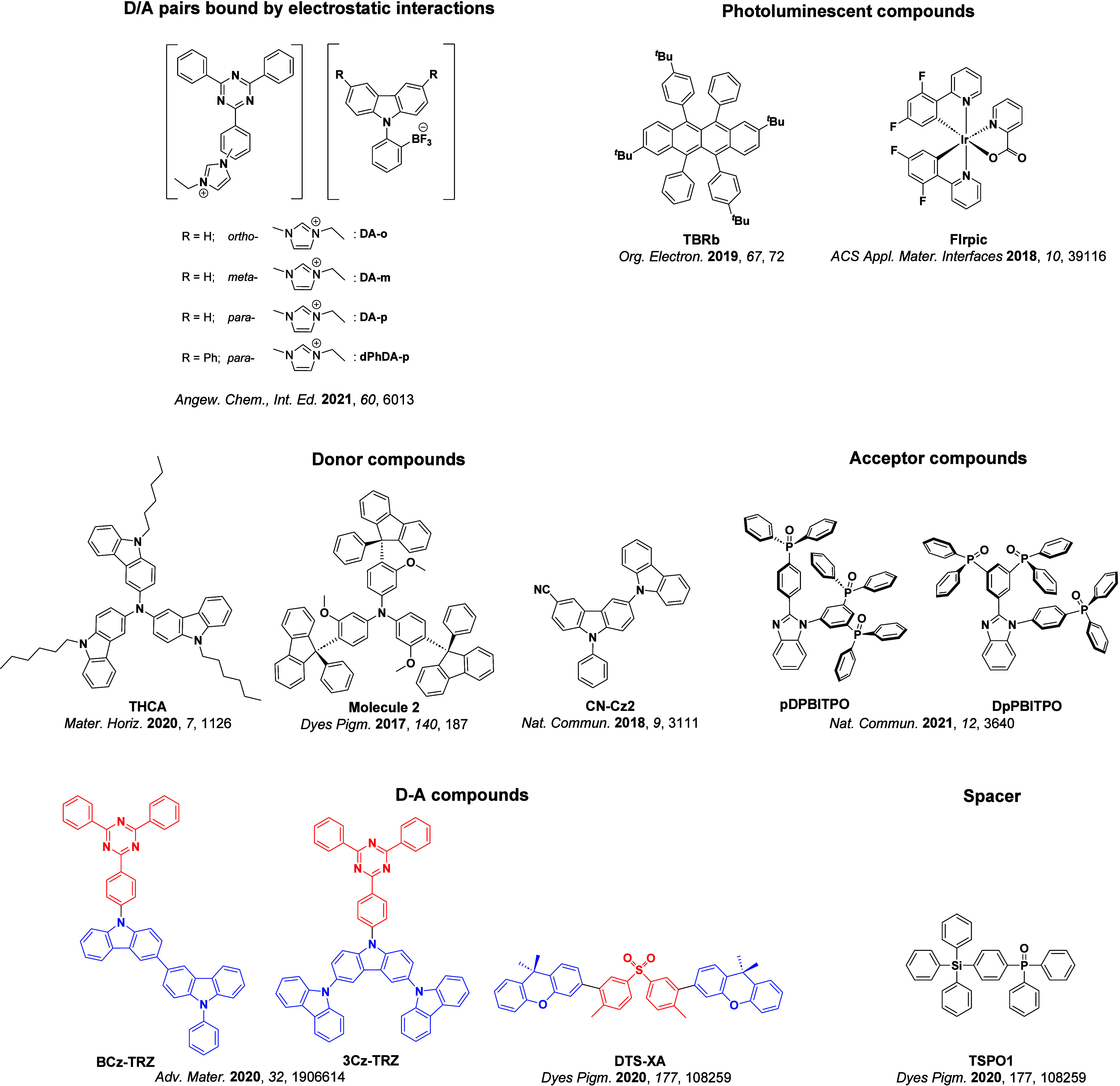
Molecular structures of D/A compounds bound by electrostatic interactions, photoluminescent compounds, donor and acceptor compounds, D-A type compounds and spacers used in WOLEDs or applied toward fundamental studies of TADF exciplex systems (the blue color signifies donor moieties, while the red color signifies acceptor moieties).

A kinetic model proposed by Grüne *et al*. is particularly suited for exciplexes and was applied to explain the photophysics of **m-MTDATA:​3TPYMB**. The model accounted for the fact that triplet-triplet annihilation (TTA) is the main second-order effect, which contributes significantly to triplet depopulation. As the efficiency of the TTA is strongly influenced by temperature, this led to a constraint of the overall efficiency of the device at room temperature.^100^

Moon *et al*. explored how the formation and photophysical properties of the **TCTA:​B4PyMPM** CT state are influenced by the distance between the D and A molecules, their relative orientation, and the D:A ratio.^734^ According to the study, the exciplex emission wavelength is determined by the configuration of the molecules in the system, which also strongly affects Δ*E*_ST_, kinetic rate constants, and emission dipole orientations. Short distances between donor and acceptor molecules results in lower exciplex energy due to the Coulomb interaction, which is proportional to r^–1^.

Bunzmann *et al*. used electron paramagnetic resonance (EPR) to study the involvement of different spin states in the RISC of TADF exciplex systems. Both electroluminescence and photoluminescence detected magnetic resonance (ELDMR, PLDMR) were used to probe the photophysics of three exciplex system: **m-MTDATA:​3TPYMB**, **m-MTDATA:​Bphen**, and **THCA:​BPhen** (Figure 90, Table S9), which emit at λ_PL_ of 545, 560, and 570 nm, respectively. Of these three the exciplex **m-MTDATA:​3TPYMB** has the highest Φ_PL_ of 45%, and the devices with this exciplex emitted at λ_EL_ of 550 nm and showed EQE_max_ of 11.0%. However, the OLED performance was not the focus of this study, but rather it was whether the investigation of the activation energy for delayed fluorescence correlated with either the Δ*E*_ST_ of the exciplex system or with the molecular triplet states of the donor or acceptor materials. The authors found that in all three systems exciplex states formed at the interface of donor and acceptor molecules in the blend led to TADF emission and that molecular (local) triplet exciton only formed under optical and not electrical excitation.^735^

As with molecular TADF materials, most of the exciplex TADF studies published to date have not focused on device stability despite its importance for commercial applications. However, a few studies do exist that correlate device stability with the properties of the exciplex. For example, Nguyen *et al*. aimed to optimize the stability of exciplex OLEDs by varying the nature of the triazine-based acceptor with **Tris-PCz** as the donor. The acceptors were divided into three classes: molecules without a significant electron-donating group, D-A compounds that are not TADF, and D-A TADF compounds. The most stable devices featured exciplexes where the acceptor partner is itself TADF, and OLEDs with **Tris-PCz:​BCz-TRZ** and **Tris-PCz:​3Cz-TRZ** (Figure 90) showed the longest device lifetimes (LT_50_ of 292 and 337 hours, respectively), ascribed to multichannel RISC processes. Both devices showed green electroluminescence at CIE coordinates at (0.26, 0.50) and (0.26, 0.53) respectively, however the device with **Tris-PCz:​BCz-TRZ** showed the highest EQE_max_ of 11.9%, compared to 8.9% for the device with **Tris-PCz:​3Cz-TRZ.** ^736^

A major challenge in exciplex design is the control of distance between donor and acceptor molecules. He *et al*. demonstrated a unique strategy that exploits electrostatic interactions (Coulombic attraction) rather than the circumstances of deposition to control these D-A distances. The exciplexes consisted of carbazole-based anionic donors ([**CAZ-o-BF_3_^–^]** or **dPhCAZ-o-BF_3_^–^]**) and 2,4,6-tri­phen­yl-1,3,5-tria­zine-based cationic acceptors (**[TRZ-o-ImEt^+^]**, **[TRZ-m-Im-Et^+^]** or **[TRZ-p-ImEt^+^]**), with the blends named **DA-o**, **DA-m**, **DA-p**, and **dPhDA-p** (Figure 90). The films of these D/A pairs in doped at 1 wt% in PMMA films emit at λ_PL_ of 498, 481, 484, and 504 nm and have ΔE_ST_ of 0.02, 0.10, 0.18, and 0.20 eV, respectively, leading to τ_d_ in the range of 3.1–7.8 μs and associated *k*_RISC_ in the range of 1.8–5.2 × 10^5^ s^–1^. The authors documented that the distance and interaction between the ionic donor and the acceptor could be modified as a function of the position of the acceptor imidazolium moiety. This also tunes the overlap of the frontier orbitals and thus the radiative decay rate of the exciplex singlet, reflected in the differing τ_p_/τ_d_ of 165 ns/3.1 ms, 114 ns/3.7 ms, 186 ns/6.2 ms, and 185 ns/7.8 ms for the blends with **DA-o**, **DA-m**, **DA-p** and **dPhDA-p**, respectively. The *k*_RISC_ was found to decrease from **DA-o** (5.2 × 10^5^ s^–1^) and **DA-m** (4.2 × 10^5^ s^–1^), to **DA-p** (2.2 × 10^5^ s^–1^) and to **dPhDA-p** (1.8 × 10^5^ s^–1^), which follows the trend in their Δ*E*_ST_. The so-called isolated exciplexes exhibited a considerably higher Φ_PL_ (24–52%) in PMMA film than neat exciplex blends (8–11%). As **dPhDA-p** has the highest Φ_PL_ of 52% it was then evaluated as the emitter in a solution-processed OLED, which emitted at λ_EL_ of 510 nm [CIE coordinates of (0.25, 0.44)] and showed an EQE_max_ of 6.1%.^737^

### 8.8 White TADF Exciplex OLEDs

Fabricating white organic light emitting diodes (WOLEDs) with high CRI, high efficiency, low operating voltage, and low efficiency roll-off is not a trivial task (Section 6). Cekaviciute *et al*. demonstrated a new approach to fabricate a WOLED using multiple exciplexes. In this study a blue-emitting exciplex layer made of 3:7 **Molecule 2**:​**BPhen** (Figure 90) was sandwiched between two layers of the green exciplex **m-MTDATA:​BPhen**. The maximum values of EQE, CE, and PE were as high as 2.55%, 6.34 cd A^–1^, and 4.09 lm W^–1^, respectively.^738^ Another study by Tian *et al*. reported a multi-layer device using a bulk exciplex system composed of bipolar donor **26DCzPPy** and acceptor **B4PyMPM** acting together as the host for **FIrpic** (Figure 90) in sky-blue phosphorescent and white OLEDs. The blue OLED showed λ_EL_ of 472 nm [CIE coordinates of (0.17, 0.36)] and a PE_max_ of 48 lm W^–1^. The white device contained an extra layer of phosphorescent orange emitter **PO-01** doped in **26DCzPPy**, having the structure **26DCzPPy:​PO-01/26DCzPPY:​B4PyMPM:​** 15wt%**FIrpic**, and emitting at CIE coordinates of (0.45, 0.48) with EQE_100_ of 27.3% and corresponding CE of 79.0 cd A^–1^ and PE of 89.0 lm W^–1^.^739^

Yao *et al*. documented a different design for fabricating high efficiency WOLEDs, where a blue emitting exciplex is used as a host for a yellow fluorescent compound. An additional hole transport layer is also inserted on top of the emissive layer, which is essential to improve the overall efficiency and efficiency roll-off at high luminance. The additional interfacial exciplex established in the EML regulates exciton distribution and enhances the energy transfer to fluorescent guest. The specific device contained blue emitter exciplex host **26DCzPPy:​PO-T2T** with fluorescent yellow dopant **TBRb** (Figure 90), and a thin interlayer of **TCTA**. This device showed an EQE_max_ of 10.1% at CIE coordinates of (0.36,0.53), with CE_max_ of 32.6 cd A^–1^ and PE_max_ of 35.9, lm W^–1^ (Table S10).^740^

Guo *et al*. demonstrated TADF exciplex WOLEDs by sandwiching a yellow exciplex layer between two blue exciplex layers. The emission spectra and device performance could then be tuned by changing the mass ratio of the intermediate yellow exciplex layer, and/or thickness of the two blue exciplex layers. The optimized device used **mCP:​PO-T2T** (1:1, 4 nm)/**PO-T2T:​TPD** (3:1, 3 nm)/**Bphen:​TPD** (1:1, 4 nm), and showed EQE_max_ of 5.21%, CE_max_ of 12.78 cd A^–1^, and PE_max_ of 12.12 lm W^–1^ at CIE coordinates of (0.245, 0.320).^741^

Tan *et al*. fabricated exciplex WOLEDs by layering separate blue and orange TADF interface exciplexes. A newly designed donor composed of a 4,4′-sul­fon­yl­bis­(meth­yl­ben­zene) central electron acceptor moiety and two peripheral 9,9-di­meth­yl-9*H*-xan­thene groups (**DTS-XA**, Figure 90) was combined with the donor compounds **TCTA** and **m-MTDATA** to form the interfacial exciplex systems **DTS-XA/TCTA** and **DTS-XA/m-MTDATA**. The devices based on **DTS-XA/TCTA** and **DTS-XA/m-MTDATA** emitted in the blue (λ_EL_ = 433 nm) and green-yellow (λ_EL_ = 524 nm) regions, showing EQE_max_ of 9.1 and 8.3%, respectively. WOLEDs were then fabricated by layering the two exciplexes using the following configuration: **(DTS-XA/TCTA)**/spacer/(**DTS-XA/m-MTDATA**), where the spacer consisted of a thin layer of diphenyl-4-triphenylsilylphenyl-phosphineoxide (TSPO1, Figure 90) acting as a hole and electron-transporting modulator. The highest-efficiency WOLED showed an EQE_max_ of 10.6% at CIE coordinates of (0.29, 0.37).^742^

Han *et al*. reported an exciplex WOLED composed of a single emissive layer featuring two phosphine oxide-based acceptors (**pDPBITPO** and **DpPBITPO**, Figure 90). The large triplet energy gap (0.6 eV) between the **mCP** donor and these acceptors limited donor-acceptor triplet coupling, which in turn led to dual triplet levels accessible in the exciplex blend. The authors confirmed by transient emission spectroscopy that cascade triplet energy transfer takes place from the high-lying triplet level of the exciplex to the blue emitter, then to the low-lying triplet level of the acceptor, and finally to the yellow emitter. This arrangement and energy transfer between excited states led to 100% exciton harvesting, and, hence, the single-emissive layer design based on **mCP**:​**pDPBITPO** and **mCP**:​**DpPBITPO** produced TADF WOLEDs with a tantalizing EQE_max_ of 32.7%, PE_max_ of 108.2 lm W^–1^, and CIE coordinates of (0.31, 0.35).^743^

### 8.9 Outlook

Exciplexes are intermolecular assemblies that frequently show TADF due to the intrinsic separation of HOMO and LUMO on separate molecules. The optoelectronic properties of these blends can also be straightforwardly manipulated through the choice of specific donor and acceptor materials. However, the very weak ‘through-space’ electronic coupling of chromophores in exciplexes tends to generate low Φ_PL_, which typically limits their intrinsic performance as emissive materials and hence also affected the relatively limited degree of attention this class of material has historically received from the research community.

Nonetheless, the performance of exciplex OLEDs has been more extensively studied in recent years and the overall stability and efficiency of these devices have progressively improved, with some now achieving performance metrics comparable to those of D-A TADF OLEDs (Figure 91). For example, the most efficient exciplex OLEDs reported within the scope of this review include one with **mCP:​PO-T2T** as the emitter that showed an EQE_max_ of 16% at CIE coordinates of (0.16, 0.28).^186^ One of the most efficient green devices employed an exciplex host, showing an EQE_max_ of 34.2% using **TCTA:​B3PyMPM** as the host and **DACT-II** as the TADF dopant.^716^ Red OLEDs using exciplex hosts showed EQE_max_ as high as 28.1% at CIE coordinates of (0.66, 0.34) using **CBP:​PO-T2T** host and **TPA-PZCN** as the TADF emitter.^507^ An efficient WOLED with an EQE_max_ of 32.7% at CIE coordinates of (0.44, 0.47) was reported using the exciplex system **mCP:​DpPBITPO** as the host, with **DMAC-DPS** as an assistant dopant and **4CzTPNBu** as the terminal emitter in a single-emissive-layer device.^743^ The high density of suitable recombination sites in exciplex emissive layers (analogous to high loading of TADF guests in conventional hosts) can also contribute to improved efficiency roll-off.^740^

**Figure 91:**
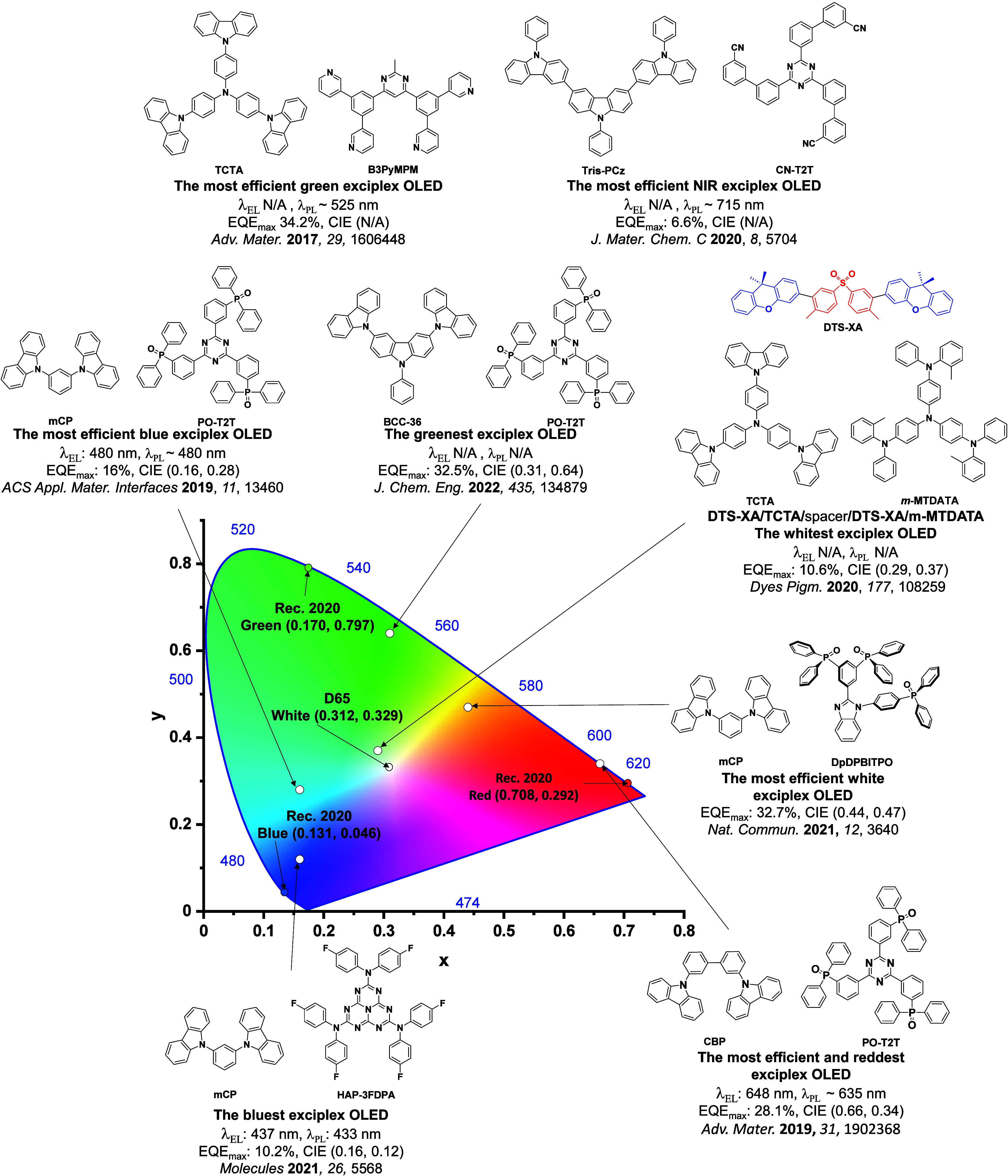
CIE color coordinates of high-performance TADF exciplex devices. The white circles illustrate the spread of the emission color of the device. Selected devices and their associated CIE coordinates are highlighted, illustrating the structures of the emitters of the “bluest”, “greenest”, “reddest”, and “whitest” devices and the structures of the emitters used in the devices showing the highest efficiency blue, green, red, NIR, and white emission. The most efficient devices are quantified by the EQE_max_ at λ_EL_ < 490 nm for blue, λ_EL_ = 490–580 nm for green, λ_EL_ > 580 nm for red, and CIE coordinates close to (0.33, 0.33) for white. The device with the CIE coordinates closest to the Rec. 2020 defined coordinates for blue, (0.131, 0.046), is defined as the “bluest”. The device with the CIE coordinates closest to the Rec. 2020 defined coordinates for green, (0.170, 0.797), is defined as the “greenest”. The device with the CIE coordinates closest to the Rec. 2020 defined coordinates for red, (0.708, 0.292), is defined as the “reddest”. The device with the CIE coordinates closest to the Rec. 2020 defined coordinates for white, (0.33, 0.33), is defined as the “whitest”. In the chemical structures, the blue color signifies donor moieties, while the red color signifies acceptor moieties.

Despite this progress, we believe that applications of TADF exciplexes in OLEDs still have significant unrealised potential. As with D-A TADF molecules, color purity in exciplex OLEDs is frequently undermined by broad emission arising from the long-range charge-transfer character of the emissive excited state. Nonradiative decay processes intrinsic to intermolecular contact interfaces can also negatively impact device performance, particularly for red OLEDs. Most notably, the development of efficient deep-blue and blue exciplex OLEDs remains elusive, largely because of the challenge in designing (or discovering) donor and acceptor molecules with appropriate HOMO, LUMO, and T_1_ energy levels. Even with the use of donor/acceptor materials that can themselves harvest triplet excitons either by TADF or phosphorescence, many studies only employ conventional hole or electron transport materials as exciplex components, with this limited range of chemical space explored likely restricting recorded performance compared to more innovative D-A TADF, TSCT TADF, or MR-TADF emitter designs. A breakthrough specifically in blue emissive materials would be particularly valuable, allowing the use of TADF-active exciplexes as hosts for many other emissive species to generate narrowband blue or white emission (examples throughout Sections 6, **11**, **17**, **18**), while providing the excellent charge transporting properties of the individual exciplex components.

Unique amongst other TADF materials, fabrication methods critically control the performance of exciplex OLEDs. The choice of bulk heterojunction or bilayer deposition influences the degree of interaction of the donor and acceptor molecules and thus the emission color and performance of the exciplex. Exploiting this feature, controlling the distance and/or orientation between donor and acceptor with a spacer layer^689^ or diluting material^698^ influences the potential energy surfaces of the exited states, and can improve the EQE_max_.^695,699^ Controlling the exciplex state using covalently bonded scaffolds now forms the basis of related TSCT emitters (Section 12). We note that controlled self-assembly (Section 19) of the donor and acceptor units to form the exciplex may become a powerful tool to achieve finer control of this in future, with currently only a few reports of self-assembled exciplexes.^708,744–746^

These examples therefore highlight both the promise and current limitations of exciplexes as both hosts and emitters. While their often low Φ_PL_ represents a major drawback as emitters in their own right, their balanced charge transport properties and ability to harvest both singlet and triplet excitons make them significantly more appealing than conventional ‘inactive’ OLED hosts. Indeed, we speculate that future uses of TADF exciplexes will increasingly focus on their use as hosts for other emissive materials, exploiting their ambipolar charge transporting properties while also largely circumventing their low Φ_PL_ and broad emission.

## 9 Metal-Based TADF Emitters

### 9.1 Introduction

The majority of the sections of this review have focused on organic TADF molecules, reflecting their key advantage in their ability to harvest triplet excitons without the need for scarce and expensive heavy metals central to both the structure and function of organometallic phosphors. However, TADF emission is observed in a range of metal complexes as well, including those based on Earth-abundant metals. Indeed, the majority of the reported examples of TADF complexes are copper(I) complexes, although there are also numerous examples of silver(I), gold(I and III), palladium(II), and zinc(II) complexes. Examples based on each of these metals will be discussed in detail in this section. There are also examples of TADF emission emanating from complexes of abundant alkali metals, d^0^ transition metals, d^10^ transition metals, and main group compounds, which are also briefly discussed. An overview of the metals that have been incorporated into TADF compounds is shown in Figure 92. Like organic TADF emitters, organometallic TADF compounds have found wide applications in OLEDs, LEECs, and as photocatalysts. While this section focuses on metal-containing TADF emitters used in OLEDs, their use in LEECs and photocatalysis are covered in Sections 16 and 23.

**Figure 92:**
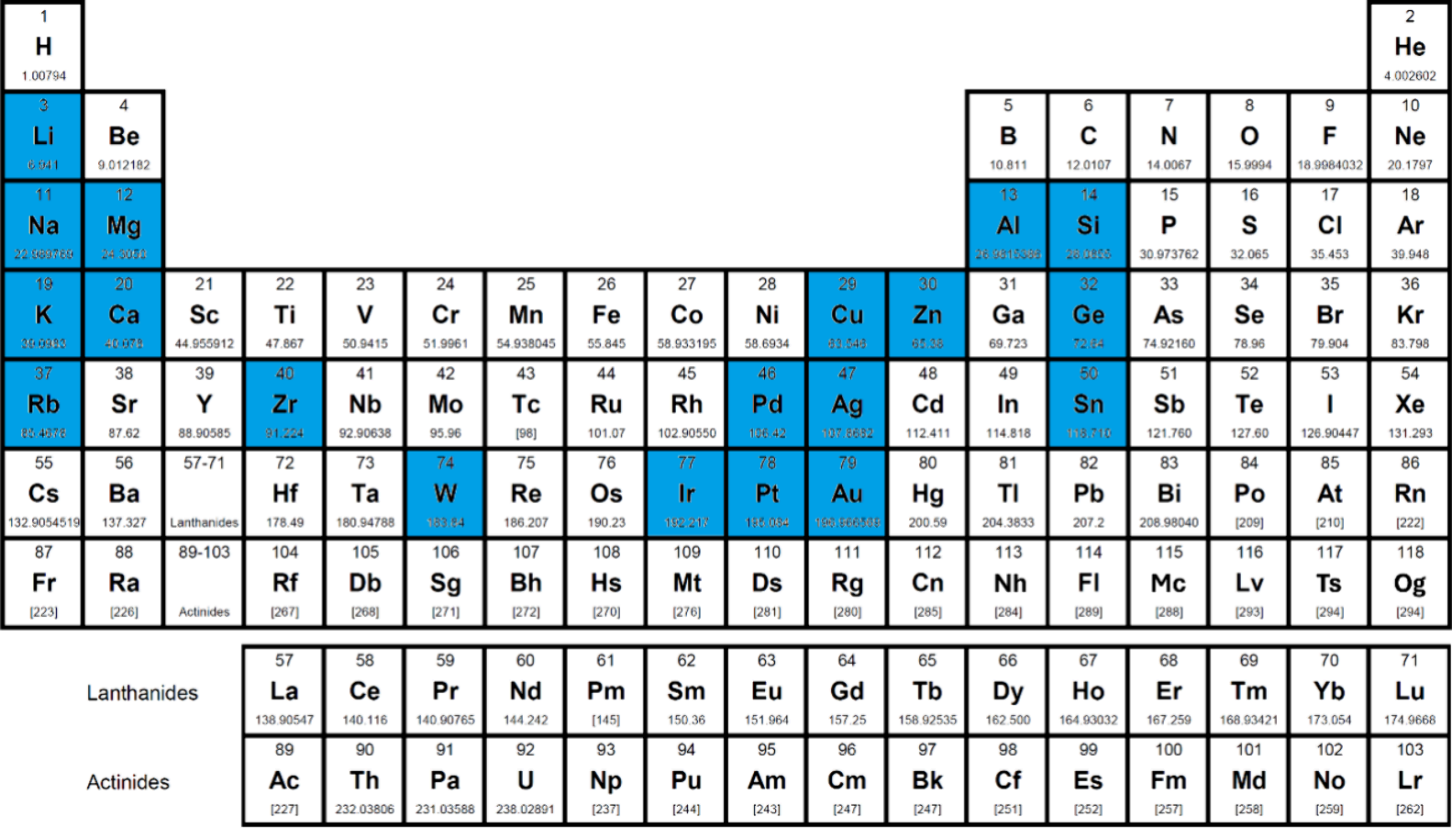
Periodic table with metals that feature in TADF-active materials colored in blue.

The existence of TADF in metal complexes has been known since the pioneering work of McMillin, who identified that the time-resolved PL decays of **[Cu(dmp)_2_]BF_4_** were temperature dependent, indicating interconversion between singlet and triplet excited states (Figure 93).^70,747^ Following these initial reports, a number of copper(I) complexes with similar photophysical behavior were disclosed. There was little interest/application for these complexes though until 2004 when the first bright OLED was fabricated using **[Cu(dnbp)(DPEphos)]BF_4_**, which showed a current efficiency of 10.5 cd A^–1^ and a maximum luminance of 1663 cd m^–2^.^74^ This marked the starting point for a rapid expansion in research into emissive copper complexes and their use in OLEDs (Figure 93).^748–754^ A notable milestone in the steady improvement in OLED performance was the use of **[Cu_2_I_2_(dppb)_2_]**, where the EQE_max_ reached 4.8%.^75^ However, at that time no copper-based OLEDs surpassed the 5% EQE limit that would have permitted confident assertion that triplet excitons were being harvested for emission.

**Figure 93:**
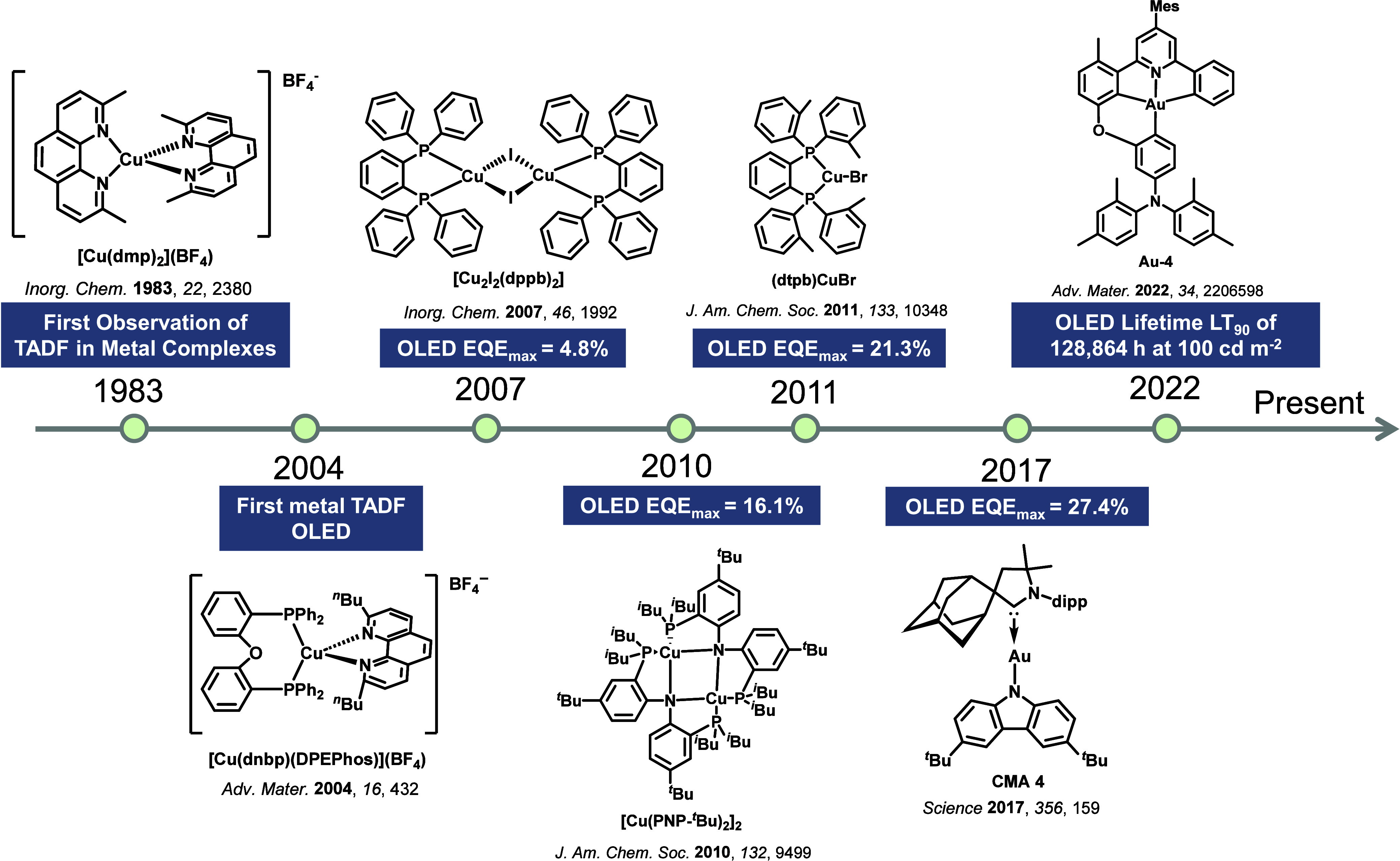
Timeline of milestones achieved using metal-containing (Cu and Au) TADF emitters discussed in the Introduction. The OLED EQE_max_ has increased from 4.8% to 27.4% over a period of 10 years.

In 2010 Deaton *et al*. reported an OLED with **[Cu(PNP-^t^Bu)_2_]_2_** that showed an EQE_max_ of 16.1% (Figure 93).^73^ At this point TADF was well established as an operational photoluminescence emission mechanism for copper complexes, and this result also established the same for electroluminescence. Identification of TADF in other metal complexes rapidly expanded, especially in other coinage metal complexes.^755,756^ The development of copper(I) complexes as TADF emitters also continued, with the monometallic 3-coordinate complex **(dtpb)CuBr** used in an OLED that showed an EQE_max_ = 21.3% in 2011.^757^ The report of highly emissive linear carbene metal amide (CMA) complexes of copper(I) and gold(I) in 2017 threw the field of metal-containing TADF materials into overdrive, as the solution-processed OLEDs with the gold(I) complex **CMA-4** could reach an EQE_max_ = 27.4% and showed very low efficiency roll-off.^194^ Since the first report of TADF emission from gold(III) complexes in 2015,^758^ materials development has continued apace, exemplified currently by **Au-4**, where the OLED showed an EQE_max_ of 27.3% along with low efficiency roll-off and long lifetime.^759^ These and other reports of similar performance put metal-containing TADF materials on equal footing with all-organic TADF emitters.

Analogous to all-organic TADF emitters, the emissive excited states in metal-containing TADF emitters have dominant charge-transfer character. However frequently these CT states involve transitions to/from metal-based orbitals. Categorized by the electronic and structural role of the metal center, there are several different classes of metal-TADF complexes, illustrated in Figure 94. The most common type of metal-containing TADF emitter benefits from a large contribution of metal d-orbitals to the excited state, either resulting in metal-to-ligand charge transfer (MLCT) or ligand-to-metal charge transfer (LMCT) states. The CT states of some metal complexes instead have no, or minimal, involvement of the metal d-orbitals, which are best described as ligand-to-ligand charge transfer (inter-ligand charge transfer, LLCT) excited states. In these complexes SOC from the metal center is subdued, and they behave comparably to organic Donor-Bridge-Acceptor TADF compounds with the metal center acting as the bridging element between different ligands acting as the donor and acceptor. A final class of metal-containing TADF emitter has the excited state localized on a single ligand in an intra-ligand charge transfer (ILCT) excited state, with this ligand itself effectively comprising a D-A TADF molecule. In these cases the metal acts as a Lewis acid, stabilizing the orbitals compared to those of the free ligand, and may also enhance the SOC between the excited states. Of particular interest are examples where the ligand is non-emissive, but coordination of a metal is capable of “turning on” both emission and TADF.^760^

**Figure 94:**
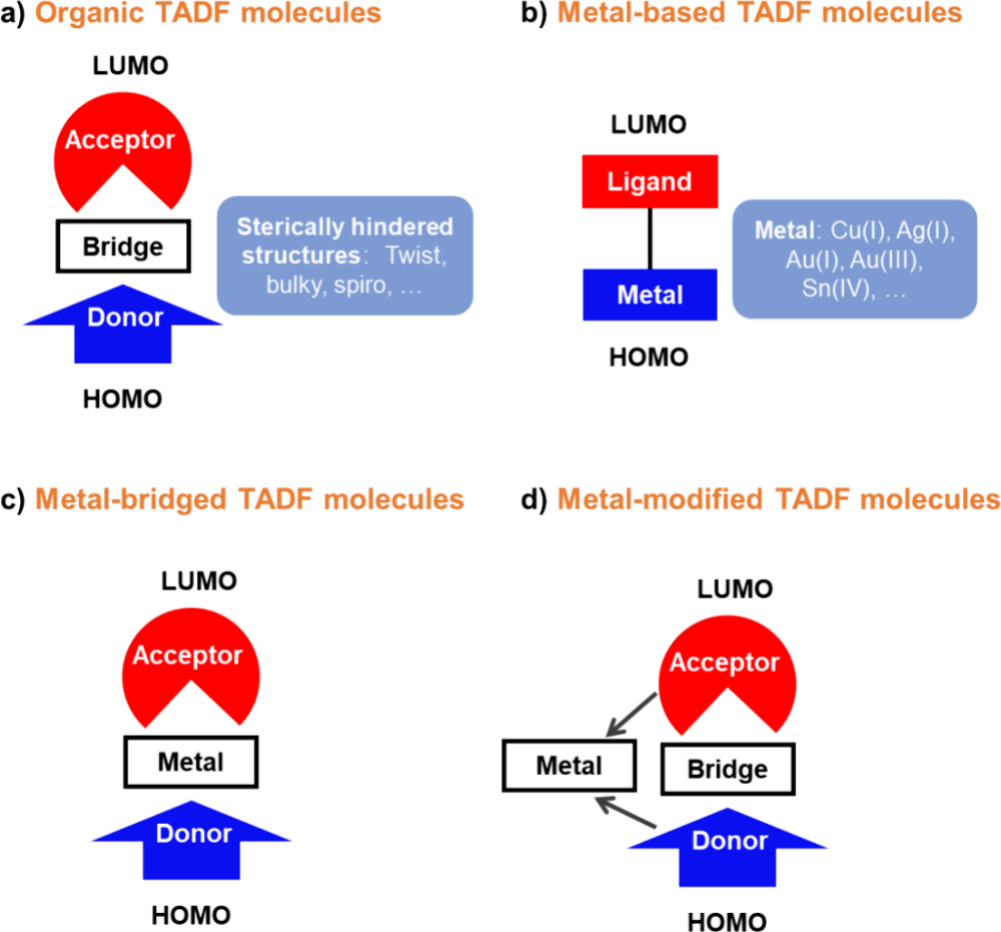
Diagrams representing the different forms of metal-containing TADF complexes. a) Organic Donor-Acceptor TADF molecule for comparison. b) Metal-containing TADF emitter with a metal directly involved in the charge transfer excited state. c) Metal-bridged TADF complexes, in which the metal bridges donor and acceptor moieties where, respectively, the HOMO and LUMO are located. d) Metal complex with a D-A TADF ligand. Taken and adapted with permission from ref 761. Copyright [2020/Advanced Optical Materials] John Wiley & Sons.

The presence of metals with very large atomic mass typically results in the SOC between low-lying singlet and triplet states becoming significantly larger than in purely organic TADF emitters. This impacts and simplifies the excited-state kinetics of these molecules in several ways. When SOC accelerates *k*_ISC_ to > 10^10^ s^–1^, intersystem crossing can outcompete radiative emission from the S_1_ state. This results in the rate of TADF emission becoming independent of further small changes in *k*_ISC_ and instead dependent primarily on the radiative decay rate (*k*_S1_) and the equilibrium constant for ISC/RISC cycling between S_1_ and T_1_ (*K*_eq_).^762^ This situation mirrors the emission kinetics for organometallic phosphors, in which heavy-atom SOC also enables ultrafast initial ISC. SOC will also simultaneously increase radiative decay from the triplet state, and as a result phosphorescence can become a competitive radiative process alongside TADF in these materials, even at room temperature. Emission properties (time-resolved PL decays and others, see below) must be carefully analyzed to determine if emission is purely TADF, purely phosphorescence, or a combination of the two. A representation of the combined emission is seen in Figure 95.^763^ The balance of these two processes has been studied in detail for a number of metal complexes, in particular copper(I) complexes.^763–772^

**Figure 95:**
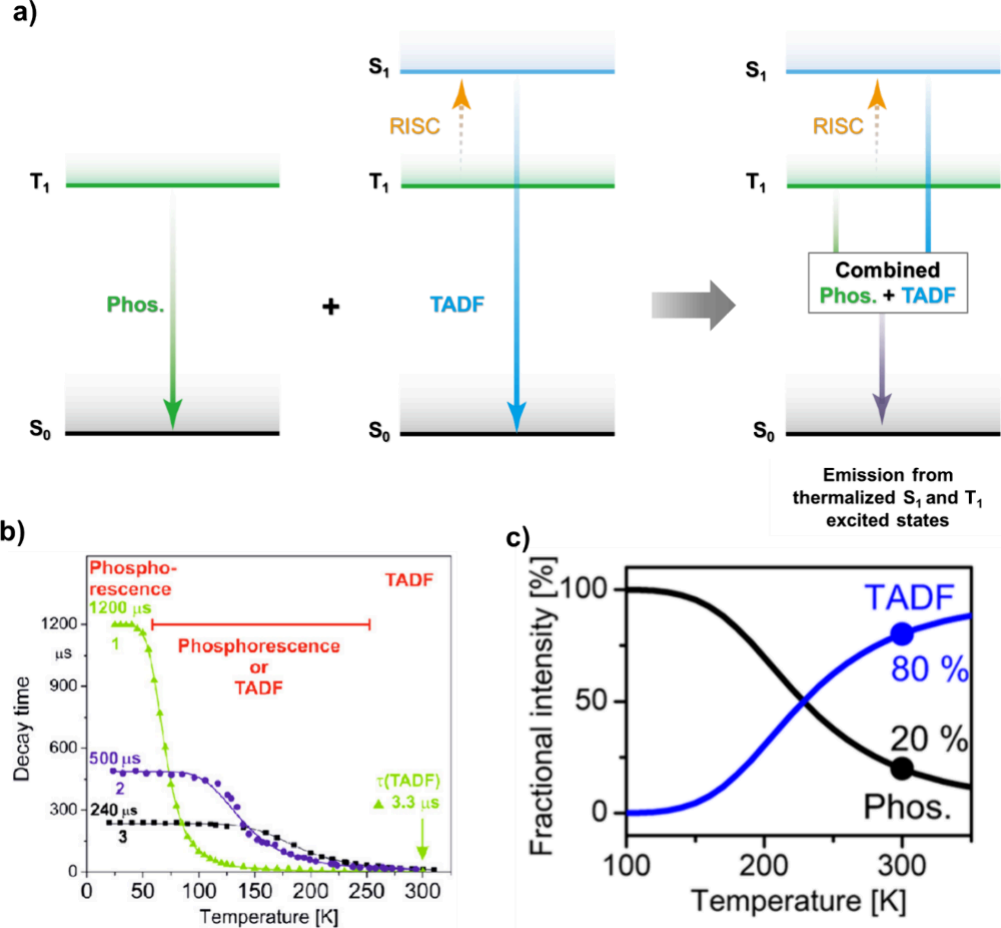
a) Representation of the excited states involved in emission for metal complexes that exhibit both TADF and phosphorescence at room temperature, taken from ref 763. Copyright [2015/Journal of the American Chemical Society] American Chemical Society. b) Example of a comparison between emission lifetime and temperature, along with the derived τ(S_1_) and ΔE_ST_ via the Boltzmann equation shown in equation 1, taken from Inorg. Chem. 2015, 54, 4322 25894718 10.1021/ic503072u with permission.^773^ c) Simulation of the emission fractions from TADF and phosphorescence and their temperature dependence for Cu_2_Cl_2_(N^∧^P)_2_, taken from ref 763. Copyright [2015/Journal of the American Chemical Society] American Chemical Society.

Determining the nature of the emission process occurring in a metal complex can be difficult. Both TADF and phosphorescence in metal complexes may have similar emission lifetimes and spectra, and even room temperature steady-state emission cannot always be unambiguously assigned to either singlet (TADF) or triplet (phosphorescence) excited states. In some cases, TADF can be inferred through comparison of the room temperature steady-state and delayed fluorescence spectra to the low temperature time-gated phosphorescence spectra, where these are distinguishable. In other cases, identifying TADF or phosphorescence emission (or mixed emission) in metal complexes relies on a detailed study of the temperature dependence of the emission lifetimes.^763,764^ The overall decay time of the system can be modelled according to equation 18, which models the decay kinetics according to a three-state model under the assumption of SOC-assisted rapid thermal equilibrium between the populations of T_1_ and S_1_.^774^ At very low temperatures that deactivate TADF the measured lifetime of the system corresponds to the pure phosphorescence lifetime τ(T_1_), allowing the TADF lifetime τ(S_1_) and Δ*E*_ST_ to be inferred by fitting the emission lifetime across different temperatures (for an example see Figure 95c).^748,763,764,773,774^

18 $$\tau_{obs}=\frac{1+\frac{1}{3}\;exp\left(-\frac{\Delta E_{ST}}{k_{b}T}\right)}{\frac{1}{\tau \left(T_{1}\right)}+\frac{1}{3\tau \left(S_{1}\right)}\;exp\left(-\frac{\Delta E_{ST}}{k_{b}T}\right)}$$

As well as the emission lifetime, the intensity contributions of TADF and phosphorescence emission have a strong temperature dependence, as illustrated in Figure 95c. Hence, when the spectra are readily resolvable, modelling this ratio at different temperatures can also be used to extract Δ*E*_ST_. There is a similar temperature dependence observed in organic TADF compounds as well; however, as the SOC in organic compounds is much smaller, the rate of phosphorescence is normally so slow that it is not observed at room temperature (or reasonably assumed to be negligible).^102^ As SOC alone is also not able to instantly establish an equilibrium between singlets and triplets in all-organic TADF emitters following photoexcitation, more involved modelling procedures are typically required.^99,102^

Likely because of these challenges, many emissive complexes are reported without explicit assignment of the emission to either fluorescence, TADF, phosphorescence, or some combination thereof. Many reports also lack the photophysical data needed for a reader to reasonably infer this assignment. As a result, we propose that the number of TADF metal complexes is likely significantly under-reported, especially considering that many luminescent metal complexes emit from CT states, often with small anticipated Δ*E*_ST_. Despite these technical challenges the field of emissive metal complexes is sufficiently mature to have been covered in many reviews, with several reviews focused on TADF metal complexes.^761,775–779^ Metal-containing TADF emitters are additionally discussed in a number of reviews that encompass either metal emitters^780–782^ or TADF emitters^35,86,783^ more widely. Given the early discovery and extensive study of TADF emission from copper complexes, there are several reviews covering this topic specifically,^748–754^ along with others that focus more widely on the photophysical properties of coinage metal complexes.^784–786^

In this section, we review reported metal complexes where the authors have explicitly assigned the emission to TADF. The survey is divided into different sub-sections based on the metal in the complex. The copper, silver, and gold sub-sections cover selected complexes that demonstrate the history of coinage metal TADF emitters, highlighting key structural motifs or reports of particularly notable emission properties and OLED performance. Sub-sections concerning Carbene-metal-amide (CMA), palladium and platinum, zinc and other metals are comprehensive in scope and include all examples of metal complexes that have experimentally reported TADF emission. This section does not discuss the TADF properties of large metallic clusters^787–790^ and coordination polymers^791,792^ that have recently been shown to exhibit TADF (albeit likely with more exotic underlying emission mechanisms). The emission properties of the complexes discussed here are also summarized in Table S11, and the performance of OLEDs fabricated from metal-containing TADF emitters are collated in Table S12.

### 9.2 Copper

Since the first report of delayed fluorescence by McMillin and co-workers,^70^ copper(I) complexes have emerged as the most abundant group of metal-containing TADF emitters. To give some indication of the scale of reported emissive copper complexes, a recent review provides absorption and emission data for more than 1200 photoactive monometallic copper(I) complexes,^753^ although only a portion of these exhibit TADF. In 1999, the first report of a copper(I) emitter used in an OLED concerned the phosphorescent complex **Cu_4_(C≡CPh)_4_(L)_2_**;^793^ however, by 2007 examples of copper(I) TADF OLEDs had been reported using **[Cu(μ-I)(dppb)]_2_** (Figure 93).^75^

The majority of luminescent copper(I) emitters are 4-coordinate tetrahedral complexes like **[Cu(dmp)_2_]BF_4_** reported by McMillin and co-workers (Figure 96).^70,748^ The weakly emissive nature of many of these complexes is due to significant non-radiative decay, arising from Jahn-Teller distortion in the MLCT excited state as copper center becomes formally Cu(II).^753,794–796^ Increasing the steric bulk of the ligands in these tetrahedral copper(I) complexes restricts this excited-state distortion (as well as addressing ligand dissociation and exciplex formation) improving the photophysical and emission properties of these complexes.^753,774,797–799^ The tetrahedral complexes summarized here are further sub-divided into categories based on their structure: cationic bis-diimine complexes, cationic diimine/diphosphine complexes, switchable neutral/cationic complexes, and neutral complexes.

**Figure 96:**
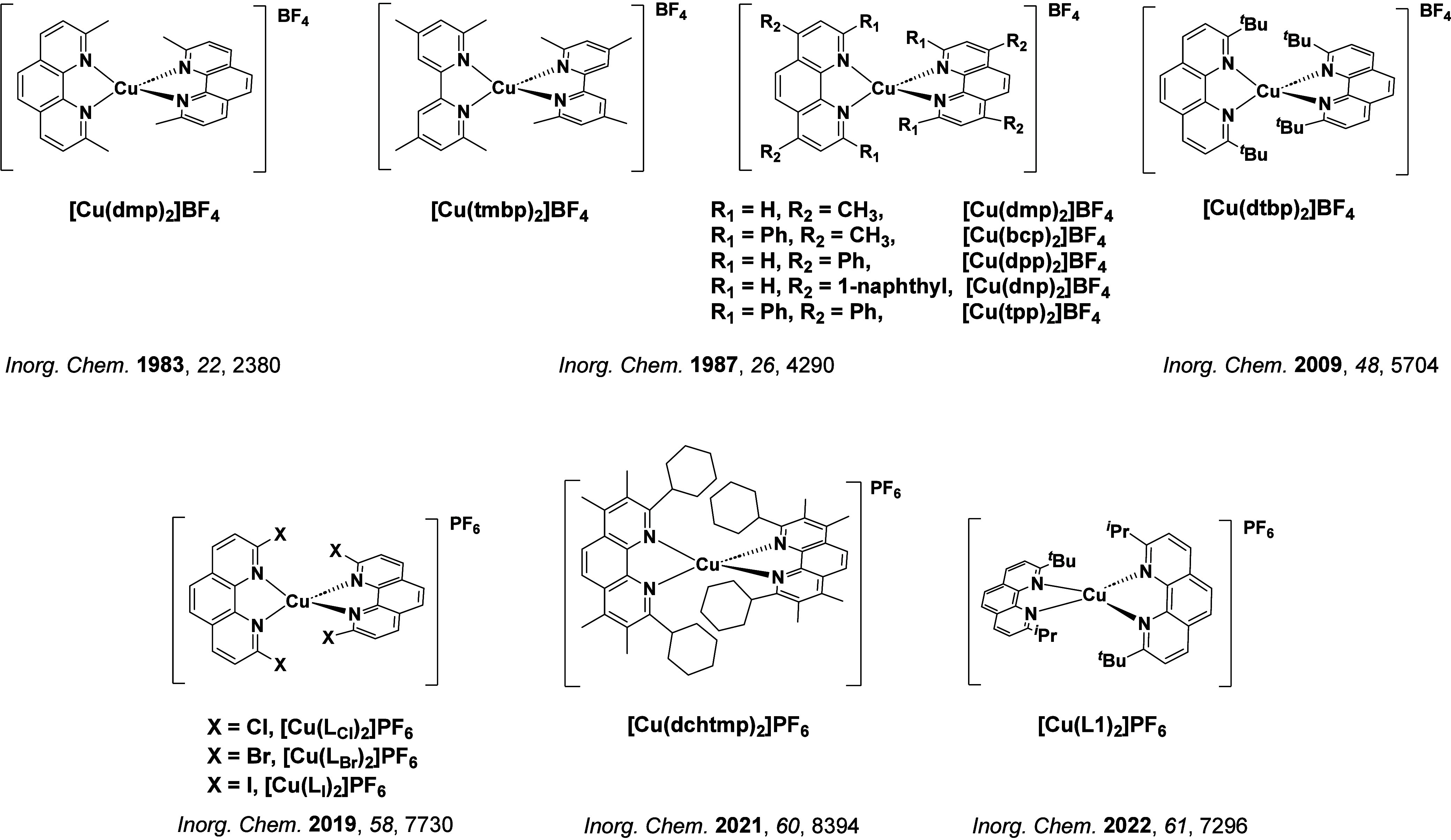
Cationic bis-diimine copper(I) complexes [Cu(N^∧^N)_2_]^+^ having TADF properties.

Following from the initial report of **[Cu(dmp)_2_]BF_4_**, numerous other cationic bis-diimine copper(I) complexes have been reported that show weak emission in solution and moderately strong emission in the solid state (Figure 96). The emission intensity in these [Cu(N^∧^N)_2_]^+^ complexes is increased when bulky ligands or solid-state interactions are used to restrict the Jahn-Teller distortion in the excited state. This was demonstrated in a series of complexes with substituents of increasing steric bulk at the 2- and 9-positions of a phenanthroline ligand, **[Cu(bcp)_2_]BF_4_**, **[Cu(dnp)_2_]BF_4_**, **[Cu(tmbp)_2_]BF_4_**, **[Cu(dpp)_2_]BF_4_**, and **[Cu(tpp)_2_]BF_4_**.^800^ In DCM solution the complexes are all red emitters (λ_PL_ = 710–750 nm) with Φ_PL_ increasing from 0.03% for the weakly emissive **[Cu(dmp)_2_]BF_4_** to 0.15% for the bulkiest complex **[Cu(tpp)_2_]BF_4_**.

Further increases in the steric bulk of the diamine ligands result in [Cu(N^∧^N)_2_]^+^ complexes showing reduced geometric reorganization in the excited state and thus are yet more emissive. An example of how emissive these complexes can become is **[Cu(dtbp)_2_]BF_4_** (Figure 96), which has a Φ_PL_ = 5.6% in DCM solution. This material, however, also undergoes ligand displacement more readily than other copper-phenanthroline complexes due to the steric demands of the *tert*-butyl groups, and thus the relatively weaker coordination of the dtbp ligands to the copper centre.^801^ The photophysical properties of **[Cu(dchtmp)_2_]PF_6_** with a bulky 2,9-dicy­clo­hex­yl-3,4,7,8-tetra­meth­yl-1,10-phen­an­thro­line ligand (Φ_PL_ = 5.5% in DCM)^802^ are similar to those of **[Cu(dtbp)_2_]BF_4_** yet this material is more chemically inert and does not suffer from ligand dissociation to the same extent. To explore the limits of the steric bulk that can be installed in the 2- and 9-positions of the phenanthroline, the asymmetric ligand 2-iso­pro­pyl-9-*tert*-but­yl-1,10-phen­an­thro­line that is sterically in between those of the dtbp and dipp was investigated. The resulting complex **[Cu(L_1_)_2_]PF_6_** ^803^ is surprisingly weakly emissive (τ_PL_ of 0.13 μs and Φ_PL_ of 0.17%) but inert to ligand dissociation. The use of this asymmetric ligand was indeed found to lead to more distortion in the excited state, increasing non-radiative decay and resulting in a shorter lifetime and weaker emission compared to those of the reference emitter **[Cu(dipp)_2_]BF_4_** (τ_PL_ = 0.34 μs; and Φ_PL_ = 0.4%).^804^

The impact of peripheral heavy atoms on the emission properties of [Cu(N^∧^N)_2_]^+^ complexes was explored by replacing the methyl groups in **[Cu(dmp)_2_]BF_4_** with halide atoms to form **[Cu(L_Cl_)_2_]PF_6_**, **[Cu(L_Br_)_2_]PF_6_**, and **[Cu(L_I_)_2_]PF_6_** (Figure 96).^805^ The chloride atoms have very little impact on the emission properties of the complex; however, in DCM both the bromine and iodine complexes have higher Φ_PL_ (0.08 and 0.09%) than non-halogenated **[Cu(dmp)_2_]BF_4_** (Φ_PL_ = 0.024%) and identical longer τ_PL_ of 0.11 μs, compared to 0.085 μs, due to increased phosphorescence radiative decay rates resulting from increased SOC. Additionally, while both **[Cu(L_Cl_)_2_]PF_6_** and **[Cu(L_Br_)_2_]PF_6_** show TADF, **[Cu(L_I_)_2_]PF_6_** emits only by phosphorescence as the high SOC of the iodine atoms significantly increased the rate of emission from T_1_.

Soon after the initial reports of emissive [Cu(N^∧^N)_2_]^+^ complexes, researchers started to explore the photophysical properties of heteroleptic complexes containing bulky phosphine (P) or diphosphine ligands (P^∧^P). Among several early reports of luminescent [Cu(N^∧^N)P_2_]^+^ complexes with photophysics incompatible with simple singlet emission, McMillin and co-workers identified TADF in **[Cu(dmp)(PPh_3_)_2_]BF_4_** (Figure 97).^806,807^ This complex is a weak green-yellow emitter (λ_PL_ = 560 nm, Φ_PL_ = 0.14%, τ_PL_ = 330 ns) in methanol. After this initial report, there were numerous reports of phosphorescent and otherwise luminescent [Cu(N^∧^N)(P^∧^P)]^+^ complexes;^74,798,808–810^ however, none of these reports claimed that the emission was TADF. In 2012, Yersin and co-workers reported that a related and previously studied complex, **[Cu(dmp)(POP)]BF_4_**,^74,798^ also emits by TADF (λ_PL_ = 538 nm, Φ_PL_ = 80%, τ_PL_ = 18 μs) with Δ*E*_ST_ of 110 meV.^811^ Since this report many more emissive [Cu(N^∧^N)(P^∧^P)]^+^ complexes have been prepared,^753^ with selected examples that have clearly identified TADF emission shown in Figure 97.

**Figure 97:**
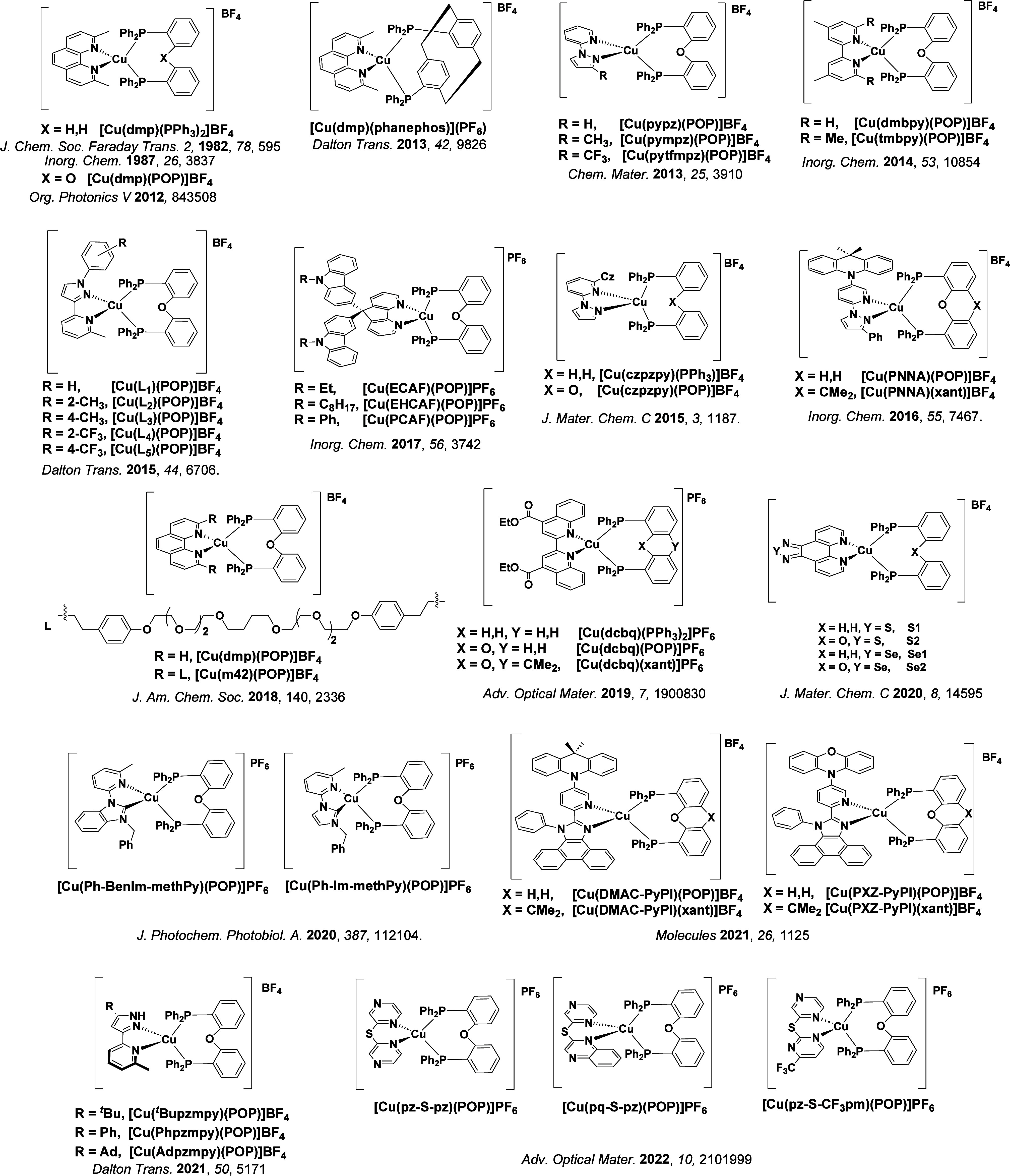
Cationic diimine/diphosphine copper(I) complexes [Cu(N^∧^N)(P^∧^P)]^+^ having TADF properties. POP = DPEPhos = bis((2-di­phen­yl­phos­phino)­phen­yl)­ether, xant = xantphos.

Yersin and co-workers later reported **[Cu(dmp)(phanephos)]BF_4_** (Figure 97), containing a bulky and rigid cyclophane-diphosphine ligand that showed green TADF emission (λ_PL_ = 530 nm, Φ_PL_ = 80%, τ_PL_ = 14 μs) as a powder.^812^ The temperature dependence of the emission lifetime was used to estimate the Δ*E*_ST_ of 140 meV. The strong emission from this complex relative to other copper complexes (especially in DCM, Φ_PL_ = 40%) was attributed to the rigid coordination environment provided by the phanephos ligand.

A series of substituted 2-pyridyl-pyrazoyl N^∧^N ligands was used to prepare complexes **[Cu(pypz)(POP)]BF_4_**, **[Cu(pympz)(POP)]BF_4_**, and **[Cu(pytfmpz)(POP)]BF_4_** (Figure 97).^813^ The electron-rich pypz ligand was used to tune the emission of the complexes to the blue through LUMO destabilization. Of the three, **[Cu(pympz)(POP)]BF_4_** has the most blue-shifted emission (λ_PL_ = 465 nm as a powder). The λ_PL_ values range from 465 to 492 nm, Φ_PL_ range from 56 to 87%, and τ_PL_ range from 12.2 to 22.8 μs, despite all having the same calculated Δ*E*_ST_ of 180 meV, which corresponds very well with the measured Δ*E*_ST_ of 170 meV for **[Cu(pytfmpz)(POP)]BF_4_**. Solution-processed OLEDs with **[Cu(pypz)(POP)]BF_4_** in 26mCPy showed EQE_max_ of 3.2% at λ_EL_ of 516 nm, while **[Cu(pympz)(POP)]BF_4_** in DPEPO showed EQE_max_ of 3.7% at λ_EL_ of 484 nm. The device with **[Cu(pytfmpz)(POP)]BF_4_** in DPEPO showed a considerably higher EQE_max_ of 8.5% at λ_EL_ of 508 nm.

A family of five complexes with differently methyl- or trifluoromethyl-substituted pyridylpyrazoyl ligands, **[Cu(L_1_)(POP)]BF_4_** to **[Cu(L_5_)(POP)]BF_4_** (**1** to **5** in that work, Figure 97) show blue TADF emission in both powder (λ_PL_ from 464 to 481 nm, Φ_PL_ from 82 to 99%, τ_PL_ from 4.1 to 16.9 μs) and doped films in PMMA.^814^ The Δ*E*_ST_ for the complexes ranged between 80 and 90 meV. A similar family of very bright blue-green emissive complexes with C3 (rather than N2) substituted pyridylpyrazolyl ligands, **[Cu(^t^Bupzmpy)(POP)]BF_4_**, **[Cu(Phpzmpy)(POP)]BF_4_**, and **[Cu(Adpzmpy)(POP)]BF_4_** were also prepared.^815^ Both families of pyridylpyrazolyl-containing complexes have very similar photophysics, with powders emitting at λ_PL_ of between 498 to 523 nm, Φ_PL_ ranging from 71 to 91%, and τ_PL_ of between 13.4 to 34.1 μs. This second set of complexes have Δ*E*_ST_ values between 90 and 100 meV, again similar to the first set.

A pair of complexes containing a substituted bipyridine as the diimine ligand, **[Cu(dmbpy)­(POP)]BF_4_** and **[Cu(tmbpy)­(POP)]BF_4_** (Figure 97), are also reported as TADF-active.^816^ Of the two, the emission in **[Cu(tmbpy)­(POP)]BF_4_** is much stronger due to reduced excited-state distortion imposed by the additional methyl groups at the 6- and 6′-positions of the bipyridine ligand. **[Cu(tmbpy)­(POP)]BF_4_** emits at λ_PL_ of 555 nm, has a Φ_PL_ of 74% and a τ_PL_ of 13 μs as a powder with an associated Δ*E*_ST_ of 78 meV, the less hindered complex **[Cu(dmbpy)­(POP)]BF_4_** has a lower Φ_PL_ of 9% as a powder.

A series of spiro-carbazole ligands was used to prepare **[Cu(ECAF)­(POP)]PF_6_**, **[Cu(EHCAF)(POP)]PF_6_**, and **[Cu(PCAF)(POP)]PF_6_** (Figure 97).^817^ These complexes are of particular interest as the bulky spirocarbazole ligands allow the cationic complexes to be sublimed to fabricate vacuum-deposited OLEDs. The complexes are green TADF emitters in the solid state (λ_PL_ = 525 to 528 nm, Φ_PL_ = 31 to 33% in PMMA films), with Δ*E*_ST_ of 90 meV for all three. The best performing OLEDs used 10 wt% **[Cu(ECAF)­(POP)]PF_6_** in mCP and showed EQE_max_ of 14.8% at λ_EL_ of 544 nm and CIE coordinates of (0.37, 0.55); however, the efficiency roll-off was severe (EQE_4000_ = 2%) and the turn-on voltage was high at 5.2 V, both attributed to poor electron confinement in the emissive layer featuring this uncommon ionic emitter.

An interesting strategy was employed for the design of **[Cu(czpzpy)­(PPh_3_)_2_]BF_4_** and **[Cu(czpzpy)­(POP)]BF_4_**, (Figure 97),^818^ with the carbazole-substituted pyridylpyrazoyl ligand also acting as a host material for OLEDs. The complexes are green TADF emitters as powders, with **[Cu(czpzpy)­(PPh_3_)_2_]BF_4_** emitting at λ_PL_ of 495 nm and having Φ_PL_ of 45% and τ_PL_ of 134 μs, while **[Cu(czpzpy)­(POP)]BF_4_** emits at λ_PL_ of 518 nm, has Φ_PL_ of 95% and a τ_PL_ of 23 μs. The TADF emission is supported by Δ*E*_ST_ of 180 meV and 130 meV, respectively. Due to its higher Φ_PL_ and shorter τ_PL_, **[Cu(czpzpy)­(POP)]BF_4_** dispersed in additional ligand czpzpy as host was used as the emitter in a solution-processed OLED. The green OLED showed an EQE_max_ of 6.3% at CIE coordinates of (0.26, 0.49), while no efficiency roll-off out to 100 cd m^–2^ was observed. Interestingly, devices with the same performance could be obtained by spin-coating a solution of **[Cu(NCMe)_2_­(POP)]BF_4_** and czpzpy, showing that the copper complex could be formed *in-situ* during the solution-processing of the device. Related complexes **[Cu(PNNA)(POP)]BF_4_** and **[Cu(PNNA)(xant)]BF_4_** (Figure 97) contain a diimine ligand decorated instead with a DMAC donor.^819^ The diimine ligand acts as an electron acceptor in this case, which resulted in an ILCT state from the electron-donating DMAC group (more so than carbazole) and TADF emission emerging from the ligand itself. The two complexes only differ by fusing of the other POP/xanthene-based ligand, and so show similar photophysics as 20 wt% doped films in mCP (λ_PL_ = 482 to 492 nm, Φ_PL_ = 70 to 74%, τ_PL_ ≈ 50 μs). The highest performance solution-processed OLED used **[Cu(PNNA)(xant)]BF_4_** and showed an EQE_max_ = 7.4% at CIE coordinates of (0.21, 0.43).

A novel strategy to reduce the kinetic lability of ligands in [Cu(N^∧^N)(P^∧^P)]^+^ complexes involves the generation of a pseudorotaxane structure, wherein the ligated diphosphine is effectively encircled by a macrocycle containing the bound N^∧^N ligand.^820,821^ Of the complexes synthesized to explore this design, **[Cu(m42)(POP)]BF_4_** (Figure 97) was found to be the most kinetically inert. This complex has a Φ_PL_ of 23% in DCM rising to 41% when doped at 1 wt% in PMMA, which is similar to reference compound **[Cu(dmp)(POP)]BF_4_** (23 and 50%, respectively). The key difference between these complexes manifests in the OLED performance, where although the EQE_max_ are similar (at 10.5 and 9.5%, respectively) the device with **[Cu(m42)(POP)]BF_4_** is found to be more stable and could attain a much higher maximum luminance of 12,800 cd m^–2^ vs. 7740 cd m^–2^ for the device with **[Cu(dmp)(POP)]BF_4_**.

Using a more strongly π-accepting 2,2′-biquinoline N^∧^N ligand, dcbq, resulted in the red-emitting complexes **[Cu(dcbq)(PPh_3_)_2_]PF_6_**, **[Cu(dcbq)(POP)]PF_6_**, and **[Cu(dcbq)(xant)]PF_6_** (Figure 97).^822^ These complexes emit at λ_PL_ ranging from 669 to 671 nm, have Φ_PL_ ranging from 26 to 56%, and τ_PL_ ranging from 0.58 to 0.71 μs, while Δ*E*_ST_ was not reported. With increasing size and rigidity of the phosphine ligand(s) and resulting suppression of Jahn-Teller distortion there was a progressive enhancement of the Φ_PL_.

The use of heavy chalcogens is another strategy to enhance SOC as illustrated by the use of thiadiazole and selenodiazole ligands in the complexes **S1**, **S2**, **Se1** and **Se2** (Figure 97).^823^ In 10 wt% doped films in PMMA, all four complexes are weakly yellow-orange emissive, with λ_PL_ between 577 and 605 nm, Φ_PL_ of between 4 to 8%, and τ_PL_ ranging from 0.8 to 1.2 μs. Notably, *k*_r_ in the Se-containing complexes is twice that of the sulfur-containing complexes. However, this was attributed not to enhanced SOC but rather to greater spatial separation of the frontier orbitals, resulting in a smaller Δ*E*_ST_. Supporting this interpretation the τ_PL_ of **Se2** is only 0.8 μs, which was the shortest emission lifetime of any [Cu(N^∧^N)(P^∧^P)]^+^ complex at the time.

Bulky NHC ligands have been widely used in emissive complexes^194,781^ but have seen limited use in tetrahedral copper(I) complexes. One of the few such reports describes complexes **[Cu(Ph-BenIm-methPy)(POP)]PF_6_** and **[Cu(Ph-Im-methPy)(POP)]PF_6_** (Figure 97), that combine a pyridyl NHC ligand with a POP P^∧^P ligand.^772^ The complexes are very bright sky-blue emitters as powders with λ_PL_ = 493 and 487 nm, Φ_PL_ > 96% for both, and τ_PL_ = 63 and 56 μs, respectively. The Δ*E*_ST_ are 128 and 108 meV, but temperature-dependent PL studies revealed that at room temperature only about 35% of the emission originates from the S_1_ state (TADF), while the remainder is concurrent phosphorescence from the T_1_ state.

Complexes **[Cu(DMAC-PyPI)­(POP)]BF_4_**, **[Cu(DMAC-PyPI)­(xant)]BF_4_**, **[Cu(PXZ-PyPI)­(POP)]BF_4_**, and **[Cu(PXZ-PyPI)­(xant)]BF_4_** (Figure 97) contain N^∧^N ligands that possess both electron-donating (DMAC/PXZ) and electron-accepting (phenanthroimidazole) groups.^824^ These four complexes emit at λ_PL_ between 534 and 564 nm, have Φ_PL_ ranging from 42 to 71%, and τ_PL_ of between 4.3 and 24.1 μs. The photophysical properties of these complexes is entirely dependent on the nature of the D-A N^∧^N ligand and all complexes emit from an ILCT state with Δ*E*_ST_ ranging from 50 and 110 meV. Interestingly, the the copper ion is not entirely decorative, with the Δ*E*_ST_ of the free ligands larger at 450 and 310 meV for DAMC-PyPI and PXZ-PyPI, respectively. This study highlights how coordination to the copper can tune the energy levels of the orbitals localized on the N^∧^N ligand to enable TADF emission. Green emitting solution-processed OLEDs with **[Cu(DMAC-PyPI)­(xant)]BF_4_** and **[Cu(PXZ-PyPI)­(POP)]BF_4_** showed EQE_max_ ranging from 3.8 to 8.0% depending on emitter and doping concentration. The best performing device employed 16 wt% **[Cu(PXZ-PyPI)­(POP)]BF_4_** doped in PYD2 to achieve an EQE_max_ = 8.0%, which was maintained to EQE_1000_ > 5.0%.^824^

Employing strongly π-accepting pyrazinyl sulfide N^∧^N ligands provided an effective strategy to achieve red emitting [Cu(N^∧^N)(P^∧^P)]^+^ complexes.^825^ **[Cu(pz-S-pz)­(POP)]PF_6_**, **[Cu(pq-S-pz)­(POP)]PF_6_**, and **[Cu(pz-S-CF_3_pm)­(POP)]PF_6_** (Figure 97) as powders emit at λ_PL_ ranging from 581 to 650 nm, have widely varying Φ_PL_ of between 7.7 and 57.8%, and τ_PL_ of 6.47 to 10.5 μs. The Δ*E*_ST_ values are between 60 and 130 meV. The most promising red emitter **[Cu(pq-S-pz)­(POP)]PF_6_** (λ_PL_ = 642 nm, and with highest Φ_PL_ = 57.8% and shortest τ_PL_ = 6.47 μs) was used in LECs that showed very good performance for a red device (See Sections 16 and 5 for discussion of the challenges associated with this type of device and color).

As an alternative to the cationic copper(I) complexes described above, neutral complexes exhibiting TADF emission are of great interest. This is particularly because these non-ionic materials aree more readily evaporable and so are more compatible with vacuum deposition fabrication for OLEDs. Neutral tetrahedral copper(I) complexes can have a range of different ligand environments, from Cu(P^∧^P)(N^∧^N) bearing anionic diimine ligands, to the use of halido ligands in combination with one to three dative ligands. Representative examples are shown in Figure 98 and are discussed below.

**Figure 98:**
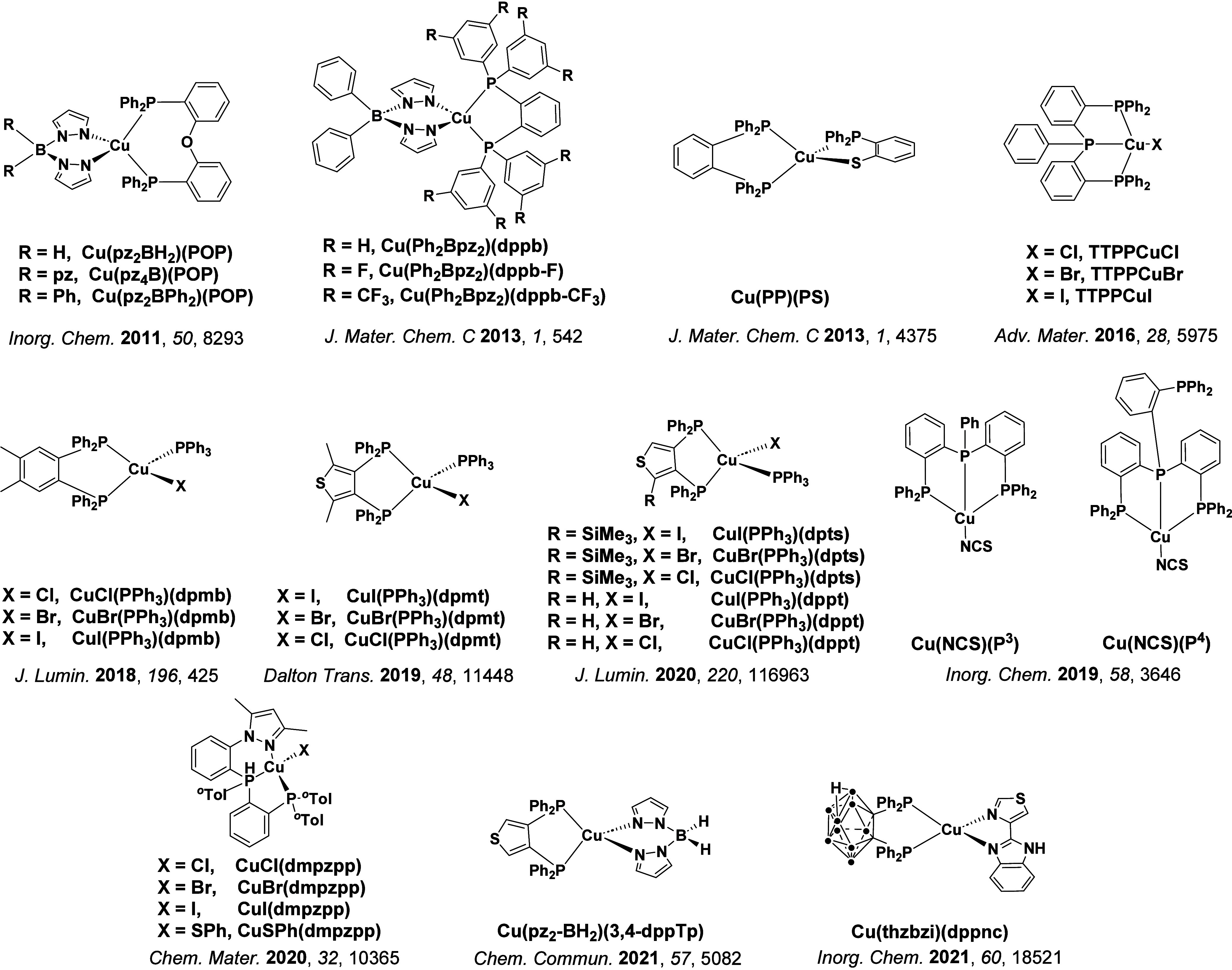
Neutral tetrahedral copper(I) complexes having TADF properties.

The first reported neutral copper(I) TADF complexes contained a POP ligand and a dipyrazolylborate ligand. Powders **Cu(POP)­(pz_2_BH_2_)**, **Cu(POP)­(pz_4_B)**, and **Cu(POP)(­pz_2_Bph_2_)** (Figure 98) are bright blue emitters (λ_PL_ = 436–464 nm, Φ_PL_ up to 90% and τ_PL_ = 13–22 μs, Δ*E*_ST_ = 99–161 meV).^774^ A similar series of three complexes contain the similar dipyra­zol­yl­di­phen­yl­borate ligand, **Cu(Ph_2_­Bpz_2_)­(dppb)**, **Cu(Ph_2_­Bpz_2_)­(dppb-F)**, and **Cu(Ph_2_­Bpz_2_)­(dppb-CF_3_)**.^826^ In doped mCP films (10 wt%), these complexes emit strongly in the green (λ_PL_ = 523 – 545 nm, Φ_PL_ = 50 – 68% and τ_PL_ = 3.6 – 8.2 μs). The green-emitting (λ_EL_ = 528 – 552 nm) OLEDs showed EQE_max_ ranging from 11.9 to 17.7%. The photophysics of **Cu(Ph_2_­Bpz_2_)­(dppb)** and **Cu(pz_2_­Bph_2_)­(POP)** were subsequently studied in more detail, confirming assignment of the emission to TADF and measuring the Δ*E*_ST_ to be 46 and 81 meV, respectively.^773^

An anionic phosphinothiolato ligand was used to prepare **Cu(PP)(PS)** (Figure 98).^756^ As a powder the complex emits at λ_PL_ of 521 nm, has a Φ_PL_ of 73%, and shows biexponential decay kinetics with τ_PL_ = 0.33 and 1.73 μs. Solution-processed OLEDs showed an EQE_max_ = 7.8% at CIE coordinates of (0.40, 0.53).

Beyond coordination environments with two bidentate ligands, 2-to-4-coordinate TADF copper complexes have been prepared using combinations of mono-, bi-, and tridentate ligands. **TTPPCuCl**, **TTPPCuBr**, and **TTPPCuI** (Figure 98) are examples of this, containing a halido ligand in combination with a triphosphine ligand.^766^ In neat films the complexes are bright green emitters (λ_PL_ = 521–530 nm, Φ_PL_ = 76–83% and τ_PL_ = 11–19 μs) and have Δ*E*_ST_ of 95–99 meV. While the Cl and Br complexes emit via TADF, the iodo complex showed mixed TADF/phosphorescence at room temperature (39% phosphorescence). The OLEDs with **TTPPCuCl**, **TTPPCuBr**, and **TTPPCuI** showed progressively increasing EQE_max_ from 9 to 12.2 and 16.3%, with the highest efficiency and lowest efficiency roll-off (6% decline at 1000 cd m^–2^) for the OLED with **TTPPCuI** attributed to the faster *k*_r_ in that emitter.

Replacing the tridentate phosphine ligand above with a combination of a P^∧^P ligands and a triphenylphosphine results in the complexes **CuCl(PPh_3_)(dpmb)**, **CuBr(PPh_3_)(dpmb)**, and **CuI(PPh_3_)(dpmb)** (Figure 98).^827^ The complexes are sky-blue emitters as powders (λ_PL_ = 464–479 nm, Φ_PL_ = 23–53% and τ_PL_ = 4.3–5.7 μs) and have calculated Δ*E*_ST_ between 98 and 152 meV. Replacement of the bridging dimethylbenzyl group with a dimethylthiophene produced the series of complexes **CuCl(PPh_3_)(dpmt)**, **CuBr(PPh_3_)(dpmt)**, and **CuI(PPh_3_)(dpmt)** (Figure 98).^828^ The thiophene was chosen as an electron-rich heteroaryl ring in an attempt to raise the LUMO energy of the complexes and blue-shift the emission. This was only modestly successful, with powder emission blue-shifted by approximately 10 nm compared to the dpmb analogues (λ_PL_ between 459 and 484 nm), and the powders were less emissive (Φ_PL_ ≤ 24%) while the calculated Δ*E*_ST_ range from 64 to 198 meV. A later report examined the effects of removing the methyl groups on the bridging thiophene as in **CuCl(PPh_3_)(dppt)**, **CuBr(PPh_3_)(dppt)**, and **CuI(PPh_3_)(dppt)**, or incorporate a trimethylsilyl group as in **CuCl(PPh_3_)(dpts)**, **CuBr(PPh_3_)(dpts)** and **CuI(PPh_3_)(dpts)** (Figure 98).^829^ These complexes show bright sky-blue to yellow-green emission (λ_PL_ = 485–535 nm). Notably, the introduction of the trimethylsilyl group increased the solubility of the complexes in most organic solvents and reduced their *k*_nr_ without affecting *k*_r_, resulting in both longer emission lifetimes (τ_PL_ increased from 4–10 μs to 20.8–48.9 μs) and higher Φ_PL_ (increased from 3–18% to 29–52%). The non-doped solution-processed OLED with **[CuBr(dpts)(PPh_3_)]** showed an EQE_max_ of 7.7% at λ_EL_ of 564 nm.

Monodentate ligands need not be limited to halido groups. Two complexes containing tridentate phosphine ligands and a thiocyanato group, **Cu(NCS)(P^3^)** and **Cu(NCS)(P^4^)** (Figure 98), showed green to yellow TADF emission.^830^ As powders the two complexes emit at λ_PL_ of 520 and 543 nm, have Φ_PL_ = 57 and 27% with τ_PL_ of 4.8 and 4.9 μs and Δ*E*_ST_ of 62 and 80 meV, all respectively.

Examples of complexes bearing another tridentate N,P,P-ligand include **CuCl(dmpzpp)**, **CuBr(dmpzpp)**, **CuI(dmpzpp)**, and **CuSPh(dmpzpp)** (Figure 98).^831^ **CuCl(dmpzpp)** is non-emissive, while the remaining complexes are bright green-yellow emitters as powders (λ_PL_ = 530–541 nm, Φ_PL_ = 82–90% and τ_PL_ = 5–9 μs). OLEDs with **CuI(dmpzpp)** and **CuSPh(dmpzpp)** doped in a mixed TCTA:​DPEPO host showed EQE_max_ between 10.8 and 16.4% across a range of doping concentrations (2–8%). The device with **CuI(dmpzpp)** showed the highest EQE_max_ of 16.4% and the lowest efficiency roll-off (EQE_1000_ = 10.2%), while the reduced performance of **CuSPh(dmpzpp)** was attributed to charge trapping in the emissive layer.

A complex with a thiophene-bridged diphosphine ligand and an anionic dipyrazolylborate ligand, **Cu(pz_2_BH_2_)(3,4-dppTp)** (Figure 98) showed both mechanochromism and vapochromism.^832^ The complex crystallizes in two polymorphs, which emit in the blue (**1B**) and yellow (**1Y**). Grinding **1B** produced a new material **1G** that is a green emitter. Exposing **1G** to solvent vapors (dichloromethane or diethyl ether) returned the emission profile to that of **1B**. The variable emission of the complex was attributed to intermolecular interactions that are modulated by grinding or exposing the material to solvent vapors.

Finally, **Cu(thzbzi)(dppnc)** (Figure 98) contains an unusual anionic diphos­phine-*nido*-car­borane ligand.^833^ As a powder this complex is a green-yellow emitter, with λ_PL_ of 547 nm, Φ_PL_ of 16%, and τ_PL_ of 26 μs. Similarly when doped at 5 wt% in PMMA the emission has λ_PL_ of 542 nm, Φ_PL_ of 10%, and τ_PL_ of 23 μs. The Δ*E*_ST_ was found to be 114 meV (powder) and 127 meV (in the doped film). The similar photophysical properties in these two media imply little aggregation in the powder form.

There are a small number of reported TADF Cu(I) complexes that can switch between cationic and neutral forms following protonation of one of the ligands (Figure 99). The first report of these switchable complexes included four neutral complexes containing (di)phosphine ligands and a pyridyltetrazolate ligand, **Cu(P^∧^P)(PyrTet)**, where (P^∧^P) = (PPh_3_)_2_, POP, xantphos, or Me_2_Xantphos. When the tetrazole of the PyrTet ligand is protonated, the complexes become charged **[Cu(P^∧^P)(PyrTetH)]BF_4_**.^834^ All eight complexes are green to yellow emitters (λ_PL_ = 510–569 nm), while the neutral complexes show more efficient, longer-lived emission (Φ_PL_ = 76–89% and τ_PL_ = 17.8–26.6 μs for the neutral complexes, compared to Φ_PL_ = 4–46% and τ_PL_ = 5.2–15.3 μs for the cationic complexes). The less efficient emission from the cationic complexes was attributed to a change in the nature of the emissive state (^1^MLCT for neutral and mixed ^1^MLCT/LLCT for charged) as well as vibrational quenching effects of the N-H bond of the protonated tetrazole ring.

**Figure 99:**
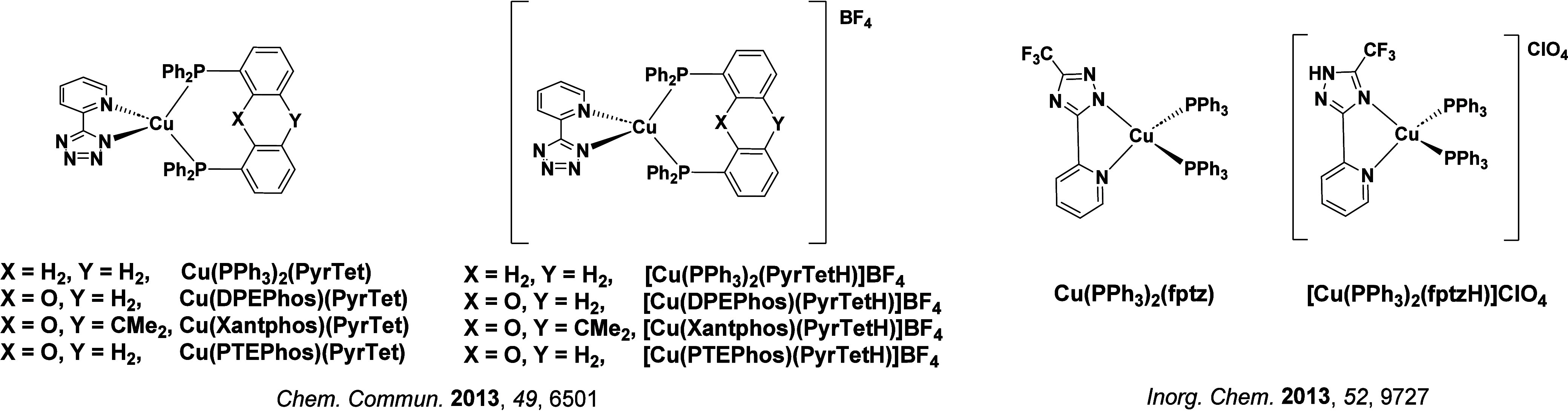
Tetrahedral copper(I) complexes that can switch between neutral and cationic forms having TADF properties.

Another pair of interconvertible complexes, **Cu(PPh_3_)_2_(fptz)** and **[Cu(PPh_3_)_2_(fptzH)]ClO_4_** (Figure 99), also show interesting photophysical properties.^835^ In this case conversion involves protonation and a ring inversion isomerism of the 1,2,4-triazole. Both complexes are emissive in solution and the solid state and moving from the cationic to the neutral complex results in a blue-shift in the solution-state emission but a red-shift in the solid-state emission. This change was attributed to the presence of the N-H bond raising the LUMO energy and blue-shifting the solution-state emission, while the more flexible structure of the neutral complex leads to greater excited-state relaxation and a lower energy excited state in the solid state. The contrasting electronic and geometric impact on the emission highlights the sensitivity of the photophysical properties of copper(I) complexes to the ligand environment.

TADF emission has also been reported for 3-coordinate trigonal planar copper(I) complexes, with selected examples shown in Figure 100. The use of bulky phosphine or carbene ligands is popular to restrict the pseudo Jahn-Teller Y-to-T excited-state distortion in 3-coordinate complexes, which contributes to non-radiative decay and ligand dissociation as in tetrahedral complexes.^836^ The first report of TADF trigonal copper complexes featured **(L_Me_)CuCl**, **(L_Me_)CuBr**, and **(L_Me_)CuI** (L_Me_ = dtpb = 1,2-bis­(*o*-di­tol­yl­phos­phino)­ben­zene), although these were misattributed as phosphorescent likely due to the exceptional device performance (EQE_max_ = 21.3%, λ_EL_ = 517 nm for the bromo complex) that predated the key early reports of all-organic TADF OLEDs.^757,837^ The photophysics of these three complexes and additional related complexes **(L_Et_)CuBr** and **(L_iPr_)CuBr** were later analyzed in greater detail and confirmed to arise from TADF.^837^ As powders all five complexes show bright sky-blue to green emission (λ_PL_ = 473–517 nm, Φ_PL_ = 38–95%, τ_PL_ = 4.6–8.9 μs). **(L_Et_)CuBr** and **(L_iPr_)CuBr** were used in green OLEDs (EQE_max_ = 22.5 and 18.6%, and λ_EL_ = 529 and 515 nm respectively). The related complexes **(L_Me_)Cu(SPh)** and **(L_iPr_)Cu(SPh)** with thiolates replacing the halido ligands are also TADF-active and have near unity Φ_PL_ as powders (Φ_PL_ = 95%).^838^ The emissive excited states in these materials were assigned to have LLCT character, in contrast to the MLCT states of **(L_Me_)CuBr**.^838^

**Figure 100:**
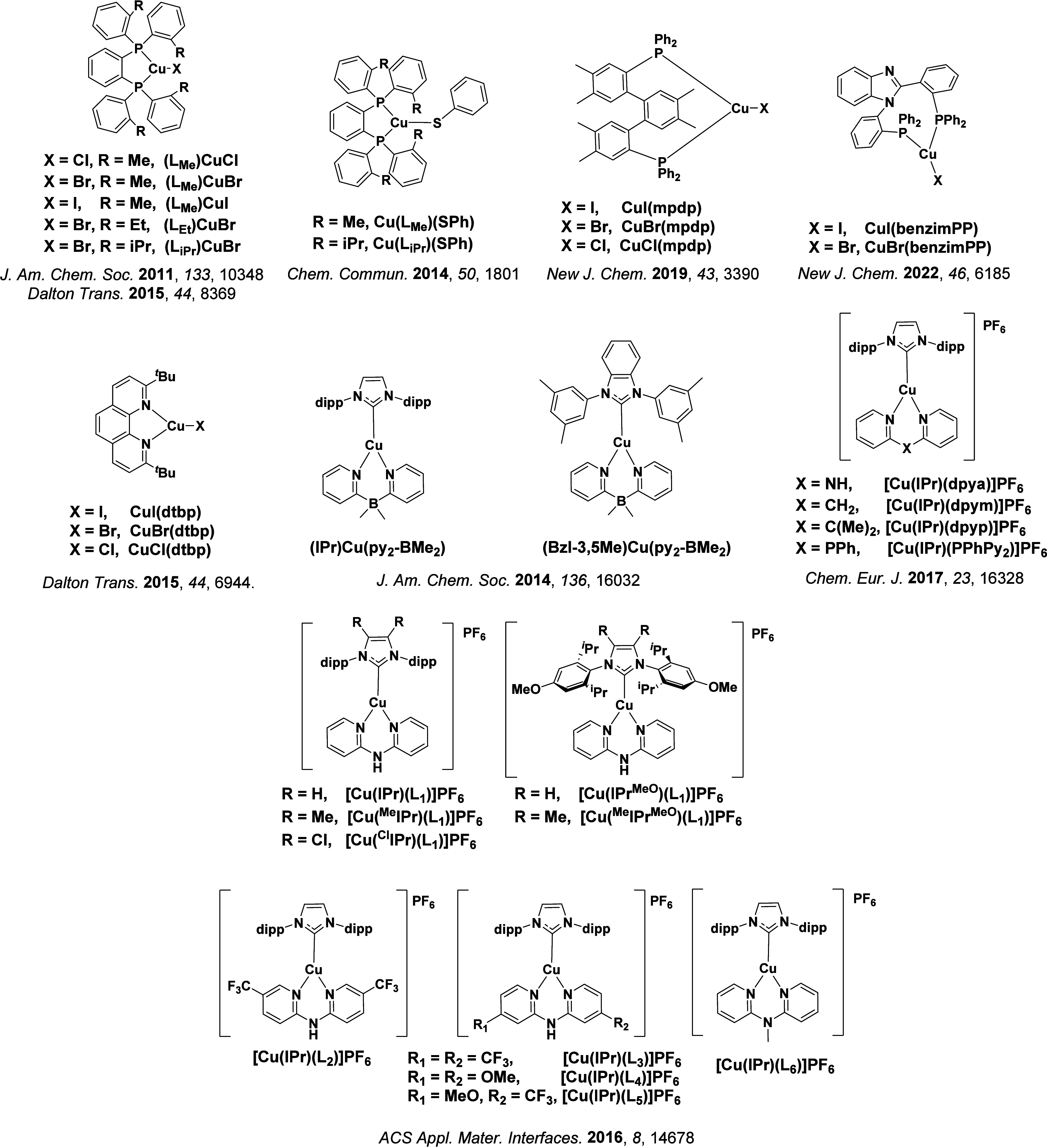
Trigonal planar copper(I) complexes having TADF properties.

Cu(P^∧^P)X complexes employing bulky diphosphine ligands also have been shown to emit by TADF, for instance in the family of **CuI(mpdp)**, **CuBr(mpdp)**, and **CuCl(mpdp)** (mpdp = hexa­meth­yl-bis­(di­phen­yl­phos­phino)-ter­phenyl, Figure 100).^839^ These complexes are only weakly emissive though (Φ_PL_ = 1–5.4%), and OLEDs with **CuI(mpdp)** showed an unsurprisingly low EQE_max_ of 0.26%. The use of an unusual benzimidazole-linked diphosphine ligand instead resulted in two highly emissive complexes **CuI(benzimPP)** and **CuBr(benzimPP)** (named **1** and **2** in the initial publication, Figure 100).^840^ These complexes are orange-red emitters as powders, that show bright emission and long lifetimes (λ_PL_ = 630 and 615 nm, Φ_PL_ = 65 and 72%, τ_PL_ = 143 and 228 μs, all respectively). Other trigonal planar copper(I) complexes using the bulky diimine ligand dtbp such as **CuX(dtbp)** (Figure 100) have been shown to exhibit TADF, albeit with low Φ_PL_ ≤ 15%.^841^

Over time the sterically bulky class of NHC ligands have become the most popular for 3-coordinate copper complexes. The first example, **(IPr)Cu(py_2_-BMe_2_)** (Figure 100), was initially reported as phosphorescent along with related complexes **(BzI-3,5Me)Cu(py_2_-BMe_2_)** and **(PzI-3,5Me)Cu(py_2_-BMe_2_)**.^842^ A subsequent study showed that **(IPr)Cu(py_2_-BMe_2_)** in fact emits by TADF, while **(BzI-3,5Me)Cu(py_2_-BMe_2_)** emits by phosphorescence.^764^ It was determined that the emission mechanism is controlled by the different steric demands of the aryl groups on the NHC ligands – 2,6-diisopropylphenyl (dipp) vs 3,5-dimethylphenyl (xylyl). The bulkier dipp groups in **(IPr)Cu(py_2_-BMe_2_)** locked the ligands in a co-planar orientation, while the less bulky xylyl groups in **(BzI-3,5Me)Cu(py_2_-BMe_2_)** resulted in a perpendicular conformation. DFT calculations revealed that the Δ*E*_ST_ is smaller in the co-planar ligand orientation (67 meV, enabling TADF) and higher when the ligands adopt an orthogonal conformation (459 meV), accounting for the different emission mechanisms observed in the complexes.

In addition to the neutral 3-coordinate copper(I) complexes described above, there are a number of cationic 3-coordinate copper(I) complexes that show TADF. Elie *et al.* documented the first examples of cationic trigonal copper(I) complexes in the structural form of [Cu(N^∧^N)(NHC)]PF_6_.^843^ The 13 reported examples contained various combinations of 5 different NHC ligands and 6 different dipyridylamine ligands, and are blue to green emitters as powders (λ_PL_ = 455 to 521 nm). Varying the electronics of the NHC ligand had minimal impact on the emission color (λ_PL_ = 463 to 481 nm), while the emission color was sensitive to substitution of the pyridyl rings of the dipyridylamine ligands. Electron-withdrawing CF_3_ groups on the pyridyl rings stabilized the LUMO of the complexes, red-shifting the emission for **[Cu(IPr)(L2)]PF_6_** and **[Cu(IPr)(L3)]PF_6_** above 505 nm (Figure 100), while electron-donating OMe groups stabilized the LUMO of **[Cu(IPr)(L4)]PF_6_**, blue-shifting the emission to 420 nm. The Φ_PL_ varied with the rigidity of the molecule, with Φ_PL_ as high as 64% for **[Cu(^Me^­IPr^MeO^)­(L_1_)]PF_6_**.

The same group later investigated a similar series of complexes by varying the bridge between the two pyridyl rings of the diimine ligand.^844^ Of the compounds studied, powders of **[Cu(IPr)(dpyp)]PF_6_** and **[Cu(IPr)(Pphpy_2_)]PF_6_** (Figure 100) have the highest Φ_PL_ as these contained the most rigid bridges between the pyridyl rings (isopropyl and phenylphosphinyl, Φ_PL_ = 73 and 86% respectively). These two compounds emit in the blue (λ_PL_ = 474 nm) and green (λ_PL_ = 503 nm), respectively.

2017 marked the first reports of linear copper(I) complexes that emit via TADF. Among these, linear carbene-metal-amide (CMA) complexes (including previously mentioned copper complex **CMA2**) showed the most desirable emission properties for OLED applications.^845^ CMA complexes have consequently been the focus of an extensive research effort for the last 5 years, with notable early Cu containing materials summarized here. Aside from CMA complexes, a small number of other noteworthy linear copper(I) complexes emitting by TADF have also been reported (Figure 101).

**Figure 101:**
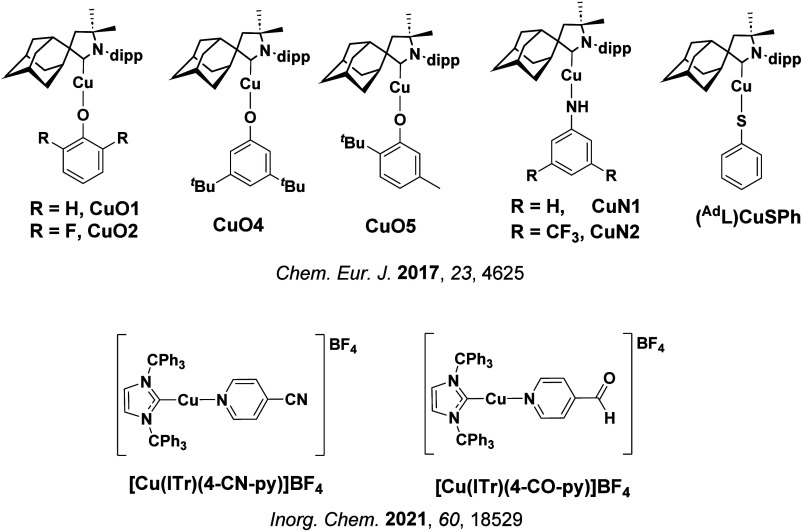
Linear copper(I) complexes having TADF properties.

Romanov *et al*. made important early contributions to the research field by reporting copper complexes based on the adamantyl-substituted Cyclic Alkyl Amino Carbene (CAAC) ligand ^Ad^L.^845^ While TADF is not explicitly mentioned in the paper, a number of the complexes, namely **CuO1-CuO5**, **(^Ad^L)CuSPh**, **CuN1**, and **CuN2** (Figure 101) showed efficient emission and Φ_PL_ up to 62% that is characterized by biexponential decay kinetics with associated prompt (τ_p_ = 2 to 9.7 ns) and delayed emission (τ_d_ = 0.29 to 0.66 μs) consistent with TADF. Further improvements in TADF performance have also been observed for CMA complexes employing other metal centers, particularly Au, and are summarized later in this section.

The sterically demanding trityl groups of the NHC ligand (ITr) were used in a series of linear copper(I) complexes with pyridyl and quinoline ligands. Of these complexes only two exhibited TADF, **[Cu(ITr)­(4-CN-py)]­BF_4_** and **[Cu(ITr)­(4-CO-py)]­BF_4_** (Figure 101).^846^ In 10 wt% doped films in PMMA the complexes emit with λ_PL_ of 525 and 545 nm, have modest Φ_PL_ of 25 and 12%, and short τ_PL_ of 2.1 and 3.2 μs, all respectively.

Instead of co-doping a copper(I) complexes with a host material in the EML of the OLED, Thompson and co-workers pioneered an approach of co-depositing an OLED host that could coordinate directly with separate copper(I) precursors to form the emissive complex *in-situ*.^847^ The initial report involved co-depositing **mCPy** and **CuI** to produce a green phosphorescent film (λ_PL_ = 528 nm, Φ_PL_ = 64% and τ_PL_ = 10.7 μs). The OLED showed an EQE_max_ of only 4.4%; however, a number of the films were shown to be TADF-active (Figure 102).

**Figure 102:**
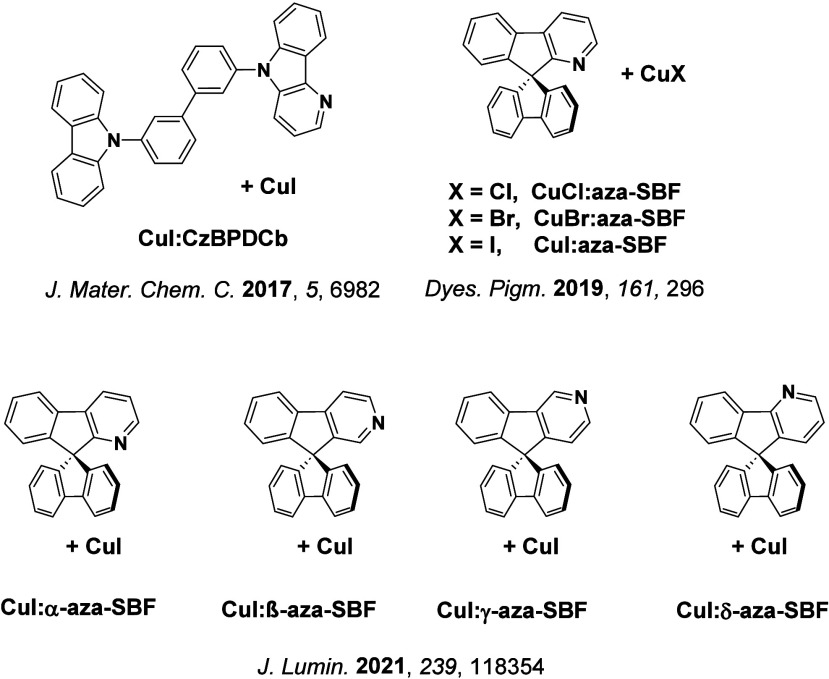
Co-deposited copper(I) halide/ligand films having TADF properties.

Wang *et al*. similarly prepared co-deposited films of **CuI** with two different carboline containing host materials.^848^ One of the films, **CuI:​CzBPDCb** (Figure 102) showed green TADF (λ_PL_ = 520 nm, Φ_PL_ = 22%, τ_d_ = 1.05 μs, Δ*E*_ST_ = 120 meV). Many OLEDs were fabricated using different ratios of **CuI:​CzBPDCb**, and the best device with 6 mol% **CuI** in the EML showed an EQE_max_ = 17.5%.^848^

A spiro-bifluorene compound with a coordinating nitrogen in one of the aryl rings, **aza-SBF**, was similarly co-deposited with **CuCl**, **CuBr**, and **CuI**.^849^ **CuCl:​aza-SBF**, **CuBr:​aza-SBF**, and **CuI:​aza-SBF** (Figure 102) all showed green TADF with doping ratios of between 5–11 mol% of CuX. There is a small blue-shift in the λ_PL_ as the halide increased in mass (λ_PL_ = 550, 537, 526 nm for the Cl, Br and I films, respectively), while all complexes showed similar delayed emission lifetimes (τ_d_ = 3.9 to 5.8 μs). The Φ_PL_ varied significantly as a function of halide ligands and doping concentration, with the highest Φ_PL_ being 78% for the 7 mol% **CuBr:​aza-SBF** film. The highest performing device used 7 mol% **CuBr:​aza-SBF** (EQE_max/100_ = 13.6/13.6%). A subsequent study used aza-SBF analogues with the nitrogen atom in 4 different positions of the aryl ring to produce four different co-deposited films **CuI:​α-aza-SBF**, **CuI:​β-aza-SBF**, **CuI:​γ-aza-SBF**, and **CuI:​δ-aza-SBF**.^850^ The co-deposited films showed yellow to red emission (λ_PL_ = 550 to 625 nm) with a wide range of range Φ_PL_ (4.6 to 92.2%), the most efficient emitter being the yellow (λ_PL_ = 550 nm) emitting **CuI:​δ-aza-SBF**. The emission at room temperature of all complexes was determined to be a mixture of TADF and phosphorescence (ranging from 20% phosphorescence contribution for **CuI:​α-aza-SBF** to 58% for **CuI:​γ-aza-SBF**). The most efficient OLED of the series used **CuI(8 mol%):​δ-aza-SBF** (λ_EL_ = 540 nm) and achieved EQE_max_ of 16.8%.^850^

Although the concept of TADF in simpler copper complexes has been observed for decades, the first TADF OLED in 2007 used a dinuclear copper complex with bridging iodo ligands, **[Cu(μ-I)dppb]_2_** (Figure 103).^75^ As a powder **[Cu(μ-I)dppb]_2_** showed two delayed fluorescence components with τ_PL_ of 1.5 and 4.0 μs, and has a small Δ*E*_ST_ of 90 meV. Devices showed low EQE_max_ of 4.8% at λ_EL_ of 560 nm. The dinuclear structure nonetheless prevents the formation of a formally d^9^ copper center and large changes to the geometry in the excited, with the electron density being delocalized over both centersstate.^73,851^ Similar to the use of bulky ligands, this is a widely exploited tactic to produce highly efficient TADF emitting copper complexes. Selected dinuclear copper(I) complexes shown in Figure 103 are discussed below, with additional polynuclear Cu complexes in Figure 104.

**Figure 103:**
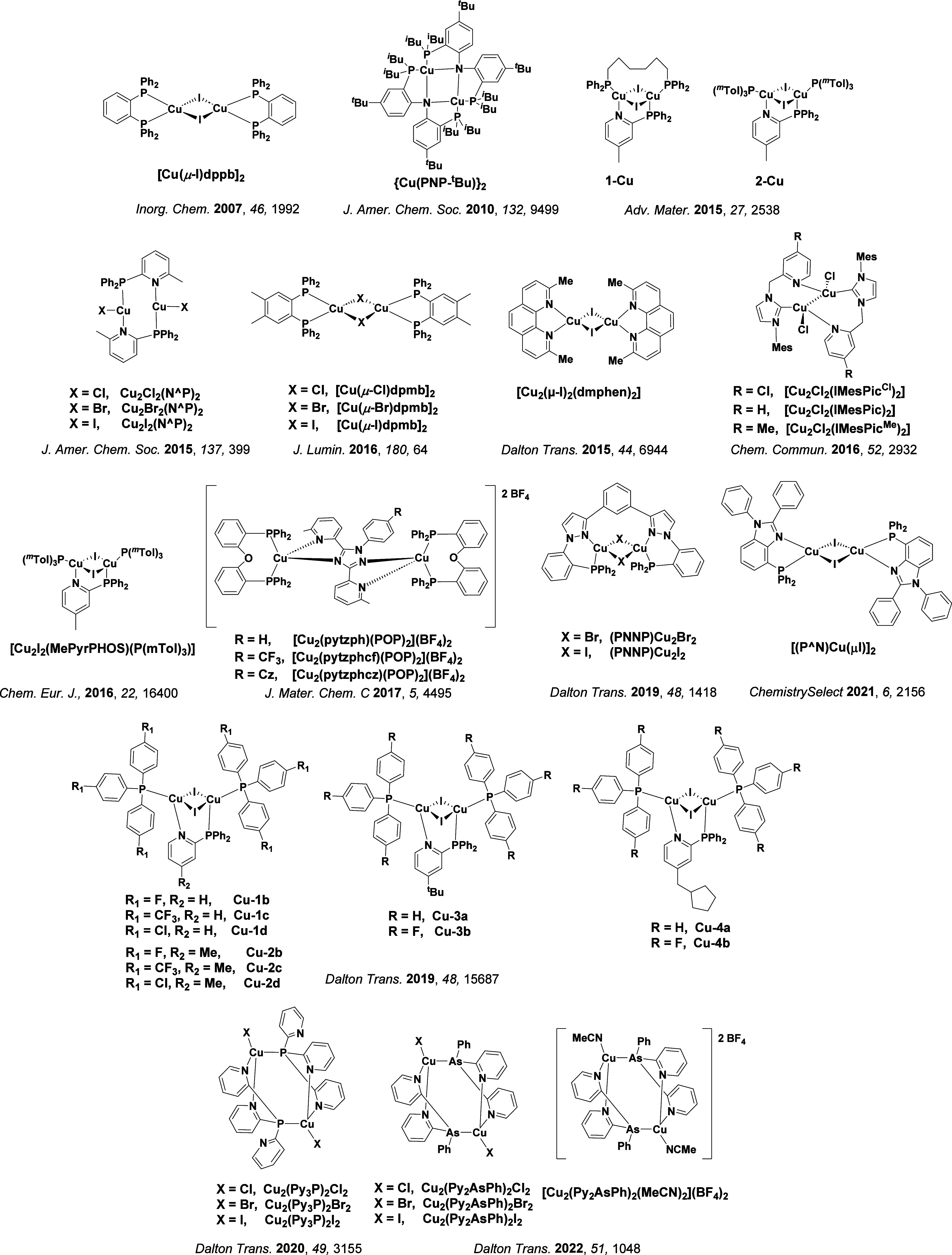
Dinuclear copper(I) complexes having TADF properties.

**Figure 104:**
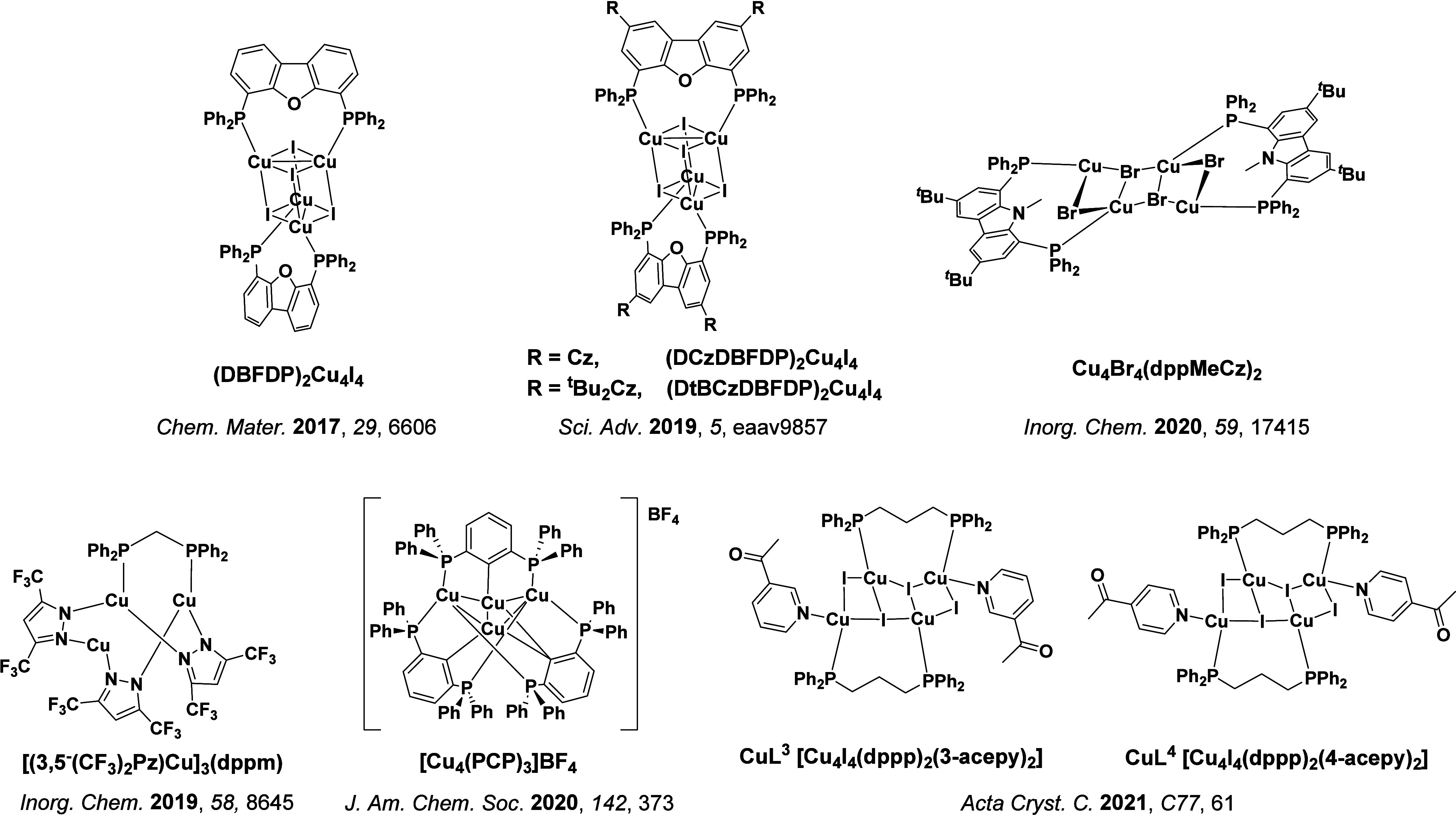
Larger copper(I) clusters having TADF properties.

The first copper OLED to surpass the 5% EQE limit of fluorescent devices was another dinuclear emitter, **[Cu(PNP-tBu)]_2_** (Figure 103).^73^ In 2-MeTHF the complex emits at λ_PL_ ≈ 510 nm, has a Φ_PL_ of 57%, a τ_PL_ of 11.5 μs, and a Δ*E*_ST_ of 100 meV. The OLED showed what was at the time a remarkable EQE_max_ of 16.1%, similar to the efficiencies of iridium(III)-based OLEDs. Volz *et al*. introduced additional dinuclear complexes **1-Cu** and **2-Cu**,^851^ the former of which showed TADF in doped films (λ_PL_ = 540 nm, Φ_PL_ = 92%, τ_d_ = 3.2 μs, and Δ*E*_ST_ = 90 meV in 30 wt% doped PYD2 films). The solution-processed OLED with **1-Cu** showed an EQE_max_ of 23% and a λ_EL_ of 555 nm. The dinuclear copper(I) complex **Cu_2_Cl_2_(N^∧^P)_2_** and analogues **Cu_2_Br_2_(N^∧^P)_2_** and **Cu_2_I_2_(N^∧^P)_2_** all have high Φ_PL_ and microsecond-long τ_PL_ (Φ_PL_ = 52 to 92%, τ_PL_ = 7.3 to 12.4 μs).^763^ Detailed photophysical studies of **Cu_2_Cl_2_(N^∧^P)_2_** show that it emits from both S_1_ and T_1_ states with 80% TADF contribution and the remaining 20% being phosphorescence.

A family of dinuclear clusters containing the bidentate ligand 1,2-bis(diphenylphosphino)-4,5-dimethylbenzene (dpmb), **[Cu(μ-Cl)dpmb]_2,_ [Cu(μ-Br)dpmb]_2_**, and **[Cu(μ-I)dpmb]_2_** (Figure 103) are all green emitters as powders (λ_PL_ = 498–527 nm, Φ_PL_ = 28–32%, τ_PL_ = 2.5–12.5 μs), with moderate Δ*E*_ST_ ranging between 120 to 140 meV.^852^ The green OLEDs using mCP as the host showed EQE_max_ ranging from 7.3 to 10.1%. The device with **[Cu(μ-Cl)dpmb]_2_** in particular showed an EQE_max_ of 8.3%, and had moderate efficiency roll-off (EQE_1000_ = 4.9%) with CIE coordinates of (0.38, 0.51). The dmp-based dinuclear complex **[Cu_2_(μ-I)_2_(dmp)_2_]** showed very complicated photophysics owing to contributions from both TADF and phosphorescence (λ_PL_ = 667 nm, Φ_PL_ = 18%, τ_PL_ = 6.4 μs).^841^

NHC ligand-based dinuclear complexes **[Cu_2_Cl_2_(IMesPic^Cl^)_2_]**, **[Cu_2_Cl_2_(IMesPic^H^)_2_]**, and **[Cu_2_Cl_2_(IMesPic^Me^)_2_]** all show TADF (Figure 103).^853^ These complexes emit at λ_PL_ = 520–550 nm, have moderate Φ_PL_ of 49 to 68%, and similar τ_PL_ of 9.2 to 11.0 μs associated with similar Δ*E*_ST_ of between 78 and 120 meV. Interestingly, the very closely related complex **[Cu_2_Cl_2_(IMesPic^OMe^)_2_]** is phosphorescent, highlighting that subtle changes in the ligand can completely change the emission mechanism of this class of complex.^853^

Wallesch *et al*. reported the complex **[Cu_2_I_2_(MePyrPHOS)(P(*m*Tol)_3_]**.^854^ This complex is a yellow-green emitter (λ_PL_ = 550 nm as a powder) with a Φ_PL_ of 75% as a powder and 56% for the 50 wt% doped film in PMMA. Solution-processed OLEDs were fabricated by either spin coating or inkjet printing. The best performance was achieved with a spin-coated device and showed an EQE_max_ = 11.4%.^854^

**[Cu_2_(pytzph)­(POP)_2_]­(BF_4_)_2_**, **[Cu_2_(pytzphcf)­(POP)_2_]­(BF_4_)_2_**, and **[Cu_2_(pytzphcz)­(POP)_2_]­(BF_4_)_2_** all contain a bridging dipyridyl-1,2,4-triazole ligand (Figure 103).^855^ The complexes emit at λ_PL_ = 503–519 nm, yet have distinct Φ_PL_ of between 29 and 79%, τ_PL_ ranging from 5.5 to 16 μs, and Δ*E*_ST_ of between 89 and 132 meV. The most efficient solution-processed OLEDs employed the complex **[Cu_2_(pytzphcz)­(POP)_2_]­(BF_4_)_2_**, which has the highest Φ_PL_ and showed an EQE_max_ of 8.3% and minimal roll-off (EQE_100_ = 8.1%) at CIE coordinates of (0.29, 0.53).

The Cu_2_X_2_ copper halide core is supported by a single tetradentate ligand in complexes **(PNNP)Cu_2_I_2_** and **(PNNP)Cu_2_Br_2_** (Figure 103).^856^ The phenyl-bridged tetradentate ligand increases the rigidity of these complexes, reducing non-radiative decay by hindering excited-state distortions.^766,851^ **(PNNP)Cu_2_I_2_** and **(PNNP)Cu_2_Br_2_** have similar emission properties as powders, with **(PNNP)Cu_2_I_2_** emitting at a λ_PL_ of 494 nm with a Φ_PL_ of 42%, and τ_PL_ of 8.8 μs. **(PNNP)Cu_2_Br_2_** emits at a slightly red-shifted at λ_PL_ of 517 nm and a slightly brighter Φ_PL_ of 58%, and has a τ_PL_ of 13 μs. The complexes also have similar Δ*E*_ST_ of 90 and 110 meV, respectively. A similar coordination environment was achieved with two unlinked ligands around the same copper halide core in **[(P^∧^N)Cu(μI)]_2_** using an unusual diphen­yl­phos­phino­benz­imida­zole ligand.^857^ The complex emits at λ_PL_ of 585 nm with a Φ_PL_ of 37% and a τ_PL_ of 5.85 μs as a powder. Related complexes with methoxy groups at the 2-position of the phenyl ring of the benzimidazole were instead found to be phosphorescent. The solution-processed OLED with **[(P^∧^N)Cu(μI)]_2_** showed an EQE_max_ of 3.0%.

Busch *et al.* synthesized a family of ten dinuclear copper(I) complexes containing bridging 2-phosphinopyridyl ligands.^858^ The complexes **Cu-1b** through **d**, **Cu-2b** through **d**, **Cu-3a** and **b**, and **Cu-4a** and **b** (Figure 103) are all bright green to yellow emitters as powders (λ_PL_ = 519 to 549 nm, Φ_PL_ = 70 to 93%, τ_PL_ = 5.5 to 10.2 μs). These results are consistent with the properties of previously reported complexes using pyridylphosphine bridging ligands,^851,854^ and also show very high solubility in organic solvents due to the presence of the fluorinated and alkyl groups on the complexes, which makes them promising materials for solution-processed OLEDs.

A series of three further complexes with pyridyl phosphine ligands, **[Cu_2_(Py_3_P)_2_X_2_] (X = Cl, Br, I)** were prepared by mechanochemical synthesis.^771^ These complexes are bright green emitters at room temperature, with the emission attributed to mixed phosphorescence and TADF. The trend in contributions of the two processes across the halide series is unusual, with more phosphorescence observed for the Cl complex (73%), while the least phosphorescence is observed for the I complex (39%). Normally, the increased SOC of the heavier halogens increases the radiative rate of the formally spin-forbidden phosphorescence.^766,856^ However, in these complexes Δ*E*_ST_ also decreases with increasing halogen mass (from 186 to 155 to 124 meV) and the resulting increase in *k*_RISC_ for the complexes with heavier halogen atoms outweighs any increase in phosphorescence rate due to enhanced SOC. In an effort increase the radiative rate and SOC of these dinuclear copper(I) complexes, a series of four similar complexes containing arsine ligands, **[Cu_2_(Py_2_AsPh)_2_X_2_]** (X = Cl, Br, I)^859^ and **[Cu_2_(Py_2_AsPh)_2_(MeCN)_2_](BF_4_)_2_** was prepared.^860^ The three As-containing complexes are all bright green emitters in the solid state (λ_PL_ = 500 to 530 nm, Φ_PL_ = 22 to 50%, τ_PL_ = 2.0 to 9.0 μs), with emission originating from TADF and phosphorescence. Due to the larger SOC, the emission lifetimes of the arsenic complexes are shorter than their isostructural phosphine analogues with similar photoluminescence quantum yields (τ_PL_ = 5 to 33 μs and Φ_PL_ = 51–55%).^860^

Beyond dinuclear copper(I) complexes, there are a number of larger copper clusters reported to display TADF emission. Selected examples of tri- and tetra-nuclear complexes are shown in Figure 104. At yet larger cluster sizes there are a number reported copper clusters that are luminescent; however, their electronic character moves away from molecular descriptions and are beyond the scope of this review.^787,789^ While there is an extensive body of work on tetranuclear copper clusters that display strong luminescence,^861,862^ it wasn’t until 2017 that the first TADF emitting cluster was reported.^767^ Most of these clusters emit from a cluster-centered (^3^CC) excited state as phosphorescence. To enable TADF emitting tetranuclear cluster, ligands that can electronically couple to the core of the cluster to delocalize the electron density across the ligands are typically required.^863^

The first tetranuclear copper(I) complex that showed TADF, **(DBFDP)_2_Cu_4_I_4_**, contained two diphosphine ligands to stabilize a Cu_4_I_4_ cubic core (Figure 104).^767^ The complex is a weak blue-green emitter in DCM (λ_PL_ = 491 nm, Φ_PL_ = 5%, τ_PL_ = 1.9 μs, Δ*E*_ST_ = 160 meV), and analysis of temperature-dependent emission shows that the dominant contribution is TADF (80%) at room temperature. Solution-processed bilayer OLEDs showed dual emission from the ^3^CC and a higher energy^1^ (M-X)LCT state. The white OLED showed very low efficiency (EQE_max_ = 0.78%) as expected for the low Φ_PL_ of the complex. Modification of the diphosphine ligand with carbazole groups to form donor-acceptor ligands DCzDBFDP and DtBCzDBFDP resulted in complexes **(DCzDBFDP)_2_Cu_4_I_4_** and **(DtBCzBFDP)_2_Cu_4_I_4_**.^863^ Compared to the original complex without carbazole groups **(DBFDP)_2_Cu_4_I_4_**, the emission is blue-shifted (λ_PL_ = 480 nm) and narrower (FWHM is reduced from 95 to 60 nm). Suppression of the ^3^CC excited state in the two carbazole-containing complexes leads to an increased Φ_PL_ from 5 to 46–65% and reduced Δ*E*_ST_ from 160 to 70–100 meV. Non-doped solution-processed OLEDs showed EQE_max_ of up to 7.9% at CIE coordinates of (0.22, 0.43).

Moving to emitters with yet higher Cu content, a TADF tri-nuclear copper(I) complex has been reported with a diphosphine ligand (dppm) completing the coordination spheres of a trimeric pyrazolate core in **(dppm)­[(3,5-(CF_3_)_2_Pz)Cu]_3_** (Figure 104).^864^ The complex is a green emitter as a powder (λ_PL_ = 514 nm, Φ_PL_ = 41%, τ_PL_ = 32.7 μs) and has a Δ*E*_ST_ = 131 meV. The emissive excited state is predominantly ^1^MLCT (metal to dppm ligand), highlighting the importance of the diphosphine ligand in generating TADF emission from the Cu_3_Pz_3_ core.

Interesting vapochromic emission was observed in a tetranuclear Cu_4_Br_4_-based complex with carbazole-bridged diphosphine ligands, **Cu_4_Br_4_­(dppMeCz)_2_** (Figure 104).^865^ Two polymorphs of the cluster, **1G** and **1Y**, are either green (λ_PL_ = 512 nm, Φ_PL_ = 8%, τ_PL_ = 8.9 and 295 μs) or yellow (λ_PL_ = 550 nm, Φ_PL_ = 13.8%, τ_PL_ = 6.7 and 473 μs) emitters, respectively. In both complexes the Cu_4_Br_4_ core has the same geometry, with the difference between the polymorphs due only to the orientation of the ligand relative to the cores. Crystals of the complex can be induced to change polymorphs by exposure of **1G** to hexane vapor or **1Y** to acetonitrile vapor. The emission of **1G** was determined to be phosphorescence, while **1Y** had a strong TADF component to the emission.

An organometallic cationic Cu_4_ cluster has been prepared with diphosphine ligands that also bond to the Cu atoms through a C_aryl_-Cu bond to form **[Cu_4_(PCP)_3_]BAr^F^_4_** (Figure 104).^866^ The cluster is a bright green emitter in both solution and as a powder (λ_PL_ = 518 nm, Φ_PL_ = 50%, τ_PL_ = 9.8 μs as a powder) with moderately narrow FWHM of 58 nm attributed to the rigid nature of the cluster. The Δ*E*_ST_ is 72 meV and TADF was determined to be responsible for 92% of the emission at room temperature. The solution-processed OLED showed an EQE_max_ = 11.2% at CIE coordinates of (0.305, 0.637), with moderate roll-off (EQE_1000_ ≈ 8.5%).

The crystals of two isomeric clusters with diphosphine and pyridine ligands, **[Cu_4_I_4_­(dppp)_2_­(3-acepy)_2_] (Cu*L_3_*)** and **[Cu_4_I_4_­(dppp)_2_­(4-acepy)_2_] (Cu*L_4_***) (Figure 104) emit TADF with Δ*E*_ST_ = 35 meV for both complexes).^867^ Both emitters show yellow emission with moderate efficiency (λ_PL_ of 562 and 580 nm, Φ_PL_ of 26 and 30%), and short τ_PL_ of 2.5 and 10.8 μs, respectively.

### 9.3 Silver

Silver(I) complexes, containing another d^10^ metal, have also gained interest recently due to their ability to engender TADF characteristics. SOC is expected to increase moving down group 11 from copper to silver, opening the potential for enhanced T_1_ → S_0_ transitions that can compete with RISC for this precious metal. This enhanced SOC may explain why only a few Ag(I) TADF emitters have been reported compared to analogous copper complexes. Excited states in Ag(I) complexes are also primarily ligand-centered rather than MLCT, resulting in large Δ*E*_ST_ and phosphorescence facilitated by the increased SOC unless the ligands are carefully designed. Compound **[Ag(dppb)(PS)]** ^756^ is the first reported TADF silver complex, although emission was demonstrated to be a mixture of TADF and phosphorescence (Figure 105). Similar to the mixed emission mechanisms in copper complexes discussed above, TADF is dominant at RT while phosphorescence prevails at lower temperatures. Complex **[Ag(dppb)(PS)]** has a Φ_PL_ of 32% and shows biexponential decay kinetics with τ_PL_ of 0.6 μs and 2.2 μs, likely associated with delayed fluorescence and phosphorescence. Owing to its poor solubility, no devices were reported. Numerous three- and four-coordinate Ag(I) complexes have since been reported that exhibit TADF. Selected examples are shown in Figure 105, and generally emit in the blue-to-green with moderate to good Φ_PL_ in the solid state (decreasing in solution). We note that very few of these complexes have been used as emitters in OLEDs.

**Figure 105:**
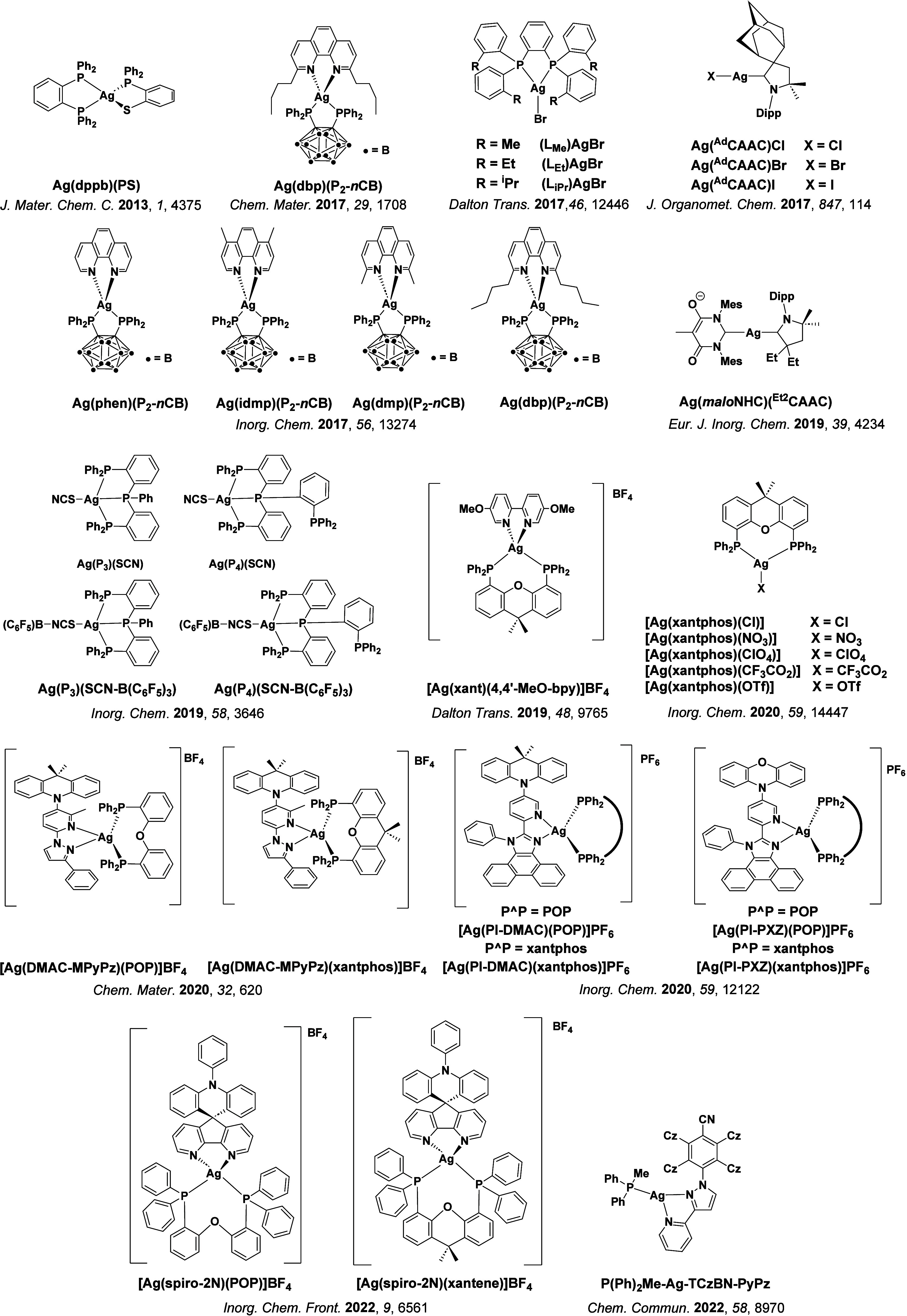
Chemical structures of monometallic silver(I) complexes having TADF properties. Cz = carbazole.

A family of complexes **Ag(phen)­(P_2_-*n*CB)**, **Ag(idmp)­(P_2_-*n*CB)**, **Ag(dmp)­(P_2_-*n*CB)**, and **Ag(dbp)­(P_2_-*n*CB)** (Figure 105) containing carborane ligands emit with λ_PL_ between 526 and 575 nm, and have powder Φ_PL_ of between 36 and 100%.^868,869^ The complexes with the higher Φ_PL_ use diimine ligands with increased steric bulk about the silver center, which reduces the capacity for the complex to distort its geometry in the excited state.

Complex **[Ag(xant)­(4,4′-MeO-bpy)]­BF_4_** emits at λ_PL_ of 493 nm and has a Φ_PL_ of 57.1% in DCM solution.^870^ This is an increase in Φ_PL_ over the analogous copper complex (*vide supra*), which was shown in theoretical studies to be due to the presence of additional low-lying non-emissive excited states in the copper complex.

A pair of silver complexes **Ag(P_3_)(SCN)** and **Ag(P_4_)(SCN)** (Figure 105) containing tridentate phosphine ligands along with an isothiocyanate ligand have been prepared by Koshevoy and co-workers.^830^ **Ag(P_3_)(SCN)** emits in the green (λ_PL_ = 538 nm, Φ_PL_ = 32%) while **Ag(P_4_)(SCN)** showed interesting behavior with different crystalline forms (polymorphs and solvates) giving a range of emission colors (λ_PL_ from 525 to 630 nm) and Φ_PL_ from 19 to 44%. Reaction of these complexes with B(C_6_F_5_)_3_ produced the isothiocyanatoborate complexes **Ag(P_3_)­(SCN-B(C_6_F_5_)_3_)** and **Ag(P_4_)­(SCN-B(C_6_F_5_)_3_)**, which resulted in a blue-shift of the emission and a drop in the powder Φ_PL_. The emission of the powders is TADF in nature, with Δ*E*_ST_ of 200 meV for **Ag(P_3_)­(SCN-B(C_6_F_5_)_3_)** and 90 meV for **Ag(P_4_)­(SCN-B(C_6_F_5_)_3_)**.

Compounds **(L_Me_)AgBr**, **(L_Et_)AgBr**, and **(L_iPr_)AgBr** (Figure 105), containing bidentate diphosphine ligands, emit in the sky-blue in both DCM solution and as powders.^871^ The emission maxima of the solutions ranged from 492 to 499 nm with Φ_PL_ of between 20 and 32%, while the powder emission was blue-shifted to 463 to 487 nm and considerably brighter with Φ_PL_ increasing to 56 to 98%. In both solution and in the solid state the Φ_PL_ increased with steric bulk of the P^∧^P ligand.

A number of interesting 4-coordinate silver(I) complexes exist where coordination of a silver(I)-diphosphine moiety to a separate donor-acceptor ligand turns on TADF from the ligand.^760,872^ **Ag(DMAC-MPyPz)­(POP)** and **Ag(DMAC-MPyPz)­(xant)** ^760^ (Figure 105) emit at λ_PL_ of 502 and 500 nm in DCM, which are blue-shifted to 472 and 471 nm in 15 wt% doped films in PMMA, with Φ_PL_ of up to 60% in solution and 99% in the same films, all respectively. The TADF nature of the emission was supported by the magnitude of the emission lifetime (τ_d_ = 6.3 and 6.5 μs, in PMMA) and a significant reduction in the Δ*E*_ST_ (from 470 meV for the ligand to 170 meV and 150 meV in the complexes, respectively). Similar materials with D-A TADF ligands **Ag(PI-DMAC)­(POP)**, **Ag(PI-DMAC)­(xant)¸** **Ag(PI-PXZ)­(POP)**, and **Ag(PI-PXZ)­(xant)** ^872^ all showed green to yellow emission in DCM (λ_PL_ = 502 to 533 nm) that red-shifted in 10 wt% doped films in DPEPO. Since the TADF in these compounds is ligand centered, there are few relevant geometric changes about the Ag(I) center and thus this is no longer a major contributor to non-radiative decay.^760^ Solution-processed OLEDs with **Ag(PI-PXZ)­(POP)** showed an EQE_max_ of 8.76% at CIE coordinates of (0.45, 0.62).

It has been demonstrated that *k*_RISC_ can be dramatically increased when an already TADF-active donor-acceptor compound is coordinated to silver ions.^873^ Both the free ligand **TCzBN-PyPz** and the silver complex **P(Ph)_2_Me-Ag-TCzBN-PyPz** (Figure 105) show similar green emission (λ_PL_ = 536 and 522 nm, respectively), with the free ligand showing higher Φ_PL_ (36 and 29%). The major difference in photophysics is in the τ_d_, which decreased from 2074 μs in the ligand to 0.59 μs in the complex. This change is largely explained by the relative magnitudes of Δ*E*_ST_ (160 meV for the ligand and 0.03 for the complex) as well as the much enhanced SOC from the Ag ion.^874^ Similar in concept, **[Ag(spiro-2N)­(POP)]­BF_4_** and **[Ag(spiro-2N)­(xanthene)]­BF_4_** contain a spiro-type TADF emitter coordinated to Ag(I).^875^ In 10 wt% doped films in PMMA both complexes exhibit strong blue-green emission, with λ_PL_ of 486 nm and Φ_PL_ of 65% for **[Ag(spiro-2N)­(POP)]­BF_4_** and 495 nm and 74% for **[Ag(spiro-2N)­(xanthene)]­BF_4_**. DFT calculations revealed that the LUMO energy of the free ligand is considerably stabilized upon coordination with the Ag(I) center, with a concomitant reduction in calculated Δ*E*_ST_ from 270 meV in the free ligand to 10 meV for **[Ag(spiro-2N)­(POP)]­BF_4_** and 13 meV for **[Ag(spiro-2N)­(xanthene)]­BF_4_**, in good agreement with the experimental Δ*E*_ST_ of 90 and 50 meV, respectively. As with the previous study, the lifetime of the free ligand is significantly shortened upon coordination to the metal, accelerating from 539 ms (1 wt% doped phenyl benzoate film) in and also showing an afterglow of 5 s, to just 5.3 and 5.8 μs for **[Ag(spiro-2N)­(POP)]­BF_4_** and **[Ag(spiro-2N)­(xanthene)]­BF_4_**, respectively, in 10 wt% doped PMMA.

Several groups have investigated linear Ag(I) carbene complexes, analogues to corresponding high-performance Cu(I) CMAs. The first examples of these were a series of carbene-halide complexes **Ag(^Ad^CAAC)X** (X = Cl, Br, I, Figure 105).^876^ These complexes emit at λ_PL_ of 432 to 443 nm and have poor Φ_PL_ of 0.5 to 5% in the solid state, and were not emissive in solution. The TADF behavior of these complexes is supported by the observation of dual photoluminescence consisting of a prompt ns emission and long-lived microsecond emission. A related carbene ligand ^Et2^CAAC was later used in combination with an unusual anionic carbene ligand *malo*NHC in the zwitterionic complex **Ag(*malo*NHC)­(^Et2^CAAC)**. This complexes was weakly blue emissive (Φ_PL_ < 1%) as both a powder and a 5 wt% doped polystyrene film.^877^ Beyond these complexes, a series of highly emissive linear silver CMA complexes are discussed together with other coinage metal CMA complexes further below.

While the previous examples of Ag(I) complexes are all mononuclear, there are also a range of reported dinuclear (Figure 106) or larger (Figure 107) TADF silver complexes. The interaction between the metal centers in these multinuclear silver(I) complexes can destabilize the antibonding d-orbitals of the silver(I) ions, thus increasing the energy of metal-centered (MC) states to such an extent that the emissive MLCT state is the lowest in energy.^878,879^ The first reported multinuclear silver(I) TADF emitters were a series of mixed phosphine/halide complexes with a bridging 1,2,4,5-tetrakis(diphenylphosphino)benzene ligand, **[Ag(PPh_3_)(X)]_2_­(tpbz)** (Figure 106).^880^ These complexes were not emissive in the solution, but showed green emission (λ_PL_ = 517–531 nm) with Φ_PL_ of up to 40% and τ_d_ of between 4.0–5.3 μs in 5 wt% doped films in PMMA. The emission as powders is blue-shifted (λ_PL_ = 471–495 nm) and the Φ_PL_ are much higher (Φ_PL_ = 74–98%). Interestingly there was little impact on the τ_PL_ when moving to iodide ions despite the expected increased SOC (X=Cl, τ_PL_ = 3.0 μs; X=I, τ_PL_ = 2.5 μs). As powders, all complexes have an Δ*E*_ST_ < 200 meV. The same bridging ligand was used to prepare a dinuclear complex with the silver atoms supported by a diphosphinocarborane ligand **[Ag_2_(tpbz)­(P_2_-*n*CB)_2_]**.^881^ This complex emits strongly at λ_PL_ of 555 nm (Φ_PL_ = 70%) as a powder and has a small Δ*E*_ST_ of 59 meV.

**Figure 106:**
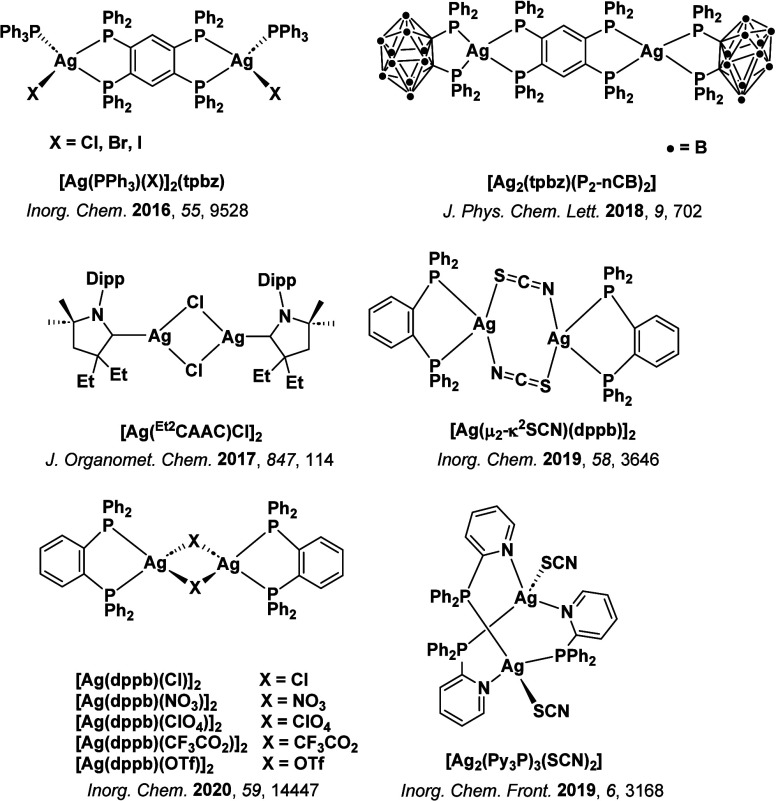
Chemical structures of dinuclear silver(I) complexes having TADF properties.

**Figure 107:**
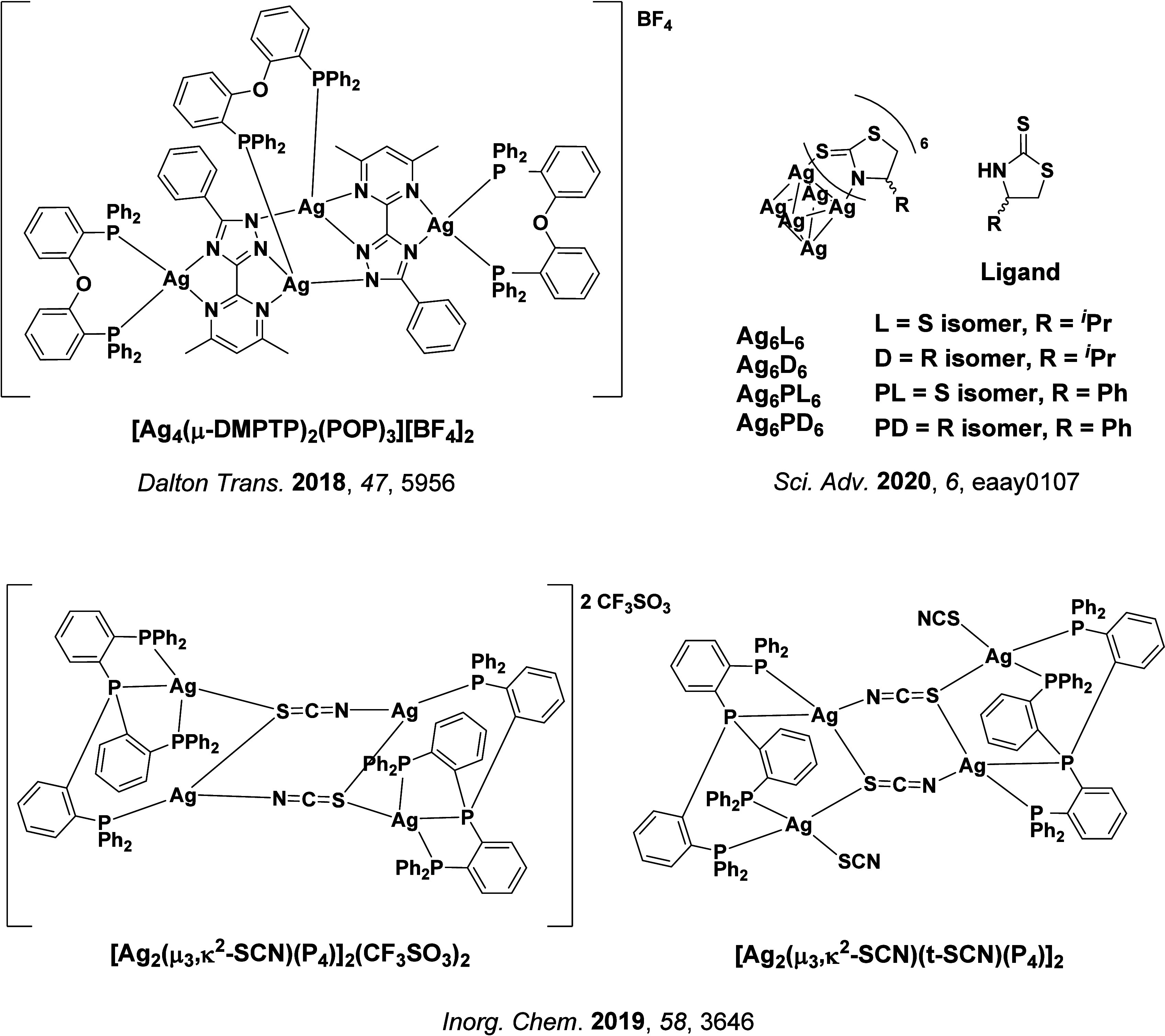
Chemical structures of multinuclear silver clusters having TADF properties.

A halide bridged dinuclear CAAC complex **[Ag(^Et2^CAAC)Cl]_2_** ^876^ (Figure 106) showed weak blue emission (λ_PL_ = 454 nm, Φ_PL_ = 5%), with a τ_d_ of 18.9 μs. Isothiocyanates can similarly act as bridging ligands, as in the case of **[Ag(μ_2_-κ^2^-SCN)­(dppb)]_2_**, which emits at λ_PL_ of 505 nm (Φ_PL_ = 35%, τ_d_ = 12 μs) as a powder and has a small calculated Δ*E*_ST_ of 90 meV.^830^ A considerably different bonding motif of similar subunits is adopted in **[Ag_2_(Py_3_P)_3_­(SCN)_2_]**,^882^ which contains two close lying silver(I) atoms bridged by three pyridylphosphine ligands, while the isothiocyanates are terminal. This complex exhibited a solvent-induced enhancement of the solid-state emission. The desiccated complex emits at λ_PL_ of 469 nm and has a low Φ_PL_ of 16%, but upon exposure to CH_2_Cl_2_ or CHCl_3_ vapors, Φ_PL_ increases to ≈ 70% along with a small (*ca.* 10 nm) red-shift in the emission. This process was reversible through heating the complex to 130 °C. Bridging isothiocyanate ligands are also present in tetranuclear silver(I) complexes^830^ **[Ag_2_(μ_3_,κ^2^-SCN)(P_4_)]_2_­(CF_3_SO_3_)_2_** and **[Ag_2_(μ_3_,κ^2^-SCN)­(t-SCN)(P_4_)]_2_** (Figure 107) that show sky-blue emission (λ_PL_ = 468 to 475 nm) and have moderate Φ_PL_ of up to 43% and τ_d_ of between 3.7 to 6.4 μs as powders.

A large series of complexes with phosphine ligands and bridging (pseudo-)halide ligands have been prepared that tune the emission color from sky blue to red,^883^ with a subset of these complexes exhibiting TADF. The dppb-terminated complexes **[Ag(dppb)(X)]_2_** (Figure 106) show sky blue-to-green emission in 5 wt% doped films in PMMA (λ_PL_ = 476–515 nm) and have τ_PL_ ranging from 6 to 63 μs and Φ_PL_ of up to 53%. The Δ*E*_ST_ of the triflate-bridged complex was experimentally found to be 74 meV.

Moving beyond dinuclear Ag(I) complexes, the tetranuclear complex **[Ag_4_(μ-DMPTP)_2_­(POP)_3_]­[BF_4_]_2_**.^878^ shows very bright green emission (λ_PL_ = 527 nm, Φ_PL_ = 76%) and has a very short lifetime (τ_PL_ = 0.65 μs), associated with its small Δ*E*_ST_ of 80 meV.

Lastly there are examples of reported hexameric clusters **Ag_6_L_6_** ^884^ containing enantiopure thiazolidine-2-thione ligands that show TADF (Figure 107). These clusters emit at λ_PL_ ranging from 556 to 575 nm in the solid state with Φ_PL_ of between 56 and 95% and τ_PL_ of 16 to 18 μs at room temperature. The Δ*E*_ST_ are 96 meV for **Ag_6_L_6_** and 41 meV for **Ag_6_PL_6_**. The chiral clusters also show moderate luminescence dissymmetry factors (*g*_lum_ = ± 4.42 × 10^–3^) and are the first silver chiral TADF emitters.

### 9.4 Gold

Unlike copper and silver, there are examples of both emissive d^10^ gold(I) and d^8^ gold(III) complexes, many of which are TADF-active. The very first reports of TADF from gold-based materials were based on nano-clusters,^884–886^ however this review will only focus on organometallic gold complexes. Further down group 11, the larger SOC of gold in comparison to the lighter coinage metals results in many emissive gold complexes showing phosphorescence rather than TADF.^887^ Here we summarize gold complexes with emission that has been explicitly identified as TADF.

As with copper and silver complexes, there are a number of tri- and tetra-coordinated gold(I) complexes that show TADF (Figure 108). Indeed, many of these examples have the same ligand environment as copper(I) TADF complexes, and similar to these the low-lying excited states in the gold(I) complexes can best be described as MLCT states with the gold is directly involved in the transition. The first reported TADF gold(I) complex was **Au(dppb)(PS)**,^756^ a gold analogue of a known copper(I) TADF emitter.^756^ The complex emits in the orange as a powder (λ_PL_ = 610 nm, Φ_PL_ = 12%, τ_PL_ = 1.66 μs), with the emission significantly red-shifted (90 nm) compared to the analogous copper(I) complex.

**Figure 108:**
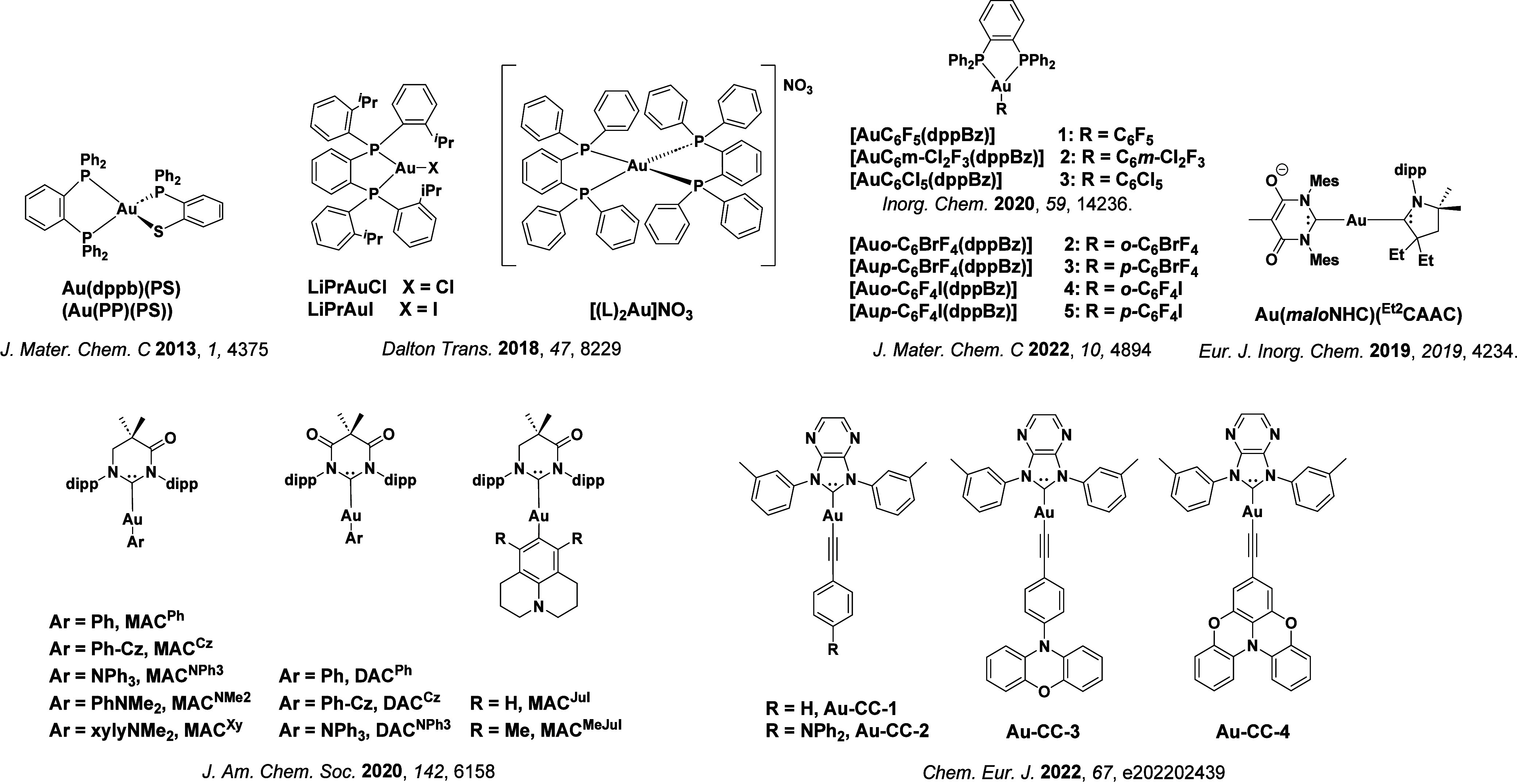
Chemical structures of gold(I) complexes having TADF properties.

A pair of trigonal planar complexes **(LiPr)AuX** and a cationic tetrahedral complex **[(dppb)_2_Au](NO_3_)** (Figure 108) all show very bright sky-blue to green emission as crystals (λ_PL_ = 485–558 nm, Φ_PL_ > 82%).^888^ The microsecond-long lifetimes (τ_PL_ = 3.8–13 μs) and small Δ*E*_ST_ of 80 to 120 meV support the assignment to TADF. The complexes are much less emissive in 2-MeTHF; the neutral complexes are weak orange emitters (λ_PL_ = 596 to 607 nm, Φ_PL_ = 2 to 4%) while **[(dppb)_2_Au](NO_3_)** is not emissive at all in solution. This red-shift and lower Φ_PL_ are attributed to the more polar solvent stabilizing the CT excited state in the former and the faster *k*_nr_ resulting from increased vibrational motion in fluid solution for the latter.

The trigonal planar series of complexes **[(dppBz)Au(Ar)]** (Figure 108) contain perhalophenyl ligands, and are yellow-emissive (λ_PL_ = 545 to 560 nm, Φ_PL_ = 11 to 29%, τ_PL_ = 10 to 21 μs) with Δ*E*_ST_ ranging from 58 to 144 meV.^889^ A subsequent study explored the impact of the addition of bromine and iodine atoms to the perfluorophenyl group at the *ortho* and *para* positions to determine if the presence of the additional heavy atom influenced the properties of the gold complexes. Ultimately there was little impact on the emission properties of the complexes, as the central gold atom already ensured very strong SOC that was not significantly increased through the use of ancillary heavy halogen atoms.^890^

The linear gold(I) carbene complex **Au(*malo*NHC)(^Et2^CAAC)** (Figure 108) contains an unusual zwitterionic mixed carbene structure and exhibits weak sky-blue emission as powder (λ_PL_ = 461 nm, Φ_PL_ = 2.7%). In 5 wt% doped PS films the emission properties are similar (λ_PL_ = 464 nm, Φ_PL_ = 3%), and biexponential decay kinetics typical of TADF were reported (τ_PL_ = 1.5 and 22 μs in the PS film).^877^

The emission of a series of linear carbene-aryl gold(I) complexes (**DAC^aryl^** and **MAC^aryl^**, named **1a–c** and **2a–e** in the original work, Figure 108) can be tuned from blue to orange (λ_PL_ = 460 to 620 nm in 1 wt% doped PS films, with Φ_PL_ of up to 77%) as a function of the structure of the aryl group.^891^ The complexes with either a phenyl ring or a phenyl carbazole for the aryl group (**DAC^Ph^**, **DAC^Cz^**, **MAC^Ph^**, and **MAC^Cz^**) have MLCT excited states that emit via phosphorescence. In contrast, the remaining complexes have LLCT excited states that lead to emission via TADF. Most of the complexes showed only modest luminescent efficiencies (Φ_PL_ < 40%), attributed to high non-radiative decay rates resulting from free rotation around the Au-C_aryl_ bond. Addition of two methyl groups *ortho* to the Au-C_aryl_ bond in either the dimethylaniline ligand in **MAC^NMe2^** or the julolidine ligand in **MAC^Jul^** blocks this rotation around the Au-C_aryl_ bond and locks these two ligands in a co-planar configuration. The resulting reduction in the non-radiative decay rate for the complexes **MAC^Xy^** and **MAC^MeJul^** leads to a significant increase in the Φ_PL_ to between 35–75%. The Δ*E*_ST_ range between 86 and 203 meV for all the TADF emissive complexes.

A series of four linear carbene-alkynyl complexes (**1–4** in the original work and renamed **Au-CC-1** to **Au-CC-4** here, Figure 108) are bright blue to green emitters.^892^ The complexes have Φ_PL_ ranging from 36 to 76%, and have τ_PL_ of up to 60 μs in 5% doped films in PMMA with Δ*E*_ST_ ranging from 82 to 162 meV. The green solution-processed OLEDs with **Au-CC-2** showed an EQE_max_ of 20.4% at CIE coordinates of (0.32, 0.54), however the efficiency roll-off was severe with an EQE_1000_ of 9.7%.

MR-TADF emitters are an emerging class of TADF materials that have garnered great interest (see Section 11). One critical issue with MR-TADF emitters is their relatively slow *k*_RISC_ compared to D-A analogues. In an effort to increase RISC several studies have added heavy atoms to the skeleton, including coordination to gold atoms.^180,191,893^ Cai *et al.* reported a series of MR-TADF emitters with a coordinated gold(I) NHC moiety.^894^ **(SIPr)AuBN**, **(BzIPr)AuBN**, **(PyIPr)AuBN**, **(PzIPr)AuBN**, **(Ipr)AuBN**, and **(BzIPr)AuBNO** (Figure 109) all have very high Φ_PL_ of up to 99%, and show narrowband emission (FWHM of 30–37 nm) in both thin films (2 wt% in PMMA) and in THF. The OLEDs with these Au(I)-MR-TADF complexes show good performance, with **(BzIPr)AuBN** achieving an EQE_max_ of 30.3% at CIE coordinates of (0.16, 0.68), with very low efficiency roll-off (EQE_1000_ = 28.1%). Further, this device showed good stability with an LT_60_ of 1210 h at 1000 cd m^–2^.^894^

**Figure 109:**
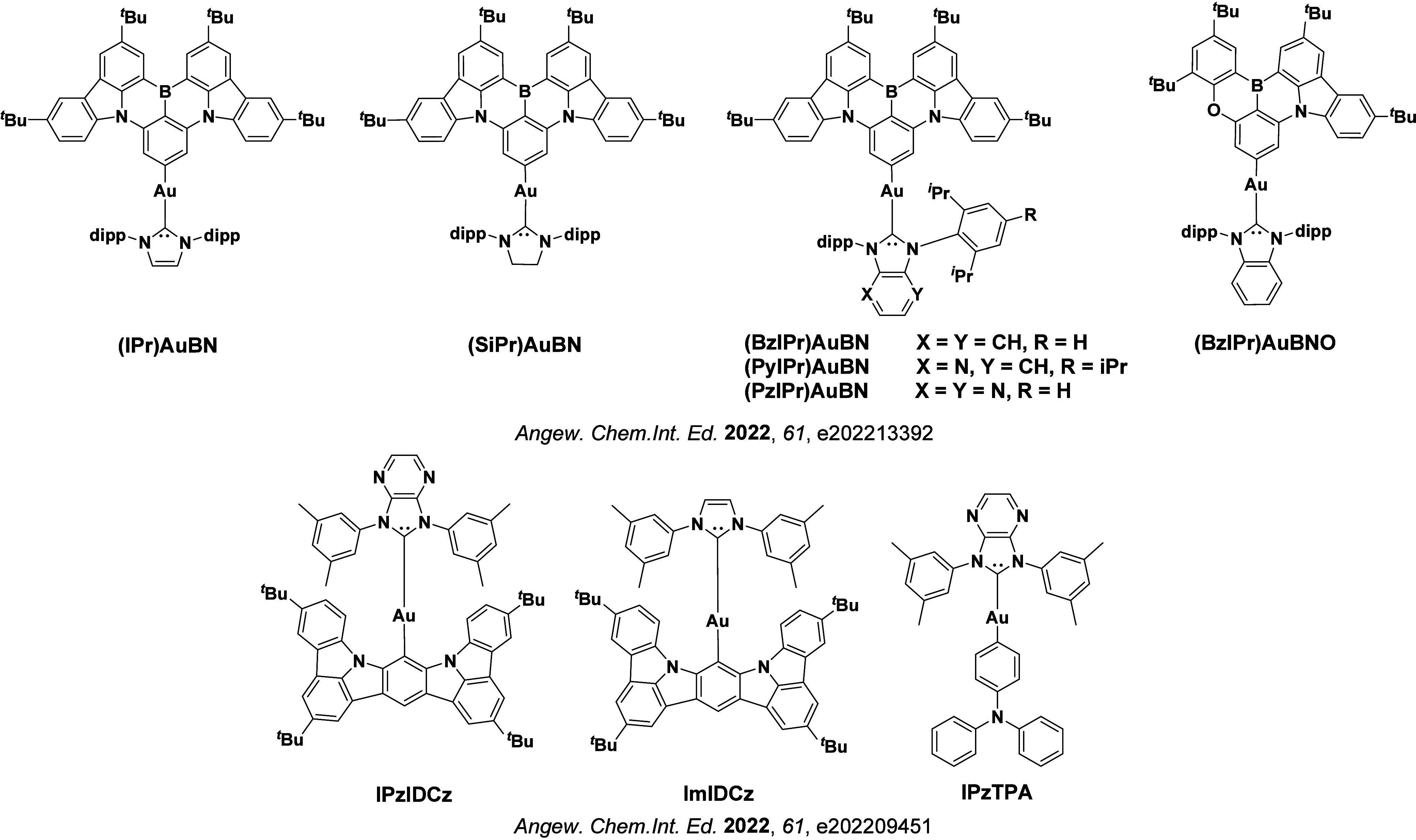
Chemical structures of TADF gold(I) complexes containing and NHC ligand and acyclometalating ligand based on MR-TADF motifs having TADF properties (**ImIDz** is a phosphorescent emitter, dipp = 1,3-di(4-imi­da­zo­lino­phen­oxyl)­pro­pane).

Feng *et al*. have also reported complexes with gold(I) carbene moieties coordinated to the narrowband fluorescent *meta*-diindolocarbazole core^161,895^ . **IPzIDCz** emits by TADF while **ImIDCz** is phosphorescent, the mechanism controlled by the carbene ligand used (Figure 109).^896^ **IPzIDCz** shows intense green emission in 10 wt% doped films in DPEPO, with Φ_PL_ = 66% and τ_PL_ = 2.9 μs. A green OLED with **IPzIDCz** (1 wt% doped in DMIC-TRZ) showed an EQE_max_ = 23.9% at CIE coordinates of (0.37, 0.57) and had only minimal efficiency roll-off (EQE = 20% at 47 000 cd m^–2^) yet, surprisingly, only a moderate LT_95_ of 27.4 h at 1000 cd m^–2^. These results suggest that coordination of gold(I) carbenes to MR-TADF compounds is a promising tactic for high performance OLEDs.

Unlike Au(I) complexes, all TADF Au(III) emitters are 4-coordinate complexes containing either a di-anionic tridentate pincer ligand with a monodentate halide, aryl, alkynyl, or amide ligand (Figure 110), or a tri-anionic tetradentate ligand (Figure 111). The first reported gold(III) TADF emitters were a series of complexes based on a diphenylpyrazine pincer ligand.^758^ These complexes are all weakly emissive in the neat film and in DCM (Φ_PL_ < 8%), with highly structured emission ranging from green to orange suggestive of emission from a LC state. The first OLEDs containing a gold(III) emitter were reported from complexes made with a diphenylpyridine pincer ligand.^897^ Of the family of eight complexes prepared, the most promising contain triphenylamine groups, **(C^∧^N^∧^C)Au(PhN(Ph)_2_)** and **(CF_2_^∧^NOEt^∧^CF_2_)Au(PhN(Ph)_2_)**. In 4 wt% doped films in PMMA these two compounds emit at λ_PL_ of 523 and 517 nm, have short τ_d_ of 1.35 and 0.72 μs, and high Φ_PL_ of 66 and 84%, respectively. Solution-processed OLEDs with **(C^∧^N^∧^C)Au(PhN(Ph)_2_)** and **(CF_2_^∧^NOEt^∧^CF_2_)Au(PhN(Ph)_2_)** showed EQE_max_ of 14.8% and 23.8% (and efficiency roll-off of only 1 or 31% at 1000 cd m^–2^) at CIE coordinates of (0.32,0.55) and (0.27,0.51), all respectively. The efficiency roll-off for the latter was reduced to 8% when a larger band gap host (PYD2) was used but the EQE_max_ simultaneously dropped to 15.7%.

**Figure 110:**
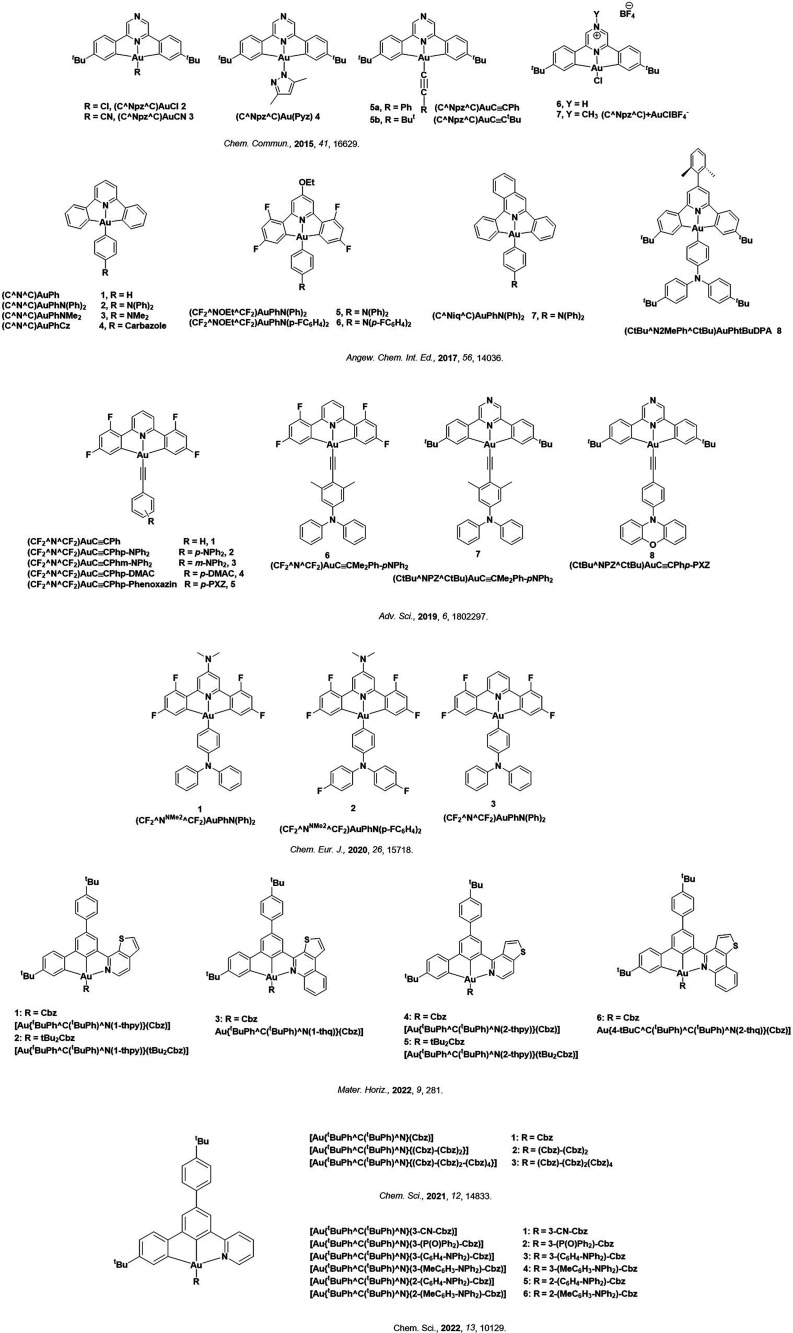
Chemical structures of TADF gold(III) complexes containing tridentate pincer ligands.

**Figure 111:**
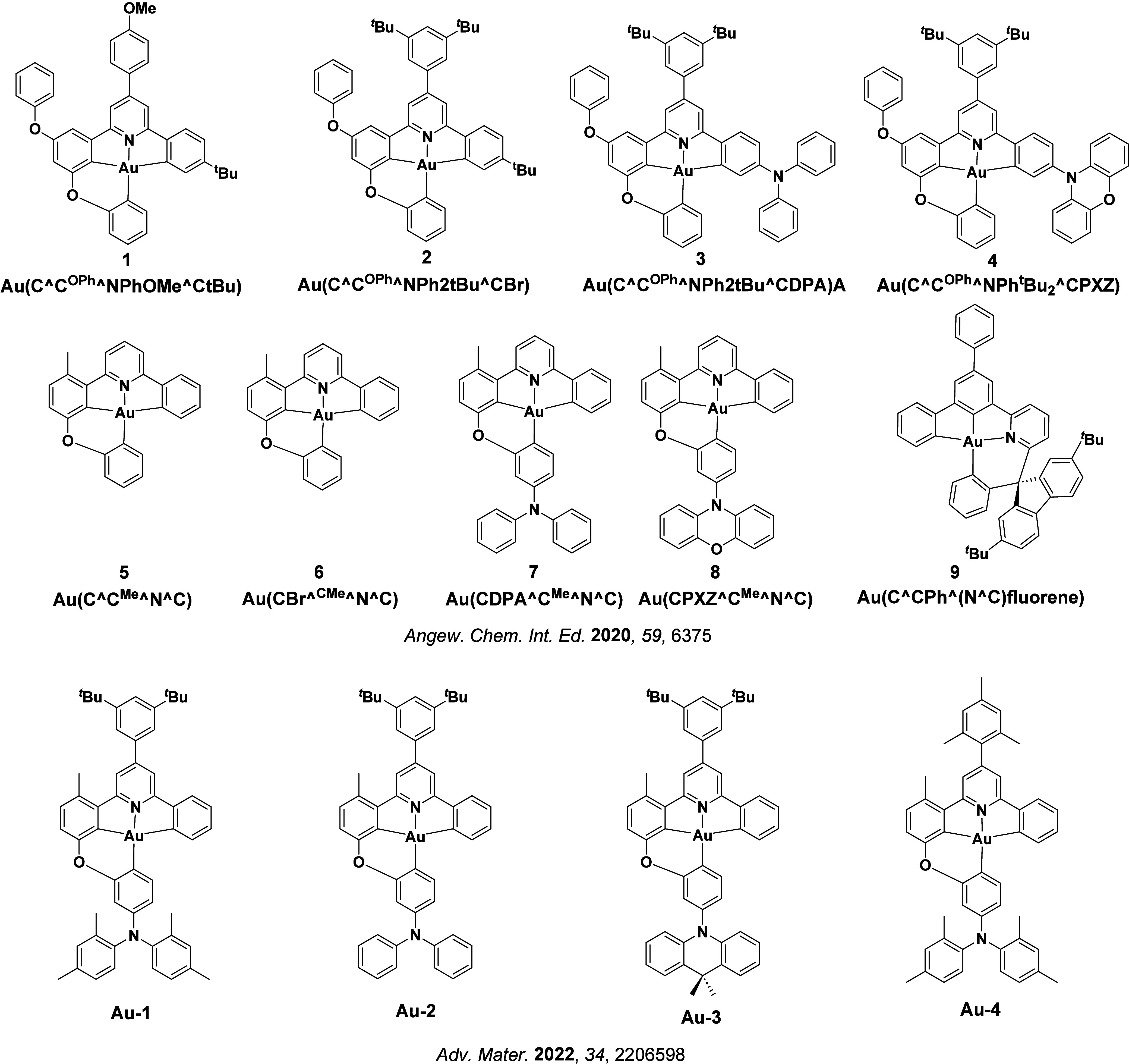
Chemical structures of tetracoordinate TADF gold(III) complexes.

A similar set of complexes, **(CF_2_^∧^N^Nme2^^∧^CF_2_)Au(PhN(Ph)_2_)** and **(CF_2_^∧^N^Nme2^^∧^CF_2_)Au(PhN(p-FC_6_H_4_)_2_)** (Figure 110), display a blue-shifted emission in 4 wt% doped PMMA films (λ_PL_ of 484 and 470 nm, Φ_PL_ of 34 and 82%, and τ_PL_ = 0.97 and 0.95 μs, all respectively) due to the increased electron density on the central pyridine ring of the acceptor pincer ligand associated with the donating dimethylamine group. Analogue **(CF_2_^∧^N^∧^CF_2_)Au(PhN(Ph)_2_)** without this substituent exhibits a red-shifted emission (λ_PL_ = 550 nm, Φ_PL_ = 81% and τ_PL_ = 0.69 μs).^898^ The solution-processed OLEDs with the sky-blue emitters showed reasonably narrow electroluminescence (FWHM = 64–67 nm) with CIE coordinates of (0.16, 0.25) and (0.16, 0.23) and EQE_max_ of 6.8 **(CF_2_^∧^N^Nme2^^∧^CF_2_)Au(PhN(Ph)_2_)** and 15.3% **(CF_2_^∧^N^Nme2^^∧^CF_2_)Au(PhN(p-FC_6_H_4_)_2_)** although with considerable efficiency roll-off of 63 and 35% at 100 cd m^–2^, respectively. The solution-processed OLED with **(CF_2_^∧^N^∧^CF_2_)Au(PhN(Ph)_2_)** showed an EQE_max_ of 24.3% at CIE coordinates of (0.35, 0.56) and moderate efficiency roll-off of 24% 1000 cd m^–2^.

A series of green-to-yellow-emitting gold(III) complexes containing diphenylpyridine pincer ligands and substituted alkynyl ligands (**(CF_2_^∧^N^∧^CF_2_)AuC≡CPh)** and others, Figure 110) have Φ_PL_ up to 88% and τ_d_ from 0.33 to 1.46 μs in solution and doped PMMA films.^899^ Calculations indicated that the lowest excited states are LLCT in nature where a diphenylamine (or a related ring-closed system such as phenoxazine or acridan) acts as the donor and the pincer ligand is the acceptor. The calculated Δ*E*_ST_ is dependent on the conformation of the arylamine with respect to the plane of the pincer ligand bound to the gold center, and varies from 2 to 330 meV, suggesting that certain conformers are TADF-active. Of the OLEDs tested, the one with **(CF_2_^∧^N^∧^CF_2_)AuC≡C-Me_2_Ph-*p*NPh_2_)** showed the best performance with an EQE_max_ of 23.4% at CIE coordinates of (0.40, 0.55,) and an efficiency roll-off of 5% at 1000 cd m^–2^.^899^ The OLEDs were also relatively stable with LT_95_ = 500 h at 100 cd m^–2^.

Beyond gold(III) aryl and acetylide complexes there are also gold(III) complexes bearing amide ligands. Yellow-to-red-emitting complexes **[Au{^t^BuPh^∧^C(^t^BuPh)^∧^N(1-thpy)}(Cbz)]** and **[Au{^t^BuPh^∧^C(^t^BuPh)^∧^N(2-thpy)}(Cbz)]** (λ_PL_ = 554 and 557 nm, respectively, Figure 110) are examples that employ pincer ligands based on isomeric thienopyridine and thienoquinoline in combination with a carbazolate ligands.^900^ In 5 wt% doped films in mCP these compounds have Φ_PL_ of 83 and 81% and τ_d_ of 4 and 7 μs, respectively. The best performing OLEDs with **[Au{^t^BuPh^∧^C(^t^BuPh)^∧^N(1-thpy)}(Cbz)]** showed EQE_max_ of 14.5% at CIE coordinates of (0.60, 0.40), and also showed excellent device stability (LT_70_ = 63 258 h at 100 cd m^–2^).

Yam and co-workers reported a series of highly efficient TADF gold(III) complexes containing carbazolate donor dendrons.^901^ A reference gold(III) TADF complex **[Au{^t^BuPh^∧^​C(^t^BuPh)^∧^​N}(Cbz)]** (**G_0_**), the first-generation dendrimer **[Au{^t^BuPh^∧^​C(^t^BuPh)^∧^​N}{(Cbz)-(Cbz)_2_}]** (**G_1_**), and the second-generation dendrimer **[Au{^t^BuPh^∧^​C(^t^BuPh)^∧^​N}{(Cbz)-(Cbz)_2_-(Cbz)_4_}]** (**G_2_**, Figure 110) emit at λ_PL_ of 547, 532, and 535 nm in 10 wt% doped mCP films. The dendrimer complexes **G_0_**, **G_1_**, and **G_2_** have Φ_PL_ of 82, 74, and 75% and τ_PL_ of 3.5, 1.2, and 1.4 μs, respectively. The solution-processed OLED based on **[Au{^t^BuPh^∧^​C(^t^BuPh)^∧^​N}{(Cbz)-(Cbz)_2_-(Cbz)_4_}]** showed an EQE_max_ of 15.8% at CIE coordinates of (0.38, 0.57).^901^ Addition of triphenylamine groups to the carbazolate ligand of **G_0_** results in a family of six further complexes showing bright green-yellow emission in 10 wt% doped films in mCP. Of these complexes, **[Au{^t^BuPh^∧^​C(^t^BuPh)^∧^​N}(2-(MeC_6_H_3_-NPh_2_)-Cbz)]** showed the highest Φ_PL_ of 79% at a λ_PL_ of 535 nm, and has a τ_PL_ of 5.9 μs.^902^

Finally, Zhou *et al*. explored gold(III) complexes containing a single tetradentate ligand, prepared via microwave synthesis.^903^ Three of these complexes, **Au(C^∧^C^OPh^^∧^​NPh2^t^Bu_2_^∧^​CPXZ)**, **Au(CDPA^∧^​C^Me^^∧^N^∧^C)**, and **Au(CPXZ^∧^​C^Me^^∧^N^∧^C)** (Figure 111) show TADF (λ_PL_ = 520–568 nm, Φ_PL_ = 71–89% and τ_d_ = 1.69–2.54 μs). Green OLEDs with **Au(CDPA^∧^C^Me^^∧^N^∧^C)** and **Au(CPXZ^∧^C^Me^^∧^N^∧^C)** showed EQE_max_ of 23 and 20% at CIE coordinates of (0.26, 0.54) and (0.34, 0.56), respectively. The OLED with **Au(C^∧^C^Oph^^∧^NPh^t^Bu_2_^∧^CPXZ)** showed an EQE_max_ of 25% with CIE coordinates of (0.43, 0.54), and showed low efficiency roll-off (EQE_1000_ = 22%) with very good stability (LT_95_ = 5 280 h at 100 cd m^–2^).

Zhou *et al*. later prepared another series of four green-emitting TADF tetradentate gold(III) complexes with trianionic (C^∧^C^∧^N^∧^C) ligands that showed excellent photophysical properties.^759^ Complexes **Au-1**, **Au-2**, **Au-3**, and **Au-4** emit between λ_PL_ of 525 to 547 nm with Φ_PL_ of up to 88% in toluene. Doped at 4 wt% in DPEPO/TCTA films (1:1 host), these complexes emit between λ_PL_ of 513 to 530 nm with Φ_PL_ up to 99%. The τ_d_ are between 0.47 and 0.69 μs in both toluene and doped films, although **Au-3** alone is not TADF-active. The OLEDs with **Au-1**, **Au-2**, and **Au-4** are bright and efficient with EQE_max_ > 24%, reaching 27.3% at CIE coordinates of (0.36, 0.60) **Au-4**.the devices showed relatively low efficiency roll-off of < 28% for all devices, and their stabilities are outstanding with the longest reported device lifetimes of any metal-based TADF OLEDs. For instance, the device with **Au-1** had an LT_90_ of 128,864 h at 100 cd m^–2^. These results confirm that TADF gold(III) emitters are a very promising class of emitter materials for OLEDs.

### 9.5 Carbene Metal Amides (CMAs)

CMA complexes (Figure 112 and Figure 113) have rapidly come to the fore as arguably the most promising class of TADF coinage metal complexes. The pioneering work of Di *et al*. in 2017 demonstrated outstanding performance for solution-processed OLEDs with Au(I) CMA complexes,^194^ and since this first study there has been a growing number of reported coinage metal CMA TADF emitters. The emissive excited states in these complexes are best characterized as LLCT, with the metal acting as a bridge between the amide donor and the carbene acceptor, but also contributing to SOC and RISC.

**Figure 112:**
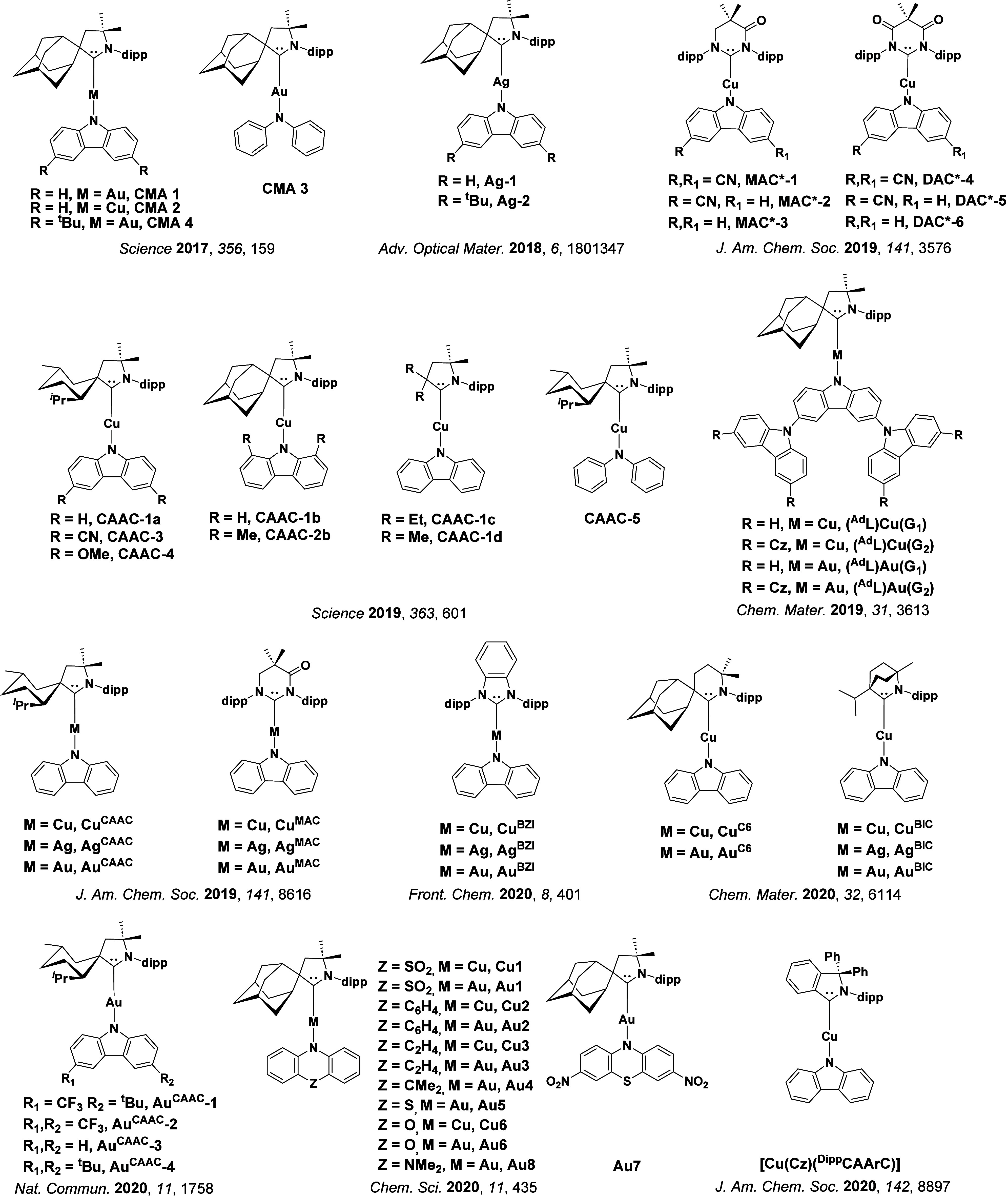
Chemical structures of linear carbene metal amide (CMA) silver(I), copper(I) and gold(I) and complexes having TADF properties published up to 2020.

**Figure 113:**
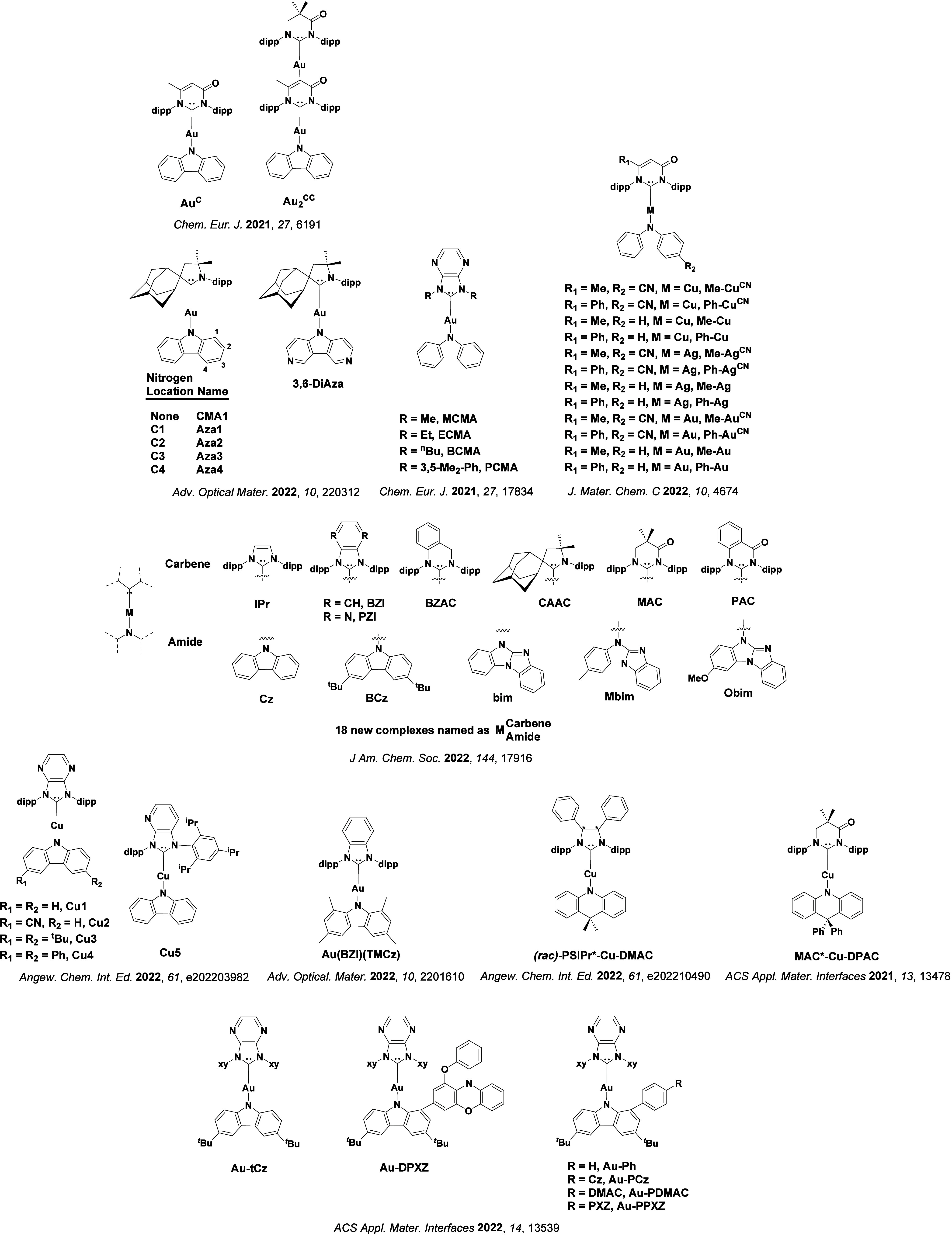
Chemical structures of linear carbene metal amide (CMA) silver(I), copper(I) and gold(I) complexes having TADF properties.

The first report of TADF CMA complexes contained an adamantyl-substituted cyclic alkyl amino carbene (CAAC) ligand as part of four separate gold and copper compounds.^194^ These complexes showed green-to-yellow emission with near unity Φ_PL_ in the solid state and very fast emission lifetimes (τ_PL_ = 0.35 μs). Solution-processed OLEDs with the gold emitter **CMA 4** (Figure 112) showed outstanding performance, with EQE_max_ of 27.5% at CIE coordinates of (0.26, 0.48). The devices with **CMA 3** and **CMA 1** showed similarly impressive EQE_max_ of 17.9% and 26.3% respectively, while copper-containing **CMA 2** showed somewhat lower EQE_max_ of 9.7%. Impressively, the devices with all four complexes showed excellent efficiency roll-off of < 5% at 1000 cd m^–2^. It was proposed that rotation between the two ligands in the excited state modified singlet and triplet energies to give a negative Δ*E*_ST_, which the authors rationalized to explain both the short lifetimes and high Φ_PL_. However, the TD-DFT calculations upon which this conclusion was built were later found to be flawed, and it was later demonstrated that these complexes possess near-zero Δ*E*_ST_.^195,196^ Building on from this work, vacuum-deposited devices using **CMA 1** showed slightly improved EQE_max_ of 26.9% at CIE coordinates of (0.24, 0.43), with efficiency roll-off at 1000 cd m^–2^ of only 7%. Non-doped device of the same showed impressive EQE_max_ of 23.1% at CIE coordinates of (0.24, 0.46) with an efficiency roll-off of 5% at 1000 cd m^–2^, making these some of the best performing non-doped TADF OLEDs to date.^904^

Silver-containing complexes **Ag-1** and **Ag-2** (Figure 112) show similar photophysical properties to their gold analogues **CMA-1** and **CMA-4**,^905^ emitting at λ_PL_ of 521 and 546 nm in toluene and having Φ_PL_ of 74 and 55% with short τ_d_ of 0.46 and 0.305 μs, all respectively. These complexes were used in both solution-processed and vacuum-deposited devices. The solution-processed device with **Ag-2** showed an EQE_max_ of 11.0% at CIE coordinates of (0.36, 0.56), with the EQE_1000_ decreasing to 8.2%. The vacuum-deposited device of the same showed an improved EQE_max_ of 13.7% at similar CIE coordinates of (0.31, 0.50), retaining a higher EQE_1000_ of 10.0%. Devices with **Ag-1** showed much poorer performance with EQE_max_ < 4% for both solution-processed and vacuum-deposited devices, demonstrating the sensitivity of device performance to the choice of ligand in these CMA emitters.

Focusing on copper CMA complexes, Hamze *et al.* explored the impact of different carbenes and substitution of the carbazolate group in eight new complexes.^906^ Decreasing the steric bulk of the carbene ligand (**CAAC-1c** and **CAAC-1d**, Figure 112) results in increased non-radiative decay, manifesting in a lower Φ_PL_ (decreasing from 56 to 11%, for **CAAC-1c** and **CAAC-1d**). Addition of electron-withdrawing CN groups to the carbazolide ligand in **CAAC-3** results in localization of the excited state onto the carbazole ligand and fluorescence from a ligand-centered state (λ_PL_ = 428 nm in 2-MeTHF). Addition of electron-donating OMe groups to the carbazolide ligand results in a red-shift in the emission by destabilizing the HOMO of the donor carbazole group. Complexes with bulky ligands showed near unity Φ_PL_ as 1 wt% doped films in polystyrene, with emission ranging from λ_PL_ = 426 to 558 nm. The OLED with **CAAC-1a** showed a leading EQE_max_ of 9.0% at λ_EL_ of 460 nm.^906^

Six-membered heterocyclic carbenes MAC* and DAC* were used in a series of six new copper CMA complexes, MAC*-1–3 and DAC*-4–6 (Figure 112).^907^ The move from the monocarbonyl MAC ligand to the dicarbonyl DAC ligand resulted in stabilization of the LUMO by ≈ 1 eV, resulting in red-shifted emission for the DAC complexes. The addition of one or two CN groups to the carbazole ligand stabilized the HOMO by up to 0.5 eV, resulting in a blue-shift of the emission compared to complexes with just Cz. Between these two structural modifications, the emission of 1 wt% doped films in PS could be tuned across the full visible spectrum, from a λ_PL_ of 432 nm for MAC*-1 to 704 nm for DAC*-6. The OLED with MAC*-3 showed an EQE_max_ of 19.4% at a λ_EL_ of 543 nm.

Complexes **(^Ad^L)Cu(G_1_)**, **(^Ad^L)Au(G_1_)**, and **(^Ad^L)Au(G_2_)** (Figure 112) contain carbazole-based donor dendrons as ligands.^908^ These complexes showed similar photophysical properties to the parent complexes, **CMA-1** and **CMA-2**, with slightly reduced Φ_PL_ arising from the greater conformational motion within the donor dendron units. Solution-processed OLEDs with **(^Ad^L)Au(G_1_)** showed EQE_max_ of 10.6% at CIE coordinates of (0.39, 0.58) with negligible efficiency roll-off (EQE_1000_ = 10.0%), while the devices with the other two emitters showed poorer performance.

A number of reports have explored different carbene ligands in combination with carbazole in CMA complexes of copper, silver, and gold.^909–912^ Hamze *et al*. explored the difference in photophysical properties between 5-membered CAAC carbenes and 6-membered MAC ligands.^909^ The use of the 6-membered MAC carbenes induced a 35 to 40 nm red-shift in the emission of the complexes (1 wt% doped PS films), to λ_PL_ of 506, 512, and 512 nm for **Cu^MAC^**, **Ag^MAC^**, and **Au^MAC^** compared to the CAAC complexes at λ_PL_ of 470, 472 and 472 nm for **Cu^CAAC^**, **Ag^CAAC^**, and **Au^CAAC^**, all respectively (Figure 112). All six complexes have near unity Φ_PL_ in the same 1 wt% doped PS films, with shorter τ_PL_ for the MAC complexes of 2.8, 0.50, and 1.14 μs for **Cu^MAC^**, **Ag^MAC^**, and **Au^MAC^** compared to the CAAC complexes with τ_PL_ of 1.40, 0.33, and 0.83 μs for **Cu^CAAC^**, **Ag^CAAC^**, and **Au^CAAC^**, all respectively. The OLEDs with **Au^MAC^** showed EQE_max_ of 18% and EQE_1000_ of 15% with λ_EL_ at 510 nm.^909^ Changing the carbene from CAAC to BZI in a subsequent report resulted in a blue-shift of *ca*. 40 nm for each of **Cu^BZI^**, **Ag^BZI^**, and **Au^BZI^**, with λ_PL_ of 434, 438, and 432 nm, respectively.^910^ The complexes retained their high Φ_PL_ of > 85%, although the emission lifetime increased to τ_PL_ of 3.2 to 4.9 μs. Deep-blue OLEDs with **Au^BZI^** showed an EQE_max_ of 12% at CIE coordinates of (0.16, 0.06).^910^

A similar and direct comparison has been reported between CMA complexes using CAAC ligands with a monocyclic 6-membered ring (C6 ligand) and those using bicyclic 6-membered rings (BIC) in the series **Cu^C6^**, **Au^C6^**, **Cu^BIC^**, **Ag^BIC^**, and **Au^BIC^** (Figure 112).^911^ In 1 wt% doped Zeonex films the BIC complexes are sky blue emitters with λ_PL_ of 490 to 496 nm, while the C6 complexes are green emitters with λ_PL_ of 519 and 523 nm for **Cu^C6^** and **Au^C6^**, respectively. The more rigid BIC ligand reduces non-radiative decay in the complexes resulting in more efficient emission (Φ_PL_ = 82 to 100% for BIC complexes, compared to 3 to 22% for the C6 complexes) as well as longer τ_d_.^911^

In a bid to blue-shift the emission, replacement of the ^*t*^Bu groups on the carbazole of **CMA-4** with an electron-withdrawing CF_3_ moiety leads to complexes **Au^CAAC^-1** and **Au^CAAC^-2** (Figure 112, named simply **1** and **2** in the original work).^913^ The emission of the complexes in toluene is blue-shifted from 552 nm in **CMA-4** to 495 and 456 nm respectively in **Au^CAAC^-1** and **Au^CAAC^-2**. Despite the blue emission, no OLED was fabricated with **Au^CAAC^-2** due to its long emission lifetime (τ_d_ > 10 μs) and moderate efficiency (Φ_PL_ = 61%). The OLED with **Au^CAAC^-1**, however, showed an EQE_max_ of 20.9% at CIE coordinates of (0.17, 0.17), and moderate efficiency roll-off with EQE_100_ of 17.8%.

Changing the amide from carbazole to 6-membered heterocycles based on acridine resulted in a series of conformationally flexible complexes of both copper and gold, including **Cu1–3**, **Cu5**, and **Au1–8** (Figure 112).^914^ The complexes emit across a wide range of wavelengths in toluene (λ_PL_ = 489 to 689 nm) and in 5 wt% doped PS films (λ_PL_ = 458 to 649 nm), with the color tuned by the electronics of the atom or bridging group at the 9-position of the acridine ligand. The bluest emitters are **Cu1** and **Au1** (λ_PL_ = 489 and 505 nm respectively, in toluene) with a strongly electron-withdrawing SO_2_ group resulting in a weakly donating amide ligand, and consequently a larger HOMO-LUMO gap. Complexes with hydrocarbon bridging groups, **Cu2–3** and **Au2–4**, all emit similarly in toluene (λ_PL_ from 589 to 629 nm). Stronger donating amines with electron-donating O and S atoms, **Cu6** and **Au5–7**, or electron-donating Nme_2_ groups, **Au8**, show the most red-shifted emission in toluene (λ_PL_ of 654 to 689 nm). The Φ_PL_ decreases significantly as the emission color moves from blue to red (from 90% to < 0.1%), consistent with the energy gap law. Complex **Au1** also showed mechanochromic behavior, moving from warm-white to sky-blue emission upon grinding. The dual emission of **Au1** was exploited in solution-processed white OLEDs, which showed EQE_max_ of 4.6% at CIE coordinates of (0.18, 0.31).^914^

The emission color of most CMA complexes has been limited to blue to green, although Gernert *et al.* showed that it is possible to tune the emission of complexes bearing aryl-fused CAAC ligands to the red.^915^ Of their reported complexes only **[Cu(Cz)(^Dipp^CAArC)]** showed TADF, with deep-red emission (λ_PL_ = 621 nm, Φ_PL_ = 32%, and τ_PL_ = 0.37 μs).

Over time the sophistication and performance of CMA design strategies have naturally increased, and notably so since 2021. In an attempt to increase the radiative decay rate Li *et al.* designed bimetallic CMA complex **Au_2_^CC^** (Figure 113) that has a faster *k_r_* in comparison to the monometallic complex **Au^MAC^**.^916^ The emission was blue-shifted to 480 nm, with Φ_PL_ = 80% and τ_d_ = 0.52 μs. Increasing the substituent steric bulk of the imidazopyrazine carbene ligands in the series **MCMA**, **ECMA**, **BCMA**, and **PCMA** (Figure 113) had surprisingly little impact on the green emission color in 1 wt% doped films in PMMA, with λ_PL_ ranging between 510 to 520 nm for the four complexes. **PCMA** has the highest Φ_PL_ of 89% and shortest τ_d_ of 0.35 μs of the series, suggesting that this is nonetheless a promising new type of carbene ligand for CMA complexes.^912^

Instead of decorating the archetypal Cz with electron-withdrawing groups to blue-shift the emission of CMA complexes, an alternative strategy involves the use of more weakly electron-donating carboline derivatives.^917^ For instance, compared to **CMA-1** (λ_PL_ = 498 nm) the emission of **3,6-DiAza** (Figure 113) is blue-shifted to a λ_PL_ of 419 nm. Of the derivatives studied, the one with the most promising photophysical properties is **Aza3**, which emits at λ_PL_ of 450 nm with a Φ_PL_ = 66% and a τ_PL_ of 2.1 μs in 3 wt% doped film in PS.^917^

To understand in greater detail the excited state kinetics within CMA complexes, Li *et al*. performed a combined experimental and theoretical study on 12 CMA complexes that featured MAC ligands with either methyl or phenyl substituents and carbazole groups with or without a cyano group in the 3-position (Figure 113).^918^ All these complexes were bright emitters (Φ_PL_ > 50%), with emission ranging from sky blue (λ_PL_ = 476 nm) to yellow (λ_PL_ = 558 nm) and having short emission lifetimes (τ_PL_ < 1.5 μs). The theoretical study identified a ‘sweet spot’ where an NTO overlap of around 0.25 to 0.3 produced the best balance of between Δ*E*_ST_ and *f* to ensure both fast TADF and *k*_r_.

Muniz *et al*. recently described a series of 18 M_Amide_^Carbene^ complexes containing both existing and new (BZAC and PAC) carbenes, along with established and new (bim, Mbim, and Obim) amides (Figure 113).^919^ In 1 wt% doped PS films the emission spans from λ_PL_ = 400 to 594 nm, and 15 of the complexes have Φ_PL_ > 75%. Importantly, this study revealed strong correlation between the theoretical electron-hole distance in the CMA complexes (determined from NTO calculations) and the experimentally observed *k_r_*. Of the complexes investigated **Au_bim_^PZI^**, **Au_bim_^BZI^**, **Au_bim_^PAC^**, and **Au_bim_^BZAC^** have the shortest τ_PL_ of 0.24, 0.25, 0.27, and 0.28 μs respectively.

The use of bulky pyrazine- and pyridine-annulated NHC ligands has been further explored in a series of copper CMA complexes **Cu1-Cu5** (Figure 113).^920^ The complexes emit across a wide range from sky-blue to orange (λ_PL_ = 470 to 660 nm in 2 wt% doped films in mCP). The use of the pyrazine-fused ligand PzIPr in **Cu1-Cu4** resulted in red-shifted emission, especially when combined with an unsubstituted (**Cu1**) or substituted carbazolate ligand (**Cu3** and **Cu4**), resulting in λ_PL_ ranging from 567 to 581 nm. Introduction of a weakly donating carbazolate ligand containing electron-withdrawing cyano groups in **Cu2** led to a blue-shifted emission with λ_PL_ of 508 nm. Moving from a pyrazine-containing carbene to a weaklier accepting pyridine-based carbene in **Cu5** led to a further blue-shift in the emission of 5 wt% doped films in mCP, to λ_PL_ of 470 nm. All the complexes showed short emission lifetimes (τ_d_ = 0.36 to 0.47 μs in 2 wt% doped films in mCP) and moderate to good efficiency (Φ_PL_ = 52 to 88% in the same). OLEDs with **Cu5** showed EQE_max_ of 23.6% at CIE coordinates of (0.14, 0.22), with low efficiency roll-off of 12% at 10,000 cd m^–2^ and very long device lifetimes (LT_90_ of up to 1300 h at 1000 cd m^–2^). These are the best-performing copper CMA OLEDs reported to date.^920^

Given the excellent performance of CMA TADF emitters, they have also been explored as assistant dopants in HF-OLEDs (See Section 17). In addition to using a known CMA complex **(BZI)Au(Cz)**,^910^ Heo *et al*. designed the related complex **(BZI)Au(TMCz)** containing a sterically modified carbazole (Figure 113).^921^ This modification resulted in a red-shift in the emission of the 5 wt% doped film in Zeonex to λ_PL_ = 466 nm, while retaining a high Φ_PL_ of 95% and short τ_PL_ of 0.38 μs that makes this complex amenable as an assistant dopant for green HF-OLED.

In a later study focusing on the impact of restricting rotation around the Au-N bond, Yang *et al.* investigated a series of six CMA complexes with substituents in the 1-position of the 4,7-di-^*t*^Bu-carbazole ligand to lock the CMA complexes into a twisted conformation.^922^ The reference complex with no substituent on the carbazole ligand, **Au-tCz** (Figure 113), has a dihedral angle between the carbene and carbazole of only 0.4°. In contrast, the other five complexes have much larger dihedral angles of between 66–74°. The high luminescence efficiency of CMA complexes is generally attributed to the high oscillator strength and fast radiative decay arising from a co-planar arrangement of the ligands around the metal centre.^194,906^ However in these twisted complexes the Φ_PL_ remains high at between 73 to 94% in 5 wt% doped films in mCP. This outcome is attributed to a decrease in the Δ*E*_ST_ and an increase in SOC of the T_1_ → S_1_ RISC process supported by the large dihedral angles, which counteracts the decrease in oscillator strength for the S_1_ → S_0_ transition moving away from a co-planar conformation.

To target CPL emission, the first chiral CMA complexes (*R,R)*-PSIPr*-Cu-DMAC and (*S,S)*-PSIPr*-Cu-DMAC (Figure 113) employed a chiral analog of the SIPr NHC ligand.^923^ Both enantiomers and the racemate showed TADF, with identical λ_PL_ = 531 nm, τ_PL_ = 0.14 μs, and Φ_PL_ = 24% in toluene solution. In dilute solution the enantiopure complexes showed no CPL signal, however in both the powder and crystal the complexes showed strong CPL signals with |*g*_lum_| values of up to 2.7 × 10^–2^ for the crystals. An extensive DFT study highlighted that aggregation-induced CPL was induced by limiting the rotation of the ligands in the solid state.^923^

Finally, Ying *et al.* replaced the common carbazole donor with the stronger electron-donor diphenylacridine to produce the red-emitting copper complex MAC*-CuDPAC (Figure 113).^924^ In toluene this complex emits at λ_PL_ of 638 nm and has a poor Φ_PL_ of 12% with a short τ_PL_ of 0.11 μs. In 1.5 wt% doped films of mixed CBP:​TPBi (1:1 co-host) it emits at λ_PL_ of 599 nm, has a much higher Φ_PL_ of 65%, and a τ_PL_ of 0.95 μs. Notably the Φ_PL_ of 65% in CBP:​TPBi films is very good for a red TADF emitter, supported by the rigid ligands reducing non-radiative decay. The complex was also shown to have a strongly horizontally orientated TDM in the same films. As a result, despite the modest Φ_PL_ the OLEDs with MAC*-Cu-DPAC showed an EQE_max_ of 21.1% at CIE coordinates of (0.58, 0.42), and the efficiency roll-off was also very low (EQE_1000_ = 20.1%).

### 9.6 Palladium and Platinum

Due to the very high SOC constant of platinum, most platinum(II) complexes are phosphorescent and have been widely used previously as emitters in OLEDs.^106,925^ Indeed, first report of triplet harvesting in an OLED used a platinum(II) porphyrin emitter.^14^ There have recently also been reports of platinum and palladium emitters that exhibit metal-assisted delayed fluorescence (MADF), where both delayed fluorescence and phosphorescence have been detected.^926^ In these examples the Δ*E*_ST_ can be moderately large (> 150 meV) but the large SOC constants of Pd and Pt nonetheless enable rapid ISC/RISC resulting in some TADF emission. In addition, a small number of bimetallic platinum(II) complexes have recently been shown to exhibit TADF.^927–929^

The first report of TADF in palladium(II) complexes presented **PdN3N** and **PdN3O** (Figure 114), containing rigid and planar tetradentate ligands.^926^ The small Δ*E*_ST_ in these ‘phosphorescent’ complexes allows an additional high-energy component in their emission spectra to emerge, that is enhanced at higher temperatures. This emission band has been attributed to fluorescence from a thermally accessible S_1_ state – i.e. TADF. This balance of emission mechanisms is of interest as it may provide a route to achieve blue emission from complexes with lower energy triplet excited states, that may also translate into more stable devices. **PdN3N** and **PdN3O** both emit at λ_PL_ of 534 nm, have Φ_PL_ of 76%, and τ_PL_ of 142 and 205 μs, respectively. The OLEDs with **PdN3N** and **PdN3O** showed EQE_max_ of 20.9 and 20.4%, however the efficiency roll-off was large at 32% at 100 cd m^–2^, attributed to the long emission lifetimes. Despite the efficiency roll-off, the OLED with **PdN3N** demonstrated excellent device stability with a very long lifetime (LT_90_ > 20 000 h at 100 cd m^–2^). There is a higher TADF contribution (30%) in **PdN3N** due to its smaller Δ*E*_ST_ of 150 meV, compared to 180 meV for **PdN3O**. The mechanism of the mixed TADF and phosphorescence process in **PdN3N** was later investigated computationally,^930^ and found to be supported by calculated T_1_ → S_1_ and T_2_ → T_1_ → S_1_ RISC rates comparable to the T_1_ → S_0_ phosphorescence rate at 300 K.

**Figure 114:**
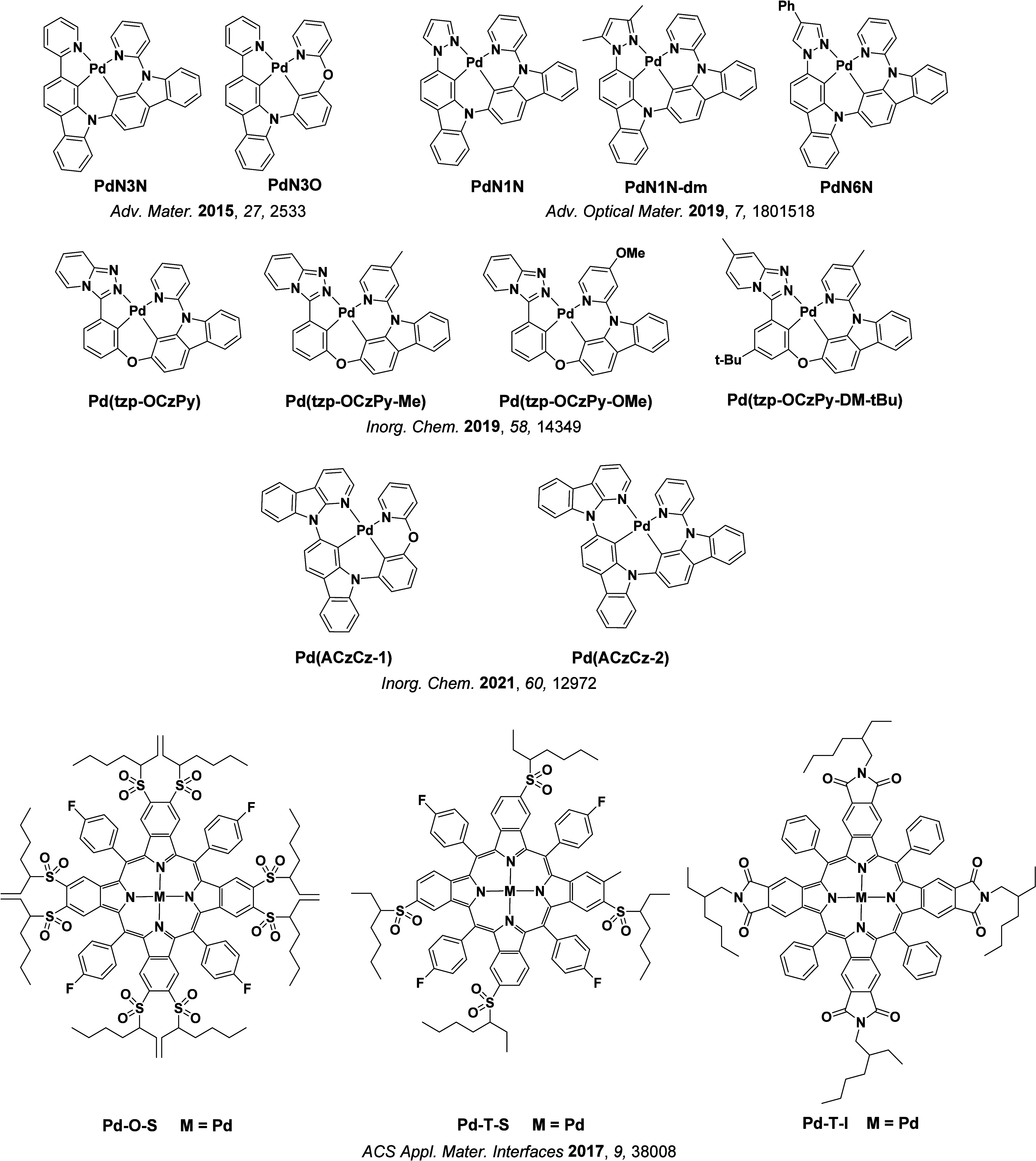
Chemical structures of TADF palladium(II) complexes.

Subsequently, a series of three complexes were reported in which one of the pyridyl groups of **PdN3N** was replaced with a pyrazolyl group to destabilize the LUMO and blue-shift the emission of **PdN1N**, **PdN1N-dm**, and **PdN6N** (Figure 114).^931^ The complex **PdN1N-dm** has the highest Φ_PL_ of 77% and the best thermal stability of the three complexes in the study, and so was used as the emitter in OLEDs. The devices showed an EQE_max_ of 25.1% at CIE coordinates of (0.14, 0.25), although there was very high efficiency roll-off (EQE_100_ = 11.1%). Another series of six complexes were also prepared in which the pyrazole within the ligand was replaced with a triazole group.^932^ The complex emitting with the highest TADF component is **Pd(tzp-OczPy-Ome)**, despite its Δ*E*_ST_ of 228 meV. The use of the same ligands to form a platinum complex produced a phosphorescent emitter with no observed TADF contribution. Similarly fusing rings to form an azacarbazolylcarbazole-based tetradentate ligand in complexes **Pd(AczCz-1)** and **Pd(AczCz-2)** led to either sky blue emission for the former (λ_PL_ = 479 nm) or green (λ_PL_ = 506 nm) for the latter. These complexes have small Δ*E*_ST_ of 57 and 112 meV, respectively in DCM, however are weak emitters with Φ_PL_ = 10–11%.^933^

A series of eight Pd and Pt porphyrin-based complexes (Figure 114 and Figure 115) have been developed for oxygen and temperature sensing.^934^ Again due to the higher SOC constant, the Pt complexes possess a smaller TADF contribution (and thus a stronger phosphorescence contribution) than the Pd complexes.^931^ The complexes all emit in the red to NIR region with well-resolved TADF (λ_PL_ = 620 to 652 nm) and phosphorescence (λ_PL_ = 742 to 800 nm) in the steady-state toluene PL spectra. The Φ_PL_ of the complexes ranged from 3 to 30%, while the τ_d_ of the platinum complexes ranged from 12 to 47 μs while those of the palladium complexes ranged from 53 to 286 μs. The complexes with the largest TADF contribution to the emission are **Pd-O-S** and **Pd-T-I**, which showed an increase in the TADF:​fluorescence ratio from 0.16 and 0.26 to 3.2 and 4.6 (respectively) as the temperature was increased from 5 to 80 °C, allowing optical readout.

**Figure 115:**
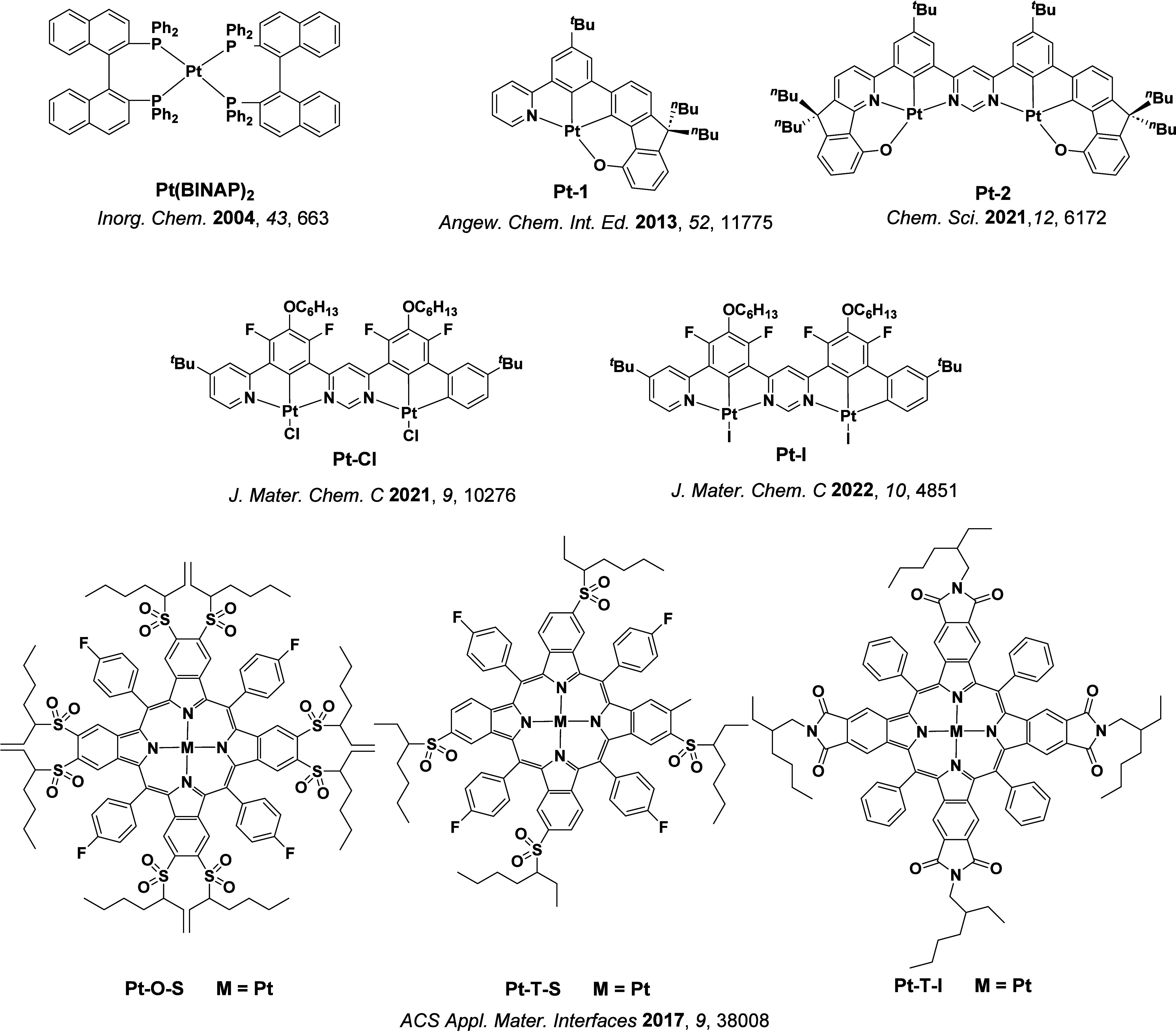
Chemical structures of TADF platinum(II) complexes.

Delayed fluorescence was first identified in a platinum(0) complex in 2004.^935^ The complex **Pt(BINAP)_2_** (Figure 115) emits at λ_PL_ of 763 nm in toluene and has a Φ_PL_ of 12%. TADF was assigned from both the biexponential decay kinetics (τ_p_ = 3.2 ps, τ_d_ = 1.025 μs) and the temperature dependent intensity of the delayed emission. DFT calculations indicated that the unusual emission properties of this complex were due to rapid ISC/RISC between the ^3^MLCT and ^1^MLCT states owing to the small Δ*E*_ST_ of 149 meV (calculated Δ*E*_ST_ = 184 meV). To date, no other TADF platinum(0) emitters have been reported.

Pander *et al.* have nonetheless shown that moving from mono- to dinuclear platinum complexes leads to much smaller Δ*E*_ST_ values, such that TADF outcompetes phosphorescence.^927^ This was initially demonstrated by comparison of the photophysical properties of **Pt-2** with its phosphorescent mononuclear analogue **Pt-1** (Figure 115),^936^ where computations revealed that the Δ*E*_ST_ decreased from 370 meV for the latter to 180 meV for the former. The smaller Δ*E*_ST_ in **Pt-2** is partially due to the use of the stronger electron-accepting pyrimidine (which coordinated both Pt atoms) compared to pyridine in the mononuclear complex, while simultaneously the calculated S_1_–T_1_ spin–orbit coupling matrix element (SOCME) drops from 88 cm^–1^ for **Pt-1** to 10 cm^–1^ for **Pt-2**. This lower SOCME value in the latter implies that directly spin-forbidden processes (such as phosphorescence) become slower in this material, giving TADF (which becomes spin-allowed through vibronic coupling) a window of opportunity to dominate the emission mechanism. Dissolved in MCH **Pt-2** emits at 602 nm, has a FWHM of 22 nm, and a small Stokes shift of only 7 nm, which is rare for third-row transition metal complexes and suggests both a small Δ*E*_ST_ and small reorganization energy in the excited state. The solution-state Φ_PL_ is 83% and the τ_PL_ is 2.1 μs. The OLEDs of **Pt-2** showed an EQE_max_ of only 7.4% at CIE coordinates (0.62, 0.37), but the emission is broadened compared to solution (FWHM of 75 nm). The low efficiency and spectral broadening were ascribed to the formation of aggregates in the film.

Related dinuclear Pt complexes bearing ancillary halogen ligands (**Pt-Cl** ^928^ and **Pt-I** ^929^, Figure 115) showed similar λ_PL_ of 635 and 633 nm with Φ_PL_ of 51 and 57% respectively in chlorobenzene. **Pt-Cl** exhibits a longer τ_PL_ (5.0 μs) in both chlorobenzene solution and 0.1 wt% doped films in PS than **Pt-I** (1.7 μs in chlorobenzene solution, 2.3 μs in PS). These results were rationalized by the smaller Δ*E*_ST_ of **Pt-I** (60 meV) compared to **Pt-Cl** (200 meV). Based on theoretical calculations, this variation in Δ*E*_ST_ was proposed to originate from a much smaller HOMO-LUMO overlap in **Pt-I**, with an MO pattern and chemical structure resembling an MR-TADF emitter. Solution-processed OLEDs with both **Pt-I** and **Pt-Cl** showed EQE_max_ of 3.1 and 2.6%, respectively. One of the devices using **Pt-Cl** at a very high doping concentration (33 wt%) is notably the first example of an excimer-based Pt(II) solution-processed OLED with NIR emission beyond 800 nm.

### 9.7 Zinc

Given the number of d^10^ coinage metal emitters discussed so far it is surprising that d^10^ zinc(II) complexes have received relatively little attention as TADF emitters. This comes despite the first example of a TADF zinc complex being reported in 2015.^937^ All the TADF zinc(II) complexes reported to date and discussed here show ligand-centered emission, with the zinc atom only minimally contributing to the excited states (charge transfer or otherwise). Adachi and co-workers reported the first zinc(II) TADF emitters **Zn(p-PX-BOX)_2_** and **Zn(m-PX-BOX)_2_** (Figure 116),^937^ using ligands that were themselves known D-A TADF emitters with phenoxazine donor and phen­yl­benz­oxa­zole acceptor. The calculated Δ*E*_ST_ are very small at 17 and 37 meV, respectively, while the HOMO and LUMO were expected to be located on the phenoxazine and phen­yl­benz­oxa­zole with no involvement from the metal center. **Zn(p-PX-BOX)_2_** and **Zn(m-PX-BOX)_2_** emit at λ_PL_ of 523 nm (Φ_PL_ = 78%) and λ_PL_ of 542 nm (Φ_PL_ = 58%) and have Δ*E*_ST_ of 60 and 180 meV, respectively, in 6 wt% doped films in mCBP. These Δ*E*_ST_ values are notably smaller than the metal-free methoxy-substituted ligand, ***p*-OMe-PX-BOX** (Δ*E*_ST_ = 310 meV), with the decrease attributed to an increase in the dihedral angle between the donor and acceptor upon coordination to the zinc. The green OLEDs with **Zn(p-PX-BOX)_2_** showed EQE_max_ of 19.6% at λ_EL_ of 542 nm.^937^

**Figure 116:**
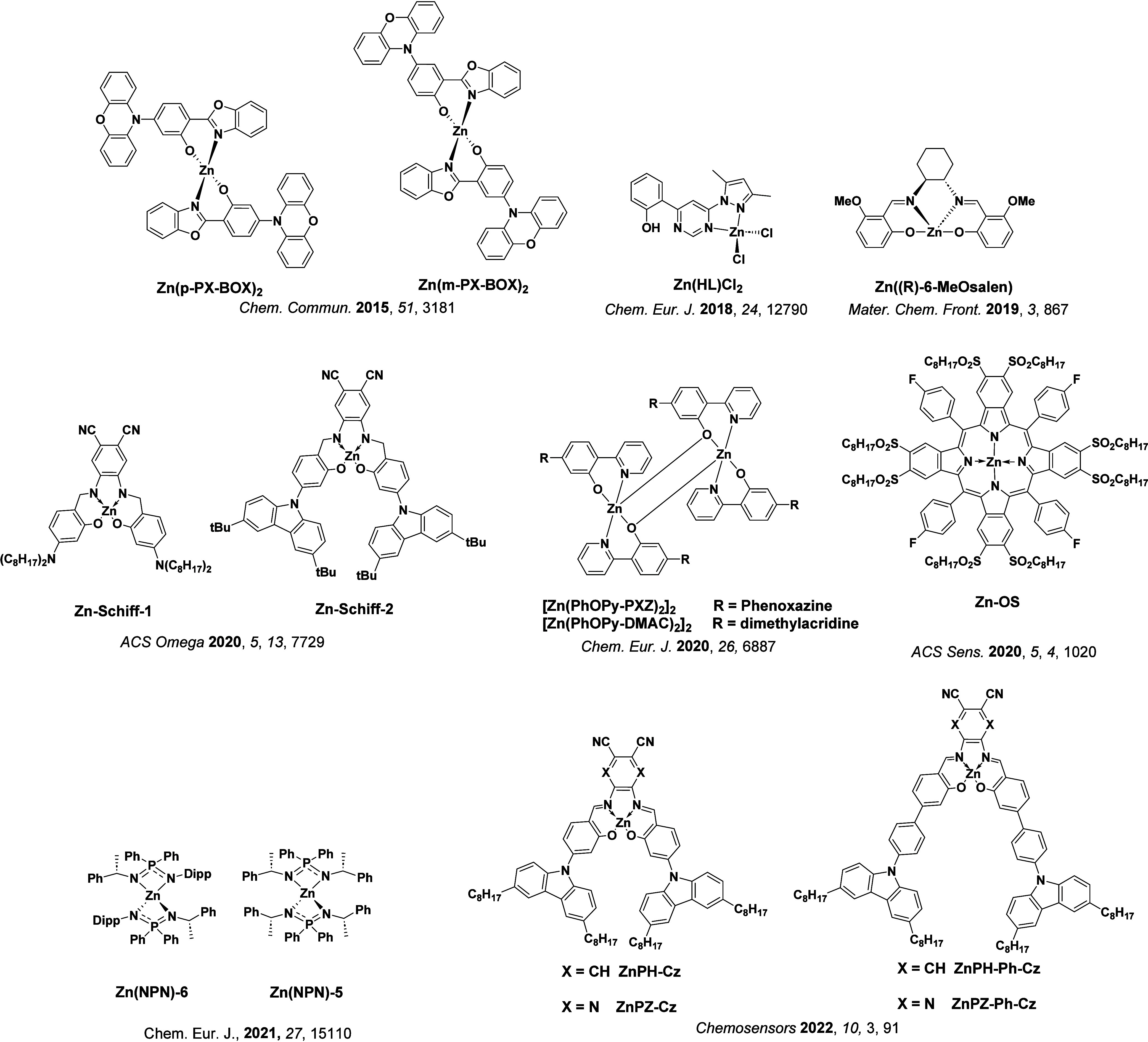
Chemical structures of TADF zinc(II) complexes.

The same strategy of metal complexation enhancing the TADF in donor-acceptor ligands was also invoked in the dimeric zinc complexes **[Zn(PhOPy-PXZ)_2_]_2_** and **[Zn(PhOPy-DMAC)_2_]_2_** (Figure 116).^938^ These two complexes showed interesting luminescence polymorphism, with different emission observed for powders (λ_PL_ of 538 and 497 nm,), crystals (500 and 444 nm), and ground powders (532 and 473 nm, all respectively). Pristine powders of **[Zn(PhOPy-PXZ)_2_]_2_** and **[Zn(PhOPy-DMAC)_2_]_2_** emit at λ_PL_ of 538 and 497 nm, have Φ_PL_ of 13 and 50%, and τ_PL_ of 2.09 and 2.45 μs, respectively. The calculated Δ*E*_ST_ of 70 and 100 meV and the temperature dependence of the TRPL both indicate that these complexes are TADF-active.^938^

The complex **Zn(HL)Cl_2_** (Figure 116) shows excitation-wavelength dependent emission, ESIPT, and TADF.^939^ As a powder, low energy excitation at 480 nm results in yellow TADF emission (λ_PL_ = 565 nm, Φ_PL_ = 7%, τ_PL_ = 6.0 μs, and Δ*E*_ST_ = 11 meV), while excitation at 380 nm results in emission at λ_PL_ of 640 nm accompanied by a significant drop in Φ_PL_ to 0.02%. Computational studies showed that either the *keto* or *enol* tautomer can be the most stable form of the molecule, depending on the excited state. Excitation at 480 nm to S_1_ of the *enol* form leads to ESIPT to the *keto* form, followed by both prompt fluorescence and TADF. In contrast, upon excitation at 380 nm to S_n>1_, the *enol* form of the complex undergoes rapid ISC to the *enol* form’s T_n_ and then subsequent ESIPT the *keto* T_1_. As there is no initial photoexcitation of the *keto* form S_1_ excited state there is no prompt emission, and all the emission is either phosphorescence from *keto* T_1_ and/or TADF from *keto* S_1_.

Chen *et al*. reported the use of the chiral TADF Zn(II) salen complex **Zn((R/S)-6-MeOsalen)** (Figure 116) as an emitter in CP-OLEDs.^940^ In THF the complex emits at λ_PL_ of 491 nm while in the neat film it showed dual emission consisting of a shoulder at 490 nm and a more intense band at λ_PL_ of 576 nm. The low-energy band was assigned to excimer emission, and was much longer-lived than the nanosecond fluorescence of the band at 490 nm, with τ_PL_ of 8.42 μs for **Zn((R)-6-MeOsalen)** and 7.39 μs for **Zn((S)-6-MeOsalen)**. CP-OLED devices showed an EQE_max_ of only ca. 0.04%, however the g_EL_ values were high on the order of 10^–2^.

Two zinc(II) Schiff base complexes with microsecond-long τ_PL_ were used as emission lifetime based optical temperature sensors.^941^ Both complexes are composed of a phthalonitrile acceptor unit and either an *N,N’*-dialkyl­ani­line (**Zn-Schiff-1**) or a di-*tert*-but­yl­car­ba­zole (**Zn-Schiff-2**) donor (Figure 116). Use of a stronger donor moiety in **Zn-Schiff-2** compared to **Zn-Schiff-1** resulted in a small red-shift in the emission (from λ_PL_ = 542 to 547 nm), a smaller Δ*E*_ST_ (from 310 to 280 meV) and a shorter τ_PL_ (from 2.1 ms to 435 μs). Delayed fluorescence was estimated to make up 30% of the total Φ_PL_ for **Zn-Schiff-2**, while this was more difficult to quantify for **Zn-Schiff-1** due to its long excited state lifetime but was nonetheless estimated to make up approximately 16% of the total Φ_PL_. More recently, similarly structured complexes of were studied^942^ containing a phenyl spacer between the donor and the Schiff base ligand backbone. The incorporation of the phenyl spacer in **ZnPH-Cz** and **ZnPH-Ph-Cz** results in a larger Δ*E*_ST_ compared with **Zn-Schiff-1** and **Zn-Schiff-2**, while introduction of a stronger 2,3-pyrazinedicarbonitrile acceptor in **ZnPZ-Cz** and **ZnPZ-Ph-Cz** produces a smaller Δ*E*_ST_. Complexes **ZnPZ-Cz** and **ZnPZ-Ph-Cz** have shorter τ_PL_ of 114 and 236 μs compared with **ZnPH-Cz** and **ZnPH-Ph-Cz** (τ_PL_ = 945 and 1040 μs, all respectively), both of which exhibited τ_PL_ closer to those of **Zn-Schiff-1** and **Zn-Schiff-2**. The presence of the strong acceptor also significantly reduced the Φ_PL_, particularly when combined with the phenyl spacer, falling from 37% for **ZnPH-Cz** to 1.9% for **ZnPZ-Ph-Cz**. This behavior can be rationalized by the larger Δ*E*_ST_ in **ZnPZ-Ph-Cz** and increased non-radiative decay in this complex due to greater conformational flexibility in the ligand.

A zinc porphyrin complex, **Zn-OS** (Figure 116), was used as a dual oxygen and temperature sensor.^943^ This complex emits at λ_PL_ of 667 nm and has a τ_d_ > 1 ms. As the τ_d_ and the intensity of the prompt and delayed fluorescence (*I*_DF_/*I*_PF_) vary differently with temperature and oxygen quenching, an empirical model was built to measure both parameters from a single measurement.

Goswami *et al*. reported blue-emitting (λ_PL_ = 480 nm) iminophosphonamide zinc complex **Zn-NPN-5** (Figure 116) that has a Δ*E*_ST_ of 120 meV.^944^ Interestingly, a very similar complex **Zn-NPN-6** was found to have no delayed emission. Analysis of single crystals of both complexes revealed that the Zn(II) center of **Zn-NPN-6** adopts a square planar geometry while **Zn-NPN-5** appears to coordinate in a distorted tetrahedron. The square planar geometry was proposed to be the origin of the poor triplet formation in **Zn-NPN-6**, hindering intraligand charge transfer. Interestingly, TADF was observed in a planar dinuclear copper complexes in the same study, for which the authors attributed the improved ISC/RISC to an enhanced SOC in this system.

### 9.8 Other Metals

Examples of TADF emitters containing metals other than those discussed above exist in smaller numbers. Among the first of these were a series of tin(IV) porphyrin complexes (Figure 117).^32^ A sufficiently small Δ*E*_ST_ of 240 meV in **SnF_2_-OEP** resulted in dominant TADF emission, although some phosphorescence was also detected at room temperature. Temperature-dependent Φ_PL_ was used to assign the emission as TADF (Φ_PL_ increasing from 0.6% at 300 K to 2.4% at 400 K). Devices were fabricated and although no EQE_max_ was mentioned, upon electrical excitation a prompt and temperature-dependent delayed emission were observed. This report by Adachi and co-workers was one of the first examples of the TADF mechanism being applied (knowingly) to OLEDs.

**Figure 117:**
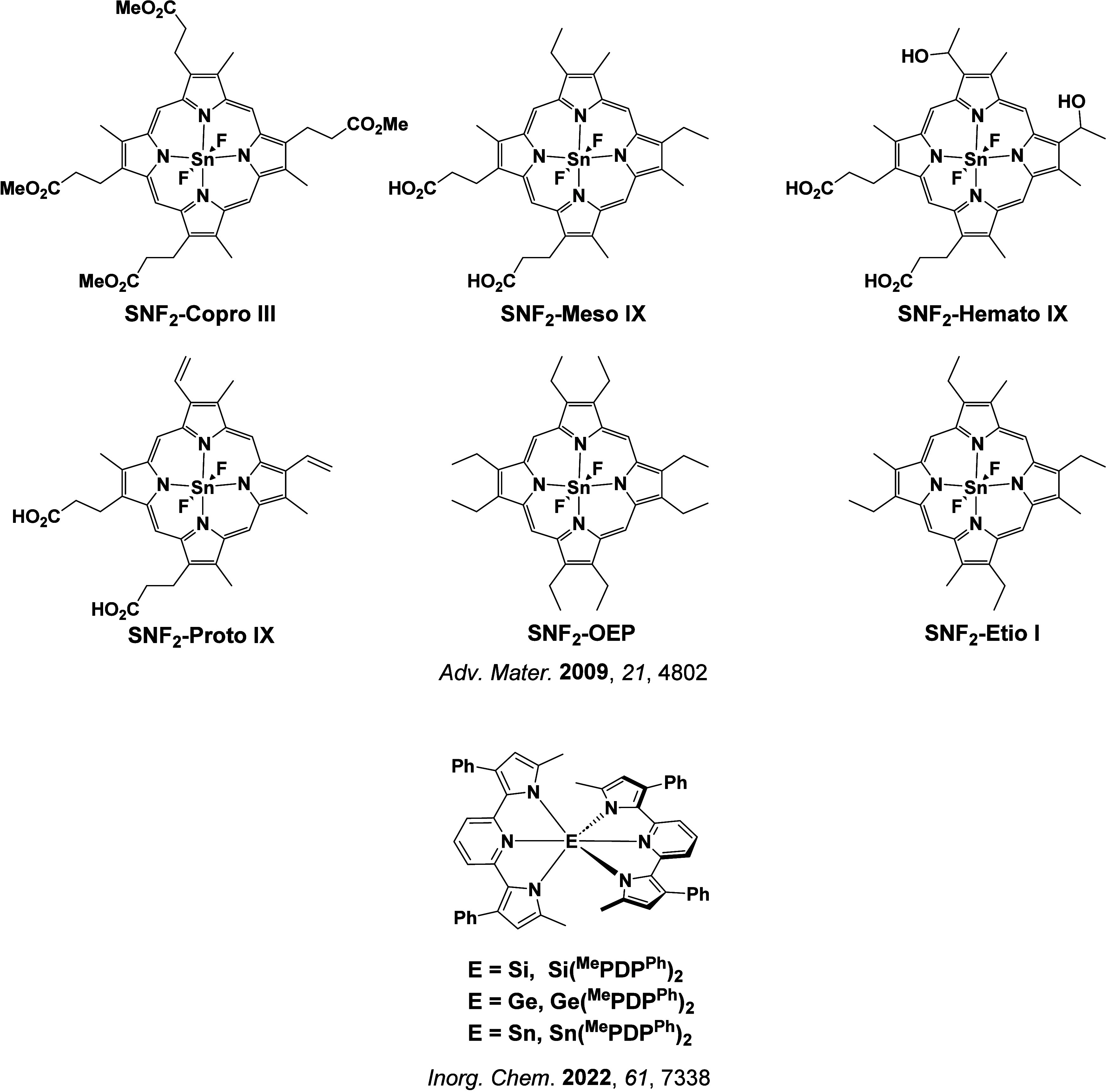
Chemical structures of tin porphyrin and other porphyrin-based group 14 metal complexes having TADF properties.

The only other report of TADF Sn complexes since this early discovery comes from Gowda *et al.*, who prepared a series of three main group polypyrrole complexes, **Si(^Me^PDP^Ph^)_2_**, **Ge(^Me^PDP^Ph^)_2_**, and **Sn(^Me^PDP^Ph^)_2_** (Figure 117).^945^ The complexes are green emitters in THF and all show TADF. Moving down the group the emission color blue-shifts from a λ_PL_ of 527 nm for **Si(^Me^PDP^Ph^)_2_** to 512 nm for **Sn(^Me^PDP^Ph^)_2_**, and the τ_PL_ increases from 0.9 ms for **Si(^Me^PDP^Ph^)_2_** to 2.0 ms for **Sn(^Me^PDP^Ph^)_2_** while the Φ_PL_ ranges between 32 and 49% for the three complexes. The Δ*E*_ST_ of the three complexes are 243, 260, and 313 meV for the lightest to the heaviest analogue. The *k*_ISC_ for the three complexes was also measured by transient absorption spectroscopy, increasing from 3.23 × 10^8^ to 4.0 × 10^9^ s^–1^ when moving from Si to Sn. This recent study is the first comparing complexes of the different group 14 elements as TADF emitters, and one of few investigating the photophysical properties of metalloid and post transition metal complexes.

There are also a small number of early transition metal complexes that exhibit TADF (Figure 118). A series of tungsten(0) isocyanide complexes of the form **w(CNdippR)_6_** were shown to have yellow to red emission and were used as photocatalysts due to their large excited state reduction potentials.^946^ In toluene these complexes have Φ_PL_ of around 40% and τ_PL_ of *ca.* 1.5 μs. The emission was shown to originate from a MLCT excited state with temperature-dependent emission lifetimes. The tungsten(VI) Schiff base complex **W(O)_2_(N-Ar_3_-Salen)** also emits via TADF, from a mixed LLCT/MLCT excited state.^947^ The calculated small Δ*E*_ST_ of 93 meV provides support for the assignment of TADF. The presence of methyl groups on the xylyl linker were essential to promote a much more strongly twisted conformation, and to spatially separate the electron densities of the HOMO and LUMO, and an analogous complex using a phenyl linker between the Salen and diarylamine has a much larger Δ*E*_ST_ of 347 meV. OLEDs with **W(O)_2_(N-Ar_3_-Salen)** showed EQE_max_ of 15.6% at CIE coordinates of (0.49, 0.49), and moderate efficiency roll-off (EQE_1000_ = 9.7%).

**Figure 118:**
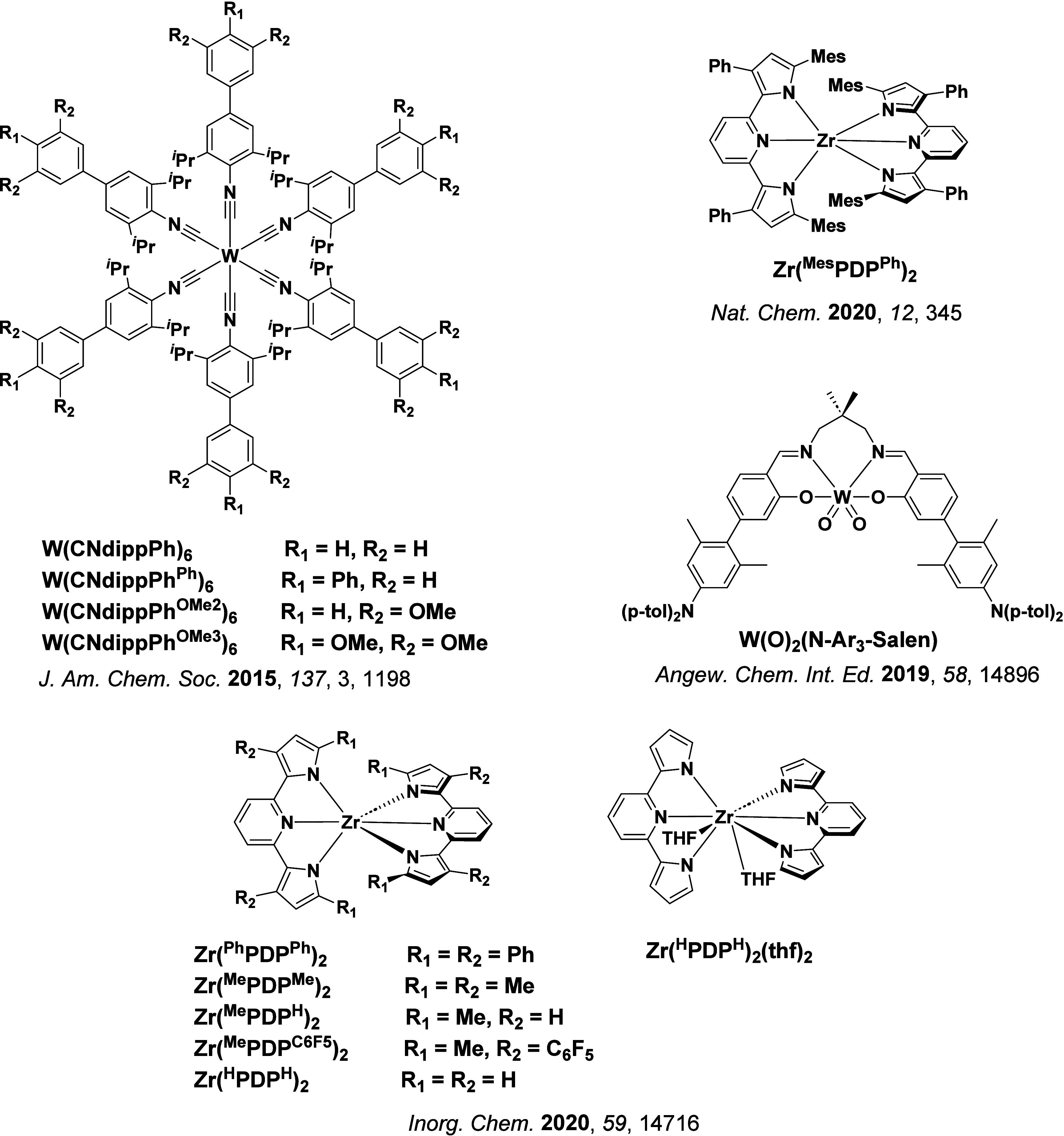
Chemical structures of TADF tungsten and zirconium complexes.

Millsman and co-workers reported a TADF-active zirconium(IV) complex. **Zr(^Mes^PDP^Ph^_)2_** (Figure 118) shows bright yellow emission in solution (λ_PL_ = 581 nm and Φ_PL_ = 45%) and has a long emission lifetime of 350 μs.^948^ Calculations revealed that the emissive excited state has mixed IL/LMCT character, and the negligible calculated TDM in the excited state helped to explain the lack of solvatochromism. Temperature-dependent emission studies supported the identification of TADF, alongside a Δ*E*_ST_ of 200 meV. The complex was used as a photocatalyst in a number of different reactions,^948^ and the same group subsequently reported six TADF complexes containing different substituents on the same pyridyl-dipyrrolide core.^949^ One of these, **Zr(^Me^PDP^Ph^)_2_**, had previously been reported without identification of its emission mechanism as TADF.^950^ These complexes are yellow to orange emitters in benzene with λ_PL_ of 568 to 629 nm, are moderately emissive (Φ_PL_ of 10 to 38%), and have long τ_PL_ of 190 to 576 μs.

Examples of TADF emitters incorporating both alkali metals and aluminum have also been reported (Figure 119). Compounds **Mg(p-PX-BOX)_2_** and **Li(p-PX-BOX)** emit at λ_PL_ of 510 and 516 nm, respectively and show a slightly blue-shifted emission relative to previously discussed **Zn(p-PX-BOX)_2_** (λ_PL_ = 542 nm) in 6 wt% doped films in mCBP. All three complexes have high Φ_PL_, ranging from 70 to 78%, and very small Δ*E*_ST_ of 60 to 80 meV.^937^ Green OLEDs with **Mg(p-PX-BOX)_2_** and **Li(p-PX-BOX)** showed EQE_max_ of 16.5 and 12.9% respectively, slightly lower than the EQE_max_ of 19.6% reported for **Zn(p-PX-BOX)**. The same study documented the first aluminum TADF emitter **[Al(p-PX-BOX)_2_(μ-OH)_2_]** (λ_PL_ = 530 nm and Φ_PL_= 86.7% in 6 wt% doped films in mCBP) that showed temperature dependent intensity of the delayed emission. The Δ*E*_ST_ of **[Al(p-PX-BOX)_2_(μ-OH)_2_]** is 60 meV in 2-MeTHF glass. The aluminum complex is thermally unstable though, and all films and devices were prepared by solution processing in contrast to the thermal evaporation methods used for the zinc, lithium, and magnesium complexes with the same ligand. The OLED showed an EQE_max_ of 6.8% at λ_EL_ of 505 nm.^937^

**Figure 119:**
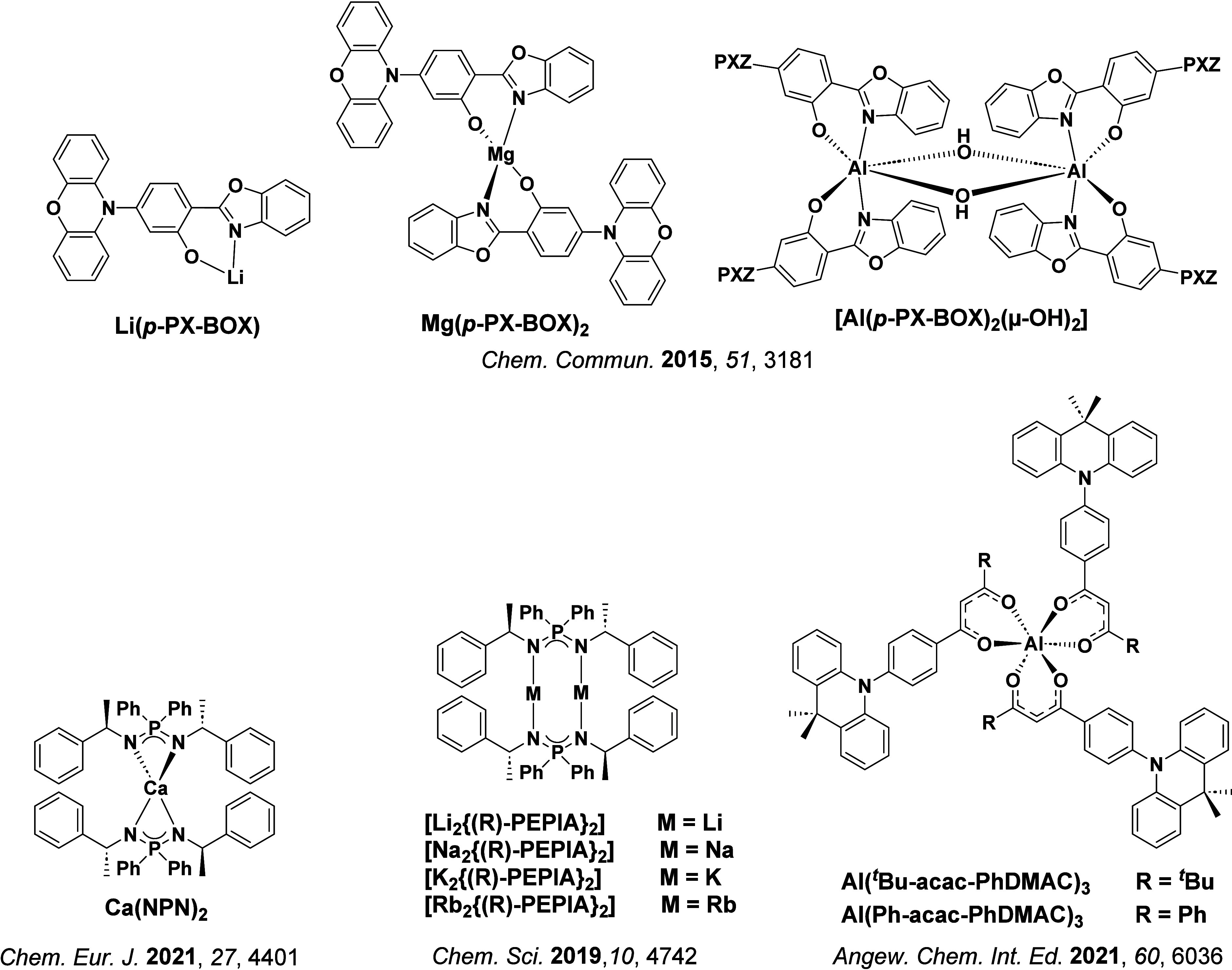
Chemical structures of TADF alkali metal and aluminum complexes.

A series of emitters based on dimeric alkali metal complexes with enantiopure imino­phos­phon­amide ligands of the form **[M_2_((R)-PEPIA)_2_]** have been reported (Figure 119).^951^ The complexes are blue emitters as neat films with low Φ_PL_ ranging from 8–21%, and τ_PL_ between 4.1 to 14.8 μs with small Δ*E*_ST_ values ranging from 73 to 90 meV. There were no clear trends in the reported optoelectronic properties of the complexes. The same imino­phos­phon­amide ligand was later used in a monometallic calcium complex **Ca((R)-PEPIA)_2_** [or **Ca(NPN)_2_**] that also showed TADF.^952^ The complex is a blue-green emitter as a neat film, has a Φ_PL_ of 22%, τ_PL_ of 24 μs and a Δ*E*_ST_ of −148 meV – approximately double that of the related dinuclear alkali metal complexes.

The first family of mononuclear aluminum complexes to show TADF, of the form **Al(R-acac-PhDMAC)_3_**, has recently been reported (Figure 119).^953^ The asymmetric acetylacetonate ligands showed weak TADF emission without complexation in 30 wt% doped films in CBP. Upon coordination to the aluminum, changes in the dihedral angle between the DMAC donor and the remainder of the molecule result in a decrease of the Δ*E*_ST_. The complexes are green emitters (λ_PL_ = 495–534 nm) in toluene, and 30 wt% doped CBP films have τ_PL_ < 4 μs with Φ_PL_ ranging from 32 to 79%. Solution-processed OLEDs with **Al(Ph-acac-PhDMAC)_3_** showed an EQE_max_ of 17.5% at CIE coordinates of (0.43, 0.55), and had small efficiency roll-off (EQE_1000_ = 14.7%).

Iridium(III) complexes are typically employed as phosphorescent emitters in OLEDs (PhOLEDs) due to their large SOC values.^954^ However, introduction of TADF-emitting ligands can produce some interesting dual-emissive complexes (Figure 120). Benjamin *et al*. reported complexes **Ir-5** and **Ir-6** that show dual TADF and phosphorescent emission in polar solvents, originating from ^3^CT and ^3^MLCT states respectively.^955^ The blue-emitting OLEDs with **Ir-5** and **Ir-6** however showed very low EQE_max_ of 1.5 and 2.1%, respectively.

**Figure 120:**
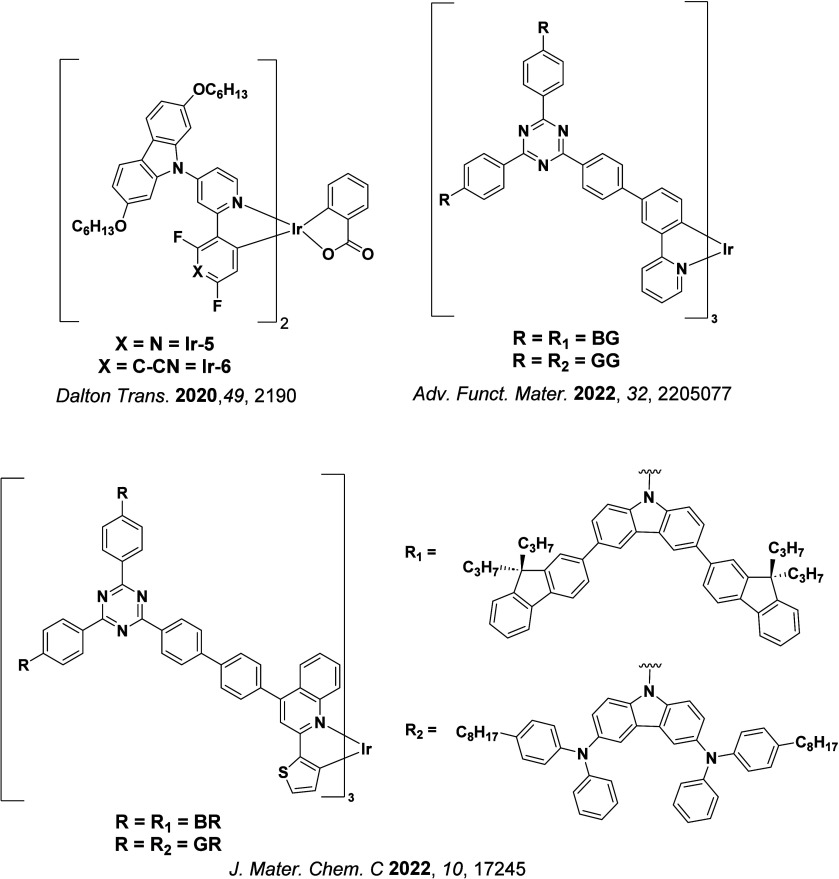
Chemical structures of TADF iridium(III) complexes.

Thamarappalli *et al.* reported two dual-emitting complexes that incorporate TADF dendrimers onto a Ir(ppy)_3_ core: **BG** and **GG** (Figure 120).^956^ In solution the complexes showed dual TADF and phosphorescent emission, and as the solvent polarity increased from cyclohexane to toluene to DCM, the contribution of the TADF emission increased as the CT excited state associated with the TADF dendrimer moiety was stabilized. An energy transfer process between the TADF donor dendrons and the phosphorescent core was confirmed by PL measurements in solutions of varying solvent and temperature. This suggests that energy transfer from long-lived excitons in TADF dendrimers can be harvested and managed through faster radiative decay of a phosphorescent core. Both non-doped and doped OLEDs showed green emission, with the non-doped OLEDs of **GG** showing an EQE_max_ of 4.7% at CIE coordinates of (0.48, 0.51). The device with **BG** showed similar EQE_max_ of 4.0% at CIE coordinates of (0.42, 0.56). Devices using doped emissive layers (0.4 mol% in mCPCN) showed higher efficiencies, with the **GG** device showing an EQE_max_ of 9.8% at CIE coordinates of (0.41, 0.56), and **BG** showing an EQE_max_ of 15.1% at CIE coordinates of (0.32, 0.63).

Similar structures have also been reported by Jang *et al*. employing blue-emitting (**BR**) and green-emitting **(GR**) TADF dendrons attached to a *fac*-tris[2-(thio­phen-2-yl)-4-(p-tol­yl)­quin­o­lin­ato]­iridium(III) (**TQIr**) phosphorescent core (Figure 120).^957^ In solution dual emission was observed from both the TADF donor dendrons and the iridium core, with the ratio between the emissions varying as a function of the polarity of the solvent. Both non-doped and doped OLEDs in TCTA showed red emission, with the non-doped **BR** OLEDs having an EQE_max_ of 2.6% at CIE coordinates of (0.68, 0.32), while the devices with **GR** showed an EQE_max_ of 0.9% at CIE coordinates of (0.67, 0.33). Devices using doped emissive layers (0.1 mol% in TCTA) showed higher efficiencies, with the **BR** OLED showing an EQE_max_ of 13.5% at CIE coordinates of (0.66, 0.34), and **GR** showing an EQE_max_ of 11.0% at CIE coordinates of (0.66, 0.35).

### 9.9 Outlook

Metal complexes whose reported emission originates in whole or in part from TADF are growing rapidly in number and show promise as emissive materials for OLEDs as well as in other applications such as photocatalysis (Section 23) and LECs (Section 16). In comparison to organic TADF materials, metal complexes can have much faster *k*ISC and *k*RISC, which can lead to very short emission lifetimes even with relatively light (and abundant) metals. The complicated origins of the emission from metal complexes, often a mixture of phosphorescence and TADF, means that special care must be taken when interpreting and reporting their photophysics. Nonetheless, the photophysical properties of metal complexes can be readily tuned by varying the electronic and steric properties of the ligand(s), giving rise to a rainbow of interesting and useful luminescent materials.

Currently the most promising classes of complexes for use in OLEDs are the coinage metal CMA and gold(III) complexes. Both have excellent optoelectronic properties that translate to high-efficiency devices on-par with state-of-the-art iridium OLEDs. Since the first report of coinage metal CMA complexes in 2017,^194^ a significant body of work on the design, synthesis, and characterization of these complexes has demonstrated their high optoelectronic performance as well as color tuning. This is exemplified in the complexes **Au_bim_^PZI^**, **Au_bim_^BZI^**,**Au_bim_^PAC^**, and **Au_bim_^BZAC^** that have τ_PL_ of 240 to 280 ns, as well as by OLEDs with **CMA4** that showed an EQEmax of 27.5% at CIE coordinates of (0.36, 0.54).^194^ Separately, while gold(III) complexes have long been thought to be purely phosphorescent, efforts to elicit TADF activity have led to OLEDs using **Au-4** showing an EQEmax of 26.8% at CIE coordinates of (0.36, 0.60), and long device lifetime of LT90 = 674 h (at an initial 1,000 cd m^–2^).

Separately, metal TADF complexes that show switchable emission based upon metal coordination are particularly relevant in the context of bioimaging and sensing (Sections 20 and 21). Underpinning such utility, several families of silver, zinc, and alkali metal complexes summarized in this section feature a geometry change of the ligand upon coordination of a metal centre, resulting in TADF-inactive ligands ‘turning on’ in response to external stimuli. Further refinement of this strategy should enable the design of new sensors, detectors, and bioimaging reagents that optically report on changes in solvent environment, or metal ion concentrations.

Combining TADF properties with the synthetic flexibility and tunable emission properties of organometallic complexes in TADF metal complexes may indeed be a viable path towards achieving the impressive performance and color targets currently available in PHOLEDs. By instead exploiting RISC and TADF, either in the ligands or in CT states, this performance may also be achievable in materials that do not rely on the heaviest and scarcest elements that promote SOC and phosphorescence, providing clear advantages in terms of device cost and sustainability.

## 10 Macromolecules TADF

### 10.1 TADF Polymers

Sections 3–9 have largely focused on low molecular weight, small molecule emitters that can be vacuum-deposited during device fabrication. This section, by contrast, summarizes the advances in macromolecules, polymers and dendrimer that show TADF, which have been designed to be processed from solution, such as by spin-coating or inkjet printing, in the context of solution-processed OLEDs. TADF polymers have emerged as a promising class of emitter materials that can be used to achieve high-performance solution-processed OLEDs (SP-OLEDs). The use of polymers as emitters had previously been explored widely for both solution-processed fluorescent and phosphorescent emitters. The major advantage of SP-OLEDs is a considerable reduction in energy use and production costs compared to thermally vacuum-deposited small molecule-based OLED materials. Furthermore, polymers can easily be designed and synthesized to incorporate various optoelectronic functional units into or pendant from the polymer backbone, such as emitters, hosts, spacers, solubilizing groups, and charge-transporting moieties. Precise control of the polymerization then allows adjustable ratios of these units to be achieved in such a way that phase separation can be avoided within the polymer chains and where the polymer can be used neat within the EML. Polymeric materials also frequently display excellent film-forming properties, allowing them in some cases to outperform SP-OLEDs containing low molecular weight emitters (i.e., small molecules). Beside their use as emitters, TADF polymers can also act as host matrices for small molecule emitter dopants, enabling triplet-harvesting from the host in these SP-OLEDs.^958^

There are a range of design strategies for TADF polymers, typically involving combinations of donor (D) and acceptor (A) components. How these subunits are engineered to interact varies according to a collection of identifiable strategies: 1) A known D-A type TADF emitter can be coupled directly to a non-conjugated or weakly conjugated polymer backbone, thus acting as a functional pendant group; 2) The polymer backbone itself can be composed of repeating donor units, which are directly coupled with acceptor components acting as pendant groups to form D-A emissive sites; 3) The polymer main chain can be composed of alternating donor and acceptor units; 4) Both donor and acceptor groups can be installed as separate pendant groups on a non-conjugated backbone, producing TADF by a through-space charge transfer interaction. Other functional groups can also be added either as pendant groups or as part of the chain to act as hosts or as non-conjugated spacers, ensuring good charge balance and triplet confinement. While these specific strategies are the most frequently reported and hence the ones highlighted in this section, this list, with properties summarized in Table S13, is by no means exhaustive. The considerable breadth and sophistication of modern polymer chemistry combined with the combinatorial nature of D-A TADF emitters allows for practically limitless innovation in this area.^959^

#### 10.1.1 Polymers with Pendant TADF Emitters

One of the simplest design strategies for TADF polymers is to attach known small-molecule TADF emitters as pendant groups to the main chain of the polymer. This main chain can be optoelectronically inert or active, and the final material can be prepared by either post-synthetic modification of the main chain polymer, or by polymerizing monomers that contain an embedded TADF motif. For example, by grafting a TADF emitter **PXZ-DP-Cz** onto a polycarbazole backbone, Xie *et al*.^960^ reported a series of efficient bluish-green polymers **PCzDP-x** (Figure 121). The polycarbazole backbone in this case not only improved the charge transport compared to aliphatic chains, additionally it also acts as a host due to its high T_1_ level so as not to quench triplet excitons of the TADF emitter group. Furthermore, incorporating *N*-eth­yl­hex­yl­car­ba­zole or *N*-hex­yl­car­ba­zole substituted monomers into the main chain electronically isolates the pendant TADF emitter and prevented aggregation-caused quenching (ACQ) in the ‘self-hosting’ non-doped polymer. Polymer **PCzDP-10** contained 10% mole fraction of TADF-containing monomers and showed the highest delayed contribution to the total emission (72%, τ_d_ = 2 μs). The Φ_PL_ are as high as 67% in toluene and 74% in the neat film, indicating their promise as materials in SP-OLEDs. The non-doped SP-OLED with **PCzDP**-**10** showed an EQE_max_ of 2.8%, although this increased to 5.9% when **PCzDP**-**10** was used in conjunction with mCP in a 1:1 ratio. By incorporating an additional small molecule TADF emitter **DMAC-DP-Cz** as a sensitizer a considerably higher EQE_max_ of 16.1% at a luminance of around 100 cd·m^–2^ was achieved (Table S13), all while maintaining CIE coordinates clustered around (0.24, 0.40).

**Figure 121:**
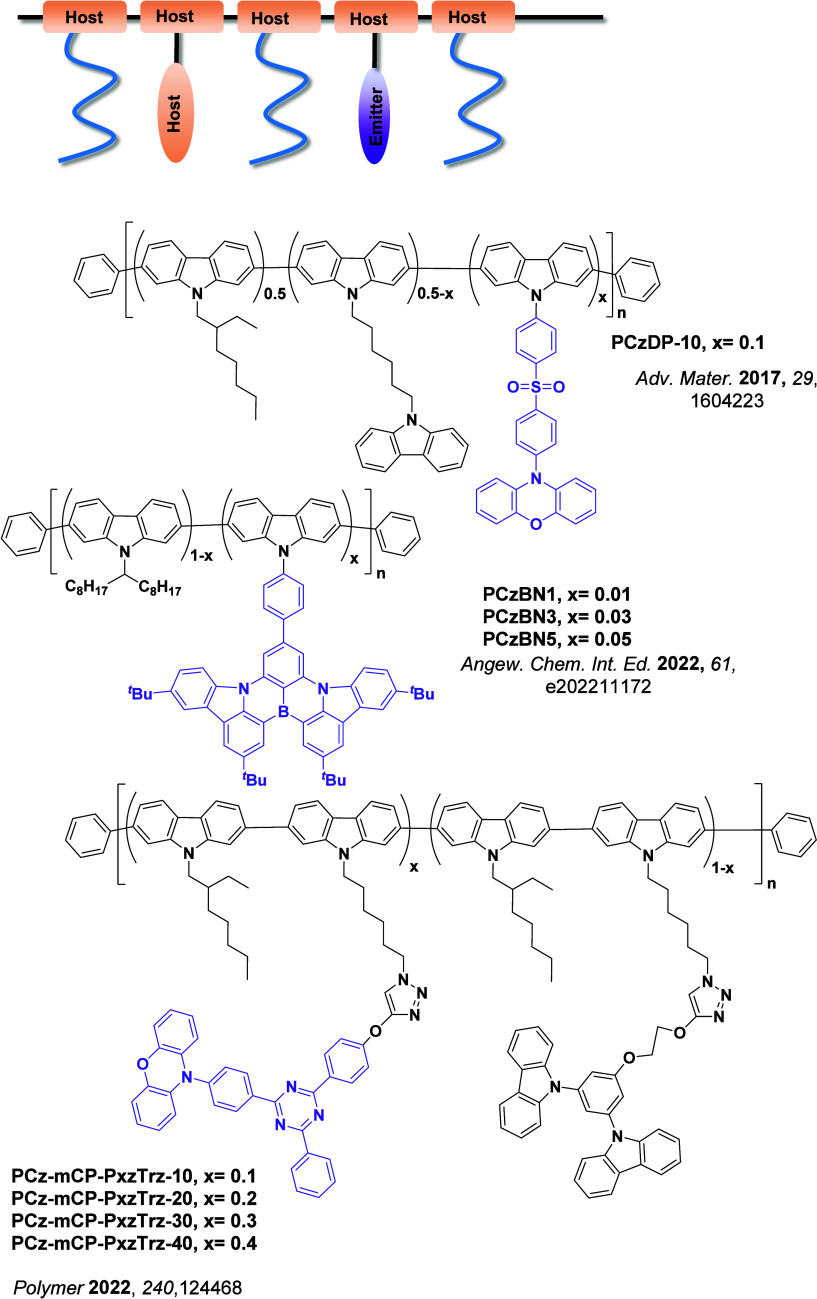
Schematic diagram and example chemical structures of conjugated polymers with pendant TADF emitter groups. All examples consist of a host type motif incorporated in the polymer backbone along with known TADF moieties attached to the backbone as side chain (purple).

Using a similar approach, the same group reported the design and synthesis of a MR-TADF polymer, using a green MR-TADF emitter (**BN**) as a pendant group onto a polycarbazole backbone with polymers of molecular weights ranging from 4.4–10.0 kDa and polydispersity indices (PDI) of between 1.40–1.87. Among the doped SP-OLEDs (60 wt% polymer in mCP), those with polymers **PCzBN1** and **PCzBN3**, with 1 or 3% of emitter-containing monomers, showed the best performance with EQE_max_ of 17.8 and 17.3%, respectively (Table S13). The SP-OLED with **PCzBN5**, containing 5% emitter monomer, showed a lower EQE_max_ of 13.3%. This decrease was attributed to increased ACQ due to the lower content of alkyl-carbazole monomers, which act to separated emissive monomers and improved solubility. The CIE coordinates of the SP-OLEDs with **PCzBN1**, **PCzBN3**, and **PCzBN5** were (0.10, 0.43), (0.12, 0.54), and (0.11, 0.53), respectively. Compared to an EQE_max_ of 16.3% for SP-OLED based on just the use of **BN** as the emitter, the EQE_max_ for the polymer-based devices with **PCzBN1** and **PCzBN3** increased to 17.8 and 17.3%, respectively. This enhancement in device performance was attributed to the improved solubility and film morphology of the polymer compared to its monomer counterpart, while maintaining the same emission color and TADF performance. The intrinsic advantage of MR-TADF emitters was also conferred to the polymer OLEDs, with a resulting narrowband emission (FWHM of around 30 nm) for all fabricated devices.^961^

Following the same design strategy, Zong *et al*.^962^ grafted both TADF and host units as pendants onto a polycarbazole backbone in an effort to decrease ACQ. Together with small-molecule TADF emitter **PXZ-TRZ**, “self-hosted” TADF polymers containing a modified mCP monomer (Figure 121) were synthesized with molecular weights of between 7.5–8.0 kDa and PDIs of between 1.5–1.6. Polymers **PCz-mCP-PxzTrz-x** with varying portions of emitter molecules (**x**, ranging from 10 to 40%) were produced, with **PCz-mCP-PxzTrz-30** (30% emitter) showing the highest Φ_PL_ of 39% in the neat film (in air) at a λ_PL_ of 540 nm. The non-doped SP-OLED showed an EQE_max_ of 15.3% and CIE coordinates of (0.35, 0.53). These polymer properties compare favorably to the intrinsic TADF performance of the **PXZ-TRZ**, reflected in the lower EQE_max_ of 12.5% in a doped vacuum-deposited device (6 wt% in CBP).^963^

To raise the triplet energy of the polymer backbone, which is especially important for blue emission using conjugated polymers like polyfluorene, Yang *et al*.^964^ inserted a 3,3′-dimethyldiphenyl ether group into the backbone to regulate the conjugation length. As a result, the triplet energy of the polymer increased from 2.16 to 2.58 eV for **PFDMPE-R01** to **PFDMPE-R10** (Figure 122) as the ratio of the nonconjugated ether component increased. The synthesized polymers had molecular weights of between 83–132 kDa with PDIs of between 1.6–1.8. When a red TADF emitter **ROC8** ^964^ was introduced as a pendant unit, effective energy transfer from the backbone to the grafted emitter was achieved, with all the polymers showing red emission similar to that of **ROC8**. The Φ_PL_ of neat films of the polymers improved from 18 to 55% with increasing ether component. The device performance with **PFDMPE-R05** (Figure 122, with 5% mole fraction of emitter-containing monomers) was the best in the study, with an EQE_max_ of 5.6% at λ_EL_ of 606 nm. Severe efficiency roll-off, decreasing by 82% at 500 cd·m^–2^, was reported and attributed to the long τ_d_ of 126 μs, thus allowing triplet quenching processes to dominate at higher luminance.

**Figure 122:**
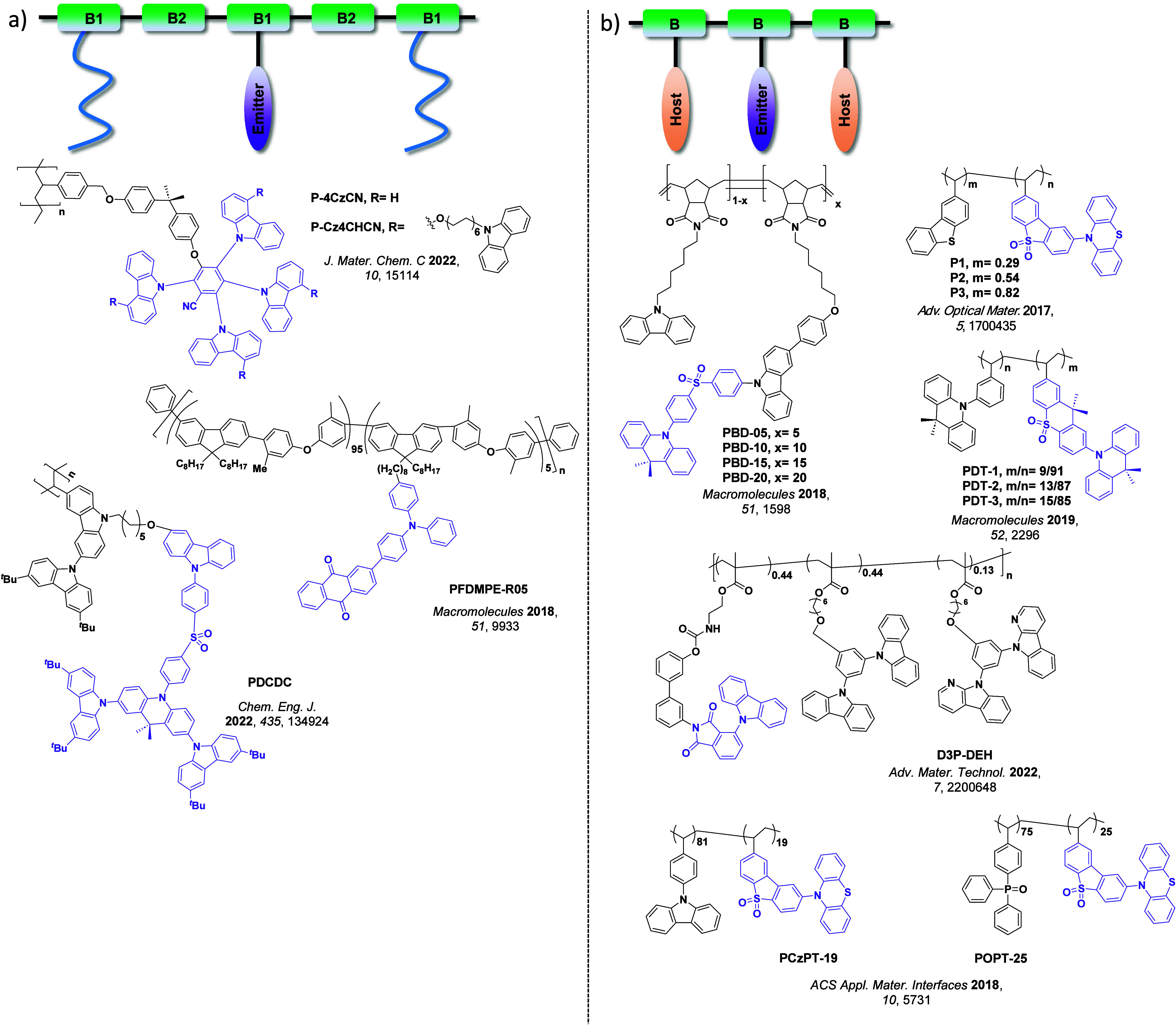
Schematic diagram and chemical structures of a) non-conjugated polymers containing pendant TADF groups and b) non-conjugated polymers containing pendant TADF and host groups. In both cases, the polymer backbone consists of a non-conjugated chain on which a known TADF emitter is the side chain (purple). In b, these TADF subunits are installed alternating with host units to improve the charge transport properties of the polymer.

Ban *et al*.^965^ similarly used a non-conjugated alkyl backbone alongside modified **4CzCN** units to create a TADF polymer with a molecular weight of 7.5 kDa and a PDI of 1.5. The bulky **4CzCN** units also showed AIE and bestowed the polymer **P-4CzCN** (Figure 122) with neat film Φ_PL_ of 37% at λ_PL_ of 472 nm and a τ_d_ of 1.5 μs. Non-doped SP-OLEDs showed an EQE_max_ of only 3.6% with CIE coordinates of (0.23, 0.39). However, when non-conjugated alkyl-carbazole were attached to the emitter unit in a semi-dendritic fashion (**P-Cz4CzCN**, Figure 122), the increased encapsulation of the TADF-core in the self-hosting monomers led to much higher neat film Φ_PL_ of 65% at λ_PL_ of 489 nm (Table S13). The EQE_max_ of the non-doped SP-OLEDs also increased significantly to 11.5%, with somewhat similar CIE coordinates of (0.24, 0.47). As well as presumably alleviating ACQ between emissive monomers, films of the encapsulated dendrimeric TADF polymer were also much more resistant to application of orthogonal solvent, which potentially simplifies the production of fully SP-OLEDs by allowing layers above the EML to also be processed from solution.

A similar approach was reported by Li *et al*.^966^ using a non-conjugated backbone decorated with carbazole groups, themselves terminally functionalized with an asymmetric dendritic TADF emitter unit (**DMAC-DPS-Cz**). The resulting polymer **PDCDC** (Figure 122), with a molecular weight of 12.7 kDa and a narrow PDI of only 1.16, showed a greenish-blue emission at 496 nm and a Φ_PL_ of 68% in neat films (Table S13). The non-doped SP-OLEDs showed an EQE_max_ of 9.0% at CIE coordinates of (0.23, 0.39). Comparing this material to previous examples, it becomes clear that careful tuning of the overall carbazole content – either through monomer constitution by incorporating host moieties like mCP in the polymer by copolymerization, or simply by blending the polymers with a supporting host – is vital to ensure bright TADF polymer films.

Polyolefin is an alternative non-conjugated backbone that has also been explored in TADF polymers. Using a monomer containing the emitter **DBTO2-PTZ**, Li *et al*.^967^ developed a series of TADF copolymers **PCzPT-x** and **POPT-x** containing either hole-transporting carbazole and electron-transporting phosphine oxide spacer monomers, respectively. These copolymers showed relatively small Δ*E*_ST_ values between 0.05–0.13 eV and Φ_PL_ up to 36%. **POPT-25** and **PCzPT-19** (with number representing mole fraction of emitter monomer, Figure 122) were identified as the best performing of their respective series among the **POPT-x** and **PCzPT-x** polymers, with molecular weights of 27 and 16 kDa and PDIs of 1.5 and 1.8, respectively. The phosphine oxide pendant of the polymer **POPT-25** was designed to work similarly to the common polar host, DPEPO. A moderate Φ_PL_ of 52% at was obtained for **POPT-25** in toluene, while the control polymer **PCzPT-19**, with only a donor pendant, only exhibited a Φ_PL_ of 25% (Table S13). Even though both polymers share the same pendant emitter moiety, the *k*_RISC_ of the two polymers deviated considerably, at 1.9 × 10^5^ s^–1^ for **PCzPT-19** and 8.1 × 10^5^ s^–1^ for **POPT-25**, revealing that the polarity of host pendants has a significant impact on the TADF kinetics. A yellow SP-device with **POPT-25** (10 wt% in mCP) showed an EQE_max_ of 5.2% with CIE coordinates of (0.36, 0.50). A lower EQE_max_ of 1.2% was obtained for the SP-OLED with **PCzPT-19**, attributed to the improved charge balance and higher Φ_PL_ for **POPT-25** (36% vs 21%) in 10 wt% doped mCP films. Non-doped devices of both polymers were also prepared; however, they displayed much lower efficiencies of under 1% EQE_max_.

Polymers **P1**, **P2**, and **P3** (Figure 122) all based on the same emitter **DBTO2-PTZ** were also developed by Li *et al.*,^968^ here using dibenzothiophene instead of carbazole as the host monomer. Polymers **P1**, **P2**, and **P3** had molecular weights of 12, 30 and 23 kDa with PDIs of 1.3, 1.7 and 1.7, and Φ_PL_ in neat films of 10.4%, 23.5% and 19.5%, respectively. This sequence of Φ_PL_ indicated that the monomer ratio in **P2** (approximately equal in TADF emitter and host monomers) gives the best environment for the TADF pendant group. The SP-OLEDs with **P2** also had a performance very close to its carbazole-based analogue **PCzPT-19**, indicating only subtle differences in hosting environment when using the co-monomer based on carbazole and dibenzothiophene.

Li *et al*.^969^ again adopted the same design strategy for blue TADF polymers **PDT-x**. These polymers incorporated a pendant 9,9-dimethyl-10-phenylacridine (BDMAc) with high triplet energy (*E*_T_ = 3.38 eV) to act as host and spacer unit, in conjunction with the intrinsically high-performance blue TADF emitter **DMA-TXO2**.^970^ The polymers **PDT-1**, **PDT**-**2** and **PDT-3** have molecular weights of 10, 11 and 21 kDa, with PDIs of 1.4, 1.7 and 1.7, respectively. In contrast to the high Φ_PL_ of this emitter (Φ_PL_ = 80% in 11 wt% in DPEPO), the Φ_PL_ of the polymer neat films decreased to 42, 54, 46% for **PDT-1**, **PDT-2**, **PDT-3**, respectively (Table S13), most likely due to concentration quenching despite the excess of BDMAc spacer monomers. The best SP-OLED based on neat **PDT-2** achieved an EQE_max_ of 5.3% at λ_EL_ of 436 nm and CIE coordinates of (0.15, 0.09), in comparison to an EQE_max_ of around 20% for the vacuum-deposited device based on **DMA-TXO2**.

Polynorbornene backbones have also been used for the development of TADF polymers. The high triplet energy (2.95 eV) of this subunit is suitable to prevent the quenching of triplets from TADF units to the polymer backbone, crucial for the design of high-triplet blue TADF polymers. A series of blue TADF polymers was reported by Zeng *et al.*,^971^ with carbazole-containing monomers acting as hole injection units, and **DMAC-DPS** derivatives as emissive monomers linked to a norbornene backbone (Figure 122). This design was chosen to avoid conjugation along the backbone and therefore suppress any red-shifting as monomer photophysics is effectively localized. The polymer molecular weight and branching can also be well-controlled because of the ring-opening metathesis polymerization conditions. The molar ratio of TADF monomers was varied (**PBD-0**, **PBD-5**, **PBD-10**, **PBD-15**, and **PBD-20**) to give polymers of molecular weight ranging from 5.8–8.0 kDa. The neat films of these polymers all showed blue emission at around 460 nm. The non-doped SP-OLEDs with **PBD-10** showed an EQE_max_ of 7.3% at CIE coordinates of (0.20, 0.29). The SP-OLEDs with **PBD-5**, **PBD-15** and **PBD-20** showed EQE_max_ of 6.0, 7.1 and 6.7%, respectively, where the EQE_max_ tracked with the Φ_PL_ of the polymers. The efficiency roll-off was severe though, with the EQE_100_ decreasing by between 45 to 53%, correlating with the relatively slow values of *k*_RISC_.

Cole *et al*.^972^ engineered the polymer **D3P-DEH**, which contains a non-conjugated alkyl backbone grafted with three different side chains (Figure 122). The emissive side chain contains a TADF emitter, while the second side chain contains the hole-transporting host material, mCP, and the third side chain contains a modified mCP unit (**NmCP1**), which is designed to act as an electron-transporting host material. The introduction of both electron and hole transporting units along the polymer backbone obviates the need for an external host material. The authors demonstrated this claim by comparing the performance of non-doped and doped devices. Even though the non-doped device exhibited a low EQE_max_ of 1.6% at CIE (0.27, 0.50), it still outperformed the doped device (30 wt% in 26DCzPPy) with an EQE_max_ of 0.6% (Table S13). This study also demonstrated the first report of a TADF “self-hosted” polymer SP-OLED deposited by inkjet-printing.

#### 10.1.2 Donor Backbone with Acceptor Pendants

Zhu *et al*.^973^ reported a TADF polymer, **PAPTC**, comprised of a donor-containing backbone where some of the donors are covalently linked to pendant acceptor groups. The conjugated backbone of **PAPTC** (Figure 123) consists of acridan and carbazole groups linked *via* the 3- and 6-positions. A pendant triazine acceptor was linked to each of the acridan monomers to form the TADF subunits, while the carbazole groups provide both spacing to avoid ACQ of the TADF emitters as well as (presumably) hole transport properties. DFT calculations confirmed that the HOMO is delocalized over the entire polymer backbone, while the LUMO is localized on the pendant acceptor. **PAPTC** has a Δ*E*_ST_ of 0.13 eV, and the Φ_PL_ in toluene was increased from 22 to 40% after bubbling with N_2_. Non-doped SP-OLEDs showed an EQE_max_ of 12.6% with λ_EL_ at 521 nm (Table S13).

**Figure 123:**
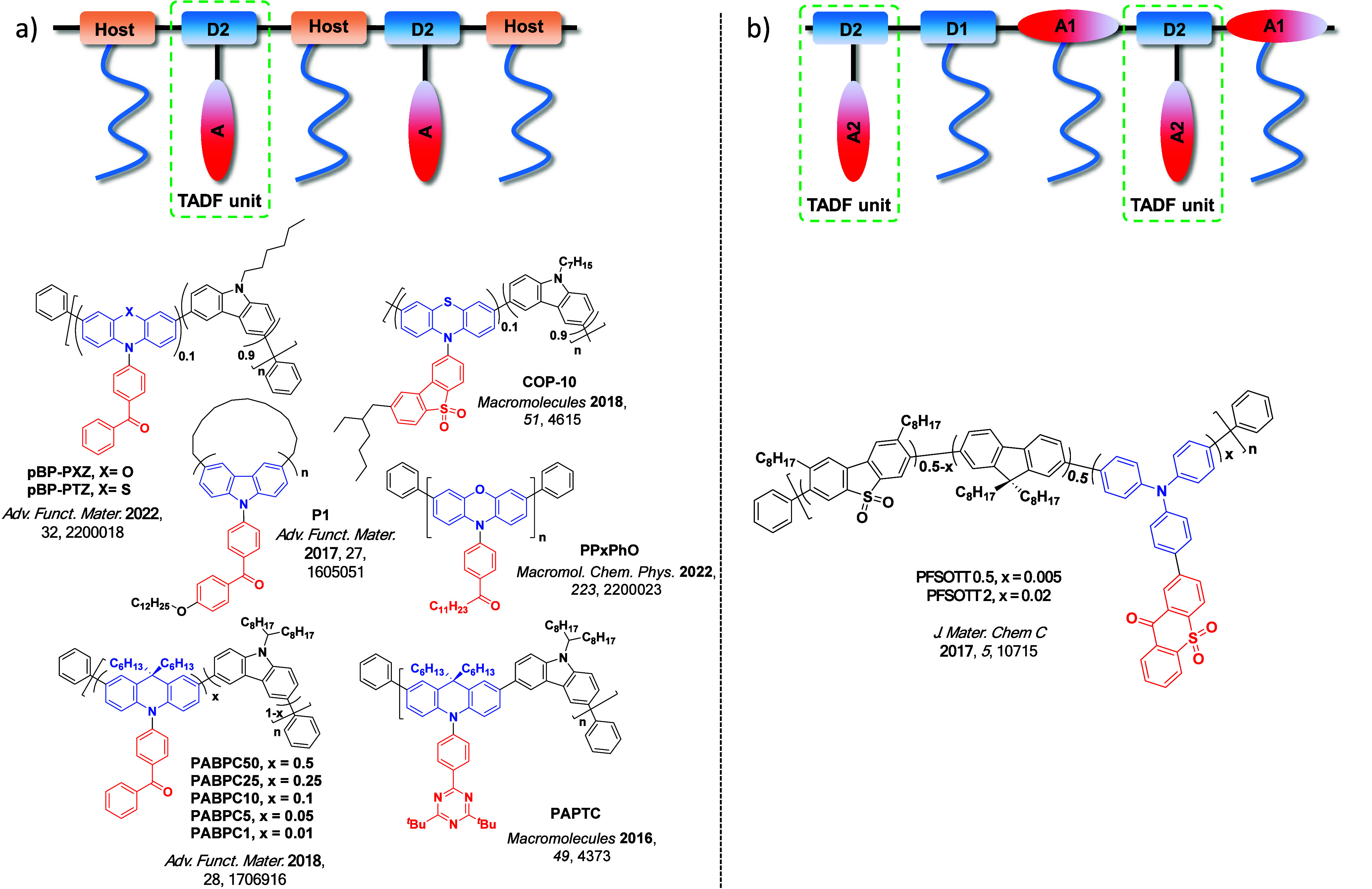
Schematic diagram and chemical structures of a) donor-backbone (blue) TADF polymers containing pendant acceptor groups (red) and b) donor-acceptor-backbone (blue) TADF polymers containing pendant acceptor groups (red).

Yang *et al*.^974^ employed a similar strategy to produce a series of TADF polymers with molecular weights of between 6.3–17.6 kDa, using the same acridan-carbazole backbone but instead incorporating a benzophenone acceptor coupled to the acridan. The best performing polymer, **PABPC5** (Figure 123), contained 5% of the TADF monomer and displayed a high Φ_PL_ of 77% (Table S13). A non-doped SP-OLED using **PABPC5** showed an EQE_max_ of 18.1% with CIE coordinates of (0.40, 0.56), and low efficiency roll-off with an EQE_1000_ of 17.8%.

A copolymer **COP-10** (Figure 123) using 10% **DBTO2-PTZ** as the TADF monomer and phenothiazine-carbazole backbone was reported by Liu *et al*.^975^ The SP-OLEDs with **COP-10** doped at 10 wt% in a mixed TCTA:​TAPC (65 wt%:​25 wt%) co-host showed an EQE_max_ of 15.7%. However, significant efficiency roll-off was observed, with a reduction in EQE of 76% at a luminance of 100 cd m^–2^.

A similar approach was used by Zhao *et al*.^976^ who reported polymers **pBP-PXZ** and **pBP-PTZ** consisting of alkyl-substituted carbazoles copolymerized with 10% of either PXZ or PTZ donors. These donors were themselves coupled to benzophenone to give TADF emissive subunits within the polymers (Figure 123), which both have a molecular weight of around 16 kDa and a PDI of 1.7. In neat films both polymers showed λ_PL_ at 550 nm yet diverging Φ_PL_ of 82% (**pBP-PXZ**) and 48% (**pBP-PTZ**) (Table S13). The oxygen containing **pBP-PXZ** showed a slight faster τ_DF_ of 1.29 μs compared to 1.55 μs for **pBP-PTZ**. The non-doped SP-OLED with **pBP-PXZ** showed a higher EQE_max_ of 13.7% [CIE coordinates of (0.52, 0.48)] compared to its sulfur-containing counterpart with an EQE_max_ of just 7.9% at CIE coordinates of (0.50, 0.49). The non-doped device with **pBP-PXZ** showed a lower efficiency roll-off of 35% at 1000 cd·m^–2^ while the **pBP-PTZ** devices were not able to achieve a brightness of 1000 cd·m^–2^. In devices using 10 wt% **pBN-PXZ** doped in CBP, the EQE_max_ increased to 23.1% and the efficiency roll-off was reduced to 16%, with the EQE_1000_ still exceeding 19%. These reported devices are the best performing SP-OLED using a TADF polymer as emitter to date, and again highlight the importance of tuning the host-monomer content.

Wei *et al*.^977^ demonstrated a new polymer **P1** with macrocycle design to achieve TADF (Figure 123), based on the linking together of non-TADF monomers and where the macrocycle gains TADF activity by virtue of the increased donor conjugation in the cyclized material. DFT calculations and photophysical characterization of **P1** (2 wt% in polystyrene) showed that this material has a Φ_PL_ of 71% (3% for the monomer) of which delayed fluorescence (Φ_DF_) contributed 51% with Δ*E*_ST_ of 0.19 eV. Unfortunately, no OLEDs were prepared to test the performance of **P1**, although a subsequent study investigated the stepwise effects of this conjugation expansion in a series of non-cyclic oligomers.^977^

Zhang *et al*.^978^ reported a polymer consisting exclusively of TADF monomers, **PxPhO** (Figure 123), with a poly(phenoxazine) backbone each bearing a ketone acceptor unit. The polymer maintained a small Δ*E*_ST_ of 0.07 eV, similar in magnitude to its monomer unit (Δ*E*_ST_ = 0.05 eV), although the emission of the polymer in toluene solution (λ_PL_ = 557 nm) is red-shifted compared to the monomer by approximately 70 nm (Table S13). A comparison of the fluorescence lifetimes (prompt and delayed) between monomer and polymer showed that the τ_p_ of the polymer is three times shorter than its the monomer (3.63 and 12.02 ns), while the claimed τ_d_ of the monomer was almost seven times longer than its polymer (0.054 and 0.008 μs). The Φ_PL_ of the polymer (> 60%) and monomer (∼50%) are both similar, implying that these differences in lifetimes did not arise from faster non-radiative decay in the polymer, but instead due to faster RISC. Furthermore, in doped SP-OLEDs with an EML consisting of 80 wt% mCP (host) and 20 wt% emitter (monomeric **PxPhO** or polymeric **PPxPhO**) the device with the polymeric emitter showed a higher EQE_max_ of 11.8% (λ_EL_ = 550 nm) compared to the device with **PxPhO** (EQE_max_ = 8.8%; λ_EL_ = 520 nm). The authors attributed the enhanced performance of the polymer OLED to the better film-forming properties of **PPxPhO** compared to **PxPhO**.

Wang *et al*.^979^ prepared a series of TADF polymers **PFSOTT-x** (Figure 123) composed of a TADF monomer containing a triphenylamine donor and a thioxanthone-dioxide acceptor, alongside an alternating fluorene and dibenzothiphene-S,S-dioxide backbone. The resulting polymers have a molecular weight of 48.2–58.3 kDa with a broad PDI of over 2. With increasing proportion of the TADF monomer, the PL spectra of the polymers gradually red-shifted from blue to orange in the neat films. Despite a remarkably high Φ_PL_ of 89% in the neat film for **PFSOTT0.5** (0.5% of the TADF unit), the non-doped OLED achieved an EQE_max_ of only 2.6% with CIE coordinates of (0.49, 0.49) (Table S13), an indication of poor charge balance in the device. Indeed, the EL performance was significantly improved when **PFSOTT2**, (2% TADF unit) was dispersed with 40 wt% in an mCP matrix, giving an EQE_max_ of 19.4% at λ_EL_ at 592 nm, which was consistent with the near unity Φ_PL_ of this polymer emitter in mCP. This result once again highlights the importance of designing TADF polymers that include functional groups to support both TADF emission and charge transport in a device context.

Chiral small molecule TADF emitters have been shown to emit circularly polarized luminescence (CPL) (See Section 7). Hu *et al*.^980^ developed the first chiral conjugated poly(carbazole-ran-acridine) polymer **P10** which contained a stereogenic alanine pendant groups alongside achiral TADF co-monomers. The polymer has a molecular weight of 10 kDa and a PDI 1.7 (Figure 124). By using a polymeric emitter instead of a small molecule emitter, better thermal stability and easier solution-processability was achieved. The *g*_lum_ of **P10** was −1.39 × 10^–3^. AIE was reported in the solid state, and neat films of **P10** exhibited a green emission with Φ_PL_ of 10.3% and τ_d_ of 1.3 μs (Table S13). The doped SP-OLED (5 wt% in mCP) showed green emission with CIE coordinates of (0.36, 0.52); however, the EQE_max_ was a rather low 0.87%, consistent with the low Φ_PL_.

**Figure 124:**
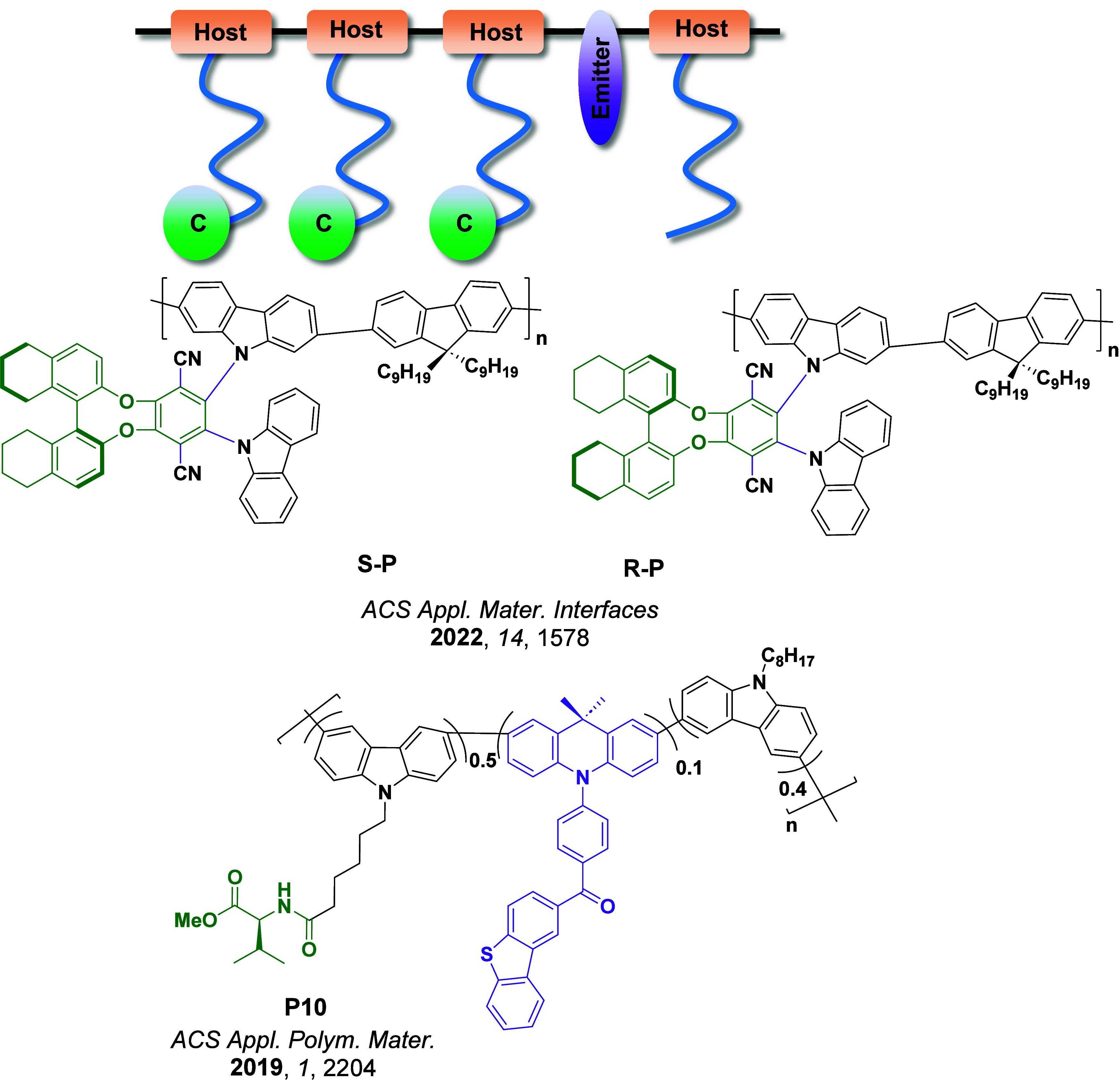
Schematic diagram and chemical structures of TADF polymers with chiral pendant groups (green) that are located on the TADF emitter (S-P and R-P) or on a separate side chain (P10) with the TADF emitter being anchored within the backbone (purple).

Inspired by this work, Teng *et al*.^981^ reported CP-TADF polymers and SP-OLEDs. In contrast to the previous example, in this case the TADF unit was itself intrinsically chiral and polymerized by one of its carbazole donor into the main chain alongside a fluorene co-monomer (**S-P** and **R-P**, Figure 124). Photophysical investigations of the neat film showed practically identical λ_PL_ of 560 and 562 nm for the **S-P** and **R-P** enantiomers, respectively. The emission of the polymers as 10 wt% doped films in mCP were blue-shifted by 13 nm compared to the neat film (Φ_PL_ of 76% for **S-P** and 72% for **R-P**, with τ_d_ of 2.3 and 1.6 μs, respectively). The *g*_lum_ values are 1.9 × 10^–3^ (**S-P**) and −1.9 × 10^–3^ (**R-P**). Doped SP-OLED devices (10 wt% in mCP) showed EQE_max_ of 15.8% for **S-P** and 14.9% for **R-P**, with a low efficiency roll-off at 1000 cd·m^–2^ of 22 and 15%, respectively. Both devices showed yellow-greenish emission with CIE coordinates of (0.41, 0.57), while the *g*_EL_ values were +1.6 × 10^–3^ (**S-P**) and −1.5 × 10^–3^ (**R-P**).

Freeman *et al*.^982^ proposed a new strategy to induce TADF in conjugated polymers by including an orthogonal acceptor group at the bridgehead position of alternating spiro-fluorene repeat units. In this way the pendant acceptor group is not directly attached to the donor units in the main chain, and instead the CT states and TADF are achieved as a result of through-space interactions. In **ASFCN** (Figure 125), the electron and hole wave functions were consequently spatially separated due to the non-conjugated sp^3^ connection between acceptor pendants and donor backbone units. The low Φ_PL_ of 16% at λ_PL_ of around 520 nm (Table S13) was attributed to a relatively high rate of non-radiative decay from a ^3^LE state and a low radiative decay rate of the singlet state, a result of the near zero overlap between frontier molecular orbitals.

**Figure 125:**
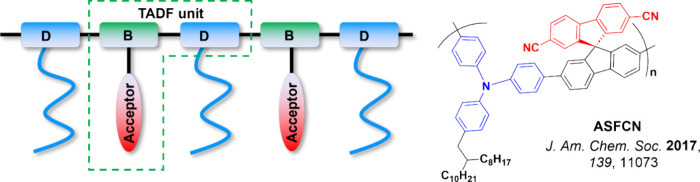
Schematic diagram and chemical structure of **ASFCN** (the blue color signifies donor moieties, while the red color signifies acceptor moieties).

#### 10.1.3 Main-Chain D-A Type TADF Polymers

Nikolaenko *et al*.^983^ proposed a series of main-chain TADF polymers based on an “*intermonomer TADF*” strategy that induces TADF properties from the linked donor and acceptor repeating units (Figure 126). The TADF polymer **LEP** (Figure 126) was synthesized *via* Suzuki polymerization using a feed ratio of 5%:50%:45% of the three monomers, containing donor (amine), acceptor (triazine), and spacer (alkyl chain) units. The triazine unit in this case supported both the TADF emission by interacting with the amine group as well as contributed to charge transport to tune the recombination zone. As highlighted in previous examples, the large content of spacer monomers helped to maintain a uniform dispersion of TADF-emitting units in the polymer and mitigate ACQ. A moderate Φ_PL_ of 43% and Δ*E*_ST_ = 0.22 eV were obtained for **LEP**. The green non-doped SP-OLED device showed an EQE_max_ of 10% at CIE coordinates of (0.32, 0.56).

**Figure 126:**
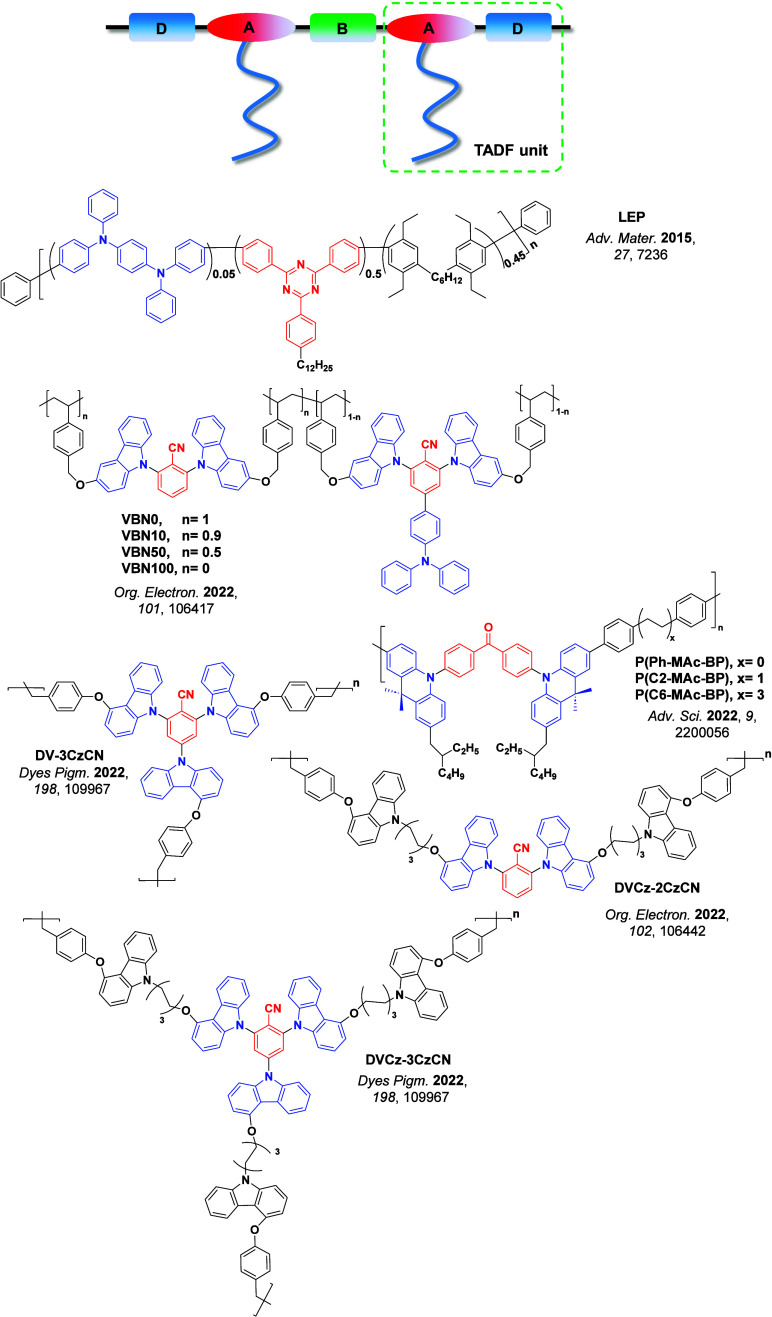
Schematic diagram and chemical structures of TADF polymers with donor (blue) and acceptor (red) emissive units within the main chain.

To observe the impact of including different spacers on the TADF properties of a main-chain-emissive polymer, Philipps *et al*.^984^ synthesized a group of three polymers consisting of a benzophenone acceptor unit and two acridan donor units, connected in each monomer with non-conjugated spacers of different length (Figure 126). Non-doped SP-OLEDs with longer non-conjugated spacer units displayed improved device performance, with EQE_max_ of 2.9% using **P** (**Ph-Mac-BP**) with a fully conjugated phenyl spacer, increasing to 6.7% for **P(C2-Mac-BP)** with an ethyl spacer and 7.1% for **P(C6-Mac-BP)** with a hexyl spacer. Furthermore, the SP-OLED with **P(C6-Mac-BP)** showed the best efficiency roll-off, with the EQE dropping by only 8% at 100 cd m^–2^ and 30% at 1000 cd m^–2^ (Table S13).

In contrast to linear polymers, Sun *et al*.^985,986^ prepared branched polymers comprised of a carbazole-benzonitrile emissive center and carbazole spacer units, that were thermally crosslinked by annealing after spin-coating. The resulting cross-linked polymer films showed blue emission at around 470 nm, with Φ_PL_ values of 36, 54 and 68% for **DV-3CzCN** (without carbazole spacing units), **DVCz-3CzCn**, and **DVCz-2CzCN** (a linear analogue, Figure 126), respectively. Non-doped thermally annealed devices exhibited sky-blue emission with CIE coordinates of (0.16, 0.31), (0.15, 0.30) and (0.16, 0.21). The SP-OLEDs with **DVCz-2CzCN** and **DVCz-3CzCn** showed an EQE_max_ of around 6%, while the device with **DV-3CzCN** only showed an EQE_max_ of 0.8% (Table S13). This difference in performance was explained as arising from the isolation of the TADF core units in the branched polymers, which minimized ACQ.

A second approach was also reported by Sun *et al.*,^987^ using two small molecule TADF subunits, **2CzBn** and **2CzTBn**. These were linked with vinyl-benzyl groups to build a copolymer with different ratios of the emitter units (**VBNx**, Figure 126). Two polymers containing different ratios of **2CzBn** and **2CzTBn**, **VBN10** containing 90% **2CzBn** and 10% **2CzTBn** and **VBN50** containing 50% **2CzBn** and 50% **2CzTBn** were reported, as well as control polymers containing each of the TADF subunits individually. According to the authors the **2CzBN** units act primarily as hosts while the **2CzTBN** units act as guest emitters in this polymer. Both copolymers showed similar photophysical properties in the neat film, with λ_PL_ at 488 nm and 497 nm and Φ_PL_ of 74% and 70% for **VBN10** and **VBN50**, respectively. The non-doped SP-OLEDs with **VBN10** exhibited an enhanced performance with an EQE_max_ of 11.4% at CIE coordinates of (0.20, 0.38) compared to the device with **VBN50**, which showed an EQE_max_ of 9.1% at CIE coordinates of (0.21, 0.41). The similar emission spectra of the two polymers indeed supported the hypothesis that one TADF subunit acts as host for the other. Comparing to control polymers containing only one of the monomers (**VBN0**, **VBN100**) the copolymers also showed enhanced Φ_PL_ and shorter τ_d_ in the neat film. Accordingly, comparison devices using the homopolymers with for **VBN0** and **VBN100** showed much lower performance with EQE_max_ of only 3.1 and 4.9%, respectively.

#### 10.1.4 Through Space TADF Polymer

In recent years it has become clear that TADF can also arise effectively as a result of through-space interactions between donor and acceptor moieties (See Section 12). Shao *et al*.^988^ applied this strategy to produce blue TADF polymers based on a nonconjugated polyethylene backbone, with TSCT interactions between the pendant acridan-based donors and the triazine acceptors. The copolymers **P-Ac95-TRZ05** and **P-TBAc95-TRZ05** (Figure 127) were synthesized accordingly, although only **P-Ac95-TRZ05** exhibited TADF, associated with a small Δ*E*_ST_ of 0.019 eV and a Φ_PL_ of 60% in the neat film. As a control system, **P-TBAc95-TRZ05** showed no TADF due to the too large inter-chromophore distances between the TBAc and TRZ pendants. The non-doped SP-OLEDs with **P-Ac95-TRZ05** showed sky-blue electroluminescence at CIE (0.18, 0.27), an EQE_max_ of 12.1% and low efficiency roll-off with an EQE_1000_ of 11.5% (Table S13).

**Figure 127:**
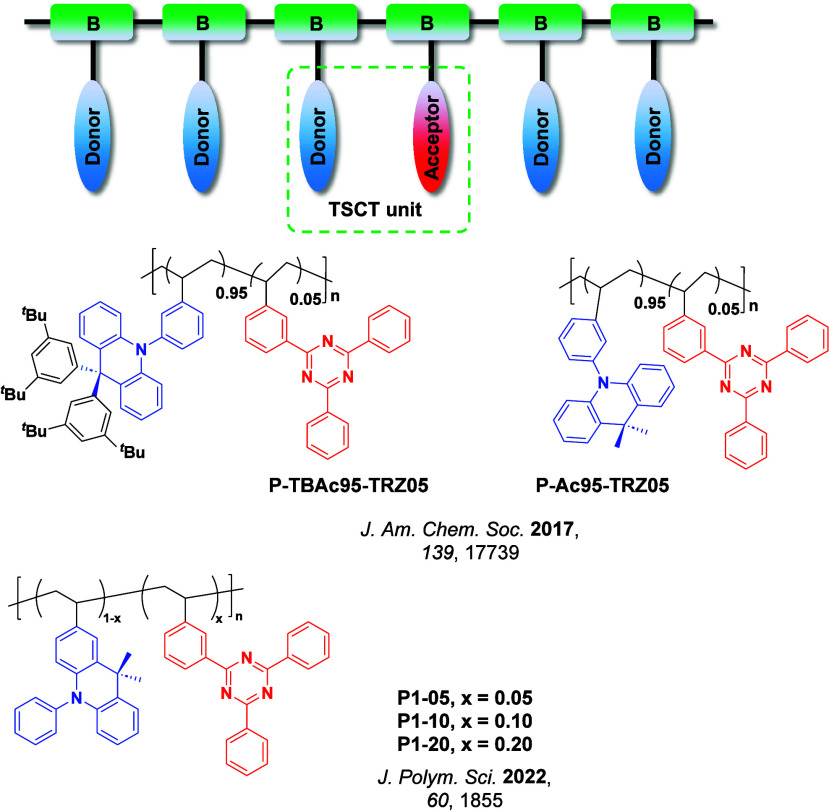
Schematic diagram and chemical structures of through-space charge transfer (TSCT) TADF polymers (the blue color signifies donor moieties, while the red color signifies acceptor moieties).

The same group reported a second class of TSCT-TADF polymers^989^ with the same donor and acceptor building blocks but different connectivity from the acridine donor to the main chain (**P1-x** series, Figure 127). Compared to their previous work where the donor unit was attached to the backbone *via* a phenyl ring attached to the N-atom of the acridine ring (10-phenyl), here the donor unit was connected directly at the 2-position of the acridine ring. Compared to **P-Ac95-TRZ05**, the co-polymers **P1-05**, **P1-10** and **P1-20** (with 5, 10 and 20% TRZ, respectively) showed red-shifted emission at 485, 489 and 492 nm as neat films and lower Φ_PL_ values of 54, 47 and 45%, respectively (Table S13). The Δ*E*_ST_ values for the **P1-x** copolymers where also twice as large as for **P-Ac95-TRZ05**, at around 0.04 eV. In good agreement with these optical properties, non-doped device performance using the standout **P1-05** polymer showed an EQE_max_ of 11.3% at CIE coordinates of (0.20, 0.37). This study demonstrates nicely that, just as in small molecule D-A TADF emitters, the relative geometries of the donor and acceptor groups play a crucial role in the design of TADF polymers. Understandably, this is all the more crucial for TSCT materials, where these geometries cannot be as directly controlled by covalent design and bond placement.

In summary, a wide range of TADF polymer design strategies has emerged in recent years, with selected relevant examples presented here to highlight their typical photophysical and electroluminescent properties. Since polymers can exhibit the advantage of reduced ACQ and superior solution processability, they remain a promising class of emitters for high-performance SP-OLEDs. However, to compete with SP-OLEDs with small molecule emitters–especially in terms of properties like color purity (FWHM) and EQE_max_–still more materials development is still required.

### 10.2 TADF Dendrimers

Fluorescent, phosphorescent and now TADF polymer-based emitters for SP-OLEDs have all been widely reported. Despite their performance and suitability for SP-OLEDs, control purity and batch-to-batch variation in materials composition, polydispersity, branching and other structural defects during polymerization are intolerable concerns for commercial display production, and polymers are themselves nearly impossible to purify following synthesis to the level required by industry by standard methods.

One alternative method that can avoid these issues associated with polymers is to instead employ dendrimers. These are large and readily solution-processable macromolecules, but which also have well-defined molecular structures that can be purified in the same manner as low molecular weight small molecules. The large and globular nature of these dendrimer emitters also aids in protecting an emissive core from aggregation and thus in suppression of non-radiative decay pathways, opening the possibility of efficient non-doped devices. TADF dendrimers are indeed usually composed of a core emissive unit surrounded by optically inert dendron units that both shield the core from intermolecular interactions, and yet can also contribute to modulating charge transport within the film. The dendron units themselves can also act as donors, thus producing D-A TADF materials. Some donor dendrons can be coupled directly to a central acceptor core in a conjugated manner (i.e. conjugated dendrimers, Figure 128, while in other cases conjugation between the donor dendrons units and the emissive core is broken for example by using alkyl chains (non-conjugated dendrimers, Figure 130). In this section we will review recent progress towards high efficiency SP-OLEDs using dendrimer materials and summarize their photophysics and device performances in Table S14.

**Figure 128:**
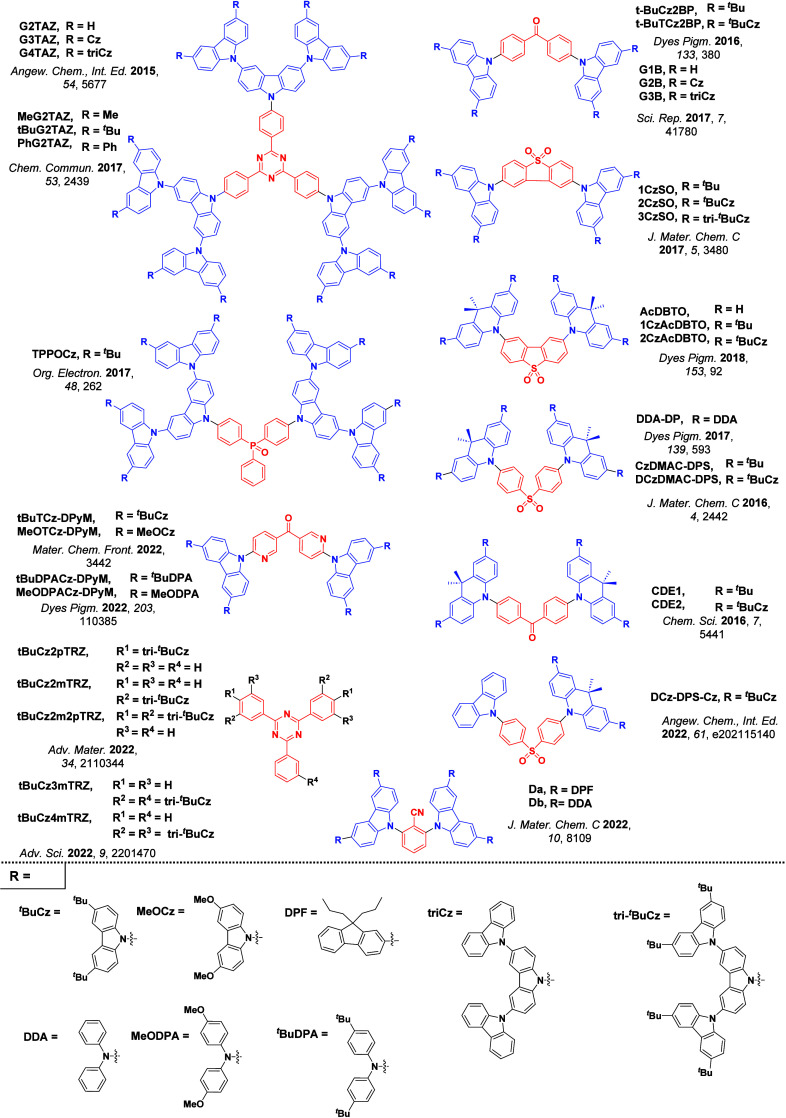
Chemical structures of conjugated TADF dendrimers consisting of a central acceptor unit (red) and peripheral donor dendrons (blue).

#### 10.2.1 Conjugated Dendrimers

The first TADF dendrimers, reported by Albrecht *et al*. in 2015, contained carbazole donor dendrons coupled to a central triazine acceptor.^990^ Dendrimers with one of three generations of donor dendrons, **G2TAZ**, **G3TAZ** and **G4TAZ** (Figure 128), were reported, with the most promising **G3TAZ** producing non-doped SP-OLEDs with a modest EQE_max_ of 3.4%. The efficiency roll-off at 100 cd·m^–2^ was ∼19%, but this increased significantly at 1000 cd·m^–2^ where the efficiency roll-off was ∼56% (Table S14). **G3TAZ** has a Φ_PL_ of 56% in the neat film, while when placed in dilute toluene solution, it increases to 100%, which suggested that the design of this dendrimer structure did not completely preventing ACQ in the neat film.

The use of an alternate donor dendron to **G2TAZ** with *tert*-butyl groups (^*t*^Bu) decorating the peripheral carbazole units, **tBuG2TAZ** (Figure 128),^991^ gave a much-improved device performance with an EQE_max_ of 9.5% and an efficiency roll-off of just 1% at 100 cd·m^–2^. SP-OLEDs using congener dendrimers where the ^*t*^Bu substituents were replaced with Me, Ph, and H, (Figure 128), produced EQE_max_ of 9.4%, 8.2% and 6.0%, respectively, demonstrating the value of these steric blocking groups at keeping the emitter dendrimers suitably isolated from each other.^990,991^ Further, each of these devices displayed excellent efficiency roll-off at 100 cd·m^–2^ where the EQE decreased by only 1%, 0% and 0%. There was also little variation in emission color between each of these devices, with λ_EL_ ranging from ∼500 nm to ∼505 nm (Table S14). Building upon the device with **tBuG2TAZ** (EQE_max_ 9.5%), a much higher EQE_max_ of 17.0% was achieved for non-doped devices based on emitter **tBuG2B**,^992^ in which a diphenylketone acceptor was used instead of triazine as the acceptor and core of the dendrimer (Figure 128). The Φ_PL_ of 74% in the neat film was higher than that measured in toluene solution (47%). The relatively low efficiency roll-off of 19% at 1000 cd·m^–2^ in the device was attributed to both the small Δ*E*_ST_ of 0.08 eV and the fast τ_d_ of 2.2 μs. The same emitter was also previously reported as **t-BuTCz2BP** by Huang *et al*.^993^ exhibiting a lower Φ_PL_ of 41% (in air), a shorter τ_d_ of 0.57 μs, and a lower EQE_max_ of only 4.3%, likely due to a less elaborate and optimized device stack. The other compounds in the study from Albrecht *et al*.^992^ involved replacement of ^*t*^Bu for Me (**MeG2B**), OMe (**MeOG2B**) and Ph (**PhG2B**) (Figure 128). The lower Φ_PL_ (34, 17 and 41%) of these dendrimers led to lower EQE_max_ of 9.0, 6.4 and 8.8%, respectively, in the SP-OLEDs. The devices suffered from serious efficiency roll-off, with both **MeG2B** and **PhG2B** unable to achieve a brightness of 1000 cd·m^–2^, while the EQE decreased by 69% for the device with **MeOG2B** at this brightness. ACQ was surmised to be responsible for the low efficiency of **MeOG2B**. The non-doped SP-OLED with **G2B** (Figure 128),^994^ showed an EQE_max_ 5.7%. The use of the higher generation dendrimer **G3B** led to a further decrease in the EQE_max_ to 2.9%, again highlighting the importance of carefully balanced charge transport in the device EML, and the role of the dendron shell in supporting this.^994^

Three dendrimers based on a triazine acceptor core and ^*t*^BuTCz donor dendrons have been recently reported by our group that showed outstanding photophysics and device performance.^995^ Two of the dendrimers are regioisomers, **tBuCz2pTRZ**, **tBuCz2mTRZ** (Figure 128) while the third possessing a combination of both *meta* and *para* donor dendrons **tBuCz2m2pTRZ**. Dendrimers **tBuCz2pTRZ**, **tBuCz2mTRZ** exhibited similar photophysical properties in neat films, with λ_PL_ at 481 nm and 483 nm and Φ_PL_ of 61% and 59% for each respectively. In contrast, **tBuCz2m2pTRZ** showed a red-shifted emission at 520 nm and a higher Φ_PL_ of 86% (Table S14). The non-doped SP-OLEDs with **tBuCz2pTRZ** and **tBuCz2mTRZ** showed EQE_max_ of 18.5 and 19.9% and associated efficiency roll-offs at 100 cd·m^–2^ of 13% and 40%, at CIE coordinates of (0.23, 0.46) and (0.27, 0.53), all respectively. The device with **tBuCz2m2pTRZ**, however, showed an excellent EQE_max_ of 28.7% at CIE coordinates of (0.37, 0.57); however, the efficiency roll-off was suboptimal at 26%. Devices that incorporated 30 wt% OXD-7 within the EML showed a much-improved efficiency roll-off of 2% at 100 cd·m^–2^ while the EQE_max_ was maintained above 28%.

In a separate study,^996^ we reported two dendrimers based on the same TRZ acceptor and different numbers of *meta*-connected ^*t*^BuCz donor dendrons, **tBuCz3mTRZ** and **tBuCz4mTRZ** (Figure 128). The non-doped SP-OLEDs with **tBuCz3mTRZ** and **tBuCz4mTRZ** showed improved performance compared to devices with **tBuCz2mTRZ** and **tBuG2TAZ**, with EQE_max_ of 23.7 and 23.8% at CIE coordinates of (0.35, 0.57) and (0.36, 0.58), respectively (Table S14). These examples showed that attaching the donor dendrons at the *meta* position and in large numbers is a good design strategy to increase device performance of TADF dendrimers, as this linking topology likely helps increase RISC.^119^

A similar structure to **tBuG2B** was reported by Zhang *et al*.^997^ who exchanged the diphen­yl­ketone acceptor with a dipyri­din­yl­ketone acceptor (DpyM) and attached two 9́H-9,3́:6́,9́́-ter­car­bazole (triCz) donor dendrons containing either peripheral ^*t*^Bu or OMe groups (**tBuTCz-DpyM**, **MeOTCz-DpyM**, Figure 128). In neat films the dendrimers emit at 495 nm for **tBuTCz-DpyM** and 514 nm for **MeOTCz-DpyM**, with Φ_PL_ of 64 and 55%, respectively (Table S14). Both compounds exhibit delayed fluorescence with τ_d_ of 9.5 μs for **tBuTCz-DpyM** and 7.9 μs for **MeOTCz-DpyM**. The SP-OLED with **tBuTCz-DpyM** (8 wt% doped in mCBP) showed an EQE_max_ of 20.4% at CIE coordinates of (0.25, 0.48), while the device with **MeOTCz-DpyM** showed an EQE_max_ of only 9.2% at CIE coordinates of (0.37, 0.54).

A very closely related structure was reported by He *et al.*,^998^ which contains diphenylamine-carbazole donor dendrons and either ^*t*^Bu or OMe groups decorating the periphery. The dendrimers **tBuDPACz-DpyM** and **MeODPACz-DpyM** (Figure 128) both exhibit a red-shifted emission in neat film of 596 nm and 645 nm and a much-decreased Φ_PL_ of only 11 and 3%, respectively, compared to their carbazole counterparts **tBuTCz-DpyM** and **MeOTCz-DpyM**. As a result of the low Φ_PL_, the EQE_max_ of the non-doped SP-OLEDs were low at 1.8% and 0.17%, respectively (Table S14).

Using the same design strategy, Li *et al*.^999^ reported a dendrimer containing donor dendrons with DMAC as the innermost donor unit and carbazole peripheral groups. The neat film of dendrimer **CDE1** (Figure 128) emits at λ_PL_ of 520 nm and has a Φ_PL_ of 77%. The non-doped devices showed an EQE_max_ of 13.8% and low efficiency roll-off at 1000 cd·m^–2^ of only 4%, which can be explained in part by the very short delayed lifetime of 0.52 μs. The CIE coordinates were (0.40, 0.54). The device emission mechanism was identified as a mixture of typical D-A TADF, along with exciplex emission occurring at the interface between the emitter and the TmPyPB electron transporting layer. A dendrimer with a higher generation donor dendron, **CDE2** (Figure 128) emits at λ_PL_ of 499 nm and has a comparable Φ_PL_ of 75% (Table S14), but did not display the extra exciplex emission. Without this exciplex contribution the non-doped SP-OLEDs showed a much smaller EQE_max_ of 5.2%. The reduction in EQE_max_ was assigned to a mismatch of the work functions of the HTL and ETL in the device.

Replacing ketone with sulfone as the central acceptor unit but using the same donor dendron as in **CDE1** gave the blue-emitting dendrimer **CzDMAC-DPS** (Figure 128), which emits at λ_PL_ of 492 nm and has a Φ_PL_ of 68% in the neat film. **CzDMAC-DPS** also has a small Δ*E*_ST_ of 0.09 eV and a short τ_d_ of 1.5 μs. The SP-OLEDs showed an EQE_max_ of 12.2% at CIE coordinates of (0.22, 0.44) (Table S14), although this was accompanied by a much stronger efficiency roll-off (63% decrease at 1000 cd·m^–2^). The non-doped device with **DCzDMAC-DPS**, an analogue dendrimer but with a higher generation of donor dendron (Figure 128), displayed a further blue-shift with CIE coordinates of (0.18, 0.27), but accompanied with a yet lower EQE_max_ of 2.2%.^1000^

A similar approach was reported by Gong *et al.*,^397^ who instead used peripheral diphenylamine units on the DMAC-based donor dendrons in **DDA-DP** (Figure 128). This compound has a small Δ*E*_ST_ of 0.04 eV as a neat film and emits at λ_PL_ at 549 nm, while in dilute toluene solution it has a Φ_PL_ of only 12.4%. Unlike the SP-OLEDs using the two previous dendrimer examples, devices with **DDA-DP** maintained the emission color of the DMAC-DPS core, with CIE coordinates of (0.36, 0.56). The EQE_max_ of the device was 8.1% and the efficiency roll-off was very low at only 1% at 1000 cd·m^–2^ (Table S14).

In order to address concentration quenching, Li *et al*.^1001^ designed a half-dendronized derivative of **CzDMAC-DPS**, **DCz-DPS-Cz** (Figure 128), which consists of a DPS acceptor core with a carbazole donor attached to one side and the CzDMAC donor dendron on the other side. While **DCz-DPS-Cz** maintained the color of **CzDMAC-DPS** with a neat film λ_PL_ at 494 nm (and similar CIE coordinates), the EQE_max_ of the SP-OLED was almost doubled to 23.3% (Table S14), and the efficiency roll-off at 100 cd·m^–2^ was halved to 23.3%, likely due to optimized charge transfer properties using the half-dendrimer material.

Huang *et al*.^1002^ designed dendrimers containing a related acceptor to DPS, dibenzothiophene-5,5-dioxide (DBTO). Attaching donor dendrons of varying size to this DBTO core afforded two green emitters, **1CzAcDBTO** and **2CzAcDBTO** (Figure 128), which in the neat film emit at λ_PL_ of 559 nm and 540 nm, have Φ_PL_ of 41 and 54%, and small Δ*E*_ST_ of 0.02 and 0.04 eV, all respectively (Table S14). Non-doped SP-OLEDs showed EQE_max_ of 3.9 and 4.5%, at CIE coordinates of (0.43, 0.54) and (0.36, 0.54), with evidence of large current leakage but low efficiency roll-off at 1000 cd·m^–2^ of 3.3% and 4.4%, all respectively.

Investigating the effects of different generation for carbazole-based dendrons, Li *et al*.^1003^ designed two green-emitting dendrimers **2CzSO** and **3CzSO** (Figure 128). As observed with other examples, the use of higher generation donor dendrons leads to a smaller Δ*E*_ST_ of 0.08 eV for **3CzSO** compared to 0.16 eV for **2CzSO** (Table S14), but the Φ_PL_ decreased from 43% (**2CzSO**) to 21% (**3CzSO**) due to the decreased oscillator strength for the S_0_-S_1_ transition in the latter. Non-doped SP-OLEDs with **2CzSO** and **3CzSO** showed EQE_max_ of 10.7 and 7.3% at CIE coordinates of (0.27, 0.52) and (0.31, 0.53), respectively, demonstrating conclusively that the use of larger donor dendrons does not always equate to better performance in the SP-OLED.

Wang *et al*.^1004^ reported a dendrimer (**TPPOCz**, Figure 128) containing tri-^*t*^BuCz donor dendrons and a phosphine oxide acceptor that emits at λ_PL_ of 400 nm with Φ_PL_ of 33% in the neat film (Table S14). The non-doped single-layer SP-OLED showed a poor EQE_max_ of only 0.27% at CIE coordinates of (0.18, 0.13). The low EQE_max_ was attributed to hindered charge injection into the emitting layer caused by a mismatch of transport layer work functions. To overcome this, the authors fabricated an SP-OLED with TmPyPB/TPBi acting as the ETL, which increased EQE_max_ to 2.0%, but also resulted in a large red-shift of the electroluminescence, with CIE coordinates of (0.26, 0.31).

Puttock *et al*.^1005^ reported two TADF dendrimers based on a benzonitrile acceptor core surrounded by two *ortho*-carbazole donor dendrons functionalised with either fluorene (**Da**, Figure 128) or diphenylamine groups (**Db**). Neat films of dendrimer **Da** emits at λ_PL_ of 463 nm and has a Φ_PL_ of 27% in the neat film, whereas **Db**, containing the stronger donor dendrons, emits at λ_PL_ of 526 nm with a Φ_PL_ of 21%. SP-OLEDs with both dendrimers showed low EQE_max_, either in non-doped or doped devices (4 wt% in mCP). The 4 wt% doped mCP device of **Db** showed the highest EQE_max_ amongst the devices in this study of 5.8% at CIE coordinates of (0.25, 0.48). The same group has also investigated the use of hybrid dendrons that themselves contain D-A TADF subunits, which then feed excitons to a central organometallic phosphorescent centre.^957,1006^

Rather than basing the dendrimer design about a central acceptor moiety, Wang *et al*.^1007^ developed a series of π-stacked dendrimers composed of cofacially aligned and alternating dendritic teracridan donor dendrons and triazine acceptors (Figure 129). The closely spaced donors and acceptors around a central benzene ring led to efficient TSCT-TADF properties. By regulating the strength of the TSCT *via* substituent effects on the acceptor, the emission color of the dendrimers was tuned from blue to yellow/red. The PL spectra of **BD-Cy**, **YD–TF** and **RD–2TF** in toluene exhibit broad CT emission at λ_PL_ of 487, 552 and 590 nm (Figure 129), respectively, a trend in line with the increased electron-withdrawing strength of the triazine acceptors containing increasing numbers of trifluoromethyl groups. The spatial separation between donor dendrons and acceptors reduces the overlap of the frontier molecular orbitals, thus leading to small Δ*E*_ST_ of 0.05, 0.04 and 0.04 eV for **BD–Cy**, **YD–TF** and **RD–2TF**, respectively. The SP-OLEDs with **BD–Cy**, **YD-TF** and **RD–2TF** showed EQE_max_ of 18.2, 21.9, and 10.3%, respectively, in good agreement with their corresponding Φ_PL_ values of 74, 86 and 49% as 10 wt% doped films in polystyrene.

**Figure 129:**
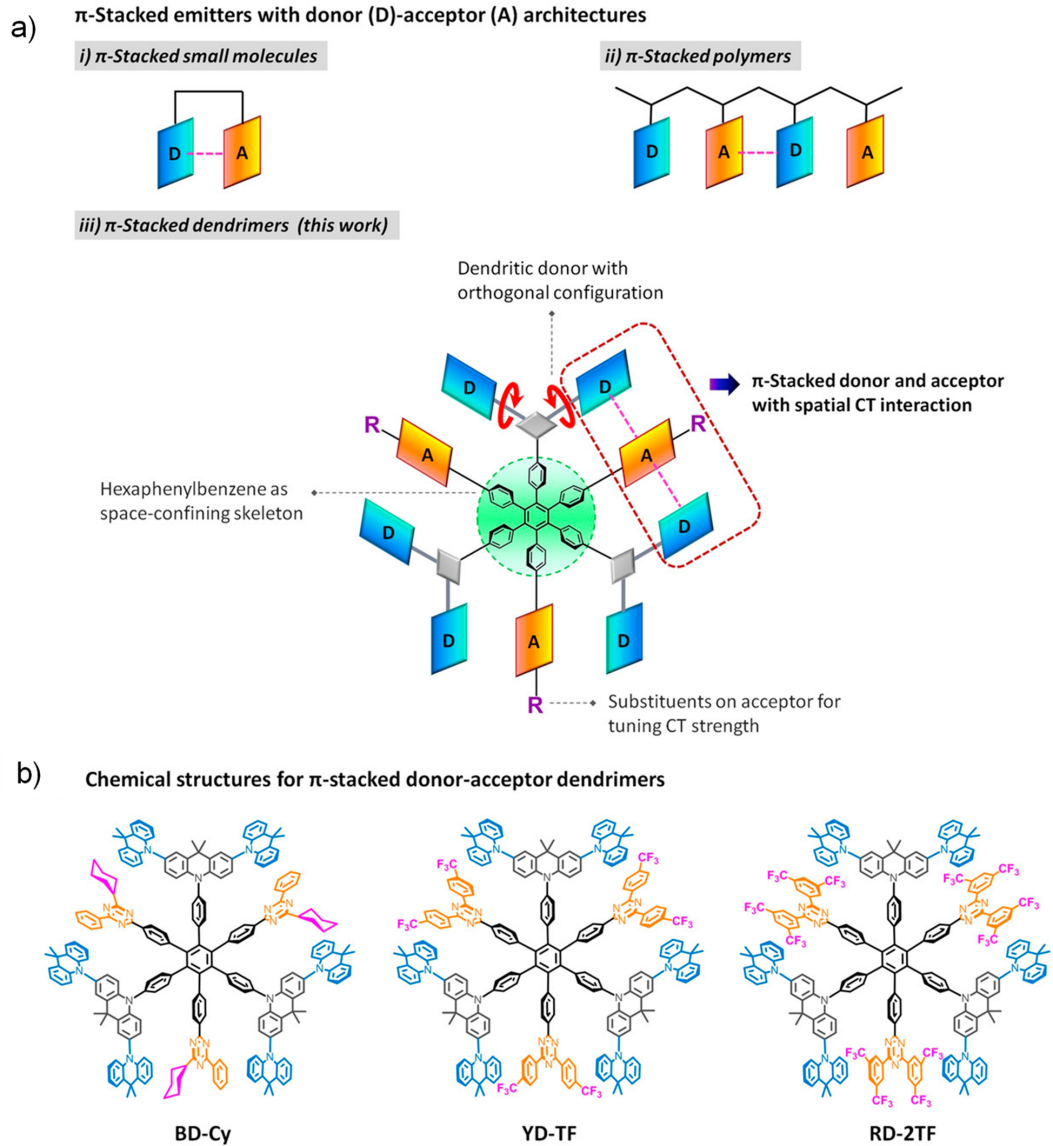
a) π-Stacked emitters with donor (D)-acceptor (A) architectures; b) chemical structures of π-stacked through-space donor–acceptor TADF dendrimers. Taken and adapted with permission from ref 1007. Copyright [2021/Angewandte Chemie International Edition] John Wiley & Sons.

#### 10.2.2 Non-conjugated Dendrimers

**Cz-CzCN** is a dendrimer consisting of a **5CzBN** TADF core decorated with carbazole at the periphery of alkyl chain tethers (Figure 130).^1008^ The neat film emits at λ_PL_ of 509 nm and has a Φ_PL_ of 52%, which is much higher than the 21% measured for **5CzBN**. The τ_d_ is short at 2.3 μs while the Δ*E*_ST_ is moderate at 0.17 eV. Non-doped SP-OLEDs with **Cz-CzCN** showed EQE_max_ of 17.1% at CIE coordinates of (0.26, 0.52), which showed a low efficiency roll-off of 11% at 1000 cd·m^–2^.

**Figure 130:**
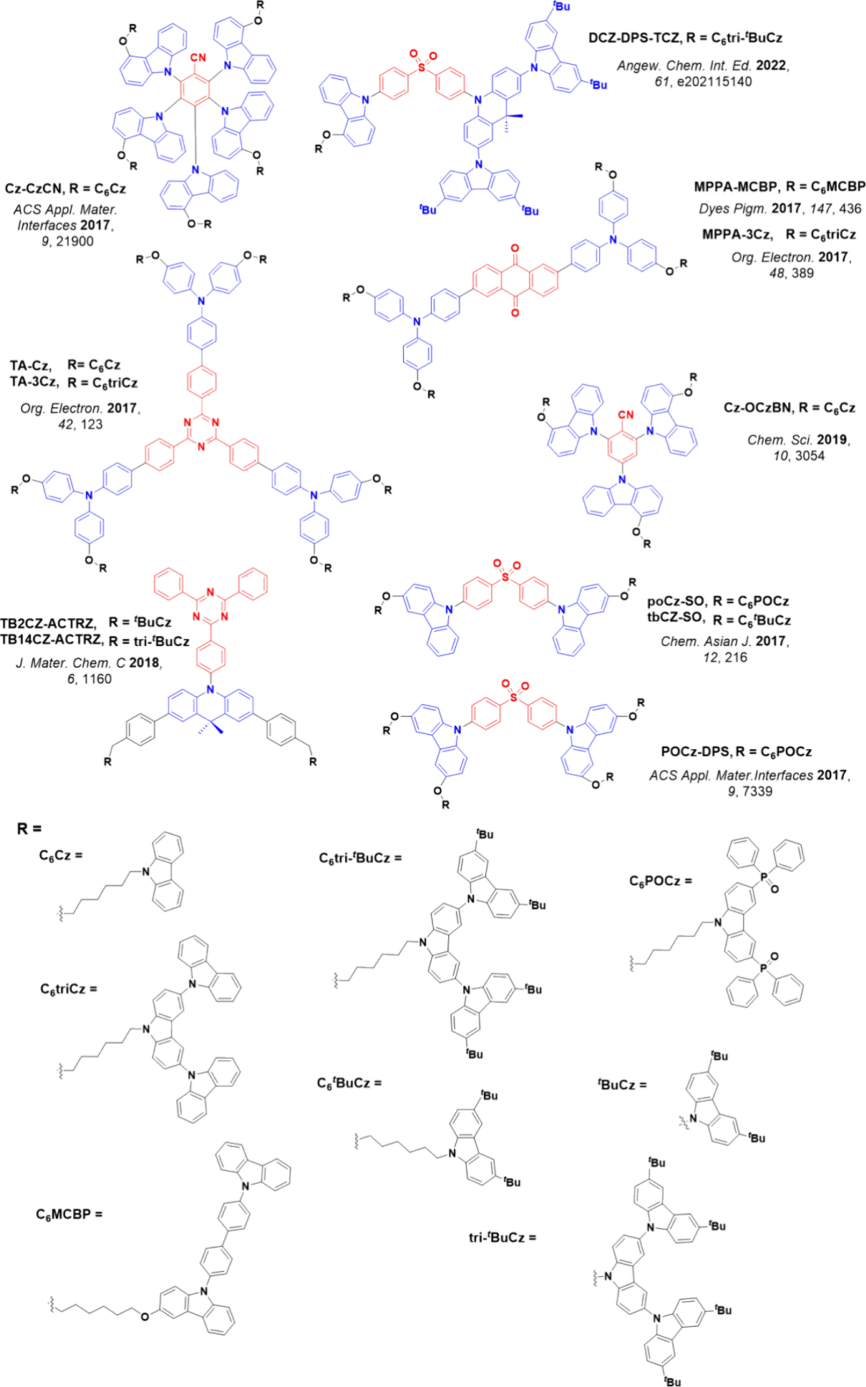
Schematic diagram and chemical structures of non-conjugated TADF dendrimers, consisting of a central donor (blue)-acceptor (red) emitter with peripheral donor dendrons connected with a non-conjugated linker.

Of a similar concept, **Cz-OCzBN** (Figure 130), using a **3CzBN** core and the same dendronized carbazole, emits at λ_PL_ of 498 nm and has a Φ_PL_ of 58% in the neat film (Table S14). The non-doped SP-OLED with the blue emitter showed an EQE_max_ of 6.6% at CIE coordinates of (0.18, 0.29) and had an efficiency roll-off of ∼26% at 1000 cd·m^–2^. This dendrimer was then also used in conjunction with a red phosphorescent emitter to produce solution processed white OLEDs. SP-WOLEDs with an EML consisting of **Cz-OCzBN** co-doped with 0.6 wt% of the iridium-based phosphorescent emitter **PO-01** showed an EQE_max_ of 17% at warm white CIE coordinates of (0.34, 0.44) and an efficiency roll-off of 30% at 1000 cd·m^–2^, which makes them one of the highest performing hybrid SP-WOLEDs to date.^1009^

Sun *et al*.^1010^ reported a dendrimer with diphenylamine donors joined to a triazine core, with triCz at the periphery (**TA-3Cz**, Figure 130). Conjugation between the core and outer dendron units was broken using hexyl chains. **TA-3Cz** emits at λ_PL_ of 541 nm, has a Φ_PL_ of 71% and a short τ_d_ of 0.8 μs in the neat film (Table S14). The non-doped SP-OLEDs showed an EQE_max_ of 11.8%, and although efficiency roll-off at 1000 cd·m^–2^ was ∼58%, a high maximum luminance of 23,145 cd·m^–2^ was achieved. The strong efficiency roll-off was attributed to the fact that triCz is unipolar, resulting in poor charge balance within the non-doped EML. The device with a smaller derivative capped with only a single generation of carbazole, **TA-Cz** (Figure 130), showed lower efficiencies with EQE_max_ 5.5%, a result of alleviated ACQ in **TA-3Cz**. An improved efficiency roll-off of ∼24% at 1000 cd·m^–2^ was also observed.

Godumala *et al*.^1011^ reported a dendrimer, **TB2CZ-ACTRZ** (Figure 130) in which a methylene bridge was used to break conjugation between the peripheral ^*t*^BuCz groups and the DMAC donor, itself connected to a triazine acceptor. In addition, a derivative with one additional generation of carbazole substituents, **TB14CZ-ACTRZ** (Figure 130) was also reported. **TB2CZ-ACTRZ** emits at λ_PL_ of 520 nm, with a Φ_PL_ of 69% in neat film (Table S14), while **TB14CZ-ACTRZ** showed a blue-shifted emission in the neat film at λ_PL_ of 494 nm and a decreased Φ_PL_ of 56%. Further, **TB14CZ-ACTRZ** has a longer τ_d_ of 25 μs compared to 2.9 μs for **TB2CZ-ACTRZ**. The devices with **TB2CZ-ACTRZ** showed an EQE_max_ of 9.9%, which was maintained at 100 cd·m^–2^, although at 1000 cd·m^–2^ the efficiency roll-off grew to ∼52%; a related device without any hole transporting or injection layers showed an EQE_max_ of 9.5%. The device incorporating **TB14CZ-ACTRZ** showed a lower EQE_max_ of 5.5%. Notably, for this material a device without hole transporting layers showed an improved EQE_max_ of 8.1%. The devices for both of these dendrimers and absent of HTLs showed large efficiency roll-off, with efficiency values at 100 cd m^–2^ dropping by ∼58% and ∼63%, respectively.

In order to address charge imbalance, Ban *et al*.^1012^ developed the self-host dendrimer emitter **POCz-DPS** (Figure 130), containing a dicarbazole diphenylsulfone emitting core surrounded by alkyl chains, and with phosphine oxide functionalized carbazole acting as the peripheral host unit. The neat film emits at λ_PL_ of 460 nm and has a Φ_PL_ of 61% (Table S14). The non-doped SP-OLED showed an EQE_max_ of 7.3% at CIE coordinates of (0.18, 0.30) and the efficiency roll-off at 1000 cd·m^–2^ was only ∼19%, although the leakage current was very high.

The same group also compared the performance of two similar self-hosting emitters, one with bipolar carbazole and phosphine oxide containing dendrimers, **poCz-SO**, and the other with just ^*t*^Bu-carbazole dendrimers, **tbCz-SO** (Figure 130).^1013^ **poCz-SO** and **tbCz-SO** emit at λ_PL_ of 458 nm and 440 nm and have very short τ_d_ of only 0.2 μs and 0.1 μs, respectively, as neat films (Table S14). The differences in EQE_max_ of 6.2 and 2.6%, for the devices respective with **poCz-SO** and **tbCz-SO** were attributed to improved charge balance in the former material with bipolar dendrons. This tuning of the charge transport properties also improved efficiency roll-off at 100 cd·m^–2^, which were 11% and 46% for the devices with **poCz-SO** and **tbCz-SO**, respectively.

Li *et al*.^1001^ subsequently reported an asymmetric dendrimer based on **tbCz-SO**, but replacing one of the ^*t*^BuCz donor dendrons with a CzDMAC donor dendron to give **DCz-DPS-TCz** (Figure 130). The neat film emission of **DCz-DPS-TCz** red-shifted to a λ_PL_ of 500 nm, while the Φ_PL_ approached unity at 96%. The τ_d_ is 1.4 μs, which is associated with the small Δ*E*_ST_ of 0.03 eV (Table S14). The non-doped SP-OLED with this dendrimer showed an EQE_max_ of 24% at CIE coordinates of (0.24, 0.45). The efficiency roll-off at 100 cd·m^–2^ was decreased significantly to only 2%, while at 1000 cd·m^–2^ it was still low at 11%. These results demonstrate that significantly enhanced device performance can be achieved by using dendrimers with asymmetrical donor dendrons that carefully balance TADF and charge transport properties.

Most of the materials highlighted so far are green emitters. An exception is the dendrimer reported by Sun *et al*.^1014,1015^ who developed a red self-host dendrimer consisting of CBP peripheral groups attached to a central TPA-anthraquinone D-A TADF core (**MPPA-MCBP**, Figure 130). This dendrimer emits at λ_PL_ of 690 nm but has a low Φ_PL_ of 10% (Table S14). Non-doped devices showed NIR emission with λ_EL_ at 698 nm and an EQE_max_ 0.62%. The efficiency of the SP-OLED was improved compared to devices using just the TADF core (EQE_max_ = 0.11%).

A similar emitter based on the same core but with triCz as the donor dendron, **MPPA-3Cz** (Figure 130), emits further to the red at λ_PL_ of 708 nm, and has a Φ_PL_ of 8% (Table S14). Devices incorporating **MPPA-3Cz** showed even worse EQE_max_ of 0.25% at λ_EL_ of 715 nm compared to **MPPA-MCBP**, owing to the reduced charge balance in this material, a result of replacing the CBP groups with carbazole.

### 10.3 Outlook

A wide range of TADF polymer design strategies has emerged in recent years. Selected relevant representative examples have been presented in this review to highlight their typical photophysical and electroluminescent properties. The majority of the OLEDs with the presented polymers emit within the sky-blue to green color region, with those with **PDT-1**, **PDT-2**, and **PDT-3** ^969^ representing the only devices to achieving blue CIE coordinates of (0.15, 0.08), (0.15, 0.09) and (0.17, 0.14), respectively; however, their EQE_max_ values were low at around 5%. There are as of the end of 2022 no reported red or near-infrared emissive TADF polymers. Of the polymer TADF OLEDs present, arguably the best device performance has been achieved using the green-emitting **PABPC5**,^974^ which showed an EQE_max_ of 18.1% and very low efficiency roll-off, with an EQE_1000_ of 17.8%. However, the emission spectrum is broad due to a combination of a CT emissive state and a distribution of polymers in the sample. Such broad emission from TADF polymers can be addressed by the incorporation of an MR-TADF emitter within the polymers, as exemplified in **PCzBN1**, **PCzBN3**, and **PCzBN5**,^961^ which all showed small FWHM of 27, 34 and 30 nm, respectively. Devices with **PCzBN1** and **PCzBN5** showed EQE_max_ of 17.8 and 17.5%, respectively, demonstrating the potential of this approach.

Another big challenge in this field remains batch-to-batch variation endemic to polymer synthesis, evidenced by the rather large PDI. Finer control of the polymerization is needed in order to achieve polymers with a narrow size distribution. In addition, the monomer ratio (or in this case emitter to host ratio) is a crucial parameter to optimize to obtain suitably high-performance devices, as demonstrated in many of the reports summarized herein. It is also clear that polymers can exhibit reduced ACQ if the ratio of monomers is chosen correctly. Polymer materials do have the advantage of producing high-quality amorphous films and so as a whole they remain a promising class of emitters for high-performance SP-OLEDs. However, to compete with SP-OLEDs using small molecule emitters – especially in terms of properties like color purity (FWHM) and EQE_max_ – still more materials development efforts are required.

Three different classes of TADF dendrimers have been illustrated as an alternative family of macromolecular materials suitable for SP-OLEDs and key relevant examples have been highlighted. The outstanding issues for SP-OLED is a generally lower EQE_max_ and their typically inferior efficiency roll-off compared to vacuum-deposited devices, as seen for a lot of the devices employing polymers and dendrimers as emitters discussed in this section. For dendrimers it has been shown that certain designs can help to address these issues. As a first example, devices incorporating the conjugated dendrimer **tBuCz2m2pTRZ** ^995^ showed the highest EQE_max_ of 28.7% of all the reported dendrimers at green CIE coordinates of (0.37, 0.57), demonstrating that dendrimer TADF-SP-OLEDs can compete with small-molecule TADF SP-OLEDs in terms of their performance. Devices containing the TSCT-dendrimer **YD-TF** ^1007^ as the emitter showed an EQE_max_ of 21.9% and a low efficiency roll-off to 18.6% at a luminance of 1000 cd m^–2^ at CIE coordinates of (0.41, 0.54) when doped into a dendrimeric host. An impressive device performance using a non-conjugated dendrimer as the emitter, employed the asymmetrically substituted dendrimer **DCz-DPS**-**TCz**,^1001^ which showed an EQE_max_ of 24.0% and an EQE_500_ of 21.3% at CIE coordinates of (0.24, 0.45). These three examples demonstrate that dendrimers represent a potent alternative class of emitters compared to small molecules and polymers in SP-OLEDs.

Dendrimers possess a balance of desirable properties that makes them attractive for SP-OLEDs. Due to their size, they are amenable for solution-processing fabrication techniques like polymers. Additionally, ACQ can be mitigated in all three classes of TADF dendrimers, exemplified by the performance of several examples of non-doped devices highlighted in this section. While likewise having good film-forming properties as do polymers, dendrimers also enjoy having a well-defined molecular structure, so there is no batch-to-batch variation and purification can be readily achieved. Dendrimers can also employ donor dendrons that have embedded charge transport units to help address charge balance in the SP-OLED, and the considerable flexibility in terms of dendron design will likely support both imaginative future material strategies as well as improved overall device performance in this area.

## 11 Multi-resonance TADF

### 11.1 Introduction

The most popular TADF emitter design strategy relies on highly twisted donor-acceptor architectures to reduce the exchange integral and hence Δ*E*_ST_. The most prominent recent examples have been documented in Sections 3–5. Though there are many examples of high-efficiency OLEDs using emitters with this design, the emission spectrum is frequently broad and unstructured, reflective of the CT nature of the excited state and inherent conformational flexibility. To account for varying bandshapes, the width of the emission is primarily quantified at half the emission intensity maximum (FWHM, Figure 131), with D-A TADF materials typically having FWHM of 80–100 nm. Narrower emission spectra can more easily achieve high color saturation, which is required for commercial displays to meet industry-standard color space coverage. Standard red blue green (sRBG) coordinates have defined CIE coordinates of (0.64, 0.33), (0.15, 0.06) and (0.30, 0.60) respectively, which for emitters with large FWHM require subtractive filtering to achieve, sacrificing overall emission efficiency.^9^ The more recent standard Rec. 2020 defines blue, green, and red CIE coordinates to be (0.13, 0.05), (0.17, 0.80) and (0.71, 0.29), respectively, which are even more challenging for D-A TADF emitters to acheive.^10^

**Figure 131:**
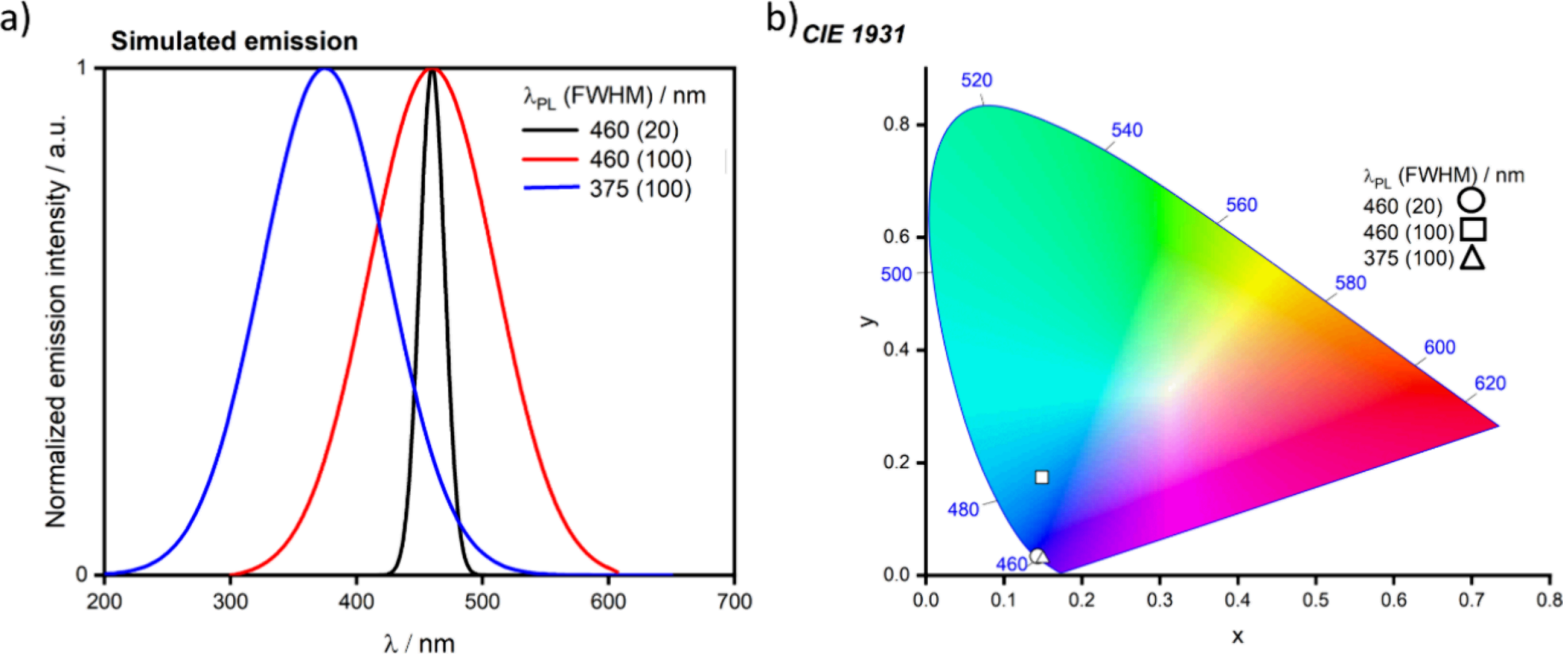
a) Simulated 460 nm emission spectra with FWHM of 100 nm (red) and 20 nm (black), and simulated 375 nm emission spectrum with FWHM of 100 nm (blue); b) Corresponding CIE color coordinates.

To demonstrate this challenge, Figure 131 shows two simulated emission spectra with the same maximum at 460 nm, and with FWHM of 20 nm or 100 nm. These emission spectra correspond to CIE coordinates of (0.14, 0.03) and (0.15, 0.17), with the narrower spectrum having a far more saturated blue emission despite a considerably lower high-energy onset – itself a significant benefit for device host choice and lifetime. To achieve similar CIE coordinates of (0.14, 0.04) with an emission spectrum with a 100 nm FWHM, the λ_PL_ would need to be 375 nm (Figure 131). An emitter design with these metrics would be very challenging and no host exits with a suitably high triplet energy to accommodate such an emitter.

In contrast to CT emission in D-A TADF molecules, narrowband emission can be achieved within an exciting sub-class of TADF materials based mainly on p- and n-doped nanographene fragments, termed multiresonant TADF (MR-TADF) emitters. Pioneered by Hatakeyama and co-workers,^118^ these materials exploit complementary resonance effects, with the electron density distribution of the HOMO and LUMO localized on neighboring atoms in the heteroacene, setting up short-range charge transfer (SRCT) excitons and ensuring a sufficiently small Δ*E*_ST_ to turn on TADF (Figure 132).^45^ Crucially, MR-TADF emitters possess a rigid structure with little change in the geometry from the ground to the excited state, resulting in small Stokes shifts and narrowband emission profiles with only minor contributions from vibronic bands.^162^ The emissive SRCT excited states also have considerably subdued solvatochromism compared to the long-range CT states in D-A compounds.^138,162^

**Figure 132:**
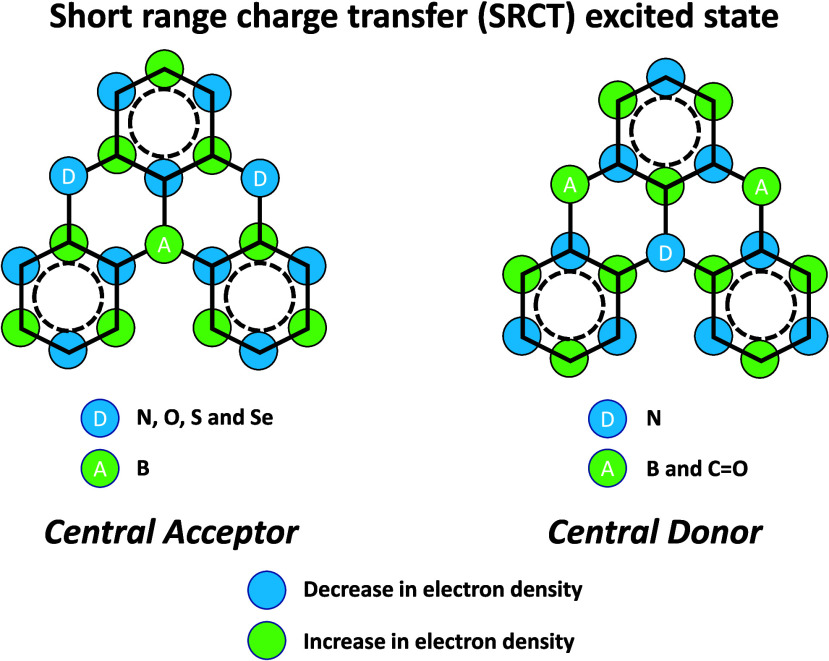
Schematic representation of the difference density plots (S_0_-S_1_) for the short-range charge transfer excited states in triangulene-based MR-TADF compounds, containing either a central acceptor (left) or donor atom (right).

The SRCT excited states in MR-TADF materials can be clearly visualised using computed difference density plots. In these, the alternating pattern of increasing and decreasing electron density on neighboring atoms in the excited states (relative to the ground state) reveals the alternating charge-transfer interactions (Figure 132). This is in stark contrast to LRCT excited states in D-A materials, in which electron density migrates large distances from the donor part of the molecule to the acceptor (See Section 2). This different category of excited states, and particularly the confinement of electrons and holes in the nanographene fragment (with significant correlation and exchange interaction), necessitates the use of multireference methods rather than simpler DFT when calculating properties of these materials.

The structural diversity of MR-TADF emitters remains small at present. However, there are nonetheless now more than 250 reported examples, including some that approach the Rec. 2020 standard for each of blue, green, and red emission. In some cases, these materials also have exceptional efficiencies in devices, especially when supported by assistant dopants in hyperfluorescence OLEDs. Examples highlighted in this section have been grouped together based on common structural motifs, with their properties discussed and OLED performance cross-compared (Table S15).

### 11.2 Central Acceptor Structures

#### 11.2.1 Early MR-TADF Emitters, DABNA and Its Derivatives

The most common design for MR-TADF emitters incorporates a central boron as the acceptor atom with oxygen or nitrogen atoms acting as the donors (Figure 132). The first examples of MR-TADF emitters reported by Hatakeyama and co-workers^232^ possess this motif as exemplified by compound **2a** (Figure 133, later called **DOBNA** or **BOO**).^179^ Measurements in solution showed a Δ*E*_ST_ of 0.15 eV (fluorescence measured in DCM and phosphorescence measured in EtOH); however, no time-resolved PL was collected to substantiate TADF activity.^232^ The photophysics of this compound was revisited recently,^179^ and the authors reported data for 1 wt% PMMA films, with a Δ*E*_ST_ of 0.18 eV and λ_PL_ of 398 nm with a τ_d_ of 66 μs. No devices were fabricated in either of these reports, likely owing to the near-UV emission and lack of suitable host. Other derivatives of **DOBNA** are discussed in the “DOBNA derivatives” sub section.

**Figure 133:**
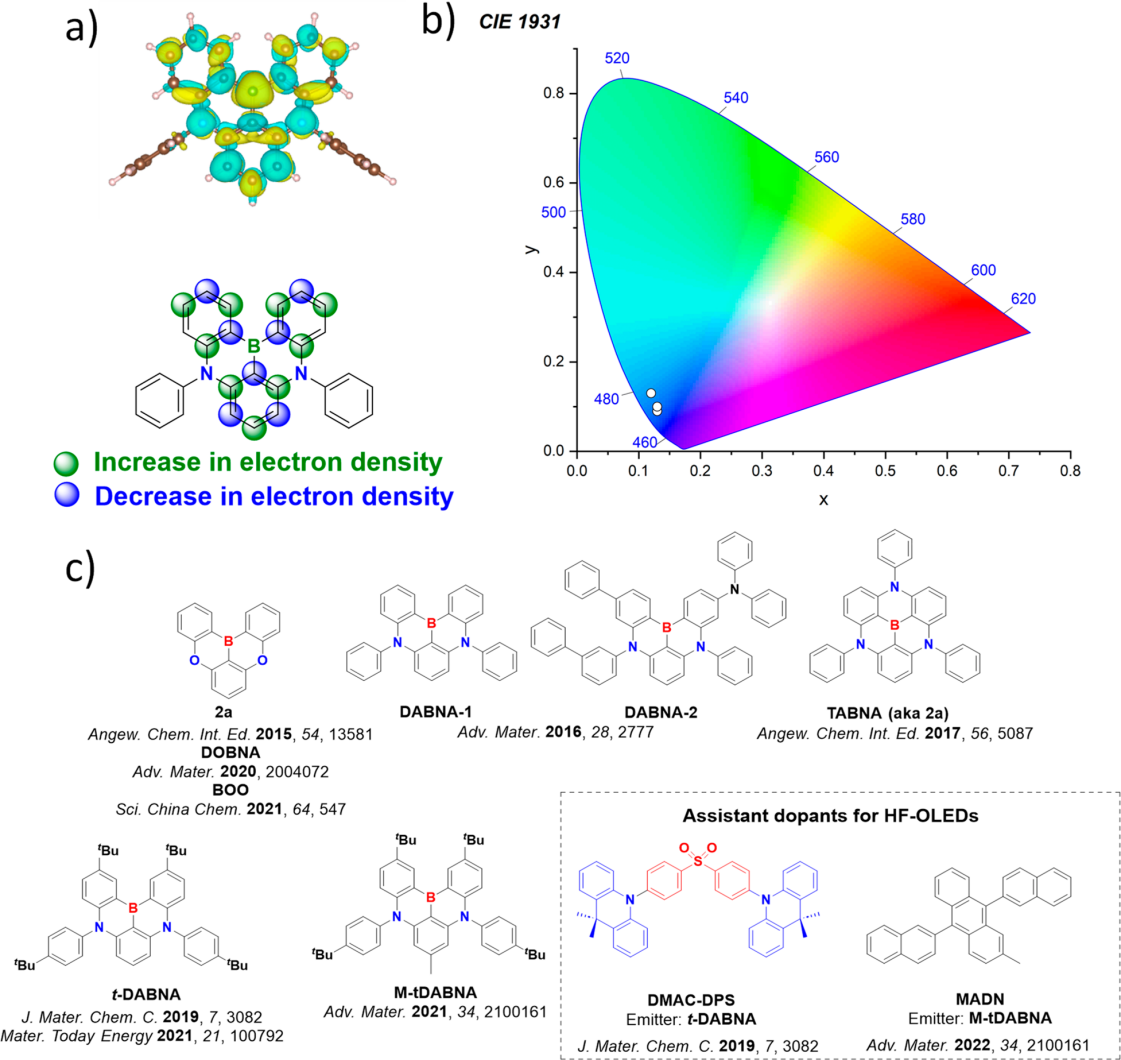
a) Computed difference density plot (top) and the schematic representation of the difference density distribution of **DABNA-1** (bottom), b) CIE coordinates of OLEDs with DABNA derivatives, and c) structures of early DABNA derivatives and HF-OLED assistant dopants (the blue color signifies donor moieties/atoms/functional groups, while the red color signifies acceptor moieties/atoms/functional groups). Difference density plots calculated at the SCS-CC2/cc-pVDZ level in the gas phase; is-value = 0.01.

Hatakeyama and co-workers subsequently reported the emitters **DABNA-1** and **DABNA-2**, where the oxygen donor atoms were replaced by nitrogen atoms within DPA groups fused to a central boron atom (Figure 133), and with **DABNA-2** featuring additional DPA and phenyl substituents.^118^ Excellent Φ_PL_ values in 1 wt% mCBP films of 88 and 90% were achieved for **DABNA-1** and **DABNA-2**, respectively, with λ_PL_ red-shifted compared to **DOBNA** (λ_PL_ of 398 nm in 1 wt% PMMA)^179^ at 460 nm for **DABNA-1** and 469 nm for **DABNA-2** in 1 wt% mCBP.^118^ As will become evident for most MR-TADF compounds, moderately large Δ*E*_ST_ of 0.18 and 0.14 eV and associated long τ_d_ of 94 and 65 μs were reported for **DABNA-1** and **DABNA-2**, respectively. Vacuum-deposited OLEDs with **DABNA-1** and **DABNA-2** as the emitter showed EQE_max_ of 13.5 and 20.2%, respectively. The most attractive feature of these OLEDs is their narrow FWHM at 28 nm, that ensured pure blue emission with CIE coordinates of (0.13, 0.09) and (0.12, 0.13) for the devices with **DABNA-1** and **DABNA-2**, respectively. This report was the first example of MR-TADF emitters employed in devices; however, despite the promising EQE_max_ values, the devices suffered from severe efficiency roll-off with EQE_100_ of 6.3 and 13.3%. A luminance of 1,000 cd m^–2^ was not achieved by either device. This efficiency roll-off stems from the large Δ*E*_ST_ and associated long τ_d_ of these materials, a feature still commonly reported for MR-TADF materials to this day. A related *D*_3_-symmetric derivative of **DABNA-1**, **TABNA** (named **2a** in the original report, Figure 133)^1016^ showed a moderate Φ_PL_ of 54% in 1 wt% PMMA and a comparable Δ*E*_ST_ of 0.21 eV to **DABNA-1**. A narrow FWHM of 28 nm at a λ_PL_ of 399 nm was observed, although no devices were reported.

***t*-DABNA**, a *tert*-butyl decorated analogue of **DABNA-1**, was reported by Han *et al.* (Figure 133).^1017^ This compound was used as both an emitter in an OLED and as the terminal emitter in a hyperfluorescence OLED with **DMAC-DPS** (Figure 133) as the assistant dopant. In 5 wt% DPEPO films the Φ_PL_ of ***t*-DABNA** is 85%, the Δ*E*_ST_ is 0.17 eV (similar to **DABNA-1** with Δ*E*_ST_ = 0.18 eV), and the τ_d_ is 83 μs. The OLED showed a promising EQE_max_ of 25.1%, but the efficiency roll-off was again severe (EQE_100_ of 6.0%) owing to the slow RISC and long delayed lifetime. The EQE_max_ of the HF device was 31.4% and the efficiency roll-off improved considerably, with EQE_100_ of 27%. The HF OLED strategy and mechanism are discussed in detail in Section 17 and has proven popular for OLEDs employing MR-TADF terminal emitters, as this strategy can mitigate the slow *k*_RISC_ in these compounds while preserving their valuable narrow FWHM emission. A related derivative of ***t*-DABNA** with a methyl substituent *para* to the boron, **M-tDABNA**,^1018^ shows improved orientation of its TDM (Figure 133). **M-tDABNA** has a λ_PL_ of 461 nm, a Φ_PL_ of 84%, and a τ_d_ of 195 μs in 3 wt% mCBP films, while the Δ*E*_ST_ in toluene is 0.11 eV. An OLED employing a TTA assistant dopant, **MADN** (Figure 133), showed an EQE_max_ of 8.6% and CIE coordinates of (0.14, 0.08) using this MR-TADF terminal emitter.

#### 11.2.2 Substituted DABNA Derivatives

***t*-DABNA** has since been revisited by Kim *et al*., where it was investigated alongside a donor-decorated derivative (***t*-DAB-DPA**, Figure 134).^1019^ Improved device performance was demonstrated compared to the previous report when ***t*-DABNA** was doped in a mixed host system (mCBP:​mCBP-CN). The OLED showed an EQE_max_ of 28.4%, but the efficiency roll-off was large (EQE_100_ of 14.8%). Increasing the concentration of the emitter from 3 to 10 wt% resulted in ACQ and the EQE_max_ reached only 21.3%. The addition of peripheral ^*t*^BuCz groups to the ***t*-DABNA** core as in **TBN-TPA** suppressed the ACQ (Figure 134 and Figure 149);^1020^ notably, the structure of the emitter was initially wrongly identified,^1021^ and it was subsequently shown that the structure was in fact that of **CzDABNA-NP-TB** (Figure 149). This emitter is discussed in detail alongside **CzBN** derivatives (*vide infra*).

**Figure 134:**
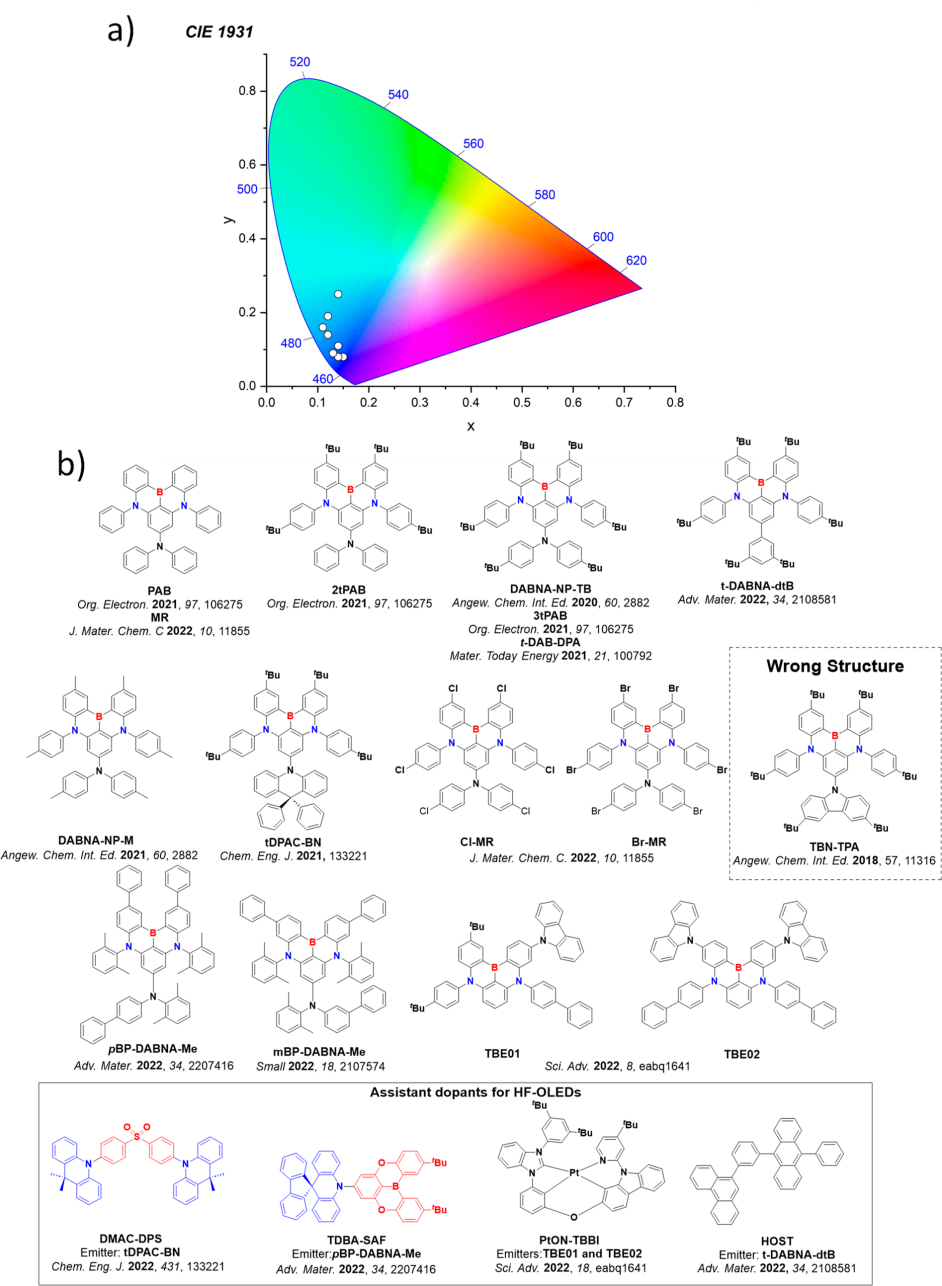
a) CIE color coordinates of OLEDs with substituted DABNA emitters, and b) structures of the substituted DABNA emitters and HF-OLED assistant dopants. The white circles of the CIE diagram illustrate the spread of the emission color of the devices. In the chemical structures, the blue color signifies donor moieties/atoms/functional groups, while the red color signifies acceptor moieties/atoms/functional groups.

Two derivatives of **DABNA-1** were reported that contained a third diarylamine donor as well as pendant methyl or ^*t*^Bu groups on the diarylamines: **DABNA-NP-M** and **DABNA-NP-TB** (Figure 134).^1021^ The addition of the third donor group in this case had minimal impact on the photophysical properties, with similar λ_PL_ of 460 and 453 nm and Φ_PL_ of 88 and 83% in 1 wt% PMMA, respectively for **DABNA-NP-M** and **DABNA-NP-TB**, compared to **DABNA-1** (λ_PL_ of 460 nm and Φ_PL_ 88% in 1 wt% mCBP).^118^ The Δ*E*_ST_ value for both emitters in 1 wt% PMMA film was 0.17 eV and each showed similar τ_d_ of 89 and 90 μs,^1021^ respectively, which are again similar to the values reported for **DABNA-1** (Δ*E*_ST_ = 0.18 eV and τ_d_ = 94 μs).^118^ This demonstrates that these structural changes can in some cases have only a minimal impact, contrary to the conclusions drawn in the original report of the wrongly identified emitter **TPN-TPA**.^1020^ Devices with **DABNA-NP-TB** showed an EQE_max_ of 19.5% at CIE (0.14, 0.11) and relatively low efficiency roll-off of 10% at 100 cd m^–2^,^1021^ which was an improvement compared to the OLED with **DABNA-1** (EQE_max_ = 13.5%, efficiency roll-off at 100 cd m^–2^ = 53%).^118^

An investigation into the effect of ^*t*^Bu substitution was recently conducted by Wang *et al*.^1022^ Three emitters including **DABNA-NP-TB** were presented, containing differing numbers of ^*t*^Bu substituents with **PAB** having none, **2tPAB** containing four ^*t*^Bu groups on the **DABNA-1** core, and **3tPAB** (identical to **DABNA-NP-TB**) substituted on both the **DABNA-1** core and the DPA donor (Figure 134). The introduction of the additional ^*t*^Bu groups resulted in a very small red-shift of the emission across the series, with λ_PL_ of 453, 457, and 458 nm for **PAB**, **2tPAB**, and **3tPAB**, respectively, alongside similar τ_d_ and Δ*E*_ST_ (τ_d_ of between 56 and 77 μs and Δ*E*_ST_ ranging from 0.06 to 0.10 eV). Aside from intrinsic optical properties, addition of the ^*t*^Bu groups helped to suppress ACQ, evidenced by the steady increase in Φ_PL_ from 61 and 67 to 75% for **PAB**, **2tPAB**, and **3tPAB**, respectively, in 3 wt% mCP films. Devices with **PAB** and **2tPAB** showed EQE_max_ of 14.7 and 16.8% at CIE (0.15, 0.08), while the device with for **3tPAB** showed a higher EQE_max_ of 19.3% at CIE (0.14, 0.08), which was correlated with the Φ_PL_.

**3tPAB** (renamed as ***t*-DAB-DPA**) was subsequently investigated alongside ***t*-DABNA** by Kim *et al*. (Figure 133 and Figure 134).^1019^ The authors focused on reducing ACQ by decorating a peripheral donor group of ***t*-DAB-DPA**. Moving from 3 to 10 wt%, the OLEDs with ***t*-DABNA** saw the EQE_max_ decrease from 28.4 to 21.3%, while for devices with ***t*-DAB-DPA** the decrease in EQE_max_ was attenuated, decreasing from 27.6 to 25.9%. For the 3 wt% emitter doped devices, there was a corresponding modest blue-shift of the emission, reflected in the CIE coordinates of (0.13, 0.10) and (0.14, 0.08) for the devices with ***t*-DABNA** and ***t*-DAB-DPA**, respectively. As will be made clear by following examples, addressing the strong ACQ in MR-TADF emitters that arises from their large, planar and electron-rich structures has become a key concern for improving the overall performance across the research community.

A similar strategy to suppress ACQ was used by Park *et al.*, where a bulky di-*tert*-butyl phenyl substituent was added *para* to the boron atom in **t-DABNA-dtB** (Figure 134).^1023^ A near unity Φ_PL_ of 99% was reported for the 3 wt% doped film in mCBP, which also showed Δ*E*_ST_ of 0.19 eV and τ_d_ of 110 μs. ACQ was found to be more severe for reference compound ***t*-DABNA**, where the Φ_PL_ of the 3 wt% film in mCBP was 87% but dropped to 65% for the 7 wt% doped film, while the Φ_PL_ of the 7 wt% doped film with **t-DABNA-dtB** remained as high as 96%. The corresponding OLEDs showed an EQE_max_ of 25.5%, but the EQE_100_ was only 5.4% and the LT_95_ < 1 hour at 100 cd m^–2^. When employed in a HF-OLED in conjunction with an anthracene-based TTA assistant dopant (**HOST**, Figure 134), the EQE_max_ was much lower at 11.4% (limited by the TTA assistant dopant) yet the device stability improved markedly with LT_95_ of 13,124 hours at 100 cd m^–2^.

Lee *et al*. investigated the effect of heavy atom inclusion on MR-TADF emission using derivatives of **PAB** (renamed **MR** here, Figure 133),^1024^ incorporating chlorine (**Cl-MR**, Figure 134) and bromine (**Br-MR**, Figure 134). Calculations revealed the profound impact of the substituents on SOC between S_1_ and T_2_, increasing from 0.19 cm^–1^ in **MR** to 0.68 cm^–1^ in **Cl-MR** and 2.21 cm^–1^ in **Br-MR**. A small red-shift in the emission with halogen substitution was observed in 10 wt% doped DPEPO films, with λ_PL_ of 456, 474, and 474 nm for **MR**, **Cl-MR**, and **Br-MR**, respectively, while each showed identical Δ*E*_ST_ of 0.13 eV. The influence of the heavy atoms was evident in the TADF kinetics, with increasing *k*_RISC_ of 2.8, 9.8, and 59 × 10^4^ s^–1^ for **MR**, **Cl-MR**, and **Br-MR**, respectively. Despite the higher *k*_RISC_ and comparable Φ_PL_ of 75–85%, the devices with **MR** and **Cl-MR** showed comparable EQE_max_ of 16 and 17%, while the device with **Br-MR** the EQE_max_ was only 4.2%. The decreased EQE_max_ of **Br-MR** was attributed to lower bond dissociation enthalpy values within the emitter, leading to its degradation under electrical stress.

Wang *et al*. reported a derivative of ***t*-DABNA** containing a DPAC donor attached to the ***t*-DABNA** core, **tDPAC-BN** (Figure 134).^1025^ The 1 wt% doped film of **tDPAC-BN** in PMMA emits at λ_PL_ of 454 nm, has a Δ*E*_ST_ of 0.17 eV in toluene and a τ_d_ of 114 μs in 1 wt% doped film. The OLED showed a modest EQE_max_ of 12.4% at CIE (0.14, 0.08), dropping to 1.6% at 100 cd m^–2^. Hyperfluorescent devices with **DMAC-DPS** (Figure 134) as the assistant dopant showed an improved EQE_max_ of 21.6% and reduced efficiency roll-off (EQE_100_ = 15.3%).

Cheon *et al*. developed a derivative of **DABNA-1** containing bulky groups designed to suppress ACQ, ***p*BP-DABNA-Me** (Figure 134).^1026^ Biphenyls were added to preventing intermolecular π-stacking, while xylyl groups were added to further reduce ACQ and supress rotational vibrations. ***p*BP-DABNA-Me** shows both a narrower FWHM of 22 nm and higher Φ_PL_ of 98% compared to **DABNA-1** (30 nm and 79%) in the same 5 wt% DPEPO:​mCBP host, but emits at λ_PL_ of 462 nm, identical to **DABNA-1**. Despite a similar Δ*E*_ST_ of 0.18 eV to **DABNA-1** (0.17 eV) the *k*_RISC_ was also faster for ***p*BP-DABNA-Me** at 6.85 × 10^4^ s^–1^, compared to 0.99 × 10^4^ s^–1^ for **DABNA-1**. The enhanced *k*_RISC_ was attributed to the introduction of closely lying ^3^LE states of the biphenyl groups, which according to computations facilitated RISC via spin-vibronic coupling. Devices showed EQE_max_ of 23.4% at CIE (0.13, 0.09). Non-doped OLEDs showed much poorer performance, with an EQE_max_ of 10.1%; however, there was little evidence of aggregation as the CIE coordinates were only slightly red-shifted to (0.14, 0.10). When utilized in HF-OLEDs with **TDBA-SAF** (Figure 134) as the assistant dopant the EQE_max_ rose to 30.1%.

A similar derivative was reported by the same group,^1027^ where the phenyl substituents of ***p*BP-DABNA-Me** were instead positioned *meta* to the nitrogen donor atoms to give **mBP-DABNA-Me** (Figure 134). In 5 wt% mCP:​DPEPO films the λ_PL_ is 467 nm and the Φ_PL_ is 97%, similar to ***p*BP-DABNA-Me**. The *k*_RISC_ of **mBP-DABNA-Me** was determined to be 1.95 × 10^4^ s^–1^, slower than that of ***p*BP-DABNA-Me** and indicating that the contribution of LE biphenyl triplet states was less effective over *meta* linkages than *para* ones. The OLEDs showed an EQE_max_ of 24.3% at λ_EL_ 468 nm, while the EQE_1000_ dropped to 9.1%. Owing to the bulky nature of the emitter, the CIE coordinates remained impressively constant at (0.12, 0.14) at 0.5, 5, and 25 wt% emitter doping in the EML of the OLEDs.

Two carbazole- and biphenyl-decorated **DABNA-1** derivatives, **TBE01** and **TBE02** (Figure 134), were developed to improve the performance of HF-OLEDs compared to ***t*-DABNA**.^1028^ These emitters were designed to have larger Förster radii compared to ***t*-DABNA**, while their bulkier size would help to suppress Dexter energy transfer. **TBE01** and **TBE02** showed identical λ_PL_ of 459 nm and FWHM of 21 nm, with Φ_PL_ of 91 and 89%, respectively in toluene. Compared to ***t*-DABNA** (0.21 eV), these derivatives have smaller Δ*E*_ST_ of 0.16 and 0.14 eV, respectively, in toluene solution, which translates into faster *k*_RISC_ of 0.27, 0.51, and 1.03 × 10^4^ s^–1^ for ***t*-DABNA**, **TBE01**, and **TBE02**, respectively (in exciplex host SiCzCz:​​SiTrzCz2 at 0.4 wt% emitter doping). HF-OLEDs with **PtON-TBBI** (Figure 134) acting as the assistant dopant showed similar EQE_max_ (EQE_1000_) of 27.9% (25.4%) and 29.1% (25.8%) for the devices with **TBE01** and **TBE02**, respectively, compared to 28.1% (23.7%) for the device with ***t*-DABNA**. The devices with the substituted emitters were considerably more stable though, with LT_95_ of 42.3 and 72.9 hours, compared to 19.8 hours for ***t*-DABNA**.

#### 11.2.3 DOBNA Derivatives

There have now been numerous derivatives of **DOBNA** reported since the initial paper by Harai *et al*. (Figure 133),^179^ with sulfur also used as a donating atom. Using **DOBNA** as the MR-TADF core (renamed **BOO**, Figure 133), Chen *et al*. reported a series of polymers containing this unit as well as two sulfur-containing analogues, **BOS** and **BSS** (Figure 135).^1029^ The monomer emitters **BOO**, **BOS**, and **BSS** emitted at λ_PL_ of 396, 434, and 457 nm respectively, with Δ*E*_ST_ 0.18, 0.17, and 0.15 eV in toluene. In 1 wt% polystyrene each monomer showed Φ_PL_ of 70, 63, and 58%, while their *k*_RISC_ steadily increased from 1.1 to 6.1 and 11.8 × 10^4^ s^–1^ for **BOO**, **BOS**, and **BSS**, respectively owing to the heavy atom effect. These were next incorporated into non-conjugated polymers **PS-BOO**, **PS-BOS**, and **PS-BSS** (Figure 135). Neat films of **PS-BOO**, **PS-BOS**, and **PS-BSS** emitted at λ_PL_ of 398, 435, and 456 nm, respectively and showed τ_d_ of 133.8, 104.9, and 67.0 μs, but no devices were fabricated. Copolymerisation with an acridan monomer to act as a hole-transporting host afforded polymers **PAc-BOO**, **PAc-BOS**, and **PAc-BSS** (Figure 135). The neat films of **PAc-BOO** and **PAc-BOS** showed significant spectral changes compared to **PS-BOO** and **PS-BOS**, with λ_PL_ of 457 and 434/475 nm, respectively, as a new LRCT state between electron-donating PAc and the MR-TADF core gave emission peaks at 457 and 475 nm; such behavior was not observed for **PAc-BSS**, which retained narrowband emission centred around 455 nm. Solution-processed devices of **PAc-BSS** likewise emitted at λ_EL_ of 458 nm and FWHM of 31 nm, with corresponding CIE coordinates of (0.16, 0.12) and an EQE_max_ of 13.1%. Several works applying MR-TADF emissive subunits within polymers are highlighted in Section 10.

**Figure 135:**
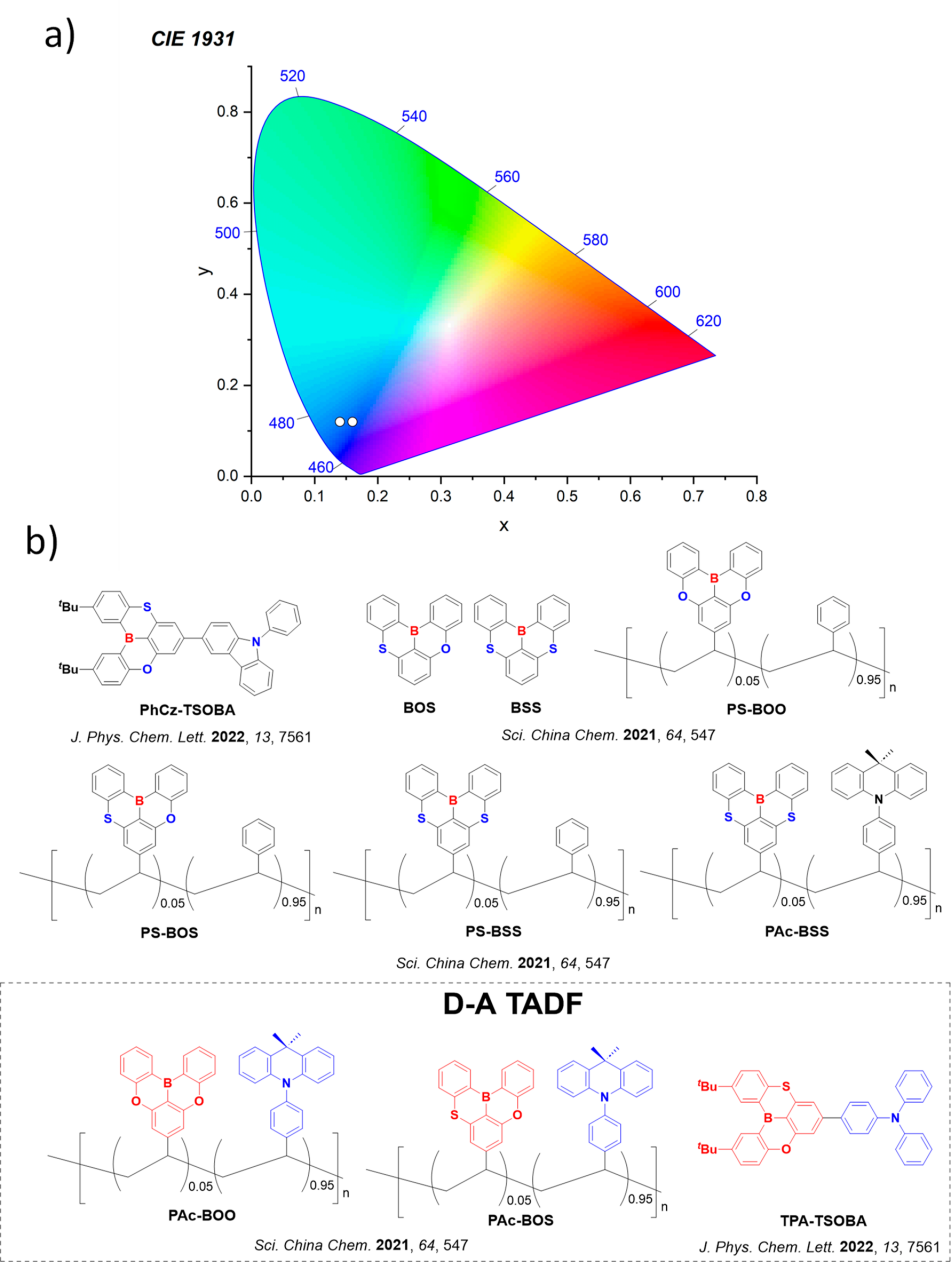
a) CIE color coordinates of OLEDs with DABNA derivatives bearing multiple acceptor atoms as emitters and b) structures of chalcogen derivatives of DOBNA. The white circles of the CIE diagram illustrate the spread of the emission color of the device. In the chemical structures, the blue color signifies donor atoms/functional groups, while the red color signifies acceptor atoms/functional groups.

Gao *et al*. reported two similar emitters based on **BOS** containing either triphenylamine and phenylcarbazole substituted *para* to the boron, **TPA-TSOBA** and **PhCz-TSOBA** (Figure 135).^317^ Based on calculations and the large observed positive solvatochromism they assigned the emission of **TPA-TSOBA** to LRCT typical of D-A compounds, while **PhCz-TSOBA** was classified as MR-TADF. **PhCz-TSOBA** emits at λ_PL_ of 444 nm (FWHM of 32 nm) and has a Δ*E*_ST_ of 0.23 eV in toluene. As a 10 wt% doped film in 2,6-DczPPy the Φ_PL_ is 61% and the *k*_RISC_ was measured to be 4.1 × 10^4^ s^–1^. The OLEDs showed an EQE_max_ of 16.7% at CIE (0.14, 0.12), while the EQE_100_ was 6.7%. Despite **TPA-TSOBA** being assigned as a D-A emitter, it showed very similar properties in both films and devices to **PhCz-TSOBA**, suggesting this assignment may not apply when dispersed in solid OLED hosts.

#### 11.2.4 DABNA Derivatives with Multiple Acceptor Atoms

Beyond materials based on the **DABNA** or **DOBNA** cores, a number of π-extended systems have been reported featuring an expanded network of donating or withdrawing atoms, Figure 136. In 2018, the group of Hatakeyama introduced this extended design strategy,^1030^ wherein they altered the number of boron atoms across the emitters **B2**, **B3**, and **B4** (Figure 136). These three compounds showed moderate Φ_PL_ values of 53, 33, and 57%, with Δ*E*_ST_ of 0.19, 0.15, and 0.15 eV as 1 wt% doped films in PMMA. **B2**, **B3**, and **B4** all showed blue emission with λ_PL_ of 455, 441, and 450 nm and FWHM of 32, 34, and 38 nm, respectively. Of the three, only **B2** was used as an emitter in a device, which performed similarly to the device with **DABNA-2** with EQE_max_ of 18.3% and EQE_100_ of 12.6% compared to 20.2 and 12.4%, respectively, for the OLED with **DABNA-2**.^118^

**Figure 136:**
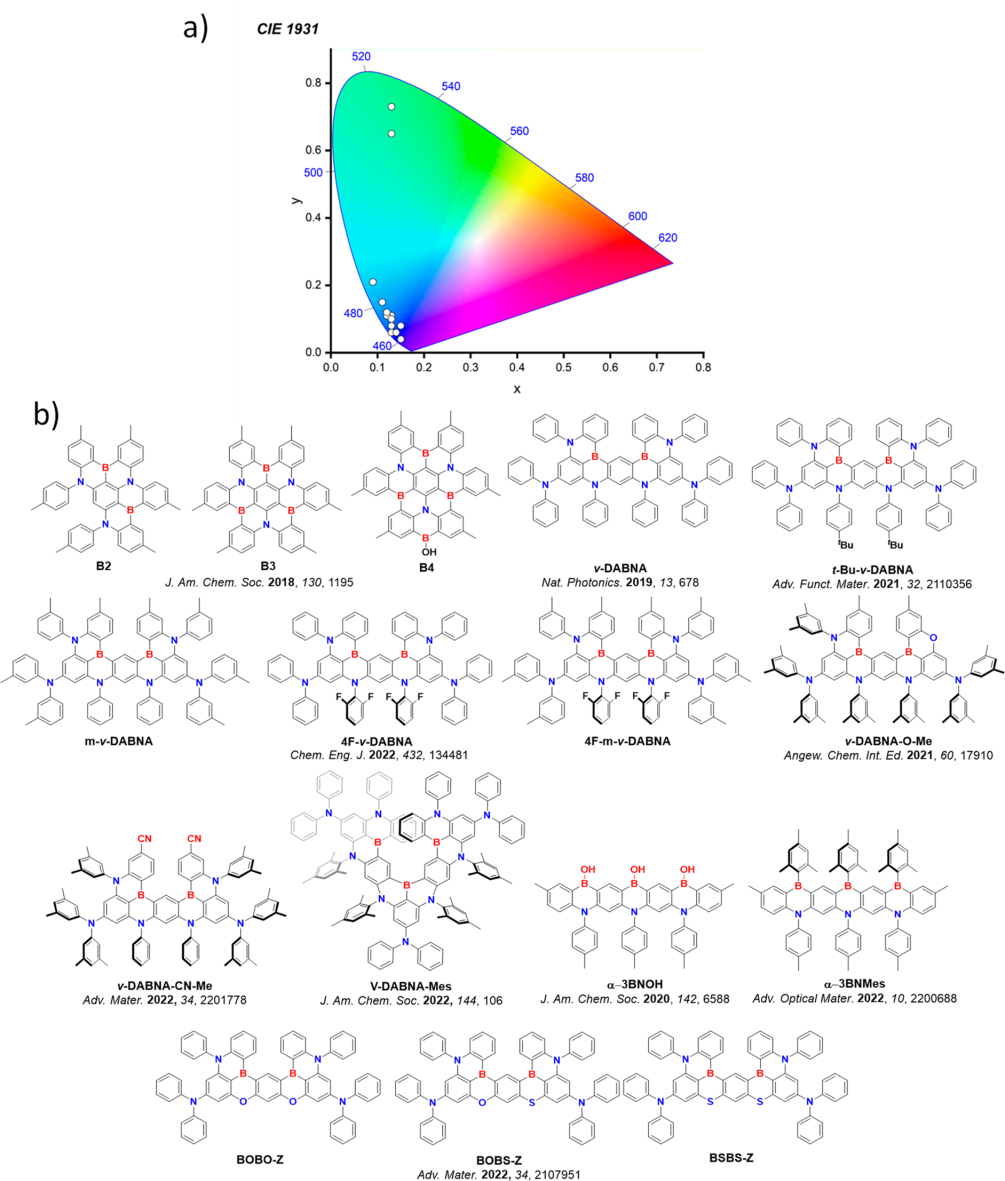
a) CIE color coordinates of OLEDs with DABNA derivatives bearing multiple acceptor atoms as emitters and b) structures of DABNA emitters with multiple acceptor atoms. The white circles of the CIE diagram illustrate the spread of the emission color of the device. In the chemical structures, the blue color signifies donor atoms/functional groups, while the red color signifies acceptor atoms/functional groups.

The blue-emissive linearly extended emitter, ***v*-DABNA** (Figure 136),^1031^ was subsequently reported by the same group. This compound emits sharply at λ_PL_ of 467 nm, has a Φ_PL_ of 90% and a small Δ*E*_ST_ of 0.02 eV at 1 wt% in the bespoke host DOBNA-OAr. DOBNA-OAr is an arylated derivative of **DOBNA** and is a rare example of an MR-TADF compound used as a host. Unlike most other MR-TADF emitters, ***v*-DABNA** showed a relatively efficient *k*_RISC_ of 2.0 × 10^5^ s^–1^ likely due to its small Δ*E*_ST_; most other MR-TADF emitters have Δ*E*_ST_ of > 0.10 eV and RISC in the range of 1–10 × 10^4^ s^–1^. There was no explanation initially provided for why this compound shows such a small Δ*E*_ST_; however, subsequent work has hypothesized that having an extended π-network is key to a small Δ*E*_ST_ in this class of compounds.^138^ The OLEDs showed an EQE_max_ of 34.4% at CIE (0.12, 0.11), representing one of the most efficient blue TADF emitters to date.^1031^ Further, the device showed minimal efficiency roll-off, with an EQE_1000_ of 26.1% owing to the efficient *k*_RISC_ that results in decreased triplet quenching that often plagues MR-TADF OLEDs. The narrowband emission of the ***v*-DABNA** combined with its excellent performance (supported by spontaneous horizontal emitter TDM alignment in films) has since sparked significant further research effort.

Following its introduction to the field, ***v*-DABNA** has been frequently used as a terminal emitter material in HF-OLEDs.^253,1032^ When a triplet harvesting assistant dopant **HDT-1** (Figure 137) was used alongside ***v*-DABNA** acting as the terminal emitter, the OLED showed an EQE_max_ of 41% at CIE (0.13, 0.16).^1032^ The device stability was improved to LT_95_ of 18 hours at 1,000 cd m^–2^, compared to < 1 hour in the parent device at 100 cd m^–2^. The *k*_RISC_ of **HDT-1** at 8.6 × 10^5^ s^–1^ is faster than that of ***v*-DABNA** (*k*_RISC_ = 2.0 × 10^5^ s^–1^), supporting efficient triplet harvesting separate to emission. When **PPCz­Trz** and **PCz­Trz** (Figure 137) were used as assistant dopants, the OLEDs showed EQE_max_ of 33.0 and 33.5%, respectively.^253^ In each device the efficiency roll-off was low, with EQE_1000_ of 25.2 and 23.8%, respectively. The device stability improved as well, with LT_50_ at 1000 cd m^–2^ of 151 and 113 hours for **PPCz­Trz** and **PCz­Trz**, respectively. In another report, HF devices with **DMT­Dac-Me** (Figure 137) as the assistant dopant performed better than the one that only included ***v*-DABNA** in the EML; the EQE_max_ of the device with 1 wt% ***v*-DABNA** was 13.3%, while the 0.5 wt% device showed an EQE_max_ of 22.2%.^1033^ This work highlighted that although the isolated performance of ***v*-DABNA** is exceptional, it still suffers considerably from ACQ and excimer formation at practical concentrations. Using the exciplex host **3Cz-TRZ**:​**Tris-PCz** (Figure 137) alongside ***v*-DABNA**, Nguyen *et al*. reported stable devices with an LT_50_ of over 300 hours at an initial luminance of 1260 cd m^–2^.^736^ The exciplex host contributed to the triplet harvesting, although broadening of the emission compared to ***v*-DABNA** alone indicates that energy transfer was not complete.

**Figure 137:**
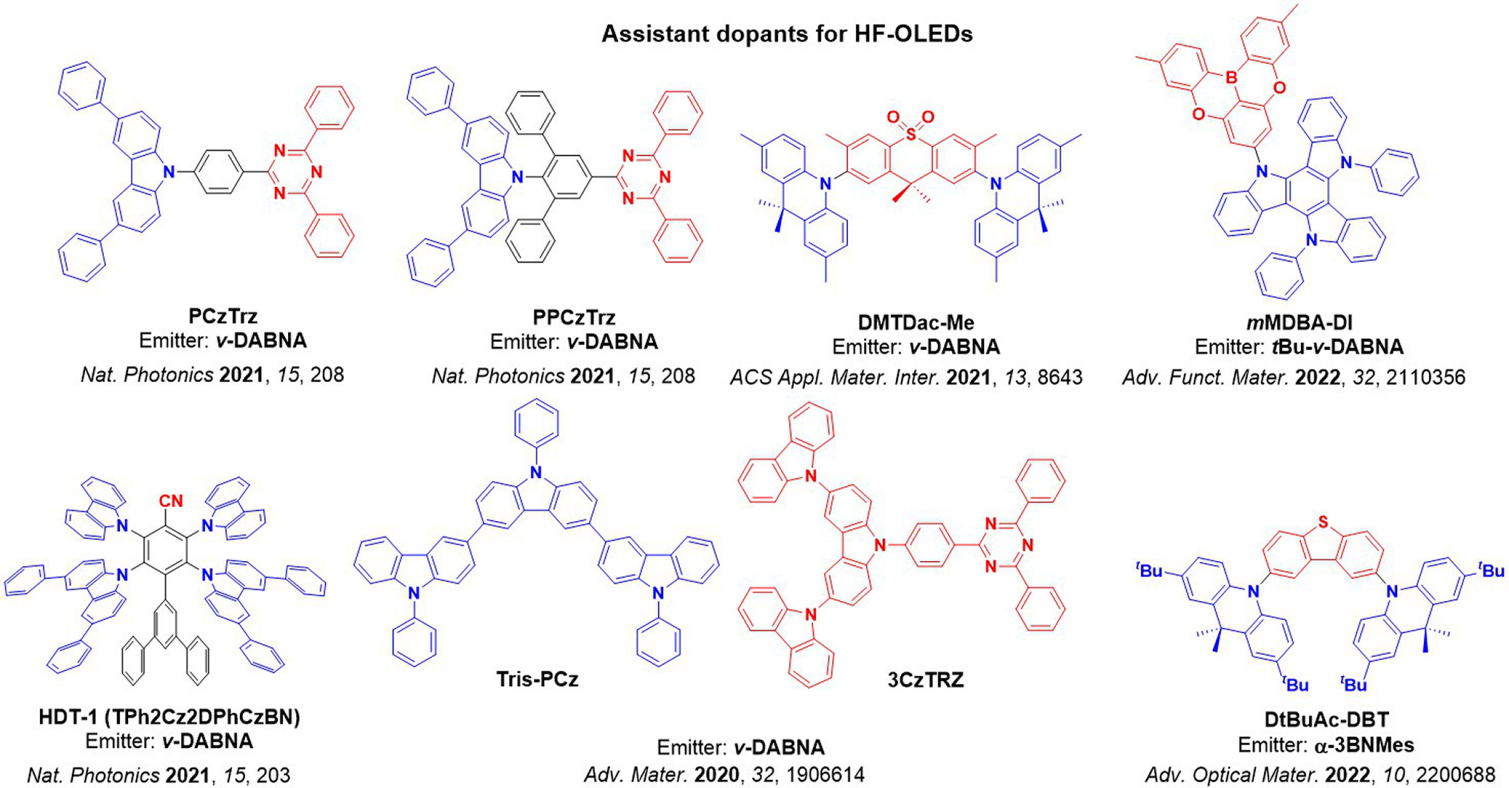
Structures of HF-OLED assistant dopants used alongside emitters in Figure 136 (the blue color signifies donor moieties, while the red color signifies acceptor moieties).

The *tert*-butyl decorated ***v*-DABNA** derivative ***t*-Bu-*v*-DABNA** (Figure 136) shows comparable photophysical properties to ***v*-DABNA** in 5 wt% DBFPO films, with Φ_PL_ of 92% and τ_d_ of 2.9 μs translating to a *k*_RISC_ of 2.5 × 10^5^ s^–1^.^306^ In toluene the λ_PL_ is 467 nm and the Δ*E*_ST_ is 0.04 eV. OLEDs using ***t*-Bu-*v*-DABNA** showed EQE_max_ of 36.3% at CIE coordinates of (0.11, 0.15). The same OLED stack using ***v*-DABNA** showed a slightly lower EQE_max_ of 35.2% at the same CIE coordinates. Unfortunately, the efficiency roll-off of the OLED with ***t*-Bu-*v*-DABNA** was large, with the EQE_1000_ of 16.5%. When used in conjunction with the assistant TADF dopant ***m*MDBA-DI** (Figure 137) the EQE_max_ reached 39.1% while the EQE_1000_ remained high at 34.3%.

Efforts by the same group to blue-shift emission towards desired Rec. 2020 coordinates focussed first on the introduction of weakly donating methyl substituents *para* to the boron atoms to destabilize the LUMO in **m-*v*-DABNA** (Figure 136).^1034^ A second strategy saw the incorporation of fluorine atoms *ortho* to the nitrogen atoms, which stabilized the HOMO in **4F-*v*-DABNA** (Figure 136). The authors also designed a third emitter that combined both modifications in **4F-m-*v*-DABNA** (Figure 136). Compared to ***v*-DABNA**, all three compounds emit slightly bluer, with λ_PL_ in toluene of 464, 457, and 455 nm for **m-*v*-DABNA**, **4F-*v*-DABNA**, and **4F-m-*v*-DABNA**, respectively, compared to 468 nm for ***v*-DABNA**. **m-*v*-DABNA**, **4F-*v*-DABNA**, and **4F-m-*v*-DABNA** showed comparable Φ_PL_ of 91, 90, and 89% and τ_d_ of 3.1, 3.1, and 3.2 μs, respectively, as 3 wt% doped films in DBFPO. The Δ*E*_ST_ values of 0.05–0.07 eV in toluene are similar to that for ***v*-DABNA** (Δ*E*_ST_ of 0.02 eV in 1 wt% DOBOA-OAr),^1031^ leading to comparably fast *k*_RISC_ ranging between 2.1–2.3 × 10^5^ s^–1^ in 3 wt% doped films in DBFPO^1034^ (*k*_RISC_ in ***v*-DABNA** was 2.0 × 10^5^ s^–1^ in 1 wt% DOBNA-OAr).^1031^ The OLEDs showed EQE_max_ of 36.2, 35.8, and 33.7% at CIE coordinates of (0.12, 0.12), (0.13, 0.08), and (0.13, 0.06) for **m-*v*-DABNA**, **4F-*v*-DABNA**, and **4F-m-*v*-DABNA**, respectively.^1034^ Despite the impressive *k*_RISC_ the efficiency roll-off was still large across all three of these emitters.

A strategy to access different color spaces by red-shifting the emission of ***v*-DABNA** involved decoration with electron-withdrawing cyano groups to generate ***v*-DABNA-CN-Me** (Figure 136).^171^ This high-performance green emitter has λ_PL_ of 498 nm, Φ_PL_ of 86%, small Δ*E*_ST_ of 0.01 eV, and τ_d_ of 10 μs in toluene. Like ***v*-DABNA**, *k*_RISC_ is fast at 1.0 × 10^5^ s^–1^. Green devices showed CIE coordinates of (0.13, 0.65) and an EQE_max_ of 31.6% with EQE_1000_ of 28.6%.

A V-shaped extended design was reported by Oda *et al*.^170^ where three boron atoms and six nitrogen atoms were incorporated within the nanographene core to create the helical structure ***v*-DABNA-Mes** (Figure 136), which is essentially three **DABNA-1** units fused together. This compound has λ_PL_ of 484 nm, Φ_PL_ of 80%, τ_d_ of 2.4 μs, and Δ*E*_ST_ of 0.009 eV as a 1 wt% doped PMMA film, which is a red-shifted emission compared to ***v*-DABNA** (λ_PL_ of 467 nm and Δ*E*_ST_ of 0.02 eV for in 1 wt% DOBNA-OAr).^1031^ Owing to the smaller Δ*E*_ST_ and Δ*E*_T2‑T1_ (calculated for ***v*-DABNA-Mes** to be 0.10 eV compared to 0.14 eV in ***v*-DABNA**), an improved *k*_RISC_ of 4.4 × 10^5^ s^–1^ was reported compared to 2.0 × 10^5^ s^–1^ in ***v*-DABNA**. Solution-processed devices were reported, likely due to the high molecular weight of the emitter at 1774.7 g mol^–1^ preventing vacuum deposition and showed EQE_max_ of 22.9% and EQE_100_ of 20.3% at CIE coordinates of (0.09, 0.21).

An alternative strategy to blue-shift the emission of ***v*-DABNA** was presented by Tanaka *et al*. using ***v*-DABNA-O-Me** (Figure 136),^173^ in which reduction in HOMO delocalisation was realized when one of the nitrogen donor atoms was replaced with an oxygen atom. This compound has a slightly blue-shifted λ_PL_ of 464 nm compared to 467 nm in ***v*-DABNA** and a similar Δ*E*_ST_ value of 0.03 eV in a 1 wt% doped film in PMMA (0.02 eV for ***v*-DABNA** in 1 wt% DOBNA-OAr). The devices showed a similar EQE_max_ of 29.5% while the efficiency roll-off was improved (EQE_1000_ of 26.9%); the CIE coordinates of the device with ***v*-DABNA-O-Me** were (0.13, 0.10), which are close to those of the device with ***v*-DABNA** (0.12, 0.11). Importantly, there is a vastly improved device lifetime (LT_50_ of 314 hours at 100 cd m^–2^) compared to the device with ***v*-DABNA** (LT_50_ of 31 hours). The differences in device lifetimes were attributed to a larger calculated SOC between S_1_ and T_2_ in ***v*-DABNA-O-Me** compared to ***v*-DABNA**, producing a more efficient TADF process.

A similar strategy to blue-shift the emission of MR-TADF compounds was reported by Park *et al*.^1035^ who replaced nitrogen atoms with oxygen or sulfur. Three emitters containing either two oxygen atoms (**BOBO-Z**), one oxygen and one sulfur atom (**BOBS-Z**), and two sulfur atoms (**BSBS-Z**) showed progressively red-shifted emission with λ_PL_ of 445, 457, and 464 nm, respectively as 3 wt% doped mCBP films (Figure 136). Each of these compounds shows a blue-shifted emission compared to ***v*-DABNA**, which has a λ_PL_ of 474 nm in the same medium. The Φ_PL_ values of the same films varied widely at 64, 93, and 88%, while the Δ*E*_ST_ and τ_d_ values in toluene were all similar at 0.15, 0.16, and 0.14 eV, and 7.7, 7.6, and 6.7 μs, all respectively. Due to the heavy atom effect *k*_RISC_ was enhanced with more sulfur atoms, at 0.7, 8.6, and 16 × 10^5^ s^–1^ for **BOBO-Z**, **BOBS-Z**, and **BSBS-Z**, respectively, in the 3 wt% doped films in mCBP. The devices with **BOBO-Z**, **BOBS-Z**, and **BSBS-Z** showed EQE_max_ of 13.6, 26.9, and 26.8%, respectively, at CIE coordinates of (0.15, 0.04), (0.14, 0.06), and (0.13, 0.08). The latter two devices outperformed the OLED with ***v*-DABNA** using the same stack [EQE_max_ of 24.6% at CIE coordinates of (0.12, 0.12)]. The efficiency roll-off was also modest, with EQE_100_ of 9.8, 24.0, and 24.0%, respectively.

Recently, our group introduced a linear boron and nitrogen-containing MR-TADF heptacene system, **α-3BNOH** (Figure 136), that emits at a λ_PL_ of 398 nm in THF and has a FWHM of 31 nm.^164^ Although this compound had a large measured Δ*E*_ST_ of 0.31 eV, a small TADF contribution was nonetheless observed with a τ_d_ of 450 ns in THF. Interestingly the activation energy for T_1_ to S_1_ conversion was much lower at 0.07 eV, with RISC here believed to involve intermediate triplet states as corroborated by calculations. At room temperature the triplet harvesting pathways are a combination of TADF and TTA. Devices were reported subsequently using an EML consisting of 10 wt% of **α-3BNOH** doped in DPEPO.^1036^ Compared to emission in THF, λ_EL_ was red-shifted and broadened (λ_EL_ at 410 nm and FWHM of 47 nm). The EQE_max_ was less than 1%, attributed to the formation of aggregates, which is consistent with the broadened and red-shifted EL spectrum. Replacement of the OH substituents with mesityl groups resulted in a red-shift of the emission in **α-3BN­Mes** (Figure 136).^1037^ In THF the λ_PL_ shifted from 398 nm for **α-3BNOH** to 442 nm for **α-3BN­Mes**. In 1 wt% doped PMMA films the Δ*E*_ST_ of **α-3BN­Mes** was identical to that of **α-3BNOH**, at 0.28 eV for each. The photophysics is complex, reflected in the presence of two lifetimes in the delayed emission (τ_d_ of 9.1 μs and 7.1 ms), the shorter one associated with a mixture of aggregate and monomer emission and the longer one linked to pure monomer emission. RISC is thus inefficient, with *k*_RISC_ of only 5.9 × 10^2^ s^–1^, and the OLED performance was poor with an EQE_max_ of 1.7%. However, when used as the terminal emitter in conjunction with **Dt­Bu­Ac-DBT** (Figure 137) as the assistant dopant in a HF-OLED, the EQE_max_ improved to 15%, with CIE coordinates of (0.15, 0.10).

### 11.3 Central Boron MR-TADF Compounds with a Carbazole Scaffold

A separate design strategy has emerged in parallel with those described above, replacing the DPA groups embedded within **DABNA-1** with other N-heterocycles. The first such derivative, **Dt­Bu­CzB** (Figure 138),^1038^ contained fused *tert*-butylcarbazole and displayed sky blue emission with λ_PL_ of 493 nm and Φ_PL_ of 88% in 1 wt% doped mCBP films, with Δ*E*_ST_ of 0.13 eV and τ_d_ of 69 μs. Compared to **DABNA-1**, the emission is red-shifted and the Δ*E*_ST_ is smaller (λ_PL_ = 460 nm and Δ*E*_ST_ = 0.18 eV for **DABNA-1** in 1 wt% doped mCBP films)^118^ owing to increased conjugation afforded by the fused structure.^1039^ The OLEDs showed an EQE_max_ of 21.6% at CIE coordinates of (0.10, 0.42). The same material was also reported as **BB­Cz-SB**,^1040^ wherein a slightly improved device performance was reported with EQE_max_ of 27.8%. Xu *et al*. presented solution processed HF-OLEDs, with an EQE_max_ of 16.3% at λ_EL_ of 490 nm reported for **Dt­Bu­CzB**, which was renamed **BCzBN** here when used alongside the assistant dopant **CzAcSF** (Figure 138).^1039^

**Figure 138:**
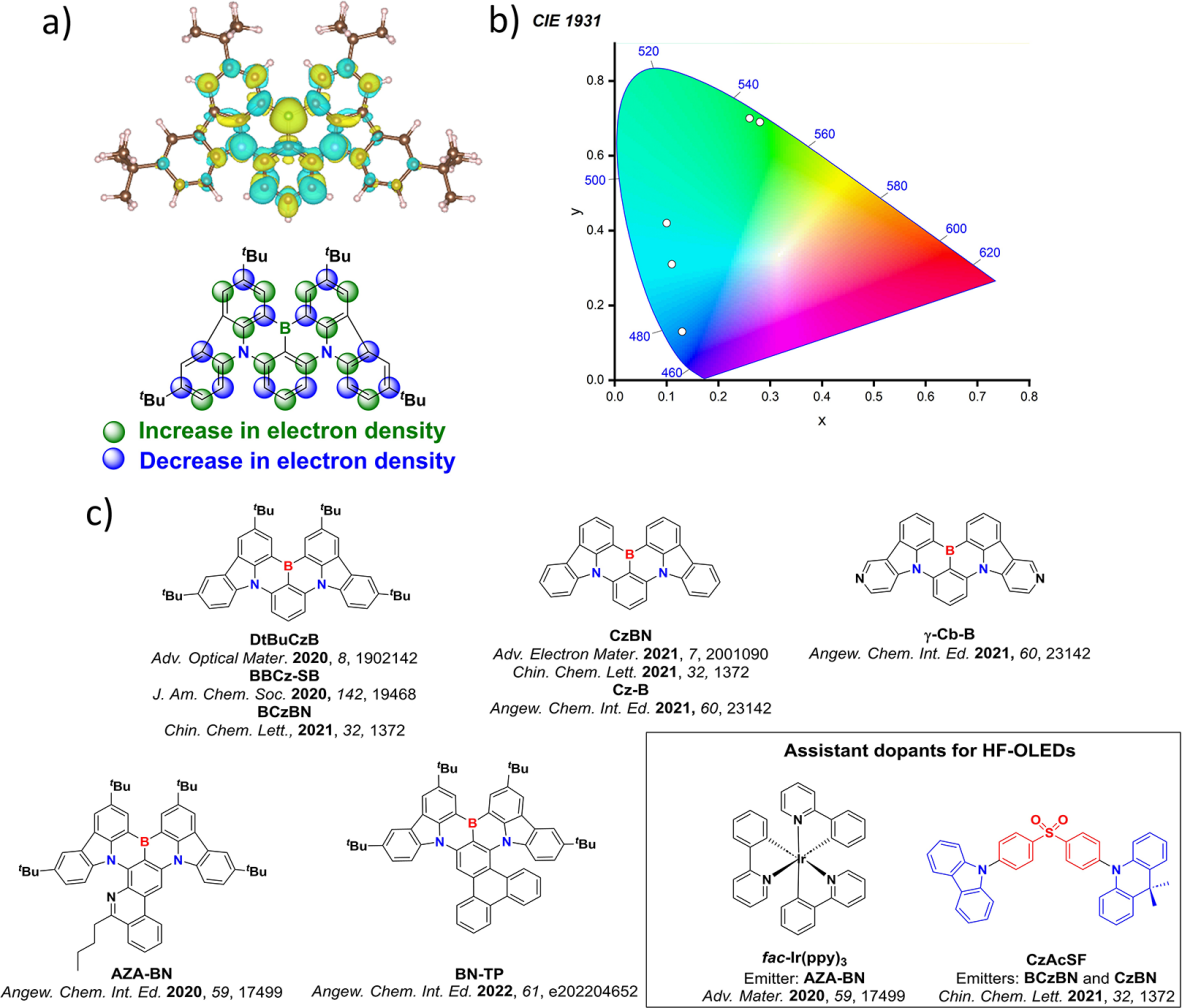
a) Computed difference density plot and the schematic representation of the difference density distribution of **Dt­Bu­CzB**, b) CIE color coordinates OLEDs with **CzBN** derivatives, and c) structures of unsubstituted **CzBN** emitters and HF-OLED assistant dopants. Difference density plots calculated at the SCS-CC2/cc-pVDZ level in the gas phase; is-value = 0.01. The white circles of the CIE diagram illustrate the spread of the emission color of the device. In the chemical structures, the blue color signifies donor moieties/atoms/functional groups, while the red color signifies acceptor moieties/atoms/functional groups.

Developing from this fused-carbazole core, an analogue without *tert*-butyl substituents has been reported by three groups, named **CzBN** ^1039,1041^ and **Cz-B** ^1042^ (Figure 138). **Cz-B** was presented alongside a carboline analogue, **γ-Cb-B** (Figure 138).^1042^ The emission of **γ-Cb-B** is blue-shifted at λ_PL_ of 461 nm compared to λ_PL_ of 484 nm in **Cz-B** in 1 wt% doped films in mCBP and oCBP, respectively, a result of weaker electron-donating character for the carboline. The Φ_PL_ and τ_d_ of **Cz-B** and **γ-Cb-B** are 97 and 89% and 32 and 44 μs, respectively, while both compounds show similar Δ*E*_ST_ values of 0.14 and 0.12 eV in toluene. The devices with **Cz-B** and **γ-Cb-B** showed EQE_max_ of 22.1 and 19.0% at CIE coordinates of (0.11, 0.31) and (0.13, 0.13), respectively. Both OLEDs showed large efficiency roll-off though, with EQE_1000_ of 6.9 and 7.7% attributed to their long-delayed lifetimes. HF solution processed OLEDs were presented with **CzBN** emitter and **CzAcSF** assistant dopant by Xu *et al.*, with a EQE_max_ of 14.7% presented at a 2 wt% doping of the emitter, with λ_EL_ of 480 nm.^1039^

A fused derivative containing an aza­phen­an­threne-type structure, **AZA-BN** (Figure 138), was reported by Zhang *et al*.^1043^ The increased conjugation produced a red-shifted emission in toluene (λ_PL_ of 522 nm) compared to **DtBuCzB** (λ_PL_ = 481 nm).^1038^ The Δ*E*_ST_ in toluene is 0.18 eV while the Φ_PL_ is essentially unity, reported as 99.7%. In the 4 wt% doped mCBP films the Φ_PL_ is 94% and there is a long τ_d_ of 160 μs. The OLEDs showed EQE_max_ of 25.7% at CIE coordinates of (0.28, 0.69), while the EQE_1000_ dropped to 9%. This is unsurprising owing to the long-delayed lifetime and was addressed in HF-OLEDs using ***fac***-**Ir(ppy)_3_** (Figure 138) as the phos­phor­escent assistant dopant, which then achieved EQE_max_ of 28.2% and a higher EQE_1000_ at 19.1%.

Triphenylene derivative **BN-TP** (Figure 138) was reported by Xu *et al*.^1044^ and prepared *via* a Scholl oxidative ring closing reaction. Compared to the parent **Dt­Bu­CzB** (λ_PL_ = 481 nm in toluene)^1038^ there was a significant red-shift to 523 nm for **BN-TP** due to the increased π-conjugation in the backbone.^1044^ In 3 wt% doped PhCzBCz films **BN-TP** has a Φ_PL_ of 96% and a *k*_RISC_ of 2.1 × 10^4^ s^–1^. The device showed an impressive EQE_max_ of 35.1% at CIE coordinates of (0.26, 0.70), which was attributed to strong horizontally aligned TDM enhancing the light outcoupling. The EQE_100_ was maintained at 32.4%, while a promising LT_50_ of 28.8 hours was reported at 4,000 cd m^–2^.

#### 11.3.1 Substituted with Peripheral Acceptor Units

Substituted analogs of **Dt­Bu­CzB** have proven to be a popular design strategy (Figure 139 and Figure 143) especially for color tuning of narrowband MR-TADF emission. Zhang *et al*.^1045^ demonstrated the first examples of green MR-TADF emitters **2F**-**BN**, **3F-BN**, and **4F-BN** with λ_PL_ of 501, 498, and 493 nm (Figure 139), respectively. The electron-withdrawing fluorophenyl groups act to stabilize the LUMO compared to the parent, reducing the emission energy from blue to green. The Δ*E*_ST_ values of 0.16, 0.08, and 0.11 eV remained similar to **Dt­Bu­CzB** while the τ_d_ and Φ_PL_ ranged between 16.7–25.6 μs, and 83–91%, respectively. Green HF-OLED devices using **2F**-**BN**, **3F-BN**, and **4F-BN** with **5TCzBN** (Figure 139) as the assistant dopant showed EQE_max_ of 22.0, 22.7, and 20.9% and efficiency roll-off of between 7–32% at 1000 cd m^–2^ at CIE coordinates (0.16, 0.60), (0.20, 0.58) and (0.12, 0.48), respectively. In a similar vein, direct substitution of a cyano group *para* to boron produced the emitter **CN-BCz-BN** (Figure 139).^486^ This compound has a modestly red-shifted emission in toluene (λ_PL_ = 496 nm) compared to **Dt­Bu­CzB** (λ_PL_ = 481 nm). No further studies were undertaken on this material.

**Figure 139:**
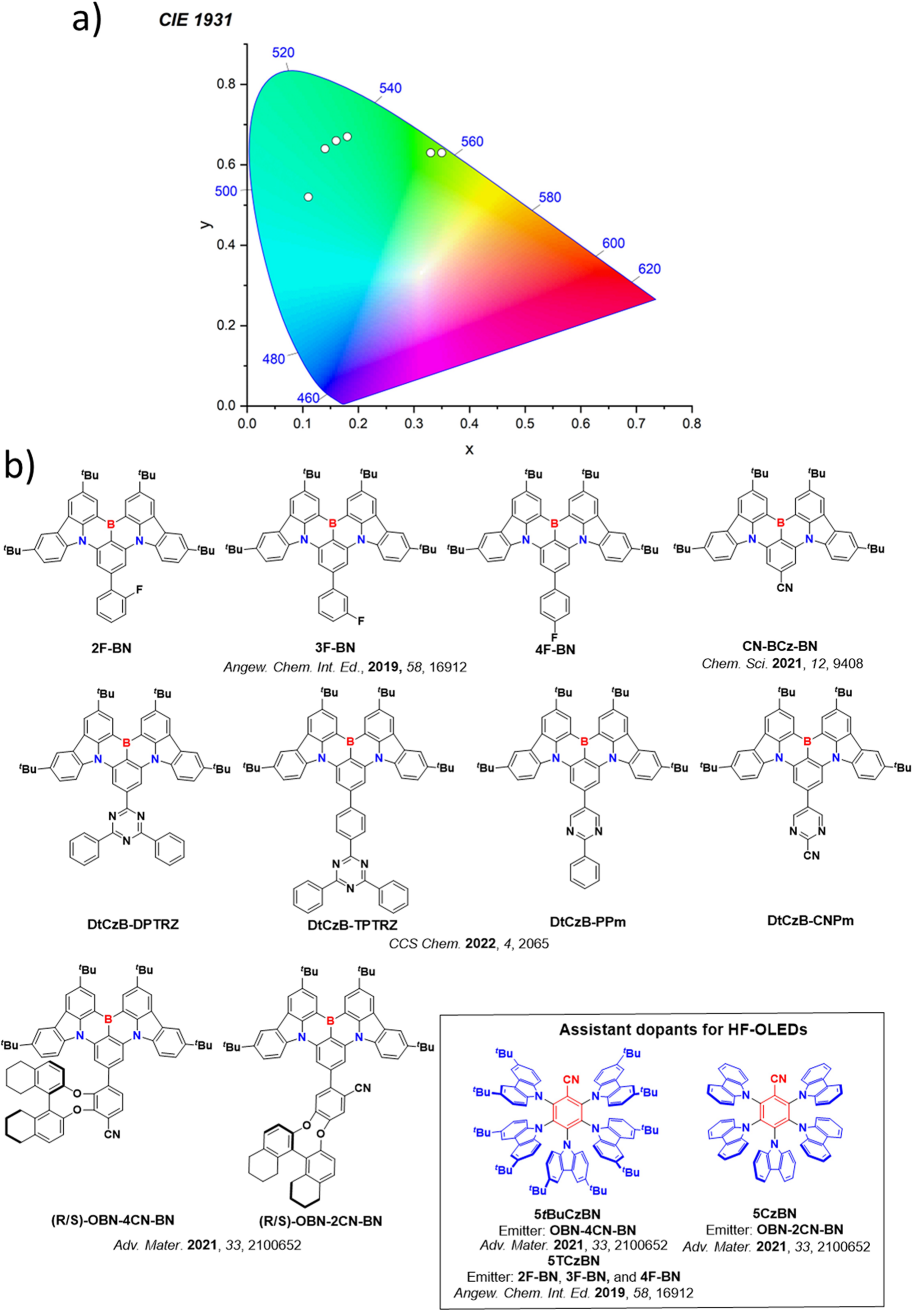
a) CIE color coordinates of OLEDs with CzBN derivatives containing acceptor moieties and b) structures of acceptor substituted CzBN emitters and HF-OLED assistant dopants. The white circles of the CIE diagram illustrate the spread of the emission color of the device. In the chemical structures, the blue color signifies donor moieties/atoms/functional groups, while the red color signifies acceptor moieties/atoms/functional groups).

Substitution with strong acceptors *para* to boron was also the subject of a study by Xu *et al*.^1046^ The acceptors included triazine, phen­yl­tri­azine, phen­yl­pyri­midine, and cyano­pyri­mi­dine, producing the emitters **DtCzB-DPTRZ**, **DtCzB-TPTRZ**, **DtCzB-PPm**, and **DtCzB-CNPm**, respectively (Figure 139). The red-shifted emission maxima in toluene were 521, 501, 499, and 515 nm, respectively. The Δ*E*_ST_ for these analogues measured in toluene ranged between 0.08–0.17 eV,^1046^ and the Φ_PL_ remained high at between 87–95% in 3 wt% doped PhCzBCz films. Devices with **DtCzB-DPTRZ**, **DtCzB-TPTRZ**, **DtCzB-PPm**, and **DtCzB-CNPm** showed EQE_max_ of 24.6, 29.8, 28.6, and 25.0%, at CIE coordinates of (0.33, 0.63), (0.18, 0.67), (0.16, 0.66), and (0.35, 0.63), respectively. Contrasting device performances were noted, with extremely high efficiency roll-off of 70% and 42% at 100 cd m^–2^ for the devices with **DtCzB-DPTRZ** and **DtCzB-CNPm**, compared to 11% and 15% for the devices with **DtCzB-TPTRZ** and **DtCzB-PPM**. This difference was attributed to faster *k*_RISC_ for the latter two emitters (ca. 1 × 10^4^ s^–1^ compared to ca. 1 × 10^3^ s^–1^ for the former). The materials with more efficient *k*_RISC_ had slightly smaller Δ*E*_ST_ of 0.11 and 0.08 eV, compared to 0.17 and 0.12 eV.

Two chiral MR-TADF compounds **(*R/S*)-OBN-2CN-BN** and **(*R/S*)-OBN-4CN-BN** containing phenylcyano substituents showed narrowband CPL (Figure 139).^658^ The phen­yl­cyano substitution red-shifted the emission compared to **DtBu­CzB**, with λ_PL_ of 498 and 510 nm for **(*R/S*)-OBN-2CN-BN** and **(*R/S*)-OBN-4CN-BN**, respectively in 3 wt% doped PhCzBCz films. The Δ*E*_ST_ of 0.12 and 0.15 eV are for the *R*-isomers in toluene while the τ_d_ are 95 and 97 μs in 3 wt% doped films in PhCzBCz; the *S*-isomers show similar photo­phys­ical behavior.^658^ Devices of R and S isomers of **OBN-2CN-BN** and **OBN-4CN-BN** were fabricated, with similar properties between R and S isomers. The CIE coordinates of **OBN-2CN-BN** and **OBN-4CN-BN** were (0.11, 0.52) and (0.14, 0.64), respectively, for both the R and S isomers. The device EQE_max_ was 29.4 and 28.8% for **OBN-2CN-BN** R and S isomers, respectively, with a modest decrease for the device with **OBN-4CN-BN** at 24.5 and 24.3% for R and S isomers, respectively. Each showcased large efficiency roll-off with EQE_100_ of 19.8% (19.2%) and 8.0% (7.9%) for **OBN-2CN-BN** and **OBN-4CN-BN**, respectively, for their R and (S) isomers. HF OLEDs of **(*R*)-OBN-2CN-BN** and (***R*)-OBN-4CN-BN** were fabricated using **5CzBN** and **5*t*BuCzBN** (Figure 139) as assistant dopants, respectively, with similar EQE_max_ at 29.8 and 24.7% compared to the 29.4 and 24.5% previously reported. However, the efficiency roll-off dramatically improved, with EQE_100_ of 27.2 and 23.5%. Their chiral optical properties are discussed in more detail in Section 7.

#### 11.3.2 Substituted with Peripheral Donor Units

Alongside acceptor substitution of MR-TADF emitters, there are now several reported examples of adding electron-donating substituents to **DtBu­CzB** to modulate or enhance its properties. A *tert*-butylcarbazole unit coupled *meta* to the boron in ***m*-Cz-BN­Cz** (Figure 140)^210^ produced a red-shifted emission with λ_PL_ of 519 nm in toluene and λ_PL_ of 528 nm in 10 wt% doped PhCz­BCz films. The stabilization of the S_1_ state is the result of a destabilized HOMO arising from the electron-donating *tert*-but­yl­car­ba­zole. The Δ*E*_ST_ is 0.08 eV in toluene, producing a remarkably rapid delayed emission with τ_d_ of 0.86 μs in 10 wt% doped PhCzBCz films and efficient *k*_RISC_ of 1.0 × 10^6^ s^–1^.^210^ However, the emission spectrum is broadened by the inclusion of the donating unit, reflected in a larger FWHM of 38 nm compared to 22 nm for **Dt­Bu­CzB** in toluene and likely arising from a hybrid SRCT/LRCT character_._ ^1038^ Efficient devices showed an EQE_max_ of 31.4% at CIE coordinates of (0.26, 0.68), and there was only a modest efficiency roll-off with EQE_100_ of 29%.^210^

**Figure 140:**
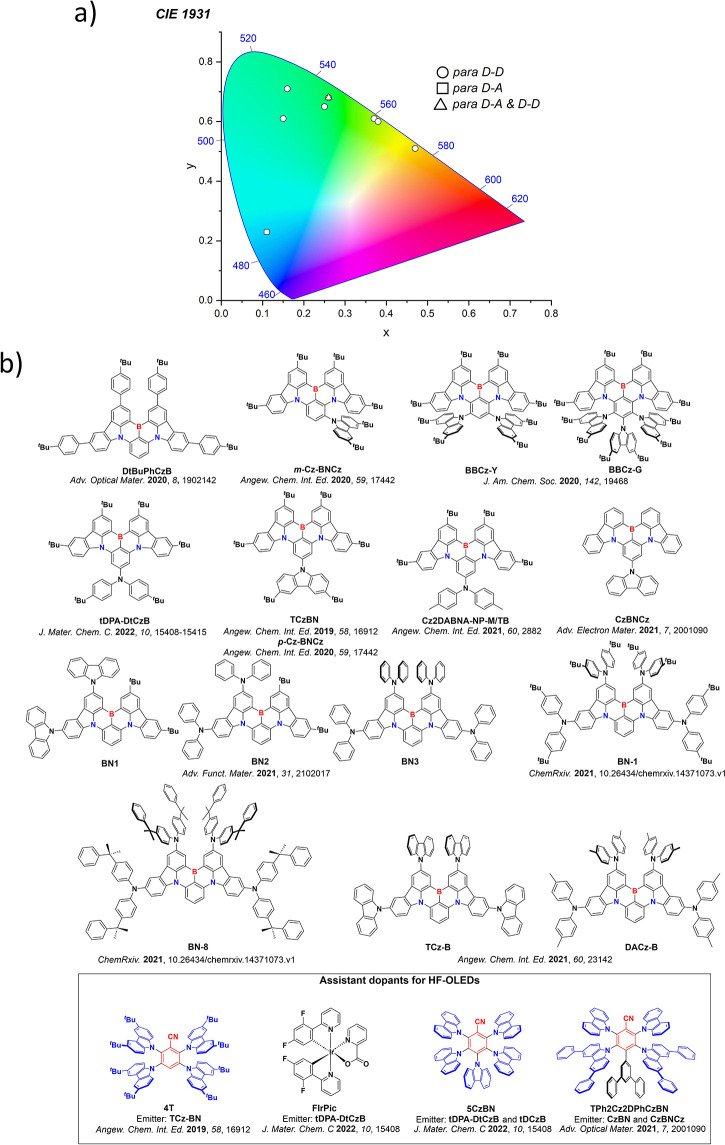
a) CIE color coordinates of OLEDs with CzBN derivatives containing donor moieties and b) structures of donor substituted CzBN emitters and HF-OLED assistant dopants. The white circles of the CIE diagram illustrate the spread of the emission color of the device for para disposed D-D, the white squares of the CIE diagram illustrate the spread of the emission color of the device for para disposed D-A, and the white triangles of the CIE diagram illustrate the spread of the emission color of the device for para disposed D-D and para disposed D-A. In the chemical structures, the blue color signifies donor moieties/atoms/functional groups, while the red color signifies acceptor moieties/atoms/functional groups.

The corresponding *para*-substituted derivative **TCz-BN** ^1045^ (or ***p*-Cz-BNCz**, Figure 140)^210^ has been investigated computationally^209^ and used in HF-OLEDs;^1045^ however, there is little documentation of its intrinsic photophysical properties. A blue-shifted emission in toluene (λ_PL_ of 477 nm) is apparent compared to ***m*-Cz-BNCz** (λ_PL_ of 519 nm).^210^ This difference is attributed to the fact that *meta* substitution leads to a destabilized HOMO more so than *para* substitution.^209^ HF-OLEDs with **TCz-BN** as the terminal emitter and **4T** (Figure 140) as the assistant dopant showed EQE_max_ of 18.9% at CIE coordinates of (0.13, 0.20).^1045^

Based on these initial reports, two derivatives with additional carbazoles were designed (**BBCz-Y** and **BBCz-G**, Figure 140).^1040^ In the former the two *meta* positions are occupied, while in the latter both *meta* and the *para* positions are occupied. Compared to ***m*-Cz-BNCz** (λ_PL_ = 519 nm), the emission of **BBCz-Y** is red-shifted in toluene to 549 nm, while the λ_PL_ of **BBCz-G** surprisingly remains at 517 nm. For **BBCz-Y**, the second donating *meta*-substituted ^*t*^BuCz further destabilizes the HOMO leading to further red-shift of the emission. For **BBCz-G** the same destabilizing interaction of the HOMO is achieved concomitantly with a destabilized LUMO resulting from the *para*-substituted ^*t*^BuCz, producing minimal net effect compared to ***m*-Cz-BNCz**. Both compounds have identical Δ*E*_ST_ of 0.14 eV, while the τ_d_ are similar at 13 and 11 μs in 2 wt% doped mCBP films. OLEDs with **BBCz-Y** and **BBCz-G** showed EQE_max_ of 29.3 and 31.8% at CIE coordinates of (0.37, 0.61) and (0.26, 0.68), respectively, and the EQE_100_ remained high at 25.8 and 29.5%.

Coupling of a Me-diphenylamine group *para* to boron in **Cz2­DABNA-NP-M/TB** (Figure 140)^1021^ resulted in a destabilized LUMO and a slight blue-shift of emission (λ_PL_ at 478 nm in 1 wt% doped PMMA films) compared to **DtBu­CzB**,^1038^ similar to that observed for **TCz-BN** (*vide supra*).^210^ The similarity of the Δ*E*_ST_ and τ_d_ (0.15 eV and 19 μs, respectively) to those of previously reported donor-substituted MR-TADF emitters suggest that the MR-TADF mechanism is largely unaffected by the nature or the regiochemistry of the donor in these materials; instead, the main impact is restricted to the emission color.^1021^ The blue devices using **Cz2­DABNA-NP-M/TB** showed EQE_max_ of 21.8% at CIE coordinates of (0.11, 0.23) while the EQE decreases by only 6% at 100 cd m^–2^.

A related *tert*-butyldiphenylamine derivative **tDPA-DtCzB** (Figure 140) was reported by Yan *et al*.^1047^ and direct comparison were made with the parent **DtBuCzB**, (named **DtCzB** in this study). In toluene the emission was blue-shifted from 481 to 470 nm for **DtCzB** and **tDPA-DtCzB**, respectively, along with a modest reduction in Δ*E*_ST_ from 0.13 to 0.11 eV. In 1 wt% doped PhCzBCz films the two emitters show comparable Φ_PL_ of 89 and 85%, while *k*_RISC_ increased from 0.74 × 10^4^ s^–1^ for **DtCzB** to 2.45 × 10^4^ s^–1^ for **tDPA-DtCzB**. The OLEDs with **DtCzB** and **tDPA-DtCzB** showed EQE_max_ of 23.2 and 25.0%, respectively, but efficiency roll-off was severe with EQE_100_ of 13.0 and 16.4%. HF-OLEDs using either **5CzBN** or **Firpic** (Figure 140) as the assistant dopant were also fabricated, and the highest performing HF-OLED showed an EQE_max_ of 31.0% with the latter.

The emitter **CzBNCz** similarly contains a carbazole attached *para* to the **CzBN** core (Figure 140).^1041^ Similar to previously discussed donor substitutions, addition of Cz *para* to the boron produced a modest blue-shift with λ_PL_ shifting from 485 nm for **CzBN** to 470 nm for **CzBNCz** in 1 wt% doped films in mCBP. The Φ_PL_ remains very high at 99% for **CzBN** and 95% for **CzBNCz** in the same films, while the Δ*E*_ST_ increase from 0.15 to 0.18 eV (both in toluene). The larger Δ*E*_ST_ resulted in a longer τ_d_, increasing from 75 for **CzBN** to 92 μs for **CzBNCz**. HF-OLEDs with **CzBNCz** and **CzBN** using **TPh2­Cz2­DPh­CzBN** (Figure 140) as the assistant dopant showed EQE_max_ of 21.9 and 20.6%, respectively, at CIE coordinates of (0.16, 0.31) and (0.14, 0.31). The efficiency roll-off was modest with EQE_100_ of 21.0 and 19.4%, respectively, although the devices showed low stability with LT_90_ at 1000 cd m^–2^ of 39 hours and 29 hours for **CzBNCz** and **CzBN**, respectively.

Incorporation of mildly donating ^*t*^Bu-Phenyl groups onto **DtBu­CzB** produced **DtBu­Ph­CzB** (Figure 140),^1038^ which shows a red-shifted emission of λ_PL_ 508 nm in 1 wt% doped mCBP films (compared to 493 nm for **Dt­Bu­CzB** in the same). Despite the change in emission color, this substitution had a minimal effect on the TADF characteristics with Δ*E*_ST_ of 0.10 eV and τ_d_ of 61 μs (compared to 0.13 eV and 69 μs reported for **Dt­Bu­CzB**). Green devices showed an EQE_max_ 23.4% at CIE coordinates of (0.15, 0.61). The EQE_max_ could be enhanced to 26.5% with the use of exciplex host TCTA:​PIM-TRZ (at 3 wt% emitter doping), but the emission color red-shifted to CIE coordinates of (0.25, 0.65). The improvements in device performance were attributed to two factors: firstly, the exciplex host is itself TADF (see Section 8), which allowed for triplet harvesting on the host as well as the emitter; secondly, the exciplex host displayed improved charge balance within the emissive layer compared to simpler mCBP devices.

Yang and co-workers reported a series of MR-TADF emitters where sequentially stronger donors were coupled *para* to the nitrogen atom.^1048^ Compounds **BN1**, **BN2**, and **BN3** contained either two carbazoles, two diphenylamines, or four diphenylamine donor groups (Figure 140). Changing the nature and number of donors had a significant impact on color, with λ_PL_ red-shifting from 499 to 538 and 563 nm in 1 wt% doped mCBP films for **BN1**, **BN2**, and **BN3**, respectively. There were only minor changes to the other photophysical properties, with a modest broadening of the emission spectrum (FWHM increasing from 38 to 44 nm from **BN1** to **BN3**) along with a slight decrease in the Φ_PL_ from 93 to 86%, while the Δ*E*_ST_ (0.09–0.13 eV) and the *k*_RISC_ (1.9–1.4 × 10^5^ s^–1^) were largely unaffected, all respectively. Devices with **BN1**, **BN2**, and **BN3** showed EQE_max_ of 17.0, 20.7, and 21.4%, although the efficiency roll-off was severe with EQE_1000_ of 8.5 and 3.3% for the devices with **BN1** and **BN2** (luminance of 1,000 cd m^–2^ was not reached for **BN3**). When mCBP:​PO-T2T exciplex host was employed, the EQE_max_ increased to 24.3, 24.5, and 24.7% for the devices with **BN1**, **BN2**, and **BN3**, at CIE coordinates of (0.15, 0.63), (0.38, 0.61), and (0.47, 0.52). Efficiency roll-off also improved, with EQE_1000_ of 18.4, 15.8, and 17.6%, respectively.

Two derivatives of **BN3** containing bulkier substituents were recently reported, **BN-1** and **BN-8** (Figure 140).^1049^ In toluene these two emitters showed similar photophysical properties, with λ_PL_ of 566 and 568 nm for **BN-1** and **BN-8** respectively, and Φ_PL_ of 95% for both. However, the Δ*E*_ST_ values were distinct at 0.11 and 0.03 eV for **BN-1** and **BN-8**, respectively. No further investigations were undertaken for these emitters.

A similar emitter design was reported by Yang *et al*. who attached carbazole (**TCz-B**, Figure 140) and tetra­meth­yl­di­phen­yl­amine donors (**DACz-B**, Figure 140) on to the core emitter **Cz-B**.^1042^ The presence of the donor groups led to a red-shifting of the emission, with λ_PL_ of 484, 517, and 576 nm for **Cz-B**, **TCz-B**, and **DACz-B**, respectively in 1 wt% doped mCBP films. Similar Δ*E*_ST_ of 0.14, 0.09, and 0.14 eV in toluene were obtained, while donor substitution produced a modest decrease in Φ_PL_ from 97 to 89 and 87% in 1 wt% doped mCBP films. There was also an increase in τ_d_ with donor substitution, from 44 to 71 and 118 μs for **Cz-B**, **TCz-B**, and **DACz-B**, respectively. OLEDs with **TCz-B** and **DACz-B** showed EQE_max_ of 29.2 and 19.6% at CIE coordinates of (0.16, 0.71) and (0.47, 0.51). The long τ_d_ translated to a large efficiency roll-off with EQE_1000_ dropping to 9.4 and 4.8%.

#### 11.3.3 Substituted with Peripheral Donor and Acceptor Units

The use of both donor and acceptor substitution can provided further control of emission color. This control was demonstrated by a range of derivatives of **BN-1** and **BN-8** (Figure 140), previously reported by Cai *et al*.^1049^ These two parent emitters were decorated with various acceptor groups *para* to the boron, including pyrimidine and triazine derivatives. The addition of the acceptor units red-shifts the emission, with λ_PL_ shifting from 566 to 586, 598, 612, 627, 618, and 629 nm for new emitters **BN-2**, **BN-3**, **BN-4**, **BN-5**, **BN-6**, and **BN-7**, respectively (Figure 141). The largest red-shifts were observed for the emitters with the strongest triazine electron acceptors. The same trend was captured with materials based on **BN-8**, with λ_PL_ shifting from 568 to 585, 595, 608, and 624 nm for new emitters **BN-9**, **BN-10**, **BN-11**, and **BN-12**, respectively (Figure 141). Addition of these acceptor units also broadened the emission (FWHM ranging between 35–47 nm). The Δ*E*_ST_ values ranged from 0.03–0.12 eV, while the emitters possessed near nearly identical Φ_PL_ of between 94 and 96%. No devices were fabricated.

**Figure 141:**
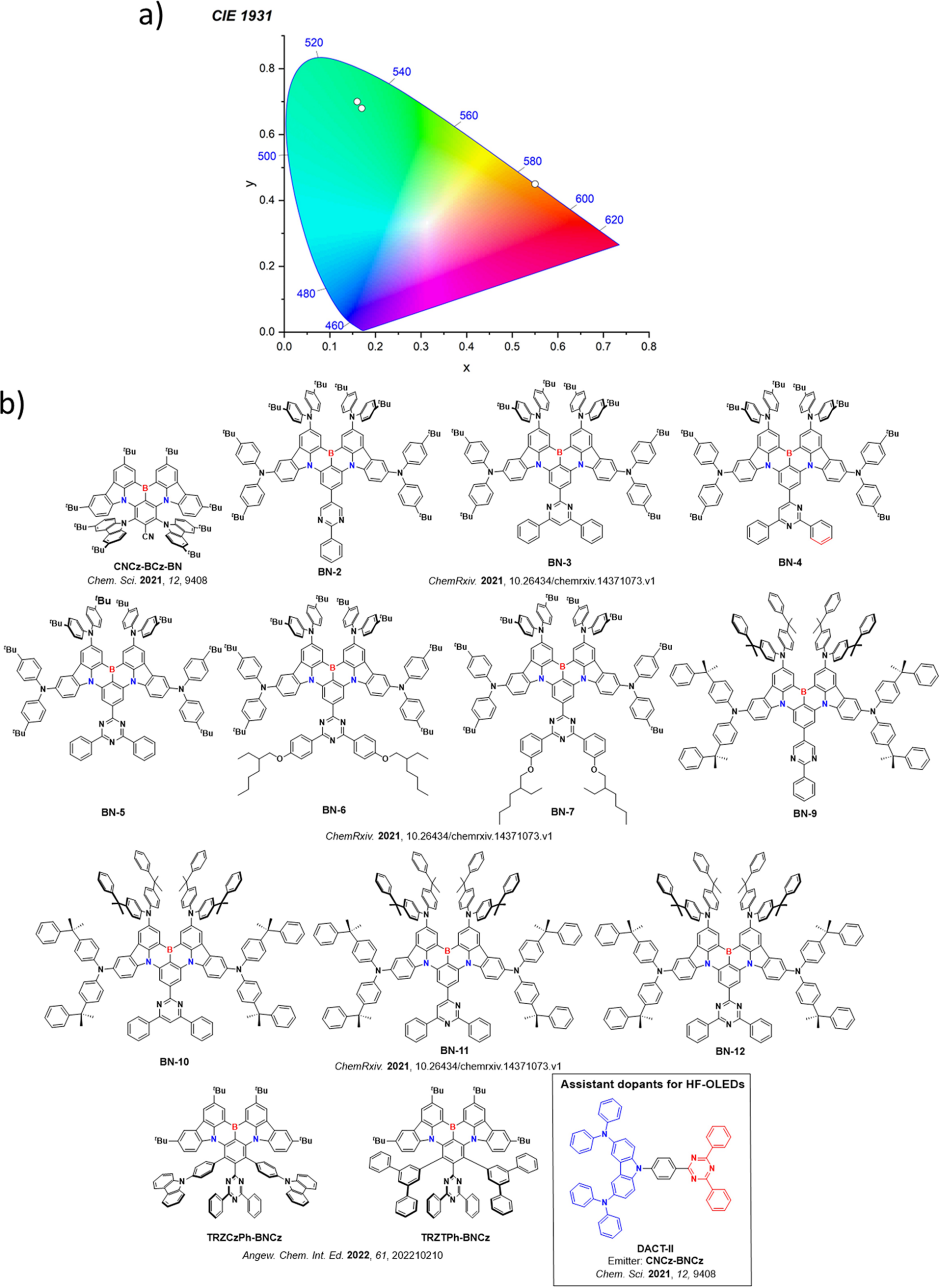
a) CIE color coordinates of OLEDs with CzBN derivatives containing both donor and acceptor moieties and b) structures of donor and acceptor substituted CzBN emitters and HF-OLED assistant dopants. The white circles of the CIE diagram illustrate the spread of the emission color of the device. In the chemical structures, the blue color signifies donor moieties/atoms/functional groups, while the red color signifies acceptor moieties/atoms/functional groups.

**TRZ­Cz­Ph-BNCz** and **TRZ­TPh-BNCz** are further derivatives of **DtBu­CzB** containing donor and acceptor substituents (Figure 141).^1050^ By replacing carbazole for triphenylene in **TRZ­TPh-BNCz** the authors aimed to introduce intermediate triplet states that could help mediate RISC while maintaining a similar steric environment. Narrowband green emission at λ_PL_ of 514 and 513 nm with corresponding FWHM of 34 and 29 nm were observed for **TRZ­CzPh-BNCz** and **TRZ­TPh-BNCz**, respectively. Solvato­chromic studies highlighted that donor and acceptor substitution of these emitters had little impact on the SRCT nature of the excited state. **TRZ­Cz­Ph-BNCz** and **TRZ­TPh-BNCz** have similar Δ*E*_ST_ values of 0.13 and 0.11 eV in toluene, and high Φ_PL_ of 98 and 99% in 3 wt% doped CBP films, respectively. Compared to **DtCzB-DPTRZ** (Figure 139), without carbazole or triphenylene substitutents, calculations revealed T_2_ and T_3_ to be close in energy to S_1_, and in addition T_2_ was calculated to have a significant SOC > 1.4 cm^–1^ in both compounds. This contributed to efficient *k*_RISC_ of 8.8 and 7.5 × 10^5^ s^–1^ for **TRZ­Cz­Ph-BNCz** and **TRZ­TPh-BNCz**, respectively, in 3 wt% doped CBP films. OLEDs with **TRZ­Cz­Ph-BNCz** and **TRZ­TPh-BNCz** showed EQE_max_ of 32.5 and 31.4%, respectively, which are higher than the device with **DTCzB-DPTRZ** (EQE_max_ = 20.2%). The efficient *k*_RISC_ also contributed to low efficiency roll-off, with the EQE_100_ remaining as high as 30.5 and 29.5%, while for the reference emitter the EQE_100_ dropped to 7.8%.

Based on **BBCz-Y** (Figure 140), Liu *et al*.^486^ reported the compound **CNCz-BNCz** that additionally contained a cyano group *para* to the boron centre (Figure 141). The emission was red-shifted from 549 nm in **BBCz-Y** to 581 nm in **CNCz-BNCz**, which was more pronounced than the red-shift from 481 nm in **BBCz-BN** to 496 nm in **CN-BCz-BN**. The addition of the cyano had minimal impact on the FWHM (42 nm for both **BBCz-Y** and **CNCz-BNCz**). **CNCz-BNCz** has a Δ*E*_ST_ of 0.18 eV, a τ_d_ of 60.4 μs, and the Φ_PL_ is 96% in 3 wt% doped CBP films. Devices showed an EQE_max_ of 23.0% at CIE coordinates of (0.55, 0.45), although there was large efficiency roll-off with EQE_100_ dropping to 10.8%. HF-OLEDs using **DACT-II** (Figure 141) as the assistant dopant showed improved EQE_max_ of 33.7% while the EQE_100_ remained high at 27.7%.

#### 11.3.4 Substituted with Peripheral Electronically Inert Substituents

A number of examples exist where there is the introduction of electronically inert substituents that are designed to have minimal impact upon the emission color (Figure 142). In most cases these groups are added to mitigate ACQ (without impacting the SRCT character of the S_1_ state) to prevent broadening of the emission spectrum at higher doping concentrations. Jiang *et al*. reported two such compounds **BN-CP1** and **BN-CP2**, containing phenyl groups *para*-substituted on the MR-TADF core (Figure 142),^1051^ and featuring two carbazole moieties either *ortho* (**BN-CP1**) or *meta* (**BN-CP2**) to the phenyl substituents (Figure 142). The two compounds showed similar photophysical properties in toluene with λ_PL_ of 490 nm for both and Δ*E*_ST_ of 0.12 and 0.13 eV for **BN-CP1** and **BN-CP2**, respectively. In 5 wt% doped DMIC-TRZ films the Φ_PL_ are 98 and 95%, while τ_d_ are 65 and 58 μs for **BN-CP1** and **BN-CP2**, respectively. Even at 30 wt% doping the Φ_PL_ remained at 84 and 61%, highlighting how these substituents can mitigate ACQ while maintaining the narrowband emission (FWHM = 23 nm for each in toluene). Devices at doping concentrations ranging between 1–30 wt% emitter in the EML were prepared for each material, with 5 wt% doping showing the highest efficiencies. The OLEDs with **BN-CP1** and **BN-CP2** showed EQE_max_ of 40.0 and 36.4% at CIE coordinates of (0.09, 0.50) and (0.10, 0.53). The EQE_100_ remained high at 34.0 and 32.6%, respectively. HF-OLEDs with **BN-CP1** were also fabricated using **5TCzBN** (Figure 142) as the assistant dopant. Despite a small drop in the EQE_max_ of 38.1%, the efficiency roll-off lessened with the EQE_100_ at 37.6%.

**Figure 142:**
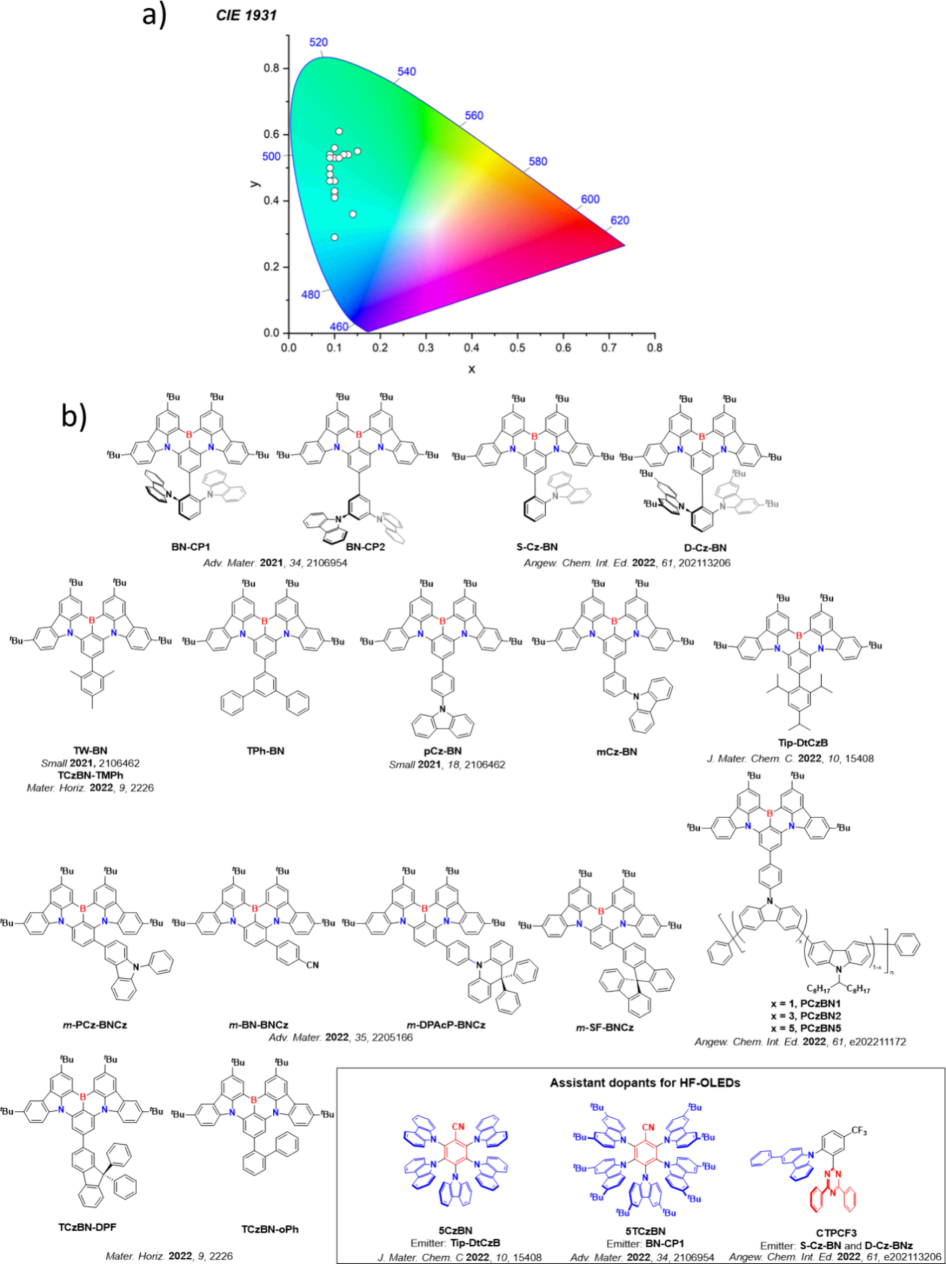
a) CIE color coordinates of substituted CzBN emitters designed to mitigate ACQ and b) structures of substituted CzBN emitters designed to mitigate ACQ and HF-OLED assistant dopants. The white circles of the CIE diagram illustrate the spread of the emission color of the device. In the chemical structures, the blue color signifies donor moieties/atoms/functional groups, while the red color signifies acceptor moieties/atoms/functional groups.

A similar design based on **BN-CP1** was presented by Zhang *et al.*,^1052^ where the DtCzBN core was substituted with an *ortho*-Czphenyl group in **S-Cz-BN** or an *ortho,ortho*-diDtCzphenyl group in **D-Cz-BN** (Figure 142). In toluene both compounds emit at λ_PL_ of 490 nm and show similar τ_d_ and Δ*E*_ST_ in 5 wt% doped CBP films at 42 and 44 μs and 0.16 and 0.14 eV for **S-Cz-BN** and **D-Cz-BN**, respectively. For **S-Cz-BN** the Φ_PL_ decreased only modestly from 95 to 84% for 1–30 wt% doped films, while for **D-Cz-BN** there was an even smaller attenuation in Φ_PL_ from 98 to 90% across the same doping range. In neat films the Φ_PL_ were 47 and 54%, yet the emission remained narrow with FWHM of 40 and 26 nm for **S-Cz-BN** and **D-Cz-BN**, respectively. Devices were fabricated at a range of doping concentrations, with 5 wt% doping offering the best performances with EQE_max_ of 22.1 and 28.7% at CIE coordinates of (0.10, 0.42) and (0.10, 0.41) for the devices with **S-Cz-BN** and **D-Cz-BN**, respectively. At 1,000 cd m^–2^ the roll-off was significant, with EQE_1000_ of 12.4 and 11.4%, owing largely to their inefficient *k*_RISC_ of 3.0 × 10^4^ s^–1^ for both. Non-doped devices with **S-Cz-BN** and **D-Cz-BN** showed EQE_max_ of 12.8 and 14.8% at CIE coordinates of (0.16, 0.59) and (0.10, 0.42), with the latter showing the narrowest emission of any non-doped TADF OLED to date with a FWHM of 21 nm. Additionally, both emitters were used in HF-OLEDs with **CTPCF3** (Figure 142) as the assistant dopant. The HF-OLEDs with **S-Cz-BN** and **D-Cz-BN** showed EQE_max_ of 30.5 and 37.2%, respectively, while the EQE_1000_ remained high at 26.2 and 34.3%.

A similar series of emitters containing mesityl (**TW-BN**), triphenyl (**TPh-BN**), *para*-phenylcarbazole (**pCz-BN**), and *meta*-phenylcarbazole (**mCz-BN**) designed to mitigate ACQ and maintain narrowband emission have been reported (Figure 142).^1053^ In 3 wt% doped mCBP films all four compounds emit similarly, with λ_PL_ of 485–495 nm (FWHM 25–30 nm) and Δ*E*_ST_ minimally varying at 0.12, 0.09, 0.15, and 0.14 eV. The spread of τ_d_ is larger at 112, 62, 89, and 95 μs for **TW-BN**, **TPh-BN**, **pCz-BN**, and **mCz-BN**, all respectively. The OLEDs with **TW-BN**, **TPh-BN**, **pCz-BN**, and **mCz-BN** showed EQE_max_ of 27.8, 28.9, 27.2, and 25.9% at CIE coordinates of (0.14, 0.36), (0.10, 0.46), (0.13, 0.54), and (0.15, 0.55), respectively. Efficiency roll-off of each was significant, with EQE_1000_ dropping to 10.7, 15.6, 12.2, and 14.0%, for the same devices. Device lifetimes were also assessed where the LT_50_ from an initial luminance of 500 cd m^–2^ were 10.4, 36.5, 27.3, and 18.6 hours, respectively.

A similar derivative was reported containing bulky triisopropyl (Tip) groups on a benzene substitutent *para* to the boron, **Tip-DtCzB** (Figure 142).^1047^ Compared to the parent emitter **DtBuCzB**, there is a modest blue-shift of the emission from 481 nm for **DtBuCzB** to 477 nm for **Tip-DtCzB** while both compounds have identical Δ*E*_ST_ of 0.13 eV in toluene. Their Φ_PL_ are also similar at 89 and 95%, as are the *k*_RISC_ values of 8.3 and 9.6 × 10^3^ s^–1^, respectively for **DtBuCzB** and **Tip-DtCzB**. The OLEDs showed improved efficiencies with the EQE_max_ increasing from 23.2% for the device with **DtBuCzB** to 28.9% for the device with **Tip-DtCzB**; both devices showed large efficiency roll-off, with EQE_100_ of 13.0 and 18.2%, respectively. Not surprisingly the efficiency roll-off in the HF-OLED with **Tip-DtCzB** and **5CzBN** (Figure 142) as assistant dopant improved, with EQE_max_ of 29.0% and EQE_100_ of 23.2%.

An alternative strategy using *meta* positioning of bulky groups has also been pursued, exemplified by ***m*-PCz-BNCz**, ***m*-DPAcP-BNCz**, ***m*-BN-BNCz**, and ***m*-SF-BNCz** (Figure 142).^1054^ Owing to their highly twisted geometry and use of phenyl spacer, the functional groups were weakly coupled to the MR-TADF core, resulting in a modest red-shift of the emission compared to the parent emitter **DtBuCzB** (481 nm in toluene),^1038^ with λ_PL_ in toluene ranging from 488–494 nm.^1054^ The four compounds possess similar Δ*E*_ST_ of 0.14–0.16 eV and high Φ_PL_ of 93–97%. Despite moderate *k*_RISC_ of around 1 × 10^4^ s^–1^ for each, the devices with ***m*-PCz-BNCz**, ***m*-DPAcP-BNCz**, ***m*-BN-BNCz**, and ***m*-SF-BNCz** showed very high EQE_max_ of 36.8, 42.0, 35.0, and 41.1%. The high EQE_max_ was supported by preferentially horizontally aligned TDM that enhanced the light-outcoupling in the devices. Efficiency roll-off was considerable though with the EQE_1000_ dropping to 19.0, 17.5, 10.9, and 17.9%, respectively.

Extending this approach, Wang *et al*. developed a family of conjugated polymers consisting of a polycarbazole backbone with pendant MR-TADF emitter **DtBuCzB** molar ratios of 1, 3, and 5% for polymers **PCzBN1**, **PCzBN3** and **PCzBN5**, respectively (Figure 142).^1055^ Beyond mitigation of ACQ the polymers facilitate the fabrication of solution-processed OLEDs. The neat film emissions of the three polymers are very similar, with λ_PL_ of 491–501 nm and FWHM of 33–43 nm. Both **PCzBN1** and **PCzBN3** also showed emission from the polycarbazole backbone; however, complete energy transfer occurred from the polycarbazole to the **DtBuCzB** in **PCzBN5**. The Φ_PL_ of the neat films ranged from 43–58%, while *k*_RISC_ varied between 8.2–21.8 × 10^4^ s^–1^. Non-doped solution-processed OLEDs with **PCzBN1**, **PCzBN3**, and **PCzBN5** showed only EQE_max_ of 3.7, 5.3, and 10.3%. Positively, these devices showed narrowband EL (FWHM of 33, 41, and 43 nm, respectively), representing rare examples of non-doped OLEDs possessing saturated color. The authors demonstrated that the EQE could be improved when using a doped EML with the polymer dispersed in mCP as the host. Optimal doping concentrations of 60, 70, and 40% for the devices with **PCzBN1**, **PCzBN3**, and **PCzBN5**, respectively, were identified. At these concentrations the Φ_PL_ increased to 65, 71, and 77%, while the EQE_max_ increased to 17.8, 17.5, and 13.3%, respectively, at CIE coordinates of (0.10, 0.43), (0.12, 0.54), and (0.11, 0.53). Additional examples of both D-A and MR-TADF emitters incorporated into polymers are presented in Section 10.1.

A series of compounds were investigated that contained different substituents *para* to the boron atom of **DtBuCzB**, designed to probe the origins of spectral broadening and annihilation pathways at higher emitter doping ratios.^1056^ The authors incorporated diphenylfluorene (**TCzBN-DPF**), mesityl (identical structure to **TW-BN**, Figure 142, but renamed **TCzBN-TMPh** here) and biphenyl (**TCzBN-oPh**, Figure 142). Together with reference emitter **DtBuCzB** these four compounds showed similar photophysics, with λ_PL_ 483, 491, 486, and 489 nm, and with Δ*E*_ST_ of 0.14, 0.13, 0.12, and 0.13 eV for **DtBuCzB**, **TCzBN-DPF**, **TCzBN-TMPh**, and **TCzBN-oPh**, all respectively in toluene. The changes in photophysics of the emitters was investigated as a function of the doping concentration (from 1–20 wt%) in SF3TRZ. A small degree of emission broadening was observed for **DtBuCzB** and **TCzBN-DPF**, which was less pronounced for **TCzBN-oPh** and absent for **TCzBN-TMPh**. The observed broadening was attributed to exciplex formation and decreases in Φ_PL_ mirrored the increasing emission contribution from the exciplex, where the Φ_PL_ of **DtBuCzB** and **TCzBN-TMPh** decreased from 93 and 94%, respectively at 1 wt% loading, to 70 and 74% at 20 wt% loading. In **TCzBN-DPF** and **TCzBN-oPh** the decrease was less pronounced, falling from 97 and 96%, to 92 and 86% at the same concentrations. An optimal doping ratio of 5 wt% was identified for the devices with **DtBuCzB** and **TCzBN-DPF**, which showed EQE_max_ of 26.3 and 26.4%, respectively. Instead, a doping ratio of 1 wt% was needed for the devices with **TCzBN-TMPh** and **TCzBN-oPh**, where the EQE_max_ were 25.1 and 26.0%, respectively. Even at the optimal doping concentrations, the devices showed strong efficiency roll-off, with EQE_1000_ dropping to 9.0, 12.0, 6.5, and 10.4% for the devices with **DtBuCzB**, **TCzBN-DPF**, **TCzBN-TMPh**, and **TCzBN-oPh**, respectively.

#### 11.3.5 Substitution of CzBN to Modulate Its Photophysics or Stability

Other examples have emerged where substitution of **CzBN** has been employed to alter other properties, including improving the energy transfer efficiency in HF-OLEDs, increasing the stability and enhancing the SOC. In examples of conspicuously non-inert substituents, Lee *et al*.^1057^ demonstrated how the coupling of naphthalene (**CzBNNa**) and pyrene (**CzBNPyr**) *para* to **CzBN** improved the stability of HF-OLEDs (Figure 143). The Δ*E*_ST_ in toluene of **CzBN** and **CzBNNa** remained the same at 0.15 eV, but that of **CzBNPyr** was significantly larger at 0.61 eV due to the low T_1_ localised on the pyrene unit. **CzBNNa** emits at λ_PL_ of 487 nm in 1 wt% doped films in mCBP, which is modestly red-shift compared to that of **CzBN** (λ_PL_ = 483 nm), while the Φ_PL_ of both compounds are near unity at 99 and 98% for **CzBN** and **CzBNNa**, respectively. Both compounds have similar *k*_RISC_ of 1.2 and 3.1 × 10^4^ s^–1^. **CzBNPyr** displayed no TADF behavior owing to its large Δ*E*_ST_ but did show similar λ_PL_ and Φ_PL_ of 485 nm and 90%, respectively, in 1 wt% doped films in mCBP to those of **CzBN** and **CzBNNa**. The devices with **CzBN**, **CzBNNa**, and **CzBNPyr** showed modest EQE_max_ of 6.3, 5.6, and 2.4%, respectively. The low EQE_max_ for the device with **CzBNPyr** was due to it not being TADF. In HF-OLEDs using **HDT-1** (Figure 143) as the assistant dopant, the EQE_max_ improved to between 19.4–22.0%. Interestingly and likely the intended outcome of the work, the device with **CZBNPyr** showed the best device stability, with LT_95_ of 29.1 hours compared to 4.7 and 6.8 hours for the other two devices. The improved device stability was attributed to the rapid clearing of long-lived triplets by the pyrene group, with detrimental impacts on EQE but at least alleviating device degradation mechanisms.

**Figure 143:**
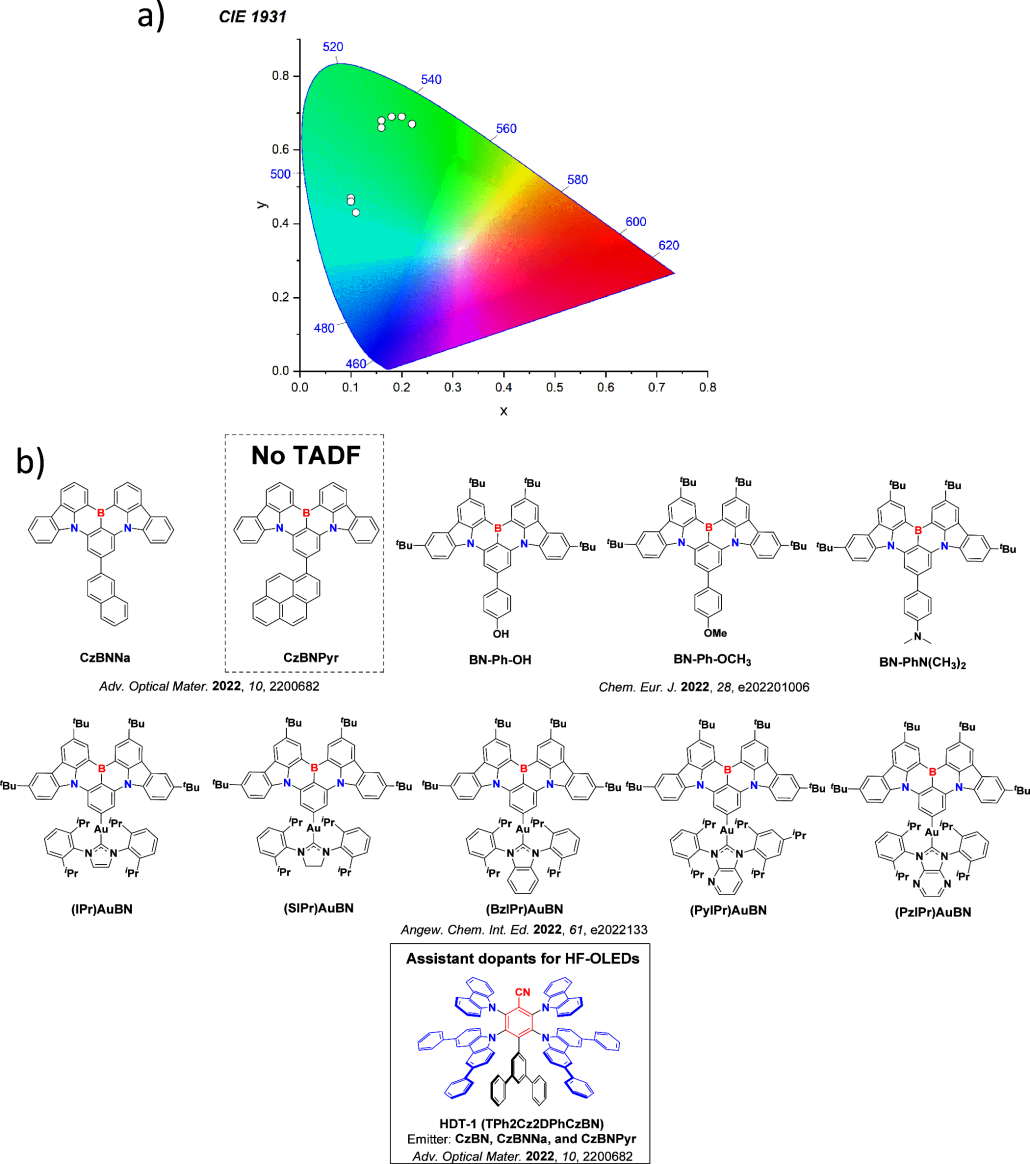
a) CIE color coordinates of OLEDs with substituted CzBN emitters where the substituents modulate either the SOC or the nature of the emissive S_1_ state and b) structures of substituted CzBN emitters where the substituents modulate either the SOC or the nature of the emissive S_1_ state, and the structure of the HF-OLED assistant dopant and an emitter that was not TADF active. The white circles of the CIE diagram illustrate the spread of the emission color of the device. In the chemical structures, the blue color signifies donor moieties/atoms/functional groups, while the red color signifies acceptor moieties/atoms/functional groups.

The influence of peripheral decoration on a MR-TADF core was further investigated by Xue *et al.*,^1058^ for three derivatives of **DtBuCzB** that contained PhOH (**BN-Ph-OH**), PhOMe (**BN-Ph-OCH_3_**), or PhNMe_2_ (**BN-PhN(CH_3_)_2_**) substituents *para* to the boron (Figure 143). The three compounds emit similarly in toluene with λ_PL_ of 485, 485, and 486 nm for **BN-PhOH**, **BN-PhOCH_3_**, and **BN-PhN(CH_3_)_2_**, respectively, all with FWHM of 24–26 nm, and Δ*E*_ST_ between 0.14 and 0.15 eV. The *k*_RISC_ ranged from 2.9 to 3.0 and 8.1 × 10^4^ s^–1^ for **BN-PhOH**, **BN-PhOCH_3_**, and **BN-PhN(CH_3_)_2_**, respectively in 3 wt% doped mCBP films. The OLEDs with **BN-PhOH**, **BN-PhOCH_3_**, and **BN-PhN(CH_3_)_2_** showed EQE_max_ of 19.0, 25.6, and 24.1%, with similar λ_EL_ between 491–493 nm. The device degradation mechanism was investigated using a combination of UV-vis absorption, transient PL, and Raman spectroscopies. **BN-PhOH** was found to be the least stable following UV irradiation, while **BN-PhN(CH_3_)_2_** was determined to be the most stable, with conformational and packing structure changes between the two ascribed as the key factor for differing degradation rates.

Cai *et al*. developed a strategy to improve the *k*_RISC_ of **DtBuCzB** derivates by incorporating as a ligand of a Au(I) NHC complex, designed to enhance SOC.^894^ Five analogues containing differing NHC ligands, **(SIPr)AuBN**, **(IPr)AuBN**, **(BzIPr)AuBN**, **(PyIPr)AuBN**, and **(PzIPr)AuBN** (Figure 143) were reported. All five emitters show similar λ_PL_ of 513–515 nm and FWHM of 30–31 nm in 2 wt% doped PMMA films, which were modestly red-shifted compared to **DtBuCzB** (λ_PL_ of 505 nm in 1 wt% doped PMMA film and FWHM of 26 nm in THF). The five emitters have similar Φ_PL_, Δ*E*_ST_, and τ_d_ of 87–92%, 0.08–0.09 eV, and 5.5–5.9 μs, revealing that the specific NHC ligand used has a minimal impact on the photophysical properties of the emitters. Nonetheless, due to the enhanced SOC associated with the gold atom (computed SOC between S_1_ and T_1_ increased from 0.05 cm^–1^ in **DtBuCzB** to 1.62 cm^–1^ in **(BzIPr)AuBN**), only delayed emission was observed in the 2 wt% doped PMMA films, implying that *k*_ISC_ ≫ *k*_r_. This is in contrast to the reference emitter **DtBuCzB**, which shows dual emission with τ_p_ and τ_d_ of 13.8 ns and 114 μs, respectively. The enhanced SOC contributed to fast *k*_RISC_ of 3.2–5 × 10^6^ s^–1^ for each of **(IPr)AuBN**, **(BzIPr)AuBN**, **(PyIPr)AuBN**, compared to 2.9 × 10^4^ s^–1^ for **DtBuCzB** in MeCN. Devices using 0.5, 0.5, 2, 4, and 1 wt% of **(SIPr)AuBN**, **(IPr)AuBN**, **(BzIPr)AuBN**, **(PzIPr)AuBN**, and **(PyIPr)AuBN** (respectively) showed EQE_max_ of 24.8, 24.0, 30.3, 24.0, and 27.6%. Owing to their short triplet lifetimes, the EQE_1000_ remained greater than 20% (20.2–28.1%) in all cases. By contrast, the OLED with **DtBuCzB** showed an EQE_max_ of 13.6% (1 wt% doping), which decreased to 7.8% at 1,000 cd m^–2^. The LT_95_ at 1,000 cd m^–2^ of the devices with **(SIPr)AuBN** and **(BzIPr)AuBN** were 47.4 and 50.2 hours, respectively.

### 11.4 Fused Indolocarbazole Emitters

Recently, a range of MR-TADF emitters have been published where a central boron is used alongside a fused indolocarbazole unit (Figure 144). Zhang *et al*. reported two emitters also based on the fusing of carbazole units to a **DtBuCzB** core: an unsubstituted compound **BN-ICz-1** and carbazole-substituted **BN-ICz-2** (Figure 144).^1059^ Both emit at λ_PL_ of 520 nm as 3 wt% doped mCBP films, and both showed narrow FWHM of 21 and 22 nm in toluene. In 3 wt% mCBP films Φ_PL_ are 95 and 93% with τd of 239 and 160 μs for **BN-ICz-1** and **BN-ICz-2**, respectively, and the Δ*E*_ST_ are similar at 0.22 and 0.18 eV in toluene. Devices with **BN-ICz-1** and **BN-ICz-2** showed EQE_max_ of 24.1 and 22.2%, respectively, at CIE coordinates of (0.24, 0.73) and (0.23, 0.72); the EQE_1000_ decreased to 10.6 and 14.4%. Using **3CTF** (Figure 144) as the assistant dopant, HF-OLEDs with **BN-ICz-1** and **BN-ICz-2** showed EQE_max_ of 30.5 and 29.8%, and improved efficiency roll-off with EQE_1000_ of 17.2 and 26.1%.

**Figure 144:**
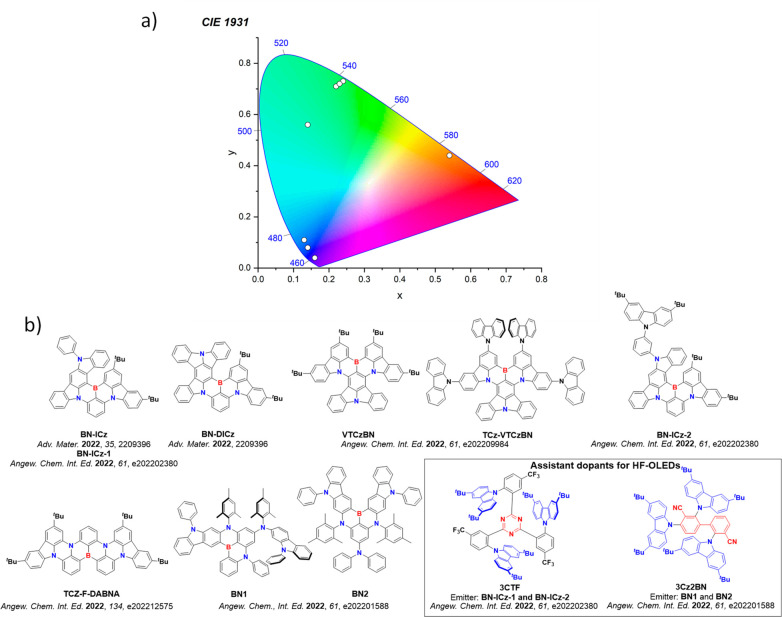
a) CIE color coordinates of OLEDs with fused indolocarbazole boron acceptor emitters and b) structures of reported fused indolocarbazole boron acceptor emitters and HF-OLED assistant dopants. The white circles of the CIE diagram illustrate the spread of the emission color of the device. In the chemical structures, the blue color signifies donor moieties/atoms/functional groups, while the red color signifies acceptor moieties/atoms/functional groups.

The same group reported a derivative of **BN-ICz** using instead an extended diindolocarbazole, **BN-DICz** (Figure 144).^1060^ This compound emits at λ_PL_ (FWHM) of 533 nm (20 nm), a modest red-shift compared to **BN-ICz-1** (renamed **BN-ICz** here), which emits at 517 nm (21 nm), both in toluene. In toluene the Δ*E*_ST_ was 0.26 eV, translating to delayed lifetimes of 496 μs and *k*_RISC_ 7.8 × 10^4^ s^–1^, while the Φ_PL_ is 99%. HF-OLEDs with **BN-DICz** using **3CTF** (Figure 144) as the assistant dopant showed very high EQE_max_ of 31.5% at CIE coordinates of (0.30, 0.58).

Luo *et al*. reported an emitter containing indolocarbazole (ICz) embedded centrally within the core of the emitter, and where the nitrogen atom of the ICz is positioned *para* to the boron. **VTCzBN** and a second analogue relacing the ^*t*^Bu groups with additional carbazole donors, **TCz-VTCzBN**, were both investigated (Figure 144).^169^ Compared to **DtBuCzB**, both compounds show a red-shifted emission in toluene with λ_PL_ at 496 nm for **VTCzBN** and 521 nm for **TCz-VTCzBN**,^169^ while the respective Δ*E*_ST_ are very small at 0.06 and 0.01 eV for leading to *k*_RISC_ of 1.0 and 0.9 × 10^6^ s^–1^. Both compounds have high Φ_PL_ of 98% in 4 wt% doped 2,6-DCzppy films. OLEDs with **VTCzBN** and **TCz-VTCzBN** showed EQE_max_ of 31.7 and 32.2% at CIE coordinates of (0.14, 0.56) and (0.22, 0.71), respectively, which decreased to 24.8% (19.8%) and 18.0% (16.0%) at 100 cd m^–2^ (1,000 cd m^–2^). The high EQE_max_ of the devices was attributed to both the high Φ_PL_ and strongly horizontally orientated TDM of the emitters.

Cheng *et al*. designed a derivative of DABNA, **TCZ-F-DABNA** (Figure 144),^1061^ where two *tert*-butylcarbazoles were fused onto the DABNA core. This increased the conjugation within the emitter, red-shifting λ_PL_ to 558 nm in toluene, while its highly twisted structure ensured that ACQ was suppressed (*vide infra*). The Δ*E*_ST_ in toluene was 0.12 eV, while in 8 wt% doped PhCzBCZ films the Φ_PL_ was 99% and the τ_d_ was 20.2 μs. Promisingly, the Φ_PL_ remained high at 92% in 40 wt% doped films, and the *k*_RISC_ in the film was measured to be 7.8 × 10^4^ s^–1^. Due to the preferential horizontally oriented TDM of the emitter, the OLEDs showed exceptionally high EQE_max_ of 39.2% at CIE coordinates of (0.54, 0.44); however, significant efficiency roll-off was apparent, with EQE decreasing to 24.4 and 7.84% at 100 cd m^–2^ and 1,000 cd m^–2^ arising from the still relatively small *k*_RISC_.

Lv *et al*. reported a series of emitters, **BN1**, **BN2**, and **BN3** (Figure 144), accessed by changing the stoichiometry of borylating reagent used.^1062^ These three compounds emit at λ_PL_ of 454, 464, and 456 nm and show narrow FWHM of 18, 15, and 17 nm, all respectively in toluene. The influence of the different π-frameworks becomes apparent in the Δ*E*_ST_, which decrease from 0.20 to 0.16 and 0.15 eV for **BN1**, **BN2**, and **BN3**, respectively. In 1 wt% doped DBFPO films there is a progressive increase in Φ_PL_ from 91 to 93 and 98% that is concurrent with an increased delay emission contribution (from 35, 45, and 76%) and faster *k*_RISC_ of 1.3, 2.6, to 25.5 × 10^4^ s^–1^ for **BN1**, **BN2**, and **BN3**, respectively. **BN3** is discussed in more detail in the “Central donor” sub-section. OLEDs with **BN1** and **BN2** showed EQE_max_ of 30.0 and 32.9%, respectively; however, the efficiency roll-off was severe, with the EQE_100_ dropping to 8 and 14.7%. To address the efficiency roll-off, HF-OLEDs using **3Cz2BN** (Figure 144) as the assistant dopant showed much improved EQE_100_ of 18.3 and 25.5%.

### 11.5 CzBN Derivatives with Multiple Acceptor Atoms

A number of doubly borylated MR-TADF compounds containing carbazole-based skeletons have also been designed (Figure 145). Similar in concept to the emitter **B2** (Figure 136),^1030^ compounds **CzB2-M/TB** and **CzB2-N/P** contain 3 nitrogen and 2 boron atoms, and 1 carbazole, while **Cz2B2-M/TB** has an extra fused carbazole unit (Figure 145).^1021^ **CzB2-M/TB, CzB2-N/P**, and **Cz2B2-M/TB** emit at λ_PL_ of 491, 504, and 483 nm in 1 wt% doped PMMA films. The addition of phenyl substituents in **CzB2-N/P** results in a smaller Δ*E*_ST_ of 0.06 eV compared to 0.12 and 0.11 eV for **CzB2-M/TB** and **Cz2B2-M/TB**. The OLED with **CzB2-N/P** showed an EQE_max_ of 26.7% at CIE coordinates of (0.15, 0.57). The efficiency roll-off was relatively low, decreasing by only 9%, at 100 cd m^–2^.

**Figure 145:**
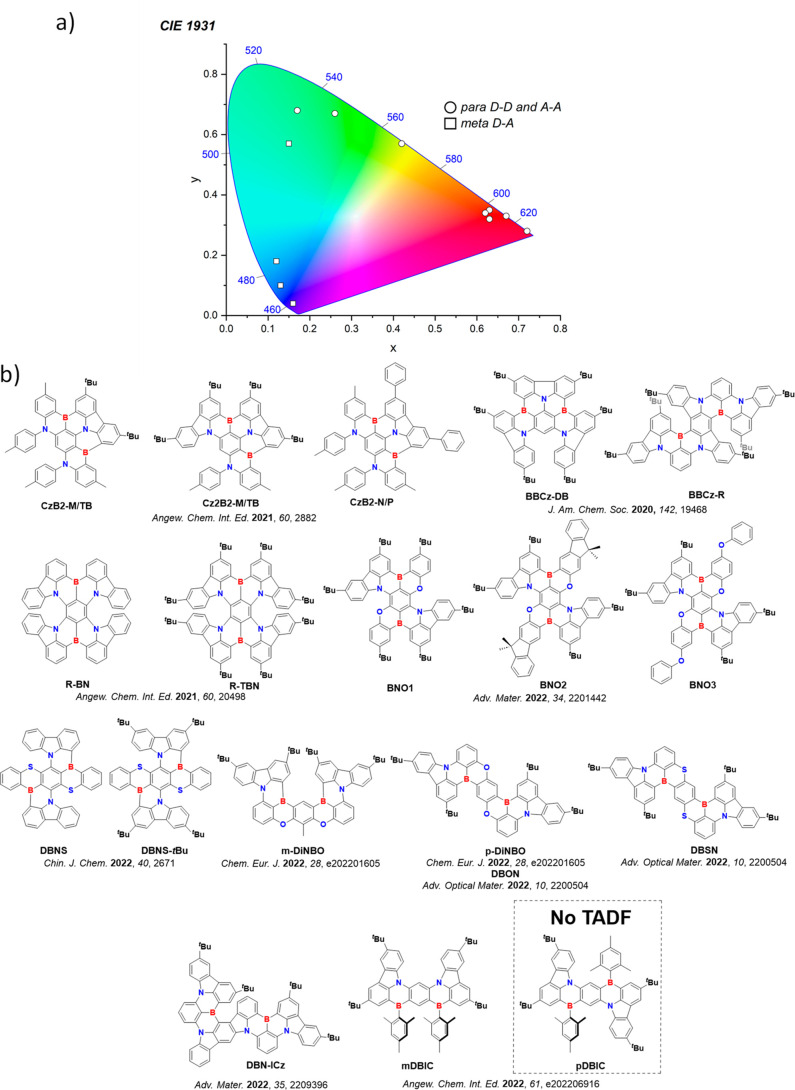
a) CIE color coordinates of OLEDs with CzBN emitters with multiple acceptor atoms and b) structures of reported CzBN emitters with multiple acceptor atoms and a derivative that is not TADF active. The white circles of the CIE diagram illustrate the spread of the emission color of the device. In the chemical structures, the blue color signifies donor atoms/functional groups, while the red color signifies acceptor atoms/functional groups.

An emerging motif to achieve relatively rare red MR-TADF emitters is to install two boron atoms *para* to each other.^177,1040^ Illustrative of the impact of the regiochemistry of the boron substitution, **BBCz-DB** and **BBCz-R** (Figure 145)^1040^ have strongly contrasting emission colors of 471 and 619 nm in 2 wt% doped mCBP films. The synergistic *para* disposition of both the electron-donating nitrogen atoms and electron-accepting boron atoms results in a destabilized HOMO and stabilized LUMO, thus decreasing the band gap, while the opposite effect is observed when the nitrogen and boron atoms are *meta*-disposed. Hence a blue-shift in the emission is observed for **BBCz-DB** (λ_PL_ = 471 nm) compared to **BBCz-SB** (λ_PL_ = 490 nm, Figure 138) while for **BBCz-R** the emission is strongly red-shifted (λ_PL_ = 619 nm, all 2 wt% doped mCBP films). Compounds **BBCz-DB** and **BBCz-R** possess Δ*E*_ST_ of 0.15 and 0.19 eV in toluene, and τ_d_ of 35 and 53 μs in the same respective films. Devices with **BBCz-DB** and **BBCz-R** showed EQE_max_ of 29.3 and 22.0% at CIE coordinates of (0.12, 0.18) and (0.67, 0.33), respectively, with the latter being the first reported red MR-TADF emitter. Very severe efficiency roll-off was observed for **BBCz-R** though, which could not attain 1000 cd m^–2^. Similarly, the EQE_1000_ dropped precipitously to 5.5% for the device with **BBCz-DB**.

The same approach to red-shift emission was also adopted by Zhang *et al*.^177^ with the emitters **R-BN** and **R-TBN** (Figure 145). These two compounds emit at λ_PL_ of 672 and 698 nm and have unity Φ_PL_ in 3 wt% doped CBP films. The same films of **R-BN** and **R-TBN** have Δ*E*_ST_ of 0.18 and 0.16 eV in toluene, and very long τ_d_ of 310 and 710 μs, respectively. The OLEDs showed EQE_max_ of 25.6 and 24.7% at CIE coordinates of (0.72, 0.28) for both devices. HF-OLEDs using the assistant dopant **Ir(mphmq)_2_tmd** (Figure 146) emitted at identical CIE coordinates and with EQE_max_ of 28.4 and 28.1% for the devices with **R-BN** and **R-TBN**, respectively.

**Figure 146:**
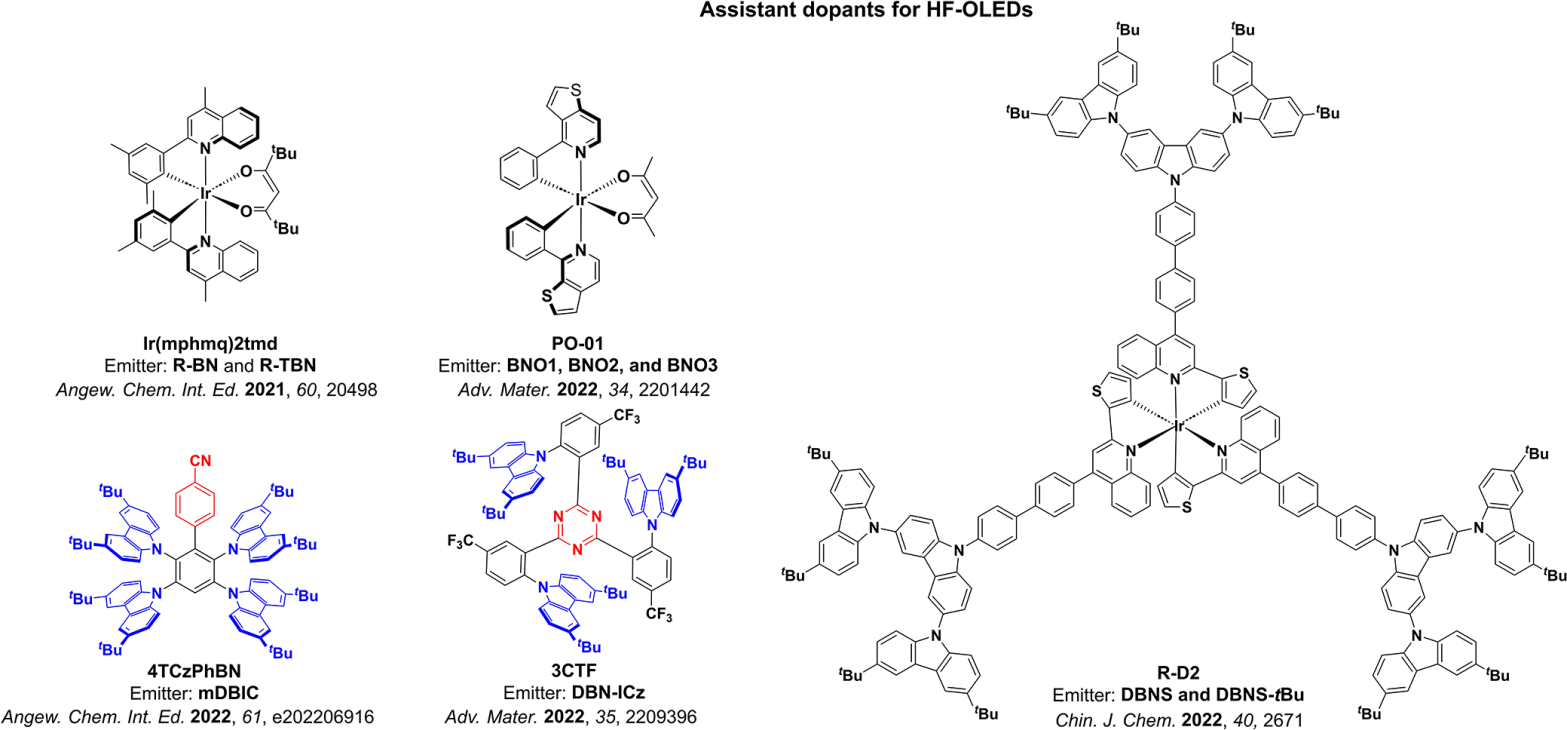
Structures of HF-OLED assistant dopants used alongside emitters in Figure 145 (the blue color signifies donor moieties/atoms/functional groups, while the red color signifies acceptor moieties/atoms/functional groups).

A similar design concept with *para* boron atoms, *para* nitrogen atoms, and *para* oxygen atoms alongside substituents ^*t*^Bu (**BNO1**), fluorene (**BNO2**), and phenoxy (**BNO3**) was also explored by Zou *et al*. (Figure 145).^1063^ Similarly to other reports, the presence of *para*-disposed donors/acceptors ensured red emission with λ_PL_ of 610, 618, and 624 nm for **BNO1**, **BNO2**, and **BNO3**, respectively in 1 wt% doped DMIC-TRZ films. These three compounds have large Δ*E*_ST_ of between 0.25 and 0.27 eV, with τ_d_ over 100 ms for each. Devices with **BNO1**, **BNO2**, and **BNO3** showed EQE_max_ of 14.9, 12.0, and 15.1%, respectively, and had significant efficiency roll-off (EQE_1000_ < 5.0% for each). Nonetheless, HF-OLEDs using **PO-01** (Figure 146) as the assistant dopant showed very high EQE_max_ of 35.6, 34.4, and 36.1%, and low efficiency roll-off (EQE_1000_ of 31.1, 29.8, and 32.1%) for the devices with **BNO1**, **BNO2**, and **BNO3**, respectively.

In a later report from Wang *et al*. **DBNS** and **DBNS-*t*Bu** (Figure 145)^1064^ containing *para*-disposed boron, nitrogen, and sulfur atoms were shown to emit similarly in the red at λ_PL_ of 631 and 641 nm in toluene, and to have similar Δ*E*_ST_ and Φ_PL_ in DCM of 0.20 and 0.19 eV, and 80 and 85%, respectively. Attributed to the presence of the heavier sulfur atoms that increase SOC, there is reasonably fast *k*_RISC_ of 2.1 and 2.2 × 10^5^ s^–1^ for these MR-TADF emitters. Solution-processed HF-OLEDs using red iridium dendrimeric sensitiser **R-D2** (Figure 146) showed surprisingly low EQE_max_ of 5.8 and 7.8% at CIE (0.64, 0.34) and (0.65, 0.34), respectively.

Two “dimeric” derivatives that contain either *meta*-disposed or *para*-disposed boron atoms (**m-DiNBO** and **p-DiNBO**, Figure 145) were reported by Liu *et al*.^1065^. Compared to the parent emitter **NBO** (Figure 150) a slight red-shift of the emission was observed, from λ_PL_ of 448 to 456 nm for **m-DiNBO**, and more so for **p-DiNBO** at 500 nm, all in toluene. These extended structures also led to narrowed emission, with FWHM decreasing from 25 nm in **NBO** to 17 and 19 nm for **m-DiNBO** and **p-DiNBO**, respectively, attributed to suppression of vibronic coupling in the latter two. The Δ*E*_ST_ decreased slightly from 0.10 eV for **NBO** to 0.06 eV for both **m-DiNBO** and **p-mDiNBO**, while comparable *k*_RISC_ were reported for all emitters in the range of 1.2–3.1 × 10^4^ s^–1^ in 3 wt% doped mCBP films. The OLEDs with **NBO**, **m-DiNBO**, and **p-DiNBO** showed EQE_max_ of 16.8, 24.2, and 21.6%, respectively, with the enhanced EQE in the dimers due to enhanced light outcoupling and improved charge balance within the EML; however, efficiency roll-off was still severe (an EQE_1000_ of 9.2% was reported only for the device with **p-DiNBO**).

**p-DiNBO** was reported again by Luo *et al*. renamed **DBON**, they also presented the S-π-S **(DBSN)** derivative Figure 145,^1066^ with λ_PL_ in toluene of 505 and 553 nm, respectively. The mono-borylated analogues of these, **SBON** and **SBSN**, showed a blue-shifted emission with λ_PL_ of 463 and 489 nm, respectively. **DBON** and **DBSN** have identical Δ*E*_ST_ of 0.13 eV in toluene and near unity Φ_PL_ of 98% in 4 wt% doped mCBP films. *k*_RISC_ was found to be faster in **DBSN** due to the larger SOC associated with the heavier chalcogen (*k*_RISC_ of 0.8 and 1.9 × 10^5^ s^–1^ for **DBON** and **DBSN**, respectively). OLEDs with **DBON** and **DBSN** showed EQE_max_ of 26.7 and 21.8% at CIE coordinates of (0.17, 0.58) and (0.42, 0.57), respectively. Despite the higher EQE_max_ for **DBON** device, it also showed larger efficiency roll-off with an EQE_1000_ of 12.0%, compared to 16.9% for the device with **DBSN**.

Zhang *et al*. reported an indolocarbazole di-borylated emitter which was two **DtBuCzB** fused together, **DBN-ICz** (Figure 145).^1060^ In toluene they report a λ_PL_ (FWHM) 542 nm (18 nm) and Δ*E*_ST_ of 0.20, while in 3 wt% doped in mCBP they reported a τ_d_ of 48 μs and a Φ_PL_ of 96%. HF-OLEDs with **DBN-ICz** using **3CTF** (Figure 146) as the assistant dopant showed very high EQE_max_ of 37.4% at CIE coordinates of (0.36, 0.59).

Wang *et al*. reported the deep blue emitter **mDBIC** (Figure 145) that contains *meta*-disposed pairs of boron and nitrogen atoms.^175^ The compound emits at λ_PL_ of 431 nm and has a Φ_PL_ of 68% in 3 wt% doped mCP films; however, in toluene the Δ*E*_ST_ is large at 0.31 eV, which translates to a slow *k*_RISC_ of 5.0 × 10^3^ s^–1^. The congener that instead has each of the boron and nitrogen atoms *para* to each other, **pDBIC** (Figure 145), was also reported and has an even larger Δ*E*_ST_ of 0.35 eV and no observable TADF. Illustrative of the challenges faced by MR-TADF emitters with slow RISC, devices with **mDBIC** showed a low EQE_max_ of 5.7% at nonetheless desirable CIE coordinates of (0.16, 0.04). HF-OLEDs with **4TCzPhBN** (Figure 146) as the assistant dopant showed much improved EQE_max_ of 13.5%.

### 11.6 Other Bridging Atoms and Groups

Instead of simply substituting or extending established MR-TADF core groups, several derivatives where carbazole or diphenylamine are replaced by other donor groups such as DMAC, PXZ, and PTZ have been explored (Figure 147). Jiang *et al*.^1067^ reported the DMAC and DPAC congeners of **DtBuCzB**, **BN-DMAC** and **BN-DPAC** (Figure 147), which emit at λ_PL_ of 485 and 490 nm in toluene and have Δ*E*_ST_ of 0.14 and 0.11 eV, respectively. In 1 wt% doped mCBP films they showed Φ_PL_ of 63 and 86% and τ_d_ of 13.9 and 11.6 μs. OLEDs with **BN-DMAC** and **BN-DPAC** showed EQE_max_ of 21.1 and 28.2% at CIE coordinates of (0.14, 0.54) and (0.14, 0.56), respectively, while the efficiency roll-off was severe, with EQE_1000_ decreasing to 12.5 and 19.1%. When TADF exciplex host mCBP:​PO-T2T was employed the EQE_max_ rose to 25.5 and 30.2% for the same devices, while the EQE_1000_ remained as high as 16.0 and 22.1%. In this exciplex host the LT_80_ were 82 and 8 hours at 500 cd m^–2^ for the devices with **BN-DMAC** and **BN-DPAC**, respectively.

**Figure 147:**
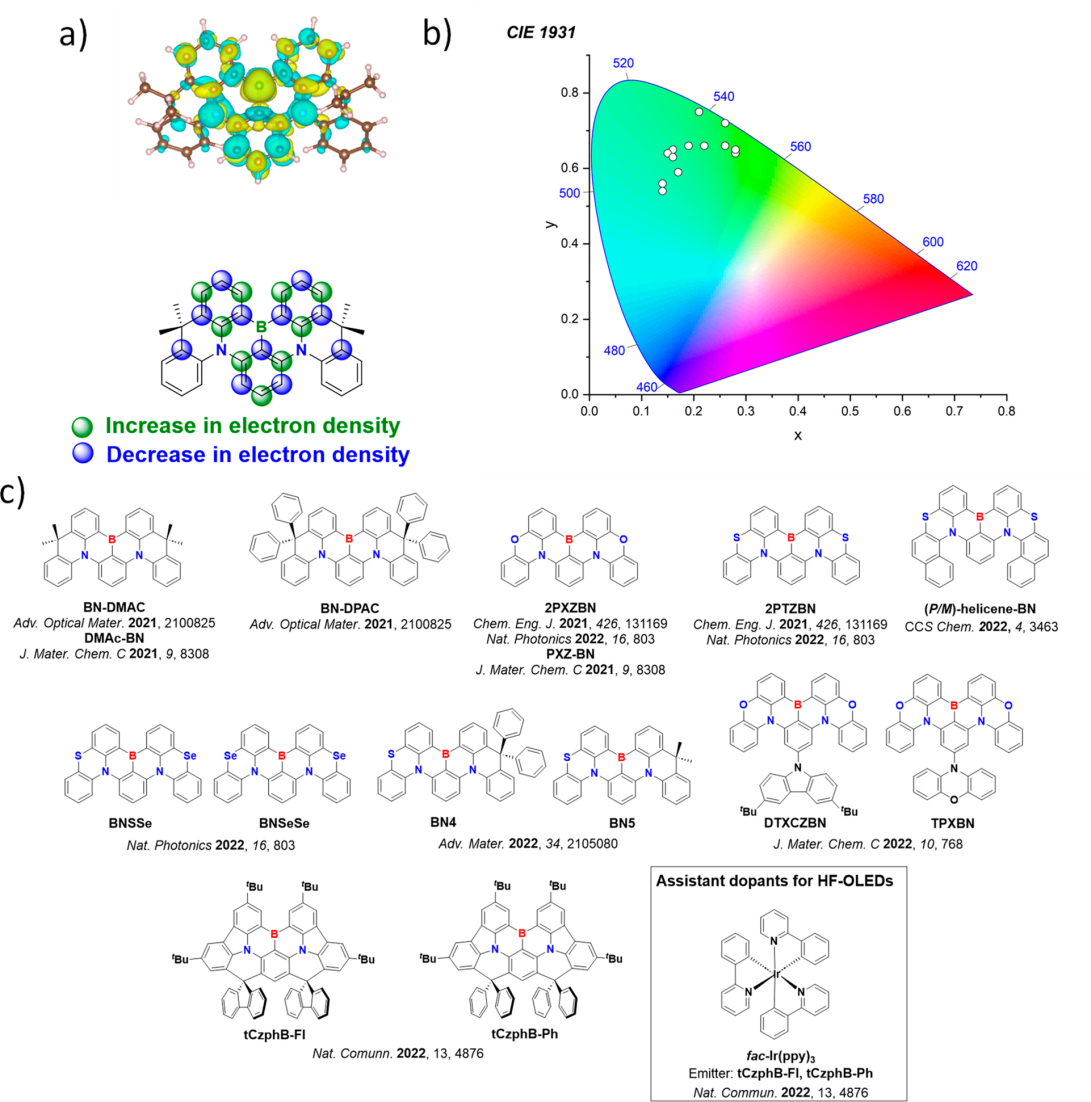
a) Computed difference density plot (top) and the schematic representation of the difference density distribution (bottom) of **BN-DMAC**, b) CIE color coordinates of OLEDs with bridged BN emitters, and c) structures of reported bridged BN emitters. Computational picture calculated S_1_ excited state from SCS-CC2/cc-pVDZ; isovalue = 0.001. The white circles of the CIE diagram illustrate the spread of the emission color of the device. In the chemical structures, the blue color signifies donor moieties/atoms/functional groups, while the red color signifies acceptor moieties/atoms/functional groups. Difference density plots calculated at the SCS-CC2/cc-pVDZ level in the gas phase; is-value = 0.01.

PXZ and PTZ analogues **2PXZBN** and **2PTZBN** (Figure 147) were reported by Hua *et al*.^1068^ Both emit in the green, with λ_PL_ of 515 and 519 nm and Φ_PL_ of 84 and 80%, respectively, in 1 wt% mCBP:​PO-TCTA doped films. In toluene both compounds have similar Δ*E*_ST_ of 0.19 and 0.15 eV, but the incorporation of the heavier sulfur decreases the τ_d_ from 25.3 to 16.1 μs and improves the *k*_RISC_ from 0.56 to 1.17 × 10^5^ s^–1^. The improved *k*_RISC_ translated into improved device performance with EQE_max_ of 17.1 and 25.5% for the devices with **2PXZBN** and **2PTZBN**, respectively, at CIE coordinates of (0.28, 0.65) and (0.28, 0.64). Efficiency roll-off is also less severe in the device with **2PTZBN** (EQE_1000_ = 17.2% compared to 7.4% in the device with **2PXZBN**).

Both **BN-DMAC** and **2PXZBN** were included in a subsequent report,^1069^ each renamed **DMAc-BN** and **PXZ-BN**, (Figure 147) where some small differences in their photophysics were recorded, likely owing to the different media.^1067,1068^ Improvements in device performance were observed for the device with **PXZ-BN** with EQE_max_ increasing to 23.3% (17.2% previously) while the device with **DMAc-BN** was lower than before, at 20.3% (21.1% previously).^1067–1069^ A similar study from Hu *et al*.^180^ extended the series containing **2PXZBN** and **2PTZBN** with analogues containing either mixed S/Se or double Se insertion, **BNSSe** and **BNSeSe** (Figure 147). The Se atoms were added to help improve RISC via a further enhanced SOC due to the ‘heavier’ atom effect of Se compared to S. The four compounds emit similarly at λ_PL_ of 523, 525, 520 and 514 nm for **2PXZBN**, **2PTZBN**, **BNSSe**, and **BNSeSe** in respective 1 wt% DMIC-TRZ doped films. The four emitters also have similar Δ*E*_ST_ between 0.12–0.15 eV. There was a progressive increase in Φ_PL_ from 71 to 91, 99, and 100% for **2PXZBN**, **2PTZBN**, **BNSSe**, to **BNSeSe**, respectively, and a concomitant decrease in τ_d_ from 38.1 to 20.7, 12.7, and 9.9 μs. The higher Φ_PL_ of **BNSSe** and **BNSeSe** was attributed to supressed ACQ due to their twisted geometry. The most striking difference between the emitters was their *k*_RISC_ rates, being 0.04 and 0.19 × 10^6^ s^–1^ for **2PXZBN** and **2PTZBN** and increasing to 0.6 and 2.0 × 10^6^ s^–1^ for **BNSSe** and **BNSeSe**, respectively, owing to their enhanced SOC from Se and confirmed by calculations. OLEDs with **BNSSe** and **BNSeSe** showed EQE_max_ of 35.7 and 36.8% at CIE coordinates of (0.22, 0.66) and (0.19, 0.66). Strikingly, the EQE_1000_ remained very high at 32.0 and 34.0%. The devices with **2PXZBN** and **2PTZBN** showed lower EQE_max_ of 30.7 and 34.6%, which dropped to 24.0 and 29.5%at 1,000 cd m^–2^. Interestingly, despite their higher *k*_RISC_, the LT_50_ of the devices with **2PTZBN**, **BNSSe**, and **BNSeSe** were much lower than that with **2PXZBN**, at 5.6, 4.8, 4.1, and 158 hours respectively. A HF-OLED using **BNSeSe** as the assistant dopant and **BN3** (Figure 140) as the terminal emitter showed an outstanding EQE_max_ of 40.5%.

A CPL-active derivative of **2PTZBN** containing naphthalene groups that induce chirality, **(*P/M*)-helicene-BN** (Figure 147).^647^ In dilute toluene **(*P/M*)-helicene-BN** emits at λ_PL_ of 520 nm, has a rather large FWHM for an MR-TADF emitters of 46 nm and a Δ*E*_ST_ of 0.18 eV in the same medium. No reason was provided for the broader emission. The photophysical properties of the M isomer were investigated in 1 wt% doped films in DMIC-TRZ, with Φ_PL_ of 98%, τ_d_ of 71.8 μs and a corresponding *k*_RISC_ of 4.6 × 10^4^ s^–1^. OLEDs of both P and M isomers were reported with EQE_max_ of 31.5% and 30.7%, respectively, at identical CIE coordinates of (0.26, 0.66). Each showed large efficiency roll-off, with EQE_1000_ of 18.7 and 17.9% for P and M isomers respectively. Its CPL properties are discussed in Section 7.

Another CPL active series was presented by Wu *et al*. where they presented two similar asymmetric compounds featuring S/N, **BN4** and **BN5**.^646^ These emitters incorporated a sulfur bridge on one side (similar to **PTZBN**, Figure 147) alongside DPAC (**BN4**) and DMAC (**BN5**) units on the other side (Figure 147). Both **BN4** and **BN5** have similar photophysical properties, with λ_PL_ of 522 and 512 nm, τ_d_ of 25 μs for both, Φ_PL_ of 96 and 92%, and Δ*E*_ST_ of 0.20 and 0.14 eV as 3 or 1 wt% doped mCPCN films, respectively. As with **PTZBN**, increased SOC from the heavy sulfur atom produced enhanced *k*_RISC_ of 1.6 and 0.7 × 10^5^ s^–1^ for **BN4** and **BN5**, respectively in toluene. Devices with both emitters and both enantiomers were fabricated, with EQE_max_ of 20.6% (19.0%) and 22.0% (26.5%) reported for **+(−)-BN4** and **+(−)-BN5**, respectively. The OLEDs were green with CIE coordinates of (0.19, 0.63) and (0.21, 0.64) for **+** and **– BN4** and (0.17, 0.59) and (0.17, 0.60) for **+** and **- BN5**, respectively. Their chiroptical properties are discussed in Section 7.

Employing **PXZ-BN** as the core, Hu *et al*. reported of emitters where ^*t*^BuCz and PXZ donors were positioned *para* to the boron centres (**TPXZBN** and **DPXZCZBN**, Figure 147).^1070^ These two compounds emit at λ_PL_ of 502 and 500 nm in toluene and have Φ_PL_ of 99 and 94% in 5 wt% doped mCBP films. They also have similar τ_d_ of 27 and 15 μs, which are correlated with their similar Δ*E*_ST_ of 0.16 and 0.13 eV in toluene and *k*_RISC_ of 0.48 and 1.11 × 10^5^ s^–1^. Devices with **TPXZBN** and **DPXZCZBN** showed EQE_max_ of 21.3 and 19.2%, respectively, at CIE coordinates of (0.16, 0.65) and (0.15, 0.64). The efficiency roll-off was modest where the EQE_1000_ was maintained at 17.4 and 17.2%.

An alterative bridging strategy was reported by Liu *et al*, where **DtBuCzB** was modified with spiro bridging units, locking all the rings.^1071^ They reported a bis phenyl spiro bridging unit, **tCzphB-Ph**, and a fluorene bridge derivative, **tCzphB-Fl** (Figure 147). The spiro linkage was added to prevent the in-plane phenyl distortion reported in **DtBuCzB**, generating planar compounds, with near pure green emission. In 2 wt% doped TPSS films, λ_PL_ of 527 nm and 535 nm was reported for **tCzphB-Ph** and **tCzphB-Fl**, respectively, with narrow FWH of 23 and 25 nm, possible due to supressed vibronic modes. Despite their small Δ*E*_ST_ of 0.04 eV, long τ_d_ of 372 and 412 μs for for **tCzphB-Ph** and **tCzphB-Fl** reported, respectively were reported, in 2 wt% doped TPSS films. Slow τ_d_ was attributed to their small Huang-Rhys factors from a result of their rigid structure. OLED devices were reported, with high EQE_max_ of 29.3% and 26.2% at CIE (0.21, 0.75) and (0.26, 0.72) for **tCzphB-Ph** and **tCzphB-Fl**, respectively. Coordinates of (0.21, 0.75) for **tCzphB-Ph** are the closest to Rec. 2020 for green (0.17, 0.80) of any reported MR-TADF emitter. Long delayed lifetime resulted in large roll-off with EQE_1000_ of 9.2% for **tCzphB-Ph** and 8.2% for **tCzphB-Fl**. HF-OLEDs using Ir(ppy)_3_ phosphorescent sensitizer improved roll-off, with **tCzphB-Ph** having an EQE at 10,000 cd m^–2^ of 30.6%.

### 11.7 Asymmetric MR-TADF Emitters

#### 11.7.1 Asymmetric MR-TADF Emitters with Nitrogen Donor Atoms

While the designs of most MR-TADF emitters are symmetrical in and around the central core unit, there are also now a range of unsymmetric analogues of **DABNA-1** or **DtBuCzB** reported (Figure 148). Qui *et. al*.^1072^ reported a family of compounds, **DPACzBN1**, **DPACzBN2**, and **DPACzBN3** (Figure 148) based on a fused carbazole and different substituted diphenylamines around the same MR-TDAF core. **DPACzBN1** emits at λ_PL_ of 479 nm in 3 wt% doped 26DczPPy films, which lies between **DABNA-1** (λ_PL_ = 460 nm in 1 wt% doped mCBP films),^118^ and **DtBuCzBN** (λ_PL_ = 493 nm in the same)^1038^ Substitution of the DPA moiety resulted in a modest blue-shift of the emission, with λ_PL_ of 470 and 475 nm for **DPACzBN2** and **DPACzBN3**, respectively. The Δ*E*_ST_ of 0.11–0.13 eV in toluene are similar to that of **DtBuCzB** (0.13 eV in toluene)^1038^ and smaller than that of **DABNA-1** (0.18 eV in 1 wt% doped mCBP film).^118^ The τ_d_ decreased from 116 μs in **DPACzBN1** to 54 and 69 μs in **DPACzBN2** and **DPACzBN3**, translating to accelerated *k*_RISC_ in the latter two compounds, from 1.2 to 2.9 and 2.1 × 10^4^ s^–1^. The Φ_PL_ range between 92–98% in 3 wt% doped 26DczPPy films. The OLEDs with **DPACzBN1**, **DPACzBN2**, and **DPACzBN3** showed EQE_max_ of 23.6, 24.0, and 27.7% at CIE coordinates of (0.14, 0.30), (0.13, 0.16), and (0.12, 0.18). Slow and inefficient *k*_RISC_ was the primary cause of the large efficiency roll-off, with EQE_1000_ of 9.6, 14.3, and 6.3%, respectively.

**Figure 148:**
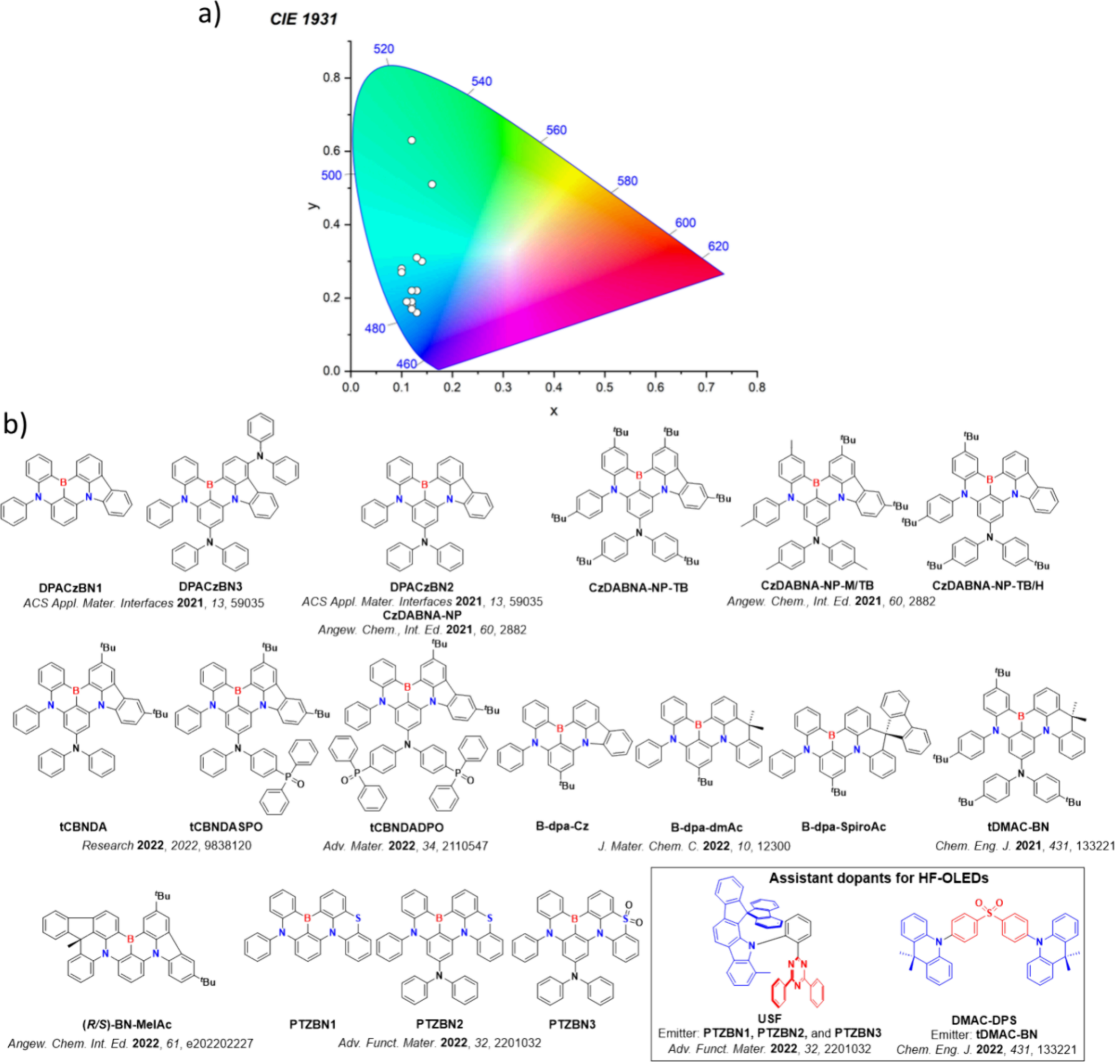
a) CIE color coordinates of reported asymmetric BN emitters with nitrogen donor atoms and b) structures of reported asymmetric BN emitters with nitrogen donor atoms and HF-OLED assistant dopants. The white circles of the CIE diagram illustrate the spread of the emission color of the device. In the chemical structures, the blue color signifies donor moieties/atoms/functional groups, while the red color signifies acceptor moieties/atoms/functional groups.

A series of similar derivatives of **DPACzBN2**; **CzDABNA-NP-M/TB**, **CzDABNA-NP-TB/H**, and **CzDABNA-NP** (Figure 148) have also been reported.^1021^ The substitution pattern around the periphery had negligible effect on the emission spectra, with these three compounds emit narrowly with λ_PL_ ranging from 461–468 nm. Similarly the Φ_PL_ range from 80–86% and all three have the same Δ*E*_ST_ of 0.18 eV. No devices were fabricated, however **CzDABNA-NP-TB** is the actual structure of the previously incorrectly identified **TBN-TPA** (Figure 149).^1020,1021^ The correct structure was confirmed by later NMR spectroscopy studies, and although the structure was wrongly identified in the initial report, the data pointed to an emitter with excellent potential, with λ_PL_ at 470 nm in toluene, a high Φ_PL_ of 98%, and a small Δ*E*_ST_ of 0.14 eV in 8 wt% doped 26-DCzppy films where the τ_d_ is 51 μs.^1020^ The OLEDs using this emitter showed an EQE_max_ of 32.1% at CIE coordinates (0.12, 0.19), while efficiency roll-off was moderate with a loss of 15% at 100 cd m^–2^.

**Figure 149:**
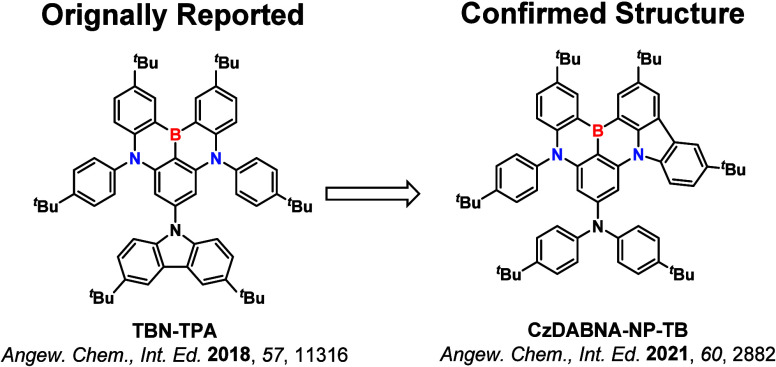
Originally reported structure of **TBN-TPA**, and the confirmed asymmetric structure **CzDABNA-NP-TB** (the blue color signifies donor moieties/atoms/functional groups, while the red color signifies acceptor moieties/atoms/functional groups).

Another derivative of **CzDABNA-NP-TB** contained fewer ^*t*^Bu substituents, **tCBNDA** and **tCBNDASPO** with a phosphine oxide group (Figure 148) were investigated by Bian *et al*..^1073^ These two compounds emit at λ_PL_ of 467 nm and with the same FWHM of 28 nm in DCM. **tCBNDA** and **tCBNDASPO** likewise have similar Δ*E*_ST_ of 0.05 and 0.04 eV in 7 and 20 wt% doped DBFDPO films, respectively. The presence of the phosphine oxide led to an increase in Φ_PL_ from 72 to 92% for **tCBNDASPO**. The presence of the phosphine oxide group also supressed ACQ, with Φ_PL_ of **tCBNDA** in 20 wt% films dropping to 29% compared to 92% for **tCBNDASPO**. The sky-blue devices have identical CIE coordinates of (0.12, 0.17), but the EQE_max_ values differed at 20.2 and 28.0%, reflecting the shorter τ_d_, higher Φ_PL_, and faster *k*_r_ of **tCBNDASPO**. The OLEDs showed moderate roll-off with EQE_100_ of 12.4 and 20.6% for the devices with **tCBNDA** and **tCBNDASPO**, respectively.

The same group reported a similar emitter, **tCBNDADPO**, which contains two phosphine oxide units attached to the DPA unit of **tCzBNDA** (Figure 148).^1074^ Addition of the phosphine oxide unit was expected to increase the ambipolar character of the emitter, assisting exciton and charge trapping. A similar λ_PL_ of 466 nm for **tCBNDADPO** compared to 467 nm in **tCBNDA** and **tCBNDASPO** was reported in DCM, with the second phosphine oxide unit having a minimal impact on the emission. At an optimal doping of 30 wt% emitter in DBFDPO, **tCBNDADPO** emits at λ_PL_ of 472 nm, has a Φ_PL_ 99%, a Δ*E*_ST_ of 0.04 eV and a *k*_RISC_ of 2.4 × 10^4^ s^–1^. Devices showed a similar emission to previous emitters, with CIE coordinates of (0.14, 0.22) here compared to (0.12, 0.17) previously reported, but with an improved EQE_max_ of 30.8%. The EQE_100_ was 23.3% reflecting a similar efficiency roll-off to **tCBNDA** and **tCBNDASPO** (*vide supra*).

A related emitter design incorporated both DPA and PTZ groups where **PTZBN1** is the parent in this series, **PTZBN2** contains a DPA *para* to the boron, and **PTZBN3** is an oxidized version of **PTZBN2** (Figure 148).^1075^ The emission of **PTZBN2** is blue-shifted at λ_PL_ of 483 nm compared to that of **PTZBN1** (490 nm in toluene), ascribed to destabilisation of the LUMO in the former, while **PTZBN3** emits at λ_PL_ of 468 nm. Interestingly, the emission spectrum of **PTZBN3** has the smallest FWHM of 30 nm compared to 41 nm in the other two, attributed to reduced structural relaxation in the excited state for this compound. All three compounds have similar Δ*E*_ST_ of 0.15–0.17 eV in toluene, along with high Φ_PL_ of 95–98% and τ_d_ of 22.4–33.5 μs in 2 wt% doped 2,6-DCzppy films. Owing to the presence of the heavy sulfur atom, SOC was enhanced and reflected in the *k*_RISC_ of 1.11, 4.51, and 1.08 × 10^5^ s^–1^ for **PTZBN1**, **PTZBN2**, and **PTZBN3**, respectively. Devices showed EQE_max_ of 26.9, 30.5, and 19.9%, respectively, at CIE coordinates of (0.16, 0.51), (0.13, 0.31), and (0.13, 0.22). The HF-OLEDs using **USF** (Figure 148) as the assistant dopant showed yet higher EQE_max_ of 32.7, 34.8, and 32.0%.

Park *et al*. reported three structurally related emitters **B-dpa-Cz**, **B-dpa-dmAc**, and **B-dpa-SpiroAc** (Figure 148), in which fused ring extensions of carbazole, dimethylacridine, or spirofluroacridine were compared.^1076^ These three compounds emit at λ_PL_ of 469, 476, and 476 nm in 3 wt% doped mCBP films, and have high Φ_PL_ of 92–98%. They all have similar Δ*E*_ST_ of 0.14–0.16 eV, while the *k*_RISC_ of the DMAC analogues improved slightly at 2.7, 4.5, and 5.0 × 10^4^ s^–1^ for **B-dpa-Cz**, **B-dpa-dmAc**, and **B-dpa-SPiroAc**, respectively. Devices showed EQE_max_ of 20.1, 24.2, and 25.1% at CIE coordinates of (0.11, 0.19), (0.10, 0.28), and (0.10, 0.27).

A similar molecular design from Wang *et al*. generated **tDMAC-BN** (Figure 148),^1025^ and borylation of the DPAc derivative produced **tDPAc-BN** (Figure 134) discussed earlier in this section. **tDMAC-BN** emits at λ_PL_ of 468 nm and has a Δ*E*_ST_ of 0.15 eV in toluene, while the Φ_PL_ is 90% and the τ_d_ is 64 μs in 1 wt% doped PMMA films. Devices showed an EQE_max_ of 19.8% at CIE coordinates of (0.12, 0.22), but the efficiency roll-off was severe. HF-devices with **DMAC-DPS** as the assistant dopant showed an EQE_max_ to 22.3%, and efficiency roll-off improved (EQE_1000_ = 10.4%).

An unusual CPL-active emitter containing a carbazole moiety and a chiral acridine unit, **(*R*/*S*)-BN-MeIAc** (Figure 148), was reported by Yang *et al*.^624^ Unlike other reported CP-MR-TADF emitters, the chirality was achieved from a stereocenter, which also, according to calculations, suggested a large SOC between S_1_ and T_2_. In toluene, **(*R*/*S*)-BN-MeIAc** emits at λ_PL_ of 497 nm (FWHM of 30 nm), and has a small Δ*E*_ST_ of 0.11 eV in 2-MeTHF. In 1 wt% doped films in DMIC-TRZ, it has a high Φ_PL_ of 96% and a *k*_RISC_ of 6.3 × 10^4^ s^–1^. The devices with the R and S isomers showed EQE_max_ of 37.2 and 36.1%, respectively, with highly horizontally orientated TDMs the key to their impressive efficiencies. At 1,000 cd m^–2^ the EQE was maintained at 26.1 and 25.1% for the R and S isomers, respectively. Their chiroptical properties are discussed in detail in Section 7.

#### 11.7.2 Asymmetric MR-TADF Emitters with Mixtures of Donating Atoms

As well as asymmetric substituents or fixed extensions, there are also now several examples of boron-based MR-TADF emitters that contain both nitrogen and oxygen donor atoms in the main core. Compound **B-O-dpa** and its congeners **B-O-Cz**, **B-O-dmAC**, and **B-O-dpAc** exemplify this design strategy (Figure 150).^1077^ For these materials the Δ*E*_ST_ decrease from 0.18 eV in **B-O-dpa** to 0.15, 0.11, and 0.06 eV for **B-O-Cz**, **B-O-dmAc**, and **B-O-dpAc** in frozen THF. The Φ_PL_ range from 86–94% in 10 wt% doped DPEPO films. The emission in PhMe red-shifts progressively from λ_PL_ = 433 nm for **B-O-dpa** to λ_PL_ of 441, 461, and 463 nm for **B-O-Cz**, **B-O-dmAc**, and **B-O-dpAc**, respectively. In the same DPEPO films all four compounds have long τ_d_ of 224, 51, 123, and 83 μs for **B-O-dpa**, **B-O-Cz**, **B-O-dmAc**, and **B-dpAc**, respectively, with the shorter τ_d_ of the latter three attributed to their smaller Δ*E*_ST_. Devices with **B-O-dpa**, **B-O-Cz**, **B-O-dmAc**, and **B-O-dpAc** showed EQE_max_ of 16.3, 13.4, 16.2, and 17.0% at CIE coordinates of (0.15, 0.05), (0.13, 0.22), (0.12, 0.21), and (0.12, 0.20), respectively. Efficiency roll-off was significant for all devices, with EQE_100_ of 2.2, 5.9, 8.4, and 9.6%, respectively. The devices were not stable either, as LT_50_ at 10 cd m^–2^ did not surpass 20 minutes, attributed to the instability of the host and the charge transport materials.

An analogous series of emitters was reported by Han *et al.*, and contained a ^*t*^Bu substituent instead *para* to the oxygen atom (**CzBNO**, **DMAcBNO**, and **DPAcBNO**, Figure 150).^1078^ These three compounds emit at λ_PL_ of 450, 470, and 468 nm with Φ_PL_ of 96, 99, and 98%, respectively in 3 wt% doped 26DczPPy films. The Δ*E*_ST_ in toluene are 0.21, 0.23, and 0.19 eV, which are larger than those of the previous series, however the τ_d_ are on average shorter at 48, 129, and 100 μs, translating to *k*_RISC_ of 3.5, 1.4, and 1.8 × 10^4^ s^–1^ for **CzBNO**, **DMAcBNO**, and **DPAcBNO**, respectively. The blue devices the same showed EQE_max_ of 13.6, 20.4, and 23.0% at CIE coordinates of (0.14, 0.08), (0.13, 0.19), and (0.13, 0.14), respectively, and the EQE_1000_ decreased to only 5.0, 8.6 and 9.1%. In HF-OLEDs using **USF** (Figure 150) as the assistant dopant the EQE_max_ increased to 25.9, 28.3, and 29.6%, while the EQE_1000_ improved to 23.0, 16.7, and 23.1%.

**Figure 150:**
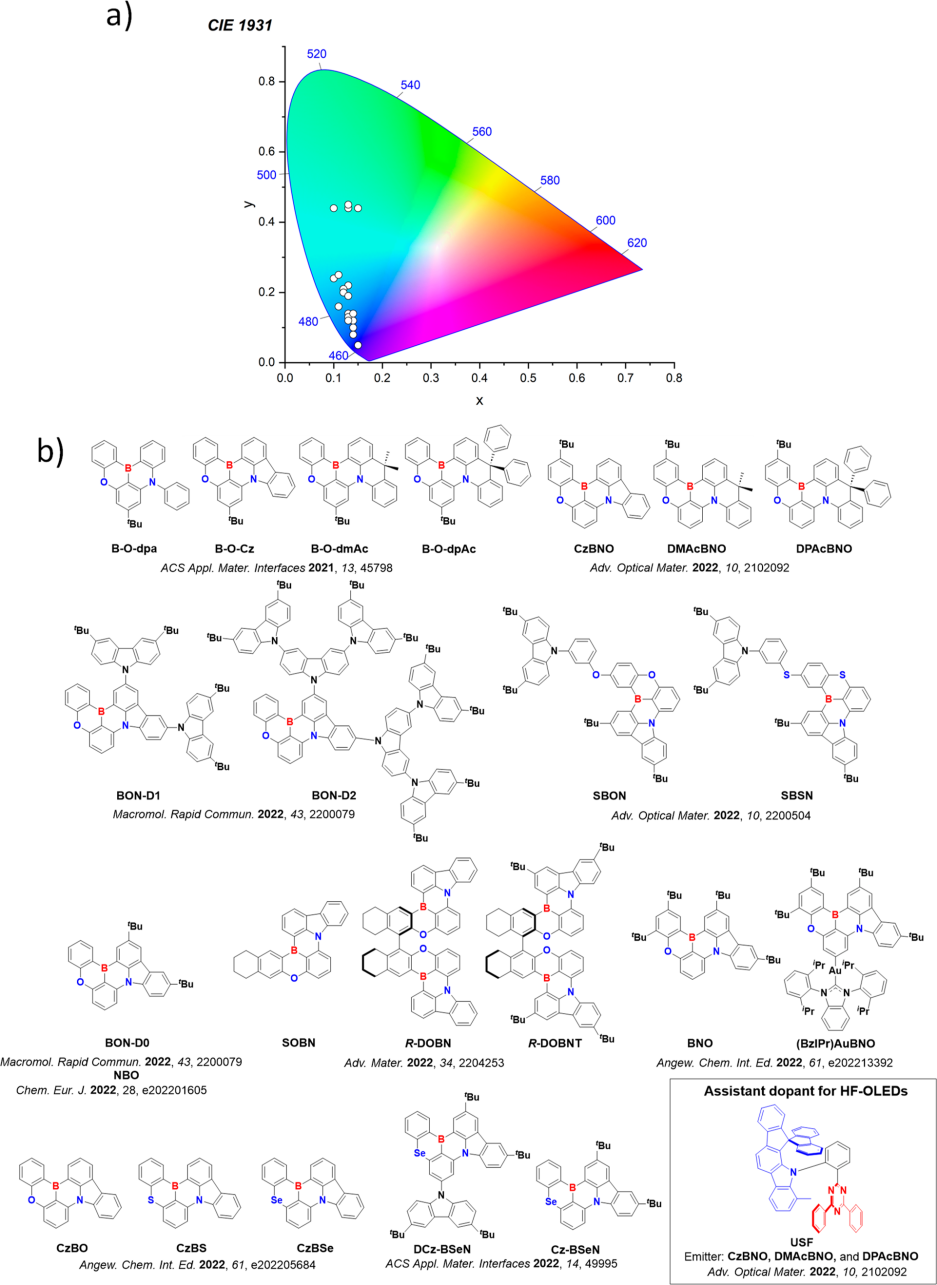
a) CIE color coordinates of OLEDs with asymmetric CzBN emitters with mixtures of donor atoms and b) structures of reported asymmetric CzBN emitters with mixtures of donor atoms and HF-OLED assistant dopants. The white circles of the CIE diagram illustrate the spread of the emission color of the device. In the chemical structures, the blue color signifies donor moieties/atoms/functional groups, while the red color signifies acceptor moieties/atoms/functional groups.

Based on a similar core to **B-O-Cz**, Liu *et al*.^1079^ investigated the impact of donor dendronisation by comparing the performance of **BON-D0** with **BON-D1** and **BON-D2** featuring 1^st^- and 2^nd^- generation carbazole-based donor dendrons (Figure 150). The use of these donor dendrons produces a red-shift of the emission from 450 nm for **BON-D0** to 476 and 472 nm for **BON-D1** and **BON-D2**, respectively in toluene. An increase in Φ_PL_ from 85% for **BON-D0** to 94 and 98% for **BON-D1** and **BON-D2** was attributed to a reduction in ACQ in the larger species. Similar *k*_RISC_ of 6.7, 7.7, and 9.8 × 10^4^ s^–1^ were reported for **BON-D0**, **BON-D1**, and **BON-D2** as 5 wt% doped mCP films. Solution-processed devices with **BON-D0**, **BON-D1**, and **BON-D2** showed EQE_max_ of 9.7%, 13.4, and 14.9% at CIE coordinates of (0.14, 0.12), (0.13, 0.44), and (0.15, 0.45), respectively. No explanation was provided by the authors to explain the significant color change from that observed in the PL. Further examples of TADF emitters containing donor dendrons are summarised in Section 10.

**BON-D0**, also reported by Liu *et al*. and renamed **NBO** here, and was presented alongside two emitters with multiple acceptor atoms, **m-DiNBO** and **p-DiNBO** (Figure 150).^1065^ The photophysics of **NBO** were reported in 3 wt% doped in mCBP, with λ_PL_ of 458 nm, Φ_PL_ of 99%, τ_d_ of 87 μs, *k*_RISC_ of 1.2 × 10^4^ s^–1^ and Δ*E*_ST_ of 0.10 eV, each comparable to the previous report.^1079^ Using their OLED stack, an improved EQE_max_ of 16.8% at CIE coordinates of (0.14, 0.14); however, efficiency roll-off was large, with EQE at 1,000 cd m^–2^ not reported. The differences in EQE_max_ may be attributed to the differing fabrication methods, with vacuum deposition used here, compared to solution processing in the previous report.

Luo *et al*. reported a decorated analogue of **BON-D0** and a sulfur-based congener, **SBON** and **SBSN**, both of which contain a pendant DtBuCzPh group (Figure 150).^1066^ These were formed as by-products of incomplete borylation during the synthesis of **DBON** and **DBSN**, respectively (Figure 150). **SBON** and **SBSN** emit at λ_PL_ of 463 and 489 nm, respectively in toluene, with FWHM of 24 and 27 nm. The diborylated analogues show significantly red-shifted emission, with λ_PL_ of 505 nm for **DBON** and 553 nm for **DBSN**. **SBON** and **SBSN** have Δ*E*_ST_ of 0.16 and 0.10 eV in toluene, Φ_PL_ of 74 and 76%, and associated *k*_RISC_ of 0.5 and 1.5 × 10^5^ s^–1^ respectively, in 4 wt% doped mCBP films. The higher *k*_RISC_ for **SBSN** was attributed to a combination of increased SOC due to the presence of the sulfur atoms, and its smaller Δ*E*_ST_. Devices with **SBON** and **SBSN** showed EQE_max_ of 13.7 and 17.6% at CIE coordinates of (0.13, 0.13) and (0.10, 0.44), respectively, while the EQE_1000_ decreased to 6.7 and 12.0%.

A CPL-active family of compounds was reported by Yan *et al*.^632^ based on a dimerized version of **SOBN**. Compounds ***R*-DOBN** and ***R*-DOBNT** (Figure 150) differ only in that the latter contains ^*t*^Bu substituents. A modest red-shift in the emission compared to **SOBN** was observed for both, with λ_PL_ of 449, 453, and 459 nm for **SOBN**, ***R*-DOBN**, and ***R*-DOBNT**, respectively, while Δ*E*_ST_ decreased from 0.19 to 0.14 and 0.12 eV in toluene. The CPL properties of ***R*-DOBN** and ***R*-DOBNT** are discussed in Section 7. In 5 wt% doped 2,6-DCzPPy films the Φ_PL_ of **SOBN** is 82% and the *k*_RISC_ is 1.4 × 10^4^ s^–1^. OLEDs with **SOBN** showed an EQE_max_ of 14.6% at CIE coordinates of (0.14, 0.14). Higher EQE_max_ of 23.9% and 25.6% were obtained for ***R*-DOBN**, and ***R*-DOBNT**, respectively, attributed to the preferential horizontal orientation of their TDMs. The efficiency of the device deceased to an EQE_100_ of 10.2% for **SOBN**.

The influence of the size of the chalcogen atom was probed by Park *et al*.^893^ across a family of oxygen (**CzBO**), sulfur (**CzBS**), and selenium containing (**CzBSe**) emitters (Figure 150). Calculations revealed that SOC between S_1_ and T_1_ expectedly increased with heteroatom size **CzBO** < **CzBS** < **CzBSe**, while the increase in SOC was even larger between S_1_ and T_2_. In 1 wt% doped mCBP films the three compounds emit at λ_PL_ of 448, 472, and 479 nm, with a slight broadening of the emission spectrum across the series from 29 to 30 and 34 nm, all respectively. The three compounds have comparable Δ*E*_ST_ of between 0.14 and 0.16 eV, but their TADF properties are remarkably different. **CzBO** and **CzBS** showed prompt fluorescent quantum yields of 83 and 15%, respectively, while it was only ∼0.1% for **CzBSe** as *k*_ISC_ is very fast in the latter. With increasing SOC, *k*_RISC_ increases from 0.9 to 22 × 10^4^ s^–1^ and 1.8 × 10^8^ s^–1^ for **CzBO**, **CzBS**, and **CzBSe**, respectively, with the latter, if accurate, being the fastest *k*_RISC_ reported to date in MR-TADF systems and one that is comparable to the most efficient D-A TADF compounds. Devices with **CzBO**, **CzBS**, and **CzBSe** showed EQE_max_ of 13.4, 23.1, and 23.9%, respectively, at CIE coordinates of (0.15, 0.05), (0.11, 0.16), and (0.10, 0.24). The efficiency roll-off followed the same trend as *k*_RISC_, with EQE_1000_ of 3.5, 15.0, and 20.0% for the same devices.

Li *et al*. reported two derivatives of **CzBSe**, **Cz-BSeN** and **DCz-BSeN** (Figure 150).^1080^ As in the previous example, inclusion of Se was designed to increase SOC, a hypothesis that was corroborated by calculations. In toluene **Cz-BSeN** and **DCz-BSeN** emit at λ_PL_ of 479 and 472 nm, with FWHM of 30 and 28 nm, while Δ*E*_ST_ are 0.15 and 0.14 eV, all respectively. Despite their Δ*E*_ST_ values, fast *k*_RISC_ of 7.5 and 8.8 × 10^6^ s^–1^ were measured for 1 wt% doped PMMA films in of **Cz-BSeN** and **DCz-BseN**, respectively. OLEDs containing 1 wt% emitter in mCBP with **Cz-BSeN** and **DCz-BSeN** showed EQE_max_ of 17.7 and 19.1%, respectively, at CIE coordinates of (0.10, 0.39) and (0.11, 0.16). When the emitter doping was increased to 5 wt% the EQE_max_ increased to 20.3 and 22.3%, but this was accompanied by a red-shift of the emission with CIE coordinates of (0.13, 0.45) and (0.11, 0.25). Both **Cz-BSeN** and **DCz-BSeN** showed improved efficiency roll-off of 32.5 and 30.0% at 500 cd m^–2^ compared to a comparable device with 5 wt% **DtBuCzB** doped in mCBP (efficiency roll-off = 62.9%), attributed to the enhanced *k*_RISC_ resulting from the Se heavy atom effect and associated increased SOC.

An alternative approach to increase SOC was introduced by Cai *et al.*, where the authors prepared Au(I) complexes with the gold centre attached *para* to the boron of the **BNO** core.^894^ The linear coordination sphere of the Au(I) was completed with a bulky NHC ligand, producing **(BzIPr)AuBNO** (Figure 150). Compared to free ligand **BNO**, **(BzIPr)AuBNO** emits at slightly longer wavelength with λ_PL_ of 471 nm compared to 454 nm for **BNO**, while narrowband emission was conserved with FWHM of 28 and 30 nm in THF for **BNO** and **(BzIPr)AuBNO**, respectively. The Δ*E*_ST_ are 0.17 and 0.11 eV for **BNO** and **(BzIPr)AuBNO** in 2 wt% doped PMMA films, and the enhanced SOC brought by the gold atom in the latter results in *k*_RISC_ accelerating from 3.7 to 110 × 10^4^ s^–1^. No devices were reported using these emitters.

### 11.8 Four-Coordinate Boron Emitters

A new family of central boron MR-TADF emitters was reported by Wang and co-workers where the central boron is four-coordinate instead of the usual trigonal planar 3-coordinate geometry (**BN1**, **TCz-BN1**, **BN2**, and **TCz-BN2**, Figure 151).^163^ **BN1** and **TCz-BN1** emit at λ_PL_ of 492 and 491 nm in 2 wt% doped mCBP films, while **BN2** and **TCz-BN2** emit at 559 and 560 nm in 5 wt% doped mCBP films. The FWHM of these four compounds are larger (FWHM = 82–108 nm) than most other MR-TADF systems while their MR-TADF character was inferred from the calculated difference density plots. The Δ*E*_ST_ values range from 0.17–0.20 eV in 5 wt% doped PMMA films, while the Φ_PL_ are moderate at 53–75%. The τ_d_ are shorter in **BN1** and **TCz-BN1** at 4.5 and 3.0 μs, respectively (2 wt% doped films mCBP) compared to 20.4 and 15.1 μs, for **BN2** and **TCz-BN2** in mCBP, all respectively. The *k*_RISC_ of this series of four-coordinate boron compounds are faster than most MR-TADF systems at 2.10–4.67 × 10^5^ s^–1^. OLEDs with **BN1**, **TCz-BN1**, **BN2**, and **TCz-BN2** showed low EQE_max_ of 5.5, 5.7, 6.7, and 7.8%, respectively, at CIE coordinates of (0.27, 0.49), (0.27, 0.45), (0.40, 0.57), and (0.41, 0.56). The HF-devices using either **TCTPCF3** (with **BN1** and **TCz-BN1**, Figure 151) and **DACT-II** (with **BN2** and **TCz-BN2**, Figure 151) as assistant dopants showed improved EQE_max_ of 9.9, 11.5, 19.9, and 25.5%, respectively, while the EQE_1000_ remained at 7.0, 10.2, 13.5, and 18.7%.

**Figure 151:**
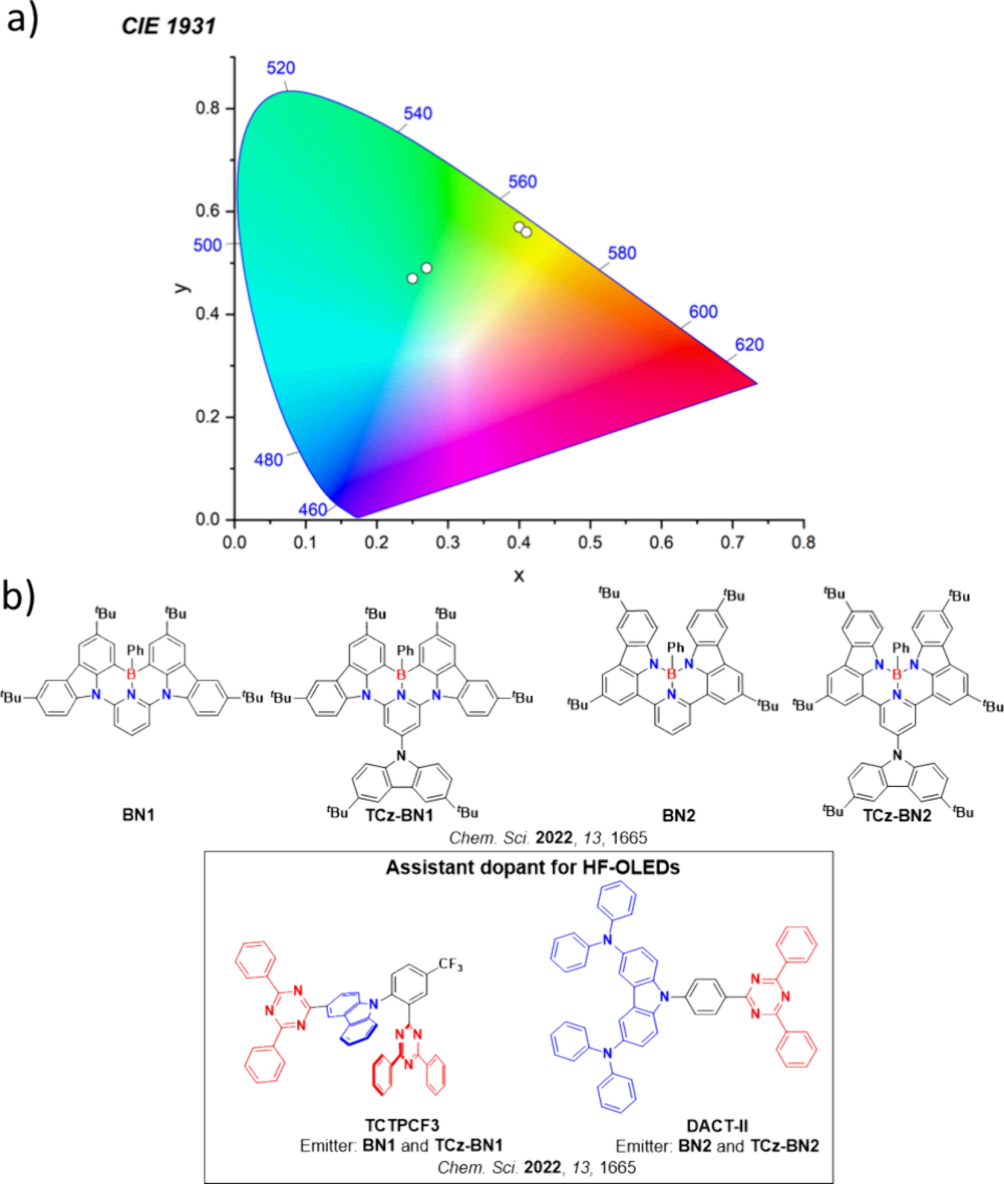
a) CIE color coordinates of OLEDs with MR-TADF emitters containing a four-coordinate boron atom and b) structures of reported four-coordinate boron MR-TADF emitters and HF-OLED assistant dopants. The white circles of the CIE diagram illustrate the spread of the emission color of the device. In the chemical structures, the blue color signifies donor moieties/atoms/functional groups, while the red color signifies acceptor moieties/atoms/functional groups.

### 11.9 Central Donor Structures with Nitrogen Donor and Boron Acceptor Compounds

An ‘inversion’ of **DABNA-1** that contains a central nitrogen donor and peripheral boron acceptors was reported by Hatakeyama and co-workers (Figure 152).^1081^ Compared to **DABNA-1** with central boron (λ_PL_ = 460 nm in mCBP), the emitters **ADBNA-Me-Mes** and **ADBNA-Me-Tip** (Figure 152) showed a red-shifted emission with λ_PL_ of 482 and 479 nm, in respective 1 wt% doped DOBNA-OAr films. **ADBNA-Me-Mes** and **ADBNA-Me-Tip** have similar Δ*E*_ST_ of 0.18 eV, and τ_d_ of 165 and 147 μs, respectively, similar to **DABNA-1** (0.18 eV and 94 μs). Sky-blue OLEDs showed EQE_max_ of 16.2 and 21.4% at CIE coordinates of (0.10, 0.27) and (0.11, 0.29), respectively. The superior performance of **ADBNA-Me-Tip** was ascribed to reduced concentration quenching due to the presence of the bulkier Tip groups.

**Figure 152:**
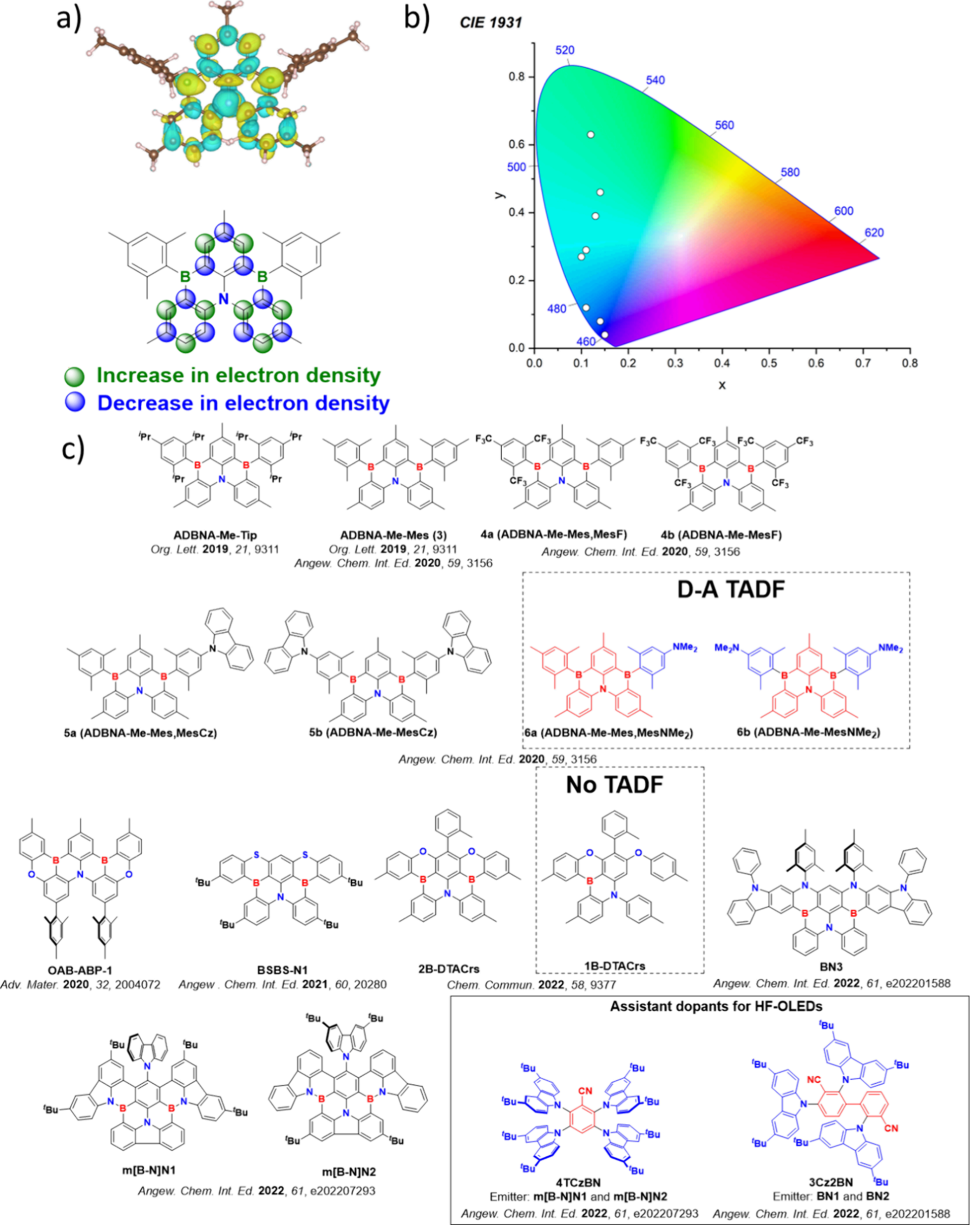
a) Computed difference density plot (top) and the schematic representation of the difference density distribution (bottom) of **ADBNA-Me-Mes**, b) CIE color coordinates of OLEDs with central nitrogen BN MR-TADF emitters, and c) structures of reported central nitrogen BN MR-TADF emitters, HF-OLED assistant dopants, a derivative which was not TADF active and emitters which showed D-A emission and not MR-TADF. Difference density plots calculated at the SCS-CC2/cc-pVDZ level in the gas phase; is-value = 0.01. The white circles of the CIE diagram illustrate the spread of the emission color of the device. In the chemical structures, the blue color signifies donor moieties/atoms/functional groups, while the red color signifies acceptor moieties/atoms/functional groups.

Further exploring this inverted design, symmetric (**4b**, **5b** and **6b**) and asymmetric (**4a**, **5a** and **6a**) derivatives of these compounds were reported, in which the methyl groups of the mesityl substituent were replaced either entirely with CF_3_ groups or by carbazole or NMe_2_ at positions *para* to the borons (Figure 152).^150^ Notably, the photophysical behavior of **6a** and **6b** was markedly different to that of the other derivatives, with emission spectra that are much broader and with much lower Φ_PL_ values, reflecting a change in the nature of the excited states from SRCT to LRCT, a reflection of the D-A structure of these compounds. The change in nature of the S_1_ state was also reflected in the much more pronounced positive solvatochromism. Indeed, the use of moieties previously identified as having MR-TADF character as acceptors in D-A TADF systems has since been reported frequently.^138,304,310,314,1082,1083^ Compounds **4a**, **4b**, **5a** and **5b** showed red-shifted emission compared to **DABNA-1** with λ_PL_ in 1 wt% doped PMMA films between 485–491 nm, and with similar Φ_PL_ of 86–93% and Δ*E*_ST_ of 0.17–0.19 eV. No devices were fabricated in this study.

Ikeda *et al*.^179^ reported the green-emitting compound **OAB-ABP-1** (λ_PL_ = 506 nm) that contains both oxygen and nitrogen donor atoms in conjunction with two boron acceptor atoms (Figure 152). In 1 wt% doped DOBNA-OAr films this compound has a Δ*E*_ST_ of 0.12 eV, a τ_d_ of 32 μs, and a Φ_PL_ of 90%. Solution-processed OLEDs showed an EQE_max_ of 21.8% at CIE coordinates of (0.12, 0.63). The OLEDs showed excellent efficiency roll-off with the EQE_1000_ remaining as high as 17.4%, while the lifetime was measured to be 11 hours (LT_50_) at a luminance of 300 cd m^–2^. This report showed that color tuning by extension of the π-system was indeed possible with central-donor MR-TADF emitters, similar to the central-acceptor MR-TADF materials discussed previously. Nagata *et al*.^1084^ reported a similar structure **BSBS-N1** (Figure 152) that contains two sulfur donor atoms in addition to the central nitrogen donor. In 2 wt% doped mCBP films this compound emits at λ_PL_ of 478 nm, has a Δ*E*_ST_ of 0.14 eV, a Φ_PL_ of 89%, and a short τ_d_ of 5.6 μs. As a result of the enhanced SOC associated with the heavy S atoms, the *k*_RISC_ is much faster than most MR-TADF emitters at 1.9 × 10^6^ s^–1^. The devices showed an EQE_max_ of 21.0% at CIE coordinates of (0.11, 0.22), yet despite the efficient *k*_RISC_, the reported EQE_100_ was only 16.3%.

Nagata *et al*.^1084^ reported the similar structure **BSBS-N1** (Figure 152) that contains two sulfur donor atoms in addition to the central nitrogen donor. In 2 wt% doped mCBP films this compound emits at λ_PL_ of 478 nm, has a Δ*E*_ST_ of 0.14 eV, a Φ_PL_ of 89%, and a short τ_d_ of 5.6 μs. As a result of the enhanced SOC associated with the heavy S atoms, the *k*_RISC_ is much faster than most MR-TADF emitters at 1.9 × 10^6^ s^–1^. The devices showed an EQE_max_ of 21.0% at CIE coordinates of (0.11, 0.22), yet despite the efficient *k*_RISC_, the reported EQE_100_ was only 16.3%.

We likewise reported a similar structure, **2B-DTACrs**, in which oxygen donors were used instead of sulfur (Figure 152).^159^ This compound emits in the deep blue, with λ_PL_ and FWHM of 448 nm and 24 nm and with Φ_PL_ of 74% in 5 wt% doped mCBP films. Interestingly, while TADF was evident in **2B-DTACrs** (τ_d_ of 13.1 μs and *k*_RISC_ of 1.3 × 10^5^ s^–1^), the corresponding mono-borylated emitter **1B-DTACrs** (Figure 152) did not show TADF. OLEDs with **2B-DTACrs** showed EQE_max_ of 14.8% with CIE coordinates of (0.15, 0.04), while the TADF-inactive devices with **1B-DTACrs** showed EQE_max_ of only 1.3%. Despite the promising EQE_max_ and *k*_RISC_ for **2B-DTACrs**, efficiency roll-off was severe and an EQE_1000_ could not be recorded.

Lv *et al*. reported an emitter, **BN3**, with indolocarbazole in the skeleton (Figure 152), which was accessed by changing the stoichiometry of the borylating reagent (two other emitters, **BN1** and **BN2**, are discussed in detail in the Fused Indolocarbazole Emitters section).^1062^ **BN3** emits at λ_PL_ of 456 nm and has a narrow FWHM 17 nm in toluene. Compared to **BN1** and **BN2**, **BN3** displays the smallest Δ*E*_ST_ of 0.15 eV owing to its longer π-framework (0.20 and 0.16 eV for **BN1** and **BN2**, respectively). In 1 wt% doped DBFPO films **BN3** has a larger Φ_PL_ of 98% and faster *k*_RISC_ of 2.55 × 10^5^ s^–1^ compared to **BN1** and **BN2** (91 and 93% for Φ_PL_ and 1.3 and 2.6 × 10^4^ s^–1^ for *k*_RISC_, all respectively). The RISC efficiency in **BN3** particularly benefits not only from the smallest Δ*E*_ST_ but also from significant SOC between S_1_ and a closely lying T_2_ state. OLEDs with **BN3** showed EQE_max_ 36.8%, larger than the others, and representing the joint highest EQE_max_ of a blue MR-TADF emitter.^306^ However, the efficiency roll-off was severe, with the EQE_100_ dropping 19.0%. To address the efficiency roll-off, HF-OLEDs using **3Cz2BN** (Figure 152) as the assistant dopant showed much improved EQE_100_ of 34.0% for the same emitter.

Most boron-based MR-TADF compounds contain three C-B bonds. By contrast, two emitters containing B-N bonds, **m[B-N]N1** and **m[B-N]N2**, were reported by Meng *et al*. (Figure 152).^1085^ **m[B-N]N1** and **m[B-N]N2** differ only in the positions of the peripheral ^*t*^Bu groups, and both emit in the sky blue with λ_PL_ of 481 and 490 nm in toluene, respectively, where the red-shifted emission of **m[B-N]N2** was attributed to the stronger donating ability of ^*t*^BuCz donor compared to Cz. Both compounds have comparable Δ*E*_ST_ of 0.15 and 0.13 eV and high Φ_PL_ of 91 and 90% in 2 wt% doped mCPBC films, which translate into comparable *k*_RISC_ of 1.59 and 1.44 × 10^4^ s^–1^. Devices with **m[B-N]N1** and **m[B-N]N2** showed EQE_max_ of 18.1 and 17.3% at CIE coordinates of (0.13, 0.39) and (0.14, 0.46), respectively. Unfortunately, both devices showed severe efficiency roll-off, with EQE_1000_ of around 6%. In HF-OLEDs with **4TCzBN** (Figure 152) as the assistant dopant the EQE_max_ increased to 36.0 and 33.4% while the EQE_1000_ improved to 27.6 and 24.7%. Further, the LT_50_ at 1,000 cd m^–2^ for the HF-OLEDs were an impressive 602 and 535 hours.

### 11.10 Single Donor Acceptor Atoms

So far most of the emitters contain at least three functional dopant atoms within the PAH skeleton, but recently two papers have emerged where the MR-TADF skeleton contains only one boron and one nitrogen atom (Figure 153). Bae *et al*.^1086^ reported a family, **BN1**, **BN2**, **BN3**, and **BN4** (Figure 153). These four compounds emit at λ_PL_ of 401, 415, 420, and 417 nm, respectively, with modest FWHM of between 25–36 nm. Their Δ*E*_ST_ in 1 wt% doped DPEPO films are large at 0.36, 0.28, 0.29, and 0.24 eV for **BN1**, **BN2**, **BN3**, and **BN4**, respectively. No delayed emission was observed for **BN1**, however long τ_d_ of 16.2, 13.3, and 4.2 ms were measured for **BN2**, **BN3**, and **BN4**, corresponding to slow *k*_RISC_ of 0.4, 0.6, and 2.0 × 10^2^ s^–1^. Devices with **BN4** showed an EQE_max_ of 9.1% at CIE coordinates of (0.17, 0.04), however the efficiency roll-off was severe and an EQE_100_ was not reported.

**Figure 153:**
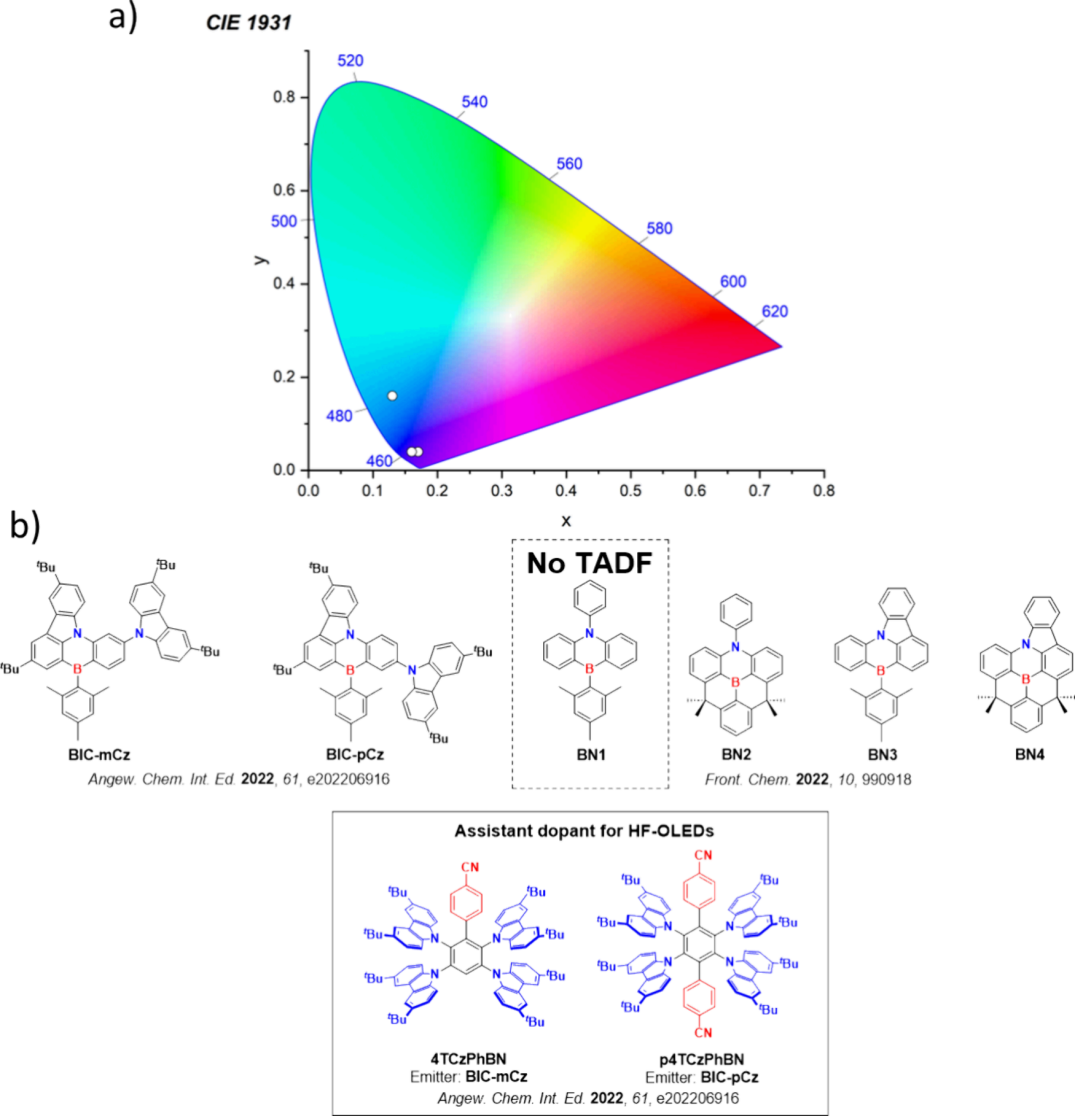
a) CIE color coordinates of OLEDs with single donor and acceptor MR-TADF emitters and b) structures of reported single donor and acceptor MR-TADF emitters, HF-OLED assistant dopants and a derivative that is not TADF active. The white circles of the CIE diagram illustrate the spread of the emission color of the device. In the chemical structures, the blue color signifies donor moieties/atoms/functional groups, while the red color signifies acceptor moieties/atoms/functional groups.

A similar series was reported by Wang *et al.*,^175^ but where the compounds contained an extra carbazole donor decorated either *meta* or *para* to the nitrogen (**BIC-mCz** and **BIC-pCz**, Figure 153). **BIC-mCz** and **BIC-pCz** emit at λ_PL_ of 432 and 471 nm in 2 wt% doped mCP films, respectively, while the Δ*E*_ST_ are 0.29 and 0.15 eV in toluene. The origins of the unexpected contrast in Δ*E*_ST_ were not discussed, and both compounds showed slow but surprisingly similar *k*_RISC_ at 4.0 and 3.1 × 10^3^ s^–1^. The devices showed EQE_max_ of 7.0 and 13.3% at CIE coordinates of (0.16, 0.04) and (0.13, 0.16), accompanied by strong efficiency roll-off. An EQE_1000_ was not observed for the device with **BIC-mCz**, and was only 1.2% for the device with **BIC-pCz**. In HF-OLEDs with **BIC-mCz** and **BIC-pCz** using **4TCzPhBN** (with **BIC-mCz**) and **p4TCzPhBN** (with **BIC-pCz**) as assistant dopants (Figure 153), the EQE_max_ increased to 19.4 and 39.8%, respectively, at CIE coordinates of (0.16, 0.05) and (0.14, 0.16).

### 11.11 Central Nitrogen Donor with Ketone Acceptor

#### 11.11.1 QAO and Substituted QAO

Another important class of MR-TADF emitters contain carbonyl groups as electron-acceptors instead of boron atoms (Figure 154 to Figure 156). The electron-accepting planar carbonyl groups act in concert with a central donating nitrogen atom to ensure the complimentary HOMO-LUMO pattern that supports MR-TADF emission. The first carbonyl-containing MR-TADF compound was reported in 2019 in the form of **QAO** ^1087^ (also known as **QAD** ^1088^ and **DiKTa**,^162^ Figure 154). This compound emits at λ_PL_ of 466 nm (FWHM of 32 nm), has a Δ*E*_ST_ of 0.18 eV in toluene, and τ_d_ of 93 μs in 5 wt% doped mCP films,^1087^ with comparable Δ*E*_ST_ of 0.19 eV and τ_d_ of 23 μs elsewhere reported in toluene.^162^ Devices showed an EQE_max_ of 19.4% at CIE coordinates of (0.13, 0.18).^1087^ However, the efficiency roll-off was severe with an EQE_100_ of 9.2%. The EQE_max_ was even lower using an alternate stack,^162^ at 14.5%, however the maximum luminance was vastly improved from 1,100 cd m^–2^ in the original report,^1087^ to 10,385 cd m^–2^ in the subsequent study.^162^

**Figure 154:**
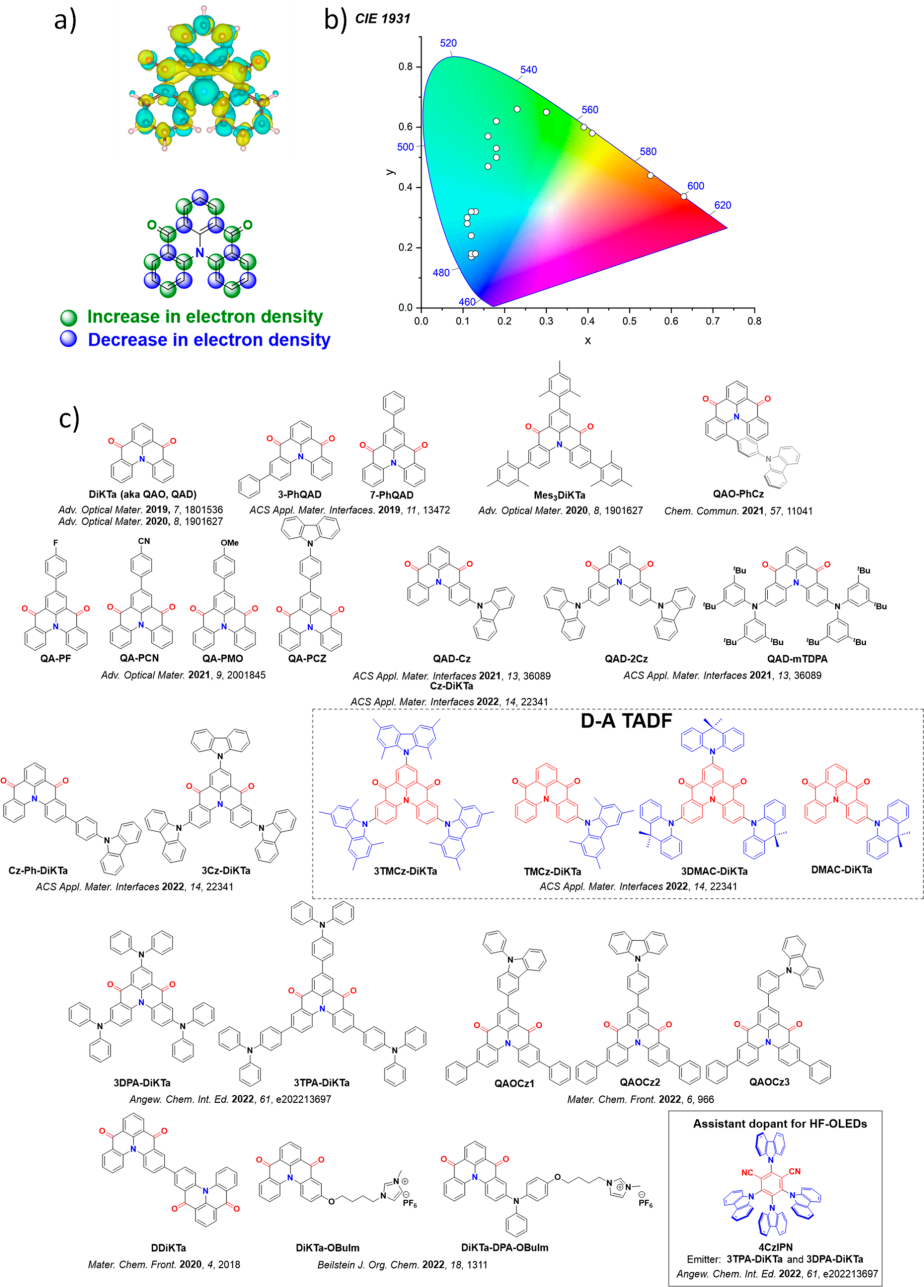
a) Computed difference density plot (top) and the schematic representation of the difference density distribution (bottom) of **QAO**, b) CIE color coordinates of OLEDs with **QAO** derivatives, and c) structures of reported **QAO**-based emitters, HF-OLED assistant dopants and emitters which showed D-A emission and not MR-TADF. Difference density plots calculated at the SCS-CC2/cc-pVDZ level in the gas phase; is-value = 0.01. The white circles of the CIE diagram illustrate the spread of the emission color of the device. In the chemical structures, the blue color signifies donor moieties/atoms/functional groups, while the red color signifies acceptor moieties/atoms/functional groups.

Two similar emitters were reported with phenyl groups located about the periphery, **3-PhQAD** and **7-PhQAD** (Figure 154).^1088^ The location of the phenyl substituent did not significantly affect the λ_PL_, the Δ*E*_ST_ or the Φ_PL_. **QAD**, **3-Ph-QAD**, and **7-Ph-QAD** emit narrowly with λ_PL_ ranging between 464–466 nm, similar Δ*E*_ST_ of 0.18–0.19 eV in toluene, and Φ_PL_ of 68–73% in 2 or 5 wt% doped mCP films. However, in the OLEDs the phenyl-substituted derivatives experienced a red-shifted and broader emission with λ_EL_ of 480 and 472 nm and FWHM of 44 and 34 nm for the devices with **3-Ph-QAD** and **7-Ph-QAD**, respectively, compared to 466 and 32 nm for the device with **QAD**. The presence of the phenyl substituent did not impact tangibly the EQE_max_ though, with values of 19.1 and 18.7% for devices with **3-Ph-QAD** and **7-Ph-QAD** respectively, compared to 19.4% reported for the device with the parent compound. Efficiency roll-off was again substantial at 46 and 71%, respectively at 100 cd m^–2^.

A strategy to mitigate ACQ in carbonyl-based MR-TADF OLEDs was introduced by our group.^162^ The decoration of three mesityl groups about the core **DiKTa** structure in **Mes_3_DiKTa** were key to both suppression of aggregate emission and reducing ACQ (Figure 154). However, the mesityl groups promote a modest red-shift in the emission, with λ_PL_ shifting from 453 nm in **DiKTa** to 468 nm in **Mes_3_DiKTa** (in toluene), while having a minimal effect on both Δ*E*_ST_ at 0.20 and 0.19 eV and τ_d_ of 23 and 33 μs for the same, respectively. The effect of mesityl substitution was most apparent in the changes of Φ_PL_ with concentration. The Φ_PL_ of **DiKTa** decreased substantially with increasing concentration, while for **Mes_3_DiKTa** the Φ_PL_ remained high up to about a 10 wt% doping. Furthermore, in neat films a distinct excimer emission was observed for **DiKTa**, which was not seen for **Mes_3_DiKTa**. Mirroring the changes in PL, the device with **Mes_3_DiKTa** showed a red-shifted emission with CIE coordinates of (0.12, 0.32) compared to that of the device with **DiKTa** (0.14, 0.18). The devices with **Mes_3_DiKTa** showed an improved EQE_max_ of 21.1% compared to those with **DiKTa** at 14.7%, while the efficiency roll-off was also improved to 31% at 100 cd m^–2^ compared to 43% for the **DiKTa**-based device.

A series of aryl-substituted derivatives were introduced onto the **QAO** core in **QA-PF**, **QA-PCN**, **QA-PMO**, and **QA-PCz** (Figure 154) in an effort to tune the photophysical properties by external substitution.^203^ The incorporation of electron-withdrawing substituents induced a modest blue-shift in emission, with λ_PL_ of 478 and 477 nm in **QA-PF** and **QA-PCN** compared to 485 and 480 nm in **QA-PMO** and **QA-PCZ**, respectively, and with respective Φ_PL_ of 89, 68, 66, and 71% in 3 wt% doped mCP films. The Δ*E*_ST_ and τ_d_ values ranged between 0.18 and 0.25 eV and 224 and 484 μs. The effect of substitution on the broadness of the emission was also probed computationally, where the authors suggested that the addition of these peripheral groups helped to suppress high-energy vibrations responsible for the broadened emission. OLEDs with **QA-PF**, **QA-PCZ**, **QA-PMO**, and **QA-PCz** showed similar EQE_max_ of 16.8, 16.9, 15.0, and 17.5% at CIE coordinates of of (0.12, 0.17), (0.12, 0.18), (0.11, 0.30), and (0.11, 0.28), all respectively. Efficiency roll-off was severe though, at between 44 and 77% at 100 cd m^–2^.

A chiral derivative, **QAD-PhCz** (Figure 154) was reported where the chirality was induced as a result of its helical structure.^642^ Although emission was attributed to a TSCT state, it would appear that it is in fact from the MR-TADF core, owing to its similar emission properties to those of **QAO**. In toluene **QAD-PhCz** emits at λ_PL_ of 460 nm while **QAO** emits at 453 nm, while the subdued observed solvatochromsim is again suggestive of an excited state of SRCT character.^162^ Owing to these observations it is included alongside MR-TADF materials. In 5 wt% mCBP films, the Φ_PL_ is 47% and τ_d_ is 40 μs, while Δ*E*_ST_ is 0.11 eV in toluene, representing modest changes compared to those reported for **QAO** (72%, 93 μs and 0.19 eV in 5 wt% mCP films).^1087^ The devices showed an EQE_max_ of 14.0% at CIE (0.13, 0.18); however, the efficiency roll-off was large, with an EQE_100_ of 8.6%. Its chiroptical properties are discussed in Section 7.

The effects of donor substitution were investigated by Huang *et al.*,^1089^ where the **QAD** core was substituted with one carbazole (**QAD-Cz**), two carbazoles (**QAD-2Cz**), or *tert*-but­yl­di­phen­yl­amines **(QAD-mTDPA)**, Figure 154. The emission of **QAD-Cz**, **QAD-2Cz**, and **QAD-mTDPA** is red-shifted compared to **DiKTa** (λ_PL_ = 463 nm in 3.5 wt% mCP)^162^ at λ_PL_ of 500, 526, and 587 nm in 1 or12 wt% doped mCP films, or 1.5 wt% doped CBP films respectively. Similar to B/N MR-TADF compounds, addition of peripheral donor groups leads to broadened emission spectra, with FWHM of 50, 50, and 62 nm, respectively, attributed to a combination of increased structural flexibility and increased LRCT character of the excited state. **QAD-Cz**, **QAD-2Cz**, and **QAD-mTDPA** have very high Φ_PL_ of 100, 100, and 97%, while the Δ*E*_ST_ values are 0.17, 0.17, and 0.33 eV in the doped films. Devices with **QAD-Cz**, **QAD-2Cz**, and **QAD-mTDPA** showed EQE_max_ of 20.3, 27.3, and 26.3% at CIE coordinates of (0.16, 0.47), (0.30, 0.65), and (0.55, 0.44), respectively. The EQE_100_ diverged considerably at 5.4, 23.9, and 12.9%, respectively. Investigation of the efficiency roll-off identified a combination of TTA and SPA as the primary detrimental factors, allowed by the inefficient *k*_RISC_ in **QAD-Cz** and **QAD-mTDPA**.

Recently we demonstrated how the strength of the peripheral donor group impacts the nature of the emissive excited state.^1090^ With DMAC (**DMAC-DiKTa** and **3DMAC-DiKTa**) and tetramethylcarbazole (**TMCz-DiKTa** and **3TMCz-DiKTa**) as the donor substituents (Figure 154) the excited state character become LRCT and the emission resembled D-A TADF systems, reflected in the broad FWHM (71–116 nm) in 2 wt% doped mCP films. However, when carbazole (**Cz-DiKTa** and **3Cz-DiKTa**) and 4-*N*-carbazolylphenyl (**Cz-Ph-DiKTa**) were used, narrowband emission (FWHM 47–54 nm) associated with SRCT excited states was observed (Figure 154). The emission also red-shifted from λ_PL_ of 502 nm in mono-substituted (**Cz-DiKTa** (same structure as **QAD-Cz**) to 539 nm in tri-substituted **3Cz-DiKTa** in 2 wt% doped mCP films. The presence of the phenyl spacer in **Cz-Ph-DiKTa** instead contributed to a blue-shifted emission at λ_PL_ of 486 nm. Each of these carbazole-based derivatives has a similar Δ*E*_ST_, ranging between 0.10–0.16 eV, and τ_d_ ranging from 153 to 286 μs. These lifetimes are substantially longer than the D-A TADF DMAC and tetra­meth­yl­car­ba­zole derivatives, that ranged from 3.0 to 22 μs. The devices with **Cz-DiKTa**, **Cz-Ph-DiKTa**, and **3Cz-DiKTa** showed EQE_max_ of 24.9, 23.0, and 24.4%, respectively, while the efficiency roll-off was moderate with EQE_100_ of 20.4, 19.3, and 17.3%, for the same.

Building upon this work, we reported two new emitters, each with three diphenylamine (**3DPA-DiKTa**, Figure 154) or triphenylamine (**3TPA-DiKTa**, Figure 154) donors about a **DiKTa** core.^165^ In 2 wt% doped mCP films **3TPA-DiKTa** and **3DPA-DiKTa** emit at λ_PL_ of 551 and 617 nm, respectively. The emission spectra are relatively broad, with FWHM of 58 and 56 nm reflecting excited states of mixed LRCT and SRCT character. **3TPA-DiKTa** and **3DPA-DiKTa** have Δ*E*_ST_ of 0.13 and 0.20 eV and τ_d_ of 131 and 323 μs, which translate to *k*_RISC_ of only 0.14 and 2.49 × 10^4^ s^–1^, respectively. Devices with **3TPA-DiKTa** and **3DPA-DiKTa** nonetheless showed EQE_max_ of 30.8 and 16.7%, respectively, at CIE coordinates of (0.41, 0.58) and (0.63, 0.37)–efficiencies amongst the highest reported for carbonyl-containing MR-TADF emitters. The devices suffered from severe roll-off though, with EQE_100_ dropping to 18.1 and 3.4%, likely due to their inefficient *k*_RISC_. HF-OLEDs using **4CzIPN** (Figure 154) as the assistant dopant showed much improved efficiency roll-off, with the EQE_100_ of 27.4 and 8.7%. The exceptional efficiency of the device with **3TPA-DiKTa** was partly attributed to the preferential horizontal alignment of its TDM, which was less pronounced for **3DPA-DiKTa**.

The impact of changing donor strength on photophysical properties of **DiKTa** analogues was also investigated by Liu *et al*.^1091^ In **QAOCz1** carbazole was attached directly to the **QAO** core, while in **QAOCz2** and **QAOCz3** phenyl spacers were set between the **QAO** core and the carbazole donors, with **QAOCz2** containing a *para* disposed carbazole and **QAOCz3** having a *meta* linked carbazole (Figure 154). **QAOCz1**, **QAOCz2**, and **QAOCz3** emit at λ_PL_ of 502, 500, and 492 nm, respectively, in toluene, with the blue-shift trend in emission linked by the authors to a decreasing D-A interaction within the molecules. The Δ*E*_ST_ was similarly reported to decrease from 0.26 to 0.18 and 0.16 eV, respectively. The Φ_PL_ in 5 wt% doped CBP films were 86, 87, and 99%, with the increase attributed to enhanced rigidity across the series. OLEDs with **QAOCz1**, **QAOCz2**, and **QAOCz3** showed EQE_max_ of 16.9, 19.4, and 21.1% respectively, with the increase in line with both the trends in Φ_PL_ and Δ*E*_ST_. All of the devices showed significant efficiency roll-off though, with EQE_1000_ < 3% for each.

Two emitters based on **DiKTa** with charged side groups were presented by us for use in LECs.^166^ One was directly coupled *via* an oxygen bridge to an alkyl-imidazolium ionic group (**DiKTa-OBuIm**, Figure 154), while the other was coupled *via* a diphenylamine donor (**DiKTa-DPA-OBuIm**, Figure 154). In 1 wt% mCP the λ_PL_ were 500 and 578 nm, with Φ_PL_ of 71 and 61% for **DiKTa-OBuIm** and **DiKTa-DPA-OBuIm** respectively. Their FWHM was broad for MR-TADF emitters at 66 and 95 nm, with both oxygen and DPA acting as donating groups. TADF was observed for both emitters in 1 wt% mCP films, with very similar Δ*E*_ST_ of 0.19 and 0.20 eV and slow *k*_RISC_ of 2.9 and 3.0 × 10^3^ s^–1^. Their LEC properties are discussed further in the LEC section.

A dimeric analogue of **DiKTa**, (**DDiKTa**, Figure 154) was reported by us and showed blue-green emission at λ_PL_ 490 nm in 9 wt% doped DPEPO films.^55^ This extended design produced a red-shifted emission and a suppression of the ACQ that is apparent in the parent **DiKTa** (λ_PL_ = 463 nm).^162^ **DDiKTa** has Δ*E*_ST_ of 0.16 eV and a relatively fast τ_d_ of 1.2 μs in the same films.^55^ The activation energy for triplet to singlet up-conversion was even smaller at 0.04 eV, suggestive of the involvement of intermediate triplet states in the RISC process and confirmed by calculations.^83^ Devices showed EQE_max_ of 19.0% at CIE coordinates of (0.18, 0.53), which at the time of publication was only the second example of a green-emitting MR-TADF OLED.^55^ The efficiency roll-off was severe and the OLED could not attain a brightness of 1,000 cd m^–2^.

#### 11.11.2 Exotic QAO Derivatives

Other ketone-based MR-TADF emitters with more elaborate structures have also been developed over time (Figure 155). For example, rigid and planar analogues have been designed that embed one of a DMAC, PXZ, or PTZ groups within the **QAO** skeleton,^1092^ producing **DQAO**, **OQAO**, and **SQAO** (Figure 155). The introduction of O and S atoms resulted in a substantial red-shift and a modest broadening of the emission, with λ_PL_ (FWHM) in toluene of 465 (33), 520 (36), and 552 (54) nm, respectively. **SQAO** also showed a more pronounced positive solvatochromism, suggesting that the excited state contains greater LRCT character than the other two compounds. **DQAO**, **OQAO**, and **SQAO** have similar Δ*E*_ST_ of 0.19, 0.16, and 0.16 eV, while τ_d_ varied more considerably at 111, 205, and 78 μs, with the latter likely shorter due to increased SOC from the heavier sulfur atom. The devices with **DQAO**, **OQAO**, and **SQAO** showed EQE_max_ of 15.2, 20.3, and 17.8%, respectively, at CIE coordinates of (0.12, 0.18), (0.32, 0.65), and (0.47, 0.52). In terms of efficiency roll-off, the EQE_100_ decreased somewhat to 8.5, 15.1, and 13.6%.

**Figure 155:**
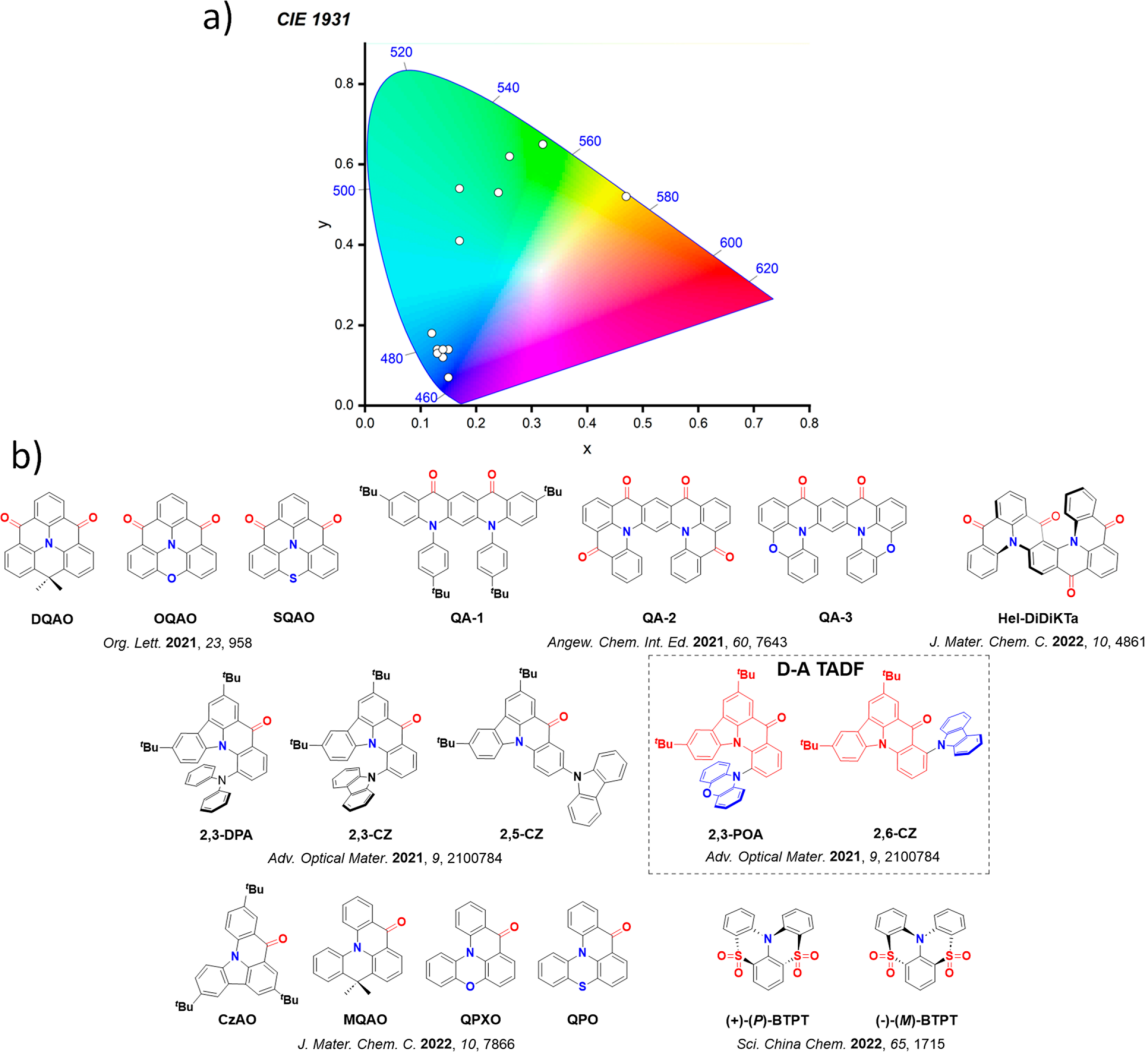
a) CIE color coordinates of OLEDs with “exotic” derivatives of **QAO** and b) structures of reported exotic derivatives of **QAO** and emitters that exhibit LRCT emission and not SRCT emission associated with MR-TADF emitters. The white circles of the CIE diagram illustrate the spread of the emission color of the device. In the chemical structures, the blue color signifies donor moieties/atoms/functional groups, while the red color signifies acceptor moieties/atoms/functional groups.

Yasuda *et al*. reported a family of linearly extended emitters, **QA-1**, **QA-2**, and **QA-3** (Figure 155).^1093^ In 3 wt% doped PPCz films their Δ*E*_ST_ are 0.29, 0.19, and 0.19 eV respectively, with associated τ_d_ of 655, 48, and 307 μs. The long τ_d_ for **QA-1** can be rationalized by its much larger Δ*E*_ST_, while the differences in delayed lifetimes between **QA-2** and **QA-3** were attributed to the presence of intermediate triplet states in **QA-2** that contribute to enhanced *k*_RISC_. **QA-1** and **QA-2** emit at λ_PL_ of 457 and 465 nm, while replacing two of the carbonyl groups with oxygen atoms led to a red-shifted emission at λ_PL_ of 523 nm for **QA-3**. Devices with **QA-1**, **QA-2**, and **QA-3** showed EQE_max_ of 17.1, 19.0, and 16.6% respectively, at CIE coordinates of (0.14, 0.12), (0.13, 0.14), and (0.26, 0.62). The very long τ_d_ for **QA-1** contributed to significant efficiency roll-off of 93% at 100 cd m^–2^, with TTA and STA quenching pathways proposed as the key culprits. The efficiency roll-off was considerable but less severe for both **QA-2** and **QA-3**, at 42 and 40% at 100 cd m^–2^.

A helical isomer of **QA-2**, **Hel-DiDiKTa**, was reported by dos Santos *et al*.^160^ (Figure 155). In 1 wt% doped films in mCP, the compound emits at λ_PL_ of 480 nm, has a Δ*E*_ST_ of 0.15 eV and a τ_d_ of 5.4 μs. However, as the Φ_PL_ is very low at 4.1%, *k*_RISC_ is very slow at 4.1 × 10^2^ s^–1^ and no devices were reported. Its chiroptical properties are discussed in Section 7.

Replacing the ketone functionalities of **QAO** with sulfone moieties produced the near-UV emitter **BTPT** (Figure 155).^645^ In 1 wt% doped PMMA films this compound emits at λ_PL_ of 400 nm with modest FWHM of 56 nm, while its Δ*E*_ST_ is 0.14 eV and it has a τ_d_ of 109 μs. In the crystal this compound shows both RTP and CPL from different enantiomers, although with TADF not apparent. No devices were fabricated.

A series of compounds containing only one carbonyl groups was reported by Luo *et al*.^464^ Of the five compounds in the study, three were demonstrated to be MR-TADF (**2,3-CZ**, **2,5-CZ**, and **2,3-DPA**, Figure 155), while **2,6-CZ** and **2,3-POA** behaved as D-A TADF compounds (Figure 155). MR-TADF was inferred from the smaller FWHM of 36, 41, and 57 nm for **2,3-CZ**, **2,5-CZ**, and **2,3-DPA**, respectively in toluene, compared to 80 and 92 nm for **2,6-CZ** and **2,3-POA** in the same medium. Further, HOMO-LUMO density distribution of **2,3-CZ**, **2,5-CZ**, and **2,3-DPA** each showed patterns reminiscent of MR-TADF. The measured Δ*E*_ST_ in toluene are 0.26, 0.29, and 0.19 eV for **2,3-CZ**, **2,5-CZ**, and **2,3-DPA**, respectively, while τ_d_ are 436, 619, and 373 μs for the same in 3.5 wt% doped mCBP films. **2,3-CZ**, **2,5-CZ**, and **2,3-DPA** emit at λ_PL_ of 449, 459, and 496 nm in toluene and have Φ_PL_ of 40, 81, and 51% in 3.5 wt% doped mCBP films. Shorter delayed lifetimes of 6.2 and 28.1 μs for **2,6-CZ** and **2,3-POA** in the same films were attributed to their much smaller Δ*E*_ST_ of 0.00 and 0.01 eV. Devices of **2,3-CZ** and **2,5-CZ** in mCBP showed EQE_max_ of 6.3 and 22.3% at CIE coordinates of (0.15, 0.14) and (0.13, 0.13). When the EML of the OLED instead consisted of 10 wt% **2,3-CZ** or **2,3-DPA** in 26DCzPPy, the EQE_max_ increased to 8.1 and 11.7% at CIE coordinates of (0.13, 0.15) and (0.17, 0.54).

Huang *et al*.^1094^ investigated a related series of mono-ketone compounds, **CzAO**, **MQAO**, **QPXO**, and **QPO** (Figure 155). A progressive red-shifting of the emission was observed across the series, which emit at λ_PL_ of 431, 447, 485, and 501 nm respectively, while their FWHM also increased from 36 to 61, 76, and 86 nm. Calculations confirmed that this trend in FWHM was mainly due to increasing LRCT content. Large Δ*E*_ST_ of 0.27–0.40 eV led to relatively inefficient TADF, reflected in τ_d_ of 1.0–2.4 ms. Devices with **CzAO**, **MQAO**, **QPXO**, and **QPO** hence showed relatively low EQE_max_ of 8.6, 10.3, 7.1, and 15.3%, respectively.

#### 11.11.3 Tri-ketone Emitters

The tri-ketone fused derivative of **QAO**, **TOAT** (Figure 156), has generated similar levels of attention as a MR-TADF emitter with λ_PL_ of 417 nm and a large Δ*E*_ST_ of 0.34 eV in toluene.^1095^ Previous reports have highlighted the same core (**TANGO**) as an RTP emitter in the crystalline form.^1096^ The addition of peripheral donating groups can alter the nature of the emissive S_1_ state from SRCT to LRCT associated with D-A TADF systems.^1095^

**Figure 156:**
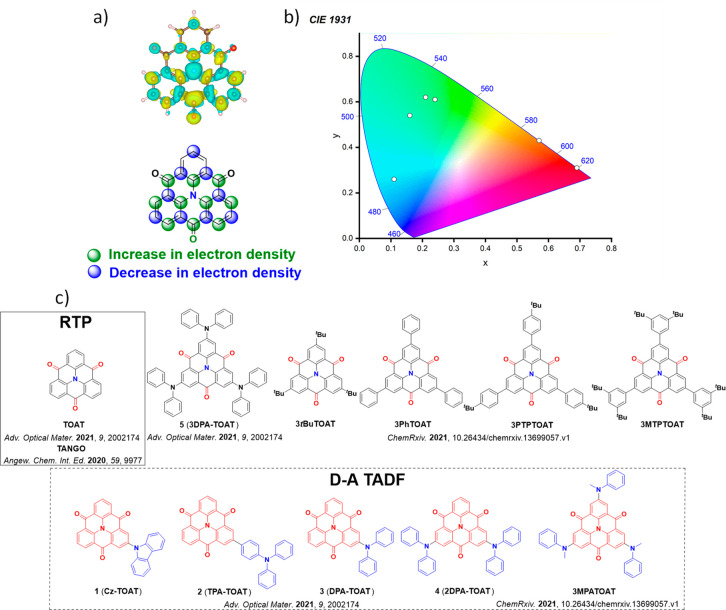
a) Computed difference density plot (top) and the schematic representation of the difference density distribution (bottom) of **TOAT**, b) CIE color coordinates of OLEDs with **TOAT** derivatives, and c) structures of reported **TOAT**-based emitters and emitters which showed D-A emission and not MR-TADF. Difference density plots calculated at the SCS-CC2/cc-pVDZ level in the gas phase; is-value = 0.01. The white circles of the CIE diagram illustrate the spread of the emission color of the device. In the chemical structures, the blue color signifies donor atoms/functional groups, while the red color signifies acceptor atoms/functional groups.

For example, upon addition of one carbazole (originally **1**, here renamed **Cz-TOAT**, Figure 156) and triphenylamine (**2**, here **TPA-TOAT**, Figure 156) the emission broadened, with the authors classifying these materials to be D-A TADF rather than MR-TADF. Increasing the number of DPA units instead induced a narrowing of the emission in **3**, **4**, and **5** (here **DPA-TOAT**, **2DPA-TOAT**, and **3DPA-TOAT**, Figure 156), with FWHM decreasing from 84 to 75 and 45 nm, respectively. It was concluded that **3DPA-TOAT** was indeed MR-TADF while the other species were of D-A character. The emission of **3DPA-TOAT** in toluene is red-shifted at λ_PL_ of 590 nm compared to that of **TOAT** (λ_PL_ of 417 nm), accompanied by an increase in Φ_PL_ from < 1% for **TOAT** to 7% for **3DPA-TOAT**. The Φ_PL_ similarly increased to 46% in 3 wt% doped mCBP films. In toluene the Δ*E*_ST_ of **3DPA-TOAT** is 0.34 eV, identical to that of **TOAT**, which resulted in a long τ_d_ of 2.1 ms and low *k*_RISC_ of 9.3 × 10^2^ s^–1^. Devices with **3DPA-TOAT** showed very low EQE_max_ of 1.2% at CIE coordinates of (0.57, 0.43). Devices with the D-A TADF emitters, **Cz-TOAT**, **TPA-TOAT**, **DPA-TOAT**, and **2DPA-TOAT** showed much improved EQE_max_ of up to 17% for the device with **2TPA-TOAT**. The authors attributed the low efficiency of the device with **3DPA-TOAT** to poor charge balance and undesired host-guest interactions.

A similar series of compounds using the same **TOAT** core but with different substituents (^*t*^Bu, phenyl, *p*-^*t*^Bu-Phenyl, *m*-Di^*t*^Bu-Phenyl, and NMePh in **3*t*BuTOAT**, **3PhTOAT**, **3PTPTOAT**, **3MTPTOAT**, and **3MPATOAT** respectively, Figure 156) was reported by Wang *et al*.^1097^ In toluene **3*t*BuTOAT**, **3PhTOAT**, **3PTPTOAT**, and **3MTPTOAT** emit at λ_PL_ ranging from 439–468 nm and have large Δ*E*_ST_ ranging from 0.30–0.40 eV; a large red-shift in the emission was observed for **3MPATOAT** with λ_PL_ at 580 nm, as this system is a D-A TADF emitter due to the strongly electron-donating NPhMe groups. In doped films large changes in photophysical properties were observed, which the authors attribute to the formation of dimer species. These differences were particularly apparent in the phenyl series of **3PhTOAT**, **3PTPTOAT**, and **3MTPTOAT**. In 15 wt% doped mCP films the emission of **3PhTOAT**, **3PTPTOAT**, and **3MTPTOAT** was red-shifted compared to toluene solution, from 442, 449, and 446 nm to 516, 520, and 502 nm, respectively, with the Φ_PL_ increasing from 16, 23, and 20% to 97, 93, and 92%. The large changes in Φ_PL_ were rationalized in terms of the differences in HOMO-LUMO electron density distributions between the monomolecular and the dimer species. As an isolated species, the S_1_ state has n−π* character, with low oscillator strength, while for the dimeric species the excited state is π–π* with much larger oscillator strength. The Δ*E*_ST_ decreased from 0.32, 0.32, and 0.30 eV in toluene to 0.12, 0.16, and 0.14 eV in the 15 wt% doped mCP films. The devices with **3PhTOAT**, **3PTPTOAT**, and **3MTPTOAT** hence showed EQE_max_ of 29.2, 27.6, and 31.2%, respectively, at CIE coordinates of (0.16, 0.54), (0.24, 0.61), and (0.21, 0.62), compared to the much lower efficiencies of 13.0 and 11.3% for the devices with **3*t*BuTOAT** and **3MPATOAT** at CIE coordinates of (0.11, 0.26) and (0.69, 0.31). Strongly horizontal orientated TDMs of **3PTPTOAT** and **3MTPTOAT** in the film contributed to the high EQE_max_ values in the devices.

### 11.12 Acceptor-Free MR-TADF Emitters

Recently three publications have emerged that demonstrate that MR-TADF compounds need not necessarily contain acceptor groups (Figure 157).^161,167,172^ These reports all centre on diindocarbazole units with *para*-disposed nitrogen atoms, with the differences in structure only extending to the peripheral substituents at present. Patil *et al*. reported **BisICz**, **tBisICz**, and **tPBisICz**, containing no substitution, *tert*-butyl groups, and di-*tert*-butylphenyl substituents, respectively (Figure 157).^172^ **tBisICz** and **tPBisICz** are MR-TADF with long τ_d_ of 12.5 and 1.7 ms, respectively, owing to their large Δ*E*_ST_ of 0.29 and 0.27 eV in 1 wt% doped mCP:​TSPO1 films. High Φ_PL_ of 95 and 91% and narrow blue λ_PL_ of 442 and 450 nm suggested that these two compounds would be promising materials for blue OLEDs. RISC was inferred to proceed *via* a T_2_ state as SOC between S_1_ and T_1_ was calculated to be very small, however both **tBisICz** and **tPBisICz** show inefficient *k*_RISC_ of 0.15 and 1.47 × 10^3^ s^–1^, with the difference in magnitude attributed to a calculated smaller Δ*E*_T2T1_ in **tPBisICz**. Deep blue OLEDs with **tBisICz** and **tPBisICz** showed EQE_max_ of 15.1 and 23.1% at CIE coordinates of (0.16, 0.05) and (0.15, 0.05). However, owing to their inefficient *k*_RISC_, efficiency roll-off was catastrophic with EQE_100_ dropping to 3.0 and 4.8% respectively.

**Figure 157:**
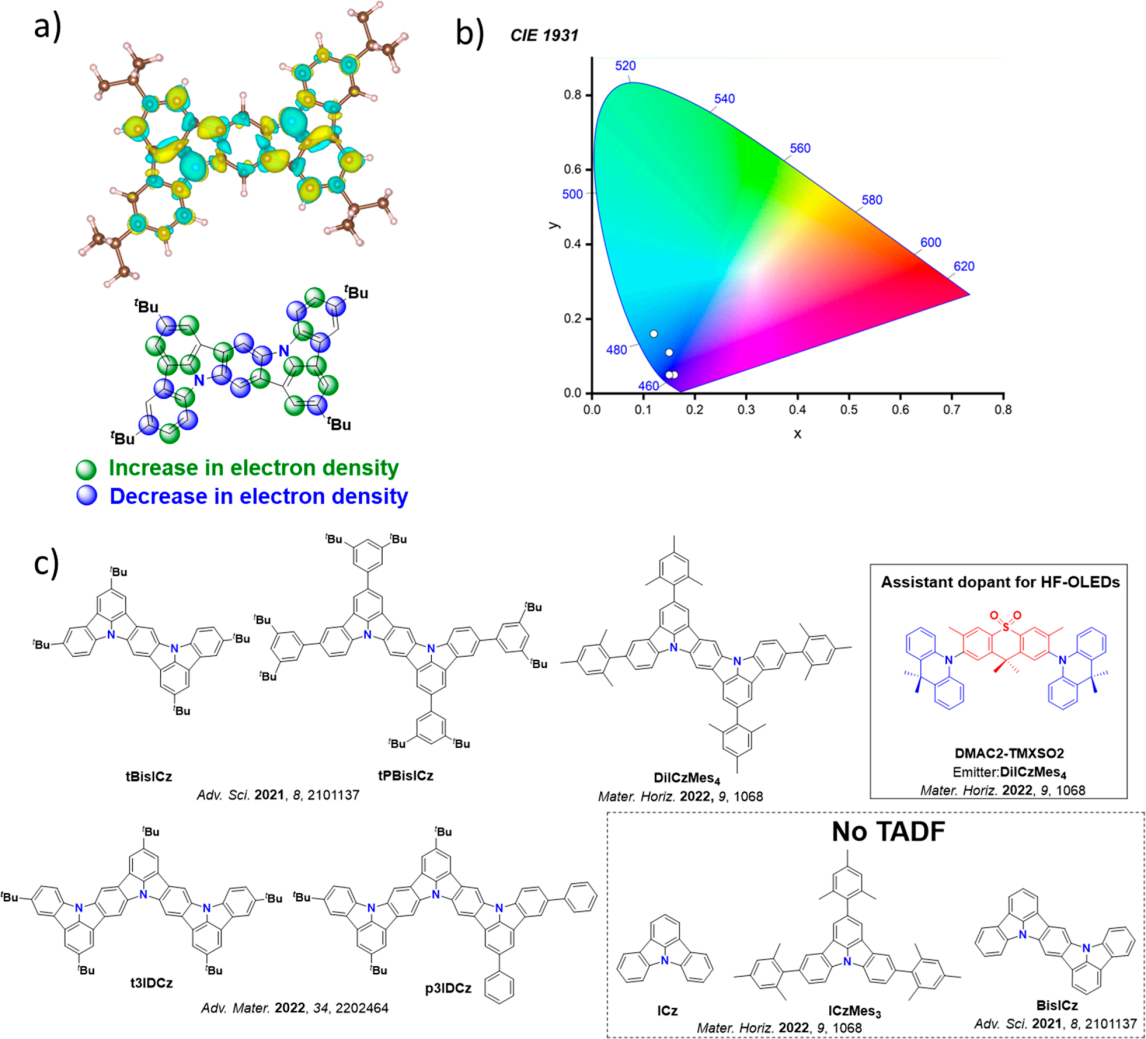
a) Computed difference density plot (top) and the schematic representation of the difference density distribution (bottom) of **tBisICz**, b) CIE color coordinates of OLEDs with acceptor free MR-TADF emitters, and c) structures of reported acceptor free MR-TADF emitters, HF-OLED assistant dopant and derivatives that are not TADF active. Difference density plots calculated at the SCS-CC2/cc-pVDZ level in the gas phase; is-value = 0.01. The white circles of the CIE diagram illustrate the spread of the emission color of the device. In the chemical structures, the blue color signifies donor moieties/atoms/functional groups, while the red color signifies acceptor moieties/atoms/functional groups.

We reported a mesityl-substituted diindolocarbazole as part of a wider study that contrasted the photophysics of **ICz**, **ICzMes_3_**, and **DiICzMes_4_** (Figure 157).^161^ Red-shifted emission and progressively decreasing Δ*E*_ST_ were observed across the series, with λ_PL_ of 374, 387, and 441 nm and Δ*E*_ST_ of 0.47, 0.39, and 0.26 eV in **ICz**, **ICzMes_3_**, and **DiICzMes_4_**, respectively. As predicted computationally, there is an increase in Φ_PL_ across the series from 58 to 66 and 70% in toluene. Owing to their large Δ*E*_ST_, **ICz** and **ICzMes_3_** are not TADF-active; however, when doped in 3 wt% mCP films TADF is apparent in **DiICzMes_4_** with Δ*E*_ST_ of 0.26 eV, τ_d_ of 433 μs, and Φ_PL_ of 82% and a *k*_RISC_ of 1.9 × 10^2^ s^–1^. OLEDs with **DiICzMes_4_** showed an EQE_max_ of only 3.0% at CIE coordinates of (0.15, 0.11), but the device performance was measurably improved in HF-OLEDs using **DMAC2-TMXSO2** (Figure 157) as the assistant dopant where the EQE_max_ increased to 16.5% at the same CIE coordinates.

A further extension of the ICz core with three nitrogen atoms was reported by Lee *et al*.^167^ Emitters **t3IDCz** and **p3IDCz** (Figure 157) were reported, the first with all ^*t*^Bu substituents and the latter with two phenyl substituents. These two larger systems showed a red-shifted emission at λ_PL_ of 470 nm compared to **tBisICz**, (λ_PL_ of 442 nm) in doped films.^172^ The Δ*E*_ST_ are smaller though at 0.21 and 0.19 eV for **t3IDCz** and **p3IDCz**, respectively in THF. Coupled with the high Φ_PL_ of 92 and 100%, *k*_RISC_ in **t3IDCz** and **p3IDCz** reached 0.84 and 1.1 × 10^4^ s^–1^ in 1 wt% doped mCP:​mCBP-1CN films. The OLEDs with **t3IDCz** and **p3IDCz** showed EQE_max_ of 30.0 and 30.9% at CIE coordinates of (0.12, 0.16) for both. Despite the improved *k*_RISC_, efficiency roll-off was still high with EQE_1000_ of just 5.0 and 4.7%.

### 11.13 Outlook

Despite their very recent rise to prominence the efficiencies achievable by MR-TADF devices are already impressive, with EQE_max_ regularly exceeding 30%. We particularly highlight representative, green, and red MR-TADF OLEDs employing **m-DPAcP-BNCz**, and **TCZ-F-DABNA**, with EQE_max_ of 36.1, 42.0, and 39.2%, respectively, while for blue two emitters, ***t*-Bu-*v*-DABNA** and **BN3** have identical EQE_max_ of 36.3% (Figure 158). However, the efficiencies at display-relevant luminances are often undercut by significant efficiency roll-off for these devices, with few reports of MR-TADF OLEDs having EQE_1000_ above 20%. It is unclear at present whether the relatively slow *k*_RISC_ in reported MR-TADF emitters (typically ∼100 times smaller than leading D-A emitters) is an intrinsic limitation, or merely reflective of the limited region of chemical space thus-far explored.

**Figure 158:**
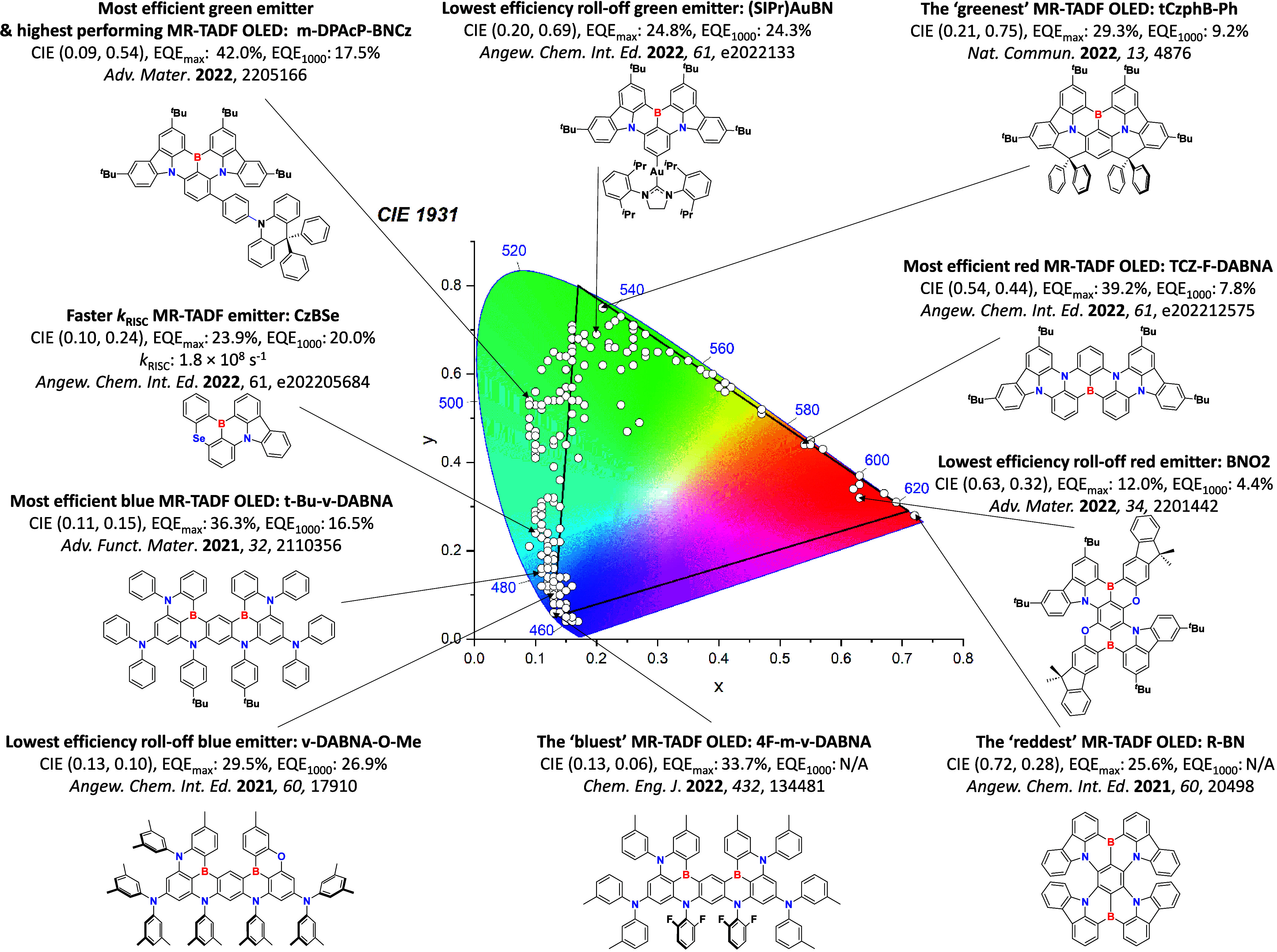
CIE color coordinates of all reported MR-TADF OLEDs. The white circles illustrate the spread of the emission color of the device. Rec. 2020 points are connected by a black line. Selected devices and their associated CIE coordinates represented by gray squares are highlighted, illustrating the structure of the emitter used in the “bluest”, “greenest”, and “reddest” device. Selected devices and their associated CIE coordinates represented by gray triangles are highlighted, illustrating the structure of the emitter of the highest efficiency blue, green, and red OLEDs quantified by the EQE_max_. Selected devices and their associated CIE coordinates represented by gray stars are highlighted, illustrating the structure of the emitter with the fastest *k*_RISC_. The device with the CIE coordinates closest to the Rec. 2020 defined coordinates for blue, (0.131, 0.046), is defined as the “bluest”. The device with the CIE coordinates closest to the Rec. 2020 defined coordinates for green, (0.170, 0.797), is defined as the “greenest”. The device with the CIE coordinates closest to the Rec. 2020 defined coordinates for red, (0.708, 0.292), is defined as the “reddest”. In the chemical structures, the blue color signifies donor atoms/functional groups, while the red color signifies acceptor atoms/functional groups.

Promising current MR-TADF design strategies include extending the π-system as exemplified by ***v*-DABNA** and its derivatives (Section 11.2.4), although with this singular approach reaching an apparent ceiling for *k*_RISC_ of ∼10^5^ s^–1^. Inclusion of heavy sulfur and selenium atoms can also accelerate *k*_RISC_, for example in **CzBSe** (Figure 158) with a reported *k*_RISC_ of 10^8^ s^–1^, surpassing even the most efficient D-A TADF systems. Mechanistically, we note that the role of upper triplet states in mediating RISC for MR-TADF emitters has only recently begun to be appreciated and explored, with further fundamental and computational studies likely to inspire and refine new design insights. In the meantime, the relatively slow RISC of reported MR-TADF emitters is frequently overcome through the use of established D-A assistant dopants in hyperfluorescence devices (Section 17).

In parallel with raw efficiency, while there are now examples of MR-TADF compounds that emit across the entire visible spectrum, challenges and mysteries remain with respect to color tuning. Indeed, one of the key benefits of narrowband MR-TADF emitters is their ability to reach highly saturated color coordinates that are practically inaccessible to D-A CT emitters, for example with red **R-TBN** and blue **4F-m-*v*-DABNA** nearing the Rec. 2020 CIE coordinatesion of (0.13,0.05) and (0.71, 0.29), respectively (Figure 158). **tCzphB-Ph** does provide the greenest MR-TADF emitter, nearing Rec. 2020 for green (0.17, 0.80), however, progress to this color point is still behind the red and blue counterparts with further development needed (Figure 158). The emission color can be tuned by judicious decoration of (or substitution within) the MR-TADF core. On a fundamental level, mixing of some LRCT character into the SRCT emissive excited state can both tune the color (most commonly to the red) and increase RISC, albeit at the expense of a somewhat broadened emission.^150^ Here we note that wavefunction-based computational methods, recently demonstrated to be necessary for the accurate modelling of MR-TADF excited-state energies (Section 2), will become more popular in the coming years to inform molecular design regarding color tuning. This will be a particularly welcome development, as outside the visible spectrum there exist precious few near-UV and near-IR MR-TADF emitters, with potential for such materials to advance significant applications in, for instance, sensing, security, and (bio)imaging.

Lastly, we note that most of the OLEDs summarized in this section were fabricated by vacuum deposition. Owing to their planar structures and strong propensity to aggregate, MR-TADF OLEDs typically require evaporative doping fabrication at low emitter concentration (<5 wt%) to retain high device performance. Nonetheless, recent works using polymers or very bulky derivatives have sought to mitigate this issue and there are now examples of bright, narrowband MR-TADF emitter films at doping concentrations exceeding 40 wt%. Similar strategies can also enable MR-TADF emitters to be solution processable, and there are now a small but increasing number of reports of solution-processed MR-TADF OLEDs. Mirroring earlier developments for D-A TADF emitters, we now also see diversification in the properties and uses of MR-TADF materials, including with chiral centers and CPL emission (Section 7), in LECs (Section 16), organic lasing (Section 22), and as photocatalysts (Section 23). Ultimately MR-TADF materials – acting either as hosts, emitters, or otherwise – are an exciting class of compounds with potential yet to be fully realized.

## 12 Through-Space Charge Transfer (TSCT) Interactions in TADF

### 12.1 Introduction

Arguably the fundamental criteria for designing effective and efficient TADF OLED materials is to realize both fast *k*_RISC_, necessitating a small Δ*E*_ST_, and large Φ_PL_. The former requires a small exchange integral, while the latter relies on high oscillator strength for the excited states involved in emission. Reflecting the vast majority of reported TADF emitters, Sections 2–7 highlight examples of highly twisted donor-acceptor molecular designs to achieve a small Δ*E*_ST_, forming emissive charge transfer states with “through bond” electronic conjugation through the π-network.

An alternative strategy to achieve weak electronic coupling between donor and acceptor motifs is to exploit “through-space” (TS) conjugation, where the π-systems of donor and acceptor moieties are aligned and interact without direct covalent bonding. Similar to TADF exciplex blends of separate donor and acceptor molecules (see Section 8), molecular scaffolding can be used to controllably engineer through-space charge transfer (TSCT) states that translate into molecules with small Δ*E*_ST_. There are also a small number of compounds where donor and acceptor groups are electronically coupled through homoconjugated linkers, which also leads to a small Δ*E*_ST._ The number of reported TSCT TADF molecules has increased rapidly, especially so since 2020, offering examples with fast *k*_RISC_ and outstanding OLED performance. In this section we summarise recent developments in the design and understanding of TSCT materials, categorized based on the structural units used as scaffolds to mediate the interaction of the donor and acceptor groups.

### 12.2 TSCT Featuring Non-conjugated Bridges

Many TSCT TADF emitters are constructed using a non-conjugated bridge to hold donor and acceptor subunits in a co-facial arrangement. Tsujimoto *et al*. were the first to explore this concept in a series of compounds containing a xanthene bridge that co-orients a triazine acceptor with donor units (phenothiazine for **XPT**, carbazole for **XCT**, or *tert*-butyl-carbazole for **XtBuCT**, Figure 159).^1098^ The distances between donor and acceptor were 3.3–3.5 Å, allowing TSCT states to exist. With increasing donor strength, a progressive red-shift in the emission was observed for **XCT**, **XtBuCT**, and **XPT** (λ_PL_ of 419 to 451 and 562 nm, respectively, in toluene). When doped at 10 wt% in DPEPO the emission of **XtBuCT** and **XPT** red-shift slightly to 453 and 566 nm, with Φ_PL_ of 35 and 66%, and τ_d_ of 4.1 and 3.3 μs, respectively (no Δ*E*_ST_ values were reported). The resulting OLEDs emitted at λ_EL_ of 488 and 584 nm for **XtBuCT** and **XPT**, and showed a modest EQE_max_ of 4 and 10%, respectively.

**Figure 159:**
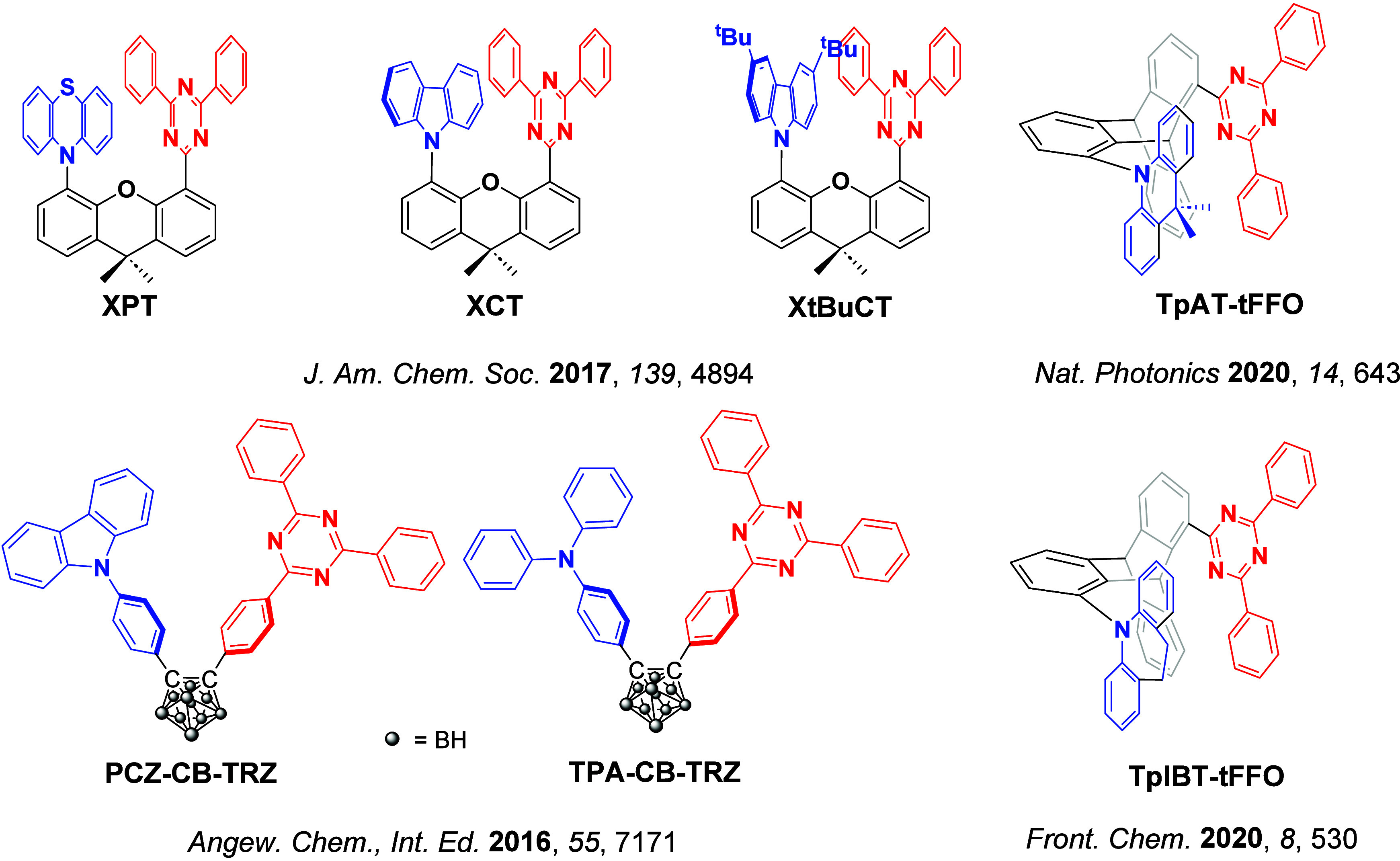
Structures of TSCT TADF emitters containing non-conjugated bridges (the blue color signifies donor moieties, while the red color signifies acceptor moieties).

*o*-Carboranes are another group that can bridge donor and acceptor to achieve TSCT.^1099^ Two emitters, **PCZ-CB-TRZ** and **TPA-CB-TRZ** (Figure 159), displayed small DFT-calculated Δ*E*_ST_ of 0.003 and 0.018 eV, respectively. The emitters also showed AIE (see Section 13 for more discussion), exhibiting yellow emission with λ_PL_ of 557 and 571 nm and having Φ_PL_ of 97 and 94% in neat film, all respectively. The non-doped OLEDs with **PCZ-CB-TRZ** and **TPA-CB-TRZ** emitted at λ_EL_ of 631 and 586 nm, and showed EQE_max_ of 11 and 9.2%, respectively. These EQE_max_ are still lower than what might be expected from the high Φ_PL_, suggesting further optimization in the device structure is needed.

Wada *et al*. reported three ‘tilted face-to-face with optimal distance’ (tFFO) TADF emitters, **TpAT-tFFO**, **TpMAT-tFFO**, and **TpPXT-tFFO** (Figure 159).^98^ Near-degenerate ^1^CT, ^3^CT, and ^3^LE states were realized by controlling the distance and orientation between the donors and acceptors using a triptycene scaffold, and large spin–orbit coupling values were realized with the donors and acceptors not perfectly co-facially oriented. With this strategy the three emitters all showed very fast *k*_RISC_; for example, **TpAT-tFFO** has a remarkably fast *k*_RISC_ of 1.2×10^7^ s^–1^ alongside a high Φ_PL_ of 76% in 25 wt% doped films in mCBP. An OLED with **TpAT-tFFO** showed blue emission at λ_EL_ of 462 nm with an EQE_max_ of 19.2%. The same group further exploited this strategy using 10,11-dihydro-5H-dibenzo[*b, f*]azepine (IB) as the donor, giving the structure **TpIBT-TFFO**.^1100^ This compound also has a very fast *k*_RISC_ of 6.9 × 10^6^ s^–1^, emits at λ_PL_ at 477 nm, and has a Δ*E*_ST_ of 0.076 eV in toluene, while the Φ_PL_ is 71.4% and the τ_d_ is 6.7 μs in 9 wt% doped films in CzSi (Table S16). A device with **TpIBT-TFFO** showed an EQE_max_ of 12.2%, emitting at λ_EL_ of 462 nm, with CIE coordinates of (0.16, 0.26).

### 12.3 TSCT Featuring Spiro-fluorene Bridges

Spiro-fluorenes can also be used to build TSCT skeletons, taking advantage of the perpendicular attachment point offered by the spiro centre. Attaching the donor or acceptor at the spiro position also leads to rigid structures with limited vibrational flexibility, thereby decreasing non-radiative decay and resulting in narrower emission bands. Tang *et al*. developed a series of pseudo co-facial TSCT emitters, **DM-B**, **DM-Bm**, and **DM-G** (Figure 160), where the spacing and relative orientation of the donor and acceptor subunits were controlled by using a rigid spiro-fluorene as a linker.^1101^ Using this approach, ground-state electronic coupling was strengthened, and non-radiative decay channels were suppressed. The resulting molecules **DM-B**, **DM-Bm**, and **DM-G** emit at 493, 495, and 504 nm, have short τ_d_ of 5.0, 4.5, and 3.3 μs, high Φ_PL_ of 96, 92, and 88%, and have reported Δ*E*_ST_ of 0.17, −0.08, and −0.11 eV in 20 wt% doped films in DPEPO. These apparent inverted singlet-triplet gaps likely reflect an energy gap between singlet and triplet states of different species. The OLEDs with **DM-B**, **DM-Bm**, and **DM-G** showed EQE_max_ of 27.4, 21.7, and 18.5% and low efficiency roll-off of only 10.9, 9.2 and 16.8% at 1000 cd m^–2^, at CIE coordinates of (0.20, 0.44), (0.22, 0.48), and (0.24, 0.50), respectively.

**Figure 160:**
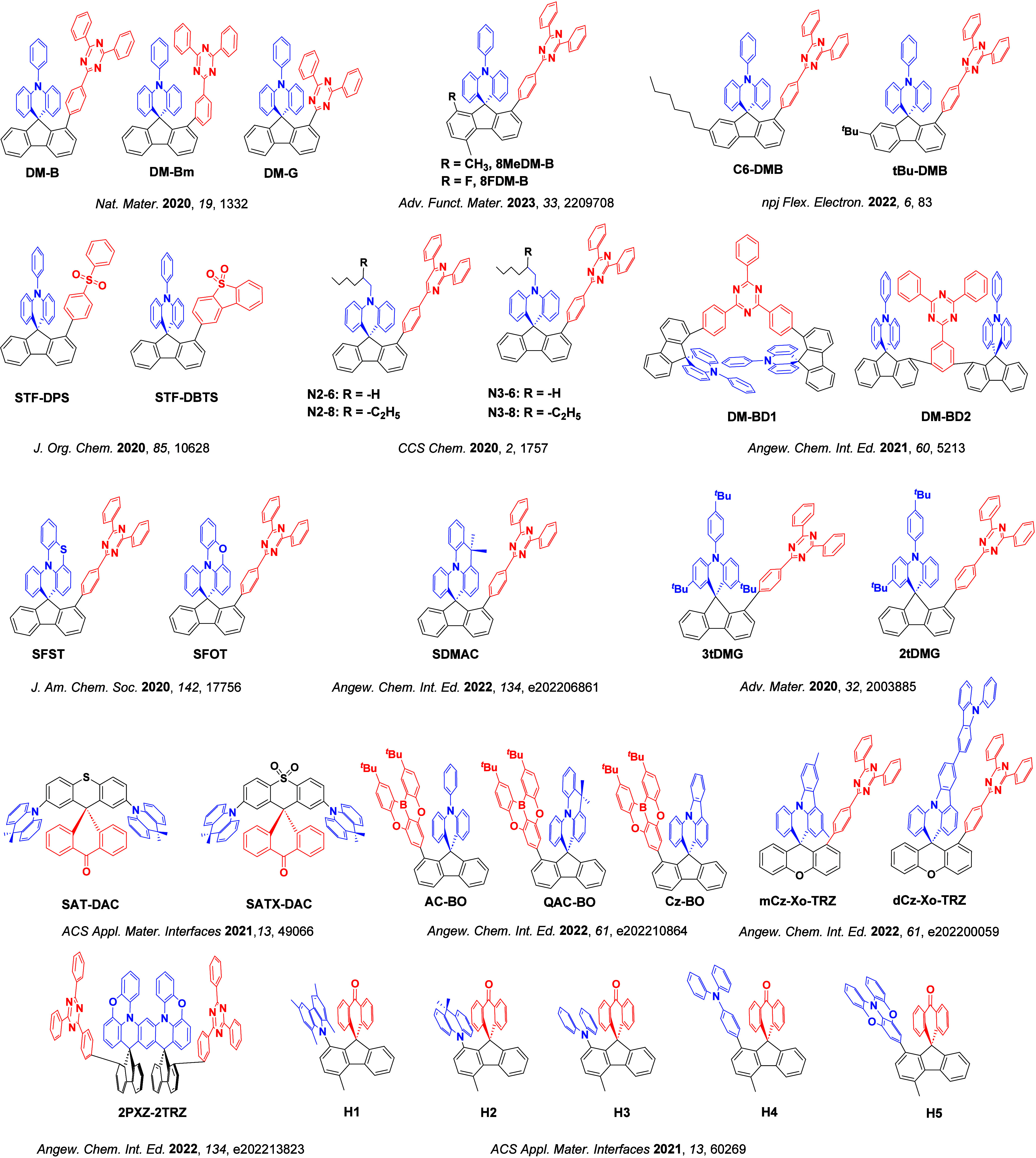
Structures of TSCT TADF emitters containing spiro-fluorene bridges (the blue color signifies donor moieties, while the red color signifies acceptor moieties).

The same group subsequently reported two similar emitters, **8MeDM-B** and **8FDM-B** (Figure 160), with methyl or fluorine groups substituted at the C8 site of the spiro-fluorene bridge.^1102^ Interestingly, the presence of fluorine atoms gave stronger electrostatic repulsion than the methyl group, distorting the TPA unit away from the C8 position. Therefore, compared to **8MeDM-B**, **8FDM-B** has an additional interaction between the D and A groups and the acceptor adopts a more planar conformation. According to their DFT study, the HOMOs and LUMOs of both compounds are primarily located on the respective donors and acceptors, whereas there is nearly no electron density on the fluorene bridge. **8FDM-B** emission is slightly red-shifted compared to **8MeDM-B** (λ_PL_ of 483 and 480 nm in toluene), which was attributed to stronger charge transfer due to the shorter donor-acceptor distance. Both compounds have a Δ*E*_ST_ of 0.15 eV in toluene glass at 77 K. In a 30 wt% doped PPF matrix, **8MeDM-B** and **8FDM-B** showed similar τ_d_ of 4.6 and 4.0 μs and very high Φ_PL_ of 97 and 98%, respectively. The photophysical properties of **8MeDM-B** and **8FDM-B** supported exceptional device performance, with high EQE_max_ of 28.8 and 31.7%, and similar λ_EL_ of 492 and 496 nm, respectively. Both compounds exhibited similar efficiency roll-off of around 17.7% at 1000 cd m^–2^.

Zheng *et al*. introduced solubilizing *tert*-butyl and *n*-hexyl groups at the C7 position of the spiro-fluorene core to construct **C6-DMB** and **tBu-DMB** (Figure 160).^1103^ The solubilizing substituents had minimal influence on the photophysical properties of the parent emitter **DM-B**, with λ_PL_ of 447 and 446 nm and the same Δ*E*_ST_ of 0.17 eV in toluene. Both emitters exhibited short τ_d_ of 5.99 and 5.58 μs, and high Φ_PL_ of 89 and 98% in 30 wt% doped films in mCP, respectively. Solution-processed OLEDs with **C6-DMB** and **tBu-DMB** showed EQE_max_ of 21.0 and 21.7%, and the same CIE coordinates of (0.21, 0.38). Both devices, however, also showed severe efficiency roll-off of 60 and 63% at 1000 cd m^–2^.

Following this report, the same group developed dibenzothiophene sulfone as an acceptor to construct the TSCT emitter **STF-DBTS** (Figure 160).^1104^ A more conformationally flexible acceptor, diphenylsulfone, was also included in reference emitter **STF-DPS**. **STF-DBTS** emits at λ_PL_ of 460 nm, has a rather large Δ*E*_ST_ of 0.27 eV in toluene, a Φ_PL_ of 53%, and yet a τ_d_ of only 24.3 μs in 30 wt% doped films in CBP. This contrasts with **STF-DPS** which did not show TADF and has a low Φ_PL_ of 16.0%, and shows a blue-shifted λ_PL_ at 441 nm in toluene. The rigid structure of **STF-DBTS** gave a shorter distance between the donor and acceptor (3.586 Å), which allowed for a more effective TSCT state to form than in **STF-DPS**, with a donor-acceptor separation of 3.752 Å. OLEDs with **STF-DBTS** displayed sky-blue emission at λ_EL_ of 488 nm and achieved an EQE_max_ of 10.3%.

Using the same spiro-fluorene scaffold, the same group developed four additional emitters containing alkyl chains of different lengths, **N2-6**, **N3-6**, **N2-8**, **and N3-8**.^1105^ The different alkyl chains, *n*-hexyl (noted in emitter name with a −6) and 2-ethylhexyl (noted with a −8), were introduced to modulate the donor-acceptor distance and to improve the solubility of the emitters for solution-processed OLEDs. Different acceptors 2,4,6-triphenylpyrimidine (N2) and 2,4,6-triphenyl-1,3,5-triazine (N3) were also compared. The emitters **N2-6** and **N2-8** have slightly blue-shifted emission (λ_PL_ of 461 and 470 nm, respectively) compared with **N3-6** and **N3-8** (λ_PL_ of 485 and 495 nm) in toluene solution due to the weaker electron-withdrawing ability of N2 group. The Δ*E*_ST_ of **N2-6**, **N2-8**, **N3-6**, and **N3-8** are 0.27, 0.16, 0.18, and 0.14 eV respectively, revealing that shorter donor-acceptor distance can be beneficial for narrowing the Δ*E*_ST_. Also attributed to the short donor-acceptor distances, **N2-8** and **N3-8** have higher Φ_PL_ (82 and 91%) than those of **N2-6** and **N3-6** (76 and 83%). All emitters showed very short τ_d_: 1.01 μs for **N2-6**, 1.18 μs for **N2-8**, 1.29 μs for **N3-6**, and 1.50 μs for **N3-8**. Solution-processed OLEDs with **N3-8** (λ_EL_ of 488 nm) and **N2-8** (λ_EL_ of 479 nm) consequently showed superior device performances with EQE_max_ of 18.9 and 17.6%, relative to 14.2 and 14.7% for devices based on **N2-6** (λ_EL_ of 480 nm) and **N3-6** (λ_EL_ of 490 nm), respectively.

Wang *et al.* later refined their emitter design further, reporting sandwich type TSCT D-A-D systems **DM-BD1** and **DM-BD2** (Figure 160).^1106^ These compounds contain a multi-layer π-stacked arrangement that spatially confines the central acceptor between one or two peripheral donor groups. **DM-BD1** possesses a bilayer structure with both donor groups on the same side of the acceptor, while **DM-BD2** has a tri-layer structure. The congested geometry in each of the two emitters results in a short distance between the donor and acceptor units of 3.11 and 3.05 Å for **DM-BD1** and **DM-BD2**, respectively, and the same λ_PL_ at around 495 nm in toluene. Similar Φ_PL_ of 94.2 and 92.8% and τ_d_ of 3.1 and 2.8 μs were reported in 30 wt% doped films in DPEPO. OLEDs with **DM-BD1** and **DM-BD2** exhibited λ_EL_ at around 500 nm with CIE coordinates of (0.21, 0.47) and (0.20, 0.46), and achieved EQE_max_ of 28.0 and 26.6%, while the EQE_1000_ decreased to 18.9 and 15.8%, respectively.

Chiral emitters **SFST** and **SFOT** were reported by the same group using a similar spiro-skeleton containing an sp^3^-hybridized spiro carbon (Figure 160).^1107^ Sulfur and oxygen atoms were introduced into the donor to tune the photophysical and chiroptical properties (see Section 7). Compared with **SFOT**, the larger sulfur atom in **SFST** resulted in enhanced SOC and led to a distortion of the molecular backbone that lengthened the donor-acceptor distance, resulting in a lower Φ_PL_ and faster non-radiative decay. **SFST** and **SFOT** both emit at λ_PL_ of around 512 nm, have small Δ*E*_ST_ of 0.052 and 0.053 eV in toluene, and Φ_PL_ of 53.1 and 89.7% in 30 wt% doped films in mCBP, respectively. The OLEDs with **SFST** and **SFOT** both emitted at λ_EL_ of 508 nm and showed EQE_max_ of 12.5 and 23.1%, reflecting their differing Φ_PL_. The devices also showed low efficiency roll-off of 9.6 and 7.8% at 1000 cd m^–2^, respectively.

Yang *et al*. replaced the oxygen atom with a bridging Me_2_C group in the multi-stimulus response-active emitter **SDMAC** (Figure 160).^1108^ **SDMAC** exhibited aggregation-induced emission enhancement (AIEE), solvatochromism, piezochromism, and CPL under different external stimuli. **SDMAC** emits in the sky-blue at λ_PL_ of 468 nm and has a small Δ*E*_ST_ of 0.034 eV in toluene. In 30 wt% doped films in PPF the Φ_PL_ is 90% and the τ_d_ is 4.17 μs, leading to *k*_RISC_ of 1.94×10^–5^ s^–1^. The device with **SDMAC** showed an EQE_max_ of 28.4%, with λ_EL_ of 492 nm and CIE coordinates of (0.18, 0.41). The same group reported two other derivatives using the same backbone, **2tDMG** and **3tDM*G*** (Figure 160).^1109^ *t*-Butyl groups were introduced at different positions and in different numbers to improve the emitter solubility. Both compounds emit similarly at λ_PL_ of 502 and 505 nm and have Δ*E*_ST_ of 0.03 and 0.01 eV in toluene, all respectively. In 40 wt% doped films in DPEPO, **2tDMG** and **3tDMG** have τ_d_ of 3.43 and 2.28 μs. The OLEDs with **2tDMG** and **3tDMG** exhibited λ_EL_ at 504 and 518 nm and showed EQE_max_ of 30.8 and 26.3%, all respectively. Notably, the respective EQE_1000_ remained high at 28.5 and 23.2%.

Using an unconjugated spiro-anthrone backbone as the acceptor and DMAC as the donor, Wang *et al.* reported emitters **SAT-DAC** and **SATX-DAC** (Figure 160).^470^ With the ketone as the primary accepting unit both inter- (exciplex) and intramolecular (TSCT) excited states were inferred from close contacts revealed in the X-ray diffraction studies, with **SAT-DCA** and **SATX-DAC** emitting at λ_PL_ of 510 and 517 nm in toluene and having Φ_PL_ of 76.8 and 68.1% in 10 wt% doped DPEPO films in DPEPO, respectively. In 1 wt% doped PMMA films in PMMA, both emitters have the same small Δ*E*_ST_ of 0.02 eV. The OLEDs with these two emitters showed green emission with λ_EL_ of 520 and 524 nm and high EQE_max_ of 22.6 and 20.9%. The EQE_1000_ also remained high at 17.9 and 17.0%.

Zhao *et al*. reported three blue emitters by combining a spiro-fluorene skeleton with a boron/oxygen heterocycle acceptor (BO, aka DOBNA).^1110^ To improve the rigidity of the donor unit in **AC-BO**, the conformation of the amine donor was locked using either with a single bond (**Cz-BO**) or Me_2_C (**QAC-BO**, Figure 160). Thus, the minimum donor-acceptor distance could be tuned from 3.1 Å in **AC-BO**, to 3.0 Å in **Cz-BO**, and 2.6 Å in **QAC-BO**, which enabled progressively stronger π-orbital overlap between the donor and acceptor moieties leading to higher Φ_PL_. **AC-BO**, **QAC-BO**, and **Cz-BO** emit at λ_PL_ of 446, 428, and 411 nm, and show Φ_PL_ of 76.9, 82.8, and 88.7%, respectively in 10 wt% doped films in PMMA (Table S16). However, the Δ*E*_ST_ (in toluene) also increased from 0.13 eV in **AC-BO** to 0.20 and 0.29 eV for **QAC-BO** and **Cz-BO**. The largest Δ*E*_ST_ of **Cz-BO** hindered RISC and this compound did not show TADF. **AC-BO** has a τ_d_ of 11.7 μs, while **QAC-BO** showed a surprisingly much shorter τ_d_ of 0.11 μs. Benefiting from both its high Φ_PL_ and very short τ_d_, **QAC-BO** showed a very high *k*_RISC_ of 1.6 × 10^7^ s^–1^ – almost two orders of magnitudes larger than for **AC-BO** (2.3×10^5^ s^–1^). Even though the emitters exhibited impressive photophysical properties, the device with **QAC-BO** showed an EQE_max_ of only 15.8% and serious efficiency roll-off of 70% at 100 cd m^–2^, which likely implies that the reported τ_d_ is not accurate. The device showed emission at λ_EL_ of 448 nm and with CIE coordinates of (0.145, 0.076). As a purely fluorescent dopant, the device with **Cz-BO** showed an EQE_max_ of 5.5% at λ_EL_ of 412 nm with CIE coordinates of (0.163, 0.034). The device with **AC-BO** showed the highest EQE_max_ of 19.3% with λ_EL_ of 456 nm and CIE coordinates of (0.148, 0.122).

Based on a spiro-xanthene bridging unit, Huang *et al*. reported the TSCT emitters **mCz-Xo-TRZ** and **dCz-Xo-TRZ** (Figure 160).^1111^ In these compounds the triazine acceptor is almost perpendicular to the xanthene bridge and co-planar with the triphenylamine donor, resulting in short donor-acceptor distances in the range of 2.7 to 3.3 Å for **mCz-Xo-TRZ** and 2.8 to 3.3 Å for **dCz-Xo-TRZ**. **dCz-Xo-TRZ** emits at λ_PL_ of 461 nm in toluene, which is red-shifted compared to **mCz-Xo-TRZ** (λ_PL_ of 456 nm) due to the stronger electron-donating ability of the dCz group compared to the mCz donor. **mCz-Xo-TRZ** and **dCz-Xo-TRZ** have Δ*E*_ST_ of 0.16 and 0.24 eV, with τ_d_ of 7.2 and 7.5 μs and high Φ_PL_ of 90 and 92%, respectively in 30 wt% doped films in PPT. Devices with **mCz-Xo-TRZ** and **dCz-Xo-TRZ** showed EQE_max_ of 21.0 and 27.8% at λ_EL_ of 464 and 477 nm, with CIE coordinates of (0.15, 0.20) and (0.16, 0.29), all respectively. Crucially, low efficiency roll-off was observed with EQE_1000_ of 17.1 and 23.9% for the two devices.

Xie *et al*. reported the TSCT material **2PXZ-2TRZ**, involving the linkage of a central biphenoxazine (2PXZ) donor and two triazine acceptors across two spiro-fluorene bridges in a so called “twin-locking” strategy (Figure 160).^1112^ This design efficiently suppresses intramolecular rotations and vibrations, and **2PXZ-2TRZ** emits at λ_PL_ of 509 nm with a small Δ*E*_ST_ of 0.01 eV as the neat film. Due to its small Δ*E*_ST_, **2PXZ-2TRZ** has a τ_d_ of 5.3 μs and a high Φ_PL_ of 94% in 30 wt% doped films in PPF. The doped and non-doped OLEDs showed EQE_max_ of 27.1 and 10.2%, at λ_EL_ of 508 and 518 nm with CIE coordinates of (0.26, 0.54) and (0.31, 0.58), all respectively. The EQE of the doped device decreased to 18.4% at 500 cd m^–2^, representing a rather sever efficiency roll-off.

Song *et al*. used spirofluorene-linked benzophenone as the acceptor unit and installed different donors at the C1 position of the fluorene to obtain emitters **H1**, **H2**, **H3**, **H4**, and **H5** (Figure 160).^1113^ The single crystal X-ray structures of **H1**–**H5** have donor-acceptor distances ranging from 3.3–3.8 Å, indicative of strong face-to-face π-π stacking interactions. The compounds emit with λ_PL_ of 493–550 nm, have Φ_PL_ ranging from 55–92%, τ_d_ ranging from 3.3–6.8 μs, and Δ*E*_ST_ all smaller than 0.07 eV. OLEDs with **H1**–**5** showed sky-blue to yellow emission with λ_EL_ of 494, 527, 503, 507, and 550 nm and CIE coordinates of (0.20, 0.42), (0.31, 0.56), (0.22, 0.48), (0.24, 0.50), and (0.41, 0.55), and with EQE_max_ of 20.9, 16.1, 17.7, 20.0, and 13.2%, all respectively. The EQE_1000_ remained as high as 13.7, 13.5, 13.3, 15.6, and 11.7% for devices with **H1**–**5**, demonstrating the versatility of this kind of molecular design towards different donor groups.

### 12.4 TSCT Featuring Carbazole Bridges

Carbazole and its derivatives have somewhat similar molecular structures to fluorene and have thus similarly been used as scaffolds for TSCT emitters. Moreover, the C1, C8, and N9 positions of carbazole are chemically accessible to decorate, allowing for facile syntheses of a diverse range of targets. It should be noted though that the donating ability of carbazole can in some cases provide a competing through-bond CT state, and the single C-N linkage is significantly more vibrationally active than the spiro linkages in the previous fluorene examples.

Wu *et al*. linked donor and acceptor units *via* a carbazole bridge to construct the TSCT emitters **PXZ-CTZ**, **DPXZ-CTZ**, and **DPXZ-BO** (Figure 161).^1114^ To explore the changes in the photophysical properties as a function of donor and acceptor structure, the donor was varied from PXZ to DPXZ and the acceptor was varied from CTZ to BO moieties. Through this modification the D-A conformations were tuned from orthogonal **(PXZ-CTZ)** to co-facial (**DPXZ-BO**), leading to closer π-π stacking in **DPXZ-BO** and suppressing non-radiative decay. **PXZ-CTZ**, **DPXZ-CTZ**, and **DPXZ-BO** emit at λ_PL_ at 525, 524, and 511 nm in toluene. In 20 wt% doped films in DPEPO, these emitters have Φ_PL_ of 55, 78, and 99%, and small Δ*E*_ST_ of 0.07, −0.03, and 0.03 eV, with short τ_d_ of 3.41, 3.38 and 11.3 μs, all respectively. The apparent negative Δ*E*_ST_ of **DPXZ-CTZ** is likely the result of spectroscopic measurements of different conformers in its fully relaxed singlet and triplet. OLEDs with **PXZ-CTZ**, **DPXZ-CTZ**, and **DPXZ-BO** showed similar green emission at λ_EL_ of ca. 528, 530, and 537 nm with CIE coordinates of (0.33, 0.56), (0.39, 0.57) and (0.26, 0.58), and EQE_max_ of 16.6, 19.7, and 24.0%, all respectively. For **DPXZ-BO** the EQE_1000_ remained above 20%, showing a small efficiency roll-off of 16%.

**Figure 161:**
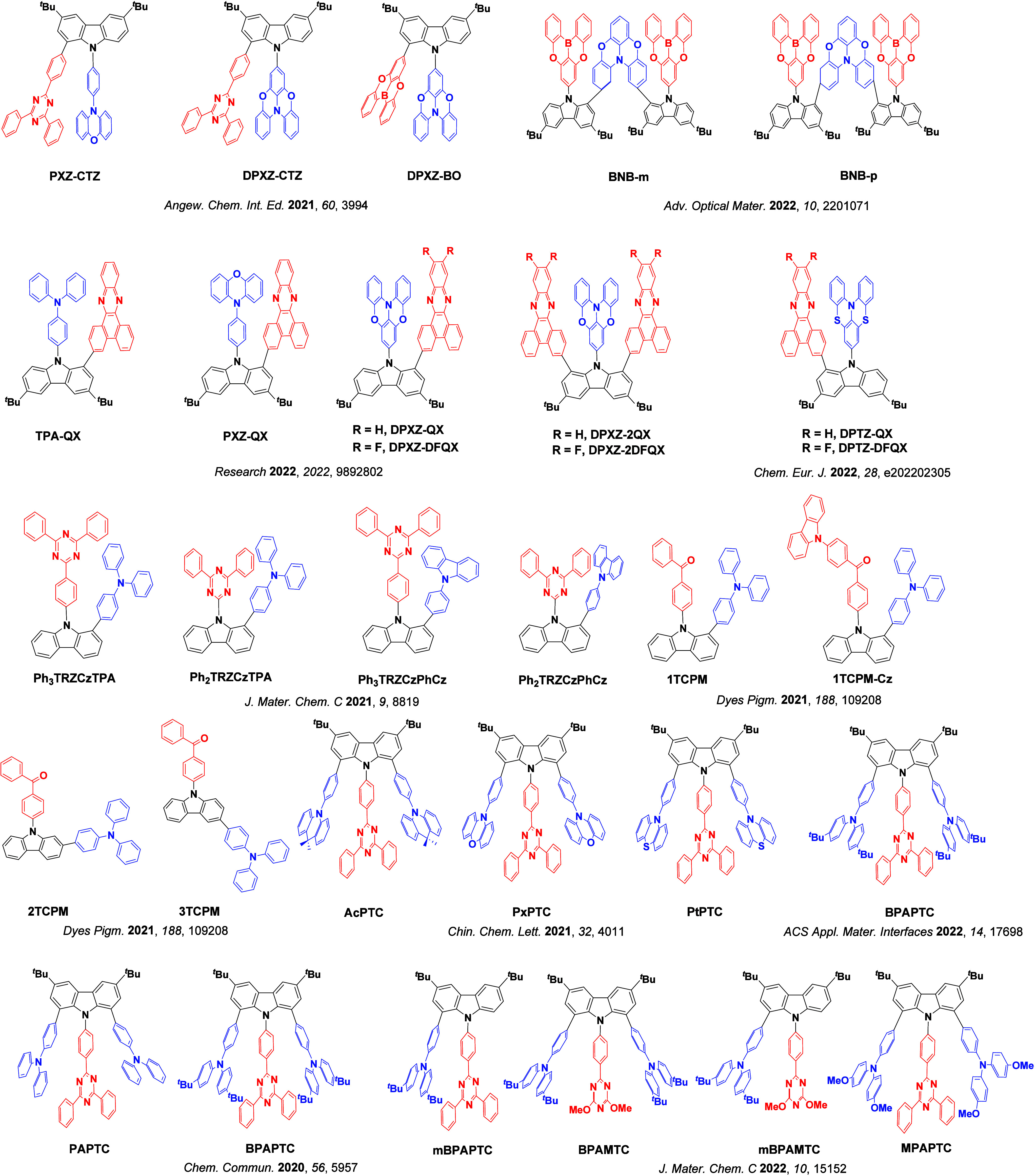
Structures of TSCT emitters containing a carbazole bridge (the blue color signifies donor moieties, while the red color signifies acceptor moieties).

Using a similar strategy Wang *et al*. reported two sandwich-type derivatives, **BNB-m** and **BNB-p** (Figure 161), containing a planar DPXZ donor connected through carbazole groups to two BO acceptors at either the *meta*- or *para*- position of DPXZ unit.^1115^ **BNB-m** and **BNB-p** emit at λ_PL_ of 502 and 518 nm and have high Φ_PL_ of 100 and 86%, respectively in 10 wt% doped films in mCP. The Δ*E*_ST_ are 0.03 and 0.11 eV, with τ_d_ of 11.2 and 25.4 μs and *k*_RISC_ of 16.0 and 9.88×10^4^ s^–1^, also respectively. The OLED with **BNB-m** showed green emission at λ_EL_ of 508 nm and CIE coordinates of (0.23, 0.54), and an outstanding EQE_max_ of 34.9% and EQE_1000_ of 27.4%.

The same group also used carbazole as a bridge to investigate a combinatorial series of TSCT emitters featuring three new donor and two new acceptor groups (**TPA-QX**, **PXZ-QX**, **DPXZ-QX**, **DPXZ-DFQX**, **DPXZ-2QX**, and **DPXZ-2DFQX**, Figure 161).^1116^ By increasing the electron-donating ability of the donor unit, the emission could be red-shifted from 535 nm for **TPA-QX** to 582 nm for **DPXZ-QX** (in 5 wt% doped films in mCP), which was accompanied by an increase in the Φ_PL_ from 44 to 74% and a decrease in the Δ*E*_ST_ from 0.38 to 0.01 eV, therefore leading to a shorter τ_d_ of 6.8 μs for **TPA-QX** compared to 26.9 μs for **DPXZ-QX**. Modulation of the acceptor strength likewise increased the *k*_RISC_ to 4.33×10^5^ s^–1^ for **DPXZ-DFQX** compared to 1.86×10^5^ s^–1^ for **DPXZ-QX**. With a goal to further accelerating *k*_RISC_, sandwich A-D-A structures **DPXZ-2QX** and **DPXZ-2DFQX** were synthesized. These two compounds emit at λ_PL_ of 594 and 599 nm, have Φ_PL_ of 87 and 91%, and have τ_d_ of 8.7 and 4.9 μs correlated to their Δ*E*_ST_ of 0.02 and −0.05 eV, all respectively, leading to fast *k*_RISC_ of 8.21 and 4.64 × 10^5^ s^–1^; again, the apparent negative Δ*E*_ST_ is likely a reflection of not accurately measuring the phosphorescence energy, where at 77 K delayed fluorescence may still also exist. The OLEDs with **DPXZ-QX**, **DPXZ-DFQX**, **DPXZ-2QX**, and **DPXZ-2DFQX** emitted at λ_EL_ of 597, 602, 609, and 616 nm, and the device with **DPXZ-2QX** showed the best performance with an EQE_max_ of 23.2% (6 wt% doped in mCBP matrix). When the emitter concentration was increased to 12 wt% the EQE_max_ was maintained at a comparable value of 21.1%, and these devices retained a higher EQE_1000_ of 19.9%, compared to 14.4% for the 6 wt% device; however, the higher doping was accompanied by a red-shifted λ_EL_ of 616 nm and CIE coordinates of (0.60, 0.39).

The same group also investigated the impact of the addition of heavy atoms on TSCT-TADF properties by substituting the oxygen atom in the DPXZ donor with sulfur in **DPTZ-QX** and **DPTZ-DFQX** (Figure 161).^1117^ In 5 wt% mCP, **DPTZ-DFQX** and **DPTZ-QX** emit at λ_PL_ of 565 and 561 nm, which are blue-shifted relative to their PXZ analogues. Simultaneously, the T_1_ states became more LE in nature, inducing larger Δ*E*_ST_ of 0.14 and 0.15 eV respectively. Consequently, longer τ_d_ of 255.0 and 114.3 μs and lower Φ_PL_ of 49 and 61% were observed for **DPTZ-QX** and **DPTZ-DFQX**, compared with τ_d_ of 26.9 and 6.8 μs, and Φ_PL_ of 74 and 71% for the previously reported **DPXZ-QX** and **DPXZ-DFQX**,^1116^ all respectively. This work emphasizes the importance of fully considering the multifaceted influences of heavy atoms on TSCT excited states and RISC.

Using the same carbazole bridge, Miranda-Salinas *et al*. reported four TSCT emitters using triphenylamine and phenylcarbazole donors and TRZ as the acceptor.^1118^ By increasing the electron-donating strength of the donor groups, the dominant CT state was tuned from through-bond between the carbazole bridge and the TRZ acceptor to through-space between the co-facially aligned decorated donor and acceptor groups. Compounds **Ph_3_TRZCzTPA** and **Ph_2_TRZCzTPA** (Figure 161) showed onsets of their respectively emission spectra at 2.89 and 2.79 eV, and have small Δ*E*_ST_ of 90 and −130 meV in 10 wt% doped films in DPEPO; again, the apparent negative Δ*E*_ST_ likely reflects that the S_1_ and T_1_ energies were measured for different species as there is no photophysical reason for inverted singlet-triplet gaps in this class of material. The devices with **Ph_3_TRZCzTPA** and **Ph_2_TRZCzTPA** showed green emissions at λ_EL_ of 522 and 529 nm and EQE_max_ of 13.3 and 16.3%, respectively, while the QE_1000_ decreased to 9.7 and 10.9%.

Ma *et al*. reported four emitters containing carbazole bridges, but with substituents connected at different positions to permit fine-tuning of the CT interaction from through-bond to through-space.^1119^ **1TCPM**, **2TCPM**, and **1TCPM-Cz** all show dual-emissions at 411/510 nm, 425/464 nm and 411/521 nm, while **3TCPM** emits at λ_PL_ of 478 nm in toluene. As neat films the Δ*E*_ST_ of **1TCPM** (0.02 eV) and **1TCPM-Cz** (0.04 eV) are much smaller than **3TCPM** (0.26 eV) and **2TCPM** (0.37 eV, Figure 161), arising from the greater separation of electron density on the donor and acceptor groups. Restricted intramolecular motion in **1TCPM** and **1TCPM-Cz** suppresses the non-radiative decay pathways, resulting in higher Φ_PL_ of 50 and 57% compared to 35 and 20% for **3TCPM** and **2TCPM** in neat film, respectively. All emitters have short τ_d_ of 2.1, 2.3, 1.7, and 1.6 μs for **3TCPM**, **2TCPM, 1TCPM**, and **1TCPM-Cz**, respectively. The devices with **3TCPM**, **2TCPM, 1TCPM**, and **1TCPM-Cz** showed EQE_max_ of 3.4, 1.8, 7.6, and 13.3% at λ_EL_ of 478, 471, 489, and 505 nm, respectively.

Li *et al*. reported a series of D-A-D sandwich TSCT emitters by employing carbazole as the bridge and decorating different donors on either side of a central TRZ acceptor, giving the compounds **AcPTC**, **PxPTC**, and **PtPTC** (Figure 161).^1120^ DFT calculations and single crystal X-ray diffraction analysis revealed that the three emitters all showed clear edge-to-face π-π interactions. By increasing the electron donating ability of the donors, the emission is red-shifted from 485 nm for **AcPTC** to 522 nm for **PxPTC** and 561 nm for **PtPTC**, all in 20 wt% doped films in SimCP2. **AcPTC**, **PxPTC**, and **PtPTC** have high Φ_PL_ of 73, 61, and 51%, small Δ*E*_ST_ of 0.05, 0.03, and 0.03 eV, along with τ_d_ of 10.5, 3.0, and 11.4 μs, all respectively. The devices with **AcPTC**, **PxPTC**, and **PtPTC** achieved EQE_max_ of 10.0, 11.0, and 5.6% at λ_EL_ of 483, 533, and 564 nm, respectively. Using the same sandwich D-A-D strategy the same group also investigated the use of triphenylamine or 4,4′-di-(*tert*-but­yl)­tri­phen­yl­amine as the donors in **PAPTC** and **BPAPTC** (Figure 161).^406^ The introduction of the *tert*-butyl groups shortened the donor-acceptor distance to 3.081 from 3.139 Å, leading to improved TADF properties. In 20 wt% doped films in SimCP2 **BPAPTC** showed a red-shifted emission at 519 nm, a higher Φ_PL_ of 90%, and similar Δ*E*_ST_ of 0.06 eV and τ_d_ of 0.62 μs compared with **PAPTC** (λ_PL_ at 509 nm, Φ_PL_ of 78%, Δ*E*_ST_ of 0.07 eV, and τ_d_ of 0.61 μs). The solution-processed devices with **PAPTC** and **BPAPTC** showed EQE_max_ of 17.4 and 24.3% at identical λ_EL_ of 520 nm. Notably, the device with **PAPTC** still retained an EQE of 11.6% at 3000 cd m^–2^, and the device with **BPAPTC** retained 19.8% at 3000 cd m^–2^ and even 13.7% at 10000 cd m^–2^. The vastly superior performance of the device with **BPAPTC** results from the slight difference in the molecular structure instilled by the *tert*-butyl group.

To compare the sandwich D-A-D concept to equivalent D-A ‘open sandwich’ materials, the same group reported another set of emitters: **mBPAPTC**, **BPAMTC**, **mBPAMTC**, and **MPAPTC** (Figure 161).^1121^ The D-A-D sandwich compounds **MPAPTC** and **BPAMTC** showed slightly red-shifted emission profiles with λ_PL_ of 546 and 492 nm, compared to the open sandwich analogues with λ_PL_ of 510 and 491 nm for **mBPAPTC** and **mBPAMTC**, all in respective 20 wt% doped films in SimCP2. From an analysis of these λ_PL_ values it is evident that introduction of methoxy groups significantly red-shifts the emission. The Δ*E*_ST_ values range from 0.002 to 0.13 eV, which are all sufficiently small to support TADF. The Φ_PL_ are 90% for **BPAPTC** (a relevant structure from the previous examples) and 90% for **mBPAPTC**, 63% for **BPAMTC**, 69% for **mBPAMTC**, and 44% for **MPAPTC**. The Φ_PL_ of **BPAPTC/BPAMTC** D-A-D sandwich compounds are therefore higher than those of the corresponding open sandwich emitters **mBPAPTC/mBPAMTC**, revealing useful practical design rules for this class of emitter. The origin of the higher Φ_PL_ was attributed to their shorter π-π interaction distances and more rigid structures. The OLEDs with **BPAPTC**, **mBPAPTC**, **BPAMTC**, **mBPAMTC**, and **MPAPTC** emitted at λ_EL_ of 520, 520, 486, 484, and 564 nm, respectively. The devices with **BPAPTC** and **BPAMTC** showed higher EQE_max_ of 23.3 and 14.7%, compared to devices with **mBPAPTC** and **mBPAMTC** possessing EQE_max_ of 17.8 and 9.5% respectively, which were correlated with the higher Φ_PL_ of the former. The device with **MPAPTC** showed a lower EQE_max_ of 9.1%, while the EQE of the devices with **BPAPTC**, **mBPAPTC**, **BPAMTC**, **mBPAMTC**, and **MPAPTC** decreased to 20.4, 13.2, 8.8, 5.3 and 6.2% at 1000 cd m^–2^.

### 12.5 TSCT featuring other bridges

In some of these TSCT materials the lowest-energy excited state can be described as a combination of both TSCT and through-bond CT (TBCT), as the bridging unit itself can be directly involved in the electronic transitions. In parallel, as TSCT becomes more deeply understood over time, examples of TBCT materials can sometimes be ‘rediscovered’ as having TSCT character.^474^ As an illustrative example of this evolving understanding, Chen *et al*. reported **B-oCz** and **B-oTC** (Figure 162).^1122^ These two emitters have either a carbazole or a *tert*-butylcarbazole donor group that is *ortho*-disposed to an aryl boron acceptor. Such a structure may simply be assumed to be a TBCT emitter, although this structure also arranges the donor and acceptor in a co-facial array. Indeed, the crystal structures revealed a short intramolecular donor-acceptor distance of 2.76–3.55 Å.^119^ **B-oCz** and **B-oTC** emit at λ_PL_ of 465 and 476 nm and have Δ*E*_ST_ of 0.06 and 0.05 eV as neat films, respectively. The Φ_PL_ of **B-oCz** is 61% as the neat film but significantly increases to 94% when the Cz donor is replaced with the sterically bulkier *tert*-butylcarbazole in **B-oTC**. Although this substitution does impact the donor strength, it is implausible for this alone to result in such large changes in Φ_PL_. The higher Φ_PL_ for **B-oTC** was instead attributed to the increased steric bulk of the donor that inhibits both intermolecular and intramolecular π–π stacking, favoring the TBCT excited state. The solution-processed non-doped OLEDs with **B-oCz** and **B-oTC** showed blue emission with λ_EL_ of 463 and 474 nm and CIE coordinates of (0.15, 0.17) and (0.50, 0.26), and EQE_max_ of 8.0 and 19.1% respectively. However, these two devices exhibited serious efficiency roll-off with low EQE_1000_ of only 2.6 and 9.7%.

**Figure 162:**
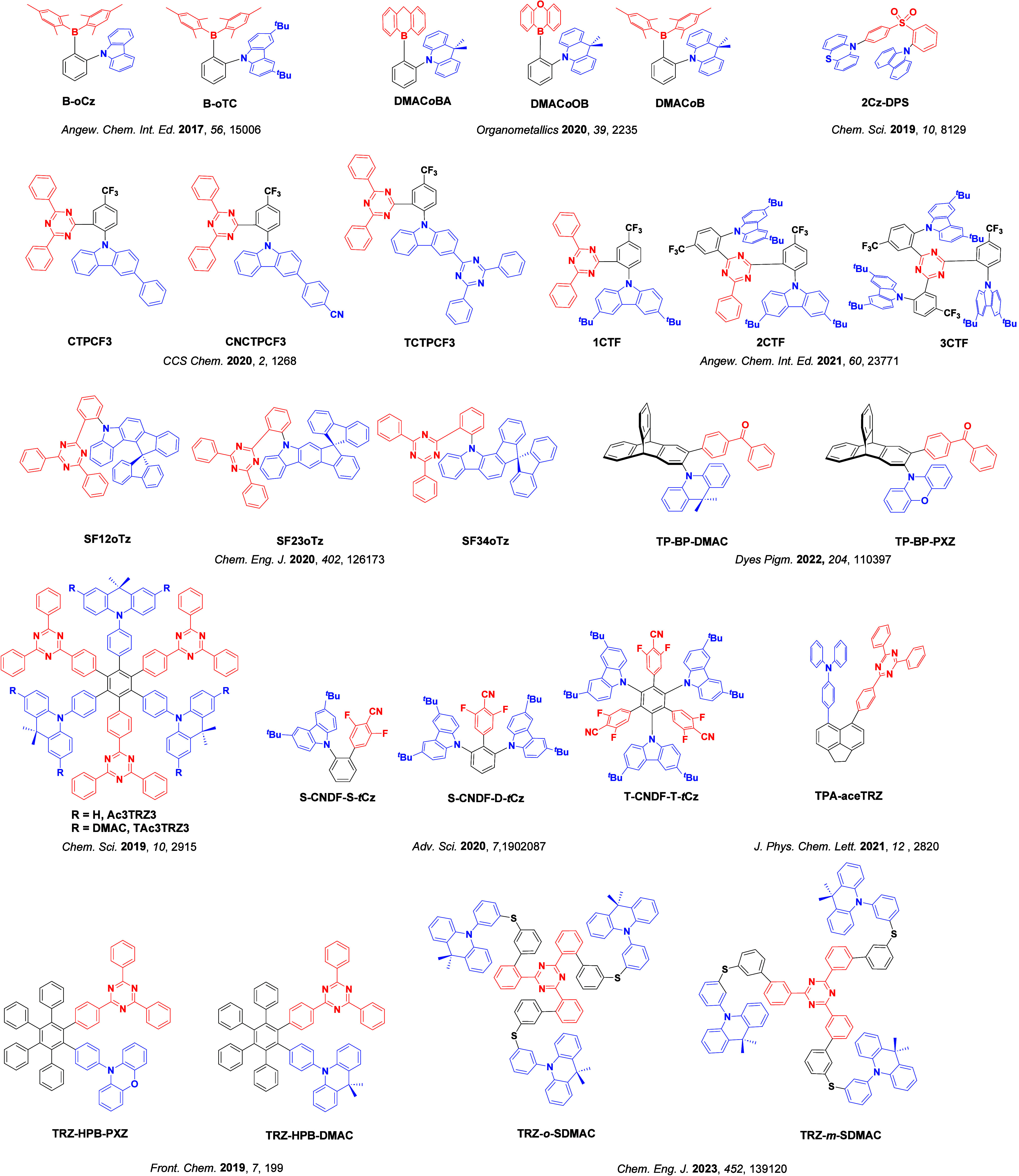
Structures of TSCT TADF emitters using other types of bridges (the blue color signifies donor moieties, while the red color signifies acceptor moieties).

Kim *et al*. reported similar *ortho*-D-A compounds containing different boryl acceptors: **DMAC*o*BA**, **DMAC*o*OB**, and **DMAC*o*B** (Figure 162).^1123^ Compared with **DMAC*o*B**, **DMAC*o*BA** and **DMAC*o*OB** exhibited weaker N–B and C–H·π non-bonding interactions between the DMAC donor and the boryl moieties. Due to the planar structure of the cyclic boryl acceptors of **DMAC*o*BA** and **DMAC*o*OB**, these fragments are orientated perpendicular relative to the phenylene ring in these compounds, which leads to an excited state of almost purely TSCT character. The boryl plane in **DMAC*o*B** is instead tilted relative to the phenyl ring, which according to the authors leads to mixed TSCT and TBCT character. Compared to the blue emission of **DMAC*o*BA** and **DMAC*o*OB** (λ_PL_ of 488 and 481 nm, Table S16), **DMAC*o*B** hence shows red-shift emission at λ_PL_ of 518 nm, and all three emitters show high Φ_PL_ of 100% in 10 wt% doped films in PMMA. **DMAC*o*BA**, **DMAC*o*OB**, and **DMAC*o*B** have τ_d_ of 15.1, 10.8, and 8.5 μs in the same films, while their respective Δ*E*_ST_ are 0.014, 0.004, and 0.001 eV in toluene at 77 K. No devices were fabricated with these emitters.

Yang *et al*. reported a derivative of **PTZ-DPS** that places a carbazole donor at the *ortho* position of sulfone acceptor, **2Cz-DPS** (Figure 162).^1124^ Through this approach, both TSCT (with the carbazole) and TBCT (with the PTZ) excited states co-exist. The steric congestion resulting from the *ortho*-carbazole donor also leads to reduced molecular motion and suppressed non-radiative decay pathways. **2Cz-DPS** emits in the green at λ_PL_ of 520 nm as the neat film, implying an excited state of mainly TBCT character (as the TSCT state would be expected to be higher in energy by comparison to the previously reported of **dtCzDPS**).^230^ **2Cz-DPS** has a large Δ*E*_ST_ of 0.32 eV and a high Φ_PL_ of 91.9% as a neat film. Even with this large Δ*E*_ST,_ the emission decays with τ_p_ and τ_d_ of 4.4 ns and 19.1 μs. The **2Cz-DPS**-based non-doped OLED emitted at λ_EL_ of 518 nm and showed an EQE_max_ of 28.7%, with EQE_300_ decreased significantly to 8.4%.

Duan and co-workers reported three TSCT emitters with *ortho*-disposed Cz and TRZ units attached to a (trifluoromethyl)benzene linker.^1125^ The dipole moments are reduced with the addition of electron-deficient substituents on the donor group. Through this strategy, emitters possessing combined TBCT and TSCT character were developed; however, owing to the highly twisted conformation between donor and acceptor groups the TSCT state often becomes dominant. **CTPCF3**, **CNCTPCF3**, and **TCTPCF3** (Figure 162) all have donor-acceptor distances sufficiently short to enable TSCT (2.85–3.81 Å), and emit at λ_PL_ of 494, 475, and 468 nm, respectively. The trends in the emission spectra can be attributed to the increasing strength of the electron-withdrawing groups on the donor, weakening the overall electron-donating ability of the carbazole. The Δ*E*_ST_ are 0.04, 0.28, and 0.06 eV for **CTPCF3**, **CNCTPCF3**, and **TCTPCF3**, respectively, where the larger Δ*E*_ST_ of **CNCTPCF3** was attributed to the increased LE character of its triplet excited state. **CTPCF3**, **CNCTPCF3**, and **TCTPCF3** have τ_d_ of 3.21, 6.04, and 2.52 μs (Table S16) that mirror the trend in Δ*E*_ST_, and have high Φ_PL_ of 96, 67, and 65%, suggesting that the twisted structure can effectively suppress concentration-quenching effects. Benefitting from the efficient TADF of these three emitters, HF devices employing **CTPCF3**, **CNCTPCF3**, and **TCTPCF3** as sensitizers and **2F-BN** as the MR-TADF terminal emitter showed green emission at λ_EL_ of 495, 497, and 495 nm, and high EQE_max_ of 33.1, 25.6, and 23.2%, respectively.^1126^ The **CTPCF3** based device showed modest efficiency roll-off, with EQE_1000_ remaining at 27%.

The same group developed three other related emitters with different numbers of *tert*-butylcarbazole groups as donors and TRZ as the acceptor, **1CTF**, **2CTF**, and **3CTF** (Figure 162).^1127^ A secondary trifluoromethyl (CF_3_) acceptor group was also incorporated to modulate the contributions from the TSCT and TBCT states. Benefiting from the steric clash of the Cz-donor perpendicularly linked to the acceptor plane, **2CTF** and **3CTF** both show face-to-face donor-acceptor interactions. Only edge-to-face donor-acceptor interactions were observed for **1CTF** as a result of the less crowded steric environment. **1CTF**, **2CTF**, and **3CTF** emit at λ_PL_ of 500, 507, and 514 nm in toluene and have near unity Φ_PL_ of 99, 98, and 99%, small Δ*E*_ST_ of 0.03, 0.03, and 0.04 eV, and short τ_d_ of 2.4, 1.8, and 1.2 μs, all respectively. Devices with **1CTF**, **2CTF**, and **3CTF** showed good EQE_max_ of 17.5, 19.8, and 22.6% at λ_EL_ of 490, 503, and 508 nm with CIE coordinates of (0.23, 0.45), (0.26, 0.54), and (0.29, 0.57), all respectively. The efficiency roll-off of the devices was modest, with EQE_1000_ of 14.5, 17.6, and 21.0% for the devices with **1CTF**, **2CTF**, and **3CTF**.

Lv *et al*. reported three emitters, **SF12oTz**, **SF23oTz**, and **SF34oTz**, consisting of spiro-fluorene-fused carbazole donors attached a the *ortho*-position of TRZ acceptors (Figure 162).^1128^ By changing the position of the fused fluorene, the molecular geometry and subsequent ratio of TBCT/TSCT character for each molecule could be modulated. DFT calculations predicted that **SF34oTz** has dominant TSCT character (96.8%), whereas **SF23oTz** and **SF12oTz** contain mixed TBCT and TSCT character, with the ratio of TBCT increasing from 21 to 32% in the latter. Due to the presence of a stronger donor, **SF34oTz** has the most red-shifted emission with λ_PL_ of 479 nm in toluene, whereas **SF23oTz** and **SF12oTz** exhibit dual-emission in toluene with respective λ_PL_ of 383/473 and 371/491 nm; the higher energy λ_PL_ at 383/371 nm arises from LE emission of the donors. The Δ*E*_ST_ values in 20 wt% doped films in DPEPO are 0.29 eV for **SF34oTz**, 0.08 eV for **SF23oTz**, and 0.05 eV for **SF12oTz**, leading to τ_d_ of 8.2, 4.3, and 4.6 μs for the same. High Φ_PL_ of 92 and 86% were observed for **SF12oTz** and **SF23oTz**, respectively, while **SF34oTz** has a lower Φ_PL_ of 65%. The resulting solution-processed green OLEDs with **SF12oTz**, **SF23oTz**, and **SF34oTz** exhibited EQE_max_ of 22.4, 19.6, and 14.6% at λ_EL_ of 496, 484, and 482 nm, all respectively. The devices with **SF12oTz** and **SF23oTz** showed minimal efficiency roll-off, with EQE_1000_ of 20.0 and 15.9%, respectively. Due to the long delayed lifetimes, the device with **SF34oTz** showed more serious efficiency roll-off with an EQE_1000_ of 3.1%.

Huang *et al*. reported two other emitters, **TP-BP-DMAC** and **TP-BP-PXZ** (Figure 162), in which a benzophenone acceptor and DMAC or PXZ donor units are attached at adjacent positions on a triptycene bridge.^1129^ The *ortho*-linkage of the donor and acceptor leads to face-to-face alignment and strong intramolecular donor-acceptor interactions. The non-planar triptycene scaffold was chosen to limit concentration-related aggregation and quenching, and to improve film quality. **TP-BP-DMAC** and **TP-BP-PXZ** emit at λ_PL_ of 508 and 531 nm in 20 wt% doped films in DPEPO, respectively. The RISC activation energies, determined using an Arrhenius analysis of the variable-temperature time-resolved PL decays, are 6.7 and 10.8 meV, while the Φ_PL_ of **TP-BP-DMAC** and **TP-BP-PXZ** are 80 and 40%, all respectively. OLEDs with **TP-BP-DMAC** and **TP-BP-PXZ** showed EQE_max_ of 20.5 and 13.8%, which remained as high as 9.6 and 9.3% at 1000 cd m^–2^, while the EL spectra are consistent with the λ_PL_ at λ_EL_ of 488 and 531 nm with CIE coordinates of (0.21, 0.38) and (0.35, 0.53), all respectively.

The strategy of attaching donor and acceptor units *ortho* to each other has also been expanded with the use of hexaphenylbenzene scaffolds (HPB). HPB has a non-planar propeller shaped structure, where peripheral groups sit orthogonal to the central benzene due to steric constraints. This conformation forces the peripheral donor and acceptor groups to adopt co-facial arrangements, which in turn enables TSCT states. Two examples of this design strategy are the emitters **Ac3Trz3** and **TAc3Trz3** (Figure 162), which emit at λ_PL_ of 505 and 535 nm, respectively in 10 wt% doped films in AC-6 as the host.^1130^ Both materials have small Δ*E*_ST_ of 0.08 and 0.04 eV and moderate Φ_PL_ of 54 and 63%, respectively. The resulting solution-processed green OLEDs with **Ac3Trz3** and **TAc3Trz3** showed EQE_max_ of 11.0 and 14.2% at λ_EL_ of 520 and 538 nm, while the EQE_100_ remained at 10.4 and 13.5%, all respectively.

Zheng *et al*. employed a similar design strategy, reporting a series of emitters that build step-wise to the fully substituted HPB: **S-CNDF-S-*t*Cz**, **S-CNDF-D-*t*Cz**, and **T-CNDF-T-*t*Cz** (Figure 162).^327^ This multi-chromophore approach was claimed by the authors to increase *k*_RISC_ by exploiting the presence of degenerate triplet states that form on the different donors and acceptors. The emitter **T-CNDF-T-*t*Cz** contains three donor and three acceptor units and emits in the sky-blue at λ_PL_ of 472 nm and has the smallest Δ*E*_ST_ of 0.03 eV of the series of compounds studied (Table S16), a high Φ_PL_ of 76%, and *k*_RISC_ of 5.07 ± 0.65 × 10^5^ s^–1^ as the neat film. Non-doped OLEDs with **T-CNDF-T-*t*Cz** showed an EQE_max_ of 21% at λ_EL_ of 466 nm.

Using the same HPB scaffold to bridge triazine to different donors (acridine and phenoxazine), Tang and co-workers reported the two emitters **TRZ-HPB-PXZ** and **TRZ-HPB-DMAC** (Figure 162).^1131^ These two compounds emit at λ_PL_ of 576 and 484 nm, reflective of the relative strength of the donor group, have Φ_PL_ of 61.5 and 51.8%, and small Δ*E*_ST_ of 0.02 and 0.09 eV, all respectively as neat films. The non-doped devices with **TRZ-HPB-PXZ** and **TRZ-HPB-DMAC** showed EQE_max_ of 12.7 and 6.5%, which decreased to 12.3 and 6.0% at 1000 cd m^–2^. The device with **TRZ-HPB-PXZ** and **TRZ-HPB-DMAC** showed λ_EL_ of 544 and 521 nm and CIE coordinates of (0.39, 0.57) and (0.28, 0.58), all respectively. These results indicate that the HBP-based TSCT emitters can effectively suppress exciton annihilation processes by inhibiting aggregation.

Li *et al*. reported a series of propeller-shaped isomers with a triazine acceptor and three donor units linked via diphenylsulfides.^1132^ Highlighting two of these compounds, **TRZ-o-SDMAC** and **TRZ-m-SDMAC** (Figure 162) emit at λ_PL_ of 496 and 499 nm and both have small Δ*E*_ST_ of 0.01 eV as neat films. **TRZ-m-SDMAC** has a Φ_PL_ of 52% while that of **TRZ-o-SDMAC** is much lower at Φ_PL_ of 13%, likely due increased non-radiative decay processes arising from the donors being connected *meta* to the triazine. Devices with **TRZ-m-SDMAC** exhibited blue-green emission at λ_PL_ of 510 nm with CIE coordinates of (0.24, 0.49) and an EQE_max_ of 20.3% but with a very large efficiency roll-off of 78.5% at 1000 cd m^–2^. The **TRZ-o-SDMAC** device showed inferior EQE_max_ of only 1.1% at λ_EL_ of 518 nm with CIE coordinates of (0.30, 0.47).

Zysman-Colman, Monkman, and co-workers have also used acenaphthene as a scaffold, employing TPA as a donor and TRZ as an acceptor in the emitter **TPA-ace-TRZ** (Figure 162).^1133^ The structure of **TPA-ace-TRZ** places the donor and acceptor highly coplanar and at quite short distances compared to other examples in this section. The spectroscopic study evidenced conclusively the presence of both TSCT and TBCT states, while the TSCT interaction is frequently only inferred from a combination of DFT calculations and structural information derived from X-ray structure analysis in other works. **TPA-ace-TRZ** emits at λ_PL_ of 518 nm and has a Φ_PL_ of only 17% in toluene.^1133^ In 1 wt% zeonex film **TPA-ace-TRZ** emits at λ_PL_ of 505 nm but has a large Δ*E*_ST_ of 0.48 eV and low Φ_PL_ of only 12%. No delayed emission lifetime was observed due to the large Δ*E*_ST_.

### 12.6 TADF and CT States Featuring Homoconjugation

Somewhat distinct from both TBCT and TSCT states, in homoconjugated systems the donor and acceptor moieties are connected via a bridge where the electronic coupling is mediated by co-aligned sigma bonds, while the distances between these fragments are too large to mediate direct TSCT interactions via their π-network. Triptycene is a specific bridge that permits this type of homoconjugation to occur and this strategy was first explored by Swager and co-workers in the compounds **TPA-QNX(CN)2** and **TPA-PRZ(CN)_2_** (Figure 163).^1134^ In these materials the triphenylamine donor and the dicyanoquinoxaline or dicyanopyrazine acceptor units are fixed at 120° relative to one another across the three arms of the bridge. The homoconjugated CT excited states resulted in predicted Δ*E*_ST_ of 0.11 and 0.08 eV, respectively. **TPA-QNX(CN)2** emits at λ_PL_ of 487 nm, has a moderate Φ_PL_ of 44%_,_ and a τ_d_ of 2.4 μs in cyclohexane. The OLEDs showed a significantly red-shifted emission at λ_EL_ = 573 nm and CIE coordinates of (0.45, 0.54), but nonetheless showed an EQE_max_ of 9.4% (10 wt% doped in mCP). The large red-shift was ascribed by the authors to the sensitivity of the CT state to the polarizability of the surrounding medium.

**Figure 163:**
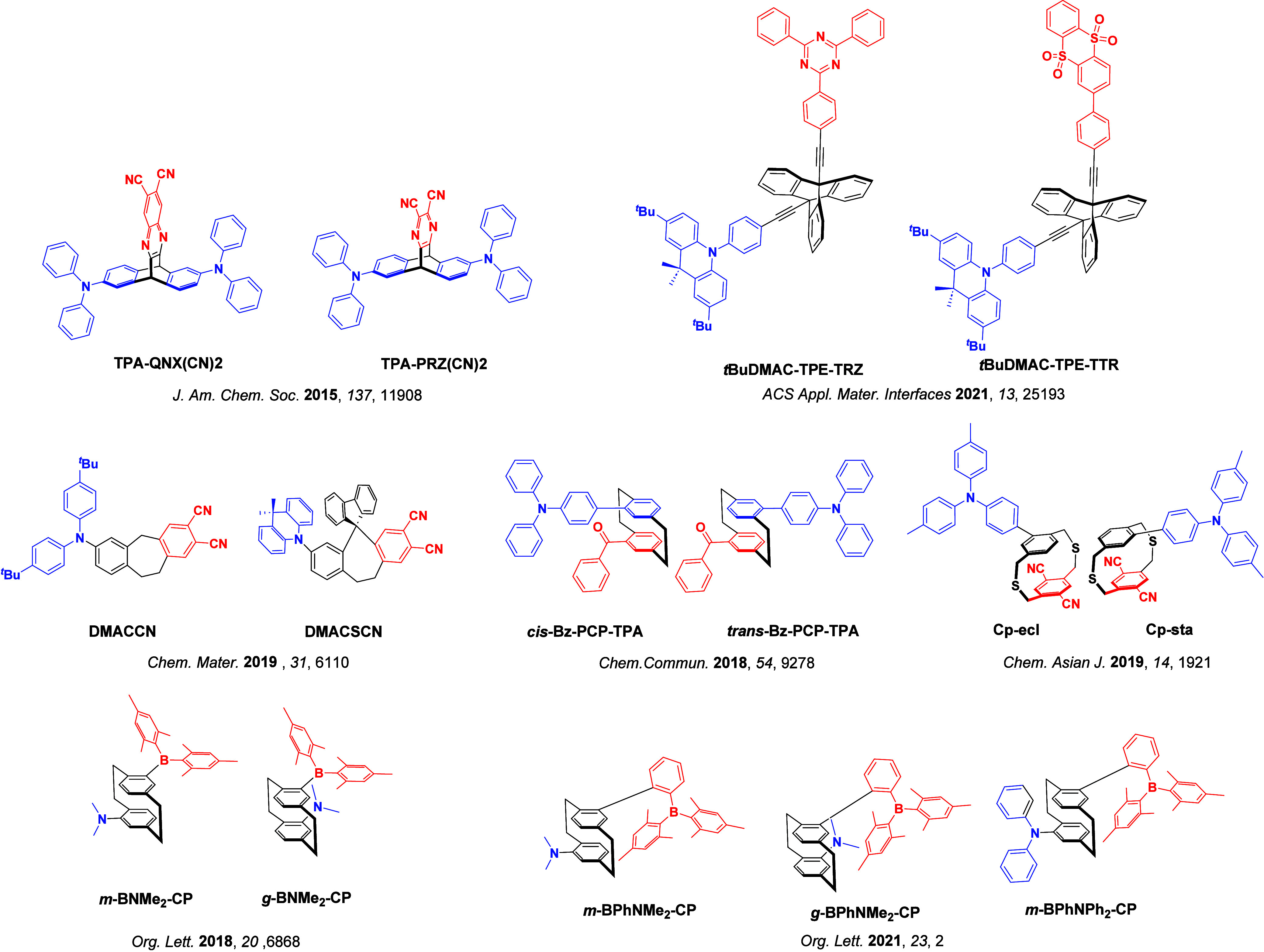
Structures of TADF emitters featuring homoconjugation between the donor and the acceptor (the blue color signifies donor moieties, while the red color signifies acceptor moieties).

Zhang and co-workers reported the emitters ***t*BuDMAC-TPE-TRZ** and ***t*BuDMAC-TPE-TTR** (Figure 163), where the donor and acceptor units were also separated with a triptycene bridge.^1135^ Uniquely though, the donors and acceptors are positioned more remote from the triptycene, with the donor separated by an ethynyl bridge to the bridgehead carbon of the triptycene, and the acceptor attached to one of the arms. ***t*BuDMAC-TPE-TRZ** emits at 500 nm and has a Φ_PL_ of 43.7% (Table S16)_._ Using transient PL measurements at different concentrations in PMMA the authors demonstrated that intra and intermolecular CT channels both play roles in the emission process. The non-doped devices with ***t*BuDMAC-TPE-TRZ** and ***t*BuDMAC-TPE-TTR** showed green and red emission at λ_EL_ of 532 and 600 nm and showed EQE_max_ of 10.0 and 1.3%, respectively.

Yersin *et al*. bridged a TPA donor and a dicyanobenzene acceptor through a non-conjugated alkyl spacer in the compound **DMACCN** (originally named **1**, renamed here for clarity, Figure 163), and also reported a derivative that contains a spiro-fluorene between donor and acceptor groups to mediate a TSCT interaction in **DMACSCN** (originally named **2**).^1136^ **DMACCN** and **DMACSCN** have very small calculated Δ*E*_ST_ of 6 and 2 meV, respectively. **DMACCN** emits at λ_PL_ of 476 nm and has a τ_d_ of 9 μs in toluene solution while **DMACSCN** emits at λ_PL_ of 468 nm, has a Φ_PL_ of 65%, and a very short τ_d_ of 420 ns. The authors claimed that the introduction of a plurality of high lying states for coupling resulted in the apparent disappearance of any long-lived TADF due to the very fast RISC between the pseudo-degenerate ^1^CT and ^3^CT states.

Spuling *et al*. explored intramolecular TSCT using a [2.2]paracyclophane (PCP) bridging unit (Figure 163).^1137^ The reduced Van der Waals distance of 3.09 Å between the the two benzenes of the PCP is sufficiently small to mediate electronic communication between the donor and acceptor groups positioned on the benzene rings. The structure of the *cis*-linked (pseudo geminal) ***cis*-Bz-PCP-TPA**, or the *trans*-linked (pseudo anti) ***trans*-Bz-PCP-TPA** has a significant impact on the optical properties. ***Cis*-Bz-PCP-TPA** and ***trans*-Bz-PCP-TPA** exhibited blue emission in solution (two peaks of 404/492 and 404/455 nm in toluene, respectively), while the 15 wt% doped films in mCP emit λ_PL_ of 480 and 465 nm and have Δ*E*_ST_ of 0.13 and 0.17 eV (Table S16), leading to small τ_d_ of 1.8 and 3.6 μs, all respectively. Unfortunately, the Φ_PL_ of these emitters remained quite low in the solid state (12% for ***cis*-Bz-PCP-TPA** and 15% **for *trans*-Bz-PCP-TPA** in 15 wt% mCP film), and thus OLEDs were not fabricated. Adachi and co-workers reported emitters using the related dithia[3.3]paracyclophane bridging moiety, **Cp-ecl** and **Cp-sta** (Figure 163).^1138^ **Cp-ecl** and **Cp-sta** each emit at λ_PL_ of ∼520 nm and have Φ_PL_ of 61 and 2%, with Δ*E*_ST_ of 0.03 and 0.05 eV, all respectively.

Zhang *et al*. reported a series of structurally similar chiral green TADF molecules containing PCP bridging units, ***g*-BNMe_2_-Cp** and ***m*-BNMe_2_-Cp** (Figure 163). These emit at λ_PL_ of 531 (with Φ_PL_ = 72% in cyclohexane, Δ*E*_ST_ = 0.17 eV, and τ_d_ = 0.38 ms in toluene) and at 521 nm (with Φ_PL_ = 39% in cyclohexane, Δ*E*_ST_ = 0.12 eV, and τ_d_ = 0.22 ms in toluene), respectively.^638^ Recently the same group introduced a phenylene spacer between the PCP and the acceptor moiety to obtain sky-blue emitters showing an enhanced Φ_PL_ in cyclohexane of 83% for ***g*-BPhNMe_2_-Cp** (λ_PL_ = 488 m), 93% for ***m*-BPhNMe_2_-Cp** (λ_PL_ = 461 nm), and 82% for ***g*-BPhNPh_2_-Cp** (λ_PL_ = 455 nm).^1139^ To our knowledge there are not yet any reports of efficient OLEDs using PCP bridged TADF materials, widely stymied by low Φ_PL_.

### 12.7 Outlook

This section offers a comprehensive overview of TSCT TADF materials, providing an in-depth analysis of optoelectronic properties and their performance as emitters in OLEDs. The field of TSCT TADF design has witnessed significant advancements since its initial report by Tsujimoto *et al*. in 2017,^1098^ marked by the development of emitters with near-unity Φ_PL_, and with examples covering the entire visible spectrum.

Triazine, which is frequently used in other classes of TADF compounds, stands out as the most commonly used acceptor in the TSCT donor-acceptor motif. This preference is due to its planar geometry, readily forming co-facial or tilted co-facial interactions with the donor moiety. To date, the acceptor triazine has showcased its versatility in creating high-efficiency emitters on diverse backbones from non-conjugated bridges like xanthene and triptycene to conjugated counterparts like carbazole and spirofluorene. In these reported examples, fine-tuning the donor and acceptor structures has been instrumental in exploring and optimizing the CT strength between them. Differing from this design, Kaji and coworkers have delved into the impact of the distance and orientation between the donor and acceptor units on emitter performance, employing DMAC and triazine as the donor and the acceptor, respectively, attached to a triptycene bridge. This work provides a clue as to how the alignment of the ^3^LE state relative to the ^3^CT and ^1^CT states affects the RISC rate. However, it is worth noting that, similar to the conventional donor-acceptor TADF design, due to their long-range CT nature, TSCT emitters seem unavoidably to show broad emission, posing challenges in terms of the color purity of the device. Therefore, a promising avenue for future exploration lies in improving color purity, by supressing molecular vibration and possibly by incorporation of emissive excited state of SRCT character, like the strategies employed in MR-TADF emitter design.

Spirofluorene and carbazole by far have been used as the most popular backbones to anchor the electron donor and acceptor units, with the aim of achieving efficient TSCT. These advances have pushed the EQE_max_ of the devices beyond the theoretical value of 25–30%. For example, Wang *et al*.^1115^ demonstrated that the devices featuring sandwich-like emitters, **BNB-m** and **BNB-p**, achieving an impressive EQE_max_ of approximately 35%. Despite these remarkable achievements, there remains a need for research that explores the impact of backbone rigidity and stability on device performance, particularly in terms of device roll-off, color purity, and operational lifetime, particularly as most TSCT TADF emitter reports focus on decorating donors and acceptors with the objective to improve device efficiency.

In many of the examples presented here, the emissive excited states possess mixed TBCT/TSCT character; further, it is difficult to spectroscopically disentangle the contributions, if any, from these two excited states. In forming these states, the magnitude of the electronic coupling between donor and acceptor moieties in TSCT TADF compounds is mediated not only by the distance between the two but also their relative orientation, both of which are modulated by the choice of bridging scaffold. As one of the most recently popularized classes of TADF emitters, it is particularly exciting to imagine the novel and innovative molecular designs that will arise in this area in the coming years.

## 13 Compounds Displaying Both Aggregation-Induced Emission (AIE) and TADF

### 13.1 Introduction

One of the main challenges in luminophore design is their propensity to form aggregates, both in high-concentration solutions and during film deposition. This frequently leads to aggregation-caused quenching (ACQ), which results frequently in a significant decrease in the Φ_PL_ and a red-shifted emission. ACQ is observed to some extent in most aromatic emitters in the solid state, unless dispersed at low doping concentration into a host medium to disrupt intermolecular interactions between emitter molecules. This is a primary reason why the vast majority of examples reported in Sections 3–7 and 9–12 involve TADF molecules doped into a host matrix within the emissive layer of the device. The host molecules effectively keep the emitter molecules separated, preventing the short-range π-system overlap that drives ACQ; however, use of a host increases the complexity of OLED fabrication as well as the cost.

In 2001 a new mechanism to circumvent ACQ was introduced by Tang and co-workers.^1140^ Molecules with flexible functional groups, which were poorly emissive in solution due to non-radiative decay associated with molecular motion (rotations and vibrations), were shown to become very emissive in the solid state where these rotations are restricted. Aggregation of these emitters hinders these motions, limiting non-radiative decay, and hence enhances the emission of the aggregate – the complete opposite of ACQ. This phenomenon is known as aggregation-induced emission (AIE). In recent years this effect has been incorporated into TADF emitter design, offering the potential to deliver efficient non-doped OLEDs and sidestep the technical challenges and limitations associated with hosts.

### 13.2 Sulfone-Based AIE-TADF Emitters

Sulfone-based TADF emitters represent a large class of those that also show AIE. The structures of emitters containing a sulfone acceptor moiety are shown in Figure 164 and relevant photophysical and device data are tabulated in Table S17. The first AIE-TADF emitters containing a sulfone acceptor moiety, **TXO-TPA** and **TXO-PhCz**, were reported by Wang and co-workers.^1141^ The compounds were poorly emissive in toluene, with Φ_PL_ of 24 and 25% at λ_PL_ of 586 and 522 nm, respectively. AIE was demonstrated through changes in the emission color and intensity in acetonitrile/water mixtures, a now commonplace technique that allows the properties of the isolated and aggregated molecules to be determined as the mixed solvent is gradually changed from ‘good’ to ‘poor’ in terms of its capacity to solubilize the emitter. The neat films of each emitter showed enhanced Φ_PL_ of 36 and 93% at 625 and 570 nm for **TXO-TPA** and **TXO-PhCz**, respectively. Green-emitting OLEDs [CIE coordinates of (0.45, 0.53) and (0.31, 0.56), respectively] were fabricated incorporating both emitters and showed EQE_max_ of 18.5 and 21.5%, although these devices had EML consisting of 5 wt% emitters doped in mCP. This is a rather common theme for most of the reported AIE-TADF emitters; frequently, only doped devices are investigated, even when AIE is present, while non-doped OLEDs are neglected. We speculate that this arises from a desire to publish the highest possible EQE_max_ values for new emitters, with non-doped devices frequently struggling to surpass the performance doped devices.

**Figure 164:**
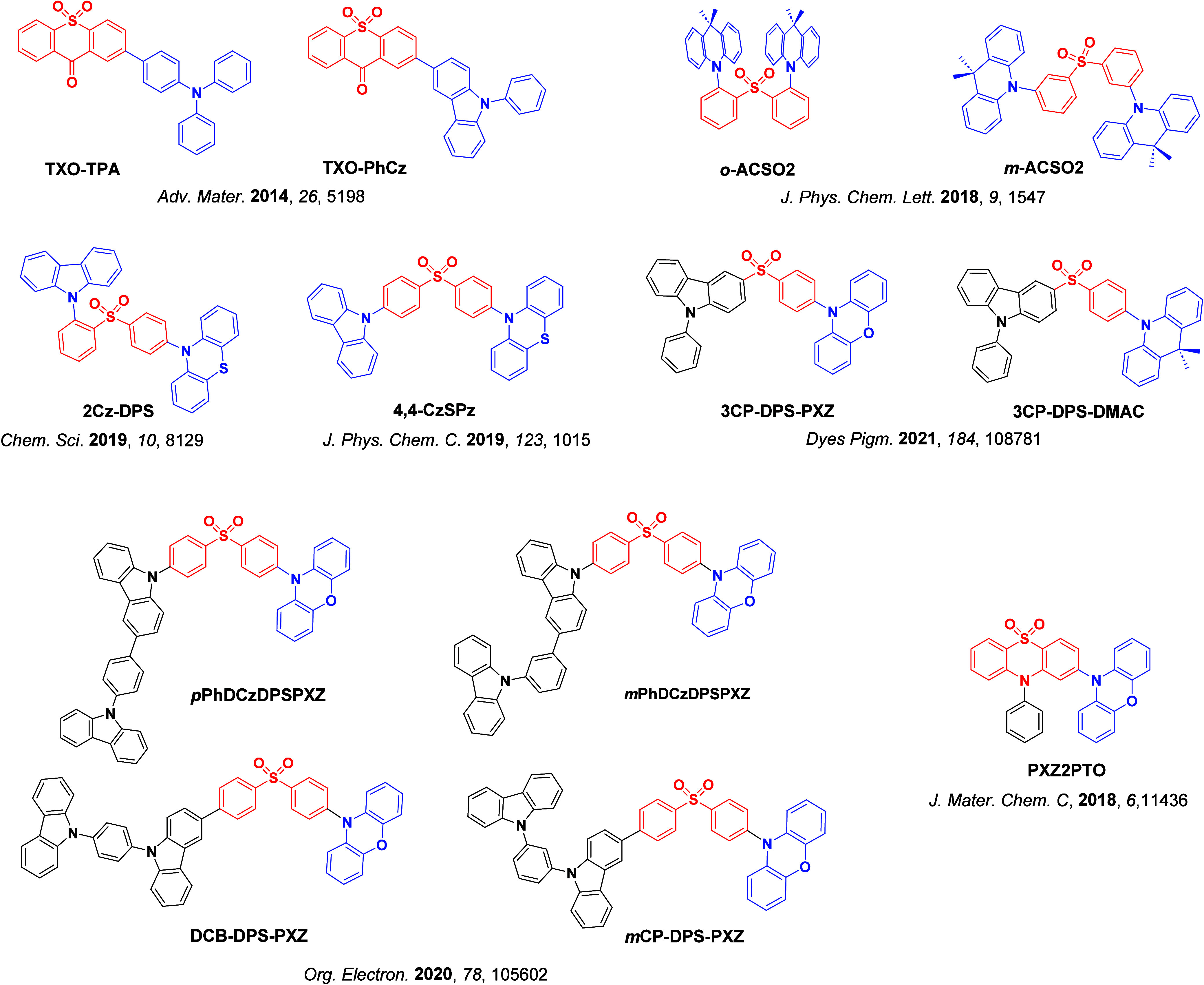
Structures of sulfone-based AIE-TADF emitters (the blue color signifies donor moieties, while the red color signifies acceptor moieties).

A blue non-doped solution-processed AIE-TADF device (λ_EL_ = 486 nm) using ***m*-ACSO2** (Figure 164) showed an EQE_max_ of 17.2% and a mild efficiency roll-off of 5% at 100 cd m^–2^. The high EQE_max_ results from a Φ_PL_ of 76% in neat films, along with a relatively short τ_d_ of 3.2 μs and a small Δ*E*_ST_ of 0.07 eV.^1142^ Devices with the analogue ***o*-ACSO2** showed much poorer performance in non-doped devices with an EQE_max_ of only 5.9%, which cannot be fully explained by the lower Φ_PL_ of 66%. This was observed despite the emitter having a short τ_d_ of 1.8 μs and a small Δ*E*_ST_ of 0.04 eV in the neat film, which highlights the challenges in rational molecular design of AIE-TADF emitters, which must simultaneously perform emission, triplet harvesting, and charge transport in non-doped devices.

Chi and co-workers reported **4,4-CzSPz**, an emitter containing two different donor groups, which has a near unity Φ_PL_ of 97.3% in the neat film (Figure 164).^1143^ A non-doped OLED based on **4,4-CzSPz** reached an EQE_max_ of 20.7% at λ_EL_ of 526 nm, attributed to the dual AIE and TADF character of the emitter. A doped device (10 wt% in CBP) did perform better with an EQE_max_ of 26.2% at λ_EL_ of 518 nm. Another example of a highly efficient non-doped OLED was reported with the similar emitter **2Cz-DPS**, which places the Cz donor at the *ortho* position to the sulfone. The non-doped device of **2Cz-DPS** showed a record-high EQE_max_ of 28.7% at λ_EL_ of 518 nm. The contributing factors for the excellent performance are the high Φ_PL_ of 91.9% at λ_PL_ of 520 nm, and the relatively short τ_d_ of 19.1 μs in the neat film. The inclusion of a carbazole donor also likely contributed to the improved intrinsic charge transport properties in the non-doped emissive layer. However, the non-doped devices suffered from a severe efficiency roll-off, with the EQE decreasing by 71% at 300 cd m^–2^.^1124^

Guo *et al*.^1144^ reported two AIE-TADF emitters, **3CP-DPS-PXZ** and **3CP-DPS-DMAC** (Figure 164), composed of a diphenylsulfone core within an asymmetrical D-A-D′ configuration. These two emitters showed Φ_PL_ of 52% at λ_PL_ of 518 nm and 65% at λ_PL_ of 472 nm in the neat film, respectively. The non-doped device with **3CP-DPS-PXZ** showed an EQE_max_ of 17.9% at λ_EL_ of 508 nm, which remained as high as 14.5% at 1000 cd m^–2^. The EQE_max_ of the **3CP-DPS-DMAC**-based blue non-doped OLED was comparatively lower at 9.1% (λ_EL_ = 484 nm). These examples highlight the popular yet poorly understood design strategy of using non-identical donors to achieve high performance in non-doped devices.

Leng *et al*.^1145^ consciously integrated host-like substituents (DCB, *m*CP, *p*PhDCz and *m*PhDCz) with AIE-TADF chromophores to generate the ‘self-hosting’ TADF emitters **DCB-DPS-PXZ**, ***m*CP-DPS-PXZ**, ***m*PhDCzDPSPXZ** and ***p*PhDCzDPSPXZ** (Figure 164) that have Φ_PL_ of 40, 47, 56 and 55%, with λ_PL_ of 547, 547, 548, and 548 nm, respectively, in the neat film. The host moieties were found not to be involved in CT transitions, and instead effectively dispersed the luminophoric centres, which led to the realization of high-performance non-doped OLEDs with EQE_max_ of 13.9, 14.7, 18.1 and 17.1% and λ_EL_ of 520, 520, 523 and 521 nm for the devices with **DCB-DPS-PXZ**, ***m*CP-DPS-PXZ**, ***m*PhDCzDPSPXZ** and ***p*PhDCzDPSPXZ**, respectively. The efficiency roll-off was found to be lower for the device with ***m*PhDCzDPSPXZ** (7.7%) than for the device with ***p*PhDCzDPSPXZ** (9.9%) at 1000 cd m^–2^; however, more severe efficiency roll-off was observed for the devices with **DCB-DPS-PXZ** (20.8%) and ***m*CP-DPS-PXZ** (17.7%) at 1000 cd m^–2^. While it is not clear whether these materials were intrinsically AIE-active, the strategy of using peripheral substitutions that preserve emission in the solid state overlaps strongly with the AIE approach.

The potential of 10-phen­yl-10*H*-phen­o­thia­zine 5,5-dioxide (2PTO) as an acceptor for AIE-TADF emitters was demonstrated by Wang and co-workers.^1146^ An emitter comprised of 2PTO and phenoxazine donors, **PXZ2PTO** (Figure 164), has a Φ_PL_ of 61.5% at λ_PL_ of 512 nm in the neat film. The non-doped device showed an EQE_max_ of 16.4% at λ_EL_ of 504 nm. Interestingly, the doped device (80 wt% doped in DPEPO) showed nearly the same EQE_max_ of 16.3% at 500 nm, demonstrating the utility of the AIE approach. Both devices exhibited low-efficiency roll-off of 4.9% for the doped and 7.9% for the non-doped device at 100 cd m^–2^.

### 13.3 Carbonyl-Based AIE-TADF Emitters

Although the reasons are at present unclear, many of the reported high-performance AIE-TADF materials feature carbonyl-based acceptor groups such as benzophenone and xanthone. The structures of the emitters are shown in Figure 165 and Figure 166, and relevant photophysical and device data are tabulated in Table S17. Tang and co-workers reported the asymmetric D-A-D′ emitter **DBT-BZ-DMAC**, which decorates a benzoyl core with and a dibenzothiophene and has an acridine donor.^1147^ This compound has a Φ_PL_ of 8.3% in THF, which increases to Φ_PL_ of 66% in 6 wt% doped CBP film and to 80% as the neat film, a clear indication of AIE. The EQE_max_ of the device containing 6 wt% **DBT-BZ-DMAC** in CBP was 17.9%, compared to 14.2% for the non-doped device. The non-doped device shows a lower efficiency roll-off, with an EQE_1000_ of 10.9% and 14.2% for the 6 wt% and non-doped devices, respectively.

**Figure 165:**
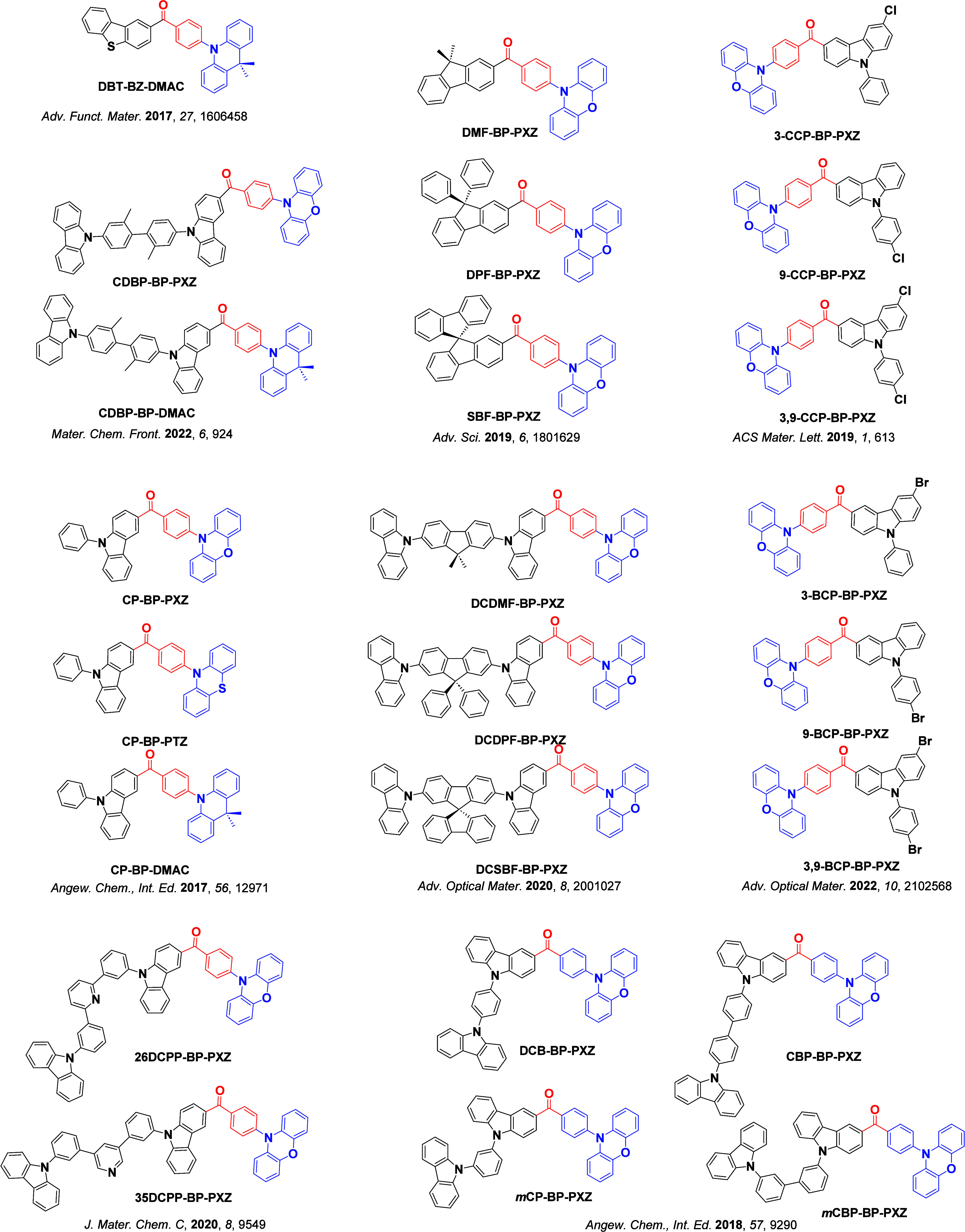
Structures of carbonyl-containing AIE-TADF emitters (the blue color signifies donor moieties, while the red color signifies acceptor moieties).

**Figure 166:**
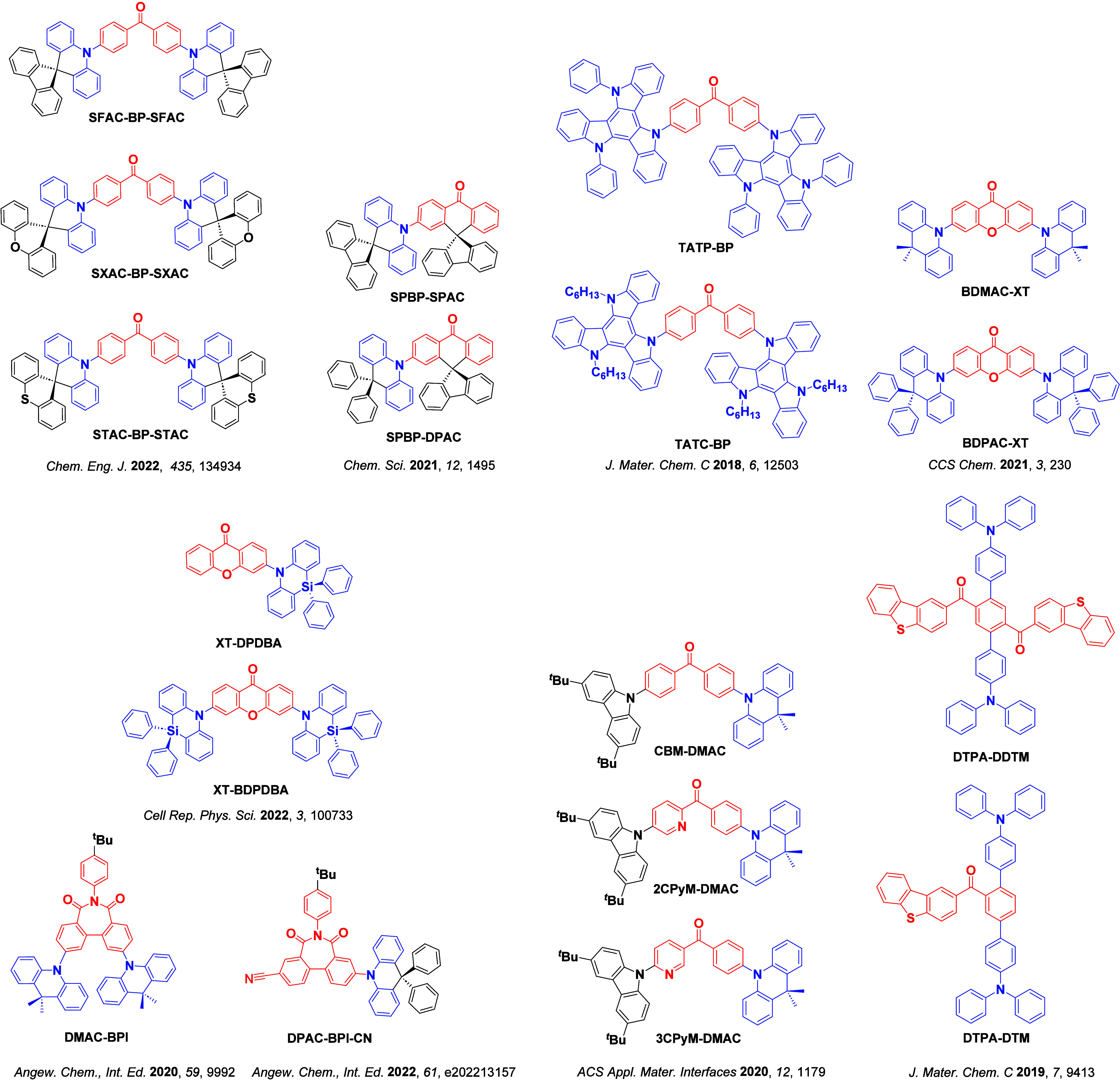
Structures of other carbonyl-containing AIE-TADF emitters (the blue color signifies donor moieties, while the red color signifies acceptor moieties).

Chen *et al*.^1148^ designed two emitters, **CDBP-BP-PXZ** and **CDBP-BP-DMAC** (Figure 165) using the same asymmetric D-A-D′ configuration strategy and appending a CDBP unit that has good OLED hosting properties. In the neat film the Φ_PL_ is 77.4% (λ_PL_ = 523 nm) for **CDBP-BP-PXZ** and 59.2% (λ_PL_ = 488 nm) for **CDBP-BP-DMAC**. The non-doped devices with **CDBP-BP-PXZ** and **CDBP-BP-DMAC** showed EQE_max_ of 15.5% at λ_EL_ of 536 nm and 9.5% at λ_EL_ of 496 nm, respectively, with corresponding efficiency roll-off of 0.6 and 2.1% at 1000 cd m^–2^. Huang *et al*.^1149^ reported the emitters **CP-BP-PXZ**, **CP-BP-PTZ**, and **CP-BP-DMAC** (Figure 165), which showed AIE activity in THF/water mixtures. Further, the magnitude of the delayed fluorescence contribution increased upon aggregate formation, thus revealing aggregation-induced delayed fluorescence (AIDF) acting not just on Φ_PL_ but also on RISC in these compounds. These compounds have Φ_PL_ ranging from 45.3 to 67.4% and λ_PL_ ranging from 490 to 538 nm in the neat films. The non-doped OLEDs showed EQE_max_ and λ_EL_ of 18.4% and 548 nm for the device with **CP-BP-PXZ**, 15.5% and 554 nm for the device with **CP-BP-PTZ**, and 15% and 502 nm for the device with **CP-BP-DMAC**. The devices exhibited a relatively small efficiency roll-off of 1.2, 16.7 and 0.2% at 1000 cd m^–2^, respectively, attributed to greatly suppressed emission quenching in the neat films.

Tang and co-workers reported three compounds **DMF-BP-PXZ**, **DPF-BP-PXZ**, and **SBF-BP-PXZ** (Figure 165) all containing a PXZ donor and carbonyl acceptors with progressively bulkier fluorene substituents.^1150^ The three compounds all emit similarly at λ_PL_ of 548–551 nm, have Φ_PL_ ranging from 45 to 49% in the neat film and short τ_d_ of 1.1–1.4 μs. Solution-state TAS showed no signal while the neat films showed a broad excited-state absorption in the range of 800–1000 nm, indicating the formation of a triplet state upon aggregation. From theoretical calculations and experimental observations, the authors claimed that AIDF originates from the S_2_ excited state rather than the S_1_ excited state, implying an anti-Kasha behavior of the compounds. The non-doped devices showed EQE_max_ ranging from 12.3 to 14.3%, with small efficiency roll off of 0.8–6% at 1000 cd m^–2^. Using a similar molecular design Liu *et al*.^1140^ reported three AIDF emitters **DCDMF-BP-PXZ**, **DCDPF-BP-PXZ**, and **DCSBF-BP-PXZ** (Figure 165) that have Φ_PL_ of 88.5, 89.0, and 39.6% and λ_PL_ of 540, 530, and 527 nm, respectively, in neat film. The lower Φ_PL_ of **DCSBF-BP-PXZ** was attributed to the relatively poor π–conjugation as well as strong intermolecular π–π interactions. Non-doped OLEDs with **DCDMF-BP-PXZ** and **DCDPF-BP-PXZ** showed EQE_max_ of 19.0% at λ_EL_ of 540 nm and 18.5% at λ_EL_ of 544 nm, respectively. The device with **DCSBF-BP-PXZ** showed a much lower EQE_max_ value of 3.3% (λ_EL_ at 548 nm) due to both the low Φ_PL_ and unbalanced carrier transport within the EML.

Fu *et al*.^1151^ developed AIDF materials **35DCPP-BP-PXZ** and **26DCPP-BP-PXZ** (Figure 165) by integrating an AIDF moiety, 4-(phenoxazin-10-yl)benzoyl, with the bipolar carrier transport materials, 3,5-bis((9H-carbazol-9-yl)-3,1-phenylene)pyridine (35DCPP) and 2,6-bis(3-(9H-carbazol-9-yl) phenyl)pyridine (26DCPP). In neat films these two compounds have Φ_PL_ of 66.5 and 67.9% at λ_PL_ of 530 and 533 nm, respectively. In contrast, and demonstrating their AIE-activity, the respective Φ_PL_ are very low in THF at 2.2 and 2.7%. Non-doped OLEDs with **35DCPP-BP-PXZ** and **26DCPP-BP-PXZ** showed EQE_max_ of 17.3 and 16.1% at λ_PL_ of 538 and 542 nm, respectively. Remarkably, the former device showed a very low efficiency roll-off of 0.6, 7.5 and 16.2%, at 1000, 5000 and 10000 cd m^–2^, respectively, which can be partially attributed to the balanced charge transfer ability embedded within the emitter design.

Zhao *et al*.^1152^ reported an emitter that combines AIDF with enhanced SOC through the incorporation of heavy halogen atoms that leads to faster *k*_RISC._ The three AIDF emitters **3-CCP-BP-PXZ**, **9-CCP-BP-PXZ** and **3,9-CCP-BP-PXZ** (Figure 165) contain the popular PXZ-BP core coupled to suitably halogen-decorated Cz donors. These compounds have Φ_PL_ of 73.0, 70.4 and 72.6% at λ_PL_ of 541, 543 and 536 nm, respectively, as neat films. Exceptionally short τ_d_s ranging from 0.42 to 0.76 μs result from the fast *k*_RISC_ of between 1.73 × 10^6^ – 3.10 × 10^6^ s^–1^, while the control compound without the halogen substituents possesses a longer τ_d_ of 2.10 μs and slower *k*_RISC_ of 0.63 × 10^6^ s^–1^. Non-doped OLEDs with **3-CCP-BP-PXZ**, **9-CCP-BP-PXZ** and **3,9-CCP-BP-PXZ** showed EQE_max_ of 21.7, 20.4 and 20.6% at λ_EL_ of 540, 537 and 541 nm, respectively; the corresponding efficiency roll-offs in the devices were 4.4–8.7% at 1000 cd m^–2^. Replacement of the chloro substituents for bromine produced analogs **3-BCP-BP-PXZ**, **9-BCP-BP-PXZ** and **3,9-BCP-BP-PXZ**.^1153^ These three compounds have slightly attenuated Φ_PL_ of 61.0, 53.4 and 50.7% and modestly blue-shifted λ_PL_ at 521, 531 and 540 nm, respectively in the neat films. Reflecting the lower Φ_PL_, the non-doped devices with **3-BCP-BP-PXZ**, **9-BCP-BP-PXZ** and **3,9-BCP-BP-PXZ** showed EQE_max_ of 19.5, 14.3 and 16.4% with λ_EL_ of 544, 540 and 544 nm, respectively. The efficiency roll-off of these devices was also low at between 3.5–6.1% at 1000 cd m^–2^.

Although AIE is an important property to consider when designing non-doped emitters, ensuring balanced transport and efficient charge recombination is paramount to obtaining efficient devices. Similar to the strategy of Leng *et al*.^1145^, the addition of host-like components to a TADF emitter helped to prevent ACQ and support non-doped device performance for **DCB-BP-PXZ**, **CBP-BP-PXZ**, ***m*CP-BP-PXZ** and ***m*CBP-BP-PXZ** (Figure 165).^1154^ These compounds are poorly emissive in THF solution, with Φ_PL_ of 3.9, 3, 3.1 and 2.8% respectively; however, their neat films showed much higher Φ_PL_ of 69, 71.6, 66 and 71.2%, respectively. Notably, the incorporation of the host-like groups negligibly impacted the λ_PL_, with the compounds displaying nearly identical emission maxima of between 529–532 nm in the neat film. The Δ*E*_ST_ values of the four compounds are around 0.02 eV in the neat film, which was correlated with the short τ_d_ of between 2.3–2.6 μs. Increased delayed emission was also found for aggregates in water-rich THF/water mixtures, demonstrating AIDF. These optical and aggregation properties in turn produced excellent green devices (λ_EL_ = 542–548 nm), with EQE_max_ of 22.6% for the device with **DCB-BP-PXZ** and 21.4% for the device with **CBP-BP-PXZ**. The OLEDs showed low efficiency roll-off of between 9.9 and 11.4% at 5000 cd m^–2^. These excellent results were attributed to the combination of AIDF and ambipolar charge transport in the emitter materials.

The use of symmetric D-A-D emitters also works well to obtain highly efficient non-doped OLEDs. Zhao *et al*.^1155^ reported three AIDF emitters, **SFAC-BP-SFAC**, **SXAC-BP-SXAC**, and **STAC-BP-STAC** (Figure 166) constructed from spiro-acridine-based donors and a benzophenone acceptor. These three compounds have Φ_PL_ of 52–58% at λ_PL_ of 500–511 nm in the neat films. A relatively short τ_d_ of 3.6–4.0 μs linked to the miniscule Δ*E*_ST_ ranging from 36–52 meV were observed for these emitters in the neat films. Non-doped OLEDs based on these emitters showed EQE_max_ ranging from 17.1 to 18.6% at λ_EL_ of between 504–508 nm. The devices with 30 wt% emitter doped in PPF showed higher EQE_max_ ranging from 34.3 to 35.3% due to the preferentially horizontally oriented TDM of the emitter.

Triazatruxene-based **TATC-BP** and **TATP-BP** (Figure 166) exhibited combined TADF, AIE and MCL.^1156^ Although the Φ_PL_ of **TATC-BP** and **TATP-BP** in THF solution are quite low at 0.8 and 1.9%, respectively, these increased considerably to 22.0 and 24.2% in the neat film, which was attributed to their AIE activity (λ_PL_ of 524 and 520 nm, respectively). Solution-processed non-doped OLEDs with **TATC-BP** and **TATP-BP** showed EQE_max_ of 5.9 and 6.0%, respectively; however, the λ_EL_ were red-shifted to 549 and 541 nm, respectively. The doped OLEDs using **H2** (a dendritic oligocarbazole host) showed an enhanced EQE_max_ of 15.9 and 15.4%, respectively for **TATC-BP** and **TATP-BP**. In what is a widely observed trend, the efficiency roll-off for the non-doped OLEDs of **TATP-BP** (3.3%) is much lower than that of many doped devices, likely due to the large number of TADF molecules being able to harvest triplets more rapidly. The efficiency roll-off of the device with **TATC-BP** was 18.6% at 1000 cd m^–2^.

Fusing the benzophenone with an oxygen bridge to give xanthenone (XT), produces a more rigid acceptor that should translate to higher Φ_PL_. Chen *et al*.^1157^ reported the AIDF emitters **BDMAC-XT** and **BDPAC-XT** (Figure 166) that have high Φ_PL_ of 96% at λ_PL_ of 518 nm and 94% at λ_PL_ of 495 nm in the neat film, respectively. Non-doped OLEDs with **BDMAC-XT** and **BDPAC-XT** showed EQE_max_ of 21% at λ_EL_ of 526 nm and 21% at λ_EL_ of 496 nm, respectively. The devices also showed negligible efficiency roll-off where the EQE_1000_ remained remarkably high at 21 and 18%. He *et al*.^1158^ reported two similar blue AIDF emitters, **XT-DPDBA** and **XT-BDPDBA**, composed of a XT acceptor and weak electron-donor 10-dihydrodibenzo[b,e][1,4]azasiline groups. Compounds **XT-DPDBA** and **XT-BDPDBA** have Φ_PL_ of 77 and 86% at λ_PL_ of 472 and 480 nm, respectively, in the neat film. The non-doped OLEDs showed EQE_max_ values of 8.9 and 13.1% at λ_EL_ of 472 and 488 nm, respectively, with corresponding efficiency roll-off at 1000 cd m^–2^ of 10% and 16%. In a similar effort to increase the rigidity of the emitter, Wu *et al*.^1159^ designed **SPBP-DPAC** and **SPBP-SPAC** (Figure 166) containing a carbonyl acceptor with a fused spirofluorene bridging group. Compounds **SPBP-DPAC** and **SPBP-SPAC** have Φ_PL_ of 93 and 98% at λ_PL_ of 495 and 504 nm, in the neat films and non-doped OLEDs showed EQE_max_ of 22.8 and 21.3% at λ_EL_ of 504 and 516 nm, all respectively. Once again and typical of efficient non-doped OLEDs, the devices showed extremely small respective efficiency roll-off of 1.8 and 2.3% at 1000 cd m^–2^.

Fulong *et al*.^1160^ rationally designed a series of AIE-TADF emitters by employing phenyl(pyridyl)methanone as the acceptor moiety that contained intramolecular H-bonding, and compared this to a control phenyl-linked compound where H-bonding cannot occur. Compounds **3CPyM-DMAC** (Φ_PL_ = 66.8%; λ_PL_= 514 nm; Δ*E*_ST_ = 0.04 eV) and **2CPyM-DMAC** (Φ_PL_ = 53.3%; λ_PL_ = 536 nm; Δ*E*_ST_ = 0.03 eV) showed higher Φ_PL_ and smaller Δ*E*_ST_ in the neat film compared to the parent emitter **CBM-DMAC** (Φ_PL_ = 46.7%; λ_P L_= 501 nm; Δ*E*_ST_ = 0.1 eV). Solution-processed non-doped OLEDs with **3CPyM-DMAC** (EQE_max_ = 11.4%; λ_EL_ = 532 nm) and **2CPyM-DMAC** (EQE_max_ = 9.1%; λ_EL_ = 544 nm) showed better performance than the device with **CBM-DMAC** (EQE_max_ = 6.7%; λ_EL_ = 499 nm), demonstrating the effective role that the intramolecular H-bonds may play in enhancing the Φ_PL_ of the emitter – although some doubt remains on this interpretation.^122^

Huang *et al*.^1161^ reported an AIDF emitter based on a new heptagonal diimide acceptor (BPI). **DMAC-BPI** (Figure 166) has a Φ_PL_ of 95.8% at λ_PL_ of 510 nm in the neat film, which decreased to 16.2% in THF, reflecting its AIE activity. In the neat film, **DMAC-BPI** has a τ_d_ of 3.1 μs linked to a small Δ*E*_ST_ of 0.02 eV (determined from toluene solution). The non-doped OLED showed an EQE_max_ of 24.7% at λ_EL_ of 511 nm and had an exceptionally low efficiency roll-off of 1% at 1000 cd m^–2^. Using the same acceptor, these authors also rationally designed **DPAC-BPI-CN**, based on a “medium-ring”-lock strategy, which has a Φ_PL_ of 90.1% at λ_PL_ of 525 nm, a τ_d_ of 3 μs and a Δ*E*_ST_ of 0.35 eV in the neat film. The non-doped device showed an EQE_max_ of 26.2% at λ_EL_ of 531 nm.^476^

Finally, Qi *et al*.^1162^ reported AIE-TADF emitters with dual charge-transfer states (TBCT and TSCT), **DTPA-DTM** and **DTPA-DDTM** (Figure 166). These compounds have moderately large Δ*E*_ST_ of 0.18 and 0.17 eV in toluene yet retain relatively high Φ_PL_ of 38.6 and 60.5% in the neat film, all respectively. The higher Φ_PL_ of **DTPA-DDTM** is due to effective suppression of intramolecular vibrational relaxation, resulting from the enhanced intramolecular D–A interaction with the additional donor. The Φ_PL_ of **DTPA-DTM** and **DTPA-DDTM** in THF are only 8.4 and 5.1%, respectively. Non-doped device of **DTPA-DTM** exhibited green emission with λ_EL_ at 494 nm and a low EQE_max_ of 4.4%, while the device with **DTPA-DDTM** exhibited an EQE_max_ of 8.2% and yellow emission with λ_EL_ at 555 nm, in line with their respective Φ_PL_. Doped devices with **DTPA-DTM** and **DTPA-DDTM** (30 wt% doped in mCP) showed moderately improved performance, with EQE_max_ of 7.1 and 13.6%, respectively.

### 13.4 AIE-TADF Emitters Based on Other Acceptors

The structures of AIE-TADF emitters with other assorted acceptors are shown in Figure 167, and the relevant photophysical and device data are shown in Table S17. Wang *et al*.^1163^ reported two AIDF emitters, **CzTAZPO** and **sCzTAZPO**, composed of carbazole donor dendrons and a triazine acceptor that is decorated with a secondary phosphine oxide acceptor to improve the electron transport properties of the emitters. The two compounds have Φ_PL_ of 71 and 57% and λ_PL_ at 512 and 502 nm, respectively, in the neat film. The non-doped solution-processed OLEDs with **CzTAZPO** and **sCzTAZPO** showed EQE_max_ of 12.8 and 9.6% at λ_EL_ of 537 and 531 nm, with remarkably low efficiency roll-off at 1.8 and 0.97% at 1000 cd m^–2^, all respectively. This level of performance was attributed to their small Δ*E*_ST_ of 0.08 and 0.10 eV and short τ_d_ of 1.1 and 0.81 μs, respectively.

**Figure 167:**
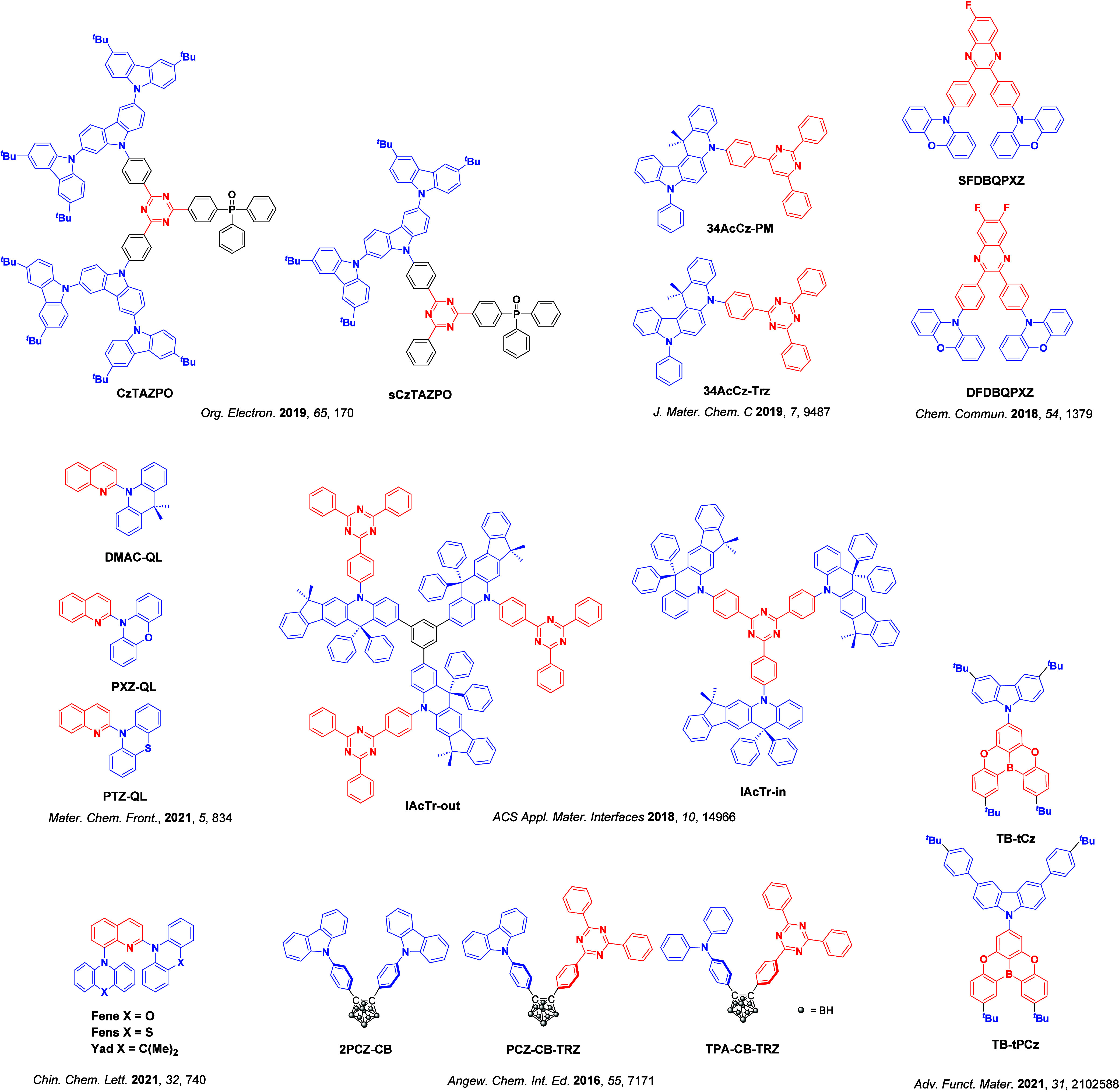
Structures of AIE-TADF emitters based on acceptors other than those containing carbonyl or sulfonyl groups (the blue color signifies donor moieties, while the red color signifies acceptor moieties).

Park *et al*.^1164^ reported two large, three-armed structures, **IAcTr-in** and **IAcTr-out** (Figure 167), composed of triazine and indenoacridine moieties that showed dual AIE and TADF. **IAcTr-in** has a higher Φ_PL_ (64.5%) at λ_PL_ of 525 nm than **IAcTr-out** (Φ_PL_ = 47.7% and λ_PL_ **=** 524 nm). **IAcTr-in** and **IAcTr-out** both have short τ_d_ of 1.6 and 1.3 μs and associated small Δ*E*_ST_ of 0.069 and 0.052 eV as neat films. The non-doped solution-processed OLED with **IAcTr-in** showed an EQE_max_ of 10.9%, increasing to 18.4% in the doped device (35 wt% emitter in mCP). An even more pronounced change in EQE_max_ was observed for the devices with **IAcTr-out**, with the doped device showing an efficiency of 17.5%, while the non-doped device showed an EQE_max_ of only 3.8%. The poor efficiency of the non-doped devices was attributed in part to the lower Φ_PL_ (64.5 *vs*. 47.7%), and mainly to the poorer charge balance in **IacTr-out** associated with its different ratio of donor/acceptor subunits, making charge recombination less favourable.

Zhang *et al*.^1165^ designed AIE-TADF emitters containing a novel acridine–carbazole fused donor, combined with either a pyrimidine or triazine as the acceptor to give **34AcCz-PM** and **34AcCz-Trz** (Figure 167). The compounds have short τ_d_ of 0.64 and 0.75 μs at λ_PL_ of 538 and 556 nm in the neat film, respectively. **34AcCz-PM** has a higher Φ_PL_ of 67% and faster *k*_RISC_ of 8.97 × 10^5^ s^–1^ than **34AcCz-Trz** (Φ_PL_ = 42%; *k*_RISC_ = 1.79 × 10^5^ s^–1^). Consequently, the non-doped device with **34AcCz-PM** showed superior performance with EQE_max_ of 14.1% at λ_EL_ of 548 nm, while the device with **34AcCz-Trz** showed an EQE_max_ of 7.3% at λ_EL_ 576 nm.

Yasuda *et al*.^1166^ reported the three carborane-based AIDF emitters **PCZ-CB-TRZ**, **TPA-CB-TRZ**, and **2PCZ-CB** (Figure 167). In neat film these have Φ_PL_ of 97, 55 and 94% at λ_PL_ of 557, 624 and 571 nm, respectively. Despite the strongly varying Φ_PL_ values, the non-doped devices with **PCZ-CB-TRZ**, **TPA-CB-TRZ**, and **2PCZ-CB** all showed similar EQE_max_ of 11.0, 10.1 and 9.2%, respectively. The emitters **SFDBQPXZ** and **DFDBQPXZ** also showed combined AIE and TADF behavior, having neat film Φ_PL_ of 43.4 and 33.2% at λ_PL_ of 546 and 551 nm, respectively. The corresponding non-doped devices showed EQE_max_ of 10.1 (λ_EL_ = 584 nm) and 9.8% (λ_EL_ = 584 nm). However, the doped OLEDs (10 wt% **SFDBQPXZ** and **DFDBQPXZ** doped in mCP) showed much improved performance due to the much higher Φ_PL_ of 99.6 and 88.3%, giving EQE_max_ of 23.5 and 16.8%, all respectively.^1167^

Three quinoline-based TADF emitters, **DMAC-QL**, **PXZ-QL** and **PTZ-QL** (Figure 167) have moderate Φ_PL_ of 32.6, 64.7 and 52.3%, and emit at λ_PL_ of 489, 531 and 537 nm in the neat films, all respectively.^1168^ Of these, RISC was most efficient in **PXZ-QL**, which has the shortest τ_d_ (1.86 μs) compared to **DMAC-QL** (2.15 μs) and **PTZ-QL** (15.76 μs). The non-doped OLEDs with **DMAC-QL**, **PXZ-QL** and **PTZ-QL** showed EQE_max_ of 7.7, 17.3 and 14.8%, respectively, at λ_EL_ of 522, 536 and 546 nm. With the fastest RISC the efficiency roll-off was most attenuated in the **PXZ-QL** device, with a decrease of only 12% at 1000 cd m^–2^. Zhang and co-workers^1169^ reported similar quinoline-based AIDF emitters, **Fene**, **Fens** and **Yad** that have Φ_PL_ ranging from 36.1 to 79.6% at λ_PL_ ranging from 544 to 591 nm, and small Δ*E*_ST_ ranging from 0.03 to 0.04 eV as neat films. The non-doped OLEDs showed EQE_max_ ranging from 13.1 to 17.4% at λ_EL_ of between 534–570 nm. These results illustrate the potential of quinoline-based AIDF emitters for non-doped OLEDs.

Finally, Kim *et al*.^1170^ reported two blue AIDF emitters **TB-tCz** and **TB-tPCz** bearing organoboron-based cores as acceptors and 3,6-substituted carbazoles as donors. Compounds **TB-tCz** and **TB-tPCz** have Φ_PL_ of 41.4 and 51.9% at λ_PL_ of 433 and 445 nm, respectively, in the neat films. Owing to the closely aligned ^1^CT and ^3^LE states, both emitters exhibit relatively fast k_RISC_ (∼10^6^ s^–1^). Solution-processed non-doped OLEDs with **TB-tCz** and **TB-tPCz** showed EQE_max_ of 8.21 and 15.8% along with narrowband emission, with λ_EL_ at 416 (FWHM = 44 nm) and 428 nm (FWHM = 42 nm), respectively. The higher performance of the device with **TB-tPCz** is due in part to its faster RISC and more efficient upconversion of triplet into singlet excitons.

### 13.5 Outlook

This section has highlighted the recent advances in AIE-TADF and AIDF emitter design, and particularly their application towards non-doped OLEDs. While the majority of AIDF emitters contain sulfonyl- or carbonyl-based acceptors, diverse strategies including asymmetric D-A-D′ configurations, incorporation of intramolecular hydrogen bonding, and integration of host moieties within the emitters have all been explored in efforts to enhance the photophysical performance of the emitter and hence the device performance. Among sulfonyl-containing derivatives, the non-doped device with **2Cz-DPS** showed the highest EQE_max_ of 28.7% at λ_EL_ of 518 nm amongst this family of emitters. Among carbonyl-containing derivatives, the non-doped device with **DPAC-BPI-CN** showed the highest EQE_max_ of 26.2% at λ_EL_ of 531 nm. Though many examples of carbonyl-containing AIE-TADF emitters also employ a phenoxazine donor, it remains at present difficult to identify general design rules for the construction of AIE-TADF emitters.

Promising AIE-TADF or AIDF emitters must show high ΦPL along with small Δ*E*ST and short τd as neat films. However, promising photophysical properties do not always translate to high performance non-doped OLEDs – charge transport is also critical, and difficult to assess from optical measurements alone. AIDF emitters nonetheless provide a promising route to non-doped OLEDs, and frequently show significant resistance to efficiency roll-off at high luminance. We also note that most of the AIDF emitters discussed in this section emit in the blue and green spectral region, while there is an apparent paucity of recognized examples of red/deep red AIDF emitters. This need not be a serious limitation though, as the alternate use of AIDF emitters as hosts and sensitizers for other terminal emitters can readily access longer wavelengths (Section 17 and 18). Ultimately, this progress in the area of AIDF emitters demonstrates the ability of the TADF research community to weaponize apparently inescapable molecular properties (ACQ) and exploit new and unexpected understanding (e.g, the existence of AIE) towards enhanced material properties and performance.

## 14 Excited-State Intramolecular Proton Transfer (ESIPT) Based TADF

### 14.1 Introduction

Excited-state intramolecular proton transfer (ESIPT) is a photochemical process that produces a tautomer with a different electronic structure from the initial ground state.^1171,1172^ ESIPT emission in this context involves the rapid photo-induced tautomerization of a molecule in its electronic excited state and subsequent emission from this second tautomer, or in some cases from both tautomers; the latter case is often described as a dual ESIPT-based emission. The most frequently reported systems are those that show a tautomerization between enol and ketone-type molecules (**A** and **B**, respectively, in Figure 168), with the enol species frequently being the most stable in the ground state and the ketone tautomer the most stable in the excited state. This tautomerization occurs faster than the radiative decay from the vertical excited state, particularly when no other geometric reorganization is required prior to the proton transfer. Hence the radiative decay occurs from the B* species and not from the A* species (Figure 168), each with distinct energy levels and orderings.

**Figure 168:**
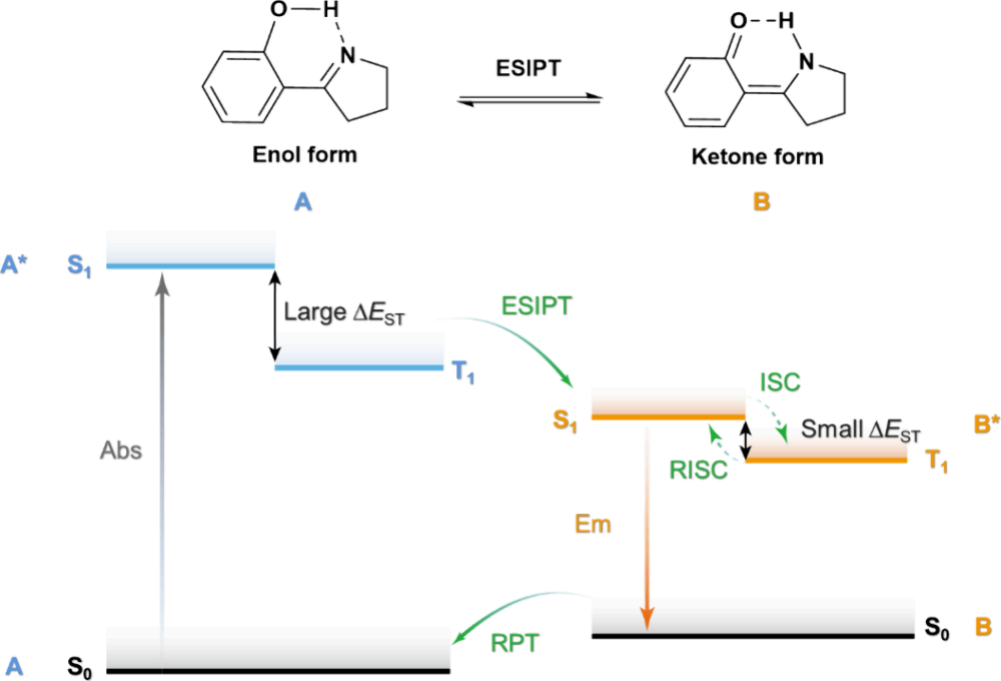
Simplified mechanism of ESIPT emission combined with TADF characteristics; RPT is rapid proton transfer.

Indeed, a key consequence of ESIPT is that the electron density distribution of the frontier molecular orbitals can change significantly between the two tautomeric forms, leading to changes in both the singlet and triplet energies and thus also Δ*E*_ST,_ which can then induce TADF and vary its efficiency.^1172,1173^ Hence, ESIPT-induced TADF represents a distinct, alternative pathway to achieving the well-separated HOMO-LUMO distributions that are established by either highly twisted D-A conformations (See Sections 3–5), by engineering π-stacking interactions between donor and acceptor motifs in either an intermolecular (Section 8) or intramolecular (Section 12) design, or in systems possessing alternating networks of donating and accepting atoms (Section 11). Due to the large electronic effects associated with proton transfer, ESIPT luminescence is characterized by very large Stokes shift (as absorption and emission occur from distinct tautomers) and an emission that can often be tuned via the local environment. Due to these photophysical properties, ESIPT molecules are attractive for fluorescence sensing,^1174,1175^ bioimaging,^1174,1176^ NIR emitters,^1177–1179^ latent fingerprint detection,^1178^ UV absorbers^1180^ as well as for lighting materials.^1181–1184^ A small number of reports exist that use ESIPT-based fluorophores as emitters in OLEDs; however, the performance of these devices is generally poor in part due to their inefficient harvesting of triplet excitons.^1181,1183,1185–1188^

### 14.2 ESIPT Materials Development

In 2007 the first example of a molecule exhibiting both ESIPT and TADF (**HPI-Ac**) was reported and compared with the non-ESIPT derivative (**MeOPI-Ac**), in which the phenolic proton was replaced with a non-labile methyl substituent to prevent the ESIPT (Figure 169 and Table S18).^1189^ Surprisingly, in **MeOPI-Ac** no TADF behavior was observed, which was rationalized by the absence of the phenolic proton and, thus, the inhibition of ESIPT. By contrast, in **HPI-Ac** a delayed lifetime of 25 μs was observed in CHCl_3_, along with a λ_PL_ of 465 nm and a modest Φ_PL_ of 22% (reduced to 18% in air). Unfortunately, no OLEDs were fabricated based on either of these two emitters, although understandably so as this report predated the key work establishing the utility of TADF in OLEDs by several years.^31^

**Figure 169:**
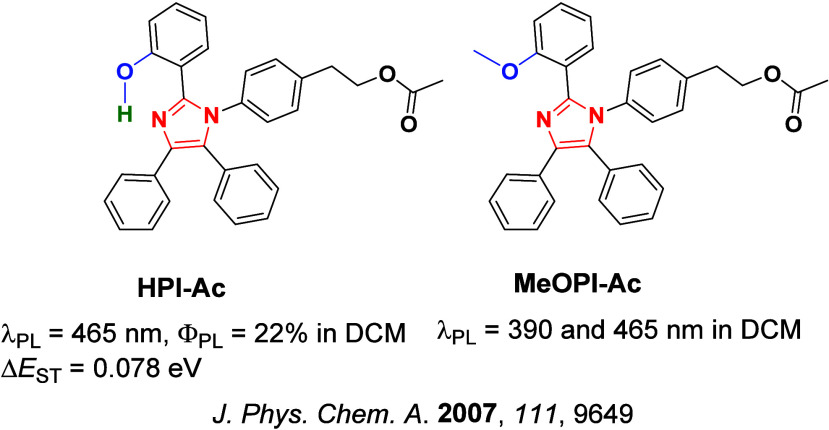
Structures of the ESIPT TADF emitter (**HPI-Ac**) and the fluorescent emitter **MeOPI-Ac**.

Mamada *et al*. reported the first use of a TADF ESIPT emitter not based on a donor-acceptor system, triquolonobenzene (**TQB**, Figure 170), in an OLED.^1190^ **TQB** displayed a λ_PL_ at 516 nm with Φ_PL_ = 55% in 10 wt% CzSi doped film, while the OLED achieved an EQE_max_ of 14% at λ_EL_ of 518 nm. The ground state species (**A**) has a large Δ*E*_ST_ (> 0.5 eV), a consequence of the large spatial overlap of the electron densities of the HOMO and LUMO for this tautomer. ESIPT leads to the formation of B*, a tautomer that has spatially separated HOMO and LUMO orbitals and hence a small Δ*E*_ST_ (< 0.2 eV), which enables RISC to occur (Figure 170 and Table S18). In subsequent studies, Cao *et al*. demonstrated through computations that the proton in **TQB** is transferred within 20 femtoseconds upon photoexcitation, suggesting the direct action of proton transfer itself plays little role in triplet harvesting. However, proton transfer dynamics from **TQB-TA** to **TQB-TB** provides access to multiple triplet states, with a decisive influence on the efficiency of the triplet harvesting (^3^ **TQB-TA** → ^1^ **TQB-TB)**.^1173^ Supporting Cao’s theoretical study, Long *et al*. performed transient absorption and time-resolved photoluminescence studies on **TQB** and demonstrated that the RISC in **TQB** occurs from T_2_ to S_1_, alongside induced absorptions and quenching bands associated with tautomers from secondary and additional proton transfers.^1191^

**Figure 170:**
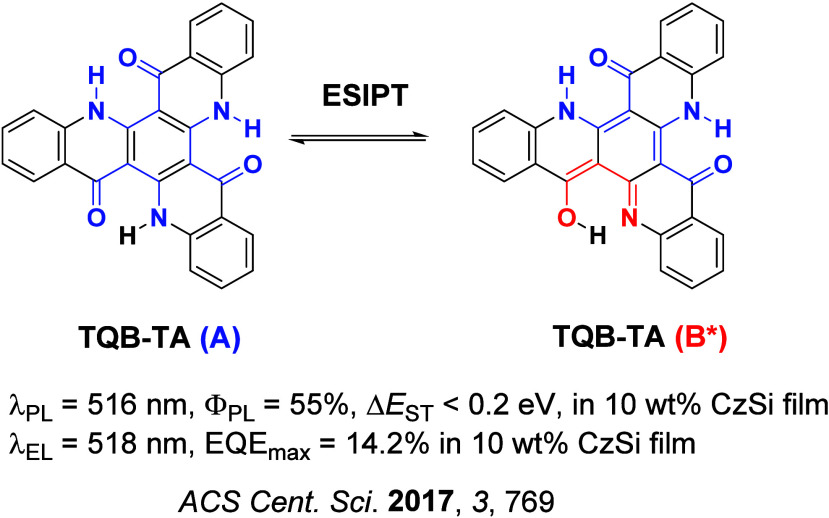
Molecular structure and ESIPT mechanism of **TQB**.

Recently Wu *et al*. reported TADF emitters **PXZPDO** and **DMACPDO**, and claimed that these compounds also showed an ESIPT (Figure 171 and Table S18).^1192^ The symmetry of the two compounds and the presence of the enol tautomer in the ground state, however, preclude ESIPT as an operational mechanism. The vacuum-deposited OLEDs employing these two emitters achieved an EQE_max_ of 18.8% at 560 nm and 23.3% at 536 nm, respectively. In the same report, the control non-ESIPT TADF emitters **PXZDMePDO** and **DMACDMePDO** were also synthesized and used for comparison. The TADF efficiency was not affected by the methylation; however, the EQE_max_ was lower but still high overall (12.2% for **PXZDMePDO** at 544 nm and 14.6% for **DMACDMePDO** at 518 nm). The improved device performance for **PXZPDO** and **DMACPDO** was attributed to the presence of the intramolecular hydrogen bond that was proposed to produce a more rigid structure. As a result, superior Φ_PL_ and *k*_RISC_ could be achieved; for example, **PXZPDO** has a *k*_RISC_ of 1.3 × 10^6^ s^–1^ compared to 2.2 × 10^5^ s^–1^ for the non-ESIPT emitter **(PXZDMePDO)**, in 1 wt% CBP films. Similarly, in 6 wt% CBP films a *k*_RISC_ of 8.8 × 10^5^ s^–1^ for **DMACPDO** and 4.5 × 10^5^ s^–1^ for non-ESIPT emitter (**DMACDMePDO**), was observed. Each of these OLEDs showed a similar efficiency roll-off at 100 cd m^–2^; indeed, only a slight improvement was observed in the efficiency roll-offs of 6, 7, 11 and 18% for the OLEDs using **PXZPDO**, **PXZDMePDO**, **DMACPDO** and **PXZMePDO**, respectively (Table S18).

**Figure 171:**
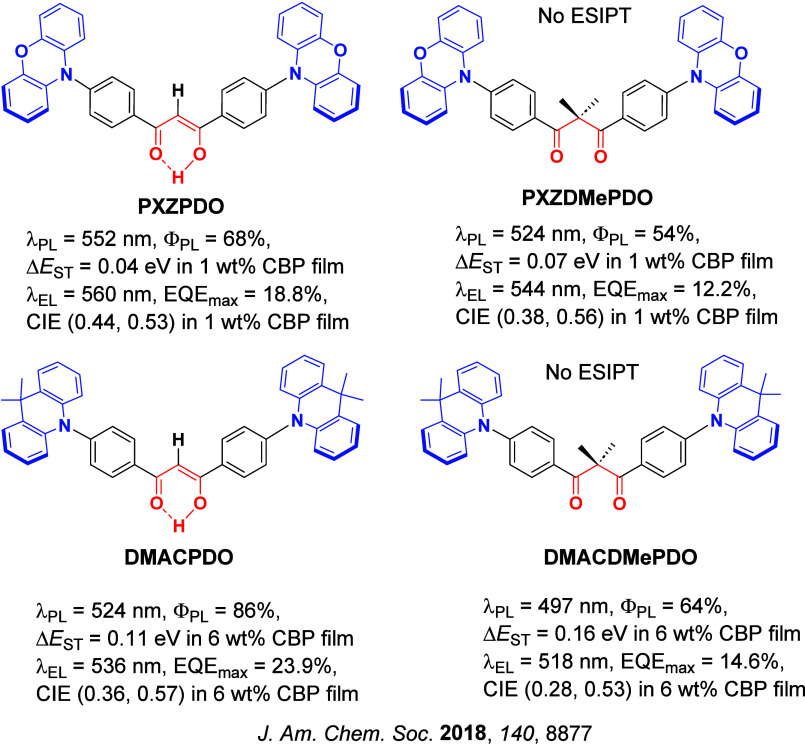
Molecular structure of the TADF based ESIPT and non-ESIPT emitters reported in ref 1192 (the blue color signifies donor moieties, while the red color signifies acceptor moieties).

Inspired by this work, Gupta *et al*. reported a new ESIPT-based TADF emitter (**TPXZBM**) that contains phenoxazine donor groups in combination with a *β*-triketone – a stronger acceptor moiety than the one found in **PXZPDO**.^1193^ The molecular design produced a more acidic methine proton, which pushed the equilibrium position in the ground state towards the presence of both tautomers, unlike that observed for **PXZPDO** (Figure 172 and Table S18) where only the enol tautomer was observed by ^1^H NMR spectroscopy. ESIPT was observed for **TPXZBM**, which showed a red-shifted emission at 650 nm in comparison to **PXZPDO** (604 nm) in toluene. Cross-comparison of the optoelectronic properties and OLED device performance using this compound revealed significant differences to those of **PXZPDO** and of the *β*-tetraketone non-ESIPT reference emitter, **BPXZBM**. The latter compound exists as only one tautomer due to the absence of an enolizable proton but retains TADF activity, presumably arising through its D-A-D structure. The emitter **TPXZBM** showed both ESIPT and TADF, with the enol tautomer dominant in the excited state, resulting in a Δ*E*_ST_ of 0.020 eV, Φ_PL_ of 30% and *τ*_d_ of 1.44 μs in 1 wt% CBP host. The solution-processed OLEDs of **TPXZBM** showed an EQE_max_ = 12.7% at 582 nm with a low efficiency roll-off (the EQE at 10,000 cd m^–2^ reached 4.7%), while for **PXZPDO**, a much better device performance was observed (EQE_max_ = 20.1%, comparable to thermally evaporated devices of **PXZPDO** previously) with low efficiency roll-off (the EQE at 10,000 cd m^–2^ reached 12.7%). The non-ESIPT control emitter **BPXZBM** showed poor Φ_PL_= 17% and a *τ*_d_ = 1.01 μs in 1 wt% in 1 wt% CBP, and thus the device performance suffered, with an EQE_max_ of 7% at 598 nm. This was also the first report of a solution-processed ESIPT-based TADF OLED.

**Figure 172:**
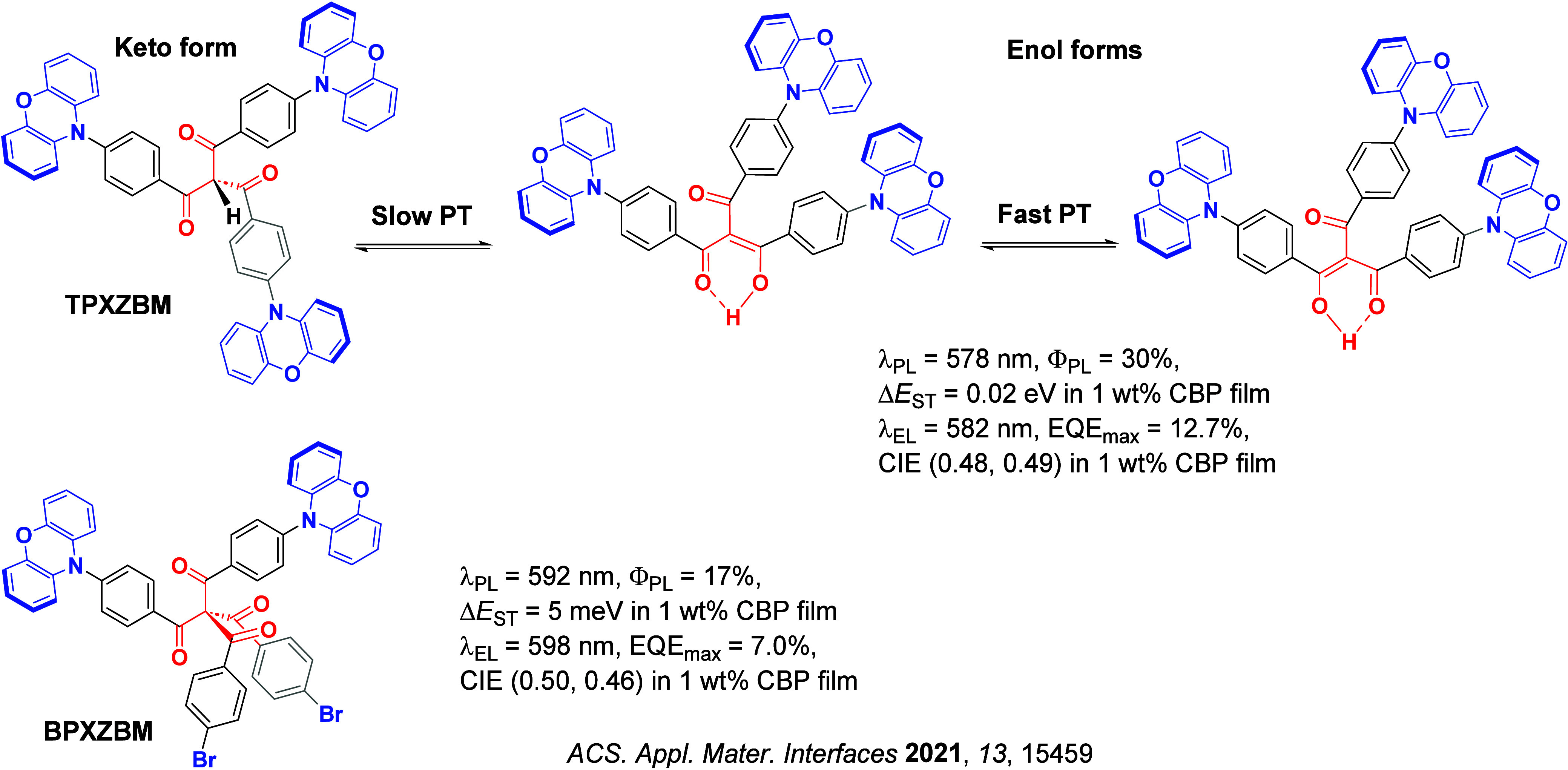
Molecular structure and ESIPT mechanism of the TADF ESIPT emitter **TPXZBM** and the non-ESIPT TADF emitter **BPXZBM** reported in ref 1193 (the blue color signifies donor moieties, while the red color signifies acceptor moieties).

The high sensitivity of ESIPT emitters to their surrounding environment inspired Kim and co-workers to devise ESIPT-based compounds that could switch between room temperature phosphorescence (RTP) and TADF, depending on the substitution about the core structure.^1194^ The introduction of both an aromatic carbonyl and an adjacent bromo substituent to (2′-hydroxyphenyl)benzimidazole (**HBI**), as in **BrA-HBI** (Figure 173), increased the SOC and resulted in RTP from the enol form. In contrast, the keto form of **BrA-HBI** exhibited a mixture of prompt fluorescence and TADF with λ_PL_ of 450 nm, Φ_PL_ that grew from 10 to 31% upon degassing, and with an associated *τ*_d_ of 1.90 ms in 1 wt% PMMA doped film at room temperature. At 77 K, the same **BrA-HBI** film showed a new emission band at around 505 nm, which was assigned to phosphorescence (τ_ph_ ≈ 13 ms) from the enol form of **BrA-HBI**, and the Δ*E*_ST_ was measured to be 0.31 eV. The phosphorescence from the enol-form was further confirmed by doping 1 wt% **BrA-HBI** in polyacrylic acid (PAA), which can inhibit intramolecular proton transfer through competitive intermolecular hydrogen bonding. The non-ESIPT control molecule “methylated **BrA-HBI**” showed similar photophysical behavior to the enol form of **BrA-HBI**. In contrast, the non-functionalized parent molecule **HBI** showed high Φ_PL_ of 70%, but no delayed emission. However, the authors assigned the emission of aldehyde-substituted **A-HBI** to be TADF (albeit with a reduced Φ_PL_ of 53%), while **Br-HBI** mostly showed phosphorescence with Φ_PL_ of 23% independent of temperature (Figure 173). The authors then used **Br-HBI** in a photochromic photo-patterning system, and as hydrogen chloride vapor detection system with optical readout.

**Figure 173:**
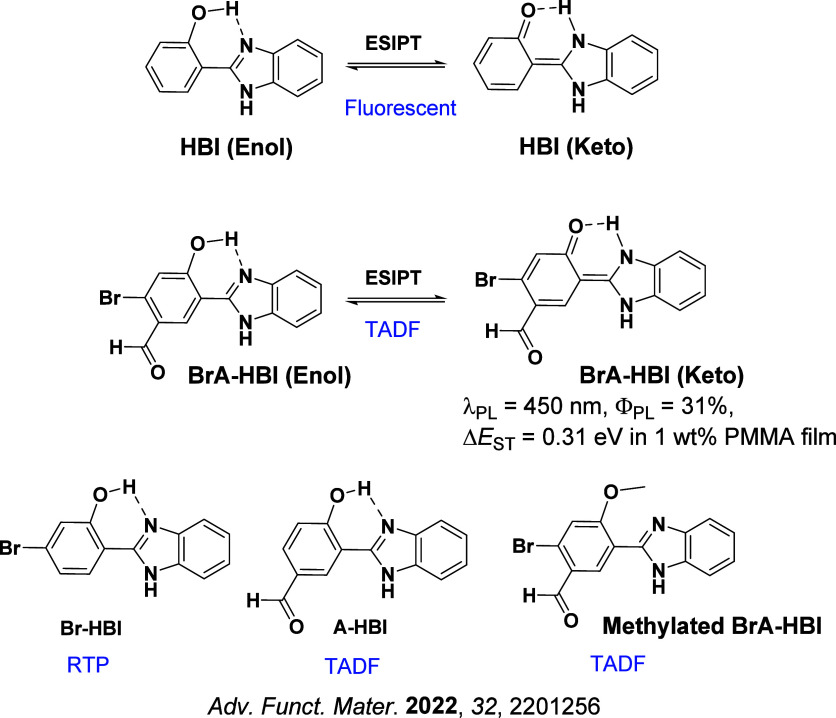
Molecular structures of the emitters **HBI**, **BrA-HBI**, **Br-HBI**, **A-HBI** and **methylated BrA-HBI**.

Berezin *et al*. demonstrated TADF behavior in the pyrimidine-based ESIPT ligand 2-[6-(3,5-dimethyl-1*H*-pyrazol-1-yl)pyrimidin-4-yl]phenol (**HL**) and its Zn complex **[Zn(HL)]Cl_2_** (Figure 174).^939^ The **HL** ligand features a short O-H···N intramolecular H-bond (O···N ca 2.6 Å) that enables the ESIPT, and a separate *N,N*-chelating pocket for binding metal ions. Complex **[Zn(HL)]Cl_2_** showed excitation wavelength-dependent emission, ESIPT, and TADF, while **HL** alone also showed both ESIPT and TADF. DFT calculations revealed that the presence of the Zn^2+^ ions facilitate S_2_ → T_2_ → T_1_ and S_2_ → T_1_ ISC. The neat powder of **HL** emits with λ_PL_ of 555 nm; however, **[Zn(HL)Cl_2_]** showed emission at 640 nm which shifted to 565 nm on changing the excitation wavelength from 420 to 480 nm. Compound **HL** showed a short τ_p_ of 2 ns and a τ_d_ of 890 μs at 300 K, the latter of which increased to 1500 μs at 220 K. The DFT calculated a small Δ*E*_ST_ of 0.10 eV, which explains the TADF behavior of **[Zn(HL)Cl_2_]**. Based on a theoretical study, the authors suggested that the ESIPT process in both compounds is barrierless and results in an abnormal anti-Kasha fluorescence (S_2_ → S_0_) and anti-Kasha phosphorescence (T_2_ → S_0_) associated with relatively low S_2_ → S_1_ and T_2_ → S_1_ internal conversion rates.

**Figure 174:**
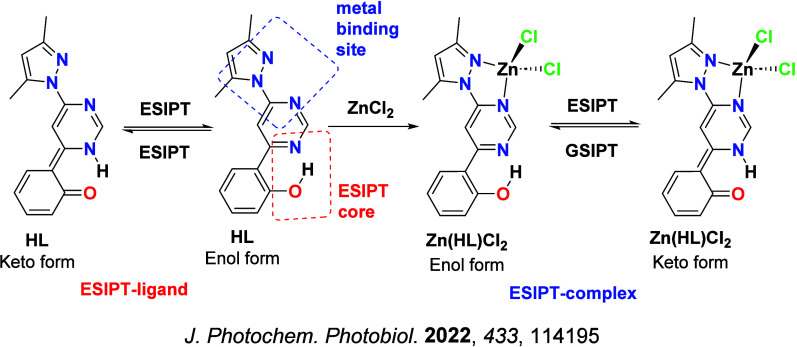
Excited-state intramolecular proton transfer (ESIPT) and ground-state intramolecular proton transfer (GSIPT) mechanisms operational in **HL** and **Zn(HL)Cl_2_**.

### 14.3 Outlook

The examples summarized in this section reveal that it is possible to design molecules in which ESIPT supports TADF. The ESIPT process paves the way for HOMO and LUMO separation and a small singlet-triplet gap, in a way that is fundamentally distinct from the strongly twisted conformation adopted in most D-A TADF emitters. For example, **TQB** is a compound far outside the donor-acceptor design paradigm (Figure 170), which was employed in green OLEDs that showed an EQE_max_ of 14.2%. Looking to the future, it is an open question whether the relatively small molecular reorganisation energies associated with ESIPT might eventually enable faster RISC rates than the large-amplitude dihedral motions associated with vibronic coupling in D-A TADF emitters.

ESIPT-active chromophores can also be flexibly deployed in hybrid designs, for example combined with electron-donating fragments in **PXZPDO**, **DMACPDO**, and **TPXZBM** (Figure 171). These display ESIPT in their acceptor moiety and each have small Δ*E*_ST_, which translated to devices with higher efficiency than non-ESIPT counterparts. The large Stokes shifts inherent in ESIPT-based emitters may also be harnessed towards the design of deep-red emitters. Especially considering their unique RISC pathway, we find it surprising that ESIPT materials have not received more research attention. Although impossible to predict, it may well be that a few high-performance materials – potentially discovered just outside the small regions of chemical space currently explored – could ignite global efforts and rapid development in this area.

## 15 Mechanochromism/Mechanoluminescence and TADF

### 15.1 Introduction

Mechanochromism and mechanochromic luminescence (MCL) involve changes of the emission spectrum and color of a material when external force is applied. This is distinct from triboluminescence, in which mechanical force directly causes the emission of light. The applied force in this context produces a change in the bulk material, usually in the packing arrangement such as a transition from the crystalline to the amorphous state or a crystal-to-crystal phase transition, which impacts the electronic structure of the molecules and hence their emission color.^1195^ The force that triggers these changes in packing can be applied physically, such as by shearing and grinding, indirectly through heating, or involve various crystallization techniques including changing the solvent system or exposure to solvent vapor. Some of these structural changes can be reversible, resulting in switching behavior that is valuable in sensing and other applications.

Because of their sensitivity to D-A molecular geometries, as exemplified in emitters throughout Sections 3–5, mechanochromism has been observed in a number of D-A TADF materials, which are summarized in this section. In some examples the excited-state decay mechanism may change entirely depending on the packing arrangement, for example switching from TADF to fluorescence; however, most reports neglect to probe the operational emission mechanism of each of the different morphologies. Most of the reported examples also exhibit both AIE and MCL, with both properties arising from changes in molecular geometry.^1196^ To date, there are only a few reports of TADF materials that have been observed to be mechanoresponsive (Figure 175–Figure 177). A subset of these have also been used as emitters in OLEDs. Table S19 summarizes materials and their photophysical properties for which no OLEDs were fabricated, while Table S20 collates TADF compounds that show MCL and which were also used in or towards OLED applications.

**Figure 175:**
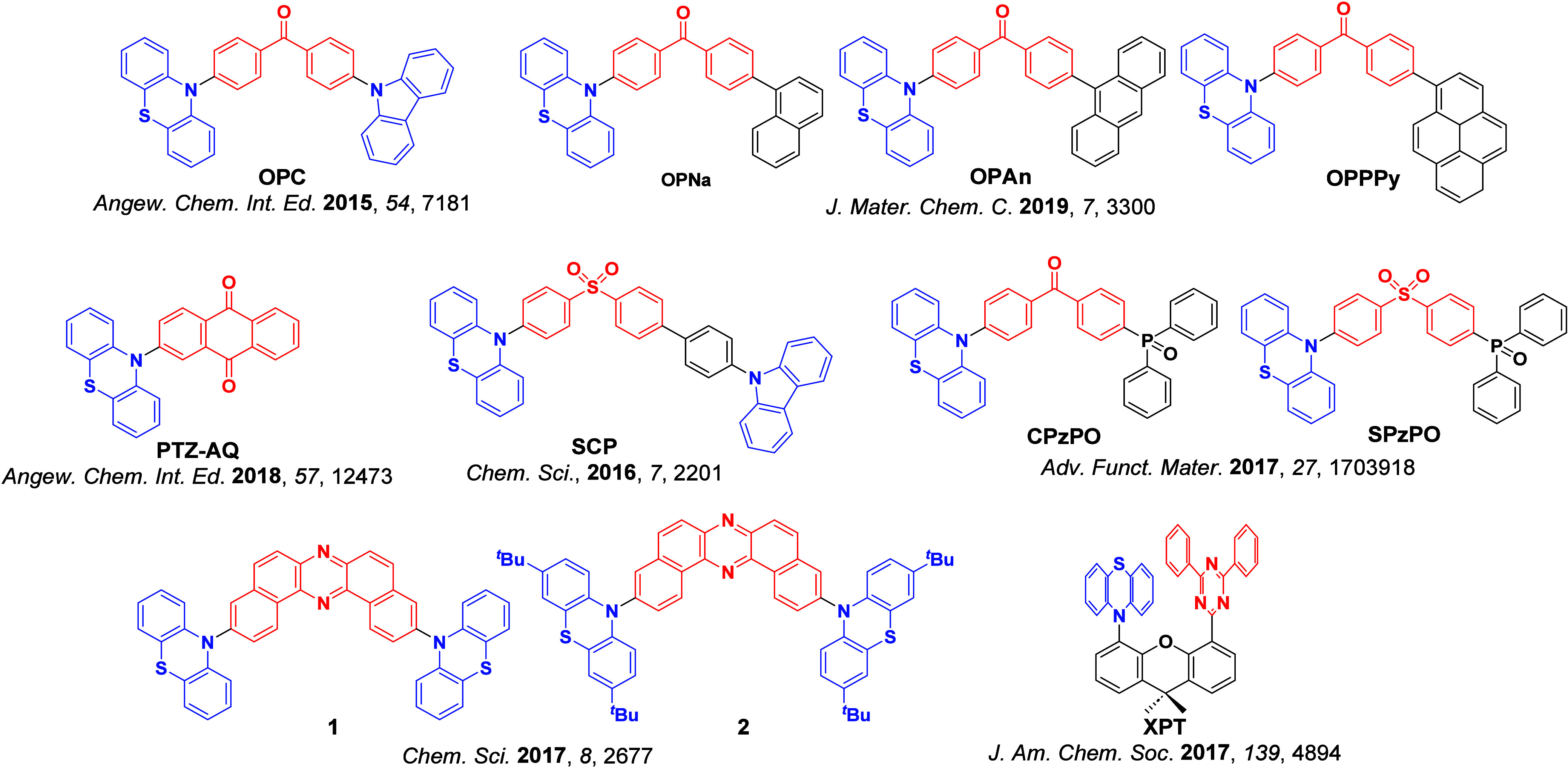
Structures of MCL emitters containing PTZ donors (the blue color signifies donor moieties, while the red color signifies acceptor moieties).

### 15.2 Materials Development

The first reported example of a TADF emitter undergoing MCL was the D-A-D′ compound **OPC** (Figure 175). It displayed dual-channel white emission with bands at 456 nm and 554 nm, the latter of which exhibited TADF.^604^ The dual emission was found to be due to the coexistence of two different excited-state conformers associated with quasi–axial or quasi–equatorial conformations that are now commonly observed for the PTZ donor group. The emission at 456 nm is from the quasi-axial conformer that has a calculated Δ*E*_ST_ of 0.56 eV, while the emission at 554 nm is from the quasi-equatorial conformer with a calculated Δ*E*_ST_ of 0.01 eV. Upon grinding the quasi-axial conformer was converted to the quasi-equatorial conformer, culminating in exclusive emission from this lower energy species in the ground powder. In single crystals the quasi-equatorial conformer is the dominant species, with two emission bands once again observed.

Using a similar D-A design, Xie *et al*. reported dual emission for a series of PTZ-ketone emitters,^1197^ where additional π-conjugated groups such as naphthalene (**OPNa**), pyrene (**OPPy**) and anthracene (**OPAn**) were also coupled to the acceptor (Figure 175). In the crystalline state each compound displayed dual emission, with a high-energy high-intensity fluorescent band at between 429–454 nm. A second low-energy band located at around 570–587 nm was assigned to arise from TADF. Upon grinding the crystals, the high-energy band decreased in intensity and the low-energy band dominated the emission spectrum. This spectral change was assigned to increased intermolecular hydrogen bonding between the donor and acceptor components across neighboring molecules, turning on an intermolecular CT transition in the ground powder state. This CT state and low-energy emission band was indeed found to be TADF-active for **OPNa**, **OPPy** and **OPAn**. Additionally, these bulky groups proved to be essential to achieve MCL, as with just phenyl substitution only the low-energy intermolecular CT band was present, even in the crystal.

The D-A compound **PTZ-AQ** (Figure 175) displays five different crystal morphologies, each with different photophysical properties.^1198^ The five morphologies were described by their yellow, orange or red color and labelled as Y-Solid, Y-crystal, O-Crystal, R-crystal and R-solid by the authors, with λ_PL_ of 545, 554, 568, 606 and 649 nm, respectively. Each of the samples was obtained using different crystal growth techniques, while heating of the R-solid yielded the Y-solid. The reverse transition (Y-solid to R-solid) was possible through exposure to CH_2_Cl_2_ vapours. Color changes in the crystals were understood to arise from alteration in the π–π interactions in these systems. Interestingly, each solid displayed distinct Δ*E*_ST_ and Φ_PL_ values, with Δ*E*_ST_ varying between 0.01 and 0.42 eV and Φ_PL_ varying between 3 and 85% (Table S19). TADF was observed in the Y-Solid, Y-crystal, O-Crystal and R-crystal, while R-solid showed no TADF owing to its larger Δ*E*_ST_ of 0.42 eV (0.01–0.25 eV for the others).

Two phosphine oxide-containing emitters, **CPzPO** and **SPzPO** (Figure 175), showed dual emission in the crystalline state with λ_PL_ of 459 and 564 nm for **CPzPO** and 433 and 546 nm for **SPzPO**.^1199^ The two emission bands displayed different emission mechanisms, with the lower energy bands showing TADF and τ_d_ of 62 and 29 μs, for **CPzPO** and **SPzPO** respectively. The higher energy bands were simply fluorescent in both materials. Upon grinding to an amorphous state, the intensity of the higher energy fluorescence band decreased. The contrasting intensities and TADF behaviour of the high and low energy bands were a result of changes in the packing arrangements, with intermolecular hydrogen bonding becoming more prominent in the ground species. Similar to the previous examples with OPC and related emitters, this enhancement of intramolecular interactions was proposed to be responsible for the enhancement of the low-energy TADF-active emission channel.

A similar effect was reported by Xu *et al*. for the emitter **SCP** (Figure 175),^1200^ where again dual emission was observed in the pristine form. Emission bands at 415 nm and 545 nm were observed, where emission from the high-energy band is purely fluorescent while emission from the low-energy band is TADF-active. Upon grinding, the intensity of the two peaks changed, with the longer wavelength TADF band dominating the spectrum, resulting in a significant color change. The ratio of these emission bands could be tuned to achieve white light emission. The high-energy band at 415 nm was assigned to the Cz-Ph → sulfone transition, while the emission at 545 nm was attributed to the PTZ → sulfone transition. Two contrasting calculated Δ*E*_ST_ values of 0.99 eV (Cz-Ph CT state) and 0.44 eV (PTZ CT state), explain the differences in TADF properties, with TADF only observed in the latter despite the relatively large Δ*E*_ST_. The Cz-Ph conformation was proposed to planarize upon grinding, affecting the photophysical properties associated with this fragment and increasing the probability of energy transfer to the PTZ-centred excited CT state associated with the low-energy band, which then dominates emission.

Once again exploiting the two accessible conformers of PTZ,^1201^ two MCL compounds using nitrogen-rich acceptors were developed by Okazaki *et al.*, (**1** and **2**, Figure 175), which emitted in the green and deep red, respectively. Using different solvent systems, distinct yellow or orange crystals of 1 were grown (1_Y and 1_O) with λ_PL_ of 568 nm and 640 nm, respectively. Upon grinding of either 1_Y or 1_O, a different red-emitting form 1_R was generated with λ_PL_ of 673 nm. Thermal annealing of 1_R produced 1_O2 (λ_PL_ of 646 nm), while exposure of 1_R to CH_2_Cl_2_ generated 1_YO (λ_PL_ of 646 nm). Grinding of either of 1_O2 or 1_YO reformed 1_R. The substantial color changes for **1** with different processing conditions were explained as a result of changing one or both of the PTZ conformation in each crystalline form, with 1_Y and 1_YO composed of axial-axial donors, 1_O2 and 1_O having axial-equatorial PTZ conformations, and 1_R being equatorial-equatorial (Figure 176). Compound **2** has a similar structure but with -^*t*^Bu substitution on the PTZ donors and showed a total of four colored forms. 2_YG (λ_PL_ of 547 nm) was obtained from recrystallization from hexane:​CHCl_3_, 2_R (λ_PL_ 663 nm) was obtained from grinding of 2_YG, heating of 2_R to 240 °C formed 2_R2, while exposure of 2_R to CH_2_Cl_2_ vapor resulted in the formation of 2_Y. All generated samples reverted to 2_YG upon recrystallization from hexane:​CHCl_3_. Both emitters **1** and **2** displayed TADF when doped at 10 wt% in CBP films, and efficient devices with EQE_max_ of 16.8% and 11.2% for the OLEDs with **1** and **2**, respectively were demonstrated.

**Figure 176:**
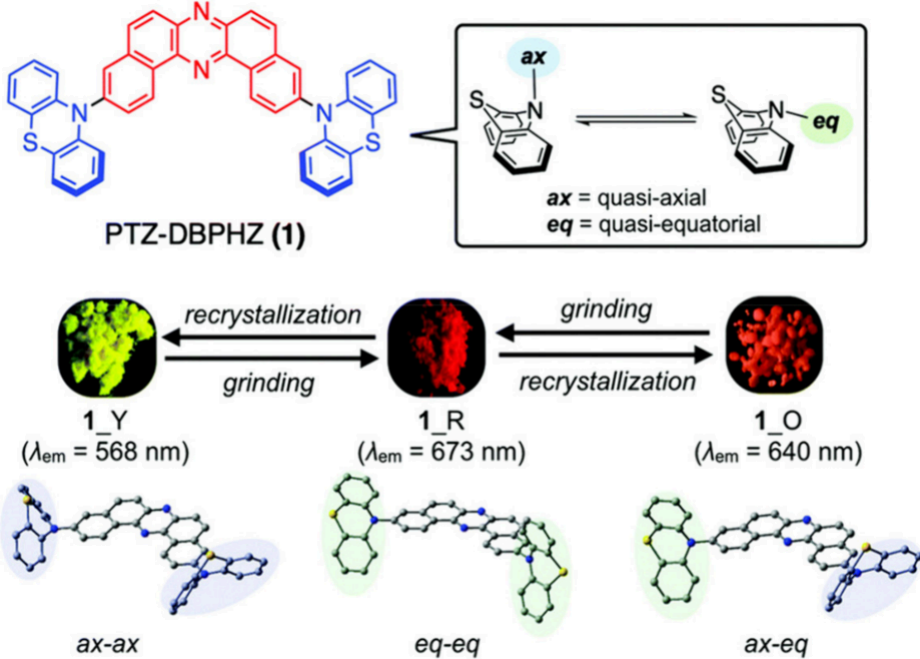
MCL properties of **PTZ-DBPHZ (1)**. Taken and adapted with permission from ref 1202. Copyright [2019/Journal of Materials Chemitry C] Royal Society of Chemistry.

A TSCT emitter, **XPT** (Figure 175),^1098^ was also reported to undergo MCL. A red-shift of the emission from λ_PL_ of 536 nm to 569 nm was observed upon grinding of the single crystal to form a powder. Sublimation of **XPT** produced a similar spectral shift with λ_PL_ of 566 nm, similar to the powder form. In both ground and sublimed samples the original λ_PL_ of 536 nm could be reconstituted upon exposure to CH_2_Cl_2_ vapor. Compound **XPT** was used as the emitter in an OLED (EML: 10 wt% **XPT** in DPEPO) that showed an EQE_max_ of 10%. Although TADF was claimed in this report, it is not clear whether the TADF was also observed in the powder samples displaying MCL.

The same strategy of using axial and equatorial conformation changes to induce MCL was employed using a phosphine derivative of PTZ in the compound **DPPZS-DBPHZ** (Figure 177).^1203^ This compound showed strong color tuning from 496 nm to 704 nm between different conformers. Reversible color tuning was also demonstrated by recrystallization of the 1-BG conformer from four other accessible conformers, themselves accessed by various combinations of grinding, heating, or solvent vapor fuming. The conformers 1-BG, 1-G1, 1-G2, 1-Y and 1-DR emitted at λ_PL_ of 497, 518, 520, 534 and 740 nm (and with Φ_PL_ of 6%, 9%, 9%, 16% and 3%) respectively. Different combinations of equatorial and axial donors were responsible for the different emission colors, with 1-BG equatorial-equatorial, 1-Y axial-equatorial, and 1-G1 and 1-G2 ascribed to be axial-axial with potential axial-equatorial conformers also present. The decay mechanism of each conformer was not investigated, although the compound in a 10 wt% Zeonex matrix showed both TADF and RTP.

**Figure 177:**
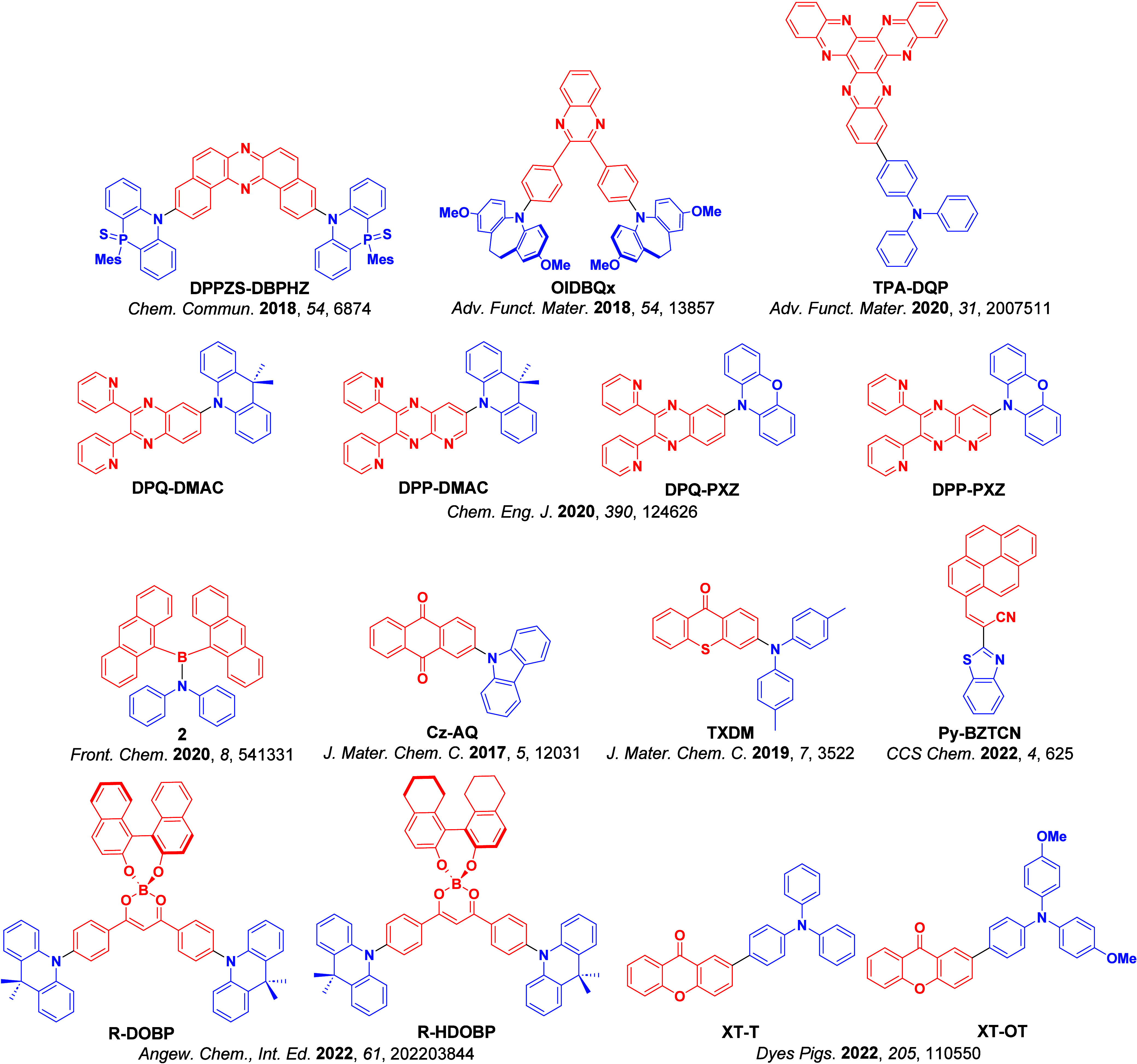
Structures of TADF materials undergoing MCL (the blue color signifies donor moieties, while the red color signifies acceptor moieties).

Pashazadeh *et al.*, documented MCL in samples of **OIDBQx** (Figure 177),^1204^ where the λ_PL_ of the powder red-shifted from 494 to 522 nm upon grinding. The original emission was restored upon exposure to CH_2_Cl_2_ vapor. Only fluorescence was observed for this compound in the powder forms, although both TADF and RTP were present in 1 wt% Zeonex films with an average delayed lifetime of 128 ms.

A series of materials presenting reversible MCL properties was reported by Yang *et al.*, composed of a combination of planar acceptors (DPP and DPQ) and donors (DMAC and PXZ).^1205^ **DPP-DMAC**, **DPQ-DMAC**, **DPP-PXZ** and **DPQ-PXZ** (Figure 177) were each ground, fumed with CH_2_Cl_2_ or heated to achieve distinct color changes in each example. Switching between the colors was shown to be completely reversible for each emitter. Following grinding, a loss of crystallinity was observed and the materials became amorphous, while subsequent fuming and heating produced new crystalline packing motifs compared to the original sample. The most significant color change occurred following grinding, where a red-shift of the emission was observed from 554 to 608 nm, 548 to 571 nm, 589 to 616 nm and 628 to 682 nm for **DPP-DMAC**, **DPQ-DMAC**, **DPQ-PXZ** and **DPP-PXZ**, respectively. In each solid-state environment a delayed emission component was observed in the μs regime, with lifetimes ranging from 1.1 to 8.3 μs and assigned to TADF. Using **DPQ-DMAC** the authors demonstrated ‘ink-free rewritable paper’, where with the application of mechanical pressure it was possible to write text using the color change of the material. Upon exposure to CH_2_Cl_2_ vapor it was also possible to restore the color of the ‘written’ material, thus deleting the text. The potential of **DPQ-DMAC** as an emitter for OLEDs was also assessed, with the material doped at 10 wt% in DPEPO showing an EQE_max_ of 11.3% at 556 nm, and exhibiting low efficiency roll-off (EQE_100_ of 10.5%).

Another D-A system (material **2**, Figure 177) composed of diphenylamine as the donor and a boron atom linked to two anthracene units as the acceptor was reported by Pandey *et al.*.^1206^ This compound displayed dual emission as pristine powder with λ_PL_ of 455 and 530 nm. Upon grinding, only a single peak centred at around 540 nm remained. Exposing this new form to CH_2_Cl_2_, CHCl_3_ or hexane vapor though restored the original dual emission. No explanation was offered for this behavior, however different powder XRD patterns were observed for each form. The compound also displayed dual emission in PhMe solution, with both emission peaks at 430 and 530 nm being TADF-active, with τ_d_ of 5.9 and 5.8 μs.

MCL was observed for the emitter **TPA-DQP** (Figure 177), which showed two distinct polymorphs. Crystal-Y and Crystal-R have λ_PL_ of 576 and 694 nm, respectively, and Crystal-Y was identified as the thermodynamic product.^1207^ For Crystal-Y, CH−π interactions between the acceptor and the donor as well as π–π stacking between the acceptor groups were identified, while for Crystal-R, the packing structure was composed entirely of π–π stacking between the acceptor units. Upon grinding Crystal-Y the emission red-shifted from 576 to 698 nm, and the emission at 576 nm could be restored upon heating the sample. For Crystal-R, the color shifts were much less pronounced, with grinding red-shifting λ_PL_ from 694 to 706 nm, which was restored to 694 nm upon CH_2_Cl_2_ fuming. MCL observed in both polymorphs was rationalized as due to transferring from crystalline to an amorphous packing arrangement. Both crystals displayed prompt and delayed emission assigned as TADF, with τ_d_ of 1.1 and 2.4 μs for the Y and R-crystals, respectively. TADF was also observed in 10 wt% doped films in Bepp2, with a corresponding small Δ*E*_ST_ of 0.11 eV. OLEDs fabricated with **TPA-DQP** showed an EQE_max_ of 18.3% at CIE coordinates of (0.67, 0.32).

For crystalline **Cz-AQ**,^1208^ two emission peaks with λ_PL_ of 604 and 541 nm were documented corresponding to two distinct crystal packing regimes (R-crystal and Y-crystal, Figure 177). The red-shifted emission was associated with a morphology featuring strongly π–π overlapped H-aggregates, while the higher energy band was linked to weaker J-aggregates. Both R-crystal and Y-crystal were interconvertable, with heating of R-crystal producing Y-crystal, while grinding or haloalkane fuming of Y-crystal recovering R-crystal. TADF was observed for both Y-crystal and R-crystal forms with τ_d_ of 1.8 and 1.9 μs while the Φ_PL_ were 59 and 28%, respectively. The different packing regimes were subsequently exploited in solution-processed OLEDs. When dichloroethane was used to spin-coat the films, a device λ_EL_ of 680 nm was observed, while when a dichloro­ethane:​ethanol (1:1) solution was employed, λ_EL_ was 600 nm. The EQE_max_ of the non-doped devices were low at 0.75 and 1.15%, respectively, and while doped devices showed higher EQE_max_ they did not have the color tuning potential of the non-doped devices. The change in both the λ_EL_ and the λ_PL_ was attributed to different aggregation states in the neat thin films, analogous to the Y-crystal and R-crystal forms.

A similar derivative using DPA as the donor and thioxanthone as the acceptor, **TXDM** (Figure 177), was reported by Mane *et al.*. Exploiting both the MCL and oxygen sensitivity of this material, this work reported a logic gate based on the PL of this compound.^1209^ Supporting this application, starkly contrasting photophysical properties were obtained for different morphologies, with the crystalline form of **TXDM** showing significantly quenched emission and no TADF (λ_PL_ of 470 nm, Φ_PL_ of 1.8%). In the amorphous state the emission was much brighter (λ_PL_ of 486 nm, Φ_PL_ of 27%) and exhibited TADF. These changes in the photophysics were ascribed to suppression of π–π stacking interactions in the amorphous state. Δ*E*_ST_ was 0.30 eV for the amorphous form and increased to 0.42 eV for the crystal, with these differing Δ*E*_ST_ responsible for the contrasting TADF activity. MCL was achieved upon grinding, heating, and fuming, with each of these external forces accessing a different output in the logic gate system.

Zhou *et al*. reported two pairs of enantiomeric emitters,^653^ each containing a tetracoordinate boron acceptor, and chiral binaphthol or octahydro-binaphthol and DMAC donor groups. ***R/S*-DOBP** and ***R/S*-HDOBP** (Figure 178), showed multifunctional properties, including CPL, mechanochromism, and piezochromism (Figure 178). A significant spectral change was observed for ***R*-DOBP** upon grinding, with the emission red-shifted from 580 nm for the crystalline sample to 647 nm for the ground form. Application of pressure from 0 to 5.9 GPa the crystalline ***R*-DOBP** in a diamond anvil cell also led to a red-shifted emission with increased intensity. A further red-shift of the emission accompanying a gradual decrease of its intensity was observed when the pressure exceeded 5.9 Gpa. Interestingly, the emission spectrum associated with atmospheric pressure could be gradually recovered when the pressure was released. By contrast, ***R/S*-HDOBP** did not show any mechanochromism. The authors also fabricated solution-processed non-doped near infrared OLEDs that showed EQE_max_ of 1.9 and 0.7% with λ_EL_ of 716 and 700 nm using ***R*-DOBP** and ***R*-HDOBP**, respectively.

**Figure 178:**
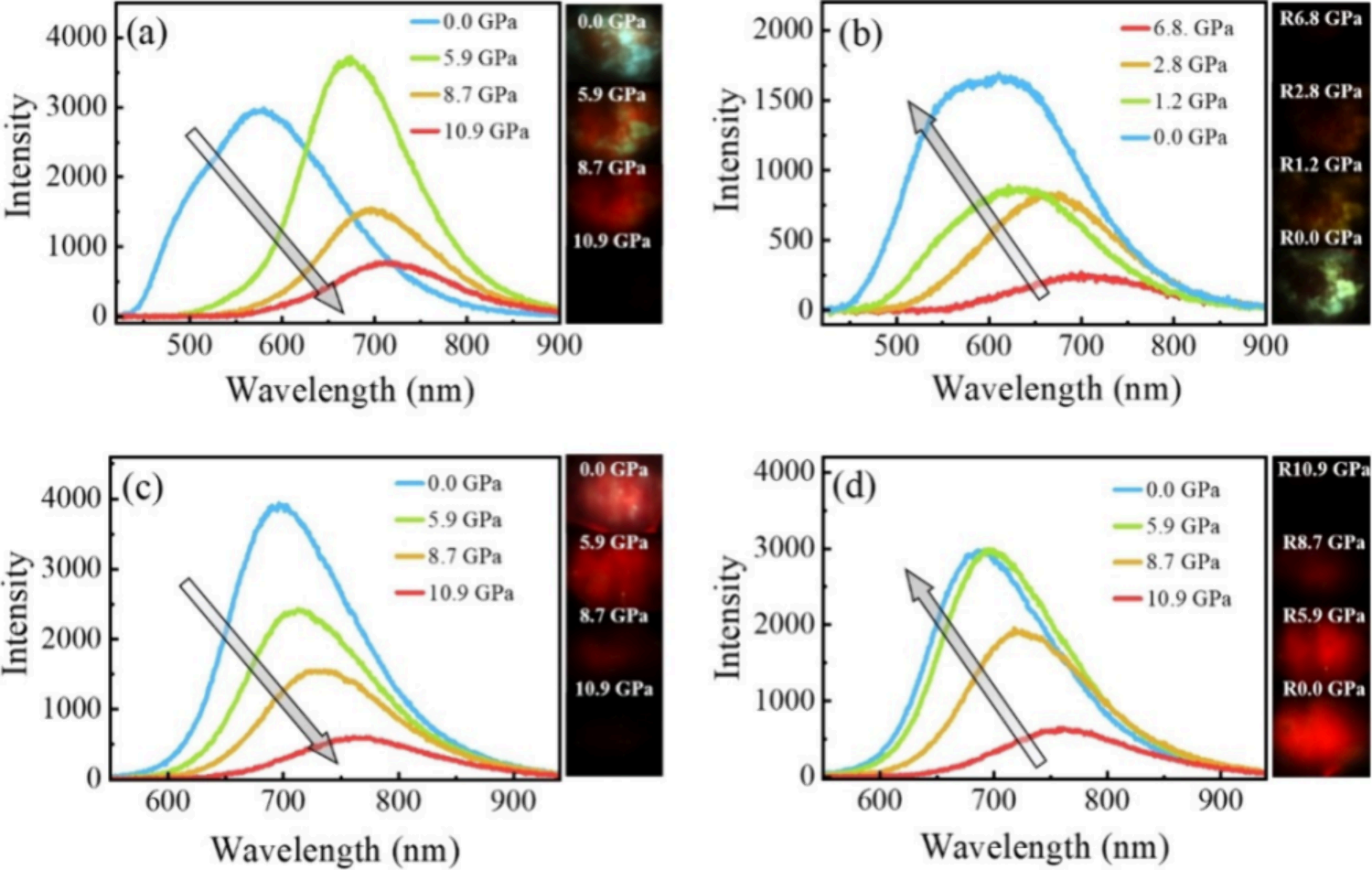
a) Pressure-dependent PL spectra and microphotographs and b) PL spectra taken upon releasing the pressure and microphotographs for **R-DOBP**; c) Pressure-dependent PL spectra and microphotographs and d) PL spectra taken upon releasing the pressure and their micrographs for R-HDOBP. Taken and adapted with permission from ref 653. Copyright [2021/Angewandte Chemie International Edition] John Wiley & Sons.

Two additional MCL-active TADF emitters, **XT-T** and **XT-OT**, were constructed from a xanthone acceptor and triphenylamine (T) or 4,4′-di­meth­oxy­tri­phen­yl­amine (OT) donors (Figure 177)^1210^. The crystals of **XT-OT** showed a large red-shift from blue-green (λ_PL_ = 466 nm, Φ_PL_ = 42.5%) to yellow (λ_PL_ = 567 nm, Φ_PL_ = 53.4%) upon grinding. However, crystals of **XT-T** exhibited a much more attenuated spectral shift from blue (λ_PL_ = 478 nm, Φ_PL_ = 38.1%) to green emission (λ_PL_ = 510 nm, Φ_PL_ = 40.7%) upon grinding. The ground powders were fumed with DCM solvent vapors which restored the original emission. PXRD analysis indicated that the change in emission color was due to a crystalline-to-amorphous transition caused by the grinding. This study highlights how slight differences in chemical composition can have a large impact on the conformation of the compounds, on the intermolecular interactions and packing arrangements in the crystal and ground forms, and thus on the extent of MCL response. OLEDs were also explored using 10 wt% emitter doping in CBP host. Devices with **XT-OT** showed superior performance, with EQE_max_ 9.4% and λ_EL_ of 532 nm compared to **XT-T** (EQE_max_ 3.3% and λ_EL_ of 488 nm). This performance dichotomy was likely caused by the larger Δ*E*_ST_ and longer τ_d_ of **XT-T**.

The compound **Py-BZTCN** is another example of a TADF material where the emission mechanism changes upon grinding (Figure 177).^1211^ The pristine crystalline powder showed orange-yellow fluorescence at λ_PL_ of 581 nm (Φ_PL_ of 52.8%; τ_p_ 2.37 ns). The emission of the ground powder was red-shifted to λ_PL_ of ∼676 nm with an associated smaller Φ_PL_ of 5.3%, and also showed TADF (τ_p_ = 7.97 ns; τ_d_ = 2.30 μs) with a small ΔE_ST_ of 0.087 eV. Similar to other systems, grinding disturbs the ordered π–π stacking of the pristine crystalline powder, converting it into an amorphous form as evidenced by the changes in the PXRD pattern. The original emission is again recovered by solvent fuming or heat treatment.

Beyond MCL, the broad category of mechanoluminance (ML) also encompasses triboluminance and fractoluminance.^1212^ These emission categories progress in the same way as photoluminescence, with the only difference being the method of exciton generation.^1213^ At present, compound **1** is the only example of ML exhibited by a TADF material (Figure 179).^1214^ Emission was observed upon scratching the powder sample, which shared the same spectrum as typical photoluminescence. While delayed emission was not explicitly measured for this material upon mechanical stimulus, it was shown to be TADF-active in the powder form upon photoexcitation. In the powder, the compound emits at λ_PL_ of 518 nm, with τ_d_ of 1.2 ms and has a Δ*E*_ST_ of 0.20 eV.

**Figure 179:**
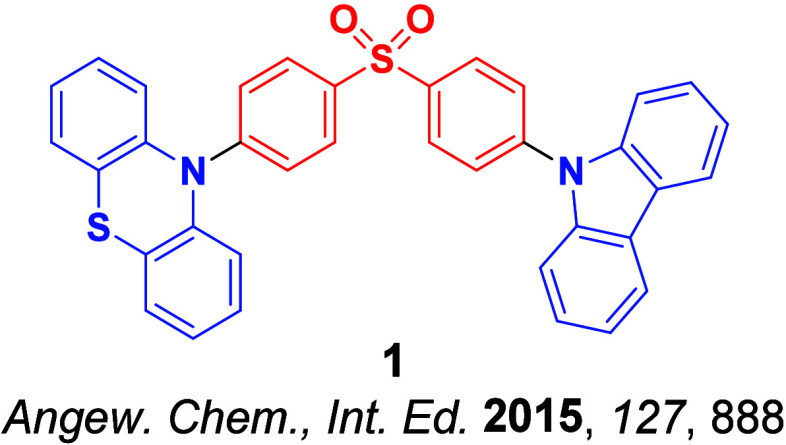
Structure of the TADF emitter undergoing mechanically excited emission reported in ref 1214 (the blue color signifies donor moieties, while the red color signifies acceptor moieties).

### 15.3 Outlook

As explored in this section, MCL and mechanoluminescence have been reported for a moderate number of TADF emitter systems. Although materials containing PTZ donors feature heavily, MCL can arise across a diverse range of emitter structures, and it remains difficult to predict *a priori* which compounds will show MCL or what underlying conformational changes alter the photophysics. This phenomenon also highlights how solid-state packing of TADF molecules can significantly and unpredictably impact the optical properties, which are so closely tied to molecular geometry adopted by D-A TADF emitters.

**Figure 180:**
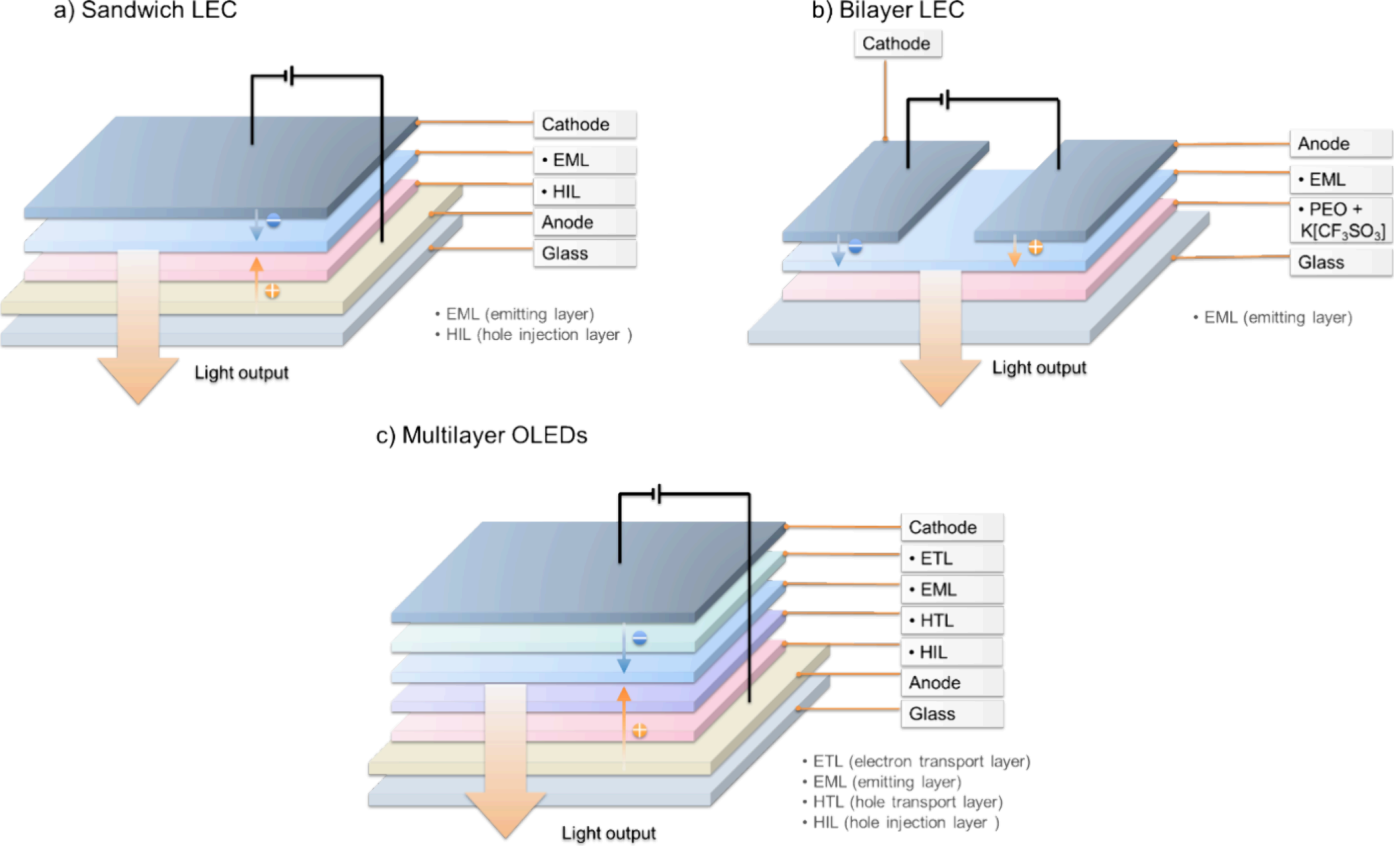
A comparison of the device structure of LECs and OLEDs.

We note that although some niche applications have been demonstrated, the observation of MCL remains largely an academic curiosity. It is not clear how this property might ever be utilised in thin, fragile, and encapsulated OLEDs. Nonetheless, materials exhibiting MCL and TADF properties can have independent applications of each property, and we propose that harnessing both simultaneously may unlock future utility. For example, since the lifetime of a TADF-MCL emitter can be used to distinguish between different forms of the material, time-resolved measurements could present a quantitative detection method with potential in anti-tampering and advanced anti-counterfeiting applications beyond simple ratiometric colorimetry. Pressure sensitive materials are also desirable considering the demand for stress/strain sensors, and TADF materials could present appealing candidates for *in situ* optical readout in both emission color and kinetics.

Despite the current lack of compelling applications for this class of compounds, they should be recognized as occupying the crossover region between optoelectronic and mechanically responsive material. Additionally, we speculate that a vast number of reported TADF emitters may have as-yet undiscovered MCL activity, which would escape the notice of the wider OLED community. Indeed, even venerable **4CzIPN** shows such properties, which were not discovered until relatively recently^125^ – likely because grinding and solvent vapor exposure are simply not widespread characterization techniques. It is therefore reasonable to expect that MCL may support innovative applications in the future beyond our current imaginations, with a wide library of candidate materials already available to be deployed.

## 16 TADF Light-Emitting Electrochemical Cells (LECs)

### 16.1 Introduction

In contrast to the OLEDs that are the electroluminescent device of focus in the previous sections, light-emitting electrochemical cells (LECs) have emerged as an alternative class of electroluminescent devices. Although a few examples of analogous single-layer OLEDs exist (e.g., single-layer PhOLEDs),^1215^ LECs offer an overall simplified device structure and are generally fabricated using solution-processing techniques. The most common architecture is a sandwich LEC, with an active layer and a hole injection layer (HIL) sandwiched between an air-stable cathode and a transparent anode (Figure 180a). The active layer is typically a blend of luminescent materials, ion transporting materials, and inorganic salts. There are also examples of LECs using polymers,^1216^ ionic transition-metal complexes,^1217,1218^ and organic small molecules (SMs) as emitter materials.^1219^ Similar to solution-processed OLEDs, the HIL in LECs is typically a blend of poly(3,4-ethylenedioxythiophene)-poly(styrenesulfonate) (PEDOT:​PSS). This water-soluble polymer is coated onto a glass substrate with transparent indium tin oxide (ITO) anode, and provides a smooth electrode surface with increased work function (WF) to promote charge injection, while also being impervious to subsequent depositions from organic solvents.^1220^ A metallic cathode such as aluminium is then deposited on top of the active layer to complete the device, typically by thermal evaporation.

A key feature of LECs that distinguishes them from OLEDs is the use of ions in the active layer to achieve charge transport, rather than relying on direct transport of electrons and holes through static layers. When an external bias is applied to the LEC, the separation of the ions in the active layer reduces the injection barrier, which enables the use of air-stable cathodes and established an *in situ* electrochemical doping of the organic semiconductor, forming a p-n junction across the active material.^1216^ The addition of salts into the emitter layer (EML) enables balanced electron and hole flow, translating into a high recombination rate of these particles into excitons. However, this operational mechanism also leads to increased exciton-polaron annihilation, affecting device performance much more acutely than in OLEDs. Thus, it remains an open research question whether a LEC can show high efficiency at high luminance.^1221^

In 2010 Sandström *et al.* reported a second LEC device structure based on a planar bilayer architecture (Figure 180b) that is similar to a bottom-gate top-contact transistor.^1222^ Thanks to this architecture the luminescent materials is largely separated from the electrolyte, which typically consists of a mixture of K[CF_3_SO_3_] and poly(ethylene oxide) (PEO). This planar structure with charge transport along rather than through the layers also permits observation of the temporal evolution of the luminance of the device, and insights into the device degradation mechanism.^1223,1224^

For LECs there are two principal models that explain the microscopic working mechanism (Figure 181).^1225^ The first is known as the electrochemical doping model (ECD, Figure 181a).^1226^ Upon application of a voltage, electrolyte anions start migrating towards the positively charged electrode while holes are injected into emitter molecules, producing radical cations. The opposite processes occur at the negatively charged electrode, forming a very thin electric double layer (EDL) of approximately 1 nm on each side of the device. The presence of these EDLs causes a substantial drop of the electric potential at the electrodes and facilitates further charge injection into the active layer. At the cathode the injection of electrons is compensated by diffusion of cations, which results in the formation of an n-type doped region. At the opposite electrode the extraction of electrons at the anode attracts anions and forms a p-type doped region. Such p- and n-doped type regions grow from the electrodes towards the centre of the cell, where radiative recombination takes place and steady-state emission is eventually established. The reliance on diffusion and growth of doped regions in the device aligns with their relatively long experimental turn-on times (t_on_), typically reaching maximum brightness over a few seconds or minutes.^1227^

**Figure 181:**
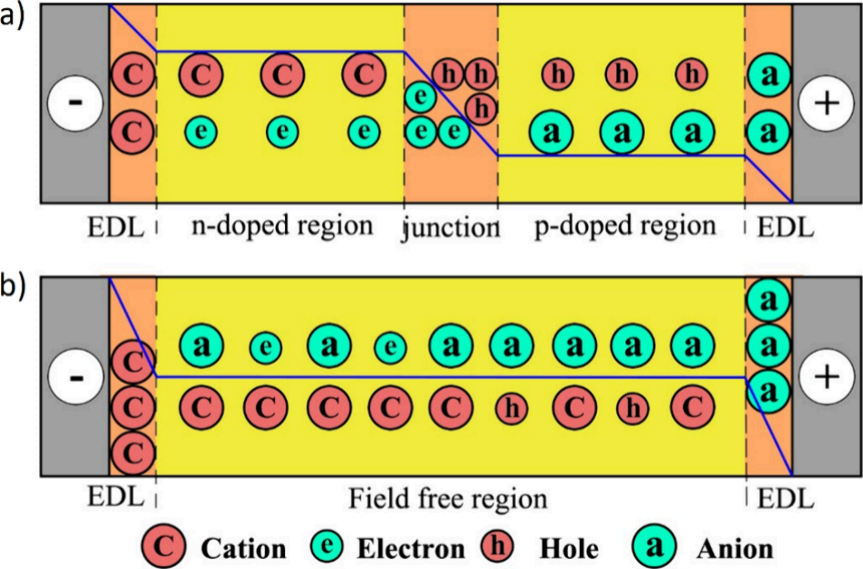
Illustration of the potential profiles and electronic/ionic charge distribution in an LEC during steady-state operation. Potential profiles and charge distributions as predicted by the a) ECD and b) ED models. The thick blue line represents the potential profile across the device; electronic and ionic charge carriers are represented by cyan (negatively charged) and red (positively charged) symbols. High- and low-field regions in the bulk are highlighted in orange and yellow, respectively. In the low-field regions, negative and positive centres are mutually compensated. Taken and adapted with permission from^1218^. Copyright [2010/Journal of the American Chemical Society] American Chemical Society.

The second LEC model is known as the electrodynamical model (ED) (Figure 181b).^1228^ As in the ECD model, charge injection is also made possible by the formation of the EDL at the electrodes. When the applied voltage is high enough, electrons and holes can additionally travel through the LUMO and HOMO levels of the semiconductors, respectively, and recombine to form excitons in the central field-free region and emit light.

Although the mechanisms are microscopically different, experimentally both models have been shown to be feasible.^1229,1230^ Indeed, van Reenen *et al*. revealed that a changeover in operating regimes occurs depending on the ability of the device to form non injection-limited ohmic contacts.^1218^ When ohmic contacts are formed the LEC follows the electrochemical doping model, but when the injection of charge carriers is limited the device instead follows the electrodynamical model.

As with an OLED, the EQE of a LEC is defined in terms of equation 19:

19 $$\mathrm{EQE}=\frac{b\varphi }{2n^{2}}$$

where b is the fraction of holes and electrons that recombine to excitons (analogous to *γ* for OLEDs, Section 1 equation 1, *φ* is the fraction of electrically produced excitons that can decay radiatively (*β*·*Φ*_PL_ for OLEDs), and $\frac{1}{2n^{2}}$ describes the outcoupling efficiency with n the glass substrate refractive index.^1231^ Typically, there is unitary recombination of holes and electrons in LECs (b = 1),^1232^ and therefore the EQE will depend primarily on the emitter’s inherent ability to harvest excitons and convert these to light – i.e., its ability to harvest singlets and triplets, and its emission efficiency. Clearly the use of emitters that are capable of harvesting both singlet and triplet excitons is highly desirable, and therefore TADF materials have attracted increasing attention within the LEC community. This section reviews progress in the development of both all-organic TADF emitters and copper(I) TADF complexes for LECs. Data of the emitters and devices summarised in this section are also collected in Table S21.

### 16.2 Ionic TADF LECs

In parallel with the development of OLEDs, LECs have historically employed cationic phosphorescent emitters to manage triplet excitons, mostly based on Ru(II) and Ir(III) complexes.^1233,1234^ There have also been a few examples involving the use of cationic organic fluorescent compounds as emitters in LECs.^1235^ Our group reported the first example of an LEC using a cationic organic TADF emitter in 2015 (Figure 182).^1236^ The emitter skeleton was derivative of **2CzPN** with pendant imidazolium groups linked to the carbazole donors, **2CzPN-LEC** (originally named **2** in that work). This compound has a Φ_PL_ of 90% as a 10 wt% doped film in PMMA and emits with λ_PL_ of 536 nm. In neat film the Φ_PL_ drops to 21% but with unchanged λ_PL_. LECs using a neat film of **2CzPN-LEC** as the active layer showed an EQE_max_ of 0.4% at λ_EL_ = 538 nm. The LEC showed a very low luminance of 12 cd m^–2^, as well as a decreasing driving voltage and luminance with time. An LEC incorporating the ionic liquid [Bmim][PF_6_] as additional electrolyte performed even more poorly, with an EQE_max_ of 0.12%. This result was surprising considering literature precedents of improved LEC performance when ionic transition metal complex emitters (iTMC) are co-doped with ionic liquids in the EML.^1237^ The same ionic TADF emitter was also used as a host in combination with a yellow fluorescent cyanine dye.^1238^ The reported EQE_max_ for this host-guest device was 1.9%, implying very efficient exciton utilization and high FRET efficiency from the TADF host to the cyanine dye, mirroring the hyperfluroescence approach developed for OLEDs. We later developed a blue-emitting LEC using the same cationic carbazole donor in combination with a weaker sulfone acceptor, **imCzDPS** (Figure 182).^1239^ The LEC emits at λ_EL_ = 470 nm but showed a very low maximum luminance of 2.5 cd m^–2^ under an average current density of 200 A m^–2^, and an EQE_max_ of 1.14%. The low luminance was attributed to the electrochemical instability of the emitter.

**Figure 182:**
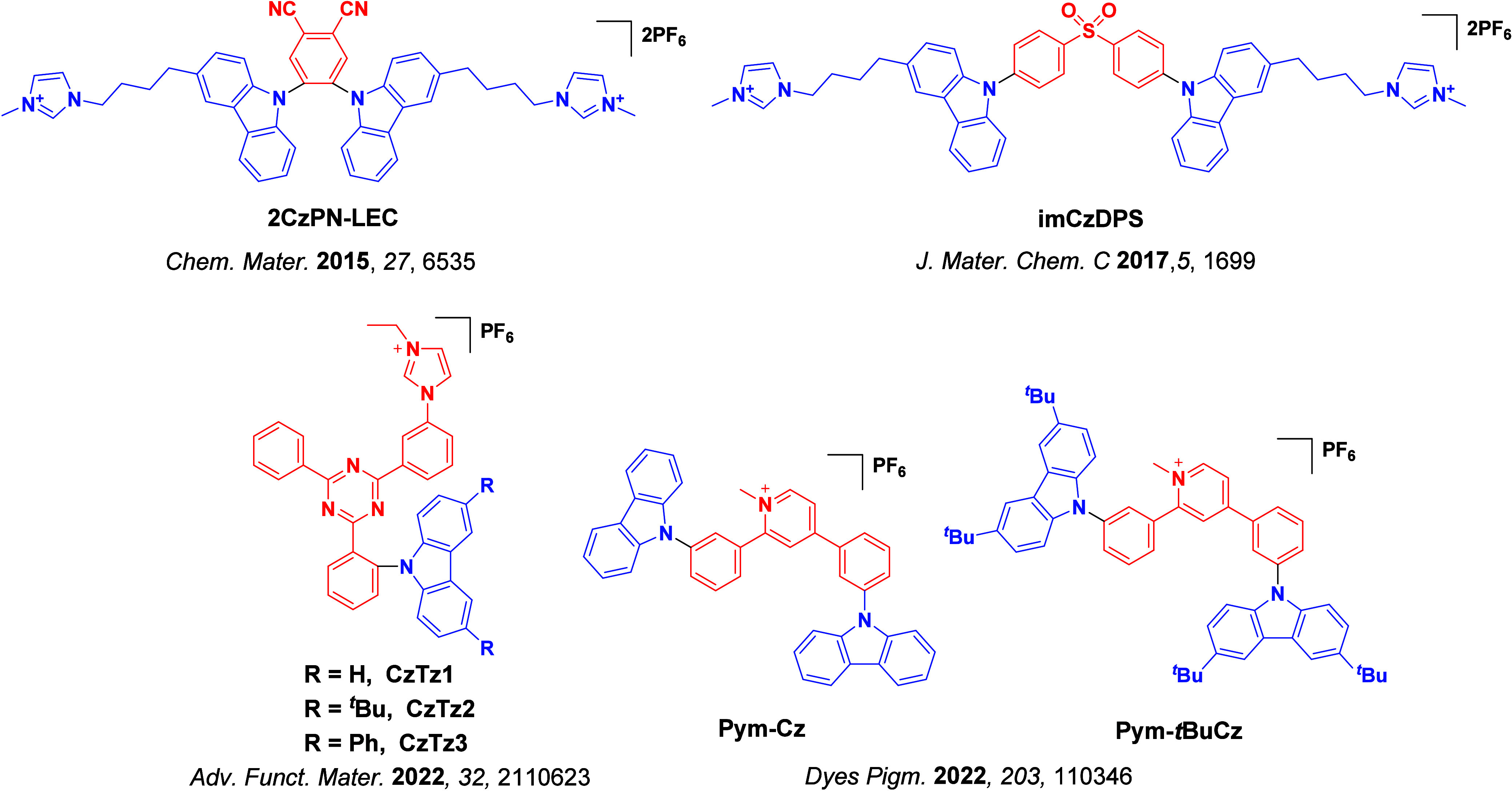
Structures of ionic TADF emitters used in LECs (the blue color signifies donor moieties, while the red color signifies acceptor moieties).

In 2022 a series of ionic D-A TADF compounds, **CzTz1**, **CzTz2** and **CzTz3** (originally named **1**, **2** and **3** in that work) were reported by Yu *et al*. as green emitters in LECs (Figure 182).^1240^ The architecture of the devices was ITO/PEDOT:​PSS/neat emitter/Al, and the most efficient LEC with **CzTz2** as the emitter showed an EQE_max_ of 6.8%, and Lum_max_ of 572 cd m^–2^ at CIE coordinates of (0.34,0.57). The device LT_50_ was 11.4 h under the at 4V and could be increased to 218 h when the device was driven at a lower constant current of 10 A m^–2^. The t_on_ of the **CzTz2**-based LEC under a constant driving voltage of 4.0 V was 740 min, which was rationalized due to the slow motion of the [PF_6_]^−^ anions, while under a constant current of 50 A m^–2^, the t_on_ for the same device was only 5.5 min.

Another two ionic emitters, **Pym-CZ** and **Pym-*t*BuCZ** (Figure 182) were used in orange-red emitting LECs by Shen *et al*.^1241^ The design strategy of these materials was distinct, as rather than appending an ionic group to a TADF-active core, instead an ionic methylpyridinium unit also formed the central acceptor of the D-A TADF structure. Orange-red emission from aggregates of **Pym-CZ** was present at the high doping concentrations used in the EML. The most efficient device with **Pym-CZ** showed an EQE_max_ of 1.19%, a CE_max_ of 2.48 cd A^–1^ under 3.0 V, and a Lum_max_ of 8.69 cd m^–2^ under 4.0 V. The t_on_ of this device under 4 V was about 9 min, longer than the device with **Pym-*t*BuCZ** (about 5 min).

### 16.3 Neutral TADF LECs

A green-emitting device was reported by Lundberg *et al.* in 2017, utilising **4CzIPN** (Figure 183).^1242^ In this example the emissive layer contained a mixture of host CBP, electrolyte (K[CF_3_SO_3_] in PEO), and polystyrene which helped to produce a homogeneous film. The optimal ratio of materials in the emissive layer was found to be 10:3:2.6:0.78:1.81 for CBP:​**4CzIPN**:​PEO:​K[CF_3_SO_3_]:​poly­styrene. The inclusion of a layer of PEDOT:​PPS between the ITO and the emitting layer proved essential to prevent short-circuiting of the devices. The LEC showed an EQE_max_ of 0.17% under a constant current of 770 A m^–2^ and an impressive Lum_max_ of 760 cd m^–2^ during a voltage ramp, which constitutes a much-improved brightness compared to TADF LECs using charged emitters. The t_on_ of this device was also less than 15 s, while the low EQE was attributed to the high electrolyte loading of 18.5 wt%.

**Figure 183:**
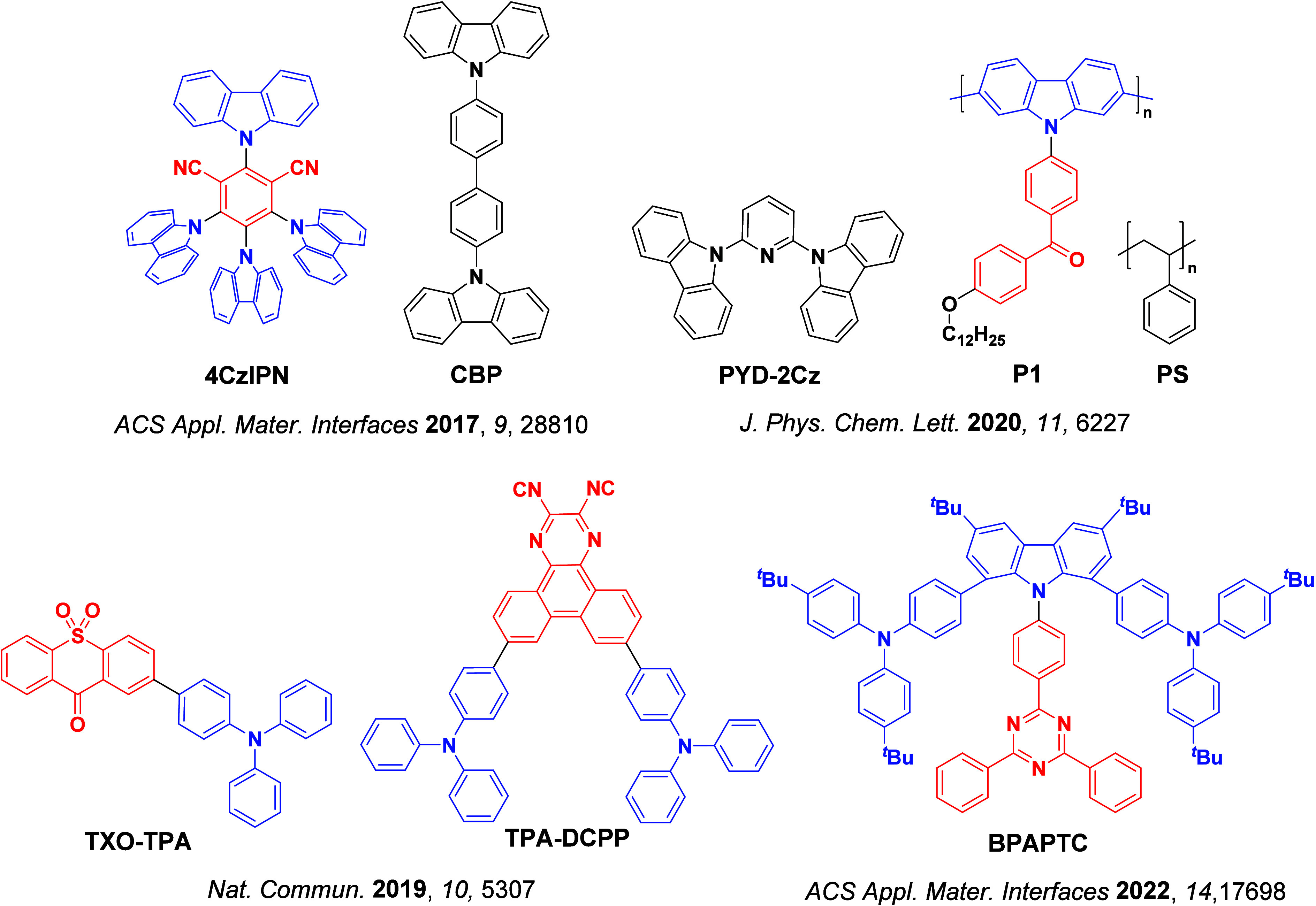
Structures of hosts and neutral TADF emitters used in LECs (the blue color signifies donor moieties, while the red color signifies acceptor moieties).

The same group later reported the first example of a LEC employing a TADF polymer as the emitter.^1243^ The EML of the LEC incorporated the ambipolar host, **PYD-2Cz**, and the TADF polymer emitter, **P1**, along with ionic liquid tetrahexylammonium tetrafluoroborate (THABF_4_) as electrolyte in a ratio of 66:17:8:9 (Figure 183). The LEC showed a luminance of 96 cd m^–2^ at 4 V, a CE of 1.4 cd A^–1^, and >600 cd m^–2^ at 6 V.

Exciplexes are excited states that can form when mixtures of donor and acceptor molecules interact to produce intermolecular charge-transfer (CT) excited states.^671^ Exciplexes frequently show TADF (see Section 8) and have been widely employed as both hosts and emitters in OLEDs. In 2019, Lundberg *et al*. demonstrated highly efficient LECs using each of the TADF emitters **4CzIPN**, **TXO-TPA** and **TPA-DCPP** (Figure 183), all using the same polymer exciplex host material composed of a blend of p-type PVK and n-type OXD-7 and driven by 100 A m^–2^.^1244^ The balanced hole and electron transport from the host blend significantly improved the efficiency of the devices by reducing exciton–polaron quenching.^1221^ The most efficient LEC in this report was obtained with **TXO-TPA** as the emitter, and showed an EQE_max_ of 7.0% and a CE of 16.0 cd A^–1^ at 120 cd m^–2^ with CIE coordinates of (0.46, 0.50). In comparison, the OLED with **TXO-TPA** showed an EQE_max_ of 18.5%, a CE_max_ of 43.3 cd A^–1^, and a Lum_max_ up to 16300 cd cm^–2^,^1141^ illustrating that LECs still require significant development to match the efficiency of corresponding OLED. The device with **TPA-DCPP** showed the highest performance for a red TADF LEC to date, with λ_EL_ = 618 nm, CIE coordinates of (0.54, 0.44), and an EQE_max_ of ca. 4% and a Lum_max_ of 380 cd m^–2^. The turn-on time to a luminance of 100 cd m^–2^ (t_on100_) ranged between 20–25 s for all three devices under a current density of 100 A m^–2^.

Similarly, using PVK:​OXD-7 as the co-host materials, Ye *et al*. reported a green TADF LEC with **BPAPTC** (Figure 183) as the emitter.^1245^ The active layer in the most efficient device used a blend of PVK:​OXD-7:​**BPAPTC**:​THABF_4_ in a 23.4:15.6:9:2 ratio. This LEC showed an EQE_max_ of 7.67%, a Lum_max_ of 3696 cd m^–2^ and a CE_max_ of 23.64 cd A^–1^ at a λ_EL_ of 533 nm, representing the highest EQE_max_ and Lum_max_ reported for TADF LECs to date. The exceptional performance in this device was attributed to intramolecular π–π stacking and hydrogen bond interactions in **BPAPTC**, which contribute to reduced ACQ. As such, a greater emitter doping concentration (18 wt%) could be exploited, translating to improve exciton-harvesting efficiency and higher EQE and luminance.

Bai *et al*. reported the first example of an LEC using an ionic exciplex system as a host material (Figure 184).^1246^ The exciplex host was formed between cationic donor ([***t*BuCAZ-ImMe][PF_6_]**) and acceptor ([**TRZ-ImEt][PF_6_]**), which was used in combination with **[Ir(buoppy)_2_(dmapzpy)][PF_6_]** as an emissive dopant in the EML of the LEC. The device with only the exciplex showed green emission and an EQE_max_ of 2.6%, a CE_max_ of 6.4 cd A^–1^ under 5.0 V, and a Lum_max_ of 231 cd m^–2^ under a constant current of 50 A m^–2^. The best device with the iridium complex co-dopant in the EML showed an EQE_max_ of 11.5% at 4.0 V, a low Lum_max_ of 45 cd m^–2^, but a high current efficiency of 25.8 cd A^–1^, at λ_EL_ of 473 nm.

**Figure 184:**
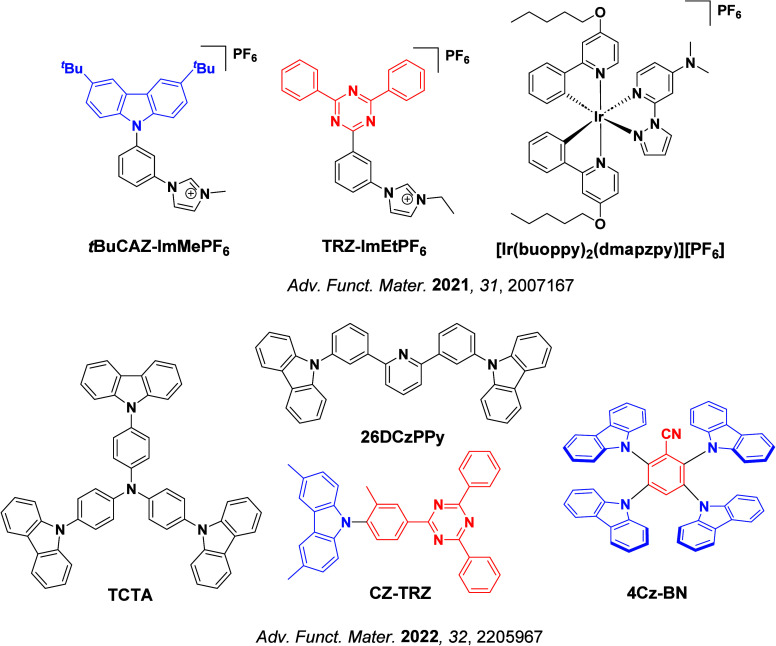
Structures of materials used in exciplex TADF LECs, including co-dopants, hosts, and transporting materials.

Adopting an EML more reminiscent of an OLED, several blue host-guest TADF LECs were reported by Tang *et al*. These devices used TCTA and 26DczPPy as host materials and **CZ-TRZ** or **4CZ-BN** as TADF emitters (Figure 184),^1247^ with the best device consisting of 31.2:31.2:31.2:6.4 for TCTA:​26DczPPy:​**CZ-TRZ**:​THABF_4_, and TCTA and 26DczPPy acting as co-hosts. This LEC showed an EQE_max_ of 5.0% at a constant current density of 7.7 mA cm^–2^, a Lum_max_ of 740 cd m^–2^, and a CE of 9.6 cd A^–1^. Emitting at λ_EL_ of 475 nm, this device shows the highest performance for a blue TADF LEC to date due to the use of the exciplex-forming co-host materials that can harvest both singlet and triplet excitons and then transfer these to the guest TADF emitter by FRET.^1248^

### 16.4 MR-TADF LECs

Due to their narrowband emission MR-TADF emitters have quickly become a hot topic for OLED applications (see Section 11). With LEC development once again mirroring OLEDs, we were the first to use ionic MR-TADF compounds (**DiKTa-ObuIm** and **DiKTa-DPA-ObuIm**, Figure 185) as emitters in LECs.^1249^ The device with **DiKTa-ObuIm** showed a Lum_max_ of 15 cd m^–2^ at 5 V and emits at λ_EL_ = 534 nm, while the Lum_max_ of the device with **DiKTa-DPA-ObuIm** was only 2 cd m^–2^ at 8 V and emitted at λ_EL_ = 656 nm. Much like the initially inferior performance of MR-TADF emitters in OLEDs, we expect that use of these in LECs will rapidly progress as design and application strategies are discovered. The red-emitting device based on **DiKTa-DPA-ObuIm** should be highlighted, as examples of red LECs using organic emitters are rare.

**Figure 185:**
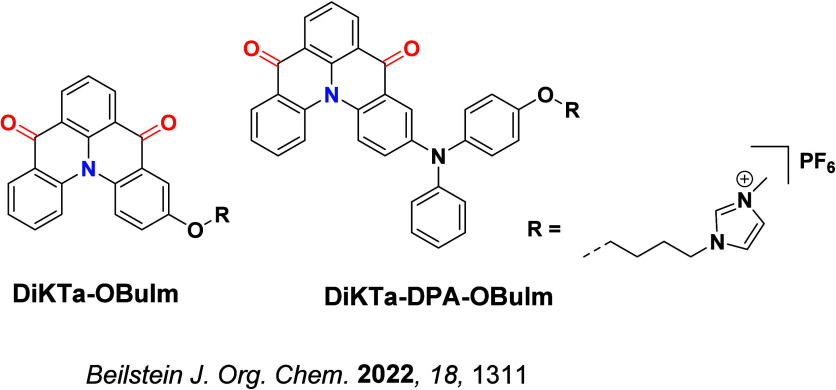
Structures of cationic MR-TADF emitters used in LECs (the blue color signifies donor atoms/functional groups, while the red color signifies acceptor atoms/functional groups).

### 16.5 Cu(I) Complex TADF LECs

In addition to fully organic TADF molecules, copper-based ionic transition metal complexes that display TADF (Cu-iTMCs) are also attractive as triplet-harvesting LEC emitters.^1250^ Similar to some TADF Carbene-Metal-Amides used in OLEDs (see Section 9), instead of relying only on the large spin–orbit coupling conferred by a heavy metal centre in phosphorescent materials, many copper(I) complexes emit via TADF or a combination of TADF and phosphorescence as a result of the small singlet–triplet energy gap (Δ*E*_ST_) and the MLCT nature of the lowest-lying excited states.^752^ Unlike other 3d-metal complexes, the d^10^ electronic configuration in Cu(I) means that there are no non-radiative d→d* electronic transitions, rendering this family of complexes unusually luminescent. The smaller SOC coefficient of Cu compared to 4d- and 5d- elements results in relatively slower phosphorescence and ISC rates, which allows TADF to become a competitive processes in the Cu(I) complexes.^1251^ All these characteristics make copper(I) complexes an attractive alternative to iridium complexes for their use as emitters in electroluminescent devices.

One consequence of the low-lying MLCT states is that upon excitation the Cu(I) formally is oxidized to Cu(II), which then undergoes Jahn-Teller distortion to a flattened geometry that is both more susceptible to nucleophilic attach and which leads to greater non-radiative decay. Such nucleophilic reaction leads to a pentacoordinate excited complexes, which also relaxes via non-emissive deactivation paths that lead to a reduction in the Φ_PL_.^1252^ The solution to both concerns is to use bulky ligands that limit the degree of geometric distortion in the excited state, preserving the tetrahedral geometry of the ground state and sterically shielding the metal centre from additional coordination. The most widely investigated family of copper complexes consequently contain both a diimine (N^∧^N) and a diphosphine (P^∧^P) ligand, [Cu(N^∧^N)(P^∧^P)]^+^, within which the P^∧^P ligands are typically very bulky bis(2-(di­phen­yl­phos­phino)­phen­yl)­ether (POP aka DPEPhos) or 4,5-bis­(di­phen­yl­phos­phino)-9,9-di­meth­yl­xan­thene (xantphos) derivatives (Figure 186a). The N^∧^N ligand is usually a derivative of 2,2′-bipy­ri­dine (bpy) or 1,10-phen­an­thro­line (phen) (Figure 186b).

**Figure 186:**
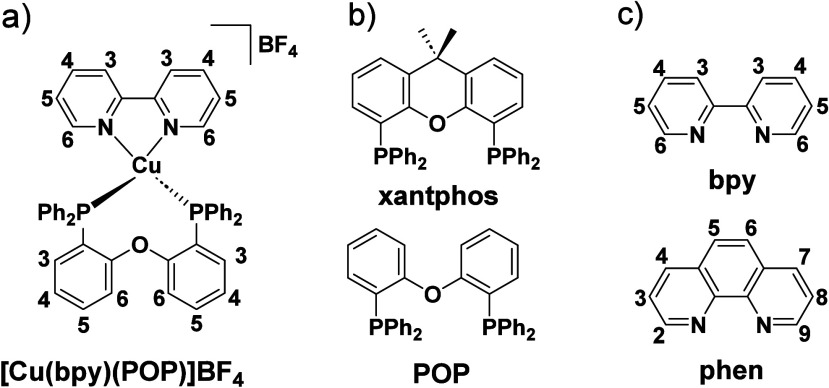
Structures of widely investigated families of copper(I) complexes: a) a typical [Cu(N^∧^N)(P^∧^P)]^+^ complex, b) the structure of common P^∧^P ligands used in TADF LECs, and c) the structure of common N^∧^N ligands used in TADF LECs.

In [Cu(N^∧^N)(P^∧^P)]^+^ complexes, substitution at the 6- and 6′-positions of bpy (the 2- and 9-positions of phen, Figure 186b) is often desirable to further limit geometric changes in the excited state.^1253,1254^ Such restriction leads to a blue-shifted emission, higher Φ_PL_ and longer excited state lifetime than complexes that do not contain substituents at these positions,^1255^ as substitution at these positions help to prevent relaxation to lower energy geometries with more active non-radiative decay.^816^

Keller *et al*. explored the impact of alkyl substitution of the N^∧^N ligand of [Cu(N^∧^N)(P^∧^P)]][PF_6_] complexes on the performance of the LECs.^1256^ They found that **[Cu(6,6′-Me_2_bpy)­(POP)]** (Figure 187, λ_PL_ = 564 nm, dissolved in CH_2_Cl_2_) had a more blue-shifted emission than **[Cu(4,5,6-Me_3_bpy)­(POP)]** (λ_PL_ = 598 nm). Meanwhile **[Cu(6,6′-Me_2_bpy)­(xantphos)]** (λ_PL_ = 606 nm) had a more red-shifted emission than **[Cu(4,5,6-Me_3_bpy)­(xantphos)]** (λ_PL_ = 582 nm). For thin films consisting of 4:1 [Cu(N^∧^N)(P^∧^P)]^+^:​[Emim][PF_6_], **[Cu(6,6′-Me_2_bpy)­(POP)]** showed the highest Φ_PL_ of 38%, **[Cu(xantphos)­(6,6′-Me_2_bpy)], [Cu(4,5,6-Me_3_bpy)­(xantphos)]** and **[Cu(4,5,6-Me_3_bpy)­(POP)]** had Φ_PL_ of 22, 19 and 16%, respectively. A possible explanation for these differences in Φ_PL_ is that for complexes with POP, which has a more flexible structure than xantphos, two methyl groups (or other substituents) next to the nitrogen atoms of the bpy are necessary to efficiently stabilize the tetrahedral complex geometry. However, for xantphos, a single alkyl substituent on the N^∧^N chelating ligand is sufficient to stabilize the geometry. Further, the total number of alkyl groups on the N^∧^N chelating ligand had a stronger impact on the HOMO–LUMO gap than their position. The best performing devices in this report used **[Cu(4,5,6-Me_3_bpy)­(xantphos)]­[PF_6_]** and **[Cu(2-Etphen)­(POP)]­[PF_6_]**. The device with **[Cu(4,5,6-Me_3_bpy)­(xantphos)]­[PF_6_]** showed an EQE_max_ of 1.7% and Lum_max_ of 462 cd m^–2^ under an average current density of 100 A m^–2^ at λ_EL_ = 570 nm, and had a t_on_ of 13 min. Under the same average current density, the LEC based on **[Cu(2-Etphen)­(xantphos)]­[PF_6_]** showed an EQE_max_ of 1.8% and a Lum_max_ of 451 cd m^–2^ at λ_EL_ = 582 nm, with a t_on_ of 25 min. However, this latter device was more stable and had a longer LT_50_ of 34.0 h. This improved stability was attributed to the similar structure of 2-Etphen and 6-Etbpy ligands, the later of which has been proven to lead to a long lifetime.^1257^ Although the electron-donating ability of the alkyl substituent at the α-position to the nitrogen atom of the N^∧^N ligand typically leads to a blue-shifted emission and higher Φ_PL_ for the complex compared to analogues without this substituent, substitution with a bulky ^*t*^Bu group led to a lower Φ_PL_ and shorter excited state lifetime due to the steric crowding about the metal centre. This excessive crowding results in elongated Cu–N bonds that affects both the non-radiative decay rates and the LUMO level that is localized on the N^∧^N ligand and demonstrates that careful management of the steric environment is required for this category of materials.

**Figure 187:**
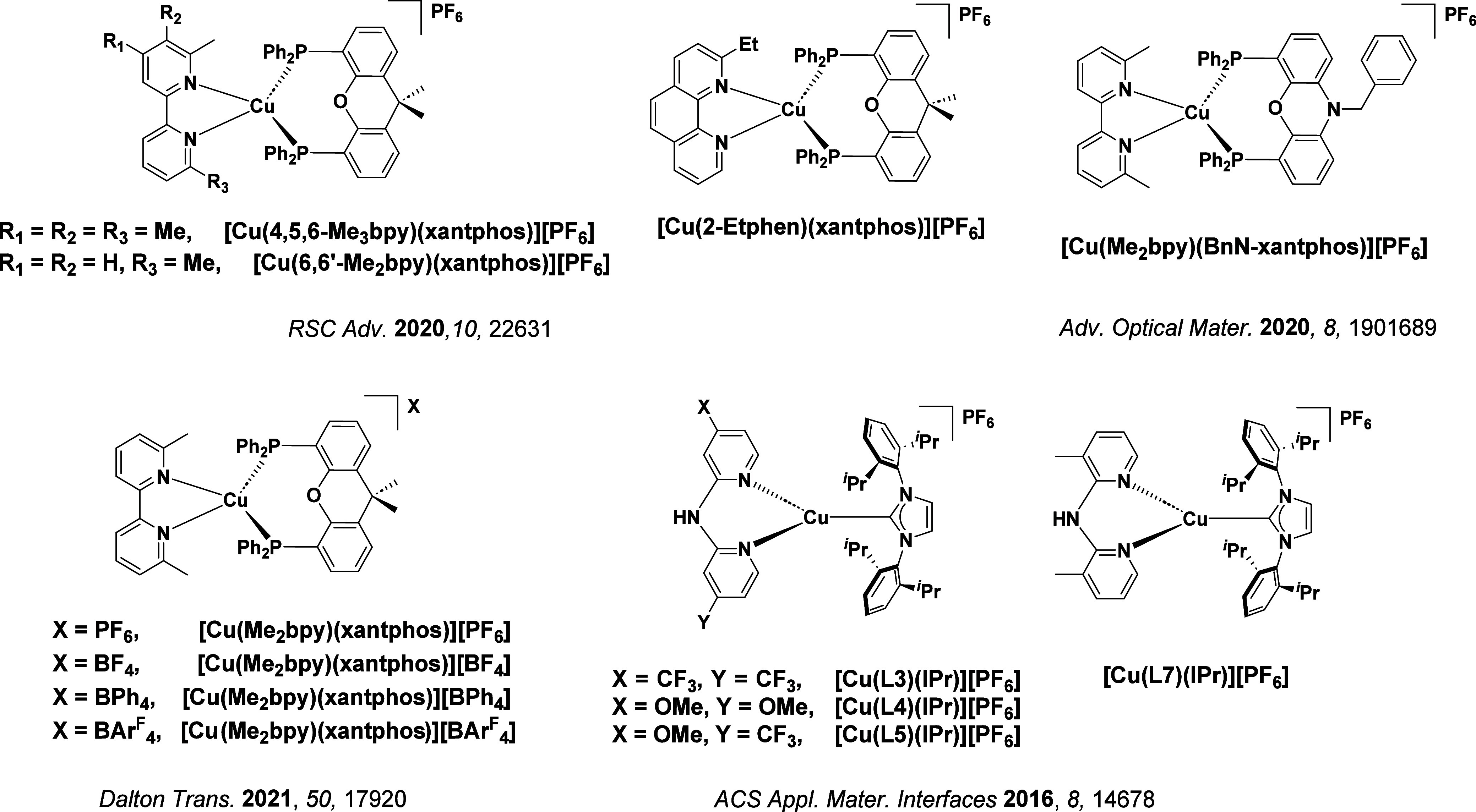
Structures of copper(I) TADF complexes containing a combination of N^∧^N and P^∧^P ligands or a combination of N^∧^N and NHC ligands used in LECs.

Arnosti *et al*. investigated the influence of hole injection layers on the efficiency of Cu(I) LEC devices.^1220^ Different compositions of PEDOT:​PSS (CLEVIOS P VP CH 8000, PEDOT:​PSS = 1:20 w/w and CLEVIOS P VP AI 4083, PEDOT:​PSS = 1:6 w/w) were employed as hole injection layers in devices with **[Cu(Me_2_bpy)­(BnN-xantphos)]­[PF_6_]** as the emitter (Figure 187). The device using the CLEVIOS P VP CH 8000 film – which has a higher PSS content, lower conductivity and a higher work function (WF) – showed the best performance with an EQE_max_ of 1.2% and Lum_max_ of 355 cd m^–2^ under a current density of 100 A m^–2^ at λ_EL_ = 567 nm. The improved performance of this device was attributed to the larger injection layer WF that can facilitate hole injection from the anode to the adjacent layers. The lower conductivity of the CLEVIOS P VP CH 8000 was also hypothesized to decrease the rate of non-radiative recombination at the PEDOT:​PSS interface, which may result in lower exciton quenching.

The influence of counterions on the performance of Cu(I) LECs was investigated by Meyer and co-workers.^1220,1258^ A family of [Cu(N^∧^N)­(P^∧^P)]^+^ complexes where the counterion differed between [BF_4_]^−^, [PF_6_]^−^, [BPh_4_]^−^ and [Bar^F^_4_]^−^ were studied, and the complexes with the larger [BPh_4_]^−^ and [Bar^F^_4_]^−^ counterions were found to be more loosely packed. Π-Stacking interactions between copper complexes has been shown to enable higher Φ_PL_ and can assist with restricting molecular geometry distortions, and these interactions can be disrupted with bulkier counteranions.^1259^ The LEC devices with **[Cu(Me_2_bpy)­(xantphos)]^+^** (Figure 187) as the emitter and smaller counterions [BF_4_]^−^ and [PF_6_]^−^ showed rapid turn-on times (t_on_ of 15 s and 58 s to reach a luminance of 100 cd m^–2^), while the devices with larger counterions [BPh_4_]^−^ and [Bar^F^_4_]^−^ failed to turn on at all, presumably because of the poor charge injection caused by the lower ionic mobility of the large counterions.

A series of 3-coordinate Cu(I) complexes have also been developed, employing N-heterocyclic carbenes (NHCs) as the monodentate ligand along with a bidentate N^∧^N ligand. Unlike most transient carbenes, the lone pair located in the plane of the heterocyclic ring of NHCs makes these compounds nucleophilic, excellent σ-donors, and able to easily bind transition metals.^1260^ NHCs have therefore become an attractive class of ligands in copper(I) complexes due to strong bonds to the metal and the ability to easily modify its structure, allowing for wide emission color tunability.^842^ Previous research has also demonstrated that the combination of NHC and dipyridylamine (dpa) in copper(I) complexes can lead to high-efficiency emitters.^1261^

To further investigate the impact of ligand modification on the photophysical properties, Elie *et al*. reported several emitters with both NHC and dpa-type ligands for blue LECs.^1262^ The study revealed that the dpa ligands have a more significant impact on emission in Cu(I) complexes than the NHC. The λ_em_ in emitters with the same NHC ligand and different dpa ligands indeed varied widely from 420 to 550 nm. With the same dpa ligand, different NHC ligands instead had little effect on λ_em_, ranging only from 465 to 481 nm. However, the different NHCs did lead to significant changes in Φ_PL_, varying from 17 to 64%. This implies that substituting the NHC ligand could potentially increase the radiative rate constant and/or reduce the non-radiative rate constant, thereby increasing Φ_PL_ without affecting λ_em_ in the blue region. By comparing **[Cu(L3)(Ipr)]­[PF_6_]**, **[Cu(L4)(Ipr)]­[PF_6_]** and **[Cu(L5)(Ipr)]­[PF_6_]** (Figure 187), which have different substituents at the same 4,4′-position of dpa, it was additionally demonstrated that this asymmetrical substitution leads to a significant enhancement in Φ_PL_. Specifically, the Φ_PL_ was found to be < 5% in **[Cu(L3)(Ipr)]­[PF_6_]** and **[Cu(L4)(Ipr)]­[PF_6_]**, but increased to 20% in **[Cu(L5)(Ipr)]­[PF_6_]**. This impact of asymmetrical substitution at the 4,4′-position of dpa, which the authors termed a push-pull effect, was attributed to the more distinct intraligand charge transfer character in **[Cu(L5)(Ipr)]­[PF_6_]**. The best device in this report used **[Cu(L7)(Ipr)]­[PF_6_]** and showed green emission at 497 nm, a Lum_max_ of 80.3 cd m^–2^ at 33.2 mA cm^–2^ and a high current efficiency of 0.29 cd A^–1^ at 16.65 mA cm^–2^. A long LT_50_ of 16.5 min was also achieved at a low constant current of 9.97 mA cm^–2^. The observed red-shift of the EL spectrum over time and an inability for the device to relight after power cycling reflects strong degradation of the emitters in the device.

### 16.6 Outlook

The development of organic TADF emitters for LECs has evolved rapidly since the first report in 2015 ^1236^ while there are now a large number of three- and four-coordinate Cu(I) complexes that have been used in LECs. LECs offer the promise of a low-cost alternative to OLEDs due to their simpler device structure.^1263^ Of the organic TADF LECs reported to date, the highest EQE_max_ devices employed **BPAPTC** as the emitter, showing an EQE of 7.7% and λ_EL_ of 533 nm.^1245^ However, the performance of LECs still lags significantly behind that of OLEDs, even when using the same emissive material. For instance, one of the most efficient LECs was reported with the emitter **TXO-TPA**, showing an EQE_max_ of 7.0% at CIE coordinates of (0.46, 0.50 (Figure 180).^1244^ The OLED with the same emitter showed an EQE_max_ of 18.5.^1244^ Issues surrounding ACQ and exciton polaron annihilation will need to be addressed for LECs performance to begin to rival that of OLEDs. Similar to that observed with OLEDs, the performance of blue and red LECs is much poorer than for green devices. The highest efficiency blue LEC employed **CZ-TRZ** as the emitter, showing an EQE_max_ of 5.0% and emitting at 475 nm.^1247^ The champion red LEC [CIE coordinates of (0.54, 0.44)] used **TPA-DCPP** as the emitter and showed an EQE_max_ of 4%.^1244^ Indeed, porting over successful OLED device strategies to LECs, such as the use of exciplex hosts and HF, are certainly worth deeper exploration in a bid to improve the performance of these devices. The relatively small number of reports to date make it hard to predict the potential value of TADF emitters in LECs. However, the fact that there are already examples that rival some of the highest efficiency iridium-based LECs should provide impetus to continue to develop improved organic emitter materials and device architectures for this alternative electroluminescence technology.

Alongside organic TADF LECs, recent works using cationic Cu(I) complexes have focused on correlating structure to device performance in the case of four-coordinate complexes and exploring the potential of three-coordinate complexes as a superior class of organometallic emitters. Most of the copper(I) complexes incorporating bidentate N^∧^N, N^∧^P or P^∧^P ligands are red, orange, or yellow emitters, meanwhile examples of blue and green copper complexes used in LEC are much more scarce and frequently contain strongly σ-donating NHC ligands. There are, up to the end of 2022, no reports of deep blue or near-infrared copper LECs using Cu(I) complexes. Nonetheless, the performance of Cu(I)-based LECs presently rival that of the well-studied iridium(III)-based LECs^1233^ and thus still drives interest in this area.

## 17 TADF Assistant Dopant and Hyperfluorescence

### 17.1 Introduction

There is an inescapable compromise in the design of D-A TADF emitters for OLED applications. While decoupling of the HOMO and LUMO in orthogonal conformations helps to minimise ΔE_ST_ and promote RISC, it can also inhibit emission by decoupling S_1_ from S_0_, attenuating the oscillator strength of the emissive transitions. This fundamental trade-off means that D-A emitters typically excel at either RISC or Φ_PL_, or attempt to balance both. Inadequate performance in either aspect has detrimental impact on device performance, either in terms of the EQE_max_ (relying on high Φ_PL_) or the efficiency roll-off at higher current densities (relying on fast *k*_RISC_).

One solution that has gained prominence is to decouple exciton harvesting from emission by employing separate materials, each individually optimized to handle these processes within the emission layer. In this context the TADF material acts as an assistant dopant or sensitiser in the OLED, typically supporting singlet emission from another fluorescent emitter in the EML (TAF or TSF OLEDs);^1264^ this same strategy has been coined by Adachi as hyperfluorescence (HF).^1265–1267^ Upon electrical excitation, RISC occurs on the TADF assistant dopant, harvesting triplet excitons, followed by Förster resonant energy transfer (FRET) from the singlet state of the TADF assistant dopant to the terminal emitter (itself either purely fluorescent or TADF), with resultant radiative decay from the latter (Figure 188). Particularly effective in this regard is the use of MR-TADF compounds as the terminal emitters (Section 11), which can provide a solution to producing devices having narrowband emission and a horizontally aligned transition dipole moment in the HF-OLED without undermining performance through otherwise slow *K*_RISC_. The key advantage of this mechanism is that the TADF assistant dopant is no longer required to simultaneously possess two fundamentally incompatible photophysical properties (i.e., fast *k*_RISC_ and high Φ_PL_).^1268^

**Figure 188:**
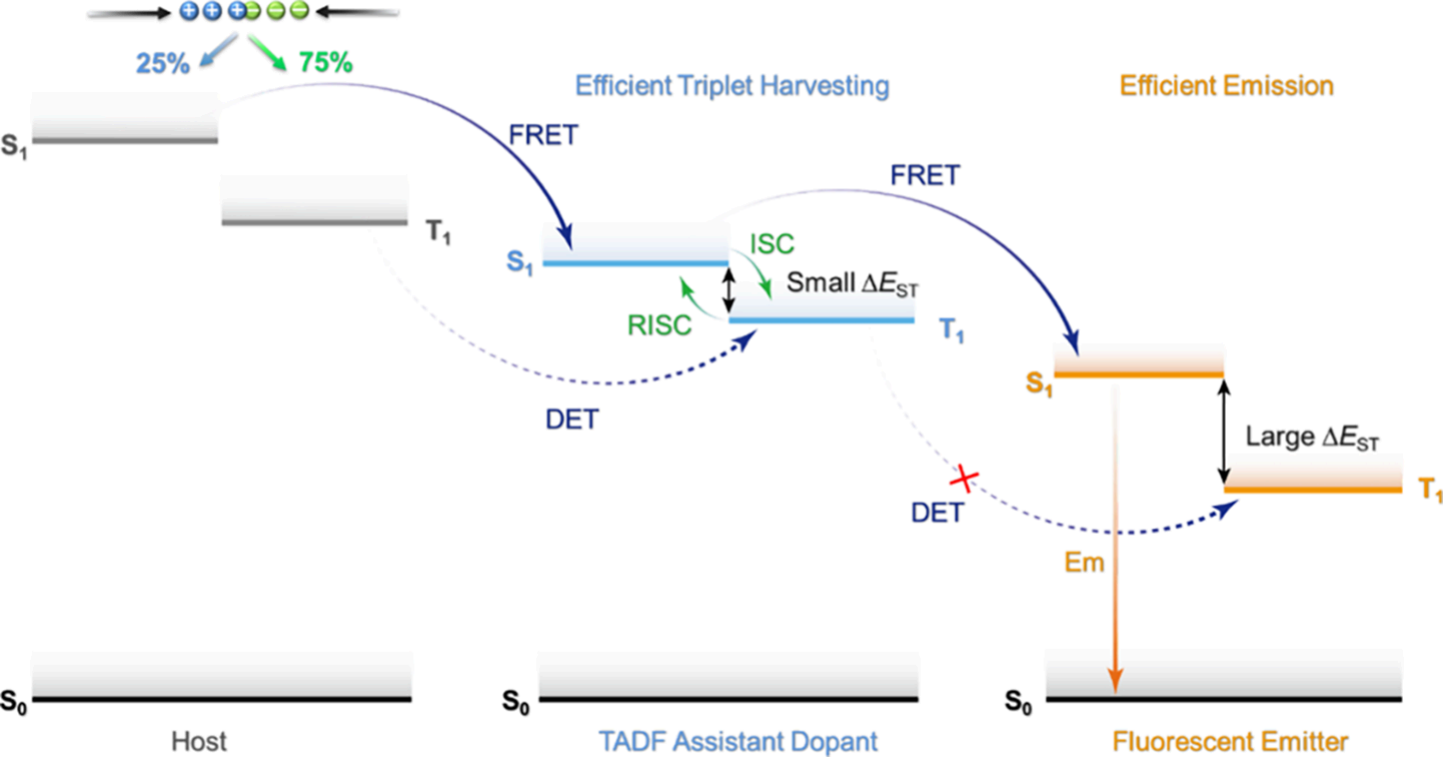
Schematic illustration of the mechanism of TADF-sensitized emission using a TADF assistant dopant and a fluorescent terminal emitter, both embedded in a host matrix (aka Hyperfluorescence^TM^).

Paramount to achieving efficient HF-OLEDs is the requirement for rapid FRET between the assistant TADF dopant and the terminal emitter, which is most favorable with strong overlap between the emission spectrum of the assistant dopant and the absorption spectrum of the terminal emitter. This concept is not new, having been exploited in PhOLEDs using a phosphorescent assistant dopant coupled with a fluorescent terminal emitter;^1269^ however, since the first promising example of this strategy using a TADF assistant dopant,^1270^ there has been a surge in the number of reports of HF-OLEDs. It must be noted, however, that competing processes such as Dexter energy transfer (DET) or direct hole-electron recombination to form triplet excitons on the terminal emitter can also take place. These processes open new quenching channels not applicable to regular TADF OLEDs, leading to sometimes poorer efficiencies in HF-OLEDs that are particularly challenging to study due to the complexity of the multi-component emissive layer.^1271^ Nevertheless, these competing processes can be somewhat mitigated by lowering the doping concentration of the terminal emitter.^1269^ A summary of the device performance of the examples discussed in this section is summarized in Table S22.

### 17.2 Materials Development

The first examples of HF-OLEDs were reported in 2014, where a series of emitters was used by Nakanotan *et al*. covering blue, green, yellow, and red emission.^1270^ The four systems involved combinations of fluorescent terminal emitters (**TBPe**, **TTPA**, **TBRb**, and **DBP**) paired appropriately with TADF assistant dopants (**ACRSA**, **ACRXTN**, **PXZ-TRZ**, and **Tri-PXZ-TRZ**) (Figure 189) to ensure the appropriate spectral overlap and thus efficient FRET. To mitigate DET, the fluorescent terminal emitters were doped at 1 wt% concentration, whereas the TADF assistant dopants were used at higher concentrations optimized separately in normal TADF-OLEDs. Blue-emitting HF devices consisted of **TBPe** with 15 wt% TADF assistant dopant **ACRSA** in the DPEPO host and showed an EQE_max_ of 13.4% at CIE coordinates of (0.17, 0.30). This was much higher than the performance of an OLED containing only the terminal emitter, although less than what had been previously reported for the device with 20 wt% **ACRSA** by itself in DPEPO, which showed an EQE_max_ of 16.5%.^472^ The green-emitting HF devices contained **TTPA** and 50 wt% **ACRXTN** as the TADF assistant dopant in mCP and showed an EQE_max_ of 15.8% at CIE coordinates of (0.29, 0.49). The yellow-emitting devices were obtained using **TBRb** with 25 wt% **PXZ-TRZ** assistant dopant in mCBP and showed an EQE_max_ of 18.0% at CIE coordinates of (0.45, 0.53). Finally, **DBP** with 15 wt% of assistant dopant **Tri-PXZ-TRZ** in CBP produced red-emitting devices with an EQE_max_ of 17.5% at CIE coordinates of (0.61, 0.39). Efficiency roll-off was low-to-moderate at 32, 26, 4, and 38% for the blue-, green-, yellow-, and red-emitting devices, respectively, at 1000 cd m^–2^. Further, the device stability improved, exemplified by the blue-emitting OLED LT_50_ of 194 hours at an initial luminance of 3225 cd m^–2^, suggesting rapid utilization of excitons.

**Figure 189:**
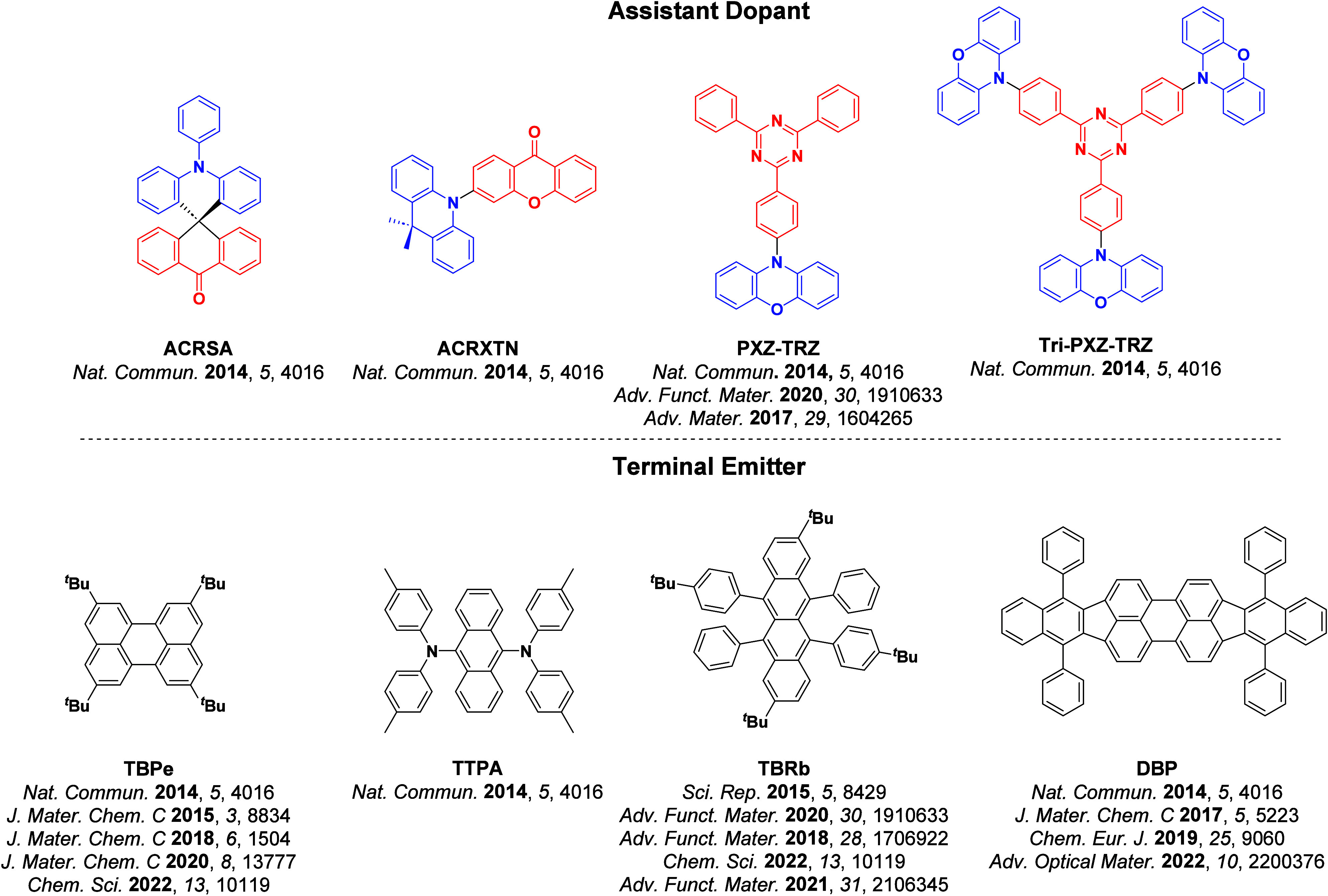
Chemical structures of TADF assistant dopants and fluorescent terminal emitters used in reported HF-OLEDs (the blue color signifies donor moieties, while the red color signifies acceptor moieties).

Despite these promising results, the color of these HF-devices became less saturated where, for instance, the HF device containing **TBPe** had CIE coordinates of (0.17, 0.30), redder than the assistant dopant [CIE coordinates of (0.15, 0.21)]. With the later advent of narrowband MR-TADF materials and their use as terminal emitters (examples below and in Section 11), it has since become possible for HF-OLEDs to possess a more saturated emission color compared to the TADF assistant dopant, and even to ‘upconvert’ the perceived emission color as the emission spectrum narrows (with lower energy onset).^1272–1274^ This approach may even help address current challenges in designing appropriate host materials for blue TADF OLEDs^1275^ (see Section 18).

A different TADF assistant dopant, **CzAcSF** (Figure 190), was used by Lee *et al*.^1276^ With an EML composed of 50 wt% **CzAcSF** and 0.1 wt% **TBPe** (Figure 189) in a DPEPO host, an EQE_max_ of 18.1% was achieved, while the color point improved due to efficient FRET, reflected in the CIE coordinates of (0.15, 0.22) having become much closer to those of the fluorescent device. The improved efficiency was attributed to not only the more efficient FRET for this HF pair, but also reduction of charge trapping on the terminal emitter resulting from the low doping concentration (0.1 wt% compared to 1.0 wt%) and higher doping concentration of the TADF assistant dopant (50 wt% compared to 15 wt% in the previous example).

**Figure 190:**
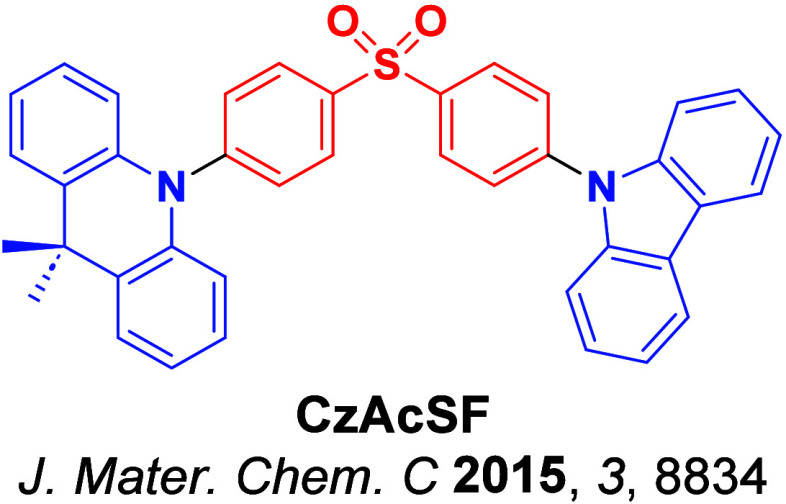
Chemical structure of the TADF assistant dopant **CzAcSF** (the blue color signifies donor moieties, while the red color signifies acceptor moieties).

Ahn *et al*.^1277^ reported an HF-OLED using 0.4 wt% **BPPyA** (Figure 191) as the terminal emitter in conjunction with 40 wt% **DMAC-DMT** (Figure 191) as the assistant dopant, all in DBFPO host. These devices showed an EQE_max_ of 19.0% at CIE coordinates of (0.14, 0.15) along with a low-efficiency roll-off of 8% at 500 cd m^–2^ and improved device lifetime (LT_50_ = 2.8 h at an initial luminance of 400 cd m^–2^), compared to the device with **DMAC-DMT** alone (LT_50_ = 0.7 h) that showed an EQE_max_ of 22.5%. This study illustrated that the HF strategy could reduce the probability of singlet-triplet annihilation (STA) and triplet-triplet annihilation (TTA) processes, with rapid FRET from the assistant dopant to the terminal emitter in the HF device effectively reducing the triplet exciton population and thus the chance of multi-excitonic quenching. This was evidenced by the shorter τ_d_ of 2.49 ms in the **BPPyA**:​**DMAC-DMT**:​DBFPO emissive system compared to **DMAC-DMT**:​DBFPO system, although this analysis has since been demonstrated to be unexpectedly complex in a similar HF system.^1271^

**Figure 191:**
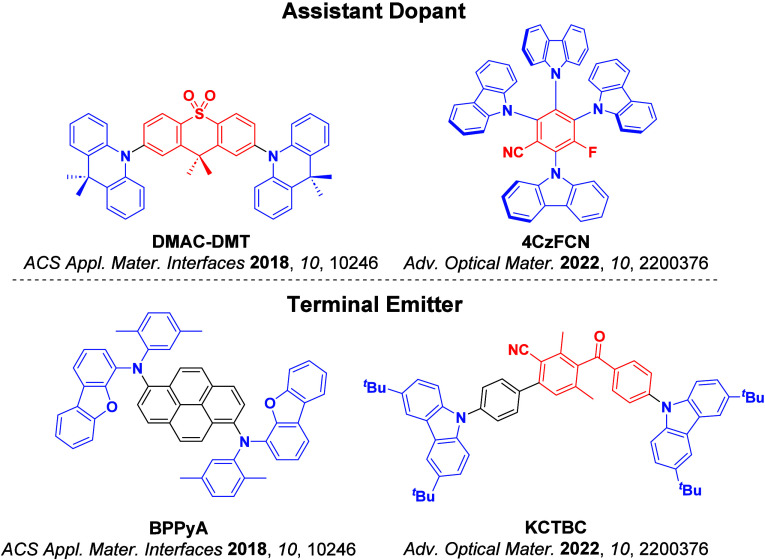
Chemical structures of the TADF assistant dopants and fluorescent terminal emitters. The terminal emitter **BPPyA** and the TADF assistant dopant **DMAC-DMT** were used in the high-performance HF-OLED, while **KCTBC** as the terminal emitter and **4CzFCN** as the TADF assistant dopant were used in a solution-processed HF-OLED. The blue color signifies donor moieties, while the red color signifies acceptor moieties.

HF-OLEDs can also be fabricated using solution-processing methods, as demonstrated by Alam *et al*.^1278^ The blue-emitting device contained 3 wt% **KCTBC** (Figure 191) as the terminal emitter and 12.5 wt% **4CzFCN** (Figure 191) as the assistant dopant in CBP host, and showed an improved EQE_max_ of 13.9% compared to that of the device with 3 wt% **KCTBC** alone, which exhibited an EQE_max_ of 9%. As well as reducing production costs, in the context of HF-OLEDs solution-processing also significantly simplifies the challenging 3-way co-deposition processes for the EML compared to vacuum deposition, which becomes particularly challenging for ultralow terminal emitter doping ratios <1%.

Delicately modulating the concentrations of both the assistant dopant and the terminal emitter is paramount for controlling energy transfer and achieving optimal results in HF-OLEDs. However, from a molecular design standpoint, the introduction of bulky functional moieties such as *tert*-butyl groups can also help to control intermolecular spacing, and so reduce the likelihood of undesired DET processes. Following this principle, Yun *et al*. designed a molecule, **FTrzTCz** (Figure 192), using 3,6-di-*tert*-butylcarbazole as the donor and triazine as the acceptor.^1279^ The HF-OLED using 20 wt% **FTrzTCz** as the assistant dopant and 1 wt% **6tBPA** as terminal emitter showed an EQE_max_ of 17.9% with CIE coordinates of (0.24, 0.58). The same group further investigated the influence of steric hindrance by introducing three 3,6-*tert*-butylcarbazole donors about the triazine unit to produce *tert*-butyl-functionalized donor-acceptor compound **TbCzTrz**.^1280^ As a comparison, **TmCzTrz** was also synthesized, which contained methyl substituents on the carbazole donor as opposed to *tert*-butyl groups. The TADF device based on **TbCzTrz** showed a much lower intrinsic EQE_max_ than that based on **TmCzTRz** (13.7% *vs*. 28.1%); however, using 20 wt% **TbCzTrz** or **TmCzTrz** as assistant dopants with 0.5 wt% **6tBPA** (Figure 192) as the terminal emitter gave EQE_max_ values of 14.6 and 18.5%, respectively. This demonstrated the utility of the ^*t*^Bu substitution outside of the direct TADF performance of the **TbCzTrz** emitter. HF-OLEDs using **C545T** as the terminal emitter similarly showed EQE_max_ of 16.1 and 15.9%, again higher for the device employing the more sterically shielded TADF assistant dopant, which presumably contributed to suppressing DET between the assistant dopant and the fluorescent terminal emitter.

**Figure 192:**
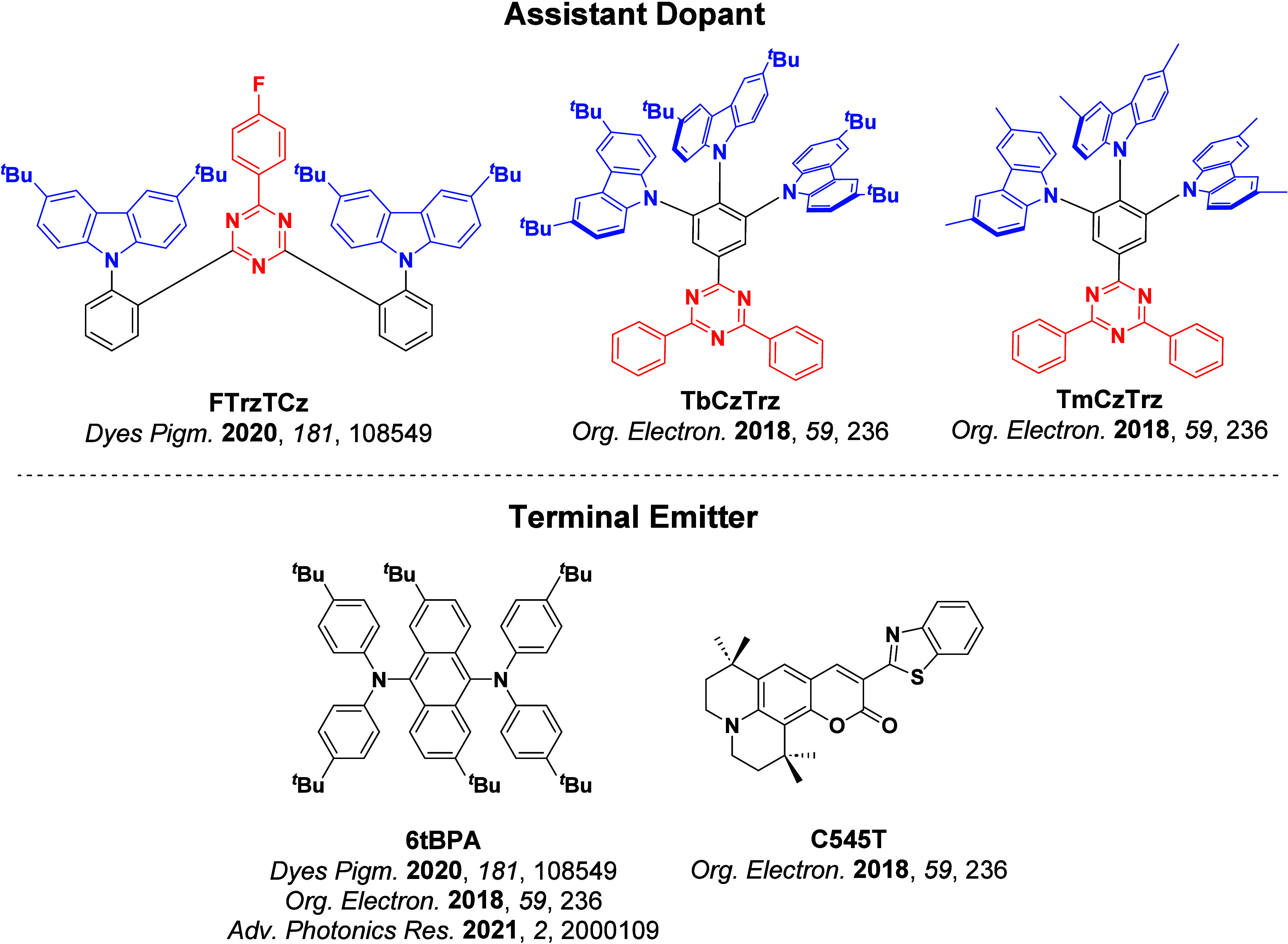
Chemical structures of TADF assistant dopants and fluorescent terminal emitters containing bulky moieties featuring both *tert*-butyl and methyl groups (the blue color signifies donor moieties, while the red color signifies acceptor moieties). In these reports, the bulky moieties were used to modulate intermolecular spacing.

Blocking undesired DET in HF-OLEDs was also studied by Xie *et al*.^1281^ using TADF compounds **PXZ-DBPZ** and **FPXZ-DBPZ** (Figure 193) as assistant dopants. Photophysical investigations and Kinetic Monte Carlo simulations revealed that the inert phenyl-fluorene substituents on **FPXZ-DBPZ** could effectively suppress DET process compared to **PXZ-DBPZ**. The device with 9 wt% **FPXZ-DBPZ** as the assistant dopant and 0.6 wt% **DBP** as terminal emitter in CBP showed an EQE_max_ of 18.1%, which was higher than that with **PXZ-DBPZ** (EQE_max_ = 15.2%) as the assistant dopant.

**Figure 193:**
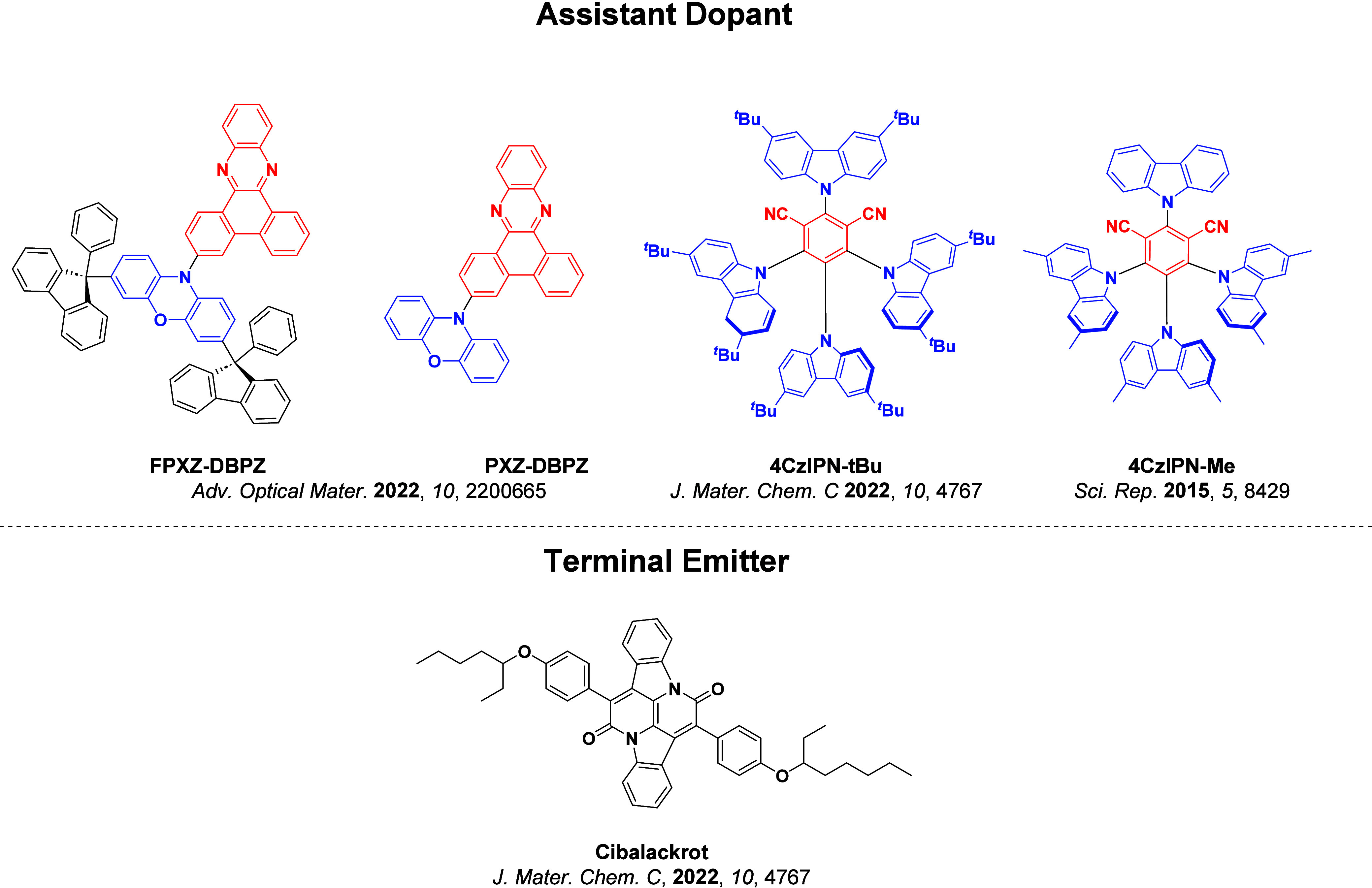
Chemical structures of TADF assistant dopants and fluorescent terminal emitters (the blue color signifies donor moieties, while the red color signifies acceptor moieties). These HF-OLEDs show suppressed DET between the assistant dopant and the terminal emitter.

Similarly to reduce the DET, a *tert*-butyl-functionalized **4CzIPN-tBu** (Figure 193), was employed as TADF assistant dopant in HF-OLEDs by Wallwork *et al*.^1282^ The best-performing solution-processed device with 0.5 mol% **cibalackrot** (Figure 193) as the terminal emitter and 29.5 mol% **4CzIPN-tBu** as the assistant dopant in mCP showed an EQE_max_ of 15.3%, and EQE_100_ of 14.9%.

Taking the structure of the fluorescent emitter into account, the TADF compound **4CzIPN-Me** (Figure 193) was used in a similar study by Furukawa *et al.*,^1283^ alongside the emitter **TBRb** (Figure 189), which contains four *tert*-butyl groups. **4CzIPN-Me** showed an efficient *k*_RISC_ of 7.7 × 10^5^ s^–1^ and the yellow-emitting HF device with 0.65 wt% **TBRb** and 6.3 wt% **4CzIPN-Me** in mCBP showed an EQE_max_ of 19.1% at CIE coordinates of (0.43, 0.54), with EQE_1000_ of 16.7%. Importantly, the LT_50_ at initial 1000 cd m^–2^ was 1470 h for the device with only **4CzIPN-Me**, which increased to 3775 hours when the HF-OLED architecture was used. The authors attributed the improved device stability to the rapid and efficient FRET, which effectively reduced the triplet population on **4CzIPN-Me** and alleviates device degradation.

**TBRb** was also employed as the terminal emitter in conjunction with the TADF sensitizer **34AcCzTrz** (Figure 194) and a TADF host **3CzPhpPM** by Lv *et al*..^287^ The yellow-emitting HF device with 4 wt% **34AcCzTrz** and 1 wt% **TBRb** in the emissive layer displayed an EQE_max_ of 19.1% with a near zero efficiency roll-off at 1000 cd m^–2^. This HF device performance was also remarkably improved compared to the non-HF counterpart with 3 wt% **34AcCzTRz** alone as the emitter, which showed an EQE_max_ of 14.5% and had an efficiency roll-off 9.1% at 1000 cd m^–2^.

**Figure 194:**
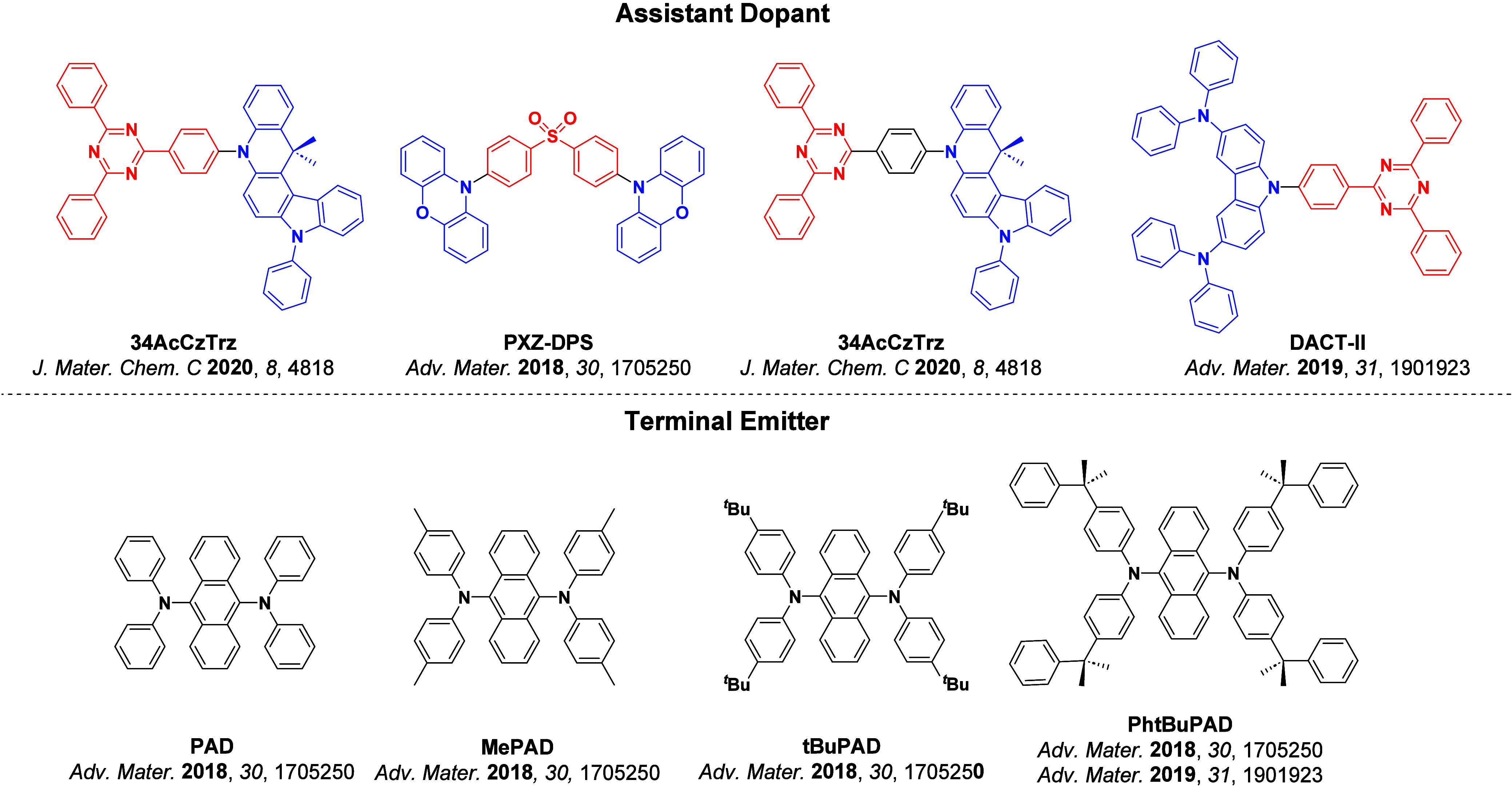
Chemical structures of TADF assistant dopants and fluorescent terminal emitters containing bulky groups, except for the compound terminal emitter PAD. The blue color signifies donor moieties, while the red color signifies acceptor moieties.

Zhang *et al.* adopted a similar strategy by introducing sequentially bulkier groups on the terminal emitter to suppress DET pathways.^1264^ Four fluorescent dyes, **PAD**, **MePAD**, **tBuPAD**, and **PhtBuPAD** (Figure 194), were investigated as terminal emitters in combination with TADF assistant dopant **PXZ-DPS** (Figure 194). The green HF-OLEDs with 1 wt% **PAD**, **MePAD**, **tBuPAD**, and **PhtBuPAD** in combination with 30 wt% **PXZ-DPS** as the assistant dopant showed increasing EQE_max_ of 18.6, 20.2, 22.7 and 24.0%, respectively, with λ_EL_ ranging from 525–540 nm. When the doping concentration of the terminal emitter was between 5–8%, excellent efficiency roll-off out to 5,000 cd m^–2^ was observed for all devices. The same group also reported a device with 3 wt% **PhtBuPAD** as the terminal emitter and 40 wt% **DACT-II** (Figure 194) as the assistant dopant that showed EQE_max_ and PE_max_ of 23.2% and 76.9 lm W^–1^, respectively, where the EQE_5000_ remained as high as 20.0%, and with CIE coordinates of (0.36, 0.60).^1284^

Kim *et al*. reported four orange-colored TADF emitters, **tBIQAC**, **tBIQAP**, **DtBIQAC**, and **DtBIQAP** (Figure 195). Compared to **DMAC**-decorated **tBIQAC** and **tBIQAP**, **DtBIQAC**, and **DtBIQAP** possess a bulkier diphenylacridan donor. Their relative device performance as assistant dopants in conjunction with **DBP** (Figure 189) as the terminal emitter in PBICT host was investigated.^1285^ The **DtBIQAP**- and **DtBIQAC**-based devices showed the highest EQE_max_ of 18.2% and 17.5% with CIE coordinates of (0.62, 0.38) and (0.64, 0.36), respectively, both higher than **tBIQAC**- and **tBIQAP**-based devices with EQE_max_ of 16.8 and 14.7% at CIE coordinates of (0.63, 0.37), respectively. These results again demonstrate that the use of bulky groups on the assistant dopant can effectively increase the EQE of the TADF-assisted fluorescent OLEDs.

**Figure 195:**
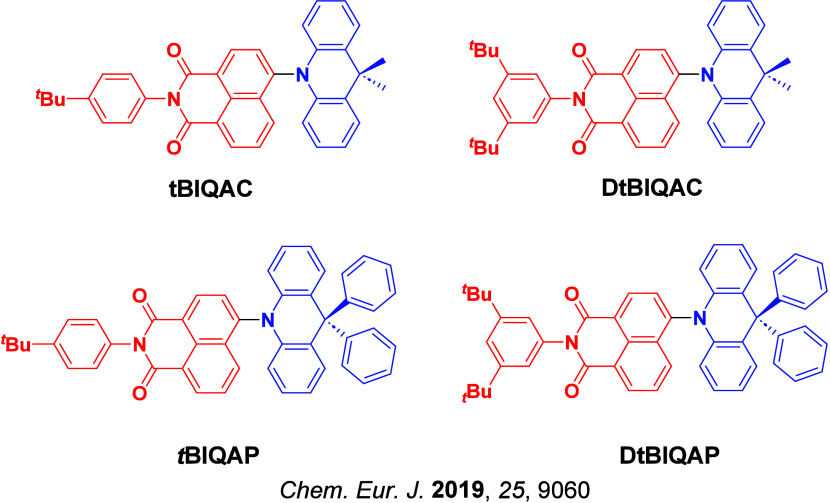
Chemical structures of orange-emitting TADF assistant dopants reported in ref 1285 (the blue color signifies donor moieties, while the red color signifies acceptor moieties).

Besides introducing steric blocking groups, the physical separation of the assistant dopant and the terminal emitter has been used to suppress DET channels. Han *et al*. engineered a multi-layered emissive layer in the OLED, where the assistant dopant and the terminal emitter in DPEPO were alternately deposited.^1286^ The device with 50 wt% **DMAC-DPS** (Figure 196) as the assistant dopant and 1 wt% **TBPe** (Figure 189) as the terminal emitter showed an EQE_max_ of 18.8% at CIE coordinates of (0.14, 0.25), higher than that of the conventional HF-device (EQE_max_ = 13.1%), where **DMAC-DPS** and **TBPe** were co-deposited simultaneously. Later, Chen *et al*. used **DMAC-DPS** as the assistant dopant at the optimized doping concentration of 20 wt% in combination with 1 wt% **TBPe** to produce a blue HF-OLED. The device only exhibited an EQE_max_ of 14.1% at CIE coordinates of (0.14, 0.17).^574^ The results indicate that the strategy employing alternate deposition of assistant dopant and terminal emitter is a possible solution to suppressing DET.

**Figure 196:**
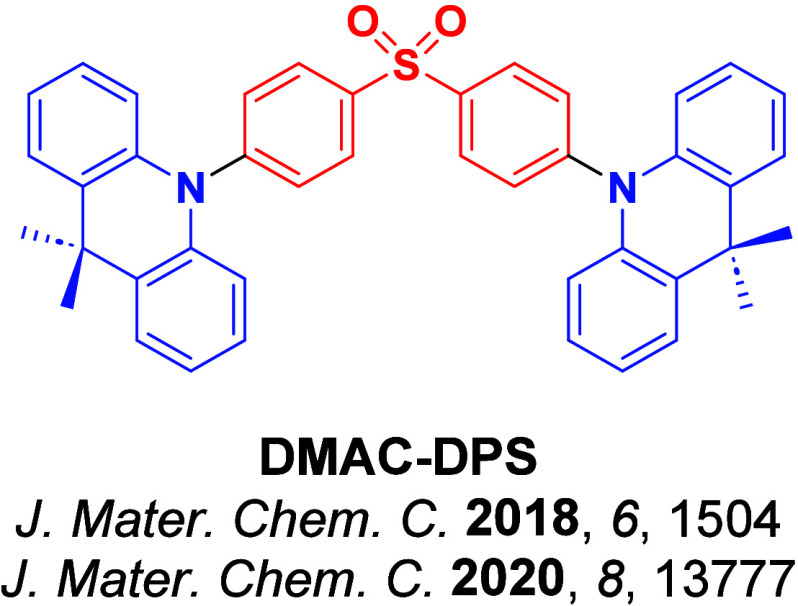
Chemical structure of the TADF assistant dopant **DMAC-DPS** used in refs 1286 and 574 (the blue color signifies donor moieties, while the red color signifies acceptor moieties).

Somewhat different from the conventional sensitization strategy, Ma *et al*. reported an OLED using **Pr-1** (Figure 197) as the assistant dopant and fluorescent luminophore **DCJTB** (Figure 197) as the terminal emitter, both doped in the TADF exciplex **mCBP:​PO-T2T**.^1287^ A competing exciplex was also formed between **Pr-1** and **PO-T2T** in the **mCBP:​PO-T2T** exciplex host. Therefore, three RISC channels in this system could act simultaneously to mitigate TTA and DET, each then feeding into FRET to the terminal emitter. The OLED with 10 wt% **Pr-1** and 1 wt% **DCJTB** in the **mCBP:​PO-T2T** exciplex host as the emissive layer showed CE_max_, PE_max_ and EQE_max_ of 22.6 cd A^–1^, 29.5 lm W^–1^, and 13%, respectively, with an LT_50_ at 1000 cd m^–2^ reaching 415 hours.

**Figure 197:**
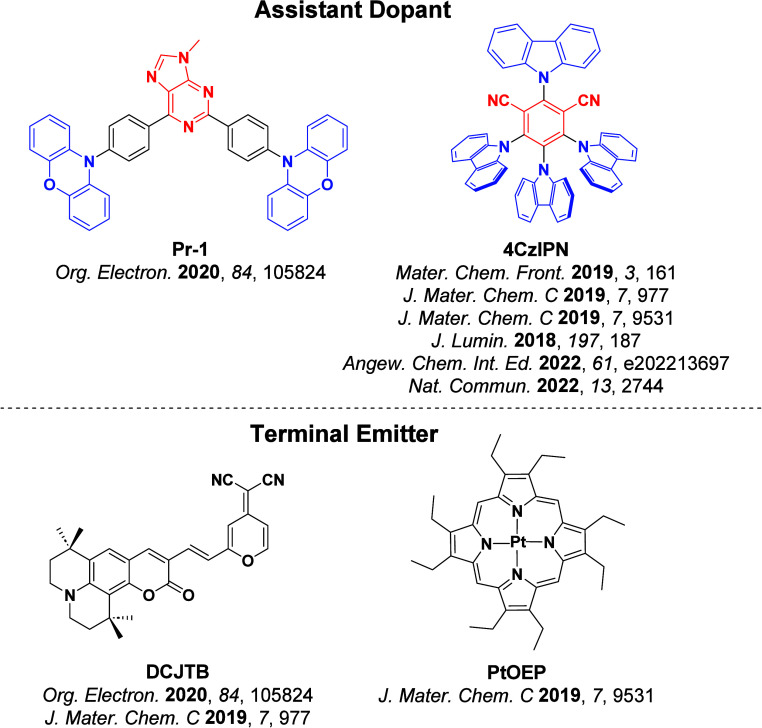
Chemical structures of TADF assistant dopants and fluorescent (**DCJTB**) and phosphorescence (**PtOEP**) terminal emitters in HF-OLEDs (the blue color signifies donor moieties, while the red color signifies acceptor moieties).

Li *et al*.^1266^ similarly explored the use of multiple TADF materials, with **4CzIPN** (Figure 197) and a TADF exciplex **TCTA:​B4PyMPM** host used together with fluorescent terminal emitter, **DCJTB**. Three FRET processes were proposed by the authors, which occur between the exciplex and **DCJTB** (FRET1), the exciplex and **4CzIPN** (FRET2) and **4CzIPN** and **DCJTB** (FRET3), all of which contributed to the harnessing of triplet excitons. The red-emitting device with 2 wt% **4CzIPN** as the assistant dopant and 0.5 wt% **DCJTB** as the terminal emitter showed EQE_max_ of up to 12.9% at CIE coordinates of (0.58, 0.41). A negligible efficiency roll-off of 3.9% was recorded at 100 cd m^–2^. By contrast, a device without the co-assistant dopant **4CzIPN** reached an EQE_max_ of only 7.3%, and a non-HF device with only **4CzIPN** as the TADF dopant exhibited an EQE_max_ of only 6.6%.

Employing the same exciplex co-host methodology, Liao *et al*. reported a novel deep red-emitting (∼650 nm) HF-OLED where the EML was composed of exciplex co-host (**CBP**:​**B4PyMPM**, 1:1), TADF emitter **4CzIPN** (Figure 197) as the assistant dopant, and a phosphorescent complex **PtOEP** (Figure 197) as the terminal emitter.^1288^ In this design, the excitons first form on the exciplex co-host, followed by the energy transfer to **4CzIPN** and to **PtOEP**. The triplet harvesting ability of the phosphorescent terminal emitter also means that this kind of device does not suffer quenching through DET channels. The optimized device used 4 wt% **4CzIPN** and 4 wt% **PtOEP** in **CBP**:​**B4PyMPM** as the EML, and showed an EQE_max_ of 21.5%. The EQE_max_ of the device with just 4 wt% **PtOEP** in **CBP**:​**B4PyMPM** and the device with 6 wt% **PtOEP** in **CBP** were instead ∼17 and 9.1%, respectively. Furthermore, the LT_50_ at 550 cd m^–2^ of an HF-OLEDs using a staircase-doping strategy was improved to 90 hours, double that of the device using a uniformly doped emitting layer.

Jang *et al*. investigated the impact of the dihedral angle of the TADF sensitizer on FRET efficiency, and hence the performance of the HF-OLED devices.^1289^ Two TADF emitters, **BPAc** and **BPAcCz** (Figure 198), both containing a benzophenone acceptor and either DMAc/DMAc or DMAc/carbazole donor groups were used as assistant dopants. The calculated molecular geometries showed a dihedral angle of 89° between DMAc and benzophenone for **BPAc**, while a more planar conformation was observed between carbazole and benzophenone (dihedral angle 49°) in **BPAcCz**. The devices with 20 wt% **BPAc** or **BPAcCz** and 0.5 wt% **6tBPA** (Figure 192) as the terminal emitter in DPEPO showed EQE_max_ of 16.6 and 15.0%, respectively. The authors asserted that the planar geometry of **BPAcCz** should be responsible for enhanced DET in the HF-OLEDs, this geometry allowing increased short-distance interactions in the emissive layer. To validate this hypothesis, 1 wt% of the blue emitter **AnTP** (Figure 198) was also dispersed in DPEPO alongside 20 wt% of **BPAc** or **BPAcCz**. The **AnTP**-doped system was chosen as it only allowed for DET to occur but, with inhibited FRET due to the large singlet energy of **AnTP**. Time-resolved decay measurements verified that the more perpendicularly structured **BPAc** could indeed suppress DET compared to more planarized **BPAcCz**.

**Figure 198:**
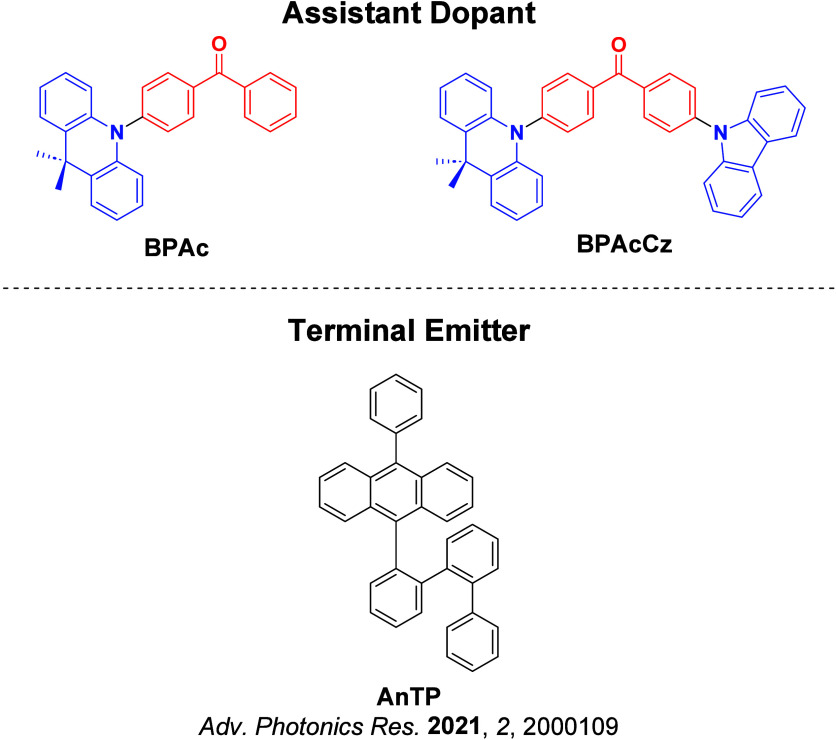
Chemical structures of TADF assistant dopants used with the **AnTP** terminal emitter in a study examining the impact of the TADF sensitizer’s dihedral angle on FRET efficiency, used in ref 1289. The blue color signifies donor moieties, while the red color signifies acceptor moieties.

To date, it remains highly challenging to achieve both large singlet radiative rate (k_r_^s^) and small electron exchange energy (*J*) to produce high-performance red emitters for OLEDs (see Section 5). To overcome this issue, the TADF sensitization strategy was employed by Chen *et al*.^1290^ Solution-processed red-emitting HF-OLEDs using conventional red fluorescent emitter **DBP** (2 wt%) (Figure 189) and the green-emitting TADF assistant dopant **DC-TC** (15 wt%) (Figure 199) in CBP host showed an EQE_max_ of 8.0% at CIE coordinates of (0.61, 0.38). An alternative TADF emitter, **DC-ACR** (Figure 199), was also used as an alternative assistant dopant, which demonstrated a much lower efficiency (EQE_max_ = 4.25%) due to trapping of excitons on the **DBP** emitter directly, which limited the FRET process.

**Figure 199:**
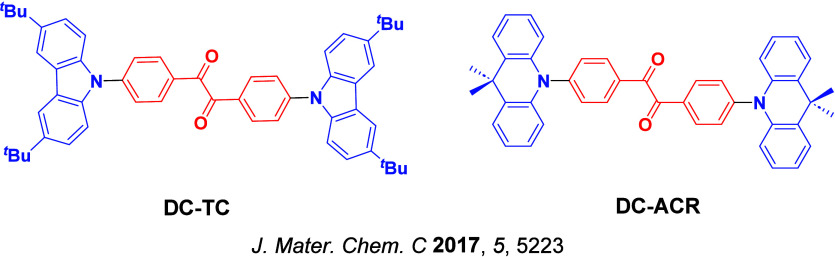
Chemical structures of green-emitting TADF assistant dopants **DC-TC** and **DC-ACR** used in ref 1290.

Wang *et al*. fabricated a red-emitting device (λ_EL_ = 612 nm) using 0.5 wt% **OTPA-BT-CN** (Figure 200) as the terminal emitter alongside 25 wt% **OSTFB** (Figure 200) as the assistant dopant in mCP, resulting in an EQE_max_ of 12.4%.^1265^ The use of **4CzIPN** (Figure 197) as an alternative assistant dopant, resulted in an HF-OLED with much lower EQE_max_ of 6.3%, despite having similar spectral overlap with the terminal emitter. The higher *k*_r_ in **OSTFB** was determined to be the main reason for these differences in device performance, which generally leads to a high-efficiency energy transfer.

**Figure 200:**
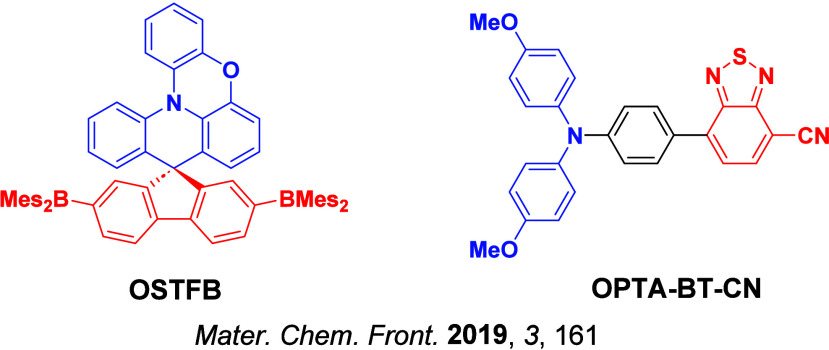
Chemical structures of the TADF assistant dopant **OSTFB** and the TADF terminal emitter **OPTA-BT-CN** used in the HF-OLEDs reported in ref 1265 (the blue color signifies donor moieties, while the red color signifies acceptor moieties).

TADF assistant dopants have also been used with phosphorescent complexes as terminal emitters, where energy transfer still occurs via FRET between the S_1_ state of the TADF compound to the S_1_ state of the phosphorescent emitter. Singlet FRET is followed by ISC to the T_1_ state and phosphorescence emission, while DET to the phosphorescent emitter (or direct recombination) can also lead to emission. Exotic triplet-to-singlet energy transfer may also be active in such systems.^1291,1292^ Liao *et al*. reported a solution-processed WOLED using 10 wt% of TADF dendrimer **BPS** (Figure 201) as the assistant dopant and 0.5 wt% **Ir(bt)_2_acac** (Figure 201) as the emitter in co-host **DCzPPy:​OXD-7** (100:40 ratio).^1293^ The device achieved an EQE_max_ of 6.6%, CE_max_ of 17.34 cd A^–1^ and the CIE coordinates varied by only (0.02, 0.02) across the luminance range of 100 to 10000 cd m^–2^, indicating good color stability and energy transfer within the device. Another example of this strategy involved combination of the red phosphorescent terminal emitter, **Hex-Ir(phq)_2_(acac)** (Figure 201), and **4CzIPN** (Figure 197) together in CBP host to produce red phosphorescent OLEDs.^1294^ The device with 1.5 wt% of terminal emitter and 7.5 wt% of TADF assistant dopant showed an EQE_max_ of 9.8% and an impressive maximum brightness of 52,204 cd m^–2^, compared to 7.9% and 12,200 cd m^–2^ in devices without **4CzIPN**. The enhanced brightness resulted from improved exciton utilisation resulting from efficient triplet harvesting. However, FRET was incomplete with some emission still observed directly from **4CzIPN** (a trait also observed by Wang *et al*. in a previously discussed example).^1265^

**Figure 201:**
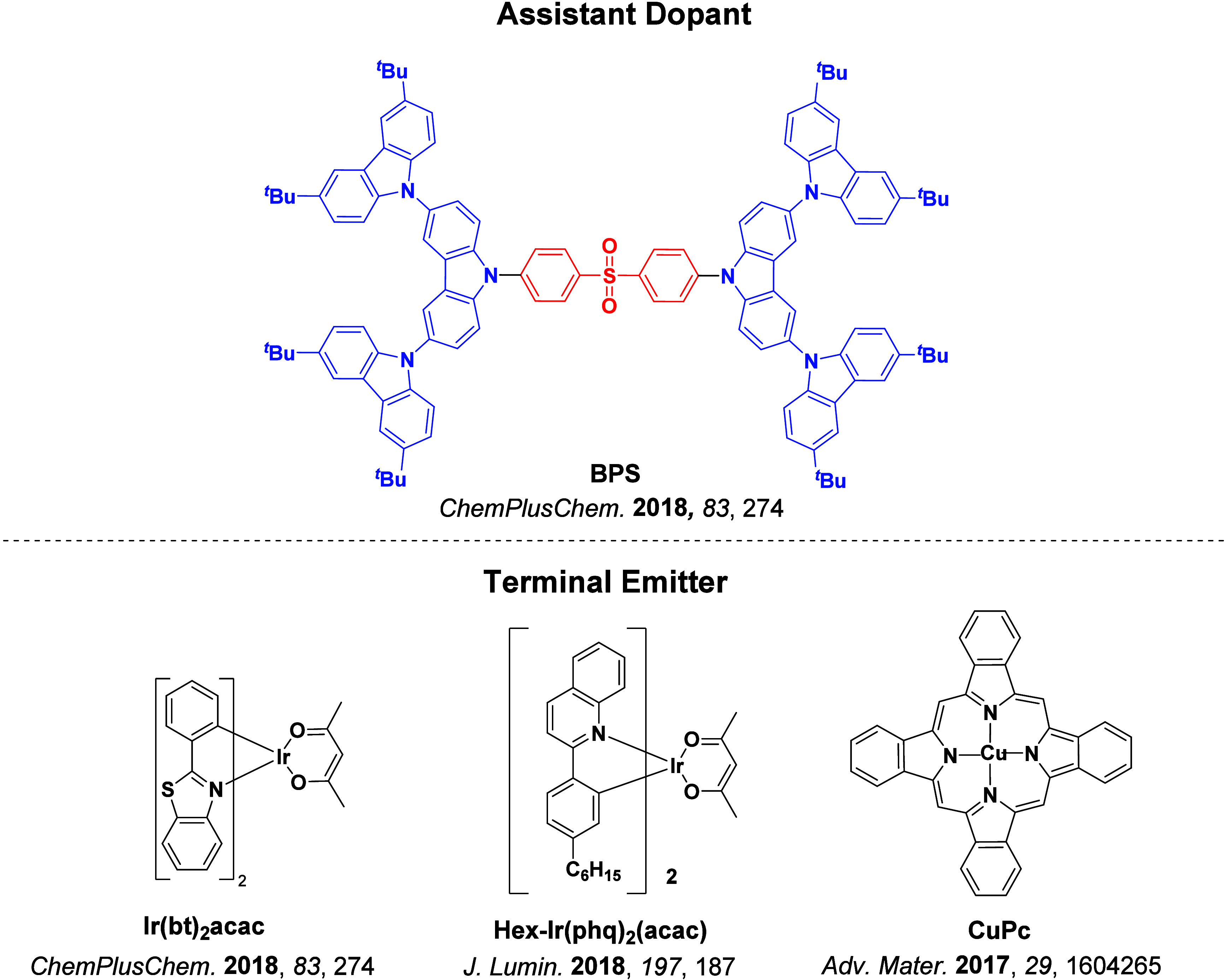
Chemical structures of the TADF dendrimer **BPS** as assistant dopants and the iridium(III) and Cu(II) phosphorescent terminal emitters (the blue color signifies donor moieties, while the red color signifies acceptor moieties).

Aside from iridium complexes, copper complexes have also been used as terminal phosphorescent emitters in OLEDs of this type, though some copper complexes can also emit via TADF or dual TADF/phosphorescence (see Section 9). Nagata *et al*. used **PXZ-TRZ** (Figure 189) as the assistant dopant and **CuPc** (Figure 201) as NIR phosphorescent terminal emitter,^1295^ both dispersed into mCBP host to produce an OLED that showed an EQE_max_ of 0.037%. Despite further advances in NIR OLEDs in the years since (see Section 5), this low EQE performance was still impressive at the time. Due to the large distance between the assistant dopant and the **CuPc** terminal emitter at the relative doping concentrations used, and the large separation between the triplet levels (T_1_PXZ‑TRZ_ – T_1_CuPc_ = 1.1 eV), the main energy transfer route between the two was assigned to FRET.

NIR OLEDs are of particular interest for applications as light sources for optical communication, medical and biological imaging systems, and for military use, including night vision goggles. Shahalizad *et al*. designed the red TADF emitter, **TPAM-BF2** (Figure 202), which emits variously at λ_PL_ of 746 nm, 752 nm, and 764 nm with associated Φ_PL_ of 41.9, 25.0, and 13.7% when doped in CBP at concentrations of 6, 10 and 20 wt%, all respectively.^1296^ When 20 wt% **TPAM-BF2** in CBP was used as the assistant dopant in conjunction with 0.5 wt% of the NIR fluorescent emitter, **BPPC-Ph** (Figure 202), the solution-processed OLED showed an EQE_max_ of 3.5% and with notably narrowband emission (FWHM < 40 nm) at 840 nm. **BPPC-Ph** was also used in another report,^1297^ now named as **BPPC**, as the terminal emitter at 0.8 wt% doping in combination with 20 wt% **TPA-DCPP** (Figure 202) as the TADF assistant dopant in **B3PYMPM** host, which gave an impressive EQE_max_ of 5.4% at λ_PL_ of 790 nm.^1297^ Notably, most organic NIR OLEDs have a considerable fraction of their spectral power density (>50%) in the visible range, while the **TPA-DCPP**-based NIR device displayed narrowband NIR emission with 90% of total emission beyond 750 nm, and an NIR cut-on wavelength (corresponding to 10% of the peak PL intensity) of 790 nm.

**Figure 202:**
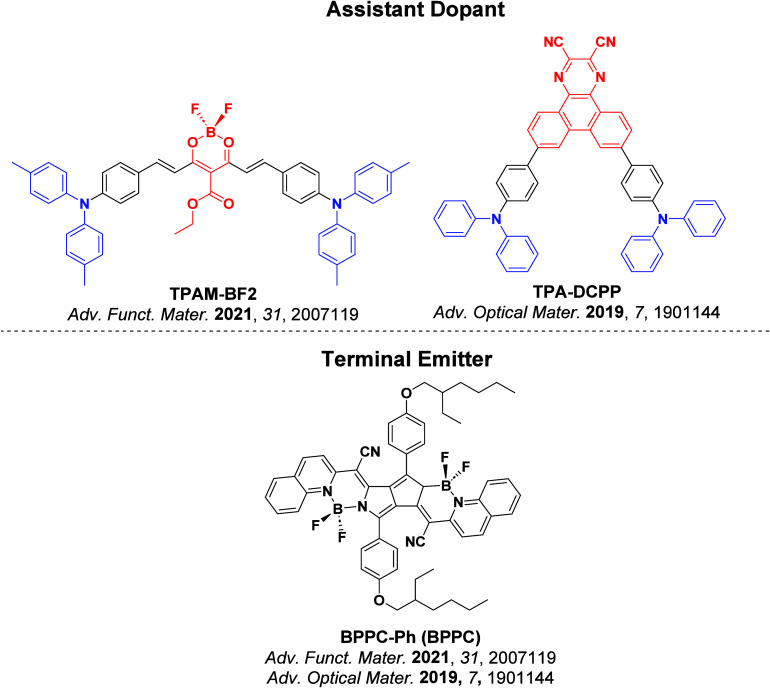
Chemical structures of TADF assistant dopants and the terminal emitter **BPPC-Ph** (aka **BPPC**) used in NIR HF-OLEDs in refs 1296 and 1297. The blue color signifies donor moieties, while the red color signifies acceptor moieties.

Bartkowski *et al*.^1298^ employed a D-A TADF compound (**tBuCz-σ-NI**, **2** in that work) as the assistant dopant and structurally analogous but rigidified fused-aromatic emitter (**tBuCz-π-NI**, **5** in that work) (Figure 203) as the terminal emitter to realize a narrowband emitting HF-OLED. The green-emitting device with 10 wt% **tBuCz-σ-NI** and 0.6 wt% as **tBuCz-π-NI** in mCP showed an EQE_max_ of 27% and FWHM of 40 nm. A similar popular strategy of obtaining narrowband emission is to use MR-TADF compounds as terminal emitters. Examples of HF-OLEDs using MR-TADF compounds as terminal emitters are summarised in Section 11.

**Figure 203:**
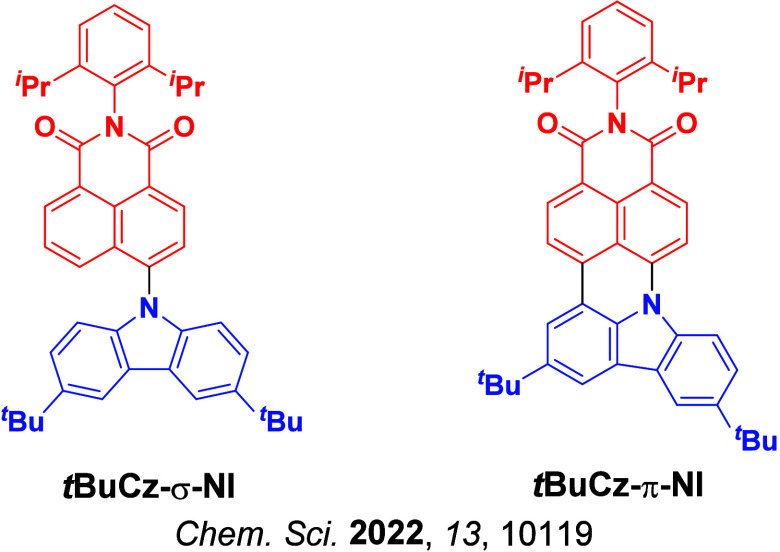
Chemical structures of the TADF assistant dopant **tBuCz-σ-NI** and the TADF terminal emitter **tBuCz-π-NI** used in the narrowband emitting HF-OLED from ref 1298 (the blue color signifies donor moieties, while the red color signifies acceptor moieties).

Although most HF-OLEDs contain purely organic TADF assistant dopants, organometallic TADF complexes have also been used as co-dopants. Zhan *et al*. employed a copper-based CMA complex (MAC*)Cu(Cz) (Figure 204) at 20 wt% doping with 1 wt% **TBRb** (Figure 189) as the terminal emitter in mCBP host.^1299^ The yellow-emitting HF-OLED (λ_EL_ = 566 nm) showed an EQE_max_ of 14.6%, with a very low-efficiency roll-off of 12% at 1000 cd m^–2^. The LT_50_ of the device was 767 hours at an initial luminance of 100 cd m^–2^. Further, a device with MR-TADF emitter **BN3** (Figure 204) instead of **TBRb** exhibited an improved EQE_max_ of 26.5%, which only decreased to 10.5% at a luminance of 10,000 cd m^–2^.

**Figure 204:**
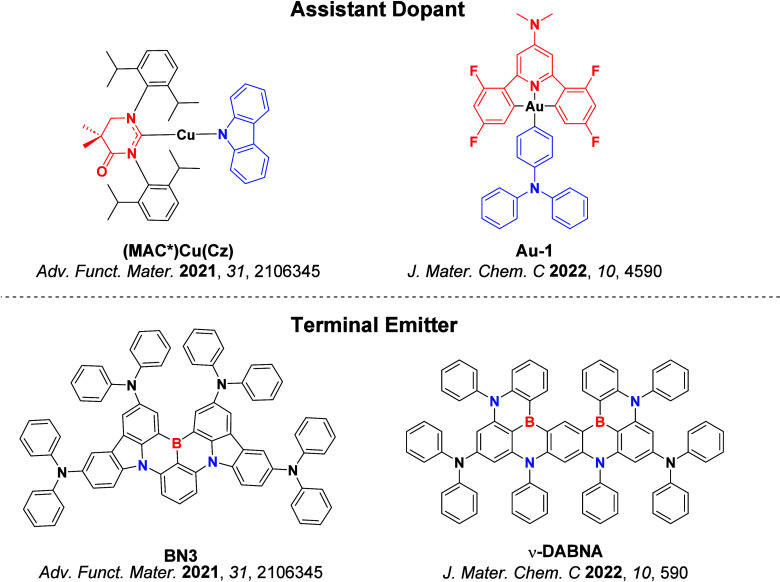
Chemical structures of the organometallic TADF assistant dopants and the MR-TADF terminal emitters used in refs 1299 and 1300 (the blue color signifies donor moieties/atoms, while the red color signifies acceptor moieties/atoms).

A second example of the same strategy saw the use of a gold (III) TADF complex **Au-1** (Figure 204) as the assistant dopant.^1300^ The blue-emitting HF device with 10 wt% of **Au-1** as the sensitizer and 0.5 wt% ***v*-DABNA** (Figure 204) as the terminal emitter in PYD2 host showed an EQE_max_ of 16.6%, which remained as high as 14.4% at 1000 cd m^–2^.

Finally, TADF assistant dopants have recently been used in conjunction with doublet organic radical emitters in an HF-OLED. The red-emitting HF-OLED contained 3 wt% of the radical emitter **TTM-3PCz** (Figure 205) and 25 wt% **4CzIPN** (Figure 197) in CBP,^1301^ and showed an EQE_max_ of 16.4% with a broad emission band ranging from 680–800 nm. The LT_50_ at 0.4 mA cm^–2^ of the HF-OLED was only 42 min, likely due to the instability of the radical species. This was nonetheless a higher efficiency than the device with only **TTM-3PCz**, which showed an EQE_max_ of 10.7%, although theoretically doublet OLEDs entirely avoid the problem of triplet harvesting, and so may not need to rely on HF-OLED strategies once sufficiently developed.

**Figure 205:**
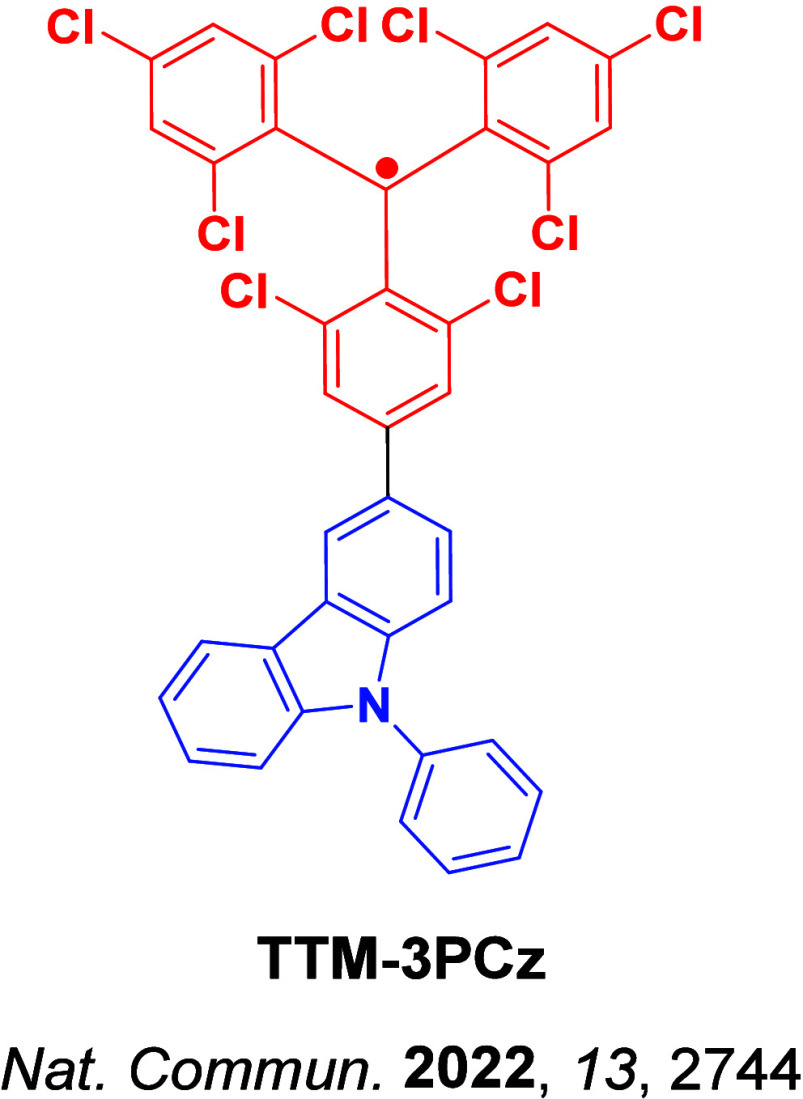
Chemical structure of the doublet terminal emitter **TTM-3PCz** from ref 1301 (the blue color signifies donor moieties, while the red color signifies acceptor moieties).

### 17.3 Outlook

From an assessment of the performance of HF-OLEDs compared to normal TADF OLEDs, it is clear that this mixed-materials approach can achieve significant improvements in efficiency, efficiency roll-off, and color purity. As an emblematic example, EQE_max_ of over 38% at blue CIE coordinates of (0.12,0.15) have been reported using leading D-A TADF sensitizers with the MR-TADF terminal emitter **ν-DABNA** (Figure 203 and Figure 206).^1302^ Similarly high efficiency green and red devices have been fabricated. For instance, an EQE_max_ of 27.0% at CIE coordinates of (0.38,0.59) was achieved for a green OLED with the D-A TADF sensitizer ***t*BuCz-σ-NI** and the fluorescent terminal emitter ***t*BuCz-π-NI**,^1303^ while the highest efficiency red OLED showed an EQE_max_ of 21.5% at CIE coordinates of (0.72, 0.30) with the D-A TADF sensitizer **4CzIPN** and the phosphorescent terminal emitter **PtOEP**.^1288^ This trend in efficiency results from the fact that fast *k* ^s^_r_ and high Φ_PL_ of the terminal emitter can be decoupled from the exciton harvesting efficiency provided by the TADF assistant dopant, allowing the HF systems to benefit from advances in both separate fields.^1288^ Despite the additional challenges associated with multiple material depositions and complex energy transfer pathways, it is likely that HF-TADF OLEDs will continue to claim record device efficiencies, particularly at higher brightness, for the foreseeable future. This includes at extreme color coordinates, a recent development supported with the use of MR-TADF materials as narrowband terminal emitters and explains the prominence of the HF-OLED strategy seen across MR-TADF research activity (see Section 11).

**Figure 206:**
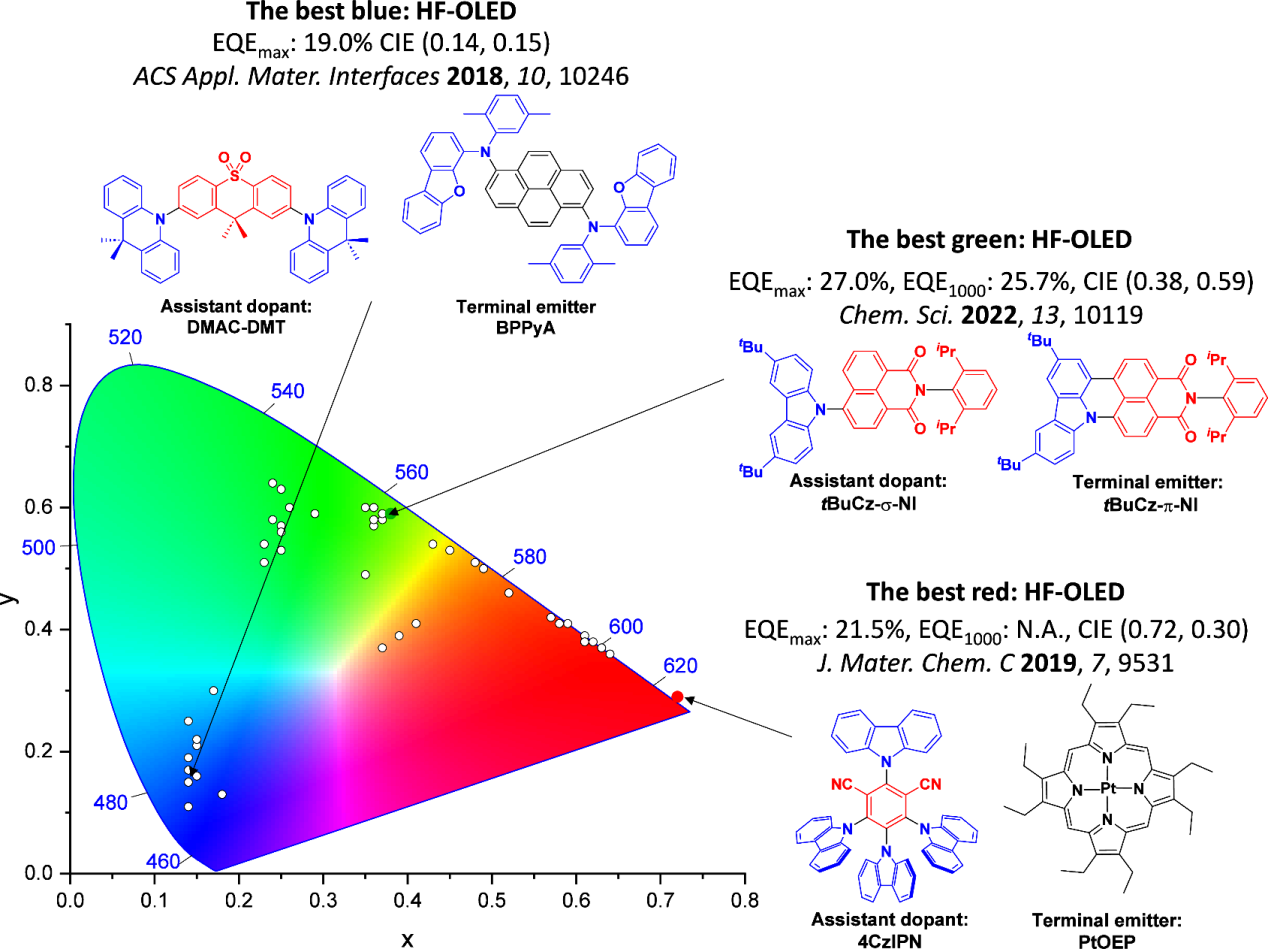
CIE color coordinates of high-performance HF-OLEDs. The white circles illustrate the spread of the emission color of the device. Selected devices and their associated CIE coordinates are highlighted, illustrating the structures of the emitter and HF-OLED assistant dopants of the highest efficiency blue, green, and red emission quantified by the EQE_max_. In the chemical structures, the blue color signifies donor moieties, while the red color signifies acceptor moieties.

As well as enabling progressive improvements in efficiency and color purity, it has recently been shown that HF sensitiser/emitter pairs featuring surprisingly low FRET overlap can nonetheless efficiently drive blue OLED emission using green-emitting sensitisers. This development allows the use of lower energy (and intrinsically more stable) emitters and hosts for blue OLEDs and may hence unlock significant gains in device stability that have persistently eluded research efforts at this wavelength range.^1037,1304^ At the same time, the underlying processes that control HF-OLED device performance remain poorly understood and is an area ripe for new experimental methods to be developed to yield new insights into device and materials design. In this context, we anticipate that HF-OLED development will continue to grip the attention of applied, fundamental, and computational research in the short and medium term.

## 18 TADF Materials as Hosts

### 18.1 Introduction

Due to their ambipolar character resulting from these materials comprising both electron-donating and electron-accepting moieties, many of the D-A TADF materials employed as emitters in previous sections are potentially also useful as host materials in OLEDs. The bipolar nature of TADF materials as hosts allows them to promote balanced charge transport into the EML.^1305^ The TADF host can also assist in exciton harvesting via RISC, followed by FRET to the guest emitter.^1306,1307^ This FRET process remains feasible even at low doping concentrations of the terminal emitter, which is particularly beneficial for improving the efficiency of OLEDs employing fluorescent emitters in the hyperfluorescence category of devices (see Section 17). In phosphorescent devices, the use of TADF host systems has also been demonstrated to lead to devices with improved efficiency and stability, even at <1 wt% doping of the emitter.^1308^ Of course, the capacity to support guest emitters of a particular energy necessitates that the TADF host itself has a sufficiently high triplet energy, so that excitons are confined on the terminal emitter. There are two classes of TADF compounds that have been explored as hosts: D-A TADF compounds and exciplexes (intermolecular donor-acceptor mixtures, see Section 8). Examples of OLEDs using D-A TADF hosts are examined here and are split into three groups based on the nature of the emissive material: phosphorescent, fluorescent, and TADF. The device performance for the OLEDs discussed in this section are collated in Table S23.

### 18.2 TADF Hosts with Phosphorescent Emitters

The first reported OLEDs with TADF materials used as hosts featured phosphorescent emitters (Figure 207). Zhang *et al*. demonstrated early on that the device lifetime is less sensitive to the doping concentration of ***fac***-**Ir(ppy)_3_** (≤3 wt%) when a TADF host is used, compared to a conventional host such as CBP.^1248^ The authors compared to the TADF host **PBICT** (λ_PL_ = 488 nm, E_T_ = 2.66 eV in neat film, and Δ*E*_ST_ = 0.10 eV in DCM), consisting of an indolocarbazole donor and triazine acceptor, which was doped with the phosphorescent green emitter (λ_PL_ = 507 nm in CHCl_3_). In thin films with very low emitter doping concentrations (0.5 – 3.0 wt%), the energy transfer process is mainly governed by long-range FRET from the TADF host to the phosphorescent guest. This supports the improved efficiency of the **PBICT:​Ir(ppy)_3_** devices (EQE_max_ = 23.9% at 3 wt% of the emitter) compared to the **CBP:​Ir(ppy)_3_** devices (EQE_max_ = 14.5% at 3 wt% of the emitter).^1248^ The efficiency could be further improved by employing **DIC-TRZ** as the TADF host, in part due to an even smaller Δ*E*_ST_ (0.06 eV in DCM). The authors compared the EQE among devices featuring differing dopant concentrations (EQE_max,x%_) with respect to the highest EQE_max_ recorded (EQE_max,all_). In the case of devices using **DIC-TRZ**, the EQE_max,all_ was achieved at a dopant concentration of 2 wt%. On the other hand, for devices using **PBICT**, the highest EQE_max,all_ was attained at a dopant concentration of 3 wt%. Moreover, at very low doping levels (0.5 wt%), the devices using **DIC-TRZ** attained ∼92% of the EQE_max,all_. In contrast, for the devices using **PBICT** only 80% of the EQE_max,all_ was attained at such a low loading (0.5 wt%). The authors thus concluded that a faster RISC rate and a higher RISC efficiency were enabled by the smaller Δ*E*_ST_ of **DIC-TRZ**, supporting greater device efficiency for the eventual phosphorescence emission.

**Figure 207:**
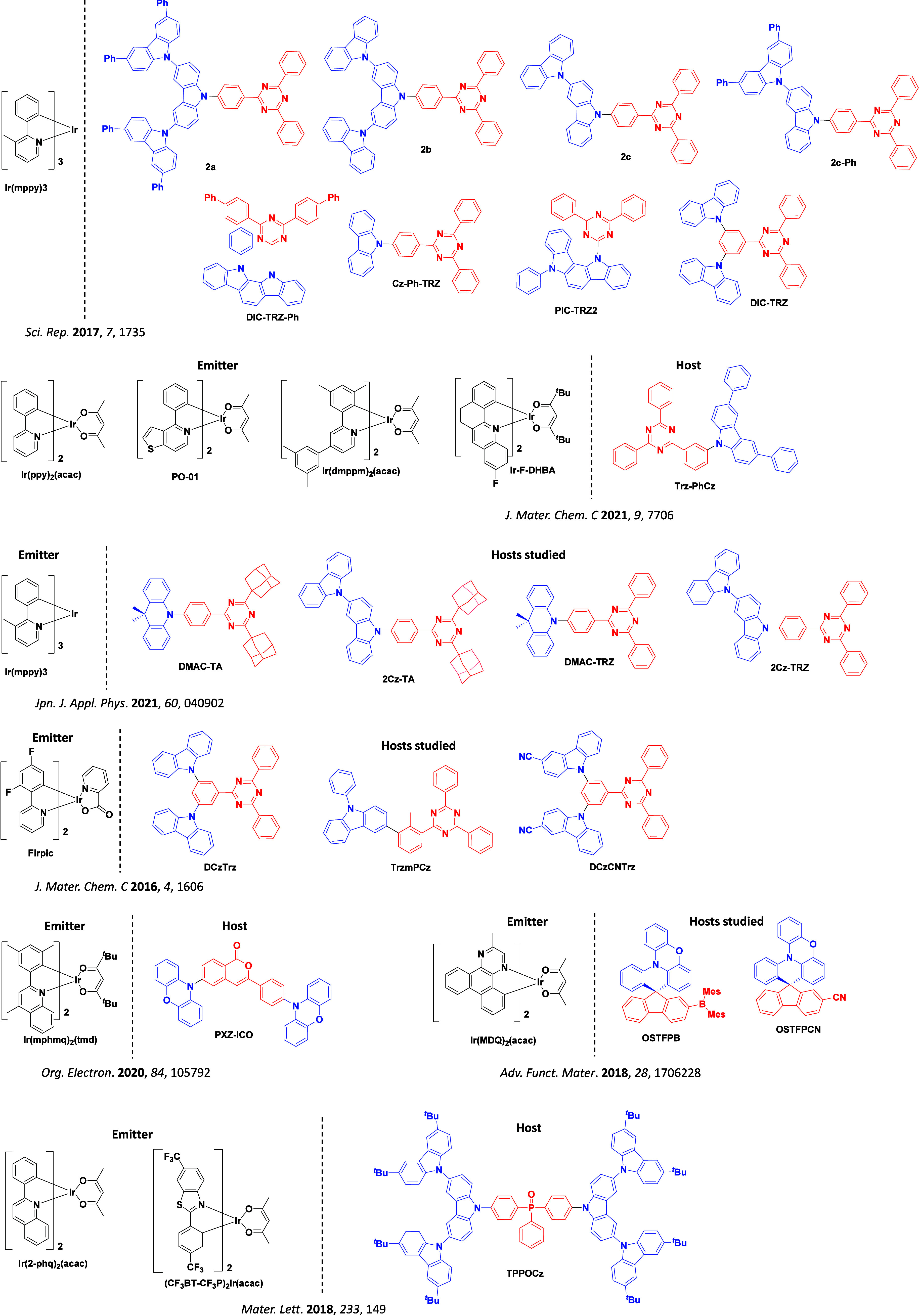
Chemical structures of TADF hosts used with phosphorescent emitters (the blue color signifies donor moieties, while the red color signifies acceptor moieties).

In a subsequent study the same group investigated the device performance using a phosphorescent orange emitter (**PO-01**, Figure 207) doped in a series of indolocarbazole-triazine TADF hosts.^1309^ **POBICT**, **BICT**, **PBICT**, and **BBICT** have Δ*E*_ST_/*E*_T_ of 0.34/2.70, 0.28/2.70, 0.10/2.66 and 0.06/2.47 eV, respectively, in DCM. The devices were compared using the same doping concentration of 10 wt% **PO-01** in the hosts, and it was found that **PBICT** with a combination of low Δ*E*_ST_ and high *E_T_* translated into the best device performance. A low efficiency roll-off was also observed for this OLED, with an EQE_max_ = 24.5%, EQE_1000_ = 24.2%, and EQE_10,000_ = 23.8%. Despite having the smallest Δ*E*_ST_, the device employing **BBICT** showed poorer performance (EQE_max_ = 13.7%, EQE_1000_ = 13.6% and EQE_10,000_ = 13.1%), attributed to the energy mismatch with **PO-01**. The LUMO level of **BBICT**, E_LUMO_ = −2.80 eV, is lower than that of **PO-01**, E_LUMO_ = −2.70 eV, which leads to inefficient charge recombination and confinement on the phosphorescent emitter. Encouraged by their preliminary success with **PBICT** as a host material, the same group sought to further optimize the host through the addition of nitrile groups on the phenyl rings of the triazine to generate **BCPICT**.^1310^ The OLED using **BCPICT** (λ_PL_ = 575 nm, *E*_T_ = 2.76 eV, and Δ*E*_ST_ = 0.08 eV in toluene) as the host showed an EQE_max_ = 10.5% and an EQE_1000_ = 9.9% in combination with the phosphorescent red emitter **Ir(mphmq)_2_(tmd)** at 2 wt% doping concentration.

Duan and co-workers have investigated the blue TADF emitter **DMAC-DPS** (Figure 207) as a host material.^562^ In their study white OLEDs were fabricated by combining the blue-emitting TADF host with orange-emitting phosphor **PO-01**, and controlling the degree of energy transfer from host to guest and overall EL color by modulating the doping level. The best white OLED, doped with 0.8 wt% **PO-01**, showed EQE_max_/EQE_1000_ of 20.8/19.6% and PE_1000_ of 38.7 lm W^–1^ at CIE coordinates of (0.398, 0.456), and even at 5000 cd m^–2^ the EQE remained above 15%.^562^ **DMAC-DPS** was also used as a host to demonstrate efficient green, red, and white OLEDs using green **Ir(ppy)_2_(acac**) and red **Ir(mphmq)_2_(tmd)** phosphorescent dopants.^580^ The red and green devices showed similarly high EQE_max_ of 22.4 and 19.5%, with the efficiencies remaining as high as 19.6 and 18.7%, respectively, at 5000 cd m^–2^. The EML of the white devices contained **DMAC-DPS** with 0.2 wt% of both the green **Ir(ppy)_2_(acac)** and red **Ir(mphmq)_2_(tmd)** dopants, and displayed CIE coordinates of (0.360, 0.390), (0.352, 0.387) and (0.364, 0.390) at voltages of 5, 7, and 9 V, respectively. The EQE_max_ reported for this white device was 20.2%, and the efficiency roll-off was very low with an EQE_1000_ of 19.4%.

A *meta*-linked isomeric variant of **DMAC-DPS**, **mSOAD** (Figure 207), was used by Wang *et al.* as a host for the red phosphorescent emitter **Ir(pq)_2_acac**.^1311^ **mSOAD** possesses a high triplet energy of 2.91 eV, a small Δ*E*_ST_ of 0.01 eV, and a short t_d_ of 2.11 μs in the crystalline state.^1312^ The best red device was achieved using 4 wt% of the emitter within the EML, and showed an EQE_max_ of 20.3% and an EQE_1000_ of 10.8%.^1311^ By reducing the concentration of the red emitter to between 0.4–1.5 wt%, incomplete energy transfer occurs and white emission is produced from the combined emissions of the blue host and red guest. The EQE_max_ of the WOLEDs ranged from 12.2–17.4%, and the EQE_1000_ varied from 4.8–13.0%, depending strongly on the emitter doping concentration. The emission CIE coordinates for devices with dopant concentration of 1.5, 0.8, and 0.4 wt% were (0.549, 0.399), (0.448, 0.400) and (0.032, 0.415), respectively. Further evaluation of sulfone-based TADF compounds as host materials was conducted by Xia *et al.*,^1313^ using both **mSOAD** or the carbazole analogue ***t*Bu-mSOCz** (λ_PL_ = 440 nm, *E*_T_ = 2.88 eV and ΔE_ST_ = 0.42 eV in toluene). These hosts were combined with sky-blue (**FIrpic**, λ_EL_ = 471 nm), green (***fac*-Ir(ppy)_3_**, λ_EL_ = 515 nm), or red (**Ir(pq)_2_acac**, λ_EL_ = 606 nm) phosphorescent dopants to produce PhOLEDs.^1313^ The best blue device employed ***t*Bu-mSOCz** with 6 wt% of **FIrpic**, and showed *L*_max_, EQE_max_, and EQE_1000_ of 6176 cd m^–2^, 14.7%, and 13.3%, respectively. The best green devices employed **mSOAD** with 4 wt% of ***fac*-Ir(ppy)_3_**, and achieved *L*_max_, EQE_max_, and EQE_1000_ of 35,530 cd m^–2^, 19.0%, and 18.4%. **mSOAD** was also the host of choice for the red PhOLEDs with 4 wt% of **Ir(pq)_2_acac**, with corresponding *L*_max_, EQE_max_, and EQE_1000_ of 19,420 cd m^–2^, 20.3%, and 10.6%. Related A-D-D-A carbazole-sulfone TADF material **BCz-2SO** (λ_PL_ = 410 nm, *E*_T_ = 2.91 eV, and Δ*E*_ST_ = 0.35 eV in toluene) has also been explored as a host in PhOLEDs with the sky-blue emitter **FIrpic**.^1314^ The solution-processed device with 1 wt% doping of the emitter showed an EQE_max_ of 7.8% and a *L*_max_ of 16,537 cd m^–2^.

Lin *et al*. reported deep-blue TADF materials **BT-01** (λ_PL_ = 396 nm, *E*_T_ = 3.00 eV, and Δ*E*_ST_ = 0.45 eV in neat film) and **BT-02** (λ_PL_ = 375 nm, *E*_T_ = 3.03 eV, and Δ*E*_ST_ = 0.52 eV in neat film, Figure 207) and demonstrated their potential as hosts for phosphorescent and TADF OLEDs.^1315^ Both compounds are composed of a sulfone acceptor and carbazole donor that are electronically decoupled through a *m*-bitolyl bridge. The cyano group attached to the carbazole in **BT-02** explains its blue-shifted emission compared to **BT-01**, and likely also contributes to charge transport as an OLED host material. Both molecules showed delayed emission despite their large Δ*E*_ST_ values (0.45 and 0.52 eV for **BT-01** and **BT-02**, respectively, in neat film). This result implies the involvement of higher-lying triplet states enabling RISC, which was further supported by lower measured TADF activation energies of 0.067 and 0.109 eV, and surprisingly short t_d_ of 1.3 and 1.8 μs for **BT-01** and **BT-02**, respectively. Devices with **FIrpic** as the emitter using **BT-01** or **BT-02** as the host showed EQE_max_/EQE_1000_ of 31.8/31.2% and 30.7/29.9%, respectively. The combination of a rather high dopant concentration of 10 wt%, bipolar charge transport by the host, and orbital alignment between host and guest led not only to these high efficiencies but also to the very low efficiency roll-off. Indeed, the efficiency roll-off was much higher when the emitter was switched to **2CzPN**, with the efficiency sharply decreasing from EQE_max_ of 25.5 and 22.3% to EQE_1000_ of 10.0 and 6.2% for **BT-01** and **BT-02** as hosts, respectively.

The first use of pyrimidine-based TADF compounds as hosts was reported by Wang *et al.*, containing either acridine (**DMAC-BPP**: λ_PL_ = 502 nm, *E*_T_ = 2.50 eV, and Δ*E*_ST_ = 0.03 eV in toluene) or δ-carboline (**DCb-BPP**: λ_PL_ = 452 nm, *E*_T_ = 2.54 eV, and Δ*E*_ST_ = 0.20 eV in toluene, Figure 207) as the donor units.^1316^ These hosts were used in conjunction with 5 wt% of phosphorescent emitter **PO-01**. The device using **DCb-BPP** as the host exhibited an EQE_max_ of 21.5% and an EQE of 17.7% at 10,000 cd m^–2^. In addition, the OLED showed a long operational lifetime with an LT_50_ of 424 h at initial brightness of 1000 cd m^–2^. The device using **DMAC-BPP** as the host showed similar performance (EQE_max_ of 19.8% and EQE_10,000_ of 17.9%) to that of **DCb-BPP**; however, the lifetime of the **DMAC-BPP** device was only about 5% of the **DCb-BPP** device.

The influence of the host on the operational stability of OLEDs was assessed by Fukagawa *et al*. by investigating a series of triazine-containing TADF hosts with the same green phosphorescent emitter ***fac*-Ir(mppy)_3_**.^233^ Across hosts **2a**, **2b**, **2c**, **2c-Ph**, **Cz-Ph-TRZ**, **PIC-TRZ2**, **DIC-TRZ**, and **DIC-TRZ-Ph** (Figure 207), the authors found that the *k*_RISC_ of the host strongly indicates the device lifetime. This conclusion applies most strongly when the emitter is doped at very low concentration within the EML, as the FRET rate between host and guest, *k*_FRET_, also affects the device lifetimes. The highest performing device used **2c** as the host and had a *k*_FRET_ of 10.0 × 10^8^ s^–1^; the authors did not however provide the value for the host *k*_RISC_. This device showed an EQE_max_ of 21.5% as well as an excellent lifetime (LT_50_) of 20,000 h from an initial 1000 cd m^–2^. To better understand the role that the TADF host plays in the success of the guest emitter, the authors also investigated TADF-inactive **Cz-Ph-TRZ** as a reference host. The LT50 of the PhOLED using **Cz-Ph-TRZ**, in which triplet up-conversion on the host is suppressed, is about 500 hours: 40-fold shorter than the device using **2c** as the host. Additionally, triazine-containing hosts were shown to form exciplexes with the platinum-based emitter **PtN7N**, which adversely impacts the device performance and color. To overcome this problem, sterically hindered triazines within the host material can be employed to suppress exciplex formation. Accordingly, devices with **PIC-TRZ2** as the host did not suffer the same degree of exciplex formation as was observed with **DIC-TRZ**.

The related triazine-containing TADF host material **Trz-PhCz**, was reported by Sun *et al.*, containing a 3,6-di­phen­yl-9H-car­ba­zole donor linked at *meta* position on the TRZ phenylene linker (Figure 207).^1317^ **Trz-PhCz** (λ_PL_ = 470 nm, *E*_T_ = 2.85 eV, and Δ*E*_ST_ = 0.19 eV in 2-MeTHF) exhibits a short τ_d_ of 2.5 μs in neat film, which should help to support triplet harvesting and reduce efficiency roll-off in devices. This host was employed with **Ir(ppy)_2_(acac)**, **PO-01**, **Ir(dmppm)_2_(acac)**, and **Ir-F-DHBA** to fabricate green, yellow, orange, and red PhOLEDs, respectively. All the resultant devices showed extremely low efficiency roll-off, with EQEs of over 20% at 10,000 cd m^–2^. Notably, the orange device showed a record-high efficiency and low roll-off with EQE_max_ = 31.4%, and EQE_10,000_ = 25.5%.

Ito *et al*. reported a comparative study on the operational lifetime of PhOLEDs using one of four triazine-based TADF host materials with the phosphorescent green dopant **Ir(mppy)_3_** at 3 wt% loading: **DMAC-TA**, **2Cz-TA**, **DMAC-TRZ**, and **2Cz-TRZ** (Figure 207).^1318^ The LT_50_ of the devices were found to be 45, 180, 2500, and 13000 h for the different respective hosts, from an initial brightness of 1000 cd m^–2^. Such significant variations in the LT_50_ were correlated with the bond dissociation energies (BDE) of the C-N and C-C bonds in the host.

Jeon *et al*. studied a range of triazine-based hosts in combination with the sky-blue iridium complex **FIrpic** (*E*_T_ = 2.65 eV) to gauge the importance of the singlet and triplet energies of the host in relation to the triplet energy of the emitter.^1319^ TADF compounds **DCzTrz** (*E*_T_ = 2.64 eV) and **TrzmPCz** (*E*_T_ = 2.79 eV, Figure 207), and the fluorescent compound **DCzCNTrz** (*E*_T_ = 2.68 eV) were investigated along with the commercially available host mCP (*E*_T_ = 2.90 eV). The EQE_max_/EQE_1000_ values of the devices were found to be 15.6/15.3, 15.7/15.0, 16.3/14.1, and 2.8%/1.3% for the devices using mCP, **DCzTrz**, **TrzmPCz**, and **DCzCNTrz**, respectively, showing similarity in performance across all hosts except for **DCzDNTrz**. According to the authors, in the charge trapping process triplet excitons of FIrpic may decay directly to the ground state or transfer energy to triplet excitons of **DCzTrz** because of the high triplet energy of **FIrpic** (*E*_T_ = 2.65 eV) compared to that of **DCzTrz** (*E*_T_ = 2.64 eV). In addition, the smaller bandgap host **DCzTrz** performed the best in terms of lower driving voltage and higher current density of the device, with this outcome proposed to arise from its shallow HOMO and deep LUMO, which facilitates suitable hole and electron injection. In the **DCzTrz** host, emission arises exclusively from the phosphorescent dopant (based on the spectra) but with a fitted delayed emission component that is suggested to be associated with TADF. This indicates that TADF hosts can upconvert triplets to singlets and transfer energy to the near-isoenergetic guest by FRET, supporting the device performance.

Qian *et al*. reported the TADF host **PXZ-ICO** (Figure 207), consisting of a phenoxazine donor and an isocoumarin acceptor.^1320^ This host has a small Δ*E*_ST_ of 0.14 eV (λ_PL_ = 560 nm) in 2-MeTHF glass and a τ_d_ of 343 μs in 10 wt% doped films in DPEPO. The OLED with 3.5 wt% doping of red phosphorescent emitter **Ir(mphmq)_2_(tmd)** in **PXZ-ICO** host exhibited CIE coordinates of (0.62, 0.37) and showed an EQE_max_ of 18.6%. These device metrics surpass those of a similar OLED using the conventional host CBP (EQE_max_ = 15.3%).^1320^

Dendritic TADF molecules have also been explored as hosts, for example **TPPOCz** (Figure 207)^1004^ with sky-blue **FIrpic**, orange **Ir** (**CF_3_BT-CF_3_P)_2_(acac)**, and red **Ir(2-phq)_2_(acac**).^1321^ **TPPOCz** contains a second-generation carbazole donor dendron and a central phosphine oxide acceptor, and has a high *E*_T_ of 2.98 eV with Δ*E*_ST_ of 0.22 eV and λ_PL_ of 400 nm, all in neat film. Of the devices reported using 4 wt% doping of **FIrpic** as the emitter, the highest EQE_max_ was 20.4% and the maximum luminance was 13,235 cd m^–2^. The devices employing 3 wt% of either **Ir(CF_3_BT-CF_3_P)_2_(acac)** or **Ir(2-phq)_2_(acac**) showed EQE_max_ of 14.9 and 12.4%, respectively. This work is one of the rare examples of a solution-processed TADF dendrimer host for OLEDs, with dendrimer TADF materials explored further in Section 10.^1321^

Two spiro-based TADF hosts, **OSTFPB** (λ_PL_ = 495 nm, *E*_T_ = 2.59 eV, and Δ*E*_ST_ = 0.21 eV in toluene) and **OSTFPCN** (λ_PL_ = 460 nm, *E*_T_ = 2.68 eV, and Δ*E*_ST_ = 0.20 eV in toluene, Figure 207) were used by Wang *et al*. in red PhOLEDs with **Ir(MDQ)_2_(acac)**.^4^ Devices using 2 wt% of the dopant in **OSTFPB** or **OSTFPCN** as hosts showed very high EQE_max_ of 29.1 and 31.2%, and low efficiency roll-off at 100 cd m^–2^ of 0.3 and 2.6%, respectively.^4^

### 18.3 TADF Hosts with Fluorescent Emitters

In addition to PhOLEDs employing TADF materials as hosts, several groups have worked to produce efficient devices using the same strategy for fluorescent emitters. The hosts and emitters used in these devices are shown in Figure 208. For example, Zhang *et al*. used **DIC-TRZ** (*E*_T_ = 2.82 eV and Δ*E*_ST_ = 0.06 eV)^1322^ and **PIC-TRZ** (*E*_T_ = 2.70 eV and Δ*E*_ST_ = 0.11 eV; 6 wt% in mCP)^76^ as TADF host materials for 1 wt% **DDAF** in yellow OLEDs (Figure 208).^1268^ The device based on **DIC-TRZ:​DDAF** achieved an EQE_max_ of 12.2% and an EQE_1000_ of 5.5%, while the combination of **PIC-TRZ:​DDAF** resulted in EQE_max_ of just 4.7% and EQE_1000_ of 3.9%. This lower efficiency can be attributed to the larger Δ*E*_ST_ of **PIC-TRZ** in comparison to **DIC-TRZ**, leading to inefficient triplet harvesting from the host.

**Figure 208:**
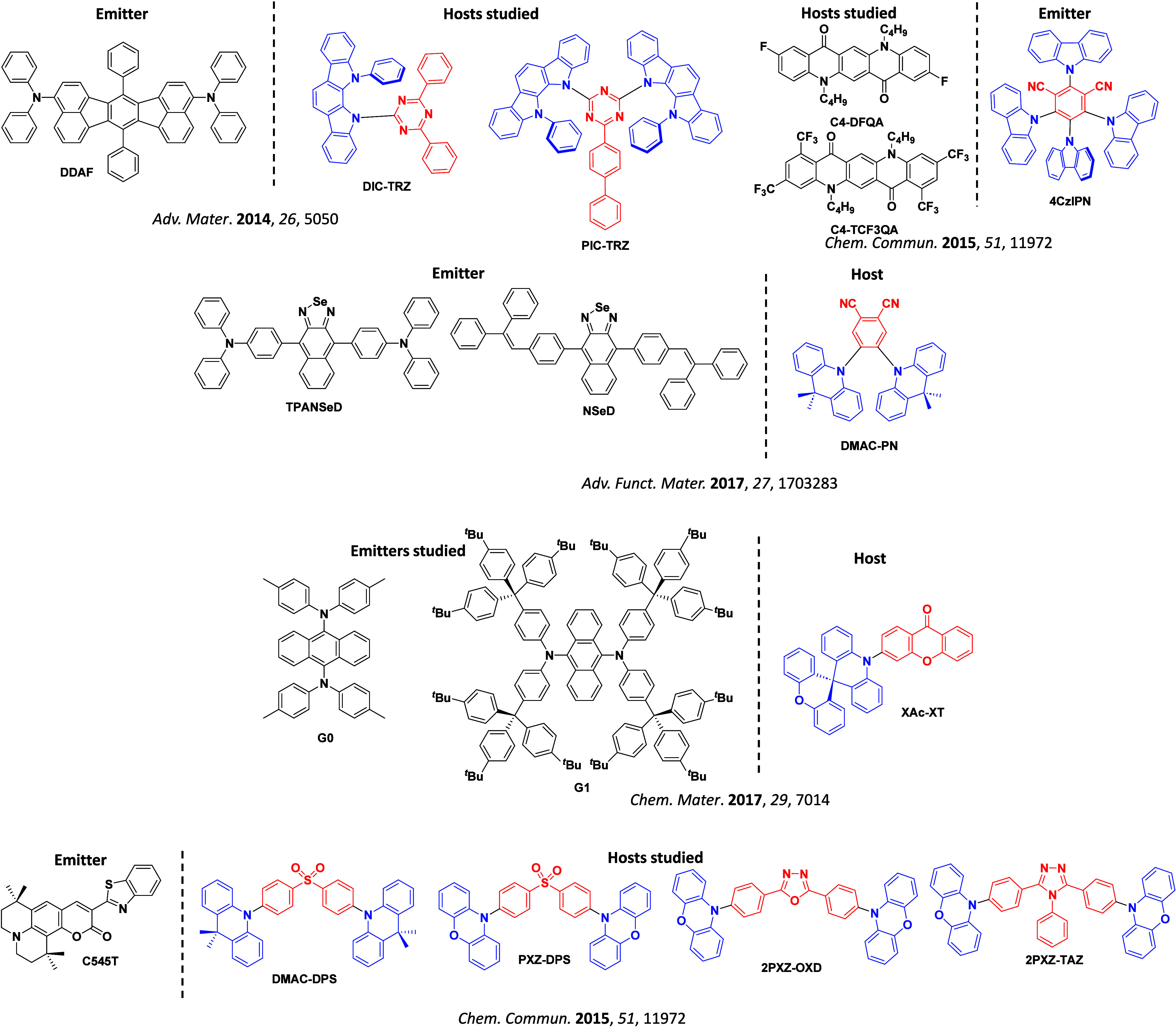
Chemical structures of the TADF hosts used with fluorescent emitters (the blue color signifies donor moieties, while the red color signifies acceptor moieties).

The orange TADF compound **DMAC-PN**, with λ_PL_ = 557 nm in 5 wt% doped CBP films and Δ*E*_ST_ = 0.27 eV in toluene,^1323^ was used as a host for two near infra-red (NIR) dyes containing naphthoselenadiazole moieties (**TPANSeD** and **NSeD**, Figure 208).^1324^ The devices with 4 wt% **TPANSeD** showed an EQE_max_ of 2.7% at λ_EL_ of 730 nm and an *L*_max_ of 10,569 cd m^–2^. Upon replacing the side group of 4-(diphenylamino)phenyl in **TPANSeD** with the bulkier 4-(2,2-diphenylvinyl)phenyl) in **NSeD**, non-radiative DET pathways were suppressed resulting in higher EQE_max_ of 3.8% at λ_EL_ 664 nm, and an *L*_max_ 16,956 cd m^–2^.^1324^

Wang *et al*. used **4CzIPN** as a host in combination with structurally similar quinacridone derivatives **C_4_-DFQA** and **C_4_-TCF_3_QA** (Figure 208) as yellow-green fluorescent dopants.^1325^ At 0.5 wt% loading the devices showed EQE_max_ of 13.5 and 14.6% respectively, and even at these very low doping concentrations only emission from the dopant was observed, indicating very efficient energy transfer between the host and guest. Excellent efficiency roll-off at 1000 and 5000 cd m^–2^ was noted for both emitters due to efficient RISC within the host and subsequent FRET from the host to the guest. The **4CzIPN:​C_4_-DFQA** and **4CzIPN:​C_4_-TCF_3_QA** devices showed EQE_1000_/EQE_5000_ of 12.6/11.0 and 13.7/12.3% respectively.^1325^

A dual TADF sensitizing strategy was used to transfer energy within OLEDs to fluorescent green emitter **C545T** through FRET.^1326^ Initial devices were fabricated using a series of TADF hosts (**DMAC-DPS**, **PXZ-DPS**, **2PXZ-OXD**, and **2PXZ-TAZ**, Figure 208); however, only **DMAC-DPS** and **PXZ-DPS** were selected for further investigation due to their superior performance in the preliminary studies. The FRET and DET energy transfer rates were measured with and without the auxiliary **PXZ-DPS** sensitizer to understand its effect in the energy transfer process, with the final compared devices composed of **DMAC-DPS:​PXZ-DPS** (30 wt%):​**C545T** (1.5 wt%), and **DMAC-DPS:​C545T** (1.5 wt%). Changes in the prompt and delayed emission components in thin films were measured to understand the FRET and DET rates. After introducing the second TADF host, the FRET rates increased from 9.26 × 10^7^ to 1.43 × 10^8^ s^–1^, while the authors quote DET rates to be on the order of 10^6^ s^–1^. The devices consequently showed an EQE_max_ of 11.1% in the dual host system, and only 9.0% in the absence of the second TADF host.

Aizawa *et al*. designed dendritic fluorescent emitter **G1** (Figure 208), with the aim of preventing Dexter energy transfer to the emitter from TADF hosts **XAc-XT**.^1327^ The solution-processed OLEDs with 1 mol% of reference emitter **G0** showed an EQE_max_ of only 3.2% (at 442 cd m^–2^), while with **G1** the device performance improved measurably to EQE_max_ = 5.2% (at 417 cd m^–2^).

### 18.4 Exciplex Hosts with Fluorescent Emitters

Exciplex-forming co-host systems with TADF properties have been used in conjunction with phosphorescent emitters to generate efficient and highly stable EL.^1328–1330^ Inspired by this, Liu *et al*. employed exciplex TADF host **TAPC:​DPTPCz** (Figure 209) in combination with fluorescent dopant **C545T**.^1331^ Devices were fabricated using a range of doping concentrations (0.2–1.0 wt%), and surprisingly the best efficiency was obtained with only 0.2 wt% of emitter. The devices achieved EQE_max_ and EQE_100_ of 14.5 and 12.0%, respectively. However, at such low doping concentrations the color purity of the device was low due incomplete energy transfer to the emitter, with residual exciplex emission observed in the EL. At the higher doping concentration of 1 wt% of **C545T**, only its emission was observed although the EQE_max_ and EQE_100_ were significantly lower at 7.5 and 5.3%, respectively.

**Figure 209:**
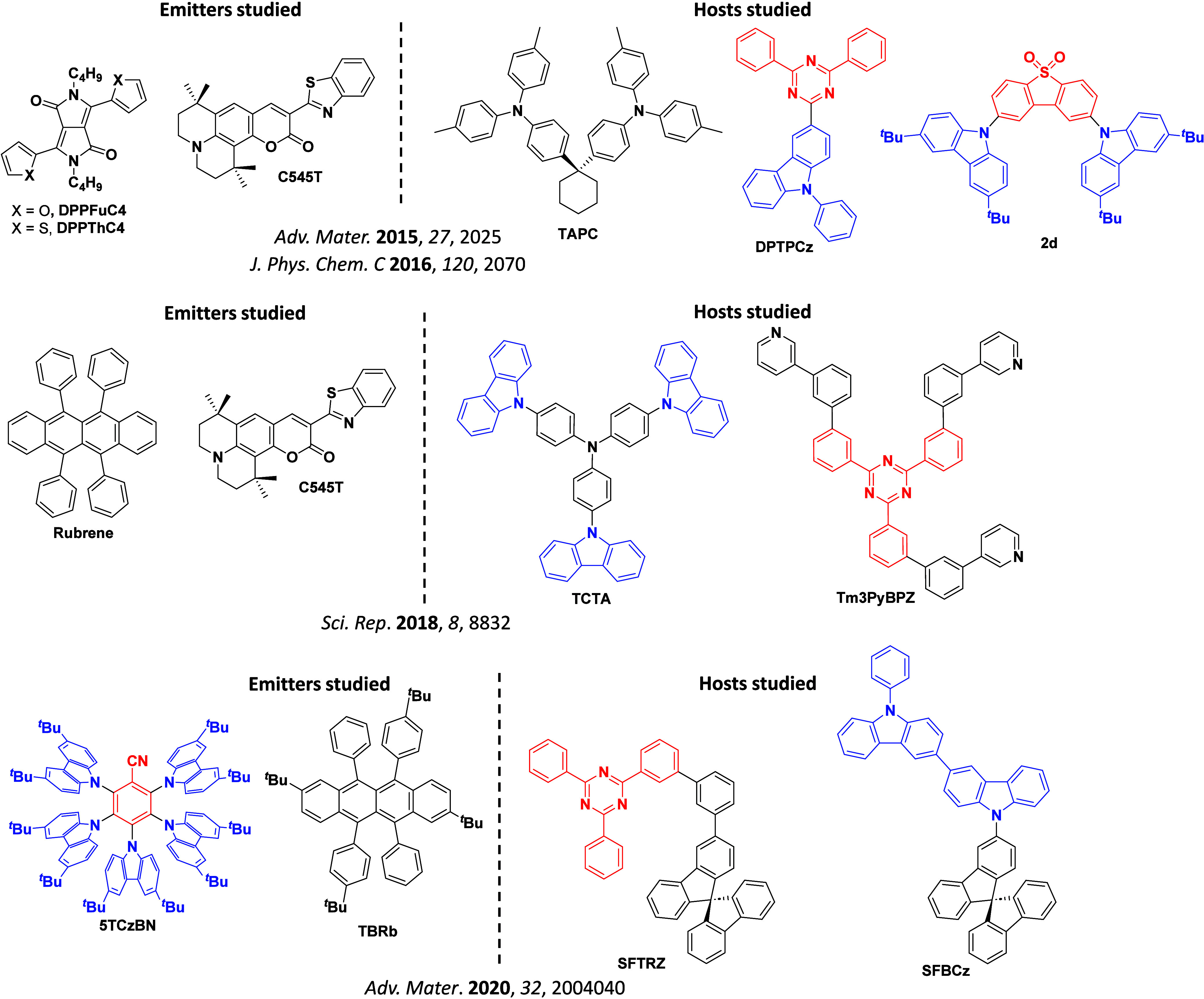
Chemical structures of the exciplex TADF hosts used with fluorescent emitters (the blue color signifies donor moieties, while the red color signifies acceptor moieties).

A multichannel exciplex-TADF host composed of **TAPC:2d** (7:3), was used alongside a series of diketodipyrolopyrole emitters (Figure 209).^1332^ In this case both the exciplex itself and the exciplex component **2d** are TADF active, giving multiple channels for RISC and triplet harvesting to occur. The most efficient green devices used **DPPFuC4** as the fluorescent dopant with an EQE_max_/EQE_100_ of 12.1/11.2%. The efficiency increased when the dopant was a thiophene analogue **(DPPThC4)**, perhaps due to more efficient triplet harvesting of the exciplex influenced by an external heavy atom effect from the sulfur of the thiophene, with an EQE_100_ of 11.1%, and a high *L*_max_ of 9983 cd m^–2^.

Zhang *et al*. also reported fluorescent devices using a TADF exciplex host.^1333^ In this case **TCTA:​Tm3PyBPZ** (λ_PL_ = 514 nm in 1:1 film, Figure 209) was used to transfer energy to the green and red emitters **C545t** and **rubrene**. The green devices with 1 wt% of **C545t** showed *L*_max_, EQE_max_, and EQE_1000_ of 20,640 cd m^–2^, 10.4%, and 7.9%, respectively. The red **rubrene** devices showed comparable values of 22,170 cd m^–2^, 10.0%, and 8.4%. All the devices exhibited higher efficiencies than previous reports which used the non-doped TADF exciplex as the emitter (1:1 ratio), which had *L*_max_, EQE_max_, and EQE_1000_ of 12,800 cd m^–2^, 13.1%, and 8.8%, respectively.^1334^

A pair of π-D and π-A exciplex forming materials were designed for WOLED purposes, envisioned to operate by partial energy transfer from the co-host to both a blue TADF sensitizer (**5TCzBN**) and a yellow fluorescent emitter (**TBRb**, Figure 209).^566^ This reported **SFTRZ:​SFBCz** exciplex system also incorporates a bulky bipolar π-group as a spacer, which achieves two objectives: i) an increased separation distance between the D and A subunits of the π–D and π–A molecules, resulting in a blue-shifted emission; and ii) the retention of the superior charge transporting ability characteristic of exciplex systems. When **SFTRZ:​SFBCz** was used in combination with 20 wt% **5TCzBN** (λ_EL_ = 485 nm), 0.2 wt% **TBRb** (λ_EL_ = 552 nm), and 0.05 wt% red fluorescent dopant, **RD** (RD = 1,3,7,9-tetra­kis­(4-(*tert*-but­yl)­phen­yl)-5,5-di­fluoro-10-(2-meth­oxy­phen­yl)-5H-4λ4,5λ4-di­pyr­rolo­[1,2-c:2′,1′-f]­[1,3,2]di­aza­bor­inine) (λ_EL_ = 614 nm), the best performing device achieved EQE_max_, EQE_1000_, and lifetime (LT_80_ at initial 5000 cd m^–2^) of 16.7, 16.5, and 203 h, respectively, at warm white CIE coordinates of (0.439, 0.452).

### 18.5 TADF Hosts with TADF Emitters

The aforementioned examples demonstrate the value of TADF hosts used in combination with phosphorescent and fluorescent emitters. Similar device performance improvements can also be achieved in OLEDs that employ TADF hosts for other TADF emitters (Figure 210). In a study by Duan and co-workers, the TADF host **DMIC-TRZ** was used in combination with TADF emitters **5TCzBN** (blue, λ_EL_ = 486 nm), **DMAC-BP** (green, λ_EL_ = 513 nm) and **4TCzTPN** (orange, λ_EL_ ∼548 nm) to produce efficient devices across the visible spectrum.^1335^ The devices showed EQE_max_ of 19.2, 21.0, and 23.2% respectively, and the efficiency roll-offs at 2,000 cd m^–2^ were excellent, ranging from 3–7%. Reference devices produced with conventional hosts CBP and 26DCzPPy showed reduced performance and increased efficiency roll-off compared to those with the triplet-harvesting TADF hosts.

**Figure 210:**
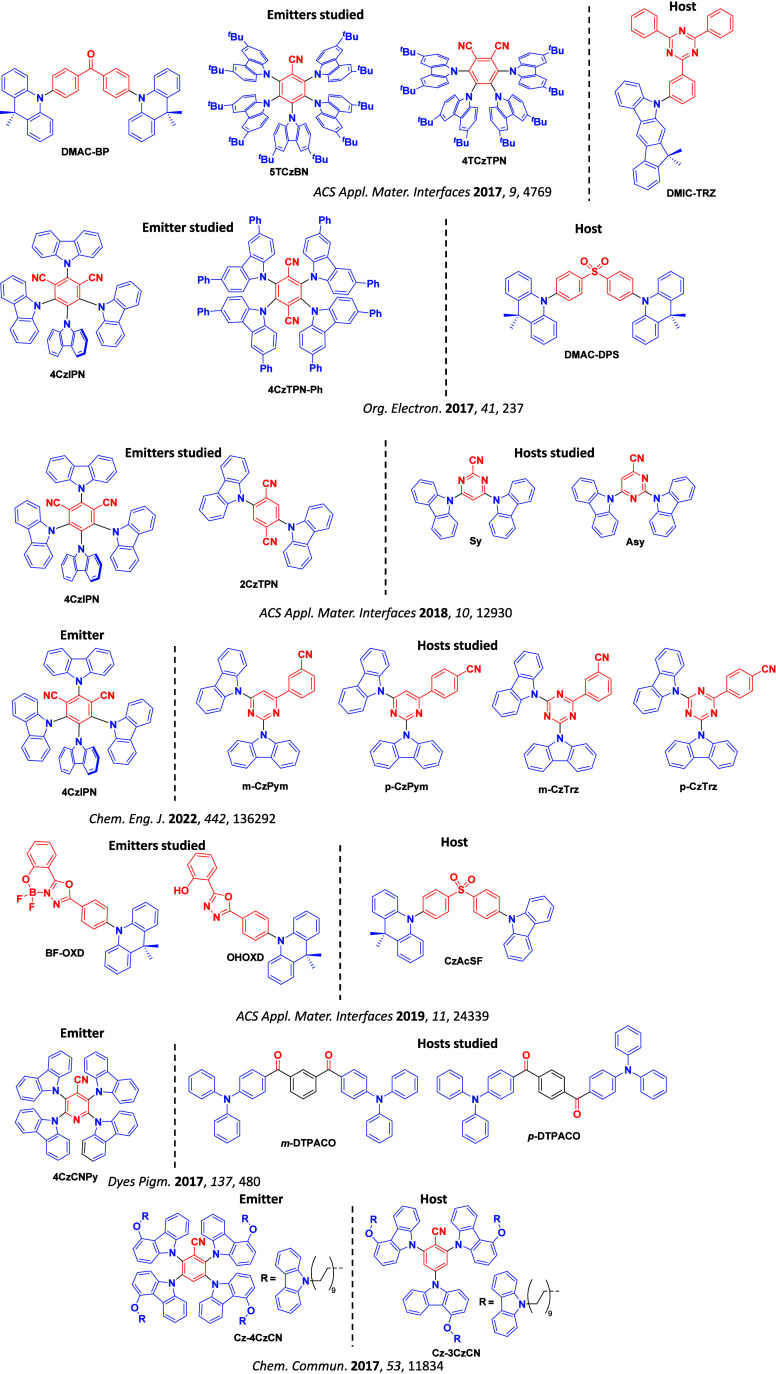
Chemical structures of the TADF hosts used with other TADF emitters (the blue color signifies donor moieties, while the red color signifies acceptor moieties).

Using **DMAC-DPS** as a host, Liu *et al*. fabricated a series of green and orange devices with **4CzIPN** or **4CzTPN-Ph** as the TADF emitters (Figure 210).^585^ The best device with **4CzIPN** was achieved using 6 wt% doping, showing an EQE_max_ of 10.9% and an EQE_1000_ of 9.1%. By contrast, the best device with **4CzTPN-Ph** required a higher doping concentration of 9 wt% and showed an EQE_max_ of 11.0% and an EQE_1000_ of 10.1%. By exploiting partial energy transfer from the host to the guest, white-emitting devices were also fabricated, the best of which using 0.2 wt% **4CzTPN-Ph** in **DMAC-DPS** showed an EQE_max_ of 14.7% and EQE_1000_ of 12.0% with CIE coordinates of (0.25, 0.37).

Symmetric and asymmetric hosts **Sy** and **Asy** (Figure 210) were reported by Li *et al*. incorporating carbazole and cyanopyrimidine.^1336^ These were used along with **2CzTPN** (blue)^1337^ and **4CzIPN** (green) emitters.^31^ Both the blue and green devices using the **Sy** host (λ_PL_ = 471 nm, *E*_T_ = 3.06 eV, and Δ*E*_ST_ = 69 meV in toluene) performed better than using **Asy** (λ_PL_ = 518 nm, *E*_T_ = 2.92 eV, Δ*E*_ST_ = 114 meV in toluene). In the device with 10 wt% **2CzTPN** in **Sy** the *L*_max_, EQE_max_, and EQE_1000_ were 122,100 cd m^–2^, 20.4, and 16.9%, while the corresponding values for the **4CzIPN:Sy** device were 221,500 cd m^–2^, 24.0, and 22.1%. Factors such as its smaller Δ*E*_ST_ and more efficient energy transfer to the emitters may explain the improved performance of the **Sy** devices. Notably, the TDMs of both emitters were preferentially horizontally aligned (88% in both hosts), contributing to the high efficiencies.

Chen *et al*. reported TADF host materials **m-CzPym**, **p-CzPym**, **m-CzTrz**, and **p-CzTrz** (Figure 210). Comprised of carbazole as the donor and benzonitrile-substituted heteroarenes (triazine or pyrimidine) as the acceptor, these had measured Δ*E*_ST_ in toluene of 0.44, 0.46, 0.31, and 0.40 eV respectively.^1338^ High-performance green TADF OLEDs were fabricated using these hosts in combination with **4CzIPN** as the emitter.^31^ Amongst all the devices, **m-CzPym** was found to be the best host with the device showing an EQE_max_ of 31.5%, PE_max_ of 116.5 lm W^–1^, a turn-on voltage of 2.5 V, and low efficiency roll-off (EQE_1000_ = 29.0%). The high EQE_max_ was linked to the outstanding light outcoupling efficiency of over 31–35%, as verified through angle-dependent PL intensity measurement.

Zhou *et al*. reported two new TADF emitters that contain a DMAC donor and oxadiazole acceptor either with or without a chelated BF_2_ group. **OHOXD** (Figure 210) has λ_PL_ = 473 nm, Δ*E*_ST_ = 0.16 eV, and τ_d_ = 1.9 μs in toluene with Φ_PL_ = 30% in 10 wt% doped **CzAcSF** films, while boron-chelated **BFOXD** has identical λ_PL_ = 473 nm, smaller Δ*E*_ST_ = 0.09 eV, yet slower τ_d_= 4.3 μs, in toluene, with much larger Φ_PL_ = 66% in the same **CzAcSF** host.^1339^ Solution-processed devices with **OHOXD** showed *L*_max_, EQE_max_, and EQE_1000_ of 1520 cd m^–2^, 12.1, and 4.3%, which rose considerably for **BFOXD** at 4518 cdm^–2^, 20.1, and 12.7%.^1339^

Hu *et al*. reported two isomeric phthaloyl/triphenylamine TADF materials as hosts for solution-processed devices.^1340^ ***m*-DTPACO** and ***p*-DTPACO** (Figure 210) consist of triphenylamine as end-capping electron-donating groups and isophthaloyl or terephthaloyl as the central electron-withdrawing moieties. ***m*-DTPACO** (λ_PL_ = 477 nm as the neat film) and ***p*-DTPACO** (λ_PL_ = 522 nm as the neat film) have Δ*E*_ST_ of 0.21 and 0.05 eV, and t_d_ of 8.29 and 9.60 μs with Φ_PL_ of 75 and 39%, respectively. Non-doped solution-processed devices with ***m*-DTPACO** and ***p*-DTPACO** as emitters exhibited *L*_max_ of 10,005 and 7354 cd m^–2^, and EQE_max_ of 2.4 and 3.7% respectively. Their potential as host materials was then investigated by doping green TADF emitter **4CzCNPy** ^1341^ at 10 wt%.^1340^ The emission spectrum of ***m*-DTPACO** showed better overlap with the absorption spectrum of **4CzCNPy**, allowing more efficient energy transfer from the host to the guest. This is reflected in the solution-processed device performance, with high *L*_max_ of 22,322 cd m^–2^ and EQE_max_/EQE_1000_ of 13.0/10.3% for the device using ***m*-DTPACO**. By contrast, the device performance was lower using ***p*-DTPACO** with *L*_max_ and EQE_max_/EQE_1000_ values of 15,510 cd m^–2^ and 9.0/5.6%, respectively.

Lastly, Ban *et al*. employed encapsulated TADF materials as both host (**Cz-3CzCN**, λ_PL_ = 445 nm as the neat film) and guest (**Cz-4CzCN**, λ_PL_ = 475 nm as the neat film, Figure 210) in solution-processed devices.^1342^ Alkyl chains connected to a peripheral carbazole donor were used to insulate the emissive **3CzCN** and **4CzCN** cores. **Cz-3CzCN** and **Cz-4CzCN** have promising Δ*E*_ST_ values of 0.24 and 0.22 eV and Φ_PL_ of 25 and 78%, respectively, in toluene. Solution-processed green devices with 4 wt% **Cz-4CzCN** in **Cz-3CzCN** showed an EQE_max_ of 23.5%; however, the efficiency roll-off was significant, with EQE_100_/EQE_1000_ of 15.5 and 7.8%, respectively. Reference devices were also made with conventional non-encapsulated TADF host (**3CzBN**) and guest (**4CzBN**) for comparison. These non-encapsulated host-guest devices showed greatly reduced EQE_max_, EQE_100_, and EQE_1000_ values of only 5.9, 3.1 and 1.9%, respectively, demonstrating the effectiveness of the encapsulation strategy for improving device performance.

### 18.6 Outlook

This section details examples of TADF-active molecules or exciplex blends acting as promising host materials for both vacuum-deposited and solution-processed OLEDs. The intrinsically electron-donating and electron-accepting chemical groups associated with the charge-transfer excited states of these TADF materials provide balanced charge transport as well as RISC pathways for triplet harvesting for separate fluorescent, phosphorescent, or TADF guest emitters. This application of TADF materials can therefore support improvements in device lifetime and efficiency, with strong conceptual overlap to hyperfluorescence (Section 17), AIE emitters (Section 13), and both exciplex and through-space charge transfer emitters (Sections 8 and 12). Emblematic of the examples of this concept summarized here, we re-highlight the use of **m-CzPym** as a host for the emitter **4CzIPN**.^1338^ While **4CzIPN** doped into standard hosts such as CBP can achieve EQE_max_ of ∼20%, with the added support of the TADF active **m-CzPym** host the the device with the same emitter can have an EQE_max_ that exceeds 30% and also shows remarkably low efficiency roll-off.

Given the promise of this approach, it seems evident that significant future performance gains across a wide range of OLED technologies will likely be enabled by a better understanding and the application of TADF materials not just as emitters, but also as hosts. While this is already demonstrated for various green and red terminal emitters, we note that this concept is yet to be fully realised for blue emission, which would nominally require very high triplet energy hosts (>3.0 eV). Rather than an intrinsic limitation though, we anticipate that the as-yet undiscovered host materials required to replace DPEPO and related phosphine oxide compounds that are the most commonly used hosts in supporting deep-blue and UV TADF OLEDs will likely arise from this area of research, with D-A TADF emitters (and likely poor emitters) finding successful repurposing as hosts that directly contribute to triplet management.

## 19 Supramolecular Assemblies of TADF Materials

### 19.1 Introduction

Supramolecular chemistry is now widely recognised as a powerful and fascinating strategy to bestow molecules with new structural features and properties outside the scope of covalent bonding.^1343–1347^ This provides an additional dimension of materials design compared to the combinatorial strategies associated with D-A TADF emitters (Sections 3–5), and the still evolving understanding of MR-TADF compounds (Section 11). Naturally, researchers have applied supramolecular strategies to TADF emitters with the aim to significantly alter their photophysical properties, in some cases leading to emergent properties unseen in their discrete counterparts. We recently reviewed this area in detail.^56^ It is worth noting that despite the wide range of supramolecular structures shown to exhibit TADF, the intersection of these research fields is still relatively young and so there are at present still few examples from each class of supramolecular system.

Here, we firstly discuss a TADF core comprised of carbazole and benzophenone as an illustrative example that has been incorporated in three different supramolecular systems, each showing vastly different properties and functionalities. We then divide and summarise other notable examples of TADF supramolecular assemblies into two categories: architectures that involve co-ordination to metal centres, and non-co-ordinating systems operating via aggregation and/or encapsulation.

### 19.2 CzBP – One Core in Three Systems

Three distinct supramolecular structures have been formed using the same parent TADF emitter, **CzBP**: gels, metallocages, and rotaxanes. Each structure possesses different photophysical properties, highlighting the potential of supramolecular chemistry to modulate to properties of other TADF emitters when integrated into distinct assemblies.

The first examples of TADF gels were formed by appending 4-pyridyl groups to the carbazole moiety to give **4PyCzBP** and mixing this compound with diacids (Figure 211).^1348^ **4PyCzBP** itself shows blue emission in both degassed DCM (λ_PL_ = 477 nm, Φ_PL_ = 52%) and in 10 wt% PMMA-doped films (λ_PL_ = 449 nm, Φ_PL_ = 21%). Mixing **4PyCzBP** with one equivalent of succinic acid gave a yellow/green gel with enhanced emission at λ_PL_ = 500 nm, with the pyridine moieties hydrogen bonding with the diacid hydrogen atoms, though the gel was only weakly bound with a critical gel concentration (CGC) of 5 mg mL^–1^. A stronger gel formed when using (L)-tartaric acid due to the greater number of hydrogen bonds that could be formed, with a red-shifted emission at λ_PL_ = 510 nm and a CGC of 3 mg mL^–1^. Compared to isolated **4PyCzBP**, there was an 11-fold enhancement in the emission when using 0.5 equivalents of the (L)-tartaric acid and a 60-fold enhancement when using 1 equivalent of the same. However, using a greater excess of diacid resulted in a decrease of the emission intensity due to disruption of the intramolecular hydrogen bonding within the gel structure. The TADF nature of the 1:1 **4PyCzBP:**​(L)-tartaric acid was confirmed by transient photoluminescence measurements, which showed biexponential decay kinetics with τ_PL_ = 20 ns and 2.3 μs. The Φ_PL_ of the xerogel is six times higher than the neat film (Φ_PL_ = 36% vs. 6%).

**Figure 211:**
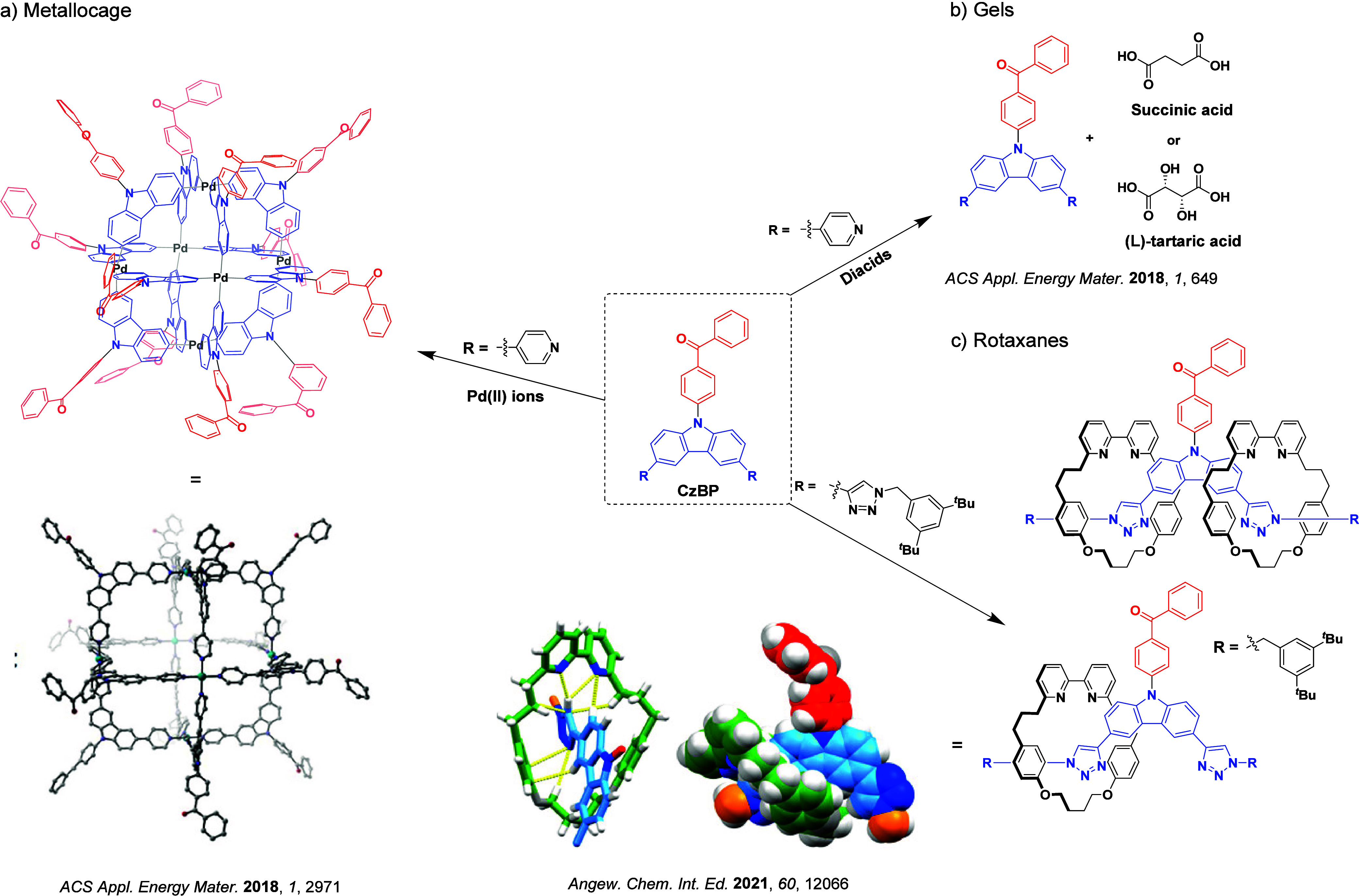
Derivatives of **CzBP** incorporated into different supramolecular assemblies: a) A Pd(II) M_6_L_12_ metallocage. b) Combined with diacids to form gels. c) Assemblies with either one or two macrocyclic rings to give rotaxanes (the blue color signifies donor moieties, while the red color signifies acceptor moieties). Taken and adapted with permission form ref 1348. Copyright [2018/ACS Applied Energy Materials], American Chemical Society.

The same **4PyCzBP** was separately employed as a ligand in conjunction with Pd^2+^ to give the M_6_L_12_ metallocage, **4PyCzBP-Pd** (Figure 211).^1349^ This metallocage geometry formed from the combination of the square planar palladium centres along with the angle between co-ordinating pyridines of **4PyCzBP** being 93.5°, which has previously been shown by Fujita and co-workers to facilitate M_6_L_12_ metallocages.^1350^ The resulting cuboctahedron was calculated to have an internal volume of 6400 Å^3^, showed a significant reduction in Φ_PL_ and an accompanying red-shift (**PyCzBP-Pd**; λ_PL_ = 555 nm, Φ_PL_ = 4% in DCM) compared to the free emitter in degassed DCM (**4PyCzBP**; λ_PL_ = 477 nm, Φ_PL_ = 52%). No delayed emission was observed for **4PyCzBP-Pd**, with only prompt biexponential lifetimes of τ_PL_ = 3 ns and 30 ns. This significant change in optical properties was rationalised by DFT calculations, showing that while the HOMO was still distributed over the carbazole moiety, the LUMO became localized at the palladium(II) centres rather than the benzophenone acceptor. The ligand-to-metal charge transfer into antibonding *d*-orbitals was therefore identified as the likely source of emission quenching. We note that the dynamic nature of supramolecular structure association often allows them to support reversible stimulus-responsive behaviour. The demonstrated ability of these assemblies to then modulate emissive and TADF properties therefore provides an attractive pathway to optical readout of such stimulus responses.

The cavity of the **PyCzBP-Pd** metallocage was also used to host two emissive xanthene dyes: fluorescein and Rose Bengal. Electrospray ionization mass spectrometry (ESI-MS) revealed that up to three molecules of neutral fluorescein can be held within **4PyCzBP-Pd**. This host-guest complex also engaged in photoinduced electron transfer (PET) from host to guest, giving **[F]^.+^[4PyCzBP**]^−^ and completely quenching the emission of both species. ESI-MS analysis of the Rose Bengal complex showed that two molecules of the open dianion quinoid form of Rose Bengal are held within **4PyCzBP-Pd**. This host-guest complex similarly engaged in photoinduced energy transfer (PEnT) in DMSO solution, where the green emission of the host was quenched along with emergence of orange Rose Bengal emission. Förster energy transfer was proposed as the proposed PEnT mechanism, quantified by a quenching rate constant of *k*_q_ = 4.07 x 10^11^ M^–1^ s^–1^.

Incorporation of triazole groups to extend the carbazole of the CzBP core was employed to produce mechanically interlocked macrocycle rotaxanes **TzCzBP**⊂**R_1_** and **TzCzBP**⊂**R_2_** (Figure 211).^1351^

Through-space interactions between the triazole protons and the macrocyclic bipyridine nitrogen lone pairs led to an increase in the Φ_PL_ under N_2_, a decrease in Δ*E*_ST_, and increased photostability under UV irradiation in toluene (**TrCzBP**; Φ_PL_ = 11%, **TzCzBP**⊂**R_1_**; Φ_PL_ = 31%, **TzCzBP**⊂**R_2_**; Φ_PL_ = 30%). DFT calculations revealed that the LUMO of the rotaxanes remains on the benzophenone moiety, whereas the carbazole-based HOMO is destabilised due to the aforementioned hydrogen bonding. This example demonstrates the ability of supramolecular approaches to finely modulate the photophysical properties of the emitter via rotaxane formation.

Of the remaining examples of supramolecular TADF materials reviewed here, we may broadly categorize these into those which are co-ordinated or covalently bound to give a supramolecular assembly, and assemblies formed non-covalently through aggregation or through-space interactions.

### 19.3 Coordinatively Bound Supramolecular TADF Assemblies

As seen for **4PyCzBP-Pd**, TADF emitters with moieties capable of co-ordination may form supramolecular assemblies templated by metal ion vertices. Metallocages are not the only assembled structures that can form between co-ordinating emitters and metal centres though; indeed, other TADF emissive supramolecular systems exist, including one Zr(IV) and two Zn(II) metal organic frameworks (MOFs), a platinum(II) metallocycle, and a cobalt-containing dendritic photocatalyst.

#### 19.3.1 MOFs

The first TADF MOF was reported by Adachi and co-workers using Zr(IV) centres and an organic diacid linker. This linker, **A**, was chosen due to its small calculated Δ*E*_ST_ of 0.2 eV (measured 0.24 eV) (Figure 212).^1350^ A red-shift of the emission and a decrease in the delayed lifetime of MOF **Zr-A-MOF** in the solid state (λ_PL_ = 501 nm, τ_PF_ = 17 ns, τ_DF_ = 180 μs, Φ_PL_ = 30% (N_2_), 18% (air)) was observed compared to the emission of the free linker **A** in 2 wt% doped PMMA films [λ_PL_ = 481 nm, τ_PF_ = 18 ns, τ_DF_ = 199 ms, Φ_PL_ = 39% (N_2_), 32% (air); **Zr-A-MOF** in the solid state]. The decrease of the MOF Φ_PL_ in air was ascribed to more active quenching of the triplet excited state by oxygen. The decrease in lifetime and red-shift of emission in **Zr-A-MOF** compared to **A** was attributed to co-ordination to the electron-poor Zr(IV) centres, acting similarly to an auxiliary acceptor in D-A TADF materials.

**Figure 212:**
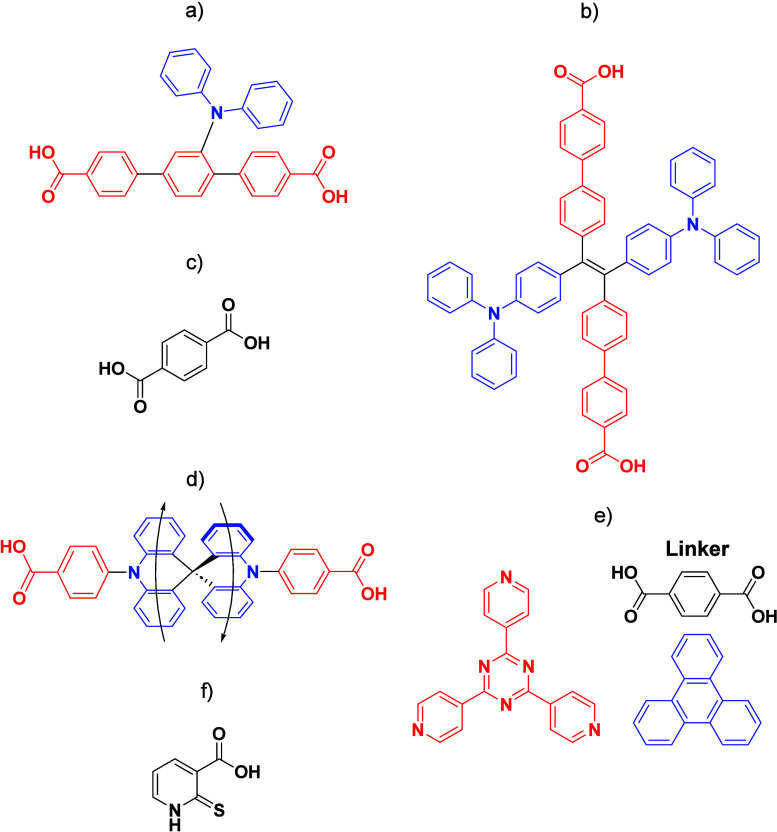
The structures of the organic components commonly used in TADF MOFs. Where relevant, electron donors have been colored blue, with electron acceptors in red.

Haldar *et al*. formed a TADF MOF between a diphenylaminoteraphenylethylene linker, **B**, and Zn(II) ions, **Zn-B-MOF** (Figure 212).^1352^ In dilute ethanol solution, linker **B** is poorly emissive due to non-radiative decay via free rotation of the phenyl groups (τ_PL_ = 1.5 ns). This process is supressed in powder samples of **B**, (τ_PL_ ≈ 200 μs). Crystalline and orientated thin-films of **Zn-B-MOF** showed the same delayed lifetime of ≈ 200 μs, with a Φ_PL_ of 14%. The TADF nature of the emission was confirmed using variable temperature photoluminescence studies. This thin-film MOF system was furthermore incorporated into an OLED, although this displayed a high turn-on voltage of 5.8 eV and a low maximum luminance of 270 cd m^–2^ at 14 V. Using time-dependant DFT, the authors inferred that the electroluminescence actually originated from a hot-exciton mechanism, made possible by the small energy gap between the T_2_ and S_1_ states compared to the relatively large energy gap for coupling between T_1_ and T_2_.

Small changes in the structure of a system may have substantial effect on the mode of emission, as demonstrated by Gutiérrez *et al*. (Figure 212).^1353^ MOFs formed from Pb(II) and terephthalic acid **C** were synthesised to study what effect crystallizing the MOF from either water, (**MOF-C-H_2_O)**, or DMF (**MOF-C-DMF**) would have on their optoelectronic properties. Green photoluminescence centred at ∼525 nm, was observed for **MOF-C-H_2_O**, with an associated Φ_PL_ of 59% for the powder. For **MOF-C-DMF**, the emission blue-shifts to 480 nm, with a Φ_PL_ of 32% in the solid state. The authors hypothesized that differences in the crystal packing were the source of the difference in the photophysical properties, with XRD analysis of **MOF-C-H_2_O** revealing a more densely packed structure. At 77 and 298 K **MOF-C-H_2_O** showed similar multiexponential emission decay kinetics, with lifetimes of *τ*_PL_ of 39 ns, 145 μs, and 1.53 ms. The lifetime for **MOF-C-DMF** was instead temperature-dependant, with *τ*_PL_ of 43 ns, 0.29 μs, and 15 μs at 298 K; however, at 77 K only the nanosecond lifetime component was observed. The authors proposed that the more densely packed **MOF-C-H_2_O** features many inter-linker interactions, leading to relatively temperature-insensitive RTP dominating the emission. In the less densely packed **MOF-C-DMF**, larger inter-linker distances begin to favour TADF, as evidenced by the temperature-dependant nature of emission.

Bie *et al*. synthesised an organic diacid linker, 4,4′′-(10*H*,10′′*H*-9,9′′-spiro­bi[acri­dine]-10,10′′-di­yl)di­ben­zoic acid (**D**) and coordinated this with zirconium clusters to give **MOF-D**, which displayed oxygen-insensitive TADF (Figure 212).^1354^ The design of **D** is an A-D-σ-D-A structure. The rigid backbone prevents rotation and increases the rigidity of the system, with the two electronically isolated A-D units separated by a quaternary sp^3^-carbon in the centre and additionally giving a twist to the compound.^1355^ The linker **D** has an emission centred at around 453 nm, and is a promising TADF material in its own right with a Δ*E*_ST_ value of 0.02 eV and a τ_d_ of 1.18 μs in degassed THF, with this long-lived emission disappearing upon exposure to air. A slight increase in the ΔE_ST_ was noted, from 0.02 eV to 0.14 eV, along with a red-shift to 490 nm in the emission of **MOF-D** compared to the free linker. The rigid design was chosen to counteract the decrease in emission lifetime observed by Adachi and co-workers for their emitter **A** upon incorporation into a MOF,^1356^ which the authors of that work believed to be driven by the flexibility of the linker within the MOF. Indeed, for **MOF-D** the τ_d_ is 0.72 μs in the powder, only modestly different compared to 0.23 μs for **D** in the solid state. Indeed, the lengthening of the emission lifetime upon complexation is likely due to the increased rigidity of the linker, resulting in a suppression of the non-radiative pathways.

Liu *et al*. reported the encapsulation of an electron-rich triphenylene donor within a Cd(II) MOF (NKU-11) containing electron-poor triazine panels, giving rise to exciplex-like TADF between the panels and the guest (Figure 212).^1357^ The host⊂guest MOF system, herein called **MOF-E**, was formed via the solvothermal synthesis of Cd(II) ions, triazine, triphenylene, and terephthalic acid. The triphenylene sits within triangular prism cages in the MOF, formed of two triazine ligands and three terephthalic acid linkers. **MOF-E** shows a broad, featureless emission centred around 492 nm, pointing to emission originating from a charge-transfer state. Temperature-dependant photoluminescence studies showed a 20-fold increase in the emission intensity upon heating from 77 K to 297 K, supporting a TADF mechanism. **MOF-E** displayed a triexponential excited-state lifetime with τ_PL_ of 17.5 ns, 1.29 μs, and 4.21 μs indicating the presence of both prompt and delayed emission, and with a Δ*E*_ST_ of 0.11 eV.

A silver cluster-containing MOF was formed upon co-ordination of 2-mercaptonic acid, **F**, with Ag(I) ions to give hexameric silver clusters, which may organise into a MOF upon complexing with Ca^2+^ ions, **MOF-F**.^1358^ The discrete silver nanoclusters possessed poor Φ_PL_ of ∼2%, but upon complexation to the calcium ions a 10-fold increase in the Φ_PL_ to ∼20% was seen. **MOF-F** proved to be pH-sensitive; protonation occurs on the carbonate co-ordinating moieties on the silver clusters at low pH thus breaking **MOF-F** apart, which can then reform under basic conditions. **MOF-F** showed green emission centred at around 590 nm in the thin film with *τ_PL_* of 557 ns, 8.61 μs. The intensity of the delayed emission was found to be temperature-dependent, confirming the TADF behaviour of this system.

### 19.4 Metallocycles

A TADF platinum (II) metallocycle with coordinating organic ligand, **BTZPy**, was reported by Lv *et al.*, showing promise as a photodynamic therapy and chemotherapy drug (Figure 213).^1359^ Compound **BTZPy** by itself showed efficient fluorescence with Φ_PL_ = 78%, τ_PL_ = 8.65 ns and λ_PL_ = 569 nm in degassed ethanol solution. Co-ordination of **BTZPy** to Pt(II) centres, **cPt**, afforded the triangular metallocycle **BTZPy-Pt**, which also showed a high Φ_PL_ but with a blue-shifted emission (Φ_PL_ = 60%, λ_PL_ = 550 nm, and an average τ_PL_ = 8.65 ns). Nanosecond transient absorption spectroscopy revealed the lifetimes of **G** and **PtG** to be 1.87 μs and 1.76 μs, respectively (λ_exc_ = 532 nm), with temperature-dependant emission studies of **G** and **PtG** uncovering a higher delayed emission intensity with increasing temperature, again suggesting the metallocycle is TADF-active. Both **G** and **PtG** displayed excellent singlet oxygen generating ability in ethanol solution [measured relative to a *meso*-tetrakis(*p*-sulfonato-phenyl) standard], with quantum yields of singlet oxygen generation of 95% and 86%, respectively.

**Figure 213:**
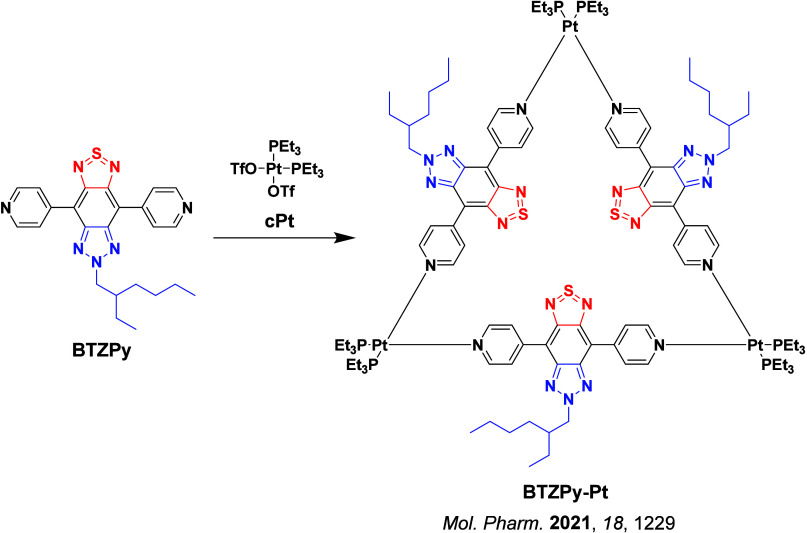
Incorporation of **BTZPy** into a TADF platinum(II) metallocycle for use as a dual chemo- and photodynamic therapy drug (the blue color signifies donor moieties, while the red color signifies acceptor moieties).

### 19.5 Cobalt-Containing Dendrimeric Antenna Complex

Combining a typical D-A TADF core **4CzPN** with terminal pyridine groups (in this context **H)**, and binding to cobaloxime centres gave the photocatalytic assembly **H-Co**. This material was used to promote the catalytic acceptorless dehydrogenation (CAD) of secondary amines (Figure 214).^1360^ DFT calculations of **H** revealed the HOMO is centred on the carbazole moieties and the LUMO is localised on the phthalonitrile core as expected from the D-A structure, with a ΔE_ST_ of 0.13 eV. Compound **H** emits at λ_PL_ = 591 nm with CT emission profile, and has a τ_d_ of 17.4 μs and a Φ_PL_ of 7% in degassed CH_2_Cl_2_ at 298 K. Upon complexation to the cobaloxime groups, a decrease in both the lifetime and photoluminescence quantum yield is observed (τ_PL_ = 13.8 μs, Φ_PL_ = 2.9%), arising from PET from **H** to the cobalt centres that supports its catalytic activity. The ability of **H-Co** to generate hydrogen using blue LEDs (450 nm ± 10 nm, 3W) was demonstrated with a 0.04% catalyst loading of **H-Co** in dry degassed THF and gave a turnover number (TON) of 305 after 12 h. An uncomplexed mixture of cobaloxime and **H** under the same conditions gave a TON of only 53, with the increase in performance upon complexation attributed to more efficient absorption and electron transfer between adjacent subcomponents of the well-defined bound structure.

**Figure 214:**
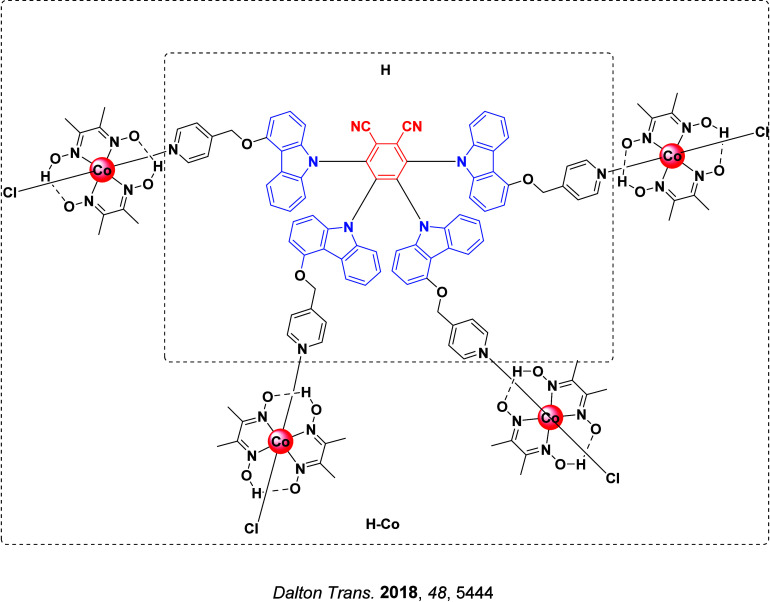
**H** co-ordinating to cobalt(II) centres to give supramolecular photocatalyst **H-Co**. The carbazole electron donors have been colored blue, while the dicyanobenzene acceptor has been colored red. Structure taken and adapted with permission from ref 1360. Copyright [2019/Dalton Transactions] Royal Society of Chemistry.

### 19.6 Non-coordinatively Bound Supramolecular TADF Systems

As well as covalent or coordinate bonding interactions, supramolecular assemblies may form through non-bonding interactions such as aggregation or encapsulation. Both approaches have the potential to significantly modulate the photophysical properties of the photoactive TADF emitters compared to isolated molecules.

### 19.7 Aggregation-Based TADF Assemblies

There are a small number of reported examples of organic and carbon dots TADF emitters. These are small micelle-like spherical particles that form in poor solvents such as water and are then decorated with water-solubilising side chains. Common organic emitters used for dot preparation include derivatives of **4CzIPN** and structurally similar emitters,^1361–1364^ as well as materials using phenoxazine and phenothiazine,^1365,1366^ anthraquinone,^1367^ and benzophenone^1368^ (Figure 215). Polyethylene glycol (PEG) chains are commonly used as water-solubilising groups and may be covalently linked to the organic emitter or mixed with the particles in solution to self-assemble into a particle coating that prevents further particle aggregation and sedimentation. Such assemblies are not only water soluble, but their excited states are sufficiently long-lived to outcompete biological autofluorescence. As the emitters are shielded within micelle or hydrophilic coating, the presence of oxygen in these biological systems does not necessarily contribute to the quenching of the excited state. In cases where oxygen does diffuse into the micelle, emission intensity can even be used as an optical probe for cellular oxygen concentration.^1369^ One such micellar system was reported by Zhu *et al*. who employed peptide chains as the water-solubilising groups, allowing the material to pass through cellular and nuclear membranes and further demonstrating their versatility and suitability as biological probes.^1370^

**Figure 215:**
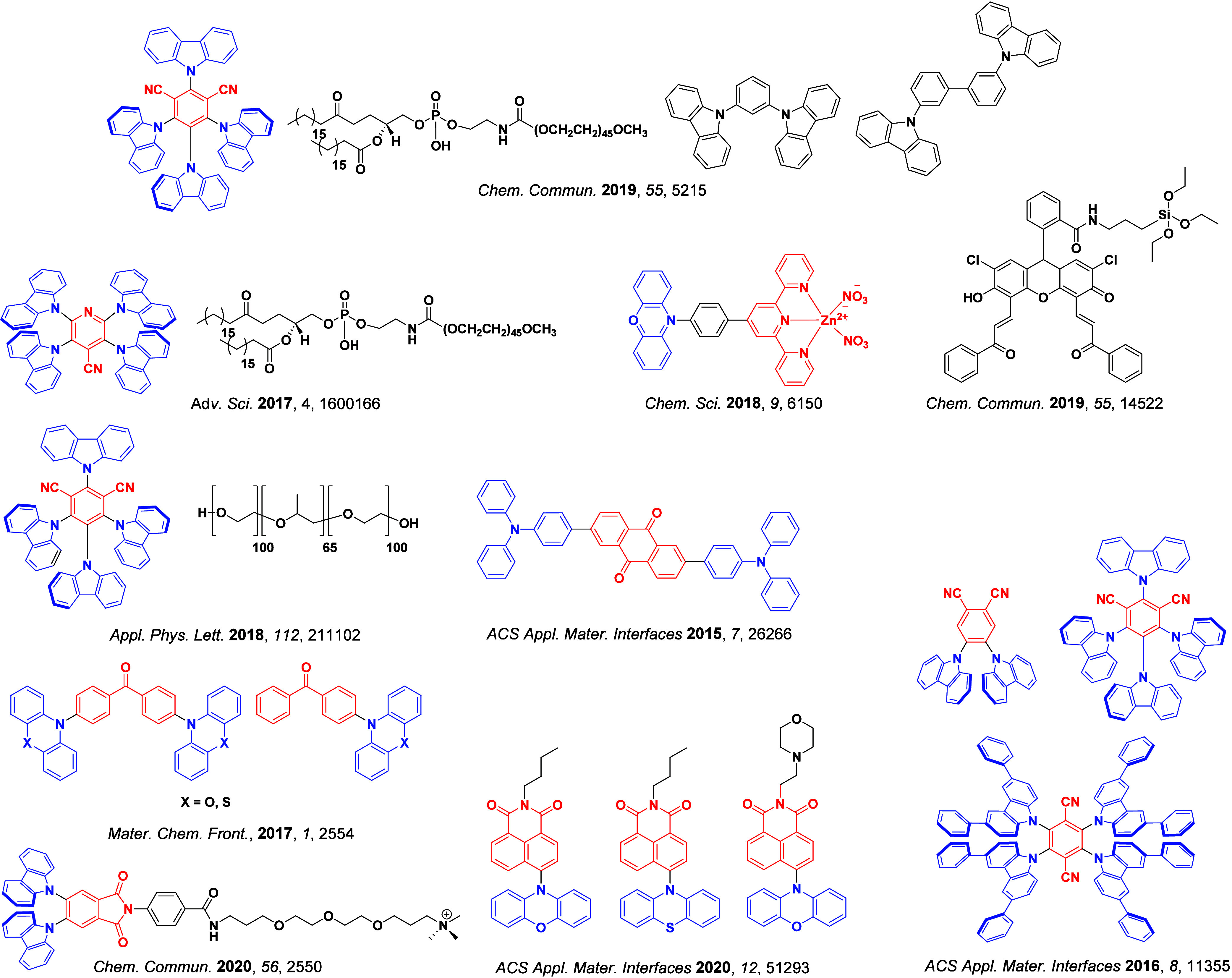
Examples of compounds used in the construction of TADF organic/carbon dots (the blue color signifies donor moieties, while the red color signifies acceptor moieties).^56^

Another example of a supramolecular TADF system formed via aggregation was reported by Qi *et al.*, where the emitter, **CDPA**, formed nanorod needles in thin neat films that were several hundred nanometres thick and several hundred micrometres long (Figure 216).^1371^ The emission of these nanorod needles in the thin film (λ_PL_ of 645 nm) is slightly red-shifted relative to the powder (λ_PL_ of 640 nm), and the needles showed an enhanced Φ_PL_ of 26% compared to 13% for the unassembled thin film. Transient photoluminescence decay measurements of the thin film show prompt (2.3 ns) and delayed (10.0 μs) emission, the latter of which showed a temperature dependence.^1372^

**Figure 216:**
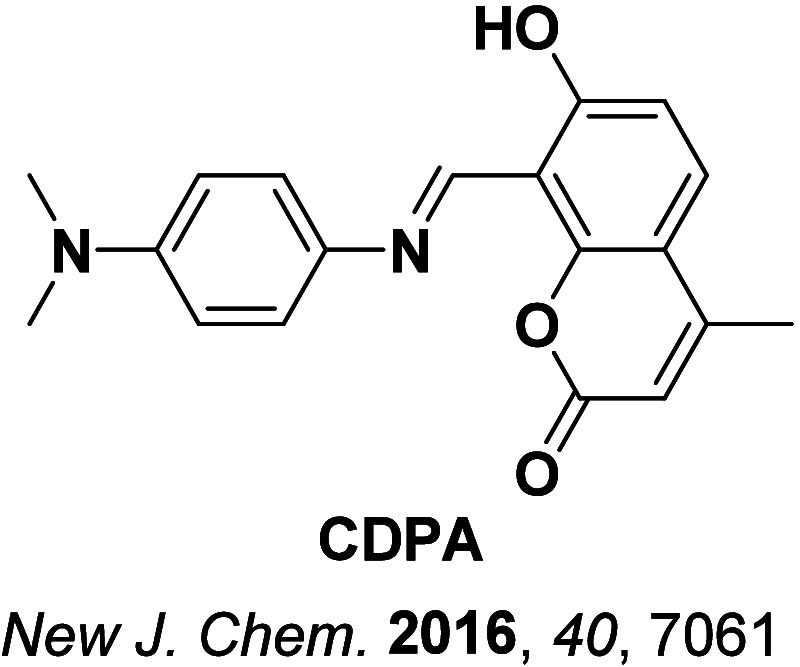
Structure of emitter **CDPA**, which forms TADF nanorod needles in the neat films.

### 19.8 TADF from Zeolite-Encapsulated Emitters

Other non-covalently bound supramolecular TADF emitter systems can be formed by the encapsulation of carbon dots into a zeolite host. Multiple reports from Yu, Li, and co-workers have demonstrated this self-assembly approach, forming a zeolite host and carbon dot in a one-pot reaction, leading to trapped dots within the zeolitic framework. This encapsulation leads to millisecond excited state lifetimes and high photoluminescence quantum yields of the materials, assisted by shielding from external atmospheric oxygen and restricting internal conformational and vibrational degrees of freedom of the carbon dots, thus suppressing non-radiative decay.

The authors first reported three dots-in-zeolite systems, **CD1**, **CD2**, and **CD3**, formed under solvothermal conditions.^1373^ System **CD1** used triethylamine, aluminum tri-*iso*-propoxide, phosphoric acid, triethylene glycol, and hydrofluoric acid to form dots trapped within a zeolite matrix. System **CD2** was formed under similar conditions, but with 4,7,10-trioxa-1-13-tridecanediamine in place of triethylamine, and no hydrofluoric acid. System **CD3** had a similar preparation to **CD2**, but with the addition of dimagnesium phosphate. Upon excitation at 370 nm all three systems displayed deep-blue emission [(**C1**: λ_PL_ = 430 nm, Φ_PL_ = 15%, CIE (0.17, 0.13); **C2**: λ_PL_ = 440 nm, Φ_PL_ = 52%, CIE (0.17, 0.14); **C3**: λ_PL_ = 425 nm, Φ_PL_ = 23%, CIE (0.17, 0.13)]. Additionally, all three systems showed delayed emission, with τ_d_ = 350 ms, 197 ms, and 216 ms the characteristic temperature dependence associated with TADF for **CD1**, **CD2**, **and CD3**, respectively. The measured Δ*E*_ST_ values for these systems are 0.22 eV, 0.23 eV, and 0.22 eV, respectively.

The authors then reported another dot-in-zeolite system showing TADF using 4,7,10-tri­oxa-1-13-tri­de­cane­amine as a template, **CD4**.^1374^ Excitation at 370 nm led to emission at λ_PL_ = 440 nm with Φ_PL_ = 29%, and excitation wavelength-dependent emission intensity (excitation at 350 nm led to the brightest emission). Temperature-dependent transient photoluminescence decay measurements showed an increase in the delayed emission intensity with increasing temperature. Combined with the delayed emission (τ_d_ = 153 ms) and the measured Δ*E*_ST_ of 0.18 eV, these data confirmed TADF as the emission mechanism. By contrast, the unconfined carbon dot in the mother liquor shows no delayed emission at room temperature, and a slightly larger Δ*E*_ST_ of 0.21 eV. The authors suggested that encapsulation of the carbon dot within the zeolite led to the suppression of nonradiative decay along with a stabilisation of the triplet state, switching on TADF.

Yu, Li, and co-workers have also reported four dots-in-zeolites systems, **CD5–8**, whereby two dots are encapsulated within the same zeolite framework in varying ratios.^1375^ The ratio of two carbon dot templates, *m*-phen­ylene­di­amine and 4,7,10,-tri­oxa-1-13-tri­de­cane­di­amine, was varied from 0:1, 0.007:1, 0.0014:1, and 0.042:1 in the starting mixtures for self-assembly to give four systems; **CD5**, **CD6**, **CD7**, and **CD8**. As the ratio of *m*-phen­ylene­di­amine to 4,7,10-tri­oxa-1-13-tri­de­cane­di­amine increased, the emission red-shifted (**CD5–8**; λ_PL_ = 425 nm, 484 nm, 498 nm, 515 nm, respectively) while the delayed lifetime increased and the prompt lifetime decreased (**CD5**: τ_PL_ = 24.43 ns, 271 ms, **CD6**: τ_PL_ = 37.54 ns, 578 ms, **CD7**: τ_PL_ = 11.76 ns, 801 ms, **CD8**: τ_PL_ = 7.70 ns, 860 ms). The photoluminescence quantum yields also increased with increasing *m*-phen­ylene­di­amine inclusion (**CD5–8**: Φ_PL_ = 20.9%, 25.1%, 37.1%, 42.0%, respectively). Temperature-dependant time-resolved emission studies of the dots-in-zeolites systems confirmed TADF, with ΔE_ST_ for **CD5** and **CD8** measured between 0.20 and 0.14 eV. The authors propose that FRET from the 4,7,10-trioxa-1-13-tridecanediamine dots to the *m*-phen­ylene­di­amine dots within the confined matrix occurs, with the *m*-phen­ylene­di­amine dots emitting via TADF.

In a similar manner, Koninti *et al*. demonstrated TADF behavior from benzophenone once encapsulated within mesoporous silica nanostructures (MSN).^1376^ Benzophenone is known to phosphoresce at 77 K, but once encapsulated the non-radiative pathways are supressed and Δ*E*_ST_ is reduced from 0.11 eV (in MeCN) to between 0.048 eV and 0.060 eV, depending on the MSN used. The PL spectrum of free benzophenone in aerated MeCN shows weak fluorescence at around 450 nm, owing to rather efficient ISC followed by quenching of the T_1_ state by oxygen, as well as non-radiative deactivation by free rotation of the phenyl rings. Upon adding MSN to the MeCN solution, the emission intensity increases due to the effects of encapsulation of benzophenone: reducing the Δ*E*_ST_, increasing the RISC rate to give emission from the S_1_, and suppressing non-radiative decay via molecular rotations/vibrations due to increased environmental rigidity. The emission intensity is further increased in degassed solution, further supporting the expectation of triplet involvement in the emission. It should be noted that the presence of MSN being linked to the increase of the emission intensity suggests some shielding from oxygen upon encapsulation within the MSN framework. A τ_d_ of between 22 and 44 μs was observed, depending on the MSN used, with Φ_PL_ of 1.7% in all systems compared with a Φ_PL_ of 0.2% for benzophenone in MeCN; the τ_d_ in each of the MSN-based systems was 5 μs.

### 19.9 Co-crystallized Host⊂Guest Donor-Acceptor TADF

A TADF host⊂guest system of α-cyclodextrin (**α-CD**) and diphenylacetylene (**DPA**) was reported by Huang *et al.*, which exhibited TADF through ‘long-range charge transportation’ giving ‘long persistent luminescence’ (Figure 217).^1377^ Three related systems were synthesised, using unsubstituted **DPA** (**DPA-1**), 4-(4-fluorophenylethynyl)phenol (**DPA-2**), and 4-4′-difluorodiphenylacetylene (**DPA-3**). For each **DPA** derivative a 1:1 aqueous solution with **α-CD** was prepared and crystals grown via slow evaporation, while an alternate method of forming the host⊂guest complexes was also explored involving grinding the host and guest with a small amount of water. Using either method, host⊂guest complexes **α-CD-DPA-n** (n = 1, 2, or 3) were formed, each showing dual TADF and RTP with an afterglow of more than 2 seconds. The complexes showed two oxygen-sensitive emission maxima at 360 nm and 460 nm, with the 360 nm peak (associated with the TADF) having very long τ_d_ of 134 ms, 282 ms, and 256 ms, for **α -CD-DPA-1**, **2**, and **3**, respectively. The emission at 460 nm results from phosphorescence and exhibits similarly long lifetimes of 354 ms, 292 ms, and 245 ms for **α -CD-DPA-1**, **2**, and **3**, respectively, with associated Φ_PL_ values of 47.4%, 33.5%, and 29%. The authors ascribed the TADF to originate from the **α-CD** host, while the phosphorescence is from the **DPA** guest, giving the observed dual emission.

**Figure 217:**
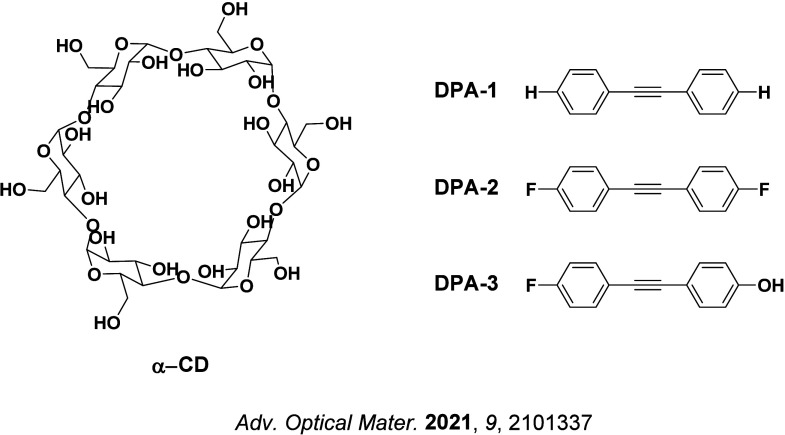
Structures of **α-CD**, **DPA-1**, **DPA-2**, and **DPA-3**, showing long-range charge transfer upon formation of TADF host⊂guest complexes.

Recently the co-crystallisation of a calix[3]-acridian ring (**C[3]A**) with dicyanobenzene (**DCB**) was shown to produce a host⊂guest complex exhibiting TADF (Figure 218).^1378^ Dissolving equimolar **C[3]A** and **DCB** in *n*-hexane/CHCl_3_ gave green crystals upon evaporation, with bright green/blue photoluminescence under UV light. X-ray crystallography showed multiple C-H···π interactions between host and guest, producing a tightly bound **C[3]A**⊂**DCB** complex. The crystals of **C[3]A**⊂**DCB** crystals showed an absorption maximum at around 400 nm and an emission at 500 nm, which is significantly red-shifted compared to **C[3]A** (λ_PL_ = 385 nm). Transient photoluminescence measurements revealed biexponential decay kinetics, with a τ_p_ of 152 ns and a τ_d_ of 5.2 μs, where the delayed emission demonstrated the expected temperature dependence associated with TADF. The Φ_PL_ = 70% was notably large, which the authors attributed to the rigidity of the crystal structure inhibiting non-radiative decay processes. The Δ*E*_ST_ was measured to be 0.014 eV, which translated into a *k*_RISC_ of 9.42 x 10^4^ s^–1^.

**Figure 218:**
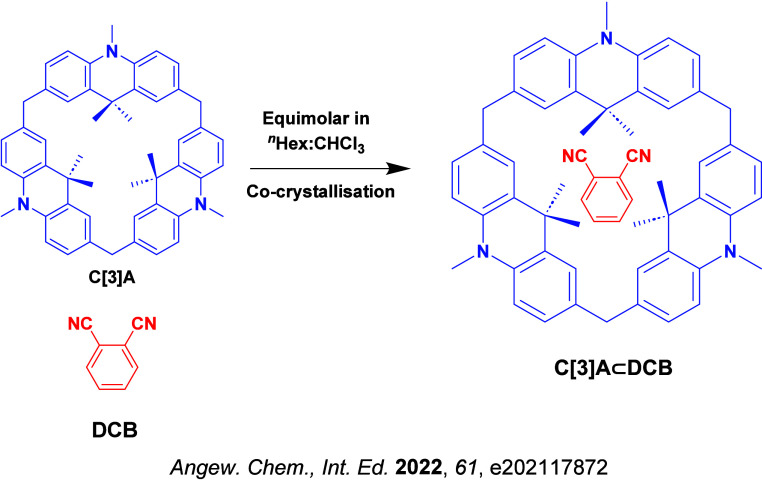
Structures of donor host **C[3]A** (blue) and acceptor guest **DCB** (red), which can co-crystallise to give a TADF host⊂guest complex (the blue color signifies donor moieties, while the red color signifies acceptor moieties).^1378^

### 19.10 Outlook

Incorporating a TADF unit within a supramolecular assembly can have an impact on the photophysical properties that are as diverse as the different supramolecular structures themselves. Given the small but growing number of examples to date, it is difficult to project what functional properties each of these classes of assemblies may ultimately unlock.

The **CzBP** core, which featured in gels, rotaxanes, and a metallocage, provides insight into the wide range of supramolecular assemblies discrete TADF emitters may be incorporated into. The gel formed from **4PyCzBP** allowed for the production of xerogel films with higher photoluminescence quantum yields than their neat film counterparts, while incorporation into a rotaxane gave fine control over the emission wavelength from the **CzBP** core and though the metallocage constructed from **4PyCzBP** and Pd^2+^ ions was poorly emissive, nonetheless it could act as a photoactive host where either photoinduced energy or electron transfer could occur depending on the nature of the encapsulated substrate. These emergent properties are as diverse as the supramolecular structures which give rise to them and demonstrate the vast potential this field may hold for the many TADF emitters that already exist. What is required at present is an increased effort to explore this field. Considerable work will be needed to correlate the properties of discrete emitters and their surpramolecular counterparts to identify trends and emergent properties. What is certain is that this is an area ripe for further exploration and innovation.

## 20 TADF Sensors

### 20.1 Introduction

As a consequence of the underpinning photophysics, TADF emission is acutely sensitive towards both temperature and oxygen, which has been exploited in sensing applications.^1379^ Indeed, establishing and calibrating correlations between emission properties (spectrum, intensity, or lifetime) and temperature is the foundation of optical molecular thermometry.^1380^ For example, the temperature-dependent nature of RISC and thus the ratio of delayed fluorescence to phosphorescence of a TADF emitter can be exploited for optical readout of temperature. Similarly the quenching of triplet excitons of TADF materials by oxygen and the associated drop in emission intensity can be harnessed for use as an oxygen sensor.^1381,1382^ Oxygen sensing is highly relevant in medicine, where the oxygen level in exhaled air, patient blood, or even within cells is a key physiological parameter that sometimes requires continuous monitoring. Similarly, the measurement of the oxygen concentration is important in industries that use metabolizing organisms, such as yeast for brewing and baking,^1383^ and in biotechnology, where microorganisms are used to produce antibiotics and anticancer drugs.^1384^

### 20.2 Materials Development

Firster *et al*. demonstrated the first TADF-based temperature sensor in 1995, using **acridine yellow** embedded within a saccharide host matrix for optical thermometry (Figure 219).^1380^ One of the major advantages of employing TADF materials as thermosensors is that the delayed emission lifetimes follow an Arrhenius behavior, in contrast to the more complex models required in other fluorescence-based sensors.^1380,1385^ As well as from delayed emission lifetimes, temperature can also be inferred from the relative emission intensities of delayed fluorescence and phosphorescence. For **acridine yellow** the average temperature sensitivities of the delayed fluorescence lifetime and of the delayed fluorescence-to-phosphorescence intensity ratio were 2.5 and 4.5% °C^–1^, respectively (confirmed across −50 to 50 °C). According to the authors, these sensitivities were ∼10 times higher than typical optical thermometer materials available at that time. Beyond this temperature range the error increases as material stability deteriorates at higher temperatures.

**Figure 219:**
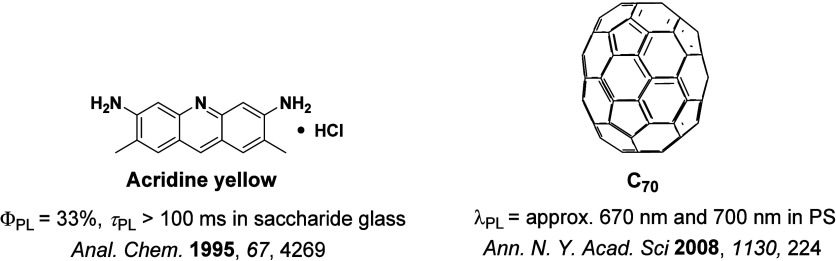
Structures of acridine yellow and C_70_ used in a TADF-based temperature sensor.

**C_70_** doped in PtBMA polymer acts as a temperature sensor with an expanded temperature range, evaluated from the ratio of intensities between delayed fluorescence and phosphorescence.^1386^ The working device possessed sensitivity of 0.5% K^–1^ across a temperature range of −80 °C to 140 °C. The device could also undergo numerous heating/cooling cycles, showing less than 2% change in readout over several weeks. In this system degassing is also essential given that the triplet excitons undergoing RISC are oxygen sensitive. By taking advantage of this oxygen sensitivity, oxygen sensors were also developed using ^13^ **C_70_**, showing detection limits down to parts per billion (ppb).^1382,1387^ This was the first example of an oxygen sensor with an optical readout. Reversibility and reusability of the sensor film was emphasized along with good stability over many months.

DeRosa *et al*. reported biological oxygen sensing using TADF- and RT-active derivatives of difluoro­bor­on-*β*-di­ke­ton­ate-poly­(lactic acid) (**BF_2_bdk-PLA**) and difluoro­bor­on­di­ben­zoyl­meth­ane-poly­(lactic acid) [**BF_2_dbm (X)PLA** (X = H, Br, I)] (Figure 220).^1388^ The non-halogenated **BF_2_dnmPLA** is a highly efficient TADF emitter under N_2_ atmosphere at room temperature, while the halogenated polymers **BF_2_dnm(Br)PLA** and **BF_2_dnm(I)PLA** are phosphorescent due the higher SOC resulting from the presence of these heavy atoms. The polymer **BF_2_dnm(I)PLA** enabled ratiometric oxygen-sensing and imaging due to its distinguishable dual-emission in fluorescence (green) and phosphorescence channels (orange), depicted in Figure 220. Further, **BF_2_dnm(I)PLA** nanoparticles were used to detect differences in intracellular oxygen concentrations demonstrated for *in vitro* ratiometric imaging of T41 mouse mammary cells.^1388^

**Figure 220:**
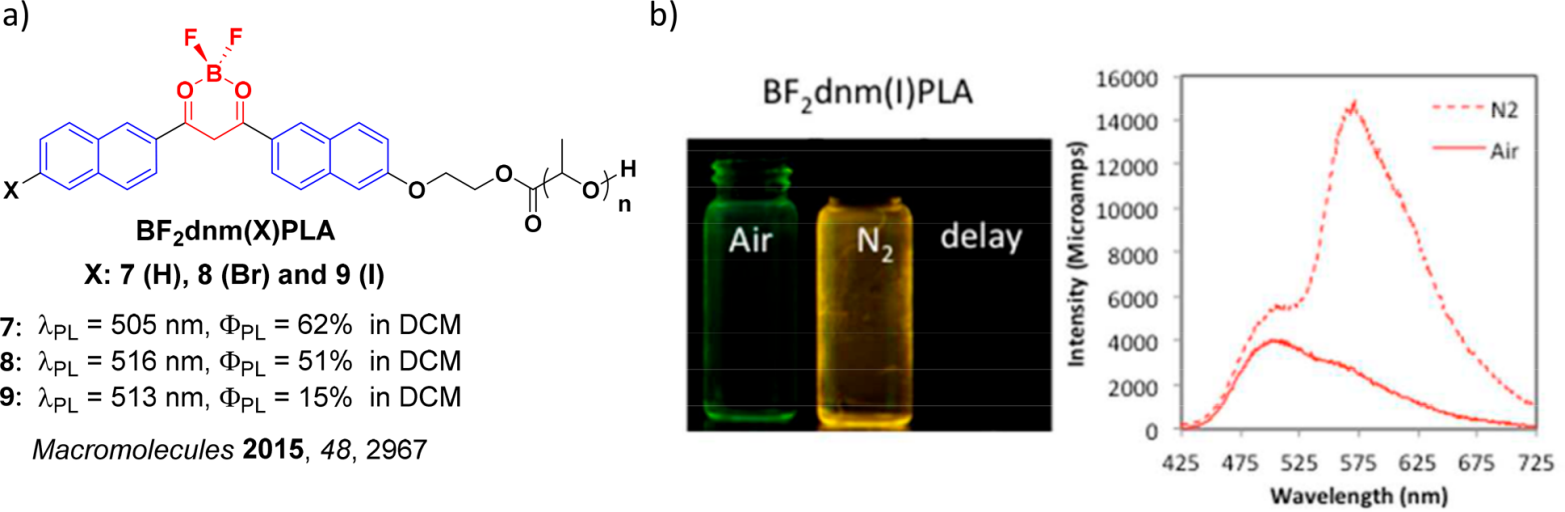
a) Structures of TADF compounds designed as biological oxygen sensors reported in ref 1388. b) Images and total emission spectra of **BF_2_dnm(I)PLA** in air and N_2_. Photographs were taken with UV lamp excitation (λ_exc_ = 354 nm); delayed emission images were captured after the UV lamp was turned off. Taken and adapted with permission from ref 1388. Copyright [2015/Macromolecules] American Chemical Society.

Steinegger *et al*. reported a series of carbazole-substituted dicyanobenzenes (**b, c, d1**, and **d2**) and diphenylamine-substituted anthraquinones (**a1**-**a7** and **e**) for use as oxygen and temperature sensors (Figure 221).^1389^ In toluene the dicyanobenzene-based emitters emit strongly between 506 and 546 nm and have Φ_PL_ of between 61 and 79%, while the series of anthraquinone-based emitters emit weakly between 609 and 678 nm (Φ_PL_ of between 0.1 and 15%). The τ_d_ of the dicyanobenzene-based emitters varies from 5 to 15 μs while the τ_d_ of the anthraquinone-based emitters varies over a much wider range (τ_PL_ of between 11 and 583 μs). For the preparation of oxygen-sensitive materials, 1 wt% dyes were immobilized in oxygen-permeable polystyrene (PS). The emission bands of these emitters in PS shift hypsochromically to between 577 and 614 nm for the anthraquinone-based emitters and to between 493 and 531 nm for the dicyanobenzene-based emitters, coupled with increases in their respective *τ*_d_ (42 μs to 5.5 ms for anthraquinones and 9 μs to 40 μs for dicyanobenzenes) and in their Φ_PL_ (26 to 48% for anthraquinones and 59 to 96% for dicyanobenzenes). Oxygen sensitivity was calibrated using Stern-Volmer (SV) quenching analysis. The oxygen sensitivity of these materials varied from moderate to very high and was proportional to the τ_d_ of the compound. For temperature sensing these emitters were incorporated into gas-impermeable poly­(vin­yl­idene chlor­ide-*co*-acrylo­nitrile) [P(VDC-*co*-AN)] and temperature was calibrated against corresponding change in *τ*_d_. These temperature sensors demonstrated sensitivities from −1.4 to −3.7% K^–1^, determined from the change of *τ*_d_ per unit change in temperature. Further, the authors also prepared a fiber-optic mini sensor by using **d2** as the temperature reporting emitter incorporated in P(VDC-*co*-AN), enabling rapid and high-resolution temperature monitoring. Additionally, the authors made temperature-sensitive nanoparticles based on **c** and **d2** for use in temperature imaging at the cellular level.

**Figure 221:**
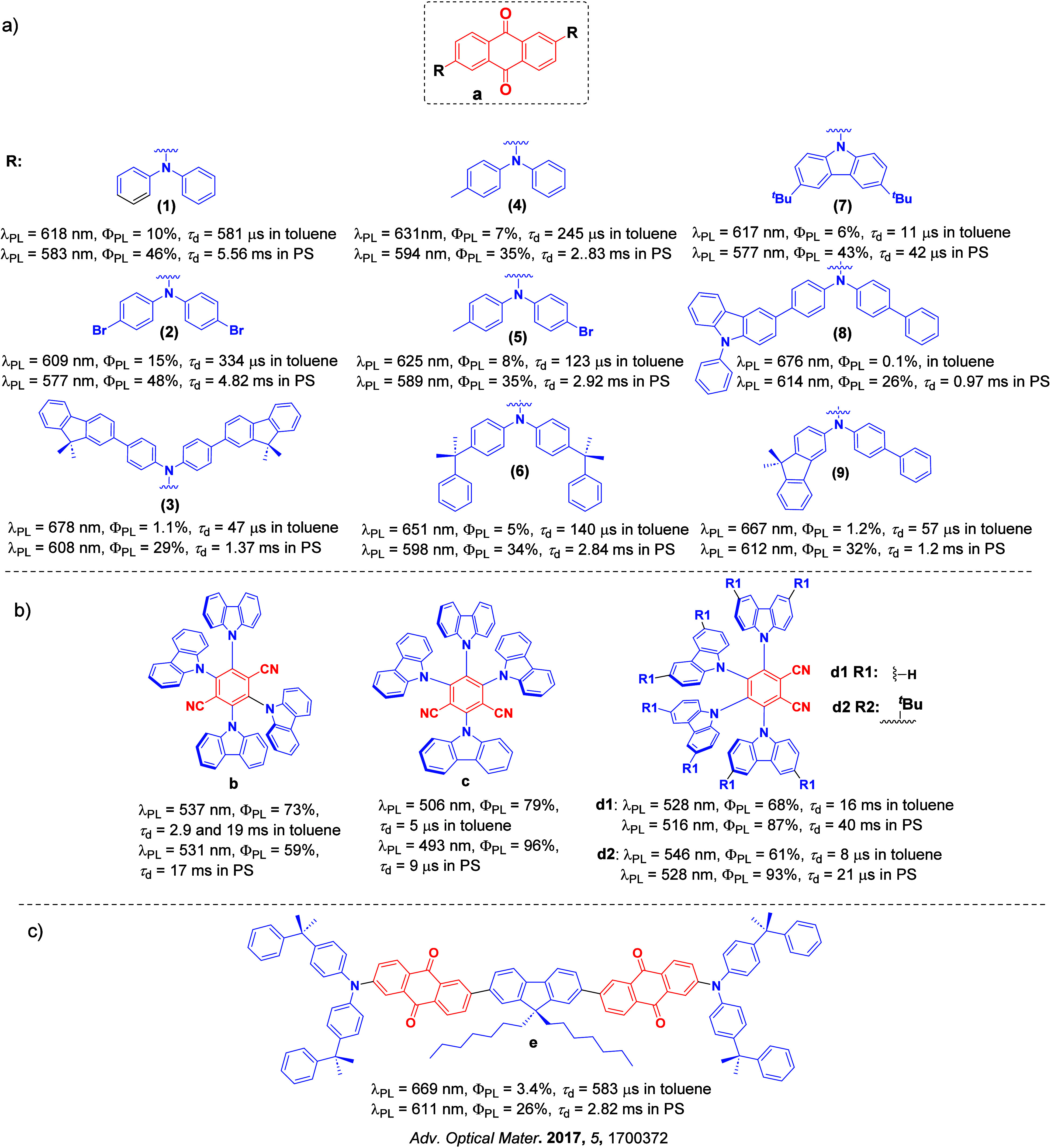
Structures of a, c) anthraquinone (**a1** to **a7** and **e**) and b) carbazole-substituted dicyanobenzenes (**b**, **c**, **d1**, and **d2**) emitters and their photophysical properties in toluene and immobilized in PS (the blue color signifies donor moieties, while the red color signifies acceptor moieties).

Zach *et al*. designed a family of Pt(II) and Pd(II) tetra­phen­yl­tetra­ben­zo­por­phyrin (**TPTBP**)-based TADF complexes for oxygen and temperature sensing (Figure 222).^934^ The Pt(II) and Pd(II) benzoporphyrin complexes were decorated with four (tetra-) or eight (octa-) alkylsulfonyl groups (**Pt-T-S**, **Pd-T-S Pt-O-S** and **Pd-O-S**), although this was eventually shown to have minimal effects on the sensing properties. Related imide-modified Pt(II) and Pd(II) benzoporphyrin complexes (**Pt-T-I** and **Pd-T-I**) were also studied, and the performance of each of these complexes was compared to the parent Pt(II) and Pd(II) benzophorphyrin complexes (**Pt-TPTBP** and **Pd-TPTBP**).^1390^ At room temperature, all Pt(II) and Pd(II) complexes emit between 620 and 652 nm and simultaneously show phosphorescence ranging between 742–786 nm and thus have Δ*E*_ST_ ranging from 0.29–0.36 eV in degassed toluene at 25 °C. The Φ_PL_ of the Pt(II) complexes (8.2 to 34%) were higher than those of the corresponding Pd(II) complexes (3.2 to 10.5%), although the phosphorescence lifetimes (*τ*_Ph_) of the Pd complexes (τ_Ph_ of between 53 and 286 μs) were much longer than of those of the Pt complexes (ranging from 12 to 47 μs), all in toluene at 25 °C. The Pt(II) complexes also show much less efficient delayed fluorescence than the Pd(II) analogues, and faster deactivation of the T_1_ state via phosphorescence. For optical temperature and oxygen sensing, these complexes were immobilized in PS at between 1–2.5 wt% doping ratios. When increasing temperature from 23 °C to 133 °C the intensity of the red TADF dramatically increased while the phosphorescence intensity decreased (Figure 223). Simultaneously, the delayed lifetimes of all complexes were significantly affected by temperature, and the observed temperature sensitivity was found to be in the range of 0.102% K^–1^ to 0.537% K^–1^. The phosphorescence lifetime was significantly affected by the presence of oxygen, and due to the greater intensity and longer lifetime of the phosphorescence band in the Pd(II) complexes, their oxygen sensitivity was found to be higher than that of Pt(II) complexes. While PS optical sensors based on Pt(II) dyes are suitable for measurement from 1 to 1000 hPa O_2_, those based on the Pd(II) complexes permit much lower oxygen partial pressure readouts. The authors further demonstrated the applicability of these dyes for simultaneous oxygen and temperature measurements.^934^

**Figure 222:**
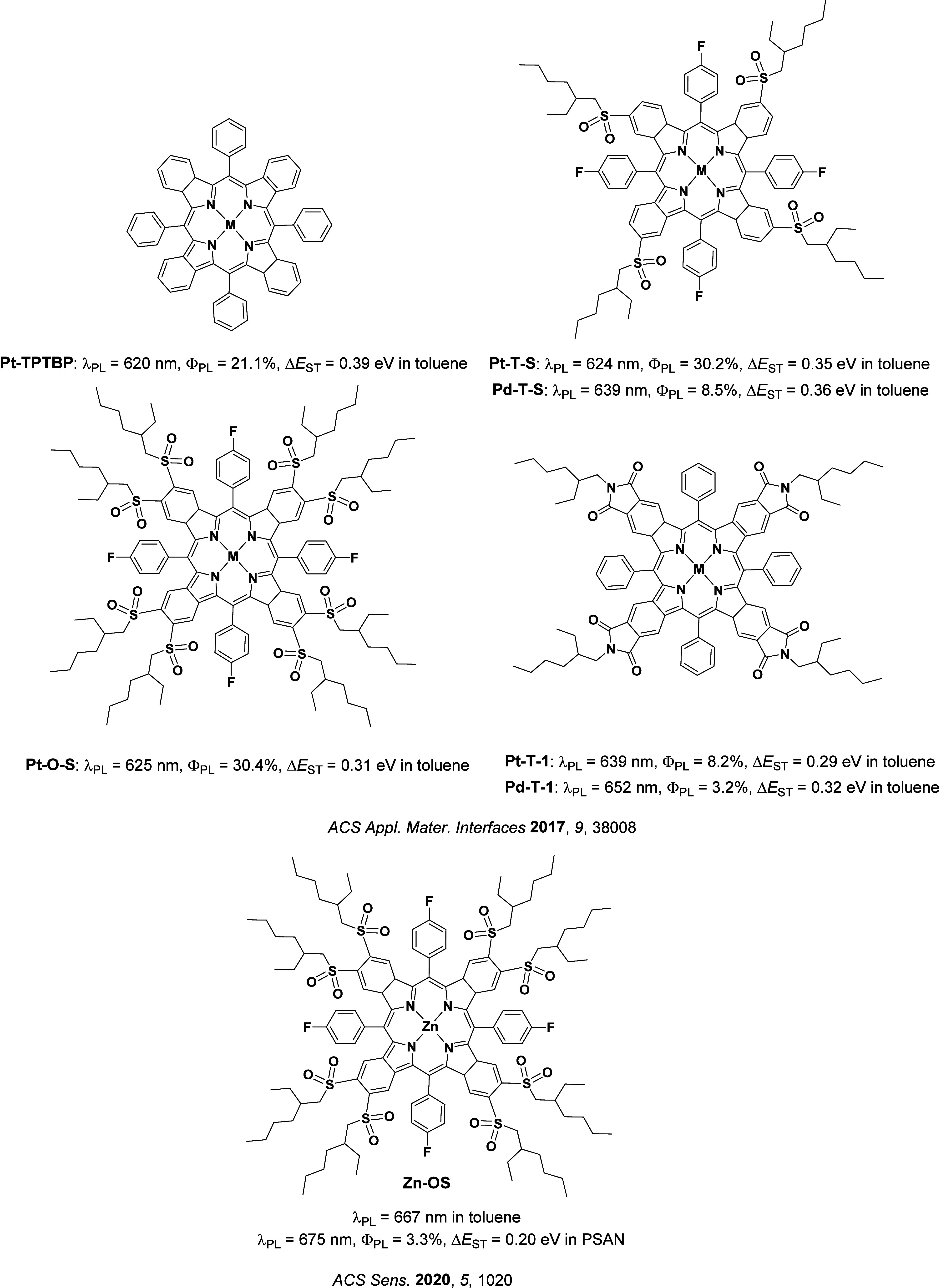
Structures of TADF Pt, Pd, and Zn benzoporphyrins and their photophysical properties in toluene.

**Figure 223:**
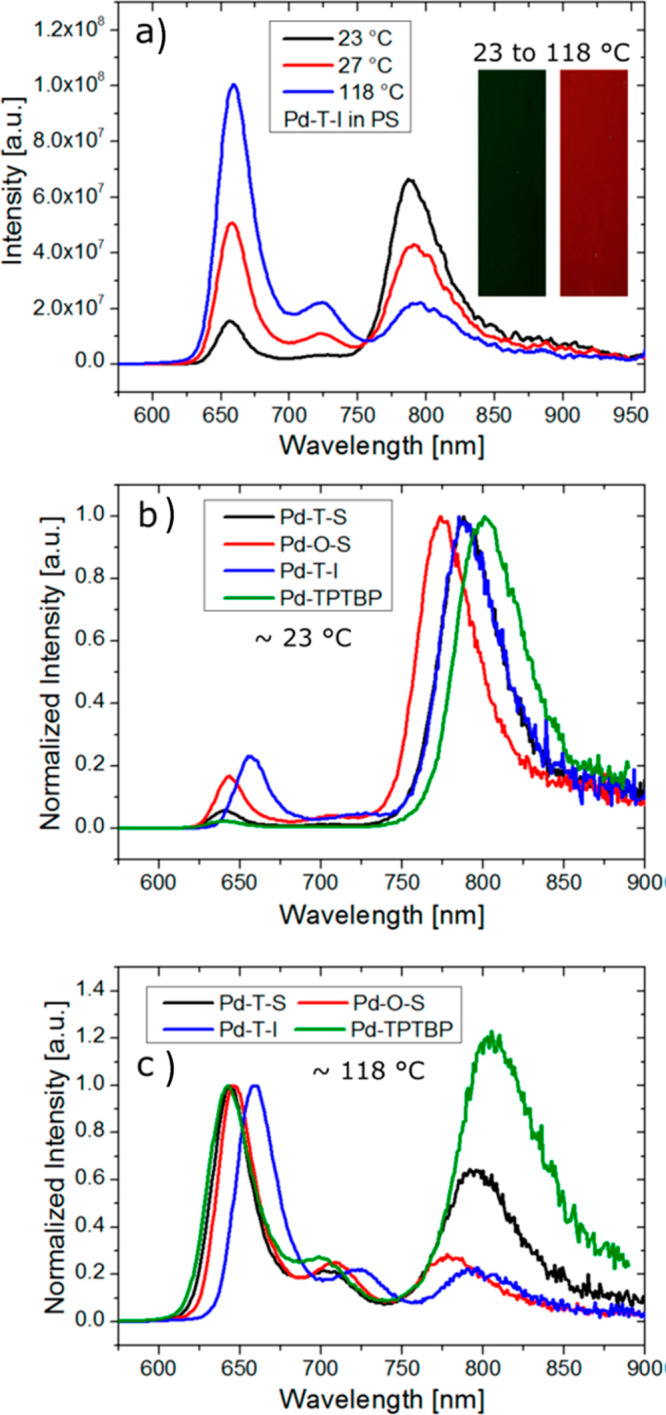
Photoluminescence properties of Pd-containing TADF sensors whose structures are shown in Figure 222. a) Temperature dependence of the emission intensity and spectra of **Pd-T-1** in polystyrene, and photographic images of the same material at 23 and 118 °C excited with a UV-Lamp at 365 nm (all under N_2_ atmosphere). b) Normalized emission spectra of **Pd-T-S**, **Pd-O-S**, **Pd-T-1**, and **Pd-TPTBP** at 23–25 °C and c) at 116–130 °C in PS under N_2_. Taken and adapted with permission from ref 934. Copyright [2017/ACS Applied Materials & Interface] American Chemical Society.

Zieger *et al*. prepared a related Zn(II) benzoporphyrin-based TADF complex, **Zn-OS** for temperature and oxygen sensing (Figure 222).^943^ Complex **Zn-OS** emits at 667 nm in toluene and has a *τ*_d_ ≥ 1 ms. An optical oxygen sensor containing **Zn-OS** (1 wt%) immobilized in poly­(sty­rene-co-acrylo­nitrile (PSAN) emits at 675 nm and has a Φ_PL_ of 3.3% and a *τ*_d_ = 7.87 ms under degassed conditions. The prompt fluorescence was affected by neither molecular oxygen nor temperature and used as internal reference. However, the *τ*_d_ and delayed fluorescence intensity (*I*_DF_) decreased significantly due to dynamic quenching by oxygen, with this quenching calibrated to achieve an optical readout of the oxygen concentration. The limit of detection at 26 °C was estimated to be 0.002 hPa O_2_. With increasing temperature, the *τ*_d_ of **Zn-OS** decreases whereas *I*_DF_ was enhanced (Figure 224). **Zn-OS** could therefore be used for simultaneous sensing of oxygen and temperature using a single material with a single-wavelength readout.

**Figure 224:**
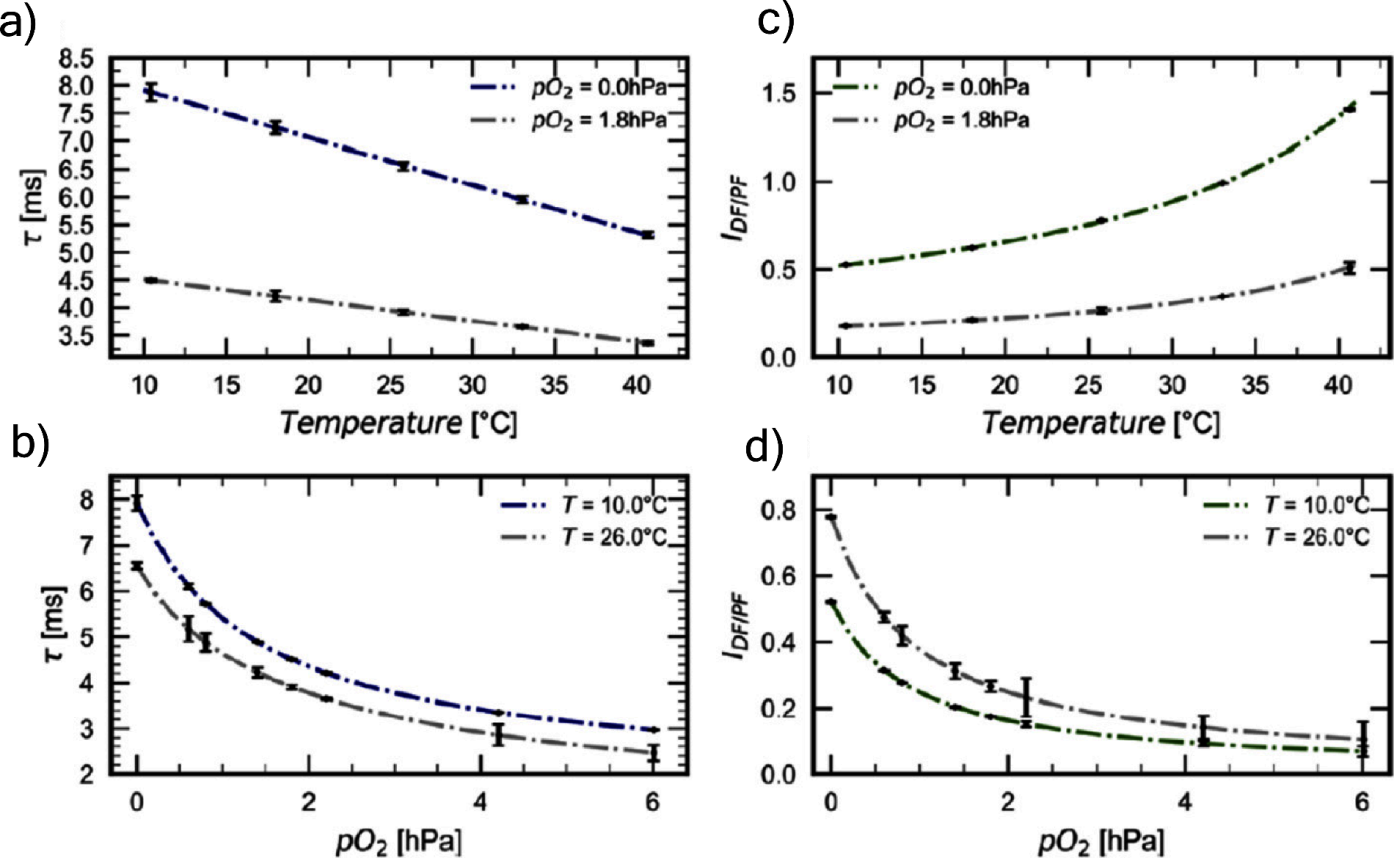
Response of luminescence decay time τ a, b) and the intensity ratio (I_DF/PF_) c, d) for Zn-OS in response to changes in temperature a, c) and oxygen b, d). The response is exemplified for two different temperatures and oxygen partial pressures. Taken and adapted with permission from ref 943. Copyright [2020/ACS Sensors] American Chemical Society.

TADF Schiff base complexes of Zn(II) (**Zn-1** and **Zn-2**, Figure 225) have similarly been developed as temperature sensors.^941^ Immobilized in PS films, **Zn-1** emits at 542 nm and has a Φ_PL_ of 30% and a τ_d_ of 7.41 ms, whereas carbazole-containing **Zn-2** emits at 547 nm and has a Φ_PL_ of 65% and a τ_d_ of 1.45 ms. The temperature sensitivities at 25 °C were 3.7 and 3.5% K^–1^ based on the changes in delayed lifetime, respectively, with temperature resolution of at least 0.03 °C. To eliminate competitive oxygen quenching, the PS-immobilized **Zn-1** was covered with an additional layer of off-stoichiometry thiol–ene polymer (OSTE) as an oxygen-consuming layer and then a layer on P(VDC-*co*-AN). Changes in the τ_d_ of the **Zn-1**/PS/OSTE/P(VDC-*co*-AN) device were tracked as a function of temperature, with sensitivities of 4.1%K^–1^ over a temperature range of 5–45 °C. The probe was stable to oxygen quenching for more than 60 days during storage under ambient air.^941^

**Figure 225:**
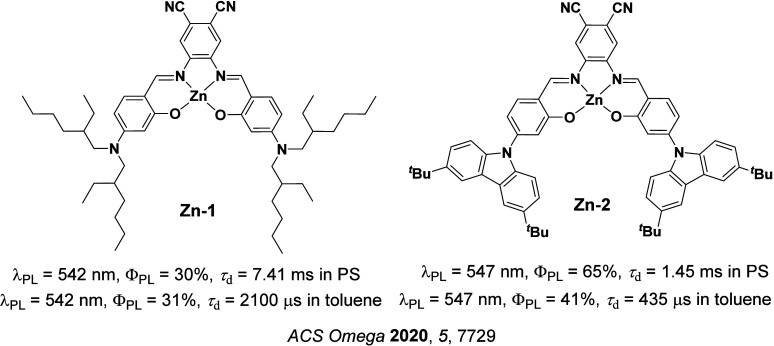
Structures of TADF **Zn-1** and **Zn-2** Schiff base complexes and their photophysical properties in toluene and polystyrene (PS) at 25 °C.

Christopherson *et al*. demonstrated that TADF polymers containing acrylate-functionalized oxadiazole-based donor–acceptor monomers such as **ACR-ODA**, **PXZ-ODA**, **PTZ-ODA**, **PAZ-ODA**, and **TTAC-ODA** can be used as oxygen sensors (Figure 226).^1391,1392^ These monomers were copolymerized with a carbazole host co-monomer (CzBA) using Cu(0) reversible deactivation radical polymerization (RDRP). These polymers have high molecular weight (*M*_n_ > 20 kDa), with polydispersities ranging from 1.11 to 1.45. As neat films the TADF polymers emit from 449–457 nm for **ACR-ODA**, 496–507 nm for **PXZ-ODA**, 510–517 nm for **PTZ-ODA**, 566–584 nm for **PAZ-ODA**, and 490–502 nm for **TTAC-ODA**. These emission wavelengths are each dependent on the attached donor moiety and on the doping concentration of the TADF-active ODA monomer, which ranged from 5 to 15 wt%. Of these polymers, **TTAC-ODA** has the highest Φ_PL_ (42% for **TTAC-ODA_0.15_**) which can be explained by the rigidity of the diphenylamine-carbazole donor dendron. The calculated Δ*E*_ST_ for these polymers is <0.011 eV, except for **TTAC-ODA** where the calculated Δ*E*_ST_ is much larger at 0.21 eV. The overall emission intensity of the polymers typically decreased as the film was coOLED and was aerated, indicating that TADF and not phosphorescence was the operational emission mechanism at room temperature.^1391^ However, **PAZ-ODA_0.15_** showed no delayed emission, which the authors attributed to the low triplet energy of the strongly donating PAZ moiety. **PTZ-ODA_0.15_** was shown to act as a single-component ratiometric oxygen sensor, able to be calibrated from the changing ratio of prompt and delayed fluorescence as a function of O_2_ concentration. This ratiometric emission behavior of **PTZ-ODA_0.15_** arose from the presence of the pseudoaxial and pseudoequatorial conformers of the phenothiazine donor (Figure 227). The **PTZ-ODA_0.15_** film was demonstrated to be able to sense oxygen concentrations from 0 to 50% and was also incorporated into water-soluble polymer dots (Pdots) to sense O_2_ in biological systems.

**Figure 226:**
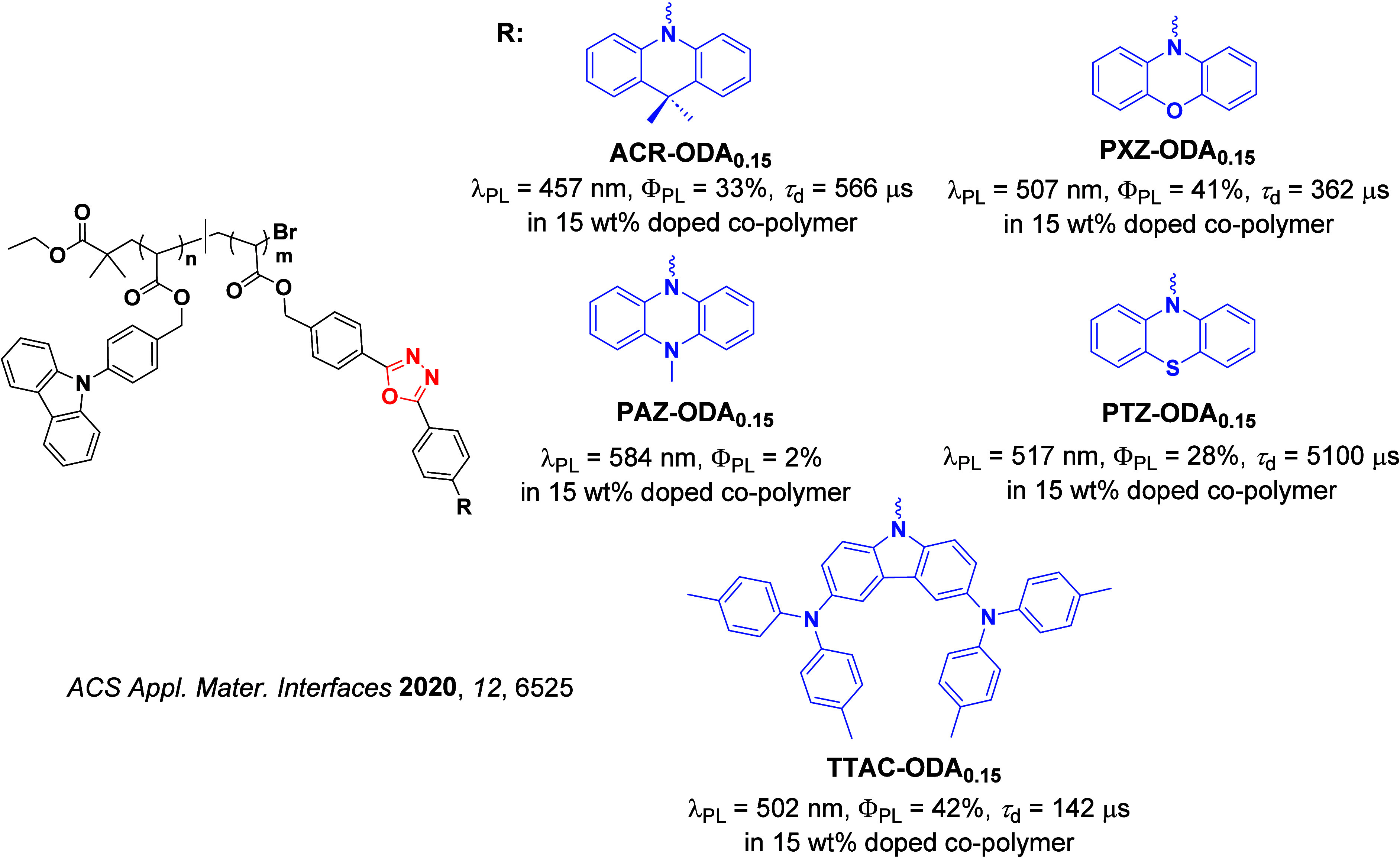
Structures of **ACR-ODA**, **PXZ-ODA**, **PTZ-ODA**, **PAZ-ODA** and **TTAC-ODA** and their photophysical properties in co-polymers with 15% TADF monomer content (the blue color signifies donor moieties, while the red color signifies acceptor moieties).

**Figure 227:**
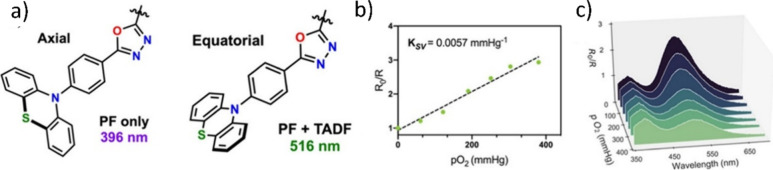
a) Illustration of pseudoaxial and pseudoequatorial conformers of **PTZ-ODA** and their respective emission wavelengths. b) Stern–Volmer plot calibrating I_516_/I_396_ emission ratios against partial pressures of O_2_ for a thin film of **PTZ-ODA_0.15_**. c) Fluorescence emission response of **PTZ-ODA_0.15_** to O_2_ concentrations. PF = prompt fluorescence. Taken and adapted with permission from refs 943 and 1391 . Copyright [2020/ACS Applied Materials & Interfaces] American Chemical Society.

Christopherson *et al*. developed additional temperature sensing materials by co-polymerizing naphthalimide (NAI)-based red-emissive TADF acrylic monomers (**NAI-DMAC**, **NAI-PTZ** and **NAI-POZ**) with an acrylate-functionalized 1,3-bis­(*N*-carba­zol­yl)­ben­zene (mCPA) co-monomer as a host (Figure 228).^1392^ Both star-shaped and linear polymers (**P1-P17**) were synthesized, showing high molecular weight (12,000 < *M*_n_ < 22,000) and narrow polydispersities between 1.07 and 1.25. The star-shaped polymers (**P1-P8**) were obtained from polymerization of the mCPA host monomer and TADF dopant monomers (1–12 mass%) with a four-arm initiator (4-BriBu), while the linear polymers (**P9-P11**) were obtained from polymerization of the mCPA host monomer and TADF dopant monomers (12 mass%) with ethyl α-bromo­iso­butyrate (EBiB) as the initiator.^1393^ All three TADF monomers show broad CT emission in toluene at 630, 582, and 594 nm for **NAI–DMAC**, **NAI–PTZ**, and **NAI–POZ**, respectively. Each monomer emission spectrum also exhibits peaks at 343 and 362 nm, attributed to fluorescence from the LE state of the NAI core. The star-shaped polymer with 12 mass% **NAI-DMAC** (**P5**) emits at 638 nm and has a Φ_PL_ of 58%, 12 mass% **NAI-PTZ** (**P7**) emits at 700 nm and has a Φ_PL_ of 11%, and 12 mass% **NAI-POZ (P8)** emits at 675 nm and has a Φ_PL_ of 4.5% in toluene. Surprisingly, only **P5** showed evidence of delayed fluorescence, with τ_d_ of 6.1 μs in toluene. However, all three polymers **P5**, **P7** and **P8** as neat films show delayed fluorescence with emission at around λ_PL_ of 610, 650, and 660 nm, and with τ_d_ of 6.14, 76.66, and 51.97 μs, respectively, which track with their respective Δ*E*_ST_ of 0.12, 0.22 and 0.21 eV. Like their monomers, these polymers exhibited dual-emission consisting of a high-energy fluorescence from the NAI acceptor (λ_PL_ = 340 nm in toluene) and a lower-energy long-lived TADF from a CT excited state (λ_PL_ = 633–711 nm in toluene).

**Figure 228:**
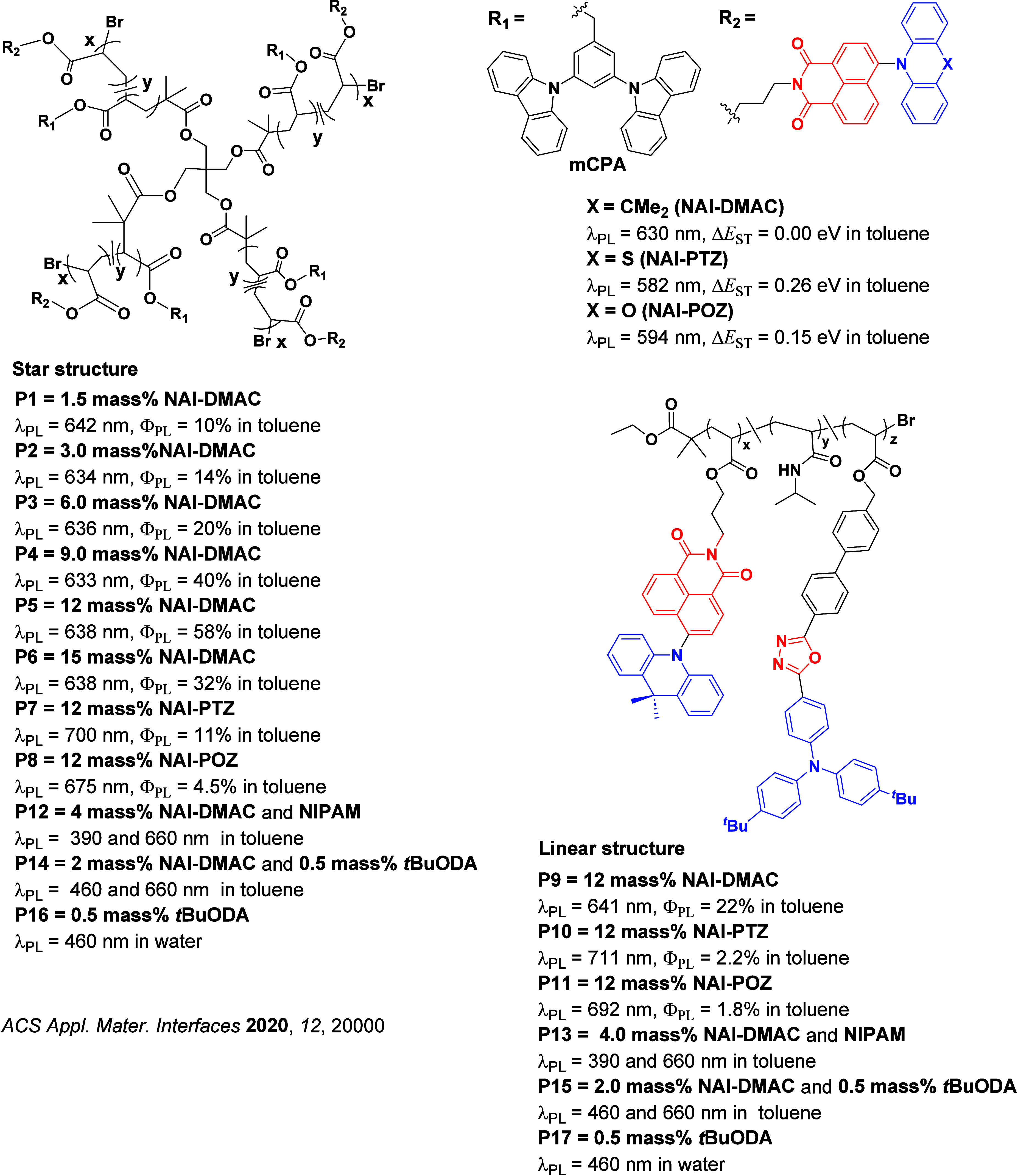
Structures of temperature-responsive linear and star-shaped TADF polymers **P1** to **P17** and their photophysical properties (the blue color signifies donor moieties, while the red color signifies acceptor moieties).

The dual-emission behavior of the **NAI-DMAC** monomer was exploited to develop ratiometric temperature-responsive polymers **P12** and **P13** (Figure 229). Star-shaped polymer **P12** and linear polymer **P13** were developed by copolymerizing 4.0% **NAI-DMAC** and *N-iso*propylacrylamide (NIPAM) with 4-BriBu and EBiB initiators, respectively. The *I*_390_/ *I*_660_ ratio of emission peaks at 390 nm (NAI emission) and 660 nm (CT state) increases linearly with temperature from 20 to 70 °C for **P12** and **P13** (Figure 229). To make an all-visible sensor the authors employed a second blue triphenylamine-oxadiazole co-dopant ***t*BuODA** that would emit as a result of FRET from the UV-emitting NAI-based LE state, and prepared star-shaped polymer **P14** by co-polymerizing 2.0% **NAI-DMAC** and 0.5 mass% ***t*BuODA** with 4-BriBu initiator. A linear analog **P15** contained 0.5 mass% ***t*BuODA** and 2.0% **NAI-DMAC** and used EBiB initiator. These two polymers show dual-emission with characteristic TADF from **NAI-DMAC** at 660 nm and fluorescence from ***t*BuODA** at 460 nm at 20 °C. Upon increasing the temperature to 70 °C a large increase in the blue emission intensity was registered in both polymers, which was attributed to increased FRET from the **NAI-DMAC** to ***t*BuODA** (Figure 230). The ratiometric optical response to temperature of **P14** is 32 ± 4% K^–1^ and 30 ± 6% K^–1^ for **P15**; fluorescent star-shaped **P16** and linear polymer **P17** with only **tBuODA** monomers did not show such a temperature-dependent behavior.

**Figure 229:**
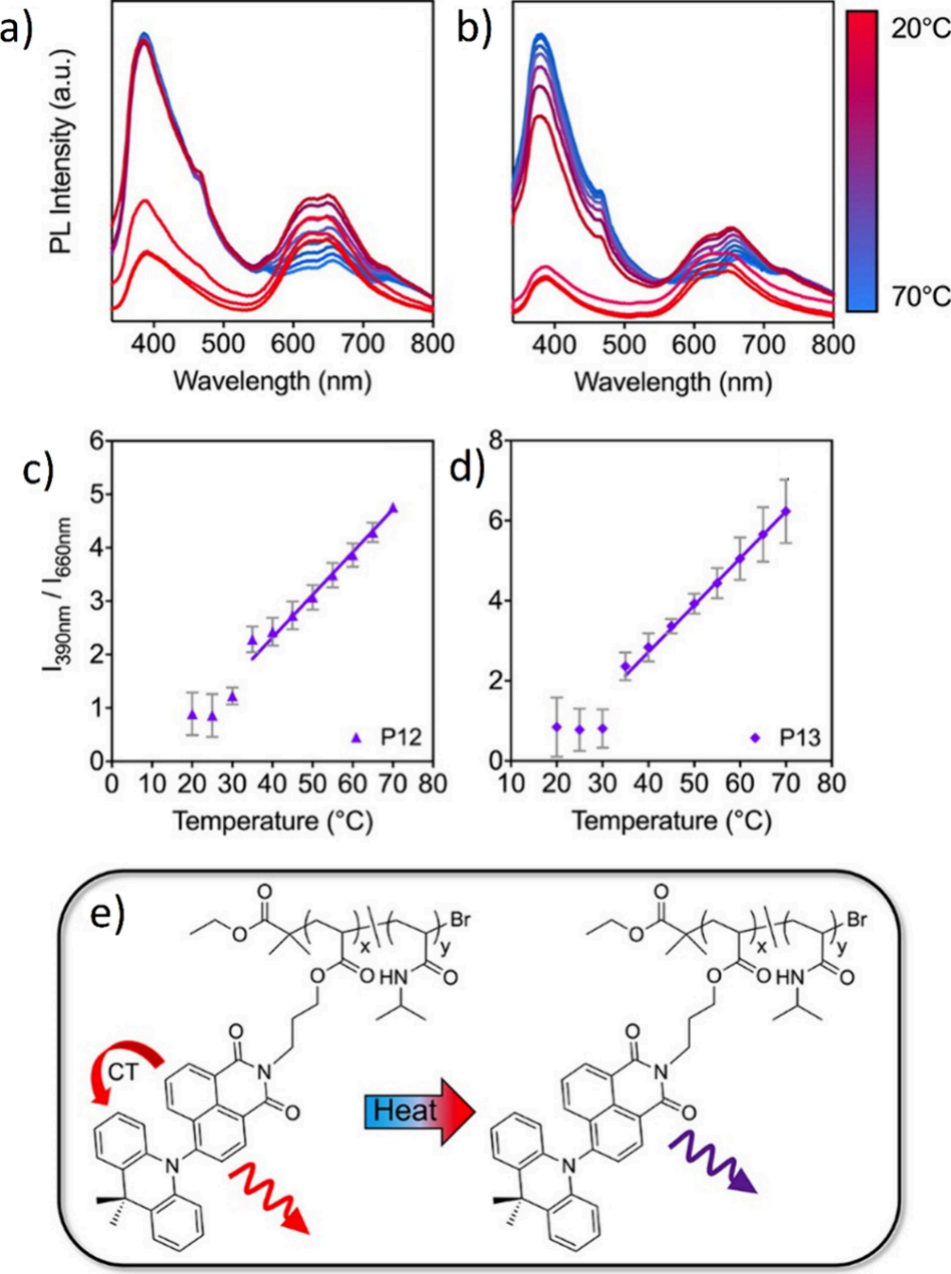
Temperature-dependent emission spectra of a) **P12** and b) **P13**. Ratiometric plot of I_390_/I_660_ vs temperature for c) **P12** and d) **P13**. e) Schematic representation of the thermal response of these materials. Taken and adapted with permission from ref 1392. Copyright [2020/ACS Applied Materials & Interfaces] American Chemical Society.

**Figure 230:**
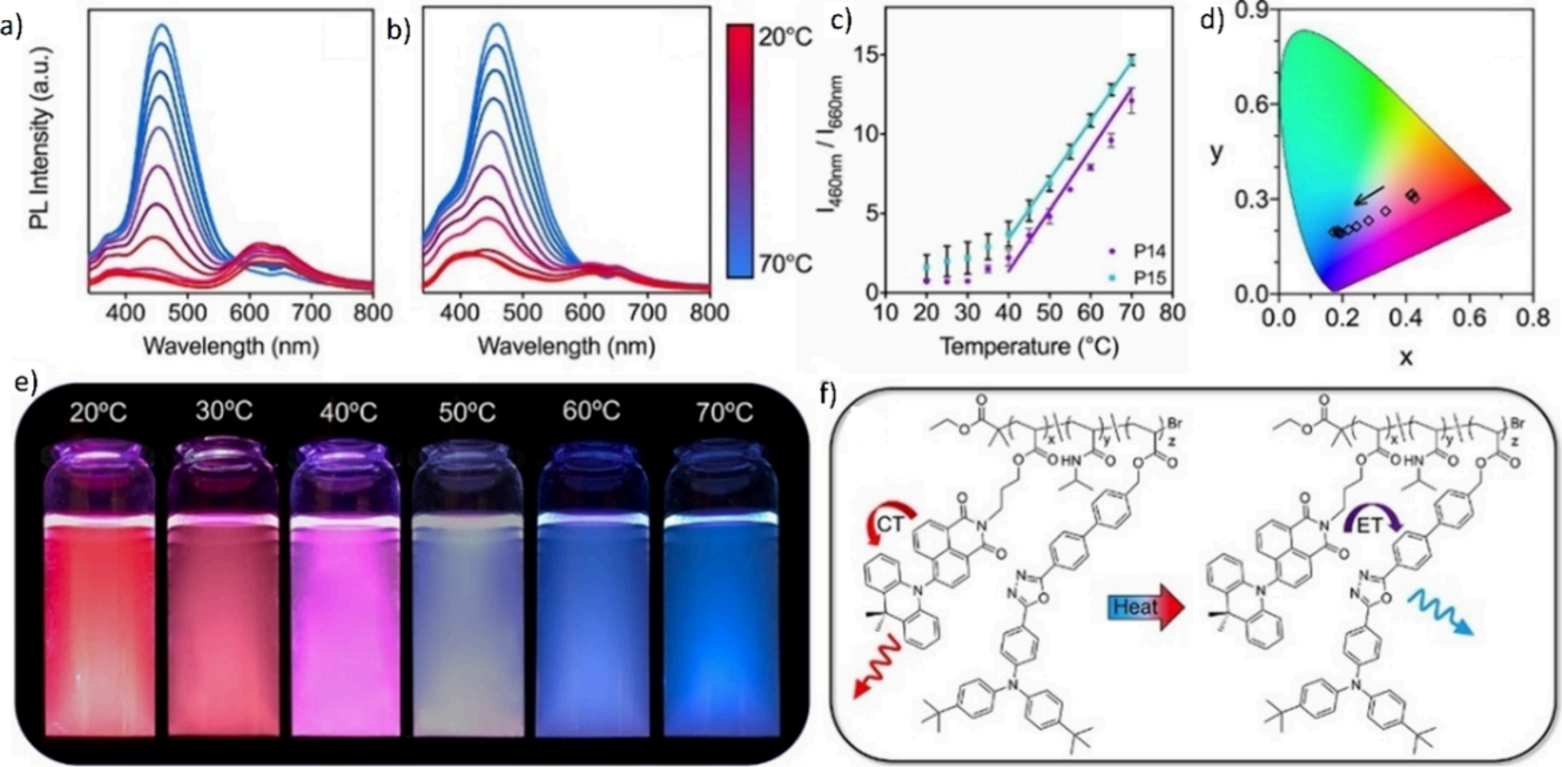
Temperature-dependent emission spectra of a) **P14** and b) **P15**. c) Ratiometric plot of I_460_/I_660_ vs temperature for **P14** and **P15**. d) CIE plot of **P14** at measured temperature points. e) Visual representation of **P14** color changes at various temperatures. f) Schematic representation of the thermal response for these materials. Taken and adapted with permission from ref 1392. Copyright [2020/ACS Applied Materials & Interfaces] American Chemical Society.

Li *et al*. developed three emitters containing diphenylsulfone (DPS) as an acceptor attached to different donors such as, 5-nitroindole (**1**), 5-aminoindole (**2**) and 5-acetaminoindole (AMID, **3**), with the goal of developing sensors for solvent polarity (Figure 231).^1394^ Emitters **1** and **2** are purely fluorescent while emitter **3** is TADF-active. Compound **3** shows dual-emissions at 332 nm (strong LE fluorescence) and 435 nm (weak CT emission with TADF) in DCM under air. Upon degassing the Φ_PL_ of **3** increased from 12 to 33% and the τ_d_ increased from 55 μs to 167 μs, linked to a small Δ*E*_ST_ of 0.17 eV that is with TADF; the τ_PL_ of 332 nm band is 22 ns and is insensitive to oxygen.^1394^ Using the invariant LE fluorescence as an internal reference, the ratio of the intensities of the LE and CT bands as well as the ratios of the prompt and delayed lifetimes were used to calibrate against solvent polarity (Figure 231). Increasing solvent polarity from hexane to DMF caused the ratio of emission wavelengths for the CT and LE states to increase from 1.17 to 1.45, and the lifetime decreased from 55 μs in toluene to 1.6 μs in DMF, showing that **3** can act as a sensitive optical probe of polarity. Further, the authors employed a 3-D ratiometric luminescent sensing strategy to detect the microenvironment polarity in a biological membrane.

**Figure 231:**
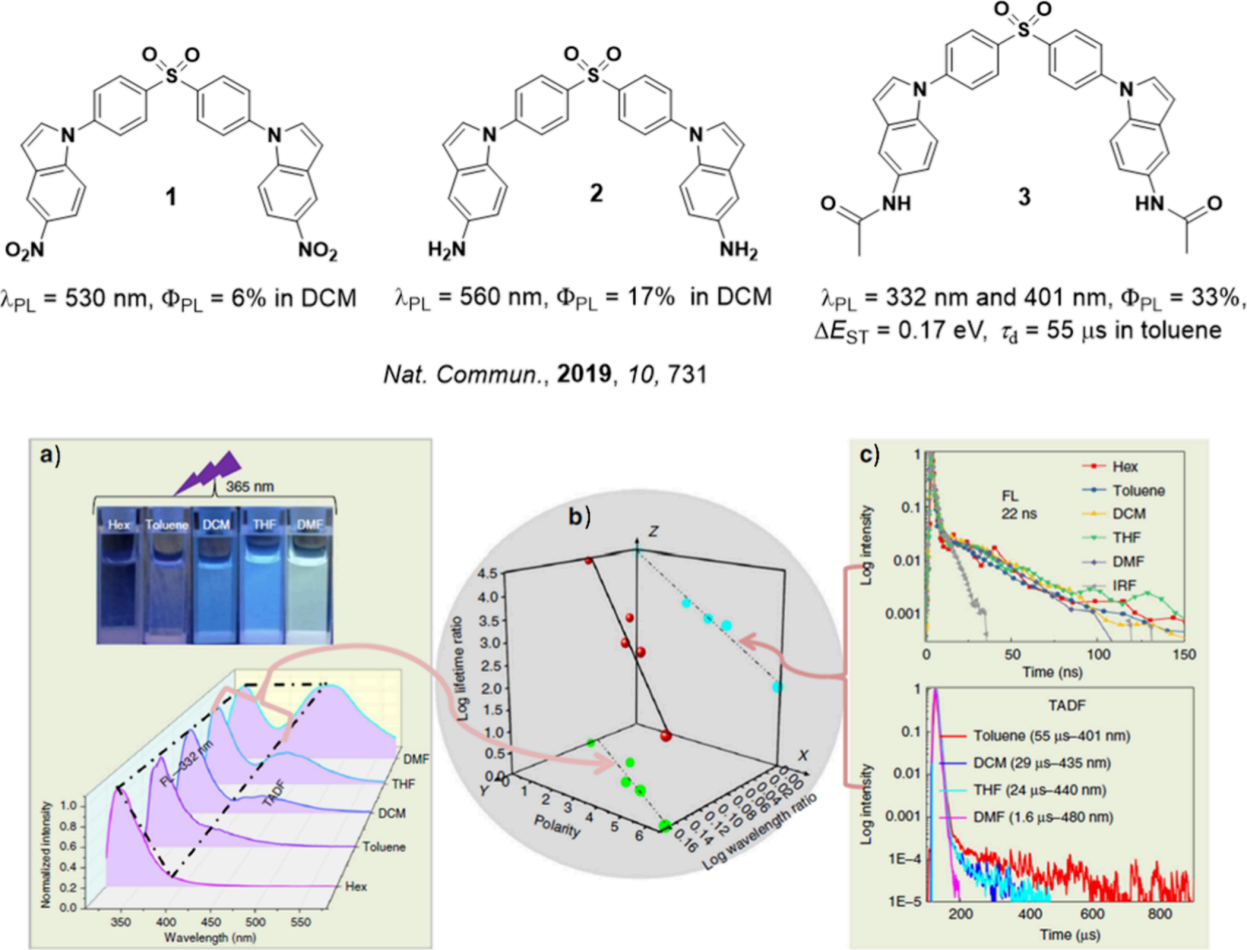
Top: Structures of TADF diphenyl sulfone-based solvent polarity sensors **1**, **2**, and **3** and their photophysical properties. Bottom: Correlation of wavelength and lifetime of TADF and FL emission with polarity. a) Emission spectra of **3** in solvents of differing polarity under ambient conditions (λ_exc_ = 300 nm) and corresponding photographs of **3** under UV light (λ_exc_ = 365 nm). b) Linear fitting of the log of wavelength and lifetime ratios (TADF to FL) as a function of solvent polarity. c) Time-resolved PL decays of FL and TADF bands in different solvents. Taken and adapted with permission from ref 1394. Copyright [2019/Nature Communications] Springer Nature.

Aside from temperature and oxygen sensing, TADF compounds have also been used for anion and cation sensing. Yin *et al*. developed a fluorescein-based fluorescence turn-on chemosensor **DCF-MPYM-lev** for sulfite ion (SO_3_^2–^) detection (Figure 232).^1395^ Compound **DCM-MPYM-lev** is very weakly emissive; however, on the addition of [SO_3_]^2–^ into the 3.0 μM **DCM-MPYM-lev** solution in CH_3_CN/PBS buffer (1/1), the fluorescence intensity significantly increases and dual-emissions at 535 nm (weak emission) and 640 nm (strong emission) was observed, providing a detection limit of 2.98 μM of [SO_3_]^2–^. The mode of action of this sensor is the sulfite-mediated deprotection of the levulinyl group, thereby releasing the luminescent **DCF-MPYM** (Figure 232). Previously, **DCF-MPYM** was reported to be TADF, with a *τ*_d_ = 22.11 μs in deaerated ethanol and a Δ*E*_ST_ of 28 meV. This compound was also used as a bioimaging reagent of breast cancer MCF-2 cells.^1396^ Additionally, **DCF-MPYM** was used as a chemosensor to detect cysteine and hypochlorite.^1397,1398^ Compound **DCF-MPYM-lev** was used to monitor the exogenous [SO_3_]^2–^ concentration in living cells.

**Figure 232:**
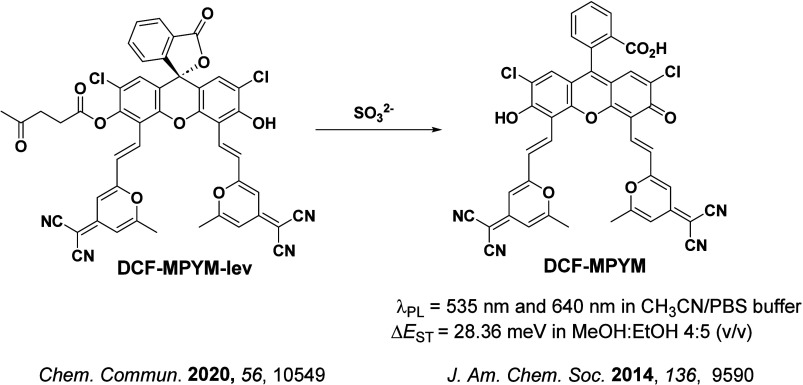
The sensing mechanism of **DCF-MPYM-lev** to [SO_3_]^2–^ ions, forming the TADF emitter **DCF-MPYM.**

**Figure 233:**
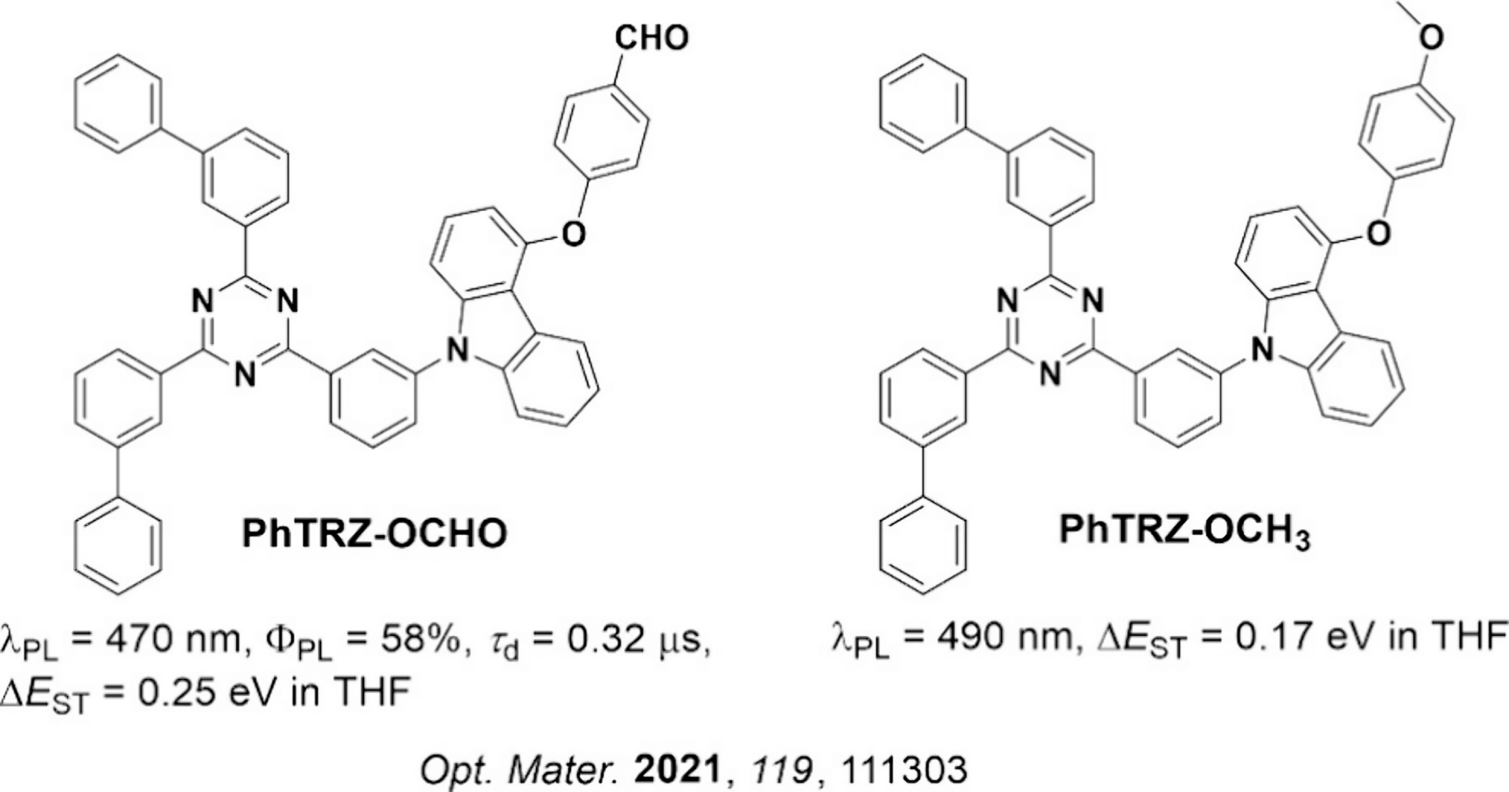
Chemical structures and the photophysical properties of the TADF emitters **PhTRZ-OCHO** and **PhTRZ-OCH_3_** used as sensors for metal ion sensing (i.e., Ba^+^, Ca^+^, Cd^2+^, Co^2+^, Cr^2+^, Cu^2+^, Fe^3+^, Hg^2+^, K^+^, Mg^2+^, Mn^2+^, Na^+^, Ni^2+^, Pb^+^).

Qiu *et al*. reported carbazole-triazine TADF emitter **PhTRZ-OCHO** (Figure 233) as a fluorescence turn-off/fluorescence quenching sensor for the detection of Na^+^, Mg^2+^ and Fe^3+^ ions.^1399^ **PhTRZ-OCHO** emits at 470 nm in THF with a Φ_PL_ of 58% and *τ*_d_ of 0.32 μs, while a control TADF compound **PhTRZ-OCH_3_** emits at 490 nm. **PhTRZ-OCHO** has a Δ*E*_ST_ 0.25 eV while **PhTRZ-OCH_3_** has a Δ*E*_ST_ of 0.17 eV. Though the emission intensity of **PhTRZ-OCHO** at 470 nm decreased upon the addition of many of the metal ions tested (Ba^+^, Ca^+^, Cd^2+^, Co^2+^, Cr^2+^, Cu^2+^, Fe^3+^, Hg^2+^, K^+^, Mg^2+^, Mn^2+^, Na^+^, Ni^2+^, Pb^+^), the strongest emission quenching occurred upon addition of Na^+^, Mg^2+^ and Fe^3+^, with detection limits of 7.03 × 10^–7^, 6.7 × 10^–7^ and 5.9 × 10^–7^ mol/L, respectively. The authors hypothesized that the excellent fluorescence quenching behavior was due to the presence of the metal-binding aldehyde group in **PhTRZ-OCHO**, which stabilized the CT band and becomes non-emissive upon complexation. As such an interaction is not possible in the control emitter **PhTRZ-OCH_3_**, it does not show any selective sensing of these cations.

Recently, Ma *et al*. reported an unusual application of TADF emitters **DMAC-TRZ**, **4CzIPN**, and **4CzTPN-Bu** (Figure 234c) as scintillators for the detection and imaging of X-ray radiation.^1400^ X-ray photons initially interact with atoms in organic molecules through both the photoelectric effect and Compton scattering, which causes ejection of high-energy electrons. These high-energy electrons further interact with emitter molecules and generate a cascade of secondary lower-energy electrons that ionize or excite other molecules to generate electron–hole pairs (Figure 234). Directly analogous to exciton formation following electrical excitation in OLEDs, in the scintillation process X-ray irradiation and subsequent recombination of ionized molecules (holes) with uncorrelated ejected electrons favors triplet states over singlet states in a 1:3 ratio. While fluorescent scintillators waste these triplet excitons, the use of phosphorescent emitters in scintillators is undesirable as it leads to significant deadtime between detector events (due to long exciton lifetimes). In TADF emitters, these triplet excitons can be harvested through rapid RISC and increase the amount of light available to the detector electronics (Figure 234b). As scintillators, **DMAC-TRZ**, **4CzIPN**, and **4CzTPN-Bu** embedded in 10 wt% sucrose octaacetate (SO) exhibit internal X-ray-to-light conversion efficiencies of 73,500 ± 400, 33,200 ± 60 and 44,900 ± 210 photons MeV^–1^, supported both by efficient conversion of triplet excitons to produce light, and reduced self-absorption. The limit of detection (LOD) of the TADF scintillator for **DMAC-TRZ** is 103.2 ± 2.9 nGy_air_ s^–1^, 250 ± 12 nGy_air_ s^–1^ for **4CzIPN**, and 208 ± 4 nGy_air_ s^–1^ for **4CzTPN-Bu**, which are much lower than a competing TTA compound (anthracene, 506 ± 21 nGy_air_ s^–1^ in SO). To demonstrate the practical application of TADF scintillators for X-ray imaging, the TADF emitters were embedded in a SO matrix at 0.5 to 10 wt% doping to produce solid-state scintillator screens (Figure 235). The radioluminescence (RL) intensity of these TADF emitters was 612–743% higher than that of anthracene in SO. The 0.5% **DMAC-TRZ**:SO scintillator screen was the used to produce X-ray images of industrial and biological samples at a high resolution of 16.6 line pairs (lp) mm^–1^ (Figure 235).

**Figure 234:**
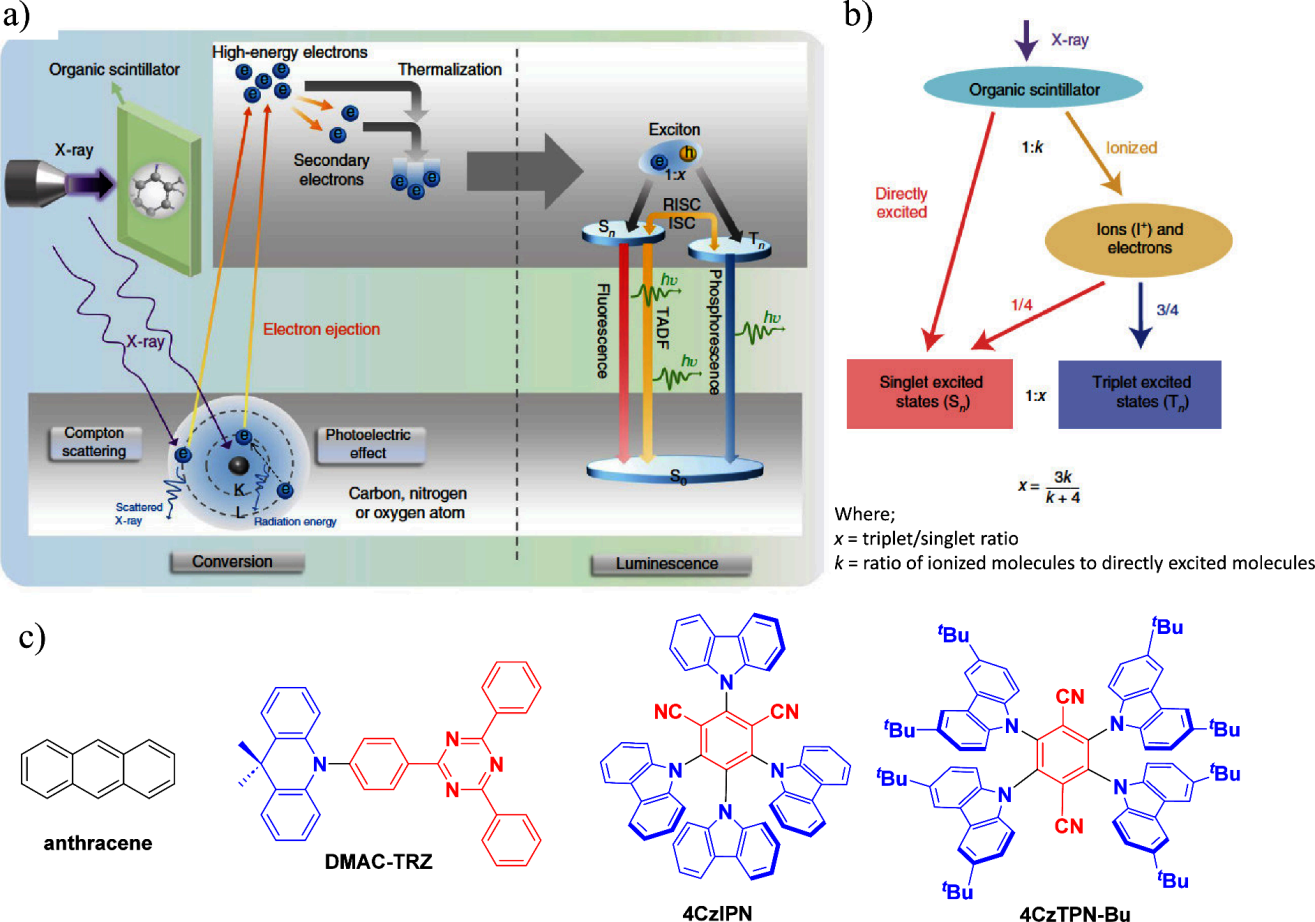
a) Schematic mechanism of X-ray-induced emission in organic TADF scintillators. b) Production ratio of S and T excited states in an organic TADF scintillator under X-ray irradiation. c) Molecular structures of anthracene, **DMAC-TRZ**, **4CzIPN**, and **4CzTPN-Bu** (the blue color signifies donor moieties, while the red color signifies acceptor moieties). Taken and adapted with permission from ref^1400^. Copyright [2020/Nature Materials] Springer Nature.

**Figure 235:**
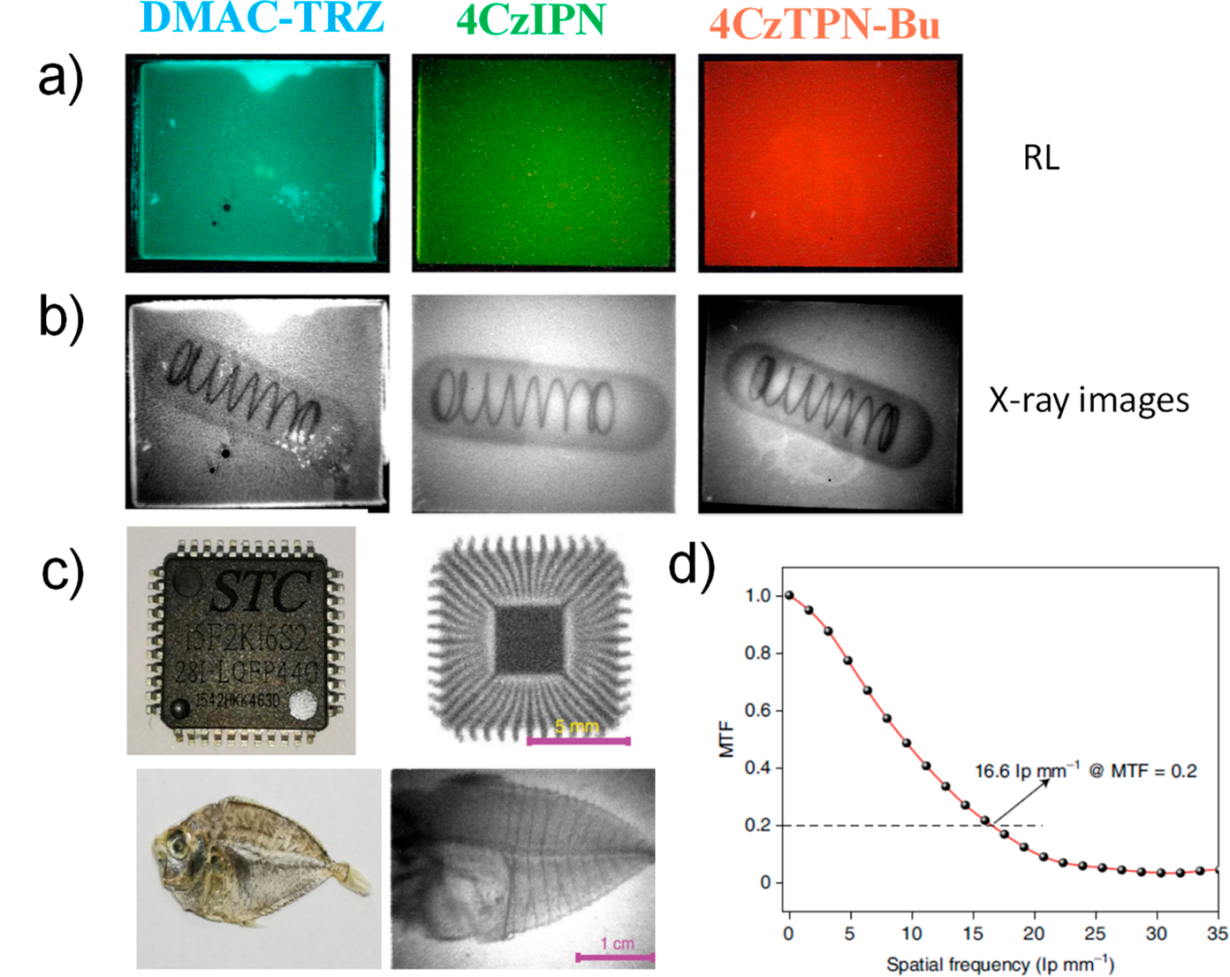
Photographs and X-ray images of TADF scintillator screens used for imaging. a) photographs under X-ray irradiation of 10 wt% **DMAC-TRZ**:SO, **4CzIPN**:SO, and **4CzTPN-Bu**:SO scintillator screens. b) X-ray images of an encapsulated metallic spring collected using the same scintillator screens. c) Bright field (left) and X-ray (right) images of a microchip and a fish using a 0.5wt% **DMAC-TRZ**:SO scintillators screen. d) Modulation transfer functions (MTFs) of X-ray images. Taken and adapted with permission from ref^1400^. Copyright [2022/Nature Materials] Springer Nature.

Highly efficient and reliable scintillators with low detection limits could be achieved by using organic scintillation materials with high X-ray absorption capability, high exciton utilization efficiency, and high photoluminescence quantum yield. Recognizing that larger atoms usually have larger X-ray absorption cross-sections, Wang *et al*. introduced heavy halogen atoms (Cl, Br and I) generating the **4CzIPN** derivatives **TADF-H**, **TADF-Cl**, **TADF-Br**, and **TADF-I**, which were used to fabricate organic scintillator screens for X-ray imaging (Figure 236).^1401^ The four emitters each emit at approximately 505 nm in 1 wt% doped PMMA films. Additionally, the τ_d_ of 4.53 μs for **TADF-H**, 2.99 μs for **TADF-Cl**, 2.22 μs for **TADF-Br**, and 1.42 μs for **TADF-I** systematically decrease due to the heavy-atom effect enhancing SOC and accelerating RISC. To illustrate the application of these TADF emitters in the detection and imaging of X-rays, scintillation screens consisting of 60 wt% emitter doped in PMMA were fabricated with different thicknesses (0.1 to 0.5 mm). Due to presence of the heavy atoms, the X-ray absorptivity of the films with **TADF-I** and **TADF-Br** is higher than the others, and the relative light yields also increase (∼18000 photons MeV^–1^ for **TADF-I** and **TADF-Br**, 7076 photons MeV^–1^ for **TADF-Cl**, and 1892 photons MeV^–1^ for **TADF-H**, Figure 236b-c). The role of the increased X-ray cross section is highlighted by relatively uniform Φ_PL_ of the scintillation screens, ranging from 44–65% and highest for **TADF-H**. Due to the high relative light yield in **TADF-I** and **TADF-Br**, the LOD is significantly improved in these (both ∼45 nGy s^–1^) in comparison to **TADF-H** (438.5 nGy s^–1^ and **TADF-Cl** (100.6 nGy s^–1^), and is comparable to a reference scintillator material LYSO:Ce (34.8 nGy s^–1^). The RL intensities of these TADF scintillators was also found to be linearly correlated with the X-ray dosages, allowing for X-ray imaging applications (Figure 236d). **TADF-Br** exhibited high X-ray imaging resolution of 12.0 lp mm^–1^ in comparison to **TADF-H** (5.1 lp mm^–1^), **TADF-Cl** (6.8 lp mm^–1^), and **TADF- I** (9.4 lp mm^–1^, Figure 236e-f).

**Figure 236:**
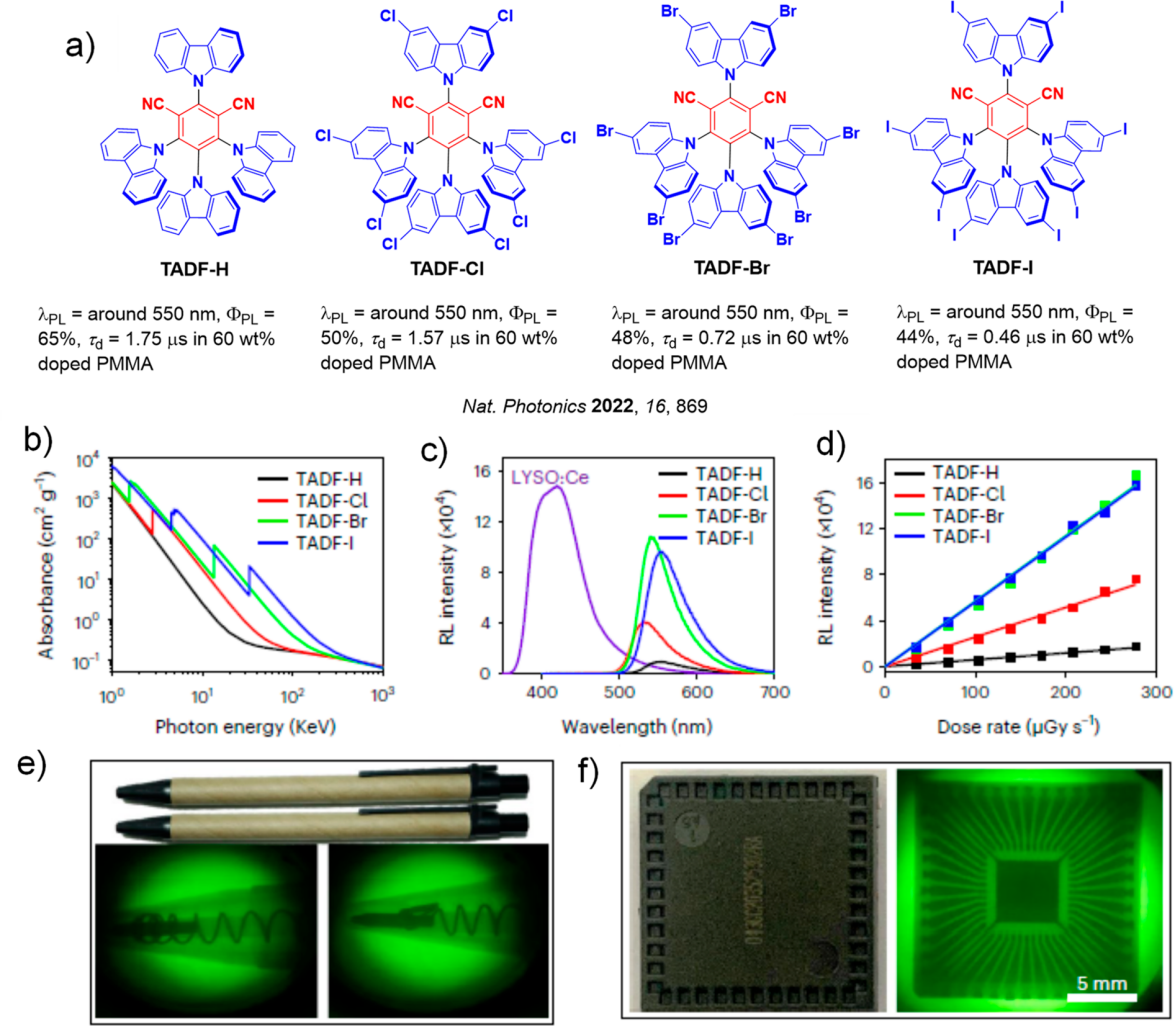
a) Molecular structures of **4CzIPN** (**TADF-H**) derivatives **TADF-Cl**, **TADF-Br**, and **TADF-I** and their photophysical properties in 60 wt% doped films in PMMA. b) X-ray absorption spectra of **TADF-H**, **TADF-Cl**, **TADF-Br**, and **TADF-I** measured as a function of X-ray energy. c) RL spectra of these four TADF chromophores at the optimal thickness compared with the reference scintillator LYSO:Ce (dose rate, 174 μGy s^–1^). d) Detection limits of the **TADF-H**, **TADF-Cl**, **TADF-Br**, and **TADF-I** emitters. e) and f) Bright- and darkfield photographs of a pen e) and an electronic chip f) before and after X-ray exposure (dose rate, 174 μGy s^–1^). Taken and adapted with permission from ref 1401. Copyright [2022/Nature Photonics] Spring Nature.

To develop reabsorption-free X-ray imaging scintillators (required for high-quality images at low detection limits) along with achieving air and light stability, Wang *et al*. reported the design of a nanocomposite film (**Zr-fcu-BADC-MOF-TADF**), Figure 237, consisting of a combination of a luminescent MOF (**Zr-fcu-BADC-MOF**) and TADF chromophores (**4CzTPN-Bu**, Figure 234c).^1402^ The authors demonstrated that there was nearly 100% energy transfer from the fluorescent MOF to the TADF co-dopant, which, coupled with direct harnessing of singlet and triplet excitons mediated by the co-dopant, translated into a remarkable enhancement of the radioluminescence upon X-ray irradiation (Figure 237). The detection limit of the optimized **D-A_0.4_** nanocomposite film (D = MOF, A = **4CzTPN-Bu**) at 256 nGy s^–1^ is significantly improved compared to the undoped **Zr-fcu-BADC-MOF** film (15,000 nGy s^–1^) and a reference **4CzTPN-Bu** film at (1,600 nGy s^–1^). This detection limit is approximately 22 times lower than the standard dosage for X-ray diagnostics (5.5 mGy s^–1^) (Figure 238), while the **D-A_0.4_** nanocomposite film simultaneously exhibits excellent photostability (Figure 238e). The **D-A_0.4_** nanocomposite film-based scintillator could thus be used for imaging of a steel famework (Figure 238g).

**Figure 237:**
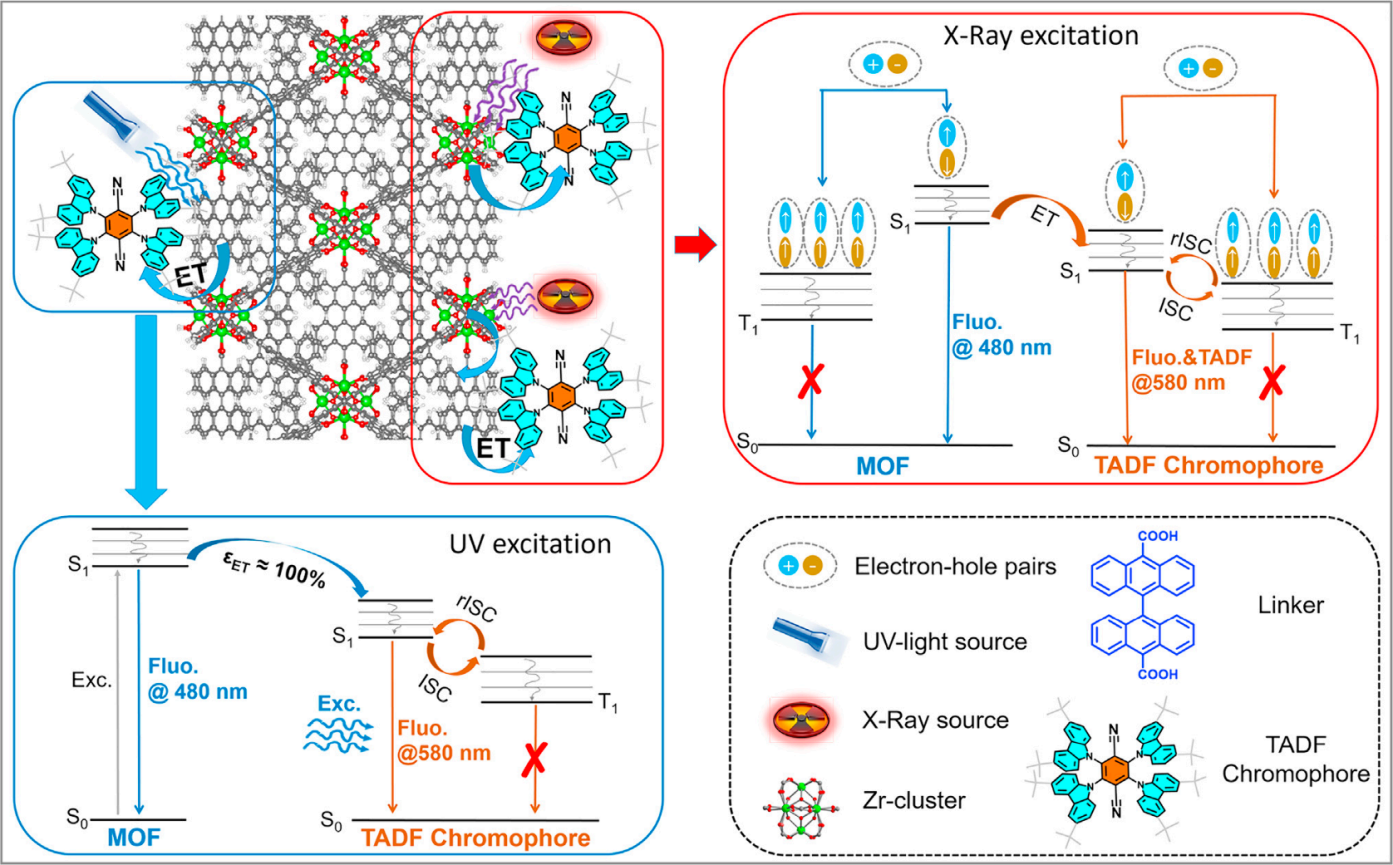
Schematic representation of the radioluminescence mechanism of **Zr-fcu-BADC-MOF-4CzTPN-Bu** nanocomposite materials. Illustration of highly efficient energy transfer from the **Zr-fcu-BADC-MOF** to **4CzTPN-Bu** under ultraviolet light irradiation (bottom left) and the significantly enhanced radioluminescence efficiency of **4CzTPN-Bu** by combining the efficient energy transfer from the **Zr-fcu-BADC-MOF** and its direct harnessing of the singlet and triplet excitons upon X-ray radiation (upper right). Taken and adapted with the permission from ref 1402. Copyright [2022/ Mater] Elsevier under Creative Commons Attribution 4.0 International License https://creativecommons.org/licenses/by/4.0/.

**Figure 238:**
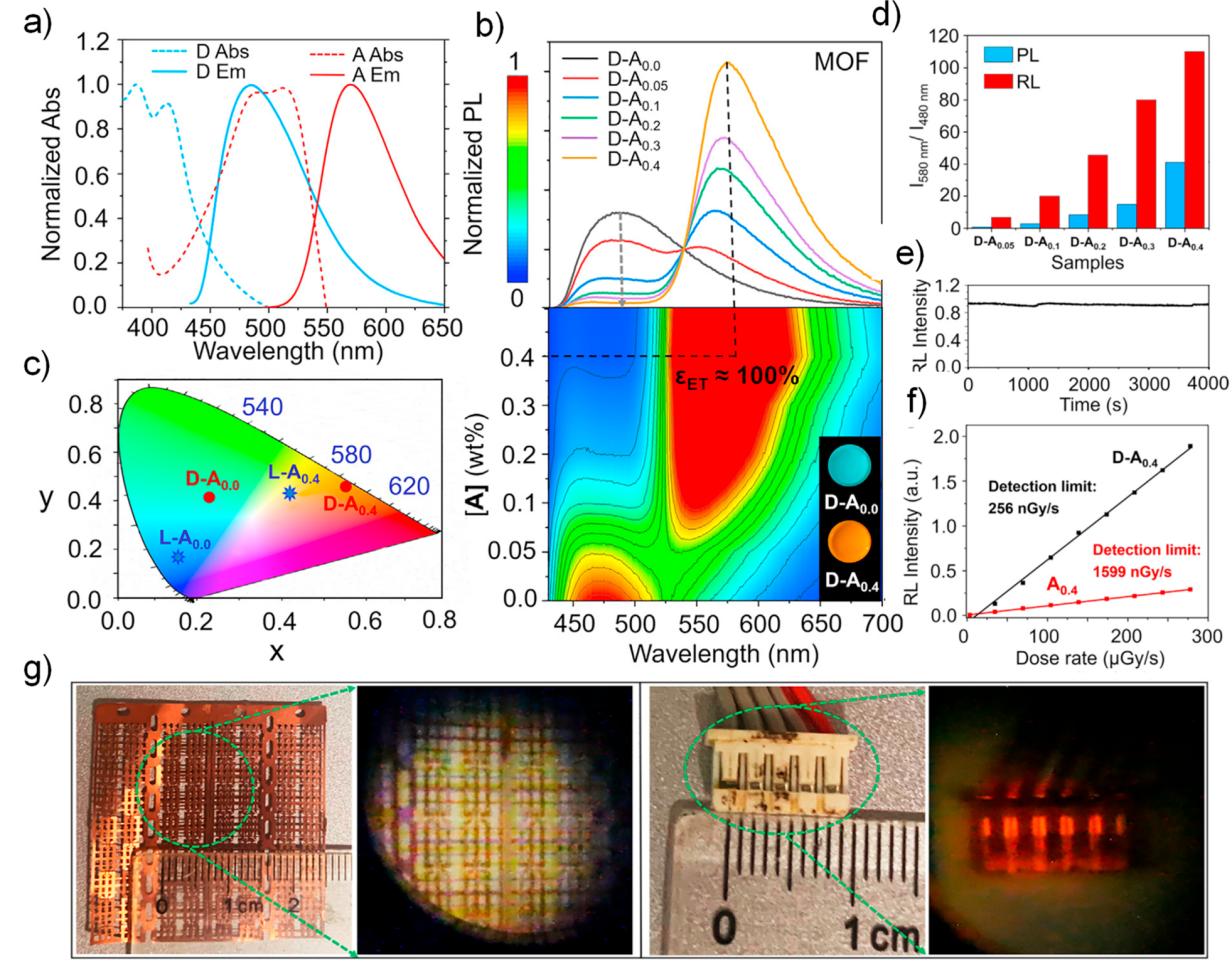
a) Spectral overlap between the emission spectrum of the **Zr-fcu-BADC-MOF** nanoparticles (**D**) and the absorption spectrum of **4CzTPN-Bu** (**A**). b). Emission spectra of the nanocomposite films containing different **D** to **A** ratios (D-A_n_, where n is the wt% of the TADF chromophore in PMMA and the concentration of **D** is 1 wt% in PMMA). Inset shows corresponding photo images of D-A nanocomposite films. c) CIE 1931 coordinates of emission from (**D-A_0.0_**) and (**D-A_0.4_**). d) Ratios of I_580 nm_/I_480 nm_ under the excitation of UV and X-rays. e) RL intensity at 580 nm of the **D-A_0.4_** nanocomposite film under continuous X-ray irradiation at a dose rate of 174 mGy s^–1^. f) Detection limit of the **D-A_0.4_** nanocomposite film (black line) and **A_0.4_** film (red line) g) Bright- and dark-field photographs of a steel framework (left) and electronic component (right) before and after X-ray exposure (dose value: 174 mGy/s). Taken and adapted with permission from ref^1402^. Copyright [2022/Matter] Elsevier under Creative Commons Attribution 4.0 International License https://creativecommons.org/licenses/by/4.0/.

Abraham *et al*. reported polyvinyltoluene (PVT) based cross-linked plastic scintillators for the detection of γ-rays, containing 1 wt% **4CzIPN** or ***t*BuCzDBA** as a TADF dye alongside various amounts (0, 5, 10, 40 wt%) of (caprot­ed)di-(meth­acryl­ate)­bis­muth (CMB) cross-linkable compounds, and either with (4.5 wt%) or without cross-linker divinyl benzene (DVB, Figure 239).^1403^ The TADF dye acts to harvest triplet excitons *via* RISC to achieve 100% luminescence quantum yield, and thus increase the light yield. The plastic scintillator containing **4CzIPN** without CMB emits close to 500 nm and has a τ_d_ of 3.3 μs, while the emission of **4CzIPN** is slightly red-shifted around 515 nm (τ_d_ = 3.0 μs) in the plastic scintillator containing 40 wt% CMB. Similar optical behavior of ***t*BuCzDBA** was observed in the plastic scintillator (λ_PL_ = 550 nm and τ_d_ = 2.9 μs, without CMB), while the τ_d_ decreased slightly to 2.6 μs in scintillator containing 40 wt% CMB. The scintillator without CMB but containing ***t*BuCzDBA** exhibited higher relative light yield (0.25) than **4CzIPN** (0.11). The authors suggested that the higher light yield in ***t*BuCzDBA** could be due to either a higher fraction of horizontal emitting dipoles or reduced internal scattering within the bulk for the **tBuCzDBA** sample. Another reason for the higher light yield could be more efficient energy transfer from the PVT matrix to the **tBuCzDBA** dye. Even though the CMB loading adversely affected the light yield, the cross-linking approach nonetheless improved the mechanical robustness with a uniaxial yield strength up to ≈ 66 MPa for the scintillators loaded with 40% CMB.

**Figure 239:**
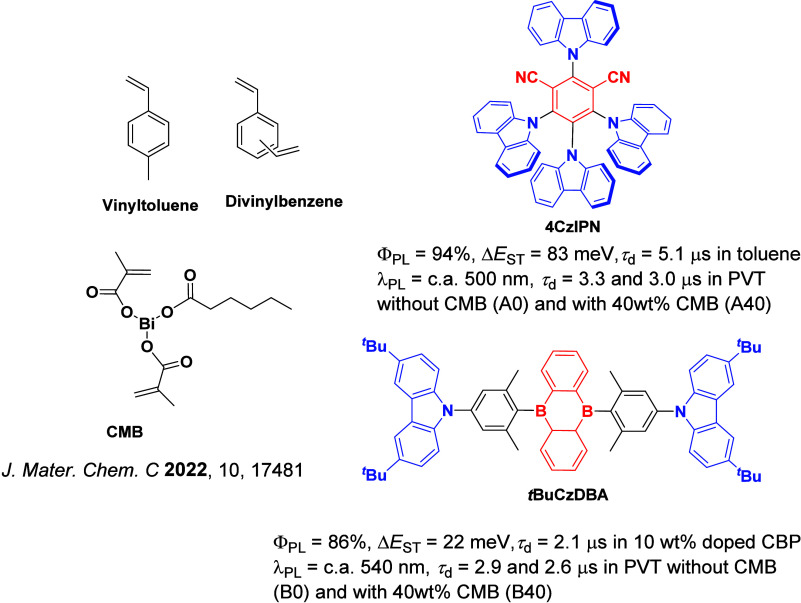
Chemical structures of the components of polyvinyltoluene (PVT) based cross-linked plastic scintillators – vinyltoluene (monomer), divinylbenzene (cross-linker), CMB, **4CzIPN**, and **tBuCzDBA** – and photophysical properties of **4CzIPN** and **tBuCzDBA** (the blue color signifies donor moieties, while the red color signifies acceptor moieties).

### 20.3 Outlook

The sensitivity of the spectral response of TADF materials to temperature and O_2_ make them particularly attractive for sensing applications in comparison to typical fluorescent probes. Simultaneously, many of the reported TADF sensors are dual emissive, with one of the bands insensitive to oxygen and/or temperature and so act as a convenient internal reference for the other band. Aside from the examples highlighted here, we note that the long-lived nature of TADF itself can be exploited to increase signal-to-noise in sensing applications, and for use in time-resolved fluorescence imaging (see Section 21). There are now also multiple reports of TADF materials employed as optical sensors for a number of chemical analytes, as well as for X-ray detection.

Despite the relative infancy of TADF sensors, the examples to date nonetheless demonstrate the potential of this class of materials. Considering that any optical sensing ability arises from changes to photophysical in response to external environment, we predict that there will be additional demonstrations of sensing utility developed in the coming years. These may include solvent polarity and trace concentrations of aqueous hydrocarbons (through CT emission red-shift), viscosity (through impacts on vibronically-coupled RISC), pH (especially in ESIPT TADF materials, see Section 14), and as electrochromic redox sensors. The sensing utility of TADF materials is primarily limited by our understanding of their intricate photophysical mechanisms, with both expected to continue growing and developing over time.

## 21 TADF Bioimaging Agents

### 21.1 Introduction

On the scale of individual cells, most living tissue is both optically transparent and has minimal intrinsic contrast (in refractive index or otherwise) between different cellular components. Bioimaging dyes and stains are therefore a frequently necessary tool for observing cell structures, offering the potential to visualize internal organelles and biological processes optically, and often without damaging the cell.^1404,1405^

While conventional fluorescent emitters are established as contrast agents in bioimaging, issues can arise as a result of the autofluorescence of cells. Autofluorescence is the emission from photoactive materials endogenous to the cell itself, which can mask the desired signal from the contrast bioimaging agent.^1406^ One strategy to overcome this issue is to employ phosphorescent emitters, as by virtue of their long-lived emission they can allow autofluorescence and phosphorescence to be distinguished in the time-domain. However, while phosphorescent metal complexes have the additional complication of potential toxicity,^46,1407–1409^ all-organic TADF materials can potentially also address autofluorescence with their suitably long-lived emission. Similar to their desirability in replacing organometallic complexes in electroluminescent devices, the use of all-organic TADF materials as bioimaging reagents also carries benefits in terms of sustainability, bioavailability, and cost (which dictate accessibility in biomedical contexts). The large Stokes shifts for D-A TADF materials can also potentially allow autofluorescence to be addressed and eliminated in the spectral domain. However, for both phosphorescent and TADF materials, quenching of triplet states and any delayed emission by physiological dissolved oxygen must also be carefully considered.

To date, there is a small but rapidly growing body of work in which organic TADF compounds have been developed for bioimaging applications, including for time-resolved luminescence imaging (TRLI) for living cells. Many TADF compounds can also emit in the red to NIR region, which is especially transparent to living tissue, even in bulk. These wavelengths are therefore advantageous for *in vivo* bio-imaging because of reduced photo-damage to the biological samples, greater deep tissue penetration allowing optical signal to emerge, and minimal interference from background (typically blue) autofluorescence from biomolecules in the living systems. In this section we will discuss recent examples of TADF emitters that have been used as bioimaging agents.

### 21.2 TADF Emitters Capped with Bovine/Human Serum Albumin (BSA/HSA)

One strategy to circumvent the quenching of TADF emission by oxygen is to use either human serum albumin (HSA) or bovine serum albumin (BSA). Both contain tryptophan, which is a chromophoric amino acid that can react with singlet oxygen, preventing the quenching of the triplet excited states and thus the delayed fluorescence of emitters.^1396^ BSA has been used in living-cell imaging experiments to enhance the signal originating from the bioimaging agent and also to help cellular uptake by masking the hydrophobic TADF molecule and rendering the TADF-BSA assembly more hydrophilic,^1410,1411^ increasing their solubility and stability in aqueous media.^1396,1411^ In addition, BSA can also protect the emitters from degradation by cellular enzymes and improve their biological compatibility, making them less toxic to cells. In 2014,^1396,1412^ Xiong *et al*. were the first to propose a TADF emitter, **DCF-MPYM** (Figure 240a), that was used in conjunction with BSA. This adduct was employed as the contrast agent in TRLI of MCF-7 cells, and showed long-lived luminescence (*τ*_PL_ = 22.11 μs in deaerated ethanol) at λ_PL_ of 649 nm with a small Δ*E*_ST_ (0.03 eV) in 5:4 MeOH:​EtOH (v/v).^1396,1410^ The BSA protein provides a hydrophobic cavity and a reductive environment that shields the emitter from oxygen, thus permitting the long-lived delayed emission of **DCF-MPYM** to persist in the cells. TRLI of MCF-7 cancer cells using this contrast agent much stronger red luminescence signals and significantly suppressed background signal in time-resolved imaging mode (Figure 240b), compared to equivalent images obtained in steady-state mode (Figure 240c).

**Figure 240:**
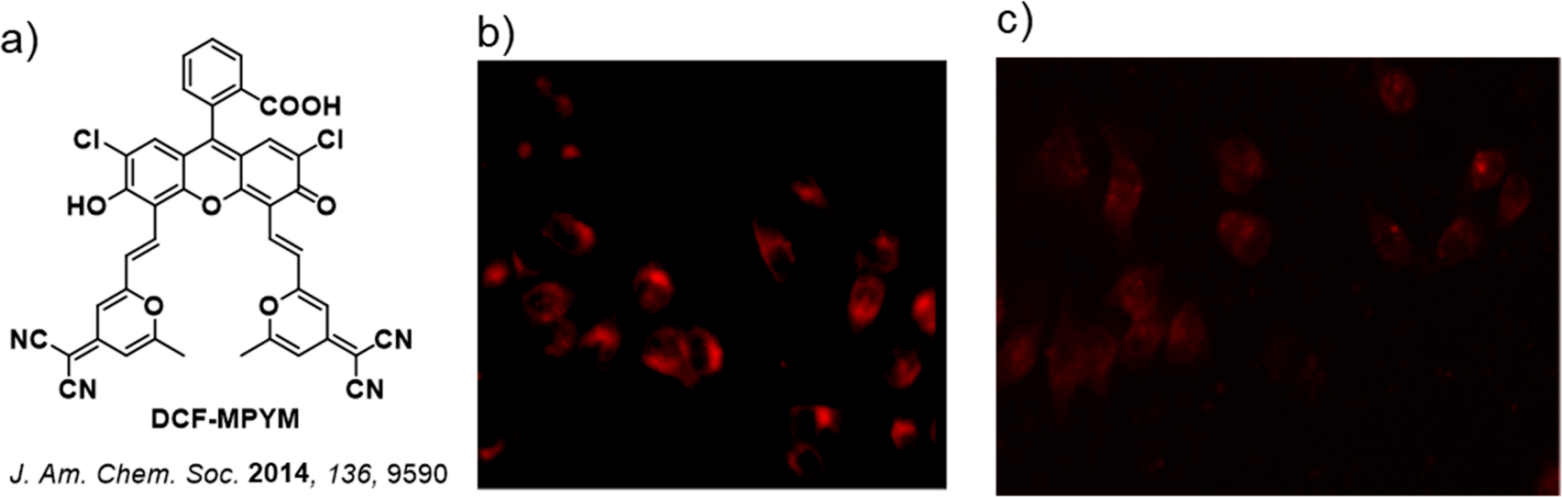
a) Chemical structure of **DCF-MPYM**. b) Time-resolved photoluminescence and c) steady-state photoluminescence (λ_exc_ = 510–560 nm) images of MCF-7 stained with **DCF-MPYM** (20 μM) and BSA (40 μL, 10 mM) at 37 °C. Taken and adapted with permission from ref 1396. Copyright [2014/Journal of the American Chemical Society] American Chemical Society.

The same group later developed two derivatives of **DCF-MPYM** through the introduction of aromatic carbonyl groups, with the goal of enhancing the ISC process to increase the population of triplet excitons and the DF contribution to total emission by augmenting SOC (Figure 241).^1413^ Indeed, derivatives **DCF-MPYM-Ph** and **DCF-MPYM-Th** possess much longer τ_d_ of 31.29 μs and 52.05 μs, respectively, than that of **DCF-MPYM** (*τ*_d_ = 22.11 μs). With the assistance of HSA, these two emitters were also used in the TRLI of MCF-7 cells.^1413^

**Figure 241:**
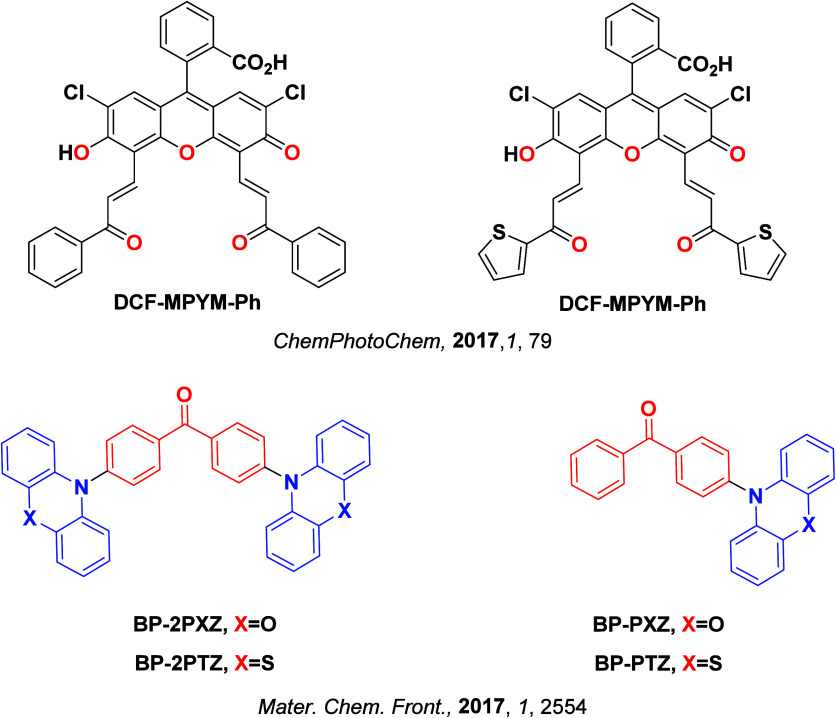
Chemical structures of organic TADF molecules used as imaging agents with the assistance of BSA/HSA (the blue color signifies donor moieties, while the red color signifies acceptor moieties).

**Figure 242:**
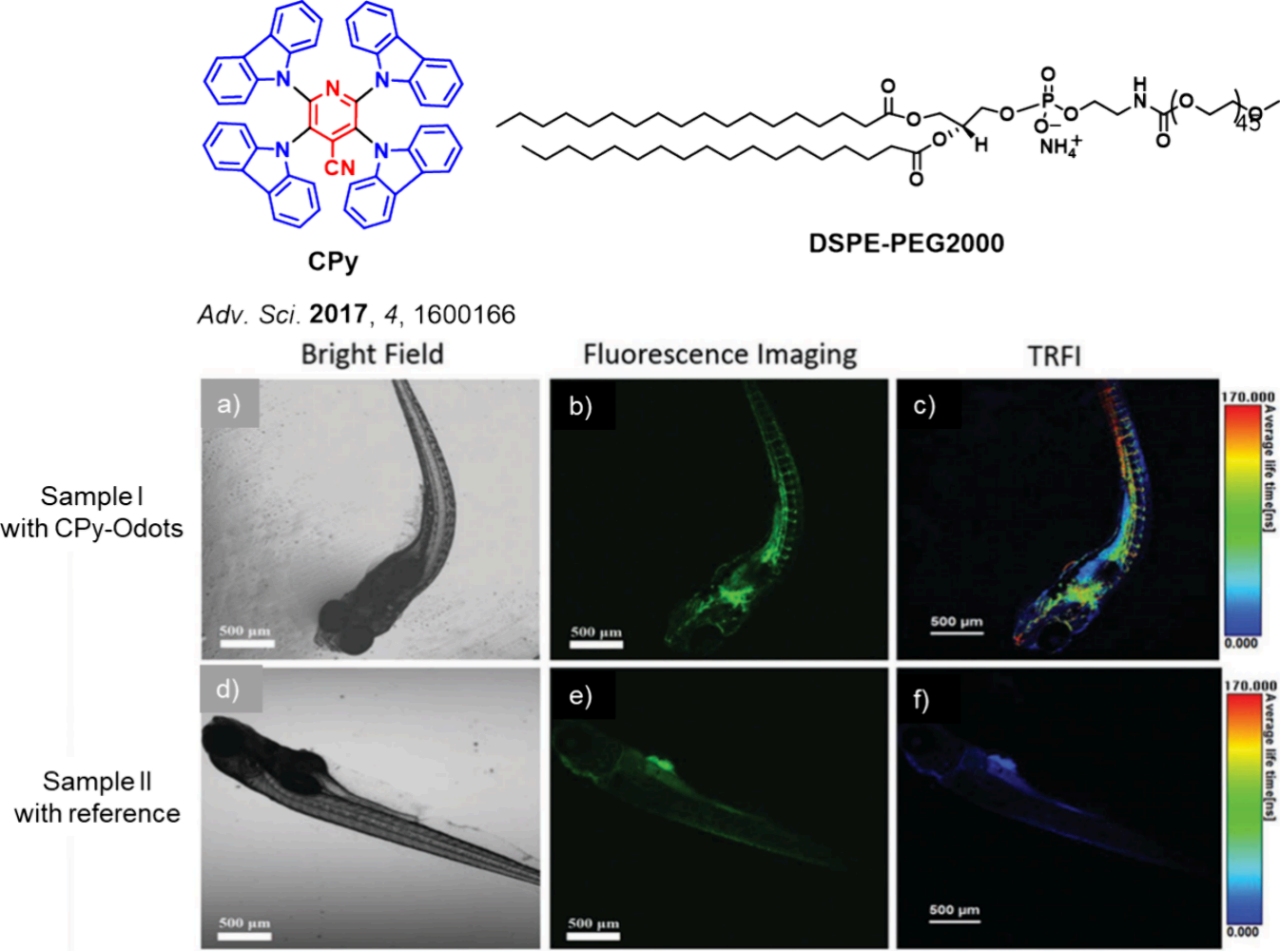
Top: chemical structure of **CPy** and DSPE-PEG2000. Bottom: confocal fluorescence images of zebrafish: a–c) zebrafish injected with **CPy-Odots**; d–f) zebrafish reference non-TADF Odots. Taken and adapted with permission from ref 1422. Copyright [2017/Advanced Science] John Wiley & Sons under Creative Commons Attribution 4.0 International License https://creativecommons.org/licenses/by/4.0/.

Another family of TADF emitters used as contrast agents through encapsulation with BSA include **BP-PXZ**, **BP-2PXZ**, **BP-PTZ**, and **BP-2PTZ** (Figure 241). These compounds reflect typical D-A TADF emitter designs developed for OLED applications, containing benzophenone (**BP**) as the acceptor (A) and **PXZ** or **PTZ** as donor. As documented in Section 13, this motif also confers AIE properties to the molecule, especially active in aqueous environments.^12^ **BP-2PXZ**, **BP-PXZ**, **BP-2PTZ**, and **BP-PTZ** in the neat films possess *τ*_d_ of 0.73, 0.96, 0.66 and 1.36 μs at λ_PL_ of 558, 546, 551 and 544 nm, respectively. After their encapsulation within BSA, the obtained water-soluble nanoparticles demonstrated strong green or yellow luminescence, low cytotoxicity, and good performance in fluorescence lifetime imaging which provided a clear map of intracellular viscosity.^1365,1368^

### 21.3 Organic Dots (Odots)

Organic dots (Odots) have emerged as a class of fluorescent nanoprobes for biological imaging as they are very bright, possess good photostability, do not blink, and are nontoxic.^1414–1416^ Currently, Odots have mainly been used in cell imaging, biosensing, drug and gene delivery, photothermal and photodynamic therapy, and two-photon-excited fluorescence imaging.^1417–1419^ However, these applications largely rely on the fluorescence intensity signals instead of their fluorescence lifetime.^1419–1421^ Odots based on TADF emitters would combine the merits of fluorescent Odots but also feature much longer-lived fluorescence suitable for time-domain microscopy.^1361,1363,1370,1422,1423^ Li *et al*. fabricated CPy-based Odots (**CPy-Odots**) by encapsulating the high-performance TADF emitter CPy^1341^ into DSPE-PEG2000, an amphiphilic and biocompatible polymer that was chosen as the encapsulation matrix due to its ability to encapsulate small, neutral, organic compounds (Figure 242).^1424^ The **CPy-Odots** are water-soluble and bright (Φ_PL_ of 38% in Milli-Q water), with a *τ*_d_ = 9.3 μs under ambient atmosphere. **CPy-Odots** were consequently employed in time-resolved and confocal fluorescence imaging of living Hela cells and in living zebrafish. As shown in Figure 242, by comparing the images captured with fluorescence lifetime imaging microscopy (FLIM), the vivid green-to-red signals of the **CPy-Odots** were easily distinguished from the autofluorescence (bioluminescence) as the latter possesses a τ_PL_ shorter than 3 ns (λ_ex_ = 405 nm). This study demonstrated that **CPy-Odots** can be used as bright microangiography agents for FLIM in living zebrafish.^1422^

In addition to **CPy**, another well-known TADF emitter **4CzIPN** was reported to show two-photon absorption as an Odot in HeLa cells.^1425^ Odots of **4CzIPN** were formed upon encapsulation into PEG-*b*-PPG-*b*-PEG (Figure 243). This Odot material possessed a τ_d_ ≈ 1.47 μs and has good biocompatibility and biodegradability, low toxicity, and shows specificity for uptake into malignant cells that were imaged by confocal fluorescence imaging in living cells.^1425^ Ran and co-workers similarly prepared nanoprobe micelles by coating **Al-Cz** (Figure 243) in glucose-PEG2000-DSPE, which were then used for malignant cell imaging diagnosis.^1426^ The Glucose-PEG2000-DSPE TADF micelles emitted at λ_PL_ ∼ 500 nm and were nontoxic, biocompatible, and even biodegradable. They could be efficiently transported into the cancer cells by an over-expressed glucose transporter on the tumor cell membrane, and then once taken into the HepG2 tumor cells localized in the lysosome.

**Figure 243:**
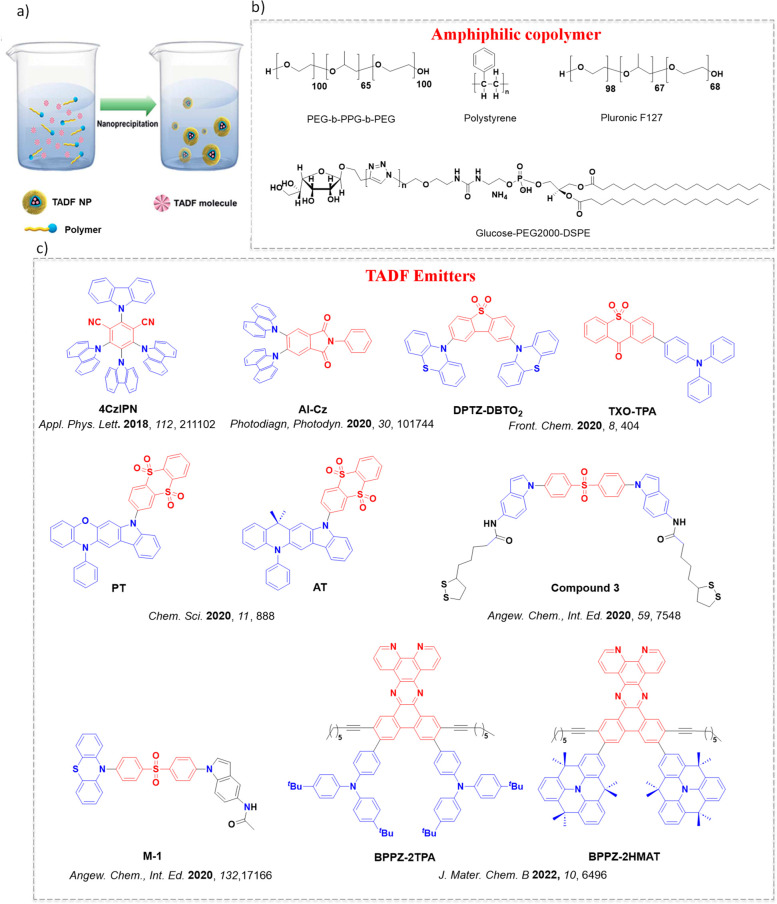
a) Schematic illustration of the nanoprecipitation for nanoparticle preparation, taken and adapted with permission from ref 1426. Copyright [2020/Chemical Science] The Royal Society of Chemistry. b) Chemical structures of amphiphilic copolymer and c) organic TADF molecules used for fluorescence imaging applications (the blue color signifies donor moieties, while the red color signifies acceptor moieties).

Xiao *et al*. prepared two TADF molecules, **PT** and **AT**, containing different electron-donating moieties to demonstrate a rational design of photosensitizers and fluorescence imaging agents, respectively. The proposed TADF emitters exhibit a tailored balance between two-photon singlet oxygen generation and fluorescence emission (Figure 243).^1426,1427^ **PT** possesses both a smaller calculated Δ*E*_ST_ of 0.06 eV and *f* of 0.03 compared to a larger calculated Δ*E*_ST_ of 0.1 eV and an *f* of 0.07 for **AT**. In a mixture of 1:99 THF:water, the Φ_PL_ of **PT** and **AT** were 2.2% and 9.1%, respectively, while in the corresponding neat thin films, the Φ_PL_ of **PT** and **AT** increased to 7.9% and 17%, respectively. In this study, DSPE-PEG2000 was employed to encapsulate **AT** and **PT** to produce nanoparticles (**PT NPs** and **AT NPs**) which improved both the stability and biocompatibility of **PT** and **AT** in aqueous environment. The cell studies further indicated that, in line with their contrasting Δ*E*_ST_ and Φ_PL_ values, the **PT NPs** show much stronger singlet oxygen generation capability and photodynamic therapy (PDT) performance compared to the **AT NPs**, while the **AT NPs** produced a much brighter fluorescence image.^1426^

Besides DSPE-PEG2000, polystyrene has also been used to encapsulate TADF emitters. In one study **DPTZ-DBTO_2_** and **TXO-TPA** (Figure 243) were encapsulated in order to conserve their photophysical properties as nanoparticles in biological media.^29^ While for **DPTZ-DBTO_2_** the effect of encapsulation on its photophysical properties was not significant (e.g., λ_PL_ = 563 nm and λ_PL_-_NP_ = 556 nm), for **TXO-TPA** the emission was markedly blue-shifted when the dye was incorporated in the nanomaterial (e.g., λ_PL_ = 629 nm and λ_PL‑NP_ = 555 nm). Both NPs possessed microsecond-long *τ*_d_ = 2.89 μs for **DPTZ-DBTO_2_ NP** and *τ*_d_ = 9.56 μs for **TXO-TPA**, respectively. The authors found that upon using amino-modified NPs the reagents could be more efficiently internalised with more uniform dispersion inside the cells.

Zhu and co-workers designed an asymmetric donor-acceptor-donor compound that showed dual TADF emission resulting from CT states from each of the phenothiazine and N-(1*H*-indole-5-yl) acetamide donors with the diphenylsulfone acceptor (Figure 243).^1428^ The two emission bands of **M-1** were at λ_PL_ = 420 nm and λ_PL_ = 580 nm, each showing distinct τ_d_ of 5.2 μs and 12.9 μs, respectively with a total Φ_PL_ of 20.1% in THF. Compound **M-1** was encapsuled within the amphiphilic block copolymer Pluronic F-127, and dispersion of **M-1** in the cell culture medium led to an enhanced average *τ*_PL_ of 33 μs and 36 μs, respectively, in the dual-channel luminescence imaging studies [the DAPI (4′,6-di­ami­dino-2-phen­yl­indole) and FITC (*fluorescein isothiocyanate*) channels, dual-channel luminescence imaging here referring to capturing separate images from different spectral bands, usually blue (DAPI) and green (FITC)]. By calibrating the two time-resolved signals, serialized and integrated intracellular local imaging information could also be observed.^1428^ The same group also designed a new TADF emitter based on an indole-derived D-A-D skeleton linked with long α-lipoic alkyl chains (Figure 243). **Compound 3** exhibited blue emission at λ_PL_ = 487 nm with DF in both pure DMF (*τ*_PL_ = 1.4 μs, Φ_PL_ of 35.3%) and DMF:H_2_O 1:99 mixtures (*τ*_PL_ = 3.6 μs, Φ_PL_ of 30.8%). Both the aggregates of **Compound 3** and NPs formed by encapsulation into Pluronic F-127 were investigated as imaging reagents by TRLI, which demonstrated that the dual-emission was conserved in the cells.^1429^

Moving away from emitters in non-doped aggregated states, Tsuchiya *et al*. recently reported an alternative strategy where the Odot is composed of an emitter (**4CzIPN**), a host (mCP) and a surfactant (DSPE-PEG2k).^1361,1430^ This design mitigates possible aggregation-caused quenching (ACQ) by effectively diluting the emitter within the micelle in an analogous manner to the emissive layers in OLEDs. These Odots showed near unity Φ_PL_ of 94% and an associated *τ*_d_ of 3.1 μs under air-free conditions in water. The conditions and ratios involved in the preparation of the ODots affected the properties, where oxygen-free processing gave ODots with higher Φ_PL_ and greater photostability. Further, upon using a host to surfactant ratio of 10:1, the best photostability was achieved, with photo-degradation causing emission to drop to 50% of the initial intensity after 360 mins, which was superior to a reference blue quantum dot sample. Once the Odot is formed, the photophysics was observed to be insensitive to external oxygen. HEK293 cell imaging was demonstrated and the Odots remained stable for at least 7 days after uptake into the cells (Figure 244).^1361^

**Figure 244:**
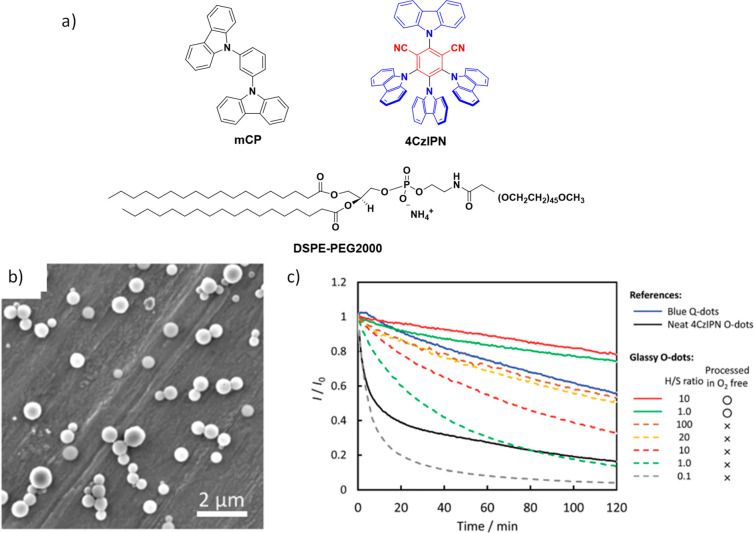
a) Chemical structures of materials for Odot preparation. b) SEM images of 6 wt% **4CzIPN**/mCP glassy Odots. c) Photo-degradation properties of 6 wt% **4CzIPN**/mCP glassy Odots under various preparation conditions: neat **4CzIPN** Odots and blue Qdots in water (air saturated); the monitored emission wavelengths were λ_PL_ = 515, 548, and 450 nm, respectively; λ_exc_ = 300–400 nm; excitation light intensity 5 mWcm^–2^. Taken and adapted with permission from ref 1361. Copyright [2019/Chemical Communications] The Royal Society of Chemistry.

Using a similar methodology as Tsuchiya and co-workers, Hudson and co-workers developed two TADF emitters, **BPPZ-2TPA** and **BPPZ-2HMAT** (Figure 243). Based on a rigid and strongly electron-withdrawing diben­zo[a,c]di­pyri­do­[3,2-h:20-30-j]­phena­zine-12-yl (BPPZ) motif, they demonstrated two approaches for the encapsulation of these emitters to yield water-dispersible nanoparticles suitable for TRLI.^1431^ Although Odots prepared with the undoped emitters did not show long-lived emission, their prompt fluorescence lifetimes were long, ranging from 8.5 to 11.9 ns in aqueous solution. Glassy organic nanoparticles (g-Odots) were also prepared with 5 wt% doped emitters in mCP surrounded by the amphiphilic polymer DSPE-PEG2000. The g-Odots by contrast showed long-lived emission in aerated aqueous solutions, with τ_PL_ of 123 ms for **TPA g-Odots**, and 85 ms for **HMAT g-Odots**. Both approaches yielded nanoparticles suitable for imaging of human cervical (HeLa) and liver (HepG2) cancer cell lines.

Hudson and co-workers also explored other g-Odots based on heptazine-type TADF emitters (Figure 245).^1430^ In this study three s-heptazine TADF materials, **HAP-3Cz**, **HAP-3MeOTPA**, and **HAP-3MeOCz**, showed green to deep-red emission (λ_PL_ = 525–664 nm) and variable Φ_PL_ (Φ_PL_ = 73% for 2 wt% **HAP-3Cz**, 33% for 2 wt% **HAP-3MeOTPA**, and 7% for 2 wt% **HAP-3MeOCz** in *poly*-mCP). For **HAP-3MeOCz** and **HAP-3Cz**, the g-Odots synthesized in air showed both shorter emission lifetimes and substantially lower Φ_PL_ values (30–41%) relative to those synthesized under nitrogen (Φ_PL_ = 99–100%). By contrast, unity Φ_PL_ was observed for the **HAP-3MeOTPA** g-Odots for samples synthesized both under air and under nitrogen. Similar delayed fluorescence lifetimes were observed for the **HAP-3MeOTPA** (50 μs under air, 52 μs under nitrogen) and **HAP-3Cz** g-Odots (1.1 μs under air, 1.2 μs under nitrogen), but no delayed fluorescence was observed for **HAP-3MeOCz** g-Odots. These g-Odots were then used as biological imaging probes of immortalized human kidney cancer (HEK293) cells, and both for single- and multi-photon excited microscopy coupled with time-gated luminescence measurements (Figure 245). This work therefore not only described new routes to efficient heptazine-based TADF materials, but also demonstrated their potential as nanoparticle-based bioimaging probes.

**Figure 245:**
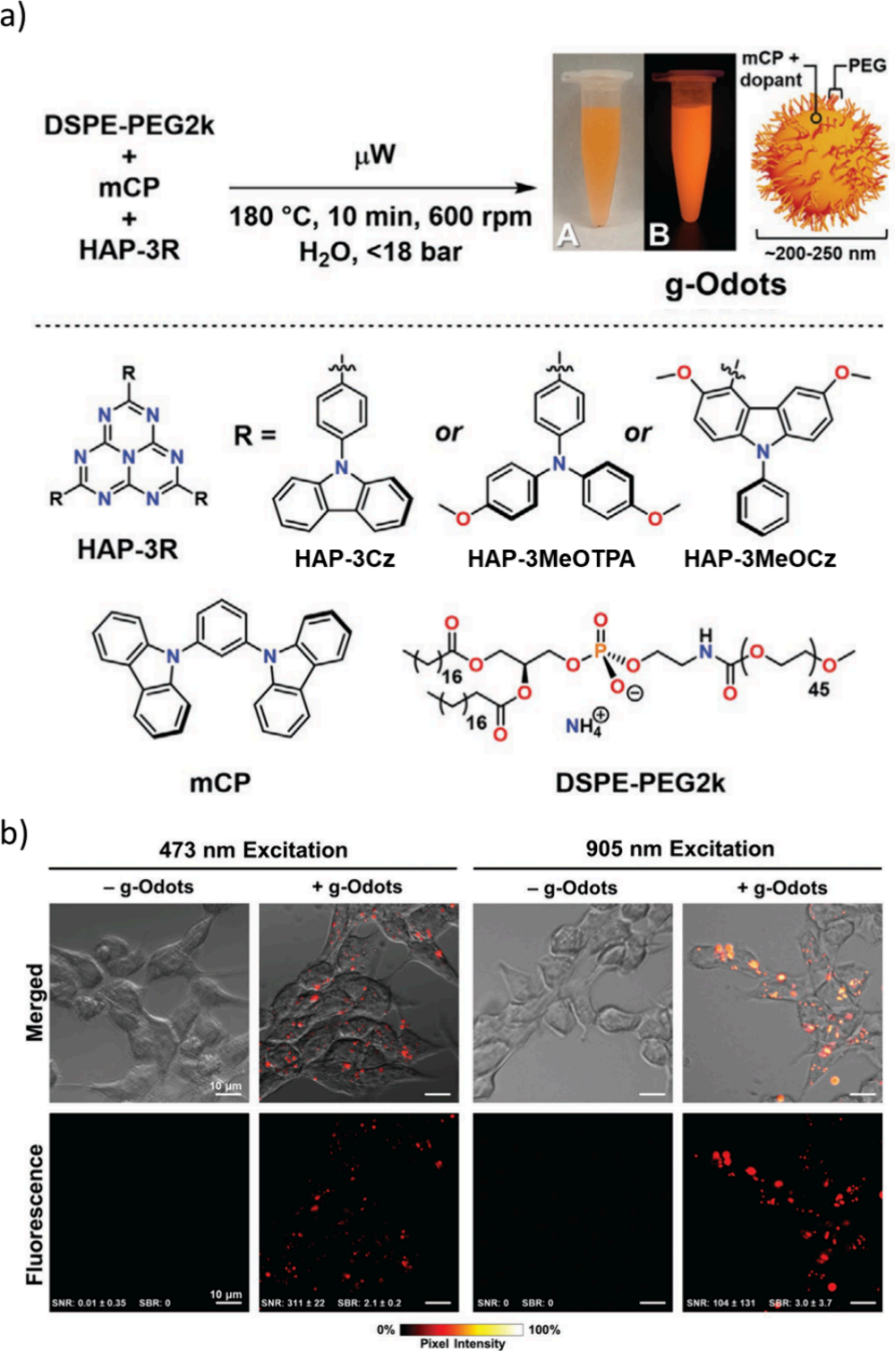
a) Synthesis of g-Odots with examples of isolated nanoparticle suspensions shown for dopant **HAP-3MeOTPA**, where A is photographed under ambient room lighting and B under 365 nm irradiation. μW = microwave irradiation; ))) = ultrasonication. b) Single-photon excitation (λ_exc_ = 473 nm, λ_em_ = 485–545 nm) and multi-photon excitation (λ_exc_ = 905 nm, λ_em_ = 575–630 nm) of HEK293 cells incubated with **HAP-3MeOTPA** g-Odots (+g-Odots) for 24 h. Corresponding control samples (−g-Odots) are shown as well. SNR and SBR calculated with N = 8 cells for 473 nm excitation samples and N = 22 cells for 905 nm excitation samples. Taken and adapted with permission from ref 1437. Copyright [2022/Advanced Functional Materials] John Wiley & Sons. Taken and adapted with permission from ref 1430. Copyright [2022/Advanced Functional Materials] John Wiley & Sons.

Hudson and co-workers also reported two boron difluoride curcuminoid (BFC)-based polymers, **CzBN-co-DtaB** and **CzBN-co-HmatB** (Figure 246a), exhibiting TADF in the deep red/NIR region with λ_PL_ of 694 nm and 717 nm in toluene, respectively. **CzBN-co-DtaB** and **CzBN-co-HmatB** showed τ_d_ of 4.7 and 5.2 μs and Φ_PL_ of 18% and 12%, respectively, in the solid state. Both polymers were incorporated into water-soluble Odots using the amphiphilic polymer poly(styrene-co-maleic anhydride) [PSMA; PS11-co-MA6)] that had an average diameter of 65 nm and 58 nm for the Odots with **CzBN-co-DtaB** and **CzBN-co-HmatB**, respectively. There was only a small red-shift in the emission noted for the Odots compared to the neat films (λ_PL_=730 and 752 nm and 731 and 764 nm in the neat film and in the Odots for **CzBN-co-DtaB** and **CzBN-co-HmatB**, respectively), while the delayed lifetimes were considerably shortened compared to those in the solid state, with τ_d_ of 0.86 μs and 0.95 μs, respectively. These Odots were used in specific extracellular immunolabeling experiments with human SK-BR3 cells and showed nonspecific binding.^1432^ Using a similar experimental design strategy, Hsu *et al*. prepared serval types of NIR-II emissive Odots using polymer TADF emitters, with a **DMAC-TRZ** derivative as a TADF monomer and 3-alkoxy-substituted thiophene as conjugated linker, encapsulated within amphiphilic lipids (Figure 246b). These Odots exhibited λ_PL_ of 1064–1100 nm and Φ_PL_ of 0.40–1.58% in aqueous solution, a significant departure from the typical properties of the **DMAC-TRZ** monomer. Although no delayed fluorescence was detected for these Odots, they were nonetheless used successfully in *in vivo* whole-body vascular imaging and 3D bond mapping.^1433^

**Figure 246:**
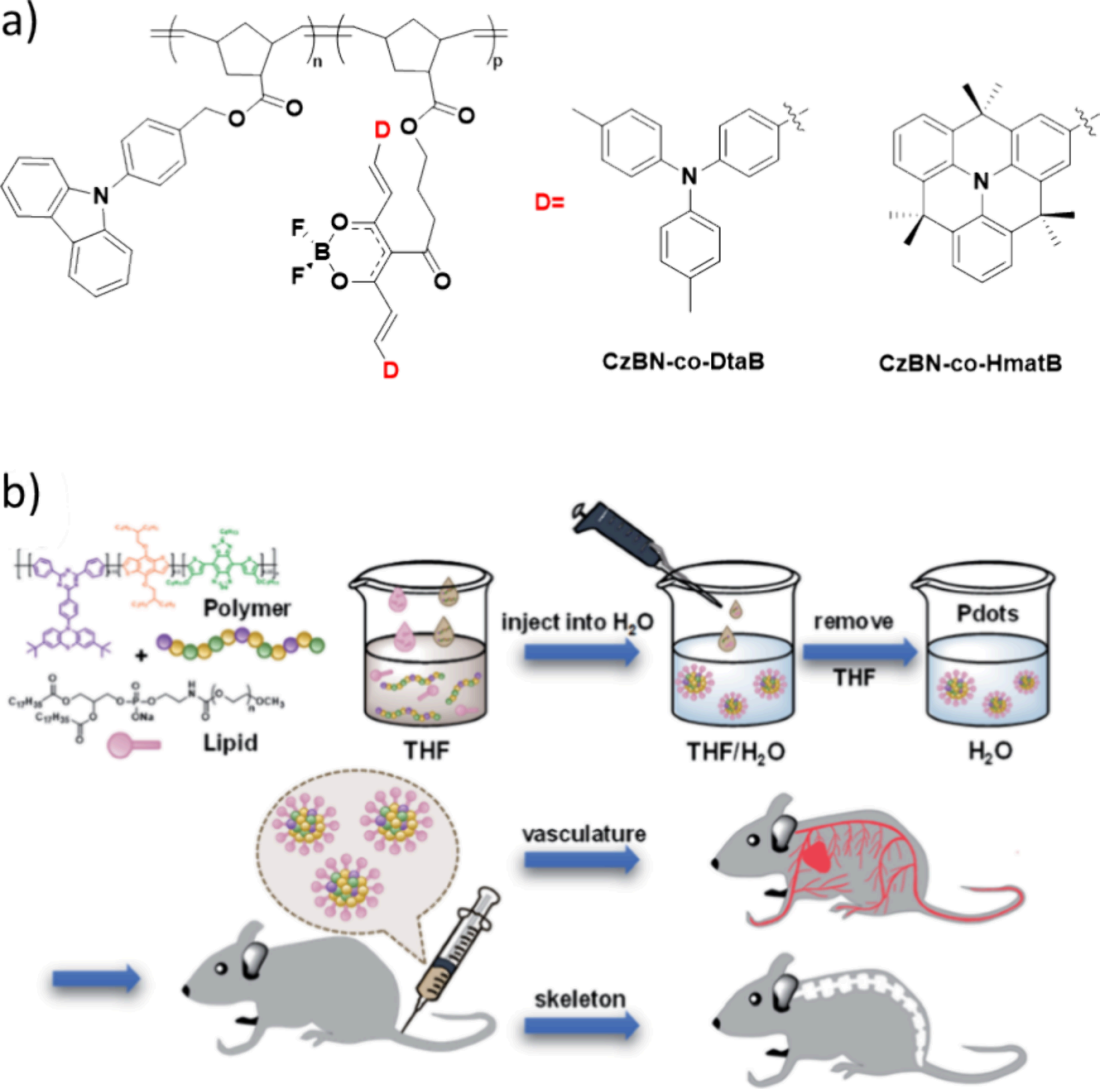
a) Chemical structures of **CzBN-co-DtaB** and **CzBN-co-HmatB**.^1432^ b) Schematic diagram representing the preparation of lipid-encapsulated Odots for *in vivo* vascular and bone imaging. Taken and adapted with permission from ref 1433. Copyright [2022/Chemical Science] Royal Society of Chemistry.

Besides using amphiphilic molecules or polymers to encapsulate luminophores, amphiphilic peptides have also been used as a delivery vector in the construction of NPs containing TADF emitters. Zhu *et al*. reported the use of the amphiphilic cell-penetrating peptide (CPP), [F_6_G_6_(rR)_3_R_2_] (Figure 247), to transport hydrophobic fluorophores across cellular barriers. Three known TADF molecules, **4CzIPN**, **NAI-DPAC**, and **BTZ-DMAC**, were incorporated in well-dispersed nanoparticles (NPs) employing CPP in aqueous solution. The CPP-functionalized NPs of **4CzIPN**, **NAI-DPAC**, and **BTZ-DMAC** showed much lower Φ_PL_ of 12%, 2.5% and 0.8% in aqueous solution at λ_PL_ of 555, 607, and 657 nm, respectively, compared to that observed for the emitters in dilute toluene or doped thin films [(**4CzIPN**: λ_PL_ = 507 nm, Φ_PL_ = 94%),^1432^ (**NAI-DPAC**: λ_PL_ = 570 nm, Φ_PL_ = 94% in 6 wt% doped into mCPCN film),^1433^ [(**BTZ-DMAC**: λ_PL_ = 638 nm, Φ_PL_ = 56% in 3 wt% doped CBP film)^31^]. These three NPs still maintained long-lived luminescence with *τ*_d_ of 1.8, 6.1 and 31.0 *μ*s for the NPs based on **4CzIPN**, **NAI-DPAC**, and **BTZ-DMAC**, respectively. The low cytotoxicity and high cytomembrane permeability of the NPs enabled them to be exploitated for TRLI of living cells.^1370^ These findings expanded the applications of cell-penetrating peptides for delivery of molecules and NPs using only noncovalent interactions.

**Figure 247:**
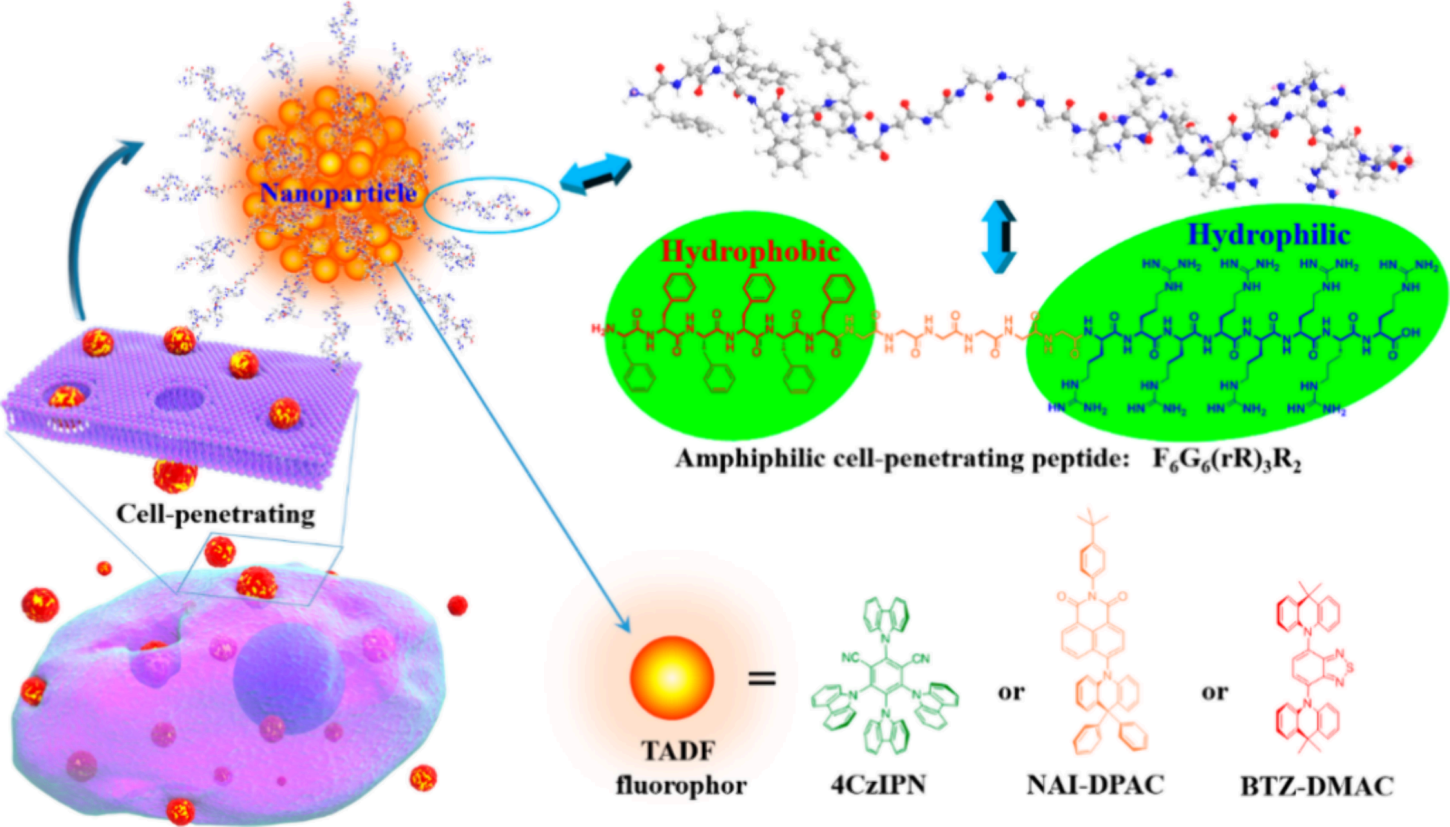
Schematic illustration showing the cell-penetrating NPs assembled from the amphiphilic peptide [**F6G6(rR)3R2**] and TADF molecules **4CzIPN**, **NAI-DPAC**, or **BTZ-DMAC**. Taken and adapted with permission from ref 1370. Copyright [2018/Journal of the American Chemical Society] American Chemical Society.

### 21.4 Silica Nanoparticles

Silica-based nanoparticles (SiNPs) have been extensively used as delivery vectors of conventional fluorescent dyes in optical imaging and sensing applications.^1434–1436^ Avó *et al*. described a method for producing TADF emitter-doped SiNPs that conserve their delayed luminescence in aqueous media. SiNPs (Figure 248a) were prepared using a modified Stöber process from tetraethoxysilane and Compound **3** in water.^1437^ The SiNPs emitted at ca. 585 nm and with a *τ*_d_ of 1.20 ms and a Φ_PL_ of 6% in H_2_O. To address the low Φ_PL_ of the SiNPs, a modified silica source bearing small PEG chains was prepared. The Φ_PL_ of PEG-SiNPs was higher at 20% and these SiNPs possess a longer *τ*_d_ of 1.25 ms, but with an accompanied red-shift in the λ_PL_ to 610 nm. The TADF PEG-SiNPs were effectively internalized by human cells, even at low incubation concentration, localizing primarily in the cytosol and enabling fluorescence microscopy imaging at low dye concentrations.^1438^ Mo *et al*. encapsulated fluorine and nitrogen co-doped carbon dots (FCDs, NCDs) within amorphous silica using a sol–gel method to obtained TADF materials in aqueous solution (**F, NCDs@SiO_2_**).^1439^ The presence of a hydrogen bond network between the CDs and the amorphous silica contributed to reducing non-radiative transitions and producing a long-lived afterglow. The **F, NCDs@SiO_2_** had a Δ*E*_ST_ of 0.32 eV, a high Φ_PL_ of 58.8%, and a *τ*_d_ = 0.48 s. This versatile material was used separately in optical information encryption, temperature monitoring, and TRLI studies.

**Figure 248:**
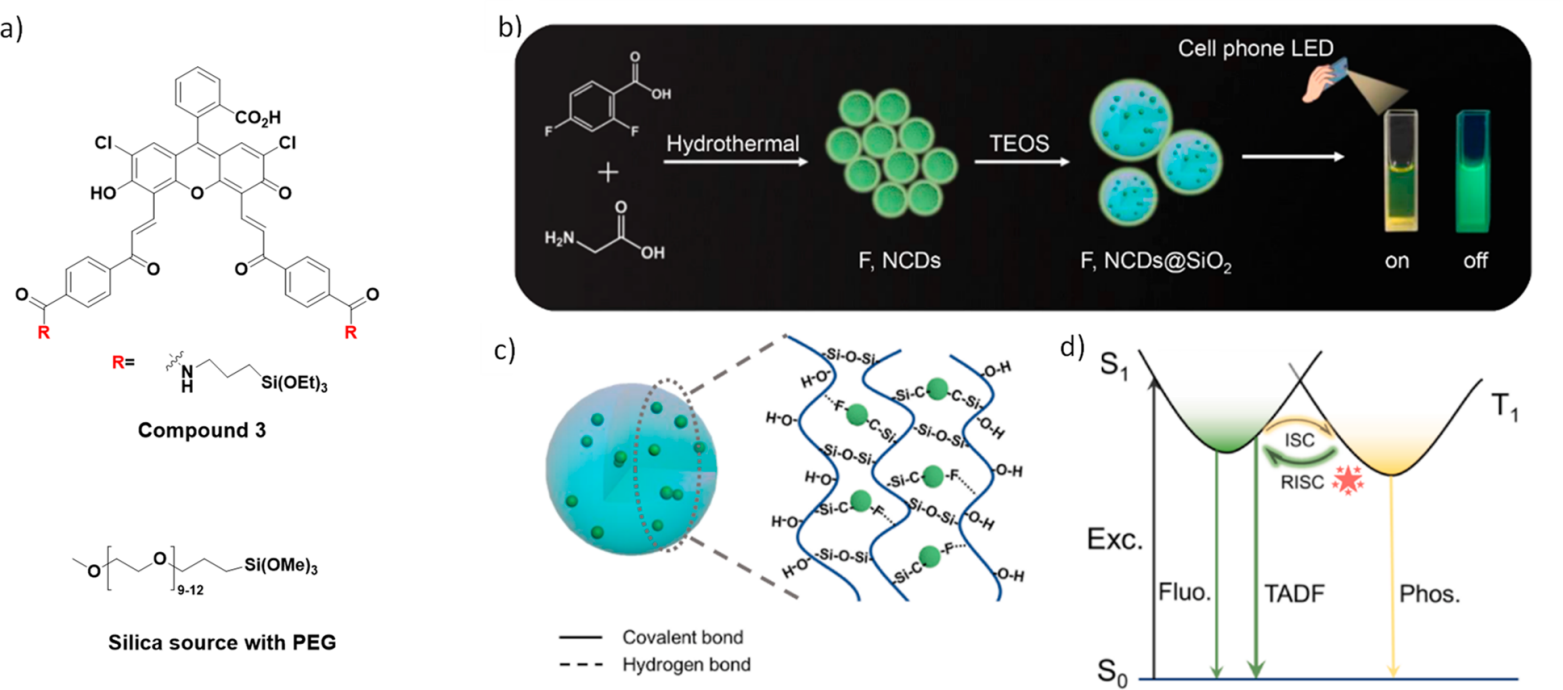
a) Chemical structures of materials for Odot preparation.^1437^ b) Schematic illustration of the preparation procedure for **F, NCDs@SiO_2_** and photographs of **F, NCDs@SiO_2_** in aqueous solution after LED excitation. c) Schematic illustration of the possible structural formation. d) Delayed fluorescence mechanism of **F, NCDs@SiO_2_**. Taken and adapted with permission from ref 1439. Copyright [2021/Chemical Engineering Journal] Journal of the American Chemical Society] Elsevier.

### 21.5 Self-Assembled Nanoparticles

Small-molecule fluorescent organic nanoprobes (FONs) have emerged as promising competitors to inorganic semiconductor quantum dots and fluorescent polymer dots in terms of their wide structural variability, low toxicity, and good biodegradability.^1417,1440–1442^ Self-assembled water-dispersible TADF nanostructures based on three known TADF emitters (**2CzPN**, **4CzIPN**, and **4CzIPN-Ph**) were reported by Lee *et al*. that relied on the limited water solubility of these compounds to form the nanostructures (Figure 249).^39^ The FONs were made by self-assembly of each of these three TADF emitters into water dispersible NPs/nanorods (NRs), with sizes ranging from 80 to 200 nm. Under nitrogen environment the reference λ_PL_ (and Φ_PL_ and τ_d_) of **2CzPN**, **4CzIPN**, and **4CzIPN-Ph** in toluene solutions are λ_PL_ = 473 nm (Φ_PL_ = 21.5%; τ_d_ = 166 μs), 507 nm (Φ_PL_ = 33.5%; τ_d_ = 5.1 μs), and 577 nm (Φ_PL_ = 6.6% τ_d_ = 1.1 μs), respectively. All three FONs showed a slight red-shift in their λ_PL_ compared to those in toluene (λ_PL_ = 503 nm for **2CzPN NRs**, λ_PL_ = 518 nm for **4CzIPN NPs** and λ_PL_ = 588 nm for **4CzTPN-Ph NPs**) coupled with a slight decrease of their respective Φ_PL_ (Φ_PL_ = 19.4% for **2CzPN NRs**, Φ_PL_ = 11.9% for **4CzIPN NPs** and Φ_PL_ = 3.6% for **4CzTPN-Ph NPs**). In order to evaluate the imaging capabilities of the three FONs, one- and two-photon fluorescence images were obtained in an A549 cell using fluorescence microscopy and laser scanning confocal fluorescence, respectively. Figure 249b shows the strong cytoplasmic blue, green, and red fluorescence signals from the **2CzPN**, **4CzIPN**, and **4CzTPN-Ph** nanoprobes, respectively. Two-photon fluorescence imaging for FONs showed greater cytoplasmic details and no fluorescence signal from the nucleus, indicating that the FONs do not penetrate into the cell nucleus. These results suggest that self-assembled nanostructures of carbazole-containing TADF emitters are also promising high-performance fluorescence probes for bioimaging.^1443^

**Figure 249:**
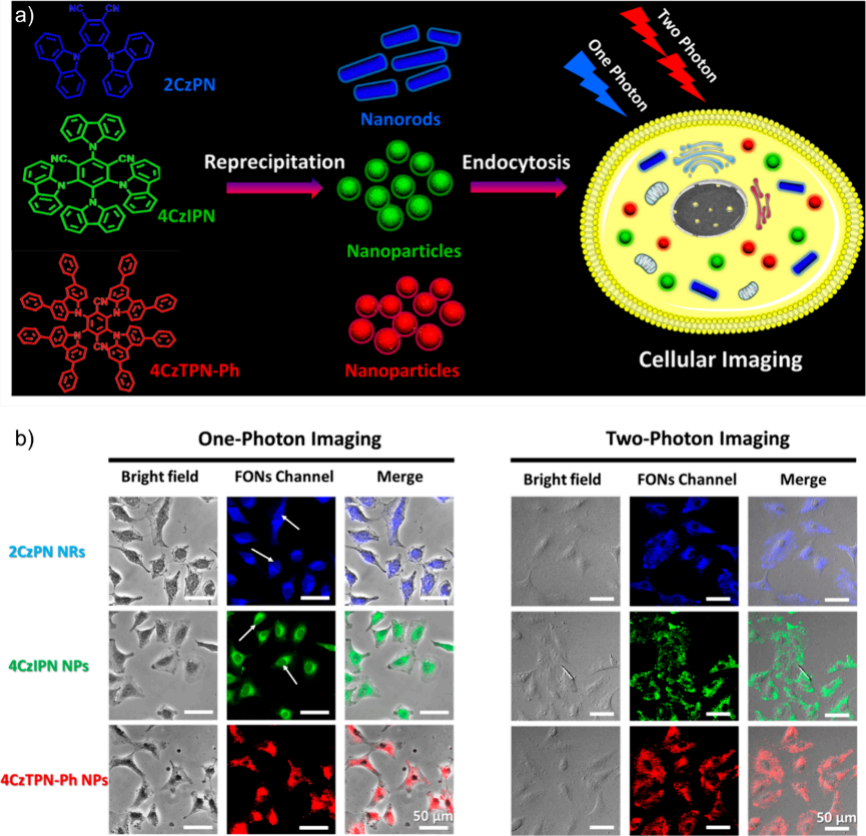
a) Schematic illustration of the preparation of blue/green/red FONs by a reprecipitation method for one- and two-photon cellular imaging. b) Cellular imaging and localization of the three FONs, monitored with a fluorescence microscope (one-photon λ_exc_: 380–420 nm) and a laser scanning confocal fluorescence microscope (two-photon λ_exc_: 800 nm) in an A549 cell (final concentration: 8 μg/mL): left column, bright-field channel; middle column, FON channel; right column, overlay of the bright and FON images. The scale bar is 50 μm. Taken and adapted with permission from ref 1443. Copyright [2016/Journal of the American Chemical Society] American Chemical Society.

Another reported self-assembled TADF amphiphilic monomer (**AI-Cz-AM**) is based on the coupling of the lipophilic aromatic imide-based TADF emitter (**AI-Cz**) with a hydrophilic chain containing a positively charged ammonium terminus.^1444^ Its amphiphilic nature allowed this TADF monomer to spontaneously form a water-soluble and biocompatible nanoprobe, **AI-Cz-NP** (Figure 250). The λ_PL_ of **AI-Cz-AM** and **AI-Cz-NP** were nearly identical at 517 and 514 nm, and the two materials had moderately small and similar Δ*E*_ST_ values of 0.10 and 0.12 eV although with very low Φ_PL_ of 1.36% and 0.94%, respectively. Interestingly, the τ_d_ increased from 6.08 μs for **AI-Cz-AM** in degassed THF to 10.68 μs for **AI-Cz-NP** in oxygenic aqueous solution, indicating significant resistance to ambient oxygen quenching. The latter material was subsequently used in FLIM studies of HepG2cells where long-lived fluorescence signals lasting about 15 ms were detected. This study illustrated how a self-assembly strategy could be used to effectively eliminate emission quenching by oxygen in living cells, without the need for either a polymerization step or biooligomer encapsulation.

**Figure 250:**
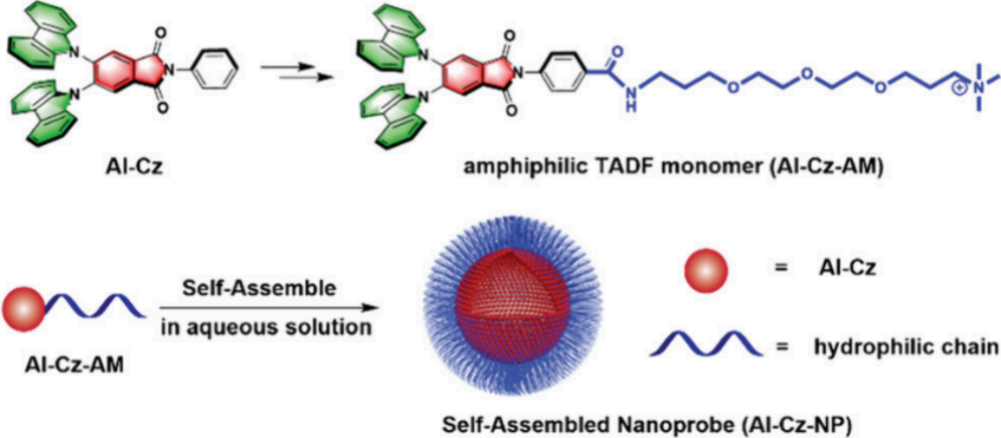
Chemical structure of the amphiphilic TADF monomer (**AI-Cz-AM**) based on **AI-Cz** and the design of the single component self-assembled TADF nanoprobe **AI-Cz-NP**. Taken and adapted with permission from ref 1445. Copyright [2020/Chemical Communications] The Royal Society of Chemistry.

**Figure 251:**
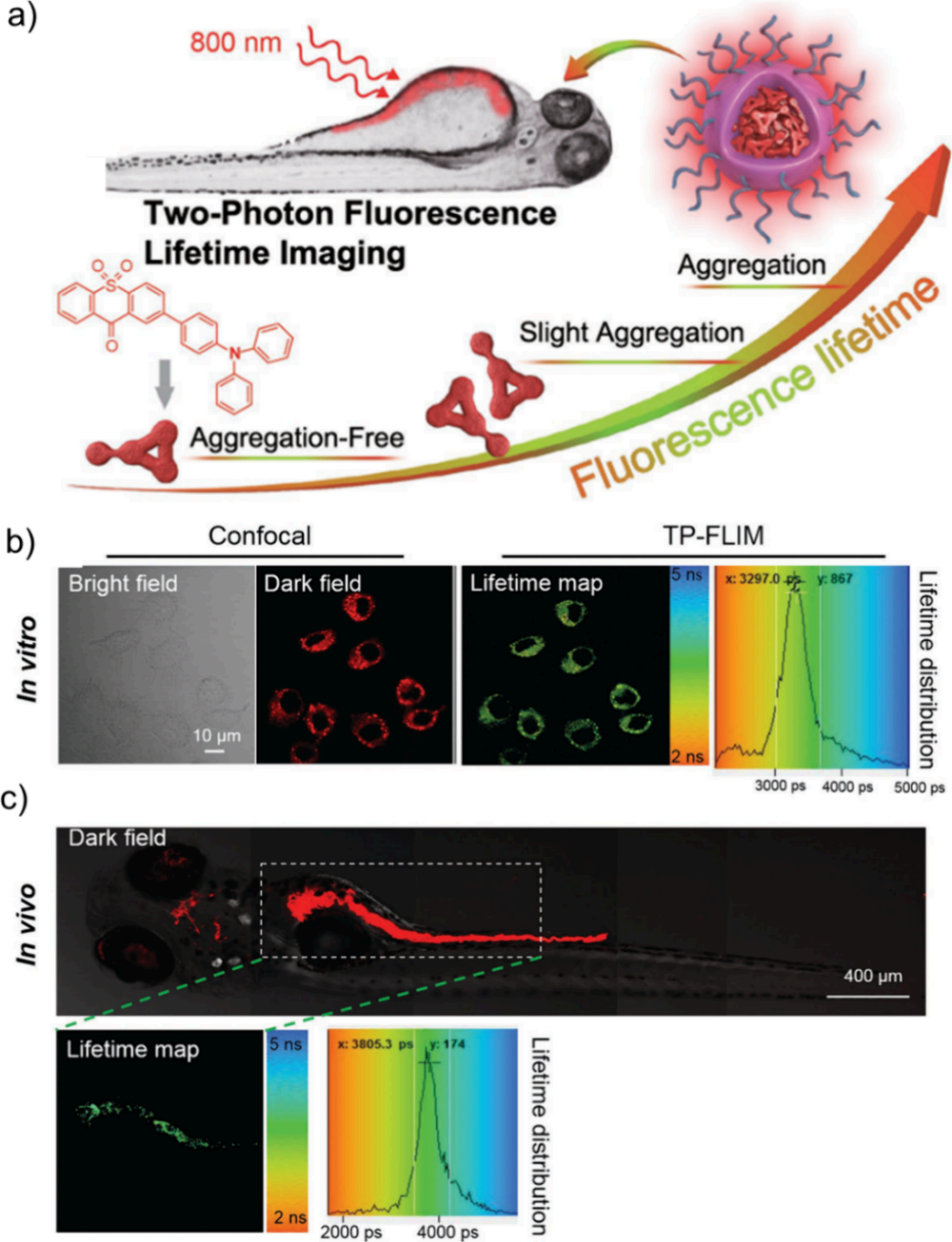
a) Schematic illustration for maximizing aggregation of organic fluorophores to prolong fluorescence lifetime for two-photon FLIM. b) *In vitro* two-photon confocal fluorescence imaging and two-photon FLIM of HepG2 cells stained with **TXO NPs** (10 μg mL^–1^) after 2 h incubation. Using λ_exc_ = 760 nm the fluorescence was recorded between 600–650 nm. c) *In vivo* two-photon confocal fluorescence imaging and two-photon FLIM of zebrafish stained with **TXO NPs** (10 μg mL^–1^) after 4 h incubation. The λ_exc_ = 760 nm, and fluorescence emissions were recorded 600–650 nm. Taken and adapted with permission from ref 1447. Copyright [2018/Advanced Healthcare Materials] Wiley & Sons.

### 21.6 Aggregation-Induced Delayed Fluorescence

One strategy to bypass the oxygen sensitivity of delayed fluorescence in TADF emitters is to use aggregated states. The quenching of emission by oxygen can be suppressed due to its limited ability to make physical contact with the emitter in the aggregated state, as demonstrated in some of the previous examples (Figure 243 and Figure 244).^1446^ As noted in Section 13, ACQ often takes place in TADF emitters in their aggregated states though, which would be detrimental for bioimaging applications. Therefore, emitters that enjoy AIE instead of suffering from ACQ (Figure 251a) are viewed as particularly advantageous. For example, the known AIDF emitter **TXO** was encapsulated in the amphiphilic polymer PEG-*b*-PPG-*b*-PEG.^1447^ As shown in Figure 251b, **TXO NPs** can readily enter the cytoplasm and exhibit strong red emission by two-photon confocal fluorescence imaging. The two-photon FLIM of **TXO NPs** revealed localization in the cytoplasm, where the lifetime of the **TXO NPs** was distributed over a range from ∼2.8 to 3.8 ns. Furthermore, **TXO NPs** were used for *in vivo* two-photon FLIM of living zebrafish.

Qi *et al*. reported the use of three AIDF emitters, **PXZ-NI**, **PTZ-NI** and lysosome-targeting **Lyso-PXZ-NI** (Figure 252), each based on a 1,8-naphthalimide (NI) acceptor.^1448^ The τ_d_ of the 10 wt% PMMA films of **PXZ-NI**, **PTZ-NI**, and **Lyso-PXZ-NI** were 1.0, 1.7, and 1.3 μs, respectively. In aqueous solutions that produced the aggregated form, all three TADF materials demonstrated markedly enhanced delayed fluorescence when concentrations were increased. Subsequently, confocal fluorescence and lifetime imaging studies were performed using laser scanning microscopy and time-resolved fluorescence microscopy. The confocal fluorescence and lifetime images of HeLa cells after incubation with **PXZ-NI**, **PTZ-NI**, and **Lyso-PXZ-NI** for 2 h were captured and exhibited not only strong red fluorescence signals but also long fluorescence lifetime signals in the HeLa cells.^1449^ In another report, Xu and co-workers presented two phosphine oxide-decorated TADF molecules, **CzPOTCF** and **tBCzPOTCF** (Figure 252).^1439^ **CzPOTCF** and **tBCzPOTCF** in neat films exhibited red emission with λ_PL_ = 634 and 647 nm, small Δ*E*_ST_ values of 0.05 and 0.07 eV, moderate Φ_PL_ of 24.5% and 32.7%, and *τ*_d_ of 8.95 and 8.69 μs, respectively. Steady-state and time-resolved luminescence imaging of HeLa cells was demonstrated using these emitters in their aggregated form. The delayed lifetimes were slightly shortened compared to the neat films at *τ*_d_ = 6.69 and 7.41 μs for **CzPOTCF** and **tBCzPOTCF**, respectively, yet nonetheless leading to high signal-to-noise ratios in the microscopy applications.^1450^

**Figure 252:**
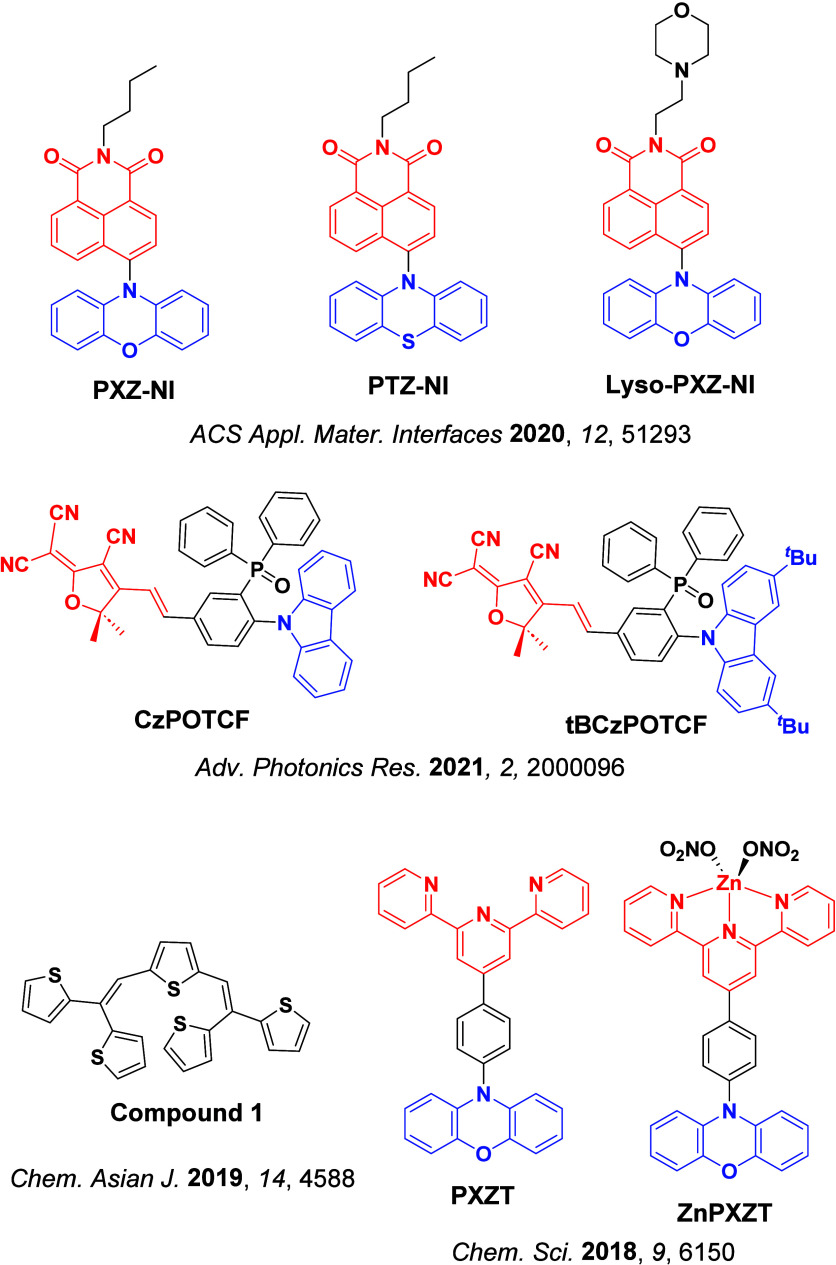
Chemical structures of organic TADF molecules having aggregation-induced delayed fluorescence used as imaging reagents (the blue color signifies donor moieties, while the red color signifies acceptor moieties).

Sarkar *et al*. reported the first example of a TADF material that is not a D-A structure but rather an oligothiophene derivative, **Compound 1** (Figure 252).^1451^ In DMSO solution the compound acts as a conventional fluorophore, however in a DMSO/H_2_O mixture the emitter aggregates exhibiting AIDF at λ_PL_ = 600 nm (*τ*_PL_ = 4.2 μs and 8.0 μs, Φ_PL_ = 11%). Time-dependent luminescence imaging and cytotoxicity studies of **Compound 1** were carried out in HeLa cells, showing low cytotoxicity to the cells and excellent signal-to-noise ratios.^1451^

Instead of preparing aggregates before entry into cells as described in the previous examples, Ni *et al*. proposed a strategy where aggregates would only form within the cells.^1365,1408^ With an increase of the water content in THF/water mixtures, the lifetimes and the fraction of delayed emission contribution both increased for **PXZT** (Figure 252), demonstrating AIDF. In 20 wt% PMMA films, **PXZT** likewise showed a long-lived emission with a τ_d_ of 1.4 μs. However, as with most organic TADF emitters this compound was insoluble in water, which limits its applications to biological microscopy. Incorporation of [Zn(NO_3_)_2_]^4–^ resulted in the formation of a water-soluble complex, although the emission was also quenched. It was proposed that once in the cell the complex becomes kinetically labile and the zinc ions dissociate when the complex is close to a channel or protein that acts upon zinc. Dissociation of the metal complex thus leads to precipitation of the ligand, and the formation of TADF aggregates which can then be visualized. When the compound was added to HeLa cells and allowed to incubate for 5 hours TADF could indeed be observed, suggesting zinc complex dissociation. The same method was then used for detection of chelating ligand EDTA, as EDTA complexes strongly with zinc leading to dissociation of the zinc from the TADF complex and turning on that ligand’s own TADF.^1365^

### 21.7 Other TADF Bioimaging Reagents

Another strategy to render small molecule TADF emitters biocompatible for imaging studies is to develop hydrophilic TADF luminophores, which can be achieved through the introduction of a hydrophilic group.^11,46^ Ni *et al.*, for instance, designed a hydrophilic TADF luminophore (**NID-TPP**) by introducing a triphen­yl­phos­phonium (TPP^+^) group onto 6-(9,9-di­meth­yl­acri­din-10(9*H*)-yl)-2-phen­yl-1H-ben­zo[*de*]iso­quin­o­line-1,3(2*H*)-dione (**NID**), Figure 253a.^1452^ The pristine **NID** exhibits TADF with an emission at λ_PL_ of 610 nm, a small Δ*E*_ST_ of 0.03 eV, and a *τ*_d_ of 5.58 μs in toluene. **NID-TPP** possesses the same Δ*E*_ST_ value of 0.03 eV, but with a shorter *τ*_d_ of 902 ns in the solid state. The Φ_PL_ of the **NID-TPP** is 0.015% in aqueous solution; however, a 40-fold enhancement was observed (Φ_PL_ = 0.6%) upon addition of sodium tetraphenylborate. Due to the strong electrostatic interactions between the TPP^+^ group and BPh_4_^–^, **NID-TPP** aggregates and there is a resulting AIE associated with an emission peak at 618 nm and *τ*_d_ of 1.2 μs. In both the plasma and mitochondria the membrane potential is negatively charged, allowing positively charged species such as **NID-TPP** to gradually accumulate in the cytoplasm as well as into the mitochondrial matrix through passive transport. Thus, **NID-TPP** was utilized for TRLI and two-photon luminescence imaging of HeLa cells and their substructures (Figure 253b). As shown in Figure 253, at short incubation times no fluorescence signal could be detected in the extracellular medium, while with longer incubation time enhanced fluorescence signals could be observed in HeLa cells.^46^

**Figure 253:**
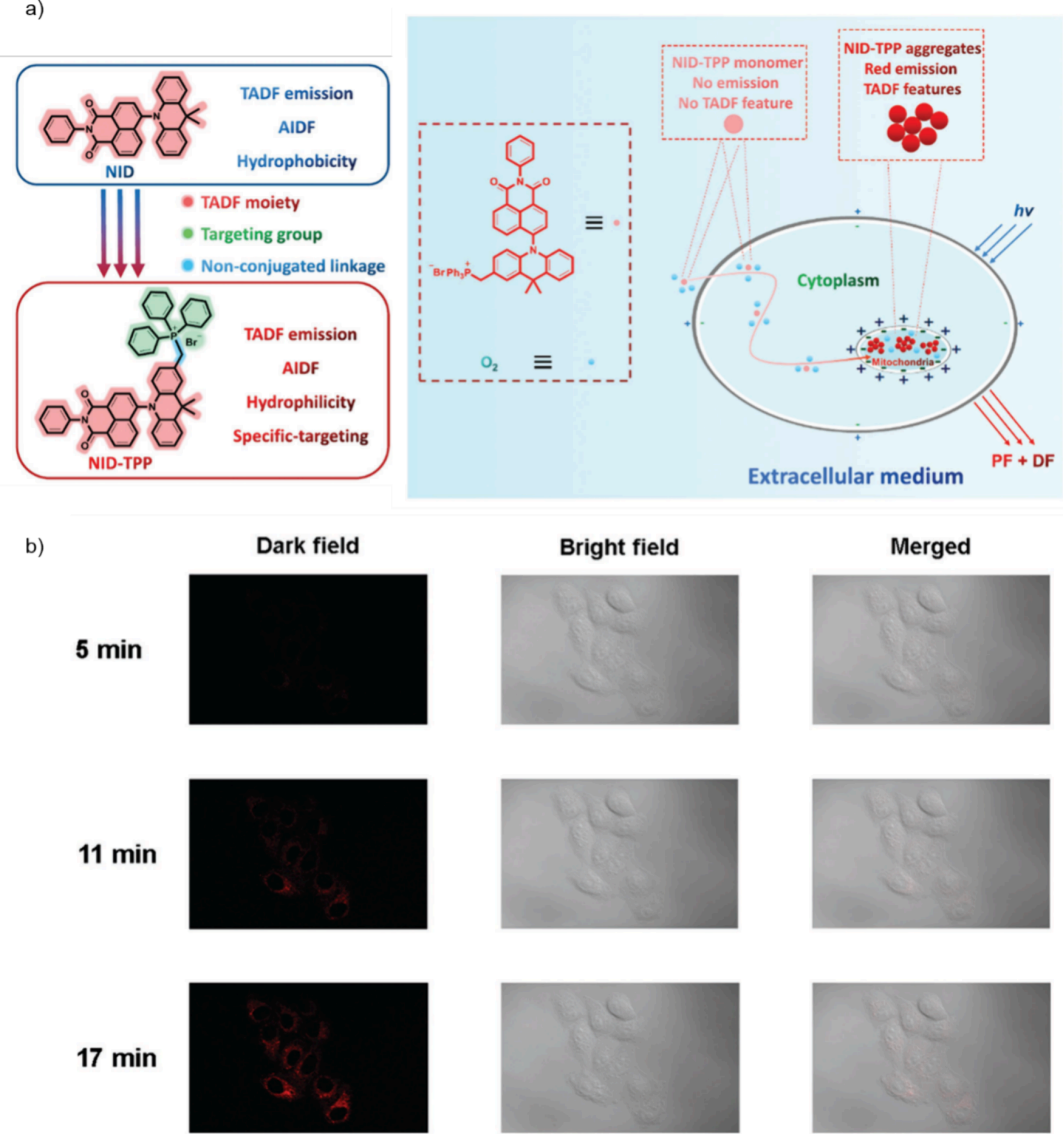
a) Design and proposed uptake mechanism of **NID-TPP** for TRLI of mitochondria in HeLa cells. b) Two-photon luminescent images of HeLa cells incubated with 10 × 10^–6^ M NID-TPP for 5 min, 11 min, and 17 min. λ_exc_ = 810 nm, λ_PL_ = 540–660 nm. Taken and adapted with permission from ref 1452. Copyright [2020/Advanced Optical Materials] John Wiley & Sons.

Hudson and co-workers developed multifunctional compartmentalized nanoparticles based on block copolymers, with a hydrophilic cell-penetrating corona surrounding the TADF-active co-monomer block (Figure 254).^1453^ This was the first system to employ a single polymer as both the emitter and the cell-penetrating moiety. The polymer nanoparticles (Pdot), **BGN_10_-*b*-P_20_Pdot**, exhibited a Φ_PL_ of up to 19% in water and significant delayed fluorescence (τ_d_ > 26 μs) under both air and inert atmospheres. These all-organic polymer nanoparticles were shown to efficiently enter HeLa, CHO, and HepG2 cells within 30 min, with cell viabilities remaining high for Pdot concentrations of up to 25 mg mL^–1^. When used for fixed cellular imaging, Pdot-incubated cells showed high signal-to-background ratios compared to control samples with no Pdot exposure. Using time-resolved spectroscopy, the delayed emission of the Pdots was effectively separated from that of both a biological serum as well as from a secondary fluorescent dye.

**Figure 254:**
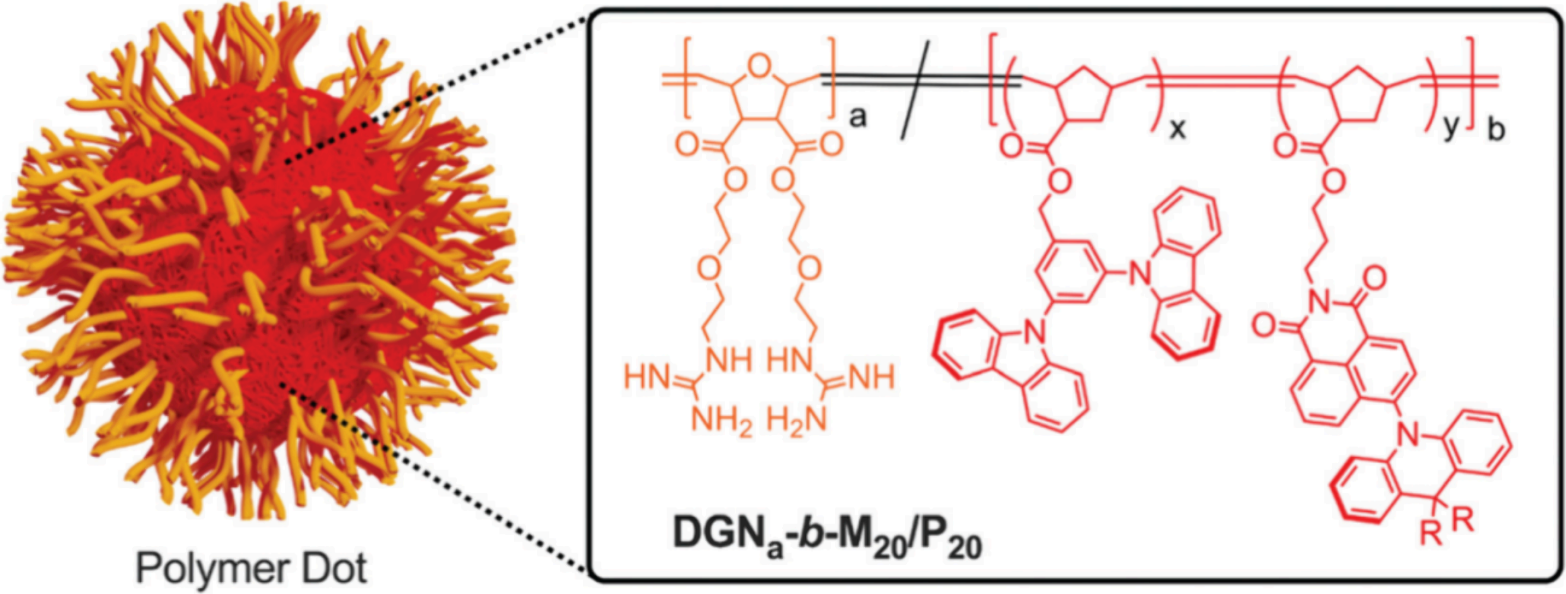
Polymer dots formed by self-assembly of an amphiphilic block copolymer containing a water-soluble cell-penetrating guanidine unit, and a hydrophobic host material and TADF emitter used to deliver TADF emitters to biological targets. Taken and adapted with permission from ref 1453. Copyright [2021/Journal of the American Chemical Society] American Chemical Society.

**Figure 255:**
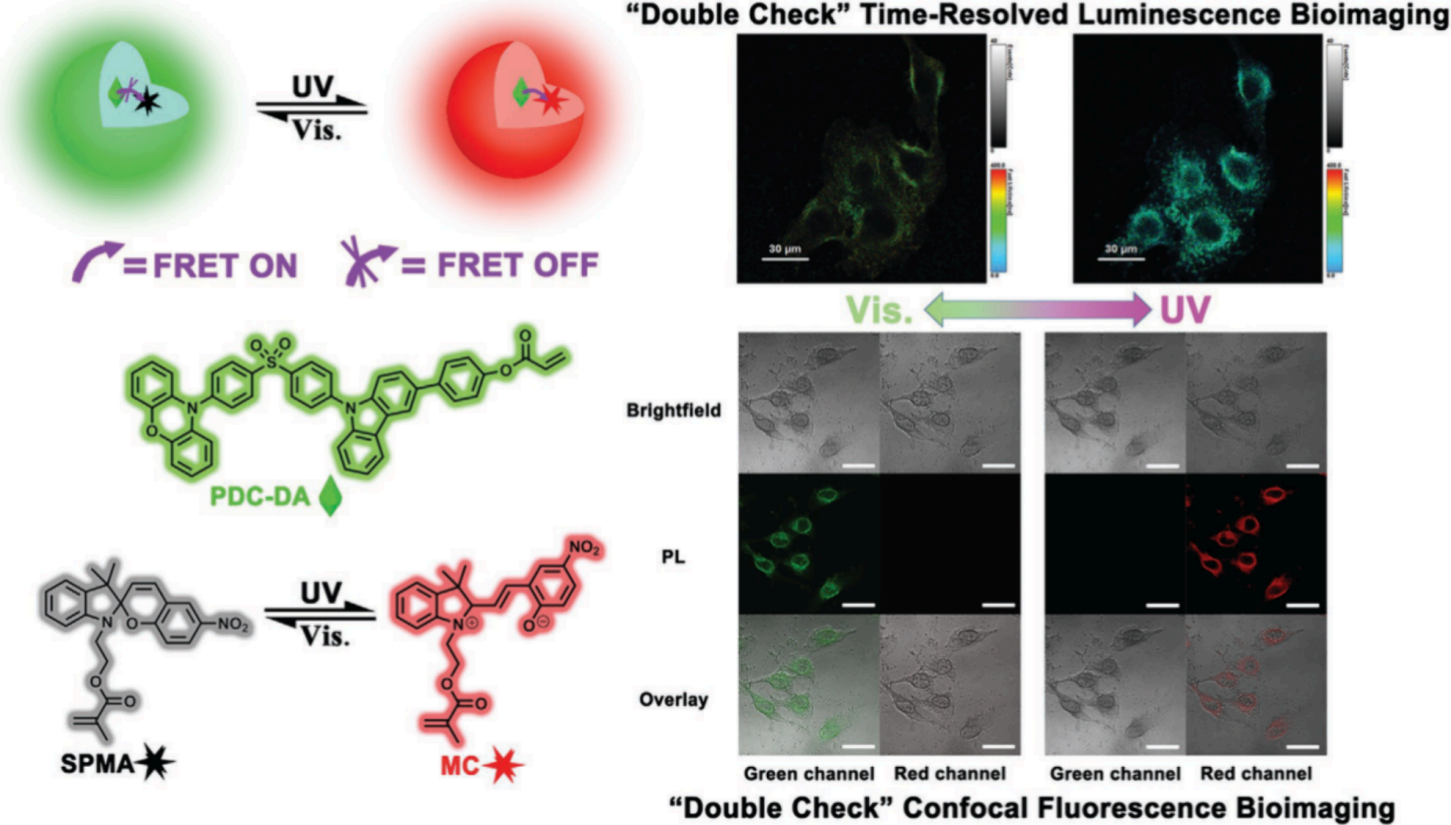
Schematic of photochromism and long-lived luminescent “double-check” bioimaging using the TADF polymeric nanoparticle **PDFPNs**. Taken and adapted with permission from ref 1454. Copyright [2022/Advanced Optical Materials] John Wiley & Sons.

By covalently incorporating a TADF monomer (**PDC-DA**) and a photochromic spiropyran derivative (**SPMA**), Yang and co-workers reported a two-component photoswitchable TADF polymeric nanoparticle (**PDFPNs**) (Figure 255)^1454^. The polymerizable luminophore, **PDC-DA**, was used as the energy donor while the photoresponsive SPMA was employed as the energy acceptor. The green emission of **PDC-DA** can be converted into red fluorescence when the SP unit is converted into its red-emissive ring-open merocyanine (**MC**) state using 365 nm UV light, enabling FRET from **PDC-DA** to **MC**. Subsequently, 525 nm visible light can be used to efficiently recyclize the SPMA into a FRET-inactive form, recovering the green emission of **PDC-DA**. The **PDFPNs** possesses a τ_d_ of 3.3 μs under degassed conditions and a shorter lifetime of 2.73 μs in aerated aqueous solution. After being irradiated by 365 nm light, the τ_d_ was remarkably reduced to 1.61 μs, which the authors ascribed to an efficient FRET process that was switched on between the donor and acceptor. These nanoparticles showed negligible oxygen-sensitivity, high FRET efficiency, rapid and reversible photo responsiveness, and long-term fluorescence stability. They were thus used to realize reversible dual-color confocal and time-resolved luminescence imaging.

### 21.8 Outlook

Organic TADF compounds with long-lived excited states have emerged as highly promising bioimaging agents. Their distinctive advantage, compared to fluorescent emitters, stems from their ability to eliminate interference from short-lived autofluorescence background signals in TRLI. By developing materials with a suitable delay time between short-lived biological autofluorescence and the long-lived TADF emission of the dye, accurate detection and imaging of various biologically relevant species is enabled. The examples included above illustrate the promise and several direct applications of TADF materials as versatile cellular and tissue stains.

Aside from long-lived emission arising from relatively slow RISC (in contrast to the requirements of TADF materials for OLEDs), good biocompatibility and tolerance of both atmospheric and intracellular oxygen are required in these applications. To achieve these properties, the design strategies reviewed in this section include: 1) TADF emitters capped with BSA/HSA; 2) TADF-based Odots formed by encapsulating TADF emitters in an amphiphilic polymer; 3) Silica-based nanoparticles as hosts for the encapsulation of the TADF emitter; 4) Self-assembled nanoparticles; 5) Aggregation-induced delayed fluorescence; and 6) Other TADF bioimaging agents such as water-soluble TADF polymers. While successful examples of each of these strategies exist, because these target properties are so different from those sought by ‘mainstream’ TADF OLED research, design rules to produce optimal imaging agents are still rapidly developing. Consequently, TADF materials offer significant opportunities for future innovation, although various unique challenges must be addressed before deployment in preclinical/clinical context. Of these we highlight in particular the following: 1) improving the inherent water solubility and poor bioavailability of these organic emitters; 2) designing high brightness deep red or NIR TADF emitters for deep-tissue theranostics, thereby mitigating optical tissue attenuation *in vivo*; 3) Design of a wider library of TADF bioimaging agents that show targeted uptake for imaging of specific organelles. In contrast to collaboration between physicists and chemists underpinning much of the work in other sections of this review, in this arena it will be growing collaboration between chemists, biologists, and medical researchers that spurs deeper understanding, progress, and utility of these materials.

## 22 Organic Solid-State Laser Using TADF Components

### 22.1 Introduction

Including vertical excitation, the four-level energy structure of TADF materials makes it practical to achieve population inversion of excited states, which is the very first step for lasing. In addition, TADF emitters and organic semiconductor materials more widely possess distinct advantages over their inorganic counterparts, such as wide range of tunability for their emission spectrum, light weight, mechanical flexibility, and potential for low-cost fabrication of large-area arrays. Their strong optical transitions lead to high gain, and they can have high Φ_PL_ in the solid state. These properties (purely fluorescent and not yet involving TADF activity) have driven the recent interest in organic solid-state lasers (OSSLs), which are promising devices with applications in scanners, printers, sensors, and as cutting-edge light sources with high spectral, spatial, and temporal resolution.^39,1455,1456^

Although the OSSL has been demonstrated under pulsed or even quasi-continuous-wave optical excitation, producing an electrically operated OSSL remains a challenging but tantalizing goal.^1457^ Development of commercial applications for OSSL requires overcoming this bottleneck. Compared to optical excitation, where the majority of generated excitons are singlets that possess a fast radiative decay rates, under electrical excitation spin-statistics of uncorrelated charge recombination governs the exciton ratio between the emissive singlets and dark triplets. As with OLED applications discussed in most of the previous sections, this leads to a 1:3 singlet:triplet exciton ratio under electrical excitation, with triplets typically unable to contribute to lasing. As a result, to achieve amplified spontaneous emission (ASE) from the gain medium, extremely high current densities (> kA/cm^2^) are needed to produce the singlet exciton densities required to reach the lasing threshold under optical excitation.^1458,1459^ These high current densities and long-lived triplet excitons induce detrimental effects on the ASE process and material. These effects include 1) exciton loss due to the bi-excitonic interactions, such as exciton-exciton annihilation, and exciton-polaron quenching; 2) gain loss due to the excited state absorption of singlet, triplet, polaron, and other species; 3) material degradation through Joule heat generation, and photo- and electrochemical bond cleavage under high current and excitation density. For all the above, managing the population of triplet exciton plays a crucial role in maintaining an achievably low lasing threshold.

There are two approaches for the management of triplet excitons. The first is triplet quenching, in which triplet excitons are actively quenched by a doped triplet scavengers in the gain medium, such as oxygen, anthracene, and cyclooctatetraene (COT).^1460–1462^ These scavengers have a higher singlet exciton energy but lower triplet exciton energy than the dye molecules, so that they do not interfere with lasing of the singlet excitons and only quench the triplets. Direct removal of triplet excitons helps alleviate some of the gain loss and material degradation pathways that rely on these, but does not avoid their initial formation, meaning that high current densities are still required. The second approach to managing triplets is harvesting, in which triplet excitons are either made emissive with the help of heavy metal atoms such as those found in phosphorescent metal complexes, or are converted to singlets by RISC in TADF materials, allowing the triplet excitons (and up to 100% of total excitons) to be used in the same way this is achieved in OLEDs.

Although reports of ASE directly from the triplet states of organic phosphorescent molecules are rare,^1463^ ASE has been observed in TADF molecules. In this section we document the recent progress on OSSLs employing TADF molecules, categorized key examples as: 1) TADF molecules used as the gain medium; 2) TADF molecules used as the triplet harvester; and 3) lasing from TADF molecules (see material structures in Figure 256).

**Figure 256:**
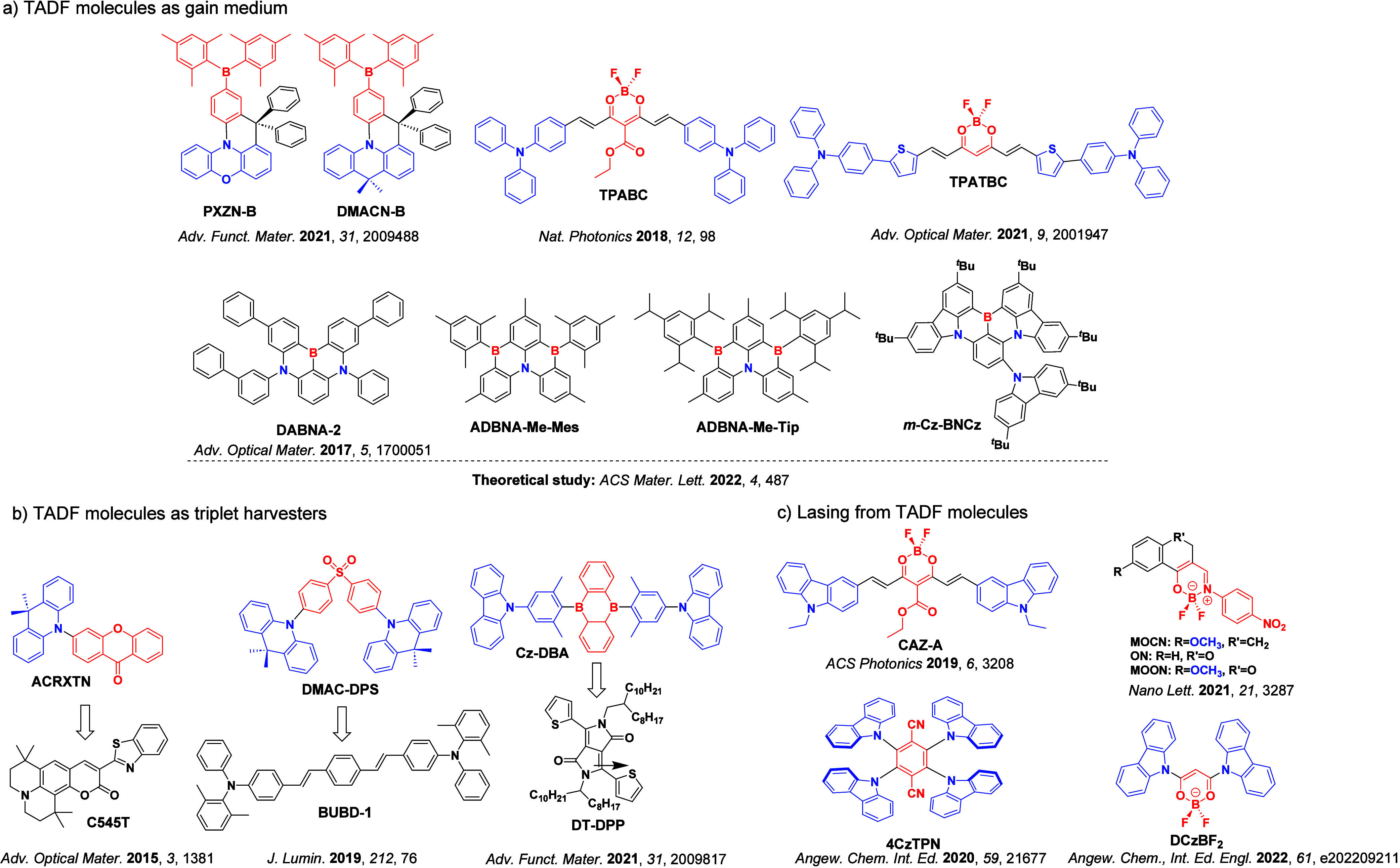
TADF molecules used in lasing applications. a) TADF molecules as gain media; b) TADF molecules as triplet harvesters (the arrows represent the direction of the FRET process); c) lasing from TADF molecules. The blue color signifies donor moieties/atoms/functional groups, while the red color signifies acceptor moieties/atoms/functional groups.

### 22.2 TADF Molecules as the Gain Medium

A promising gain medium must have gain exceeding loss. Typically this involves materials with sufficiently high Φ_PL_, absorption separated from emission, and low waveguide loss, leading to low threshold for ASE. Applying these conditions, a number of TADF materials have been reported.

The effects of the molecular structure on the ASE were investigated in a comparative study between MR-TADF (**DABNA-2**) and D-A-TADF (**3CzTrz**) molecules.^1464^ Although both compounds have similar Φ_PL_ in 6 wt% doped mCBP films (∼80%), similar peak λ_PL_ (∼470 nm), and similar singlet exciton lifetime (∼5 ns), ASE was only observed in the **DABNA-2** (Figure 256a) doped thin films under pulsed optical excitation. As the excitation energy increased above the threshold energy (*E*_th_ = 1.6 μJ cm^–2^), an increase in PL intensity accompanied by a spectral narrowing was observed, confirming an ASE process. The ASE wavelength (λ_ASE_) was 494 nm, corresponding to the 0-1 electronic transition. The absence of ASE in **3CzTrz** was attributed to its much broader PL spectrum (FWHM = 80 nm) compared to that of **DABNA-2** (FWHM = 30 nm), which decreased the stimulated emission cross-section, thereby increasing the possibility of both self-absorption and triplet absorption.

Recently, the molecular properties of 17 different **BN**-cored molecules were theoretically computed to give molecular design rules for TADF-based gain materials.^1465^ Four key screening parameters including the oscillator strength, net optical emission cross-section, singlet lifetime, and *k*_RISC_ were considered, resulting in four promising candidates for lasing, **DABNA-2**, ***m*-Cz-BNCz**, **ADBNA-Me-Mes**, and **ADBNA-Me-Tip**. Depending on the amplification modes, specific molecular design strategies were proposed to minimize the self-absorption; either to introduce additional vibrational modes for the 0-1 transition, or to optimize the substitution position to induce a large Stokes shift for the 0-0 transition.

To engineer high Φ_PL_, conventional D-A-TADF emitters can be rigidified using a proposed intramolecular-lock strategy as in **PXZN-B** and **DMACN-B** (Figure 256a), in which a diphenylmethylene group was inserted to adjust the torsion angle and restrict the intramolecular relaxation.^1466^ The locked TADF emitters not only showed high Φ_PL_ of 93% and 90%, but also narrow PL emission with FWHM of 48 nm and 29 nm in doped films for **PXZN-B** and **DMACN-B**, respectively. Under pulsed optical excitation, the doped thin films of **PXZN-B** and **DMACN-B** in mCP showed ASE behavior with λ_ASE_ at 470 nm and 448 nm, and low ASE thresholds (*E*_th_) of 4.0 and 12.0 μJ cm^–2^, respectively.

Since 2018, high efficiency solution-processable red TADF emitters based on borondifluoride curcuminoid (**BC**) derivatives have drawn increasing attention, which also exhibit ASE behavior.^1467^ These molecules possess a linear donor-acceptor-donor (D-A-D) structure and usually show photophysical properties that are highly dependent on the doping concentration, implying strong intermolecular interaction. In **TPABC** (Figure 256) doped thin films in CBP, ASE behavior was observed across a wide range of doping concentrations from 2 wt% to 40 wt%, with *E*_th_ all below 100 μJ cm^–2^. The lowest *E*_th_ of 7.5 μJ cm^–2^ (λ_ASE_ = 750 nm) was obtained in the film, which had the lowest doping concentration (2 wt%) and also the highest Φ_PL_ of 70%. The origin of ASE was subsequently identified by the same group as resulting from a low energy dimeric structure.^485^ In addition, the gain loss due to the triplet absorption was found to be negligible in those molecules. To push the λ_ASE_ beyond 800 nm, a thiophene ring was inserted between the D and A moieties to give **TPATBC** (Figure 256a), which reduced the HOMO–LUMO energy gap.^1468^ When doped into a low triplet energy host, F8BT, **TPATBC** showed a Φ_PL_ of about 45%, a short singlet lifetime of 1.3 ns, and λ_PL_ at 724 nm. Under pulsed optical excitation, ASE was observed with *E*_th_ of 13.3 μJ cm^–2^ and λ_ASE_ of 807 nm. The large red-shift of the λ_ASE_ with respect to the PL was attributed to the strong singlet-singlet absorption of **TPATBC**, which inhibits the electronic transition to the lowest vibrational level of the ground state. With a second-order DFB (distributed feedback) resonator, lasing was observed, with a further reduced *E*_th_ of 6.2 μJ cm^–2^.

### 22.3 TADF Molecules as the Triplet Harvester

The potential of TADF molecules for ASE has also been explored in co-doped thin films, in which triplet excitons are harvested by TADF molecules and then transferred to a fluorescent laser dye via FRET. Excitons accumulate on the laser dye, allowing ASE to occur, in so-called TADF-assisted ASE with strong parallels to HF-OLED approaches. In this manner, ASE has been observed with the same λ_ASE_ but reduced *E*_th_ compared with the system without the inclusion of the TADF molecules. This approach is highly appealing for applications in electrically-pumped lasing, where the TADF material may be able to convert triplet excitons to singlets before FRET, thus theoretically reducing the threshold current density by as much as a factor of 4. As an additional consideration, for efficient FRET to occur there must be a sufficient spectral overlap between the emission spectrum of TADF molecules and absorption spectrum of laser dyes.

The high efficiency TADF molecule **ACRXTN** possesses considerable FRET overlap with the green laser dye **C545T** (Figure 256b), implying that an efficient FRET process can occur. Indeed, when co-doped into an mCBP host (**ACRXTN**: 6 wt%, **C545T**: 1 wt%), *E*_th_ was decreased from 1.2 μJ cm^–2^ (**C545T** only) to 0.8 μJ cm^–2^ (TADF assisted), with λ_ASE_ of 535 nm in both cases.^1469^ A similar effect was observed when using a sky-blue fluorescent molecule **BUBD-1** (Figure 256b) as the laser dye.^1470^ A slightly different strategy was adopted wherein instead of co-doping, the TADF molecule **DMAC-DPS** (Figure 256b) was itself used as the host. The doped thin films of **BUBD-1** (2 wt%) showed similar high Φ_PL_ of 82% in both CBP and **DMAC-DPS** hosts. However, *E*_th_ was reduced from 1.51 μJ cm^–2^ in CBP to 1.19 μJ cm^–2^ in **DMAC-DPS**, with λ_ASE_ of 500 nm for both. It was proposed that the TADF host could not only harvest triplet excitons, but also promote FRET through better overlap with the dopant, together resulting in a lower *E*_th_. Recently, the co-doping strategy was explored with the use of a red laser dye, dithio­phenyl-diketo­pyrrolo­pyrrole (**DT-DPP**).^1471^ The green TADF emitter **Cz-DBA** (10 wt%) was used as the assistant dopant with **DT-DPP** (1 wt%) together in CBP as the bulk host (Figure 256b). With the same λ_ASE_ of 620 nm, Φ_PL_ and *E*_th_ were both improved from 65% and 7.3 μJ cm^–2^ in the simple doped film to 77% and 4.0 μJ cm^–2^ in the TADF-assisted thin film.

With the co-doped TADF molecule acting as a triplet harvester, a lower *E*_th_ was achieved in fluorescent dye molecules without varying λ_ASE_. These results show that TADF molecules can not only minimize the detrimental effect of triplet excitons on ASE, but also promote exciton energy transfer to dye molecules, resulting in higher Φ_PL_ and lower *E*_th_. A brief synopsis of ASE parameters for TADF molecules is shown in Table S24. In all of these examples though it should be noted that the TADF assistant dopant is only harvesting the relatively small number of triplet excitons that are generated by photoexcitation (some of which form directly on or because of the TADF emitter itself). In the ultimate application of this strategy in electrically-pumped OSSLs, the density of triplet excitons will be orders of magnitude larger, requiring TADF materials with outstanding RISC rates in order to convert these triplets sufficiently quickly to avoid quenching and material damage. Although this concept is already thoroughly demonstrated in high *k*_RISC_ OLEDs with small efficiency roll-off, the higher exciton densities required for lasing operation means that such a device remains yet to be demonstrated.

### 22.4 Lasing from TADF Molecules

With well-designed optical resonators that act as cavities to tune wavelengths and more easily reach threshold, lasing has been observed from several TADF molecules. So far, the optical resonators used have been a microring array,^1472^ a microsphere array,^1473^ and a Fabry–Pérot type microcrystal/microwire structure^1474^ (Figure 257). All these resonators show strong optical confinement with high Q-factors near or above 1000. When the excitation energy reaches above threshold energy (*E*_th_^laser^), lasing with characteristically narrow FWHM (< 1 nm) can be observed.

**Figure 257:**
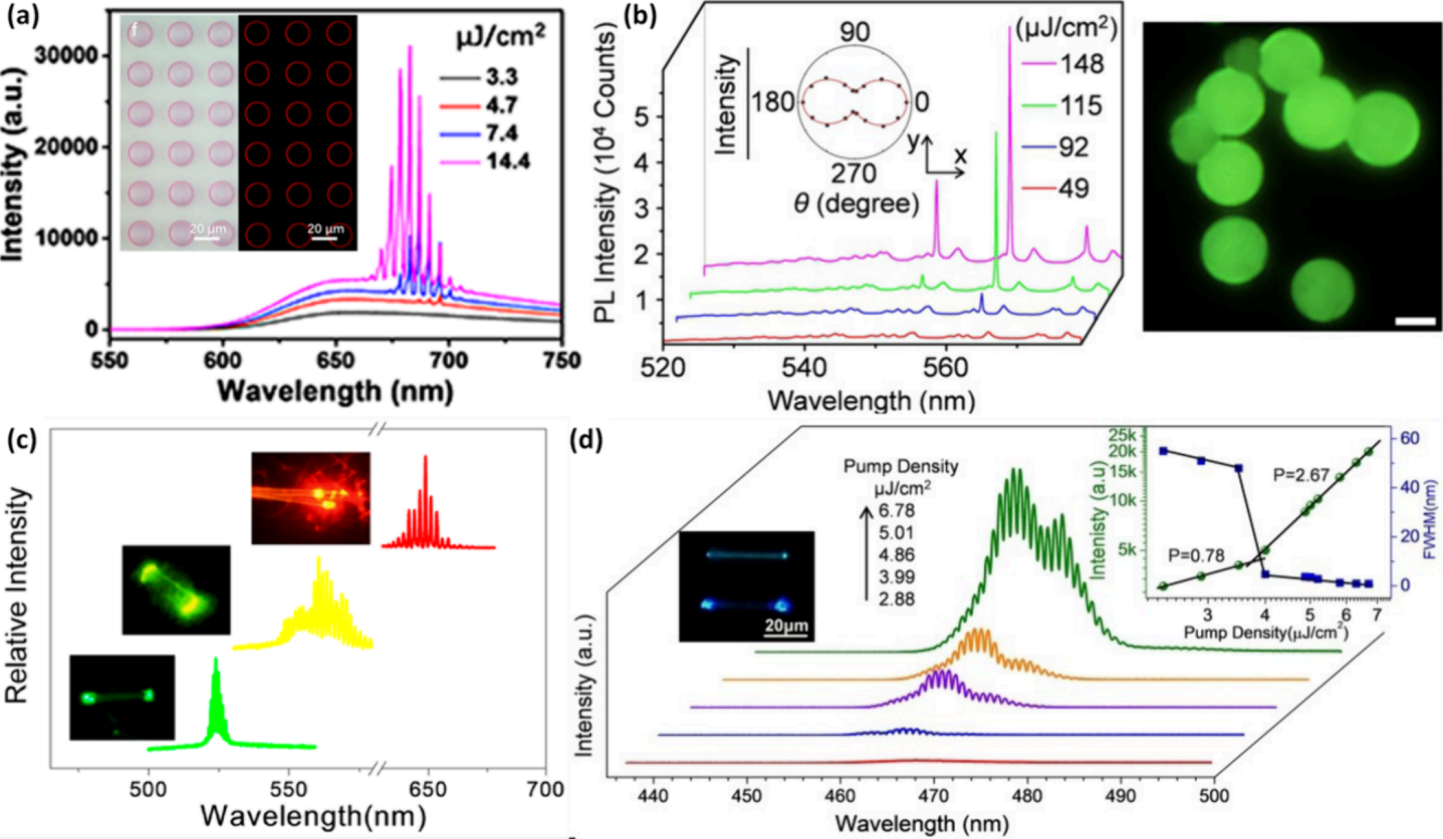
Reported resonator structures for lasing using TADF molecules: a) 4 wt% **CAZ-A** doped in CBP thin films with microring array; b) 3 wt% **4CzTPN** doped in PS thin films with microsphere array; c) faceted **MOON, ON**, and **MOCN** single-crystalline microcrystals; d) **DCzBF_2_** 1D single-crystalline microwires. Taken and adapted with permission from ref 1472 (Copyright [2019/ACS Photonics] American Chemical Society); ref 1473 (Copyright [2020/Angewandte Chemie International Edition] John Wiley & Sons); ref 1474 (Copyright [2021/Nano Letters] American Chemical Society); ref 1476 (Copyright [2022/Angewandte Chemie International Edition] John Wiley & Sons), respectively.

By using a confined solution-growth method, a whispering-gallery mode (WGM) microring resonator array was fabricated with a high Q-factor of 1300 at 683 nm.^1472^ The gain material was a red **BC** derivative, **CAZ-A** (Figure 256c), which was doped in CBP host. With the strong optical confinement of the microring resonator, lasing was observed with *E*_th_^laser^ of 3.69 μJ cm^–2^, λ_lasing_ of 683 nm as a single narrow peak (FWHM = 0.52 nm). By careful tuning of the microring size from 11.5 to 29.0 μm, the lasing mode spacing (Δλ) was successfully modulated from 8.12 nm to 2.85 nm. It was found that *E*_th_^laser^ increased with decreasing temperature, which was attributed to the slower *k*_RISC_ at lower temperature, resulting in more accumulated triplets. It should be noted that complex thermal effects on the lasing material, cavity, or otherwise can often confound the accurate identification of lasing behavior,^1475^ and so whether a change in *E*_th_ arises as a consequence of changes in RISC requires further study.

Using an emulsion-solvent-evaporation method, another WGM resonator was fabricated based on polymeric microspheres with circular boundaries and smooth surfaces. The gain medium was a green TADF molecule, **4CzTPN** (Figure 256c) doped in PS (polystyrene).^1473^ Under pulsed optical excitation, lasing at 563 nm with FWHM of 0.21 nm was observed when the excitation energy increased above 88 μJ cm^–2^. A similar resonator size dependence of Δλ was observed in microsphere as well, confirming the lasing is indeed WGM-type. From the transient absorption spectrum and temperature dependence of *E*_th_^laser^, the authors concluded that triplet absorption was negligible and fast RISC had a positive influence on the lasing threshold.

Recently, faceted microcrystals of boron difluoride-based TADF molecules were fabricated by a facile reprecipitation method.^1474^ These microcrystals not only covered emission wavelengths from red (**MOON**), yellow (**ON**), to green (**MOCN**) (Figure 256c), but also possessed a high-quality intrinsic Fabry-Pérot resonator with Q factor larger than 1000. Under pulsed optical excitation, lasing and lasing oscillation were observed with *E*_th_^laser^ (λ_lasing_) of 3.04 μJ cm^–2^ (650 nm), 4.96 μJ cm^–2^ (561 nm), and 3.49 μJ cm^–2^ (525 nm) for **MOON**, **ON**, and **MOCN**, respectively. Beside the temperature dependence of *E*_th_^laser^, triplet up-conversion was supported by transient PL measurements, in which a plateau structure was attributed to the extra generated singlet exciton via RISC.

Besides microcrystals, single-crystalline microwires of the TADF material **DCzBF_2_** (Figure 256c) were also fabricated using a solution self-assembly method.^1476^ The molecular geometry changed from a highly twisted D-A structure in solution to a nearly coplanar conformation in the microwires, which showed AIE originating from locally excited states. The 1D microwires, exhibiting herringbone-like molecular packing, smooth surface, and high Φ_PL_ of 48%, had a uniform diameter of about 1 μm and a length of 10–100 μm. These microwires thus served as natural Fabry-Pérot resonators with estimated Q factor of 930. Under pulsed optical excitation, a series of sharp cavity mode peaks at around 465 nm were observed with *E*_th_^laser^ of 3.74 μJ cm^–2^ and FWHM below 0.5 nm. The temperature dependence of *E*_th_^laser^ and the microwire length-modulated cavity modes confirmed the TADF behavior and the internal microcavity effect, respectively. For a summary of TADF materials with lasing properties, see Table S25.

### 22.5 Outlook

Although ASE has been observed in both TADF molecules and in TADF-assisted laser dye systems, key advancements that RISC is expected to support in this area are yet to be achieved – most notably the harvesting of triplet excitons for electrically driven lasing. Even exploiting well-designed resonators such as microcrystal/microwire Fabry-Pérot cavities, DFBs, microrings, and microspheres, lasing thresholds from TADF molecules are typically an order higher than those of conventional fluorescent laser dyes. This is likely a consequence of their slower radiative rates and their active ISC channels (generating triplet excitons) outweighing any beneficial ability to harvest triplets by RISC. The relatively low absorption cross section in the lowest charge-transfer bands of D-A TADF emitters also hinders population inversion, as does the broad emission bands of their CT emission for ASE. In contrast though, the strong absorption as well as efficient narrowband emission and high radiative rates of MR-TADF materials we speculate will find increasing use for optically-driven lasing films in the coming years, sharing these advantageous properties with purely fluorescent emitters.

Returning to electrically driven lasing, the large accumulation of triplets when operating at the necessary high currents is detrimental to stability, and likely incompatible with the delicate materials currently deployed in TADF OLEDs. While this appears discouraging for the use of TADF materials in laser systems, further enhancements in RISC (as sought by the OLED research community) could eventually overcome this issue. Some promising results have already been seen with the aid of LE states,^1476^ proving there is a sizeable contribution from RISC to ASE and lasing, but more significant advances in increasing RISC rates will be required in order for these materials to contribute meaningfully towards the prized development of electrically-pumped OSSLs. Considering the significant applications such a technology could unlock, we anticipate only an intensification of research activity in this area over the remainder of the decade.

## 23 TADF Materials as Photocatalysts

### 23.1 Introduction

The use of organic donor-acceptor (D-A) TADF compounds as photocatalysts (PCs) has gained considerable attention since the first report in 2016.^1477^ Visible light photocatalysis has been known since the late 19^th^ century, but has seen a resurgence of interest over the last 15 years, especially as a tool for developing ‘green’ chemistry.^1478–1481^ From water splitting^1482^ to degradation of pollutants,^1483^ the applications of photocatalysis are vast and potentially deeply impactful. Historically employing transition metal-based complexes, the use of TADF PCs, however, has thus far only been investigated with respect to small molecule photocatalysis or photopolymerization.

Photocatalysis proceeds by recruiting the excited state of the PC, generated by electronic excitation upon absorption of light, to engage in either energy or electron transfer with an organic substrate (Figure 258). In the photoinduced energy transfer (PEnT) mechanism, the substrate receives energy from the excited PC through a Förster or Dexter energy transfer process, regenerating the ground state of the PC. In this way the PC is able to undergo many catalytic cycles, with overall turnover limited by its own intrinsic photostability. When the PC is instead involved in a photoinduced electron transfer (PET) this is termed photoredox catalysis and can occur through either an oxidative or reductive quenching mechanism. A second single electron transfer (SET) step is subsequently required to close the photocatalytic cycle. It is common, though not essential, for sacrificial electron donors or acceptors to be employed in reactions to allow catalytic turnover of the photoredox catalytic cycle.

**Figure 258:**
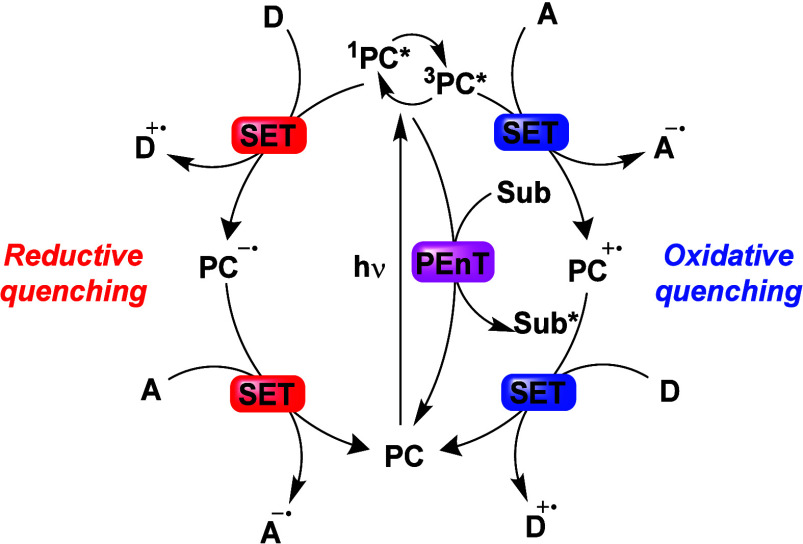
General photocatalytic cycle showing the possible photon induced energy and electron transfer events, where D = donor, A = acceptor, sub = substrate, SET = single electron transfer, and PEnT = photoinduced energy transfer.

Traditionally, iridium(III) and ruthenium(II) complexes (Figure 259) have been used as PCs and dominate much of the literature in homogeneous photocatalysis.^1484,1485^ However, the search for cheaper and less toxic PCs has led to investigations into both Earth-abundant metal complexes,^1486,1487^ and purely organic compounds as suitable alternatives.^36,1488^ To be a useful PC, the compound should exhibit appreciable light absorption (preferably in the visible region) to allow for selective photoexcitation of the PC in the presence of the organic reagents (that typically only absorb in the UV). Excited state lifetimes on the order of at least a few nanoseconds are necessary to allow for diffusion of the excited PC to encounter and undergo PEnT or PET with the substrate in solution. For photoredox catalysis, a wide redox window is also important to facilitate SET with a large range of organic molecules, while for PEnT an appropriate spectral overlap between the emission of the PC and the absorption of the substrate is necessary for efficient energy transfer. From these required properties it is unsurprising that TADF compounds, both organic (see for instance Sections 3–5) and organometallic (Section 9) have been used to great effect as PCs. It is, however, unclear at this point what intrinsic value TADF provides with respect to photocatalysis aside from generating PCs with excited-state lifetimes with sufficiently long-lived excited states to enable the photochemistry. Indeed, different photophysical properties may prove to be ideal for photocatalysis (fast ISC and slow RISC), as opposed to the OLEDs where high photoluminescence quantum yield and fast RISC are desired traits of the emitter..

**Figure 259:**
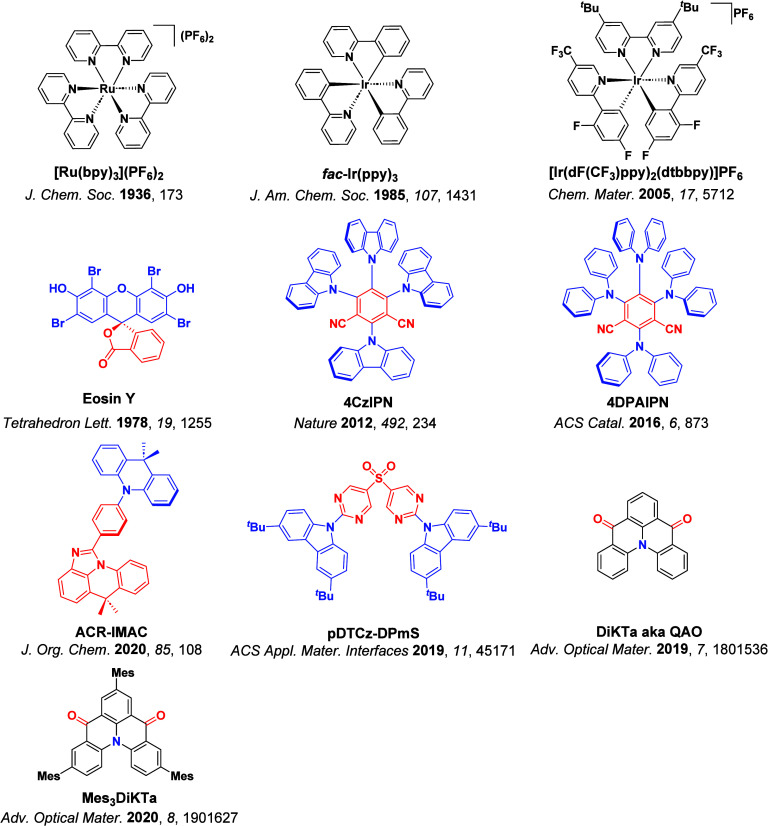
Structures of commonly used visible light organometallic PCs, and a range of popular and recently used TADF PCs. References correspond to first reported publication of the compound, except for **Eosin Y**, whereby the reference corresponds to the first reported use of this organic dye as a PC. The blue color signifies donor moieties, while the red color signifies acceptor moieties.

**Eosin Y** (Figure 259) was the first TADF compound to be used as a PC, where it was employed to photocatalyze the reduction of phenacylonium salts by 1,4-dihydropyridines,^1489^ and to this day remains a staple in the library of commonly used visible-light PCs. Since 2016, organic D-A TADF compounds based on the carbazolyl dicyanobenzene (CDCB) family and related derivatives have been shown to act as potent PCs,^1477^ with **4CzIPN** proving to be a viable replacement for cationic heteroleptic iridium(III) complexes across a diverse range of organic reactions (Figure 259 and Table S26).^1490^ Other organic D-A TADF compounds beyond the CDCB family are yet to receive comparable interest in small molecule photocatalysis, with only two examples known to date: an imidazoacridine-based structure (Figure 259) used in [2+2] PEnT cycloadditions,^1491^ and a pyrimidyl sulfone compound (Figure 259), which showed a broad range of mechanistically distinct photocatalytic reactions.^1492^ A small selection of other D-A TADF compounds have also found applicability in photopolymerizations reactions.^1493^ One of the particular benefits of using D-A TADF compounds as PCs is that facile tuning of the redox properties is possible through judicious and combinatorial choice of each of the donor or acceptor moieties. This kind of tunability has thus far been difficult to achieve with many other organic PCs, where the HOMO and LUMO are not as obviously inferred from the molecular structure.^1494^ Organometallic TADF compounds based on, for instance, copper(I), zirconium(IV) and gold(I) have additionally been explored as Earth-abundant metal PCs.^948,1495–1497^

Recently, we reported that MR-TADF compounds can also be employed as PCs. Both **DiKTa** and **Mes_3_DiKTa** (Figure 259) have been shown to be effective PCs across a wide variety of PEnT and PET reactions, rivalling the efficiency and applicability of **4CzIPN**.^1498^ One particular advantage of using MR-TADF compounds over D-A TADF emitters is that the former displays only modest positive solvatochromism, translating to less solvent stabilization of the excited state, and hence preserving more of the excited state energy for driving reactions. This may contribute to the greater reactivity of MR-TADF PCs, particularly in polar aprotic solvents such as MeCN and DMF which are typically employed in photocatalysis reactions. In a follow up study, **DiKTa** was found to be the most efficient PC in a dual NHC/photoredox reaction for the synthesis of unsymmetric 1,4-diketones.^1499^

Throughout the myriad of reports that use TADF compounds as PCs, it should be noted that the vast majority make no attempt to correlate PC activity with other key TADF photophysical properties. Consequently, no real mechanistic investigation has been undertaken to understand what, if anything, TADF activity contributes to their success as PCs. Indeed, it is reasonable to expect that small Δ*E*_ST_ and fast RISC – prized for OLED applications – would be counterproductive in photocatalysis as these properties would more rapidly depleted excited states through emission channels that compete with PEnT and PET. Regardless, we here present a select few examples of TADF compounds used as PCs, primarily to highlight their versatility. The focus of these examples is based upon the use of CDCB TADF compounds and particularly **4CzIPN_,_** since these compounds have become notably popular in the photocatalysis literature over the last seven years. A comprehensive overview of the wide range of photocatalytic transformations mediated by organic TADF compounds and a summary of the different organic D-A TADF compounds employed as PCs has been recently reviewed by Bryden and Zysman-Colman.^36^

### 23.2 TADF Compounds as PCs

From a survey of the photocatalyst literature it becomes apparent that **4CzIPN** has entered the pantheon of common photocatalysts assessed for photochemical transformations since the first report of its use in 2016.^1477^ The popularity of **4CzIPN** as a PC is therefore clearly evident, although its excellent properties for OLED applications means that its photophysical properties can be further refined for photocatalysis.^1490^ The related material **4DPAIPN** is rapidly becoming a popular alternative, given the stronger reducing capacity of **4DPAIPN** relative to **4CzIPN** (Table S26).^1500^

The scope of these reported TADF photocatalyzed reactions encompasses a large range of organic transformations, from polymerizations^1501,1502^ to cyclizations,^1503,1504^ although the most frequently encountered class of reactions involves C(sp^2^)-C(sp^3^) cross-coupling reactions. A wide range of radical precursors such as carboxylic acids,^1505,1506^ trifluoroborate salts^1507^ and 4-alkyl-1,4-dihydropyridine derivatives (DHPs),^1508^ can be used to reductively quench the excited PC, releasing a C(sp^3^)-centred alkyl radical. This requires the PC to be a relatively strong photooxidant (e.g., E_ox_ of carboxylates typically ranges from 1.2–1.5 V vs SCE).^1509^ Through a radical addition or radical coupling mechanism, a C-C bond is formed, usually facilitated through SET from the reduced PC. Given the strong photooxidizing ability of **4CzIPN** in comparison to other common PCs (E*_red_ = 1.35 V vs SCE, see Table S26), this compound typically is able to act as a photocatalyst for this class of reactions and more widely. For example, in the hydrosilylation of alkenes (Figure 260a), **4CzIPN** yielded 82% of product, while the next best PC in the study, [Ir(dF(CF_3_)ppy)_2_(dtbbpy)]PF_6_, affording only 45% of product.^1510^ This difference in yield is likely due to the suitable redox potentials of **4CzIPN** to oxidize the silacarboxylate radical precursor, as this step is thermodynamically difficult for this iridium PC (e.g., E_ox_(Ph_2_MeSiCO_2_^•^/Ph2MeSiCO_2_^–^) = 1.32 V vs SCE and E*_red_ = 1.35 V and 1.21 V vs SCE for **4CzIPN** and [Ir(dF(CF_3_)ppy)_2_(dtbbpy)]PF_6_, respectively).

**Figure 260:**
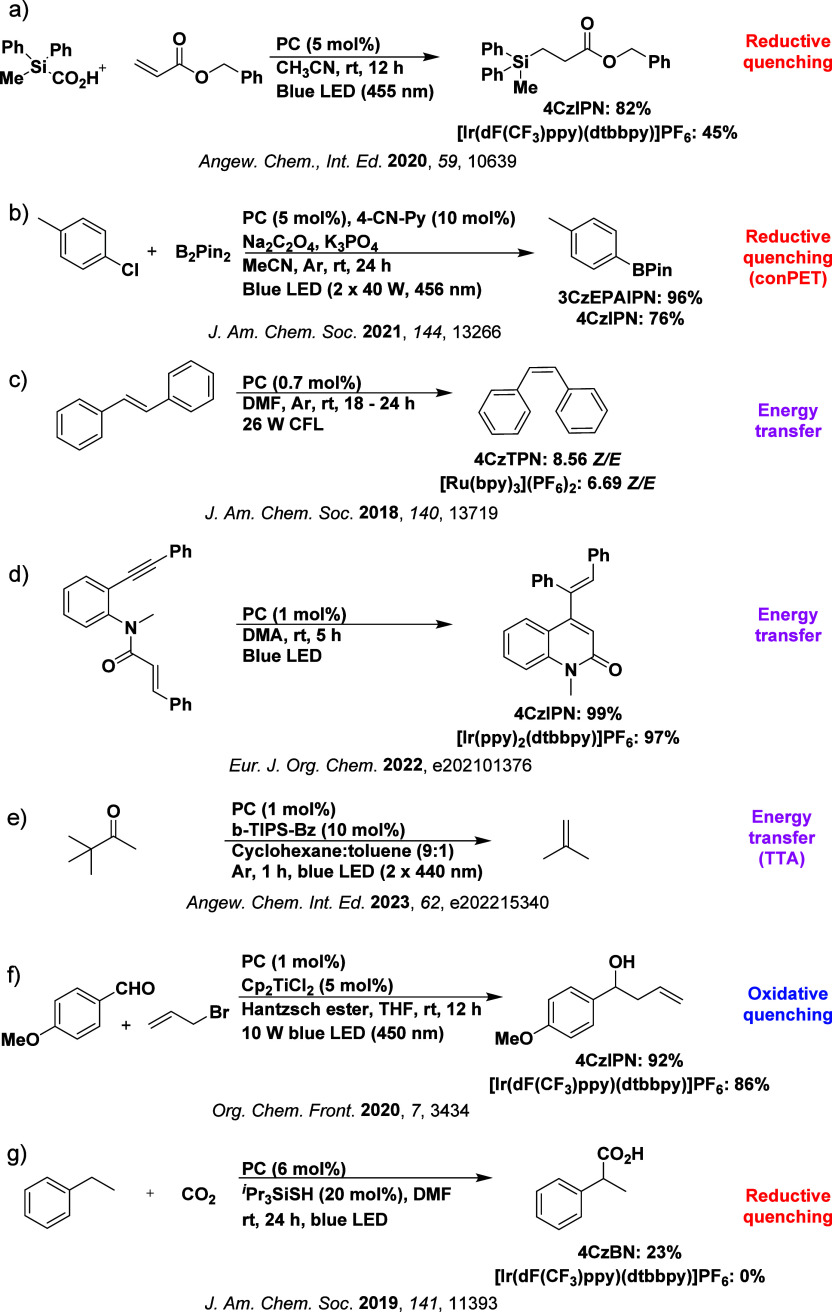
Examples of photocatalysis reactions for which CDCB PCs typically outperform commonly used transition metal PCs. The yields given reflect the highest yielding TADF PC and the highest yielding non-TADF PC. Yields given are obtained from the PC screen; further optimization may have occurred in some cases. CFL = compact fluorescent light.

The reductive dehalogenation of aryl chlorides is a challenging transformation that has also been shown to proceed efficiently using **4CzIPN** and related D-A cyanoarenes as PCs, this time through a proposed consecutive photoinduced electron transfer (conPET) mechanism.^1511^ The PC is initially reductively quenched by a sacrificial electron donor, before the resultant radical anion of the PC is photoexcited again to yield an incredibly strong photoreductant capable of reducing substrates such as 4-chloroanisole (E_red_ = −2.90 V vs SCE).^1512^ The aryl radical formed can then be coupled with a variety of partners, including boronate esters, phosphines and phosphites. While no alternative PCs outside of the CDCB family were screened in this reaction, a total of 13 **4CzIPN**-based PCs were trialled, all of which could borylate 4-chlorotoluene (Figure 260b) to varying degrees (6–96%).

Although less well documented, TADF compounds can act as PCs in energy transfer processes, such as the *E*/*Z* isomerisation of alkenes,^1513^ which proceeds through a Dexter energy transfer mechanism. The *Z*/*E* ratio can be optimized by tuning the triplet energy, E_T_, of the PC, while moderate ISC provides a suitable quantity of triplet excitons from photoexcitation. In the *E/Z* isomerisation of stilbene for example (Figure 260c), **4CzTPN** showed the highest selectivity, even greater than that of the literature PC [Ru(bpy)_3_](PF_6_)_2_ (*Z/E* = 8.56/1 and 6.69/1 and E_T_ = 2.34 eV and 2.03 eV, respectively). Other CDCB PCs, such as **2,4,6-3CzBN** (E_T_ = 2.87 eV), with unsuitably high E_T_, tended to show poorer selectivity with this substrate. More recently, **4CzIPN** has been used to photocatalyze the intramolecular [2+2] cycloaddition reaction of enynes to generate 1,3-diene-quinolinone products (Figure 260d), affording the same yield as [Ir(ppy)_2_(dtbbpy)]PF_6_, but at a fraction of the cost.^1514^ In the endeavour to develop more sustainable photocatalytic protocols, the authors also demonstrated the recyclability of **4CzIPN**, with no loss in the yield of product even after reuse of the PC three times.

Similar to the use of TADF materials as PC for energy transfer reactions, these materials are also gaining recognition as useful triplet sensitisers for other photophysical processes. In TTA upconversion solutions, triplet excitons are generated from low energy photons typically using metalloporphyrin sensitisers. Excitons are then transferred to a separate emitter species, pairs of which diffuse and undergo TTA to generate anti-Stokes shifted emission.^1515^ Recently it has been shown that TADF materials can be used in a similar way,^1516^ with their significantly higher triplet energies (compared to metalloporphyrins) particularly useful for generating UV TTA emission.^1517,1518^ MR-TADF emitters also show promise for this kind of application,^1519^ expanding significantly the tunability and range of potential designs for triplet photosensitisers. An example of this in photocatalysis involves **4CzIPN**, which undergoes a DET to the benzene-based annihilator 1,4-bis­((tri-*iso*-pro­pyl­si­lyl)­eth­yn­yl)­ben­zene (bTIPS-Bz).^1520^ Subsequently, ^1^bTIPS-Bz* is formed through TTA, which can then undergo FRET sensitization of UVB-absorbing carbonyls, such as pinacolene, to generate isobutylene (Figure 260e). Although proof of the necessity of both **4CzIPN** and ^1^bTIPS-Bz to the reaction was shown, no yields were provided, nor any comparison of performance with other PCs.

### 23.3 TADF Compounds as PCs in Dual Catalysis

There is now a wide body of literature discussing the overlap of transition metal catalysis with photocatalysis, aptly termed metalla­photo­catal­ysis or metallo­photo­redox catalysis.^1521^ Specifically, **4CzIPN** has been shown to be compatible with this form of synergistic dual catalysis, working in tandem with nickel(II),^1522–1525^ palladium(II),^1526–1528^ cobalt(II),^1529–1532^ titanium(IV),^1533–1535^ iron(II and III),^1536,1537^ chromium(II)^1538,1539^ and copper(II)^1540–1542^ catalysts. Of these metal-based co-catalysts, examples with Nickel catalysts have been the most widely documented, typically involving C(sp^2^)-C(sp^3^) cross-coupling reactions. These dual metalla­photo­redox catalysis reactions have been reported to work via a reductive quenching mechanism for the CDCB PCs. **4CzIPN** typically performs well for this class of reactions^1522,1543,1544^ as it is capable of reducing the *in-situ* Ni(I) species (E_red_ ≈ −1.1 V vs SCE).^1545^

CDCB PCs can additionally perform well in oxidative quenching cycles, as is in operation when the co-catalyst used is the titanium(IV) complex TiCp_2_Cl_2._ For instance, in the Barbier allylation of aldehydes (Figure 260f),^1533^ **4CzIPN** can replicate the success of iridium(III) PCs, such as [Ir(dF(CF_3_)ppy)_2_(dtbbpy)]PF_6_, since photoreduction of TiCp_2_Cl is facile (E_red_ = −0.22 V vs SCE). Regeneration of the PC occurs through SET with a sacrificial reductant such as a Hantzsch ester (Hantzsch ester E_ox_ = 1.10 V vs SCE).^1546^

Aside from dual catalysis with transition metals, CDCB compounds have been documented to work alongside organic catalysts, including hydrogen atom transfer (HAT) catalysts,^1547–1550^ *N*-heterocyclic carbenes (NHCs)^1499,1551,1552^ and bromine catalysts like cinnamyl bromide.^1553^ The photocatalytic formation of carbanion equivalents has received attention over the last few years^1554^ as an accessible way to form C-C bonds without the need for stoichiometric reductants like low-valent metals. An example of this involves photo­car­box­ylation of benzylic C-H bonds (Figure 260g), which Meng *et al*. found to be possible using CDCB compounds as the PC in the presence of tri-*iso*-pro­pyl­si­lane­thiol as the HAT catalyst,^1555^ whereas both cationic and neutral iridium(III) PCs were incapable of completing the transformation. The success of the CDCB compounds here is thought to be due to their ability to form a strongly reducing *in-situ* PC, 2,3,4,6-tetra­(9H-car­ba­zol-9-yl)-5-(1-phen­yl­eth­yl)­ben­zo­nitrile, by photosubstitiution of one of the cyano groups. The resultant photosubstituted photoactive product, possessing significantly different photophysical and electrochemical properties, is hypothesised to be the active photocatalyst in reactions such as this.^1556^

### 23.4 Outlook

From these examples it is clear that although the use of organic TADF compounds as PCs is still in its relative infancy, especially compared to organometallic PCs, the results obtained thus far are promising. These early studies, using TADF materials unoptimized for photocatalytic activity, indicate that organic TADF compounds can act routinely as replacements for heavy metal PCs. Unquestionably, the CDCB family of D-A TADF PCs, most commonly exemplified by **4CzIPN**, has been most influential in this research area; however, there have been an increasing number of other examples, both D-A and MR-TADF systems, that suggests a generalisability of using TADF compounds, originally designed for use in OLEDs, as PCs. The wealth of TADF compounds available to synthetic chemists should provide broad scope for tuning the reactivities of a TADF PC for a specific transformation, providing similar versatility to the status quo organometallic PCs.

Additionally, the enhanced light absorption typically displayed by TADF materials in the visible light region compared to organometallic ruthenium(II) and iridium(III) polypyridyl complexes should be beneficial to improve reaction kinetics. Due to the vast number of published structures, it is likely that high-throughput experimentation would be needed to efficiently explore which TADF materials would be the most useful as PCs. Understanding how to appropriately tune the excited-state properties of TADF PCs to best support the desired photocatalytic pathways is imperative and will require detailed mechanistic understanding of both the TADF material and the reactions/substrates that they act upon. It will also be important to understand exactly how the TADF mechanism is implicated and influences photocatalysis reactions; indeed, investigating whether the triplet or singlet excited states, or both, are involved will require thorough mechanistic and spectroscopic studies. PC stability under photocatalytic reaction conditions is also an important consideration: popular organometallic and TADF PCs have been observed to photodegrade during photocatalytic reactions (a phenomenon that is easily observed via UV-Vis absorption spectroscopy).^1556–1558^ It would be highly desirable for new PCs to be designed showing improved photocatalytic stability. Alternatively, suspension on solid supports could enable the design of more recyclable heterogeneous PCs. With an enhanced understanding of the factors governing both the operational photocatalysis mechanism and the stability of the PC, it is expected that it would be possible to design TADF compounds for their explicit use as PCs, rather than simply repurposing known emitters that were developed for OLED applications.

## 24 Conclusions and Outlook

Over the last decade TADF has dominated the optoelectronic materials and applications literature, with over 3,500 articles and 1,000 patents published up to the end of 2022.^1559^ The majority of these reports have focussed on the design and exploitation of TADF materials for use in OLEDs, building upon the seminal 2012 work of Adachi and co-workers.^31^ Indeed, steady and sometimes breakthrough improvements in device performance have been made, with examples of blue, green, red, and white TADF OLED efficiencies (Sections 3–6) now rivalling PhOLED counterparts, especially at low brightness. Attention has also increasingly shifted to addressing outstanding barriers to commercialization, such as poor device lifetime and efficiency roll-off at practical brightnesses. Of particular note, specifically during the scope of this review, a solution to the undesirably broad emission spectra of D-A TADF emitters has emerged with the emergence of MR-TADF materials (Section 11), and especially their tandem application in hyperfluorescence device architectures (Sections 17 and 18).

Beyond the visible spectrum there is still considerable work required to improve the performance of near-IR TADF OLEDs, for which there are currently few studies or standout materials. This improved performance is required to unlock the distinct applications of near-IR emission, particularly for night vision, biological sensing and imaging. On the other wavelength extreme, despite a tremendous research effort focussed on developing high-performance deep-blue TADF OLEDs, their efficiencies, color purity, and roll-off behavior remain sub-optimal (or at best, individually optimized). The recent rise to prominence of the ‘blue-backplane’ concept makes progress at this particular color point all the more valuable as it can support next-generation performance in both display and lighting applications. Simultaneously, performance gains in UV-emitting OLEDs could support new applications in biological sterilisation, security, and lithography applications. Despite current challenges, we predict that sustained experimental effort, increasingly guided by theoretical studies, will result in improved emitter designs as the years progress, and a more nuanced understanding of the structural features that control the efficiency of the TADF process.

Alongside the mainstream research efforts linked to OLEDs, this review also documents the increasingly widespread use of TADF materials in broader research spheres. Improved performance of related LEC devices (Sections 16) employing TADF emitters continues in parallel with OLEDs, albeit with a more limited materials set leading to a more relaxed pace of development. TADF materials have also made promising early inroads as active materials in bioimaging (Section 21) and sensing (Section 20), and we predict a significant expansion of multi-disciplinary research activity in this specific area in the near future. The utility of TADF compounds in photocatalysis has also now become widely recognized (Section 23); however, the chemical space explored in terms of photocatalyst design remains stubbornly limited around a small set of established phthalonitrile-based OLED emitters. Assessing the wider panoply of reported TADF materials as photocatalysts will likely result in powerful additions to the synthetic chemist’s toolbox. Indeed, as the wide scope of this review attests, investigations into TADF materials design and various applications are only broadening and accelerating as this field reaches adolescence. We are certainly excited to witness further developments on what is surely a bright horizon.

## Supplementary Material



## Notes

Summary tables of the photophysical properties and device performance and the photoluminescence and electroluminescence properties of the emitters (PDF)
