# Supplementary material for: The Golden Age of Thermally Activated Delayed Fluorescence Materials: Design and Exploitation
Source: Chem Rev. 2024 Dec 12;124(24):13736–4110. doi: 10.1021/acs.chemrev.3c00755 (PMC12132800; doi:10.1021/acs.chemrev.3c00755)
Supplement: Supplementary file 1 [file cr3c00755_si_001.pdf]

The Golden Age of Thermally Activated Delayed Fluorescence Materials: Design and Exploitation

John Marques dos Santos[1†], David Hall[1†], Biju Basumatary[1], Megan Bryden[1], Dongyang Chen[1], Praveen Choudhary[1], Thomas Comerford[1], Ettore Crovini[1], Andrew Danos[5], Joydip De[1], Stefan Diesing[1,2], Mahni Fatahi[1], Maire Griffin[1], Abhishek Kumar Gupta[1], Hassan Hafeez[2], Lea Hämmerling[1], Emily Hanover[1,7], Janine Haug[3], Tabea Heil[1], Durai Karthik[1], Shiv Kumar[1,6], Oliver Lee[1,2], Haoyang Li[1], Fabien Lucas[1], Campbell Frank Ross Mackenzie[1], Aminata Mariko[1], Tomas Matulaitis[1], Francis Millward[1], Yoann Olivier[4], Quan Qi[1], Ifor D.W. Samuel[2], Nidhi Sharma[1,2], Changfeng Si[1], Leander Spierling[1], Pagidi Sudhakar[1], Dianming Sun[1], Eglė Tankelevičiūtė[1,2], Michele Duarte Tonet[1,2], Jingxiang Wang[1], Tao Wang[1], Sen Wu[1], Yan Xu[1], Le Zhang[1,2], Eli Zysman-Colman[1]*

[†] These authors contributed equally.

*Corresponding author email address: eli.zysman-colman@st-andrews.ac.uk

[1] Organic Semiconductor Centre, EaStCHEM School of Chemistry, University of St Andrews, St Andrews, Fife, KY169ST (UK).

[2] Organic Semiconductor Centre, SUPA School of Physics and Astronomy, University of St Andrews, St Andrews, Fife, KY169SS (UK).

[3] Institute of Organic Chemistry (IOC), Karlsruhe Institute of Technology (KIT), Fritz-Haber-Weg 6, 76131 Karlsruhe, Germany.

[4] Laboratory for Computational Modeling of Functional Materials, Namur Institute of Structured Matter, Université de Namur, Rue de Bruxelles, 61, 5000 Namur, Belgium.

[5] Department of Physics, Durham University, Durham DH1 3LE, UK.

[6] Department of Chemistry, University of Delhi, Delhi – 110007, India.

[7] EaStCHEM School of Chemistry, The University of Edinburgh, Edinburgh, EH9 3FJ.

Electronic Supporting Information

Contents

1. Tables 1

References: 89

1. Tables

Table **S1.** Summary of photophysical and device performance of blue TADF emitters reviewed in Section **3**.

**Compound**	**Medium**	**λ_{PL} / nm**	**Φ_{PL} / %**	**τ_d / µs**	**ΔE_{ST} / eV**	**λ_{EL} / nm**	**CIE_{XY}**	**EQE_{max} / %**	**Roll-off 100 cd m^{-2} / %**	**Roll-off 1000 cd m^{-2} / %**	**Ref**
**Triazine**											
***p*CzTPTZ**	10% in DPEPO	438	71	N.A.	0.36	N.A.	0.15.0.23	2.0	30	80	1-3
**BuCz-TRZ**	6% in DPEPO	439	83	68.1	0.29	459	0.15, 0.15	9.3	69	N.A.	4
**DPFCz-TRZ**	40% DPEPO	429[a]	88	0.64	0.21	445	0.15, 0.10	15.5	37	47	5
**IndCzpTr-1**	10% in mCBP	492[b]	75[b]	35	0.13[g]	472	0.17, 0.27	14.5	24	N.A.	6
**IndCzpTr-2**	20% in mCBP	510[b]	71[b]	34	0.11[g]	496	0.23, 0.50	30.0	49	N.A.	6
**DBTCz-Trz**	15 wt% in DPEPO	461[g]	90	13.1	0.23[g]	472	0.17, 0.28	21.7	~65	88	7
**DBFCz-Trz**	15 wt% in DPEPO	460[g]	89	12.8	0.23[g]	472	0.17, 0.28	21.6	~63	85	7
**BDBFCz-Trz**	15 wt% in DPEPO	465[g]	98	10.7	0.20[g]	488	0.18, 0.35	21.5	~36	80	7
**Trz-BFCzCz**	20% in DPEPO	455	75	37.4	0.13	N.A.	0.18, 0.32	23.3	N.A.	43.8	8
**DCBTRZ**	10% in CBP	435	39	0.1	0.39[e]	440	0.15, 0.059	6.7	N.A.	N.A.	9
**PPCTRZ**	10% in CBP	411	38	0.3	0.38[e]	442	0.15, 0.063	6.5	N.A.	N.A.	9
**InCz23FlTz**	10% in DPEPO	470	86	98	0.19	468	0.15, 0.18	17.2	63	82	10
**InCz23DPhTz**	10% in DPEPO	471	91	87	0.17	472	0.15, 0.22	17.9	55	83	10
**InCz23DMeTz**	10% in DPEPO	488	93	77	0.15	480	0.16, 0.30	22.8	43	75	10
**InCz34DPhTz**	10% in DPEPO	475	98	70	0.11	472	0.15, 0.24	25.9	45	80	10
**TrzBFCz**	10% in DPEPO<br>20% in DPEPO	385[b]	99	26.3	0.27	447<br>451	0.15, 0.09<br>0.15, 0.10	19.0<br>18.0	N.A.<br>N.A.	77<br>55	11
**TrzCNBFCz**	10% in DPEPO<br>20% in DPEPO	407[b]	100	9.4	0.13	473<br>481	0.16, 0.24<br>0.17, 0.31	13.1<br>20.9	N.A.<br>N.A.	72<br>37	11
**dBFCzTrz**	20% DPEPO	425[a]	90	30.4	0.13	476	0.16, 0.27	22.6	N.A.	46	12
**dBFCzCNTrz**	20% DPEPO	475[a]	80	4.9	0.09	497	0.22, 0.47	27.5	N.A.	12	12
**Bzimim_FCNTz**	20% in DPEPO	461[e]	99	7.9	0.17	N.A.	0.17, 0.27	22.6	11	N.A.	13
**Cz-TRZ2**	6 wt% in DPEPO	465[a]	98	3.5	0.07[a]	N.A.	0.16, 0.24	22.0	23	42	14
**Cz-TRZ3**	6 wt% in DPEPO	435[a]	92	13.0	0.17[a]	N.A.	0.15, 0.10	19.2	23	68	14
**Cz-TRZ4**	6 wt% in DPEPO	432[a]	85	10.3	0.15[a]	N.A.	0.15, 0.10	18.3	19	49	14
**TCZ-TRZ(Me)**	15% in DPEPO	457	40	38	0.27	456	0.16, 0.14	10.4	N.A.	N.A.	15
**TCZ-TRZ(Me')**	15% in DPEPO	451	32	51	0.16	448	0.17, 0.18	11.1	N.A.	N.A.	15
**TRZ-CF**	20% in DPEPO	474	86	7.3	0.22	476	0.17, 0.27	20	N.A.	43	16
**TRZ-CzF**	20% in	458	69	11	0.31	460	0.15, 0.16	13.3	N.A.	72	16

	DPEPO										
**PhBuCz-TRZ**	6% in DPEPO	440[e]	82	44.1	0.27	458	0.15, 0.16	12.1	49	N.A.	4
**PyBuCz-TRZ**	6% in DPEPO	434[e]	87	35.8	0.30	455	0.15, 0.13	15.3	57	N.A.	4
**IDB-TRZ**	Neat	481	99	1.7	0.02[f]	487	0.15, 0.36	22.8	1	17	17
**IDB-TRZ-Me**	Neat	466	98	2.4	0.02[f]	474	0.14, 0.23	21.3	14	62	17
**IDB-TRZ-Me$_2$**	20% in PPF	472[a]	98	28	0.093	496	0.21, 0.44	28.3	14	N.A.	17
**IDB-TRZ-Me$_4$**	20% in PPF	456[a]	37	6	~0	484	0.20, 0.30	16.4	12	N.A.	17
**TAZ-1**	20% in PPF	468	88	8.4	0.15	478	0.16, 0.25	17.7	10	40	18
**TAZ-2**	20% in PPF	476	100	6.6	0.10	479	0.16, 0.27	21.2	10	38	18
**o12BFCzTrz**	20% DPEPO	473[a]	83	4.1	0.015	478	0.16, 0.29	19.2	N.A.	11	19
**o23BFCzTrz**	20% DPEPO	476[a]	86	4.2	0.042	484	0.18, 0.39	21.1	N.A.	20	19
**oBFCzTrz**	20% DPEPO	477[a]	89	3.1	0.002	488	0.17, 0.34	22.9	N.A.	13	19
**PPCzTrz**	20% in DPEPO	444	93	25.0	0.16	N.A.	N.A.	10	N.A.	N.A.	20
**DMAC-TRZ**	8% in mCPCN	495	90	1.9	0.05	-	-	26.5	5	N.A.	21, 22
***a*-DMAc-TRZ**	20% in DPEPO	479	86	4.1	0.20	N.A.	0.19, 0.34	28.9	56	88	21
**DTPPDDA**	8wt% in mCP: TSPO1	439	38	0.02	0.04	N.A.	0.15, 0.09	4.7	50	N.A.	23
**TTSA**	10% in mCP:TSPO1	481	99	3.5	0.06	480	0.16, 0.28	27.9	19	N.A.	24
**TXSA**	10% in mCP:TSPO1	475	91	3.7	0.06	476	0.15, 0.25	20.7	22	N.A.	24
**TTAZ**	10% in DPEPO	465	68	4.7	0.16[e]	464	0.15, 0.16	23.7	39	N.A.	24
**TXAZ**	10% in DPEPO	460	50	4.6	0.18[e]	456	0.15, 0.13	16.0	47	N.A.	24
**DTPDDA**	10% in mCP:TSPO1	468	70	5.1	0.16	N.A.	N.A.	N.A.	N.A.	N.A.	25
**SAzTrz**	10 wt% in DPEPO	469	68	165	0.20	468	0.15, 0.18	20.6	64	N.A.	25
**TspiroS-TRZ**	30% in DPEPO	470[a]	81.6	3.03	0.05[a]	481	0.17, 0.33	31.1	6.6	29	26
**TspiroF-TRZ**	30% in DPEPO	479[a]	74.8	4.57	0.08[a]	493	0.19, 0.39	28.1	1.4	18	26
**DspiroAc-TRZ**	Non-Doped	482	83	3.2	0.04	484	0.18, 0.38	25.7	8	36	27
**CzTrzPh**	15% in CNmCBPCN	458	34	15.3	0.16	459	0.16, 0.19	12.5	25[f]	N.A.	28
**CzTrzBp**	15% in CNmCBPCN	457	35	30.3	0.31	448	0.16, 0.14	9.2	48[f]	N.A.	28
**CzTrzDbf**	15% in CNmCBPCN	466	49	26.3	0.26	457	0.16, 0.19	12.4	51[f]	N.A.	28
**MA-TA**	10% in CzSi	469	99	18.3	0.14	N.A.	0.15, 0.19	22.1	37	N.A.	29
**FA-TA**	10% in	454	76	44.4	0.16	N.A.	0.15, 0.13	11.2	N.A.	N.A.	29

	CzSi									
**PA-TA**	10% in CzSi	451	70	69.5	0.17	N.A.	0.15, 0.10	6.7	N.A.	N.A.	29
**moTrSAc**	10% DPEPO	482	70	3.4	0.01	472	0.17, 0.27	21.3	N.A.	N.A.	30
**motmTrSAc**	10% DPEPO	469	51	3.0	0.01	468	0.16, 0.22	19.5	N.A.	N.A.	30
**oCzPO$_2$TPTZ**	10% in DPEPO	472	25	15.7	0.01	N.A.	0.17, 0.27	6.7	15	66	31
**mCzPO$_2$TPTZ**	10% in DPEPO	476	53	24.5	0.03	N.A.	0.16, 0.20	11.6	38	74	31
**pCzPO$_2$TPTZ**	10% in DPEPO	482	74	19.4	0.01	N.A.	0.15, 0.27	20.9	22	51	31
**CzDCbTrz**	6 wt% in DPEPO	469[a]	85	7.2	0.12	471	0.16, 0.23	22.0	~23	57	32
**DCzCbTrz**	40% in DBFPO	451[a]	79	15.6	0.13[a]	N.A.	0.16, 0.23	22.1	~17	54	33
**Tris-Cz-TRZ**	7 wt% in mCP	457	71	5.0	0.03	468	0.15, 0.22	16.5	0	5	34
**23CT**	30% in DPEPO	N.A.	80	8.7	N.A.	N.A.	0.17, 0.33	21.8	0	5	35
**24CT**	30% in DPEPO	N.A.	92	21, 241	N.A.	N.A.	0.15, 0.26	22.4	9	32	35
**34CT**	30% in DPEPO	N.A.	N.A.	N.A.	N.A.	N/A	0.15, 0.17	13.3	29	58	35
**245CzTrz**	30% DPEPO	450	98	9.7	0.17	490	0.17, 0.39	22	N.A.	37	36
**5Cz-Trz**	15% mCBP	486	99	2.1	0.02	486	N.A.	29.3	N.A.	3	37
**p2Cz2Trz**	30% in DPEPO	430[h]	82[i]	16.6[i]	0.18[h]	534	0.39, 0.58	12.5	N.A.	26	38
**m2Cz2Trz**	30% in DPEPO	410[h]	91[i]	12.2[i]	0.09[h]	493	0.20, 0.47	18.5	N.A.	14	38
**TrzoCz**	20% DPEPO	450	96	20	0.2	484	0.15, 0.32	28	15	60	39
**DPA-MeTRZ**	12% in mCPCN	466	100	N.A.	0.30	474	0.14, 0.23	19.1	65[f]	N.A.	40
**c-NN-TRZ**	12% in mCPCN	476	100	N.A.	0.32	481	0.14, 0.32	26.3	70[f]	N.A.	40
**c-NN-MeTRZ**	12% in mCPCN	467	100	N.A.	0.24	467	0.14, 0.22	32.2	59[f]	N.A.	40
**TTT-Ac**	20% in mCP	426	63	27.2	0.35	470	0.16, 0.21	9.2	N.A.	N.A.	41
**TTT-2Ac**	20% in mCP	446	47	20.0	0.19	474	0.17, 0.26	8.1	N.A.	N.A.	41
**TTT-DMAC**	5% in CzSi	468	21	19.5	0.20	480	0.19, 0.29	6.2	N.A.	5.3	42
**TTT-PXZ**	5% in CzSi	522	39	36.0	0.07	530	0.32, 0.52	1.9	N.A.	N.A.	42
**TTT-Ph-Cz**	3% in DPEPO	422	42	N.A.	0.43	432	0.16, 0.07	3.2	N.A.	N.A.	42
**TTT-Ph-Ac**	3% in DPEPO	464	79	142.7	0.24	480	0.16, 0.27	9.7	N.A.	N.A.	42
**TTT-Ph-BAc**	3% in DPEPO	470	32	150.7	0.09	486	0.20, 0.34	3.8	N.A.	N.A.	42
**3,4,5-3TCz-TTT**	15% in CzSi	485	80	3100	0.21	N.A.	0.17, 0.28	5.8	N.A.	N.A.	43
**3DMAC-TTT**	15% in CzSi	475	79	4700	0.27	N.A.	0.16, 0.23	11.0	N.A.	N.A.	43
**Pyrimidine, pyrazine**											
**Ac-1MHPM**	20 wt% in DPEPO	481	75	50	0.23	N.A.	0.17, 0.28	24	21	53	44
**Ac-2MHPM**	20% in DPEPO	477	71	44	0.22	N.A.	0.17, 0.27	19.8	20	52	44
**Ac-**	20% in	454	47	45	0.24	N.A.	0.16, 0.15	17.8	42	N.A.	44

**3MHPM**	DPEPO										
**3CbzPYR**	10% DPEPO	464	81	868	0.32	473	0.16, 0.23	19.7	55%	77%	45
**2SPAc-HPM**	20 wt% in DPEPO	469[a]	97	52.2	0.15[a]	487	0.18, 0.34	25.6	26	53	46
**2SPAc-MPM**	20 wt% in DPEPO	460[a]	82	53.3	0.19[a]	479	0.17, 0.29	24.3	34	62	46
**2SPAc-PPM**	20 wt% in DPEPO	464[a]	97	56.9	0.16[a]	484	0.18, 0.32	31.5	42	66	46
**MFAc-PPM**	18 wt% in PPF	464	87	38	0.25	470	0.16, 0.23	20.4	24	~65	47
**4,6-PhPMAF**	22 DPEPO	448	17	301.15	0.27	458	0.15, 0.11	2.95	N.A.	N.A.	48
**Ac-26DPPM**	10% in DPEPO	491	81	87	0.17	N.A.	0.18, 0.33	19.3	38	67	49
**CzAc-26DPPM**	10% in DPEPO	488	81	55	0.12	N.A.	0.21, 0.38	23.7	37	61	49
**2NPMAF**	22 wt% in DPEPO	482	73	1.8	0.11	481	0.19, 0.34	23.6	23	~34	50
**DPAc-4PyPM**	10 wt% in DPEPO	486	86	22.7	0.23	484	0.19, 0.36	24.3	28	65	51
**DPAc-6PyPM**	10 wt% in DPEPO	468	83	16.3	0.20	472	0.15, 0.24	22.4	50	88	51
**SFI34pPM**	10% in DPEPO	424[a]	74	118	0.24	460	0.15, 0.09	8.2	N.A.	N.A.	52
**SFI23pPM**	20% in DPEPO	414[a]	72	189	0.36	460	0.15, 0.10	5.1	N.A.	N.A.	52
**pBFCz-2,6DPPM**	10 wt% in DPEPO	437	71	200	0.23[b]	N.A.	0.15, 0.05	6.2	~29	N.A.	53
**pBTCz-2,6DPPM**	10 wt% in DPEPO	433	75	383	0.24[b]	N.A.	0.15, 0.05	5.4	N.A.	N.A.	53
**BFCZPZ1**	7% PPT	430	68	910	0.37	436	0.15, 0.06	6.5	20	27	54
**BFCZPZ2**	7% PPT	462	91	420	0.31	464	0.15, 0.16	21.3	53	76	54
**BTCZPZ1**	7% PPT	477	91	90	0.24	472	0.16, 0.26	21.1	26	58	54
**BTCZPZ2**	7% PPT	462	98	440	0.31	468	0.15, 0.18	19.7	50	70	54
**CZ9CZPZ**	7% PPT	458	94	300	0.31	468	0.16, 0.20	20	48	70	54
**DCz-ND**	20% in DPEPO	475	46	6.0	0.12	469	0.15, 0.21	18.1	44	70	55
**DCz-ND-Cz**	20% in DPEPO	471	74	4.4	0.13	468	0.16, 0.21	20.8	36	54	55
**DCz-ND-DCz**	20% in DPEPO	468	72	7.0	0.13	464	0.16, 0.20	20.8	35	67	55
**CN1**	Neat	482	76	2.4	0.01	481	0.16, 0.27	8.4	N.A.	N.A.	56
**CN4**	Neat	490	27	1.8	0.05	476	0.17, 0.24	5.5	N.A.	N.A.	56
**Boron**											
**Cz*o*B**	20 wt% in DPEPO	466	84	56	0.12	N.A.	0.14, 0.15	22.6	19	77	57
**CzAZB**	10 wt% in mCP	452	99	N.A.	0.31	454	0.14, 0.10	5.5	~20	~35	58
**tmCzAZB**	10 wt% in mCP	451	56	162	0.26	464	0.14, 0.15	12.4	~56	~90	58
**dmAcAZB**	10 wt% in mCP	469	95	197	0.11	469	0.14, 0.19	20.8	~38	~73	58
**MPAc-BS**	Neat film	487	99	1.7	0.02	487	0.15, 0.36	22.8	1	N.A.	59
**MPAc-BO**	Neat film	466	98	2.4	0.02	474	0.14, 0.23	21.3	14	N.A.	59
**MCz-BS**	50 wt% in PPF	476	92	1.9	0.11	478	0.14, 0.26	21.6	1	6	60
**MPASi-BS**	50 wt% in PPF	479	100	2.7	0.08	478	0.14, 0.26	27.6	0.1	5	60
**MFASi-BS**	50 wt% in PPF	483	100	5.3	0.06	484	0.14, 0.32	23.9	1	8	60

**MPAGe-BS**	50 wt% in PPF	468	92	10.4	0.11	476	0.14, 0.22	15.7	1	16	60
**PXB-DI**	20 wt% in PPBI	470	79	3.3	0.09	470	0.16, 0.34	37.4	N.A.	15	61
**PXB-mIC**	20 wt% in PPBI	425	51	4	0.19	450	0.15, 0.08	12.5	N.A.	58	61
**QBO**	20 wt% in PPF	455	83	0.65	0.01	460	0.14, 0.12	20.5	20.2	17.7	62
**MCz-BOBO**	20 wt% in PPF	476	100	0.78	0.01	473	0.13, 0.20	20.1	1	12	63
**MCz-BSBS**	20 wt% in PPF	483	93	2.7	0.17	484	0.14, 0.33	25.9	9	25	63
**SAC-SBS**	20 wt% in PPF	491	81	22	0.12	489	0.17, 0.39	20.9	16	56	64
**SAC-OBO**	20 wt% PPF	470	28	140	0.30	471	0.16, 0.22	5.2	75	N.A.	64
**NOBF2-Cz**	10 wt% in DPEPO	475	99	132	0.20	467[c]	0.14, 0.16[c]	11.0	N.A.	N.A.	65
**NOBF2-DTCz**	10 wt% in DPEPO	488	74	126	0.20	471[c]	0.14, 0.21[c]	12.7	N.A.	N.A.	65
**NOBF2-DPCz**	10 wt% in DPEPO	496	70	110	0.22	483[c]	0.14, 0.28[c]	15.8	N.A.	N.A.	65
**TDBA–Ac**	20 wt% in PPBI	458	93	1	0.06	448	0.15, 0.06	21.5	N.A.	55	66
**TDBA–DI**	20 wt% in PPBI	456	99	6.2	0.11	460	0.14, 0.15	32.2	N.A.	17	66
**DBA-DI**	30 wt% in mCBP	467[a]	95.3	1.25	0.03[a]	470	0.15, 0.33	23.1	N.A.	1	67
***p*MDBA-DI**	30 wt% DBFPO	460[a]	97.8	1.60[a]	0.07[a]	483	0.15, 0.31	33.1	N.A.	32.3	68
***m*MDBA-DI**	30 wt% DBFPO	451[a]	97.3	1.90[a]	0.12[a]	474	0.14, 0.23	32.8	N.A.	28.4	68
**FTAT-MBO**	20 wt% in mCP	448	55	3.6	0.16	445	0.15, 0.08	5.1	N.A.	N.A.	69
**FTAT-HBO**	20 wt% in mCP	454	66	3.5	0.11	458	0.15, 0.12	7.7	N.A.	N.A.	69
**FTAT-FBO**	20 wt% in mCP	473	90	1.8	0.04	473	0.15, 0.25	17.3	N.A.	N.A.	69
**TB-3Cz**	Non-doped	433	99	4.4	0.06	424	0.17, 0.07	9.9	N.A.	N.A.	70
**TB-P3Cz**	Non-doped	460	93	4.7	0.11	428	0.15, 0.08	6.1	N.A.	N.A.	70
**TB-P3Cz**	20 wt% DBFPO	433[a]	88	9.32	0.23[a]	470	0.14, 0.19	29.1	N.A.	54	70
**M3CzB**	20 wt% DBFPO	445[a]	93	7.84	0.14[a]	478	0.14, 0.26	30.7	N.A.	30	71
**TMCz-BO**	30 wt% in PPF	467	98	0.75	0.02	471	0.14, 0.18	20.7	2	16	72
**TMCz-3P**	30 wt% in PPF	477	76	14.5	0.13	479	0.14, 0.26	20.4	10	37	72
**TDBA-PAS**	20 wt% in DPEPO	427[a]	92.3	2.49	0.06	435	0.155, 0.042	22.35	17.99	N.A.	73
**TDBA-DPAC**	20 wt% in DPEPO	444[a]	87.8	1.63	0.02	450	0.148, 0.085	24.61	11.03	2.42	73
**OBO-I**	20 wt% in PPF	451	81	1.6	0.07	457	0.14, 0.10	21.7	20.6	16.2	74
**OBO-II**	20 wt% in PPF	465	98	1.7	0.13	464	0.14, 0.13	31.7	29.0	20.8	74
**TDBA-SBA**	20 wt% in DBFPO	450	89	1.47	0.01[a]	467	0.13, 0.15	29.3	28.5	26.0	75
**2TDBA-SBA**	20 wt% in DBFPO	448	87	1.38	0.01[a]	475	0.13, 0.21	18.3	17.3	15.0	75
**p-AC-DBNA**	10 wt% BCPO	496	96	1.8	0.09	488	0.17, 0.36	20.5	8	20	76
**TDBA-Cz**	10 wt% in DPEPO	456	100	6.1[b]	0.14[a]	466	0.134, 0.129	31.1	N.A.	N.A.	77
**DBA-Cz**	10 wt% in	450	90	8.2[b]	0.03[a]	467	0.136,	30.3	N.A.	N.A.	77

	DPEPO						0.147				
**3TBO**	30 wt% in mCBP	476	78.4	1.42	78.4	484	0.120, 0.294	17.3	N.A.	10.3	78
**5TBO**	30 wt% in mCBP	466	96.7	1.90	96.7	484	0.125, 0.275	26.2	N.A.	16.7	78
**PhCz-TOSBA**	10 wt% in 2,6-DczPPy	454	39.3	114.37	0.23[a]	456	0.14, 0.15	16.69	N.A.	N.A.	79
**TPA-TOSBA**	10 wt% in 2,6-DczPPy	467	42.8	140.02	0.36[a]	456	0.14, 0.12	16.65	N.A.	N.A.	79
**Nitrile**											
**δ-2CbPN**	20 wt% in mCP	453[a]	93	180	0.13	486	0.19, 0.34	22.5	~24	66	32
**DCzBN1**	10% in DPEPO	415	28	18.0	0.31[a]	418	0.15, 0.05	2.5	N.A.	N.A.	80
**DCzBN2**	10% in DPEPO	432	66	11.2	0.22[a]	436	0.15, 0.07	7.7	N.A.	N.A.	80
**DCzBN3**	10% in DPEPO	424	76	13.5	0.26[a]	428	0.16, 0.06	10.3	N.A.	N.A.	80
**DCzBN4**	10% in DPEPO	462	86	5.5	0.14[a]	468	0.16, 0.23	18.0	42	77	80
**DPAc-DCzBN**	20 wt% DPEPO	476	71	3.03	0.13	472	0.17, 0.25	22.8	21	~55	81
**DPAc-DCzBN**	Non-doped	482	51	5.46	N.A.	476	0.16, 0.26	22	2.5	~50	81
**DPAc-DtCzBN**	30 wt% DPEPO	472	64	3.08	0.08	456	0.16, 0.15	23.1	20.7	N.A.	81
**DPAc-DtCzBN**	Non-doped	471	55	7.12	N.A.	460	0.16, 0.19	10.1	16.8	N.A.	81
**DBFCzCN**	20% DPEPO	451[d]	100	89	0.28	479	0.15, 0.29	25.2	N.A.	55	82
**3Ph2CzCzBN**	20 wt% in mCBP	464[a]	91	10[a]	0.19[a]	486	0.18, 0.39	17.9	2	4	83
**Cy-2Cz**	8 wt% in DPEPO	420[a]	57	9.4	0.18	445	0.16, 0.10	11.9	8	~21	84
**2PhCz2CzBN**	20% mCBP	470	86	9.6	0.17	464	0.15, 0.20	22.6	35	56	85
**2PhCz2CzBN**	Non-doped	N.A.	45	N.A.	N.A.	480	0.20, 0.33	3.9	N.A.	N.A.	85
**2tCz2CzBN**	30% mCBP	470	87	15.7	0.15	464	0.15, 0.19	23.8	28	48	85
**2tCz2CzBN**	Non-doped	N.A.	66	N.A.	N.A.	470	0.17, 0.25	21.6	29	50	85
**2Cz2tCzBn**	Non-doped	488	78	3.9	0.13	488	0.21, 0.42	25.8	N.A.	75	86
**p4TCzPhBN**	15% DPEPO	457	64	9.2	0.1	468	0.15, 0.19	22.8	N.A.	11	87
**3Cz3BN**	20 wt% in mCBP	412[a]	11[a]	N.A.	0.24[a]	N.A.	N.A.	N.A.	N.A.	N.A.	88
**2Cz4BN**	20 wt% in mCBP	416[a]	18[a]	N.A.	0.33[a]	N.A.	N.A.	N.A.	N.A.	N.A.	88
**4Cz2BN**	20 wt% in mCBP	439[a]	13[a]	27[a]	0.22[a]	N.A.	N.A.	N.A.	N.A.	N.A.	88
**3tBuCz3BN**	20 wt% in mCBP	425[a]	11[a]	N.A.	0.20[a]	N.A.	N.A.	N.A.	N.A.	N.A.	88
**2tBuCz4BN**	20 wt% in mCBP	435[a]	19[a]	N.A.	0.19[a]	N.A.	N.A.	N.A.	N.A.	N.A.	88
**4tBuCz2BN**	20 wt% in mCBP	456[a]	45[a]	29[a]	0.07[a]	N.A.	N.A.	N.A.	N.A.	N.A.	88
**3PhCz3BN**	20 wt% in mCBP	438[a]	13[a]	24[a]	0.21[a]	N.A.	N.A.	N.A.	N.A.	N.A.	88
**2PhCz4BN**	20 wt% in mCBP	455[a]	16[a]	N.A.	0.18[a]	N.A.	N.A.	N.A.	N.A.	N.A.	88

**4PhCz2BN**	20 wt% in mCBP	479	87	1.6	0.04	477	0.15, 0.23	18.2	16.8	14.5	88
**T-CNDF-T-tCz**	Non-doped	477	76	7.79	0.03	484	0.19, 0.35	21	N.A.	N.A.	89
**S-CNDF-S-tCz**	Non-doped	445	42	54.3	0.24	466	0.16, 0.18	2.6	N.A.	N.A.	89
					**Oxadiazole**						
**2CzdOXD 4CF$_3$Ph**	10% in PPT	474	55	4,500 26,000	0.32	474	0.17, 0.25	11.2	78	N.A.	90
**2CzdOXD Ph**	10% in PPT	454	44	2,400 18,000	0.25	455	0.16, 0.15	6.8	71	79	90
**2CzdOXD Me**	10% in mCP	435	29	1,000 4,800	0.30	446	0.16, 0.12	4.7	45	62	90
**2CzdOXD 4MeOPh**	10% in mCP	449	15	1,700 10,000	0.46	446	0.15, 0.11	6.6	70	88	90
**2,4,6CzDPO**	25% in DPEPO	472	32	309	0.12[a]		0.17, 0.30	6.1	~57	~89	91
**2,3,4,6CzDPO**	25% in DPEPO	484	54	166	0.07[a]	N.A.	0.18, 0.36	17.8	~36	~71	91
**2,3,4,5,6CzDPO**	25% in DPEPO	479	61	75	0.12[a]	N.A.	0.16, 0.29	24.4	~23	62	91
**5*t*CzDPO**	12.5% in DPEPO	474	20[a]	71	0.01[a]	N.A.	0.18, 0.36	29	N.A.	13	92
**5CzMPO**	12.5% in DPEPO	462	21[a]	105	0.12[a]	N.A.	0.21, 0.32	15	N.A.	7.2	92
**5*t*CzMPO**	12.5% in DPEPO	466	30[a]	69	0.03[a]	N.A.	0.18, 0.34	23	N.A.	16	92
**BAcOX**	10% in DPEPO	472	93	84.1	0.26	475	0.16, 0.24	22.3	N.A.	N.A.	93
					**Sulfone**						
**ACR-BPSBP**	10% in DPEPO	460	82	5.0	0.14	468	0.16, 0.21	24.6	~47	N/A	94
**DAc-DSO2**	30 wt% in DPEPO	N.A.	N.A.	2.1	0.01	N.A.	0.18, 0.33	25.4	4	20	95
**DDPhMesA-DDPS**	10 wt% in PMMA	450	79	70	N.A.	456	0.16, 0.08	8.5	2	9	96
**TXADO-Spiro-DMACF**	nondopen	445	42	106	0.28	444	0.16, 0.09	5.3	N.A.	N.A.	97
**SBA-2DPS**	30% in DPEPO	459	60	4.3	0.09	467	0.15, 0.20	25.5	10	39	98
**DMA-ThX**	mCBP	448[a]	N.A.	0.08[b]	0.07[b]	458	0.14, 0.10	2.9	0	N.A.	99
**DMAC-mTXO2**	35 wt% DPEPO	440[a]	88	3	0.05	455	0.15, 0.18	22.6	0.4	11.9	100
**CZ-MPS**	10% in TCTA+10% in CzSi	392	46	4.85	0.49	389	0.18, 0.06	9.3	N.A.	N.A.	101
**DMAC2PTO**	15% DPEPO	452	62	4.2	0.03	448	0.15, 0.11	15.2	N.A.	N.A.	102
**3,4'ACSO2**	10% DPEPO	464[a]	77	5.4	0.09	480	0.17, 0.29	20.5	10	35	103
**3SO2**	5% in 26DCzPPY	414	17	8.6	0.18	413	0.15, 0.04	2.6	N.A.	N.A.	104

**ICz-DPS**	10 wt% DPEPO	438	72	616.1	0.22	435	0.15, 0.08	11.6	N.A.	6	105
**DPS-BF-Ac**	25 wt% mCP	477	74	14.5	0.03	482	0.16, 0.28	24.7	~60	N.A.	106
**SeDF-B**	10 wt% in mCBP	490	3	18.5	0.08	490	0.17, 0.14	25.6	N.A.	10	107
**D-A-Pi**	10 wt% in DPEPO	448	62	6.6	0.10	447	0.15, 0.11	17.2	48	N.A.	108
**D-A-MeO**	10 wt% in DPEPO	450	82	6.2	0.08	451	0.16, 0.14	21.3	25	57	108
**D-A-CH_3**	10 wt% in DPEPO	457	81	5.2	0.06	456	0.16, 0.15	21.2	24	53	108
**D-A-H**	10 wt% in DPEPO	460	82	4.8	0.04	461	0.16, 0.16	21.5	26	60	108
**D-A-CF_3**	10 wt% in DPEPO	478	95	3.6	0.01	478	0.17, 0.27	23.4	9	38	108
**D-A-CN**	10 wt% in DPEPO	488	96	2.1	0.01	489	0.18, 0.37	23.9	14	40	108
**pCz-BTO**	10 wt% in DPEPO	438	59	0.2	0.18	440	0.15, 0.09	9.5	N.A.	N.A.	109
**Ketone**											
**MXAc-BF**	50% PPF	478	88	4.1	0.08[j]	478	0.17, 0.29	16.2	3	26	110
**MXAc-CM**	50% PPF	484	77	2.8	0.08[j]	478	0.16, 0.29	15.0	3	26	110
**XAc-CM**	50% PPF	462	74	3.9	0.11[j]	462	0.15, 0.19	12.1	4	41	110
**CzX**	6 wt% in PPF	453[a]	54[a]	N.A.	N.A.	482	0.18, 0.32	19.9	~25	~60	111
**CCX-II**	6 wt% in PPF	460	97	9.7	0.03	471	0.15, 0.22	25.9	13	34	112
**3DPyM-pDTC**	7wt% in mCBP	464	98	10	0.02	464	0.14, 0.18	31.9	18	~49	113
**2BPy-mDTC**	7 wt% in mCBP	480[a]	92	8.3	0.05[a]	476	0.15, 0.28	24.6	~13	~52	114
**3BPy-pDTC**	7 wt% in mCBP	453[a]	93	25.9	0.19	458	0.14, 0.13	25.3	29	80	114
**5tCzMeB**	20% DPEPO	483	99	1.8[a]	0.08	481	0.19, 0.32	24.6	33	N.A.	115
**QXT**	20 wt% PPF	489	96	1.0	0.02	481	0.16, 0.30	24.9	2	13	116
**33PCX**	20 wt% PPF	472	92	5.2	0.16[a]	469	0.16, 0.25	27.5	1	33	117
**CP-BP-SFAC**	25 wt% in DPEPO	481	99	5.5	0.06	484	0.17, 0.30	36.6	~12	~20	118
**mCP-BP-SFAC**	20 wt% in DPEPO	478	99	5.5	0.06	478	0.16, 0.28	38.0	~12	~20	118
**TCP-BP-SFAC**	25 wt% in DPEPO	482	99	5.6	0.06	480	0.16, 0.28	38.6	~12	~20	118
**Other acceptors**											
***m*2tBCzPO**	30% in DPETPO	472	75	16.7	0.02[j]	460	0.16, 0.17	21.0	7	26	119
**4tBCzDPDPO2A**	30% in DBFDPO	479	81	19.3	0.03	472	0.18, 0.30	23.7	6	22	120
**5CzDPhCF3**	10 wt% in DPEPO	435[b]	27[a]	0.14	0.23	431	0.16, 0.07	2	75	N.A.	121
**4CzIPN-CF3**	1% in SimCP:oCF3-T2T	492	77	3.6	0.08	487	0.20, 0.41	23.1	N.A.	10	122
**TN4T-PCZ**	6 wt% in 2,6-DCzppy	411[a]	87	N.A.	0.10	420	0.16, 0.03	20.4	N.A.	N.A.	123
**ICzDAc**	10wt% in DPEPO	446	96	8.5	0.17	477	0.15, 0.16	19.7	N.A.	50	124
**PhICzDP**	10%	468	96	249	0.12	482	0.13, 0.32	30.4	43	N.A.	125

A	DPEPO									
**PA(OOQ)**	5 wt% in mCBP	489	74	1480	0.35	488	0.19, 0.37	19.5	73	84	126

[a] Toluene, [b] Neat film, [c] 10% in mCBP, [d] THF, [e] 10% in mCP:TSPO1, [f] 50% in PPF, [g] MeTHF, [h] 1% in PMMA, [i] 10% in DPEPO, [j] 25% in PPF, N.A. = Not Available.

Table **S2**. Summary of photophysical and device performance of green TADF emitters reviewed in Section **4**.

**Compound**	**Medium**	**λ_{PL} / nm**	**Φ_{PL} / %**	**τ_D / μs**	**ΔE_{ST} / eV**	**λ_{EL} / nm**	**CIE_{XY}**	**EQE_{MAX} / %**	**Roll-off 100 cd m^{-2} / %**	**Roll-off 1000 cd m^{-2} / %**	**Ref**
**Cyano containing acceptors**											
**mPTBC**	2 wt% mCP	518 [b]	58	9.6	0.006	516	0.29, 0.56	18.1	0.6	13	127
**oPTBC**	2 wt% mCP	561 [b]	58	4.6	0.007	540	0.35, 0.56	17.8	5	18	127
**oAcTBC**	10 wt% mCP	509 [b]	84	17.4	0.03	512	0.23, 0.53	20.9	≈6	17	128
**oSpTBC**	16 wt% mCP	497 [b]	93	16.9	0.02	508	0.21, 0.49	26.8	≈17	29	128
**mAcTBC**	27 wt% mCP	480 [b]	77	15.5	0.02	496	0.19, 0.44	19.2	≈17	37	128
**mSpTBC**	24 wt% mCP	475 [b]	65	13.3	0.01	492	0.17, 0.37	18.9	≈8	26	128
**4tBuCzPN**	25 wt% DPEPO	476	74	4.0	0.05	500	N/A	20.8	N/A	N/A	129
**3Cz2DPhCzBN**	20 wt% mCBP	495	80	5.7	0.15 [b]	≈500	0.21, 0.44	20.9	≈0	0.5	130
**PyPhBN**	15 wt% PPT	486 [b]	92 [b]	10.5 [b]	0.13 [b]	501	0.21, 0.46	20.6	4	17	131
**4mCzBN-BP**	10 wt% mCBP	491	95	N/A	N/A	496	0.20, 0.45	23.1	N/A	N/A	132
**5PCzCN**	10 wt% mCP	489 [b]	96.5	2.68	0.03	504	0.21, 0.49	32.1	N/A	9.3	133
**5CzBN-ESF**	Neat film	480	80	1.82	0.06	508	0.27, 0.55	30.6	2	33	134
**5CzBN-BSF**	Neat film	486 [b]	78	1.97	0.12	508	0.26, 0.54	26.5	≈1	39	134
**5CzBN-HSF**	Neat film	488 [b]	70	2.3	0.11	508	0.25, 0.54	20.9	≈1	23	134
**Boron containing acceptors**											
**ACBM**	8 wt% 2,6-DCzppy	527	76	3.0	0.11	≈531	0.33, 0.56	11.2	9	≈63	135
**SACBM**	4 wt% 2,6-DCzppy	518	99	2.6	0.11	≈514	0.22, 0.59	19.1	2	25	135
**B-2DMAC**	4 wt% 2,6-DCzppy	505	47 [b]	3.5	0.03	507	0.25, 0.53	19.3	6	21	136
**B-2PTZ**	4 wt% 2,6-DCzppy	549	18 [b]	2.0	0.06	556	0.43, 0.54	7.6	12	43	136
**B-2PXZ**	4 wt% 2,6-DCzppy	542	26 [b]	1.9	0.04	544	0.40, 0.56	10.1	2	12	136
**PXZBM**	4 wt% 2,6-DCzppy	N/A	36 [b]	3.6 [b]	0.13 [b]	567	0.45, 0.51	10.9	10	63	137

**PXZPBM**	4 wt% 2,6-DCzppy	538 [b]	80 [b]	2.2 [b]	0.08 [b]	505	0.25, 0.54	22.6	1	6	137
**PX-SF-B**	5 wt% CBP	533	84	3.83	0.013 [b]	535	0.37, 0.55	24.8	0	5.6	138
**CzCF3oB**	20 wt% TCTA:B3PYMPM	498	86	23.5	0.081	517	0.24, 0.57	22.9	2	23	139
**BuCzCF3oB**	20 wt% TCTA:B3PYMPM	518	87	15.3	0.07	521	0.330, 0.598	29.9	0	16	139
**BuCzTF7oB**	20 wt% TCTA:B3PYMPM	534	76	19.3	0.075	550	0.33, 0.60	21.9	0.4	16	139
**MPAc-BS**	50 wt% PPF	497	100	1.3	0.023	503	0.20, 0.51	25.3	1.2	6.3	140
**CzDBA**	10 wt% CBP	524	100	3.2	0.03	528	0.31, 0.61	37.8	≈0	0.3	141
***t*BuCzDBA**	10 wt% CBP	553	86	2.1	0.02	542	0.37, 0.60	32.4	≈8	3	141
**oB-2Cz**	10 wt% DPEPO	482	92.9	2.4	0.06	486	0.18, 0.37	28.1	N/A	51	142
**oB-2tCz**	10 wt% DPEPO	495	96.3	1.2	0.03	498	0.22, 0.49	27.5	N/A	44	142
**TMCzBCO**	5 wt% mCBP	526	94	0.99	0.007	532	0.33, 0.59	24.7	N/A	7.3	143
**DMACBCO**	5 wt% mCBP	520	93	1.05	0.011	556	0.43, 0.54	28.4	N/A	5.6	143
**CzDBA**	Neat film	540	90.6	1.0	0.03 [J]	557	0.44, 0.55	13.5	N/A	7.4	144
***t*BuCzDBA**	Neat film	561	84	1.2	0.02 [J]	558	0.44, 0.55	26.9	N/A	1.1	144
**iCzDBA**	Neat film	538	88	1.4	0.03 [J]	540	0.38, 0.59	18.7	N/A	0.5	144
**fppyBTPA**	8 wt% mCPCN	494	72	2.0	0.00	N/A	N/A	20.2	23	≈59	145
**dfppyBTPA**	25 wt% mCPCN	508	100	2.4	0.00	N/A	N/A	26.6	5	≈33	145
**Sulfone containing acceptors**											
**DDA-DP**	15 wt% mCP	549 [e]	45	0.45	0.04	550	0.36, 0.56	8.1	≈0	1	146
**CzTTR**	6.5 wt% mCP	487	56	28.1	0.10	492	0.21, 0.35	14.4	≈43	≈83	147
**DCZ-TTR**	6.5 wt% mCP	502	47	25.8	0.03	512	0.25, 0.50	20.2	≈16	≈40	147
**DMAC-TTR**	10 wt% mCP	555	43	5.2	0.01	≈545	0.33, 0.50	13.9	≈12	≈39	148
**DMAC-PTR**	10 wt% mCP	572	59	3.4	0.01	≈553	0.40, 0.56	18.2	≈14	≈44	148
**SADF-TTR**	10 wt% mCP	530	51	5.2	0.04	≈545	0.35, 0.57	20.2	≈17	≈31	148
**2,3-TXO-PhCz**	5 wt% CBP	540	62	131	0.24	≈548	0.42, 0.45	11.9	≈45	≈74	149
**3,6-TXO-PhCz**	5 wt% CBP	544	85	74	0.22	≈558	0.43, 0.53	18.1	≈41	≈76	149
**2,6-TXO-PhCz**	5 wt% CBP	526	84	77	0.20	≈548	0.40, 0.55	23.2	≈59	≈83	149
**2,7-TXO-PhCz**	5 wt% CBP	530	89	63	0.01	≈548	0.39, 0.55	24.4	≈51	≈76	149
**TAT-3DBTO$_2$**	10 wt% BCPO	≈512	100	11.7	0.03	≈508	0.26, 0.46	30.9	29	47	150
**DMAC-Cz-TTR**	10 wt% CBP	550	69.5	14.7	0.07	568	0.40, 0.54	21.2	13.7	31.6	151
**PS-BZ-DMAC**	5 wt% CBP	574	76.0	2.83	0.005	537	0.37, 0.55	20.6	31.2	N/A	152

**CzPXZ**	Neat film	533	79	3.6	0.03	520	N/A	21.8	N/A	N/A	153
**t-CzPXZ**	Neat film	528	77	1.4	0.04	514	N/A	17.4	N/A	N/A	153
**Triazine acceptor**											
**TRZ-*p*-ACRSA**	20 wt% DPEPO	≈500	97	4.7	N/A	≈494	0.19, 0.42	28.0	1	21	154
**TRZ-*m*-ACRSA**	20 wt% DPEPO	≈500	70	5.7	N/A	≈494	0.22, 0.45	17.7	3	24	154
***p*tBCzTPTZ**	80 wt% DPEPO	446	25	N/A	0.24	492	0.21, 0.35	4.4	23	66	155
***p*tBCzPO$_2$TPTZ**	80 wt% DPEPO	494	96	N/A	0.01	488	0.18, 0.42	28.9	10	43	155
**PAPTC**	20 wt% SimCP2	509	78	0.6	0.07	520	N/A	17.4	N/A	17	156
**BPAPTC**	20 wt% SimCP2	519	90	0.6	0.06	520	N/A	24.3	N/A	7	156
**BP-phIDID**	8 wt% CBP	520 [b]	57	22.8 [i]	0.20 [b]	497	0.25, 0.43	13.9	56	≈81	157
**Tria-phIDID**	8 wt% CBP	526 [b]	70	18.4 [i]	0.12 [b]	504	0.28, 0.49	20.8	49	≈78	157
**TRZ-TPDICz**	20 wt% DBFPO	N/A	96	8.57	0.14	509	0.25, 0.53	30.3	N/A	39	158
**HPy**	10 wt% DPEPO	500	94 (THF)	5.08	0.22	N/A	0.22, 0.44	23.6	N/A	63	159
**CH3Py**	10 wt% DPEPO	500	88.7 (THF)	3.42	0.1	N/A	0.24, 0.44	24.6	N/A	28	159
**DTRZ-DI**	25 wt% TCTA:Bepp$_2$	521	83	1.47	0.03	526	0.32, 0.58	26.2	N/A	≈7	160
**TRZ-DI**	25 wt% TCTA:Bepp$_2$	521	87	1.32	0.02	526	0.31, 0.57	31.4	N/A	≈4	160
**dBFCzCNTrz**	20 wt% DPEPO	490	80.7	4.9	0.09	497	0.22, 0.47	27.5	N/A	12	12
**dBFCzTrz**	20 wt% DPEPO	455	89.4	30.4	0.13	476	0.15, 0.18	22.6	N/A	46	12
**p2Cz2Trz**	10 wt% DPEPO	502 [i]	86	16.6	0.18 [i]	534	N/A	12.5	≈2	26	38
**m2Cz2Trz**	10 wt% DPEPO	465 [i]	96	12.2	0.09 [i]	493	N/A	18.5	≈3	13	38
**BCzTRzDBF**	5 wt% mCBPTrz	≈485	82	5.4	0.06	503	0.24, 0.52	20.1	≈0	≈15	161
**TCzTrzDBF**	5 wt% mCBPTrz	≈499	86	4.4	0.01	511	0.27, 0.57	23.5	≈0	≈13	161
**IDCzTrzDBF**	5 wt% mCBPTrz	≈475	85	2.8	0.05	500	0.22, 0.48	12.2	≈0	≈4	161
**D2T-TRZ**	10 wt% DPEPO	489	97	2.17	0.10	N/A	0.20, 0.45	27.1	11.1	29.5	162
**D2Y-TRZ**	10 wt% DPEPO	491	71	64	0.41	N/A	0.23, 0.48	16.4	42.7	76.8	162
**BmTrzCz**	Neat film	N/A	85	5.5	0.07	481	0.15, 0.30	20.3	N/A	11.3	163
**BmTrzCNCz**	Neat film	N/A	84	3.9	0.05	494	0.20, 0.46	21.9	N/A	11.9	163
**BTrzICz**	20 wt% CzTrz	490	97	3.7	0.00	N/A	0.29, 0.56	20.7	19	N/A	164
**BTrzBCz**	20 wt% CzTrz	500	92	7.2	0.04	N/A	0.30, 0.57	20.5	N/A	N/A	164
**PXZ-Ph-PyTRZ**	10 wt% CBP	538	76	N/A	0.09	540	0.38, 0.56	22.2	N/A	18.9	165
**BTrztCz**	10 wt%	N/	70.0	33.4	0.16	496	N/A	21.4	N/A	N/A	166

	DPEPO	A									
**TDMAC-PM**	10 wt% DPEPO	505	77	5.0	0.048	505	0.32, 0.53	24.2	50.8	78.5	167
**TDMAC-TRZ**	10 wt% DPEPO	525	82	1.7	0.045	525	0.32, 0.54	24.2	18.6	45.5	167
**DTPTCzDP**	7 wt% DPEPO	508	68	2.78	0.14 [f]	510	0.30, 0.50	20.13	7.5	28.5	168
**DTPTCzDP-CN**	7 wt% CBP	545	62	1.47	0.03 [f]	548	0.40, 0.54	17.81	4.1	20.7	168
**DTPTCzDP-Py**	7 wt% DPEPO	532	70	1.76	0.08 [f]	538	0.37, 0.54	16.85	11.9	35.1	168
**PFDMAC-TRZ**	30 wt% DPEPO	501 [b]	93	1.6	0.16	521	0.32, 0.55	35.1	N/A	29.1	169
**DPFDMAC-TRZ**	30 wt% DPEPO	508 [b]	97	1.53	0.16	524	0.32, 0.55	37.0	N/A	9.5	169
**Trz-Py-NCS**	Neat film	505	100	1.2	0.059	520	N/A	30.8	N/A	5.5	170
**Trz-Py-SAC**	Neat film	510	100	1.3	0.058	524	N/A	30.3	N/A	7.3	170
**Pyrimidine-based acceptor**											
**PXZPM**	mCPCN 6wt%	521	100	2.56	0.04	528	0.33, 0.58	29.5	N/A	31	171
**PXZPyPM**	mCPCN 6wt%	524	100	2.77	0.07	528	0.33, 0.58	33.9	N/A	31	171
**PXZTAZPM**	mCPCN 6wt%	528	93	2.79	0.05	528	0.33, 0.58	30.1	N/A	38	171
**2PXZ-PRZ**	10 wt% CBP	519	65	54	0.21	≈517	0.31, 0.55	21.4	19	59	172
**ClPPM**	1.5 wt% CBP	547	93	1.4	0.06	546	0.4, 0.55	25.3	N/A	12	173
**BrPPM**	1.5 wt% CBP	546	91	1.3	0.07	544	0.39, 0.56	23.6	N/A	16	173
**PXZ-PYR**	1 wt% PMMA	543 [b]	42 [b]	1.6 [b]	0.01	536	0.35, 0.56	27.9	35	56	174
**PXZ-muPYR**	1 wt% PMMA	530 [b]	52 [b]	4.2	0.07	529	0.32, 0.55	29.1	14	30	174
**PXZ-mdPYR**	1 wt% PMMA	528 [b]	38 [b]	1.7	0.15	514	0.27, 0.49	27.5	24	42	174
**PXZ-2dPYR**	1 wt% PMMA	519 [b]	53 [b]	4.3	0.13	502	0.23, 0.42	26.3	36	66	174
**12BTAc-PM**	30 wt% DPEPO	509	87	3.8	0.17	503	0.23, 0.50	25.6	N/A	14	175
**34BFAc-PM**	30 wt% DPEPO	519	95	3.5	0.08	503	0.23, 0.51	27.7	N/A	11	175
**34BTAc-PM**	30 wt% DPEPO	521	92	3.4	0.08	509	0.25, 0.55	25.8	N/A	2	175
**22bpmAc**	20 wt% DPEPO	471 [k]	75	37.5	0.28 [k]	517	0.30, 0.54	15.7	≈47	≈83	176
**25bpmAc**	20 wt% DPEPO	472 [k]	98	17.1	0.24 [k]	524	0.31, 0.58	20.5	≈24	≈63	176
**55bpmAc**	10 wt% DPEPO	466 [k]	99	21.8	0.29 [k]	512	0.25, 0.53	24.9	≈49	≈80	176
**DMAC-PymCN**	8 wt% mCPCN	522	84	14.6	0.20	N/A	0.28, 0.51	19.8	35.4	71.2	177
**DMAC-PyoCN**	8 wt% mCPCN	546	91	6.8	0.13	N/A	0.41, 0.55	25.9	19.3	51.7	177
**DMAC-PmmCN**	8 wt% mCPCN	542	83	9.6	0.14	N/A	0.36, 0.55	22.3	15.2	55.6	177
**DMAC-PmoCN**	8 wt% mCPCN	588	66	5.1	0.11	N/A	0.43, 0.54	18.8	15.4	53.2	177
**SpiroAC-PyoCN**	8 wt% mCPCN	548	100	10.6	0.16	N/A	0.31, 0.58	33.7	18.1	53.4	177

**SBAC-PyoCN**	6 wt% mCPCN	553	100	16.6	0.22	N/A	0.31, 0.58	36.1	21.3	63.7	177
**Other N-heterocycles acceptors**											
**NyDPO**	5 wt% CBP	530	79	11.9	0.09	540	0.36, 0.58	29.9	23	53	178
**NyDPt**	5 wt% CBP	533	45	10	0.016	544	0.38, 0.56	25.8	41	76	178
**NyDPAc**	10 wt% DPEPO	510	57	451	0.29	516	0.28, 0.53	20.9	N/A	N/A	179
**26AcINN**	10 wt% DPEPO	495	79	117	0.28	501	0.22, 0.45	21.6	36	66	180
**26PXZINN**	10 wt% CBP	522	79	26.9	0.06	527	0.34, 0.58	22.7	3	26	180
**Ac-2TP**	10 wt% DPEPO	457	53	319	0.38	≈449	0.16, 0.18	9.2	63	88	181
**AcDPA-2TP**	10 wt% CBP	526	62	15	0.03	≈525	0.35, 0.57	23.7	10	8	181
**PPZTPI**	5 wt% CBP	527	73	127	0.11	528	0.36, 0.58	20.5	≈33	≈74	182
**PPZPPI**	5 wt% CBP	533	99	118	0.12	528	0.36, 0.57	21.1	≈21	≈70	182
**FDQCNAc**	1 wt% polystyrene	549	87	24.0	0.087 [b]	554	0.42, 0.55	27.6	N/A	N/A	183
**FBPCNAc**	1 wt% polystyrene	607	79	11.0	0.048 [b]	597	0.55, 0.44	23.8	N/A	N/A	183
**5TzPMPXZ**	0.7 wt% CBP	527	64	2.9	0.10	542	0.37, 0.54	9.3	13	22	184
**7TzPMPXZ**	0.7 wt% CBP	532	49	2.8	0.07	552	0.40, 0.53	9.3	33	2	184
**5,7TzPmPXZ**	0.7 wt% CBP	543	66	2.6	0.06	560	0.43, 0.53	14.3	6	13	184
**Ph-PO**	20 wt% DPEPO	525	78	5.36	0.14	524	0.31, 0.55	20.8	10	N/A	185
**4BPy-*m*DTC**	7 wt% mCBP	495 [b]	97	18.0	0.01 [b]	490	0.17, 0.37	28.1	≈28	≈58	114
**2BPy-*m*DTC**	7 wt% mCBP	494 [b]	96	6.9	0.02 [b]	490	0.16, 0.37	28.0	≈21	≈53	114
**TCZPBOX**	40 wt% PYD2	527	89	4.0	0.03	≈523	0.40, 0.56	27.9	5	13	186
**DQBC**	20 wt% mCPBC	551	95	5.5	0.06	534	0.38, 0.55	39.1	7.7	25.6	187
***p*CF35tCzOXD**	10 wt% o-CzOXD	535	66.7	2.16	0.120	494	0.19, 0.41	20.3	N/A	N/A	188
***m*CF35tCzOXD**	10 wt% o-CzOXD	532	66.2	1.90	0.005	494	0.19, 0.42	22.1	N/A	N/A	188
**dCF35tCzOXD**	10 wt% o-CzOXD	N/A	87.8	2.04	0.005	496	0.20, 0.45	23.3	N/A	N/A	188
**5*t*CzDPO**	12.5 wt% DPEPO	474	79	71	0.01 [b]	N/A	0.18, 0.36	29	N/A	55.2	92
**CN-BP-TPA**	10 wt% CBP	578	98	760	0.19	580	0.51, 0.47	26.0	N/A	86.9	189
**TPZ**	10 wt% CBP	539	54	284	0.29 [b]	534	0.32, 0.61	8.3	33.7	59.0	190
**DPZ**	10 wt% CBP	564	67	240	0.34 [b]	550	0.40, 0.57	11.6	53.4	66.4	190
**APZ**	10 wt% CBP	577	86	298	0.20 [b]	562	0.44, 0.55	27.5	81.8	85.8	190
**4HQ-PXZ**	6 wt% CBP	504	67	48.3	0.19	511	0.25, 0.54	20.2	22	54	191
**4PQ-PXZ**	6 wt% CBP	510	68	40.1	0.22	518	0.28, 0.57	20.5	37	71	191

**2HQ-PXZ**	6 wt% CBP	523	81	35.9	0.10	538	0.36, 0.57	16.0	14	38	191
**2PQ-PXZ**	6 wt% CBP	523	74	28.3	0.09	538	0.36, 0.56	17.1	27	59	191
**TBQ-DPXZ**	20 wt% BCPO	537	91	3.6	0.07	533	0.35, 0.55	25.1	22.7	53.0	192
**PXZ-PCN**	10 wt% CBP	565	57	1.6	0.01	568	0.48, 0.51	15.1	≈0	21	193
**DPmP-PXZ**	Neat film	524	90	1.11	0.10	556	0.43, 0.55	23.6	N/A	8.5	194
**PyDCN-PXZ**	15 wt% CBP	532 [b]	89.6	6.4	0.06	519	0.30, 0.53	26.9	N/A	25.3	195
**Ph-DMAC**	10 wt% CBP	532	89	2.5	0.06	539	0.37, 0.56	29.1	N/A	33	196
**Na-DMAC**	10 wt% CBP	531	56	0.68	0.15	554	0.42, 0.54	21.2	N/A	61	196
**3Py-DMAC**	10 wt% CBP	538	60	1.5	0.04	567	0.47, 0.52	21.5	N/A	22	196
**Me-DMAC**	10 wt% mCP	542	96	2.7	0.12	517	0.28, 0.51	25.8	N/A	29	197
**TPAPPC**	10 wt% mCPCN	531	100	133.9	0.21	520	0.28, 0.56	37.5	N/A	N/A	198
**TPAmPPC**	10 wt% mCPCN	533	100	3.4	0.009	537	0.35, 0.57	39.8	N/A	N/A	198
**tTPAmPPC**	10 wt% mCPCN	549	97	3.4	0.010	556	0.42, 0.55	29.8	N/A	N/A	198
**3CPDA-MPC**	Neat film	522	89	1.65	0.11	525	0.34, 0.54	26.5	23.0	25.3	199
**9CPDA-MPC**	Neat film	509	92	2.05	0.13	510	0.28, 0.48	29.6	14.5	34.1	199
**BTPDIDCz**	5 wt% CzTrz	N/A	83	4.1	0.01 [f]	530	0.38, 0.57	24.5	N/A	N/A	200
**BTPBFCz-D1**	DPEPO	480	84	16.3	0.10 (THF)	491	0.18, 0.39	20.7	N/A	43	201
**BTPBFCz-D2**	DPEPO	480	85	22	0.23(THF)	491	0.18, 0.37	20.0	N/A	63	201
**BTPBFCz-D3**	DPEPO	480	92	15.6	0.12(THF)	493	0.19, 0.41	22.7	N/A	35	201
**Carbonyl acceptors**											
**AI-Cz**	5 wt% mCBP	510	84	81	0.09	510	0.32, 0.55	23.2	34	70	202
**AI-TBCz**	5 wt% mCBP	545	72	64	0.08	540	0.37, 0.56	21.1	45	74	202
**5PXZ-PIDO**	1.5 wt% CBP	535	72	2.4	0.11	≈526	0.39, 0.42	14.4	≈5	25	203
**5,6PXZ-PIDO**	1.5 wt% CBP	544	76	2.0	0.06	≈534	0.42, 0.53	16.9	≈4	16	203
**BPO-BDMAc**	25 wt% mCPCN	516	89.1	3	0.03	522	0.29, 0.54	22.5	N/A	N/A	204
**SBF-BP-DMAC**	Neat film	518	72.1	4.9	0.06	528	0.32, 0.58	24.5	N/A	16.8	205
**BZC-PXZ**	5 wt% mCP	561 [b]	93	4.47	0.02 [b]	N/A	N/A	22	N/A	7.3	206
**OPDPO**	10 wt% CBP	589 [e]	N/A	N/A	0.02 [e]	552	N/A	26.7	10.1	32.6	207
**TBP-PXZ**	5 wt% CBP	592	68	11.9	0.02	564	0.45, 0.53	17.7	≈0	10	208
**PBCz-BP-DMAC**	10 wt% PPF	488	92.3	9.2	0.02	492	0.21, 0.42	27.5	N/A	8	209
**TCO-DMAC**	20 wt% BCPO	499	92	4.94	0.04	499	0.23, 0.45	21.2	3.8	17.0	210
**SeDF-G**	10 wt% mCBP	N/A	7.6	3.9	0.15	N/A	0.31, 0.53	30.8	N/A	N/A	107

**SeDF-YG**	10 wt% mCBP	N/A	8.5	4.6	0.15	N/A	0.33, 0.48	18.8	N/A	N/A	107
**PHzMCO**	8 wt% mCP	510	47	17.9	0.018	508	0.26, 0.50	17.8	≈1	14	211
**PHzBCO**	8 wt% mCP	524	52	9.3	0.006	520	0.32, 0.50	19.6	≈6	13	211
**2,3-POA**	3.5 wt% mCBP	547	82.5	6.2	0.01	528	0.30, 0.62	21.7	N/A	N/A	212
**3,6-DPXZ-AD**	7 wt% CBP	563 [b]	94.9	1.6	0.02	552	0.43, 0.55	30.6	5.6	26.8	213
**PDAD-DMAC**	20 wt% PPF	524	94	4.8	0.029	492	0.19, 0.46	24.1	N/A	N/A	214
**3-DMAC-6-CF_3-AD**	7 wt% DPEPO	514 [b]	85	3.5	0.07 [f]	512	0.27, 0.55	21.6	17.1	N/A	215
**3-PXZ-AD**	7 wt% CBP	555 [b]	86	2.2	-0.06 [f]	519	0.32, 0.57	21.1	4.7	N/A	215
**3-PXZ-6-Me-AD**	7 wt% CBP	533 [b]	91	2.3	0 [f]	505	0.27, 0.53	23.3	5.2	N/A	215
**3,6-DMAC-AD**	7 wt% DPEPO	504 [b]	81.1	3.4	0 [f]	495	0.20, 0.42	23.5	7.2	19.6	216
**3,6-DMAC-AD-CF_3**	7 wt% DPEPO	508 [b]	74.4	2.2	0 [f]	505	0.24, 0.55	21.6	0.4	5.1	216
**MCz-TXT**	10 wt% mCBP	490	92	0.75	0.30	497	0.21, 0.46	25.8	0	4.7	217
**SAT-DAC**	30 wt% DPEPO	510 [e]	76.8	N/A	0 [e]	520	N/A	22.6	N/A	19.9	218
**SATX-DAC**	30 wt% DPEPO	517 [e]	68.1	N/A	0.05 [e]	524	N/A	20.9	N/A	18.6	218
**PXZANQ**	10 wt% DPEPO	522	71	10.2	0.03	528	0.33, 0.54	22.1	24.9	37.1	219
**DPAC-BPI-CN**	Neat film	525	90.1	3	0.07 [b]	531	0.34, 0.56	26.2	N/A	43.1	220
**DPAC-BPI**	Neat film	472	11.2	27	0.15 [b]	494	0.19, 0.39	10.5	N/A	94.3	220
**Other acceptors**											
**7CzFDCF_3DPh**	Neat film	555	55	2.02	0.05	N/A	0.36, 0.56	20.8	11	19	221
**DBCP**	5 wt% mCP	557 [b]	89	30	0.1	544	0.38, 0.57	20.2	N/A	N/A	222
**FAP**	5 wt% mCP	580 [b]	54	489.4	0.32	568	0.48, 0.51	12.8	N/A	N/A	222

[a]10% in mCP:TSPO1; [b] Toluene; [c] 50% doped in PPF; [d] 25% PPF; [e]Neat films; [f] MeTHF; [g] DCM; [h] BLYP/TD-DFT; [I] PMMA. [J] 10% in mCP [K] 1% in polystyrene film.

Table **S3**. Summary of photophysical and device performance of red TADF emitters reviewed in Section **5**.

**Compound**	**Medium**	**λ_{PL} / nm**	**Φ_{PL} / %**	**τ_d / µs**	**ΔE_{ST} / eV**	**λ_{EL} / nm**	**CIE_{XY}**	**EQE$_{max}$ / %**	**Roll-off 100 cd m^{-2} / %**	**Roll-off 1000 cd m^{-2} / %**	**Ref.**
**Pyridine-3,5-dicarbonitrile Acceptors**											

**bis-PXZ-PCN**	10 wt% CBP	565	36	1.40	0.04	600	0.55, 0.44	9.8	N/A	15	193
**tri-PXZ-PCN**	10 wt% CBP	601	34	1.48	0.05	608	0.56, 0.44	9.7	N/A	18	193
**PXZ-PCN**	10 wt% CBP	606	57	1.58	0,01	568	0.48, 0.51	15.1	N/A	N/A	193
**NP**	10 wt% CBP	560	50	0.65	0.14	591	0.53, 0.46	17.1	N/A	41	223
**TP**	10 wt% CBP	555	40	0.80	0.15	591	0.52, 0.47	12.4	N/A	32	223
**Quinoxaline Acceptors**											
**TPA-QCN**	15 wt% TPBi	649	~70	285	0.23	644	0.62, 0.38	14.5	N/A	N/A	224
**6,7-DCQx-Ac**	1 wt% polystyrene	~550	91	8.99	0.13	578	0.49, 0.50	21.1	~5	~19	225
**5,8-DCQx-Ac**	1 wt% polystyrene	~600	72	3.12	0.11	602	0.55, 0.44	16.4	~9	~21	225
**5,8-DCQx-DICz**	1 wt% polystyrene	651	40	1.6	0.028	578	0.55, 0.44	12.5	N/A	N/A	226
**6,7-DCQx-DICz**	1 wt% polystyrene	603	73	2.5	0.064	603	0.49, 0.50	23.9	N/A	N/A	226
**TFM-QP**	5 wt% CBP	N/A	61	5	0.04	584	0.50, 0.48	10.7	N/A	N/A	227
**CN-QP**	5 wt% CBP	N/A	62	1.6	0.03	584	0.50, 0.48	9.7	N/A	N/A	227
**PXZ-PQM**	5 wt% DCzDPy	588	70	5.37	0.03	592	0.53, 0.46	20.4	14	45	228
**DPXZ-PQM**	5 wt% DCzDPy	586	88	3.77	0.02	590	0.51, 0.48	26	23	47	228
**Acenaphtho[1,2-b]pyrazine Acceptors**											
**APDC-DTPA**	10 wt% TPBi	687	63	N/A	0.14	693	N/A	10.2	92	N/A	229
**TPAAP**	1wt%TPBi	648	98	5530	0.149	700	N/A	14.1	70	84	230
**TPAAQ**	1 wt% TPBi	585	95	35700	0.296	630	N/A	15.8	81	89	230
**CAT-1**	10 wt% CBP	763	8.83	80	0.4	719	N/A	N/A	N/A	N/A	231
**ANQDC-DMAC**	1.5 wt% CBP:TPBi	596	95	4.2	0.06	615	0.58, 0.41	27.5	30	58	232
**ANQDC-MeFAC**	1.5 wt% CBP:TPBic	604	77	2.8	0.05	614	0.60, 0.40	26.3	16	36	232
**AQTC-DTPA**	10 wt% mCBP	718	19.10	7.95	0.18	694	N/A	9.28	N/A	N/A	233
**ANQDC-MSTA**	1.5 wt% CBP:TPBi	623	65	3.4	0.07	622	0.61, 0.38	21.8	16.8	43	234
**ANQDC-PSTA**	1.5 wt% CBP:TPBi	618	72	3.7	0.08	622	0.61, 0.38	24.7	19.7	42	234
**Pyrazino- or Quinoxalino- Expanded Phenanthrene Acceptors**											
**DMAC-DCPP**	3 wt% mCPPy2PO	618	33	2.4	0.08	0	0.60, 0.40 (100)	10.1	N/A	N/A	235
**DPA-Ph-DCPP**	3 wt% mCPPy2PO	628	55	82.1	0.1	0	0.64, 0.36 (100)	15.1	N/A	N/A	235
**DMAC-Ph-DCPP**	3 wt% mCPPy2PO	594	65	3.2	0.05	0	0.53, 0.46 (100)	16.9	N/A	N/A	235
**PXZ-DCPP**	5 wt% CBP	564	11.9	3.3	0.09	608	0.56, 0.43	17.4	N/A	26	236

**PTZ-DCPP**	5 wt% CBP	580	17.4	3.7	0.18	640	0.62, 0.36	12.3	N/A	50	236
**DDPhCz-DCPP**	10 wt% CBP	593	66	15.6	0.08	584	0.51, 0.48	20.8	N/A	N/A	237
**DDTPACz-DCPP**	10 wt% CBP	663	53	9.7	0.16	646	0.61, 0.38	13.6	N/A	N/A	237
**Da-CNBQx**	6 wt% CBP	633	85	60 us	0.18	617	0.59, 0.41	20	63	N/A	238
**Ac-CNBQx**	6 wt% CBP	561	75	9.3	0.1	585	0.51, 0.48	14	1	N/A	238
**Da-CNBPz**	6 wt% CBP	688	72	49	0.11	670	0.66, 0.34	15	75	N/A	238
**Ac-CNBPz**	6 wt% CBP	615	67	6.9	0.03	630	0.61, 0.39	16.2	10	N/A	238
**FBPCNAc**	1 wt% polystyrene	607	79	9.02	0.047	597	0.55, 0.44	23.8	~16	~58	183
**TPA–PZCN**	15 wt% CBP	610	98	133	0.13	628	0.65, 0.35	27.4	63	83	239
**1DMAC−BP**	20 wt% mCBP	549	42	12.6	0.22[b]	560	0.43, 0.54	10.1	60	85	240
**2DMAC−BP**	20 wt% mCBP	565	84	7.5	0.21	576	0.49, 0.49	11.8	21	64	240
**3DMAC−BP**	20 wt% mCBP	590	89	2.9	0.05	606	0.58, 0.41	22	20	56	240
**1PXZ-BP**	7 wt% CBP	578	73	4.8	N/A	590	0.52, 0.46	26.3	35	64	241
**2PXZ-BP**	7 wt% CBP	596	63	4.3	N/A	606	0.57, 0.42	19.2	24	53	241
**tDBBPZ-DPXZ**	10 wt % CBP	625	83	9.78	0.03	608	0.58, 0.42	17	22	N/A	242
**DBPZ-DPXZ**	10 wt % CBP	620	84	6.47	0.03	608	0.57, 0.43	17.8	4	N/A	242
**pDBBPZ-DPXZ**	8 wt% CBP	622 [b]	49	53.3	0.23	608	0.59, 0.40	8	61	N/A	243
**pDTBPZ-DPXZ**	8 wt% CBP	621 [b]	66	5.6	0.1	604	0.58, 0.41	16	34	N/A	243
**oDTBPZ-DPXZ**	8 wt% CBP	622 [b]	87	3.3	0.04	604	0.59, 0.41	20.1	30	N/A	243
**DPXZ-DPPM**	5 wt% DCzDPy	630	61	3.53	0.05	630	0.61, 0.38	11.5	9	41	228
**BPPZ-PXZ**	14 wt% CBP	607	100	3.6	0.03	604	0.57, 0.43	25.2	N/A	28	244
**mDPBPZ-PXZ**	14 wt% CBP	638	95	7.4	0.04	624	0.62, 0.38	21.7	N/A	33	244
**DPXZ-BPPZ**	18 wt% CBP	610	97.1 ± 1.1	10.3	0.03	612	0.6, 0.4	20.1	5	17	245
**IDAC-BPPZ**	11 wt% CBP	583	84	14	0.07	580	0.5, 0.49	18.3	42	70	246
**ACID-BPPZ**	11 wt% CBP	596	75	12	0.02	588	0.52, 0.47	14.7	16	39	246
**DMAC-11-DPPZ**	10 wt% CBP	576	57	1.53	0.11	588	0.53, 0.46	23.8	~37	~66	247
**PXZ-11-DPPZ**	10 wt% CBP	617	41	0.72	0.06	627	0.63, 0.37	8.7	~20	~66	247
**TAT−DBPZ**	20 wt% CBP	576	76	2.3	0.16	604	0.56, 0.44	15.4	20	47	248
**TAT−FDBPZ**	20 wt% CBP	593	62	1.51	0.1	611	0.58, 0.41	9.2	1	20	248
**TPA-PZTCN**	1 wt % mCBP	672	45.6	19.2	0.10	651	N/A	19.3	N/A	N/A	249
**Ac-BPCN**	1 wt% polystyrene	618	66	11.1	0.13	597	0.54, 0.45	20.7	~42	~61	250

**DACz-BPCN**	1 wt% polystyrene	654	47	7.2	0.03	631	0.60, 0.39	11	~27	~55	250
**3,6,11-triAC-BPQ**	15 wt% CBP	589	75	2.5	N/A	581	0.51, 0.48	22	45	82	251
**3,6,12-triAC-BPQ**	15 wt% CBP	607	53	2.25	N/A -	616	0.58, 0.39	16.5	33	82	251
***o*TPA-DPPZ**	30 wt% DBFDPO	605	75	17	0.07	600	N/A	18.5	17	48	252
***p*TPA-DPPZ**	neat	644	87	5	0.1	652	0.67, 0.33	12.3	3	15	252
**ACFO**	3 wt% CBP	N/A	31	3.8	0.13	593	0.51, 0.45	10.8	43	76	253
**DBPz-2spAc**	1 wt% mCBP	632	65.6	2.74	-0.04	630	0.62, 0.36	13.3	1	N/A	254
**oPDM**	8 wt% mCBP	582	100	72.7	0.11	596	0.56, 0.44	28.2	N/A	N/A	255
**pPDM**	8 wt% mCBP	573	88	73.7	0.15	582	0.52, 0.47	11.8	N/A	N/A	255
**oDMAC–DPPZ**	1 wt% polystyrene	652 [b]	63	1.76	0.037	614	0.59, 0.40	13.4	N/A	N/A	256
**pDMAC–DPPZ**	1 wt% polystyrene	669 [b]	15	5.03	0.079	638	0.64, 0.35	4	N/A	N/A	256
**3DMAC-BP-Br**	15 wt% mCBP	612 [b]	83	3.8	0.04	596	0.55, 0.45	18.97	N/A	N/A	257
**3DMAC-BP-CN**	5 wt% mCBP	617 [b]	92	4.6	0.02	586	0.51, 0.47	22.4	N/A	N/A	257
**TPA-PZTCN**	1 wt % mCBP	672	45.6	19.2	0.10	651	N/A	19.3	N/A	N/A	249
**pDBBPZ-DPXZ**	8 wt% CBP	622	49	53.3	0.23	608	0.59, 0.40	18	3.1	N/A	243
**pDTBPZ-DPXZ**	8 wt% CBP	621	66	5.6	0.10	604	0.58, 0.41	16	10.5	N/A	243
**oDTBPZ-DPXZ**	8 wt% CBP	622	87	3.3	0.04	604	0.59, 0.41	20.1	14.1	N/A	243
**Phenanthro[4,5-abc]phenazine-11,12-dicarbonitrile or Phenanthro[4,5-fgh]quinoxaline Acceptors**											
**TPA-PPDCN**	10 wt% CBP	663	87	2.36	0.23 (tol)	664	0.68, 0.32	20.2	77	N/A	64
**T-DA-1**	10 wt% CBP	601	78	1.59	0.16	596	0.54, 0.46	22.62	38	N/A	258
**T-DA-2**	10 wt% CBP	640	89	0.88	0.05	640	0.62, 0.37	26.3	9	N/A	258
**C-DA-1**	10 wt% CBP	649	12	1.15	0.02	648	0.66, 0.34	3.52	N/A	N/A	258
**C-DA-2**	10 wt% CBP	689	14	0.94	0.02	684	0.67, 0.31	3.13	N/A	N/A	258
**1,8-naphthalimide acceptors**											
**NAI-DMAC**	1.5 wt% mCPCN	582 [b]	59.9	14.7	0.09	597	0.56, 0.44	23.4	42	80	259
**NAI-DPAC**	6 wt% mCPCN	570 [b]	78.9	48.7	0.07	584	0.52, 0.47	29.2	55	92	259
**BTDMAc-NAI**	1.5 wt% mCPCN	~590	39	69.3	0.07	641	0.62, 0.38	9.2	~32	N/A	260
**BFDMAc-NAI**	1.5 wt% mCPCN	~570	73	55.8	0.16	590	0.54, 0.45	20.3	~48	~80	260
**NAI_R1**	1.5 wt% mCP	~585	63	3.95	0.09	616	0.58, 0.41	9	N/A	52	261
**NAI_R2**	1.5 wt% mCP	~585	65	3.98	0.092	610	0.57, 0.42	11.5	N/A	66	261
**NAI_R3**	1.5 wt%	~59	66	3.14	0.058	622	0.60, 0.40	22.5	N/A	85	261

	mCP	0									
**PXZ-NAI**	10 wt% o-mCPBI	605	43.6	1.2	0.06	624	0.61, 0.39	13	N/A	28	262
**PTZ-NAI**	10 wt% o-mCPBI	617	30.5	1.6	0.08	632	0.60, 0.37	11.4	N/A	47	262
**PhNAI-MFAC**	1.5 wt% mCPCN	603	61	2.9	0.06	610	0.59, 0.41	22.5	33	66	263
**PhNAI-PMSBA**	1.5 wt% mCPCN	606	55	2.7	0.05	615	0.60, 0.40	22.3	36	74	263
**BFDMAc-PhNAI**	1.5 wt% mCPCN	~575	77	52.5	0.16	590	0.54, 0.46	19.8	46	N/A	260
**BTDPAc-PhNAI**	3 wt% mCPCN	~590	63	54.7	0.08	601	0.57, 0.43	18.7	44	N/A	264
**BTDMAc-PhNAI**	1.5 wt% mCPCN	~590	42	40.4	0.07	642	0.61, 0.39	10.1	37	N/A	260
**Other Miscellaneous Examples**											
**2TPA-BF2**	6 wt% CBP	721	70	28	0.06[a]	721	N/A	9.7	NA	N/A	265
**4TPA-2BF2**	6 wt% CBP	760	45.2	N/A	0.32[a]	758	N/A	5.1	NA	N/A	266
**DPhCzB**	5 wt% mCP	538[b]	87	19.5	0.15[b]	587	N/A	6.7	~3.7	~9.0	267
**DTPAB**	5 wt% mCP	560[b]	97	55.7	0.17[b]	605	N/A	8.2	~3.7	~9.0	267
**DMAC2oDBA**	20 wt% CBP	602	44	6.6	0.054	615	0.59, 0.40	10.1	~14	~51	268
**dPhADBA**	12 wt% CBP	614	53	1.16	0.04	613	0.60, 0.39	11.1	1.7	24.8	269
**dmAcDBA**	12 wt% CBP	587	71	3.97	0.06	583	0.51, 0.48	24.9	4.5	26.4	269
**SpAcDBA**	12 wt% CBP	570	85	3.35	0.08	567	0.46, 0.52	30.0	2.0	15.9	269
**PzTDBA**	5 wt% TCTA/Bepp2 (1:1)	599	99.8	2.63	0.06	576	0.53, 0.46	30.3	N/A	3.6	270
**PzDBA**	5 wt% TCTA/Bepp2 (1:1)	610	85.4	2.0	0.05	595	0.60, 0.40	21.8	N/A	3.2	270
**TPACNBz**	30 wt% CBP	710	52	1.52	0.0[6c]	712	0.68, 0.29	6.57	N/A	58	271
**PT-TPA**	15 wt% doped CBP	597[b]	99.7	58.0	0.26[b]	632	0.64, 0.35	29.7	N/A	N/A	272
**PT-Az**	10 wt% CBP	586[b]	68.9	27.7	0.28[b]	612	0.61, 0.38	14.1	N/A	N/A	272
**TPA-DBT12**	5 wt% 35DCzPPY	597	44.7	21.7	0.06[b]	608	0.56, 0.43	14.5	N/A	N/A	273
**TPA-DBT3**	5 wt% 35DCzPPY	616	55.5	241.1	0.17[b]	612	0.56, 0.44	15.0	N/A	N/A	273
**DTPA-DBT**	5 wt% 35DCzPPY	632	42.1	70.3	0.17[b]	628	0.58,0.41	11.8	N/A	N/A	273
**2SO-AD**	10 wt% 35DCzPPY	581	25.0	553.0	0.27[d]	599	N/A	3.2	N/A	N/A	274
**2SO-TBU**	10 wt% 35DCzPPY	615	58.3	272.1	0.14[d]	608	N/A	16.3	N/A	N/A	274
**2SO-F-TBU**	10wt% 35DCzPPY	591	53.0	577.5	0.20[d]	612	N/A	14.5	N/A	N/A	274
**a1**	1 wt% CBP	593	50	4600	0.29	NA	NA	NA	NA	NA	275

**a2**	1 wt% CBP	603	42	1560	0.27	NA	NA	NA	NA	NA	275
**a3**	1 wt% CBP	575	52	62	0.17	NA	NA	NA	NA	NA	275
**a4**	1 wt% CBP	600	8	1.6	0.08	NA	NA	NA	NA	NA	275
**b1**	1 wt% CBP	594	80	416	0.24	624[e]	0.61, 0.39	12.5	35	82	275
**b2**	1 wt% CBP	601	76	185	0.22	637[e]	0.63, 0.37	9.0	37	81	275
**b3**	1 wt% CBP	550	65	16/156	NA	574[e]	NA	9.0	NA	NA	275
**b4**	1 wt% CBP	564	50	6.5	0.07	584[e]	NA	6.9	NA	94	275
**AQ-PhDMAC**	5 wt% CBP	N/A	89	63.6	0.22	580	0.49, 0.49	18.1	N/A	N/A	276
**26DPXZBPy**	10 wt% mCBP	568	76	1	0.04	590	0.49, 0.49	13.7	~5	~20	277

[a] DFT calculations, [b] Toluene solution, [c] 10 wt% doped films in PMMA, [d] 2-MeTHF.

Table **S4**. Summary of photophysical and device performance of CP-TADF emitters reviewed in Section **7**.

**Compound**	**Medium**	**λ_{PL} / nm**	**Φ_{PL} / %**	**τ_D / µs**	**ΔE_{ST} / eV**	**g_{PL}**	**λ_{EL} / nm**	**CIE$_{XY}$**	**EQE$_{max}$ / %**	**roll-off 100 cd m^{-2} / %**	**roll-off 1000 cd m^{-2} / %**	**g_{EL}**	**Ref**
**CP-TADF emitters containing stereogenic centers**													
**DPHN**	Toluene	522	4	N/A	0.26	1.1 × 10^{-3}	N/A	N/A	N/A	N/A	N/A	N/A	278
**(*R*)-TTR-PMAc**	Toluene	577	N/A	0.43	0.36[b]	N/A	N/A	N/A	N/A	N/A	N/A	N/A	279
**(*S*)-TTR-PMAc**	Toluene	577	N/A	0.47	0.39[b]	N/A	N/A	N/A	N/A	N/A	N/A	N/A	279
**(*R*)-TRZ-MeIAc**	12 wt% mCPCN	473	89	82.3	0.19	5.9 × 10^{-4}	494	0.18, 0.38	20.3	N/A	N/A	6.4 × 10^{-4}	280
**(*S*)-NID-MeIc**	6 wt% mCPCN	565	86	235.3	0.22	2.0 × 10^{-3}	589	0.53, 0.47	23.7	N/A	N/A	-2.4 × 10^{-3}	280
**(*R*)-BN-MeIAc**	1 wt% DMIC-TRZ	497[c]	96	28.1	0.11[c]	2.5× 10^{-4}	504	0.12, 0.63	37.2	3	30	2.7× 10^{-4}	281
**(*S*)-SFOT**	30 wt% mCBP	512[c]	89	6.78	0.053[c]	2.2 × 10^{-3}	508	N/A	23.1	N/A	8	1.0 × 10^{-3}	282
**(*S*)-SFST**	30 wt% mCBP	512[c]	53	7.98	0.052[c]	3.4 × 10^{-3}	508	N/A	12.5	N/A	4	1.30 × 10^{-3}	282
**(*S*)-OSFSO**	25 wt% DPEPO	470[c]	81.20	4.7	0.022	1.4 × 10^{-3}	472	0.16, 0.24	20.0	N/A	4	3.1×10^{-3}	283
**(*R*)-FAC-SIC**	Toluene	507	99	5.8	0.075	-2.0× 10^{-4}	517	0.30, 0.52	28.5	16	51	N/A	284
**(*S*)-NPE-AcDPS**	12 wt% DPEPO	451	86	3.4	0.05	~10^{-4}	458	N/A	18.5	13	N/A	N/A	285
**(*R*/*S*)-SCN**	Toluene	522	89	2.1	0.01	1.4/−1.3 × 10^{-3}	519	0.34, 0.53	23.0	N/A	36	−1.4/1.8× 10^{-3}	286

**(*R*/*S*)-SPHCN**	Toluene	505	67	14.6	0.16	$3.5/-3.6 \times 10^{-3}$	512	0.30, 0.52	15.4	N/A	36	$-3.6/3.6 \times 10^{-3}$	286
**(*R*/*S*)-SCFPY**	Toluene	526	89	1.6	0.04	$3.5/-3.5 \times 10^{-3}$	517	0.31, 0.51	23.3	N/A	30	$-3.7/3.6 \times 10^{-3}$	286
**CP-TADF emitters with axial chirality**													
**(*R*)-1**	15% in TCTA	568	18.5	1.03	0.059	-7.2×10^{-4}	580	0.49, 0.48	0.85	N/A	N/A	-0.9×10^{-3}	287
***p*-BAMCN**	8wt% in TCTA/8 wt% in 26DCzPPy	537	86	170	0.18	0.5×10^{-3}	530	0.33, 0.62	27.6	N/A	N/A	N/A	288
***o*-BAMCN**	8wt% in TCTA/8 wt% in 26DCzPPy	503	77	75	0.15	4.3×10^{-3}	506	0.22, 0.57	20.5	N/A	N/A	4.6×10^{-3}	288
**(*R*)-DOBN**	5 wt% in 26DCzPPy	453 [c]	91	51.8	0.14	1.0×10^{-3}	459	0.14, 0.10	23.9	48	68	-0.9×10^{-3}	289
**(*R*)-DOBNT**	5 wt% in 26DCzPPy	459 [c]	96	122.2	0.12	0.9×10^{-3}	464	0.13, 0.12	25.6	46	68	-1.0×10^{-3}	289
**(-)-(*S*)-Cz-*Ax*-CN**	20 wt% DPEPO	460	68.2	12.6	0.029	4.8×10^{-3}	468	N/A	12.5	11	30	1.2×10^{-2}	290
**(+)-4tBuCzPN**	25 wt% DPEPO	476	74	4.0	0.05	5.4×10^{-3} [c]	500	N/A	20.8	N/A	N/A	N/A	129
**(*R*)-BPPOACZ**	20 wt% 26DCzPPy	543 [c]	86.1 [c]	1.1 [c]	0.04 [c]	1.85×10^{-2}	537	0.36, 0.57	15.1	N/A	2	4×10^{-3}	291
**(*R*)-M-BPCZ4**	25% in DPEPO	485	64	6.7	0.09	-5.2×10^{-3}	483	N/A	16.7	N/A	6	-3.8×10^{-3}	292
**(*R*)-P-BPCZ4**	25% in DPEPO	485	76	7.0	0.05	-4.7×10^{-3}	480	N/A	18.3	N/A	6	-5.5×10^{-3}	292
**(+)-Ax-o-CHO**	in DPEPO	460	3	1.04	0.19	1×10^{-3} [c]	N/A	N/A	N/A	N/A	N/A	N/A	293
**(+)-Hel-*o*-CHO**	in DPEPO	436	2	0.80	0.39	2×10^{-3} [d]	N/A	N/A	N/A	N/A	N/A	N/A	293
**B^1TPNF_2**	toluene	529	11	0.79	0	0.7×10^{-3}	N/A	N/A	N/A	N/A	N/A	N/A	294
**B^2TPNF_2**	toluene	530	29	0.953	0	2×10^{-3}	N/A	N/A	N/A	N/A	N/A	N/A	294
**$B^2CNPyrF_2$**	toluene	492	23	0.569	0.22	0.8×10^{-3}	N/A	N/A	N/A	N/A	N/A	N/A	294
**CP-TADF emitters with planar chirality**													
***g*-BNMe₂-Cp**	Toluene	531	46	0.38	0.17 [b]	4.2×10^{-3}	N/A	N/A	N/A	N/A	N/A	N/A	295
***rac*-CzpPhTrz**	10 wt% DPEPO	470 [c]	69	7	0.16	N/A	480	0.17, 0.25	17	29	N/A	N/A	296
**(*S*)-PXZp-Ph-TRZ**	10 wt% CBP	527	60	12.1	0.03	$+3.3 \times 10^{-3}$	539	0.42, 0.54	7.8	N/A	N/A	$+4.3 \times 10^{-3}$	297
**(*R*)-PXZ-PT**	10 wt% CBP	565 [c]	78	75	0.19 [b]	$+1.9 \times 10^{-3}$	557	0.44, 0.55	20.1	N/A	N/A	$+1.5 \times 10^{-3}$	298
**(*R*)-Czp-tBuCzB**	5 wt% 2,6DCzPPy	478 [c]	98	41.8	0.09 [c]	1.6×10^{-3}	479	0.11, 0.21	32.1	9	4	$+1.54 \times 10^{-3}$	299
**(*R*)-Czp-POAB**	8 wt% 2,6DCzPPy	497 [c]	96	62.4	0.13 [c]	1.4×10^{-3}	513	0.23, 0.65	28.7	2	29	$+1.30 \times 10^{-3}$	299
**(*P*)-QAO-PhCz**	5 wt% mCBP	460 [c]	46.6	40.36	0.11	1.1×10^{-3}	467	0.13, 0.18	14	N/A	N/A	$+1.50 \times 10^{-3}$	300
**(*M*)-QPO-PhCz**	Toluene	446	51	536	0.23	1.6×10^{-3}	488	0.17, 0.34	10.6	N/A	N/A	$+1.60 \times 10^{-3}$	301
**(*P*)-Hel-DiDiKTa**	1 wt% mCP	478	4.1	5.4	0.15	4.0×10^{-4}	N/A	N/A	N/A	N/A	N/A	N/A	302

**(*P*)-BTPT**	1 wt% PMMA	400	9	109	0.01	-2.8×10^{-3}	N/A	N/A	N/A	N/A	N/A	N/A	303
**BN4**	toluene	500	88	8.3	0.14	$(1\sim2)\times10^{-3}$	510	0.186, 0.632	20.6	0.4	48	$+3.70\times10^{-3}$	304
**BN5**	toluene	497	87	23.1	0.14	$(1\sim2)\times10^{-3}$	506	0.167, 0.603	26.5	34	58	-1.6×10^{-3}	304
**(*M*)-helicene-BN**	1 wt% DMIC-TRZ	525	98	71.8	0.15	-2.1×10^{-3} [c]	524	0.26, 0.66	30.7	8	42	2.2×10^{-3}	305
**R-BN**	toluene	662	100	0.18	16.6	2.1×10^{-3} [h]	664	0.719, 0.280	28.1	N/A	N/A	N/A	306,307
**R-TBN**	toluene	692	100	0.16	46.4	2.1×10^{-3} [h]	686	0.721, 0.278	27.6	N/A	N/A	N/A	306,307
**CP-TADF emitters featuring chiral perturbation**													
**(*R*)/(*S*)-1**	toluene	517	53	2.9	N/A	1.3×10^{-3}	~535 [a]	N/A	9.1 [a]	N/A	N/A	N/A	308
**(*S*)-BN-CF**	10 wt% mCP	493	32	24	N/A	1.2×10^{-3} [c]	496	0.25, 0.47	9.3	N/A	53	0.026	309
**(*S*)-BN-CCB**	10 wt% mCP	534	38	21	N/A	1.1×10^{-3}	527	0.34, 0.57	6.3	N/A	42	0.025	309
**(*S*)-BN-DCB**	10 wt% mCP	540	45	17	N/A	1.0×10^{-3}	547	0.42, 0.65	3.5	N/A	22	0.027	309
**(*S*)-BN-AF**	10 wt% mCP	571	13	32	N/A	1.3×10^{-3}	571	0.48, 0.51	1.7	N/A	8	0.016	309
**(*S*)-CPDCz**	10 wt% mCP	511	20	18	0.08	-3.3×10^{-4}	496	N/A	10.1	N/A	N/A	-3.7×10^{-3}	310
**(*S*)-CPDCB**	10 wt% mCP	533	55.2	10	0.04	-4.0×10^{-4}	516	N/A	10.6	N/A	N/A	-3.9×10^{-3}	310
**B1**	toluene	469	7 [e]	10	0.31 [b]	2.1×10^{-3}	N/A	N/A	N/A	N/A	N/A	N/A	311
**B2**	toluene	490	16 [e]	45	0.18 [b]	1.6×10^{-3}	N/A	N/A	N/A	N/A	N/A	N/A	311
**B3**	toluene	506	18 [e]	39	0.16 [b]	0.2×10^{-3}	N/A	N/A	N/A	N/A	N/A	N/A	311
**B4**	toluene	516	30 [e]	16	0.1 [b]	$<0.1\times10^{-3}$	N/A	N/A	N/A	N/A	N/A	N/A	311
**C'1**	toluene	481	29 [e]	6	0.28 [b]	1.1×10^{-3}	N/A	N/A	N/A	N/A	N/A	N/A	311
**C'2**	toluene	504	46 [e]	19	0.13 [b]	1.0×10^{-3}	N/A	N/A	N/A	N/A	N/A	N/A	311
**C'3**	toluene	510	42 [e]	17	0.11 [b]	1.1×10^{-3}	N/A	N/A	N/A	N/A	N/A	N/A	311
**C1**	toluene	493	25 [e]	18	0.22 [b]	$<0.1\times10^{-3}$	N/A	N/A	N/A	N/A	N/A	N/A	311
**C2**	toluene	511	31 [e]	40	0.11 [b]	$<0.1\times10^{-3}$	N/A	N/A	N/A	N/A	N/A	N/A	311
**C3**	toluene	519	47 [e]	22	0.1 [b]	0.7×10^{-3}	N/A	N/A	N/A	N/A	N/A	N/A	311
**(*R*)-DOBP**	Neat film	670	11	0.9	0.14	2.5×10^{-4} [f]	716	N/A	1.9	N/A	N/A	N/A	312
**(*R*)-HDOBP**	Neat film	662	9	0.7	0.08	1.5×10^{-4} [f]	700	N/A	0.7	N/A	N/A	N/A	312

**(*R*)-BDTPA**	10 wt% mCP	600	15.8	53.5 [c]	0.14 [c]	-4.7 × 10^{-3} [c]	598	0.57, 0.43	2.0	N/A	N/A	-1.6 × 10^{-3}	313
**OBNCz**	10 wt% 26DCzPPy	504	92	11.4	0.04 [c]	N/A	526	0.33, 0.59	32.6	N/A	3	-2.3 × 10^{-3}	314
**(*R*)-OBN-DPA**	10 wt% 26DCzPPy	538 [c]	84.67	13.5	0.09	1.88 × 10^{-3}	544	0.39, 0.57	12.3	6.5	6.5	2.3 × 10^{-3}	315
**(*R*)-OBS-Cz**	15 wt% mCP	504 [c]	73	3.2	0.04	+7.0× 10^{-3}	502	0.21, 0.49	15.0	N/A	3.3	+0.80 × 10^{-3}	316
**(*R*)-OBS-TCz**	15 wt% mCP	520 [c]	87	2.7	0.05	+6.4× 10^{-3}	514	0.26, 0.56	20.3	N/A	1	+0.50 × 10^{-3}	316
**(*R*)-OBN-2CN-BN**	3 wt% PhCzBCz	498	99 [c]	95.3	0.12	+9.0× 10^{-4}	496	0.11, 0.52	29.4	32	72	+1.43 × 10^{-3}	317
**(*R*)-OBN-4CN-BN**	3 wt% PhCzBCz	510	96 [c]	97.4	0.13	+8.0× 10^{-4}	508	0.14, 0.64	24.5	67	91	+4.6× 10^{-4}	317
**(*R*)-P**	10 wt% mCP	549	72	1.6	0.045 [b]	-5.8× 10^{-4}	546	0.408, 0.566	14.9	N/A	15	-1.5× 10^{-3}	318
**(*S*)-P**	10 wt% mCP	547	76	2.3	0.061 [b]	+6.4× 10^{-4}	544	0.404, 0.569	15.8	N/A	22	+1.6× 10^{-3}	318
**(*R*)-ODQPXZ**	15 wt% CBP	589 [c]	92	3.6	0.16 [c]	-4.6× 10^{-4}	548	0.38, 0.56	28.3	2	54	6.0× 10^{-4}	319
**(*R*)-ODPPXZ**	7 wt% CBP	630 [c]	89	3.7	0.07 [c]	-1.4× 10^{-3}	600	0.53, 0.45	20.3	15	55	2.4× 10^{-3}	319
**(+)-(*R*,*R*)-MC**	In neat film	505	78	1.76	0.069	+2.2× 10^{-3}	522	0.28, 0.54	17.1	N/A	4	+1.5× 10^{-3}	320
**(*R*)-OBN-AICz**	13 wt% mCBP	509	81	4	0.08 [g]	+2.6× 10^{-3}	514	0.27, 0.52	19.0	N/A	N/A	+4.7× 10^{-4}	321
**(+)-(*S*,*S*)-CAI-Cz**	15 wt% mCBP	528	98	130	0.06	-1.1 × 10^{-3}	520	N/A	19.7	29	72	-1.7 × 10^{-3}	322
**(-)-(*R*,*R*)-CAI-Cz**	15 wt% mCBP	N/A	N/A	N/A	N/A	1.1× 10^{-3}	520	N/A	19.8	28	68	2.3 × 10^{-3}	322
**(-)-(*R*,*R*)-CAI-DMAC**	6 wt% CBP	583 [c]	39.9	37.4	0.07	9.2 × 10^{-4}	592	N/A	12.4	22	67	N/A	323
**(*R*)-CzTA**	THF	528	4.7	0.48	N/A	+0.84× 10^{-3}	N/A	N/A	N/A	N/A	N/A	N/A	324
**(*S*)-CzTA**	THF	528	5.0	0.40	N/A	-1.03× 10^{-3}	N/A	N/A	N/A	N/A	N/A	N/A	324
**(*S*,*S*)-(+)-TpAc-TRZ**	In neat film	541	85	1.1	0.03	+1.9× 10^{-3}	534	0.35, 0.59	25.5	34	94	+1.5× 10^{-3}	325
**(*R*,*R*)-pTpAcDPS**	15 wt% mCP	487	63	1.07	0.03	-1.4× 10^{-3}	496	N/A	12.0	N/A	N/A	-1.1× 10^{-3}	326
**(*R*,*R*)-pTpAcBP**	10 wt% mCP	532	92	1.75	0.01	-1.0× 10^{-3}	534	N/A	22.1	N/A	N/A	-1.0× 10^{-3}	326
**chiral TADF exciplexes and LC emitters**													
**1:A**	1:A (1:2)	N/	19	0.7	0.16	7× 10^{-3}	N/A	N/A	N/A	N/A	N/A	N/A	327

		A											
**(*R*)-TRZ:TAPC**	1:1	520	39.5	5.81	0.012	2.24×10^{-3}	520	0.28, 0.57	12.7	N/A	24	7.25×10^{-3}	[328]
**FAC-PDMLM**	5wt% in 5CB LC	490 [c]	18 [c]	2.3 [c]	0.02 [c]	7.26×10^{-2}	N/A	N/A	N/A	N/A	N/A	N/A	[329]
**PXZ-PDMLM**	3wt% in 5CB LC	546 [c]	13 [c]	0.61 [c]	0.02 [c]	-5.45×10^{-2}	N/A	N/A	N/A	N/A	N/A	N/A	[329]

[a] in 20 wt% doped mCP film, [b] in MeTHF, [c] in Toluene, [d] in $CHCl_3$, [e] in PMMA, [f] in 1,4-dioxane, [g] in neat film, [h] in DCM.

Table **S5.** Photophysical properties of typical TADF exciplex systems and their OLEDs, reviewed in Section **8.2**.

**Compound**	**Medium**	**λ_{PL} / nm**	**Φ_{PL} / %**	**τ_d / µs**	**ΔE_{ST} / eV**	**λ_{EL} / nm**	**CIE_{XY}**	**EQE_{max} / %**	**Ref**
***m*-MTDATA:*t*-Bu-PBD**	1:1 Bulk	540	20	N/A	0.05	N/A	N/A	2.4	[330]
***m*-MTDATA:3TPYMB**	1:1 Bulk	540	26	N/A	N/A	~545	N/A	5.4	[330]
***m*-MTDATA:PPT**	30 mol%:1 Bulk	510	N/A	N/A	N/A	N/A	N/A	~6	[331]
***m*-MTDATA:PPT**	50 mol%:1 Bulk	510	28.5	N/A	N/A	N/A	N/A	10	[331]
***m*-MTDATA:PPT**	70 mol%:1 Bulk	510	N/A	N/A	N/A	N/A	N/A	~8	[331]
**TCTA:3P-T2T**	Interface	544	N/A	N/A	N/A	544	0.40, 0.55	7.7	[332]
**TCTA:3P-T2T**	1:1 Bulk	544	N/A	2.4	N/A	544	0.40, 0.45	7.8	[332]
**mCP:HAP-3MF**	8:92 Bulk	538	66.1	1.7,5.7	N/A	538	N/A	11.3	[333]
**mCP:HAP-3MF**	25:75 Bulk	550	55.7	N/A	N/A	550	N/A	N/A	[333]
**mCP:HAP-3MF**	50:50 Bulk	550	44	N/A	N/A	555	N/A	N/A	[333]
**TSBPA:PO-T2T**	1:1 Bulk	~540	100	2.2	≈0	528	0.33, 0.57	20	[334]
**NPB:PO-T2T**	1:1 Bulk	~600	4	0.53	≈0	585	0.53, 0.47	1.7	[334]
**TPD:PO-T2T**	1:1 Bulk	~600	8	0.37	≈0	585	0.52, 0.46	2.4	[334]
**TCBPA:PO-T2T**	1:1 Bulk	~545	93	2.2	≈0	542	0.38, 0.56	12.8	[334]
**mCP:PO-T2T**	1:1 Bulk	~480	55	1.96	≈0.01	480	0.16, 0.28	16	[334]
**CzSi:PO-T2T**	1:1 Bulk	~465	24	6.3	≈0.1	465	0.16, 0.21	6.1	[334]
**mCPPO1:PO-T2T**	1:1 Bulk	~480	20	2.63	≈0.02	480	0.16, 0.29	6.5	[334]
**Compound 1 :PO-T2T**	1:1 Bulk	489	4 (air)	0.86	N/A	541	N/A	6.5	[335]
**Compound 2 :PO-T2T**	1:1 Bulk	470	2 (air)	0.88	N/A	546	N/A	7.8	[335]
**mCP:PO-T2T**	1:1 Bulk	472	7.3	N/A	0.010	476	0.17, 0.26	8.6	[336]
**MAC:PO-T2T**	1:1 Bulk	514	8	N/A	0.014	516	0.31, 0.55	13.3	[336]
**MAC:PO-T2T**	7:3 Bulk	514	8	N/A	0.014	516	0.31, 0.55	17.8	[336]
**TAPC:CzPyCN**	1:1 Bulk	530	N/A	1.097	N/A	536	0.38, 0.53	7.4	[337]
**TAPC:CbPyCN**	1:1 Bulk	520	N/A	1.014	N/A	520	0.33, 0.52	9.1	[337]
**BFPD:TAPC**	10:90 Bulk	488	55.1	N/A	0.04	508	N/A	7.6	[338]
**BFPD:TAPC**	15:85 Bulk	490	60	N/A	0.04	509	N/A	9.8	[338]
**BFPD:TAPC**	20:80 Bulk	492	56.6	N/A	0.04	N/A	N/A	N/A	[338]
**BFPD:TAPC**	50:50 Bulk	511	50.2	N/A	0.04	518	N/A	10.5	[338]
**BFPD:TCTA**	10:90 Bulk	475	19.1	N/A	0.2	N/A	N/A	N/A	[338]
**BFPD:TCTA**	50:50 Bulk	485	25.4	N/A	0.2	N/A	N/A	N/A	[338]

**BFPD**:**m-MTDATA**	10:90 Bulk	565	10.4	N/A	0.06	N/A	N/A	N/A	338
**BFPD**:**TPD**	10:90 Bulk	494	13.9	N/A	N/A	N/A	N/A	N/A	338
**BFPD**:**Spiro-BPA**	10:90 Bulk	475	21.6	N/A	N/A	N/A	N/A	N/A	338
**BFPD**:**m-CBP**	10:90 Bulk	407	20	N/A	N/A	N/A	N/A	N/A	338
**BPD**:**m-MTDATA**	10:90 Bulk	548	9	N/A	N/A	N/A	N/A	N/A	338
**BPD**:**TAPC**	10:90 Bulk	472	26.6	N/A	N/A	N/A	N/A	N/A	338
**BPD**:**TAPC**	20:80 Bulk	480	33.7	N/A	N/A	N/A	N/A	N/A	338
**BPD**:**TAPC**	50:50 Bulk	493	35.4	N/A	N/A	N/A	N/A	N/A	338
**TCTA**:**DCNSFX**	1:1 Bulk	520	31	4.67	0.05	520	0.33, 0.52	3.0	339
**TCTA**:**TRZSFX**	1:1 Bulk	510	81	0.18	0.03	520	0.35, 0.60	22.5	340
**TCTA**:**DTRZSFX**	1:1 Bulk	539	41	0.28	0.06	560	0.44, 0.51	9.7	340
**mCP**:**DPOPht$_2$**	1:1 Bulk	498	0.8	~0.14	N/A	N/A	N/A	N/A	341
**mCP**:**CyPh$_2$Pht$_2$**	1:1 Bulk	496	2	~0.15	N/A	N/A	N/A	N/A	341
**mCP**:**2-BpPht**	1:1 Bulk	494	18	~0.42	N/A	N/A	N/A	N/A	341
**mCP**:**3-BpPht**	1:1 Bulk	495	20	~0.42	N/A	N/A	N/A	N/A	341
**mCP**:**4-BpPht**	1:1 Bulk	497	26	~0.42	0.06	N/A	0.24, 0.41	2.9	341
**CBP**:**DPOPht$_2$**	1:1 Bulk	510	1.6	~0.10	N/A	N/A	N/A	N/A	341
**CBP**:**CyPh$_2$Pht$_2$**	1:1 Bulk	503	4	~0.13	N/A	N/A	N/A	N/A	341
**CBP**:**2-BpPht**	1:1 Bulk	504	17	~0.19	N/A	N/A	N/A	N/A	341
**CBP**:**3-BpPht**	1:1 Bulk	503	19	~0.18	N/A	N/A	N/A	N/A	341
**CBP**:**4-BpPht**	1:1 Bulk	510	22	~0.17	N/A	N/A	N/A	N/A	341
**PVK**:**DPOPht$_2$**	1:1 Bulk	540	2.5	~0.20	N/A	N/A	N/A	N/A	341
**PVK**:**CyPh$_2$Pht$_2$**	1:1 Bulk	530	3	~0.25	N/A	N/A	N/A	N/A	341
**PVK**:**2-BpPht**	1:1 Bulk	525	6	~0.35	N/A	N/A	N/A	N/A	341
**PVK**:**3-BpPht**	1:1 Bulk	528	6	~0.39	N/A	N/A	N/A	N/A	341
**PVK**:**4-BpPht**	1:1 Bulk	533	8	~0.29	N/A	N/A	N/A	N/A	341
**TCTA**:**DPOPht$_2$**	1:1 Bulk	570	2	~0.12	N/A	N/A	N/A	N/A	341
**TCTA**:**CyPh$_2$Pht$_2$**	1:1 Bulk	566	2	~0.14	N/A	N/A	N/A	N/A	341
**TCTA**:**2-BpPht**	1:1 Bulk	569	2	~0.23	N/A	N/A	N/A	N/A	341
**TCTA**:**3-BpPht**	1:1 Bulk	563	3	~0.17	N/A	N/A	N/A	N/A	341
**TCTA**:**4-BpPht**	1:1 Bulk	575	5	~0.18	N/A	N/A	N/A	N/A	341
**Ir-817**: **B2PyMPM**	2:8 Bulk	606	13.7	11.8	0.012	620	0.58, 0.42	3.10	342
**Ir-817**: **B3PyMPM**	2:8 Bulk	632	7.2	9.65	0.020	640	0.62, 0.37	1.45	342
**Ir-817**: **B4PyMPM**	2:8 Bulk	642	4.6	9.11	0.015	672	0.66,0.33	1.03	342
**Ir-817**:**TRZ-1SO$_2$**	2:8 Bulk	647	N/A	N/A	0.020	658	0.64, 0.35	0.26	342
**Ir-817**:**TRZ-2SO$_2$**	2:8 Bulk	666	N/A	N/A	0.017	700	0.68, 0.31	0.22	342
**Ir-817**:**TRZ-3SO$_2$**	2:8 Bulk	698	N/A	N/A	0.019	746	N/A	0.20	342

Table **S6.** Photophysical properties and device performance of exciplexes with a TADF molecule as an elemental component, reviewed in Section **8.3**.

**Compound**	**Medium**	**λ_{PL} / nm**	**Φ_{PL} / %**	**τ_d / μs**	**ΔE_{ST} / eV**	**λ_{EL} / nm**	**CIE_{XY}**	**EQE_{max} / %**	**Ref**
**mCP**:**PO-T2T**	1:1 Bulk	472	7.3 (air)	N/A	0.01	476	0.17, 0.26	8.6	336

**MAC**:**PO-T2T**	1:1 Bulk	514	8.0 (air)	N/A	0.014	516	0.31, 0.55	13.3	[336]
**MAC**:**PO-T2T**	8:2 Bulk	N/A	N/A	N/A	N/A	516	0.31, 0.55	13.1	[336]
**MAC**:**PO-T2T**	7:3 Bulk	N/A	N/A	N/A	N/A	516	0.31, 0.55	17.8	[336]
**DBT-SADF**:**PO-T2T**	6:4 Bulk	516	38	20.5	0.032	524	0.29, 0.55	16.9	[343]
**DBT-SADF**:**PO-T2T**:**CDBP**	2:5:3 Bulk		61	16.9	N/A	516	0.26, 0.53	20.5	[343]
**13AB**:**PO-T2T**	5:5 Bulk	522	15	14	0.047	540	0.37, 0.57	12.4	[343]
**13AB**:**PO:T2T**:**CDBP**	1:1:1 Bulk	N/A	40	18.6	N/A	532	0.32, 0.56	15.5	[343]
**CDBP**:**PO-T2T**	50:50 Bulk	494	53	17.2	N/A	494	0.19, 0.37	8.9	[344]
**PO-T2T**:**DABNA-1**	50:50 Bulk	550	46	14.7	N/A	550	0.45, 0.56	8.9	[344]
**CDBP:PO-T2T**: **DABNA-1**	47.5:47.5:5 Bulk	~550	67	15.1	N/A	N/A	0.31, 0.58	17.5	[344]
**CDBP:PO-T2T**:**DABNA-1**	45:45:10 Bulk	~550	69	15.1	N/A	N/A	0.34, 0.60	16.9	[344]
**CDBP**:**PO-T2T**:**DABNA-1**	40:40:20 Bulk	~550	50	15.1	N/A	N/A	0.37, 0.60	13.4	[344]
**NPD/Xan-Cbz**	Interface	N/A	N/A	N/A	N/A	525 (at 9 V)	N/A	N/A	[345]
**DPSTPA**:**2CzPN**	3:1 Bulk	540	79	13.9	0.095	544	0.38, 0.55	19.0	[346]
**DPSTPA**:**CzDBA**	3:1 Bulk	592	59	10.1	0.078	592	0.53, 0.46	14.6	[346]
**DPSTPA**:**4CzIPN**	3:1 Bulk	596	10	6.8	0.066	590	0.50, 0.48	3.8	[346]
**TXO-TPA**:**APDC-tPh**	1:1 Bulk	690	0.09	~0.3	N/A	704	N/A	1.27	[347]
**TXO-TPA**:**APDC-tPh**	4:1 Bulk	N/A	N/A	N/A	N/A	689	N/A	N/A	[347]
**TXO-TPA**:**APDC-tPh**	2:3 Bulk	N/A	N/A	N/A	N/A	710	N/A	N/A	[347]
**TCTA**:**APDC-tPh**	1:1 Bulk	720	N/A	0.06	N/A	730	N/A	0.09	[347]
**TCTA**:**APDC-tPh**	2:1 Bulk	N/A	N/A	N/A	N/A	~700	N/A	N/A	[347]
**TCTA**:**APDC-tPh**	1:2 Bulk	N/A	N/A	N/A	N/A	~770	N/A	N/A	[347]
**PO-01**:**AQDC-tPh**	5:95 Bulk	727	N/A	0.72	N/A	732	N/A	0.16	[348]
**PO-01**:**AQDC-tPh**	10:90 Bulk	737	N/A	N/A	N/A	744	N/A	0.17	[348]
**PO-01**:**AQDC-tPh**	15:85 Bulk	742	N/A	N/A	N/A	750	N/A	0.23	[348]
**PO-01**:**AQDC-tPh**	25:75 Bulk	752	N/A	0.48	N/A	756	N/A	0.20	[348]
**PO-01**:**APDC-tPh**	5:95 Bulk	N/A	N/A	N/A	N/A	800	N/A	0.14	[348]
**PO-01**:**APDC-tPh**	15:85 Bulk	N/A	N/A	N/A	N/A	824	N/A	0.16	[348]
**PO-01**:**APDC-tPh**	20:80 Bulk	N/A	N/A	N/A	N/A	844	N/A	0.12	[348]
**Ir(ppy)$_2$acac**:**AQDC-tPh**	5:95 Bulk	717	N/A	0.81	N/A	733	N/A	0.14	[348]
**Ir(ppy)$_2$acac**:**AQDC-tPh**	10:90 Bulk	721	N/A	N/A	N/A	737	N/A	0.15	[348]
**Ir(ppy)$_2$acac**:**AQDC-tPh**	15:85 Bulk	727	N/A	N/A	N/A	742	N/A	0.17	[348]
**Ir(ppy)$_2$acac**:**AQDC-tPh**	25:75 Bulk	735	N/A	0.48	N/A	748	N/A	0.19	[348]

**TAPC:3Cz-o-TRz**	1:1 Bulk	510	68	1.8	N/A	520	N/A	12.1	349
**TPA-PXZ:3Cz-o-TRz**	1:1 Bulk	530	36	N/A	N/A	540	N/A	10.1	349
**m-MTDATA: 3Cz-o-TRz**	1:1 Bulk	580	5	N/A	N/A	588	N/A	1.1	349
**3Cz-o-TRz: B3PyMPM**	1:1 Bulk	465	19	N/A	N/A	516	N/A	5.5	349
**3Cz-o-TRz: B4PyMPM**	1:1 Bulk	475	25	N/A	N/A	520	N/A	6.2	349
**3Cz-o-TRz: PO-T2T**	1:1 Bulk	510	66	1.5	N/A	516	N/A	11.8	349

Table **S7.** Photophysical properties of bulk and interfacial exciplexes used to understand and improve TADF exciplex systems and their device performance, reviewed in Section **8.4**.

**Compound**	**Medium**	λ_{PL} **/ nm**	Φ_{PL} **/ %**	τ_d **/ μs**	ΔE_{ST} **/ eV**	λ_{EL} **/ nm**	**CIE**$_{XY}$	**EQE**$_{max}$ **/ %**	**Ref**
**DSDTAF:3N-T2T**	1:1 Bulk	535	59	2.54	N/A	535	0.37, 0.58	13.2	350
**DTAF:3N-T2T**	1:1 Bulk	535	51	2.91	N/A	551	0.42, 0.55	11.6	350
**CPTBF:PO-T2T**	1:1 Bulk	~480	44	5.86	N/A	~480	0.17, 0.29	12.5	350
**CPF:PO-T2T**	1:1 Bulk	~480	41	2.8	N/A	~480	0.18, 0.31	9.5	350
**m-MTDATA:4**	1:1 Bulk	584	3.8	0.19	N/A	N/A	N/A	N/A	351
**TCTA:4**	1:1 Bulk	490	43.8	0.31	0.05	N/A	N/A	N/A	351
**4/Bphen**	Interface	N/A	N/A	N/A	N/A	¬475	0.17, 0.28	2.0	351
**m-MTDATA/TCTA:4/Bphen**	1:1 Bulk	N/A	N/A	N/A	N/A	560	0.40, 0.52	5.8	351
**TCTA:4/4/Bphen**	1:1 Bulk/Interface	N/A	N/A	N/A	N/A	¬490	N/A	4.2	351
**m-MTDATA:4/4/Bphen**	1:1 Bulk/Interface	N/A	N/A	N/A	N/A	¬600	N/A	3.2	351
**TSBPA:PO-T2T**	1:1 Bulk	~535	58	2.4	N/A	~545	N/A	14.8	352
**TSBPA:PO-T2T**	1:1 Bulk 10 wt% in UGH-3	~535	63	2.2	N/A	~545	N/A	~15.5	352
**TSBPA:PO-T2T**	1:1 Bulk 30 wt% in UGH-3	~535	65	2.8	N/A	~540	N/A	~16.5	352
**TSBPA:PO-T2T**	1:1 Bulk 50 wt% in UGH-3	~530	80	3.1	N/A	~535	N/A	19.2	352
**TSPBA:PO-T2T**	1:1 Bulk 70 wt% in UGH-3	~525	70	3.1	N/A	~530	N/A	~14.5	352
**TSPBA:PO-T2T**	1:1 Bulk 90 wt% in UGH-3	~490	35	N/A	N/A	~520	N/A	N/A	352
**TSBPA:PO-T2T**	1:1 Bulk 10 wt% in DPEPO	~530	68	2.4	N/A	~530	N/A	~15.5	352
**TSBPA:PO-T2T**	1:1 Bulk 30 wt% in DPEPO	~530	45	2.5	N/A	~530	N/A	~16.0	352

**TSBPA**:**PO-T2T**	1:1 Bulk 50 wt% in DPEPO	~525	28	2.3	N/A	~530	N/A	~17.0	352
**TSPBA**:**PO-T2T**	1:1 Bulk 70 wt% in DPEPO	~520	38	2.9	N/A	~525	N/A	~13.5	352
**TSPBA**:**PO-T2T**	1:1 Bulk 90 wt% in DPEPO	~510	0.07	2.8	N/A	~520	N/A	N/A	352
**TCTA**:**PO-T2T**:**mCP**	1:1:0 Bulk	~565	13.8	1.1315	0.0695	558	N/A	3.9	353
**TCTA**:**PO-T2T**:**mCP**	1:1:0.5 Bulk	~550	21.4	1.5596	N/A	542	N/A	5.7	353
**TCTA**:**PO-T2T**:**mCP**	1:1:1 Bulk	~550	31.2	2.0103	0.0231	535	N/A	6.1	353
**TCTA**:**PO-T2T**:**mCP**	1:1:3 Bulk 5wt% **Ir(tptpy)$_2$(acac)**	~525	37.9	2.3787	N/A	523	N/A	8.0	353
**TCTA**:**PO-T2T**:**mCP**	1:1:0 Bulk **Ir(tptpy)$_2$(acac)**	N/A	N/A	N/A	N/A	~555	N/A	19.3	353
**TCTA**:**PO-T2T**:**mCP**	1:1:0.5 Bulk **Ir(tptpy)$_2$(acac)**	N/A	N/A	N/A	N/A	~555	N/A	20.9	353
**TCTA**:**PO-T2T**:**mCP**	1:1:1 Bulk **Ir(tptpy)$_2$(acac)**	N/A	N/A	N/A	N/A	~555	N/A	21.0	353
**TCTA**:**PO-T2T**:**mCP**	1:1:3 Bulk **Ir(tptpy)$_2$(acac)**	N/A	N/A	N/A	N/A	~555	N/A	21.7	353
**TAPC**:**DMA**	1:1 Bulk	475	9.8	0.284	N/A	N/A	N/A	N/A	354
**DMA**:**DCA**	1:1 Bulk	470	39.0	0.125	N/A	N/A	N/A	N/A	354
**TAPC**:**DCA**	1:1 Bulk	550	0.5	0.299	N/A	N/A	N/A	N/A	354
**TAPC/DMA/DCA**	20/0/10 (nm) Interfacial	534	N/A	0.160	N/A	445/550	N/A	0.14	354
**TAPC/DMA/DCA**	20/10/10 (nm) Interfacial	534	N/A	0.160	N/A	445/550	N/A	0.43	354
**TAPC/DMA/DCA**	20/20/10 (nm) Interfacial	534	N/A	0.253	N/A	445/550	N/A	0.37	354
**TAPC/DMA/DCA**	20/30/10 (nm) Interfacial	534	N/A	0.292	N/A	445/550	N/A	0.33	354
**TAPC/DMA/DCA**	20/40/10 (nm) Interfacial	534	N/A	N/A	N/A	445/550	N/A	0.42	354
**TAPC/DMA/DCA**	20/50/10 (nm) Interfacial	534	N/A	0.242	N/A	445/550	N/A	0.48	354
**TAPC/DMA/DCA**	20/60/10 (nm) Interfacial	534	N/A	N/A	N/A	445	N/A	0.69	354
**TAPC/DMA/DCA**	20/70/10 (nm) Interfacial	534	N/A	0.232	N/A	445	N/A	0.86	354
**TAPC/CBP/DCA**	20/1/10 (nm) Interfacial	N/A	N/A	0.162	N/A	550	N/A	0.33	354
**TAPC/CBP/DCA**	20/5/10 (nm) Interfacial	N/A	N/A	0.145	N/A	550	N/A	0.55	354
**TAPC/CBP/DCA**	20/10/10 (nm)	N/A	N/A	0.111	N/A	550	N/A	0.72	354

	Interfacial								
**TAPC/CBP/DCA**	20/15/10 (nm) Interfacial	N/A	N/A	0.0849	N/A	550	N/A	1.6	354
**TAPC/CBP/DCA**	20/20/10 (nm) Interfacial	N/A	N/A	0.0161	N/A	550	N/A	3.0	354
**PO-T2T**:**Ir(ppy)$_3$**	92:8 Bulk	N/A	23.3	2.8	0.026	604	0.55, 0.44	5	355
**13PXZB:PO-T2T**	60:40 Bulk	N/A	8.6	13.9	N/A	592	0.52, 0.47	1.9	355
**PIPAQ**	-	650	12.3	40.5	0.13	650	0.64, 0.36	2.10	356
**Tr-Ph**:**3P-T2T**	1:1 Bulk	553	39	1.95	0.10	558	0.43, 0.53	10.3	357
**Tr-Ph**:**3P-T2P**	1:1 Bulk	526	41	1.77	0.18	522	0.33, 0.54	10.4	357
**Tr-Ph**:**3P-Pyr**	1:1 Bulk	480	8	0.07	0.34	469	0.19, 0.24	0.54	357
**Tr-Tol**:**3P-T2T**	1:1 Bulk	553	33	1.73	0.10	566	0.44, 0.53	9.7	357
**Tr-Tol**:**3P-T2P**	1:1 Bulk	525	40	2.39	0.10	526	0.35, 0.54	12.8	357
**Tr-Tol**:**3P-Pyr**	1:1 Bulk	491	10	1.0	0.33	467	0.19, 0.25	0.55	357
**Tr-Ph**:**3P-T2T**	10wt% **(DT)$_2$BTh2CN**	N/A	40	0.15	N/A	674	0.69, 0.31	4.64	357
**Tr-Ph**:**3P-T2P**	10wt% **(DT)$_2$BTh2CN**	N/A	50	0.12	N/A	671	0.68, 0.31	5.52	357
**Tr-Tol**:**3P-T2T**	10wt% **(DT)$_2$BTh2CN**	N/A	37	0.10	N/A	669	0.69, 0.31	3.48	357
**Tr-Tol**:**3P-T2P**	10wt% **(DT)$_2$BTh2CN**	N/A	47	0.12	N/A	667	0.67, 0.32	4.42	357
**Tr-Me**:**PO-T2T**	1:1 Bulk (1:2 for Devices)	615	3	0.294	0.11	633	0.62, 0.40	0.48	358
**Tr-Me**:**PO-T2P**	1:1 Bulk (1:2 for Devices)	562	14	0.294	0.02	606	0.53, 0.46	2.20	358
**Tr-Me**:**PO-Pyr**	1:1 Bulk (1:2 for Devices)	549	21	0.054	0.04	551	0.41, 0.54	5.76	358
**Tr-iBu**:**PO-T2T**	1:1 Bulk (1:2 for Devices)	580	10	0.706	0.03	580	0.51, 0.48	2.28	358
**Tr-iBu**:**PO-T2P**	1:1 Bulk (1:2 for Devices)	552	37	2.34	0.02	560	0.43, 0.54	8.27	358
**Tr-iBu**:**PO-Pyr**	1:1 Bulk (1:2 for Devices)	516	32	2.39	0.10	516	0.27, 0.50	7.54	358
**Tr-iBu**:**PO-Pyr**	1:2 Bulk 3wt% **DPy2CN**	N/A	44	0.14	N/A	674	0.63, 0.35	6.28	358
**Tr-iBu**:**PO-Pyr**	1:2 Bulk 5wt% **DPy2CN**	N/A	44	0.14	N/A	678	0.65, 0.34	4.43	358
**Tr-iBu**:**PO-Pyr**	1:2 Bulk 10wt% **DPy2CN**	N/A	44	0.14	N/A	680	0.66, 0.33	4.06	358
**mCP**:**TXO-P-Si**	7:1 Bulk (96:4 for Devices)	~520	55.4	2.9	0.024	N/A	N/A	16.9	359
**CBP**:**TXO-P-Si**	7:1 Bulk (99:1 for Devices)	~534	47.6	2.1	0.0063	N/A	N/A	13.7	359
**3,5-DCzPPy**: **TXO-P-Si**	7:1 Bulk (99:1 for Devices)	~534	47.7	20	0.055	N/A	N/A	16.1	359

**PPO21**:**TXO-P-Si**	7:1 Bulk (90:10 for Devices)	~510	25.8	30	0.00064	N/A	N/A	7.3	359
**CPTBF**:**3,4-CN**	1:1 Bulk	522	18	0.12	N/A	524	0.35, 0.58	6.87	360
α **−CPTBF**:**3,4-CN**	1:1 Bulk	504	21	0.10	N/A	508	0.30, 0.56	7.57	360
β **−CPTBF**:**3,4-CN**	1:1 Bulk	504	25	0.10	N/A	510	0.30, 0.56	7.34	360
**13PXZB**:**B4PyMPM**	7:3 Bulk	543	69.6	9.8	0.035	560	0.41, 0.55	14.6	361
**13PXZB**:**B3PyMPM**	7:3 Bulk	529	46.5	12.4	0.037	552	0.40, 0.55	10.0	361
**13PXZB**:**B2PyMPM**	7:3 Bulk	525	33.1	14.0	0.032	548	0.39, 0.55	6.2	361
**13AB**:**B4PyMPM**	7:3 Bulk	N/A	29.8	13.6	N/A	548	0.36, 0.55	6.0	361
**13AB**:**B3PyMPM**	7:3 Bulk	N/A	28.1	12.4	N/A	540	0.31, 0.50	5.4	361
**13AB**:**B2PyMPM**	7:3 Bulk	N/A	25.3	11.9	N/A	524	0.29, 0.47	5.3	361
**1**:**B01**	1:1 Bulk	461	N/A	45.1	N/A	536	N/A	5.4	362
**BTCz-PCz**: **TmPyPB**	99:1 Bulk	N/A	N/A	N/A	N/A	436	0.19, 0.19	0.96	363
**BTDCb-PCz**:**TmPyPB**	99:1 Bulk	N/A	N/A	0.052	N/A	468	0.16, 0.21	2.36	363
**DCb-PCz**: **TmPyPB**	99:1 Bulk	N/A	N/A	N/A	N/A	436	0.19, 0.18	0.56	363
**BTCz-PCz/ TmPyPB**	Interface	N/A	N/A	N/A	N/A	435/500	0.24, 0.32	1.89	363
**BTDCb-PCz/TmPyPB**	Interface	N/A	N/A	N/A	N/A	435/500	0.20, 0.31	1.05	363
**DCb-PCz/ TmPyPB**	Interface	N/A	N/A	N/A	N/A	435/500	0.19, 0.22	2.43	363
**BTDCb-PCz**:**TmPyPB**	50:50 Bulk	N/A	N/A	N/A	N/A	500	0.21, 0.32	1.0	363
**mCP/PO-T2T**	Interface	472	N/A	N/A	N/A	497	N/A	8.2	364
**mCP/PO-T2T**	Interface Annealed	472	N/A	N/A	N/A	570	N/A	8.2	364
**FCF/PO-T2T**	Interface	475	N/A	N/A	N/A	492	N/A	6.2	364
**FCF/PO-T2T**	Interface Annealed	482	N/A	N/A	N/A	575	N/A	6.2	364
**CFC2/PO-T2T**	Interface	483	N/A	N/A	N/A	522	N/A	2.6	364
**CFC2/PO-T2T**	Interface Annealed	524	N/A	N/A	N/A	576	N/A	2.6	364
**FFF/PO-T2T**	Interface	470	N/A	N/A	N/A	477	N/A	0.8	364
**FFF/PO-T2T**	Interface Annealed	537	N/A	N/A	N/A	570	N/A	0.8	364
**TCz1/PO-T2T**	Interface	501	N/A	N/A	N/A	524	N/A	18.0	364
**TCz1/PO-T2T**	Interface Annealed	517	N/A	N/A	N/A	524	N/A	18.0	364
**NPB/TPBi**:**PPh3O/PPh3O**	5:1 Bulk	~455	N/A	1.99	N/A	435	N/A	4.0	365
**NPB/NPB**:**TPBi**	1:1 Bulk	~455	N/A	0.6	N/A	434	N/A	1.5	365
**NPB/NPB**:**TPBi/TPBi**	1:1 Bulk	~455	N/A	0.6	N/A	442	N/A	1.9	365
**mCP**:**HAP-3FDPA**:	92:8 Bulk	433	53.2	0.09	N/A	437	0.16, 0.12	10.2	366

Table **S8.** Photophysical properties and device performance of TADF exciplexes used either as hosts, or in solution-processed devices, reviewed in Sections **8.5** and **8.6**.

Compound	Medium	λ_{PL} / nm	Φ_{PL} / %	τ_d / µs	ΔE_{ST} / eV	λ_{EL} / nm	CIE_{XY}	EQE_{max} / %	Ref
**Tris-PCz**:**CN-T2T**	1:1 Bulk 1wt% **NOz-TPA**	680	42	0.131	0.025	670	N/A	4.6	367
**Tris-PCz**:**CN-T2T**	1:1 Bulk 5wt% **NOz-TPA**	697	29	0.043	0.025	689	N/A	5.0	367
**Tris-PCz**:**CN-T2T**	1:1 Bulk 10wt% **NOz-TPA**	698	29	0.031	0.025	700	N/A	4.3	367
**Tris-PCz**:**CN-T2T**	1:1 Bulk 1wt% **NOz-t-TPA**	709	28	0.252	0.025	683	N/A	7.4	367
**Tris-PCz**:**CN-T2T**	1:1 Bulk 5wt% **NOz-t-TPA**	710	26	0.091	0.025	697	N/A	6.9	367
**Tris-PCz**:**CN-T2T**	1:1 Bulk 10wt% **NOz-t-TPA**	715	20	N/A	0.025	710	N/A	6.6	367
**DPSF**:**CN-T2T**	1:1 Bulk	569	15	2.1	0.09	584	N/A	6.0	368
**DTSF**:**CN-T2T**	1:1 Bulk	594	12	2.0	0.05	607	N/A	4.9	368
**DPSF**:**CN-T2T**	1:1 Bulk 7wt% **TTDSF**	746	26	0.49	N/A	774	N/A	5.3	368
**DTSF**:**CN-T2T**	1:1 Bulk 7wt% **TTDSF**	746	25	0.76	N/A	774	N/A	4.0	368
**CBP**:**PO-T2T**	1:1 Bulk	480	N/A	N/A	N/A	N/A	N/A	N/A	239
**CBP**	15wt% **TPA–PZCN**	~635	N/A	N/A	N/A	628	0.65, 0.35	27.4	239
**CBP**:**PO-T2T**	1:1 Bulk 15wt% **TPA–PZCN**	~635	N/A	N/A	N/A	648	0.66, 0.34	28.1	239
**CDBP**:**PO-T2T**	1:1 Bulk 6 wt% **DBBPZ-DPXZ**	620	87	1.3	0.04	628	0.62, 0.38	20.8	369
**CDBP**:**PO-T2T**	1:1 Bulk 0.2 wt% **DBBPZ-DPXZ**	N/A	N/A	N/A	N/A	479,609	0.40, 0.37	20.7	369
**TCTA**:**B3PYMPM**	1:1 Bulk 7 wt% **DBBPZ-DACT-II**	~525	96	N/A	N/A	N/A	N/A	34.2	370
**CBP/B4PyMPM**	Interfacial	436	N/A	N/A	N/A	N/A	N/A	N/A	371
**CBP/B4PyPPM**	Interfacial	442	N/A	N/A	N/A	N/A	N/A	N/A	371
4 wt% **DACT-II**:**CBP/B4PyMPM**	Interfacial	N/A	N/A	N/A	N/A	N/A	N/A	30.4	371
9 wt% **DACT-II**:**CBP/B4PyMPM**	Interfacial	N/A	N/A	N/A	N/A	N/A	N/A	31.7	371
15 wt% **DACT-II**:**CBP/B4PyMPM**	Interfacial	N/A	N/A	N/A	N/A	N/A	N/A	30.8	371
9 wt% **DACT-**	Interfacial	N/A	N/A	N/A	N/A	N/A	N/A	29.8	371

**II**:**CBP**/**B4PyPPM**									
20 wt% **DACT-II**:**CBP**/**B4PyPPM**	Interfacial	N/A	N/A	N/A	N/A	534	0.37, 0.59	26.8	371
20 wt% **DACT-II**:**CBP**/**B4PyMPM**	Interfacial	N/A	N/A	N/A	N/A	N/A	N/A	27.6	371
20 wt% **DACT-II**:**CBP**/**B3PyMPM**	Interfacial	N/A	N/A	N/A	N/A	N/A	N/A	27.0	371
9 wt% **DACT-II**:**TCTA**/**B4PyPPM**	Interfacial	N/A	N/A	N/A	N/A	N/A	N/A	28.6	371
9 wt% **DACT-II**:**TCTA**/**B4PyMPM**	Interfacial	N/A	N/A	N/A	N/A	N/A	N/A	30.3	371
**PhCNCzp-Me**:**B3PyMPM**	1:1 Bulk 8wt% **Ir(ppy)$_2$(acac)**	437	N/A	0.077	0.35	N/A	0.334, 0.623	15.66	372
**PhCzp-Me**:**B3PyMPM**	1:1 Bulk 8wt% **Ir(ppy)$_2$(acac)**	449	N/A	0.160	0.26	N/A	0.338, 0.627	27.84	372
**PhCNCzp-MeCzPh**:**B3PyMPM**	1:1 Bulk 8wt% **Ir(ppy)$_2$(acac)**	438	N/A	0.114	0.28	N/A	0.335, 0.630	25.59	372
**PhCzm-Me**:**B3PyMPM**	1:1 Bulk 8wt% **Ir(ppy)$_2$(acac)**	454	N/A	0.143	0.36	N/A	0.330, 0.633	29.38	372
**PhCNCzm-MeCzPh**:**B3PyMPM**	1:1 Bulk 8wt% **Ir(ppy)$_2$(acac)**	449	N/A	0.146	0.32	N/A	0.316, 0.641	31.52	372
**m-MTDATA**: **TmPyPB**	70:30 Bulk	479	12.3	0.073	0.04	N/A	N/A	N/A	373
**m-MTDATA**: **TmPyPB**	50:50 Bulk	484	10.5	N/A	N/A	N/A	N/A	N/A	373
**m-MTDATA**: **TmPyPB**	30:70 Bulk	490	10.2	N/A	N/A	N/A	N/A	N/A	373
**m-MTDATA**: **TmPyPB**	70:30 Bulk 2wt% **FIrpic**	N/A	N/A	N/A	N/A	N/A	0.06, 0.25	0.9	373
**m-MTDATA**: **TmPyPB**	70:30 Bulk 2wt% **Ir(ppy)$_3$**	N/A	N/A	N/A	N/A	~500	0.32, 0.61	10	373
**m-MTDATA**: **TmPyPB**	70:30 Bulk 2wt% **Ir(bt)$_2$(acac)**	N/A	N/A	N/A	N/A	N/A	0.51, 0.49	18.5	373
**m-MTDATA**: **TmPyPB**	70:30 Bulk 2wt% **Ir(piq)$_2$(acac)**	N/A	N/A	N/A	N/A	~620	0.67, 0.33	10	373
**m-MTDATA**: **TmPyPB**/10wt% **FIrpic**:**TCTA**	70:30 Bulk 2wt% **Ir(bt)$_2$(acac)**	N/A	N/A	N/A	N/A	N/A	0.36, 0.41	11.9	373
**BCzPh**:**3P-T2T**	2:1 Bulk	536	68	N/A	N/A	N/A	N/A	13.5	374
**BCzPh**:**3P-T2T**	2:1 Bulk 1wt% **C545T**	516	97	N/A	N/A	N/A	N/A	15.5	374
**BCzPh**:**3P-T2T**	2:1 Bulk	523	85	N/A	N/A	N/A	N/A	29.7	374

	8wt% **Ir(ppy)2(acac)**								
**CBP**	8wt% **Ir(ppy)2(acac)**	N/A	96	0.785	N/A	N/A	N/A	29.5	374
**TAPC**:**PIM-TRZ**	1:3 Bulk	521	93.6	5.12	N/A	~525	0.35, 0.58	21.7	375
**TCTA**:**PIM-TRZ**	1:2 Bulk	~530	90.9	3.67	N/A	~515	0.31, 0.56	19.1	375
**Tris-PCz**:**PIM-TRZ**	1:2 Bulk	~540	65.4	5.40	N/A	~500	0.26, 0.51	18.6	375
**TAPC**:**PIM-TRZ**	1:3 Bulk 0.6wt% **C545T**	N/A	N/A	N/A	N/A	~520	0.29, 0.62	20.2	375
**26DCzPPy**/**PO-T2T**	1:1 Bulk	~610	N/A	3.0	0.09	N/A	N/A	N/A	376
**26DCzPPy**: **Ir(dmpq)2acac** /**PO-T2T**	Interfacial 1wt% **Ir(dmpq)2acac**	N/A	N/A	N/A	N/A	~610	N/A	24.5	376
**26DCzPPy**: **Ir(dmpq)2acac** /**PO-T2T**	Interfacial 4wt% **Ir(dmpq)2acac**	N/A	N/A	N/A	N/A	~630	N/A	28.6	376
**26DCzPPy**: **Ir(dmpq)2acac** /**PO-T2T**	Interfacial 10wt% **Ir(dmpq)2acac**	N/A	N/A	N/A	N/A	~630	N/A	26.0	376
**26DCzPPy**: **Ir(dmpq)2acac**/ spacer /**PO-T2T**	Interfacial 4wt% **Ir(dmpq)2acac**	N/A	N/A	N/A	N/A	~630	N/A	18.6	376
**26DCzPPy**: **Ir(dmpq)2acac**/ **TPBi**	Interfacial 4wt% **Ir(dmpq)2acac**	N/A	N/A	N/A	N/A	~630	N/A	21.3	376
**26DCzPPy**: **Ir(dmpq)2acac**/ **BCP**	Interfacial 7wt% **Ir(dmpq)2acac**	N/A	N/A	N/A	N/A	~630	N/A	15.3	376
**DEX**:**PO-T2T**	1:1 Bulk	520	N/A	1.2	N/A	~520	0.29, 0.55	11.2	377
**TCTA**:**PO-T2T**	1:1 Bulk	520	N/A	N/A	N/A	~520	0.29, 0.55	4.0	377
**DEX**:**PO-T2T**	1:1 Bulk 1wt% **Ir(MDQ)2(acac)**	N/A	N/A	N/A	N/A	604	0.54, 0.42	19.7	377
**DEX**:**PO-T2T**	1:1 Bulk 3wt% **Ir(MDQ)2(acac)**	N/A	N/A	N/A	N/A	N/A	0.60, 0.38	22.1	377
**DEX**:**PO-T2T**	1:1 Bulk 5wt% **Ir(MDQ)2(acac)**	N/A	N/A	N/A	N/A	N/A	0.62, 0.37	21.7	377
**DEX**:**PO-T2T**	1:1 Bulk 7wt% **Ir(MDQ)2(acac)**	N/A	N/A	N/A	N/A	N/A	0.63, 0.36	20.8	377
**DEX**:**PO-T2T**/**PO-T2T**(5 nm)	1:1 Bulk 5wt% **Ir(MDQ)2(acac)**	N/A	N/A	N/A	N/A	N/A	0.62, 0.37	23.0	377
**DEX**:**PO-T2T**/**PO-T2T**(10 nm)	1:1 Bulk 5wt% **Ir(MDQ)2(a**	N/A	N/A	N/A	N/A	N/A	0.62, 0.37	23.3	377

	**cac)**								
**DEX**:**PO-T2T**/**PO-T2T**(15 nm)	1:1 Bulk 5wt% **Ir(MDQ)$_2$(acac)**	N/A	N/A	N/A	N/A	N/A	0.62, 0.37	24.5	377
**DEX**:**PO-T2T**/**PO-T2T**(20 nm)	1:1 Bulk 5wt% **Ir(MDQ)$_2$(acac)**	N/A	N/A	N/A	N/A	N/A	0.62, 0.37	23.8	377
**CDBP**:**2DBSOSPO**	1:1 Bulk	471	26	4.3	0.08	476	0.17, 0.23	0.82	378
**CDBP**:**3DBSOSPO**	1:1 Bulk	472	24	4.9	0.11	472	0.18, 0.23	0.75	378
**CDBP**:**4DBSOSPO**	1:1 Bulk	447	17	4.5	0.18	468	0.17, 0.21	0.55	378
**CDBP**:**DBSO**	1:1 Bulk	413	1	4	0.01	N/A	N/A	N/A	378
**CDBP**:**2DBSOSPO**	1:1 Bulk 3wt% **4CzTPNBu**	570	97	6.8	0.02	564	0.48, 0.49	30.3	378
**CDBP**:**3DBSOSPO**	1:1 Bulk 3wt% **4CzTPNBu**	571	91	11.1	0.01	564	0.47, 0.51	23.3	378
**CDBP**:**4DBSOSPO**	1:1 Bulk 3wt% **4CzTPNBu**	569	79	7.3	0. 03	568	0.48, 0.51	20.0	378
**CDBP**:**DBSO**	1:1 Bulk 3wt% **4CzTPNBu**	568	78	5.2	0.04	N/A	N/A	N/A	378
**CDBP**/**B4PyPPM**	Interface	N/A	19.0	0.176	N/A	N/A	N/A	N/A	379
**CDBP**/**B4PyPPM**	Interface 5wt% **Coumarin 6** In CDBP	N/A	92.2	0.011	N/A	N/A	N/A	4.0 at 100 dc/m$_2$	379
**CDBP**/**B4PyPPM**	Interface 5wt% **Ir(ppy)$_2$acac** In CDBP	N/A	93.0	1.3	N/A	N/A	N/A	7.9 at 100 dc/m$_2$	379
**CDBP**/**B4PyPPM**	Interface 5wt% **4CzIPN** In CDBP	524	98.9	6.4	N/A	536	N/A	20.5	379
**TCTA**:**PO-T2T**	8:2 Bulk	538	59.4	0.21	N/A	556	N/A	7.4	380
**TCTA**:**PO-T2T**	8:2 Bulk 1 wt% **67dTPA-FQ**	~545	27.9	0.125	N/A	552	N/A	8.4	380
**TCTA**:**PO-T2T**	8:2 Bulk 1 wt% **267tTPA-FQ**	~545	42.5	0.148	N/A	524	N/A	9.6	380
**TCTA**:**PO-T2T**	8:2 Bulk 1 wt% **DMQA**	N/A	N/A	N/A	N/A	540	N/A	4.8	380
**H2**: **B4PyMPM**	1:1 Bulk	486	37.4	0.192	0.09	N/A	N/A	N/A	381
**H2**: **B3PyMPM**	1:1 Bulk	481	47.1	0.226	0.11	N/A	N/A	N/A	381
**H2**: **tBuCzDBA**/ **B4PyMPM**	Interfacial 5wt% **tBuCzDBA**	N/A	N/A	N/A	N/A	~550	0.37, 0.57	17.5	381
**H2**: **tBuCzDBA**/ **B4PyMPM**	Interfacial 10wt% **tBuCzDBA**	N/A	N/A	N/A	N/A	~550	0.42, 0.55	20.0	381
**H2**: **tBuCzDBA**/	Interfacial 15wt%	N/A	N/A	N/A	N/A	~550	0.42, 0.56	17.4	381

**B4PyMPM**	**tBuCzDBA**								
**H2**: **tBuCzDBA/ B4PyMPM**	Interfacial 20wt% **tBuCzDBA**	N/A	N/A	N/A	N/A	~550	0.42, 0.56	16.8	381
**H2**:**tBuCzDBA/ B3PyMPM**	Interfacial 5wt% **tBuCzDBA**	N/A	N/A	N/A	N/A	~550	0.37, 0.57	22.3	381
**H2**:**tBuCzDBA/ B3PyMPM**	Interfacial 10wt% **tBuCzDBA**	N/A	N/A	N/A	N/A	~550	0.42, 0.55	26.4	381
**H2**:**tBuCzDBA/ B3PyMPM**	Interfacial 15wt% **tBuCzDBA**	N/A	N/A	N/A	N/A	~550	0.42, 0.56	23.5	381
**H2**:**tBuCzDBA/ B3PyMPM**	Interfacial 20wt% **tBuCzDBA**	N/A	N/A	N/A	N/A	~550	0.42, 0.55	21.5	381
**mCP**:**OXD-7**	1:1 Bulk 10wt% **AQ-b1**	N/A	N/A	N/A	N/A	632	0.61, 0.38	2.55	382
**mCP**:**TDP-TRZ**	1:1 Bulk 10wt% **AQ-b1**	N/A	N/A	N/A	N/A	622	0.60, 0.39	2.74	382
**mCP**:**DTDP-TRZ**	1:1 Bulk 10wt% **AQ-b1**	N/A	N/A	N/A	N/A	622	0.59, 0.39	2.48	382
**mCP**:**TDP-TRZ: OXD-7**	1:1:1 Bulk 10wt% **AQ-b1**	N/A	N/A	N/A	N/A	628	0.60, 0.39	2.44	382
**mCP**:**DTDP-TRZ:OXD-7**	1:1:1 Bulk 10wt% AQ-b1	N/A	N/A	N/A	N/A	628	0.60, 0.39	1.61	382
**TAPC**:**DCz-DBTO$_2$**	30:70 Bulk (thickness: 15 nm)	~550	N/A	N/A	N/A	~550	N/A	2.7	383
**TAPC**:**DCz-DBTO$_2$**	30:70 Bulk (thickness: 21 nm)	~550	N/A	N/A	N/A	~550	N/A	6.1	383
**TAPC**:**DCz-DBTO$_2$**	30:70 Bulk (thickness: 30 nm)	~550	N/A	N/A	N/A	~550	N/A	5.4	383
**TAPC**:**DCz-DBTO$_2$**	30:70 Bulk (thickness: 60 nm)	~550	N/A	N/A	N/A	~550	N/A	8.9	383
**BCC-36:PO-T2T**	5:1 Bulk	490	90	1.1	0.04	N/A	0.29, 0.52	20.0	384
**BCC-36:PO-T2T**	5:1 Bulk 1wt% **C545T**	N/A	N/A	N/A	N/A	N/A	0.24, 0.57	12.5	384
**BCC-36:PO-T2T**	5:1 Bulk 12.5wt% **Ir(ppy)$_2$(acac)**	N/A	N/A	N/A	N/A	N/A	0.31, 0.64	32.5	384
**BCC-36:PO-T2T**	5:1 Bulk 7.5wt% **4CzIPN**	N/A	N/A	N/A	N/A	N/A	0.26, 0.56	26.5	384
**BCC-36:3P-T2T**	5:2 Bulk	492	80	1.8	0.12	N/A	0.27, 0.51	5.4	384

Table **S9.** Photophysical properties and device performance of exciplexes applied towards fundamental studies of TADF exciplex systems, reviewed in Section **8.7**.

Compound	Medium	λ_{PL} / nm	Φ_{PL} / %	τ_d / µs	ΔE_{ST} / eV	λ_{EL} / nm	CIE_{XY}	EQE_{max} / %	Ref
**m-MTDATA:3TPYMB**	1:1 Bulk	534	N/A	N/A	N/A	~550	N/A	12.9	385
**CN-Cz2:PO-T2T**	2:1 Bulk	N/A	56	N/A	N/A	N/A	N/A	13.7	386
**CN-Cz2:PO-T2T**	1:1 Bulk	N/A	55	3.39	N/A	~490	0.20, 0.40	16.0	386
**CN-Cz2:PO-T2T**	1:2 Bulk	N/A	51	N/A	N/A	N/A	N/A	14.9	386
**m-MTDATA:3TPYMB**	1:1 Bulk	N/A	45	N/A	0.023	N/A	N/A	N/A	387
**m-MTDATA:3TPYMB**	1:1 Bulk	545	45	12	N/A	550	N/A	11.0	388
**m-MTDATA:Bphen**	1:1 Bulk	~560	N/A	0.2	N/A	~560	N/A	N/A	388
**THCA:BPhen**	1:1 Bulk	~570	N/A	3	N/A	~600	N/A	N/A	388
**Tris-PCz:3Cz-TRZ**	1:1 Bulk	511	N/A	2.8	N/A	510	0.26, 0.53	8.9	389
**Tris-PCz:BCz-TRZ**	1:1 Bulk	499	N/A	2.8	N/A	505	0.26, 0.50	11.9	389
**Tris-PCz:Cz-TRZ**	1:1 Bulk	517	N/A	3.0	N/A	515	0.29, 0.55	9.5	389
**Tris-PCz:T2T**	1:1 Bulk	518	N/A	3.5	N/A	510	0.28, 0.54	11.5	389
**Tris-PCz:SF3-TRZ**	1:1 Bulk	504	N/A	2.6	N/A	505	0.25, 0.52	8.7	389
**DA-o**	1 wt% in PMMA	498	24	3.1	0.02	N/A	N/A	N/A	390
**DA-m**	1 wt% in PMMA	481	34	3.7	0.10	N/A	N/A	N/A	390
**DA-p**	1 wt% in PMMA	484	41	6.2	0.18	N/A	N/A	N/A	390
**dPhDA-p**	1 wt% in PMMA	504	52	7.8	0.20	N/A	N/A	6.1%	390

Table **S10.** Photophysical properties and device performance of TADF exciplex WOLEDs, reviewed in Section **8.8**.

Compound	Medium	λ_{PL} / nm	Φ_{PL} / %	τ_d / µs	ΔE_{ST} / eV	λ_{EL} / nm	CIE_{XY}	EQE_{max} / %	Ref
**1:BPhen**	1:1 Bulk	475	13.64	0.12	N/A	N/A	N/A	N/A	391
**2:BPhen**	1:1 Bulk	442	12.62	0.08	N/A	N/A	N/A	N/A	391
**3:BPhen**	1:1 Bulk	483	12.64	0.13	N/A	N/A	N/A	N/A	391
**4:BPhen**	1:1 Bulk	447	15.71	0.08	N/A	N/A	N/A	N/A	391
**1:BCP**	1:1 Bulk	447	N/A	0.10	N/A	N/A	N/A	N/A	391
**2:BCP**	1:1 Bulk	427	12.68	0.05	N/A	N/A	N/A	N/A	391
**3:BCP**	1:1 Bulk	460	N/A	0.15	N/A	N/A	N/A	N/A	391
**4:BCP**	1:1 Bulk	447	23.96	0.04	N/A	N/A	N/A	N/A	391
**26DCzPPY: B4PyMPM**	1:1 Bulk	439	N/A	6.9	N/A	N/A	N/A	N/A	392
**mCP:B4PyMPM**	1:1 Bulk 15wt% FIrpic	N/A	N/A	N/A	N/A	472	0.17, 0.35	N/A	392
**26DCzPPY: B4PyMPM**	1:1 Bulk 15wt%	N/A	N/A	N/A	N/A	472	0.17, 0.36	N/A	392

	FIrpic								
**26DCzPPy**:**PO-01**/**26DCzPPY**: **B4PyMPM**:15wt%**FIrpic**	0.5wt% **PO-01**	N/A	N/A	N/A	N/A	N/A	0.36, 0.44	21.9	392
**26DCzPPy**:**PO-01**/**26DCzPPY**: **B4PyMPM**:15wt%**FIrpic**	1.0wt% **PO-01**	N/A	N/A	N/A	N/A	N/A	0.41, 0.46	24.4	392
**26DCzPPy**:**PO-01**/**26DCzPPY**: **B4PyMPM**:15wt%**FIrpic**	2.0wt% **PO-01**	N/A	N/A	N/A	N/A	N/A	0.45, 0.47	25.3	392
**26DCzPPy**:**PO-01**/**26DCzPPY**: **B4PyMPM**:15wt%**FIrpic**	4.0wt% **PO-01**	N/A	N/A	N/A	N/A	N/A	0.45, 0.48	28.5	392
**26DCzPPy**:**PO-T2T**	1:1 Bulk	477	N/A	N/A	N/A	N/A	0.17, 0.33	7.8	393
**26DCzPPy**:**PO-T2T**	1:1 Bulk 0.3wt% TBRb	N/A	N/A	N/A	N/A	N/A	N/A	6.2	393
**26DCzPPy**:**PO-T2T**	1:1 Bulk 0.5wt% TBRb	N/A	N/A	N/A	N/A	N/A	N/A	6.0	393
**26DCzPPy**:**PO-T2T**	1:1 Bulk 0.8wt% TBRb	N/A	N/A	N/A	N/A	N/A	N/A	5.8	393
**26DCzPPy**:**PO-T2T**	1:1 Bulk 1.0wt% TBRb	N/A	N/A	N/A	N/A	N/A	N/A	5.6	393
**26DCzPPy**:**PO T2T**:0.3wt%**TBRb**/**26DCzPPy**: **PO-T2T**	1:1 Bulk	N/A	N/A	N/A	N/A	N/A	0.27,0.43	7.0	393
**26DCzPPy**:**PO T2T**:0.3wt%**TBRb**/**26DCzPPy**: **PO-T2T**: 0.3wt%**TBRb**	1:1 Bulk	N/A	N/A	N/A	N/A	N/A	0.36,0.53	10.1	393
**26DCzPPy**:**PO T2T**:0.3wt%**TBRb**/**26DCzPPy**: **PO-T2T**	TCTA interlayer	N/A	N/A	N/A	N/A	N/A	N/A	8.6	393
**mCP**:**PO-T2T**	1:1 Bulk	471	17.8	7.38	N/A	476	0.18, 0.28	6.85	394
**TPD**:**PO-T2T**	1:1 Bulk	565	4.9	0.05	N/A	592	0.53, 0.46	1.47	394
**TPD**:**Bphen**	1:1 Bulk	462	6.2	0.04	N/A	480	0.20, 0.46	0.46	394
**mCP**:**PO-T2T**/**PO-T2T**:**TPD** /**Bphen**:**TPD**	1:1/1:1/1:1	N/A	N/A	N/A	N/A	N/A	0.24, 0.33	4.53	394
**mCP**:**PO-T2T**/**PO-T2T**:**TPD** /**Bphen**:**TPD**	1:1/3:1/1:1	N/A	N/A	N/A	N/A	N/A	0.24, 0.32	5.21	394
**DTS-XA**/**TCTA**	Interface	~450	N/A	N/A	N/A	433	N/A	9.1	395
**DTS-XA**/**m-MTDATA**	Interface	~525	N/A	N/A	N/A	524	N/A	8.3	395
**DTS-XA**/**TCTA** /spacer/**DTS-XA**/**m-MTDATA**	Spacer = TSPO1	N/A	N/A	N/A	N/A	N/A	0.29, 0.37	10.6	395
**mCP**:**pDPBITPO**	65:35 Bulk	415	11	5.7	0.01	N/A	N/A	N/A	396
**mCP**: **DpPBITPO**	65:35 Bulk	412	14	5.5	0.01	N/A	N/A	N/A	396

**mCP**:**pDPBITPO**	65:35 Bulk 35% **DMAC-DPS**:0.5% **4CzTPNBu**	471, 555 (30% DMAC-DPS)	92 (30% DMAC-DPS)	0.62, 1.1/0.74 (30% DMAC-DPS)	N/A	476, 564	0.43, 0.48	30.6	[396]
**mCP**:**DpPBITPO**	65:35 Bulk 35% **DMAC-DPS**:0.2% **4CzTPNBu**	N/A	N/A	N/A	N/A	476, 556	0.31, 0.35	26.5	[396]
**mCP**:**DpPBITPO**	65:35 Bulk 35% **DMAC-DPS**:0.5% **4CzTPNBu**	474, 551 (30% DMAC-DPS)	97 (30% DMAC-DPS)	0.39, 0.69/0.37 (30% DMAC-DPS)	N/A	476, 560	0.44, 0.47	32.7	[396]

Table **S11.** Photophysical properties of the Metal TADF complexes reviewed in Section **9**.

	**Solution**					**Solid State**					
**Complex**	**Solvent**	**λ_{PL} / nm**	**Φ_{PL} / %**	**τ_{PL} / ms**	**ΔE_{ST} / meV**	**Medium**	**λ_{PL} / nm**	**Φ_{PL} / %**	**τ_{PL} / ms**	**ΔE_{ST} / meV**	**Reference**
**Copper**											
**[Cu(dmp)$_2$]BF$_4$**	DCM	670	0.021	0.09	-	-	-	-	-	-	397
**[Cu(bcp)$_2$]BF$_4$**	DCM	770	0.032	0.08	-	-	-	-	-	-	398
**[Cu(dnp)$_2$]BF$_4$**	DCM	715	0.12	0.25	-	-	-	-	-	-	398
**[Cu(tmbp)$_2$]BF$_4$**	DCM	775	0.007	0.018	-	-	-	-	-	-	398
**[Cu(dpp)$_2$]BF$_4$**	DCM	710	0.11	0.25	-	-	-	-	-	-	398
**[Cu(tpp)$_2$]BF$_4$**	DCM	745	0.145	0.23	-	-	-	-	-	-	398
**[Cu(dhp)$_2$]BF$_4$**	DCM	724	0.10	0.132	-	-	-	-	-	-	399
**[Cu(dpOHp)$_2$]BF$_4$**	DCM	724	0.08	0.107	-	-	-	-	-	-	399
**[Cu(dpOAcp)$_2$]BF$_4$**	DCM	718	0.11	0.152	-	-	-	-	-	-	399
**[Cu(dbp)$_2$]BF$_4$**	DCM	718	0.12	0.224	-	-	-	-	-	-	399
**[Cu(dbpp)$_2$]BF$_4$**	DCM	745	0.04	0.130	-	-	-	-	-	-	399
**[Cu(dtbp)$_2$]BF$_4$**	DCM	599	5.6	3.26	-	-	-	-	-	-	400
**[Cu(dchtmp)$_2$]PF$_6$**	DCM	631	5.5	2.6	-	-	-	-	-	-	401
**[Cu(dipp)$_2$]BF$_4$**	DCM	679	0.4	0.342	-	-	-	-	-	-	402
**[Cu(L$_1$)$_2$]PF$_6$**	DCM	678	0.17	0.127	-	-	-	-	-	-	403
**[Cu(L$_{Cl}$)$_2$]PF$_6$**	DCM	715	0.024	0.063	-	-	-	-	-	-	404
**[Cu(L$_{Br}$)$_2$]PF$_6$**	DCM	700	0.081	0.106	-	-	-	-	-	-	404
**[Cu(L$_I$)$_2$]PF$_6$**	DCM	700	0.088	0.11	-	-	-	-	-	-	404
**[Cu(dmp)(PPh$_3$)$_2$]BF$_4$**	DCM	560	0.014	0.33	-	-	-	-	-	-	405
**[Cu(dmp)(POP)]BF$_4$**	DCM	570	15	14.3	-	powder	538	80	18	113	406,407
**[Cu(dmp)(phanephos)]BF$_4$**	DCM	558	40	10	-	powder	530	80	14	124	408
**[Cu(pypz)(POP)]BF$_4$**	DCM	590	2.1	1.6	-	powder	490	56	20.4	180	409
**[Cu(pympz)(POP)]BF$_4$**	DCM	536	45	11.9	-	powder	465	87	12.2	170	409
**[Cu(pytfmpz)(POP)]BF$_4$**	DCM	540	30	13.3	-	powder	492	75	22.8	180	409
**[Cu(dmbpy)(POP)]BF$_4$**	Ethanol	655	<1	0.02	-	powder	575	9	-	-	410
**[Cu(tmbpy)(POP)]BF$_4$**	Ethanol	575	6	2.5	-	powder	555	74	13	89	410
**[Cu(L$_1$)(POP)]BF$_4$**	-	-	-	-	-	powder	464	82	41	80	411
**[Cu(L$_2$)(POP)]BF$_4$**	-	-	-	-	-	powder	477	99	39	80	411
**[Cu(L$_3$)(POP)]BF$_4$**	-	-	-	-	-	powder	479	83	42	90	411
**[Cu(L$_4$)(POP)]BF$_4$**	-	-	-	-	-	powder	471	84	34	90	411
**[Cu(L$_5$)(POP)]BF$_4$**	-	-	-	-	-	powder	481	87	33	90	411
**[Cu(ECAF)(POP)]PF$_6$**	DCM	616	-	-	-	powder	550	22.4	5.7	80	412
**[Cu(EHCAF)(POP)]PF$_6$**	DCM	616	-	-	-	powder	549	18.5	5.7	90	412
**[Cu(PCAF)(POP)]PF$_6$**	DCM	616	-	-	-	powder	556	20.0	5.7	40	412
**[Cu(czpzpy)(PPh$_3$)$_2$]BF$_4$**	DCM	540	-	-	-	powder	495	45	134	180	413

**[Cu(czpzpy)(POP)]BF_4**	DCM	569	-	-	-	powder	518	98	23	130	[413]
**[Cu(PNNA)(POP)]BF_4**	DCM	518	16	4.6	-	powder	493	60.9	145	120	[414]
**[Cu(PNNA)(xant)]BF_4**	DCM	518	19	7.2	-	powder	475	40.7	51	120	[414]
**[Cu(m42)(POP)]BF_4**	DCM	568	19.7	9.0	-	powder	538	24	17.1	111	[415]
**[Cu(dcbq)$(PPh_3)_2$]PF_6**	DCM	704	-	-	-	powder	669	26	0.71	-	[416]
**[Cu(dcbq)(POP)]PF_6**	DCM	709	-	-	-	powder	676	35	0.58	-	[416]
**[Cu(dcbq)(xant)]PF_6**	DCM	701	-	-	-	powder	671	56	0.63	-	[416]
**S1**	DCM	610	-	-	-	10% in PMMA	577	4	1.1	210	[417]
**S2**	DCM	615	-	-	-	10% in PMMA	592	8	1.1	170	[417]
**Se1**	DCM	624	-	-	-	10% in PMMA	594	6	1.2	140	[417]
**Se2**	DCM	637	-	-	-	10% in PMMA	605	8	0.8	120	[417]
**[Cu(Ph-BenIm-methPy)(POP)]PF_6**	-	-	-	-	-	powder	493	96	63	128	[418]
**[Cu(Ph-Im-methPy)(POP)]PF_6**	-	-	-	-	-	powder	487	100	56	108	[418]
**[Cu(DMAC-PyPI)(POP)]BF_4**	DCM	514	6	-	-	10% in DPEPO	534	21.4	62	110	[419]
**[Cu(DMAC-PyPI)(xant)]BF_4**	DCM	518	9.5	-	-	10% in DPEPO	533	24.1	71	80	[419]
**[Cu(PXZ-PyPI)(POP)]BF_4**	DCM	534	5	-	-	10% in DPEPO	565	5.8	48	70	[419]
**[Cu(PXZ-PyPI)(xant)]BF_4**	DCM	537	4.2	-	-	10% in DPEPO	564	4.3	42	50	[419]
**[Cu(^{t}Bupzmpy)(POP)]BF_4**	-	-	-	-	-	powder	523	91	13.4	100	[420]
**[Cu(Phpzmpy)(POP)]BF_4**	-	-	-	-	-	powder	500	71	32.9	90	[420]
**[Cu(Adpzmpy)(POP)]BF_4**	-	-	-	--	-	powder	498	77	34.1	90	[420]
**[Cu(pz-S-pz)(POP)]PF_6**	-	-	-	-	-	powder	581	23.7	10.5	74	[421]
**[Cu(pq-S-pz)(POP)]PF_6**	-	-	-	-	-	powder	642	57.8	6.47	128	[421]
**[Cu(pz-S-CF_3pm)(POP)]PF_6**	-	-	-	--	-	powder	650	7.7	7.74	64	[421]
**Cu(POP)(pz_2BH_2)**	DCM	535	9	1.3	-	powder	436	45	20	-	[422]
**Cu(POP)(pz_4B)**	DCM	500	2	0.5	-	powder	447	90	22	-	[422]
**Cu(POP)(pz_2BPh_2)**	DCM	498	8	1.8	-	powder	464	90	23	81 [a]	[422,423]
**Cu(dppb)(Ph_2Bpz_2)**	2-MeTHF	609	0.1	0.08	-	10% in mCP	545	50	3.8	46 [a]	[423,424]
**Cu(dppb-F)(Ph_2Bpz_2)**	2-MeTHF	614	0.5	0.13	-	10% in mCP	534	63	3.6	-	[424]
**Cu(dppb-CF_3)(Ph_2Bpz_2)**	2-MeTHF	616	2	0.35	-	10% in mCP	523	68	8.2	-	[424]
**Cu(PP)(PS)**	-	-	-	-	-	powder	521	52	1.73	-	[425]
**TTPPCuCl**	-	-	-	-	-	Neat Film	530	76	19	-	[426]
**TTPPCuBr**	-	-	-	-	-	Neat Film	523	79	16	-	[426]
**TTPPCuI**	-	-	-	-	-	Neat Film	521	83	11	-	[426]

CuCl(PPh_3)(dpmb)	-	-	-	-	-	powder	479	53	5.1	-	427
CuBr(PPh_3)(dpmb)	-	-	-	-	-	powder	465	28	5.7	-	427
CuI(PPh_3)(dpmb)	-	-	-	-	-	powder	464	23	4.3	-	427
CuI(PPh_3)(dpmt)	-	-	-	-	-	powder	459	24	361	-	428
CuBr(PPh_3)(dpmt)	-	-	-	-	-	powder	465	4	209	-	428
[CuCl(PPh_3)(dpmt)]	-	-	-	-	-	powder	484	4	106	-	428
Cu(NCS)(P^3)	-	-	-	-	-	powder	520	57	4.8	62	429
Cu(NCS)(P^4)	-	-	-	-	-	powder	543	27	4.9	80	429
[CuI(dpts)(PPh_3)]	-	-	-	-	-	powder	485	41	36.4	59	430
[CuBr(dpts)(PPh_3)]	-	-	-	-	-	powder	506	52	48.9	127	430
[CuCl(dpts)(PPh_3)]	-	-	-	-	-	powder	535	29	20.8	128	430
[CuI(dppt)(PPh_3)]	-	-	-	-	-	powder	515	18	9.0	57	430
[CuBr(dppt)(PPh_3)]	-	-	-	-	-	powder	535	7	10.0	104	430
[CuCl(dppt)(PPh_3)]	-	-	-	-	-	powder	516	3	4.2	105	430
[Cu(dmpzpp)Br]	-	-	-	-	-	powder	541	83	9	-	431
[Cu(dmpzpp)I]	-	-	-	-	-	powder	530	82	7	-	431
[Cu(dmpzpp)SPh]	DCM	544	1	4	-	powder	540	90	5	74	431
[Cu(3,4-dppTp)(pz_2-BH_2)] 1B	-	-	-	-	-	Neat Film	472	59	22	-	431
[Cu(3,4-dppTp)(pz_2-BH_2)] 1Y	-	-	-	-	-	Neat Film	528	78	19	-	432
[Cu(3,4-dppTp)(pz_2-BH_2)] 1G	-	-	-	-	-	Neat Film	550	34	15	-	432
[Cu(dppnc)(thzbzi)]	-	-	-	-	-	Solid state	547	16	25.8	114	433
Cu(PPh_3)$_2$(PyrTet)	-	-	-	-	-	Solid state	512	85	20.6	-	434
Cu(DPEPhos)(PyrTet)	-	-	-	-	-	Solid state	510	78	19.9	-	434
Cu(Xantphos)(PyrTet)	-	-	-	-	-	Solid state	545	76	26.6	-	434
Cu(PTEPhos)(PyrTet)	-	-	-	-	-	Solid state	502	89	17.8	-	434
[Cu(PPh_3)$_2$(PyrTetH)]BF_4	-	-	-	-	-	Solid state	522	46	13.5	-	434
[Cu(DPEPhos)(PyrTetH)]BF_4	-	-	-	-	-	Solid state	518	21	15.3	-	434
[Cu(Xantphos)(PyrTetH)]BF_4	-	-	-	-	-	Solid state	559	6	5.9	-	434
[Cu(Xantphos)(PyrTetH)]BF_4	-	-	-	-	-	Solid state	569	4	5.2	-	434
Cu(PPh_3)$_2$(fptz)	DCM	529	2.7	6.9	-	Solid state	496	53.3	30.3	-	435
[Cu(PPh_3)$_2$(fptzH)]ClO_4	DCM	547	4.8	7.9	-	Solid state	472	67.1	20.2	-	435
(L_{Me})CuCl	DCM	534	43	4.9	-	10% in mCP	520	68	6.1	-	436
(L_{Me})CuBr	DCM	527	47	5.4	-	10% in mCP	514	71	5.5	-	436
(L_{Me})CuI	DCM	517	60	6.5	-	10% in mCP	504	57	3.2	-	436
(L_{Et})CuBr	DCM	516	43	3.8	-	powder	487	80	6.5	74	437
(L_{iPr})CuBr	DCM	517	50	8.3	-	powder	486	95	8.9	88	437
Cu(L_{Me})(SPh)	2-MeTHF	592	24	1.4	-	powder	488	95	6.6	85	438
Cu(LiPr)(SPh)	2-MeTHF	546	15	1.0	-	powder	500	95	5.0	78	438
CuI(mpdp)	-	-	-	-	-	powder	493	5.4	7.2	69.4	439
CuBr(mpdp)	-	-	-	-	-	powder	527	<1	16.6	43.8	439

**CuCl(mpdp)**	-	-	-	-	-	powder	533	1.1	415.9	51.3	439
**CuI(benzimPP)**	-	-	-	-	-	powder	630	65	64.4	-	440
**CuBr(benzimPP)**	-	-	-	-	-	powder	615	72	61.6	-	440
**CuI(dtbp)**	-	-	-	-	-	powder	624	15	3.7	-	441
**CuBr(dtbp)**	-	-	-	-	-	powder	658	2	1.1	-	441
**CuCl(dtbp)**	-	-	-	-	-	powder	677	<1	0.9	-	441
**(IPr)Cu(py2-BMe2)**	-	-	-	-	-	powder	475	76	11	88	442
**[Cu(IPr)(L_1)]PF_6**	-	-	-	-	-	powder	463	22	13	-	443
**[Cu(^{Me}IPr)(L_1)]PF_6**	-	-	-	-	-	powder	475	48	24	-	443
**[Cu(IPr^{MeO})(L_1)]PF_6**	-	-	-	-	-	powder	481	31	29	-	443
**[Cu(^{Me}IPr^{MeO})(L_1)]PF_6**	-	-	-	-	-	powder	479	64	30	-	443
**[Cu(^{Cl}IPr)(L_1)]PF_6**	-	-	-	-	-	powder	467	17	10	-	443
**[Cu(IPr)(L_2)]PF_6**	-	-	-	-	-	powder	505	55	32	-	443
**[Cu(IPr)(L_3)]PF_6**	-	-	-	-	-	powder	550	<5	5	-	443
**[Cu(IPr)(L_4)]PF_6**	-	-	-	-	-	powder	420	<5	20	-	443
**[Cu(IPr)(L_5)]PF_6**	-	-	-	-	-	powder	521	20	7	-	443
**[Cu(IPr)(L_6)]PF_6**	-	-	-	-	-	powder	473	36	25	-	443
**[Cu(IPr)(dpya)]PF_6**	-	-	-	-	-	powder	481	22	13	-	444
**[Cu(IPr)(dpym)]PF_6**	-	-	-	-	-	powder	483	15	6	-	444
**[Cu(IPr)(dpyp)]PF_6**	-	-	-	-	-	powder	482	73	14	-	444
**[Cu(IPr)($PPhPy_2$)]PF_6**	-	-	-	-	-	powder	519	86	13	-	444
**CuO1**	-	-	-	-	-	powder	467	57	0.47	-	445
**CuO2**	-	-	-	-	-	powder	460	22	0.58	-	445
**CuO4**	-	-	-	-	-	powder	466	57	0.66	-	445
**CuO5**	-	-	-	-	-	powder	467	38	0.45	-	445
**CuN1**	-	-	-	-	-	powder	500	11	0.45	-	445
**CuN2**	-	-	-	-	-	powder	513	4.5	0.28	-	445
**$^{(Ad)}$LCuSPh**	-	-	-	-	-	powder	484	62	0.50	-	445
**[Cu(ITr)(4-CO-py)]BF_4**	DCM	600	-	-	-	10 wt% in PMMA	545	25	4.0	-	446
**[Cu(ITr)(4-CN-py)]BF_4**	DCM	665	-	-	-	10 wt% in PMMA	531	12	5.7	-	446
**CuI:CzBPDCb**	-	-	-	-	-	8 wt% CuI in CzBPDCb	520	22	1.05	120	447
**CuCl:azaSPF**	-	-	-	-	-	7 wt% CuCl in azaSPF	553	48.8	5.3	-	448
**CuBr:azaSPF**	-	-	-	-	-	7 wt% CuBr in azaSPF	537	77.9	5.5	-	448
**CuI:azaSPF**	-	-	-	-	-	9 wt% CuI in azaSPF	526	63.7	5.3	-	448
**CuI:α-aza-SBF**	DMF	517 ^{c}	-	55.8 ^{c}	-	5 wt% CuI in	548	4.6	5.5	-	449

						α-aza-SBF					
**CuI:β-aza-SBF**	DMF	625 [c]	-	18.1[c]	-	5 wt% CuI in β-aza-SBF	580	52.4	2.2	-	[449]
**CuI:γ-aza-SBF**	DMF	580 [c]	-	21.1[c]	-	5 wt% CuI in γ-aza-SBF	538	60.4	2.9	-	[449]
**CuI:δ-aza-SBF**	DMF	550 [c]	-	40.6 [c]	-	5 wt% CuI in δ-aza-SBF	550	92.2	4.2	-	[449]
**[Cu(mI)dppb]$_2$**	-	-	-	-	-	powder	495	-	10.4	-	[450]
**{Cu(PNP-^{t}Bu)}$_2$**	2-MeTHF	-	57	11.5	100	1 wt% TAPC	524	-	-	-	[451]
**1-Cu**	-	-	-	-	-	neat film	540	83	3.6 [a]	-	[452]
**Cu$_2$Cl$_2$(N^P)$_2$**	-	-	-	-	-	powder	485	92	8.3	124	[453]
**Cu$_2$Br$_2$(N^P)$_2$**	-	-	-	-	-	powder	501	52	12.4	118	[453]
**Cu$_2$I$_2$(N^P)$_2$**	-	-	-	-	-	powder	484	76	7.3	136	[453]
**[Cu(μ-Cl)dpmb]$_2$**	-	-	-	-	-	powder	498	32	2.5	120	[454]
**[Cu(μ-Br)dpmb]$_2$**	-	-	-	-	-	powder	511	28	12.5	120	[454]
**[Cu(μ-I)dpmb]$_2$**	-	-	-	-	-	powder	527	29	4.8	140	[454]
**[Cu$_2$(μ-I)$_2$(dmphen)$_2$]**	-	-	-	-	-	powder	667	18	6.4	-	[441]
**[Cu$_2$Cl$_2$(IMesPic^{Cl})$_2$]**	-	-	-	-	-	powder	550	49	11.0	92	[455]
**[Cu$_2$Cl$_2$(IMesPic)$_2$]**	-	-	-	-	-	powder	520	59	11.0	88	[455]
**[Cu$_2$Cl$_2$(IMesPic^{Me})$_2$]**	-	-	-	-	-	powder	523	68	9.2	79	[455]
**[Cu$_2$I$_2$(MePyrPHOS)(P(mTol)$_3$]**	toluene	593	-	-	-	powder	550	75	6.9	-	[456]
**[Cu$_2$(pytzph)(POP)$_2$](BF$_4$)$_2$**	-	-	-	-	-	crystalline	509	43	5.5	89	[457]
**[Cu$_2$(pytzphcf)(POP)$_2$](BF$_4$)$_2$**	-	-	-	-	-	crystalline	519	29	16	139	[457]
**[Cu$_2$(pytzphcz)(POP)$_2$](BF$_4$)$_2$**	-	-	-	-	-	crystalline	503	79	5.5	94	[457]
**(PNNP)Cu$_2$Br$_2$**	-	-	-	-	-	powder	517	42	13	90	[458]
**(PNNP)Cu$_2$I$_2$**	-	-	-	-	-	powder	494	58	8.8	100	[458]
**[(P^N)Cu(m-I)]$_2$**	-	-	-	-	-	powder	585	36.9	5.85	20	[459]
**Cu-1b**	-	-	-	-	-	powder	524	93	5.8	-	[460]
**Cu-1c**	-	-	-	-	-	powder	541	70	5.5	-	[460]
**Cu-1d**	-	-	-	-	-	powder	528	80	10.2	-	[460]
**Cu-2b**	-	-	-	-	-	powder	519	89	5.5	-	[460]
**Cu-2c**	-	-	-	-	-	powder	524	90	5.5	-	[460]
**Cu-2d**	-	-	-	-	-	powder	524	76	6.8	-	[460]
**Cu-3a**	-	-	-	-	-	powder	549	73	5.1	-	[460]
**Cu-3b**	-	-	-	-	-	powder	539	73	7.3	-	[460]
**Cu-4a**	-	-	-	-	-	powder	547	79	5.5	-	[460]
**Cu-4b**	-	-	-	-	-	powder	519	88	6.3	-	[460]
**Cu$_2$(Py$_3$P)$_2$Cl$_2$**	-	-	-	-	-	crystalline	550	55	14.5	186	[461]
**Cu$_2$(Py$_3$P)$_2$Br$_2$**	-	-	-	-	-	crystalline	530	53	18.3	155	[461]
**Cu$_2$(Py$_3$P)$_2$I$_2$**	-	-	-	-	-	crystalline	520	51	20.0	124	[461]
**Cu$_2$(Py$_2$AsPh)$_2$Cl$_2$**	-	-	-	-	-	powder	530	50	9	167	[462]

**$Cu_2(Py_2AsPh)_2Br_2$**	-	-	-	-	-	powder	510	20	3.5	157	[462]
**$Cu_2(Py_2AsPh)_2I_2$**	-	-	-	-	-	powder	500	22	2	136	[462]
**$[Cu_2(Py_2AsPh)_2(MeCN)_2](BF_4)_2$**	-	-	-	-	-	powder	465	2	4	-	[462]
**$(DBFDP)_2Cu_4I_4$**	DCM	496	5.7	-	-	neat film	494	5.1	1.96	160	[463]
**$(DCzDBFDP)_2Cu_4I_4$**	DCM	514	53	-	-	neat film	491	46	1.05	70	[464]
**$(DtBDCzDBFDP)_2Cu_4I_4$**	DCM	510	70	-	-	neat film	480	65	10.2	100	[464]
**$Cu_4Br_4(dppMeCz)_2$ (1Y)**	-	-	-	-	-	powder	549	3.7	3.6	-	[465]
**$[(3,5\text{-}(CF_3)_2Pz)Cu]_3(dppm)$**	DCE	532	-	1.6	-	powder	514	-	32.7	-	[466]
**$[Cu_4(PCP)_3]BF_4$**	THF	525	79	11		powder	518	50	9.8	72	[467]
**CuL^3 $[Cu_4I_4(dppp)_2(3\text{-}acepy)_2]$**	-	-	-	-	-	powder	562	26.3	2.52	36	[468]
**CuL^3 $[Cu_4I_4(dppp)_2(4\text{-}acepy)_2]$**	-	-	-	-	-	powder	580	30.6	10.78	35	[468]
**Silver**											
**Ag(dppb)(PS)**	-	-	-	-	-	neat film	505	32	0.6, 2.2	20	[425]
**Ag(phen)(P2-nCB)**	DCM	-	< 1	-	-	1% in PMMA	577	26	2.0 [a]	-	[51]
**Ag(idmp)(P2-nCB)**	DCM	-	< 1	-	-	1% in PMMA	565	38	1.7 [a]	-	[51]
**Ag(dmp)(P2-nCB)**	DCM	-	-	-	-	1% in PMMA	540	75	2.8 [a]	80 [a]	[51]
**Ag(dbp)(P2-nCB)**	DCM	585	3	-	-	1% in PMMA	535	85	1.4 [a]	80 [a]	[50]
**[Ag(xant)(4,4'-MeO-bpy)]BF4**	DCM	493	57	-	-	-	-	-	-	-	[469]
**$Ag(P_3)(SCN)$**	-	-	-	-	-	neat film	538	32	5.5, 16.2	-	[429]
**$Ag(P_4)(SCN)$**	-	-	-	-	-	neat film	552	27	1.3, 5.4	-	[429]
**$Ag(P_3)(SCN\text{-} B(C_6F_5)_3)$**	-	-	-	-	-	neat film	475	11	1.3, 4.7	-	[429]
**$Ag(P_4)(SCN\text{-} B(C_6F_5)_3)$**	-	-	-	-	-	neat film	482	12	24.1, 10.1	-	[429]
**$(L_{Me})AgBr$**	DCM	499	20	-	-	powder	487	0.56	-	105	[470]
**$(L_{Et})AgBr$**	DCM	499	25	-	-	powder	463	0.70	-	110	[470]
**$(L_{iPr})AgBr$**	DCM	492	32	-	-	powder	463	0.98	-	99	[470]
**Ag(DMAC-MPyPz)(POP)**	DCM	502	61	4.23	-	15 wt% PMMA	472	95	6.3	170	[471]
**Ag(DMAC-MPyPz)(xant)**	DCM	500	60	4.24	-	15 wt% PMMA	471	99	6.5	150	[471]
**Ag(PI-DMAC)(POP)**	DCM	502	-	-	-	10 wt% DPEPO	520	48 [b]	4.7	210	[472]
**Ag(PI-DMAC)(xant)**	DCM	506	-	-	-	10 wt% DPEPO	524	34 [b]	7.4	210	[472]
**Ag(PI-PXZ)(POP)**	DCM	533	-	-	-	10 wt% DPEPO	573	62 [b]	3.5	160	[472]
**Ag(PI-PXZ)(xant)**	DCM	533	-	-	-	10 wt% DPEPO	535	50 [b]	3.7	220	[472]

**$P(Ph)_2Me$-Ag-TCzBN-PyPz**	-	-	-	-	-	powder	522	28.5	0.59	30	473
**[Ag(spiro-2N)(POP)]BF_4**	-	-	-	-	-	10 wt% PMMA	486	65	5.3	90	474
**[Ag(spiro-2N)(xanthene)]BF_4**	-	-	-	-	-	10 wt% PMMA	495	74	5.8	50	474
**Ag(^{Ad}CAAC)Cl**	-	-	-	-	-	powder	432	4.5	15.0	-	475
**Ag(^{Ad}CAAC)Br**	-	-	-	-	-	powder	437	5	8.1	-	475
**Ag(^{Ad}CAAC)I**	-	-	-	-	-	powder	443	0.5	6.7	-	475
**Ag(Et_2CAAC)(maloNHC)**	Toluene	459	-	-	-	5 wt% PS	440	0.3	11.6	-	476
**[Ag(PPh_3)(Cl)]$_2$(tpbz)**	-	-	-	-	-	5 wt% PMMA	531	34	5.4	-	477
**[Ag(PPh_3)(Br)]$_2$(tpbz)**	-	-	-	-	-	5 wt% PMMA	519	40	4.5	-	477
**[Ag(PPh_3)(I)]$_2$(tpbz)**	-	-	-	-	-	5 wt% PMMA	517	16	4.0	-	477
**Ag_2(tpbz)(P_2-*n*CB)$_2$**	-	-	-	-	-	powder	555	70	1.9	59	478
**[Ag(^{Et2}CAAC)Cl]$_2$**	-	-	-	-	-	powder	454	5	18.9	-	475
**[Ag(μ_2-κ^2-SCN)(dppb)]$_2$**	-	-	-	-	-	powder	505	35	12.0	90	429
**[Ag_2(Py_3P)$_3$(SCN)$_2$]**	Chloroform	478	70	9	-	powder	469	16	2.2	-	479
**[Ag_2(Py_3P)$_3$(SCN)$_2$]**	DCM	483	71	12	-	-	-	-	-	-	479
**[Ag_2(μ_3,κ^2-SCN)(P_4)]$_2$(CF_3SO_3)$_2$**	-	-	-	-	-	powder	468	43	4.7	113	429
**[Ag_2(μ_3,κ^2-SCN)(t-SCN)(P_4)]$_2$**	-	-	-	-	-	powder	475	11	18.3	-	429
**[Ag(dppb)(Cl)]$_2$**	-	-	-	-	-	5 wt% PMMA	515	47	35	-	480
**[Ag(dppb)(NO_3)]$_2$**	-	-	-	-	-	5 wt% PMMA	482	24	21	-	480
**[Ag(dppb)(ClO_4)]$_2$**	-	-	-	-	-	5 wt% PMMA	492	44	63	-	480
**[Ag(dppb)(CF_3COO)]$_2$**	-	-	-	-	-	5 wt% PMMA	496	53	61	-	480
**[Ag(dppb)(CF_3SO_3)]$_2$**	-	-	-	-	-	5 wt% PMMA	500	28	41	-	480
**[Ag_4(μ-DMPTP)$_2$(POP)$_3$][BF_4]$_2$**	-	-	-	-	-	powder	527	76	0.65	80	61
**Ag_6L_6/D_6**	-	-	-	-	-	powder	556	56	18.6	96	481
**Ag_6PL_6/PD_6**	-	-	-	-	-	powder	575	95	16.3	41	481
**Gold**											
**Au(dppb)(PS)**	-	-	-	-	-	Neat film	610	12	1.66	-	425
**(LiPr)AuCl**	2-MeTHF	607	2		-	Neat film	558	82	13	118	482
**(LiPr)AuI**	2-MeTHF	596	4		-	Neat film	549	92	9	108	482
**[(dppb)$_2$Au](NO_3)**	-	-	-	-	-	Neat film	485	95	3.8	77	482
**[AuC_6F_5(dppBz)]**	-	-	-	-	-	Neat film	560	29	10.3	58	483

**[AuC$_6$m-Cl$_2$F$_3$(dppBz)]**	-	-	-	-	-	Neat film	545	16	20.8	100	483
**[AuC$_6$Cl$_5$(dppBz)]**	-	-	-	-	-	Neat film	555	11	19.9	114	483
**[Au$_O$-C$_6$BrF$_4$(dppBz)]**	-	-	-	-	-	Neat film	566	13	22.6	89	484
**[Au$_p$-C$_6$BrF$_4$(dppBz)]**	-	-	-	-	-	Neat film	590	37	9.9	-	484
**[Au$_O$-C$_6$F$_4$I(dppBz)]**	-	-	-	-	-	Neat film	616	4	9.6	-	484
**[Au$_p$-C$_6$F$_4$I(dppBz)]**	-	-	-	-	-	Neat film	595	8	9.5	-	484
**Au(*malo*NHC)(^{Et2}CAAC)**	Toluene	453	-	-	-	5 wt% PS	464	2.9	22	-	476
**AuDAC^{NPh3}**	2-MeTHF	680	0.2	0.004	-	1 wt% PS	620	38	0.65	-	485
**AuMAC^{NPh3}**	2-MeTHF	522	12	0.210	-	1 wt% PS	462	19	2.3	-	485
**AuMAC^{NMe2}**	2-MeTHF	583	3.5	0.083	-	1 wt% PS	504	28	1.3	-	485
**AuMAC^{Xy}**	2-MeTHF	594	5.5	0.310	-	1 wt% PS	520	77	4.1	-	485
**AuMAC^{Jul}**	2-MeTHF	604	1.3	0.015	-	1 wt% PS	544	61	2.5	-	485
**AuMAC^{MeJul}**	2-MeTHF	620	2.1	0.128	-	1 wt% PS	554	61	3.0	-	485
**Au-CC-1**	Toluene	435	-	0.21	330	5 wt% PMMA	427	36	65	-	486
**Au-CC-2**	Toluene	540	-	0.64	110	5 wt% PMMA	515	76	127.3	144	486
**Au-CC-3**	Toluene	536	-	0.51	0	5 wt% PMMA	520	60	53.7	82	486
**Au-CC-4**	Toluene	565	-	0.89	160	5 wt% PMMA	545	55	224.6	162	486
**(SIPr)AuBN**	THF	511	88	7.9	-	1 wt% PMMA	505	99	114	-	487
**(BzIPr)AuBN**	THF	514	86	6.9	-	2 wt% PMMA	511	90	5.8	-	487
**(PyIPr)AuBN**	THF	511	93	7.3	-	2 wt% PMMA	513	91	5.5	-	487
**(PzIPr)AuBN**	THF	510	78	6.5	-	2 wt% PMMA	514	88	5.9	-	487
**(IPr)AuBN**	THF	511	83	7.2	-	2 wt% PMMA	515	92	5.5	-	487
**(BzIPr)AuBNO**	THF	471	89	27	-	2 wt% PMMA	457	97	600	-	487
**IPzIDCz**	2-MeTHF	587	-	-	-	10 wt% DPEPO	525	66	2.9		488
**ImIDCz**	2-MeTHF	475	-	-	-	10 wt% DPEPO	478	51	704		488
**IPzTPA**	2-MeTHF	612	-	-	-	10 wt% DPEPO	529	66	1.2		488
**(C^Npz^C)AuCl**	DCM	-	-	-	-	Neat film	560 [a]	-	-	-	489
**(C^Npz^C)AuCN**	DCM	536	0.184	0.00596	-	Neat film	535	2.9	-	-	489
**(C^Npz^C)Au(PyZ)**	DCM	572	0.008	0.0154	-	Neat film	531	-	-	-	489

**(C^Npz^C)AuC≡CPh**	DCM	526	0.462	0.161	-	Neat film	523	4.5	-	-	[489]
**(C^Npz^C)AuC≡*t*Bu**	DCM	525	0.512	0.13	-	Neat film	523	8.3	-	-	[489]
**Me(C^Npz^C)AuClBF$_4^-$**	DCM	460	3.75	0.00346	-	Neat film	623	-	-	-	[489]
**(C^N^C)AuPh**	Toluene	503	14	61.5	-	4 wt% PMMA	504	14	51	-	[490]
**(C^N^C)Au(PhN(Ph)$_2$)**	Toluene	534	74	1.03	-	4 wt% PMMA	523	66	1.35	-	[490]
**(C^N^C)AuPhNMe2**	Toluene	596	18	0.32	-	4 wt% PMMA	564	42	0.74	-	[490]
**(C^N^C)AuPhCz**	Toluene	503	13	43.5	-	4 wt% PMMA	504	13	34	-	[490]
**(F$_2$C^NOEt^CF$_2$)Au(PhN(Ph)$_2$)**	Toluene	524	79	0.71	-	4 wt% PMMA	517	84	0.72	-	[490]
**(CF$_2$^NOEt^CF$_2$)AuPhN(p-FC$_6$H$_4$)$_2$**	Toluene	511	68	0.75	-	4 wt% PMMA	513	78	0.85	-	[490]
**(C^Niq^C)AuPhN(C$_6$H$_5$)$_2$**	Toluene	641	6.5	29.4	-	4 wt% PMMA	642	3.1	16.5	-	[490]
**(CtBu^N$_2$MePh^CtBu)AuPhtBuDPA**	Toluene	527	75	1.10	-	4 wt% PMMA	521	67	1.10	-	[490]
**(CF$_2^N^{Nme2}$^CF$_2$)Au(PhN(Ph)$_2$)**	Toluene	495	81	0.68	-	4 wt% PMMA	484	82	0.97	-	[491]
**(CF$_2^N^{Nme2}$^CF$_2$)Au(PhN(p-FC$_6$H$_4$)$_2$)**	Toluene	483	60	0.67	-	4 wt% PMMA	470	34	0.95	-	[491]
**(CF$_2$^N^CF$_2$)Au(PhN(Ph)$_2$)**	Toluene	566	93	0.84	-	4 wt% PMMA	550	81	0.69	-	[491]
**(CF$_2$^N^CF$_2$)Au(C≡CPh)**	Toluene	466	0.2	0.34	-	4 wt% PMMA	468	2.8	22.4	-	[492]
**(CF$_2$^N^CF$_2$)Au(C≡CPh-*p*NPh$_2$)**	Toluene	574	60	0.78	-	4 wt% PMMA	577	88	0.85	-	[492]
**(CF$_2$^N^CF$_2$)Au(C≡CPh-*m*NPh$_2$)**	Toluene	545	21	1.25	-	4 wt% PMMA	546	29	3.78	-	[492]
**(CF$_2$^N^CF$_2$)Au(C≡CPh-*p*DMAC)**	Toluene	562	49	0.80	-	4 wt% PMMA	560	67	1.43	-	[492]
**(CF$_2$^N^CF$_2$)Au(C≡C-Ph-PXZ)**	Toluene	603	57	0.84	-	4 wt% TCTA :TPBi	567	65	1.46	-	[492]
**(CF$_2$^N^CF$_2$)Au(C≡C-Me$_2$Ph-*p*NPh$_2$)**	Toluene	594	25	0.33	-	4 wt% TCTA :TPBi	568	80	1.19	-	[492]
**(C^{tBu}^N$_{pz}^{C}^{tBu}$)Au(C≡C-Me$_2$Ph-*p*NPh$_2$)**	Toluene	632	2	0.20	-	4 wt% MCP	595	56	0.77	-	[492]
**(C^{tBu}^N$_{pz}^{C}^{tBu}$)Au(C≡C-Ph-PXZ)**	Toluene	625	8	0.25	-	4 wt% PMMA	605	9	0.33	-	[492]
**[Au{^{t}BuC^C(4-^{t}BuC$_6$H$_4$)^N(1-**	Toluene	647	20	0.3	-	5 wt% mCP	554	83	3.3	-	[493]

**thpy)}(Cbz)]**											
**[Au{^{t}BuC^C(4-^{t}BuC$_6$H$_4$)^N(1-thpy)}(^{t}Bu$_2$Cbz)]**	Toluene	690	4	0.1	-	5 wt% mCP	592	60	1.4	-	493
**[Au{^{t}BuC^C(4-^{t}BuC$_6$H$_4$)^N(1-thq)}(Cbz)]**	Toluene	727	2	0.1	-	5 wt% mCP	604	57	4.5	-	493
**[Au{^{t}BuC^C(4-^{t}BuC$_6$H$_4$)^N(2-thpy)}(Cbz)]**	Toluene	635	22	0.3	-	5 wt% mCP	557	81	8.1	-	493
**[Au{^{t}BuC^C(4-^{t}BuC$_6$H$_4$)^N(2-thpy)}(^{t}Bu$_2$Cbz)]**	Toluene	671	6	0.1	-	5 wt% mCP	580	71	1.8	-	493
**[Au{^{t}BuC^C(4-^{t}BuC$_6$H$_4$)^N(2-thq)}(Cbz)]**	Toluene	710	3	0.1	-	10 wt% mCP	600	57	3.9	-	493
**[Au{ ^{t}BuC^C(4-^{t}BuC$_6$H$_4$)^N}(Cbz)]**	Toluene	607	4	0.5	3	20 wt% mCP	547	82	3.5	-	494
**[Au{^{t}BuC^C(4-^{t}BuC$_6$H$_4$)^N}{(Cbz)-(Cbz)$_2$}]**	Toluene	624	3.6	0.2	-	5 wt% mCP	532	74	1.2	-	494
**[Au{ ^{t}BuC^C(4-^{t}BuC$_6$H$_4$)^N}{(Cbz)-(Cbz)$_2$-(Cbz)$_4$}]**	Toluene	584	9.1	0.5	-	20 wt% mCP	535	75	1.4	-	494
**[Au{4-^{t}BuC^C(4-^{t}BuC$_6$H$_4$)^N}(3-CN-Cbz)]**	Toluene	540	15	2.0	-	15 wt% mCP	497, 526	41	67	-	495
**[Au{^{t}BuC^C(4-^{t}BuC$_6$H$_4$)^N}(3-(P(O)Ph$_2$)-Cbz)]**	Toluene	570	14	0.6	-	10 wt% mCP	497, 523	63	64.6	-	495
**[Au{^{t}BuC^C(4-^{t}BuC$_6$H$_4$)^N}(3-(C$_6$H$_4$-NPh$_2$)-Cbz)]**	Toluene	659	0.3	0.04	-	5 wt% mCP	557	44	1.7	-	495
**[Au{^{t}BuC^C(4-^{t}BuC$_6$H$_4$)^N}(3-(MeC$_6$H$_3$-NPh$_2$)-Cbz)]**	Toluene	637	1	0.1	-	10 wt% mCP	554	60	2.0	-	495
**[Au{^{t}BuC^C(4-^{t}BuC$_6$H$_4$)^N}(2-(C$_6$H$_4$-NPh$_2$)-Cbz)]**	Toluene	600	5	0.5	-	15 wt% mCP	539	68	7.2	-	495
**[Au{^{t}BuC^C(4-^{t}BuC$_6$H$_4$)^N}(2-(MeC$_6$H$_3$-NPh$_2$)-Cbz)]**	Toluene	599	7	0.5	-	10 wt% mCP	535	79	5.9	-	495
**Au(C^C^{Oph}^NphOMe^C^{t}Bu)**	Toluene	495, 526, 565	54	93.1	-	4 wt% PMMA	492, 523, 560	20	43.8	-	496
**Au(C^C^{Oph}^NPh^{t}Bu$_2$^CBr)**	Toluene	498, 526, 527	40	77.1	-	4 wt% PMMA	490, 522, 562	4	90.1	-	496
**Au(C^C^{Oph}^NPh^{t}Bu$_2$^CDPA)**	Toluene	524, 550	77	94.3	-	4 wt% PMMA	550	47	56.8	-	496
**Au(C^C^{Oph}^NPh^{t}Bu$_2$^CPXZ)**	Toluene	612	47	0.62	-	16 wt% TCTA	568	89	1.69	-	496
**Au(C^C^{Me}^N^C)**	Toluene	493, 521	28	225	-	4 wt% PMMA	489, 519, 555	6	147	-	496
**Au(CBr^C^{Me}^N^C)**	Toluene	485, 518,	26	152	-	4 wt% PMMA	486, 518, 555	6	90.4	-	496

		552									
**Au(CDPA^C^{Me}^N^C)**	Toluene	533	94	1.61	-	8wt% TCTA	520	82	2.08	-	496
**Au(CPXZ^C^{Me}^N^C)**	Toluene	580	74	0.79	-	4wt% TCTA	568	71	2.54	-	496
**[Au{C^CPh^(N^C)fluorene)}]**	Toluene	480, 513, 557	0.3	1.04	-	4 wt% PMMA	482, 515, 552	6	36.01	-	496
**Au-1**	Toluene	547	84	0.56	-	4 wt% DPEPO/TCTA	530	98	0.56	-	497
**Au-2**	Toluene	537	85	0.47	-	4 wt% DPEPO/TCTA	521	93	0.51	-	497
**Au-3**	Toluene	525	83	1.53	-	4 wt% PMMA	513	29	79.2	-	497
**Au-4**	Toluene	532	88	0.69	-	4 wt% DPEPO/TCTA	528	99	0.54	-	497
**CMA**											
**Ag-1**	toluene	521	74	0.46	-	neat film	496	19	0.38	80	498
**Ag-2**	toluene	546	55	0.305	-	neat film	514	45	0.42	60	498
**CAAC-1a**	2-MeTHF	492	100	2.5	-	1% in PS	474	100	2.8	-	499
**CAAC-1b**	2-MeTHF	510	68	2.3	-	-	-	-	-	-	499
**CAAC-1c**	2-MeTHF	500	56	1.8	-	-	-	-	-	-	499
**CAAC-1d**	2-MeTHF	510	11	0.54	-	-	-	-	-	-	499
**CAAC-2b**	2-MeTHF	542	12	1.46	-	-	-	-	-	-	499
**CAAC-3**	2-MeTHF	428	11	8.3	-	1% in PS	426	82	980	-	499
**CAAC-4**	2-MeTHF	558	25	0.28	-	1% in PS	518	100	2.3	-	499
**CAAC-5**	2-MeTHF	580	16	0.87	-	1% in PS	532	78	2.6	-	499
**MAC*-1**	2-MeTHF	448	24	2.3	-	1% in PS	432	80	12.4	-	500
**MAC*-2**	2-MeTHF	492	100	1.2	-	1% in PS	468	100	1.3	-	500
**MAC*-3**	2-MeTHF	542	55	1.1	-	1% in PS	506	90	1.4	-	500
**DAC*-4**	2-MeTHF	602	5	0.08	-	1% in PS	548	78	1.2	-	500
**DAC*-5**	2-MeTHF	666	2	0.052	-	1% in PS	616	30	0.75	-	500
**DAC*-6**	2-MeTHF	-	-	-	-	1% in PS	704	3	0.19	-	500
**$(^{Ad}L)Cu(G_1)$**	toluene	511	28	0.79	40	20% in PVK	502	33	3.0	-	501
**$(^{Ad}L)Cu(G_2)$**	toluene	491	2.6	0.83	30	20% in PVK	498	6	1.97	-	501
**$(^{Ad}L)Au(G_1)$**	toluene	526	52	0.67	130	20% in PVK	510	68	0.66	-	501
**$(^{Ad}L)Au(G_2)$**	toluene	505	78	0.79	30	20% in PVK	508	48	1.06	-	501
**Cu^{CAAC}**	2-MeTHF	474	100	2.5	-	1% in PS	470	100	2.8	-	502
**Ag^{CAAC}**	2-MeTHF	512	71	.37	-	1% in PS	472	100	0.50	-	502
**Au^{CAAC}**	2-MeTHF	502	95	1.20	-	1% in PS	472	100	1.14	-	502

**Cu^{MAC}**	2-MeTHF	542	55	1.10	-	1% in PS	506	90	1.40	-	[502]
**Ag^{MAC}**	2-MeTHF	568	06	0.04	-	1% in PS	512	79	0.33	-	[502]
**Au^{MAC}**	2-MeTHF	544	50	0.79	-	1% in PS	512	85	0.83	-	[502]
**Cu^{BZI}**	toluene	450	75	1.50	-	1% in PS	434	86	4.41	-	[503]
**Ag^{BZI}**	toluene	458	50	3.27	-	1% in PS	438	85	4.90	-	[503]
**Au^{BZI}**	toluene	448	94	1.11	-	1% in PS	432	100	3.17	-	[503]
**Cu^{C6}**	toluene	556	3.6	1.37	-	1% Zeonex	519	6	9.8	-	[504]
**Au^{C6}**	toluene	570	22	0.26	-	1% Zeonex	523	54	1.0	-	[504]
**Cu^{BIC}**	toluene	502	100	3.3	-	1% Zeonex	493	64	5.0	-	[504]
**Ag^{BIC}**	toluene	526	82	0.5	-	1% Zeonex	496	84	0.9	-	[504]
**Au^{BIC}**	toluene	512	100	1.1	-	1% Zeonex	490	100	1.3	-	[504]
**MCMA**	-	-	-	-	-	1% in PMMA	510	-	1.37	-	[505]
**ECMA**	-	-	-	-	-	1% in PMMA	510	-	1.87	-	[505]
**BCMA**	-	-	-	-	-	1% in PMMA	510	91	0.52	-	[505]
**PCMA**	-	-	-	-	-	1% in PMMA	520	89	0.35	-	[505]
**Au^{CAAC}-1**	toluene	495	96	0.74	110	neat film	484	-	0.99	-	[506]
**Au^{CAAC}-2**	toluene	456	61	11.5	120	neat film	425	-	10.8	-	[506]
**Au^{CAAC}-3**	toluene	528	98	1.25	200	neat film	500	-	0.76	-	[506]
**Au^{CAAC}-4**	toluene	552	95	0.84	300	neat film	540	-	0.69	-	[506]
**Cu1**	toluene	489	50	1.13	-	5% in PS	458	26	8.5	-	[507]
**Au1**	toluene	505	90	0.74	-	5% in PS	464	66	0.87	-	[507]
**Cu2**	toluene	589	20	0.58	-	5% in PS	528	37	3.6	-	[507]
**Au2**	toluene	603	20	0.15	-	5% in PS	563	40	0.61	-	[507]
**Cu3**	toluene	607	3	0.43	-	5% in PS	490	1.7	4.1	-	[507]
**Au3**	toluene	620	1.7	0.19	-	5% in PS	574	50	1.5	-	[507]
**Au4**	toluene	629	2	0.14	-	5% in PS	607	33	0.15	-	[507]
**Au5**	toluene	574	0.4	0.27	-	5% in PS	615	30	2.82	-	[507]
**Cu6**	toluene	661	-	0.11	-	5% in PS	614	-	-	-	[507]
**Au6**	toluene	672	1	0.25	-	5% in PS	637	17	0.77	-	[507]
**Au7**	toluene	654	8	0.6	-	5% in PS	649	8.5	2.2	-	[507]
**Au8**	toluene	689	<0.1	0.04	-	5% in PS	-	-	-	-	[507]
**[Cu(Cz)(^{Dipp}CAArC)**	THF	738	<0.01	0.009	-	Powder	621	32	0.37	-	[508]
**Au^{C}**	2-MeTHF	558	36	0.77	-	1% in PS	526	62	0.93	-	[509]
**Au_2^{CC}**	2-MeTHF	496	20	0.21	-	1% in PS	480	80	0.52	-	[509]
**CMA1**	toluene	528	95	1.2	240	3% in PS	498	73	1.2	-	[510]
**Aza1**	toluene	474	73	5.1	90	3% in PS	449	23	-	-	[510]
**Aza2**	toluene	500	95	1.9	70	3% in PS	478	27	-	-	[510]
**Aza3**	toluene	486	61	0.8	90	3% in PS	450	66	2.1	-	[510]
**Aza4**	toluene	506	100	1.7	30	3% in PS	454	32	-	-	[510]
**3,6-DiAza**	toluene	433	16	3.2	70	3% in PS	419	6	-	-	[510]
**Me-Cu^{CN}**	-	-	-	-	-	1% in PS	482	77	1.4	83	[511]

**Ph-Cu^{CN}**	-	-	-	-	-	1% in PS	500	83	1.1	55	[511]
**Me-Cu**	-	-	-	-	-	1% in PS	534	58	1.5	64	[511]
**Ph-Cu**	-	-	-	-	-	1% in PS	556	70	0.97	55	[511]
**Me-Ag^{CN}**	-	-	-	-	-	1% in PS	476	83	0.41	16	[511]
**Ph-Ag^{CN}**	-	-	-	-	-	1% in PS	498	88	0.60	10	[511]
**Me-Ag**	-	-	-	-	-	1% in PS	530	77	0.41	14	[511]
**Ph-Ag**	-	-	-	-	-	1% in PS	558	56	0.53	14	[511]
**Me-Au^{CN}**	-	-	-	-	-	1% in PS	484	50	0.81	78	[511]
**Ph-Au^{CN}**	-	-	-	-	-	1% in PS	504	100	0.82	61	[511]
**Me-Au**	-	-	-	-	-	1% in PS	528	50	1.1	75	[511]
**Ph-Au**	-	-	-	-	-	1% in PS	554	77	0.80	59	[511]
**Cu_{BCz}^{BZAC}**	toluene	502	>95	0.71	-	1% in PS	459	93	1.24	-	[512]
**Au_{BCz}^{BZAC}**	toluene	500	>95	0.56	-	1% in PS	484	>95	0.72	-	[512]
**Cu_{BCz}^{PAC}**	toluene	595	47	0.56	-	1% in PS	594	75	0.95	-	[512]
**Ag_{BCz}^{PAC}**	toluene	610	10	0.58	-	1% in PS	588	51	0.26	-	[512]
**Au_{BCz}^{PAC}**	toluene	588	42	0.44	-	1% in PS	586	76	0.74	-	[512]
**Au_{Cz}^{PZI}**	toluene	600	62	0.41	230	1% in PS	570	92	0.45	-	[512]
**Au_{Cz}^{PAC}**	toluene	546	73	0.74	250	1% in PS	546	>95	0.81	-	[512]
**Au_{Cz}^{BZAC}**	toluene	480	>95	0.69	230	1% in PS	479	>95	1.98	-	[512]
**Au_{bim}^{PZI}**	toluene	600	31	0.21	160	1% in PS	552	91	0.24	-	[512]
**Au_{bim}^{PAC}**	toluene	562	30	0.17	190	1% in PS	532	81	0.27	-	[512]
**Au_{bim}^{MAC}**	toluene	548	19	0.17	180	1% in PS	506	88	0.40	-	[512]
**Au_{bim}^{CAAC}**	toluene	514	87	0.63	220	1% in PS	476	>95	0.55	-	[512]
**Au_{bim}^{BZAC}**	toluene	484	>95	0.43	170	1% in PS	452	>95	0.28	-	[512]
**Au_{bim}^{BZI}**	toluene	454	>95	0.42	180	1% in PS	429	>95	0.25	-	[512]
**Au_{bim}^{IPr}**	toluene	340	<1	-	300	1% in PS	400	<5	13	-	[512]
**Au_{Mbim}^{BZI}**	toluene	460	>95	0.42	-	1% in PS	436	>95	0.29	-	[512]
**Au_{Obim}^{BZI}**	toluene	480	92	0.37	-	1% in PS	450	>95	0.38	-	[512]
**Cu1**	toluene	624	29	0.18	-	2% in mCP	567	88	0.41	-	[513]
**Cu2**	toluene	555	58	0.36	-	2% in mCP	508	89	0.41	-	[513]
**Cu3**	toluene	660	14	0.11	-	2% in mCP	581	66	0.37	-	[513]
**Cu4**	toluene	635	15	0.12	-	2% in mCP	568	76	0.36	-	[513]
**Cu5**	toluene	502	74	0.55	-	5% in mCP	470	52	0.47	-	[513]
**(BZI)Au(TMCz)**	toluene	484	79	1.1	-	5% in zeonex	466	95	0.38	-	[514]
**Au-tCz**	2-MeTHF	650	-	-	-	5% in mCP	562	75	0.37	88	[515]
**Au-Ph**	2-MeTHF	650	-	-	-	5% in mCP	565	89	0.46	-	[515]
**Au-DPXZ**	2-MeTHF	650	-	-	-	5% in mCP	570	73	1.11	-	[515]
**Au-PCz**	2-MeTHF	650	-	-	-	5% in mCP	570	78	0.67	-	[515]
**Au-PDMAC**	2-MeTHF	650	-	-	-	5% in mCP	565	94	0.61	-	[515]
**Au-PPXZ**	2-MeTHF	650	-	-	-	5% in mCP	565	82	0.57	-	[515]
**(rac)-PSIPr*-Cu-DMAC**	toluene	531	24	0.19	0.92	1% in PS	512	13	0.68	-	[516]

**MAC*-Cu-DPAC**	toluene	638	12	0.11	-	5% in PS	609	24	0.42	55	[517]
**Palladium**											
**PdN3N**	DCM	534	-	-	-	6 % in PMMA	528	72	142	-	[518]
**PdN3O**	-	-	-	-	-	6 % in PMMA	523	73	205	-	[518]
**PdN1N**	DCM	472	70	40	-	-	-	-	-	-	[519]
**PdN1N-dm**	DCM	470	77	52	-	-	-	-	-	-	[519]
**PdN6N**	DCM	476	59	61	-	-	-	-	-	-	[519]
**Pd(tzp-OCzPy)**	DCM	498, 537, 582	0.21	30.5	-	PMMA	461, 498, 536, 580	-	89.4	-	[520]
**Pd(tzp-OCzPy-Me)**	DCM	498, 536, 580	0.52	26.7	-	PMMA	462, 498, 536, 579	-	79.9	-	[520]
**Pd(tzp-OCzPy-OMe)**	DCM	513, 550	1.34	153	-	PMMA	457, 513, 548	-	110	-	[520]
**Pd(tzp-OCzPy-DM-tBu)**	DCM	513, 550	1.72	119	-	PMMA	456, 515, 551	-	57.0	-	[520]
**Pd(ACzCz-1)**	DCM	479	4	0.1	-	5% in PMMA	459	11	17.8	-	[521]
**Pd(AzCzCz-2)**	DCM	506	9	0.7	-	5% in PMMA	467	10	22.7	-	[521]
**Pd-O-S**	Toluene	640, 772	1	161	331	PS	-	19	-	-	[522]
**Pd-T-S**	Toluene	639, 786	0.3	157	363	PS	-	16	-	-	[522]
**Pd-T-I**	Toluene	652, 786	0.4	53	324	PS	-	15	-	-	[522]
**Platinum**											
**$Pt(BINAP)_2$**	Toluene	763	12	1.25	-	-	-	-	-	-	[523]
**Pt-1**	DCM	537	79	10	-	-	-	-	-	-	[524]
**Pt-2**	Toluene	612	68	1.2	-	0.1% in Zeonex	605	-	2	66	[525]
**Pt-Cl**	Toluene	617	16	1.2	-	0.1% in PS	640	11	5.7	-	[526]
**Pt-I**	Toluene	628	43	1.0	116	PS	618	-	2.5	58	[527]
**Pt-O-S**	Toluene	625, 742	0.4	33	313	PS	-	47	-	-	[522]
**Pt-T-S**	Toluene	624, 756	0.2	41	347	PS	-	34	-	-	[522]
**Pt-T-I**	Toluene	639, 755	0.2	12	298	PS	-	33	-	-	[522]
**Zinc**											
**$Zn(p-PX-BOX)_2$**	-	-	-	-	-	6 wt% mCBP	542	78.4	37.8	60	[528]

**Zn(m-PX-BOX)$_2$**	-	-	-	-	-	6 wt% mCBP	523	58.2	-	180	528
**[Zn(PhOPy-PXZ)$_2$]$_2$**	-	-	-	-	-	powder	538	13	2.09	100	529
**[Zn(PhOPy-DMAC)$_2$]$_2$**	-	-	-	-	-	powder	497	50	2.45	70	529
**Zn(HL)Cl$_2$ (*enol*)**	-	-	-	-	-	powder	565	7	-	110	530
**Zn(HL)Cl$_2$ (*keto*)**	-	-	-	-	-	powder	640	0.02	-	-	530
**Zn(R/S)-6-MeOsalen**	THF	491	-	-	-	thin film	576	-	8.42 (R), 7.39 (S)	-	531
**Zn-Schiff-1**	Toluene	542	31	2.1 x 10^3	310	PS	542	30	7.41 x 10^3	-	532
**Zn-Schiff-2**	Toluene	547	41	435	280	PS	547	65	1.45 x 10^3	-	532
**ZnPZ-Cz**	-	-	-	-	-	PS	626	7.1	132	-	533
**ZnPZ-Ph-Cz**	-	-	-	-	-	PS	611	2.3	991	-	533
**ZnPH-Cz**	-	-	-	-	-	PS	554	31.7	1.24 x 10^3	-	533
**ZnPH-Ph-Cz**	-	-	-	-	-	PS	555	10.9	3.53 x 10^3	-	533
**Zn-OS**	Toluene	667	-	> 1 x 10^3	-	PSAN	675	3.3	7.87 x 10^3	-	534
**Zn-NPN-5**	-	-	-	-	-	powder	480	100	24.5	120	535
**Other Metals**											
**SNF$_2$-Copro III**	-	-	-	-	-	Neat film	571	-	-	400	536
**SNF$_2$-Meso IX**	-	-	-	-	-	Neat film	570	-	-	400	536
**SNF$_2$-Hemato IX**	-	-	-	-	-	Neat film	576	-	-	370	536
**SNF$_2$-Proto IX**	-	-	-	-	-	Neat film	579	-	-	380	536
**SNF$_2$-OEP**	-	-	-	-	-	Neat film	571	-	-	400	536
**SNF$_2$-Etio I**	-	-	-	-	-	Neat film	569	-	-	400	536
**Si(^{Me}PDP^{Ph})$_2$**	THF	527	47	900	243	-	-	-	-	-	537
**Ge(^{Me}PDP^{Ph})$_2$**	THF	519	49	1000	260	-	-	-	-	-	537
**Sn(^{Me}PDP^{Ph})$_2$**	THF	512	32	2000	313	-	-	-	-	-	537
**W(CNdippPh)$_6$**	Toluene	617	41	1.73	-	-	-	-	-	-	538
**W(CNdippPh^{Ph})$_6$**	Toluene	629	44	1.53	-	-	-	-	-	-	538
**W(CNdippPh^{OMe2})$_6$**	Toluene	618	42	1.65	-	-	-	-	-	-	538
**W(CNdippPh^{Ome3})$_6$**	Toluene	612	41	1.83	-	-	-	-	-	-	538
**W(O)$_2$(N-Ar$_3$-Salen)**	Toluene	554	56	14.7	-	5 wt% mCP	554	84	2.0	-	539
**Zr(^{Mes}PDP^{Ph})$_2$**	THF	581	45	350	200	-	-	-	-	-	540
**Zr(^{Mes}PDP^{Ph})$_2$**	Benzene	521	34	313	-	-	-	-	-	-	541
**Zr(^{Mes}PDP^{Ph})$_2$**	Benzene	532	27	474	-	-	-	-	-	-	541
**Zr(^{Mes}PDP^{Ph})$_2$**	Benzene	524	10	260	-	-	-	-	-	-	541

**Zr(^{Mes}PDP^{Ph})$_2$**	Benzene	531	22	576	-	-	-	-	-	-	[541]
**Zr(^{Mes}PDP^{Ph})$_2$**	Benzene	547	11	190	-	-	-	-	-	-	[541]
**Zr(^{H}PDP^{H})$_2$**	Benzene	482	28	413	-	-	-	-	-	-	[541]
**Zr(^{H}PDP^{H})$_2$(THF)$_2$**	Benzene	430	12	619	-	-	-	-	-	-	[541]
**Mg(p-PX-BOX)$_2$**	-	-	-	-	-	6 wt% mCBP	516	70.6	-	80	[528]
**Li(p-PX-BOX)**	-	-	-	-	-	6 wt% mCBP	510	70.4	-	80	[528]
**[Al(p-PX-BOX)$_2$(μ-OH)$_2$]**	2-MeTHF	522	-	-	60	6 wt% mCP	530	86.7	-	-	[528]
**[Li$_2${(R)-PEPIA}$_2$]**	-	-	-	-	-	Neat film	465	8	4.1	-	[542]
**[Na$_2${(R)-PEPIA}$_2$]**	-	-	-	-	-	Neat film	476	36	6.9	78	[542]
**[K$_2${(R)-PEPIA}$_2$]**	-	-	-	-	-	Neat film	462	21	12.5	73	[542]
**[Rb$_2${(R)-PEPIA}$_2$]**	-	-	-	-	-	Neat film	476	21	14.8	90	[542]
**Ca(NPN)$_2$**	-	-	-	-	-	Neat film	470	22	24	148	[543]
**Al(*t*Bu-acac-PhDMAC)$_3$**	Toluene	495	32	2.4	-	30 wt% CBP	497	65	4.4	210	[544]
**Al(Ph-acac-PhDMAC)$_3$**	Toluene	527	61	1.0	-	30 wt% CBP	534	79	3.9	80	[544]
**Ir-5**	Chlorobenzene	461, 489, 565	30	0.83, 0.79	-	-	-	-	-	-	[545]
**Ir-6**	Chlorobenzene	469, 499, 583	13	0.52, 0.49	-	-	-	-	-	-	[545]
**BG**	Toluene	530	76	-	-	0.4 mol% mCPCN	-	76	-	-	[546]
**GG**	Toluene	540	54	-	-	0.4 mol% mCPCN	-	60	-	-	[546]
**BR**	Toluene	610	42	-	-	0.1 mol% TCTA	-	66	-	-	[547]
**GR**	Toluene	610	64	-	-	0.1 mol% TCTA	-	52	-	-	[547]

[a]Measured from a powdered sample, [b]Measured at 10% w/w doped mCP film.

Table **S12.** Performance data for OLEDs fabricated with metal TADF complexes reviewed in Section **9**.

**Complex**	**Emissive layer**	**λ_{EL} / nm**	**CIE**	**EQE$_{MAX}$ / %**	**EQE$_{100}$ / %**	**EQE$_{1000}$ / %**	**Reference**
**Copper**							
**[Cu(pypz)(POP)]BF$_4$**	Solution, 20% in 26mCPy	516	(0.21, 0.36)	3.18	-	-	[409]
**[Cu(pympz)(POP)]BF$_4$**	Solution, 20% in DPEPO	484	(0.17, 0.21)	3.72	-	-	[409]
**[Cu(pytfmpz)(POP)]BF$_4$**	Solution, 20% in 26mCPy	508	(0.21, 0.33)	8.47	-	-	[409]
**[Cu(ECAF)(POP)]PF$_6$**	10% in mCP	5444	(0.37, 0.55)	14.81	-	-	[412]

**[Cu(EHCAF) (POP)]PF_6**	10% in mCP	544	(0.38, 0.55)	11.17	-	-	412
**[Cu(PCAF)(POP)]PF_6**	10% in mCP	544	(0.38, 0.54)	6.67	-	-	412
**[Cu(czpzpy)(POP)]BF_4**	Solution, 20% in czpzpy	514	(0.26, 0.49)	6.34	6.29	-	413
**[Cu(PNNA)(POP)]BF_4**	Solution, 20% in mCP	490	(0.17, 0.37)	5.83	5.70	-	414
**[Cu(PNNA)(xant)]BF_4**	Solution, 20% in mCP	501	(0.21, 0.43)	7.42	7.41	-	414
**[Cu(m42)(POP)]BF_4**	Solution, 15% in BCPO	-	(0.32, 0.53)	10.5	-	-	415
**[Cu(DMAC-PyPI)(xant)]BF_4**	Solution, 24% in PYD2	-	(0.43, 0.54)	5.91	-	-	419
**[Cu(PXZ-PyPI)(POP)]BF_4**	Solution, 16% in PYD2	-	(0.51, 0.41)	7.96	-	-	419
**Cu(Ph_2Bpz_2)(dppb)**	10% in mCP	552	[0.40, 0.53]	11.9	-	-	424
**Cu(Ph_2Bpz_2)(dppb-F)**	10% in mCP	545	[0.37, 0.54]	16.0	-	-	424
**Cu(Ph_2Bpz_2) (dppb-CF_3)**	10% in mCP	528	[0.34, 0.54]	17.7	-	-	424
**Cu(PP)(PS)**	10% in mCP + 30% TAPC	-	[0.40, 0.53]	7.8	-	-	425
**TTPPCuCl**	10% in mCP	584	[0.47, 0.49]	9.6	9.0	7.1	426
**TTPPCuBr**	10% in mCP	584	[0.46, 0.49]	12.4	11.5	9.3	426
**TTPPCuI**	10% in mCP	584	[0.47, 0.49]	16.3	16.3	15.3	426
**[CuCl(dpmt)(PPh_3)]**	Neat Film	540	[0.35, 0.50]	2.47	-	-	428
**[CuBr(dpts)(PPh_3)]**	Neat Film	564	[0.43, 0.51]	7.74	-	-	430
**[Cu(dmpzpp)I]**	2% in DPEPO:TCTA (1:1)	521	[0.33, 0.52]	16.4	-	10.2	431
**[Cu(dmpzpp)SPh]**	4% in DPEPO:TCTA (1:1)	535	[0.36, 0.52]	12.8	-	-	431
**(L_{Me})CuBr**	10% in mCP	517	-	21.3	-	-	436
**(L_{Et})CuBr**	10% in mCP	529	[0.32, 0.54]	22.5	-	-	437
**(L_{iPr})CuBr**	10% in mCP	515	[0.26, 0.51]	18.6	-	-	437
**CuI(mpdp)**	Neat Film	-	[0.44, 0.47]	0.26	-	-	439
**CuI:CzBPDCb**	8 wt% in TCTA	520	-	17.5	15.8	-	447
**CuCl:azaSPF**	5 wt% in TCTA	576	-	9.5	-	-	448
**CuBr:azaSPF**	7 wt% in TCTA	524	-	13.6	-	-	448
**CuI:azaSPF**	9 wt% in TCTA	552	-	10.7	-	-	448
**CuI:α-aza-SBF**	8 wt% in **α-aza-SBF**	552	-	6.6	-	3.5	449
**CuI:β-aza-SBF**	8 wt% in **β-aza-SBF**	588	-	7.6	-	5.9	449
**CuI:γ-aza-SBF**	8 wt% in **γ-aza-SBF**	552	-	8.8	-	5.6	449
**CuI:δ-aza-SBF**	8 wt% in **δ-aza-SBF**	540	-	16.8	-	8.1	449
**$[Cu(mI)dppb]_2$**	10 wt% in CBP	565	-	4.8	-	-	450
**$\{Cu(PNP\text{-}^tBu)\}_2$**	0.25 wt% in CBP and 25% TAPC	512	-	16.1	-	-	451
**1-Cu**	30 wt% in PYD2	555	-	23	-	-	452
**$[Cu(m\text{-}Cl)dpmb]_2$**	10 wt% in mCP	528	[0.31, 0.54]	10	-	-	454
**$[Cu(m\text{-}Br)dpmb]_2$**	10 wt% in mCP	528	[0.31, 0.50]	7.3	-	-	454
**$[Cu(m\text{-}I)dpmb]_2$**	10 wt% in mCP	528	[0.38, 0.51]	8.3	-	-	454
**[Cu_2I_2(MePyrPHOS)(P(mTol)$_3$)]**	40 wt% in PYD2	552	-	11.4	-	-	456
**[Cu_2(pytzph)$(POP)_2$]$(BF_4)_2$**	20 wt% in PYD2	523	[0.30, 0.54]	7.6	7.5	-	457
**[Cu_2(pytzphcf)$(POP)_2$]$(BF_4)_2$**	20 wt% in PYD2	526	[0.30, 0.54]	5.9	19.4	-	457
**[Cu_2(pytzphcz)$(POP)_2$]$(BF_4)_2$**	20 wt% in PYD2	522	[0.29, 0.53]	8.1	26.2	-	457

**$[(P\hat{}N)Cu(m\text{-}I)]_2$**	10 wt% in mCP:TCTA (1:1)	516	[0.35, 0.51]	3	-	-	459
**$(DBFDP)_2Cu_4I_4$**	10 wt% in mCP	550	[0.37, 0.45]	0.73	-	-	463
**$(DCzDBFDP)_2Cu_4I_4$**	10 wt% in TmPyPB	560	[0.22, 0.44]	6	-	-	464
**$(DtBDCzDBFDP)_2Cu_4I_4$**	10 wt% in TmPyPB	560	[0.23, 0.42]	7.9	-	-	464
**$[Cu_4(PCP)_3]BF_4$**	17wt% in TAPC	-	[0.30, 0.64]	11.2	-	-	467
**Silver**							
**Ag(PI-PXZ)(POP)**	8 wt.% in PYD2	560	[0.45, 0.62]	8.76	-	-	472
**Gold**							
**Au-CC-2**	10 wt% in TCTA	-	[0.32, 0.54]	20.37	-	9.72	486
**Au-CC-4**	10 wt% in TCTA	-	[0.40, 0.56]	14.90	-	8.50	486
**(SIPr)AuBN**	0.5 wt% DMI-Cz:DMIC-TRz (1:1)	511	[0.20, 0.69]	24.8	-	2	487
**(BzIPr)AuBN**	2 wt% DMI-Cz:DMIC-TRz (1:1)	510	[0.16, 0.68]	30.3	-	7.3	487
**(PyIPr)AuBN**	2 wt% DMI-Cz:DMIC-TRz (1:1)	516	[0.19, 0.70]	27.3	-	12.8	487
**(PzIPr)AuBN**	4 wt% DMI-Cz:DMIC-TRz (1:1)	515	[0.22, 0.67]	24	-	3.8	487
**IPzIDCz**	1 wt% DMIC-TRz	531	[0.37, 0.57]	23.9	-	23.7	488
**$(C\hat{}N\hat{}C)Au(PhN(Ph)_2)$**	16 wt% PYD2	509	[0.32, 0.55]	14.8	-	14.7	490
**$(F_2C\hat{}NOEt\hat{}CF_2)Au(PhN(Ph)_2)$**	16 wt% PYD2	500	[0.27, 0.51]	23.8	-	16.5	490
**$(CF_2\hat{}N^{NMe2}\hat{}CF_2)Au(PhN(Ph)_2)$**	6 wt% PYD2	473	[0.16, 0.25]	15.25	-	9.98	491
**$(CF_2\hat{}N^{NMe2}\hat{}CF_2)Au(PhN(p\text{-}FC_6H_4)_2)$**	6 wt% PYD2	465	[0.16, 0.23]	6.76	-	2.51	491
**$(CF_2\hat{}N\hat{}CF_2)Au(PhN(Ph)_2)$**	20 wt% PYD2	534	[0.35, 0.56]	24.32	-	18.40	491
**$(CF_2\hat{}N\hat{}CF_2)Au(C\equiv CPh)$**	4 wt% TCTA:TPBi (1:1)	-	[0.34, 0.56]	23.1	-	19.2	492
**$(CF_2\hat{}N\hat{}CF_2)Au(C\equiv C\text{-}Ph\text{-}PXZ)$**	4 wt% TCTA:TPBi (1:1)	-	[0.41, 0.55]	19.7	-	15.0	492
**$(CF_2\hat{}N\hat{}CF_2)Au(C\equiv C\text{-}Me_2Ph\text{-}pNPh_2)$**	4 wt% TCTA:TPBi (1:1)	-	[0.40, 0.55]	23.4	-	22.1	492
**$[Au\{4\text{-}^tBuC\hat{}C(4\text{-}^tBuC_6H_4)\hat{}N(1\text{-}thpy)\}(Cbz)]$**	Solution 8% m-CPB	576	[0.49, 0.50]	14.3	-	-	493
**$[Au\{4\text{-}^tBuC\hat{}C(4\text{-}^tBuC_6H_4)\hat{}N(1\text{-}thpy)\}(^tBu_2Cbz)]$**	Solution 8% m-CPB	612	[0.57, 0.43]	7.1	-	-	493
**$[Au\{4\text{-}^tBuC\hat{}C(4\text{-}^tBuC_6H_4)\hat{}N(1\text{-}thq)\}(Cbz)]$**	Solution 5% m-CPB	628	[0.60, 0.40]	8.2	-	-	493
**$[Au\{4\text{-}^tBuC\hat{}C(4\text{-}^tBuC_6H_4)\hat{}N(2\text{-}thpy)\}(Cbz)]$**	Solution 11% m-CPB	568	[0.47, 0.52]	14.5	-	-	493
**$[Au\{4\text{-}^tBuC\hat{}C(4\text{-}^tBuC_6H_4)\hat{}N(2\text{-}thpy)\}(^tBu_2Cbz)]$**	Solution 11% m-CPB	600	[0.54, 0.45]	8.7	-	-	493
**$[Au\{4\text{-}^tBuC\hat{}C(4\text{-}^tBuC_6H_4)\hat{}N\}(Cbz)]$**	20 wt% CBP	548	[0.40, 0.57]	11.9	-	-	494
**$[Au\{4\text{-}^tBuC\hat{}C(4\text{-}^tBuC_6H_4)\hat{}N\}\{(Cbz)\text{-}(Cbz)_2\}]$**	20 wt% CBP	560	[0.43, 0.54]	8.7	-	-	494
**$[Au\{4\text{-}^tBuC\hat{}C(4\text{-}^tBuC_6H_4)\hat{}N\}\{(Cbz)\text{-}(Cbz)_2\text{-}(Cbz)_4\}]$**	20 wt% CBP	540	[0.38, 0.57]	15.8	-	-	494
**$[Au\{4\text{-}^tBuC\hat{}C(4\text{-}^tBuC_6H_4)\hat{}N\}(3\text{-}$**	Solution 20 wt% MCP	500, 528	[0.29, 0.56]	8.4	-	2.9	495

**CN-Cbz)]**							
**[Au{4-^{t}BuC^C(4-^{t}BuC$_6$H$_4$)^N}(3-(P(O)Ph$_2$)-Cbz)]**	20 wt% MCP	520	[0.29, 0.58]	11.7	-	-	[495]
**[Au{4-^{t}BuC^C(4-^{t}BuC$_6$H$_4$)^N}(3-(C$_6$H$_4$-NPh$_2$)-Cbz)]**	Solution 20 wt% MCP	584	[0.51, 0.48]	6.1	-	5.9	[495]
**[Au{4-^{t}BuC^C(4-^{t}BuC$_6$H$_4$)^N}(3-(MeC$_6$H$_3$-NPh$_2$)-Cbz)]**	20 wt% MCP	576	[0.49, 0.50]	10.0	-	-	[495]
**[Au{4-^{t}BuC^C(4-^{t}BuC$_6$H$_4$)^N}(2-(C$_6$H$_4$-NPh$_2$)-Cbz)]**	20 wt% MCP	560	[0.44, 0.54]	11.4	-	-	[495]
**[Au{4-^{t}BuC^C(4-^{t}BuC$_6$H$_4$)^N}(2-(MeC$_6$H$_3$-NPh$_2$)-Cbz)]**	20 wt% MCP	556	[0.42, 0.55]	10.9	-	-	[495]
**Au(C^C^{OPh}^NPh^{t}Bu$_2$^CPXZ)**	16 wt% TCTA	554	[0.43, 0.54]	25.03	-	22.01	[496]
**Au(CDPA^C^{Me}^N^C)**	8 wt% TCTA	505	[0.26, 0.54]	23.52	-	15.44	[496]
**Au(CPXZ^C^{Me}^N^C)**	4 wt% TCTA	529	[0.34, 0.56]	13.88	-	12.01	[496]
**Au-1**	4 wt% DPEPO:TCTA (1:1)	540	[0.38, 0.57]	24.9	-	20.5	[497]
**Au-2**	8 wt% DPEPO:TCTA (1:1)	529	[0.31, 0.60]	26.2	-	24.0	[497]
**Au-4**	12 wt% DPEPO:TCTA (1:1)	541	[0.36, 0.60]	26.8	-	26.2	[497]
**CMA**							
**CMA1**	20% in PVK	-	[0.26, 0.48]	26.3	26.1	25.2	[548]
**CMA2**	20% in PVK	-	-	9.7	8.9	9.2	[548]
**CMA3**	20% in PVK	-	-	17.9	17.7	15.5	[548]
**CMA4**	20% in PVK	-	[0.36,0.54]	27.5	26.6	24.5	[548]
**Ag-1**	solution<br>20% in PVK	542	[0.35, 0.52]	3.8	3.8	1.2	[498]
**Ag-1**	evaporated 20% in mCP	502	[0.25, 0.42]	4.3	2.7	-	[498]
**Ag-2**	solution<br>20% in PVK	540	[0.36, 0.56]	11.0	10.6	8.2	[498]
**Ag-2**	evaporated 20% in mCP	509	[0.28, 0.46]	13.7	12.9	10.0	[498]
**CAAC-1a**	evaporated 20% in UGH3	460	-	9.0	-	-	[499]
**MAC*3**	evaporated 40% in mCBP	543	-	19.4	-	-	[500]
**(^{Ad}L)Cu(G$_1$)**	20% in PVK	-	[0.37, 0.60]	5.6	5.5	4.6	[501]
**(^{Ad}L)Au(G$_1$)**	20% in PVK	-	[0.39, 0.58]	10.6	10.3	10.0	[501]
**(^{Ad}L)Au(G$_2$)**	20% in PVK	-	[0.37, 0.60]	3.7	3.6	2.8	[501]
**Au^{MAC}**	40% in mCBP	516	-	18.0	-	15.0	[502]
**Au^{BZI}**	5% in UGH3	430	[0.16, 0.06]	12.0	-	-	[503]
**Au^{CAAC}-1**	20% in DPEPO	450	[0.17, 0.17]	20.9	17.8	-	[506]
**Au^{CAAC}-3**	20% in TCP	500	[0.24, 0.42]	26.9	26.4	-	[506]
**Au^{CAAC}-4**	20% in DPEPO	518	[0.29, 0.49]	24.7	22.9	-	[506]
**Au1**	solution 20% in PVK	-	[0.15, 0.24]	5.8	5.5	3.6	[507]
**Au1**	solution 20% in CBP	-	[0.18, 0.31]	4.6	4.2	2.3	[507]
**Au3**	evaporated 20% in mCP	590	[0.53, 0.46]	11.0	9.8	9.1	[507]

**Cu1**	evaporated 6% in TCTA:TPBi	582	[0.51, 0.48]	18.7	-	18.3	513
**Cu2**	evaporated 4% in TCTA:DPEPO	521	[0.27, 0.57]	20.6	-	19.0	513
**Cu3**	evaporated 2% in TCTA:TPBi	619	[0.58, 0.42]	14.4	-	13.8	513
**Cu5**	evaporated 4% in TCTA:DPEPO	474	[0.14, 0.22]	23.6	-	18.7	513
**MAC*-Cu_DPAC**	evaporated 1.5% in CBP:TPBi	628	[0.58, 0.42]	21.1	21.0	20.1	517
**Palladium**							
**PdN3N**	evaporated 6% in 26mCPy	-	[0.30, 0.61]	20.9	14.3	-	518
**PdN3O**	evaporated 6% in 26mCPy	-	[0.30, 0.62]	20.4	13.9	-	518
**PdN1N-dm**	evaporated 10% in 26mCPy	476	[0.14, 0.25]	25.1	11.1	-	519
**Platinum**							
**Pt-2**	solution-processed 5% in (60%TPD:40%PBD)	607	[0.62, 0.37]	7.4	-	-	525
**Pt-Cl**	solution-processed 5% in (70%mCP:30%PO-T2T)	637, 730	-	2.64	-	-	526
**Pt-I**	solution-processed 5% in ((70%mCP:30%PO-T2T)	6112	[0.62, 0.36]	3.11	-	-	527
**Zinc**							
**Zn(R)-6-MeOsalen**	neat	576	[0.43, 0.48]	0.038	-	-	531
**Zn(R)-6-MeOsalen**	neat	576	[0.43, 0.48]	0.042	-	-	531
**Other Metals**							
**$W(O)_2(N\text{-}Ar_3\text{-}Salen)$**	30 wt% PVK : OXD-7 (9:1)	-	[0.49, 0.49]	15.56	-	9.70	539
**$Mg(p\text{-}PX\text{-}BOX)_2$**	1 wt% mCBP	-	-	16.5	-	-	528
**Li(p-PX-BOX)**	1 wt% mCBP	-	-	12.9	-	-	528
**Al(*t*Bu-acac-PhDMAC)$_3$**	10 wt% 26DCzPPy	522	[0.31, 0.54]	10.7	10.6	8.4	544
**Al(Ph-acac-PhDMAC)$_3$**	30 wt% CBP	556	[0.43, 0.55]	17.5	17.5	14.7	544
**Ir-5**	PVK:OXD-7:Ir-5 [100:50:8]	472-491	[0.22, 0.38]	1.46	-	-	545
**Ir-6**	PVK:OXD-7:Ir-5 [100:50:8]	476	[0.19, 0.38]	2.09	-	-	545
**BG**	0.4 mol% mCPCN	551	[0.41, 0.56]	9.8	-	-	546
**GG**	0.4 mol% mCPCN	541	[0.32, 0.63]	15.1	-	-	546
**BR**	0.1 mol% TCTA	420	[0.66, 0.34]	13.5	-	-	547
**GR**	0.1 mol% TCTA	-	[0.66, 0.35]	11.0	-	-	547

Table **S13.** Summary of photophysical and device performance of polymer TADF emitters reviewed in Section **10.1**.

Compound	Medium	λ_{PL} / nm	Φ_{PL} / %	τ_D / µs	ΔE_{ST} / eV	λ_{EL} / nm	CIE_{XY}	EQE_{max} / %	Roll-off 100 cd m^{-2} / %	Roll-off 1000 cd m^{-2} / %	Ref
PCzDP-10	Neat	500	74	1.7	N/A	496	0.24, 0.40	16.1	30	N/A	549
PCzBN1	Neat	491	58	38.3	0.14	491	0.10, 0.43	17.8	N/A	N/A	550
PCzBN3	Neat	496	51	28.8	0.13	496	0.12, 0.54	17.5	N/A	N/A	550
PCzBN5	Neat	501	43	8.2	0.13	496	0.11, 0.53	13.3	N/A	N/A	550
PCz-mCP-PxzTrz-10	Neat	527	22	~0.5	0.09	539	0.33, 0.51	13.0	42	N/A	551
PCz-mCP-PxzTrz-20	Neat	533	21	~0.5	0.06	543	0.34, 0.51	13.9	20	N/A	551
PCz-mCP-PxzTrz-30	Neat	540	39	~0.5	0.06	546	0.35, 0.53	15.3	1	N/A	551
PCz-mCP-PxzTrz-40	Neat	544	19	~0.5	0.05	548	0.38, 0.54	8.3	7	N/A	551
PFDMPE-R05	Neat	592	32	126	N/A	606	0.57, 0.42	5.62	~71	~87	552
P-4CzCN	Neat	472	37	1.5	0.23	515	0.23, 0.39	3.6	N/A	N/A	553
P-Cz4CzCN	Neat	489	65	1.9	0.12	492	0.24, 0.47	11.5	N/A	N/A	553
PDCDC	Neat	496	68	1.29	0.02	498	0.23, 0.39	9.0	28	N/A	551
POPT-25	10 wt% in mCP	541	36	3.0	0.11	540	0.36, 0.50	5.2	33	~65	554
PCzPT-19	10 wt% in mCP	538	21	10.2	0.06	552	0.40, 0.55	1.2	25	~25	554
P1	Neat	547	10	N/A	0.09	N/A	0.46, 0.43	1.1	36	N/A	555
P2	Neat	542	24	N/A	0.06	N/A	0.45, 0.40	4.5	71	N/A	555
P3	Neat	535	20	N/A	0.04	416/535/580	0.37, 0.38	10.4	75	N/A	555
PDT-1	Neat	441	42	5.79	0.04	434	0.15, 0.08	3.9	10	N/A	556
PDT-2	Neat	444	54	5.87	0.03	436	0.15, 0.09	5.3	8	N/A	556
PDT-3	Neat	445	46	5.11	0.04	438	0.17, 0.14	4.4	7	N/A	556
PBD-5	Neat	453	38	47	N/A	474	0.19, 0.25	6.0	53	N/A	557
PBD-10	Neat	457	42	48	N/A	478	0.20, 0.29	7.3	45	N/A	557
PBD-15	Neat	461	55	50	N/A	474	0.22, 0.29	7.1	49	N/A	557
PBD-20	Neat	464	34	51	N/A	480	0.18, 0.30	6.7	45	N/A	557
D3P-DEH	Neat	~500	44	4.8	0.03	509	0.24, 0.51	1.57	N/A	N/A	558
PAPTC	Neat	507	28	0.68	0.13		0.30, 0.59	12.6	N/A	N/A	559
PABPC50	Neat	547	58	0.72	0.17	~563	0.45, 0.52	6.8	~0	0	560
PABPC25	Neat	541	66	0.84	0.14	~560	0.44,	16.1	~0	1	560

							0.54				
PABPC10	Neat	527	74	1.2	0.09	~549	0.40, 0.56	16.2	~0	1	560
PABPC5	Neat	525	77	1.3	0.12	~549	0.40, 0.56	18.1	~0	2	560
PABPC1	Neat	518	76	1.3	0.13	~541	0.36, 0.57	15.4	~0	8	560
Cop-10	10 wt% Polymer in 65 wt% TCTA : 25 wt% TAPC	540 [a]	21	47 [a]	N/A	678	0.46, 0.49	15.7	76	~89	561
pBP-PXZ	Neat	554	81.7	1.29	0.028	584	0.52, 0.48	13.71	N/A	N/A	562
pBP-PTZ	Neat	542	48.2	1.55	0.020	578	0.50, 0.49	7.92	N/A	N/A	562
P1	2 wt% in PS	~480	71	296	0.023	N/A	N/A	N/A	N/A	N/A	563
PPxPhO	20 wt% mCP	557 [c]	62.2 [c]	7.9 [c]	0.07 [c]	550	N/A	11.8	N/A	N/A	564
PFSOTT0.5	Neat	420/546	89	7.8	N/A	602	0.49, 0.49	2.6	N/A	N/A	565
PFSOTT2	Neat	416/567	76	6.2	N/A	616	0.55, 0.43	4.8	N/A	N/A	565
P10	5 wt% in CBP	539 [b]	10	1.4	N/A	528	0.36, 0.52	0.87	~3	~41	566
R-P	10 wt% in mCP	549	72	1.6	0.045	546	0.41, 0.57	14.9	N/A	15	567
S-P	10 wt% in mCP	547	76	2.3	0.061	544	0.40, 0.57	15.8	N/A	22	567
ASFCN	Neat	~520	16	N/A	N/A	N/A	N/A	N/A	N/A	N/A	568
LEP	Neat	N/A	44	N/A	0.22	N/A	0.32, 0.56	10	N/A	N/A	569
P(C2-Mac-BP)	Neat	~500	45	N/A	0.01	545	0.39, 0.57	6.7	18	54	570
P(C6-Mac-BP)	Neat	~500	21	N/A	0.01	537	0.37, 0.58	7.1	8	30	570
P(Ph-Mac-BP)	Neat	~500	35	N/A	0.01	399,548	0.39, 0.56	2.9	14	N/A	570
DV-3CzCN	Neat/cross-linked	476/478	36	1.4	0.23	484	0.16, 0.31	0.8	N/A	N/A	571
DVCz-3CzCN	Neat/ cross-linked	469/470	54	1.7	0.16	476	0.15, 0.30	6.8	N/A	N/A	571
DVCz-2CzCN	Neat/ cross-linked	470/~470	56/68	-/2.5	N/A	~475/~475	0.16, 0.21/0.16, 0.21	4.2/5.6	N/A	N/A	572
VBN0	Neat	479	55	4.3	0.11	484	0.22, 0.36	3.1	N/A	N/A	573
VBN10	Neat	488	74	1.3	0.22	492	0.20, 0.38	11.4	N/A	N/A	573
VBN50	Neat	497	70	2.2	0.21	500	0.21, 0.41	9.1	N/A	N/A	573
VBN100	Neat	512	64	3.5	0.22	516	0.27, 0.54	4.9	N/A	N/A	573
P-Ac95-TRZ05	Neat	~474	51	1.3	0.02	472	0.18, 0.27	12.1	1	5	574
P-TBAc95-TRZ05	Neat	N/A	N/A	N/A	N/A	545/635	0.21, 0.20	0.33	58	N/A	574
P1-05	Neat	485	54	3.6	0.05	483	0.20, 0.37	11.3	N/A	N/A	575
P1-10	Neat	489	47	3.3	0.04	491	0.21, 0.42	6.5	N/A	N/A	575
P1-20	Neat	492	45	3.0	0.04	502	0.24,	4.1	N/A	N/A	575

							0.47				
P4CzCN–PA	As host doped with 40 wt% 5tCzCN	458	N/A	0.12	0.14	498	0.25, 0.49	20.9	N/A	N/A	576
P4CzCN–BCz (1:4)	As host doped with 40 wt% 5TCzCN	450	N/A	0.11	0.17	507	0.25, 0.43	3.8	N/A	N/A	576

[a] neat, [b] THF, [c] Toluene.

Table **S14.** Summary of photophysical and device performance of TADF dendrimers reviewed in Section **10.2**.

**Compound**	**Medium**	**λ_{PL} / nm**	**Φ_{PL} / %**	**τ_D / μs**	**ΔE_{ST} / eV**	**λ_{EL} / nm**	**CIE_{XY}**	**EQE_{max} / %**	**Roll-off 100 cd m^{-2} / %**	**Roll-off 1000 cd m^{-2} / %**	**Ref**
G2TAZ	Neat	N/A	52	8.4	0.03		0.25, 0.49	2.4	N/A	N/A	577
G3TAZ	Neat	~510	31	4.7	0.06	~513	0.27, 0.49	3.4	~19	~56	577
G4TAZ	Neat	N/A	8.5	6.3	0.06		0.23, 0.34	1.5	N/A	N/A	577
tBuG2TAZ	Neat	~500	44	5.3	0.12	~500	N/A	9.5	1	23	578
MeG2TAZ	Neat	~502	40	3.3	0.10	~505	N/A	9.4	0	24	578
PhG2TAZ	Neat	~494	49	1.9	0.09	~505	N/A	8.2	2	27	578
CzG2TAZ	Neat	~601	52	4.0	0.12	~503	N/A	6.0	0	52	578
tBuG2B	Neat	501	74	2.2	0.08	502	0.27, 0.52	17.0	1	19	579
MeG2B	Neat	501	34	0.5	0.08	~502	0.28, 0.48	9.0	11	N/A	579
MeOG2B	Neat	539	17	0.6	0.12	~546	0.44, 0.51	6.4	9	69	579
PhG2B	Neat	507	41	1.2	0.14	~502	0.30, 0.48	8.8	7	N/A	579
t-BuTCz2BP	Neat	505	41	0.57	0.08	506	0.26, 0.46	4.3	N/A	N/A	580
G2B	Neat	493	33	0.7	0.12	500	0.26, 0.48	5.7	~17	~39	581
G3B	Neat	500	21	0.8	0.10	516	0.31, 0.50	2.9	~14	N/A	581
tBuCz2pTRZ	Neat	481	61	1.2	0.09	500	0.23, 0.46	18.5	13	N/A	582
tBuCz2mTRZ	Neat	483	59	1.1	0.09	516	0.27, 0.53	19.9	40	N/A	582
tBuCz2m2pTRZ	Neat	520	86	1.1	0.04	540	0.37, 0.37	28.7	26	N/A	582
tBuCz3mTRZ	Neat	517[a]	62	2.2	0.08[a]	516	0.27, 0.53	19.9	40	N/A	583
tBuCz4mTRZ	Neat	532[a]	67	1.7	0.05[a]	536	0.36, 0.58	23.8	0	N/A	583
tBuTCz-DPyM	Doped 8 wt% mCBP	495	63.5	9.5	0.01	503	0.25, 0.48	20.4	N/A	N/A	584
MeOTCz-DPyM	Doped 8 wt% mCBP	514	55	7.9	0.02	530	0.37, 0.54	9.2	N/A	N/A	584

tBuDPACz-DPyM	Neat	596	11	9.4	0.04	600	0.56, 0.43	1.82	14	N/A	585
MeODPACz-DPyM	Neat	645	3.1	9.4	0.03	649	0.62, 0.37	0.17	18	N/A	585
CDE2	Neat	499	75	0.6	0.15	522	0.32, 0.51	5.2		21	586
CzDMAC-DPS	Neat	492	68	1.5	0.09	~504	0.22, 0.44	12.2	46	63	587
DCzDMAC-DPS	Neat	464	48	1.9	0.20	~479	0.18, 0.27	2.2	32	N/A	587
DDA-DP	Neat	549	12.4[b]	0.45	0.04	550	0.36, 0.56	8.1	N/A	1%	146
DCz-DPS-Cz	Neat	494	88	1.51	0.03	498	0.23, 0.42	23.3	2	20	588
1CzAcDBTO	Neat	559	41	N/A	0.02	558	0.43, 0.54	3.9	~92	2	589
2CzAcDBTO	Neat	540	54	N/A	0.04	538	0.36, 0.54	4.5	~82	15	589
2CzSO	Neat	499	43	14.2	0.16	~525	0.27, 0.52	10.7	N/A	N/A	590
3CzSO	Neat	522	21	8.3	0.08	~525	0.31, 0.53	7.3	N/A	N/A	590
TPPOCz	Neat	400	33	1.29	0.22	~515	0.26, 0.31	2.0	N/A	N/A	591
Da	Neat	463	27.2	3.10	N/A	~460	0.17, 0.22	1.2	N/A	N/A	592
Db	Neat	526	21.3	1.77	N/A	~540	0.36, 0.57	4.1	N/A	N/A	592
BD–Cy	Neat	494	74[a]	0.41	0.05[b]	477	0.18, 0.28	18.2	22	55	593
YD–TF	Neat	555	86[a]	0.76	0.04[b]	552	0.41, 0.54	21.9	7	15	593
RD–2TF	Neat	618	49[a]	1.16	0.04[b]	626	0.60, 0.37	10.3	1	24	593
Cz-CzCN	Neat	509	52	2.8	0.17	510	0.26, 0.52	17.1	~7	~11	594
Cz-OCzBN	Neat	478	58	1.5	0.17	~478	0.18, 0.29	6.6	~17	~26	595
TA-3Cz	Neat	541	71	0.8	0.20[b]	546	0.39, 0.56	11.8	N/A	~58	596
TA-Cz	Neat	545	56	0.5	0.17[b]	550	0.41, 0.54	5.5	~5	~24	596
TB14CZ-ACTRZ	Neat	494	56	25	0.13	492	0.21, 0.42	8.1	~63	~72	597
TB2CZ-ACTRZ	Neat	520	69	2.9	0.08	520	0.31, 0.57	9.9	0	25	597
POCz-DPS	Neat	460	61	N/A	0.23	480	0.18, 0.30	7.3	~19	~16	598
tbCz-SO	Neat	440	N/A	0.1	0.21	464	0.16, 0.19	2.6	46	N/A	599
poCz-SO	Neat	458	N/A	0.2	0.11	480	0.18, 0.27	6.2	11	N/A	599
DCz-DPS-TCz	Neat	500	96	1.43	0.03	500	0.24, 0.45	24.0	2	11	588
MPPA-MCBP	Neat	690	10	0.7	0.17[b]	698	0.67, 0.31	0.62	~13	N/A	600
MPPA-3Cz	Neat	708	8	N/A	0.16[b]	715	0.69, 0.30	0.25	~24	N/A	601
MPPA-Cz	Neat	715	6	N/A	0.17[b]	728	0.70, 0.29	0.06	N/A	N/A	601

[a]10 wt% in PS, [b] Toluene, [c] 5 wt% in PMMA.

Table **S15.** Photoluminescence and electroluminescence properties of all reported MR-TADF emitters reviewed in Section **11**.

**Compound**	**Medium**[a]	**λ_{PL} / nm**	**Φ_{PL} / %**	**τ_d / µs**	**ΔE_{ST} / eV**	**λ_{EL} / nm**	**CIE_{XY}**	**EQE_{max} / %**	**EQE_{100} / %**	**EQE_{1000} / %**	**Ref**
**DOBNA (BOO)**	1 wt% PMMA	398	58	66	0.18	N/A	N/A	N/A	N/A	N/A	602-604
**DABNA-1**	1 wt% mCBP	460	88	94	0.18	459	0.13, 0.09	13.5	6.3 [b]	N/A	605
**DABNA-2**	1 wt% mCBP	469	90	65	0.14	467	0.12, 0.13	20.2	13.3 [b]	N/A	605
**TABNA**	1 wt% PMMA	399	54	N/A	0.21	N/A	N/A	N/A	N/A	N/A	606
***t*-DABNA**	5 wt% DPEPO	467	85	83.3	0.17	471[b]	N/A	25.1	6.0 [b]	N/A	607
**M-tDABNA**	3 wt% mCBP	461 [b]	84	195	0.11[c]	N/A	N/A	N/A	N/A	N/A	608
**DABNA-NP-M**	1 wt% PMMA	460	88	89	0.17	N/A	N/A	N/A	N/A	N/A	609
**DABNA-NP-TB (3tPAB, tDAB-DPA)**	1 wt% DOBNA-Tol	453 [c]	83 [c]	90 [c]	0.17 [c]	457	0.14, 0.11	19.5	17.5	12.0	609-611
**PAB (MR)**	3 wt% mCP	453	61	56	0.06	456	0.15, 0.08	14.7	8.0 [b]	N/A	611
**2tPAB**	3 wt% mCP	457	67	77	0.08	456	0.15, 0.08	16.8	8.9 [b]	3.1 [b]	611,612
**t-DABNA-dtB**	3 wt% mCBP	473	97	110	0.19	473	0.11, 0.16	25.4	5.7 [b]	2.4 [b]	613
**Cl-MR**	10 wt% DPEPO	474	85	17	0.13	472	0.12, 0.19	17.0	9.6 [b]	N/A	612
**Br-MR**	10 wt% DPEPO	474	76	9.9	0.13	476	0.14, 0.25	4.2	N/A	N/A	612
**tDPAC-BN**	10 wt% DPEPO	454 [d]	94 [c]	114 [c]	0.17 [d]	460	0.14, 0.08	12.4	1.6	N/A	614
**pBP-DABNA-Me**	5 wt% mCBP:DPEPO	462	98	53	0.18	464	0.13, 0.09	23.4	17.9 [b]	5.8 [b]	615
**mBP-DABNA-Me**	mCP:DPEPO	467	97	64.5	0.13	468	0.12, 0.14	24.4	19.8	9.1 [b]	616
**TBE01**	0.4 wt% SiCzCz:SiTrzCz2	459 [d]	91 [d]	240	0.16 [d]	N/A	N/A	N/A	N/A	N/A	617
**TBE02**	0.4 wt% SiCzCz:SiTrzCz2	459 [d]	89 [d]	125	0.14 [d]	N/A	N/A	N/A	N/A	N/A	617
**BOS**	1 wt% Polystyrene	434 [d]	63	90.6	0.17 [d]	N/A	N/A	N/A	N/A	N/A	604
**BSS**	Polystyrene	457 [d]	58	85.5	0.15 [d]	N/A	N/A	N/A	N/A	N/A	604
**PS-BOO**	Neat	398	73	133.8	N/A	N/A	N/A	N/A	N/A	N/A	604
**PS-BOS**	Neat	435	65	104.9	N/A	N/A	N/A	N/A	N/A	N/A	604
**PS-BSS**	Neat	456	59	67.0	N/A	N/A	N/A	N/A	N/A	N/A	604
**PAc-BSS**	Neat	455	60	71.9	N/A	458	0.16, 0.12	13.1	10.2 [b]	1.5 [b]	604
**PhCz-TSOBA**	10 wt% 2,6-DczPPy	444 [d]	61	47.2	0.23 [d]	456	0.14, 0.12	16.7	6.7 [d]	1.3 [d]	79
**B2**	1 wt% mCBP	455 [c]	53 [b]	30.4 [c]	0.19 [c]	460	0.13, 0.11	18.3	12.4	N/A	618
**B3**	1 wt%	441	33	N/A	0.15	N/A	N/A	N/A	N/A	N/A	618

	PMMA										
**B4**	1 wt% PMMA	450	57	N/A	0.15	N/A	N/A	N/A	N/A	N/A	618
***v*-DABNA**	1 wt% in DOBNA-Oar	467	90	4.1	0.02	469	0.12, 0.11	34.4	32.7	26.1	619
***t*-Bu-*v*-DABNA**	5 wt% DBPFO	467 [d]	92	2.9	0.04 [d]	474	0.11, 0.15	36.3	34.3 [b]	16.5	68
**m-*v*-DABNA**	3 wt% DBPFO	464 [d]	91	3.1	0.07 [d]	471	0.12, 0.12	36.2	30.0 [b]	10.7 [b]	620
**4F-*v*-DABNA**	3 wt% DBPFO	457 [d]	90	3.1	0.05 [d]	464	0.13, 0.08	35.8	28.8 [b]	N/A	620
**4F-m-*v*-DABNA**	3 wt% DBPFO	446 [d]	89	3.2	0.07 [d]	461	0.13, 0.06	33.7	27.0 [b]	N/A	620
***v*-DABNA-CN-Me**	1 wt% DOBNA-Ph	496 [d]	86 [d]	10 [d]	0.01 [d]	504	0.13, 0.65	31.6	31.5	28.5	621
**V-DABNA-Mes**	1 wt% Polymer C	484 [c]	80 [c]	2.4 [c]	0.01 [c]	480	0.09, 0.21	22.9	20.3	10.9	622
***v*-DABNA-O-Me**	1 wt% DOBNA-Tol	464 [c]	90 [c]	7.7 [c]	0.03 [c]	465	0.13, 0.10	29.5	28.8	26.9	623
**BOBO-Z**	3 wt% mCBP	445	64	7.7	0.15 [d]	445	0.15, 0.04	13.6	9.8	3.3	624
**BOBS-Z**	3 wt% mCBP	457	93	7.6	0.16 [d]	456	0.14, 0.06	26.9	24.0	15.0	624
**BSBS-Z**	3 wt% mCBP	464	88	6.7	0.14 [d]	463	0.13, 0.08	26.8	24.0	15.9	624
**α-3BNOH**	MeTHF	390	50	0.45	0.31	N/A	N/A	N/A	N/A	N/A	625
**α-3BNMes**	20 wt% mCBP	441 [e]	63 [f]	9.1, 7,100 [c]	0.28 [c]	444 [b]	0.15, 0.08	1.6 [b]	1.6 [b]	1.4 [b]	626
**DtBuCzB (BBCz-SB)**	1 wt% mCBP	493	88	69	0.13 [d]	488	0.10, 0.42	21.6	15.0	5.3	627,628
**Cz-B (CzBN)**	1 wt% mCBP	484	97	44	0.14 [d]	482	0.11, 0.31	22.6	16.6	6.9	629,630
**γ-Cb-B**	1 wt% o-CBP	461	89	32	0.12 [d]	461	0.13, 0.13	19.0	16.2	7.7	630
**AZA-BN**	4 wt% mCBP	526	94	13.4	0.18 [d]	527	0.28, 0.69	25.7	20.9	9.0	631
**BN-TP**	3 wt% PhCzBCz	529	96	44.0	0.14 [d]	528	0.26, 0.70	35.1	32.4	20.8	632
**2F-BN**	6 wt% mCBPC	502	89	25.6	0.16 [d]	N/A	N/A	N/A	N/A	N/A	633
**3F-BN**	6 wt% mCBPC	503	83	16.7	0.08 [d]	N/A	N/A	N/A	N/A	N/A	633
**4F-BN**	6 wt% mCBPC	501	91	19.0	0.11 [d]	N/A	N/A	N/A	N/A	N/A	633
**CN-BCz-BN**	Toluene	496	N/A	N/A	N/A	N/A	N/A	N/A	N/A	N/A	634
**D*t*CzB-DPTRZ**	3 wt% PhCzBCz	536	87	788	0.17 [d]	532	0.33, 0.63	24.6	7.5	5.1 [b]	635
**D*t*CzB-TPTRZ**	3 wt% PhCzBCz	520	95	84	0.11 [d]	516	0.18, 0.67	29.8	26.4	12.1 [b]	635
**D*t*CzB-PPm**	3 wt% PhCzBCz	510	94	87	0.08 [d]	508	0.16, 0.66	28.6	24.3	9.9 [b]	635
**D*t*CzB-CNPm**	3 wt% PhCzBCz	543	87	524	0.12 [d]	540	0.35, 0.63	25.0	14.4	5.9 [b]	635
**OBN-2CN-BN**	3 wt% PhCzBCz	498 [g]	95 [g]	95 [g]	0.12 [d, g]	496 [g] (496) [h]	0.11, 0.52 [g] (0.11, 0.52 [h])	29.4 [g] (28.8 [h])	19.8 [g] (19.2 [h])	8.1 [g] (8.0 [h])	317
**OBN-4CN-BN**	3 wt% PhCzBCz	510 [g]	90 [g]	97 [g]	0.13 [d, g]	508 [g] (496) [h]	0.14, 0.64 [g] (0.14, 0.64 [h])	24.5 [g] (24.3 [h])	8.0 [g] (7.9 [h])	2.1 [g] (1.9 [h])	317

***m*-Cz-BNCz**	10 wt% PhCzBCz	528	96	0.86	0.08 [d]	528	0.26, 0.68	31.4	29.0	17.5	636
**TCz-BN (*p*-Cz-CzBN**	Toluene	477	N/A	N/A	N/A	N/A	N/A	N/A	N/A	N/A	633,636
**BBCz-Y**	2 wt% mCBP	549	90	11	0.14 [d]	549	0.37, 0.61[b]	29.3	25.8 [b]	19.6 [b]	628
**BBCz-G**	2 wt% mCBP	519	99	13	0.14 [d]	515	0.26, 0.68	31.8	29.5 [b]	22.3 [b]	628
**Cz2DABNA-NP-M/TB**	1 wt% PMMA	478 [c]	85 [c]	19 [c]	0.15 [c]	477	0.11, 0.23	21.8	20.4	14.9	609
***t*DPA-DtCzB**	1 wt% PhCzBCz	484	85	29.5	0.11 [d]	480	0.11, 0.23	25.0	16.4	8.3	637
**CzBNCz**	1 wt% mCBP	470	95	92	0.18	N/A	N/A	N/A	N/A	N/A	629
**D*t*BuPhCzB**	3 wt% TCTA:PIM-TRZ (1:1)	513	93	33	0.10 [f]	512	0.25, 0.65	26.5	14.3	7.3	*627*
**BN1**	1 wt% mCBP	499	93	68.6	0.11	500	0.15, 0.61	17.0	16.6	8.5	638
**BN2**	1 wt% mCBP	538	89	108	0.13	546	0.38, 0.60	20.7	5.3	3.3	638
**BN3**	1 wt% mCBP	563	86	128	0.09	566	0.47, 0.51	21.4	5.3	N/A	638
**BN-1**	Toluene	566	95	N/A	0.11	N/A	N/A	N/A	N/A	N/A	639
**BN-8**	Toluene	567	95	N/A	0.03	N/A	N/A	N/A	N/A	N/A	639
**TCz-B**	1 wt% mCBP	517	89	71	0.09 [d]	515	0.16, 0.71	29.2	24.7	9.4	630
**DACz-B**	1 wt% mCBP	576	87	118	0.14 [d]	571	0.47, 0.51	19.6	12.0	4.8	630
**BN-2**	Toluene	586	96	N/A	0.08	N/A	N/A	N/A	N/A	N/A	639
**BN-3**	Toluene	598	96	N/A	0.08	N/A	N/A	N/A	N/A	N/A	639
**BN-4**	Toluene	612	96	N/A	0.06	N/A	N/A	N/A	N/A	N/A	639
**BN-5**	Toluene	627	95	N/A	0.05	N/A	N/A	N/A	N/A	N/A	639
**BN-6**	Toluene	618	94	N/A	0.06	N/A	N/A	N/A	N/A	N/A	639
**BN-7**	Toluene	629	94	N/A	0.09	N/A	N/A	N/A	N/A	N/A	639
**BN-9**	Toluene	585	96	N/A	0.03	N/A	N/A	N/A	N/A	N/A	639
**BN-10**	Toluene	595	96	N/A	0.12	N/A	N/A	N/A	N/A	N/A	639
**BN-11**	Toluene	608	95	N/A	0.09	N/A	N/A	N/A	N/A	N/A	639
**BN-12**	Toluene	624	94	N/A	0.07	N/A	N/A	N/A	N/A	N/A	639
**TRZCzPh-BNCz**	3 wt% CBP	516	98	5.7	0.13 [d]	513	0.17, 0.68	32.5	30.5	22.9	640
**TRZTPh-BNCz**	3 wt% CBP	516	99	6.4	0.11 [d]	513	0.16, 0.70	31.4	29.5	23.1	640
**CNCz-BNCz**	3 wt% CBP	582	96	60.4	0.18 [d]	584	0.55, 0.45	23.0	10.8	5.4	634
**BN-CP-1**	5 wt% DMIC-TRZ	490 [d]	98	65	0.12 [d]	496	0.09, 0.50	40.0	28.4	18.5	641
**BN-CP-2**	5 wt% DMIC-TRZ	490 [d]	95	58	0.13 [d]	497	0.10, 0.53	36.4	32.6	19.2	641
**S-Cz-BN**	5 wt% mCBP	490 [d]	94	42	0.16	490	0.10, 0.42	22.1	22.1	12.4	642
**D-Cz-BN**	5 wt% mCBP	490 [d]	96	44	0.14	489	0.10, 0.41	28.7	22.6	11.4	642
**TW-BN (TCzBN-TMPh)**	3 wt% mCBP	485	92	112	0.12	488	0.14, 0.36	27.8	25.4	10.7	643,644
**TPh-BN**	3 wt% mCBP	495	94	62	0.09	492	0.10, 0.46	28.9	25.1	15.6	644
**pCz-BN**	3 wt% mCBP	496	95	89	0.15	496	0.13, 0.54	27.2	25.6	12.2	644

**mCz-BN**	3 wt% mCBP	494	88	95	0.14	496	0.15, 0.55	25.9	24.1	14.0	644
**Tip-DtCzB**	1 wt% PhCzBCz	486	95	100	0.13	484	0.10, 0.29	28.9	18.2	6.8	637
***m*-PCz-BNCz**	10 wt% PhCzBCz	500	98	74.6	0.15 [d]	504	0.11, 0.61	36.8	33.4	19.0	645
***m*-DPAcP-BNCz**	10 wt% PhCzBCz	498	97	80.9	0.14 [d]	496	0.09, 0.54	42.0	37.6	17.5	645
***m*-BN-BNCz**	10 wt% PhCzBCz	496	95	97.0	0.16 [d]	492	0.09, 0.48	35.0	24.4	10.9	645
***m*-SF-BNCz**	10 wt% PhCzBCz	498	98	83.1	0.16 [d]	496	0.09, 0.53	41.1	37.0	17.9	645
**PCzBN1**	60 wt% mCP	430, 492	65	40.4	0.14	409, 428, 491	0.10, 0.43	17.8	8.6 [b]	2.6 [b]	646
**PCzBN3**	70 wt% mCP	430, 497	71	27.0	0.13	496	0.12, 0.54	17.5	11.4 [b]	3.1 [b]	646
**PCzBN5**	40 wt% mCP	430, 497	77	33.3	0.13	496	0.11, 0.53	13.3	8.4 [b]	3.1 [b]	646
**TCzBN-DPF**	5 wt% SF3TRZ	491 [d]	96	56	0.10	498	0.10, 0.56	26.4	22.8	12.0	643
**TCzBN-oPh**	1 wt% SF3TRZ	486 [d]	96	112	0.10	492	0.09, 0.46	26.0	22.9	10.4	643
**CzBNNa**	1 wt% mCBP	487	98	48	0.15 [d]	489	N/A	5.6 [d]	3.7 [d]	2.6 [d]	647
**BN-PhOH**	1 wt% mCBP	491	80 [d]	40.9	0.14 [d]	491	0.11, 0.43	19.0	15.5 [b]	6.2 [b]	648
**BN-PhOCH3**	3 wt% mCBP	493	78 [d]	40.8	0.15 [d]	493	0.10, 0.47	25.6	20.4 [b]	9.9 [b]	648
**BN-PhN(CH3)2**	3 wt% mCBP	492	71 [d]	21.2	0.14 [d]	492	0.10, 0.46	24.1	20.4 [b]	11.7 [b]	648
**(SIPr)AuBN**	0.5 wt% DMIC-Cz:DMIC-TRz	515	92	5.5	0.08	511	0.20, 0.69	24.8	24.8 [b]	24.3	487
**(IPr)AuBN**	0.5 wt% DMIC-Cz:DMIC-TRz	514 [i]	90 [i]	5.8 [i]	0.08 [i]	509	0.16, 0.66	24.0	24.0 [b]	20.2	487
**(BzIPr)AuBN**	2 wt% DMIC-Cz:DMIC-TRz	513 [i]	91 [i]	5.5 [i]	0.09 [i]	510	0.16. 0.68	30.3	30.3 [b]	28.1	487
**(PzIPr)AuBN**	1 wt% DMIC-Cz:DMIC-TRz	514 [i]	87 [i]	5.6 [i]	0.08 [i]	512	0.22, 0.67	24.0	24.0 [b]	23.1	487
**(PyIPr)AuBN**	4 wt% DMIC-Cz:DMIC-TRz	513 [i]	88 [i]	5.9 [i]	0.08 [i]	515	0.18, 0.69	27.6	27.6 [b]	20.5	487
**BN-DICz**	3 wt% mCBP	534	92	156	0.26 [d]	N/A	N/A	N/A	N/A	N/A	649
**BN-ICz-1 (BN-ICz)**	3 wt% mCBP	520	95	239	0.09 [d]	522	0.24, 0.73	24.1	18.2	10.6	650
**BN-ICz-2**	3 wt% mCBP	520	93	160	0.09 [d]	523	0.23, 0.72	22.2	18.3	14.4	650
**VTCzBN**	2,6-DCzppy	496 [d]	98	9.9	0.06 [d]	499	0.14, 0.56	31.7	24.8	19.8	651
**TCz-VTCzBN**	4 wt% 2,6-Dczppy	521 [b]	98	8.7	0.01 [d]	524	0.22, 0.71	32.2	18.0	16.0	651
**TCz-F-DABNA**	8 wt% PhCzBCz	558 [d]	99	20.2	0.12 [d]	588	0.54, 0.44	39.2	24.4	7.8	652

**BN1**	1 wt% DBFPO	454 [d]	91	126.6	0.20	457 [b]	0.14, 0.06	30.0	8.0	3.1	[653]
**BN2**	1 wt% DBFPO	464 [d]	93	74.6	0.16	467 [b]	0.12, 0.10	32.9	14.7	5.2	[653]
**CzB2-M/TB**	1 wt% PMMA	491	88	50	0.12	N/A	N/A	N/A	N/A	N/A	[609]
**Cz2B2-M/TB**	1 wt% PMMA	483	88	16	0.11	N/A	N/A	N/A	N/A	N/A	[609]
**CzB2-N/P**	1 wt% DOBNA-Tol	504 [c]	87 [c]	24 [c]	0.04 [c]	497	0.15, 0.57	26.7	24.4	18.0	[609]
**BBCz-DB**	2 wt% mCBP	471	91	35	0.15 [d]	469	0.12, 0.18	29.3	16.1 [b]	5.5 [b]	[628]
**BBCz-R**	2 wt% mCBP	619	79	53	0.19 [d]	616	0.67, 0.33	22.0	5.0 [b]	N/A	[628]
**R-BN**	4 wt% CBP	672 [j]	100 [j]	310 [j]	0.18 [d]	663	0.72, 0.28	25.6	N/A	N/A	[306]
**R-TBN**	4 wt% CBP	698 [j]	100 [j]	710 [j]	0.16 [d]	686	0.72, 0.28	24.7	N/A	N/A	[306]
**BNO1**	1 wt% DMIC-TRZ	610	96 [d]	N/A	0.25 [d]	609	0.63, 0.35	14.9	4.2	3.3	[654]
**BNO2**	1 wt% DMIC-TRZ	618	95 [d]	N/A	0.27 [d]	623	0.63, 0.32	12.0	5.1	4.4	[654]
**BNO3**	1 wt% DMIC-TRZ	624	96 [d]	N/A	0.26 [d]	616	0.62, 0.34	15.1	5.3	4.5	[654]
**DBNS**	CH_2Cl_2	631	80	11.2	0.20	N/A	N/A	N/A	N/A	N/A	[655]
**DBNS-*t*Bu**	CH_2Cl_2	641	85	10.2	0.19	N/A	N/A	N/A	N/A	N/A	[655]
**m-DiNBO**	3 wt% mCBP	466	94	31.4	0.06	466	0.13, 0.10	24.2	17.3	N/A	[656]
**p-DiNBO (DBON)**	4 wt% mCBP	503 [d]	98	24.6	0.13 [d]	510	0.17, 0.68	26.7	20.2	12.0	[656,657]
**DBSN**	4 wt% mCBP	553 [d]	98	25.7	0.13 [d]	556	0.42, 0.57	21.8	20.6	16.9	[657]
**DBN-ICz**	3 wt% mCBP	545	96	48	0.20 [d]	N/A	N/A	N/A	N/A	N/A	[650]
**mDBIC**	2 wt% mCP	431	68	202	0.31 [d]	433	0.16, 0.04	5.7	N/A	N/A	[658]
**BN-DMAC (DMAc-BN)**	1 wt% mCBP	461 [d]	63	13.9	0.14 [d]	502	0.14, 0.54	21.1	19.7	12.5	[659,660]
**BN-DPAC**	1 wt% mCBP	465 [d]	86	11.6	0.11 [d]	504	0.14, 0.56	28.1	27.7	19.2	[659]
**2PXZBN (PXZ-BN)**	1 wt% mCBP:PO-T2T (1:1)	515	84	25.3	0.19 [d]	522	0.28, 0.64	17.7	15.3	7.4	[660-662]
**2PTZBN**	1 wt% mCBP:PO-T2T (1:1)	519	80	16.2	0.15 [d]	528	0.28, 0.65	25.5	21.7	17.2	[661 662]
**BNSSe**	1 wt% DMIC-TRZ	520	99	12.7	0.12	515	0.22, 0.66	35.7	35.3 [b]	32.0	[662]
**BNSeSe**	1 wt% DMIC-TRZ	514	100	9.9	0.14	512	0.19, 0.66	36.8	36.6 [b]	34.0	[662]
**(P/M)-helicene-BN**	1 wt% DMIC-TRZ	520 [d]	98	71.8	0.15	523	0.26, 0.66	31.5	29.6	18.7	[305]
**BN4**	3 wt% mCPCN	522	96	25	0.20 [d]	510 [k] (512) [l]	0.16, 0.63 [k] (0.21, 0.64) [l]	20.6 [k] (19.0) [l]	20.5 [k] (16.6) [k]	10.7 [k] (10.1) [l]	[304]
**BN5**	1 wt% mCPCN	512	92	25	0.14 [c]	506 [k]	0.17, 0.59 [k] (0.17,	22.0 [k]	15.3 [k]	10.9 [k] (11.1) [l]	[304]

						(506) [l]	0.60) [l]	(26.5) [l]	(17.6) [l]		
**TPXZBN**	5 wt% mCBP	502 [d]	99	27	0.16 [d]	506	0.16, 0.65	21.3	19.6	17.4	663
**DPXZCZBN**	5 wt% mCBP	500 [d]	94	15	0.13 [d]	505	0.15, 0.64	19.2	18.8	17.2	663
**tCzphB-Ph**	2 wt% TPSS	527	80	372	0.04	527	0.21, 0.75	29.3	22.3 [b]	9.2 [b]	664
**tCzphB-Fl**	2 wt% TPSS	535	81	412	0.04	535	0.26, 0.72	26.2	21.6 [b]	8.7 [b]	664
**DPACzBN1**	3 wt% 26DczPPy	479	98	116	0.11	475	0.14, 0.30	23.6	19.3	9.6	665
**DPACzBN2**	3 wt% 26DczPPy	470	92	54	0.12 [d]	469	0.13, 0.16	24.0	23.5	14.3	665
**DPACzBN3**	3 wt% 26DczPPy	475	94	69	0.13 [d]	472	0.12, 0.18	27.7	19.3	6.7	665
**CzDABNA-NP-M/TB**	1 wt% PMMA	468	86	106	0.18	N/A	N/A	N/A	N/A	N/A	609
**CzDABNA-NP-TB/H**	1 wt% PMMA	465	82	93	0.18	N/A	N/A	N/A	N/A	N/A	609
**CzDABNA-NP**	1 wt% PMMA	461	80	77	0.18	N/A	N/A	N/A	N/A	N/A	609
**TBN-TPA (CzDABNA-NP-TB)**	4 wt% in 2,6-Dczppy	470 [d]	98 [d]	51.0	0.03 [d]	474	0.12, 0.19	32.1	27.4	13.9	609,666
**tCBNDA**	7 wt% DBFDPO	472	72	22.8	0.05	472	0.12, 0.17	20.2	12.4	7.1	667
**tCBNDASPO**	20% DBFDPO	472	92	9.1	0.04	472	0.12, 0.17	28.0	20.6	15.3	667
**tCBNDADPO**	30 wt% DBFDPO	472	99	N/A	0.04	472	0.14, 0.22	30.8	23.3	16.2	668
**PTZBN1**	2 wt% 2,6 Dczppy	497	98	33.5	0.16 [d]	496	0.16, 0.51	26.9	25.3	17.9	669
**PTZBN2**	2 wt% 2,6 Dczppy	487	95	22.4	0.15 [d]	483	0.13, 0.31	30.5	29.7	23.0	669
**PTZBN3**	2 wt% 2,6 Dczppy	473	98	26.5	0.17 [d]	476	0.13, 0.22	19.9	15.8	12.1	669
**B-dpa-Cz**	3 wt% mCBP	469	94	78	0.15 [e]	475	0.11, 0.19	20.1	15.3	4.7	670
**B-dpa-dmAc**	3 wt% mCBP	476	98	60	0.16 [e]	481	0.10, 0.28	24.2	22.3	12.7	670
**B-dpa-SpiroAc**	3 wt% mCBP	476	92	52	0.14 [e]	481	0.10, 0.27	25.1	24.2	15.3	670
**tDMAC-BN**	5 wt% DPEPO	468 [d]	90 [c]	64 [c]	0.15	476	0.12, 0.22	19.8	13.1	N/A	614
**BN-MeIAc**	1 wt% DMIC-TRZ	497	96	28.1	0.11 [e]	504 [k] 503 [l]	0.12, 0.63 [k] (0.12, 0.63 [l])	37.2 [k] (36.1 [l])	36.1 [k] (35.2 [l])	26.1 [k] (25.1 [l])	281
**B-O-dpa**	10 wt% DPEPO	433 [d]	86	224	0.18 [e]	443	0.15, 0.05	16.3	2.2	N/A	671
**B-O-Cz**	10 wt% DPEPO	441 [d]	94	51	0.15 [e]	481	0.13, 0.22	13.4	9.5	5.9	671
**B-O-dmAc**	10 wt% DPEPO	461 [d]	91	123	0.11 [e]	475	0.12, 0.21	16.2	13.5	8.4	671
**B-O-dpAc**	10 wt% DPEPO	463 [d]	94	83	0.06 [e]	473	0.12, 0.20	17.0	14.3	9.6	671
**CzBNO**	3 wt% 26DczPPy	450	96	48	0.21 [d]	454	0.14, 0.08	13.6	11.0	5.0	672
**DMAcBNO**	3 wt% 26DczPPy	470	99	129	0.23 [d]	472	0.13, 0.19	20.4	18.1	8.6	672
**DPAcBNO**	3 wt% 26DczPPy	468	98	100	0.19 [d]	468	0.13, 0.14	23.0	22.3	9.1	672
**BON-D0 (NBO)**	5 wt% mCP	464	85	157	0.18 [d]	460	0.14, 0.12	9.7	4.2 [b]	1.4 [b]	656,673

**BON-D1**	5 wt% mCP	485	94	139	0.14 [d]	488	0.13, 0.44	13.4	7.7 [b]	1.4 [b]	673
**BON-D2**	5 wt% mCP	478	98	107	0.10 [d]	487/ 509	0.15, 0.44	14.9	11.1 [b]	3.0 [b]	673
**SBON**	4 wt% mCBP	463 [d]	74	11.5	0.16 [d]	466	0.13, 0.13	13.7	12.6	6.7	657
**SBSN**	4 wt% mCBP	489 [d]	76	32.2	0.10 [d]	492	0.10, 0.44	17.6	17.6	12.0	657
**SOBN**	5 wt% 2,6-DczPPy	452	82	47	0.19 [d]	460	0.14, 0.14	14.6	10.2	7.2	289
***R*-DOBN**	5 wt% 2,6-DczPPy	455	91	52	0.14 [d]	459	0.14, 0.10	23.9	12.5	8.6	289
***R*-DOBNT**	5 wt% 2,6-DczPPy	458	96	122	0.12 [d]	464	0.13, 0.12	25.6	13.9	8.6	289
**CzBO**	1 wt% mCBP	448	99	120	0.16	448	0.15, 0.05	13.4	8.4	3.5	674
**CzBS**	1 wt% mCBP	472	98	30	0.14	473	0.11, 0.16	23.1	21.3	15.0	674
**CzBSe**	1 wt% mCBP	479	98	14	0.15	481	0.10, 0.24	23.9	23.4	20.0	674
**Cz-BseN**	5 wt% mCBP	479 [d]	87 [c]	29.8 [c]	0.15 [d]	490	0.13, 0.45	20.3	16.9	14.0 [b]	675
**DCz-BseN**	5 wt% mCBP	472 [d]	93 [c]	28.4 [c]	0.14 [d]	481	0.11, 0.25	22.3	19.6	12.6 [b]	675
**BNO**	1 wt% PMMA	457	97	600	0.17	N/A	N/A	N/A	N/A	N/A	487
**(BzIPr)AuBNO**	2 wt% PMMA	473	84	22.8	0.11	N/A	N/A	N/A	N/A	N/A	487
**BN1**	2 wt% mCBP	492	75	4.5	0.20 [m]	505	0.27, 0.49	5.5	N/A	4.1 [b]	676
**TCz-BN1**	2 wt% mCBP	491	71	3.0	0.16 [m]	505	0.25, 0.47	5.7	N/A	5.1 [b]	676
**BN2**	5 wt% mCBP	559	53	20.4	0.19 [m]	545	0.40, 0.57	6.7	N/A	6.5 [b]	676
**TCz-BN2**	5 wt% mCBP	560	62	15.1	0.17 [m]	556	0.41, 0.56	7.8	N/A	7.5 [b]	676
**ADBNA-Me-Mes (3)**	1 wt% in DOBNA-OAr	482	89	165	0.18	480	0.10, 0.27	16.2	11.2	N/A	677
**ADBNA-Me-Tip**	1 wt% in DOBNA-OAr	479	88	147	0.18	481	0.11, 0.29	21.4	15.4	N/A	677
**4a**	1 wt% PMMA	488	91	N/A	0.19	N/A	N/A	N/A	N/A	N/A	678
**4b**	1 wt% PMMA	491	93	N/A	0.19	N/A	N/A	N/A	N/A	N/A	678
**5a**	1 wt% PMMA	485	91	N/A	0.18	N/A	N/A	N/A	N/A	N/A	678
**5b**	1 wt% PMMA	487	86	N/A	0.17	N/A	N/A	N/A	N/A	N/A	678
**OAB-ABP**	2 wt% in Polymer B	506 [c]	90 [c]	32 [c]	0.12 [c]	505	0.12, 0.63	21.8	19.6	17.4	602
**BSBS-N1**	2 wt% mCBP	478	89	5.6	0.14	478	0.11, 0.12	21.0	16.3 [b]	8.7 [b]	679
**2B-DTACrs**	5 wt% mCBP	448	74	13.1	0.16	447	0.15, 0.04	14.8	8.9 [b]	N/A	680
**BN3**	1 wt% DBFPO	456 [d]	98	17.8	0.15	458 [b]	0.14, 0.08	34.8 [b]	16.7 [b]	5.7 [b]	653
**m[B-N]N1**	5 wt% mCBPC	483	91	128.1	0.15	490	0.13, 0.39	18.1	13.1 [b]	6.1 [b]	681
**m[B-N]N2**	5 wt% mCBP	491	90	136.2	0.13	494	0.14, 0.46	17.3	13.2 [b]	5.5 [b]	681
**BN2**	1 wt% DPEPO	415 [d]	76	16,240	0.28	N/A	N/A	N/A	N/A	N/A	682
**BN3**	1 wt%	420	77	13,3	0.29	N/A	N/A	N/A	N/A	N/A	682

	DPEPO	[d]		40							
**BN4**	1 wt% DPEPO	417 [d]	80	4,190	0.24	423	0.17, 0.04	9.1	N/A	N/A	682
**BIC-mCz**	2 wt% mCP	432	82	250	0.29 [d]	431	0.16, 0.04	7.0	0.2 [d]	N/A	658
**BIC-pCz**	2 wt% mCP	471	96	328	0.15 [d]	470	0.13, 0.16	13.3	3.1 [d]	1.2 [d]	658
**QAO/QAD/DiKTa**	5 wt% mCP	466	72	93.3	0.19	468	0.13, 0.18	19.4	9.4	1.4	683-685
**3Ph-QAD**	2 wt% mCP	478	73	250	0.18	480	0.13, 0.32	19.4	10.4	3.1	683
**7Ph-QAD**	2 wt% mCP	472	68	474	0.19	472	0.12, 0.24	19.1	5.4	2.1	683
**Mes$_3$DiKTa**	3.5 wt% mCP	477	80	20	0.21	480	0.12, 0.32	21.1	14.5	4.5	684
**QA-PF**	3 wt% mCP	478	89	346.9	0.24	474	0.12, 0.17	16.8	5.8	2.1 [b]	686
**QA-PCN**	3 wt% mCP	477	68	223.9	0.18	473	0.12, 0.18	16.9	9.4	2.7 [b]	686
**QA-PMO**	3 wt% mCP	485	66	484.1	0.25	484	0.11, 0.30	15.0	3.5	2.1 [b]	686
**QA-PCZ**	3 wt% mCP	480	71	339.1	0.21	482	0.11, 0.28	17.5	7.5	2.4 [b]	686
**QAO-PhCz**	5 wt% mCBP	460 [d]	47	40	0.11 [d]	470	0.13, 0.18	12.4	8.6 [b]	1.9 [b]	300
**QAD-Cz (Cz-DiKTa)**	1 wt% mCP	500	100	205	0.17	494	0.16, 0.47	20.3	5.4	0.7	687,688
**QAD-2Cz**	12 wt% mCP	526	100	130	0.17	530	0.30, 0.65	27.3	23.9	12.4	687
**QAD-mTDPA**	1.5 wt% CBP	587	97	269	0.33	589	0.55, 0.44	26.3	12.9	4.7	687
**Cz-Ph-DiKTa**	2 wt% mCP	486	77	153	0.10	492	0.18, 0.50	23.0	19.3	10.2	688
**3Cz-DiKTa**	2 wt% mCP	539	78	286	0.16	547	0.39, 0.60	24.4	17.3	6.0	688
**3TPA-DiKTa**	2 wt% mCP	551	93	131	0.13	551	0.41, 0.58	30.8	18.1	7.3	689
**3DPA-DiKTa**	2 wt% mCP	617	60	323	0.20	613	0.63, 0.37	16.7	3.4	1.9	689
**QAOCz1**	5 wt% CBP	501	86	116.7	0.26 [d]	516	0.23, 0.66	16.9	4.4 [b]	2.9 [b]	690
**QAOCz2**	5 wt% CBP	500	87	16.3	0.18 [d]	504	0.18, 0.62	19.4	7.5 [b]	2.6 [b]	690
**QAOCz3**	5 wt% CBP	495	99	26.5	0.16 [d]	500	0.16, 0.57	21.1	9.9 [b]	2.7 [b]	690
**DiKTa-OBuIm**	1 wt% mCP	500	71	316.6	0.20	N/A	N/A	N/A	N/A	N/A	691
**DiKTa-DPA-OBuIm**	1 wt% mCP	578	61	241.7	0.19	N/A	N/A	N/A	N/A	N/A	691
**DDiKTa**	9 wt% DPEPO	490	72	1.2	0.16	500	0.18, 0.53	19.0	10.3 [b]	N/A	692
**DQAO**	8 wt% mCP	472	59	111	0.19 [d]	472	0.12, 0.18	15.2	8.5	2.3 [b]	693
**OQAO**	5 wt% CBP	534	90	205	0.16 [d]	532	0.32, 0.65	20.3	15.1	3.4 [b]	693
**SQAO**	1 wt% mCPCN	560	65	78	0.16 [d]	564	0.47, 0.52	17.8	13.6	6.3 [b]	693
**QA-1**	3 wt% PPCz	457	94	655	0.29	455	0.14, 0.12	17.1	1.2	N/A	694
**QA-2**	3 wt% PPCz	465	97	48	0.19	463	0.13, 0.14	19.0	11.0	2.6 [b]	694
**QA-3**	3 wt% PPCz	523	99	307	0.19	515	0.26, 0.62	18.6	11.1	1.8 [b]	694
**Hel-DiDiKTa**	1 wt% mCP	480	4.1	5.4	0.15	N/A	N/A	N/A	N/A	N/A	695

**BTPT**	1 wt% PMMA	401	9	109	0.14	N/A	N/A	N/A	N/A	N/A	303
**2,3-CZ**	3.5 wt% mCBP	449 [d]	40	436	0.26 [d]	458	0.15, 0.14	6.3	3.7 [b]	1.6 [b]	212
**2,5-CZ**	3.5 wt% mCBP	459 [d]	81	619	0.29 [d]	463	0.13, 0.13	22.3	5.0 [b]	3.9 [b]	212
**2,3-DPA**	10 wt% 26DCzPPY	496 [d]	51 [n]	373	0.19 [d]	503	0.17, 0.54	11.7	7.2 [b]	3.2 [b]	212
**CzAO**	3 wt% mCPCN	431 [d]	49	1340	0.40 [d]	444	0.15, 0.07	8.6	N/A	N/A	696
**MQAO**	3 wt% mCPCN	447 [d]	61	1024	0.31 [d]	460	0.14, 0.14	10.3	N/A	N/A	696
**QPXO**	3 wt% mCPCN	485 [d]	35	2394	0.29 [d]	486	0.17, 0.41	7.1	N/A	N/A	696
**QPO**	3 wt% mCPCN	501 [d]	71	1436	0.26 [d]	510	0.24, 0.53	15.3	N/A	N/A	696
**TOAT**	Toluene	417 [b]	N/A	N/A	0.34	N/A	N/A	N/A	N/A	N/A	697
**3DPA-TOAT**	3 wt% mCBP	590 [b]	46	2142	0.34 [d]	588 [b]	0.57, 0.43	1.2 [b]	0.4 [b]	0.2 [b]	697
**3*t*BuTOAT**	15 wt% SiCz	484	N/A	N/A	0.29	484	0.11, 0.26	13.0	N/A	N/A	698
**3PhTOAT**	15 wt% mCP	516	97	N/A	0.12	500	0.16, 0.54	29.2	N/A	N/A	698
**3PTPTOAT**	15 wt% mCP	520	93	N/A	0.16	516	0.24, 0.61	27.6	N/A	N/A	698
**3MTPTOAT**	15 wt% mCP	502	92	N/A	0.14	516	0.21, 0.62	31.2	N/A	N/A	698
**3MPATOAT**	15 wt% CBP	654	N/A	N/A	0.28	652	0.69, 0.31	11.3	N/A	N/A	698
**tBisICz**	1 wt% mCP:TSPO1	442	95	12500	0.29 [o]	445	0.16, 0.05	15.1	3.0 [b]	N/A	699
**tPBisICz**	1 wt% mCP:TSPO1	450	91	1740	0.27 [o]	452	0.15, 0.05	23.1	4.8 [b]	N/A	699
**DiICzMes$_4$**	10 wt% DPEPO	451 [p]	82 [p]	433 [p]	0.26 [p]	446	0.15, 0.11	3.0	1.9	N/A	700
**t3IDCz**	1 wt% mCP:mCBP-CN	470	92	376.6	0.21 [e]	472	0.12, 0.16	30.0	12.4	5.0	701
**P3IDCz**	1 wt% mCP:mCBP-CN	470	100	332.6	0.19 [e]	472	0.12, 0.16	30.9	12.6	4.7	701

[a] Medium is that of photoluminescence and electroluminescent emissive layer unless otherwise stated, [b] fitted using graphical fitting software, [c] 1 wt% doped PMMA film, [d] dilute toluene solution, [e] dilute THF solution, [f] 1 wt% doped mCP film, [g] *R* isomer, [h] *S* isomer, [i] 2 wt% doped PMMA film, [j] 3 wt% doped CBP film, [k] +ve enantiomer, [l] -ve enantiomer, [m] 5 wt% doped PMMA film, [n] 3.5 wt% doped mCBP film, [o] dilute DCM solution, [p] 3.5 wt% doped mCP film.

Table **S16.** Photoluminescence and electroluminescence properties of all reported TSCT-TADF emitters reviewed in Section **12**.

**Compound**	**Medium**	**λ_{PL} / nm**	**Φ_{PL} / %**	**τ_D / µs**	**ΔE_{ST} / eV**	**λ_{EL} / nm**	**CIE_{XY}**	**EQE_{MAX} / %**	**Roll-off 100 cd m^{-2} /**	**Roll-off 1000 cd m^{-2} / %**	**Ref**

									%		
**XPT**	10 wt% DPEPO	566	66	3.3	N/A	585	N/A	10	~14	~44	702
**XtBuCT**	10 wt% DPEPO	453	53	4.1	N/A	488	N/A	4	N/A	N/A	702
**TpAT-tFFO**	N/A	485	84%	4.42	N/A	498	0.20,0.44	19.2	1%	6%	703
**TpIBT-tFFO**	9% CzSi	477	71.4	6.7	0.076*	472	0.16,0.26	12.2	N/A	N/A	704
**DM-B**	20wt% DPEPO	493	96	5	0.17*	488	N/A	27.4	N/A	10.9	705
**DM-Bm**	20wt% DPEPO	495	92	4.5	–0.08	500	N/A	21.7	N/A	9.2	705
**DM-G**	20wt% DPEPO	504	88	3.3	-0.11	500	N/A	18.5	N/A	16.8	705
**8MeDM-B**	30 wt% PPF	480 [a]	97	4.6	0.15 [a]	492	0.20, 0.41	28.8	6	18	706
**8FDM-B**	30 wt% PPF	483 [a]	98	4.0	0.15 [a]	496	0.22, 0.43	31.7	5	18	706
**tBu-DMB**	30 wt% mCP	446	89	5.99	0.17	492	0.21, 0.38	21.0	23	0.6	707
**C6-DMB**	30 wt% mCP	447	98	5.58	0.17	492	0.21, 0.38	21.7	24	63	707
**STF-DPS**	30 wt% CBP	441	16	6.78	0.052	468	N/A	3.0	N/A	N/A	708
**STF-TBDS**	30 wt% CBP	460	53	7.98	0.053	488	N/A	21.5	N/A	N/A	708
**N2-6**	20 wt% CzAcS	461	76	1.01	0.27	NA	NA	14.2	NA	NA	709
**N2-8**	20 wt% CzAcS	470	82	1.18	0.16	NA	NA	17.6	NA	NA	709
**N3-6**	20 wt% CzAcS	485	83	1.29	0.28	NA	NA	14.7	NA	NA	709
**N3-8**	20 wt% CzAcS	495	91	1.50	0.14	NA	NA	18.9	NA	NA	709
**DM-BD1**	20wt% DPEPO	495	94.2	3.1	0	N/A	0.21,0.47	28.0	N/A	N/A	708
**DM-BD2**	20wt% DPEPO	495	92.8	2.8	-0.07	N/A	0.20,0.46	26.6	N/A	N/A	708
**SFST**	30 wt% mCBP	512	53.1	6.78	0.052	508	N/A	12.5	N/A	N/A	710
**SFOT**	30 wt% mCBP	512	89.7	7.98	0.053	508	N/A	23.1	N/A	N/A	710
**SDMAC**	30 wt% PPF	NA	90	4.17	0.034	492	0.18, 0.41	28.4	14	40	711
**2tDMG**	40wt% DPEPO	502	87	3.43	0.03	504	0.24,0.53	30.8	N/A	7.5	712
**3tDMG**	40wt% DPEPO	505	86	2.28	0.01*	518	0.29,0.57	26.3	N/A	11.8	712
**AC-BO**	10 wt% PMMA	446	77	11.7	0.13	456	0.15, 0.12	19.3	60	NA	713
**QAC-BO**	10 wt% PMMA	428	83	0.11	0.20	448	0.15, 0.08	15.8	70	NA	713
**Cz-BO**	10 wt% PMMA	411	89	NA	0.29	412	0.16, 0.03	5.5	NA	NA	713
**mCz-Xo-TRZ**	30 wt% PPF	469	90	7.2	0.16	477	0.16,0.29	27.8	1	14	714
**dCz-Xo-TRZ**	30 wt% PPF	482	92	7.5	0.24	464	0.15,0.20	21.0	4	19	714
**2PXZ-2TRZ**	30 wt% PPF	512	94	5.3	0.01	508	0.26,0.54	27.1	NA	NA	715
**H1**	30 wt% DPEPO	493	92	6.8	0.02	494	0.20, 0.42	20.9	13	34	716
**H2**	30 wt% DPEPO	520	80	10.9	0.07	527	0.31, 0.56	16.1	1	16	716
**H3**	30 wt%	497	82	5.9	0.01	503	0.22, 0.48	17.7	2	25	716

	DPEPO										
**H4**	30 wt% DPEPO	504	89	5.3	0.03	507	0.24, 0.50	20.0	5	22	716
**H5**	30 wt% DPEPO	220	52	3.3	0.03	550	0.42, 0.55	13.2	1	11	716
**PXZ-CTZ**	20wt% DPEPO	525	55	3.41	0.07	N/A	0.33,0.56	16.57	N/A	40.1	717
**DPXZ-CTZ**	20wt% DPEPO	524	78	3.38	-0.03	N/A	0.39,0.57	19.7	N/A	53.0	717
**DPXZ-BO**	20wt% DPEPO	522	99	11.3	0.03	N/A	0.26,0.58	23.96	N/A	15.6	717
**BNB-m**	10 wt% mCP	502	100	11.2	0.03	502	0.21, 0.50	31.4	NA	30	718
**BNB-p**	10 wt% mCP	518	86	25.4	0.11	508	0.23, 0.54	34.9	NA	21	718
**TPA-QX**	5 wt% mCP	535	44	NA	0.38	NA	NA	NA	NA	NA	719
**PXZ-QX**	5 wt% mCP	573	32	NA	0.24	NA	NA	NA	NA	NA	719
**DPXZ-QX**	5 wt% mCP	582	74	26.9	0.09	597	0.52, 0.44	20.6	NA	74	719
**DPXZ-DFQX**	5 wt% mCP	595	71	6.8	0.01	NA	NA	NA	NA	NA	719
**DPXZ-2QX**	5 wt% mCP	594	87	8.7	0.02	609	0.59, 0.41	23.2	NA	0.38	719
**DPXZ-2DFQX**	5 wt% mCP	599	91	4.9	–0.05	NA	NA	NA	NA	NA	719
**DPTZ-QX**	5 wt% mCP	565	49	255	0.14	NA	NA	NA	NA	NA	720
**DPTZ-DFQX**	5 wt% mCP	561	61	114	0.15	NA	NA	NA	NA	NA	720
**AcPTC**	20 wt% SimCP2	485	73	10.5	0.05	484	0.18, 0.26	10.0	NA	NA	721
**PxPTC**	20 wt% SimCP2	522	61	2.9	0.03	533	NA	11.0	NA	NA	721
**PtPTC**	20 wt% SimCP2	532	51	11.4	0.03	564	NA	5.6	NA	NA	721
**BPAPTC**	20 wt% SimCP2	519	90	4.6	0.02	520	NA	23.3	12	36	722
**mBPAPTC**	20 wt% SimCP2	510	90	3.1	0.03	520	NA	17.8	26	47	722
**BPAMTC**	20 wt% SimCP2	492	63	6.0	0.06	486, 516	NA	14.7	40	74	722
**mBPAMTC**	20 wt% SimCP2	491	69	3.2	0.13	484, 520	NA	9.5	44	69	722
**MPAPTC**	20 wt% SimCP2	546	44	1.8	0.002	564	NA	9.1	32	56	722
**B-oCz**	Neat	465	61	15	0.06	463	0.15,0.17	8.0	14	68	723
**B-oTC**	Neat	476	94	14	0.05	474	0.15,0.26	19.1	11	49	723
**DMACoBA**	10wt% PMMA	488	100	10.1	0.014	N/A	N/A	N/A	N/A	N/A	724
**DMACoOB**	10wt% PMMA	481	100	10.8	0.004	N/A	N/A	N/A	N/A	N/A	724
**DMACoB**	10wt% PMMA	518	100	8.5	0.001	N/A	N/A	N/A	N/A	N/A	724
**CTPCF3**	Toluene	494	96	3.21	0.04	495	NA	33.1	6	18	725
**CNCTPCF3**	Toluene	475	67	6.04	0.28	497	NA	25.6	12	46	725
**TCTPCF3**	Toluene	468	65	2.52	0.06	495	NA	23.2	12	19	725
**1CTF**	Toluene	500	99	2.4	0.03	490	0.23, 0.45	17.5	NA	4	726
**2CTF**	Toluene	507	98	1.8	0.03	503	0.26, 0.54	19.8	NA	1	726
**3CTF**	Toluene	514	99	1.2	0.04	508	0.29, 0.57	22.6	NA	0	726
**SF12oTz**	20 wt% DPEPO	491	92	4.6	0.29	496	0.23, 0.47	22.4	1	11	727
**SF23oTz**	20 wt%	473	86	4.3	0.08	484	0.19, 0.35	19.6	4	19	727

	DPEPO										
**SF34oTz**	20 wt% DPEPO	479	65	8.2	0.05	482	0.18, 0.37	14.6	42	79	727
**TP-BP-DMAC**	20 wt% DPEPO	508	80	8.3	0.01	488	0.21, 0.38	20.5	20	53	728
**TP-BP-PXZ**	20 wt% DPEPO	531	40	3.9	0.01	531	0.35, 0.53	13.8	11	33	728
**Ac3TRZ3**	10 wt% Ac6	486*	54	3.5	0.08	520	0.22,0.42	11.0	5	40	729
**TAc3TRZ3**	10 wt% Ac6	508*	63	3.2	0.04	538	0.25,0.47	14.2	5	27	729
**S-CNDF-S-*t*Cz**	Neat	445	42	1.83	0.24	484	0.16,0.18	2.6	N/A	N/A	89
**S-CNDF-D-*t*Cz**	Neat	441	19	2.20	0.21	466	0.16,0.17	3.7	N/A	N/A	89
**T-CNDF-T-*t*Cz**	Neat	447	76	1.65	0.03	466	0.19,0.35	21.0	N/A	N/A	89
**TRZ-*o*-SDMAC**	Neat film	496	13	1.3	0.01	518	0.30, 0.47	1.1	83.6	NA	730
**TRZ-*m*-SDMAC**	Neat film	499	52	5.2	0.01	510	0.24,0.49	20.3	24.9	79	730
**TPA-QNX(CN)2**	cyclohexane	487	44	2.4	N/A	573	0.45,0.54	9.4	N/A	N/A	731
***t*BuDMAC-TPE-TRZ**	Neat film	510	44	4.64	0.08	524	0.29, 0.52	5.0	14	NA	732
***t*BuDMAC-TPE-TTR**	Neat film	550	11	9.28	0.01	560	0.43, 0.53	7.9	41	NA	732
**Cis-Bz-PCP-TPA**	15 wt% mCP	480	12	1.8	0.13	N/A	N/A	N/A	N/A	N/A	733
**Trans-Bz-PCP-TPA**	15 wt% mCP	465	15	3.6	0.17	N/A	N/A	N/A	N/A	N/A	733
**Cp-ecl**	Toluene	520	61	1.9	0.03***	N/A	N/A	N/A	N/A	N/A	734
**Cp-sta**	Toluene	520	2		0.05***	N/A	N/A	N/A	N/A	N/A	734

[a] Dilute toluene solution.

Table **S17.** Photoluminescence and electroluminescence properties of AIE TADF emitters reviewed in Section **13**.

**Compound**	**Medium**	**λ_{PL} / nm**	**Φ_{PL} / %**	**τ_d / µs**	**ΔE_{ST} / eV**	**λ_{EL} / nm**	**CIE_{XY}**	**EQE_{max} / %**	**Roll-off at 100 cd m^{-2} / %**	**Roll-off at 1000 cd m^{-2} %**	**Ref**
**TXO-TPA**	5 wt% in mCP	580	83	78	0.04 [a]	552	0.45, 0.53	18.5	~35	68	735
**TXO-PhCz**	5 wt% in mCP	520	90	86	0.09 [a]	~517	0.31, 0.56	21.5	~44	72	735
***m*-ACSO2**	Neat	473	76	3.2	0.07 [b]	486	0.21, 0.34	17.2	6	~65	736
***o*-ACSO2**	Neat	484	66	1.8	0.04 [b]	492	0.23, 0.40	5.9	2	N/A	736
**4,4-CzSPz**	Neat	530	97	62	0.25 [b]	526		20.7	~0	50	737
**2Cz-DPS**	Neat	520	91.9	19.1	0.32 [b]	518	NA	28.7	NA	NA	738
**3CP-DPS-PXZ**	Neat	518	52	0.96	0.03 [b]	508	0.28, 0.52	17.9	NA	19	739
**3CP-DPS-DMAC**	neat	472	65	2.36	0.07 [b]	484	0.16, 0.28	9.1	NA	69	740
**DCB-DPS-PXZ**	Neat	537	40	0.62	0.08 [b]	520	0.32, 0.56	13.9	NA	20.8	740
**mCP-DPS-PXZ**	Neat	537	47	0.67	0.05 [b]	520	0.32, 0.56	14.7	NA	17.7	740
***m*PhDCzDPSPXZ**	Neat	548	56	0.76	0.03 [b]	521	0.34, 0.56	18.1	NA	7.7	740
**pPhDCzDPSPX**	Neat	548	55	0.75	0.03 [b]	523	0.34, 0.56	17.1	NA	9.9	740

**Z**											
**PXZ2PTO**	Neat	512	61	2.49	0.02 [a]	504	0.27, 0.50	16.4	7.9	NA	741
**DBT-BZ-DMAC**	Neat	505	80	3.0	0.08 [b]	508	0.26, 0.55	14.2	N/A	0.46	742
**CDBP-BP-PXZ**	Neat	523	77.4	2.5	0.039 [b]	536	0.355, 0.579	15.5	N/A	0.6	743
**CDBP-BP-DMAC**	Neat	488	59.2	6.1	0.043 [b]	496	0.229, 0.445	9.5	N/A	2.1	743
**CP-BP-PXZ**	Neat	530	58	2.1	0.024 [b]	548	0.40, 0.57	18.4	N/A	1.2	744
**CP-BP-PTZ**	Neat	538	45.3	5.7	0.033 [b]	554	0.42, 0.55	15.3	N/A	16.7	744
**CP-BP-DMAC**	Neat	490	67.4	5.5	0.016 [b]	502	0.23, 0.49	15.0	N/A	0.2	744
**DMF-BP-PXZ**	Neat	595	45	1.4	0.22 [c]	NA	0.44, 0.54	13.3	NA	6%	745
**DPF-BP-PXZ**	Neat	596	49	1.1	NA	560	0.46, 0.53	14.3	NA	1.4%	745
**SBF-BP-PXZ**	Neat	596	45	1.1	NA	NA	0.45, 0.53	12.3	NA	0.8%	745
**DCDMF-BP-PXZ-BP-PXZ**	Neat	540	88.5	0.33	0.05 [b]	540	0.39, 0.57	19.0	N/A	2.6	746
**DCDPF-BP-PXZ**	Neat	530	89.0	0.30	0.03 [b]	544	0.38, 0.57	18.5	N/A	1.6	746
**DCSBF-BP-PXZ**	Neat	527	39.6	0.1	0.09 [b]	548	0.40, 0.56	3.3	N/A	3.0	746
**35DCPP-BP-PXZ**	Neat	530	66.5	1.58	0.02 [b]	538	0.39, 0.57	17.3	NA	0.6	747
**26DCPP-BP-PXZ**	Neat	533	67.9	1.68	0.01 [b]	542	0.38, 0.57	16.1	NA	1.2	747
**3-CCP-BP-PXZ**	Neat	541	73	0.76	0.016 [b]	540	0.38, 0.58	21.7	NA	19.8	748
**9-CCP-BP-PXZ**	Neat	543	70.4	0.68	0.018 [b]	537	0.37, 0.59	20.4	NA	19.4	748
**3,9-CCP-BP-PXZ**	Neat	536	72.6	0.42	0.019 [b]	541	0.38, 0.59	20.6	NA	19.7	748
**3-BCP-BP-PXZ**	Neat	521	61	1.63	0.028 [b]	544	0.374, 0.575	19.5	NA	6.1	749
**9-BCP-BP-PXZ**	Neat	531	53.4	1.54	0.034 [b]	540	0.365, 0.577	14.3	NA	3.5	749
**3,9-BCP-BP-PXZ**	Neat	540	50.7	1.47	0.033 [b]	544	0.382, 0.573	16.4	NA	6.1	749
**DCB-BP-PXZ**	Neat	530	69	2.6	0.02 [b]	N/A	0.39, 0.57	22.6	N/A	3	750
**CBP-BP-PXZ**	Neat	532	72	2.4	0.02 [b]	N/A	0.39, 0.57	21.4	N/A	3	750
**mCP-BP-PXZ**	Neat	529	66	2.3	0.02 [b]	N/A	0.39, 0.57	22.1	N/A	3	750
**mCBP-BP-PXZ**	Neat	530	71	2.4	0.02 [b]	N/A	0.38, 0.57	21.8	N/A	1	750
**SFAC-BP-SFAC**	Neat	508	52	3.6	0.036 [b]	508	0.24, 0.53	18.6	N/A	13.4	750
**SXAC-BP-SXAC**	Neat	500	57	4.7	0.061 [b]	504	0.21, 0.46	17.1	N/A	15.8	750
**STAC-BP-STAC**	Neat	511	58	4.0	0.052 [b]	504	0.22, 0.49	18.1	N/A	12.2	750
**TATC-BP**	Neat	524	22	0.94	0.125 [b]	549	0.41, 0.54	5.9	NA	18.6	751
**TATP-BP**	Neat	520	24	0.91	0.129 [b]	541	0.38, 0.55	6	NA	3.3	751
**BDMAC-XT**	Neat	518	96	1.4	0.025 [b]	526	0.31, 0.61	21	N/A	0	752
**BDPAC-XT**	Neat	495	94	1.9	0.024 [b]	496	0.22, 0.49	21	N/A	14.3	752
**XT-DPDBA**	Neat	472	77	2.823	0.063 [b]	472	0.151,0.272	8.9	NA	10	753
**XT-BDPDBA**	Neat	480	86	2.403	0.025 [b]	488	0.185, 0.390	13.1	NA	16	753
**SPBP-DPAC**	Neat	495	93	2.5	0.03 [b]	504	0.23, 0.50	22.8	NA	1.8	754
**SPBP-SPAC**	Neat	504	98	1.3	0.02 [b]	506	0.27, 0.56	21.3	NA	2.3	754
**3CPyM-DMAC**	Neat	514	67	1.42	0.04 [b]	532	0.34, 0.51	11.4	NA	NA	755
**2CPyM-DMAC**	Neat	536	53.3	1.10	0.03 [b]	544	0.39, 0.56	9.1	NA	NA	755
**CBM-DMAC**	Neat	501	47	0.99	0.1 [b]	499	0.28, 0.47	6.7	NA	NA	755
**DMAC-BPI**	Neat	510	95.8	3.1	0.02 [b]	511	0.25, 0.51	19.4	NA	1.0	756
**DPAC-BPI-CN**	Neat	525	90.1	3	0.07 [b]	531	0.34, 0.56	26.2	N/A	44.3	756
**DTPA-DTM**	Neat	498	39	1.6	0.18 [b]	494	0.21, 0.42	4.4	NA	NA	757
**DTPA-DDTM**	Neat	539	60	2.4	0.17 [b]	555	0.41, 0.55	8.2	NA	NA	757
**CzTAZPO**	Neat	512	71	1.1	0.08 [b]	537	0.37, 0.56	12.8	NA	12.6	758
**sCzTAZPO**	Neat	502	57	0.81	0.10 [b]	531	0.36, 0.56	9.6	NA	9.1	758
**IAcTr-out**	25 wt% mCP	~505	77	7.0	0.05 [b]	NA	0.29, 0.54	17.5	4	20	759

**IAcTr-in**	35 wt% mCP	~516	80	2.8	0.07 ^{b}	NA	0.33, 0.58	18.4	1	12	759
**34AcCz-PM**	Neat	538	67	0.73	0.15 ^{d}	548	0.42, 0.55	14.1	N/A	9.2	760
**34AcCz-Trz**	Neat	556	42	0.88	0.12 ^{e}	576	0.50, 0.49	7.3	N/A	19.2	760
**PCZ-CB-TRZ**	Neat	557	97	27	0.003 ^{f}	586	N/A	11.0	N/A	N/A	761
**TPA-CB-TRZ**	Neat	624	55	–	0.146 ^{f}	631	N/A	10.1	N/A	N/A	761
**2PCZ-CB**	Neat	571	94	28	0.018 ^{f}	590	N/A	9.2	N/A	N/A	761
**SFDBQPXZ**	10 wt% CBP	546	100	1.5	0.04 ^{b}	548	N/A	23.5	28	45	762
**DFDBQPXZ**	10 wt% CBP	551	88	2.0	0.04 ^{b}	548	N/A	16.8	2	26	762
**DMAC-QL**	Neat	489	33	2.15	0.06 ^{b}	522	0.31, 0.51	7.7	23	58	763
**PXZ-QL**	Neat	531	65	1.86	0.10 ^{b}	536	0.36, 0.55	17.3	6.9	12	763
**PTZ-QL**	Neat	537	52	15.76	0.04 ^{b}	546	0.39, 0.56	14.8	14	64	763
**Fene**	Neat	584	58.24	2.75	0.04 ^{b}	570	N/A	14.9	N/A	N/A	764
**Fens**	Neat	591	36.11	3.27	0.03 ^{b}	568	N/A	13.1	N/A	N/A	764
**Yad**	Neat	544	79.63	16.05	0.04 ^{b}	534	N/A	17.4	N/A	N/A	764
**TB-tCz**	Neat	433	41.4	1.49	0.227 ^{g}	416	0.17, 0.06	8.21	N/A	N/A	765
**TB-tPCz**	Neat	445	51.9	1.06	0.169 ^{g}	428	0.16, 0.05	15.8	N/A	N/A	765

^{a} 2-MeTHF, ^{b} Neat, $^{c}\Delta E_{S2\text{-}T3}$ calculated computationally on solid, ^{d} 10 wt% in CBP, ^{e} 5 wt% in CBP, ^{f} calculated at the TD-DFT-PBEO/6-31G(d) level, ^{g} toluene.

Table **S18.** Properties of ESIPT TADF emitters and their corresponding non-ESIPT TADF emitters reviewed in Section **14**.

**Compound**	**Medium**	**λ_{PL} / nm ^{a}**	**Φ_{PL} / % ^{b}**	**τ_D / µs ^{b}**	**ΔE_{ST} / eV ^{b}**	**λ_{EL} / nm**	**CIE$_{XY}$**	**EQE$_{max}$/ %**	**Roll-off 100 cd m^{-2} / %^{e}**	**Roll-off 1000 cd m^{-2} / % ^{e}**	**Ref**
**HPI-Ac**	$CHCl_3$	465	22	25	0.08	N/A	N/A	N/A	N/A	N/A	766
**TQB**	10 wt% in CzSi	516	55	106	N/A	518 ^{c}	N/A	14.2	N/A	N/A	767
**PXZPDO**	1 wt% CBP	594	68	1.3	0.04	560 ^{c}	0.44,0.53	18.8	6	23	768
**PXZDMePDO**	1 wt% CBP	562	54	1.5	0.07	544 ^{c}	0.38,0.56	12.2	7	20	768
**DMACPDO**	6 wt% CBP	533	86	1.9	0.11	536 ^{c}	0.36,0.57	23.9	12	35	768
**DMACDMePDO**	6 wt% CBP	506	64	1.8	0.16	518 ^{c}	0.28,0.53	14.6	18	43	768
**PXZPDO**	1 wt% CBP	604	58	1.3	0.04	570 ^{d}	0.47, 0.50	20.1	N/A	8	769
**TPXZBM**	1 wt% CBP	650	30	1.4	0.02	582 ^{d}	0.48, 0.49	12.7	N/A	22	769
**BPXZBM**	1 wt% CBP	626	17	1.01	0.005	598 ^{d}	0.50, 0.46	7.05	N/A	43	769
**BrA-HBI**	1 wt% PMMA	450	31	1900	0.31	–	-	-	-	-	770

^{a} measured in solution (DCM and toluene) at room temperature. ^{b} in doped thin films except HPI-Ac compound. ^{c} OLEDs fabricated by thermally evaporation process. ^{d} OLEDs fabricated by solutionprocessing. ^{e} EQE dropped values calculated by using equation $EQE_{dropped\ values} = (EQE_{max} - EQE_{100\ or\ 1000\ cd\ /m^2})/(EQE_{max}) \times 100$.

Table **S19.** Photophysical properties of TADF MCL emitters in their different macromolecular forms, reviewed in Section **15**.

Emitter	Packing Name[a]	λ_{PL} / nm	Φ_{PL}/ %	ΔE_{ST} / eV	τ_d / µs	Mechanism[b]	Ref.
**OPC**	Crystal	456, 554	23	0.56, 0.01 [c]	N/A	Fl, TADF	771
	Amorphous	554	N/A	0.01[c]	N/A	TADF	771
**OPNa**	Amorphous	574 [e]	N/A	N/A	N/A	N/A	772
	Crystalline	429 [e], 570 [e]	N/A	N/A	N/A	N/A	772
**OPAn**	Amorphous	449, 570 [e]	N/A	N/A	N/A	N/A	772
	Crystalline	443, 587 [e]	N/A	N/A	N/A	N/A	772
**OPPy**	Amorphous	459, 573 [e]	N/A	N/A	N/A	N/A	772
	Crystalline	458[e], 570 [e]	N/A	N/A	N/A	N/A	772
**PTZ-AQ**	Y-Solid	545	72	0.11	14.7 (275) [d]	TADF	773
	Y-Crystal	554	54	0.20	13.7 (235) [d]	TADF	773
	O-Crystal	568	53	0.03	12.5 (189) [d]	TADF	773
	R-Crystal	606	85	0.01	13.8 (276) [d]	TADF	773
	R-Solid	649	3	0.42	1.4 (25.8) [d]	N/A	773
**CpzPO**	Crystalline	459[e], 564 [e]	N/A	N/A	N/A, 62	Fl, TADF	774
	Amorphous	564 [e]	N/A	N/A	62	TADF	774
**SpzPO**	Crystalline	433 [e], 546 [e]	N/A	N/A	N/A, 29	Fl, TADF	774
	Amorphous	546 [e]	N/A	N/A	29	TADF	774
**SCP**	Pristine	415, 545	41	0.99, 0.41	N/A, 51	Fl, TADF	775
	Ground	545	N/A	0.41	51	TADF	775
**1**	1_Y	568	16	N/A	N/A	N/A	776
	1_O	640	8	N/A	N/A	N/A	776
	1_R	673	12	N/A	N/A	N/A	776
	1_O2	646	11	N/A	N/A	N/A	776
	1_YO	596	5	N/A	N/A	N/A	776
**2**	2_YG	547	13	N/A	N/A	N/A	776
	2_R	663	15	N/A	N/A	N/A	776
	2_R2	675	12	N/A	N/A	N/A	776
	2_Y	564	15	N/A	N/A	N/A	776
**XPT**	Solvent Evap	536	N/A	N/A	N/A	N/A	702
	Grinding	569	N/A	N/A	N/A	N/A	702
	Sublimation	566	N/A	N/A	N/A	N/A	702
**DPPZS-DBPHZ**	1-BG	497	6	N/A	N/A	N/A	777
	1-G1	518	9	N/A	N/A	N/A	777
	1-G2	520	9	N/A	N/A	N/A	777
	1-Y	534	16	N/A	N/A	N/A	777
	1-DR	740	3	N/A	N/A	N/A	777
**OIDBQx**	Initial	494	N/A	N/A	N/A	N/A	778
	Ground	522	N/A	N/A	N/A	N/A	778
**DPP-DMAC**	Pristine	554	N/A	N/A	8.3	TADF	779
	Ground	608	N/A	N/A	7.1	TADF	779
	Fumed	563	N/A	N/A	6.4	TADF	779
	Heated	575	N/A	N/A	4.7	TADF	779
**DPQ-DMAC**	Pristine	548	N/A	N/A	4.3	TADF	779
	Ground	571	N/A	N/A	8.9	TADF	779
	Fumed	547	N/A	N/A	4.1	TADF	779
	Heated	553	N/A	N/A	5.9	TADF	779
**DPQ-PXZ**	Pristine	589	N/A	N/A	1.4	TADF	779

	Ground	616	N/A	N/A	1.8	TADF	779
	Fumed	592	N/A	N/A	1.3	TADF	779
	Heated	598	N/A	N/A	1.4	TADF	779
**DPP-PXZ**	Pristine	628	N/A	N/A	2.7	TADF	779
	Ground	682	N/A	N/A	2.4	TADF	779
	Fumed	639	N/A	N/A	3.2	TADF	779
	Heated	649	N/A	N/A	2.6	TADF	779
**2**	Pristine	455, 530	N/A	N/A	N/A	N/A	780
	Grinding	540	N/A	N/A	N/A	N/A	780
**TPA-DQP**	Crystal-Y	576	15	N/A	1.1	TADF	781
	Crystal-R	694	22	N/A	2.4	TADF	781
**Cz-AQ**	R-Crystal	604	28	N/A	1.9	TADF	782
	Y-Crystal	541	59	N/A	1.8	TADF	782
**TXDM**	Crystalline	470	1.8	0.49	N/A	Fl	783
	Amorphous	486	27	0.30	17.4	TADF	783
**R-DOBP**	Crystalline	580	N/A	N/A	N/A	N/A	312
	Amorphous	647	N/A	N/A	N/A	N/A	312
**XT-OT**	Crystalline	466	42.5	N/A	N/A	N/A	784
	Amorphous	567	53.4	N/A	N/A	N/A	784
**XT-T**	Crystalline	478	38.1	N/A	N/A	N/A	784
	Amorphous	510	40.7	N/A	N/A	N/A	784
**Py-BZTCN**	Crystalline	581	52.75	0.330	N/A	N/A	785
	Amorphous	676 [e]	5.27	0.087	2.30	TADF	785

[a] Reported name for the solid-state phenomenon, [b] Excited-state decay mechanism, where Fl is fluorescence, TADF is thermally activated delayed fluorescence, [c] Calculated at the B3LYP/6-31G* level for each conformer, [d] Two delayed lifetimes reported, [e] Approximation using graphical fitting software.

Table **S20.** Photophysical and device properties of MCL emitters in solution or doped films, reviewed in Section **15**.

**Compound**	**Medium[a]**	**λ_{PL} / nm**	**Φ_{PL} / %**	**τ_d / µs**	**ΔE_{ST} / eV**	**λ_{EL} / nm**	**CIE_{XY}**	**EQE_{max} / %**	**EQE_{100} m^{-2} / %**	**EQE_{1000} / %**	**Ref**
**CPzPO**	1 wt% PMMA	470, 545	18	0.3, 12.6, 112.8	N/A	N/A	N/A	N/A	N/A	N/A	774
**SPzPO**	1 wt% PMMA	425, 525	29	0.3, 10.7, 95.7	N/A	N/A	N/A	N/A	N/A	N/A	774
**1**	10 wt% CBP	N/A	N/A	N/A	0.14	613	N/A	16.8	15.5 [b]	11.1 [b]	776
	Powder	518	93	1200	0.20 [g]	N/A	N/A	N/A	N/A	N/A	786
**2**	10 wt% CBP	N/A	N/A	N/A	0.18	646 [b]	N/A	11.2 [b]	11.2 [b]	9.9 [b]	776
	Solid	530	42	27.2	0.06	N/A	N/A	N/A	N/A	N/A	780
**XPT**	10 wt% DPEPO	566	66	3.3	N/A	584	N/A	10	8.6 [b]	4.4 [b]	702
**DPPZS-DBPHZ**	10 wt% Zeonex	484 [c]	34 [c]	N/A	0.30	N/A	N/A	N/A	N/A	N/A	777
**OIDBQx**	CH_2Cl_2 dilute	534	31	128, 000 [d]	0.49 [d]	N/A	N/A	N/A	N/A	N/A	778
**DPQ-DMAC**	10 wt% DPEPO	554	N/A	N/A	N/A	556	N/A	11.3	10.5	5.3	779
**DPP-DMAC**	PhMe	599	N/A	N/A	N/A	N/A	N/A	N/A	N/A	N/A	779
**DPQ-PXZ**	PhMe	618	N/A	N/A	N/A	N/A	N/A	N/A	N/A	N/A	779

**DPP-PXZ**	PhMe	647	N/A	N/A	N/A	N/A	N/A	N/A	N/A	N/A	779
**TPA-DQP**	10 wt% Bepp2	676	65	107	0.11 [c]	676	0.67,0.32	18.3	N/A	1.9 [b]	781
**Cz-AQ (Dev1)[e]**	Neat	N/A	N/A	N/A	N/A	680	0.68,0.30	0.75	N/A	N/A	782
**Cz-AQ (Dev2)[f]**	Neat	N/A	N/A	N/A	N/A	600	0.50,0.42	1.15	N/A	N/A	782
**R/S-DOBP**	Neat	670	11	6	0.14	716	N/A	1.9	N/A	N/A	312
**R/S-HDOBP**	Neat	662	9	3	0.08	700	N/A	0.7	N/A	N/A	312
**XT-OT**	20 wt% CBP	517	50.8	77	0.20	532	0.315, 0.569	9.39	N/A	9.38	784
**XT-T**	20 wt% CBP	480	44.2	154	0.34	488	0.208, 0.433	3.30	N/A	2.90	784

[a] Stated medium is that used for the PL study and in the EML of the OLED unless otherwise stated, [b] Approximation using graphical fitting software, [c] Dilute toluene, [d] 1 wt% Zeonex film, [e] Solution-processed device spin-coated from dichloroethane, [f] Solution-processed device spin-coated from dichloroethane:ethanol mix (1:1), [g] TD-DFT-B3LYP/6-31G*.

Table **S21.** Photophysical and device properties of TADF LECs reviewed in Section **16**.

Compound	Medium	λ_{PL}/ nm	Φ_{PL} / %	τ_d/ μs	ΔE_{ST}/ eV	λ_{EL} / nm	CIE_{XY}	EQE_{max} / %	Lum_{max} / cd m^{-2}	$t_{1/2}$ [a]	Ref
**2CzPN-LEC**	Neat	536	21	2.7	N/A	538	0.35, 0.57	0.4	13	N/A	787
**imCzDPS**	Neat	414 [b]	44 [b]	48 [b]	N/A	470, 550	0.21, 0.25	1.14	2.5	N/A	788
**CzTz1**	Neat	501	55	3.2	0.02	510	0.29, 0.52	4	565	6.2 h	789
**CzTz2**	Neat	523	52	2.3	0.02	532	0.34, 0.57	6.8	890	11.4 h	789
**CzTz3**	Neat	524	48	1.8	0.01	542	0.39, 0.55	6.2	860	47.8 h	789
**Pym-CZ**	Neat	500 [b]	43 [a]	2.3	0.06	609	0.54, 0.46	1.19	8.69	N/A	790
**Pym-*t*BuCZ**	Neat	553 [b]	8 [a]	N/A	0.03	620	0.58, 0.42	0.14	1.96	N/A	790
**4CzIPN**	CBP:Emitter: PEO:KCF3SO3:polystyrene	512	40	N/A	N/A	~522	0.32, 0.52	0.17	760	N/A	791
**P1**	PYD-2Cz:Emitter: $THABF_4$:PS	505	71	N/A	N/A	500	0.31, 0.44	N/A	600	N/A	792
**4CzIPN**	PVK:OXD-7:Emitter:$THABF_4$	N/A	N/A	11	0.025	505	0.31, 0.47	~2	430	N/A	793
**TXO-TPA**	PVK:OXD-7:Emitter:$THABF_4$	N/A	N/A	33	0.105	585	0.46. 0.50	7	740	N/A	793
**TPA-DCPP**	PVK:OXD-7:Emitter:$THABF_4$	N/A	N/A	21	0.140	618	0.54, 0.44	~4	380	N/A	793
**BPAPTC**	PVK:OXD-7:Emitter:$THABF_4$	519	90	N/A	0.06	533	0.36, 0.54	7.67	3696	N/A	794
**[*t*BuCAZ-ImMe][PF_6]: [TRZ-ImEt][PF_6]**	Emitter:[TRZ-ImEt][PF_6]	472	65	N/A	N/A	499	0.23, 0.43	2.6	231	90 min	795
**TCTA:26DCzPPy**	Emitter:CZ-TRZ:$THABF_4$	410	11.6	N/A	N/A	475	0.19, 0.27	5	740	N/A	796
**DiKTa-ObuIm**	Neat	500 [c]	57	316.6	0.2	534	0.19, 0.79	<0.01	15	N/A	797
**DiKTa-DPA-ObuIm**	Neat	578 [c]	53	241.7	0.19	656	0.73, 0.27	<0.01	2	N/A	797
**[Cu(4,5,6-Me_3bpy) (xantphos)][P**	Emitter:[EMIM][PF6]=4:1	529 [b]	19	N/A	0.23	570	0.44, 0.55	1.7	462	<5 h	798

**F$_6$]**											
**Cu(6,6-Me$_2$bpy)(xantphos)][PF$_6$]**	Emitter:[EMIM][PF6]=4:1	529	38.4	10.9	N/A	557	0.35, 0.64	N/A	53	1.47 h	798,799
**[Cu(2-Etphen)(xantphos)][PF$_6$]**	Emitter:[EMIM][PF6]=4:1	558 ^{b}	26	N/A	0.08	582	0.53, 0.47	1.8	451	34 h	798
**[Cu(Me$_2$bpy)(BnN-xantphos)][PF$_6$]**	Emitter:[EMIM][PF6]=4:1	520 ^{b}	55 ^{a}	N/A	N/A	567	0.43, 0.53	1.2	355	155 min	800
**[Cu(Me$_2$bpy)(xantphos)][PF$_6$]**	Emitter:[EMIM][PF6]=4:1	563	44	14.7 ^{b}	N/A	N/A	N/A	N/A	173	N/A	801
**[Cu(Me$_2$bpy)(xantphos)][BF$_4$]**	Emitter:[EMIM][PF6]=4:1	563	45	8.7 ^{b}	N/A	N/A	N/A	N/A	137	N/A	801
**[Cu(Me$_2$bpy)(xantphos)][BPh$_4$]**	Emitter:[EMIM][PF6]=4:1	548	32	12.9 ^{b}	N/A	N/A	N/A	N/A	N/A	N/A	801
**[Cu(Me2bpy)(xantphos)][BarF$_4$]**	Emitter:[EMIM][PF6]=4:1	552	35	8.5 ^{b}	N/A	N/A	N/A	N/A	N/A	N/A	801
**[Cu(L3)(Ipr)][PF$_6$]**	Neat	~550	N/A	N/A	0.7	N/A	N/A	N/A	N/A	N/A	802
**[Cu(L4)(Ipr)][PF$_6$]**	Neat	~420	N/A	N/A	0.65	N/A	N/A	N/A	N/A	N/A	802
**[Cu(L5)(Ipr)][PF$_6$]**	Neat	521	N/A	N/A	0.67	N/A	0.23, 0.28	0.6	10	1.2 min	802
**[Cu(L7)(Ipr)][PF$_6$]**	Neat	458	N/A	N/A	N/A	N/A	N/A	N/A	N/A	N/A	802

^{a} Longest lifetime; ^{b} Powder; ^{c} 1% in mCP.

Table **S22.** Properties of HF-TADF devices reviewed in Section **17**.

TADF Dopant	Emitter	Emissive Layer	Turn-on/ V	EQEmax/EQE$_{100}$/EQE$_{1000}$ / %	CIE (x, y)	Ref
**ACRSA**	**TBPe**	1 wt% TBPe:15 wt% ACRSA in DPEPO	4.7	13.4/-/8.7	0.17, 0.30	803
**ACRXTN**	**TTPA**	1 wt% TTPA:50 wt% ACRXTN in mCP	3.0	15.8/-/11.7	0.29, 0.59	803
**PXZ-TRZ**	**TBRb**	1 wt% emitter:25 wt% PXZ-TRZ in mCBP	3.2	18.0/-/17.2	0.45, 0.53	803
**Tri-PXZ-TRZ**	**DBP**	1 wt% DBP:15 wt% Tri-PXZ-TRZ in CBP	3.0	17.5/-/17.2	0.61, 0.39	803
**CzAcSF**	**TBPe**	x wt% TBPe:y% CzAcSF in DPEPO, where x, y=1, 25; 1, 50; 0.1 50	-	17.2/-/-; 17.7/-/-; 18.1/-/-	0.14, 0.19; 0.15, 0.21; 0.15, 0.22	804
**DMAC-DMT**	**BPPyA**	0.7 wt% BPPyA:40 wt%DMAC-DMT in DBFPO	-	19.0/-/-	0.14, 0.15	805
**DMAC-DPS**	**BPPyA**	0.7 wt% BPPyA:40 wt%DMAC-DPS in DPEPO	-	13.0/-/-	0.15, 0.16	805
**SPAC-DMT**	**BPPyA**	0.7 wt% BPPyA:40 wt% SPAC-DMT in DBFPO	-	7.0/-/-	0.14, 0.11	805

**4CzFCN**	**KCTBC**	3 wt% KCTBC:12.5 wt% 4CzFCN in CBP	3.6	13.9/12.0/6.2	0.18, 0.13	806
**FTrzTCz**	**C545T**	1% C545T:20wt%FTrzTCz in DPEPO	7.06	11.5/-/7.1	0.24, 0.64	807
**FTrzTCz**	**6tBPA**	1 wt% 6tBPA:20 wt%FTrzTCz in DPEPO	7.11	17.9/-/14	0.24, 0.58	807
**TbCzTrz**	**6tBPA**	x wt% 6tBPA:20 wt% TbCzTrz in DPEPO, where x= 0.5; 1	-	14.6/-/9.2; 13.5/-/8.1	0.25, 0.57; 0.26, 0.60	808
**TbCzTrz**	**C545T**	0.5 wt% C545T: 20 wt% TbCzTrz in DPEPO		16.1/-/9.5	0.25, 0.63	808
**TmCzTrz**	**6tBPA**	x wt% 6tBPA:20 wt% TmCzTrz in DPEPO, where x= 0.5; 1	-	18.5/-/ 15.3; 14.0/-/10.7	0.25, 0.57; 0.26, 0.60	808
**TmCzTrz**	**C545T**	0.5 wt% C545T:20 wt% TmCzTrz in DPEPO	-	15.9/-/11.8	0.25, 0.63	808
**PXZ-DBPZ**	**DBP**	0.6 wt% DBP:9 wt% PXZ-DBPZ in CBP	3.2	15.2/-/-	0.57, 0.42	809
**FPXZ-DBPZ**	**DBP**	0.6 wt% DBP:9 wt% FPXZ-DBPZ in CBP	3.3	18.1/-/6.5	0.61, 0.38	809
**4CzIPN-tBu**	**cibalackrot**	x mol% cibalackrot:y mol% 4CzIPN-tBu in CBP, where x, y = 0.5, 29.5; 1, 29; 1.5, 28.5	5.0; 4.8; 4.4	15.3/14.9/8.4	-	810
**4CzIPN**	**cibalackrot**	1.5 mol% cibalackrot:28.5 mol% 4CzIPN-tBu:CBP	4.4	5.6/5.6/4.4	-	810
**4CzIPN-Me**	**TBRb**	0.65 wt% TBRb:6.3 wt% 4CzIPN-Me in CBP	-	19.1/-/16.7	0.43, 0.54	811
**34AcCzTrz**	**TBRb**	1 wt% TBRb: 4 wt% 34A cCzTrz in 3CzPhpPM	-	19.1/-/-	0.49, 0.50	52
**PXZ-DPS**	**PAD**	0.5 mol% PAD:30 wt% PXZ-DPS in PhCzTRZ	2.56 at 10 cd m^{-2}	18.6/-/ 18.5	0.36, 0.57	812
**PXZ-DPS**	**MePAD**	0.5mol% MePAD:30 wt% PXZ-DPS in PhCzTRZ	2.54 at 10 cd m^{-2}	20.8/-/-20.7	0.37, 0.58	812
**PXZ-DPS**	**tBuPAD**	1 mol% tBuPAD:30 wt% PXZ-DPS in PhCzTRZ	2.57 at 10 cd m^{-2}	22.7/-/-22.5	0.36, 0.58	812
**PXZ-DPS**	**PhtBuPAD**	2 mol% PhtBuPAD:30 wt% PXZ-DPS in PhCzTRZ	2.55 at 10 cd m^{-2}	24.0/-/-23.8	0.36, 0.58	812
**DACT-II**	**PhtBuPAD**	3 wt% PhtBuPAD:20 wt%DACT-II in m32aICT	-	23.2/-/-	0.36, 0.60	813
**DACT-II**	**PhtBuPAD**	3 wt% PhtBuPAD:40 wt%DACT-II in m23bICT	-	16.1/-/-	0.37, 0.59	813
**DACT-II**	**PhtBuPAD**	3 wt% PhtBuPAD:40 wt%DACT-II in CPyBP	-	18.5/-/-	0.36, 0.60	813
**DACT-II**	**PhtBuPAD**	3 wt% PhtBuPAD:40 wt%DACT-II in CBP	-	19.4/-/-	0.35, 0.60	813
**DBP**	**tBIQAC**	0.7 wt% DBP:20 wt% tBIQAC in PBICT	5.8	16.8/-/11.7	0.63, 0.37	814
**DBP**	**DtBIQAC**	0.7 wt% DBP:20 wt%	6.4	14.7/-/10.3	0.64,	814

		DtBIQAC in PBICT			0.36	
**DBP**	**tBIQAP**	0.7 wt% DBP:20 wt% tBIQAP in PBICT	6.6	17.5/-/7.0	0.63, 0.37	814
**DBP**	**DtBIQAP**	0.7 wt% DBP: 20 wt% DtBIQAP in PBICT	6.5	18.2/-/8.2	0.62, 0.38	814
**DMAC-DPS**	**TBPe**	1 wt TBPe in DPEPO: 50 wt% DMAC-DPS in DPEPO	6.0	18.8/-/13.5	0.14, 0.25	815
**DMAC-DPS**	**TBPe**	1 wt% TBPe:x wt% DMAC-DPS in DPEPO, where x= 10; 20; 30; 40	3.2;3.0;3.0;2.8	10.0/ 7.4/1.9; 14.1/11.3/7.3; 11.0/8.2/4.1; 9.7/8.0/4.9	0.14, 0.17; 0.14, 0.17; 0.14, 0.17; 0.14, 0.17	816
**Pr-1**	**DCJTB**	1 wt% DCJTB:10 wt% Pr-1 in mCBP:PO-T2T	2.4	13/10.5/8.7	-	817
**4CzIPN**	**DCJTB**	0.5 wt% DCJTB:2 wt% 4CzIPN in TCTA:B4PyMPM	3.0	12.9/12.4/10.1	0.58, 0.41	818
**4CzIPN**	**DCJTB**	0.5 wt% DCJTB:2 wt% 4CzIPN in TCTA	3.1	6.6/6.1/3.3	0.52, 0.46	818
**B4PyMPM**	**DCJTB**	0.5 wt% DCJTB:2 wt% B4PyMPM in TCTA	2.9	7.3/7.1/6.0	0.59, 0.41	818
**4CzIPN**	**PtOEP**	4 wt% PtOEP:6 wt% 4CzIPN in CBP:B4PyMPM	-	21.5/-/-	-	819
**BPAc**	**6tBPA**	x wt% 6tBPA:20 wt%BPAc in DPEPO, where x= 0.5, 1	-	16.6/-/ 15.2; 15.8/-/14.4	0.23,0.51 ; 0.23,0.54	820
**BPAcCz**	**6tBPA**	x wt%6tBPA:20 wt% BPAcCz in DPEPO, where x= 0.5, 1	-	15.0/-/13.3; 14.4/-/12.6	0.25,0.53 ; 0.25,0.56	820
**DC-TC**	**DBP**	2 wt% DBP:15 wt% DC-TC in CBP	5.2	8.0/6.65/-	0.61, 0.38	821
**DC-ACR**	**DBP**	2 wt% DBP:15 wt% DC-TC in CBP	5.4	4.25/3.62/-	0.63, 0.37	821
**OSTFB**	**OTPA-BT-CN**	x wt% OTPA-BT-CN:25 wt% OSTFB in mCP, where x= 0.5; 1; 2	3.0; 3.0; 3.4 at 100 cd m^{-2}	12.4/-/-; 10.1/-/-; 4.4/-/-	-	822
**PXZ-TRZ**	**TBRb**	1 wt% TBRb:x wt% PXZ-TRZ in SF4-TPE, where x= 10; 20; 30; 40	4.1;3.9;3.8;3.7	23.5/-/21.5; 24.7/-/23.6; 24.2/-/23.5; 23.0/-/22.9	0.48, 0.51; 0.49, 0.50; 0.49, 0.50; 0.49, 0.50	823
**PXZ-TRZ**	**TBRb and 4P-NPD**	1 wt% TBRb:30 wt% PXZ-TRZ:SF4-TPE|40 wt% 4P-NPD:SF4-TPE	3.7	17.6/15.5/14.2	0.39, 0.39	823
**4CzPN**	**TBRb and Bepp2**	0.4 wt% TBRb:6 wt% 4CzPN|0.8 wt% TBRb:10 wt% 4CzPN:mCP|40 wt% Bepp2:mCP|Bepp2	3.0	15.1/-/4.6	0.35, 0.49	824
**BPS**	**Ir(bt)2acac**	0.5 wt% Ir(bt)2acac:10wt% BPS in CBP:OXD-	3.6	4.55/2.37/4.55	0.37, 0.37	825

		7(100:40)				
**BPS**	**Ir(bt)2acac**	0.5 wt% Ir(bt)2acac:10wt% BPS in DCzPPy:OXD-7(100:4)	4.6	6.59/1.09/4.41	0.41, 0.41	825
**4CzIPN**	**Hex-Ir(phq)2(acac)**	1.5 wt% Hex-Ir(phq)2(acac):x wt% 4CzIPN in CBP, where x= 7.5; 15; 20; 50	4.3; 3.8; 3.8; 3.3	9.8/-/9.6; 6.6/-/6.3; 5.6/-/5.4; 2.4/-/2.1	-	826
**PXZ-TRZ**	**CuPc**	1 wt% CuPc:45 wt% PXZ-TRZ in mCBP	-	0.037	-	827
**TPAM-BF2**	**BPPC-Ph**	0.5% BPPC-Ph:20% TPAM-BF2 in CBP	-	3.5/-/-	-	828
**TPA-DCPP**	**BPPC**	0.8 wt% BPPC:20 wt%TPA-DCPP in B3PYMPM	-	5.4/-/-	-	829
**tBuCz-p-NI**	**tBuCz-p-NI**	0.6% wt% tBuCz-s-NI:10 wt% tBuCz-p-NI in mCP	2.7	27/-/-	-	830
**4CzIPN**	**TTM-3PCz**	3 wt% TMM-3PCz:25 wt% 4CzIPN in CBP	2.3	16.4/-/-	-	831
**(MAC*)Cu(Cz)**	**TBRb**	20 wt% (MAC*)Cu(Cz): 1 wt% TBRb in mCBP	3.0	16.6/-/16.4	-	832
**(MAC*)Cu(Cz)**	**BN3**	20 wt% (MAC*)Cu(Cz): 1 wt% BN3 in mCBP	2.8	26.5/-/-	-	832
**Au-1**	***ν*-DABNA**	10 wt% Au-1:0.5 wt% n-DABNA in PYD2		16.6/-/14.4	0.14, 0.18	833

Table **S23.** Properties of TADF emitters used as hosts in devices evieweed in Section **18**.

TADF Host	Emitter	Medium	λ_{EL} / nm	CIE_{XY}	EQE_{max} / EQE_{100} / EQE_{1000} / %	Ref
**PBICT**	**$Ir(ppy)_3$**	3 wt%	-	-	23.9/-/-	834
**DIC-TRZ**	**$Ir(ppy)_3$**	2 wt%	-	-	20.9/-/-	834
**POBICT**	**PO-01**	10 wt%	566	0.51, 0.49	22.0/-/21.7	835
**BICT**	**PO-01**	10 wt%	566	0.51,0.49	16.8/-/16.6	835
**PBICT**	**PO-01**	10 wt%	566	0.51, 0.49	24.5/-/24.2	835
**BBICT**	**PO-01**	10 wt%	566	0.51, 0.49	13.7/-/13.6	835
**BCPICT**	**$Ir(mphmq)_2(tmd)$**	2 wt%	620–624	-	10.5/-/9.9	836
**DMAC-DPS**	**PO-01**	0.8 wt%	460,560	0.39, 0.46	20.8/-/19.6	837
**DMAC-DPS**	**$Ir(ppy)_2acac$**	2.0 wt%	-	-	19.5/-/19.1	838
**DMAC-DPS**	**$Ir(mphmq)_2tmd$**	0.5 wt%	-	-	22.4/-/21.6	838
**DMAC-DPS**	**$Ir(ppy)_2acac$, $Ir(mphmq)_2$ (tmd)**	0.2wt% green, 0.2wt% red	-	0.36, 0.39	20.2/-/19.4	838

**mSOAD**	**Ir(pq)$_2$acac**	4 wt%	-	0.62, 0.38	20.3/-/10.8	839
**mSOAD**	**FIrpic**	6 wt%	471	-	16.7/-/8.0	840
**mSOAD**	**Ir(ppy)$_3$**	4 wt%	515	-	19.0/-/18.4	840
**mSOAD**	**Ir(pq)$_2$acac**	4 wt%	606	-	20.3/-/10.6	840
**tBu-mSOCz**	**FIrpic**	6 wt%	471	-	14.7/-/13.3	840
**tBu-mSOCz**	**Ir(ppy)$_3$**	4 wt%	515	-	11.2/-/10.1	840
**tBu-mSOCz**	**Ir(pq)$_2$acac**	4 wt%	606	-	13.9/-/12.2	840
**BCz-2SO**	**FIrpic**	1.0 wt%	476	-	7.8/-/-	841
**BT-01**	**FIrpic**	10 wt%	-	0.16,0.36	31.8/31.6/31.2	842
**BT-01**	**2CzPN**	10 wt%	-	0.16,0.31	25.5/19.5/10.0	842
**BT-02**	**FIrpic**	10 wt%	-	0.17,0.40	30.7/30.5/29.9	842
**BT-02**	**2CzPN**	10 wt%	-	0.17,0.31	22.3/13.9/6.2	842
**DMAC-BPP**	**PO-01**	5 wt%	564	-	19.8/-/19.7	843
**DCb-BPP**	**PO-01**	5 wt%	561	-	21.5/-/20.6	843
**2a**	**Ir(mppy)$_3$**	3 wt%	-	-	-/-/19.5	1
**2b**	**Ir(mppy)$_3$**	3 wt%	-	-	-/-/20.0	1
**2c**	**Ir(mppy)$_3$**	3 wt%	-	-	-/-/21.5	1
**2c-Ph**	**Ir(mppy)$_3$**	3 wt%	-	-	-/-/19.4	1
**Cz-Ph-TRZ**	**Ir(mppy)$_3$**	3 wt%	-	-	-/-/19.8	1
**PIC-TRZ2**	**Ir(mppy)$_3$**	3 wt%	-	-	-/-/22.0	1
**DIC-TRZ**	**Ir(mppy)$_3$**	3 wt%	-	-	-/-/21.8	1
**DIC-TRZ-Ph**	**Ir(mppy)$_3$**	3 wt%	-	-	-/-/16.0	1
**Trz–PhCz**	**Ir(ppy)$_2$(acac)**	8 wt%	-	0.35, 0.61	24.3/22.9/22.3	844
**Trz–PhCz**	**PO-01**	5 wt%	-	0.51, 0.48	27.3/26.5/25.9	844
**Trz–PhCz**	**Ir(dmppm)$_2$(acac)**	8 wt%	-	0.61, 0.39	31.4/30.9/29.1	844
**Trz–PhCz**	**Ir-F-DHBA**	3 wt%	-	0.62, 0.38	30.4/26.9/25.9	844
**DMAC-TA**	**Ir(mppy)$_3$**	3 wt%	–	-	-	845
**2Cz-TA**	**Ir(mppy)$_3$**	3 wt%	-	-	-	845
**DMAC-TRZ**	**Ir(mppy)$_3$**	3 wt%	-	-	-	845
**2CZ-TRZ**	**Ir(mppy)$_3$**	3 wt%	-	-	-	845
**DCzTrz**	**FIrpic**	-	-	0.16, 0.34	15.7/-/15.0	846
**TrzmPCz**	**FIrpic**	-	-	0.15, 0.32	16.3/-/14.1	846
**DCzCNTrz**	**FIrpic**	-	-	0.23, 0.49	2.8/-/1.3	846

**PXZ-ICO**	**$Ir(mphmq)_2(tmd)$**	3.5 wt%	605	0.62 0.37	18.6/-/-	[847]
**TPPOCz**	**FIrpic**	4 wt%	475	0.17,0.36	20.4/-/-	[848]
**TPPOCz**	**$Ir(CF_3BT-CF_3P)_2(acac)$**	3 wt%	570	0.49, 0.50	14.9/-/-	[848]
**TPPOCz**	**$Ir(2\text{-}phq)_2(acac)$**	3 wt%	612	0.62, 0.37	12.4/-/-	[848]
**OSTFPB**	**$Ir(MDQ)_2(acac)$**	2.0 wt%	-	-	29.1/-/-	[849]
**OSTFPCN**	**$Ir(MDQ)_2(acac)$**	2.0 wt%	-	0.61,0.39	31.2/-/-	[849]
**DIC-TRZ**	**DDAF**	1 wt%	-	-	12.2/-/5.5	[850]
**PIC-TRZ**	**DDAF**	1 wt%	-	-	4.7/-/3.9	[850]
**DMAC-PN**	**TPANSeD**	4 wt%	730	-	2.65/-/-	[851]
**DMAC-PN**	**NSeD**	4 wt%	664	-	3.81-/-	[851]
**4CzIPN**	**C4-DFQA**	0.5 wt%	-	0.44,0.55	13.5/-/11.0	[852]
**4CzIPN**	**C4-TCF3QA**	0.5 wt%	-	0.45,0.54	14.6/-/12.3	[852]
**DMAC-DPS**	**C545T**	1.5 wt%	-	-	9.0/-/-	[853]
**PXZ-DPS**	**C545T**	1.0 wt%	-	-	6.9/-/-	[853]
**2PXZ-OXD**	**C545T**	1.0 wt%	-	-	2.1/-/-	[853]
**2PXZ-TAZ**	**C545T**	1.0 wt%	-	-	5.6/-/-	[853]
**TAPC:DPTPCz**	**C545T**	1 wt%	-	0.24,0.58	7.5/-/5.3	[854]
**TAPC:2d**	**DPPFuC4**	1 wt%	-	-	12.1/11.2/-	[855]
**TAPC:2d**	**DPPThC4**	1 wt%	-	-	-/11.1/-	[855]
**TCTA:Tm3PyBPZ**	**C545T**	1 wt%	-	0.24,0.64	10.4/9.1/7.9	[856]
**TCTA:Tm3PyBPZ**	**rubrene**	1 wt%	-	0.44,0.54	10.0/10.0/8.4	[856]
**SFBCz: SFTRZ**	**5TCzBN: TBRb: RD**	20 wt% : 0.2 wt% : 0.05 wt%	-	0.44,0.45	16.7/-/16.5	[857]
**XAc-XT**	**G0**	1.0 mol%	-	-	3.2/-/-	[858]
**XAc-XT**	**G1**	1.0 mol%	-	-	5.2/-/-	[858]
**DMIC-TRZ**	**4TCzTPN**	2 wt%	-	-	23.2/-/-	[859]
**DMIC-TRZ**	**DMAC-BP**	12 wt%	513	-	21.0/-/-	[859]
**DMIC-TRZ**	**5TCzBN**	30 wt%	486	-	19.2/-/-	[859]
**DMAC-DPS**	**4CzIPN**	6 wt%	-	0.23, 0.50	10.9/-/9.1	[860]
**DMAC-DPS**	**4CzTPN-Ph**	9 wt%	-	0.39, 0.53	11.0/-/10.1	[860]
**Sy**	**4CzIPN**	10 wt%	-	0.28,0.55	24.0/-/22.1	[861]
**Sy**	**2CzTPN**	10 wt%	-	0.19,0.41	20.4/-/16.9	[861]
**Asy**	**4CzIPN**	10 wt%	-	0.25,0.56	22.5/-/20.4	[861]

**Asy**	**2CzTPN**	10 wt%	-	0.21,0.44	15.0/-/12.7	861
**m-CzPym**	**4CzIPN**	6 wt%	-	0.256, 0.578	31.5/-/29.0	862
**p-CzPym**	**4CzIPN**	6 wt%	-	0.286, 0.587	29.2/-/28.5	862
**m-CzTrz**	**4CzIPN**	10 wt%	-	0.312, 0.595	29.5/-/28.4	862
**p-CzTrz**	**4CzIPN**	10 wt%	-	0.271, 0.584	24.1/-/24.1	862
**CzAcSF**	**OHOXD**	10 wt%	480	0.17,0.28	12.1/-/4.3	863
**CzAcSF**	**BFOXD**	10 wt%	492	0.21,0.38	20.1/-/12.7	863
**m-DTPACO**	**4CzCNPy**	10 wt%	520	0.32,0.60	13.0/13.0/10.3	864
**p-DTPACO**	**4CzCNPy**	10 wt%	520	0.33,0.58	9.0/8.0/5.6	864
**Cz-3CzCN**	**Cz-4CzCN**	4 wt%	-	0.25,0.52	23.5/15.5/7.8	865

Table **S24.** Photophysical properties and ASE parameters of the reported TADF molecules, reviewed in Section **22**.

**Compound**	**λ_{PL} / nm**	**τ_{PL} / ns**	**Φ_{PL} / %**	**k_{RISC} / s^{-1}**	**ASE**				
					**λ_{ASE} / nm**	**FWHM / nm**	**Gain / cm^{-1}**	**E_{th} / $\mu J\ cm^{-2}$**	**excitation source**
TADF molecules as the gain medium									
**DABNA-2** (6 wt%, mCBP)[866]	467, 490	4.5	83	3.1×10^3	494	~ 6.6	--	1.6	N_2 gas laser
**PXZN-B** (10wt%, mCP)[867]	468	5.4	79	4.5×10^4	470	8.1	--	3.3	N_2 gas laser
**DMACN-B** (5wt%, mCP)[867]	444	4.0	83	2.5×10^4	444	11.3	--	12.0	N_2 gas laser
**TPABC** (6 wt%, CBP)[265]	721	4.5	70	--	747	17	--	6.7	N_2 gas laser
**TPATBC** (2wt% F8BT)[265]	724	1.3	45	--	807	21.7	--	13.3	N_2 gas laser
TADF molecules as the triplet harvester (laser dye: TADF molecules: host)									
**C545T**:**ACRXTN**:mCBP[868]	506	--	86	4.6×10^5	535	~10	25.0	0.8	N_2 gas laser
**BUBD-1**:**DMAC-DPS**[869]	470, 500	10.8	82.3	--	500	6	18.0	1.19	OPO laser
**DT-DPP**:**Cz-DBA**:CBP[870]	577, 618	9.9	77	3.1×10^5	620	~10	--	4	N_2 gas laser

Table **S25.** Photophysical properties and lasing parameters of the reported TADF molecules, reviewed in Section **22**.

**Compound**	**λ_{PL} / nm**	**τ_{PL} / ns**	**Φ_{PL} / %**	***k*$_{RISC}$ / s^{-1}**	**Lasing**					
					**λ_{lasing} / nm**	**FWHM / nm**	**Gain / cm^{-1}**	**E_{th}^{laser}/μJ cm^{-2}**	**Resonator**	**Q value**
**TPATBC** (2 wt% F8BT)[265]	724	1.3	45	--	807	0.2	--	6.2	DFB	N.A.
**CAZ-A** (4 wt%, CBP)[871]	650	1.27	~17	6.5×10^{3}	683	0.52	640	3.96	WGM microring	~10^{3}
**4CzTPN** (3 wt%, PS)[872]	520	10.7	71.4	2.2×10^{6}	563	0.21	---	88	WGM microsphere	~10^{3}
**MOON** microcrystals[873]	588	0.4	5		650			3.04		
**ON** microcrystals[873]	561	0.6	4	--	561	--	--	4.96	Microcrystals	~10^{3}
**MOCN** microcrystals[873]	539	0.4	3		525			3.49		
**DCzBF$_2$** microwires[874]	465	2.08	48	--	465	0.5	870	3.74	Microwires	~10^{3}

Table **S26.** Selected photophysical and electrochemical properties of common visible light PCs and popular TADF PCs,[a] reviewed in Section **23**.

**Compound**	**λ_{abs} / nm**	**λ_{PL} / nm**	**$E_{0,0}$ / eV**	**E_{ox} / V**	**E_{red} / V**	**E^*_{ox} / V**	**E^*_{red} / V**	**τ / ns**	**Ref**
**[Ru(bpy)$_3$](PF$_6$)$_2$**	452	615	2.10 ^{b}	1.29	-1.33	-0.81	0.77	1100	875,876
***fac*-Ir(ppy)$_3$**	375	518	2.75 ^{b}	0.77	-2.19	-1.73	0.31	1322	877-879
**[Ir(dF(CF$_3$)ppy)$_2$(dtbbpy)]PF$_6$**	380	470	2.77 ^{b}	1.69	-1.37	-0.89	1.21	2300	879-882
**Eosin Y**	520 ^{c}	537 ^{d}	2.31 ^{c}	0.78	-1.06	-1.11	0.83	2.10 ^{c} τ_S<br>160000 ^{d} τ_T	883-888
**4CzIPN**	435	535	2.56	1.52	-1.21	-1.04	1.35	18.7 τ_{pf}<br>1390 τ_{df}	889-892

**4DPAIPN**	425	523	2.62	1.34	-1.52	-1.28	1.1		[889]

[a] λ_{abs} refers to the absorption maximum of the lowest energy absorption band. λ_{PL} refers to the photoluminescence maximum. $E_{0,0}$ values given correspond to the optical gap to the S_1 state unless otherwise noted. All potentials are given in V versus SCE. $E^*_{ox} = E_{ox} – E_{0,0}$ and $E^*_{red} = E_{red} + E_{0,0}$. τ_{pf}, τ_{df}, τ_S and τ_T are the prompt fluorescence lifetime, the delayed fluorescence lifetime, the singlet excited state lifetime and the triplet excited state lifetime, respectively. Data reported in MeCN unless otherwise noted. [b] Corresponds to the optical gap to T_1 state. [c] Determined in methanol. [d] Determined in H_2O.

References:

(1) Fukagawa, H.; Shimizu, T.; Iwasaki, Y.; Yamamoto, T. Operational Lifetimes of Organic Light-Emitting Diodes Dominated by Förster Resonance Energy Transfer. *Sci. Rep.* **2017**, *7*, 1735.

(2) Byeon, S. Y.; Kim, J.; Lee, D. R.; Han, S. H.; Forrest, S. R.; Lee, J. Y. Nearly 100% Horizontal Dipole Orientation and Upconversion Efficiency in Blue Thermally Activated Delayed Fluorescent Emitters. *Adv. Optical Mater.* **2018**, *6*, 1701340.

(3) Nakanotani, H.; Masui, K.; Nishide, J.; Shibata, T.; Adachi, C. Promising Operational Stability of High-Efficiency Organic Light-Emitting Diodes Based on Thermally Activated Delayed Fluorescence. *Sci. Rep.* **2013**, *3*, 2127.

(4) Liu, B.; Li, J.; Liu, D.; Mei, Y.; Lan, Y.; Song, K.; Li, Y.; Wang, J. Electron-Withdrawing Bulky Group Substituted Carbazoles for Blue TADF Emitters: Simultaneous Improvement of Blue Color Purity and Risc Rate Constants. *Dyes Pigm.* **2022**, *203*, 110329.

(5) Feng, Q.; Tan, K.; Zheng, X.; Xie, S.; Xue, K.; Bo, Y.; Zhang, H.; Lin, D.; Rao, J.; Xie, X.et al. Simultaneous and Significant Improvements in Efficiency and Stability of Deep-Blue Organic Light Emitting Diodes through Friedel-Crafts Arylmethylation of a Fluorophore. *ChemPhotoChem* **2020**, *4*, 321-326.

(6) Xiang, S.; Lv, X.; Sun, S.; Zhang, Q.; Huang, Z.; Guo, R.; Gu, H.; Liu, S.; Wang, L. To Improve the Efficiency of Thermally Activated Delayed Fluorescence OLEDs by Controlling the Horizontal Orientation through Optimizing Stereoscopic and Linear Structures of Indolocarbazole Isomers. *J. Mater. Chem. C* **2018**, *6*, 5812-5820.

(7) Tao, W.-W.; Wang, K.; Chen, J.-X.; Shi, Y.-Z.; Liu, W.; Zheng, C.-J.; Li, Y.-Q.; Yu, J.; Ou, X.-M.; Zhang, X.-H. Dibenzofuran/Dibenzothiophene as the Secondary Electron-Donors for Highly Efficient Blue Thermally Activated Delayed Fluorescence Emitters. *J. Mater. Chem. C* **2019**, *7*, 4475-4483.

(8) Shin, D. J.; Lim, J.; Lee, J. Y. Combinatorial Donor Engineering for Highly Efficient Blue Thermally Activated Delayed Fluorescence Emitters with Low Efficiency Roll-Off. *J. Mater. Chem. C* **2021**, *9*, 15276-15283.

(9) Wang, D.-H.; Yao, J.-F.; Li, H.-Z.; Li, G.-Z.; Xie, F.-M.; Li, Y.-Q.; Hu, Y.-Y.; Tang, J.-X.; Zhao, X. New Strategy for Developing Deep Blue TADF Materials with Narrow Emission and Ciey < 0.06 Employing Host Materials as Donors. *New J. Chem.* **2023**, *47*, 3026-3036.

(10) Lv, X.; Huang, R.; Sun, S.; Zhang, Q.; Xiang, S.; Ye, S.; Leng, P.; Dias, F. B.; Wang, L. Blue TADF Emitters Based on Indenocarbazole Derivatives with High Photoluminescence and Electroluminescence Efficiencies. *ACS Appl. Mater. Interfaces* **2019**, *11*, 10758-10767.

(11) Lee, H. L.; Lee, K. H.; Lee, J. Y.; Hong, W. P. Management of Thermally Activated Delayed Fluorescence Using a Secondary Electron Accepting Unit in Thermally Activated Delayed Fluorescent Emitters. *J. Mater. Chem. C* **2019**, *7*, 6465-6474.

(12) Yun, J. H.; Lee, K. H.; Lee, J. Y. Propeller Type Dibenzofurocarbazole as a New Rigid Donor Moiety for Highly Efficient and Long Living Thermally Activated Delayed Fluorescence Emitters. *Chem. Eng. J.* **2020**, *400*, 125940.

(13) Raikwar, M. M.; Kim, S. C.; Lee, J. Y. Highly Efficient Thermally Activated Delayed Fluorescence Emitter Based on the 5h-Benzo[D]Benzo[4,5]Imidazo[1,2-a]Imidazole Donor. *Mater. Chem. Front.* **2022**, *6*, 3382-3390.

(14) Cui, L. S.; Nomura, H.; Geng, Y.; Kim, J. U.; Nakanotani, H.; Adachi, C. Controlling Singlet-Triplet Energy Splitting for Deep-Blue Thermally Activated Delayed Fluorescence Emitters. *Angew. Chem., Int. Ed.* **2017**, *56*, 1571-1575.

(15) Huang, W.; Einzinger, M.; Zhu, T.; Chae, H. S.; Jeon, S.; Ihn, S.-G.; Sim, M.; Kim, S.; Su, M.; Teverovskiy, G.et al. Molecular Design of Deep Blue Thermally Activated Delayed Fluorescence Materials Employing a Homoconjugative Triptycene Scaffold and Dihedral Angle Tuning. *Chem. Mater.* **2018**, *30*, 1462-1466.

(16) Wang, Q.; Zhang, Y.-X.; Yuan, Y.; Hu, Y.; Tian, Q.-S.; Jiang, Z.-Q.; Liao, L.-S. Alleviating Efficiency Roll-Off of Hybrid Single-Emitting Layer WOLED Utilizing Bipolar TADF Material as Host and Emitter. *ACS Appl. Mater. Interfaces* **2019**, *11*, 2197-2204.

(17) Huang, W.; Einzinger, M.; Maurano, A.; Zhu, T.; Tiepelt, J.; Yu, C.; Chae, H. S.; Van Voorhis, T.; Baldo, M. A.; Buchwald, S. L. Large Increase in External Quantum Efficiency by Dihedral Angle Tuning in a Sky-Blue Thermally Activated Delayed Fluorescence Emitter. *Adv. Optical Mater.* **2019**, *7*, 1900476.

(18) Min, H.; Park, I. S.; Yasuda, T. Dipolar and Quadrupolar Luminophores Based on 1,8-Dimethylcarbazole−Triazine Conjugates for High-Efficiency Blue Thermally Activated Delayed Fluorescence OLEDs. *ChemPhotoChem* **2020**, *4*, 82-88.

(19) Jung, M.; Lee, K. H.; Lee, J. Y. Molecular Engineering of Isomeric Benzofurocarbazole Donors for Photophysical Management of Thermally Activated Delayed Fluorescence Emitters. *Chem. Eur. J.* **2020**, *26*, 4816-4821.

(20) Jeon, S. O.; Lee, K. H.; Kim, J. S.; Ihn, S.-G.; Chung, Y. S.; Kim, J. W.; Lee, H.; Kim, S.; Choi, H.; Lee, J. Y. High-Efficiency, Long-Lifetime Deep-Blue Organic Light-Emitting Diodes. *Nat. Photonics* **2021**, *15*, 208-215.

(21) Li, W.; Cai, X.; Li, B.; Gan, L.; He, Y.; Liu, K.; Chen, D.; Wu, Y.-C.; Su, S.-J. Adamantane-Substituted Acridine Donor for Blue Dual Fluorescence and Efficient Organic Light-Emitting Diodes. *Angew. Chem., Int. Ed.* **2019**, *58*, 582-586.

(22) Tsai, W.-L.; Huang, M.-H.; Lee, W.-K.; Hsu, Y.-J.; Pan, K.-C.; Huang, Y.-H.; Ting, H.-C.; Sarma, M.; Ho, Y.-Y.; Hu, H.-C.et al. A Versatile Thermally Activated Delayed Fluorescence Emitter for Both Highly Efficient Doped and Non-Doped Organic Light Emitting Devices. *Chem. Commun.* **2015**, *51*, 13662-13665.

(23) Sun, J. W.; Baek, J. Y.; Kim, K. H.; Huh, J. S.; Kwon, S. K.; Kim, Y. H.; Kim, J. J. Azasiline-Based Thermally Activated Delayed Fluorescence Emitters for Blue Organic Light Emitting Diodes. *J. Mater. Chem. C* **2017**, *5*, 1027-1032.

(24) Woo, S. J.; Kim, Y.; Kwon, S. K.; Kim, Y. H.; Kim, J. J. Phenazasiline/Spiroacridine Donor Combined with Methyl-Substituted Linkers for Efficient Deep Blue Thermally Activated Delayed Fluorescence Emitters. *ACS Appl. Mater. Interfaces* **2019**, *11*, 7199-7207.

(25) Woo, S.-J.; Kim, Y.; Kim, Y.-H.; Kwon, S.-K.; Kim, J.-J. A Spiro-Silafluorene–Phenazasiline Donor-Based Efficient Blue Thermally Activated Delayed Fluorescence Emitter and Its Host-Dependent Device Characteristics. *J. Mater. Chem. C* **2019**, *7*, 4191-4198.

(26) Li, W.; Li, B.; Cai, X.; Gan, L.; Xu, Z.; Li, W.; Liu, K.; Chen, D.; Su, S. J. Tri-Spiral Donor for High Efficiency and Versatile Blue Thermally Activated Delayed Fluorescence Materials. *Angew. Chem., Int. Ed.* **2019**, *58*, 11301-11305.

(27) Li, W.; Li, W.; Gan, L.; Li, M.; Zheng, N.; Ning, C.; Chen, D.; Wu, Y. C.; Su, S. J. J-Aggregation Enhances the Electroluminescence Performance of a Sky-Blue Thermally Activated Delayed-Fluorescence Emitter in Nondoped Organic Light-Emitting Diodes. *ACS Appl. Mater. Interfaces* **2020**, *12*, 2717-2723.

(28) Kang, H.; Ihn, S.-G.; Kim, I.; Chung, Y. S.; Jeon, S. O.; Sim, M.; Kim, J.; Lee, H.; Son, Y.; Son, W.-J.et al. Designing Stable Deep-Blue Thermally Activated Delayed Fluorescence Emitters through Controlling the Intrinsic Stability of Triplet Excitons. *Adv. Optical Mater.* **2022**, *10*, 2102309.

(29) Wada, Y.; Kubo, S.; Kaji, H. Adamantyl Substitution Strategy for Realizing Solution-Processable Thermally Stable Deep-Blue Thermally Activated Delayed Fluorescence Materials. *Adv. Mater.* **2018**, *30*, 1705641.

(30) Lee, Y.; Woo, S.-J.; Kim, J.-J.; Hong, J.-I. Blue Thermally Activated Delayed Fluorescence Emitter Using Modulated Triazines as Electron Acceptors. *Dyes Pigm.* **2020**, *172*, 107864.

(31) Fan, X.; Li, C.; Wang, Z.; Wei, Y.; Duan, C.; Han, C.; Xu, H. Enhancing Reverse Intersystem Crossing Via Secondary Acceptors: Toward Sky-Blue Fluorescent Diodes with 10-Fold Improved External Quantum Efficiency. *ACS Appl. Mater. Interfaces* **2019**, *11*, 4185-4192.

(32) Kim, G. H.; Lampande, R.; Im, J. B.; Lee, J. M.; Lee, J. Y.; Kwon, J. H. Controlling the Exciton Lifetime of Blue Thermally Activated Delayed Fluorescence Emitters Using a Heteroatom-Containing Pyridoindole Donor Moiety. *Mater. Horiz.* **2017**, *4*, 619-624.

(33) Youn, K. M.; Ahn, D. H.; Kim, G. H.; Karthik, D.; Lee, J. Y.; Kwon, J. H. Blue Thermally Activated Delayed Fluorescence Emitters with a Δ-Pyridoindole Donor Moiety. *New J. Chem.* **2018**, *42*, 5532-5539.

(34) Cha, J.-R.; Lee, C. W.; Gong, M.-S. Effect of Increasing Electron Donor Units for High-Efficiency Blue Thermally Activated Delayed Fluorescence. *Dyes Pigm.* **2017**, *140*, 399-406.

(35) Oh, C. S.; Pereira, D. D. S.; Han, S. H.; Park, H.-J.; Higginbotham, H. F.; Monkman, A. P.; Lee, J. Y. Dihedral Angle Control of Blue Thermally-Activated Delayed Fluorescent Emitters through Donor Substitution Position for Efficient Reverse Intersystem Crossing. *ACS Appl. Mater. Interfaces* **2018**, *10*, 35420-35429.

(36) Oh, C. S.; Lee, H. L.; Han, S. H.; Lee, J. Y. Rational Molecular Design Overcoming the Long Delayed Fluorescence Lifetime and Serious Efficiency Roll-Off in Blue Thermally Activated Delayed Fluorescent Devices. *Chem. Eur. J.* **2019**, *25*, 642-648.

(37) Cui, L.-S.; Gillett, A. J.; Zhang, S.-F.; Ye, H.; Liu, Y.; Chen, X.-K.; Lin, Z.-S.; Evans, E. W.; Myers, W. K.; Ronson, T. K.et al. Fast Spin-Flip Enables Efficient and Stable Organic Electroluminescence from Charge-Transfer States. *Nat. Photonics* **2020**, *14*, 636–642.

(38) Lee, D. R.; Han, S. H.; Lee, C. W.; Lee, J. Y. Bis(Diphenyltriazine) as a New Acceptor of Efficient Thermally Activated Delayed Fluorescent Emitters. *Dyes Pigm.* **2018**, *151*, 75-80.

(39) Lee, H. L.; Lee, K. H.; Lee, J. Y. Transformation from Nonthermally Activated Delayed Fluorescence Molecules to Thermally Activated Delayed Fluorescence Molecules. *Adv. Optical Mater.* **2020**, *8*, 2001025.

(40) Lin, C. Y.; Lu, C. H.; Kuo, K. H.; Wang, M.; Tang, Y.; Dou, Y.; Hu, B.; Wu, C. C.; Wong, K. T. Highly Efficient Blue Thermally Activated Delayed Fluorescence Emitters with a Triphenylamine-Based Macrocyclic Donor. *Adv. Optical Mater.* **2023**, *11*, 2202292.

(41) Fang, Z.; Wang, S.; Liao, J.; Chen, X.; Zhu, Y.; Zhu, W.; Wang, Y. Asymmetric Sky-Blue Thermally-Activated Delayed Fluorescence Emitters Bearing Tris(Triazolo)Triazine Moiety for Solution-Processable Organic Light-Emitting Diodes. *J. Mater. Chem. C* **2022**, *10*, 4837-4844.

(42) Pathak, S. K.; Xiang, Y.; Huang, M.; Huang, T.; Cao, X.; Liu, H.; Xie, G.; Yang, C. Fused Tetracyclic Tris[1,2,4]Triazolo[1,3,5]Triazine as a Novel Rigid Electron Acceptor for Efficient Thermally Activated Delayed Fluorescence Emitters. *RSC Adv.* **2020**, *10*, 15523-15529.

(43) Hundemer, F.; Crovini, E.; Wada, Y.; Kaji, H.; Bräse, S.; Zysman-Colman, E. Tris(Triazolo)Triazine-Based Emitters for Solution-Processed Blue Thermally Activated Delayed Fluorescence Organic Light-Emitting Diodes. *Mater. Adv.* **2020**, *1*, 2862-2871.

(44) Komatsu, R.; Ohsawa, T.; Sasabe, H.; Nakao, K.; Hayasaka, Y.; Kido, J. Manipulating the Electronic Excited State Energies of Pyrimidine-Based Thermally Activated Delayed Fluorescence Emitters to Realize Efficient Deep-Blue Emission. *ACS Appl. Mater. Interfaces* **2017**, *9*, 4742-4749.

(45) Serevičius, T.; Dodonova, J.; Skaisgiris, R.; Banevičius, D.; Kazlauskas, K.; Juršėnas, S.; Tumkevičius, S. Optimization of the Carbazole–Pyrimidine Linking Pattern for Achieving Efficient TADF. *J. Mater. Chem. C* **2020**, *8*, 11192-11200.
(46) Li, B.; Li, Z.; Hu, T.; Zhang, Y.; Wang, Y.; Yi, Y.; Guo, F.; Zhao, L. Highly Efficient Blue Organic Light-Emitting Diodes from Pyrimidine-Based Thermally Activated Delayed Fluorescence Emitters. *J. Mater. Chem. C* **2018**, *6*, 2351-2359.
(47) Park, I. S.; Komiyama, H.; Yasuda, T. Pyrimidine-Based Twisted Donor–Acceptor Delayed Fluorescence Molecules: A New Universal Platform for Highly Efficient Blue Electroluminescence. *Chem. Sci.* **2017**, *8*, 953–960.
(48) Sohn, S.; Ha, M. W.; Park, J.; Kim, Y. H.; Ahn, H.; Jung, S.; Kwon, S. K.; Kim, Y. H. High-Efficiency Diphenylpyrimidine Derivatives Blue Thermally Activated Delayed Fluorescence Organic Light-Emitting Diodes. *Front. Chem.* **2020**, *8*, 356.
(49) Nakao, K.; Sasabe, H.; Komatsu, R.; Hayasaka, Y.; Ohsawa, T.; Kido, J. Significant Enhancement of Blue OLED Performances through Molecular Engineering of Pyrimidine-Based Emitter. *Adv. Optical Mater.* **2017**, *5*, 1600843.
(50) Ganesan, P.; Chen, D.-G.; Liao, J.-L.; Li, W.-C.; Lai, Y.-N.; Luo, D.; Chang, C.-H.; Ko, C.-L.; Hung, W.-Y.; Liu, S.-W.et al. Isomeric Spiro-[Acridine-9,9′-Fluorene]-2,6-Dipyridylpyrimidine Based TADF Emitters: Insights into Photophysical Behaviors and OLED Performances. *J. Mater. Chem. C* **2018**, *6*, 10088-10100.
(51) Zhang, Q.; Sun, S.; Lv, X.; Liu, W.; Zeng, H.; Guo, R.; Ye, S.; Leng, P.; Xiang, S.; Wang, L. Manipulating the Positions of Ch···N in Acceptors of Pyrimidine–Pyridine Hybrids for Highly Efficient Sky-Blue Thermally Activated Delayed Fluorescent OLEDs. *Mater. Chem. Front.* **2018**, *2*, 2054-2062.
(52) Lv, X.; Sun, S.; Zhang, Q.; Ye, S.; Liu, W.; Wang, Y.; Guo, R.; Wang, L. A Strategy to Construct Multifunctional TADF Materials for Deep Blue and High Efficiency Yellow Fluorescent Devices. *J. Mater. Chem. C* **2020**, *8*, 4818-4826.
(53) Zhang, Q.; Xiang, S.; Huang, Z.; Sun, S.; Ye, S.; Lv, X.; Liu, W.; Guo, R.; Wang, L. Molecular Engineering of Pyrimidine-Containing Thermally Activated Delayed Fluorescence Emitters for Highly Efficient Deep-Blue (Cie Y < 0.06) Organic Light-Emitting Diodes. *Dyes Pigm.* **2018**, *155*, 51-58.
(54) Cai, M.; Auffray, M.; Zhang, D.; Zhang, Y.; Nagata, R.; Lin, Z.; Tang, X.; Chan, C.-Y.; Lee, Y.-T.; Huang, T.et al. Enhancing Spin-Orbital Coupling in Deep-Blue/Blue TADF Emitters by Minimizing the Distance from the Heteroatoms in Donors to Acceptors. *Chem. Eng. J.* **2021**, *420*, 127591.
(55) Banevičius, D.; Kreiza, G.; Klioštoraitis, R.; Juršėnas, S.; Javorskis, T.; Vaitkevičius, V.; Orentas, E.; Kazlauskas, K. Enhanced Blue TADF in a D–a–D Type Naphthyridine Derivative with an Asymmetric Carbazole-Donor Motif. *J. Mater. Chem. C* **2022**, *10*, 4813-4820.
(56) Mahmoudi, M.; Gudeika, D.; Kutsiy, S.; Simokaitiene, J.; Butkute, R.; Skhirtladze, L.; Woon, K. L.; Volyniuk, D.; Grazulevicius, J. V. Ornamenting of Blue Thermally Activated Delayed Fluorescence Emitters by Anchor Groups for the Minimization of Solid-State Solvation and Conformation Disorder Corollaries in Non-Doped and Doped Organic Light-Emitting Diodes. *ACS Appl. Mater. Interfaces* **2022**, *14*, 40158-40172.
(57) Lee, Y. H.; Park, S.; Oh, J.; Shin, J. W.; Jung, J.; Yoo, S.; Lee, M. H. Rigidity-Induced Delayed Fluorescence by Ortho Donor-Appended Triarylboron Compounds: Record-High Efficiency in Pure Blue Fluorescent Organic Light-Emitting Diodes. *ACS Appl. Mater. Interfaces* **2017**, *9*, 24035-24042.
(58) Wu, T.-L.; Lo, S.-H.; Chang, Y.-C.; Huang, M.-J.; Cheng, C.-H. Steric Switching for Thermally Activated Delayed Fluorescence by Controlling the Dihedral Angles between Donor and Acceptor in Organoboron Emitters. *ACS Appl. Mater. Interfaces* **2019**, *11*, 10768-10776.
(59) Park, I. S.; Matsuo, K.; Aizawa, N.; Yasuda, T. High-Performance Dibenzoheteraborin-Based Thermally Activated Delayed Fluorescence Emitters: Molecular Architectonics for Concurrently Achieving Narrowband Emission and Efficient Triplet–Singlet Spin Conversion. *Adv. Funct. Mater.* **2018**, *28*, 1802031.

(60) Matsuo, K.; Yasuda, T. Blue Thermally Activated Delayed Fluorescence Emitters Incorporating Acridan Analogues with Heavy Group 14 Elements for High-Efficiency Doped and Non-Doped OLEDs. *Chem. Sci.* **2019**, *10*, 10687-10697.

(61) Ahn, D. H.; Lee, H.; Kim, S. W.; Karthik, D.; Lee, J.; Jeong, H.; Lee, J. Y.; Kwon, J. H. Highly Twisted Donor-Acceptor Boron Emitter and High Triplet Host Material for Highly Efficient Blue Thermally Activated Delayed Fluorescent Device. *ACS Appl. Mater. Interfaces* **2019**, *11*, 14909-14916.

(62) Min, H.; Park, I. S.; Yasuda, T. Blue Thermally Activated Delayed Fluorescence with Sub-Microsecond Short Exciton Lifetimes: Acceleration of Triplet–Singlet Spin Interconversion Via Quadrupolar Charge-Transfer States. *Adv. Optical mater.* **2022**, *10*, 2200290.

(63) Agou, T.; Matsuo, K.; Kawano, R.; Park, I. S.; Hosoya, T.; Fukumoto, H.; Kubota, T.; Mizuhata, Y.; Tokitoh, N.; Yasuda, T. Pentacyclic Ladder-Heteraborin Emitters Exhibiting High-Efficiency Blue Thermally Activated Delayed Fluorescence with an Ultrashort Emission Lifetime. *ACS Mater. Lett.* **2019**, *2*, 28-34.

(64) Matsuo, K.; Yasuda, T. Boronate- and Borinate-Based Pi-Systems for Blue Thermally Activated Delayed Fluorescence Materials. *Chem. Commun.* **2019**, *55*, 2501-2504.

(65) Li, G.; Lou, W.; Wang, D.; Deng, C.; Zhang, Q. Difluoroboron-Enabled Thermally Activated Delayed Fluorescence. *ACS Appl. Mater. Interfaces* **2019**, *11*, 32209-32217.

(66) Ahn, D. H.; Kim, S. W.; Lee, H.; Ko, I. J.; Karthik, D.; Lee, J. Y.; Kwon, J. H. Highly Efficient Blue Thermally Activated Delayed Fluorescence Emitters Based on Symmetrical and Rigid Oxygen-Bridged Boron Acceptors. *Nat. Photonics* **2019**, *13*, 540-546.

(67) Ahn, D. H.; Maeng, J. H.; Lee, H.; Yoo, H.; Lampande, R.; Lee, J. Y.; Kwon, J. H. Rigid Oxygen-Bridged Boron-Based Blue Thermally Activated Delayed Fluorescence Emitter for Organic Light-Emitting Diode: Approach Towards Satisfying High Efficiency and Long Lifetime Together. *Adv. Optical Mater.* **2020**, *8*, 2000102.

(68) Naveen, K. R.; Lee, H.; Braveenth, R.; Karthik, D.; Yang, K. J.; Hwang, S. J.; Kwon, J. H. Achieving High Efficiency and Pure Blue Color in Hyperfluorescence Organic Light Emitting Diodes Using Organo-Boron Based Emitters. *Adv. Funct. Mater.* **2022**, *32*, 2110356.

(69) Liu, Y.; Du, B.; Han, X.; Wu, X.; Tong, H.; Wang, L. Intramolecular-Locked Triazatruxene-Based Thermally Activated Delayed Fluorescence Emitter for Efficient Solution-Processed Deep-Blue Organic Light Emitting Diodes. *Chem. Eng. J.* **2022**, *446*, 137372.

(70) Kim, H. J.; Godumala, M.; Kim, S. K.; Yoon, J.; Kim, C. Y.; Park, H.; Kwon, J. H.; Cho, M. J.; Choi, D. H. Color-Tunable Boron-Based Emitters Exhibiting Aggregation-Induced Emission and Thermally Activated Delayed Fluorescence for Efficient Solution-Processable Nondoped Deep-Blue to Sky-Blue OLEDs. *Adv. Optical Mater.* **2020**, *8*, 1902175.

(71) Karthik, D.; Ahn, D. H.; Ryu, J. H.; Lee, H.; Maeng, J. H.; Lee, J. Y.; Kwon, J. H. Highly Efficient Blue Thermally Activated Delayed Fluorescence Organic Light Emitting Diodes Based on Tercarbazole Donor and Boron Acceptor Dyads. *J. Mater. Chem. C* **2020**, *8*, 2272-2279.

(72) Kim, J. U.; Park, I. S.; Chan, C. Y.; Tanaka, M.; Tsuchiya, Y.; Nakanotani, H.; Adachi, C. Nanosecond-Time-Scale Delayed Fluorescence Molecule for Deep-Blue OLEDs with Small Efficiency Rolloff. *Nat. Commun.* **2020**, *11*, 1765.

(73) Tan, H. J.; Yang, G. X.; Deng, Y. L.; Cao, C.; Tan, J. H.; Zhu, Z. L.; Chen, W. C.; Xiong, Y.; Jian, J. X.; Lee, C. S.; Tong, Q. X. Deep-Blue OLEDs with Rec.2020 Blue Gamut Compliance and EQE over 22% Achieved by Conformation Engineering. *Adv. Mater.* **2022**, *34*, 2200537.

(74) Park, I. S.; Min, H.; Kim, J. U.; Yasuda, T. Deep-Blue OLEDs Based on Organoboron–Phenazasiline-Hybrid Delayed Fluorescence Emitters Concurrently Achieving 30% External Quantum Efficiency and Small Efficiency Roll-Off. *Adv. Optical Mater.* **2021**, *9*, 2101282.

(75) Hua, T.; Liu, Y.-C.; Huang, C.-W.; Li, N.; Zhou, C.; Huang, Z.; Cao, X.; Wu, C.-C.; Yang, C. High-Efficiency and Low Roll-Off Deep-Blue OLEDs Enabled by Thermally Activated Delayed Fluorescence Emitter with Preferred Horizontal Dipole Orientation. *Chem. Eng. J.* **2021**, *433*, 133598.

(76) Meng, G.; Chen, X.; Wang, X.; Wang, N.; Peng, T.; Wang, S. Isomeric Bright Sky-Blue TADF Emitters Based on Bisacridine Decorated Dbna: Impact of Donor Locations on Luminescent and Electroluminescent Properties. *Adv. Optical Mater.* **2019**, *7*, 1900130.

(77) Han, J.; Huang, Z.; Miao, J.; Qiu, Y.; Xie, Z.; Yang, C. Narrowband Blue Emission with Insensitivity to the Doping Concentration from an Oxygen-Bridged Triarylboron-Based TADF Emitter: Nondoped OLEDs with a High External Quantum Efficiency up to 21.4. *Chem. Sci.* **2022**, *13*, 3402-3408.

(78) Meng, X.-Y.; Feng, Z.-Q.; Yu, Y.-J.; Liao, L.-S.; Jiang, Z.-Q. Highly Efficient Blue Thermally Activated Delayed Fluorescence Emitters Based on Multi-Donor Modified Oxygen-Bridged Boron Acceptor. *Molecules* **2022**, *27*, 4048.

(79) Gao, H.; Shen, S.; Qin, Y.; Liu, G.; Gao, T.; Dong, X.; Pang, Z.; Xie, X.; Wang, P.; Wang, Y. Ultrapure Blue Thermally Activated Delayed Fluorescence (TADF) Emitters Based on Rigid Sulfur/Oxygen-Bridged Triarylboron Acceptor: Mr TADF and D-a TADF. *J. Phys. Chem. Lett* **2022**, *13*, 7561-7567.

(80) Chan, C. Y.; Cui, L. S.; Kim, J. U.; Nakanotani, H.; Adachi, C. Rational Molecular Design for Deep-Blue Thermally Activated Delayed Fluorescence Emitters. *Adv. Funct. Mater.* **2018**, *28*, 1706023.

(81) Cheng, Z.; Li, Z.; Xu, Y.; Liang, J.; Lin, C.; Wei, J.; Wang, Y. Achieving Efficient Blue Delayed Electrofluorescence by Shielding Acceptors with Carbazole Units. *ACS Appl. Mater. Interfaces* **2019**, *11*, 28096-28105.

(82) Park, H.-J.; Lee, H. L.; Lee, H. J.; Lee, K. H.; Lee, J. Y.; Hong, W. P. Peripheral Decoration of Dibenzofuran with Donors and Acceptors as a New Design Platform for Thermally Activated Delayed Fluorescence Emitters. *Chem. Mater.* **2019**, *31*, 10023-10031.

(83) Chan, C.-Y.; Tanaka, M.; Nakanotani, H.; Adachi, C. Efficient and Stable Sky-Blue Delayed Fluorescence Organic Light-Emitting Diodes with Ciey Below 0.4. *Nat. Commun.* **2018**, *9*, 5036.

(84) Zhang, W.; Zhang, Y.-X.; Zhang, X.-Q.; Liu, X.-Y.; Fan, J.; Liao, L.-S. Blue Thermally Activated Delayed Fluorescence Materials Based on Bi/Tri-Carbazole Derivatives. *Org. Electron.* **2018**, *58*, 238-244.

(85) Zou, S. J.; Xie, F. M.; Xie, M.; Li, Y. Q.; Cheng, T.; Zhang, X. H.; Lee, C. S.; Tang, J. X. High-Performance Nondoped Blue Delayed Fluorescence Organic Light-Emitting Diodes Featuring Low Driving Voltage and High Brightness. *Adv. Sci.* **2020**, *7*, 1902508.

(86) Xie, F.-M.; An, Z.-D.; Xie, M.; Li, Y.-Q.; Zhang, G.-H.; Zou, S.-J.; Chen, L.; Chen, J.-D.; Cheng, T.; Tang, J.-X. Tert-Butyl Substituted Hetero-Donor TADF Compounds for Efficient Solution-Processed Non-Doped Blue OLEDs. *J. Mater. Chem. C* **2020**, *8*, 5769-5776.

(87) Zhang, D.; Song, X.; Gillett, A. J.; Drummond, B. H.; Jones, S. T. E.; Li, G.; He, H.; Cai, M.; Credgington, D.; Duan, L. Efficient and Stable Deep-Blue Fluorescent Organic Light-Emitting Diodes Employing a Sensitizer with Fast Triplet Upconversion. *Adv. Mater.* **2020**, *32*, 1908355.

(88) Mamada, M.; Katagiri, H.; Chan, C. Y.; Lee, Y. T.; Goushi, K.; Nakanotani, H.; Hatakeyama, T.; Adachi, C. Highly Efficient Deep-Blue Organic Light-Emitting Diodes Based on Rational Molecular Design and Device Engineering. *Adv. Funct. Mater.* **2022**, *32*, 2204352.

(89) Zheng, X.; Huang, R.; Zhong, C.; Xie, G.; Ning, W.; Huang, M.; Ni, F.; Dias, F. B.; Yang, C. Achieving 21% External Quantum Efficiency for Nondoped Solution-Processed Sky-Blue Thermally Activated Delayed Fluorescence OLEDs by Means of Multi-(Donor/Acceptor) Emitter with through-Space/-Bond Charge Transfer. *Adv. Sci.* **2020**, *7*, 1902087.

(90) Wong, M. Y.; Krotkus, S.; Copley, G.; Li, W.; Murawski, C.; Hall, D.; Hedley, G. J.; Jaricot, M.; Cordes, D. B.; Slawin, A. M. Z.et al. Deep-Blue Oxadiazole-Containing Thermally Activated Delayed Fluorescence Emitters for Organic Light-Emitting Diodes. *ACS Appl. Mater. Interfaces* **2018**, *10*, 33360-33372.

(91) Cooper, M. W.; Zhang, X.; Zhang, Y.; Jeon, S. O.; Lee, H.; Kim, S.; Fuentes-Hernandez, C.; Barlow, S.; Kippelen, B.; Marder, S. R. Effect of the Number and Substitution Pattern of Carbazole Donors on the Singlet and Triplet State Energies in a Series of Carbazole-Oxadiazole Derivatives Exhibiting Thermally Activated Delayed Fluorescence. *Chem. Mater.* **2018**, *30*, 6389-6399.

(92) Cooper, M. W.; Zhang, X.; Zhang, Y.; Ashokan, A.; Fuentes-Hernandez, C.; Salman, S.; Kippelen, B.; Barlow, S.; Marder, S. R. Delayed Luminescence in 2-Methyl-5-(Penta(9-Carbazolyl)Phenyl)-1,3,4-Oxadiazole Derivatives. *J. Phys. Chem. A* **2022**, *126*, 7480-7490.
(93) Tan, Y.; Rui, B.; Li, J.; Zhao, Z.; Liu, Z.; Bian, Z.; Huang, C. Blue Thermally Activated Delayed Fluorescence Emitters Based on a Constructing Strategy with Diversed Donors and Oxadiazole Acceptor and Their Efficient Electroluminescent Devices. *Opt. Mater.* **2019**, *94*, 103-112.
(94) Liu, M.; Komatsu, R.; Cai, X.; Sasabe, H.; Kamata, T.; Nakao, K.; Liu, K.; Su, S.-J.; Kido, J. Introduction of Twisted Backbone: A New Strategy to Achieve Efficient Blue Fluorescence Emitter with Delayed Emission. *Adv. Optical Mater.* **2017**, *5*, 1700334.
(95) Chen, D.; Liu, K.; Li, X.-L.; Li, B.; Liu, M.; Cai, X.; Ma, Y.; Cao, Y.; Su, S.-J. Engineering Excited-State Properties of Intramolecular and Intermolecular Charge Transfer Purely Organic Emitters Towards High-Performance Fluorescent OLEDs. *J. Mater. Chem. C* **2017**, *5*, 10991-11000.
(96) Jürgensen, N.; Kretzschmar, A.; Höfle, S.; Freudenberg, J.; Bunz, U. H. F.; Hernandez-Sosa, G. Sulfone-Based Deep Blue Thermally Activated Delayed Fluorescence Emitters: Solution-Processed Organic Light-Emitting Diodes with High Efficiency and Brightness. *Chem. Mater.* **2017**, *29*, 9154-9161.
(97) Rao, J.; Zhao, C.; Wang, Y.; Bai, K.; Wang, S.; Ding, J.; Wang, L. Achieving Deep-Blue Thermally Activated Delayed Fluorescence in Nondoped Organic Light-Emitting Diodes through a Spiro-Blocking Strategy. *ACS Omega* **2019**, *4*, 1861-1867.
(98) Zeng, X.; Pan, K.-C.; Lee, W.-K.; Gong, S.; Ni, F.; Xiao, X.; Zeng, W.; Xiang, Y.; Zhan, L.; Zhang, Y.et al. High-Efficiency Pure Blue Thermally Activated Delayed Fluorescence Emitters with a Preferentially Horizontal Emitting Dipole Orientation Via a Spiro-Linked Double D–a Molecular Architecture. *J. Mater. Chem. C* **2019**, *7*, 10851-10859.
(99) Jeon, Y. P.; Kong, B. K.; Lee, E. J.; Yoo, K.-H.; Kim, T. W. Ultrahighly-Efficient and Pure Deep-Blue Thermally Activated Delayed Fluorescence Organic Light-Emitting Devices Based on Dimethylacridine/Thioxanthene-S,S-Dioxide. *Nano Energy* **2019**, *59*, 560-568.
(100) Stachelek, P.; Ward, J. S.; dos Santos, P. L.; Danos, A.; Colella, M.; Haase, N.; Raynes, S. J.; Batsanov, A. S.; Bryce, M. R.; Monkman, A. P. Molecular Design Strategies for Color Tuning of Blue TADF Emitters. *ACS Appl. Mater. Interfaces* **2019**, *11*, 27125-27133.
(101) Luo, Y.; Li, S.; Zhao, Y.; Li, C.; Pang, Z.; Huang, Y.; Yang, M.; Zhou, L.; Zheng, X.; Pu, X.; Lu, Z. An Ultraviolet Thermally Activated Delayed Fluorescence OLED with Total External Quantum Efficiency over 9%. *Adv. Mater.* **2020**, *32*, 2001248.
(102) Sun, S.; Guo, R.; Zhang, Q.; Lv, X.; Leng, P.; Wang, Y.; Huang, Z.; Wang, L. Efficient Deep-Blue Thermally Activated Delayed Fluorescence Emitters Based on Diphenylsulfone-Derivative Acceptor. *Dyes Pigm.* **2020**, *178*, 108367.
(103) Zhan, L.; Xiang, Y.; Chen, Z.; Wu, K.; Gong, S.; Xie, G.; Yang, C. Fine-Tuning the Photophysical Properties of Thermally Activated Delayed Fluorescent Emitters Using Torsion Angles: High Performance Sky-Blue OLEDs. *J. Mater. Chem. C* **2019**, *7*, 13953-13959.
(104) Hu, Y.; Yao, J.; Xu, Z.; Wang, Z.; Li, L.; Su, S. J.; Ma, D.; Huang, F. Three-Dimensional Organic Cage with Narrowband Delayed Fluorescence. *Sci. China Chem.* **2020**, *63*, 897-903.
(105) Wang, J.; Zhang, J.; Jiang, C.; Yao, C.; Xi, X. Effective Design Strategy for Aggregation-Induced Emission and Thermally Activated Delayed Fluorescence Emitters Achieving 18% External Quantum Efficiency Pure-Blue OLEDs with Extremely Low Roll-Off. *ACS Appl. Mater. Interfaces* **2021**, *13*, 57713-57724.
(106) Zhu, Y.; Zeng, S.; Gong, W.; Chen, X.; Xiao, C.; Ma, H.; Zhu, W.; Yeob Lee, J.; Wang, Y. Molecular Design of Blue Thermally Activated Delayed Fluorescent Emitters for High Efficiency Solution Processable OLED Via an Intramolecular Locking Strategy. *Chem. Eng. J.* **2022**, *450*, 138459.
(107) Sharif, P.; Alemdar, E.; Ozturk, S.; Caylan, O.; Haciefendioglu, T.; Buke, G.; Aydemir, M.; Danos, A.; Monkman, A. P.; Yildirim, E.et al. Rational Molecular Design Enables Efficient Blue TADF−OLEDs with Flexible Graphene Substrate. *Adv. Funct. Mater.* **2022**, *32*, 2207324.

(108) Ryoo, C. H.; Han, J.; Yang, J. h.; Yang, K.; Cho, I.; Jung, S.; Kim, S.; Jeong, H.; Lee, C.; Kwon, J. E.et al. Systematic Substituent Control in Blue Thermally Activated Delayed Fluorescence (TADF) Emitters: Unraveling the Role of Direct Intersystem Crossing between the Same Charge-Transfer States. *Adv. Optical Mater.* **2022**, *10*, 2201622.

(109) Wang, J.; Yang, Y.; Jiang, C.; He, M.; Yao, C.; Zhang, J. Ultrapure Deep-Blue Aggregation-Induced Emission and Thermally Activated Delayed Fluorescence Emitters for Efficient OLEDs with Ciey < 0.1 and Low Efficiency Roll-Offs. *J. Mater. Chem. C* **2022**, *10*, 3163-3171.

(110) Lee, J.; Aizawa, N.; Yasuda, T. Isobenzofuranone- and Chromone-Based Blue Delayed Fluorescence Emitters with Low Efficiency Roll-Off in Organic Light-Emitting Diodes. *Chem. Mater.* **2017**, *29*, 8012-8020.

(111) Shizu, K.; Miwa, T.; Wada, Y.; Ogata, I.; Kaji, H. Thermally Activated Delayed Fluorescence Emitter with a Symmetric Acceptor-Donor-Acceptor Structure. *J. Photopolym. Sci. Technol.* **2017**, *30*, 475-481.

(112) Miwa, T.; Kubo, S.; Shizu, K.; Komino, T.; Adachi, C.; Kaji, H. Blue Organic Light-Emitting Diodes Realizing External Quantum Efficiency over 25% Using Thermally Activated Delayed Fluorescence Emitters. *Sci. Rep.* **2017**, *7*, 284.

(113) Rajamalli, P.; Senthilkumar, N.; Huang, P. Y.; Ren-Wu, C. C.; Lin, H. W.; Cheng, C. H. New Molecular Design Concurrently Providing Superior Pure Blue, Thermally Activated Delayed Fluorescence and Optical out-Coupling Efficiencies. *J. Am. Chem. Soc.* **2017**, *139*, 10948-10951.

(114) Rajamalli, P.; Thangaraji, V.; Natarajan, S.; Chen-Cheng, R.-W.; Lin, H.-W.; Cheng, C. H. Thermally Activated Delayed Fluorescence Emitters with Am, M-Di-Tert-Butyl-Carbazolyl Benzoylpyridine Core Achieving Extremely High Blue Electroluminescence Efficiencies. *J. Mater. Chem. C* **2017**, *5*, 2919-2926.

(115) Kreiza, G.; Banevičius, D.; Jovaišaitė, J.; Maleckaitė, K.; Gudeika, D.; Volyniuk, D.; Gražulevičius, J. V.; Juršėnas, S.; Kazlauskas, K. Suppression of Benzophenone-Induced Triplet Quenching for Enhanced TADF Performance. *J. Mater. Chem. C* **2019**, *7*, 11522-11531.

(116) Min, H.; Park, I. S.; Yasuda, T. Blue Thermally Activated Delayed Fluorescence with Sub-Microsecond Short Exciton Lifetimes: Acceleration of Triplet–Singlet Spin Interconversion Via Quadrupolar Charge-Transfer States. *Adv. Optical Mater.* **2022**, *10*, 2200290.

(117) Zhang, D.; Wada, Y.; Wang, Q.; Dai, H.; Fan, T.; Meng, G.; Wei, J.; Zhang, Y.; Suzuki, K.; Li, G.et al. Highly Efficient and Stable Blue Organic Light-Emitting Diodes Based on Thermally Activated Delayed Fluorophor with Donor-Void-Acceptor Motif. *Adv. Sci.* **2022**, *9*, 2106018.

(118) Fu, Y.; Liu, H.; Yang, D.; Ma, D.; Zhao, Z.; Tang, B. Z. Boosting External Quantum Efficiency to 38.6% of Sky-Blue Delayed Fluorescence Molecules by Optimizing Horizontal Dipole Orientation. *Sci. Adv.* **2021**, *7*, eabj2504.

(119) Liang, Q.; Han, C.; Duan, C.; Xu, H. Blue Thermally Activated Delayed Fluorescence-Emitting Phosphine Oxide Hosts for Ultrasimple and Highly Efficient White Organic Light-Emitting Diodes. *Adv. Optical Mater.* **2018**, *6*, 1800020.

(120) Gao, F.; Du, R.; Han, C.; Zhang, J.; Wei, Y.; Lu, G.; Xu, H. High-Efficiency Blue Thermally Activated Delayed Fluorescence from Donor-Acceptor-Donor Systems Via the through-Space Conjugation Effect. *Chem. Sci.* **2019**, *10*, 5556-5567.

(121) Yuan, W.; Zhang, M.; Zhang, X.; Cao, X.; Sun, N.; Wan, S.; Tao, Y. The Electron Inductive Effect of Cf3 on Penta-Carbazole Containing Blue Emitters: Trade-Off between Color Purity and Luminescent Efficiency in TADF OLEDs. *Dyes Pigm.* **2018**, *159*, 151-157.

(122) Yi, C. L.; Ko, C. L.; Yeh, T. C.; Chen, C. Y.; Chen, Y. S.; Chen, D. G.; Chou, P. T.; Hung, W. Y.; Wong, K. T. Harnessing a New Co-Host System and Low Concentration of New TADF Emitters Equipped with Trifluoromethyl- and Cyano-Substituted Benzene as Core for High-Efficiency Blue OLEDs. *ACS Appl. Mater. Interfaces* **2020**, *12*, 2724-2732.

(123) Liang, X.; Han, H.-B.; Yan, Z.-P.; Liu, L.; Zheng, Y.-X.; Meng, H.; Huang, W. Versatile Functionalization of Trifluoromethyl Based Deep Blue Thermally Activated Delayed

Fluorescence Materials for Organic Light Emitting Diodes. *New J. Chem.* **2018**, *42*, 4317-4323.
(124) Seo, J.-A.; Im, Y.; Han, S.-H.; Lee, C. W.; Lee, J. Y. Unconventional Molecular Design Approach of High Efficiency Deep Blue Thermally Activated Delayed Fluorescent Emitters Using Indolocarbazole as an Acceptor. *ACS Appl. Mater. Interfaces* **2017**, *9*, 37864-37872.
(125) Patil, V. V.; Lee, K. H.; Lee, J. Y. Universal Blue Emitters for High Efficiency Thermally Activated Delayed Fluorescence and Fluorescent Organic Light-Emitting Diodes. *Dyes Pigm.* **2020**, *174*, 108070.
(126) Zhu, Y.; Qu, C.; Ye, J.; Xu, Y.; Zhang, Z.; Wang, Y. Donor-Acceptor Type of Fused-Ring Thermally Activated Delayed Fluorescence Compounds Constructed through an Oxygen-Containing Six-Membered Ring. *ACS Appl. Mater. Interfaces* **2022**, *14*, 47971-47980.
(127) Wang, D.-Q.; Zhang, M.; Wang, K.; Zheng, C.-J.; Shi, Y.-Z.; Chen, J.-X.; Lin, H.; Tao, S.-L.; Zhang, X.-H. Fine-Tuning the Emissions of Highly Efficient Thermally Activated Delayed Fluorescence Emitters with Different Linking Positions of Electron-Deficient Substituent Groups. *Dyes Pigm.* **2017**, *143*, 62-70.
(128) Chen, Z.-P.; Wang, D.-Q.; Zhang, M.; Wang, K.; Shi, Y.-Z.; Chen, J.-X.; Tao, W.-W.; Zheng, C.-J.; Tao, S.-L.; Zhang, X.-H. Optimization on Molecular Restriction for Highly Efficient Thermally Activated Delayed Fluorescence Emitters. *Adv. Optical Mater.* **2018**, *6*, 1800935.
(129) Wang, Y. F.; Li, M.; Zhao, W. L.; Shen, Y. F.; Lu, H. Y.; Chen, C. F. An Axially Chiral Thermally Activated Delayed Fluorescent Emitter with a Dual Emitting Core for a Highly Efficient Organic Light-Emitting Diode. *Chem. Commun.* **2020**, *56*, 9380-9383.
(130) Noda, H.; Nakanotani, H.; Adachi, C. Excited State Engineering for Efficient Reverse Intersystem Crossing. *Sci. Adv.* **2018**, *4*, eaao6910.
(131) Balijapalli, U.; Tanaka, M.; Auffray, M.; Chan, C. Y.; Lee, Y. T.; Tsuchiya, Y.; Nakanotani, H.; Adachi, C. Utilization of Multi-Heterodonors in Thermally Activated Delayed Fluorescence Molecules and Their High Performance Bluish-Green Organic Light-Emitting Diodes. *ACS Appl. Mater. Interfaces* **2020**, *12*, 9498-9506.
(132) Woo, S.-J.; Ha, Y.-H.; Kim, Y.-H.; Kim, J.-J. Effect of Ortho-Biphenyl Substitution on the Excited State Dynamics of a Multi-Carbazole TADF Molecule. *J. Mater. Chem. C* **2020**, *8*, 12075-12084.
(133) Zhang, M.; Zheng, C.-J.; Wang, K.; Shi, Y.-Z.; Yang, H.-Y.; Lin, H.; Tao, S.-L.; Zhang, X.-H. Efficient and Stable Single-Emitting-Layer White Organic Light-Emitting Diodes by Employing All Thermally Activated Delayed Fluorescence Emitters. *Org. Electron.* **2022**, *101*, 106415.
(134) Zhao, G.; Liu, D.; Wang, P.; Huang, X.; Chen, H.; Zhang, Y.; Zhang, D.; Jiang, W.; Sun, Y.; Duan, L. Exceeding 30 % External Quantum Efficiency in Non-Doped OLEDs Utilizing Solution Processable TADF Emitters with High Horizontal Dipole Orientation Via Anchoring Strategy. *Angew. Chem., Int. Ed.* **2022**, *61*, e202212861.
(135) Lien, Y. J.; Lin, T. C.; Yang, C. C.; Chiang, Y. C.; Chang, C. H.; Liu, S. H.; Chen, Y. T.; Lee, G. H.; Chou, P. T.; Lu, C. W.; Chi, Y. First N-Borylated Emitters Displaying Highly Efficient Thermally Activated Delayed Fluorescence and High-Performance OLEDs. *ACS Appl. Mater. Interfaces* **2017**, *9*, 27090-27101.
(136) Tsai, C.-C.; Huang, W.-C.; Chih, H.-Y.; Hsh, Y.-C.; Liao, C.-W.; Lin, C.-H.; Kang, Y.-X.; Chang, C.-H.; Chang, Y. J.; Lu, C.-W. Efficient Donor-Acceptor-Donor Borylated Compounds with Extremely Small Δest for Thermally Activated Delayed Fluorescence OLEDs. *Org. Electron.* **2018**, *63*, 166-174.
(137) Chen, D.-G.; Lin, T.-C.; Chen, C.-L.; Chen, Y.-T.; Chen, Y.-A.; Lee, G.-H.; Chou, P.-T.; Liao, C.-W.; Chiu, P.-C.; Chang, C.-H.et al. Optically Triggered Planarization of Boryl-Substituted Phenoxazine: Another Horizon of TADF Molecules and High-Performance OLEDs. *ACS Appl. Mater. Interfaces* **2018**, *10*, 12886-12896.
(138) Qu, C.; Xia, G.; Xu, Y.; Zhu, Y.; Liang, J.; Zhang, H.; Wang, J.; Zhang, Z.; Wang, Y. Boron-Containing D–a–a Type TADF Materials with Tiny Singlet–Triplet Energy Splittings and High Photoluminescence Quantum Yields for Highly Efficient OLEDs with Low Efficiency Roll-Offs. *J. Mater. Chem. C* **2020**, *8*, 3846-3854.

(139) Kumar, A.; Lee, W.; Lee, T.; Jung, J.; Yoo, S.; Lee, M. H. Triarylboron-Based TADF Emitters with Perfluoro Substituents: High-Efficiency OLEDs with a Power Efficiency over 100 Lm W−1. *J. Mater. Chem. C* **2020**, *8*, 4253-4263.
(140) Park, I. S.; Matsuo, K.; Aizawa, N.; Yasuda, T. High-Performance Dibenzoheteraborin-Based Thermally Activated Delayed Fluorescence Emitters: Molecular Architectonics for Concurrently Achieving Narrowband Emission and Efficient Triplet–Singlet Spin Conversion. *Adv. Funct. Mater.* **2018**, *28*, 1802031.
(141) Wu, T.-L.; Huang, M.-J.; Lin, C.-C.; Huang, P.-Y.; Chou, T.-Y.; Chen-Cheng, R.-W.; Lin, H.-W.; Liu, R.-S.; Cheng, C.-H. Diboron Compound-Based Organic Light-Emitting Diodes with High Efficiency and Reduced Efficiency Roll-Off. *Nat. Photonics* **2018**, *12*, 235-240.
(142) Ouyang, M.; Xing, L.; Chen, Q.; Huang, H.; Zhu, M.; Hu, K.; Liu, Y.; Chen, W.-C.; Huo, Y.; Yang, C. Highly Efficient Thermally Activated Delayed Fluorescence Emitters Enabled by Double Charge Transfer Pathways Via Ortho-Linked Triarylboron/Carbazole Hybrids. *J. Mater. Chem. C* **2021**, *9*, 1678-1684.
(143) Lee, Y. H.; Shin, Y.-S.; Lee, T.; Jung, J.; Lee, J.-H.; Lee, M. H. Managing Local Triplet Excited States of Boron-Based TADF Emitters for Fast Spin-Flip Process: Toward Highly Efficient TADF-OLEDs with Low Efficiency Roll-Off. *Chem. Eng. J.* **2021**, *423*, 130224.
(144) Wu, T. L.; Lei, J.; Hsieh, C. M.; Chen, Y. K.; Huang, P. Y.; Lai, P. T.; Chou, T. Y.; Lin, W. C.; Chen, W.; Yu, C. H.et al. Substituent Engineering of the Diboron Molecular Architecture for a Nondoped and Ultrathin Emitting Layer. *Chem. Sci.* **2022**, *13*, 12996-13005.
(145) Shiu, Y.-J.; Chen, Y.-T.; Lee, W.-K.; Wu, C.-C.; Lin, T.-C.; Liun, S.-H.; Chou, P.-T.; Lu, C.-W.; Cheng, I.-C.; Lien, Y.-J.; Chi, Y. Efficient Thermally Activated Delayed Fluorescence of Functional Phenylpyridinato Boron Complexes and High Performance Organic Light-Emitting Diodes. *J. Mater. Chem. C* **2017**, *5*, 1452-1462.
(146) Gong, S.; Luo, J.; Wang, Z.; Li, Y.; Chen, T.; Xie, G.; Yang, C. Tuning Emissive Characteristics and Singlet-Triplet Energy Splitting of Fluorescent Emitters by Encapsulation Group Modification: Yellow TADF Emitter for Solution-Processed OLEDs with High Luminance and Ultraslow Efficiency Roll-Off. *Dyes Pigm.* **2017**, *139*, 593-600.
(147) Wang, K.; Liu, W.; Zheng, C.-J.; Shi, Y.-Z.; Liang, K.; Zhang, M.; Ou, X.-M.; Zhang, X.-H. A Comparative Study of Carbazole-Based Thermally Activated Delayed Fluorescence Emitters with Different Steric Hindrance. *J. Mater. Chem. C* **2017**, *5*, 4797-4803.
(148) Wang, K.; Zheng, C. J.; Liu, W.; Liang, K.; Shi, Y. Z.; Tao, S. L.; Lee, C. S.; Ou, X. M.; Zhang, X. H. Avoiding Energy Loss on TADF Emitters: Controlling the Dual Conformations of D–a Structure Molecules Based on the Pseudoplanar Segments. *Adv. Mater.* **2017**, *29*, 1701476.
(149) Wei, X.; Li, Z.; Hu, T.; Duan, R.; Liu, J.; Wang, R.; Liu, Y.; Hu, X.; Yi, Y.; Wang, P.; Wang, Y. Substitution Conformation Balances the Oscillator Strength and Singlet–Triplet Energy Gap for Highly Efficient D–a–D Thermally Activated Delayed Fluorescence Emitters. *Adv. Optical Mater.* **2019**, *7*, 1801767.
(150) dos Santos, P. L.; Ward, J. S.; Congrave, D. G.; Batsanov, A. S.; Eng, J.; Stacey, J. E.; Penfold, T. J.; Monkman, A. P.; Bryce, M. R. Triazatruxene: A Rigid Central Donor Unit for a D–A3 Thermally Activated Delayed Fluorescence Material Exhibiting Sub-Microsecond Reverse Intersystem Crossing and Unity Quantum Yield Via Multiple Singlet–Triplet State Pairs. *Adv. Sci.* **2018**, *5*, 1700989.
(151) Zhang, M.; Dai, G.-L.; Zheng, C.-J.; Wang, K.; Shi, Y.-Z.; Fan, X.-C.; Lin, H.; Tao, S.-L.; Zhang, X.-H. Novel D-D′-a Structure Thermally Activated Delayed Fluorescence Emitters Realizing over 20% External Quantum Efficiencies in Both Evaporation- and Solution-Processed Organic Light-Emitting Diodes. *Org. Electron.* **2021**, *99*, 106312.
(152) Ma, M.; Li, J.; Liu, D.; Li, D.; Dong, R.; Mei, Y. Low Efficiency Roll-Off Thermally Activated Delayed Fluorescence Emitters for Non-Doped OLEDs: Substitution Effect of Thioether and Sulfone Groups. *Dyes Pigm.* **2021**, *194*, 109649.
(153) Gao, S.; Chen, X.; Ge, X.; Chen, Z.; Zhao, J.; Chi, Z. Asymmetric Thermally Activated Delayed Fluorescence Materials Rendering High-Performance OLEDs through Both Thermal Evaporation and Solution-Processing. *Chem. Res. Chin. Univ.* **2022**, *38*, 1526-1531.

(154) Gan, L.; Xu, Z.; Wang, Z.; Li, B.; Li, W.; Cai, X.; Liu, K.; Liang, Q.; Su, S.-J. Utilizing a Spiro TADF Moiety as a Functional Electron Donor in TADF Molecular Design toward Efficient "Multichannel" Reverse Intersystem Crossing. *Adv. Funct. Mater.* **2019**, *29*, 1808088.
(155) Li, C.; Duan, C.; Han, C.; Xu, H. Secondary Acceptor Optimization for Full-Exciton Radiation: Toward Sky-Blue Thermally Activated Delayed Fluorescence Diodes with External Quantum Efficiency of ≈30%. *Adv. Mater.* **2018**, *30*, 1804228.
(156) Li, K.; Zhu, Y.; Yao, B.; Chen, Y.; Deng, H.; Zhang, Q.; Zhan, H.; Xie, Z.; Cheng, Y. Rotation-Restricted Thermally Activated Delayed Fluorescence Compounds for Efficient Solution-Processed OLEDs with EQEs of up to 24.3% and Small Roll-Off. *Chem. Commun.* **2020**, *56*, 5957-5960.
(157) Ryoo, C. H.; Cho, I.; Han, J.; Yang, J.-h.; Kwon, J. E.; Kim, S.; Jeong, H.; Lee, C.; Park, S. Y. Structure-Property Correlation in Luminescent Indolo[3,2-B]Indole (Idid) Derivatives: Unravelling the Mechanism of High Efficiency Thermally Activated Delayed fluorescence (TADF). *ACS Appl. Mater. Interfaces* **2017**, *9*, 41413-41420.
(158) Maeng, J. H.; Ahn, D. H.; Lee, H.; Jung, Y. H.; Karthik, D.; Lee, J. Y.; Kwon, J. H. Rigid Indolocarbazole Donor Moiety for Highly Efficient Thermally Activated Delayed Fluorescent Device. *Dyes Pigm.* **2020**, *180*, 108485.
(159) Yoon, S. J.; Lee, H. J.; Lee, K. H.; Lee, J. Y. A Study on the Effect of a Pyridine Secondary Acceptor on the Emission Properties of Thermally Activated Delayed Fluorescence Emitters. *J. Mater. Chem. C* **2020**, *8*, 7485-7491.
(160) Kim, K. J.; Kim, G. H.; Lampande, R.; Ahn, D. H.; Im, J. B.; Moon, J. S.; Lee, J. K.; Lee, J. Y.; Lee, J. Y.; Kwon, J. H. A New Rigid Diindolocarbazole Donor Moiety for High Quantum Efficiency Thermally Activated Delayed Fluorescence Emitter. *J. Mater. Chem. C* **2018**, *6*, 1343-1348.
(161) Yu, J. G.; Han, S. H.; Lee, H. L.; Hong, W. P.; Lee, J. Y. A Novel Molecular Design Employing a Backbone Freezing Linker for Improved Efficiency, Sharpened Emission and Long Lifetime in Thermally Activated Delayed Fluorescence Emitters. *J. Mater. Chem. C* **2019**, *7*, 2919-2926.
(162) Liu, Z.; Li, G.; Liu, H.; Zhou, C.; Li, K.; Wang, Z.; Yang, C. Side by Side Alignment of Donors Enabling High-Efficiency TADF OLEDs with Insensitivity to Doping Concentration. *Adv. Optical Mater.* **2021**, *9*, 2101410.
(163) Lee, H. L.; Chung, W. J.; Lee, J. Y. Efficient up-Conversion Process by Isolation of Two Chromophores in Thermally Activated Delayed Fluorescent Emitters. *Chem. Eng. J.* **2021**, *409*, 128285.
(164) Lee, H. L.; Lee, K. H.; Lee, J. Y.; Lee, H. J. Molecular Design Opening Two Emission Pathways for High Efficiency and Long Lifetime of Thermally Activated Delayed Fluorescent Organic Light-Emitting Diodes. *J. Mater. Chem. C* **2021**, *9*, 7328-7335.
(165) Zhang, S. L.; Shi, Y. Z.; Wang, K.; Fan, X. C.; Yu, J.; Ou, X. M.; Zhang, X. H. Pyridine-Substituted Triazine as an Acceptor for Thermally Activated Delayed Fluorescence Emitters Showing High Efficiency and Low Roll-Off in Organic Light-Emitting Diodes. *Mater. Today Energy* **2021**, *20*, 100581.
(166) Yun, J. H.; Lee, K. H.; Lee, J. Y. Benzoylphenyltriazine as a New Acceptor of Donor–Acceptor Type Thermally-Activated Delayed-Fluorescent Emitters. *J. Ind. Eng. Chem.* **2021**, *102*, 226-232.
(167) Zhan, Q.; Cao, C.; Huang, T.; Zhou, C.; Xie, Z.; Zou, Y.; Lee, C. S.; Yang, C. 3d Triptycene-Fused Acridine Electron Donor Enables High-Efficiency Nondoped Thermally Activated Delayed Fluorescent OLEDs. *Adv. Optical Mater.* **2021**, *9*, 2100273.
(168) Shi, C.; Liu, D.; Li, J.; He, Z.; Song, K.; Liu, B.; Wu, Q.; Xu, M. Tert-Butyltriazine-Diphenylaminocarbazole Based TADF Materials: Π-Bridge Modification for Enhanced KRISC and Efficiency Stability. *Dyes Pigm.* **2022**, *204*, 110430.
(169) Feng, Q.; Qian, Y.; Wang, H.; Hou, W.; Peng, X.; Xie, S.; Wang, S.; Xie, L. Donor Arylmethylation toward Horizontally Oriented TADF Emitters for Efficient Electroluminescence with 37% External Quantum Efficiency. *Adv. Optical Mater.* **2022**, *10*, 2102441.

(170) Fan, X.-C.; Wang, K.; Shi, Y.-Z.; Sun, D.-M.; Chen, J.-X.; Huang, F.; Wang, H.; Yu, J.; Lee, C.-S.; Zhang, X.-H. Thermally Activated Delayed Fluorescence Materials for Nondoped Organic Light-Emitting Diodes with Nearly 100% Exciton Harvest. *SmartMat* **2023**, *4*, e1122.
(171) Xiang, Y.; Li, P.; Gong, S.; Huang, Y.-H.; Wang, C.-Y.; Zhong, C.; Zeng, W.; Chen, Z.; Lee, W.-K.; Yin, X.et al. Acceptor Plane Expansion Enhances Horizontal Orientation of Thermally Activated Delayed Fluorescence Emitters. *Sci. Adv.* **2020**, *6*, eaba7855.
(172) Kato, Y.; Sasabe, H.; Hayasaka, Y.; Watanabe, Y.; Arai, H.; Kido, J. A Sky Blue Thermally Activated Delayed Fluorescence Emitter to Achieve Efficient White Light Emission through in Situ Metal Complex Formation. *J. Mater. Chem. C* **2019**, *7*, 3146-3149.
(173) Xiang, Y.; Zhao, Y.; Xu, N.; Gong, S.; Ni, F.; Wu, K.; Luo, J.; Xie, G.; Lu, Z.; Yang, C. Halogen-Induced Internal Heavy-Atom Effect Shortening Emissive Lifetime and Improving Fluorescence Efficiency of Thermally Activated Delayed Fluorescence Emitters. *J. Mater. Chem. C* **2017**, *5*, 12204-12210.
(174) Serevičius, T.; Skaisgiris, R.; Dodonova, J.; Jagintavicius, L.; Banevicius, D.; Kazlauskas, K.; Tumkevicius, S.; Jursenas, S. Achieving Submicrosecond Thermally Activated Delayed Fluorescence Lifetime and Highly Efficient Electroluminescence by Fine-Tuning of the Phenoxazine-Pyrimidine Structure. *ACS Appl. Mater. Interfaces* **2020**, *12*, 10727-10736.
(175) Zhang, Q.; Wang, Y.; Yoon, S. J.; Chung, W. J.; Ye, S.; Guo, R.; Leng, P.; Sun, S.; Lee, J. Y.; Wang, L. Fusing Acridine and Benzofuran/Benzothiophene as a Novel Hybrid Donor for High-Performance and Low Efficiency Roll-Off TADF OLEDs. *J. Mater. Chem. C* **2020**, *8*, 1864-1870.
(176) Park, H.-J.; Han, S. H.; Lee, J. Y.; Han, H.; Kim, E.-G. Managing Orientation of Nitrogens in Bipyrimidine-Based Thermally Activated Delayed Fluorescent Emitters to Suppress Nonradiative Mechanisms. *Chem. Mater.* **2018**, *30*, 3215-3222.
(177) Yi, C. L.; Lin, C. Y.; Tang, Y.; Wang, C. Y.; Huang, C. W.; Gong, X.; Gong, S.; Wu, C. C.; Wong, K. T. A Rational Molecular Design Strategy of TADF Emitter for Achieving Device Efficiency Exceeding 36%. *Adv. Optical Mater.* **2022**, *10*, 2101791.
(178) Lee, Y.; Hong, J.-I. High-Efficiency Thermally Activated Delayed Fluorescence Emitters Via a High Horizontal Dipole Ratio and Controlled Dual Emission. *J. Mater. Chem. C* **2020**, *8*, 8012-8017.
(179) Lee, Y.; Woo, S.-J.; Kim, J.-J.; Hong, J.-I. Linear-Shaped Thermally Activated Delayed Fluorescence Emitter Using 1,5-Naphthyridine as an Electron Acceptor for Efficient Light Extraction. *Org. Electron.* **2020**, *78*, 105600.
(180) Sasabe, H.; Onuma, N.; Nagai, Y.; Ito, T.; Kido, J. High Power Efficiency Blue-to-Green Organic Light-Emitting Diodes Using Isonicotinonitrile-Based Fluorescent Emitters. *Chem. Asian J.* **2017**, *12*, 648-654.
(181) Sasabe, H.; Hayasaka, Y.; Komatsu, R.; Nakao, K.; Kido, J. Highly Luminescent Π-Conjugated Terpyridine Derivatives Exhibiting Thermally Activated Delayed Fluorescence. *Chem. Eur. J.* **2017**, *23*, 114-119.
(182) Wang, L.; Huang, Z.; Xiang, S.; Zhang, Q.; Lv, X.; Ye, S.; Zhuang, S.; Guo, R. Highly Efficient Green Organic Light Emitting Diodes with Phenanthroimidazole-Based Thermally Activated Delayed Fluorescence Emitters. *J. Mater. Chem. C* **2018**, *6*, 2379-2386.
(183) Kothavale, S.; Chung, W. J.; Lee, J. Y. Rational Molecular Design of Highly Efficient Yellow-Red Thermally Activated Delayed Fluorescent Emitters: A Combined Effect of Auxiliary Fluorine and Rigidified Acceptor Unit. *ACS Appl. Mater. Interfaces* **2020**, *12*, 18730-18738.
(184) Zeng, X.; Zhou, T.; Liu, J.; Wu, K.; Li, S.; Xiao, X.; Zhang, Y.; Gong, S.; Xie, G.; Yang, C. Incorporating Thermally Activated Delayed Fluorescence into Mechanochromic Luminescent Emitters: High-Performance Solution-Processed Yellow Organic Light Emitting Diodes. *Adv. Optical Mater.* **2018**, *6*, 1801071.
(185) Kim, H.; Lee, Y.; Lee, H.; Hong, J. I.; Lee, D. Click-to-Twist Strategy to Build Blue-to-Green Emitters: Bulky Triazoles for Electronically Tunable and Thermally Activated Delayed Fluorescence. *ACS Appl. Mater. Interfaces* **2021**, *13*, 12286-12295.
(186) Zhang, X.; Cooper, M. W.; Zhang, Y.; Fuentes-Hernandez, C.; Barlow, S.; Marder, S. R.; Kippelen, B. Host-Free Yellow-Green Organic Light-Emitting Diodes with External Quantum

Efficiency over 20% Based on a Compound Exhibiting Thermally Activated Delayed Fluorescence. *ACS Appl. Mater. Interfaces* **2019**, *11*, 12693-12698.
(187) Chen, Y.; Zhang, D.; Zhang, Y.; Zeng, X.; Huang, T.; Liu, Z.; Li, G.; Duan, L. Approaching Nearly 40% External Quantum Efficiency in Organic Light Emitting Diodes Utilizing a Green Thermally Activated Delayed Fluorescence Emitter with an Extended Linear Donor-Acceptor-Donor Structure. *Adv. Mater.* **2021**, *33*, 2103293.
(188) Hu, D.; Zhu, M.; Shi, C.; Yuan, W.; Sun, N.; Huang, B.; Tao, Y. Manipulating Peripheral Non-Conjugated Substituents in Carbazole/Oxadiazole Hybrid TADF Emitters Towards High-Efficiency OLEDs. *J. Mater. Chem. C* **2021**, *9*, 13384-13391.
(189) Zhou, J. X.; Zeng, X. Y.; Xie, F. M.; He, Y. H.; Tang, Y. Q.; Li, Y. Q.; Tang, J. X. High-Efficiency Orange Thermally Activated Delayed Fluorescence by Secondary Acceptor Modification. *Mater. Today Energy* **2021**, *21*, 100819.
(190) Liu, Y.; Yang, J.; Mao, Z.; Ma, D.; Wang, Y.; Zhao, J.; Su, S. J.; Chi, Z. Donor or Acceptor: Molecular Engineering Based on Dibenzo[a,C]Phenazine Backbone for Highly Efficient Thermally-Activated Delayed Fluorescence Organic Light-Emitting Diodes. *Adv. Optical Mater.* **2023**, *11*, 2201695.
(191) Li, B.; Wang, Z.; Su, S.-J.; Guo, F.; Cao, Y.; Zhang, Y. Quinazoline-Based Thermally Activated Delayed Fluorecence for High-Performance OLEDs with External Quantum Efficiencies Exceeding 20%. *Adv. Optical Mater.* **2019**, *7*, 1801496.
(192) Ji, S.-C.; Zhao, T.; Wei, Z.; Meng, L.; Tao, X.-D.; Yang, M.; Chen, X.-L.; Lu, C.-Z. Manipulating Excited States Via Lock/Unlock Strategy for Realizing Efficient Thermally Activated Delayed Fluorescence Emitters. *Chem. Eng. J.* **2022**, *435*, 134868.
(193) Chen, Z.; Wu, Z.; Ni, F.; Zhong, C.; Zeng, W.; Wei, D.; An, K.; Ma, D.; Yang, C. Emitters with a Pyridine-3,5-Dicarbonitrile Core and Short Delayed Fluorescence Lifetimes of About 1.5 Ms: Orange-Red TADF-Based OLEDs with Very Slow Efficiency Roll-Offs at High Luminance. *J. Mater. Chem. C* **2018**, *6*, 6543-6548.
(194) Shi, Y. Z.; Wang, K.; Fan, X. C.; Chen, J. X.; Ou, X. M.; Yu, J.; Jie, J. S.; Lee, C. S.; Zhang, X. H. High-Performance Nondoped Organic Light-Emitting Diode Based on a Thermally Activated Delayed Fluorescence Emitter with 1d Intermolecular Hydrogen Bonding Interactions. *Adv. Optical Mater.* **2021**, *9*, 2100461.
(195) Dong, R.; Liu, D.; Li, J.; Ma, M.; Mei, Y.; Li, D.; Jiang, J. Acceptor Modulation for Blue and Yellow TADF Materials and Fabrication of All-TADF White OLED. *Mater. Chem. Front.* **2022**, *6*, 40-51.
(196) Liu, H.; Li, J.; Chen, W.-C.; Chen, Z.; Liu, Z.; Zhan, Q.; Cao, X.; Lee, C.-S.; Yang, C. Modulating the Acceptor Structure of Dicyanopyridine Based TADF Emitters: Nearly 30% External Quantum Efficiency and Suppression on Efficiency Roll-Off in OLED. *Chem. Eng. J.* **2020**, *401*, 126107.
(197) Li, J.; Chen, W.-C.; Liu, H.; Chen, Z.; Chai, D.; Lee, C.-S.; Yang, C. Double-Twist Pyridine–Carbonitrile Derivatives Yielding Excellent Thermally Activated Delayed Fluorescence Emitters for High-Performance OLEDs. *J. Mater. Chem. C* **2020**, *8*, 602-606.
(198) Chen, Y. K.; Jayakumar, J.; Hsieh, C. M.; Wu, T. L.; Liao, C. C.; Pandidurai, J.; Ko, C. L.; Hung, W. Y.; Cheng, C. H. Triarylamine-Pyridine-Carbonitriles for Organic Light-Emitting Devices with EQE Nearly 40. *Adv. Mater.* **2021**, *33*, 2008032.
(199) Xie, Z.; Cao, C.; Zou, Y.; Cao, X.; Zhou, C.; He, J.; Lee, C. S.; Yang, C. Molecular Engineering Enables TADF Emitters Well Suitable for Non-Doped OLEDs with External Quantum Efficiency of Nearly 30%. *Adv. Funct. Mater.* **2022**, *32*, 2112881.
(200) Lee, H. L.; Oh, C. S.; Lee, K. H.; Lee, J. Y.; Hong, W. P. Lifetime-Extending 3-(4-Phenylbenzo[4,5]Thieno[3,2-D]Pyrimidin-2-Yl)Benzonitrile Acceptor for Thermally Activated Delayed Fluorescence Emitters. *ACS Appl. Mater. Interfaces* **2021**, *13*, 2908-2918.
(201) Lee, H. L.; Chung, W. J.; Lee, J. Y. Selective Efficiency Boosting in Thermally Activated Delayed Fluorescence Emitters by a Secondary Donor. *Chem. Eng. J.* **2021**, *408*, 127293.
(202) Chen, C.-F.; Li, M.; Liu, Y.; Duan, R.; Wei, X.; Yi, Y.; Wang, Y. Aromatic Imide Based Thermally Activated Delayed Fluorescence Materials for Highly Efficient Organic Light Emitting Diodes. *Angew. Chem., Int. Ed.* **2017**, *56*, 8818-8822.

(203) Xiang, Y.; Zhu, Z.-L.; Xie, D.; Gong, S.; Wu, K.; Xie, G.; Lee, C.-S.; Yang, C. Revealing the New Potential of an Indandione Unit for Constructing Efficient Yellow Thermally Activated Delayed Fluorescence Emitters with Short Emissive Lifetimes. *J. Mater. Chem. C* **2018**, *6*, 7111-7118.

(204) Liu, Y.; Yin, Z.; Wang, X.; Baranoff, E.; Zhou, D.; Zhang, K.; Ren, Z.; Wang, S.; Zhu, W.; Wang, Y. A Novel Donor Moiety 9,9,9′9′-Tetramethyl-9,9′10,10′-Tetrahydro-2,10′-Biacridine Via One-Pot C–H Arylation for TADF Emitters and Their Application in Highly Efficient Solution-Processable OLEDs. *J. Mater. Chem. C* **2020**, *8*, 8971-8979.

(205) Zeng, J.; Guo, J.; Liu, H.; Zhao, Z.; Tang, B. Z. A Multifunctional Bipolar Luminogen with Delayed Fluorescence for High-Performance Monochromatic and Color-Stable Warm-White OLEDs. *Adv. Funct. Mater.* **2020**, *30*, 2000019.

(206) Wang, P.; Yu, J.; Chen, S.; Yu, H.; Yan, X.; Guan, Y.; Chen, J.; Li, L. 3-Benzoyl-4h-Chromen-4-One: A Novel Twisted Acceptor for Highly Efficient Thermally Activated Delayed Fluorescence Emitters. *Dyes Pigm.* **2020**, *183*, 108744.

(207) Chen, X.; Yang, Z.; Xie, Z.; Zhao, J.; Yang, Z.; Zhang, Y.; Aldred, M. P.; Chi, Z. An Efficient Yellow Thermally Activated Delayed Fluorescence Emitter with Universal Applications in Both Doped and Non-Doped Organic Light-Emitting Diodes. *Mater. Chem. Front.* **2018**, *2*, 1017-1023.

(208) Bai, M.-D.; Zhang, M.; Wang, K.; Shi, Y.-Z.; Chen, J.-X.; Lin, H.; Tao, S.-L.; Zheng, C.-J.; Zhang, X.-H. Novel Star-Shaped Yellow Thermally Activated Delayed Fluorescence Emitter Realizing over 10% External Quantum Efficiency at High Luminance of 30000 Cd M^{-2} in OLED. *Org. Electron.* **2018**, *62*, 220-226.

(209) Dong, R.; Li, J.; Liu, D.; Li, D.; Mei, Y.; Ma, M.; Jiang, J. Self-Host Thermally Activated Delayed Fluorescence Material with Aggregation-Induced Emission Character: Multi-Functional Applications in OLEDs. *Adv. Optical Mater.* **2021**, *9*, 2100970.

(210) Jing, Y.-Y.; Tao, X.-D.; Yang, M.-X.; Chen, X.-L.; Lu, C.-Z. Triptycene-Imbedded Thermally Activated Delayed Fluorescence Emitters with Excellent Film Morphologies for Applications in Efficient Nondoped and Doped Organic Light-Emitting Devices. *Chem. Eng. J.* **2021**, *413*, 127418.

(211) Chen, J.-X.; Liu, W.; Zheng, C.-J.; Wang, K.; Liang, K.; Shi, Y.-Z.; Ou, X.-M.; Zhang, X.-H. Coumarin-Based Thermally Activated Delayed Fluorescence Emitters with High External Quantum Efficiency and Low Efficiency Roll-Off in the Devices. *ACS Appl. Mater. Interfaces* **2017**, *9*, 8848-8854.

(212) Luo, X. F.; Li, F. L.; Zou, J. W.; Zou, Q.; Su, J.; Mao, M. X.; Zheng, Y. X. A Series of Fused Carbazole/Carbonyl Based Blue to Yellow-Green Thermally Activated Delayed Fluorescence Materials for Efficient Organic Light-Emitting Diodes. *Adv. Optical Mater.* **2021**, *9*, 2100784.

(213) Mei, Y.; Liu, D.; Li, J.; Li, H.; Wei, W. Acridin-9(10h)-One Based Thermally Activated Delayed Fluorescence Material: Simultaneous Optimization of RISC and Radiation Processes to Boost Luminescence Efficiency. *J. Mater. Chem. C* **2021**, *9*, 5885-5892.

(214) Xu, J.; Wu, X.; Guo, J.; Zhao, Z.; Tang, B. Z. Sky-Blue Delayed Fluorescence Molecules Based on Pyridine-Substituted Acridone for Efficient Organic Light-Emitting Diodes. *J. Mater. Chem. C* **2021**, *9*, 15505-15510.

(215) Mei, Y.; Liu, D.; Li, J.; Wang, J. Thermally Activated Delayed Fluorescence Materials Based on Acridin-9(10h)-One Acceptor for Organic Light-Emitting Diodes. *Dyes Pigm.* **2022**, *207*, 110701.

(216) Mei, Y.; Liu, D.; J.Li; Dong, R.; M.Ma; Wei, W.; Y.Lan. Acridin-9(10h)-One-Based Blue Thermally Activated Delayed Fluorescence Materials: Improvement of Color Purity and Efficiency Stability. *Mater. Today Chem.* **2022**, *23*, 100645.

(217) Aizawa, N.; Matsumoto, A.; Yasuda, T. Thermal Equilibration between Singlet and Triplet Excited States in Organic Fluorophore for Submicrosecond Delayed Fluorescence. *Sci. Adv.* **2021**, *7*, eabe5769.

(218) Wang, R.; Li, Z.; Hu, T.; Tian, L.; Hu, X.; Liu, S.; Cao, C.; Zhu, Z. L.; Tan, J. H.; Yi, Y.et al. Two-Channel Space Charge Transfer-Induced Thermally Activated Delayed Fluorescent Materials for Efficient OLEDs with Low Efficiency Roll-Off. *ACS Appl. Mater. Interfaces* **2021**, *13*, 49066-49075.

(219) Yang, D.; Huh, J.-S.; Hong, J.-I. Spiro−Type TADF Emitters Based on Acridine Donors and Anthracenone Acceptor. *Dyes Pigm.* **2022**, *197*, 109873.
(220) Huang, Z.; Lei, B.; Yang, D.; Ma, D.; Bin, Z.; You, J. Modified Intramolecular-Lock Strategy Enables Efficient Thermally Activated Delayed Fluorescence Emitters for Non-Doped OLEDs. *Angew. Chem., Int. Ed.* **2022**, *61*, e202213157.
(221) Yuan, W.; Yang, H.; Duan, C.; Cao, X.; Zhang, J.; Xu, H.; Sun, N.; Tao, Y.; Huang, W. Molecular Configuration Fixation with C–H···F Hydrogen Bonding for Thermally Activated Delayed Fluorescence Acceleration. *CHEM* **2020**, *6*, 1998-2008.
(222) Chen, J.-X.; Wang, H.; Zhang, X.; Xiao, Y.-F.; Wang, K.; Zhou, L.; Shi, Y.-Z.; Yu, J.; Lee, C.-S.; Zhang, X.-H. Using Fullerene Fragments as Acceptors to Construct Thermally Activated Delayed Fluorescence Emitters for High-Efficiency Organic Light-Emitting Diodes. *Chem. Eng. J.* **2022**, *435*, 134731.
(223) Liu, H.; Li, J.; Chen, W.-C.; Lv, X.; Zhou, C.; Lee, C.-S.; Yang, C. Efficient Yellow Thermally Activated Delayed Fluorescent Emitters Based on 3,5-Dicyanopyridine Acceptors. *J. Phys. Chem. C* **2020**, *124*, 25489-25498.
(224) Li, C.; Duan, R.; Liang, B.; Han, G.; Wang, S.; Ye, K.; Liu, Y.; Yi, Y.; Wang, Y. Deep-Red to near-Infrared Thermally Activated Delayed Fluorescence in Organic Solid Films and Electroluminescent Devices. *Angew. Chem., Int. Ed.* **2017**, *56*, 11525-11529.
(225) Kothavale, S.; Lee, K. H.; Lee, J. Y. Isomeric Quinoxalinedicarbonitrile as Color-Managing Acceptors of Thermally Activated Delayed Fluorescent Emitters. *ACS Appl. Mater. Interfaces* **2019**, *11*, 17583-17591.
(226) Kothavale, S.; Chung, W. J.; Lee, J. Y. High Efficiency and Long Lifetime Orange-Red Thermally Activated Delayed Fluorescent Organic Light Emitting Diodes by Donor and Acceptor Engineering. *J. Mater. Chem. C* **2021**, *9*, 528-536.
(227) Huang, T.; Liu, D.; Li, D.; Jiang, W.; Jiang, J. Novel Yellow Thermally Activated Delayed Fluorescence Emitters for Highly Efficient Full-TADF WOLEDs with Low Driving Voltages and Remarkable Color Stability. *New J. Chem.* **2019**, *43*, 13339-13348.
(228) Liang, J.; Li, C.; Cui, Y.; Li, Z.; Wang, J.; Wang, Y. Rational Design of Efficient Orange-Red to Red Thermally Activated Delayed Fluorescence Emitters for OLEDs with External Quantum Efficiency of up to 26.0% and Reduced Efficiency Roll-Off. *J. Mater. Chem. C* **2020**, *8*, 1614-1622.
(229) Yuan, Y.; Hu, Y.; Zhang, Y.-X.; Lin, J.-D.; Wang, Y.-K.; Jiang, Z.-Q.; Liao, L.-S.; Lee, S.-T. Over 10% EQE near-Infrared Electroluminescence Based on a Thermally Activated Delayed Fluorescence Emitter. *Adv. Funct. Mater.* **2017**, *27*, 1700986.
(230) Xue, J.; Liang, Q.; Wang, R.; Hou, J.; Li, W.; Peng, Q.; Shuai, Z.; Qiao, J. Highly Efficient Thermally Activated Delayed Fluorescence Via J-Aggregates with Strong Intermolecular Charge Transfer. *Adv. Mater.* **2019**, *31*, 1808242.
(231) Congrave, D. G.; Drummond, B. H.; Conaghan, P. J.; Francis, H.; Jones, S. T. E.; Grey, C. P.; Greenham, N. C.; Credgington, D.; Bronstein, H. A Simple Molecular Design Strategy for Delayed Fluorescence toward 1000 Nm. *J. Am. Chem. Soc.* **2019**, *141*, 18390-18394.
(232) Gong, X.; Li, P.; Huang, Y. H.; Wang, C. Y.; Lu, C. H.; Lee, W. K.; Zhong, C.; Chen, Z.; Ning, W.; Wu, C. C.et al. A Red Thermally Activated Delayed Fluorescence Emitter Simultaneously Having High Photoluminescence Quantum Efficiency and Preferentially Horizontal Emitting Dipole Orientation. *Adv. Funct. Mater.* **2020**, *30*, 1908839.
(233) Cheng, J.-F.; Pan, Z.-H.; Zhang, K.; Zhao, Y.; Wang, C.-K.; Ding, L.; Fung, M.-K.; Fan, J. Interrupted Intramolecular Donor-Acceptor Interaction Compensated by Strong through-Space Electronic Coupling for Highly Efficient near-Infrared TADF with Emission over 800 nm. *Chem. Eng. J.* **2022**, *430*, 132744.
(234) Gong, X.; Lu, C.-H.; Lee, W.-K.; Li, P.; Huang, Y.-H.; Chen, Z.; Zhan, L.; Wu, C.-C.; Gong, S.; Yang, C. High-Efficiency Red Thermally Activated Delayed Fluorescence Emitters Based on Benzothiophene-Fused Spiro-Acridine Donor. *Chem. Eng. J.* **2021**, *405*, 126663.
(235) Wang, S.; Cheng, Z.; Song, X.; Yan, X.; Ye, K.; Liu, Y.; Yang, G.; Wang, Y. Highly Efficient Long-Wavelength Thermally Activated Delayed Fluorescence OLEDs Based on Dicyanopyrazino Phenanthrene Derivatives. *ACS Appl. Mater. Interfaces* **2017**, *9*, 9892-9901.

(236) Wang, B.; Qiao, X.; Yang, Z.; Wang, Y.; Liu, S.; Ma, D.; Wang, Q. Realizing Efficient Red Thermally Activated Delayed Fluorescence Organic Light-Emitting Diodes Using Phenoxazine/Phenothiazine-Phenanthrene Hybrids. *Org. Electron.* **2018**, *59*, 32-38.
(237) Wang, B.; Yang, H.; Zhang, Y.; Xie, G.; Ran, H.; Wang, T.; Fu, Q.; Ren, Y.; Sun, N.; Zhao, G.et al. Highly Efficient Electroluminescence from Evaporation- and Solution-Processable Orange–Red Thermally Activated Delayed Fluorescence Emitters. *J. Mater. Chem. C* **2019**, *7*, 12321-12327.
(238) Furue, R.; Matsuo, K.; Ashikari, Y.; Ooka, H.; Amanokura, N.; Yasuda, T. Highly Efficient Red–Orange Delayed Fluorescence Emitters Based on Strong Π-Accepting Dibenzophenazine and Dibenzoquinoxaline Cores: Toward a Rational Pure-Red OLED Design. *Adv. Optical Mater.* **2018**, *6*, 1701147.
(239) Zhang, Y.-L.; Ran, Q.; Wang, Q.; Liu, Y.; Hänisch, C.; Reineke, S.; Fan, J.; Liao, L.-S. High-Efficiency Red Organic Light-Emitting Diodes with External Quantum Efficiency Close to 30% Based on a Novel Thermally Activated Delayed Fluorescence Emitter. *Adv. Mater.* **2019**, *31*, 1902368.
(240) Xie, F. M.; Li, H. Z.; Dai, G. L.; Li, Y. Q.; Cheng, T.; Xie, M.; Tang, J. X.; Zhao, X. Rational Molecular Design of Dibenzo[a,C]Phenazine-Based Thermally Activated Delayed Fluorescence Emitters for Orange-Red OLEDs with EQE up to 22.0. *ACS Appl. Mater. Interfaces* **2019**, *11*, 26144-26151.
(241) Xie, F.-M.; Wu, P.; Zou, S.-J.; Li, Y.-Q.; Cheng, T.; Xie, M.; Tang, J.-X.; Zhao, X. Efficient Orange–Red Delayed Fluorescence Organic Light-Emitting Diodes with External Quantum Efficiency over 26%. *Adv. Electron. Mater.* **2020**, *6*, 1900843.
(242) Chen, J. X.; Tao, W. W.; Xiao, Y. F.; Wang, K.; Zhang, M.; Fan, X. C.; Chen, W. C.; Yu, J.; Li, S.; Geng, F. X.et al. Efficient Orange-Red Thermally Activated Delayed Fluorescence Emitters Feasible for Both Thermal Evaporation and Solution Process. *ACS Appl. Mater. Interfaces* **2019**, *11*, 29086-29093.
(243) Chen, J. X.; Xiao, Y. F.; Wang, K.; Sun, D.; Fan, X. C.; Zhang, X.; Zhang, M.; Shi, Y. Z.; Yu, J.; Geng, F. X.et al. Managing Locally Excited and Charge-Transfer Triplet States to Facilitate up-Conversion in Red TADF Emitters That Are Available for Both Vacuum- and Solution-Processes. *Angew. Chem., Int. Ed.* **2021**, *60*, 2478-2484.
(244) Fan, J.; Zhang, Y.; Ma, Y.; Song, Y.; Lin, L.; Xu, Y.; Wang, C.-K. The Role of Intermolecular Interactions in Regulating the Thermally Activated Delayed Fluorescence and Charge Transfer Properties: A Theoretical Perspective. *J. Mater. Chem. C* **2020**, *8*, 8601-8612.
(245) Chen, J.-X.; Wang, K.; Zheng, C.-J.; Zhang, M.; Shi, Y.-Z.; Tao, S.-L.; Lin, H.; Liu, W.; Tao, W.-W.; Ou, X.-M.; Zhang, X.-H. Red Organic Light-Emitting Diode with External Quantum Efficiency Beyond 20% Based on a Novel Thermally Activated Delayed Fluorescence Emitter. *Adv. Sci.* **2018**, *5*, 1800436.
(246) Chen, J.-X.; Tao, W.-W.; Xiao, Y.-F.; Tian, S.; Chen, W.-C.; Wang, K.; Yu, J.; Geng, F.-X.; Zhang, X.-H.; Lee, C.-S. Isomeric Thermally Activated Delayed Fluorescence Emitters Based on Indolo[2,3-B]Acridine Electron-Donor: A Compromising Optimization for Efficient Orange–Red Organic Light-Emitting Diodes. *J. Mater. Chem. C* **2019**, *7*, 2898-2904.
(247) Zhou, C.; Chen, W.-C.; Liu, H.; Cao, X.; Li, N.; Zhang, Y.; Lee, C.-S.; Yang, C. Isomerization Enhanced Quantum Yield of Dibenzo[a,C]Phenazine-Based Thermally Activated Delayed Fluorescence Emitters for Highly Efficient Orange OLEDs. *J. Mater. Chem. C* **2020**, *8*, 9639-9645.
(248) Liu, Y.; Chen, Y.; Li, H.; Wang, S.; Wu, X.; Tong, H.; Wang, L. High-Performance Solution-Processed Red Thermally Activated Delayed Fluorescence OLEDs Employing Aggregation-Induced Emission-Active Triazatruxene-Based Emitters. *ACS Appl. Mater. Interfaces* **2020**, *12*, 30652-30658.
(249) Balijapalli, U.; Nagata, R.; Yamada, N.; Nakanotani, H.; Tanaka, M.; D'Aleo, A.; Placide, V.; Mamada, M.; Tsuchiya, Y.; Adachi, C. Highly Efficient near-Infrared Electrofluorescence from a Thermally Activated Delayed Fluorescence Molecule. *Angew. Chem., Int. Ed.* **2021**, *60*, 8477-8482.

(250) Kothavale, S.; Chung, W. J.; Lee, J. Y. Color Tuning of Dibenzo[a,C]Phenazine-2,7-Dicarbonitrile-Derived Thermally Activated Delayed Fluorescence Emitters from Yellow to Deep-Red. *J. Mater. Chem. C* **2020**, *8*, 7059-7066.

(251) Xie, F.-M.; Zeng, X.-Y.; Zhou, J.-X.; An, Z.-D.; Wang, W.; Li, Y.-Q.; Zhang, X.-H.; Tang, J.-X. Intramolecular H-Bond Design for Efficient Orange–Red Thermally Activated Delayed Fluorescence Based on a Rigid Dibenzo[F,H]Pyrido[2,3-B]Quinoxaline Acceptor. *J. Mater. Chem. C* **2020**, *8*, 15728-15734.

(252) Xu, H.; Zhao, B.; Wang, H.; Han, C.; Ma, P.; Li, Z.; Chang, P. Highly Efficient Deep-Red Non-Doped Diodes Based on T-Shape Thermally Activated Delayed Fluorescence Emitter. *Angew. Chem., Int. Ed.* **2020**, *59*, 19042-19047.

(253) Wu, K.; Zhang, T.; Zhan, L.; Wang, Z.; Zhong, C.; Gong, S.; Lu, Z.-H.; Yang, C. Highly Efficient Orange–Red Electroluminescence Enabled by Fluorenone-Based Thermally Activated Delayed Fluorescent Emitter. *J. Photon. Energy* **2018**, *8*, 032107.

(254) Zhang, K.; Zhang, X.; Fan, J.; Song, Y.; Fan, J.; Wang, C. K.; Lin, L. Novel Deep Red Thermally Activated Delayed Fluorescence Molecule with Aggregation-Induced Emission Enhancement: Theoretical Design and Experimental Validation. *J. Phys. Chem. Lett* **2022**, *13*, 4711-4720.

(255) Tan, J. H.; Jin, J. M.; Chen, W. C.; Cao, C.; Wang, R.; Zhu, Z. L.; Huo, Y.; Lee, C. S. The Role of Balancing Carrier Transport in Realizing an Efficient Orange-Red Thermally Activated Delayed-Fluorescence Organic Light-Emitting Diode. *ACS Appl. Mater. Interfaces* **2022**, *14*, 53120-53128.

(256) Kothavale, S.; Chung, W. J.; Lee, J. Y. Isomer Engineering of Dipyrido[3,2-A:3′,4′-C]Phenazine-Acceptor-Based Red Thermally Activated Delayed Fluorescent Emitters. *J. Mater. Chem. C* **2022**, *10*, 6043-6049.

(257) Huang, F.-X.; Li, H.-Z.; Xie, F.-M.; Zeng, X.-Y.; Li, Y.-Q.; Hu, Y.-Y.; Tang, J.-X.; Zhao, X. Efficient Orange-Red Thermally Activated Delayed Fluorescence Material Containing a Cyano Group. *Dyes Pigm.* **2021**, *195*, 109731.

(258) Yang, T.; Cheng, Z.; Li, Z.; Liang, J.; Xu, Y.; Li, C.; Wang, Y. Improving the Efficiency of Red Thermally Activated Delayed Fluorescence Organic Light-Emitting Diode by Rational Isomer Engineering. *Adv. Funct. Mater.* **2020**, *30*, 2002681.

(259) Zeng, W.; Lai, H. Y.; Lee, W. K.; Jiao, M.; Shiu, Y. J.; Zhong, C.; Gong, S.; Zhou, T.; Xie, G.; Sarma, M.et al. Achieving Nearly 30% External Quantum Efficiency for Orange–Red Organic Light Emitting Diodes by Employing Thermally Activated Delayed Fluorescence Emitters Composed of 1,8-Naphthalimide-Acridine Hybrids. *Adv. Mater.* **2018**, *30*, 1704961.

(260) Chen, T.; Lu, C.-H.; Huang, C.-W.; Zeng, X.; Gao, J.; Chen, Z.; Xiang, Y.; Zeng, W.; Huang, Z.; Gong, S.et al. Tuning the Emissive Characteristics of TADF Emitters by Fusing Heterocycles with Acridine as Donors: Highly Efficient Orange to Red Organic Light-Emitting Diodes with EQE over 20%. *J. Mater. Chem. C* **2019**, *7*, 9087-9094.

(261) Zeng, W.; Zhou, T.; Ning, W.; Zhong, C.; He, J.; Gong, S.; Xie, G.; Yang, C. Realizing 22.5% External Quantum Efficiency for Solution-Processed Thermally Activated Delayed-Fluorescence OLEDs with Red Emission at 622 Nm Via a Synergistic Strategy of Molecular Engineering and Host Selection. *Adv. Mater.* **2019**, *31*, 1901404.

(262) Wang, B.; Zheng, Y.; Wang, T.; Ma, D.; Wang, Q. 1,8-Naphthalimide-Based Hybrids for Efficient Red Thermally Activated Delayed Fluorescence Organic Light-Emitting Diodes. *Org. Electron.* **2021**, *88*, 106012.

(263) Zeng, X.; Huang, Y.-H.; Gong, S.; Yin, X.; Lee, W.-K.; Xiao, X.; Zhang, Y.; Zeng, W.; Lu, C.-H.; Lee, C.-C.et al. Rational Design of Perfectly Oriented Thermally Activated Delayed Fluorescence Emitter for Efficient Red Electroluminescence. *Sci. China Mater.* **2020**, *64*, 920-930.

(264) Chen, T.; Lu, C. H.; Chen, Z.; Gong, X.; Wu, C. C.; Yang, C. Modulating the Electron-Donating Ability of Acridine Donor Units for Orange-Red Thermally Activated Delayed Fluorescence Emitters. *Chem. Eur. J.* **2021**, *27*, 3151-3158.

(265) Kim, D.-H.; D'Aléo, A.; Chen, X.-K.; Sandanayaka, A. D. S.; Yao, D.; Zhao, L.; Komino, T.; Zaborova, E.; Canard, G.; Tsuchiya, Y.et al. High-Efficiency Electroluminescence and

Amplified Spontaneous Emission from a Thermally Activated Delayed Fluorescent near-Infrared Emitter. *Nat. Photonics* **2018**, *12*, 98-104.
(266) Ye, H.; Kim, D. H.; Chen, X.; Sandanayaka, A. S. D.; Kim, J. U.; Zaborova, E.; Canard, G.; Tsuchiya, Y.; Choi, E. Y.; Wu, J. W.et al. Near Infrared Electroluminescence and Low Threshold Amplified Spontaneous Emission above 800 Nm from a Thermally-Activated Delayed Fluorescent Emitter. *Chem. Mater.* **2018**, *30*, 6702-6710.
(267) Jin, J.; Wang, W.; Xue, P.; Yang, Q.; Jiang, H.; Tao, Y.; Zheng, C.; Xie, G.; Huang, W.; Chen, R. Intermolecular Locking Design of Red Thermally Activated Delayed Fluorescence Molecules for High-Performance Solution-Processed Organic Light-Emitting Diodes. *J. Mater. Chem. C* **2021**, *9*, 2291-2297.
(268) Kumar, A.; Shin, H. Y.; Lee, T.; Jung, J.; Jung, B. J.; Lee, M. H. Doubly Boron-Doped TADF Emitters Decorated with Ortho-Donor Groups for Highly Efficient Green to Red OLEDs. *Chem. Eur. J.* **2020**, *26*, 16793-16801.
(269) Hsieh, C. M.; Wu, T. L.; Jayakumar, J.; Wang, Y. C.; Ko, C. L.; Hung, W. Y.; Lin, T. C.; Wu, H. H.; Lin, K. H.; Lin, C. H.et al. Diboron-Based Delayed Fluorescent Emitters with Orange-to-Red Emission and Superior Organic Light-Emitting Diode Efficiency. *ACS Appl. Mater. Interfaces* **2020**, *12*, 23199-23206.
(270) Karthik, D.; Jung, Y. H.; Lee, H.; Hwang, S.; Seo, B. M.; Kim, J. Y.; Han, C. W.; Kwon, J. H. Acceptor-Donor-Acceptor-Type Orange-Red Thermally Activated Delayed Fluorescence Materials Realizing External Quantum Efficiency over 30% with Low Efficiency Roll-Off. *Adv. Mater.* **2021**, *33*, 2007724.
(271) Kumsampao, J.; Chaiwai, C.; Chasing, P.; Chawanpunyawat, T.; Namuangruk, S.; Sudyoadsuk, T.; Promarak, V. A Simple and Strong Electron-Deficient 5,6-Dicyano[2,1,3]Benzothiadiazole-Cored Donor-Acceptor-Donor Compound for Efficient near Infrared Thermally Activated Delayed Fluorescence. *Chem. Asian J.* **2020**, *15*, 3029-3036.
(272) Wang, Y.-Y.; Tong, K.-N.; Zhang, K.; Lu, C.-H.; Chen, X.; Liang, J.-X.; Wang, C.-K.; Wu, C.-C.; Fung, M.-K.; Fan, J. Positive Impact of Chromophore Flexibility on the Efficiency of Red Thermally Activated Delayed Fluorescence Materials. *Mater. Horiz.* **2021**, *8*, 1297-1303.
(273) Hu, X.; Qin, Y.; Li, Z.; Gao, H.; Gao, T.; Liu, G.; Dong, X.; Tian, N.; Gu, X.; Lee, C.-S.et al. Nearly 100% Exciton Utilization in Highly Efficient Red OLEDs Based on Dibenzothioxanthone Acceptor. *Chin. Chem. Lett.* **2022**, *33*, 4645-4648.
(274) Gao, T.; Shen, S.; Qin, Y.; Gao, H.; Dong, X.; Pang, Z.; Wang, P.; Wang, Y.; Hu, X. Modulating up-Conversion and Non-Radiative Deactivation to Achieve Efficient Red Thermally Activated Delayed Fluorescence Emitters. *J. Mater. Chem. C* **2022**, *10*, 17053-17058.
(275) Zhang, Q.; Kuwabara, H.; Potscavage, W. J.; Huang, S.; Hatae, Y.; Shibata, T.; Adachi, C. Anthraquinone-Based Intramolecular Charge-Transfer Compounds: Computational Molecular Design, Thermally Activated Delayed Fluorescence, and Highly Efficient Red Electroluminescence. *J. Am. Chem. Soc.* **2014**, *136*, 18070-18081.
(276) Hao, F.-Y.; Shi, Y.-Z.; Wang, K.; Fan, X.-C.; Wu, L.; Ye, J.; Zheng, C.-J.; Li, Y.-Q.; Ou, X.-M.; Zhang, X.-H. Forcing Dimethylacridine Crooking to Improve the Efficiency of Orange-Red Thermally Activated Delayed Fluorescent Emitters. *J. Mater. Chem. C* **2020**, *8*, 10416-10421.
(277) Pandidurai, J.; Jayakumar, J.; Senthilkumar, N.; Cheng, C.-H. Effects of Intramolecular Hydrogen Bonding on the Conformation and Luminescence Properties of Dibenzoylpyridine-Based Thermally Activated Delayed Fluorescence Materials. *J. Mater. Chem. C* **2019**, *7*, 13104-13110.
(278) Imagawa, T.; Hirata, S.; Totani, K.; Watanabe, T.; Vacha, M. Thermally Activated Delayed Fluorescence with Circularly Polarized Luminescence Characteristics. *Chem. Commun.* **2015**, *51*, 13268-13271.
(279) Hao, F.-Y.; Shi, Y.-Z.; Wang, K.; Xiong, S.-Y.; Fan, X.-C.; Wu, L.; Zheng, C.-J.; Li, Y.-Q.; Ou, X.-M.; Zhang, X.-H. Chiral Thermally Activated Delayed Fluorescence Emitters with Dual Conformations Based on a Pair of Enantiomeric Donors Containing Asymmetric Carbons. *Dyes Pigm.* **2020**, *178*, 108336.

(280) Ni, F.; Huang, C.-W.; Tang, Y.; Chen, Z.; Wu, Y.; Xia, S.; Cao, X.; Hsu, J.-H.; Lee, W.-K.; Zheng, K.et al. Integrating Molecular Rigidity and Chirality into Thermally Activated Delayed Fluorescence Emitters for Highly Efficient Sky-Blue and Orange Circularly Polarized Electroluminescence. *Mater. Horiz.* **2021**, *8*, 547-555.
(281) Yang, Y.; Li, N.; Miao, J.; Cao, X.; Ying, A.; Pan, K.; Lv, X.; Ni, F.; Huang, Z.; Gong, S.; Yang, C. Chiral Multi-Resonance TADF Emitters Exhibiting Narrowband Circularly Polarized Electroluminescence with an EQE of 37.2. *Angew. Chem., Int. Ed.* **2022**, *61*, e202202227.
(282) Yang, S. Y.; Wang, Y. K.; Peng, C. C.; Wu, Z. G.; Yuan, S.; Yu, Y. J.; Li, H.; Wang, T. T.; Li, H. C.; Zheng, Y. X.et al. Circularly Polarized Thermally Activated Delayed Fluorescence Emitters in through-Space Charge Transfer on Asymmetric Spiro Skeletons. *J. Am. Chem. Soc.* **2020**, *142*, 17756-17765.
(283) Zhang, Y. P.; Liang, X.; Luo, X. F.; Song, S. Q.; Li, S.; Wang, Y.; Mao, Z. P.; Xu, W. Y.; Zheng, Y. X.; Zuo, J. L.; Pan, Y. Chiral Spiro-Axis Induced Blue Thermally Activated Delayed Fluorescence Material for Efficient Circularly Polarized OLEDs with Low Efficiency Roll-Off. *Angew. Chem., Int. Ed.* **2021**, *60*, 8435-8440.
(284) Hao, Z.; Li, N.; Miao, J.; Huang, Z.; Lv, X.; Cao, X. Chiral Sulfoximine-Based TADF Emitter for Circularly Polarized Luminescence and Highly Efficient OLEDs. *Chem. Eng. J.* **2023**, *454*, 140070.
(285) Huang, Z.; Huang, C.-W.; Tang, Y.-K.; Xiao, Z.; Li, N.; Hua, T.; Cao, X.; Zhou, C.; Wu, C.-C.; Yang, C. Chiral Thermally Activated Delayed Fluorescence Emitters for Circularly Polarized Luminescence and Efficient Deep Blue OLEDs. *Dyes Pigm.* **2022**, *197*, 109860.
(286) Zhang, Y.-P.; Song, S.-Q.; Mao, M.-X.; Li, C.-H.; Zheng, Y.-X.; Zuo, J.-L. Efficient Circularly Polarized Photoluminescence and Electroluminescence of Chiral Spiro-Skeleton Based Thermally Activated Delayed Fluorescence Molecules. *Sci. China Chem.* **2022**, *65*, 1347-1355.
(287) Wang, Y.; Zhang, Y.; Hu, W.; Quan, Y.; Li, Y.; Cheng, Y. Circularly Polarized Electroluminescence of Thermally Activated Delayed Fluorescence-Active Chiral Binaphthyl-Based Luminogens. *ACS Appl. Mater. Interfaces* **2019**, *11*, 26165-26173.
(288) Yan, Z. P.; Liu, T. T.; Wu, R.; Liang, X.; Li, Z. Q.; Zhou, L.; Zheng, Y. X.; Zuo, J. L. Chiral Thermally Activated Delayed Fluorescence Materials Based on R/S-N2, N2′-Diphenyl-[1,1′-Binaphthalene]-2,2′-Diamine Donor with Narrow Emission Spectra for Highly Efficient Circularly Polarized Electroluminescence. *Adv. Funct. Mater.* **2021**, *31*, 2103875.
(289) Yan, Z. P.; Yuan, L.; Zhang, Y.; Mao, M. X.; Liao, X. J.; Ni, H. X.; Wang, Z. H.; An, Z.; Zheng, Y. X.; Zuo, J. L. A Chiral Dual-Core Organoboron Structure Realizes Dual-Channel Enhanced Ultrapure Blue Emission and Highly Efficient Circularly Polarized Electroluminescence. *Adv. Mater.* **2022**, *34*, 2204253.
(290) Li, M.; Wang, Y. F.; Zhang, D. D.; Duan, L.; Chen, C. F. Axially Chiral TADF-Active Enantiomers Designed for Efficient Blue Circularly Polarized Electroluminescence. *Angew. Chem., Int. Ed.* **2020**, *59*, 3500-3504.
(291) Tu, Z. L.; Yan, Z. P.; Liang, X.; Chen, L.; Wu, Z. G.; Wang, Y.; Zheng, Y. X.; Zuo, J. L.; Pan, Y. Axially Chiral Biphenyl Compound-Based Thermally Activated Delayed Fluorescent Materials for High-Performance Circularly Polarized Organic Light-Emitting Diodes. *Adv. Sci.* **2020**, *7*, 2000804.
(292) Tu, Z. L.; Lu, J. J.; Luo, X. F.; Hu, J. J.; Li, S.; Wang, Y.; Zheng, Y. X.; Zuo, J. L.; Pan, Y. Blue Axially Chiral Biphenyl Based Thermally Activated Delayed Fluorescence Materials for Efficient Circularly Polarized OLEDs. *Adv. Optical Mater.* **2021**, *9*, 2100596.
(293) Sumsalee, P.; Abella, L.; Roisnel, T.; Lebrequier, S.; Pieters, G.; Autschbach, J.; Crassous, J.; Favereau, L. Axial and Helical Thermally Activated Delayed Fluorescence Bicarbazole Emitters: Opposite Modulation of Circularly Polarized Luminescence through Intramolecular Charge-Transfer Dynamics. *J. Mater. Chem. C* **2021**, *9*, 11905-11914.
(294) Poulard, L.; Kasemthaveechok, S.; Coehlo, M.; Kumar, R. A.; Frederic, L.; Sumsalee, P.; d'Anfray, T.; Wu, S.; Wang, J.; Matulaitis, T.et al. Circularly Polarized-Thermally Activated

Delayed Fluorescent Materials Based on Chiral Bicarbazole Donors. *Chem. Commun.* **2022**, *58*, 6554-6557.
(295) Zhang, M.-Y.; Li, Z.-Y.; Lu, B.; Wang, Y.; Ma, Y.-D.; Zhao, C.-H. Solid-State Emissive Triarylborane-Based [2.2]Paracyclophanes Displaying Circularly Polarized Luminescence and Thermally Activated Delayed Fluorescence. *Org. Lett.* **2018**, *20*, 6868-6871.
(296) Sharma, N.; Spuling, E.; Mattern, Cornelia M.; Li, W.; Fuhr, O.; Tsuchiya, Y.; Adachi, C.; Bräse, S.; Samuel, I. D. W.; Zysman-Colman, E. Turn on of Sky-Blue Thermally Activated Delayed Fluorescence and Circularly Polarized Luminescence (CPL) Via Increased Torsion by a Bulky Carbazolophane Donor. *Chem. Sci.* **2019**, *10*, 6689-6696.
(297) Liao, C.; Zhang, Y.; Ye, S. H.; Zheng, W. H. Planar Chiral [2.2]Paracyclophane-Based Thermally Activated Delayed Fluorescent Materials for Circularly Polarized Electroluminescence. *ACS Appl. Mater. Interfaces* **2021**, *13*, 25186-25192.
(298) Zhang, D. W.; Teng, J. M.; Wang, Y. F.; Han, X. N.; Li, M.; Chen, C. F. D-Pi*-a Type Planar Chiral TADF Materials for Efficient Circularly Polarized Electroluminescence. *Mater. Horiz.* **2021**, *8*, 3417-3423.
(299) Liao, X. J.; Pu, D.; Yuan, L.; Tong, J.; Xing, S.; Tu, Z. L.; Zuo, J. L.; Zheng, W. H.; Zheng, Y. X. Planar Chiral Multiple Resonance Thermally Activated Delayed Fluorescence Materials for Efficient Circularly Polarized Electroluminescence. *Angew. Chem., Int. Ed.* **2023**, *62*, e202217045.
(300) Yang, S. Y.; Zou, S. N.; Kong, F. C.; Liao, X. J.; Qu, Y. K.; Feng, Z. Q.; Zheng, Y. X.; Jiang, Z. Q.; Liao, L. S. A Narrowband Blue Circularly Polarized Thermally Activated Delayed Fluorescence Emitter with a Hetero-Helicene Structure. *Chem. Commun.* **2021**, *57*, 11041-11044.
(301) Yang, S.-Y.; Tian, Q.-S.; Liao, X.-J.; Wu, Z.-G.; Shen, W.-S.; Yu, Y.-J.; Feng, Z.-Q.; Zheng, Y.-X.; Jiang, Z.-Q.; Liao, L.-S. Efficient Circularly Polarized Thermally Activated Delayed Fluorescence Hetero-[4]Helicene with Carbonyl-/Sulfone-Bridged Triarylamine Structures. *J. Mater. Chem. C* **2022**, *10*, 4393-4401.
(302) dos Santos, J. M.; Sun, D.; Moreno-Naranjo, J. M.; Hall, D.; Zinna, F.; Ryan, S. T. J.; Shi, W.; Matulaitis, T.; Cordes, D. B.; Slawin, A. M. Z.et al. An S-Shaped Double Helicene Showing Both Multi-Resonance Thermally Activated Delayed Fluorescence and Circularly Polarized Luminescence. *J. Mater. Chem. C* **2022**, *10*, 4861-4870.
(303) Ning, W.; Wang, H.; Gong, S.; Zhong, C.; Yang, C. Simple Sulfone-Bridged Heterohelicene Structure Realizes Ultraviolet Narrowband Thermally Activated Delayed Fluorescence, Circularly Polarized Luminescence, and Room Temperature Phosphorescence. *Sci. China Chem.* **2022**, *65*, 1715-1719.
(304) Wu, X.; Huang, J. W.; Su, B. K.; Wang, S.; Yuan, L.; Zheng, W. Q.; Zhang, H.; Zheng, Y. X.; Zhu, W.; Chou, P. T. Fabrication of Circularly Polarized MR-TADF Emitters with Asymmetrical Peripheral-Lock Enhancing Helical B/N-Doped Nanographenes. *Adv. Mater.* **2022**, *34*, 2105080.
(305) Yang, W.; Li, N.; Miao, J.; Zhan, L.; Gong, S.; Huang, Z.; Yang, C. Simple Double Hetero[5]Helicenes Realize Highly Efficient and Narrowband Circularly Polarized Organic Light-Emitting Diodes. *CCS Chem.* **2022**, *4*, 3463-3471.
(306) Zhang, Y.; Zhang, D.; Huang, T.; Gillett, A. J.; Liu, Y.; Hu, D.; Cui, L.; Bin, Z.; Li, G.; Wei, J.; Duan, L. Multi-Resonance Deep-Red Emitters with Shallow Potential-Energy Surfaces to Surpass Energy-Gap Law*. *Angew. Chem., Int. Ed.* **2021**, *60*, 20498-20503.
(307) Li, J. K.; Chen, X. Y.; Guo, Y. L.; Wang, X. C.; Sue, A. C.; Cao, X. Y.; Wang, X. Y. B,N-Embedded Double Hetero[7]Helicenes with Strong Chiroptical Responses in the Visible Light Region. *J. Am. Chem. Soc.* **2021**, *143*, 17958-17963.
(308) Feuillastre, S.; Pauton, M.; Gao, L.; Desmarchelier, A.; Riives, A. J.; Prim, D.; Tondelier, D.; Geffroy, B.; Muller, G.; Clavier, G. Design and Synthesis of New Circularly Polarized Thermally Activated Delayed Fluorescence Emitters. *J. Am. Chem. Soc.* **2016**, *138*, 3990-3993.
(309) Song, F.; Xu, Z.; Zhang, Q.; Zhao, Z.; Zhang, H.; Zhao, W.; Qiu, Z.; Qi, C.; Zhang, H.; Sung, H. H. Y.et al. Highly Efficient Circularly Polarized Electroluminescence from Aggregation-

Induced Emission Luminogens with Amplified Chirality and Delayed Fluorescence. *Adv. Funct. Mater.* **2018**, *28*, 1800051.
(310) Sun, S.; Wang, J.; Chen, L.; Chen, R.; Jin, J.; Chen, C.; Chen, S.; Xie, G.; Zheng, C.; Huang, W. Thermally Activated Delayed Fluorescence Enantiomers for Solution-Processed Circularly Polarized Electroluminescence. *J. Mater. Chem. C* **2019**, *7*, 14511-14516.
(311) Frédéric, L.; Desmarchelier, A.; Plais, R.; Lavnevich, L.; Muller, G.; Villafuerte, C.; Clavier, G.; Quesnel, E.; Racine, B.; Meunier-Della-Gatta, S.et al. Maximizing Chiral Perturbation on Thermally Activated Delayed Fluorescence Emitters and Elaboration of the First Top-Emission Circularly Polarized OLED. *Adv. Funct. Mater.* **2020**, *30*, 2004838.
(312) Zhou, L.; Ni, F.; Li, N.; Wang, K.; Xie, G.; Yang, C. Tetracoordinate Boron-Based Multifunctional Chiral Thermally Activated Delayed Fluorescence Emitters. *Angew. Chem., Int. Ed.* **2022**, *61*, e202203844.
(313) Xue, P.; Wang, X.; Wang, W.; Zhang, J.; Wang, Z.; Jin, J.; Zheng, C.; Li, P.; Xie, G.; Chen, R. Solution-Processable Chiral Boron Complexes for Circularly Polarized Red Thermally Activated Delayed Fluorescent Devices. *ACS Appl. Mater. Interfaces* **2021**, *13*, 47826-47834.
(314) Wu, Z.-G.; Han, H.-B.; Yan, Z.-P.; Luo, X.-F.; Wang, Y.; Zheng, Y.-X.; Zuo, J.-L.; Pan, Y. Chiral Octahydro-Binaphthol Compound-Based Thermally Activated Delayed Fluorescence Materials for Circularly Polarized Electroluminescence with Superior EQE of 32.6% and Extremely Low Efficiency Roll-Off. *Adv. Mater.* **2019**, *31*, 1900524.
(315) Wu, Z.-G.; Yan, Z.-P.; Luo, X.-F.; Yuan, L.; Liang, W.-Q.; Wang, Y.; Zheng, Y.-X.; Zuo, J.-L.; Pan, Y. Non-Doped and Doped Circularly Polarized Organic Light-Emitting Diodes with High Performances Based on Chiral Octahydro-Binaphthyl Delayed Fluorescent Luminophores. *J. Mater. Chem. C* **2019**, *7*, 7045-7052.
(316) Liu, T. T.; Yan, Z. P.; Hu, J. J.; Yuan, L.; Luo, X. F.; Tu, Z. L.; Zheng, Y. X. Chiral Thermally Activated Delayed Fluorescence Emitters-Based Efficient Circularly Polarized Organic Light-Emitting Diodes Featuring Low Efficiency Roll-Off. *ACS Appl. Mater. Interfaces* **2021**, *13*, 56413-56419.
(317) Xu, Y.; Wang, Q.; Cai, X.; Li, C.; Wang, Y. Highly Efficient Electroluminescence from Narrowband Green Circularly Polarized Multiple Resonance Thermally Activated Delayed Fluorescence Enantiomers. *Adv. Mater.* **2021**, *33*, 2100652.
(318) Teng, J. M.; Zhang, D. W.; Wang, Y. F.; Chen, C. F. Chiral Conjugated Thermally Activated Delayed Fluorescent Polymers for Highly Efficient Circularly Polarized Polymer Light-Emitting Diodes. *ACS Appl. Mater. Interfaces* **2022**, *14*, 1578-1586.
(319) Xie, F. M.; Zhou, J. X.; Zeng, X. Y.; An, Z. D.; Li, Y. Q.; Han, D. X.; Duan, P. F.; Wu, Z. G.; Zheng, Y. X.; Tang, J. X. Efficient Circularly Polarized Electroluminescence from Chiral Thermally Activated Delayed Fluorescence Emitters Featuring Symmetrical and Rigid Coplanar Acceptors. *Adv. Optical Mater.* **2021**, *9*, 2100017.
(320) Zhao, W.-L.; Wang, Y.-F.; Wan, S.-P.; Lu, H.-Y.; Li, M.; Chen, C.-F. Chiral Thermally Activated Delayed Fluorescence-Active Macrocycles Displaying Efficient Circularly Polarized Electroluminescence. *CCS Chem.* **2022**, *4*, 3540-3548.
(321) Wang, Y.-F.; Liu, X.; Zhu, Y.; Li, M.; Chen, C.-F. Aromatic-Imide-Based TADF Enantiomers for Efficient Circularly Polarized Electroluminescence. *J. Mater. Chem. C* **2022**, *10*, 4805-4812.
(322) Li, M.; Li, S.-H.; Zhang, D.; Cai, M.; Duan, L.; Fung, M.-K.; Chen, C.-F. Stable Enantiomers Displaying Thermally Activated Delayed Fluorescence: Efficient OLEDs with Circularly Polarized Electroluminescence. *Angew. Chem., Int. Ed.* **2018**, *57*, 2889-2893.
(323) Wang, Y.-F.; Lu, H.-Y.; Chen, C.; Li, M.; Chen, C.-F. 1,8-Naphthalimide-Based Circularly Polarized TADF Enantiomers as the Emitters for Efficient Orange-Red OLEDs. *Org. Electron.* **2019**, *70*, 71-77.
(324) Zheng, Y.; Zhang, L.; Huang, Z.; Li, S.; Zuo, L.; Liang, Y.; Liu, C.; Luo, S.; Shi, G.; Zhao, Z.et al. Bright Organic Mechanoluminescence and Remarkable Mechanofluorochromism from Circularly Polarized TADF Enantiomers with Aggregation-Induced Emission Properties. *Chem. Eur. J.* **2022**, DOI:10.1002/chem.202202594 10.1002/chem.202202594, e202202594.
(325) Wang, Y. F.; Li, M.; Teng, J. M.; Zhou, H. Y.; Chen, C. F. High-Performance Solution-Processed Nondoped Circularly Polarized OLEDs with Chiral Triptycene

Scaffold-Based TADF Emitters Realizing over 20% External Quantum Efficiency. *Adv. Funct. Mater.* **2021**, *31*, 2106418.

(326) Wang, Y. F.; Li, M.; Teng, J. M.; Zhou, H. Y.; Zhao, W. L.; Chen, C. F. Chiral TADF-Active Polymers for High-Efficiency Circularly Polarized Organic Light-Emitting Diodes. *Angew. Chem., Int. Ed.* **2021**, *60*, 23619-23624.

(327) Sumsalee, P.; Abella, L.; Kasemthaveechok, S.; Vanthuyne, N.; Cordier, M.; Pieters, G.; Autschbach, J.; Crassous, J.; Favereau, L. Luminescent Chiral Exciplexes with Sky-Blue and Green Circularly Polarized-Thermally Activated Delayed Fluorescence. *Chem. Eur. J.* **2021**, *27*, 16505-16511.

(328) Gu, Q.; Chen, Z.; Xie, W.; Qiu, W.; Peng, X.; Jiao, Y.; Li, M.; Liu, Z.; Sun, G.; Lu, Y.et al. Chiral Exciplex Acceptor Enables Circularly Polarized Electroluminescence with High Dissymmetry Factor Close to 10−2. *Adv. Optical Mater.* **2022**, *10*, 2201793.

(329) Zhu, Y.; Chen, Z.; Ying, A.; Gong, S.; Wang, T.; Yang, C. Nematic Liquid Crystals Induce and Amplify the Circularly Polarized Luminescence of Chiral TADF Emitters. *J. Mater. Chem. C* **2022**, *10*, 5065-5069.

(330) Goushi, K.; Yoshida, K.; Sato, K.; Adachi, C. Organic Light-Emitting Diodes Employing Efficient Reverse Intersystem Crossing for Triplet-to-Singlet State Conversion. *Nat. Photonics* **2012**, *6*, 253-258.

(331) Goushi, K.; Adachi, C. Efficient Organic Light-Emitting Diodes through up-Conversion from Triplet to Singlet Excited States of Exciplexes. *Appl. Phys. Lett.* **2012**, *101*, 023306.

(332) Hung, W. Y.; Fang, G. C.; Chang, Y. C.; Kuo, T. Y.; Chou, P. T.; Lin, S. W.; Wong, K. T. Highly Efficient Bilayer Interface Exciplex for Yellow Organic Light-Emitting Diode. *ACS Appl Mater Interfaces* **2013**, *5*, 6826-6831.

(333) Li, J.; Nomura, H.; Miyazaki, H.; Adachi, C. Highly Efficient Exciplex Organic Light-Emitting Diodes Incorporating a Heptazine Derivative as an Electron Acceptor. *Chem. Commun.* **2014**, *50*, 6174-6176.

(334) Chapran, M.; Pander, P.; Vasylieva, M.; Wiosna-Salyga, G.; Ulanski, J.; Dias, F. B.; Data, P. Realizing 20% External Quantum Efficiency in Electroluminescence with Efficient Thermally Activated Delayed Fluorescence from an Exciplex. *ACS Appl. Mater. Interfaces* **2019**, *11*, 13460-13471.

(335) Keruckiene, R.; Guzauskas, M.; Lapienyte, L.; Simokaitiene, J.; Volyniuk, D.; Cameron, J.; Skabara, P. J.; Sini, G.; Grazulevicius, J. V. An Experimental and Theoretical Study of Exciplex-Forming Compounds Containing Trifluorobiphenyl and 3,6-Di-Tert-Butylcarbazole Units and Their Performance in OLEDs. *J. Mater. Chem. C* **2020**, *8*, 14186-14195.

(336) Liu, W.; Chen, J. X.; Zheng, C. J.; Wang, K.; Chen, D. Y.; Li, F.; Dong, Y. P.; Lee, C. S.; Ou, X. M.; Zhang, X. H. Novel Strategy to Develop Exciplex Emitters for High-Performance OLEDs by Employing Thermally Activated Delayed Fluorescence Materials. *Adv. Funct. Mater.* **2016**, *26*, 2002-2008.

(337) Wu, Q.; Wang, M.; Cao, X.; Zhang, D.; Sun, N.; Wan, S.; Tao, Y. Carbazole/A-Carboline Hybrid Bipolar Compounds as Electron Acceptors in Exciplex or Non-Exciplex Mixed Cohosts and Exciplex-TADF Emitters for High-Efficiency OLEDs. *J. Mater. Chem. C* **2018**, *6*, 8784-8792.

(338) Mamada, M.; Tian, G.; Nakanotani, H.; Su, J.; Adachi, C. The Importance of Excited-State Energy Alignment for Efficient Exciplex Systems Based on a Study of Phenylpyridinato Boron Derivatives. *Angew. Chem., Int. Ed.* **2018**, *57*, 12380-12384.

(339) Cao, H.-T.; Zhao, Y.; Sun, C.; Fang, D.; Xie, L.-H.; Yan, M.-N.; Wei, Y.; Zhang, H.-M.; Huang, W. Novel Electron Acceptor Based on Spiro[Fluorine-9,9′-Xanthene] for Exciplex Thermally Activated Delayed Fluorescence. *Dyes Pigm.* **2018**, *149*, 422-429.

(340) Cao, H.-T.; Wan, J.; Li, B.; Zhang, H.; Xie, L.-H.; Sun, C.; Feng, Q.-Y.; Yu, W.-J.; Huang, W. Highly Efficient Exciplex-Emission from Spiro[Fluorene-9,9′-Xanthene] Derivatives. *Dyes Pigm.* **2021**, *185,* 108894.

(341) Chapran, M.; Lytvyn, R.; Begel, C.; Wiosna-Salyga, G.; Ulanski, J.; Vasylieva, M.; Volyniuk, D.; Data, P.; Grazulevicius, J. V. High-Triplet-Level Phthalimide Based Acceptors for Exciplexes with Multicolor Emission. *Dyes Pigm.* **2019**, *162*, 872-882.

(342) Zhang, M.; Zheng, C.-J.; Zhang, H.-Y.; Yang, H.-Y.; Wang, K.; Shi, Y.-Z.; Lin, H.; Tao, S.-L.; Zhang, X.-H. Thermally Activated Delayed Fluorescence Exciplexes with Phosphor Components Realizing Deep-Red to near-Infrared Electroluminescence. *J. Mater. Chem. C* **2022**, *10*, 15593-15600.

(343) Zhang, M.; Liu, W.; Zheng, C. J.; Wang, K.; Shi, Y. Z.; Li, X.; Lin, H.; Tao, S. L.; Zhang, X. H. Tricomponent Exciplex Emitter Realizing over 20% External Quantum Efficiency in Organic Light-Emitting Diode with Multiple Reverse Intersystem Crossing Channels. *Adv. Sci.* **2019**, *6*, 1801938.

(344) Jeon, S. K.; Jang, H. J.; Lee, J. Y. Ternary Exciplexes for High Efficiency Organic Light-Emitting Diodes by Self-Energy Transfer. *Adv. Optical Mater.* **2019**, *7*, 1801462.

(345) Siddiqui, Q. T.; Awasthi, A. A.; Bhui, P.; Parab, P.; Muneer, M.; Bose, S.; Agarwal, N. TADF and Exciplex Emission in a Xanthone-Carbazole Derivative and Tuning of Its Electroluminescence with Applied Voltage. *RSC Adv* **2019**, *9*, 40248-40254.

(346) Wu, T. L.; Liao, S. Y.; Huang, P. Y.; Hong, Z. S.; Huang, M. P.; Lin, C. C.; Cheng, M. J.; Cheng, C. H. Exciplex Organic Light-Emitting Diodes with Nearly 20% External Quantum Efficiency: Effect of Intermolecular Steric Hindrance between the Donor and Acceptor Pair. *ACS Appl. Mater. Interfaces* **2019**, *11*, 19294-19300.

(347) Hu, Y.; Yu, Y. J.; Yuan, Y.; Jiang, Z. Q.; Liao, L. S. Exciplex-Based Organic Light-Emitting Diodes with near-Infrared Emission. *Adv. Optical Mater.* **2020**, *8*, 1901917.

(348) Wang, X. Q.; Hu, Y.; Yu, Y. J.; Tian, Q. S.; Shen, W. S.; Yang, W. Y.; Jiang, Z. Q.; Liao, L. S. Over 800 Nm Emission Via Harvesting of Triplet Excitons in Exciplex Organic Light-Emitting Diodes. *J. Phys. Chem. Lett* **2021**, *12*, 6034-6040.

(349) Yang, H.-Y.; Zheng, C.-J.; Zhang, M.; Zhao, J.-W.; Shi, Y.-Z.; Pu, C.-P.; Lin, H.; Tao, S.-L.; Zhang, X.-H. Novel Donor-Spacer-Acceptor Compound as the Multifunctional Component of Exciplexes for Efficient Organic Light-Emitting Diodes. *Sci. China Mater.* **2022**, *64*, 460-468.

(350) Hung, W.-Y.; Wang, T.-C.; Chiang, P.-Y.; Peng, B.-J.; Wong, K.-T. Remote Steric Effect as a Facile Strategy for Improving the Efficiency of Exciplex-Based OLEDs. *ACS Appl. Mater. Interfaces* **2017**, *9*, 7355-7361.

(351) Skuodis, E.; Tomkeviciene, A.; Reghu, R.; Peciulyte, L.; Ivaniuk, K.; Volyniuk, D.; Bezvikonnyi, O.; Bagdziunas, G.; Gudeika, D.; Grazulevicius, J. V. OLEDs Based on the Emission of Interface and Bulk Exciplexes Formed by Cyano-Substituted Carbazole Derivative. *Dyes Pigm.* **2017**, *139*, 795-807.

(352) Colella, M.; Danos, A.; Monkman, A. P. Less Is More: Dilution Enhances Optical and Electrical Performance of a TADF Exciplex. *J. Phys. Chem. Lett* **2019**, *10*, 793-798.

(353) Yuan, P.; Guo, X.; Qiao, X.; Yan, D.; Ma, D. Improvement of the Electroluminescence Performance of Exciplex-Based OLEDs by Effective Utilization of Long-Range Coupled Electron–Hole Pairs. *Adv. Optical Mater.* **2019**, *7*, 1801648.

(354) Pu, Y. J.; Koyama, Y.; Otsuki, D.; Kim, M.; Chubachi, H.; Seino, Y.; Enomoto, K.; Aizawa, N. Exciplex Emissions Derived from Exceptionally Long-Distance Donor and Acceptor Molecules. *Chem Sci* **2019**, *10*, 9203-9208.

(355) Zhang, M.; Wang, K.; Zheng, C. J.; Wang, D. Q.; Shi, Y. Z.; Lin, H.; Tao, S. L.; Li, X.; Zhang, X. H. Development of Red Exciplex for Efficient OLEDs by Employing a Phosphor as a Component. *Front. Chem.* **2019**, *7*, 16.

(356) Chen, W.-C.; Huang, B.; Ni, S.-F.; Xiong, Y.; Rogach, A. L.; Wan, Y.; Shen, D.; Yuan, Y.; Chen, J.-X.; Lo, M.-F.et al. Deep-Red/near-Infrared Electroluminescence from Single-Component Charge-Transfer Complex Via Thermally Activated Delayed Fluorescence Channel. *Adv. Funct. Mater.* **2019**, *29*, 1903112.

(357) Hu, Y.-C.; Lin, Z.-L.; Huang, T.-C.; Lee, J.-W.; Wei, W.-C.; Ko, T.-Y.; Lo, C.-Y.; Chen, D.-G.; Chou, P.-T.; Hung, W.-Y.; Wong, K.-T. New Exciplex Systems Composed of Triazatruxene Donors and N-Heteroarene-Cored Acceptors. *Mater. Chem. Front.* **2020**, *4*, 2029-2039.

(358) Hu, Y. C.; Chen, L. M.; Lin, Z. L.; Lee, J. W.; Wei, W. C.; Ko, T. Y.; Lo, C. Y.; Hung, W. Y.; Wong, K. T. Suppressing Intermolecular Interactions for Enhancing the Performance of Exciplex-Based OLEDs. *J. Chin. Chem. Soc.* **2022**, *69*, 1485-1494.

(359) Wei, X.; Hu, T.; Li, Z.; Liu, Y.; Hu, X.; Gao, H.; Liu, G.; Wang, P.; Yi, Y.; Wang, Y. Rational Strategy of Exciplex-Type Thermally Activated Delayed Fluorescent (TADF) Emitters: Stacking of Donor and Acceptor Units of the Intramolecular TADF Molecule. *Chem. Eng. J.* **2021**, *433*, 133546.

(360) Chen, C.-H.; Cheng, J.-T.; Ding, W.-C.; Lin, Z.-L.; Chen, Y.-S.; Chiu, T.-L.; Lo, Y.-C.; Lee, J.-H.; Wong, K.-T. New Carboline-Based Donors for Green Exciplex-Forming Systems. *J. Chin. Chem. Soc. (Taipei, Taiwan)* **2021**, *68*, 482-490.

(361) Zhang, M.; Zheng, C. J.; Wang, K.; Shi, Y. Z.; Wang, D. Q.; Li, X.; Lin, H.; Tao, S. L.; Zhang, X. H. Hydrogen-Bond-Assisted Exciplex Emitters Realizing Improved Efficiencies and Stabilities in Organic Light Emitting Diodes. *Adv. Funct. Mater.* **2021**, *31*, 2010100.

(362) Voll, C.-C. A.; Markopoulos, G.; Wu, T. C.; Welborn, M.; Engelhart, J. U.; Rochat, S.; Han, G. G. D.; Sazama, G. T.; Lin, T.-A.; Van Voorhis, T.et al. Lock-and-Key Exciplexes for Thermally Activated Delayed Fluorescence. *Org. Mater.* **2020**, *2*, 1-10.

(363) Wang, Z.; Wang, H.; Zhu, J.; Wu, P.; Shen, B.; Dou, D.; Wei, B. Manipulation of Thermally Activated Delayed Fluorescence of Blue Exciplex Emission: Fully Utilizing Exciton Energy for Highly Efficient Organic Light Emitting Diodes with Low Roll-Off. *ACS Appl. Mater. Interfaces* **2017**, *9*, 21346-21354.

(364) Guzauskas, M.; Volyniuk, D.; Tomkeviciene, A.; Pidluzhna, A.; Lazauskas, A.; Grazulevicius, J. V. Dual Nature of Exciplexes: Exciplex-Forming Properties of Carbazole and Fluorene Hybrid Trimers. *J. Mater. Chem. C* **2019**, *7*, 25-32.

(365) Hippola, C.; Danilovic, D.; Bhattacharjee, U.; Perez-Bolivar, C.; Sachinthani, K. A. N.; Nelson, T. L.; Anzenbacher, P.; Petrich, J. W.; Shinar, R.; Shinar, J. Bright Deep Blue TADF OLEDs: The Role of Triphenylphosphine Oxide in Npb/Tpbi:Pph3o Exciplex Emission. *Adv. Optical Mater.* **2020**, *8*, 0191282.

(366) Li, J.; Gong, H.; Zhang, J.; Liu, H.; Tao, L.; Wang, Y.; Guo, Q. Efficient Exciplex-Based Deep-Blue Organic Light-Emitting Diodes Employing a Bis(4-Fluorophenyl)Amine-Substituted Heptazine Acceptor. *Molecules* **2021**, *26*, 5568.

(367) Huang, C.-Y.; Ho, S.-Y.; Lai, C.-H.; Ko, C.-L.; Wei, Y.-C.; Lin, J.-A.; Chen, D.-G.; Ko, T.-Y.; Wong, K.-T.; Zhang, Z.et al. Insights into Energy Transfer Pathways between the Exciplex Host and Fluorescent Guest: Attaining Highly Efficient 710 Nm Electroluminescence. *J. Mater. Chem. C* **2020**, *8*, 5704-5714.

(368) Chen, Y. S.; Luo, D.; Wei, W. C.; Chen, B. L.; Yeh, T. H.; Liu, S. W.; Wong, K. T. New Exciplex-Forming Co-Host System and Thienothiadazole-Based Fluorescent Emitter for High-Efficiency and Promising Stability near-Infrared OLED. *Adv. Optical Mater.* **2022**, *10*, 2101952.

(369) Chen, J.-X.; Wang, H.; Wang, K.; Zhang, X.; Zhou, L.; Shi, Y.-Z.; Yu, J.; Zhang, X.-H. High-Performance Red and White Organic Light-Emitting Diodes Based on a Novel Red Thermally Activated Delayed Fluorescence Emitter in an Exciplex Matrix. *Mater. Today Energy* **2021**, *21*, 100818.

(370) Moon, C. K.; Suzuki, K.; Shizu, K.; Adachi, C.; Kaji, H.; Kim, J. J. Combined Inter-and Intramolecular Charge-Transfer Processes for Highly Efficient Fluorescent Organic Light-Emitting Diodes with Reduced Triplet Exciton Quenching. *Adv. Mater.* **2017**, *29*, 1606448.

(371) Sasabe, H.; Sato, R.; Suzuki, K.; Watanabe, Y.; Adachi, C.; Kaji, H.; Kido, J. Ultrahigh Power Efficiency Thermally Activated Delayed Fluorescent OLEDs by the Strategic Use of Electron-Transport Materials. *Adv. Optical Mater.* **2018**, *6*, 1800376.

(372) Lee, C.-C.; Amin, N. R. A.; Xu, J.-J.; Wang, B.-C.; Luo, D.; Sutanto, K.; Biring, S.; Liu, S.-W.; Chen, C.-H. Structural Effect of Phenylcarbazole-Based Molecules on the Exciplex-Forming Co-Host System to Achieve Highly Efficient Phosphorescent OLEDs with Low Efficiency Roll-Off. *J. Mater. Chem. C* **2021**, *9*, 9453-9464.

(373) Jia, L.; Jin, L.; Yuan, K.; Chen, L.; Yuan, J.; Xu, S.; Lv, W.; Chen, R. High-Performance Exciplex-Type Host for Multicolor Phosphorescent Organic Light-Emitting Diodes with Low Turn-on Voltages. *ACS Sustain. Chem. Eng.* **2018**, *6*, 8809-8815.

(374) Shih, C. J.; Lee, C. C.; Yeh, T. H.; Biring, S.; Kesavan, K. K.; Amin, N. R. A.; Chen, M. H.; Tang, W. C.; Liu, S. W.; Wong, K. T. Versatile Exciplex-Forming Co-Host for Improving

Efficiency and Lifetime of Fluorescent and Phosphorescent Organic Light-Emitting Diodes. *ACS Appl. Mater. Interfaces* **2018**, *10*, 24090-24098.
(375) Liang, B.; Wang, J.; Cheng, Z.; Wei, J.; Wang, Y. Exciplex-Based Electroluminescence: Over 21% External Quantum Efficiency and Approaching 100 Lm/W Power Efficiency. *J Phys Chem Lett* **2019**, *10*, 2811-2816.
(376) Colella, M.; Pander, P.; Pereira, D. d. S.; Monkman, A. P. Interfacial TADF Exciplex as a Tool to Localize Excitons, Improve Efficiency, and Increase OLED Lifetime. *ACS Appl. Mater. Interfaces* **2018**, *10*, 40001-40007.
(377) Tian, Q.-S.; Zhu, X.-D.; Liao, L.-S. Highly Efficient Exciplex-Based OLEDs Incorporating a Novel Electron Donor. *Mater. Chem. Front.* **2020**, *4*, 1648-1655.
(378) Duan, C.; Han, C.; Zhang, J.; Zhang, X.; Fan, C.; Xu, H. Manipulating Charge-Transfer Excitons by Exciplex Matrix: Toward Thermally Activated Delayed Fluorescence Diodes with Power Efficiency Beyond 110 lm w−1. *Adv. Funct. Mater* **2021**, *31,* 2102739.
(379) Zhou, Z.; Chen, R.; Jin, P.; Hao, J.; Wu, W.; Yin, B.; Zhang, C.; Yao, J. Interplay between Singlet and Triplet Excited States in Interface Exciplex OLEDs with Fluorescence, Phosphorescence, and TADF Emitters. *Adv. Funct. Mater.* **2023**, *33*, 2211059.
(380) Wang, X.; Zhang, Y.; Yu, Z.; Wu, Y.; Wang, D.; Wu, C.; Ma, H.; Ning, S.; Dong, H.; Wu, Z. Overcoming Energy Loss of Thermally Activated Delayed Fluorescence Sensitized-OLEDs by Developing a Fluorescent Dopant with a Small Singlet–Triplet Energy Splitting. *J. Mater. Chem. C* **2022**, *10*, 1681-1689.
(381) Chen, L.; Lv, J.; Wang, S.; Shao, S.; Wang, L. Dendritic Interfacial Exciplex Hosts for Solution-Processed TADF-OLEDs with Power Efficiency Approaching 100 lm w−1. *Adv. Optical mater.* **2021**, *9*, 2100752.
(382) Xu, T.; Xie, G.; Huang, T.; Liu, H.; Cao, X.; Tang, Y.; Yang, C. Solution-Processed Multiple Exciplexes Via Spirofluorene and S-Triazine Moieties for Red Thermally Activated Delayed Fluorescence Emissive Layer OLEDs. *Org. Electron.* **2021**, *96,* 106184.
(383) Colella, M.; Pander, P.; Monkman, A. P. Solution Processable Small Molecule Based TADF Exciplex OLEDs. *Org. Electron.* **2018**, *62*, 168-173.
(384) Kishore Kesavan, K.; Jayakumar, J.; Lee, M.; Hexin, C.; Sudheendran Swayamprabha, S.; Kumar Dubey, D.; Tung, F.-C.; Wang, C.-W.; Jou, J.-H. Achieving a 32% EQE Solution-Processed Simple Structure OLED Via Exciplex System. *Chem. Eng. J.* **2022***, 435,* 134879.
(385) Huang, Q.; Zhao, S.; Wang, P.; Qin, Z.; Xu, Z.; Song, D.; Qiao, B.; Xu, X. Investigating the Evolution of Exciplex States in Thermally Activated Delayed Fluorescence Organic Light-Emitting Diodes by Transient Measurement. *J. Lumin.* **2018**, *201*, 38-43.
(386) Lin, T.-C.; Sarma, M.; Chen, Y.-T.; Liu, S.-H.; Lin, K.-T.; Chiang, P.-Y.; Chuang, W.-T.; Liu, Y.-C.; Hsu, H.-F.; Hung, W.-Y.et al. Probe Exciplex Structure of Highly Efficient Thermally Activated Delayed Fluorescence Organic Light Emitting Diodes. *Nat. Commun.* **2018**, *9*, 3111.
(387) Grüne, J.; Bunzmann, N.; Meinecke, M.; Dyakonov, V.; Sperlich, A. Kinetic Modeling of Transient Electroluminescence Reveals TTA as an Efficiency-Limiting Process in Exciplex-Based TADF OLEDs. *J. Phys. Chem. C* **2020**, *124*, 25667-25674.
(388) Bunzmann, N.; Weissenseel, S.; Kudriashova, L.; Gruene, J.; Krugmann, B.; Grazulevicius, J. V.; Sperlich, A.; Dyakonov, V. Optically and Electrically Excited Intermediate Electronic States in Donor:Acceptor Based OLEDs. *Mater. Horiz.* **2020**, *7*, 1126-1137.
(389) Nguyen, T. B.; Nakanotani, H.; Hatakeyama, T.; Adachi, C. The Role of Reverse Intersystem Crossing Using a TADF-Type Acceptor Molecule on the Device Stability of Exciplex-Based Organic Light-Emitting Diodes. *Adv. Mater.* **2020**, *32*, 1906614.
(390) He, L.; Bai, R.; Yu, R.; Meng, X.; Tian, M.; Wang, X. Donor/Acceptor Pairs Created by Electrostatic Interaction: Design, Synthesis, and Investigation on the Exciplex Formed within the Pair. *Angew. Chem., Int. Ed.* **2021**, *60*, 6013-6020.
(391) Cekaviciute, M.; Simokaitiene, J.; Volyniuk, D.; Sini, G.; Grazulevicius, J. V. Arylfluorenyl-Substituted Metoxytriphenylamines as Deep Blue Exciplex Forming Bipolar Semiconductors for White and Blue Organic Light Emitting Diodes. *Dyes Pigm.* **2017**, *140*, 187-202.

(392) Tian, Q. S.; Zhang, L.; Hu, Y.; Yuan, S.; Wang, Q.; Liao, L. S. High-Performance White Organic Light-Emitting Diodes with Simplified Structure Incorporating Novel Exciplex-Forming Host. *ACS Appl. Mater. Interfaces* **2018**, *10*, 39116-39123.
(393) Yao, J.; Ying, S.; Qiao, X.; Yang, D.; Chen, J.; Ahamad, T.; Alshehri, S. M.; Ma, D. High Efficiency and Low Roll-Off All Fluorescence White Organic Light-Emitting Diodes by the Formation of Interface Exciplex. *Org. Electron.* **2019**, *67*, 72-78.
(394) Guo, Y.; Zhao, Y.; Miao, Y.; Wang, L.; Li, T.; Wang, H.; Xu, B.; Yu, J. All-Exciplex-Based White Organic Light-Emitting Diodes by Employing an Interface-Free Sandwich Light-Emitting Unit Achieving High Electroluminescence Performance. *J. Mater. Chem. C* **2020**, *8*, 12247-12256.
(395) Tan, X.; Volyniuk, D.; Matulaitis, T.; Keruckas, J.; Ivaniuk, K.; Helzhynskyy, I.; Stakhira, P.; Grazulevicius, J. V. High Triplet Energy Materials for Efficient Exciplex-Based and Full-TADF-Based White OLEDs. *Dyes Pigm.* **2020**, *177*, 108259.
(396) Han, C.; Du, R.; Xu, H.; Han, S.; Ma, P.; Bian, J.; Duan, C.; Wei, Y.; Sun, M.; Liu, X.; Huang, W. Ladder-Like Energy-Relaying Exciplex Enables 100% Internal Quantum Efficiency of White TADF-Based Diodes in a Single Emissive Layer. *Nat. Commun.* **2021**, *12*, 3640.
(397) Kirchhoff, J. R.; Gamache, R. E.; Blaskie, M. W.; Del Paggio, A. A.; Lengel, R. K.; McMillin, D. R. Temperature Dependence of Luminescence from $Cu(Nn)_2^+$ Systems in Fluid Solution. Evidence for the Participation of Two Excited States. *Inorg. Chem.* **1983**, *22*, 2380-2384.
(398) Ichinaga, A. K.; Kirchhoff, J. R.; McMillin, D. R.; Dietrich-Buchecker, C. O.; Marnot, P. A.; Sauvage, J. P. Charge-Transfer Absorption and Emission of $Cu(Nn)_2^+$ Systems. *Inorg. Chem.* **1987**, *26*, 4290-4292.
(399) Felder, D.; Nierengarten, J.-F.; Barigelletti, F.; Ventura, B.; Armaroli, N. Highly Luminescent Cu(I)−Phenanthroline Complexes in Rigid Matrix and Temperature Dependence of the Photophysical Properties. *J. Am. Chem. Soc.* **2001**, *123*, 6291-6299.
(400) Green, O.; Gandhi, B. A.; Burstyn, J. N. Photophysical Characteristics and Reactivity of Bis(2,9-Di-Tert-Butyl-1,10-Phenanthroline)Copper(I). *Inorg. Chem.* **2009**, *48*, 5704-5714.
(401) Rosko, M. C.; Wells, K. A.; Hauke, C. E.; Castellano, F. N. Next Generation Cuprous Phenanthroline Mlct Photosensitizer Featuring Cyclohexyl Substituents. *Inorg. Chem.* **2021**, *60*, 8394-8403.
(402) Smith, C. S.; Mann, K. R. Void Space Containing Crystalline Cu(I) Phenanthroline Complexes as Molecular Oxygen Sensors. *Chem. Mater.* **2009**, *21*, 5042-5049.
(403) Gimeno, L.; Phelan, B. T.; Sprague-Klein, E. A.; Roisnel, T.; Blart, E.; Gourlaouen, C.; Chen, L. X.; Pellegrin, Y. Bulky and Stable Copper(I)-Phenanthroline Complex: Impact of Steric Strain and Symmetry on the Excited-State Properties. *Inorg. Chem.* **2022**, *61*, 7296-7307.
(404) Brown-Xu, S.; Fumanal, M.; Gourlaouen, C.; Gimeno, L.; Quatela, A.; Thobie-Gautier, C.; Blart, E.; Planchat, A.; Riobe, F.; Monnereau, C.et al. Intriguing Effects of Halogen Substitution on the Photophysical Properties of 2,9-(Bis)Halo-Substituted Phenanthrolinecopper(I) Complexes. *Inorg. Chem.* **2019**, *58*, 7730-7745.
(405) Palmer, C. E. A.; McMillin, D. R. Singlets, Triplets, and Exciplexes: Complex, Temperature-Dependent Emissions from $Cu(Dmp)(Pph_3)_2^+$ and $Cu(Phen)(Pph_3)_2^+$ in Solution. *Inorg. Chem.* **1987**, *26*, 3837-3840.
(406) Cuttell, D. G.; Kuang, S.-M.; Fanwick, P. E.; McMillin, D. R.; Walton, R. A. Simple Cu(I) Complexes with Unprecedented Excited-State Lifetimes. *J. Am. Chem. Soc.* **2002**, *124*, 6-7.
(407) Rand, B. P.; Yersin, H.; Adachi, C.; Czerwieniec, R.; Hupfer, A.; van Elsbergen, V. *Singlet Harvesting with Brightly Emitting Cu(I) and Metal-Free Organic Compounds,* Organic Photonics V, **2012**; p 843508.
(408) Czerwieniec, R.; Kowalski, K.; Yersin, H. Highly Efficient Thermally Activated Fluorescence of a New Rigid Cu(I) Complex [Cu(Dmp)(Phanephos)]+. *Dalton Trans.* **2013**, *42*, 9826-9830.
(409) Chen, X.-L.; Yu, R.; Zhang, Q.-K.; Zhou, L.-J.; Wu, X.-Y.; Zhang, Q.; Lu, C.-Z. Rational Design of Strongly Blue-Emitting Cuprous Complexes with Thermally Activated Delayed Fluorescence and Application in Solution-Processed OLEDs. *Chem. Mater.* **2013**, *25*, 3910-3920.

(410) Linfoot, C. L.; Leitl, M. J.; Richardson, P.; Rausch, A. F.; Chepelin, O.; White, F. J.; Yersin, H.; Robertson, N. Thermally Activated Delayed Fluorescence (TADF) and Enhancing Photoluminescence Quantum Yields of $[CuI(Diimine)(Diphosphine)]^{+}$ Complexes—Photophysical, Structural, and Computational Studies. *Inorg. Chem.* **2014**, *53*, 10854-10861.

(411) Zhang, Q.; Chen, J.; Wu, X. Y.; Chen, X. L.; Yu, R.; Lu, C. Z. Outstanding Blue Delayed Fluorescence and Significant Processing Stability of Cuprous Complexes with Functional Pyridine-Pyrazolate Diimine Ligands. *Dalton Trans.* **2015**, *44*, 6706-6710.

(412) Zhang, F.; Guan, Y.; Chen, X.; Wang, S.; Liang, D.; Feng, Y.; Chen, S.; Li, S.; Li, Z.; Zhang, F.et al. Syntheses, Photoluminescence, and Electroluminescence of a Series of Sublimable Bipolar Cationic Cuprous Complexes with Thermally Activated Delayed Fluorescence. *Inorg. Chem.* **2017**, *56*, 3742-3753.

(413) Chen, X.-L.; Lin, C.-S.; Wu, X.-Y.; Yu, R.; Teng, T.; Zhang, Q.-K.; Zhang, Q.; Yang, W.-B.; Lu, C.-Z. Highly Efficient Cuprous Complexes with Thermally Activated Delayed Fluorescence and Simplified Solution Process OLEDs Using the Ligand as Host. *J. Mater. Chem. C* **2015**, *3*, 1187-1195.

(414) Liang, D.; Chen, X. L.; Liao, J. Z.; Hu, J. Y.; Jia, J. H.; Lu, C. Z. Highly Efficient Cuprous Complexes with Thermally Activated Delayed Fluorescence for Solution-Processed Organic Light-Emitting Devices. *Inorg. Chem.* **2016**, *55*, 7467-7475.

(415) Mohankumar, M.; Holler, M.; Meichsner, E.; Nierengarten, J. F.; Niess, F.; Sauvage, J. P.; Delavaux-Nicot, B.; Leoni, E.; Monti, F.; Malicka, J. M.et al. Heteroleptic Copper(I) Pseudorotaxanes Incorporating Macrocyclic Phenanthroline Ligands of Different Sizes. *J. Am. Chem. Soc.* **2018**, *140*, 2336-2347.

(416) Fresta, E.; Weber, M. D.; Fernandez-Cestau, J.; Costa, R. D. White Light-Emitting Electrochemical Cells Based on Deep-Red Cu(I) Complexes. *Adv. Optical Mater.* **2019**, *7*, 1900830.

(417) Farias, G.; Salla, C. A. M.; S. Heying, R.; Bortoluzzi, A. J.; Curcio, S. F.; Cazati, T.; dos Santos, P. L.; Monkman, A. P.; Souza, B. d.; Bechtold, I. H. Reducing Lifetime in Cu(I) Complexes with Thermally Activated Delayed Fluorescence and Phosphorescence Promoted by Chalcogenolate–Diimine Ligands. *J. Mater. Chem. C* **2020**, *8*, 14595-14604.

(418) Wang, J.; Chen, H.; Xu, S.; Su, Q.; Zhao, F.; He, H. Highly Effective Luminescence Stemmed from Thermally Activated Delayed Fluorescence (TADF) and Phosphorescence for the New Four-Coordinate Copper(I) Complexes Containing N-Heterocyclic Carbene (Nhc) Ligands. *J. Photochem. Photobiol., A* **2020**, *387*, 112104.

(419) Teng, T.; Xiong, J.; Cheng, G.; Zhou, C.; Lv, X.; Li, K. Solution-Processed OLEDs Based on Thermally Activated Delayed Fluorescence Copper(I) Complexes with Intraligand Charge-Transfer Excited State. *Molecules* **2021**, *26*, 1125.

(420) Huang, C. H.; Yang, M.; Chen, X. L.; Lu, C. Z. Bright Bluish-Green Emitting Cu(I) Complexes Exhibiting Efficient Thermally Activated Delayed Fluorescence. *Dalton Trans.* **2021**, *50*, 5171-5176.

(421) Fresta, E.; Mahoro, G. U.; Cavinato, L. M.; Lohier, J. F.; Renaud, J. L.; Gaillard, S.; Costa, R. D. Novel Red-Emitting Copper(I) Complexes with Pyrazine and Pyrimidinyl Ancillary Ligands for White Light-Emitting Electrochemical Cells. *Adv. Optical Mater.* **2022**, *10*, 2101999.

(422) Czerwieniec, R.; Yu, J.; Yersin, H. Blue-Light Emission of Cu(I) Complexes and Singlet Harvesting. *Inorg. Chem.* **2011**, *50*, 8293-8301.

(423) Czerwieniec, R.; Yersin, H. Diversity of Copper(I) Complexes Showing Thermally Activated Delayed Fluorescence: Basic Photophysical Analysis. *Inorg. Chem.* **2015**, *54*, 4322-4327.

(424) Igawa, S.; Hashimoto, M.; Kawata, I.; Yashima, M.; Hoshino, M.; Osawa, M. Highly Efficient Green Organic Light-Emitting Diodes Containing Luminescent Tetrahedral Copper(I) Complexes. *J. Mater. Chem. C* **2013**, *1*, 542-551.

(425) Osawa, M.; Kawata, I.; Ishii, R.; Igawa, S.; Hashimoto, M.; Hoshino, M. Application of Neutral D^{10} Coinage Metal Complexes with an Anionic Bidentate Ligand in Delayed Fluorescence-Type Organic Light-Emitting Diodes. *J. Mater. Chem. C* **2013**, *1*, 4375-4383.

(426) Zhang, J.; Duan, C.; Han, C.; Yang, H.; Wei, Y.; Xu, H. Balanced Dual Emissions from Tridentate Phosphine-Coordinate Copper (I) Complexes toward Highly Efficient Yellow OLEDs. *Adv. Mater.* **2016**, *28*, 5975-5979.
(427) Zhang, W.-J.; Zhou, Z.-X.; Liu, L.; Zhong, X.-X.; Asiri, A. M.; Alamry, K. A.; Li, F.-B.; Zhu, N.-Y.; Wong, W.-Y.; Qin, H.-M. Highly-Efficient Blue Neutral Mononuclear Copper(I) Halide Complexes Containing Bi- and Mono-Dentate Phosphine Ligands. *J. Lumin.* **2018**, *196*, 425-430.
(428) Wei, Q.; Zhang, R.; Liu, L.; Zhong, X. X.; Wang, L.; Li, G. H.; Li, F. B.; Alamry, K. A.; Zhao, Y. From Deep Blue to Green Emitting and Ultralong Fluorescent Copper(I) Halide Complexes Containing Dimethylthiophene Diphosphine and Pph_3 Ligands. *Dalton Trans.* **2019**, *48*, 11448-11459.
(429) Chakkaradhari, G.; Eskelinen, T.; Degbe, C.; Belyaev, A.; Melnikov, A. S.; Grachova, E. V.; Tunik, S. P.; Hirva, P.; Koshevoy, I. O. Oligophosphine-Thiocyanate Copper(I) and Silver(I) Complexes and Their Borane Derivatives Showing Delayed Fluorescence. *Inorg. Chem.* **2019**, *58*, 3646-3660.
(430) Guo, B.-K.; Yang, F.; Wang, Y.-Q.; Wei, Q.; Liu, L.; Zhong, X.-X.; Wang, L.; Gong, J.-K.; Li, F.-B.; Wong, W.-Y.et al. Efficient TADF-OLEDs with Ultra-Soluble Copper(I) Halide Complexes Containing Non-Symmetrically Substituted Bidentate Phosphine and Pph_3 Ligands. *J. Lumin.* **2020**, *220*, 116963.
(431) Klein, M.; Rau, N.; Wende, M.; Sundermeyer, J.; Cheng, G.; Che, C.-M.; Schinabeck, A.; Yersin, H. Cu(I) and Ag(I) Complexes with a New Type of Rigid Tridentate N,P,P-Ligand for Thermally Activated Delayed Fluorescence and OLEDs with High External Quantum Efficiency. *Chem. Mater.* **2020**, *32*, 10365-10382.
(432) Yu, X.; Li, X.; Cai, Z.; Sun, L.; Wang, C.; Rao, H.; Wei, C.; Bian, Z.; Jin, Q.; Liu, Z. Mechanochromic Properties in a Mononuclear Cu(I) Complex without Cuprophilic Interactions. *Chem. Commun.* **2021**, *57*, 5082-5085.
(433) Alconchel, A.; Crespo, O.; Garcia-Orduna, P.; Gimeno, M. C. Closo- or Nido-Carborane Diphosphane as Responsible for Strong Thermochromism or Time Activated Delayed Fluorescence (TADF) in $[Cu(N^\wedge N)(P^\wedge P)]^{0/+}$. *Inorg. Chem.* **2021**, *60*, 18521-18528.
(434) Bergmann, L.; Friedrichs, J.; Mydlak, M.; Baumann, T.; Nieger, M.; Brase, S. Outstanding Luminescence from Neutral Copper(I) Complexes with Pyridyl-Tetrazolate and Phosphine Ligands. *Chem. Commun.* **2013**, *49*, 6501-6503.
(435) Chen, J. L.; Cao, X. F.; Wang, J. Y.; He, L. H.; Liu, Z. Y.; Wen, H. R.; Chen, Z. N. Synthesis, Characterization, and Photophysical Properties of Heteroleptic Copper(I) Complexes with Functionalized 3-(2'-Pyridyl)-1,2,4-Triazole Chelating Ligands. *Inorg. Chem.* **2013**, *52*, 9727-9740.
(436) Hashimoto, M.; Igawa, S.; Yashima, M.; Kawata, I.; Hoshino, M.; Osawa, M. Highly Efficient Green Organic Light-Emitting Diodes Containing Luminescent Three-Coordinate Copper(I) Complexes. *J. Am. Chem. Soc.* **2011**, *133*, 10348-10351.
(437) Osawa, M.; Hoshino, M.; Hashimoto, M.; Kawata, I.; Igawa, S.; Yashima, M. Application of Three-Coordinate Copper(I) Complexes with Halide Ligands in Organic Light-Emitting Diodes That Exhibit Delayed Fluorescence. *Dalton Trans.* **2015**, *44*, 8369-8378.
(438) Osawa, M. Highly Efficient Blue-Green Delayed Fluorescence from Copper(I) Thiolate Complexes: Luminescence Color Alteration by Orientation Change of the Aryl Ring. *Chem. Commun.* **2014**, *50*, 1801-1803.
(439) Liu, L.-P.; Zhang, R.; Liu, L.; Zhong, X.-X.; Li, F.-B.; Wang, L.; Wong, W.-Y.; Li, G.-H.; Cong, H.-J.; Alharbi, N. S.; Zhao, Y. A New Strategy to Synthesize Three-Coordinate Mononuclear Copper(I) Halide Complexes Containing a Bulky Terphenyl Bidentate Phosphine Ligand and Their Luminescent Properties. *New J. Chem.* **2019**, *43*, 3390-3399.
(440) Yin, X.; Liu, C.; Liu, S.; Cao, M.; Rawson, J. M.; Xu, Y.; Zhang, B. Structural Characterization and Luminescence Properties of Trigonal Cu(I) Iodine/Bromine Complexes Comprising Cation–Π Interactions. *New J. Chem.* **2022**, *46*, 6185-6192.
(441) Nitsch, J.; Kleeberg, C.; Frohlich, R.; Steffen, A. Luminescent Copper(I) Halide and Pseudohalide Phenanthroline Complexes Revisited: Simple Structures, Complicated Excited State Behavior. *Dalton Trans.* **2015**, *44*, 6944-6960.

(442) Leitl, M. J.; Krylova, V. A.; Djurovich, P. I.; Thompson, M. E.; Yersin, H. Phosphorescence Versus Thermally Activated Delayed Fluorescence. Controlling Singlet–Triplet Splitting in Brightly Emitting and Sublimable Cu(I) Compounds. *J. Am. Chem. Soc.* **2014**, *136*, 16032-16038.

(443) Elie, M.; Sguerra, F.; Di Meo, F.; Weber, M. D.; Marion, R.; Grimault, A.; Lohier, J. F.; Stallivieri, A.; Brosseau, A.; Pansu, R. B.et al. Designing Nhc-Copper(I) Dipyridylamine Complexes for Blue Light-Emitting Electrochemical Cells. *ACS Appl. Mater. Interfaces* **2016**, *8*, 14678-14691.

(444) Elie, M.; Weber, M. D.; Di Meo, F.; Sguerra, F.; Lohier, J. F.; Pansu, R. B.; Renaud, J. L.; Hamel, M.; Linares, M.; Costa, R. D.; Gaillard, S. Role of the Bridging Group in Bis-Pyridyl Ligands: Enhancing Both the Photo- and Electroluminescent Features of Cationic (Ipr)Cu(I) Complexes. *Chem. Eur. J.* **2017**, *23*, 16328-16337.

(445) Romanov, A. S.; Becker, C. R.; James, C. E.; Di, D.; Credgington, D.; Linnolahti, M.; Bochmann, M. Copper and Gold Cyclic (Alkyl)(Amino)Carbene Complexes with Sub-Microsecond Photoemissions: Structure and Substituent Effects on Redox and Luminescent Properties. *Chem. Eur. J.* **2017**, *23*, 4625-4637.

(446) Holzel, T.; Belyaev, A.; Terzi, M.; Stenzel, L.; Gernert, M.; Marian, C. M.; Steffen, A.; Ganter, C. Linear Carbene Pyridine Copper Complexes with Sterically Demanding *N,N'*-Bis(Trityl)Imidazolylidene: Syntheses, Molecular Structures, and Photophysical Properties. *Inorg. Chem.* **2021**, *60*, 18529-18543.

(447) Wang, Z.; Zhu, J.; Liu, Z.; Wu, P.; Wang, H.; Zhang, Z.; Wei, B. Thermally Activated Delayed Fluorescence of Co-Deposited Copper(I) Complexes: Cost-Effective Emitters for Highly Efficient Organic Light-Emitting Diodes. *J. Mater. Chem. C* **2017**, *5*, 6982-6988.

(448) Yang, L.; Xu, X.; Zhang, P.; Chen, M.; Chen, G.; Zheng, Y.; Wei, B.; Zhang, J. Photophysical Properties and Stability of Binuclear Emissive Copper(I) Complexes Co-Deposited with Cu*x* (X = Cl, Br, I) and Aza-9,9′-Spirobifluorenes. *Dyes Pigm.* **2019**, *161*, 296-302.

(449) Guo, J.; Zhang, Z.; Wu, P.; Zhu, J.; Dou, D.; Liao, Z.; Xia, R.; Wang, K.; Wang, Z. Co-Deposited Copper(I) Complexes Integrating Phosphorescence and TADF Properties for Highly Efficient OLEDs. *J. Lumin.* **2021**, *239*, 118354.

(450) Tsuboyama, A.; Kuge, K.; Furugori, M.; Okada, S.; Hoshino, M.; Ueno, K. Photophysical Properties of Highly Luminescent Copper(I) Halide Complexes Chelated with 1,2-Bis(Diphenylphosphino)Benzene. *Inorg. Chem.* **2007**, *46*, 1992-2001.

(451) Deaton, J. C.; Switalski, S. C.; Kondakov, D. Y.; Young, R. H.; Pawlik, T. D.; Giesen, D. J.; Harkins, S. B.; Miller, A. J. M.; Mickenberg, S. F.; Peters, J. C. E-Type Delayed Fluorescence of a Phosphine-Supported Cu_2(*M*-NAr_2)$_2$ Diamond Core: Harvesting Singlet and Triplet Excitons in OLEDs. *J. Am. Chem. Soc.* **2010**, *132*, 9499-9508.

(452) Volz, D.; Chen, Y.; Wallesch, M.; Liu, R.; Fléchon, C.; Zink, D. M.; Friedrichs, J.; Flügge, H.; Steininger, R.; Göttlicher, J.et al. Bridging the Efficiency Gap: Fully Bridged Dinuclear Cu(I)-Complexes for Singlet Harvesting in High-Efficiency OLEDs. *Adv. Mater.* **2015**, *27*, 2538-2543.

(453) Hofbeck, T.; Monkowius, U.; Yersin, H. Highly Efficient Luminescence of Cu(I) Compounds: Thermally Activated Delayed Fluorescence Combined with Short-Lived Phosphorescence. *J. Am. Chem. Soc.* **2015**, *137*, 399-404.

(454) Hong, X.; Wang, B.; Liu, L.; Zhong, X.-X.; Li, F.-B.; Wang, L.; Wong, W.-Y.; Qin, H.-M.; Lo, Y. H. Highly Efficient Blue–Green Neutral Dinuclear Copper(I) Halide Complexes Containing Bidentate Phosphine Ligands. *J. Lumin.* **2016**, *180*, 64-72.

(455) Nitsch, J.; Lacemon, F.; Lorbach, A.; Eichhorn, A.; Cisnetti, F.; Steffen, A. Cuprophilic Interactions in Highly Luminescent Dicopper(I)-Nhc-Picolyl Complexes - Fast Phosphorescence or TADF? *Chem. Commun.* **2016**, *52*, 2932-2935.

(456) Wallesch, M.; Verma, A.; Flechon, C.; Flugge, H.; Zink, D. M.; Seifermann, S. M.; Navarro, J. M.; Vitova, T.; Gottlicher, J.; Steininger, R.et al. Towards Printed Organic Light-Emitting Devices: A Solution-Stable, Highly Soluble Cu(I)-Nhetphos. *Chem. Eur. J.* **2016**, *22*, 16400-16405.

(457) Lin, L.; Chen, D.-H.; Yu, R.; Chen, X.-L.; Zhu, W.-J.; Liang, D.; Chang, J.-F.; Zhang, Q.; Lu, C.-Z. Photo- and Electro-Luminescence of Three TADF Binuclear Cu(I) Complexes with Functional Tetraimine Ligands. *J. Mater. Chem. C* **2017**, *5*, 4495-4504.
(458) Jia, J. H.; Chen, X. L.; Liao, J. Z.; Liang, D.; Yang, M. X.; Yu, R.; Lu, C. Z. Highly Luminescent Copper(I) Halide Complexes Chelated with a Tetradentate Ligand (PNNP): Synthesis, Structure, Photophysical Properties and Theoretical Studies. *Dalton Trans.* **2019**, *48*, 1418-1426.
(459) Cao, M.; Zhao, Y.; Gu, M.; Liu, C.; Zhu, Q.; Chen, Y.; Wei, B.; Du, C.; Zhang, B. Syntheses, Crystal Structures and Photophysical Properties of Dinuclear Copper(I) Complexes Bearing Diphenylphosphino-Substituted Benzimidazole Ligands. *ChemistrySelect* **2021**, *6*, 2156-2163.
(460) Busch, J. M.; Zink, D. M.; Di Martino-Fumo, P.; Rehak, F. R.; Boden, P.; Steiger, S.; Fuhr, O.; Nieger, M.; Klopper, W.; Gerhards, M.; Brase, S. Highly Soluble Fluorine Containing Cu(I) Alkylpyrphos TADF Complexes. *Dalton Trans.* **2019**, *48*, 15687-15698.
(461) Baranov, A. Y.; Berezin, A. S.; Samsonenko, D. G.; Mazur, A. S.; Tolstoy, P. M.; Plyusnin, V. F.; Kolesnikov, I. E.; Artem'ev, A. V. New Cu(I) Halide Complexes Showing TADF Combined with Room Temperature Phosphorescence: The Balance Tuned by Halogens. *Dalton Trans.* **2020**, *49*, 3155-3163.
(462) Artem'ev, A. V.; Demyanov, Y. V.; Rakhmanova, M. I.; Bagryanskaya, I. Y. Pyridylarsine-Based Cu(I) Complexes Showing TADF Mixed with Fast Phosphorescence: A Speeding-up Emission Rate Using Arsine Ligands. *Dalton Trans.* **2022**, *51*, 1048-1055.
(463) Xie, M.; Han, C.; Zhang, J.; Xie, G.; Xu, H. White Electroluminescent Phosphine-Chelated Copper Iodide Nanoclusters. *Chem. Mater.* **2017**, *29*, 6606-6610.
(464) Xie, M.; Han, C.; Liang, Q.; Zhang, J.; Xie, G.; Xu, H. Highly Efficient Sky Blue Electroluminescence from Ligand-Activated Copper Iodide Clusters: Overcoming the Limitations of Cluster Light-Emitting Diodes. *Sci. Adv.* **2019**, *5*, eaav9857.
(465) Wu, Y.; Wang, J. Y.; Zhang, L. Y.; Xu, L. J.; Chen, Z. N. Vapor-Triggered Green-to-Yellow Luminescence Conversion Due to the Variation of Ligand Orientations in Tetranuclear Copper(I) Complex. *Inorg. Chem.* **2020**, *59*, 17415-17420.
(466) Titov, A. A.; Filippov, O. A.; Smol'yakov, A. F.; Godovikov, I. A.; Shakirova, J. R.; Tunik, S. P.; Podkorytov, I. S.; Shubina, E. S. Luminescent Complexes of the Trinuclear Silver(I) and Copper(I) Pyrazolates Supported with Bis(Diphenylphosphino)Methane. *Inorg. Chem.* **2019**, *58*, 8645-8656.
(467) Olaru, M.; Rychagova, E.; Ketkov, S.; Shynkarenko, Y.; Yakunin, S.; Kovalenko, M. V.; Yablonskiy, A.; Andreev, B.; Kleemiss, F.; Beckmann, J.; Vogt, M. A Small Cationic Organo-Copper Cluster as Thermally Robust Highly Photo- and Electroluminescent Material. *J. Am. Chem. Soc.* **2020**, *142*, 373-381.
(468) Cao, B. J.; Li, R.; Huang, X. H. Synthesis, Structure and Photophysical Properties of Two Tetranuclear Copper(I) Iodide Complexes Based on Acetylpyridine and Diphosphine Mixed Ligands. *Acta Crystallogr., Sect. C: Cryst. Struct. Commun.* **2021**, *77*, 61-67.
(469) Carbonell-Vilar, J. M.; Fresta, E.; Armentano, D.; Costa, R. D.; Viciano-Chumillas, M.; Cano, J. Photoluminescent Cu(I) Vs. Ag(I) Complexes: Slowing Down Emission in Cu(I) Complexes by Pentacoordinate Low-Lying Excited States. *Dalton Trans.* **2019**, *48*, 9765-9775.
(470) Osawa, M.; Hashimoto, M.; Kawata, I.; Hoshino, M. Photoluminescence Properties of TADF-Emitting Three-Coordinate Silver(I) Halide Complexes with Diphosphine Ligands: A Comparison Study with Copper(I) Complexes. *Dalton Trans.* **2017**, *46*, 12446-12455.
(471) Jia, J.-H.; Liang, D.; Yu, R.; Chen, X.-L.; Meng, L.; Chang, J.-F.; Liao, J.-Z.; Yang, M.; Li, X.-N.; Lu, C.-Z. Coordination-Induced Thermally Activated Delayed Fluorescence: From Non-TADF Donor–Acceptor-Type Ligand to TADF-Active Ag-Based Complexes. *Chem. Mater.* **2020**, *32*, 620-629.
(472) Teng, T.; Li, K.; Cheng, G.; Wang, Y.; Wang, J.; Li, J.; Zhou, C.; Liu, H.; Zou, T.; Xiong, J.et al. Lighting Silver(I) Complexes for Solution-Processed Organic Light-Emitting Diodes and Biological Applications Via Thermally Activated Delayed Fluorescence. *Inorg. Chem.* **2020**, *59*, 12122-12131.

(473) Cai, X. B.; Liang, D.; Yang, M.; Wu, X. Y.; Lu, C. Z.; Yu, R. Efficiently Increasing the Radiative Rate of TADF Material with Metal Coordination. *Chem. Commun.* **2022**, *58*, 8970-8973.
(474) Liang, D.; Jia, J.-H.; Cai, X.-B.; Zhao, Y.-Q.; Wang, Z.-Q.; Lu, C.-Z. Tuning Excited State Energy Levels by Achieving Coordination-Induced Thermally Activated Delayed Fluorescence. *Inorg. Chem. Front.* **2022**, *9*, 6561-6566.
(475) Romanov, A. S.; Bochmann, M. Synthesis, Structures and Photoluminescence Properties of Silver Complexes of Cyclic (Alkyl)(Amino)Carbenes. *J. Organomet. Chem.* **2017**, *847*, 114-120.
(476) Chotard, F.; Romanov, A. S.; Hughes, D. L.; Linnolahti, M.; Bochmann, M. Zwitterionic Mixed-Carbene Coinage Metal Complexes: Synthesis, Structures, and Photophysical Studies. *Eur. J. Inorg. Chem.* **2019**, *2019*, 4234-4240.
(477) Chen, J.; Teng, T.; Kang, L.; Chen, X. L.; Wu, X. Y.; Yu, R.; Lu, C. Z. Highly Efficient Thermally Activated Delayed Fluorescence in Dinuclear Ag(I) Complexes with a Bis-Bidentate Tetraphosphane Bridging Ligand. *Inorg. Chem.* **2016**, *55*, 9528-9536.
(478) Shafikov, M. Z.; Suleymanova, A. F.; Schinabeck, A.; Yersin, H. Dinuclear Ag(I) Complex Designed for Highly Efficient Thermally Activated Delayed Fluorescence. *J. Phys. Chem. Lett* **2018**, *9*, 702-709.
(479) Artem'ev, A. V.; Shafikov, M. Z.; Schinabeck, A.; Antonova, O. V.; Berezin, A. S.; Bagryanskaya, I. Y.; Plusnin, P. E.; Yersin, H. Sky-Blue Thermally Activated Delayed Fluorescence (TADF) Based on Ag(I) Complexes: Strong Solvation-Induced Emission Enhancement. *Inorg. Chem. Front.* **2019**, *6*, 3168-3176.
(480) Calvo, M.; Crespo, O.; Gimeno, M. C.; Laguna, A.; Oliván, M. T.; Polo, V.; Rodríguez, D.; Sáez-Rocher, J.-M. Tunable from Blue to Red Emissive Composites and Solids of Silver Diphosphane Systems with Higher Quantum Yields Than the Diphosphane Ligands. *Inorg. Chem.* **2020**, *59*, 14447-14456.
(481) Han, Z.; Dong, X.-Y.; Luo, P.; Li, S.; Wang, Z.-Y.; Zang, S.-Q.; Mak, T. C. W. Ultrastable Atomically Precise Chiral Silver Clusters with More Than 95% Quantum Efficiency. *Sci. Adv.* **2020**, *6*, eaay0107.
(482) Osawa, M.; Aino, M. A.; Nagakura, T.; Hoshino, M.; Tanaka, Y.; Akita, M. Near-Unity Thermally Activated Delayed Fluorescence Efficiency in Three- and Four-Coordinate Au(I) Complexes with Diphosphine Ligands. *Dalton Trans.* **2018**, *47*, 8229-8239.
(483) López-de-Luzuriaga, J. M.; Monge, M.; Olmos, M. E.; Rodríguez-Castillo, M.; Soldevilla, I.; Sundholm, D.; Valiev, R. R. Perhalophenyl Three-Coordinate Gold(I) Complexes as TADF Emitters: A Photophysical Study from Experimental and Computational Viewpoints. *Inorg. Chem.* **2020**, *59*, 14236-14244.
(484) Soldevilla, I.; García-Camacho, A.; Nasibullin, R. T.; Olmos, M. E.; Monge, M.; Sundholm, D.; Valiev, R. R.; López-de-Luzuriaga, J. M.; Rodríguez-Castillo, M. Influence of Perhalophenyl Groups in the TADF Mechanism of Diphosphino Gold(I) Complexes. *J. Mater. Chem. C* **2022**, *10*, 4894-4904.
(485) Li, T. Y.; Muthiah Ravinson, D. S.; Haiges, R.; Djurovich, P. I.; Thompson, M. E. Enhancement of the Luminescent Efficiency in Carbene-Au((I))-Aryl Complexes by the Restriction of Renner-Teller Distortion and Bond Rotation. *J. Am. Chem. Soc.* **2020**, *142*, 6158-6172.
(486) Yu, F. H.; Song, X. F.; Liu, G. H.; Chang, X.; Li, K.; Wang, Y.; Cui, G.; Chen, Y. Highly Efficient Au(I) Alkynyl Emitters: Thermally Activated Delayed Fluorescence and Solution-Processed OLEDs. *Chem. Eur. J.* **2022**, *28*, e202202439.
(487) Cai, S.; Tong, G. S. M.; Du, L.; So, G. K.; Hung, F. F.; Lam, T. L.; Cheng, G.; Xiao, H.; Chang, X.; Xu, Z. X.; Che, C. M. Gold(I) Multi-Resonance Thermally Activated Delayed Fluorescent Emitters for Highly Efficient Ultrapure-Green Organic Light-Emitting Diodes. *Angew. Chem., Int. Ed.* **2022**, *61*, e202213392.
(488) Feng, X.; Yang, J. G.; Miao, J.; Zhong, C.; Yin, X.; Li, N.; Wu, C.; Zhang, Q.; Chen, Y.; Li, K.; Yang, C. Au···H-C Interactions Support a Robust Thermally Activated Delayed Fluorescence (TADF) Gold(I) Complex for OLEDs with Little Efficiency Roll-Off and Good Stability. *Angew. Chem., Int. Ed.* **2022**, *61*, e202209451.

(489) Fernandez-Cestau, J.; Bertrand, B.; Blaya, M.; Jones, G. A.; Penfold, T. J.; Bochmann, M. Synthesis and Luminescence Modulation of Pyrazine-Based Gold(III) Pincer Complexes. *Chem. Commun.* **2015**, *51*, 16629-16632.
(490) To, W.-P.; Zhou, D.; Tong, G. S. M.; Cheng, G.; Yang, C.; Che, C.-M. Highly Luminescent Pincer Gold(III) Aryl Emitters. Thermally Activated Delayed Fluorescence and Solution-Processed OLEDs with EQE and Luminance of up to 23.8 % and 57340 Cd M^{-2}. *Angew. Chem., Int. Ed.* **2017**, *56*, 14036-14041.
(491) Zhou, D.; Cheng, G.; Tong, G. S. M.; Che, C. M. High Efficiency Sky-Blue Gold(III)-TADF Emitters. *Chem. Eur. J.* **2020**, *26*, 15718-15726.
(492) Zhou, D.; To, W.-P.; Kwak, Y.; Cho, Y.; Cheng, G.; Tong, G. S. M.; Che, C.-M. Thermally Stable Donor–Acceptor Type (Alkynyl)Gold(III) TADF Emitters Achieved EQEs and Luminance of up to 23.4% and 70 300 Cd M^{-2} in Vacuum-Deposited OLEDs. *Adv. Sci.* **2019**, *6*, 1802297.
(493) Li, L. K.; Au-Yeung, C. C.; Tang, M. C.; Lai, S. L.; Cheung, W. L.; Ng, M.; Chan, M. Y.; Yam, V. W. Design and Synthesis of Yellow- to Red-Emitting Gold(III) Complexes Containing Isomeric Thienopyridine and Thienoquinoline Moieties and Their Applications in Operationally Stable Organic Light-Emitting Devices. *Mater. Horiz.* **2022**, *9*, 281-293.
(494) Li, L. K.; Kwok, W. K.; Tang, M. C.; Cheung, W. L.; Lai, S. L.; Ng, M.; Chan, M. Y.; Yam, V. W. Highly Efficient CarbazolylGold(III) Dendrimers Based on Thermally Activated Delayed Fluorescence and Their Application in Solution-Processed Organic Light-Emitting Devices. *Chem. Sci.* **2021**, *12*, 14833-14844.
(495) Wong, C. Y.; Tang, M. C.; Li, L. K.; Leung, M. Y.; Tang, W. K.; Lai, S. L.; Cheung, W. L.; Ng, M.; Chan, M. Y.; Yam, V. W. CarbazolylGold(III) Complexes with Thermally Activated Delayed Fluorescence Switched on by Ligand Manipulation as High Efficiency Organic Light-Emitting Devices with Small Efficiency Roll-Offs. *Chem. Sci.* **2022**, *13*, 10129-10140.
(496) Zhou, D.; To, W. P.; Tong, G. S. M.; Cheng, G.; Du, L.; Phillips, D. L.; Che, C. M. Tetradentate Gold(III) Complexes as Thermally Activated Delayed Fluorescence (TADF) Emitters: Microwave-Assisted Synthesis and High-Performance OLEDs with Long Operational Lifetime. *Angew. Chem., Int. Ed.* **2020**, *59*, 6375-6382.
(497) Zhou, D.; Tong, G. S. M.; Cheng, G.; Tang, Y. K.; Liu, W.; Ma, D.; Du, L.; Chen, J. R.; Che, C. M. Stable Tetradentate Gold(III)-TADF Emitters with Close to Unity Quantum Yield and Radiative Decay Rate Constant of up to 2×10^6 S^{-1}: High-Efficiency Green OLEDs with Operational Lifetime (Lt_{90}) Longer Than 1800 H at 1000 Cd M^{-2}. *Adv. Mater.* **2022**, *34*, 2206598.
(498) Romanov, A. S.; Jones, S. T. E.; Yang, L.; Conaghan, P. J.; Di, D.; Linnolahti, M.; Credgington, D.; Bochmann, M. Mononuclear Silver Complexes for Efficient Solution and Vacuum-Processed OLEDs. *Adv. Optical Mater.* **2018**, *6*, 1801347.
(499) Hamze, R.; Peltier, J. L.; Sylvinson, D.; Jung, M.; Cardenas, J.; Haiges, R.; Soleilhavoup, M.; Jazzar, R.; Djurovich, P. I.; Bertrand, G.; Thompson, M. E. Eliminating Nonradiative Decay in Cu(I) Emitters: >99% Quantum Efficiency and Microsecond Lifetime. *Science* **2019**, *363*, 601-606.
(500) Shi, S.; Jung, M. C.; Coburn, C.; Tadle, A.; Sylvinson, M. R. D.; Djurovich, P. I.; Forrest, S. R.; Thompson, M. E. Highly Efficient Photo- and Electroluminescence from Two-Coordinate Cu(I) Complexes Featuring Nonconventional N-Heterocyclic Carbenes. *J. Am. Chem. Soc.* **2019**, *141*, 3576-3588.
(501) Romanov, A. S.; Yang, L.; Jones, S. T. E.; Di, D.; Morley, O. J.; Drummond, B. H.; Reponen, A. P. M.; Linnolahti, M.; Credgington, D.; Bochmann, M. Dendritic Carbene Metal Carbazole Complexes as Photoemitters for Fully Solution-Processed OLEDs. *Chem. Mater.* **2019**, *31*, 3613-3623.
(502) Hamze, R.; Shi, S.; Kapper, S. C.; Muthiah Ravinson, D. S.; Estergreen, L.; Jung, M. C.; Tadle, A. C.; Haiges, R.; Djurovich, P. I.; Peltier, J. L.et al. "Quick-Silver" from a Systematic Study of Highly Luminescent, Two-Coordinate, D^{10} Coinage Metal Complexes. *J. Am. Chem. Soc.* **2019**, *141*, 8616-8626.

(503) Hamze, R.; Idris, M.; Muthiah Ravinson, D. S.; Jung, M. C.; Haiges, R.; Djurovich, P. I.; Thompson, M. E. Highly Efficient Deep Blue Luminescence of 2-Coordinate Coinage Metal Complexes Bearing Bulky NHC Benzimidazolyl Carbene. *Front. Chem.* **2020**, *8*, 401.
(504) Chotard, F.; Sivchik, V.; Linnolahti, M.; Bochmann, M.; Romanov, A. S. Mono- Versus Bicyclic Carbene Metal Amide Photoemitters: Which Design Leads to the Best Performance? *Chem. Mater.* **2020**, *32*, 6114-6122.
(505) Yang, J. G.; Song, X. F.; Wang, J.; Li, K.; Chang, X.; Tan, L. Y.; Liu, C. X.; Yu, F. H.; Cui, G.; Cheng, G.et al. Highly Efficient Thermally Activated Delayed Fluorescence from Pyrazine-Fused Carbene Au(I) Emitters. *Chem. Eur. J.* **2021**, *27*, 17834-17842.
(506) Conaghan, P. J.; Matthews, C. S. B.; Chotard, F.; Jones, S. T. E.; Greenham, N. C.; Bochmann, M.; Credgington, D.; Romanov, A. S. Highly Efficient Blue Organic Light-Emitting Diodes Based on Carbene-Metal-Amides. *Nat. Commun.* **2020**, *11*, 1758.
(507) Romanov, A. S.; Jones, S. T. E.; Gu, Q.; Conaghan, P. J.; Drummond, B. H.; Feng, J.; Chotard, F.; Buizza, L.; Foley, M.; Linnolahti, M.et al. Carbene Metal Amide Photoemitters: Tailoring Conformationally Flexible Amides for Full Color Range Emissions Including White-Emitting OLED. *Chem. Sci.* **2020**, *11*, 435-446.
(508) Gernert, M.; Balles-Wolf, L.; Kerner, F.; Muller, U.; Schmiedel, A.; Holzapfel, M.; Marian, C. M.; Pflaum, J.; Lambert, C.; Steffen, A. Cyclic (Amino)(Aryl)Carbenes Enter the Field of Chromophore Ligands: Expanded Π System Leads to Unusually Deep Red Emitting Cu(I) Compounds. *J. Am. Chem. Soc.* **2020**, *142*, 8897-8909.
(509) Li, T. Y.; Shlian, D. G.; Djurovich, P. I.; Thompson, M. E. A Luminescent Two-Coordinate Au(I) Bimetallic Complex with a Tandem-Carbene Structure: A Molecular Design for the Enhancement of TADF Radiative Decay Rate. *Chem. Eur. J.* **2021**, *27*, 6191-6197.
(510) Reponen, A. P. M.; Chotard, F.; Lempelto, A.; Shekhovtsev, V.; Credgington, D.; Bochmann, M.; Linnolahti, M.; Greenham, N. C.; Romanov, A. S. Donor N-Substitution as Design Principle for Fast and Blue Luminescence in Carbene-Metal-Amides. *Adv. Optical Mater.* **2022**, *10*, 2200312.
(511) Li, T.-y.; Schaab, J.; Djurovich, P. I.; Thompson, M. E. Toward Rational Design of TADF Two-Coordinate Coinage Metal Complexes: Understanding the Relationship between Natural Transition Orbital Overlap and Photophysical Properties. *J. Mater. Chem. C* **2022**, *10*, 4674-4683.
(512) Muniz, C. N.; Schaab, J.; Razgoniaev, A.; Djurovich, P. I.; Thompson, M. E. Π-Extended Ligands in Two-Coordinate Coinage Metal Complexes. *J. Am. Chem. Soc.* **2022**, *144*, 17916-17928.
(513) Tang, R.; Xu, S.; Lam, T. L.; Cheng, G.; Du, L.; Wan, Q.; Yang, J.; Hung, F. F.; Low, K. H.; Phillips, D. L.; Che, C. M. Highly Robust Cu(I) -TADF Emitters for Vacuum-Deposited OLEDs with Luminance up to 222 200 Cd M^{-2} and Device Lifetimes (Lt_{90}) up to 1300 Hours at an Initial Luminance of 1000 Cd M^{-2}. *Angew. Chem., Int. Ed.* **2022**, *61*, e202203982.
(514) Heo, S.; Jung, Y.; Kim, J.; Kim, I.; Bae, H. J.; Son, W. J.; Choi, H.; You, Y. High-Performance Blue Electroluminescence Devices Based on Linear Gold(I) Complexes as Ultrafast Triplet Exciton Harvesters. *Adv. Optical Mater.* **2022**, *10*, 2201610.
(515) Yang, J. G.; Song, X. F.; Cheng, G.; Wu, S.; Feng, X.; Cui, G.; To, W. P.; Chang, X.; Chen, Y.; Che, C. M.et al. Conformational Engineering of Two-Coordinate Gold(I) Complexes: Regulation of Excited-State Dynamics for Efficient Delayed Fluorescence. *ACS Appl. Mater. Interfaces* **2022**, *14*, 13539-13549.
(516) Ying, A.; Ai, Y.; Yang, C.; Gong, S. Aggregation-Dependent Circularly Polarized Luminescence and Thermally Activated Delayed Fluorescence from Chiral Carbene-Cu(I) -Amide Enantiomers. *Angew. Chem., Int. Ed.* **2022**, *61*, e202210490.
(517) Ying, A.; Huang, Y. H.; Lu, C. H.; Chen, Z.; Lee, W. K.; Zeng, X.; Chen, T.; Cao, X.; Wu, C. C.; Gong, S.; Yang, C. High-Efficiency Red Electroluminescence Based on a Carbene-Cu(I)-Acridine Complex. *ACS Appl. Mater. Interfaces* **2021**, *13*, 13478-13486.
(518) Zhu, Z.-Q.; Fleetham, T.; Turner, E.; Li, J. Harvesting All Electrogenerated Excitons through Metal Assisted Delayed Fluorescent Materials. *Adv. Mater.* **2015**, *27*, 2533-2537.

(519) Zhu, Z. Q.; Park, C. D.; Klimes, K.; Li, J. Highly Efficient Blue OLEDs Based on Metal-Assisted Delayed Fluorescence Pd(Ii) Complexes. *Adv. Optical Mater.* **2019**, *7*, 1801518.
(520) Li, G.; Chen, Q.; Zheng, J.; Wang, Q.; Zhan, F.; Lou, W.; Yang, Y. F.; She, Y. Metal-Assisted Delayed Fluorescent Pd(Ii) Complexes and Phosphorescent Pt(Ii) Complex Based on [1,2,4]Triazolo[4,3-*a*]Pyridine-Containing Ligands: Synthesis, Characterization, Electrochemistry, Photophysical Studies, and Application. *Inorg. Chem.* **2019**, *58*, 14349-14360.
(521) She, Y.; Xu, K.; Fang, X.; Yang, Y. F.; Lou, W.; Hu, Y.; Zhang, Q.; Li, G. Tetradentate Platinum(II) and Palladium(II) Complexes Containing Fused 6/6/6 or 6/6/5 Metallocycles with Azacarbazolylcarbazole-Based Ligands. *Inorg. Chem.* **2021**, *60*, 12972-12983.
(522) Zach, P. W.; Freunberger, S. A.; Klimant, I.; Borisov, S. M. Electron-Deficient near-Infrared Pt(Ii) and Pd(Ii) Benzoporphyrins with Dual Phosphorescence and Unusually Efficient Thermally Activated Delayed Fluorescence: First Demonstration of Simultaneous Oxygen and Temperature Sensing with a Single Emitter. *ACS Appl. Mater. Interfaces* **2017**, *9*, 38008-38023.
(523) Abedin-Siddique, Z.; Ohno, T.; Nozaki, K.; Tsubomura, T. Intense Fluorescence of Metal-to-Ligand Charge Transfer in [Pt(0)(Binap)$_2$] [Binap = 2,2‘-Bis(Diphenylphosphino)-1,1‘-Binaphthyl]. *Inorg. Chem.* **2004**, *43*, 663-673.
(524) Chow, P. K.; Ma, C.; To, W. P.; Tong, G. S.; Lai, S. L.; Kui, S. C.; Kwok, W. M.; Che, C. M. Strongly Phosphorescent Palladium(II) Complexes of Tetradentate Ligands with Mixed Oxygen, Carbon, and Nitrogen Donor Atoms: Photophysics, Photochemistry, and Applications. *Angew. Chem., Int. Ed.* **2013**, *52*, 11775-11779.
(525) Pander, P.; Daniels, R.; Zaytsev, A. V.; Horn, A.; Sil, A.; Penfold, T. J.; Williams, J. A. G.; Kozhevnikov, V. N.; Dias, F. B. Exceptionally Fast Radiative Decay of a Dinuclear Platinum Complex through Thermally Activated Delayed Fluorescence. *Chem. Sci.* **2021**, *12*, 6172-6180.
(526) Pander, P.; Zaytsev, A. V.; Sil, A.; Williams, J. A. G.; Lanoe, P.-H.; Kozhevnikov, V. N.; Dias, F. B. The Role of Dinuclearity in Promoting Thermally Activated Delayed Fluorescence (TADF) in Cyclometallated, N^C^N-Coordinated Platinum(II) Complexes. *J. Mater. Chem. C* **2021**, *9*, 10276-10287.
(527) Pander, P.; Zaytsev, A. V.; Sil, A.; Williams, J. A. G.; Kozhevnikov, V. N.; Dias, F. B. Enhancement of Thermally Activated Delayed Fluorescence Properties by Substitution of Ancillary Halogen in a Multiple Resonance-Like DiPlatinum(II) Complex. *J. Mater. Chem. C* **2022**, *10*, 4851-4860.
(528) Sakai, Y.; Sagara, Y.; Nomura, H.; Nakamura, N.; Suzuki, Y.; Miyazaki, H.; Adachi, C. Zinc Complexes Exhibiting Highly Efficient Thermally Activated Delayed Fluorescence and Their Application to Organic Light-Emitting Diodes. *Chem. Commun.* **2015**, *51*, 3181-3184.
(529) Xiong, J.; Li, K.; Teng, T.; Chang, X.; Wei, Y.; Wu, C.; Yang, C. Dinuclear Zn(II) Complexes Exhibiting Thermally Activated Delayed Fluorescence and Luminescence Polymorphism. *Chem. Eur. J.* **2020**, *26*, 6887-6893.
(530) Berezin, A.; Vinogradova, K.; Krivopalov, V.; Nikolaenkova, E.; Plyusnin, V.; Kupryakov, A.; Pervukhina, N.; Naumov, D.; Bushuev, M. B. Excitation-Wavelength-Dependent Emission and Delayed Fluorescence in a Proton Transfer System. *Chem. Eur. J.* **2018**, *24*, 12790-12795.
(531) Chen, Y.; Li, X.; Li, N.; Quan, Y.; Cheng, Y.; Tang, Y. Strong Circularly Polarized Electroluminescence Based on Chiral Salen-Zn(II) Complex Monomer Chromophores. *Mater. Chem. Front.* **2019**, *3*, 867-873.
(532) Steinegger, A.; Borisov, S. M. Zn(II) Schiff Bases: Bright TADF Emitters for Self-Referenced Decay Time-Based Optical Temperature Sensing. *ACS Omega* **2020**, *5*, 7729-7737.
(533) Russegger, A.; Eiber, L.; Steinegger, A.; Borisov, S. M. Zinc Donor–Acceptor Schiff Base Complexes as Thermally Activated Delayed Fluorescence Emitters. *Chemosensors* **2022**, *10*, 91.

(534) Zieger, S. E.; Steinegger, A.; Klimant, I.; Borisov, S. M. TADF-Emitting Zn(II)-Benzoporphyrin: An Indicator for Simultaneous Sensing of Oxygen and Temperature. *ACS Sens.* **2020**, *5*, 1020-1027.
(535) Goswami, B.; Feuerstein, T. J.; Yadav, R.; Lebedkin, S.; Boden, P. J.; Steiger, S. T.; Niedner-Schatteburg, G.; Gerhards, M.; Kappes, M. M.; Roesky, P. W. Thermally Activated Delayed Fluorescence and Phosphorescence Quenching in Iminophosphonamide Copper and Zinc Complexes. *Chem. Eur. J.* **2021**, *27*, 15109-15118.
(536) Endo, A.; Ogasawara, M.; Takahashi, A.; Yokoyama, D.; Kato, Y.; Adachi, C. Thermally Activated Delayed Fluorescence from Sn^{4+}–Porphyrin Complexes and Their Application to Organic Light Emitting Diodes - a Novel Mechanism for Electroluminescence. *Adv. Mater.* **2009**, *21*, 4802-4806.
(537) Gowda, A. S.; Lee, T. S.; Rosko, M. C.; Petersen, J. L.; Castellano, F. N.; Milsmann, C. Long-Lived Photoluminescence of Molecular Group 14 Compounds through Thermally Activated Delayed Fluorescence. *Inorg. Chem.* **2022**, *61*, 7338-7348.
(538) Sattler, W.; Henling, L. M.; Winkler, J. R.; Gray, H. B. Bespoke Photoreductants: Tungsten Arylisocyanides. *J. Am. Chem. Soc.* **2015**, *137*, 1198-1205.
(539) Chan, K. T.; Lam, T. L.; Yu, D.; Du, L.; Phillips, D. L.; Kwong, C. L.; Tong, G. S. M.; Cheng, G.; Che, C. M. Strongly Luminescent Tungsten Emitters with Emission Quantum Yields of up to 84 %: TADF and High-Efficiency Molecular Tungsten OLEDs. *Angew. Chem., Int. Ed.* **2019**, *58*, 14896-14900.
(540) Zhang, Y.; Lee, T. S.; Favale, J. M.; Leary, D. C.; Petersen, J. L.; Scholes, G. D.; Castellano, F. N.; Milsmann, C. Delayed Fluorescence from a Zirconium(IV) Photosensitizer with Ligand-to-Metal Charge-Transfer Excited States. *Nature Chem.* **2020**, *12*, 345-352.
(541) Zhang, Y.; Leary, D. C.; Belldina, A. M.; Petersen, J. L.; Milsmann, C. Effects of Ligand Substitution on the Optical and Electrochemical Properties of (Pyridinedipyrrolide)Zirconium Photosensitizers. *Inorg. Chem.* **2020**, *59*, 14716-14730.
(542) Feuerstein, T. J.; Goswami, B.; Rauthe, P.; Köppe, R.; Lebedkin, S.; Kappes, M. M.; Roesky, P. W. Alkali Metal Complexes of an Enantiopure Iminophosphonamide Ligand with Bright Delayed Fluorescence. *Chem. Sci.* **2019**, *10*, 4742-4749.
(543) Goswami, B.; Feuerstein, T. J.; Yadav, R.; Koppe, R.; Lebedkin, S.; Kappes, M. M.; Roesky, P. W. Enantiopure Calcium Iminophosphonamide Complexes: Synthesis, Photoluminescence, and Catalysis. *Chem. Eur. J.* **2021**, *27*, 4401-4411.
(544) Nakao, K.; Sasabe, H.; Shibuya, Y.; Matsunaga, A.; Katagiri, H.; Kido, J. Novel Series of Mononuclear Aluminum Complexes for High-Performance Solution-Processed Organic Light-Emitting Devices. *Angew. Chem., Int. Ed.* **2021**, *60*, 6036-6041.
(545) Benjamin, H.; Zheng, Y.; Kozhevnikov, V. N.; Siddle, J. S.; O'Driscoll, L. J.; Fox, M. A.; Batsanov, A. S.; Griffiths, G. C.; Dias, F. B.; Monkman, A. P.; Bryce, M. R. Unusual Dual-Emissive Heteroleptic Iridium Complexes Incorporating TADF Cyclometalating Ligands. *Dalton Trans.* **2020**, *49*, 2190-2208.
(546) Thamarappalli, A.; Ranasinghe, C. S. K.; Jang, J.; Gao, M.; Burn, P. L.; Puttock, E. V.; Shaw, P. E. Properties of Dual Emissive Dendrimers Based on Thermally Activated Delayed Fluorescence Dendrons and a Phosphorescent $Ir(Ppy)_3$ Core. *Adv. Funct. Mater.* **2022**, *32*, 2205077.
(547) Jang, J.; Ranasinghe, C. S. K.; Thamarappalli, A.; Gao, M.; Koodalingam, M.; Burn, P. L.; Puttock, E. V.; Shaw, P. E. Understanding the Emission from Dendrimers Composed of Thermally Activated Delayed Fluorescence-Based Dendrons and a Phosphorescent *Fac*-Tris[2-(Thiophen-2-Yl)-4-(Phenyl)Quinoline]Iridium(Iii) Core. *J. Mater. Chem. C* **2022**, *10*, 17245-17257.
(548) Di, D.; Romanov, A. S.; Yang, L.; Richter, J. M.; Rivett, J. P. H.; Jones, S.; Thomas, T. H.; Abdi Jalebi, M.; Friend, R. H.; Linnolahti, M.et al. High-Performance Light-Emitting Diodes Based on Carbene-Metal-Amides. *Science* **2017**, *356*, 159-163.
(549) Xie, G.; Luo, J.; Huang, M.; Chen, T.; Wu, K.; Gong, S.; Yang, C. Inheriting the Characteristics of TADF Small Molecule by Side-Chain Engineering Strategy to Enable Bluish-Green Polymers with High PLQYs up to 74% and External Quantum Efficiency over 16% in Light-Emitting Diodes. *Adv. Mater.* **2017**, *29*, 1604223.

(550) Wang, T.; Zou, Y.; Huang, Z.; Li, N.; Miao, J.; Yang, C. Narrowband Emissive TADF Conjugated Polymers Towards Highly Efficient Solution-Processible OLEDs. *Angew. Chem., Int. Ed.* **2022**, *61*, e202211172-e202211172.

(551) Zong, W.; Qiu, W.; Yuan, P.; Wang, F.; Liu, Y.; Xu, S.; Su, S. J.; Cao, S. Thermally Activated Delayed Fluorescence Polymers for High-Efficiency Solution-Processed Non-Doped OLEDs: Convenient Synthesis by Binding TADF Units and Host Units to the Pre-Synthesized Polycarbazole-Based Backbone Via Click Reaction. *Polymer* **2022**, *240*, 124468.

(552) Yang, Y.; Zhao, L.; Wang, S.; Ding, J.; Wang, L. Red-Emitting Thermally Activated Delayed Fluorescence Polymers with Poly(Fluorene-Co-3,3′-Dimethyl Diphenyl Ether) as the Backbone. *Macromolecules* **2018**, *51*, 9933-9942.

(553) Ban, X.; Zhou, T.; Cao, Q.; Zhang, K.; Tong, Z.; Xu, H.; Zhu, A.; Jiang, W. Combining Molecular Encapsulation and an AIE Strategy to Construct an Efficient Blue TADF Polymer for Solution-Processed Multilayer White OLEDs. *J. Mater. Chem. C* **2022**, *10*, 15114-15125.

(554) Li, C.; Wang, Y.; Sun, D.; Li, H.; Sun, X.; Ma, D.; Ren, Z.; Yan, S. Thermally Activated Delayed Fluorescence Pendant Copolymers with Electron- and Hole-Transporting Spacers. *ACS Appl. Mater. Interfaces* **2018**, *10*, 5731-5739.

(555) Li, C.; Nobuyasu, R. S.; Wang, Y.; Dias, F. B.; Ren, Z.; Bryce, M. R.; Yan, S. Solution-Processable Thermally Activated Delayed Fluorescence White OLEDs Based on Dual-Emission Polymers with Tunable Emission Colors and Aggregation-Enhanced Emission Properties. *Adv. Optical Mater.* **2017**, *5*, 1700435.

(556) Li, C.; Ren, Z.; Sun, X.; Li, H.; Yan, S. Deep-Blue Thermally Activated Delayed Fluorescence Polymers for Nondoped Solution-Processed Organic Light-Emitting Diodes. *Macromolecules* **2019**, *52*, 2296-2303.

(557) Zeng, X.; Luo, J.; Zhou, T.; Chen, T.; Zhou, X.; Wu, K.; Zou, Y.; Xie, G.; Gong, S.; Yang, C. Using Ring-Opening Metathesis Polymerization of Norbornene to Construct Thermally Activated Delayed Fluorescence Polymers: High-Efficiency Blue Polymer Light-Emitting Diodes. *Macromolecules* **2018**, *51*, 1598-1604.

(558) Cole, C. M.; Kunz, S. V.; Shaw, P. E.; Sampath, C.; Ranasinghe, K.; Baumann, T.; Blinco, J. P.; Sonar, P.; Barner-Kowollik, C.; Yambem, S. D.et al. Inkjet-Printed Self-Hosted TADF Polymer Light-Emitting Diodes. *Adv. Mater. Tech.* **2022**, *7*, 2200648-2200648.

(559) Zhu, Y.; Zhang, Y.; Yao, B.; Wang, Y.; Zhang, Z.; Zhan, H.; Zhang, B.; Xie, Z.; Wang, Y.; Cheng, Y. Synthesis and Electroluminescence of a Conjugated Polymer with Thermally Activated Delayed Fluorescence. *Macromolecules* **2016**, *49*, 4373-4377.

(560) Yang, Y.; Wang, S.; Zhu, Y.; Wang, Y.; Zhan, H.; Cheng, Y. Thermally Activated Delayed Fluorescence Conjugated Polymers with Backbone-Donor/Pendant-Acceptor Architecture for Nondoped OLEDs with High External Quantum Efficiency and Low Roll-Off. *Adv. Funct. Mater.* **2018**, *28*, 1706916.

(561) Liu, Y.; Wang, Y.; Li, C.; Ren, Z.; Ma, D.; Yan, S. Efficient Thermally Activated Delayed Fluorescence Conjugated Polymeric Emitters with Tunable Nature of Excited States Regulated Via Carbazole Derivatives for Solution-Processed OLEDs. *Macromolecules* **2018**, *51*, 4615-4623.

(562) Zhao, Z.; Liu, Y.; Hua, L.; Yan, S.; Ren, Z.; Zhao, Z.; Hua, L.; Yan, S.; Ren, Z.; Liu, Y. Activating Energy Transfer Tunnels by Tuning Local Electronegativity of Conjugated Polymeric Backbone for High-Efficiency OLEDs with Low Efficiency Roll-Off. *Adv. Funct. Mater.* **2022**, *32*, 2200018-2200018.

(563) Wei, Q.; Kleine, P.; Karpov, Y.; Qiu, X.; Komber, H.; Sahre, K.; Kiriy, A.; Lygaitis, R.; Lenk, S.; Reineke, S.; Voit, B. Conjugation-Induced Thermally Activated Delayed Fluorescence (TADF): From Conventional Non-TADF Units to TADF-Active Polymers. *Adv. Funct. Mater.* **2017**, *27*, 1605051.

(564) Zhang, J.; Wei, Q.; Lyu, L.; Cao, L.; Zhao, M.; Fei, N.; Wang, T.; Ge, Z. Thermally Activated Delayed Fluorescent (TADF) Mono-Polymeric OLED with Higher EQE over Its TADF Repeating Unit. *Macromol. Chem. Phys.* **2022**, *223*, 2200023-2200023.

(565) Wang, Y.; Zhu, Y.; Xie, G.; Zhan, H.; Yang, C.; Cheng, Y. Bright White Electroluminescence from a Single Polymer Containing a Thermally Activated Delayed

Fluorescence Unit and a Solution-Processed Orange OLED Approaching 20% External Quantum Efficiency. *J. Mater. Chem. C* **2017**, *5*, 10715-10720.
(566) Hu, Y.; Song, F.; Xu, Z.; Tu, Y.; Zhang, H.; Cheng, Q.; Lam, J. W. Y.; Ma, D.; Tang, B. Z. Circularly Polarized Luminescence from Chiral Conjugated Poly(carbazole-ran-acridine)s with Aggregation-Induced Emission and Delayed Fluorescence. *ACS Appl. Polym. Mater.* **2019**, *1*, 221-229.
(567) Teng, J. M.; Zhang, D. W.; Wang, Y. F.; Chen, C. F. Chiral Conjugated Thermally Activated Delayed Fluorescent Polymers for Highly Efficient Circularly Polarized Polymer Light-Emitting Diodes. *ACS Appl. Mater. Interfaces* **2022**, *14*, 1578-1586.
(568) Freeman, D. M. E.; Musser, A. J.; Frost, J. M.; Stern, H. L.; Forster, A. K.; Fallon, K. J.; Rapidis, A. G.; Cacialli, F.; McCulloch, I.; Clarke, T. M.et al. Synthesis and Exciton Dynamics of Donor-Orthogonal Acceptor Conjugated Polymers: Reducing the Singlet–Triplet Energy Gap. *J. Am. Chem. Soc.* **2017**, *139*, 11073-11080.
(569) Nikolaenko, A. E.; Cass, M.; Bourcet, F.; Mohamad, D.; Roberts, M. Thermally Activated Delayed Fluorescence in Polymers: A New Route toward Highly Efficient Solution Processable OLEDs. *Adv. Mater.* **2015**, *27*, 7236-7240.
(570) Philipps, K.; Ie, Y.; van der Zee, B.; Png, R. Q.; Ho, P. K. H.; Chua, L. L.; del Pino Rosendo, E.; Ramanan, C.; Wetzelaer, G. J. A. H.; Blom, P. W. M.; Michels, J. J. Role of Linker Functionality in Polymers Exhibiting Main-Chain Thermally Activated Delayed Fluorescence. *Adv. Sci.* **2022**, *9*, 2200056-2200056.
(571) Sun, K.; Wu, J.; Zhu, L.; Liu, H.; Zhou, Y.; Tian, W.; Cai, Z.; Jiang, W.; Sun, Y. Highly Efficient Blue All-Solution-Processed Organic Light-Emitting Diodes Based on the Strategy of Constructing a Thermally Cross-Linkable TADF Dendrimer. *Dyes Pigm.* **2022**, *198*, 109967-109967.
(572) Sun, K.; Tian, W.; Gao, H.; Bi, C.; Yao, J.; Wang, Z.; Cai, Z.; Jiang, W. Creation of a Thermally Cross-Linkable Encapsulated TADF Molecule for Highly Efficient Solution-Processed Hybrid White OLEDs. *Org. Electron.* **2022**, *102*, 106442-106442.
(573) Sun, K.; Tian, W.; Ge, C.; Gu, F.; Zhou, Y.; Wang, W.; Cai, Z.; Jiang, W.; Sun, Y. Creation of Efficient Solution-Processed OLEDs Via a Strategy of the Host-Guest System Constructing with Two Small Cross-Linkable TADF Molecules. *Org. Electron.* **2022**, *101*, 106417.
(574) Shao, S.; Hu, J.; Wang, X.; Wang, L.; Jing, X.; Wang, F. Blue Thermally Activated Delayed Fluorescence Polymers with Nonconjugated Backbone and through-Space Charge Transfer Effect. *J. Am. Chem. Soc.* **2017**, *139*, 17739-17742.
(575) Hu, J.; Chang, Y.; Chen, F.; Yang, Q.; Shao, S.; Wang, L. Design, Synthesis, and Properties of Polystyrene-Based through-Space Charge Transfer Polymers: Effect of Triplet Energy Level of Electron Donor Moiety on Delayed Fluorescence and Electroluminescence Performance. *J. Polym. Sci.* **2022**, *60*, 1855-1863.
(576) Zhou, T.; Zhang, K.; Cao, Q.; Xu, H.; Ban, X.; Zhu, P.; Li, Q.; Shi, L.; Ge, F.; Jiang, W. Benzonitrile-Based AIE Polymer Host with a Simple Synthesis Process for High-Efficiency Solution-Processable Green and Blue TADF Organic Light Emitting Diodes. *J. Mater. Chem. C* **2022**, *10*, 2109-2120.
(577) Albrecht, K.; Matsuoka, K.; Fujita, K.; Yamamoto, K. Carbazole Dendrimers as Solution-Processable Thermally Activated Delayed-Fluorescence Materials. *Angew. Chem., Int. Ed.* **2015**, *54*, 5677-5682.
(578) Albrecht, K.; Matsuoka, K.; Yokoyama, D.; Sakai, Y.; Nakayama, A.; Fujita, K.; Yamamoto, K. Thermally Activated Delayed Fluorescence OLEDs with Fully Solution Processed Organic Layers Exhibiting Nearly 10% External Quantum Efficiency. *Chem. Commun.* **2017**, *53*, 2439-2442.
(579) Matsuoka, K.; Albrecht, K.; Nakayama, A.; Yamamoto, K.; Fujita, K. Highly Efficient Thermally Activated Delayed Fluorescence Organic Light-Emitting Diodes with Fully Solution-Processed Organic Multilayered Architecture: Impact of Terminal Substitution on Carbazole–Benzophenone Dendrimer and Interfacial Engineering. *ACS Appl. Mater. Interfaces* **2018**, *10*, 33343-33352.

(580) Huang, B.; Ban, X.; Sun, K.; Ma, Z.; Mei, Y.; Jiang, W.; Lin, B.; Sun, Y. Thermally Activated Delayed Fluorescence Materials Based on Benzophenone Derivative as Emitter for Efficient Solution-Processed Non-Doped Green OLED. *Dyes Pigm.* **2016**, *133*, 380-386.
(581) Matsuoka, K.; Albrecht, K.; Yamamoto, K.; Fujita, K. Mulifunctional Dendritic Emitter: Aggregation-Induced Emission Enhanced, Thermally Activated Delayed Fluorescent Material for Solution-Processed Multilayered Organic Light-Emitting Diodes. *Sci. Rep.* **2017**, *7*, 41780.
(582) Sun, D.; Duda, E.; Fan, X.; Saxena, R.; Zhang, M.; Bagnich, S.; Zhang, X.; Köhler, A.; Zysman-Colman, E. Thermally Activated Delayed Fluorescent Dendrimers That Underpin High-Efficiency Host-Free Solution-Processed Organic Light-Emitting Diodes. *Adv. Mater.* **2022**, *34*, 2110344-2110344.
(583) Sun, D.; Saxena, R.; Fan, X.; Athanasopoulos, S.; Duda, E.; Zhang, M.; Bagnich, S.; Zhang, X.; Zysman-Colman, E.; Köhler, A. Regiochemistry of Donor Dendrons Controls the Performance of Thermally Activated Delayed Fluorescence Dendrimer Emitters for High Efficiency Solution-Processed Organic Light-Emitting Diodes. *Adv. Sci.* **2022**, *9*, 2201470.
(584) Zhang, C.; Yan, H.; He, Y.; Chai, Y.; Zhou, D. Thermally Activated Delayed Fluorescence Dendrimers Achieving 20% External Quantum Efficiency for Solution-Processed OLEDs. *Mater. Chem. Front.* **2022**, *6*, 3442-3449.
(585) He, Y.; Zhou, D.; Zhang, C.; Yan, H.; Chai, Y. Orange-Red and Saturated Red Thermally Activated Delayed Fluorescent Dendrimers for Non-Doped Solution-Processed OLEDs. *Dyes Pigm.* **2022**, *203*, 110385-110385.
(586) Li, Y.; Xie, G.; Gong, S.; Wu, K.; Yang, C. Dendronized Delayed Fluorescence Emitters for Non-Doped, Solution-Processed Organic Light-Emitting Diodes with High Efficiency and Low Efficiency Roll-Off Simultaneously: Two Parallel Emissive Channels. *Chem. Sci.* **2016**, *7*, 5441-5447.
(587) Luo, J.; Gong, S.; Gu, Y.; Chen, T.; Li, Y.; Zhong, C.; Xie, G.; Yang, C. Multi-Carbazole Encapsulation as a Simple Strategy for the Construction of Solution-Processed, Non-Doped Thermally Activated Delayed Fluorescence Emitters. *J. Mater. Chem. C* **2016**, *4*, 2442-2446.
(588) Li, C.; Harrison, A. K.; Liu, Y.; Zhao, Z.; Zeng, C.; Dias, F. B.; Ren, Z.; Yan, S.; Bryce, M. R. Asymmetrical-Dendronized TADF Emitters for Efficient Non-Doped Solution-Processed OLEDs by Eliminating Degenerate Excited States and Creating Solely Thermal Equilibrium Routes. *Angew. Chem., Int. Ed.* **2022**, *61*, e202115140.
(589) Huang, M.; Li, Y.; Wu, K.; Luo, J.; Xie, G.; Li, L.; Yang, C. Carbazole-Dendronized Thermally Activated Delayed Fluorescent Molecules with Small Singlet-Triplet Gaps for Solution-Processed Organic Light-Emitting Diodes. *Dyes Pigm.* **2018**, *153*, 92-98.
(590) Li, Y.; Chen, T.; Huang, M.; Gu, Y.; Gong, S.; Xie, G.; Yang, C. Tuning the Twist Angle of Thermally Activated Delayed Fluorescence Molecules Via a Dendronization Strategy: High-Efficiency Solution-Processed Non-Doped OLEDs. *J. Mater. Chem. C* **2017**, *5*, 3480-3487.
(591) Wang, J.; Peng, J.; Yao, W.; Jiang, C.; Liu, C.; Zhang, C.; He, M.; Liu, R.; Xia, X.; Yao, C. Carbazole-Dendrite-Encapsulated Electron Acceptor Core for Constructing Thermally Activated Delayed Fluorescence Emitters Used in Nondoped Solution-Processed Organic Light-Emitting Diodes. *Org. Electron.* **2017**, *48*, 262-270.
(592) Puttock, E. V.; Ranasinghe, C. S. K.; Babazadeh, M.; Kistemaker, J. C. M.; Jang, J.; Gao, M.; Huang, D. M.; Adachi, C.; Burn, P. L.; Shaw, P. E. Thermally Activated Delayed Fluorescence Poly(Dendrimer)S - Detrapping Excitons for Reverse Intersystem Crossing. *J. Mater. Chem. C* **2022**, *10*, 8109-8124.
(593) Wang, X.; Hu, J.; Lv, J.; Yang, Q.; Tian, H.; Shao, S.; Wang, L.; Jing, X.; Wang, F. Π-Stacked Donor–Acceptor Dendrimers for Highly Efficient White Electroluminescence. *Angew. Chem., Int. Ed.* **2021**, *60*, 16585-16593.
(594) Ban, X.; Zhu, A.; Zhang, T.; Tong, Z.; Jiang, W.; Sun, Y. Highly Efficient All-Solution-Processed Fluorescent Organic Light-Emitting Diodes Based on a Novel Self-Host Thermally Activated Delayed Fluorescence Emitter. *ACS Appl. Mater. Interfaces* **2017**, *9*, 21900-21908.
(595) Ban, X.; Chen, F.; Liu, Y.; Pan, J.; Zhu, A.; Jiang, W.; Sun, Y. Design of Efficient Thermally Activated Delayed Fluorescence Blue Host for High Performance Solution-Processed Hybrid White Organic Light Emitting Diodes. *Chem. Sci.* **2019**, *10*, 3054-3064.

(596) Sun, K.; Sun, Y.; Huang, T.; Luo, J.; Jiang, W.; Sun, Y. Design Strategy of Yellow Thermally Activated Delayed Fluorescent Dendrimers and Their Highly Efficient Non-Doped Solution-Processed OLEDs with Low Driving Voltage. *Org. Electron.* **2017**, *42*, 123-130.

(597) Godumala, M.; Choi, S.; Kim, H. J.; Lee, C.; Park, S.; Moon, J. S.; Si Woo, K.; Kwon, J. H.; Cho, M. J.; Choi, D. H. Novel Dendritic Large Molecules as Solution-Processable Thermally Activated Delayed Fluorescent Emitters for Simple Structured Non-Doped Organic Light Emitting Diodes. *J. Mater. Chem. C* **2018**, *6*, 1160-1170.

(598) Ban, X.; Jiang, W.; Sun, K.; Lin, B.; Sun, Y. Self-Host Blue Dendrimer Comprised of Thermally Activated Delayed Fluorescence Core and Bipolar Dendrons for Efficient Solution-Processible Nondoped Electroluminescence. *ACS Appl. Mater. Interfaces* **2017**, *9*, 7339-7346.

(599) Ban, X.; Lin, B.; Jiang, W.; Sun, Y. Constructing a Novel Dendron for a Self-Host Blue Emitter with Thermally Activated Delayed Fluorescence: Solution-Processed Nondoped Organic Light-Emitting Diodes with Bipolar Charge Transfer and Stable Color Purity. *Chem. Asian J.* **2017**, *12*, 216-223.

(600) Sun, K.; Sun, Y.; Liu, D.; Feng, Y.; Zhang, X.; Sun, Y.; Jiang, W. Cbp Derivatives Dendronized Self-Host TADF Dendrimer: Achieving Efficient Non-Doped near-Infrared Organic Light-Emitting Diodes. *Dyes Pigm.* **2017**, *147*, 436-443.

(601) Sun, K.; Chu, D.; Cui, Y.; Tian, W.; Sun, Y.; Jiang, W. Near-Infrared Thermally Activated Delayed Fluorescent Dendrimers for the Efficient Non-Doped Solution-Processed Organic Light-Emitting Diodes. *Org. Electron.* **2017**, *48*, 389-396.

(602) Ikeda, N.; Oda, S.; Matsumoto, R.; Yoshioka, M.; Fukushima, D.; Yoshiura, K.; Yasuda, N.; Hatakeyama, T. Solution-Processable Pure Green Thermally Activated Delayed Fluorescence Emitter Based on the Multiple Resonance Effect. *Adv. Mater.* **2020**, *32*, e2004072.

(603) Hirai, H.; Nakajima, K.; Nakatsuka, S.; Shiren, K.; Ni, J.; Nomura, S.; Ikuta, T.; Hatakeyama, T. One-Step Borylation of 1,3-Diaryloxybenzenes Towards Efficient Materials for Organic Light-Emitting Diodes. *Angew. Chem., Int. Ed.* **2015**, *54*, 13581-13585.

(604) Chen, F.; Zhao, L.; Wang, X.; Yang, Q.; Li, W.; Tian, H.; Shao, S.; Wang, L.; Jing, X.; Wang, F. Novel Boron- and Sulfur-Doped Polycyclic Aromatic Hydrocarbon as Multiple Resonance Emitter for Ultrapure Blue Thermally Activated Delayed Fluorescence Polymers. *Sci. China Chem.* **2021**, *64*, 547-551.

(605) Hatakeyama, T.; Shiren, K.; Nakajima, K.; Nomura, S.; Nakatsuka, S.; Kinoshita, K.; Ni, J.; Ono, Y.; Ikuta, T. Ultrapure Blue Thermally Activated Delayed Fluorescence Molecules: Efficient HOMO–LUMO Separation by the Multiple Resonance Effect. *Adv. Mater.* **2016**, *28*, 2777-2871.

(606) Nakatsuka, S.; Gotoh, H.; Kinoshita, K.; Yasuda, N.; Hatakeyama, T. Divergent Synthesis of Heteroatom-Centered 4,8,12-Triazatriangulenes. *Angew. Chem., Int. Ed.* **2017**, *56*, 5087-5090.

(607) Han, S. H.; Jeong, J. H.; Yoo, J. W.; Lee, J. Y. Ideal Blue Thermally Activated Delayed Fluorescence Emission Assisted by a Thermally Activated Delayed Fluorescence Assistant Dopant through a Fast Reverse Intersystem Crossing Mediated Cascade Energy Transfer Process. *J. Mater. Chem. C* **2019**, *7*, 3082-3089.

(608) Lim, H.; Woo, S. J.; Ha, Y. H.; Kim, Y. H.; Kim, J. J. Breaking the Efficiency Limit of Deep-Blue Fluorescent OLEDs Based on Anthracene Derivatives. *Adv. Mater.* **2022**, *34*, 2100161.

(609) Oda, S.; Kumano, W.; Hama, T.; Kawasumi, R.; Yoshiura, K.; Hatakeyama, T. Carbazole-Based DABNA Analogues as Highly Efficient Thermally Activated Delayed Fluorescence Materials for Narrowband Organic Light-Emitting Diodes. *Angew. Chem., Int. Ed.* **2021**, *60*, 2882-2886.

(610) Kim, J. H.; Chung, W. J.; Kim, J.; Lee, J. Y. Concentration Quenching–Resistant Multiresonance Thermally Activated Delayed Fluorescence Emitters. *Mater. Today Energy* **2021**, *21*, 100792.

(611) Wang, Y.; Duan, Y.; Guo, R.; Ye, S.; Di, K.; Zhang, W.; Zhuang, S.; Wang, L. A Periphery Cladding Strategy to Improve the Performance of Narrowband Emitters, Achieving Deep-Blue OLEDs with Ciey < 0.08 and External Quantum Efficiency Approaching 20%. *Org. Electron.* **2021**, *97*, 106275.

(612) Lee, Y.; Hong, J.-I. Multiple Resonance Thermally Activated Delayed Fluorescence Enhanced by Halogen Atoms. *J. Mater. Chem. C* **2022**, *10*, 11855-11861.
(613) Park, J.; Kim, K. J.; Lim, J.; Kim, T.; Lee, J. Y. High Efficiency of over 25% and Long Device Lifetime of over 500 H at 1000 Nit in Blue Fluorescent Organic Light-Emitting Diodes. *Adv. Mater.* **2022**, *34*, 2108581.
(614) Wang, Y.; Di, K.; Duan, Y.; Guo, R.; Lian, L.; Zhang, W.; Wang, L. The Selective Regulation of Borylation Site Based on One-Shot Electrophilic C–H Borylation Reaction, Achieving Highly Efficient Narrowband Organic Light-Emitting Diodes. *Chem. Eng. J.* **2022**, *431*, 133221.
(615) Cheon, H. J.; Woo, S. J.; Baek, S. H.; Lee, J. H.; Kim, Y. H. Dense Local Triplet States and Steric Shielding of a Multi-Resonance TADF Emitter Enable High-Performance Deep-Blue OLEDs. *Adv. Mater.* **2022**, *34*, 2207416.
(616) Cheon, H. J.; Shin, Y. S.; Park, N. H.; Lee, J. H.; Kim, Y. H. Boron-Based Multi-Resonance TADF Emitter with Suppressed Intermolecular Interaction and Isomer Formation for Efficient Pure Blue OLEDs. *Small* **2022**, *18*, 2107574.
(617) Kim, E.; Park, J.; Jun, M.; Shin, H.; Baek, J.; Kim, T.; Kim, S.; Lee, J.; Ahn, H.; Sun, J.et al. Highly Efficient and Stable Deep-Blue Organic Light-Emitting Diode Using Phosphor-Sensitized Thermally Activated Delayed Fluorescence. *Sci. Adv.* **2022**, *8*, eabq1641.
(618) Matsui, K.; Oda, S.; Yoshiura, K.; Nakajima, K.; Yasuda, N.; Hatakeyama, T. One-Shot Multiple Borylation toward Bn-Doped Nanographenes. *J. Am. Chem. Soc.* **2018**, *140*, 1195-1198.
(619) Kondo, Y.; Yoshiura, K.; Kitera, S.; Nishi, H.; Oda, S.; Gotoh, H.; Sasada, Y.; Yanai, M.; Hatakeyama, T. Narrowband Deep-Blue Organic Light-Emitting Diode Featuring an Organoboron-Based Emitter. *Nat. Photonics* **2019**, *13*, 678-682.
(620) Rayappa Naveen, K.; Lee, H.; Braveenth, R.; Joon Yang, K.; Jae Hwang, S.; Hyuk Kwon, J. Deep Blue Diboron Embedded Multi-Resonance Thermally Activated Delayed Fluorescence Emitters for Narrowband Organic Light Emitting Diodes. *Chem. Eng. J.* **2022**, *432*, 134381.
(621) Oda, S.; Sugitani, T.; Tanaka, H.; Tabata, K.; Kawasumi, R.; Hatakeyama, T. Development of Pure Green Thermally Activated Delayed Fluorescence Material by Cyano Substitution. *Adv. Mater.* **2022**, *34*, 2201778.
(622) Oda, S.; Kawakami, B.; Yamasaki, Y.; Matsumoto, R.; Yoshioka, M.; Fukushima, D.; Nakatsuka, S.; Hatakeyama, T. One-Shot Synthesis of Expanded Heterohelicene Exhibiting Narrowband Thermally Activated Delayed Fluorescence. *J. Am. Chem. Soc.* **2022**, *144*, 106-112.
(623) Tanaka, H.; Oda, S.; Ricci, G.; Gotoh, H.; Tabata, K.; Kawasumi, R.; Beljonne, D.; Olivier, Y.; Hatakeyama, T. Hypsochromic Shift of Multiple-Resonance-Induced Thermally Activated Delayed Fluorescence by Oxygen Atom Incorporation. *Angew. Chem., Int. Ed.* **2021**, *60*, 17910-17914.
(624) Park, I. S.; Yang, M.; Shibata, H.; Amanokura, N.; Yasuda, T. Achieving Ultimate Narrowband and Ultrapure Blue Organic Light-Emitting Diodes Based on Polycyclo-Heteraborin Multi-Resonance Delayed Fluorescence Emitters. *Adv. Mater.* **2022**, *34*, 2107951.
(625) Suresh, S. M.; Duda, E.; Hall, D.; Yao, Z.; Bagnich, S.; Slawin, A. M. Z.; Bassler, H.; Beljonne, D.; Buck, M.; Olivier, Y.et al. A Deep Blue B,N-Doped Heptacene Emitter That Shows Both Thermally Activated Delayed Fluorescence and Delayed Fluorescence by Triplet-Triplet Annihilation. *J. Am. Chem. Soc.* **2020**, *142*, 6588-6599.
(626) Stavrou, K.; Madayanad Suresh, S.; Hall, D.; Danos, A.; Kukhta, N. A.; Slawin, A. M. Z.; Warriner, S.; Beljonne, D.; Olivier, Y.; Monkman, A.; Zysman-Colman, E. Emission and Absorption Tuning in TADF B,N-Doped Heptacenes: Toward Ideal-Blue Hyperfluorescent OLEDs. *Adv. Optical Mater.* **2022**, *10*, 2200688.
(627) Xu, Y.; Cheng, Z.; Li, Z.; Liang, B.; Wang, J.; Wei, J.; Zhang, Z.; Wang, Y. Molecular-Structure and Device-Configuration Optimizations toward Highly Efficient Green Electroluminescence with Narrowband Emission and High Color Purity. *Adv. Optical Mater.* **2020**, *8*, 1902142.

(628) Yang, M.; Park, I. S.; Yasuda, T. Full-Color, Narrowband, and High-Efficiency Electroluminescence from Boron and Carbazole Embedded Polycyclic Heteroaromatics. *J. Am. Chem. Soc.* **2020**, *142*, 19468-19472.
(629) Lee, Y. T.; Chan, C. Y.; Tanaka, M.; Mamada, M.; Balijapalli, U.; Tsuchiya, Y.; Nakanotani, H.; Hatakeyama, T.; Adachi, C. Investigating Homo Energy Levels of Terminal Emitters for Realizing High-Brightness and Stable TADF-Assisted Fluorescence Organic Light-Emitting Diodes. *Adv. Electron. Mater.* **2021**, *7*, 2001090.
(630) Yang, M.; Shikita, S.; Min, H.; Park, I. S.; Shibata, H.; Amanokura, N.; Yasuda, T. Wide-Range Color Tuning of Narrowband Emission in Multi-Resonance Organoboron Delayed Fluorescence Materials through Rational Imine/Amine Functionalization. *Angew. Chem., Int. Ed.* **2021**, *60*, 23142-23147.
(631) Zhang, Y.; Zhang, D.; Wei, J.; Hong, X.; Lu, Y.; Hu, D.; Li, G.; Liu, Z.; Chen, Y.; Duan, L. Achieving Pure Green Electroluminescence with Ciey of 0.69 and EQE of 28.2% from an Aza-Fused Multi-Resonance Emitter. *Angew. Chem., Int. Ed.* **2020**, *59*, 17499-17503.
(632) Xu, Y.; Wang, Q.; Wei, J.; Peng, X.; Xue, J.; Wang, Z.; Su, S. J.; Wang, Y. Constructing Organic Electroluminescent Material with Very High Color Purity and Efficiency Based on Polycyclization of the Multiple Resonance Parent Core. *Angew. Chem., Int. Ed.* **2022**, *61*, e202204652.
(633) Zhang, Y.; Zhang, D.; Wei, J.; Liu, Z.; Lu, Y.; Duan, L. Multi-Resonance Induced Thermally Activated Delayed Fluorophores for Narrowband Green OLEDs. *Angew. Chem., Int. Ed.* **2019**, *58*, 16912-16917.
(634) Liu, Y.; Xiao, X.; Ran, Y.; Bin, Z.; You, J. Molecular Design of Thermally Activated Delayed Fluorescent Emitters for Narrowband Orange–Red OLEDs Boosted by a Cyano-Functionalization Strategy. *Chem. Sci.* **2021**, *12*, 9408-9412.
(635) Xu, Y.; Li, C.; Li, Z.; Wang, J.; Xue, J.; Wang, Q.; Cai, X.; Wang, Y. Highly Efficient Electroluminescent Materials with High Color Purity Based on Strong Acceptor Attachment onto B–N-Containing Multiple Resonance Frameworks. *CCS Chem.* **2022**, *4*, 2065-2079.
(636) Xu, Y.; Li, C.; Li, Z.; Wang, Q.; Cai, X.; Wei, J.; Wang, Y. Constructing Charge-Transfer Excited States Based on Frontier Molecular Orbital Engineering: Narrowband Green Electroluminescence with High Color Purity and Efficiency. *Angew. Chem., Int. Ed.* **2020**, *59*, 17442-17446.
(637) Yan, X.; Li, Z.; Wang, Q.; Qu, Y.; Xu, Y.; Wang, Y. Achieving Highly Efficient Narrowband Sky-Blue Electroluminescence with Alleviated Efficiency Roll-Off by Molecular-Structure Regulation and Device-Configuration Optimization. *J. Mater. Chem. C* **2022**, *10*, 15408-15415.
(638) Qi, Y.; Ning, W.; Zou, Y.; Cao, X.; Gong, S.; Yang, C. Peripheral Decoration of Multi-Resonance Molecules as a Versatile Approach for Simultaneous Long-Wavelength and Narrowband Emission. *Adv. Funct. Mater.* **2021**, *31*, 2102017.
(639) Cai, X.; Xu, Y.; Wang, Q.; Li, C.; Wang, Y. Constructing Narrowband Thermally Activated Delayed Fluorescence Materials with Emission Maxima Beyond 560 Nm Based on Frontier Molecular Orbital Engineering. *Angew. Chem., Int. Ed.* **2023**, *62,* e202312451.
(640) Liu, Y.; Xiao, X.; Huang, Z.; Yang, D.; Ma, D.; Liu, J.; Lei, B.; Bin, Z.; You, J. Space-Confined Donor-Acceptor Strategy Enables Fast Spin-Flip of Multiple Resonance Emitters for Suppressing Efficiency Roll-Off. *Angew. Chem., Int. Ed.* **2022**, *61*, e202210210.
(641) Jiang, P.; Miao, J.; Cao, X.; Xia, H.; Pan, K.; Hua, T.; Lv, X.; Huang, Z.; Zou, Y.; Yang, C. Quenching-Resistant Multiresonance TADF Emitter Realizes 40% External Quantum Efficiency in Narrowband Electroluminescence at High Doping Level. *Adv. Mater.* **2022**, *34*, 2106954.
(642) Zhang, Y.; Wei, J.; Zhang, D.; Yin, C.; Li, G.; Liu, Z.; Jia, X.; Qiao, J.; Duan, L. Sterically Wrapped Multiple Resonance Fluorophors for Suppression of Concentration Quenching and Spectrum Broadening. *Angew. Chem., Int. Ed.* **2022**, *61*, e202113206.
(643) Huang, F.; Fan, X. C.; Cheng, Y. C.; Wu, H.; Shi, Y. Z.; Yu, J.; Wang, K.; Lee, C. S.; Zhang, X. H. Distinguishing the Respective Determining Factors for Spectral Broadening and Concentration Quenching in Multiple Resonance Type TADF Emitter Systems. *Mater. Horiz.* **2022**, *9*, 2226-2232.

(644) Liu, F.; Cheng, Z.; Wan, L.; Feng, Z.; Liu, H.; Jin, H.; Gao, L.; Lu, P.; Yang, W. Highly Efficient Multi-Resonance Thermally Activated Delayed Fluorescence Material with a Narrow Full Width at Half-Maximum of 0.14 Ev. *Small* **2022**, *18*, 2106462.
(645) Wang, Q.; Xu, Y.; Yang, T.; Xue, J.; Wang, Y. Precise Functionalization of a Multiple-Resonance Framework: Constructing Narrowband Organic Electroluminescent Materials with External Quantum Efficiency over 40. *Adv. Mater.* **2023**, *35*, 2205166.
(646) Wang, T.; Zou, Y.; Huang, Z.; Li, N.; Miao, J.; Yang, C. Narrowband Emissive TADF Conjugated Polymers Towards Highly Efficient Solution-Processible OLEDs. *Angew. Chem., Int. Ed.* **2022**, *61*, e202211172.
(647) Lee, Y. T.; Chan, C. Y.; Tanaka, M.; Mamada, M.; Goushi, K.; Tang, X.; Tsuchiya, Y.; Nakanotani, H.; Adachi, C. Tailor-Made Multi-Resonance Terminal Emitters toward Narrowband, High-Efficiency, and Stable Hyperfluorescence Organic Light-Emitting Diodes. *Adv. Optical Mater.* **2022**, *10*, 2200682.
(648) Xue, W.; Yan, H.; He, Y.; Wu, L.; Zhang, X.; Wu, Y.; Xu, J.; He, J.; Yan, C.; Meng, H. Identifying the Molecular Origins of Green Bn-TADF Material Degradation and Device Stability Via in Situ Raman Spectroscopy. *Chem. Eur. J.* **2022**, *28*, e202201006.
(649) Zhang, Y.; Wei, J.; Wang, L.; Huang, T.; Meng, G.; Wang, X.; Zeng, X.; Du, M.; Fan, T.; Yin, C.et al. Multiple Fusion Strategy for High-Performance Yellow OLEDs with Full Width at Half Maximums Down to 23 Nm and External Quantum Efficiencies up to 37.4. *Adv. Mater.* **2022**, *35*, e2209396.
(650) Zhang, Y.; Li, G.; Wang, L.; Huang, T.; Wei, J.; Meng, G.; Wang, X.; Zeng, X.; Zhang, D.; Duan, L. Fusion of Multi-Resonance Fragment with Conventional Polycyclic Aromatic Hydrocarbon for Nearly Bt.2020 Green Emission. *Angew. Chem., Int. Ed.* **2022**, *61*, e202202380.
(651) Luo, X. F.; Song, S. Q.; Ni, H. X.; Ma, H.; Yang, D.; Ma, D.; Zheng, Y. X.; Zuo, J. L. Multiple-Resonance-Induced Thermally Activated Delayed Fluorescence Materials Based on Indolo[3,2,1-Jk]Carbazole with an Efficient Narrowband Pure-Green Electroluminescence. *Angew. Chem., Int. Ed.* **2022**, *61*, e202209984.
(652) Cheng, Y. C.; Fan, X. C.; Huang, F.; Xiong, X.; Yu, J.; Wang, K.; Lee, C. S.; Zhang, X. H. A Highly Twisted Carbazole-Fused DABNA Derivative as an Orange-Red TADF Emitter for OLEDs with Nearly 40 % EQE. *Angew. Chem., Int. Ed.* **2022**, *61*, e202212575.
(653) Lv, X.; Miao, J.; Liu, M.; Peng, Q.; Zhong, C.; Hu, Y.; Cao, X.; Wu, H.; Yang, Y.; Zhou, C.et al. Extending the Pi-Skeleton of Multi-Resonance TADF Materials Towards High-Efficiency Narrowband Deep-Blue Emission. *Angew. Chem., Int. Ed.* **2022**, *61*, e202201588.
(654) Zou, Y.; Hu, J.; Yu, M.; Miao, J.; Xie, Z.; Qiu, Y.; Cao, X.; Yang, C. High-Performance Narrowband Pure-Red OLEDs with External Quantum Efficiencies up to 36.1% and Ultralow Efficiency Roll-Off. *Adv. Mater.* **2022**, *34*, 2201442.
(655) Wang, Y.; Zhang, K.; Chen, F.; Wang, X.; Yang, Q.; Wang, S.; Shao, S.; Wang, L. Boron-, Sulfur- and Nitrogen-Doped Tridecacyclic Aromatic Emitters with Multiple Resonance Effect for Narrowband Red Emission. *Chin. J. Chem.* **2022**, *40*, 2671-2677.
(656) Liu, G.; Sasabe, H.; Kumada, K.; Arai, H.; Kido, J. Nonbonding/Bonding Molecular Orbital Regulation of Nitrogen-Boron-Oxygen-Embedded Blue/Green Multiresonant TADF Emitters with High Efficiency and Color Purity. *Chem. Eur. J.* **2022**, *28*, e202201605.
(657) Luo, X.-F.; Ni, H.-X.; Lv, A.-Q.; Yao, X.-K.; Ma, H.-L.; Zheng, Y.-X. High-Efficiency and Narrowband OLEDs from Blue to Yellow with Ternary Boron/Nitrogen-Based Polycyclic Heteroaromatic Emitters. *Adv. Optical Mater.* **2022**, *10*, 2200504.
(658) Wang, X.; Zhang, Y.; Dai, H.; Li, G.; Liu, M.; Meng, G.; Zeng, X.; Huang, T.; Wang, L.; Peng, Q.et al. Mesityl-Functionalized Multi-Resonance Organoboron Delayed Fluorescent Frameworks with Wide-Range Color Tunability for Narrowband OLEDs. *Angew. Chem., Int. Ed.* **2022**, *61*, e202206916.
(659) Jiang, P.; Zhan, L.; Cao, X.; Lv, X.; Gong, S.; Chen, Z.; Zhou, C.; Huang, Z.; Ni, F.; Zou, Y.; Yang, C. Simple Acridan-Based Multi-Resonance Structures Enable Highly Efficient Narrowband Green TADF Electroluminescence. *Adv. Optical Mater.* **2021**, *9*, 2100825.
(660) Liu, G.; Sasabe, H.; Kumada, K.; Matsunaga, A.; Katagiri, H.; Kido, J. Facile Synthesis of Multi-Resonance Ultra-Pure-Green TADF Emitters Based on Bridged Diarylamine

Derivatives for Efficient OLEDs with Narrow Emission. *J. Mater. Chem. C* **2021**, *9*, 8308-8313.
(661) Hua, T.; Zhan, L.; Li, N.; Huang, Z.; Cao, X.; Xiao, Z.; Gong, S.; Zhou, C.; Zhong, C.; Yang, C. Heavy-Atom Effect Promotes Multi-Resonance Thermally Activated Delayed Fluorescence. *Chem. Eng. J.* **2021**, *426*, 131169.
(662) Hu, Y. X.; Miao, J.; Hua, T.; Huang, Z.; Qi, Y.; Zou, Y.; Qiu, Y.; Xia, H.; Liu, H.; Cao, X.; Yang, C. Efficient Selenium-Integrated TADF OLEDs with Reduced Roll-Off. *Nat. Photonics* **2022**, *16*, 803-810.
(663) Hu, J.-J.; Luo, X.-F.; Mao, M.-X.; Ni, H.-X.; Liang, X.; Zhang, Y.-P.; Zheng, Y.-X. Green Multi-Resonance Induced Thermally Activated Delayed Fluorescence Emitters Containing Phenoxazine Units with Highly Efficient Electroluminescence. *J. Mater. Chem. C* **2022**, *10*, 768-773.
(664) Liu, J.; Zhu, Y.; Tsuboi, T.; Deng, C.; Lou, W.; Wang, D.; Liu, T.; Zhang, Q. Toward a Bt.2020 Green Emitter through a Combined Multiple Resonance Effect and Multi-Lock Strategy. *Nat Commun* **2022**, *13*, 4876.
(665) Qiu, Y.; Xia, H.; Miao, J.; Huang, Z.; Li, N.; Cao, X.; Han, J.; Zhou, C.; Zhong, C.; Yang, C. Narrowing the Electroluminescence Spectra of Multiresonance Emitters for High-Performance Blue OLEDs by a Peripheral Decoration Strategy. *ACS Appl. Mater. Interfaces* **2021**, *13*, 59035-59042.
(666) Liang, X.; Yan, Z. P.; Han, H. B.; Wu, Z. G.; Zheng, Y. X.; Meng, H.; Zuo, J. L.; Huang, W. Peripheral Amplification of Multi-Resonance Induced Thermally Activated Delayed Fluorescence for Highly Efficient OLEDs. *Angew. Chem., Int. Ed.* **2018**, *57*, 11316-11320.
(667) Bian, J.; Chen, S.; Qiu, L.; Zhang, N.; Zhang, J.; Duan, C.; Han, C.; Xu, H. Synergetic Insulation and Induction Effects Selectively Optimize Multiresonance Thermally Activated Delayed Fluorescence. *Research* **2022**, *2022*, 9838120.
(668) Bian, J.; Chen, S.; Qiu, L.; Tian, R.; Man, Y.; Wang, Y.; Chen, S.; Zhang, J.; Duan, C.; Han, C.; Xu, H. Ambipolar Self-Host Functionalization Accelerates Blue Multi-Resonance Thermally Activated Delayed Fluorescence with Internal Quantum Efficiency of 100. *Adv. Mater.* **2022**, *34*, 2110547.
(669) Hua, T.; Miao, J.; Xia, H.; Huang, Z.; Cao, X.; Li, N.; Yang, C. Sulfone-Incorporated Multi-Resonance TADF Emitter for High-Performance Narrowband Blue OLEDs with EQE of 32%. *Adv. Funct. Mater.* **2022**, *32*, 2201032.
(670) Park, J.; Moon, J.; Lim, J.; Woo, J.; Yoon, S. S.; Lee, J. Y. Fine-Tuned Asymmetric Blue Multiple Resonance Thermally Activated Delayed Fluorescence Emitters with High Efficiency and Narrow Emission Band. *J. Mater. Chem. C* **2022**, *10*, 12300-12306.
(671) Park, J.; Lim, J.; Lee, J. H.; Jang, B.; Han, J. H.; Yoon, S. S.; Lee, J. Y. Asymmetric Blue Multiresonance TADF Emitters with a Narrow Emission Band. *ACS Appl. Mater. Interfaces* **2021**, *13*, 45798-45805.
(672) Han, J.; Huang, Z.; Lv, X.; Miao, J.; Qiu, Y.; Cao, X.; Yang, C. Simple Molecular Design Strategy for Multiresonance Induced TADF Emitter: Highly Efficient Deep Blue to Blue Electroluminescence with High Color Purity. *Adv. Optical Mater.* **2022**, *10*, 2102092.
(673) Liu, J.; Chen, L.; Wang, X.; Yang, Q.; Zhao, L.; Tong, C.; Wang, S.; Shao, S.; Wang, L. Multiple Resonance Dendrimers Containing Boron, Oxygen, Nitrogen-Doped Polycyclic Aromatic Emitters for Narrowband Blue-Emitting Solution-Processed OLEDs. *Macromol. Rapid Commun.* **2022**, *43*, 2200079.
(674) Park, I. S.; Min, H.; Yasuda, T. Ultrafast Triplet-Singlet Exciton Interconversion in Narrowband Blue Organoboron Emitters Doped with Heavy Chalcogens. *Angew. Chem., Int. Ed.* **2022**, *61*, e202205684.
(675) Li, Q.; Wu, Y.; Yang, Q.; Wang, S.; Shao, S.; Wang, L. Selenium-Doped Polycyclic Aromatic Hydrocarbon Multiresonance Emitters with Fast Reverse Intersystem Crossing for Narrowband Blue Emission. *ACS Appl. Mater. Interfaces* **2022**, *14*, 49995-50003.
(676) Meng, G.; Liu, L.; He, z.; Hall, D.; Wang, X.; Peng, T.; Yin, X.; Chen, P.; Beljonne, D.; Olivier, Y.et al. Multi-Resonant Thermally Activated Delayed Fluorescence Emitters Based on Tetracoordinate Boron-Containing Pahs: Colour Tuning Based on the Nature of Chelate. *Chem. Sci.* **2022**, *13*, 1665-1674.

(677) Oda, S.; Kawakami, B.; Kawasumi, R.; Okita, R.; Hatakeyama, T. Multiple Resonance Effect-Induced Sky-Blue Thermally Activated Delayed Fluorescence with a Narrow Emission Band. *Org. Lett.* **2019**, *21*, 9311-9314.
(678) Knoller, J. A.; Meng, G.; Wang, X.; Hall, D.; Pershin, A.; Beljonne, D.; Olivier, Y.; Laschat, S.; Zysman-Colman, E.; Wang, S. Intramolecular Borylation Via Sequential B-Mes Bond Cleavage for the Divergent Synthesis of B,N,B-Doped Benzo[4]Helicenes. *Angew. Chem., Int. Ed.* **2020**, *59*, 3156-3160.
(679) Nagata, M.; Min, H.; Watanabe, E.; Fukumoto, H.; Mizuhata, Y.; Tokitoh, N.; Agou, T.; Yasuda, T. Fused-Nonacyclic Multi-Resonance Delayed Fluorescence Emitter Based on Ladder-Thiaborin Exhibiting Narrowband Sky-Blue Emission with Accelerated Reverse Intersystem Crossing. *Angew. Chem., Int. Ed.* **2021**, *60*, 20280-20285.
(680) Chan, C. Y.; Madayanad Suresh, S.; Lee, Y. T.; Tsuchiya, Y.; Matulaitis, T.; Hall, D.; Slawin, A. M. Z.; Warriner, S.; Beljonne, D.; Olivier, Y.et al. Two Boron Atoms Versus One: High-Performance Deep-Blue Multi-Resonance Thermally Activated Delayed Fluorescence Emitters. *Chem. Commun.* **2022**, *58*, 9377-9380.
(681) Meng, G.; Dai, H.; Huang, T.; Wei, J.; Zhou, J.; Li, X.; Wang, X.; Hong, X.; Yin, C.; Zeng, X.et al. Amine-Directed Formation of B-N Bonds for Bn-Fused Polycyclic Aromatic Multiple Resonance Emitters with Narrowband Emission. *Angew. Chem., Int. Ed.* **2022**, *61*, e202207293.
(682) Bae, J.; Sakai, M.; Tsuchiya, Y.; Ando, N.; Chen, X. K.; Nguyen, T. B.; Chan, C. Y.; Lee, Y. T.; Auffray, M.; Nakanotani, H.et al. Multiple Resonance Type Thermally Activated Delayed Fluorescence by Dibenzo [1,4] Azaborine Derivatives. *Front. Chem.* **2022**, *10*, 990918.
(683) Li, X.; Shi, Y. Z.; Wang, K.; Zhang, M.; Zheng, C. J.; Sun, D. M.; Dai, G. L.; Fan, X. C.; Wang, D. Q.; Liu, W.et al. Thermally Activated Delayed Fluorescence Carbonyl Derivatives for Organic Light-Emitting Diodes with Extremely Narrow Full Width at Half-Maximum. *ACS Appl. Mater. Interfaces* **2019**, *11*, 13472-13480.
(684) Hall, D.; Suresh, S. M.; dos Santos, P. L.; Duda, E.; Bagnich, S.; Pershin, A.; Rajamalli, P.; Cordes, D. B.; Slawin, A. M. Z.; Beljonne, D.et al. Improving Processability and Efficiency of Resonant TADF Emitters: A Design Strategy. *Adv. Optical Mater.* **2020**, *8*, 1901627.
(685) Yuan, Y.; Tang, X.; Du, X. Y.; Hu, Y.; Yu, Y. J.; Jiang, Z. Q.; Liao, L. S.; Lee, S. T. The Design of Fused Amine/Carbonyl System for Efficient Thermally Activated Delayed Fluorescence: Novel Multiple Resonance Core and Electron Acceptor. *Adv. Optical Mater.* **2019**, *7*, 1801536.
(686) Qiu, X.; Tian, G.; Lin, C.; Pan, Y.; Ye, X.; Wang, B.; Ma, D.; Hu, D.; Luo, Y.; Ma, Y. Narrowband Emission from Organic Fluorescent Emitters with Dominant Low-Frequency Vibronic Coupling. *Adv. Optical Mater.* **2021**, *9*, 2001845.
(687) Huang, F.; Wang, K.; Shi, Y. Z.; Fan, X. C.; Zhang, X.; Yu, J.; Lee, C. S.; Zhang, X. H. Approaching Efficient and Narrow RGB Electroluminescence from D-a-Type TADF Emitters Containing an Identical Multiple Resonance Backbone as the Acceptor. *ACS Appl. Mater. Interfaces* **2021**, *13*, 36089-36097.
(688) Wu, S.; Li, W.; Yoshida, K.; Hall, D.; Madayanad Suresh, S.; Sayner, T.; Gong, J.; Beljonne, D.; Olivier, Y.; Samuel, I. D. W.; Zysman-Colman, E. Excited-State Modulation in Donor-Substituted Multiresonant Thermally Activated Delayed Fluorescence Emitters. *ACS Appl. Mater. Interfaces* **2022**, *14*, 22341-22352.
(689) Wu, S.; Kumar Gupta, A.; Yoshida, K.; Gong, J.; Hall, D.; Cordes, D. B.; Slawin, A. M. Z.; Samuel, I. D. W.; Zysman-Colman, E. Highly Efficient Green and Red Narrowband Emissive Organic Light-Emitting Diodes Employing Multi-Resonant Thermally Activated Delayed Fluorescence Emitters. *Angew. Chem., Int. Ed.* **2022**, *61*, e202213697.
(690) Liu, J.-F.; Zou, S.-N.; Chen, X.; Yang, S.-Y.; Yu, Y.-J.; Fung, M.-K.; Jiang, Z.-Q.; Liao, L.-S. Isomeric Thermally Activated Delayed Fluorescence Emitters Based on a Quinolino[3,2,1-De]Acridine-5,9-Dione Multiple Resonance Core and Carbazole Substituent. *Mater. Chem. Front.* **2022**, *6*, 966-972.
(691) Karaman, M.; Kumar Gupta, A.; Madayanad Suresh, S.; Matulaitis, T.; Mardegan, L.; Tordera, D.; Bolink, H. J.; Wu, S.; Warriner, S.; Samuel, I. D.; Zysman-Colman, E. Ionic

Multiresonant Thermally Activated Delayed Fluorescence Emitters for Light Emitting Electrochemical Cells. *Beilstein J. Org. Chem.* **2022**, *18*, 1311-1321.
(692) Sun, D.; Suresh, S. M.; Hall, D.; Zhang, M.; Si, C.; Cordes, D. B.; Slawin, A. M. Z.; Olivier, Y.; Zhang, X.; Zysman-Colman, E. The Design of an Extended Multiple Resonance TADF Emitter Based on a Polycyclic Amine/Carbonyl System. *Mater. Chem. Front.* **2020**, *4*, 2018-2022.
(693) Zou, S. N.; Peng, C. C.; Yang, S. Y.; Qu, Y. K.; Yu, Y. J.; Chen, X.; Jiang, Z. Q.; Liao, L. S. Fully Bridged Triphenylamine Derivatives as Color-Tunable Thermally Activated Delayed Fluorescence Emitters. *Org. Lett.* **2021**, *23*, 958-962.
(694) Min, H.; Park, I. S.; Yasuda, T. Cis-Quinacridone-Based Delayed Fluorescence Emitters: Seemingly Old but Renewed Functional Luminogens. *Angew. Chem., Int. Ed.* **2021**, *60*, 7643-7648.
(695) dos Santos, J. M.; Sun, D.; Moreno-Naranjo, J. M.; Hall, D.; Zinna, F.; Ryan, S. T. J.; Shi, W.; Matulaitis, T.; Cordes, D. B.; Slawin, A. M. Z.et al. An S-Shaped Double Helicene Showing Both Multi-Resonance Thermally Activated Delayed Fluorescence and Circularly Polarized Luminescence. *J. Mater. Chem. C* **2022**, *10*, 4861-4870.
(696) Huang, J.-W.; Hsu, Y.-C.; Wu, X.; Wang, S.; Gan, X.-Q.; Zheng, W.-Q.; Zhang, H.; Gong, Y.-Z.; Hung, W.-Y.; Chou, P.-T.; Zhu, W. Influence of Charge Transfer Strength on Emission Bandwidth for Multiple-Resonance Emitters Via Systematically Tuning the Acceptor–Donor Assembly. *J. Mater. Chem. C* **2022**, *10*, 7866-7874.
(697) Tsuchiya, Y.; Ishikawa, Y.; Lee, S. H.; Chen, X. K.; Brédas, J. L.; Nakanotani, H.; Adachi, C. Thermally Activated Delayed Fluorescence Properties of Trioxoazatriangulene Derivatives Modified with Electron Donating Groups. *Adv. Optical Mater.* **2021**, *9*, 2002174.
(698) Wang, K.; Fan, X.-C.; Tsuchiya, Y.; Shi, Y.-Z.; Tanaka, M.; Lin, Z.; Lee, Y.-T.; Zhang, X.; Liu, W.; Dai, G.-L.et al. Efficient and High Colour Purity RGB OLEDs Employing Densely Packed Dimers. *Chemrxiv* **2023**, 10.26434/chemrxiv-2023-wxk36-v2.
(699) Patil, V. V.; Lee, H. L.; Kim, I.; Lee, K. H.; Chung, W. J.; Kim, J.; Park, S.; Choi, H.; Son, W. J.; Jeon, S. O.; Lee, J. Y. Purely Spin-Vibronic Coupling Assisted Triplet to Singlet up-Conversion for Real Deep Blue Organic Light-Emitting Diodes with over 20% Efficiency and Y Color Coordinate of 0.05. *Adv. Sci.* **2021**, *20*, 2101137.
(700) Hall, D.; Stavrou, K.; Duda, E.; Danos, A.; Bagnich, S.; Warriner, S.; Slawin, A. M. Z.; Beljonne, D.; Kohler, A.; Monkman, A.et al. Diindolocarbazole - Achieving Multiresonant Thermally Activated Delayed Fluorescence without the Need for Acceptor Units. *Mater. Horiz.* **2022**, *9*, 1068-1080.
(701) Lee, H. L.; Jeon, S. O.; Kim, I.; Kim, S. C.; Lim, J.; Kim, J.; Park, S.; Chwae, J.; Son, W. J.; Choi, H.; Lee, J. Y. Multiple-Resonance Extension and Spin-Vibronic-Coupling-Based Narrowband Blue Organic Fluorescence Emitters with over 30% Quantum Efficiency. *Adv. Mater.* **2022**, *34*, 2202464.
(702) Tsujimoto, H.; Ha, D.-G.; Markopoulos, G.; Chae, H. S.; Baldo, M. A.; Swager, T. M. Thermally Activated Delayed Fluorescence and Aggregation Induced Emission with through-Space Charge Transfer. *J. Am. Chem. Soc.* **2017**, *139*, 4894-4900.
(703) Wada, Y.; Nakagawa, H.; Matsumoto, S.; Wakisaka, Y.; Kaji, H. Organic Light Emitters Exhibiting Very Fast Reverse Intersystem Crossing. *Nat. Photonics* **2020**, *14*, 643-649.
(704) Kusakabe, Y.; Wada, Y.; Nakagawa, H.; Shizu, K.; Kaji, H. Conformation Control of Iminodibenzyl-Based Thermally Activated Delayed Fluorescence Material by Tilted Face-to-Face Alignment with Optimal Distance (TFFO) Design. *Front. Chem.* **2020**, *8*, 530.
(705) Tang, X.; Cui, L.-S.; Li, H.-C.; Gillett, A. J.; Auras, F.; Qu, Y.-K.; Zhong, C.; Jones, S. T. E.; Jiang, Z.-Q.; Friend, R. H.; Liao, L.-S. Highly Efficient Luminescence from Space-Confined Charge-Transfer Emitters. *Nat. Mater.* **2020**, *19*, 1332-1338.
(706) Feng, Z. Q.; Yang, S. Y.; Kong, F. C.; Qu, Y. K.; Meng, X. Y.; Yu, Y. J.; Zhou, D. Y.; Jiang, Z. Q.; Liao, L. S. Indirect Control of Donor/Acceptor Interactions for Highly Efficient Space-Confined Thermally Activated Delayed Fluorescence Emitters. *Adv. Funct. Mater.* **2023**, *33*, 2209708.

(707) Zheng, Q.; Wang, X.-Q.; Qu, Y.-K.; Xie, G.; Liao, L.-S.; Jiang, Z.-Q. Solution-Processable through-Space Charge-Transfer Emitters Via Solubilizing Groups Modification. *Npj Flex. Electron.* **2022**, *6*, 83.
(708) Yang, S. Y.; Tian, Q. S.; Yu, Y. J.; Zou, S. N.; Li, H. C.; Khan, A.; Wu, Q. H.; Jiang, Z. Q.; Liao, L. S. Sky-Blue Thermally Activated Delayed Fluorescence with Intramolecular Spatial Charge Transfer Based on a Dibenzothiophene Sulfone Emitter. *J. Org. Chem.* **2020**, *85*, 10628-10637.
(709) Wang, T.-T.; Xie, G.; Li, H.-C.; Yang, S.-Y.; Li, H.; Xiao, Y.-L.; Zhong, C.; Sarvendra, K.; Khan, A.; Jiang, Z.-Q.; Liao, L.-S. Π-Stacked Thermally Activated Delayed Fluorescence Emitters with Alkyl Chain Modulation. *CCS Chem.* **2021**, *3*, 1757-1763.
(710) Yang, S. Y.; Wang, Y. K.; Peng, C. C.; Wu, Z. G.; Yuan, S.; Yu, Y. J.; Li, H.; Wang, T. T.; Li, H. C.; Zheng, Y. X.et al. Circularly Polarized Thermally Activated Delayed Fluorescence Emitters in through-Space Charge Transfer on Asymmetric Spiro Skeletons. *J. Am. Chem. Soc.* **2020**, *142*, 17756-17765.
(711) Yang, S. Y.; Feng, Z. Q.; Fu, Z.; Zhang, K.; Chen, S.; Yu, Y. J.; Zou, B.; Wang, K.; Liao, L. S.; Jiang, Z. Q. Highly Efficient Sky-Blue Pi-Stacked Thermally Activated Delayed Fluorescence Emitter with Multi-Stimulus Response Properties. *Angew. Chem., Int. Ed.* **2022**, *61*, e202206861.
(712) Peng, C.-C.; Yang, S.-Y.; Li, H.-C.; Xie, G.-H.; Cui, L.-S.; Zou, S.-N.; Poriel, C.; Jiang, Z.-Q.; Liao, L.-S. Highly Efficient Thermally Activated Delayed Fluorescence Via an Unconjugated Donor–Acceptor System Realizing EQE of over 30%. *Adv. Mater.* **2020**, *32*, 2003885.
(713) Zhao, Z.; Zeng, C.; Peng, X.; Liu, Y.; Zhao, H.; Hua, L.; Su, S. J.; Yan, S.; Ren, Z. Tuning Intramolecular Stacking of Rigid Heteroaromatic Compounds for High-Efficiency Deep-Blue through-Space Charge-Transfer Emission. *Angew. Chem., Int. Ed.* **2022**, *61*, e202210864.
(714) Huang, T.; Wang, Q.; Meng, G.; Duan, L.; Zhang, D. Accelerating Radiative Decay in Blue through-Space Charge Transfer Emitters by Minimizing the Face-to-Face Donor-Acceptor Distances. *Angew. Chem., Int. Ed.* **2022**, *61*, e202200059.
(715) Xie, F. M.; Li, H. Z.; Zhang, K.; Shen, Y.; Zhao, X.; Li, Y. Q.; Tang, J. X. A Dislocated Twin-Locking Acceptor-Donor-Acceptor Configuration for Efficient Delayed Fluorescence with Multiple through-Space Charge Transfer. *Angew. Chem., Int. Ed.* **2022**, *61*, e202213823.
(716) Song, Y.; Tian, M.; Yu, R.; He, L. Through-Space Charge-Transfer Emitters Developed by Fixing the Acceptor for High-Efficiency Thermally Activated Delayed Fluorescence. *ACS Appl. Mater. Interfaces* **2021**, *13*, 60269-60278.
(717) Wu, C.; Liu, W.; Li, K.; Cheng, G.; Xiong, J.; Teng, T.; Che, C. M.; Yang, C. Face-to-Face Orientation of Quasiplanar Donor and Acceptor Enables Highly Efficient Intramolecular Exciplex Fluorescence. *Angew. Chem., Int. Ed.* **2021**, *60*, 3994-3998.
(718) Wang, J.; Miao, J.; Jiang, C.; Luo, S.; Yang, C.; Li, K. Engineering Intramolecular Π-Stacking Interactions of through-Space Charge-Transfer TADF Emitters for Highly Efficient OLEDs with Improved Color Purity. *Adv. Optical Mater.* **2022**, *10*, 2201071.
(719) Jiang, C.; Miao, J.; Zhang, D.; Wen, Z.; Yang, C.; Li, K. Acceptor-Donor-Acceptor Pi-Stacking Boosts Intramolecular through-Space Charge Transfer Towards Efficient Red TADF and High-Performance OLEDs. *Research* **2022**, *2022*, 9892802.
(720) Zhang, D.; Jiang, C.; Wen, Z.; Feng, X.; Li, K. Influence of Sulfur Atoms on TADF Properties from through-Space Charge Transfer Excited States. *Chem. Eur. J.* **2022**, *28*, e202202305.
(721) Li, K.; Wang, T.; Yao, B.; Chen, Y.; Deng, H.; Zhan, H.; Xie, Z.; Cheng, Y. Carbazole Ring: A Delicate Rack for Constructing Thermally Activated Delayed Fluorescent Compounds with through-Space Charge Transfer. *Chin. Chem. Lett.* **2021**, *32*, 4011-4014.
(722) Li, K.; Chen, Y.; Yao, B.; Dou, K.; Wang, T.; Deng, H.; Zhan, H.; Xie, Z.; Cheng, Y. Insight into through-Space Conjugation in Rotation-Restricted Thermally Activated Delayed Fluorescence Compounds. *J. Mater. Chem. C* **2022**, *10*, 15152-15159.
(723) Chen, X. L.; Jia, J. H.; Yu, R.; Liao, J. Z.; Yang, M. X.; Lu, C. Z. Combining Charge-Transfer Pathways to Achieve Unique Thermally Activated Delayed Fluorescence Emitters for High-Performance Solution-Processed, Non-Doped Blue OLEDs. *Angew. Chem., Int. Ed.* **2017**, *56*, 15006-15009.

(724) Kim, J.; Lee, T.; Ryu, J. Y.; Lee, Y. H.; Lee, J.; Jung, J.; Lee, M. H. Highly Emissive Ortho-Donor–Acceptor Triarylboranes: Impact of Boryl Acceptors on Luminescence Properties. *Organometallics* **2020**, *39*, 2235-2244.

(725) Yin, C.; Zhang, D.; Zhang, Y.; Lu, Y.; Wang, R.; Li, G.; Duan, L. High-Efficiency Narrow-Band Electro-Fluorescent Devices with Thermally Activated Delayed Fluorescence Sensitizers Combined through-Bond and through-Space Charge Transfers. *CCS Chem.* **2020**, *2*, 1268-1277.

(726) Huang, T.; Wang, Q.; Xiao, S.; Zhang, D.; Zhang, Y.; Yin, C.; Yang, D.; Ma, D.; Wang, Z.; Duan, L. Simultaneously Enhanced Reverse Intersystem Crossing and Radiative Decay in Thermally Activated Delayed Fluorophors with Multiple through-Space Charge Transfers. *Angew. Chem., Int. Ed.* **2021**, *60*, 23771-23776.

(727) Lv, X.; Wang, Y.; Li, N.; Cao, X.; Xie, G.; Huang, H.; Zhong, C.; Wang, L.; Yang, C. Regulating the Photophysical Properties of Highly Twisted TADF Emitters by Concurrent through-Space/-Bond Charge Transfer. *Chem. Eng. J.* **2020**, *402*, 126173.

(728) Huang, Y.; Zhang, D.-H.; Tao, X.-D.; Wei, Z.; Jiang, S.; Meng, L.; Yang, M.-X.; Chen, X.-L.; Lu, C.-Z. Triptycene-Derived Thermally Activated Delayed Fluorescence Emitters with Combined through-Bond and through-Space Charge Transfers. *Dyes Pigm.* **2022**, *204*, 110397.

(729) Wang, X.; Wang, S.; Lv, J.; Shao, S.; Wang, L.; Jing, X.; Wang, F. Through-Space Charge Transfer Hexaarylbenzene Dendrimers with Thermally Activated Delayed Fluorescence and Aggregation-Induced Emission for Efficient Solution-Processed OLEDs. *Chem. Sci.* **2019**, *10*, 2915-2923.

(730) Li, J.; Zhou, L.; He, J.; Xue, Q.; Xu, L.; Xie, G. Propeller-Shape Isomers with Turn-on through-Space Charge Transfer for Solution-Processed Non-Doped Organic Light-Emitting Diodes. *Chem. Eng. J.* **2023**, *452*, 139120.

(731) Kawasumi, K.; Wu, T.; Zhu, T.; Chae, H. S.; Van Voorhis, T.; Baldo, M. A.; Swager, T. M. Thermally Activated Delayed Fluorescence Materials Based on Homoconjugation Effect of Donor–Acceptor Triptycenes. *J. Am. Chem. Soc.* **2015**, *137*, 11908-11911.

(732) Dai, G.; Zhang, M.; Wang, K.; Fan, X.; Shi, Y.; Sun, D.; Liu, W.; Chen, J.; Yu, J.; Ou, X.et al. Nonconjugated Triptycene-Spaced Donor-Acceptor-Type Emitters Showing Thermally Activated Delayed Fluorescence Via Both Intra- and Intermolecular Charge-Transfer Transitions. *ACS Appl. Mater. Interfaces* **2021**, *13*, 25193-25201.

(733) Spuling, E.; Sharma, N.; Samuel, I.; Zysman-Colman, E.; Braese, S. (Deep) Blue through-Space Conjugated TADF Emitters Based on [2.2]Paracyclophanes. *Chem. Commun.* **2018**, *54*, 9278-9281.

(734) Auffray, M.; Kim, D. H.; Kim, J. U.; Bencheikh, F.; Kreher, D.; Zhang, Q.; D'Aleo, A.; Ribierre, J. C.; Mathevet, F.; Adachi, C. Dithia[3.3]Paracyclophane Core: A Versatile Platform for Triplet State Fine-Tuning and through-Space TADF Emission. *Chem. Asian J.* **2019**, *14*, 1921-1925.

(735) Wang, H.; Xie, L.; Peng, Q.; Meng, L.; Wang, Y.; Yi, Y.; Wang, P. Novel Thermally Activated Delayed Fluorescence Materials–Thioxanthone Derivatives and Their Applications for Highly Efficient OLEDs. *Adv. Mater.* **2014**, *26*, 5198-5204.

(736) Wu, K.; Wang, Z.; Zhan, L.; Zhong, C.; Gong, S.; Xie, G.; Yang, C. Realizing Highly Efficient Solution-Processed Homojunction-Like Sky-Blue OLEDs by Using Thermally Activated Delayed Fluorescent Emitters Featuring an Aggregation-Induced Emission Property. *J. Phys. Chem. Lett* **2018**, *9*, 1547-1553.

(737) Zhao, J.; Chen, X.; Yang, Z.; Liu, T.; Yang, Z.; Zhang, Y.; Xu, J.; Chi, Z. Highly-Efficient Doped and Nondoped Organic Light-Emitting Diodes with External Quantum Efficiencies over 20% from a Multifunctional Green Thermally Activated Delayed Fluorescence Emitter. *J. Phys. Chem. C.* **2019**, *123*, 1015-1020.

(738) Yang, Z.; Mao, Z.; Xu, C.; Chen, X.; Zhao, J.; Yang, Z.; Zhang, Y.; Wu, W.; Jiao, S.; Liu, Y.et al. A Sterically Hindered Asymmetric D–a–D′ Thermally Activated Delayed Fluorescence Emitter for Highly Efficient Non-Doped Organic Light-Emitting Diodes. *Chem. Sci.* **2019**, *10*, 8129-8134.

(739) Guo, R.; Leng, P.; Zhang, Q.; Wang, Y.; Lv, X.; Sun, S.; Ye, S.; Duan, Y.; Wang, L. Donor Engineering for Diphenylsulfone Derivatives with Both Thermally Activated Delayed Fluorescence and Aggregation-Induced Emission Properties. *Dyes Pigm.* **2021**, *184*, 108781.
(740) Leng, P.; Sun, S.; Guo, R.; Zhang, Q.; Liu, W.; Lv, X.; Ye, S.; Wang, L. Modifying the AIE-TADF Chromophore with Host-Substituents to Achieve High Efficiency and Low Roll-Off Non-Doped OLEDs. *Org. Electron.* **2020**, *78*, 105602.
(741) Xiang, S.; Huang, Z.; Sun, S.; Lv, X.; Fan, L.; Ye, S.; Chen, H.; Guo, R.; Wang, L. Highly Efficient Non-Doped OLEDs Using Aggregation-Induced Delayed Fluorescence Materials Based on 10-Phenyl-10h-Phenothiazine 5,5-Dioxide Derivatives. *J. Mater. Chem. C* **2018**, *6*, 11436-11443.
(742) Guo, J.; Li, X.-L.; Nie, H.; Luo, W.; Gan, S.; Hu, S.; Hu, R.; Qin, A.; Zhao, Z.; Su, S.-J.; Tang, B. Z. Achieving High-Performance Nondoped OLEDs with Extremely Small Efficiency Roll-Off by Combining Aggregation-Induced Emission and Thermally Activated Delayed Fluorescence. *Adv. Funct. Mater.* **2017**, *27*, 1606458.
(743) Chen, H.; Liu, H.; Xiong, Y.; He, J.; Zhao, Z.; Tang, B. Z. New Aggregation-Induced Delayed Fluorescent Materials for Efficient OLEDs with High Stabilities of Emission Color and Efficiency. *Mater. Chem. Front.* **2022**, *6*, 924-932.
(744) Huang, J.; Nie, H.; Zeng, J.; Zhuang, Z.; Gan, S.; Cai, Y.; Guo, J.; Su, S.-J.; Zhao, Z.; Tang, B. Z. Highly Efficient Nondoped OLEDs with Negligible Efficiency Roll-Off Fabricated from Aggregation-Induced Delayed Fluorescence Luminogens. *Angew. Chem., Int. Ed.* **2017**, *56*, 12971-12976.
(745) Guo, J.; Fan, J.; Lin, L.; Zeng, J.; Liu, H.; Wang, C.-K.; Zhao, Z.; Tang, B. Z. Mechanical Insights into Aggregation-Induced Delayed Fluorescence Materials with Anti-Kasha Behavior. *Adv. Sci.* **2019**, *6*, 1801629.
(746) Liu, H.; Liu, H.; Fan, J.; Guo, J.; Zeng, J.; Qiu, F.; Zhao, Z.; Tang, B. Z. An Effective Design Strategy for Robust Aggregation-Induced Delayed Fluorescence Luminogens to Improve Efficiency Stability of Nondoped and Doped OLEDs. *Adv. Optical Mater.* **2020**, *8*, 2001027.
(747) Fu, Y.; Liu, H.; Zhu, X.; Zeng, J.; Zhao, Z.; Tang, B. Z. Efficient Aggregation-Induced Delayed Fluorescent Materials Based on Bipolar Carrier Transport Materials for the Fabrication of High-Performance Nondoped OLEDs with Very Small Efficiency Roll-Off. *J. Mater. Chem. C* **2020**, *8*, 9549-9557.
(748) Xu, J.; Zhu, X.; Guo, J.; Fan, J.; Zeng, J.; Chen, S.; Zhao, Z.; Tang, B. Z. Aggregation-Induced Delayed Fluorescence Luminogens with Accelerated Reverse Intersystem Crossing for High-Performance OLEDs. *ACS Mater. Lett.* **2019**, *1*, 613-619.
(749) Xu, J.; Wu, X.; Li, J.; Zhao, Z.; Tang, B. Z. Regulating Photophysical Property of Aggregation-Induced Delayed Fluorescence Luminogens Via Heavy Atom Effect to Achieve Efficient Organic Light-Emitting Diodes. *Adv. Optical mater.* **2022**, *10*, 2102568.
(750) Liu, H.; Zeng, J.; Guo, J.; Nie, H.; Zhao, Z.; Tang, B. Z. High-Performance Non-Doped OLEDs with Nearly 100% Exciton Use and Negligible Efficiency Roll-Off. *Angew. Chem., Int. Ed.* **2018**, *57*, 9290-9294.
(751) Chen, Y.; Wang, S.; Wu, X.; Xu, Y.; Li, H.; Liu, Y.; Tong, H.; Wang, L. Triazatruxene-Based Small Molecules with Thermally Activated Delayed Fluorescence, Aggregation-Induced Emission and Mechanochromic Luminescence Properties for Solution-Processable Nondoped OLEDs. *J. Mater. Chem. C* **2018**, *6*, 12503-12508.
(752) Chen, J.; Zeng, J.; Zhu, X.; Guo, J.; Zhao, Z.; Tang, B. Z. Versatile Aggregation-Enhanced Delayed Fluorescence Luminogens Functioning as Emitters and Hosts for High-Performance Organic Light-Emitting Diodes. *CCS Chem.* **2021**, *3*, 230-240.
(753) He, J.; Chen, H.; Li, J.; Wang, J.; Xu, J.; Zhao, Z.; Tang, B. Z. Aggregation-Induced Delayed Fluorescence Molecules with Mechanochromic Behaviors for Efficient Blue Organic Light-Emitting Diodes. *Cell Rep. Phys. Sci.* **2022**, *3*, 100733.
(754) Wu, L.; Wang, K.; Wang, C.; Fan, X.-C.; Shi, Y.-Z.; Zhang, X.; Zhang, S.-L.; Ye, J.; Zheng, C.-J.; Li, Y.-Q.et al. Using Fluorene to Lock Electronically Active Moieties in Thermally Activated Delayed Fluorescence Emitters for High-Performance Non-Doped Organic Light-Emitting Diodes with Suppressed Roll-Off. *Chem. Sci.* **2021**, *12*, 1495-1502.

(755) Ma, F.; Zhao, G.; Zheng, Y.; He, F.; Hasrat, K.; Qi, Z. Molecular Engineering of Thermally Activated Delayed Fluorescence Emitters with Aggregation-Induced Emission Via Introducing Intramolecular Hydrogen-Bonding Interactions for Efficient Solution-Processed Nondoped OLEDs. *ACS Appl. Mater. Interfaces* **2020**, *12*, 1179-1189.
(756) Huang, Z.; Bin, Z.; Su, R.; Yang, F.; Lan, J.; You, J. Molecular Design of Non-Doped OLEDs Based on a Twisted Heptagonal Acceptor: A Delicate Balance between Rigidity and Rotatability. *Angew. Chem., Int. Ed.* **2020**, *59*, 9992-9996.
(757) Ma, F.; Cheng, Y.; Zheng, Y.; Ji, H.; Hasrat, K.; Qi, Z. Rational Design of Thermally Activated Delayed Fluorescence Emitters with Aggregation-Induced Emission Employing Combined Charge Transfer Pathways for Fabricating Efficient Non-Doped OLEDs. *J. Mater. Chem. C* **2019**, *7*, 9413-9422.
(758) Wang, J.; Liu, C.; Jiang, C.; Yao, C.; Gu, M.; Wang, W. Solution-Processed Aggregation-Induced Delayed Fluorescence (Aidf) Emitters Based on Strong Π-Accepting Triazine Cores for Highly Efficient Nondoped OLEDs with Low Efficiency Roll-Off. *Org. Electron.* **2019**, *65*, 170-178.
(759) Park, S. Y.; Choi, S.; Park, G. E.; Kim, H. J.; Lee, C.; Moon, J. S.; Kim, S. W.; Park, S.; Kwon, J. H.; Cho, M. J.; Choi, D. H. Unconventional Three-Armed Luminogens Exhibiting Both Aggregation-Induced Emission and Thermally Activated Delayed Fluorescence Resulting in High-Performing Solution-Processed Organic Light-Emitting Diodes. *ACS Appl. Mater. Interfaces* **2018**, *10*, 14966-14977.
(760) Zhang, Q.; Sun, S.; Liu, W.; Leng, P.; Lv, X.; Wang, Y.; Chen, H.; Ye, S.; Zhuang, S.; Wang, L. Integrating TADF Luminogens with AIE Characteristics Using a Novel Acridine–Carbazole Hybrid as Donor for High-Performance and Low Efficiency Roll-Off OLEDs. *J. Mater. Chem. C* **2019**, *7*, 9487-9495.
(761) Furue, R.; Nishimoto, T.; Park, I. S.; Lee, J.; Yasuda, T. Aggregation-Induced Delayed Fluorescence Based on Donor/Acceptor-Tethered Janus Carborane Triads: Unique Photophysical Properties of Nondoped OLEDs. **2016**, *55*, 7171-7175.
(762) Yu, L.; Wu, Z.; Xie, G.; Zhong, C.; Zhu, Z.; Ma, D.; Yang, C. An Efficient Exciton Harvest Route for High-Performance OLEDs Based on Aggregation-Induced Delayed Fluorescence. *Chem. Commun.* **2018**, *54*, 1379-1382.
(763) Shen, Y.-F.; Li, M.; Zhao, W.-L.; Wang, Y.-F.; Lu, H.-Y.; Chen, C.-F. Quinoline-Based TADF Emitters Exhibiting Aggregation-Induced Emission for Efficient Non-Doped Organic Light-Emitting Diodes. *Mater. Chem. Front.* **2021**, *5*, 834-842.
(764) Zhang, L.; Wang, Y.-F.; Li, M.; Gao, Q.-Y.; Chen, C.-F. Quinoline-Based Aggregation-Induced Delayed Fluorescence Materials for Highly Efficient Non-Doped Organic Light-Emitting Diodes. *Chin. Chem. Lett.* **2021**, *32*, 740-744.
(765) Kim, H. J.; Kang, H.; Jeong, J. E.; Park, S. H.; Koh, C. W.; Kim, C. W.; Woo, H. Y.; Cho, M. J.; Park, S.; Choi, D. H. Ultra-Deep-Blue Aggregation-Induced Delayed Fluorescence Emitters: Achieving Nearly 16% EQE in Solution-Processed Nondoped and Doped OLEDs with Ciey < 0.1. *Adv. Funct. Mater.* **2021**, *31*, 2102588.
(766) Park, S.; Kwon, O. H.; Lee, Y. S.; Jang, D. J.; Park, S. Y. Imidazole-Based Excited-State Intramolecular Proton-Transfer (Esipt) Materials: Observation of Thermally Activated Delayed Fluorescence (Tdf). *J. Phys. Chem. A* **2007**, *111*, 9649-9653.
(767) Mamada, M.; Inada, K.; Komino, T.; Potscavage, W. J., Jr.; Nakanotani, H.; Adachi, C. Highly Efficient Thermally Activated Delayed Fluorescence from an Excited-State Intramolecular Proton Transfer System. *ACS Cent. Sci.* **2017**, *3*, 769-777.
(768) Wu, K.; Zhang, T.; Wang, Z.; Wang, L.; Zhan, L.; Gong, S.; Zhong, C.; Lu, Z.-H.; Zhang, S.; Yang, C. De Novo Design of Excited-State Intramolecular Proton Transfer Emitters Via a Thermally Activated Delayed Fluorescence Channel. *J. Am. Chem. Soc.* **2018**, *140*, 8877-8886.
(769) Gupta, A. K.; Li, W.; Ruseckas, A.; Lian, C.; Carpenter-Warren, C. L.; Cordes, D. B.; Slawin, A. M. Z.; Jacquemin, D.; Samuel, I. D. W.; Zysman-Colman, E. Thermally Activated Delayed Fluorescence Emitters with Intramolecular Proton Transfer for High Luminance Solution-Processed Organic Light-Emitting Diodes. *ACS Appl. Mater. Interfaces* **2021**, *13*, 15459-15474.

(770) Shao, W.; Hao, J.; Jiang, H.; Zimmerman, P. M.; Kim, J. Metal-Free Organic Triplet Emitters with on–Off Switchable Excited State Intramolecular Proton Transfer. *Adv. Funct. Mater.* **2022**, *32*, 2201256.
(771) Xie, Z.; Chen, C.; Xu, S.; Li, J.; Zhang, Y.; Liu, S.; Xu, J.; Chi, Z. White-Light Emission Strategy of a Single Organic Compound with Aggregation-Induced Emission and Delayed Fluorescence Properties. *Angew. Chem., Int. Ed.* **2015**, *54*, 7181-7184.
(772) Xie, Z.; Su, T.; Ubba, E.; Deng, H.; Mao, Z.; Yu, T.; Zheng, T.; Zhang, Y.; Liu, S.; Chi, Z. Achieving Tunable Dual-Emissive and High-Contrast Mechanochromic Materials by Manipulating Steric Hindrance Effects. *J. Mater. Chem. C* **2019**, *7*, 3300-3305.
(773) Huang, B.; Chen, W. C.; Li, Z.; Zhang, J.; Zhao, W.; Feng, Y.; Tang, B. Z.; Lee, C. S. Manipulation of Molecular Aggregation States to Realize Polymorphism, AIE, MCL, and TADF in a Single Molecule. *Angew. Chem., Int. Ed.* **2018**, *57*, 12473-12477.
(774) Xie, Z.; Huang, Q.; Yu, T.; Wang, L.; Mao, Z.; Li, W.; Yang, Z.; Zhang, Y.; Liu, S.; Xu, J.et al. Hydrogen-Bonding-Assisted Intermolecular Charge Transfer: A New Strategy to Design Single-Component White-Light-Emitting Materials. *Adv. Funct. Mater.* **2017**, *27*, 1703918.
(775) Xu, B.; Mu, Y.; Mao, Z.; Xie, Z.; Wu, H.; Zhang, Y.; Jin, C.; Chi, Z.; Liu, S.; Xu, J.et al. Achieving Remarkable Mechanochromism and White-Light Emission with Thermally Activated Delayed Fluorescence through the Molecular Heredity Principle. *Chem. Sci.* **2016**, *7*, 2201-2206.
(776) Okazaki, M.; Takeda, Y.; Data, P.; Pander, P.; Higginbotham, H.; Monkman, A. P.; Minakata, S. Thermally Activated Delayed Fluorescent Phenothiazine-Dibenzo [a, J] Phenazine-Phenothiazine Triads Exhibiting Tricolor-Changing Mechanochromic Luminescence. *Chem. Sci.* **2017**, *8*, 2677-2686.
(777) Takeda, Y.; Kaihara, T.; Okazaki, M.; Higginbotham, H.; Data, P.; Tohnai, N.; Minakata, S. Conformationally-Flexible and Moderately Electron-Donating Units-Installed D–a–D Triad Enabling Multicolor-Changing Mechanochromic Luminescence, TADF and Room-Temperature Phosphorescence. *Chem. Commun.* **2018**, *54*, 6847-6850.
(778) Pashazadeh, R.; Pander, P.; Bucinskas, A.; Skabara, P. J.; Dias, F. B.; Grazulevicius, J. V. An Iminodibenzyl-Quinoxaline-Iminodibenzyl Scaffold as a Mechanochromic and Dual Emitter: Donor and Bridge Effects on Optical Properties. *Chem. Commun.* **2018**, *54*, 13857-13860.
(779) Yang, W.; Yang, Y.; Zhan, L.; Zheng, K.; Chen, Z.; Zeng, X.; Gong, S.; Yang, C. Polymorphism-Dependent Thermally Activated Delayed Fluorescence Materials with Diverse Three Dimensional Supramolecular Frameworks. *Chem. Eng. J.* **2020**, *390*, 124626.
(780) Pandey, U. P.; Nandi, R. P.; Thilagar, P. Design, Synthesis, and Temperature-Driven Molecular Conformation-Dependent Delayed Fluorescence Characteristics of Dianthrylboron-Based Donor-Acceptor Systems. *Front. Chem.* **2020**, *8*, 541331.
(781) Yu, H.; Song, X.; Xie, N.; Wang, J.; Li, C.; Wang, Y. Reversible Crystal-to-Crystal Phase Transitions with High-Contrast Luminescent Alterations for a Thermally Activated Delayed Fluorescence Emitter. *Adv. Funct. Mater.* **2021**, *31*, 2007511.
(782) Huang, B.; Li, Z.; Yang, H.; Hu, D.; Wu, W.; Feng, Y.; Sun, Y.; Lin, B.; Jiang, W. Bicolour Electroluminescence of 2-(Carbazol-9-Yl)Anthraquinone Based on a Solution Process. *J. Mater. Chem. C* **2017**, *5*, 12031-12034.
(783) Mane, S. K. B.; Mu, Y.; Yang, Z.; Ubba, E.; Shaishta, N.; Chi, Z. A TADF Compound with High-Contrast Mechano-Responsive Fluorescence on/Off Switching for Both Sequential and Combinational Logic Gates. *J. Mater. Chem. C* **2019**, *7*, 3522-3528.
(784) Fu, Y.; Ye, Z.; Xiao, J.; Liao, L.; Chen, L.; Mu, Y.; Ji, S.; Zhao, Z.; Zhang, H.-L.; Huo, Y. Large Effects of Tiny Structural Changes on the AIE-TADF Type Xanthone Derivatives in Mechano-Responsive Luminescence and Electroluminescence. *Dyes Pigm.* **2022**, *205*, 110550.
(785) Zhang, X.; Lu, T.; Zhou, C.; Liu, H.; Wen, Y.; Shen, Y.; Li, B.; Zhang, S.-T.; Yang, B. Thermally Activated Delayed Fluorescence of Aggregates Induced by Strong Π–Π Interactions and Reversible Dual-Responsive Luminescence Switching. *CCS Chem.* **2022**, *4*, 625-637.

(786) Xu, S.; Liu, T.; Mu, Y.; Wang, Y. F.; Chi, Z.; Lo, C. C.; Liu, S.; Zhang, Y.; Lien, A.; Xu, J. An Organic Molecule with Asymmetric Structure Exhibiting Aggregation-Induced Emission, Delayed Fluorescence, and Mechanoluminescence. *Angew. Chem., Int. Ed.* **2015**, *54*, 874-878.

(787) Wong, M. Y.; Hedley, G. J.; Xie, G.; Kölln, L. S.; Samuel, I. D. W.; Pertegás, A.; Bolink, H. J.; Zysman-Colman, E. Light-Emitting Electrochemical Cells and Solution-Processed Organic Light-Emitting Diodes Using Small Molecule Organic Thermally Activated Delayed Fluorescence Emitters. *Chem. Mater.* **2015**, *27*, 6535-6542.

(788) Wong, M. Y.; La-Placa, M.-G.; Pertegas, A.; Bolink, H. J.; Zysman-Colman, E. Deep-Blue Thermally Activated Delayed Fluorescence (TADF) Emitters for Light-Emitting Electrochemical Cells (LEECs). *J. Mater. Chem. C* **2017**, *5*, 1699-1705.

(789) Yu, R.; Song, Y.; Zhang, K.; Pang, X.; Tian, M.; He, L. Intrinsically Ionic, Thermally Activated Delayed Fluorescent Materials for Efficient, Bright, and Stable Light-Emitting Electrochemical Cells. *Adv. Funct. Mater.* **2022**, *32*, 2110623.

(790) Shen, H.-L.; Hsiao, P.-W.; Yi, R.-H.; Su, Y.-H.; Chen, Y.; Lu, C.-W.; Su, H.-C. Purely Organic Pyridium-Based Materials with Thermally Activated Delayed Fluorescence for Orange-Red Light-Emitting Electrochemical Cells. *Dyes Pigm.* **2022**, *203*, 110346.

(791) Lundberg, P.; Lindh, E. M.; Tang, S.; Edman, L. Toward Efficient and Metal-Free Emissive Devices: A Solution-Processed Host–Guest Light-Emitting Electrochemical Cell Featuring Thermally Activated Delayed Fluorescence. *ACS Appl. Mater. Interfaces* **2017**, *9*, 28810-28816.

(792) Lundberg, P.; Wei, Q.; Ge, Z.; Voit, B.; Reineke, S.; Edman, L. Polymer Featuring Thermally Activated Delayed Fluorescence as Emitter in Light-Emitting Electrochemical Cells. *J. Phys. Chem. Lett* **2020**, *11*, 6227-6234.

(793) Lundberg, P.; Tsuchiya, Y.; Lindh, E. M.; Tang, S.; Adachi, C.; Edman, L. Thermally Activated Delayed Fluorescence with 7% External Quantum Efficiency from a Light-Emitting Electrochemical Cell. *Nat. Commun.* **2019**, *10*, 5307.

(794) Ye, J.; He, Y.; Li, K.; Liu, L.; Xi, C.; Liu, Z.; Ma, Y.; Zhang, B.; Bao, Y.; Wang, W.et al. Achieving Record Efficiency and Luminance for TADF Light-Emitting Electrochemical Cells by Dopant Engineering. *ACS Appl. Mater. Interfaces* **2022**, *14*, 17698-17708.

(795) Bai, R.; Meng, X.; Wang, X.; He, L. Color-Stable, Efficient, and Bright Blue Light-Emitting Electrochemical Cell Using Ionic Exciplex Host. *Adv. Funct. Mater.* **2021**, *31*, 2007167.

(796) Tang, S.; Lundberg, P.; Tsuchiya, Y.; Ràfols-Ribé, J.; Liu, Y.; Wang, J.; Adachi, C.; Edman, L. Efficient and Bright Blue Thermally Activated Delayed Fluorescence from Light-Emitting Electrochemical Cells. *Adv. Funct. Mater.* **2022**, *32*, 2205967.

(797) Karaman, M.; Kumar Gupta, A.; Madayanad Suresh, S.; Matulaitis, T.; Mardegan, L.; Tordera, D.; Bolink, H. J.; Wu, S.; Warriner, S.; Samuel, I. D.; Zysman-Colman, E. Ionic Multiresonant Thermally Activated Delayed Fluorescence Emitters for Light Emitting Electrochemical Cells. *Beilstein J Org Chem* **2022**, *18*, 1311-1321.

(798) Keller, S.; Prescimone, A.; La Placa, M.-G.; Junquera-Hernández, J. M.; Bolink, H. J.; Constable, E. C.; Sessolo, M.; Ortí, E.; Housecroft, C. E. The Shiny Side of Copper: Bringing Copper(I) Light-Emitting Electrochemical Cells Closer to Application. *RSC Adv.* **2020**, *10*, 22631-22644.

(799) Keller, S.; Constable, E. C.; Housecroft, C. E.; Neuburger, M.; Prescimone, A.; Longo, G.; Pertegás, A.; Sessolo, M.; Bolink, H. J. [Cu(Bpy)(P^P)]+ Containing Light-Emitting Electrochemical Cells: Improving Performance through Simple Substitution. *Dalton Trans.* **2014**, *43*, 16593-16596.

(800) Arnosti, N.; Brunner, F.; Susic, I.; Keller, S.; Junquera-Hernández, J. M.; Prescimone, A.; Bolink, H. J.; Sessolo, M.; Ortí, E.; Housecroft, C. E.; Constable, E. C. Remote Modification of Bidentate Phosphane Ligands Controlling the Photonic Properties in Their Complexes: Enhanced Performance of [Cu(Rn-Xantphos)(N^N)][Pf6] in Light-Emitting Electrochemical Cells. *Adv. Optical Mater.* **2020**, *8*, 1901689.

(801) Meyer, M.; Mardegan, L.; Tordera, D.; Prescimone, A.; Sessolo, M.; Bolink, H. J.; Constable, E. C.; Housecroft, C. E. A Counterion Study of a Series of [Cu(P^P)(N^N)][a] Compounds with Bis(Phosphane) and 6-Methyl and 6,6′-Dimethyl-Substituted 2,2′-Bipyridine Ligands for Light-Emitting Electrochemical Cells. *Dalton Trans.* **2021**, *50*, 17920-17934.

(802) Elie, M.; Sguerra, F.; Di Meo, F.; Weber, M. D.; Marion, R.; Grimault, A.; Lohier, J.-F.; Stallivieri, A.; Brosseau, A.; Pansu, R. B.et al. Designing NHC–Copper(I) Dipyridylamine Complexes for Blue Light-Emitting Electrochemical Cells. *ACS Appl. Mater. Interfaces* **2016**, *8*, 14678-14691.
(803) Nakanotani, H.; Higuchi, T.; Furukawa, T.; Masui, K.; Morimoto, K.; Numata, M.; Tanaka, H.; Sagara, Y.; Yasuda, T.; Adachi, C. High-Efficiency Organic Light-Emitting Diodes with Fluorescent Emitters. *Nat. Commun.* **2014**, *5*, 4016.
(804) Ho Lee, I.; Song, W.; Lee, J. Y.; Hwang, S.-H. High Efficiency Blue Fluorescent Organic Light-Emitting Diodes Using a Conventional Blue Fluorescent Emitter. *J. Mater. Chem. C* **2015**, *3*, 8834-8838.
(805) Ahn, D. H.; Jeong, J. H.; Song, J.; Lee, J. Y.; Kwon, J. H. Highly Efficient Deep Blue Fluorescent Organic Light-Emitting Diodes Boosted by Thermally Activated Delayed Fluorescence Sensitization. *ACS Appl. Mater. Interfaces* **2018**, *10*, 10246-10253.
(806) Alam, M. I.; Nagar, M. R.; Nayak, S. R.; Choudhury, A.; Jou, J.-H.; Vaidyanathan, S. Acceptor Interlocked Molecular Design for Solution-Processed Stable Deep-Blue TADF and Hyper Fluorescence Organic Led Enabling High-Efficiency. *Adv. Optical Mater.* **2022**, *10*, 2200376.
(807) Yun, J. H.; Lee, K. H.; Lee, J. Y. Fluorine Substituted Triazine Acceptor Based Thermally Activated Delayed Fluorescent Emitter as an Assistant Dopant of Fluorescent Emitter. *Dyes Pigm.* **2020**, *181*, 108549.
(808) Jang, J. S.; Han, S. H.; Choi, H. W.; Yook, K. S.; Lee, J. Y. Molecular Design of Sensitizer to Suppress Efficiency Loss Mechanism in Hyper-Fluorescent Organic Light-Emitting Diodes. *Org. Electron.* **2018**, *59*, 236-242.
(809) Xie, W.; Peng, X.; Li, M.; Qiu, W.; Li, W.; Gu, Q.; Jiao, Y.; Chen, Z.; Gan, Y.; Liu, K. k.; Su, S.-J. Blocking the Energy Loss of Dexter Energy Transfer in Hyperfluorescence OLEDs Via One-Step Phenyl-Fluorene Substitution of TADF Assistant Host. *Adv. Optical Mater.* **2022**, *10*, 2200665.
(810) Wallwork, N. R.; Mamada, M.; Shukla, A.; McGregor, S. K.; Adachi, C.; Namdas, E. B.; Lo, S.-C. High-Performance Solution-Processed Red Hyperfluorescent OLEDs Based on Cibalackrot. *J. Mater. Chem. C* **2022**, *10*, 4767-4774.
(811) Furukawa, T.; Nakanotani, H.; Inoue, M.; Adachi, C. Dual Enhancement of Electroluminescence Efficiency and Operational Stability by Rapid Upconversion of Triplet Excitons in OLEDs. *Sci. Rep.* **2015**, *5*, 8429.
(812) Zhang, D.; Song, X.; Cai, M.; Duan, L. Blocking Energy-Loss Pathways for Ideal Fluorescent Organic Light-Emitting Diodes with Thermally Activated Delayed Fluorescent Sensitizers. *Adv. Mater.* **2018**, *30*, 1705250.
(813) Song, X.; Zhang, D.; Lu, Y.; Yin, C.; Duan, L. Understanding and Manipulating the Interplay of Wide-Energy-Gap Host and TADF Sensitizer in High-Performance Fluorescence OLEDs. *Adv. Mater.* **2019**, *31*, 1901923.
(814) Kim, J. H.; Lee, K. H.; Lee, J. Y. Design of Thermally Activated Delayed Fluorescent Assistant Dopants to Suppress the Nonradiative Component in Red Fluorescent Organic Light-Emitting Diodes. *Chem. Eur. J.* **2019**, *25*, 9060-9070.
(815) Han, S. H.; Lee, J. Y. Spatial Separation of Sensitizer and Fluorescent Emitter for High Quantum Efficiency in Hyperfluorescent Organic Light-Emitting Diodes. *J. Mater. Chem. C* **2018**, *6*, 1504-1508.
(816) Chen, Y.; Sun, Q.; Dai, Y.; Yang, D.; Qiao, X.; Ma, D. High Efficiency Blue and Color-Stable Hybrid Warm White Organic Light-Emitting Diodes Based on a Thermally Activated Delayed Fluorescent Material as an Assistant Host. *J. Mater. Chem. C* **2020**, *8*, 13777-13785.
(817) Yao, J.; Wang, Z.; Qiao, X.; Yang, D.; Dai, Y.; Sun, Q.; Chen, J.; Yang, C.; Ma, D. High Efficiency and Long Lifetime Fluorescent Organic Light-Emitting Diodes Based on Cascaded Energy Transfer Processes to Efficiently Utilize Triplet Excitons Via Sensitizer. *Org. Electron.* **2020**, *84*, 105824.
(818) Li, D.; Hu, Y.; Liao, L.-S. Triplet Exciton Harvesting by Multi-Process Energy Transfer in Fluorescent Organic Light-Emitting Diodes. *J. Mater. Chem. C* **2019**, *7*, 977-985.

(819) Li, D.; Liao, L.-S. Highly Efficient Deep-Red Organic Light-Emitting Diodes Using Exciplex-Forming Co-Hosts and Thermally Activated Delayed Fluorescence Sensitizers with Extended Lifetime. *J. Mater. Chem. C* **2019**, *7*, 9531-9536.
(820) Jang, J. S.; Han, S. H.; Lee, J. Design Rule of Assistant Dopant for High External Quantum Efficiency in Hyperfluorescence Organic Light-Emitting Diodes. *Adv. Photon. Res.* **2021**, *2*, 2000109.
(821) Chen, D.; Cai, X.; Li, X.-L.; He, Z.; Cai, C.; Chen, D.; Su, S.-J. Efficient Solution-Processed Red All-Fluorescent Organic Light-Emitting Diodes Employing Thermally Activated Delayed Fluorescence Materials as Assistant Hosts: Molecular Design Strategy and Exciton Dynamic Analysis. *J. Mater. Chem. C* **2017**, *5*, 5223-5231.
(822) Wang, Y.-K.; Huang, C.-C.; Kumar, S.; Wu, S.-F.; Yuan, Y.; Aziz Khan, A. K.; Jiang, Z.-Q.; Fung, M.-K.; Liao, L.-S. The Roles of Thermally Activated Delayed Fluorescence Sensitizers for Efficient Red Fluorescent Organic Light-Emitting Diodes with D–a–a Type Emitters. *Mater. Chem. Front.* **2019**, *3*, 161-167.
(823) Tang, X.; Li, Y.; Qu, Y. K.; Peng, C. C.; Khan, A.; Jiang, Z. Q.; Liao, L. S. All-Fluorescence White Organic Light-Emitting Diodes Exceeding 20% EQEs by Rational Manipulation of Singlet and Triplet Excitons. *Adv. Funct. Mater.* **2020**, *30*, 1910633.
(824) Wang, Z.; Li, X. L.; Ma, Z.; Cai, X.; Cai, C.; Su, S. J. Exciton-Adjustable Interlayers for High Efficiency, Low Efficiency Roll-Off, and Lifetime Improved Warm White Organic Light-Emitting Diodes (WOLEDs) Based on a Delayed Fluorescence Assistant Host. *Adv. Funct. Mater.* **2018**, *28*, 1706922.
(825) Liao, X.; Yang, X.; Cheng, J.; Li, Y.; Meng, X.; Li, J.; Pei, Q.; Li, L. Solution-Processed Warm White Organic Light-Emitting Diodes Based on a Blue Thermally Activated Delayed Fluorescence Dendrimer. *Chempluschem* **2018**, *83*, 274-278.
(826) Hu, J.; Hu, S.; Lu, C.; Huang, Y.; Xu, K.; Wang, X. Assistant Dopant System in Red Phosphorescent OLEDs and Its Mechanism Reveal. *J. Lumin.* **2018**, *197*, 187-192.
(827) Nagata, R.; Nakanotani, H.; Adachi, C. Near-Infrared Electrophosphorescence up to 1.1 μm Using a Thermally Activated Delayed Fluorescence Molecule as Triplet Sensitizer. *Adv. Mater.* **2017**, *29*, 1604265.
(828) Shahalizad, A.; Malinge, A.; Hu, L.; Laflamme, G.; Haeberlé, L.; Myers, D. M.; Mao, J.; Skene, W. G.; Kéna-Cohen, S. Efficient Solution-Processed Hyperfluorescent OLEDs with Spectrally Narrow Emission at 840 Nm. *Adv. Funct. Mater.* **2021**, *31*, 2007119.
(829) Brodeur, J.; Hu, L.; Malinge, A.; Eizner, E.; Skene, W. G.; Kéna-Cohen, S. Highly Efficient and Spectrally Narrow near-Infrared Fluorescent OLEDs Using a TADF-Sensitized Cyanine Dye. *Adv. Optical Mater.* **2019**, *7*, 1901144.
(830) Bartkowski, K.; Zimmermann Crocomo, P.; Kochman, M. A.; Kumar, D.; Kubas, A.; Data, P.; Lindner, M. Tandem Rigidification and Π-Extension as a Key Tool for the Development of a Narrow Linewidth Yellow Hyperfluorescent OLED System. *Chem. Sci.* **2022**, *13*, 10119-10128.
(831) Li, F.; Gillett, A. J.; Gu, Q.; Ding, J.; Chen, Z.; Hele, T. J. H.; Myers, W. K.; Friend, R. H.; Evans, E. W. Singlet and Triplet to Doublet Energy Transfer: Improving Organic Light-Emitting Diodes with Radicals. *Nat. Commun.* **2022**, *13*, 2744.
(832) Zhan, L.; Ying, A.; Qi, Y.; Wu, K.; Tang, Y.; Tan, Y.; Zou, Y.; Xie, G.; Gong, S.; Yang, C. Copper(I) Complex as Sensitizer Enables High-Performance Organic Light-Emitting Diodes with Very Low Efficiency Roll-Off. *Adv. Funct. Mater.* **2021**, *31*, 2106345.
(833) Zhou, D.; Wu, S.; Cheng, G.; Che, C.-M. A Gold(III)–TADF Emitter as a Sensitizer for High-Color-Purity and Efficient Deep-Blue Solution-Processed OLEDs. *J. Mater. Chem. C* **2022**, *10*, 4590-4596.
(834) Zhang, D.; Duan, L.; Zhang, D.; Qiu, Y. Towards Ideal Electrophosphorescent Devices with Low Dopant Concentrations: The Key Role of Triplet up-Conversion. *J. Mater. Chem. C* **2014**, *2*, 8983-8989.
(835) Zhang, D.; Duan, L.; Li, Y.; Li, H.; Bin, Z.; Zhang, D.; Qiao, J.; Dong, G.; Wang, L.; Qiu, Y. Towards High Efficiency and Low Roll-Off Orange Electrophosphorescent Devices by Fine Tuning Singlet and Triplet Energies of Bipolar Hosts Based on Indolocarbazole/1, 3, 5-Triazine Hybrids. *Adv. Funct. Mater.* **2014**, *24*, 3551-3561.

(836) Li, Y.; Zhang, D.; Zhang, Y.; Cai, M.; Duan, L. Red Phosphorescent Organic Light-Emitting Diodes Based on a Novel Host Material with Thermally Activated Delayed Fluorescent Properties. *Sci. China Chem.* **2016**, *59*, 684-691.
(837) Zhang, D.; Cai, M.; Zhang, Y.; Zhang, D.; Duan, L. Highly Efficient Simplified Single-Emitting-Layer Hybrid WOLEDs with Low Roll-Off and Good Color Stability through Enhanced Forster Energy Transfer. *ACS Appl. Mater. Interfaces* **2015**, *7*, 28693-28700.
(838) Wei, P.; Zhang, D.; Cai, M.; Song, X.; Wang, Z.; Duan, L. Simplified Single-Emitting-Layer Hybrid White Organic Light-Emitting Diodes with High Efficiency, Low Efficiency Roll-Off, High Color Rendering Index and Superior Color Stability. *Org. Electron.* **2017**, *49*, 242-248.
(839) Wang, Z.; Zhang, H.; Wang, Z.; Zhao, B.; Chen, L.; Li, J.; Wang, H.; Hao, Y.; Li, W. Efficient Management of Excitons in Red and White Organic Light-Emitting Diodes by Employing Blue Thermally Activated Delayed Fluorescent Emitter Based Acridine/Sulfone Derivative as the Host. *Org. Electron.* **2018**, *57*, 311-316.
(840) Xia, Y.; Liu, Z.; Li, J.; Fan, C.; Li, G.; Zhao, B.; Wu, Y.; Wang, H.; Guo, K. TADF Material with Non-Conjugated Rigid Donor for High-Performance Full-Color Phosphorescent OLEDs: Effects of Triplet Harvest and Charge Transport on Efficiency. *Org. Electron.* **2020**, *85*, 105826.
(841) Liu, Y.; Pan, J.; Chen, F.; Gao, K.; Zhu, A.; Wang, R.; Yue, X.; Ban, X. An Effective Thermally Activated Delayed Fluorescence Host Material for Highly Efficient Blue Phosphorescent Organic Light-Emitting Diodes with Low Doping Concentration. *J. Photochem. Photobiol., A* **2020**, *388*, 112178.
(842) Lin, C.-C.; Huang, M.-J.; Chiu, M.-J.; Huang, M.-P.; Chang, C.-C.; Liao, C.-Y.; Chiang, K.-M.; Shiau, Y.-J.; Chou, T.-Y.; Chu, L.-K.et al. Molecular Design of Highly Efficient Thermally Activated Delayed Fluorescence Hosts for Blue Phosphorescent and Fluorescent Organic Light-Emitting Diodes. *Chem. Mater.* **2017**, *29*, 1527-1537.
(843) Wang, H.; Zhao, H.; Zang, C.; Liu, S.; Zhang, L.; Xie, W. Stable and Efficient Phosphorescent Organic Light-Emitting Device Utilizing a Δ-Carboline-Containing Host Displaying Thermally Activated Delayed Fluorescence. *J. Mater. Chem. C* **2020**, *8*, 3800-3806.
(844) Sun, B.; Tong, K.-N.; Chen, X.; He, J.-L.; Liu, H.; Fung, M.-K.; Fan, J. A Universal Thermally Activated Delayed Fluorescent Host with Short Triplet Lifetime for Highly Efficient Phosphorescent OLEDs with Extremely Low Efficiency Roll-Off. *J. Mater. Chem. C* **2021**, *9*, 7706-7712.
(845) Ito, H.; Shimizu, T.; Wada, Y.; Kaji, H.; Fukagawa, H. Comprehensive Study on Operational Lifetime of Organic Light-Emitting Diodes: Effects of Molecular Structure and Energy Transfer. *Jpn. J. Appl. Phys.* **2021**, *60*, 040902.
(846) Jeon, S. K.; Oh, C. S.; Kim, M.; Yook, K. S.; Lee, J. Y. Triplet Exciton Recycling of a Phosphorescent Emitter by an up-Conversion Process Using a Delayed Fluorescence Type Low Triplet Energy Host Material. *J. Mater. Chem. C* **2016**, *4*, 1606-1612.
(847) Qian, S.; Zhang, H.; Lan, J.; Bin, Z. Facile Access to Isocoumarin-Based D-a-D Triad: A Thermally Activated Delayed-Fluorescence Host for Efficient Red Phosphorescent OLEDs. *Org. Electron.* **2020**, *84*, 105792.
(848) Wang, J.; Jiang, C.; Liu, C.; Liu, H.; Yao, C. Highly Efficient Blue, Orange and Red PhOLEDs with Low Roll-Off of Efficiency Using a Carbazole Dendritic Thermally Activated Delayed Fluorescence (TADF) Material as Host. *Mater. Lett.* **2018**, *233*, 149-152.
(849) Wang, Y. K.; Li, S. H.; Wu, S. F.; Huang, C. C.; Kumar, S.; Jiang, Z. Q.; Fung, M. K.; Liao, L. S. Tilted Spiro-Type Thermally Activated Delayed Fluorescence Host for ≈100% Exciton Harvesting in Red Phosphorescent Electronics with Ultralow Doping Ratio. *Adv. Funct. Mater.* **2018**, *28*, 1706228.
(850) Zhang, D.; Duan, L.; Li, C.; Li, Y.; Li, H.; Zhang, D.; Qiu, Y. High-Efficiency Fluorescent Organic Light-Emitting Devices Using Sensitizing Hosts with a Small Singlet–Triplet Exchange Energy. *Adv. Mater.* **2014**, *26*, 5050-5055.
(851) Xue, J.; Liang, Q.; Zhang, Y.; Zhang, R.; Duan, L.; Qiao, J. High-Efficiency near-Infrared Fluorescent Organic Light-Emitting Diodes with Small Efficiency Roll-Off: A Combined Design from Emitters to Devices. *Adv. Funct. Mater.* **2017**, *27*, 1703283.

(852) Wang, S.; Zhang, Y.; Chen, W.; Wei, J.; Liu, Y.; Wang, Y. Achieving High Power Efficiency and Low Roll-Off OLEDs Based on Energy Transfer from Thermally Activated Delayed Excitons to Fluorescent Dopants. *Chem. Commun.* **2015**, *51*, 11972-11975.
(853) Li, M.; Wang, J.; Dai, Y.; Zhang, Y.; Chen, L.; Jin, L.; Tao, Y.; Chen, R.; Huang, W. Evoking Synergetic Effect of Dual Thermally Activated Delayed Fluorescent Hosts for High-Efficiency Sensitized Fluorescent Organic Light-Emitting Diodes. *J. Phys. Chem. C* **2019**, *124*, 1836-1843.
(854) Liu, X.-K.; Chen, Z.; Zheng, C.-J.; Chen, M.; Liu, W.; Zhang, X.-H.; Lee, C.-S. Nearly 100% Triplet Harvesting in Conventional Fluorescent Dopant-Based Organic Light-Emitting Devices through Energy Transfer from Exciplex. *Adv. Mater.* **2015**, *27*, 2025-2030.
(855) Data, P.; Kurowska, A.; Pluczyk, S.; Zassowski, P.; Pander, P.; Jedrysiak, R.; Czwartosz, M.; Otulakowski, L.; Suwinski, J.; Lapkowski, M.; Monkman, A. P. Exciplex Enhancement as a Tool to Increase OLED Device Efficiency. *J. Phys. Chem. C* **2016**, *120*, 2070-2078.
(856) Zhang, L.; Cheah, K. W. Thermally Activated Delayed Fluorescence Host for High Performance Organic Light-Emitting Diodes. *Sci. Rep.* **2018**, *8*, 8832.
(857) Zhang, C.; Lu, Y.; Liu, Z.; Zhang, Y.; Wang, X.; Zhang, D.; Duan, L. A Pi-D and Pi-a Exciplex-Forming Host for High-Efficiency and Long-Lifetime Single-Emissive-Layer Fluorescent White Organic Light-Emitting Diodes. *Adv. Mater.* **2020**, *32*, 2004040.
(858) Aizawa, N.; Shikita, S.; Yasuda, T. Spin-Dependent Exciton Funneling to a Dendritic Fluorophore Mediated by a Thermally Activated Delayed Fluorescence Material as an Exciton-Harvesting Host. *Chem. Mater.* **2017**, *29*, 7014-7022.
(859) Zhang, D.; Zhao, C.; Zhang, Y.; Song, X.; Wei, P.; Cai, M.; Duan, L. Highly Efficient Full-Color Thermally Activated Delayed Fluorescent Organic Light-Emitting Diodes: Extremely Low Efficiency Roll-Off Utilizing a Host with Small Singlet-Triplet Splitting. *ACS Appl. Mater. Interfaces* **2017**, *9*, 4769-4777.
(860) Liu, Z.; Lei, Y.; Fan, C.; Peng, X.; Ji, X.; Jabbour, G. E.; Yang, X. Simple-Structure Organic Light Emitting Diodes: Exploring the Use of Thermally Activated Delayed Fluorescence Host and Guest Materials. *Org. Electron.* **2017**, *41*, 237-244.
(861) Li, S. W.; Yu, C. H.; Ko, C. L.; Chatterjee, T.; Hung, W. Y.; Wong, K. T. Cyanopyrimidine-Carbazole Hybrid Host Materials for High-Efficiency and Low-Efficiency Roll-Off TADF OLEDs. *ACS Appl. Mater. Interfaces* **2018**, *10*, 12930-12936.
(862) Chen, C.-H.; Lin, S.-C.; Lin, B.-Y.; Li, C.-Y.; Kong, Y.-C.; Chen, Y.-S.; Fang, S.-C.; Chiu, C.-H.; Lee, J.-H.; Wong, K.-T.et al. New Bipolar Host Materials for High Power Efficiency Green Thermally Activated Delayed Fluorescence OLEDs. *Chem. Eng. J.* **2022**, *442*, 136292.
(863) Zhou, D.; Liu, D.; Gong, X.; Ma, H.; Qian, G.; Gong, S.; Xie, G.; Zhu, W.; Wang, Y. Solution-Processed Highly Efficient Bluish-Green Thermally Activated Delayed Fluorescence Emitter Bearing an Asymmetric Oxadiazole-Difluoroboron Double Acceptor. *ACS Appl. Mater. Interfaces* **2019**, *11*, 24339-24348.
(864) Hu, J.; Zhang, X.; Zhang, D.; Cao, X.; Jiang, T.; Zhang, X.; Tao, Y. Linkage Modes on Phthaloyl/Triphenylamine Hybrid Compounds: Multi-Functional AIE Luminogens, Non-Doped Emitters and Organic Hosts for Highly Efficient Solution-Processed Delayed Fluorescence OLEDs. *Dyes Pigm.* **2017**, *137*, 480-489.
(865) Ban, X.; Zhu, A.; Zhang, T.; Tong, Z.; Jiang, W.; Sun, Y. Design of Encapsulated Host and Guest for Highly Efficient Blue and Green Thermally Activated Delayed Fluorescence OLEDs Based on Solution-Process. *Chem. Commun.* **2017**, *53*, 11834-11837.
(866) Nakanotani, H.; Furukawa, T.; Hosokai, T.; Hatakeyama, T.; Adachi, C. Light Amplification in Molecules Exhibiting Thermally Activated Delayed Fluorescence. *Adv. Optical Mater.* **2017**, *5*, 1700051.
(867) Khan, A.; Tang, X.; Zhong, C.; Wang, Q.; Yang, S. Y.; Kong, F. C.; Yuan, S.; Sandanayaka, A. S. D.; Adachi, C.; Jiang, Z. Q.; Liao, L. S. Intramolecular-Locked High Efficiency Ultrapure Violet-Blue (Cie-Y <0.046) Thermally Activated Delayed Fluorescence Emitters Exhibiting Amplified Spontaneous Emission. *Adv. Funct. Mater.* **2021**, *31,* 2009488.
(868) Nakanotani, H.; Furukawa, T.; Adachi, C. Light Amplification in an Organic Solid-State Film with the Aid of Triplet-to-Singlet Upconversion. *Adv. Optical Mater.* **2015**, *3*, 1381-1388.

(869) Liu, T.; Li, S.; Wang, W.; Liu, Y.; Du, H.; Guo, Y.; Shi, Q.; Zhang, D.; Zhao, L.; Fan, Q. Reduced Optically Pumped Amplified Spontaneous Emission Threshold of Bubd-1 Thin Films by Thermally Activated Delayed Fluorescent Materials. *J. Lumin.* **2019**, *212*, 76-82.
(870) Shukla, A.; McGregor, S. K.; Wawrzinek, R.; Saggar, S.; Moore, E. G.; Lo, S. C.; Namdas, E. B. Light Amplification and Efficient Electroluminescence from a Solution-Processable Diketopyrrolopyrrole Derivative Via Triplet-to-Singlet Upconversion. *Adv. Funct. Mater.* **2021**, *31*, 2009817.
(871) Huang, H.; Yu, Z.; Zhou, D.; Li, S.; Fu, L.; Wu, Y.; Gu, C.; Liao, Q.; Fu, H. Wavelength-Turnable Organic Microring Laser Arrays from Thermally Activated Delayed Fluorescent Emitters. *ACS Photonics* **2019**, *6*, 3208-3214.
(872) Zhou, Z.; Qiao, C.; Wang, K.; Wang, L.; Liang, J.; Peng, Q.; Wei, Z.; Dong, H.; Zhang, C.; Shuai, Z.et al. Experimentally Observed Reverse Intersystem Crossing-Boosted Lasing. *Angew. Chem., Int. Ed.* **2020**, *59*, 21677-21682.
(873) Li, Y.; Wang, K.; Liao, Q.; Fu, L.; Gu, C.; Yu, Z.; Fu, H. Tunable Triplet-Mediated Multicolor Lasing from Nondoped Organic TADF Microcrystals. *Nano Lett.* **2021**, *21*, 3287-3294.
(874) Li, S.; Chen, J.; Wei, Y.; De, J.; Geng, H.; Liao, Q.; Chen, R.; Fu, H. An Organic Laser Based on Thermally Activated Delayed Fluorescence with Aggregation-Induced Emission and Local Excited State Characteristics. *Angew. Chem., Int. Ed.* **2022**, *61*, e202209211.
(875) Kalyanasundaram, K. Photophysics, Photochemistry and Solar Energy Conversion with Tris(Bipyridyl)Ruthenium(Ii) and Its Analogues. *Coordination Chemistry Reviews* **1982**, *46*, 159-244.
(876) Juris, A.; Balzani, V.; Belser, P.; Zelewsky, A. v. Characterization of the Excited State Properties of Some New Photosensitizers of the Ruthenium (Polypyridine) Family. *Helv. Chim. Acta* **1981**, *64*, 2175-2183.
(877) Balzani, V.; Campagna, S. *Photochemistry and Photophysics of Coordination Compounds Ii*; Springer, **2007**.
(878) St-Pierre, G.; Ladouceur, S.; Fortin, D.; Zysman-Colman, E. Fraternal Twin Iridium Hemicage Chelates. *Dalton Trans.* **2011**, *40*, 11726-11731.
(879) Koike, T.; Akita, M. Visible-Light Radical Reaction Designed by Ru- and Ir-Based Photoredox Catalysis. *Inorg. Chem. Front.* **2014**, *1*, 562-576.
(880) Lowry, M. S.; Goldsmith, J. I.; Slinker, J. D.; Rohl, R.; Pascal Jr., R. A.; Malliaras, G. G.; Bernhard, S. Single-Layer Electroluminescent Devices and Photoinduced Hydrogen Production from an Ionic Iridium(Iii) Complex. *Chem. Mater.* **2005**, *17*, 5712-5719.
(881) Singh, A.; Teegardin, K.; Kelly, M.; Prasad, K. S.; Krishnan, S.; Weaver, J. D. Facile Synthesis and Complete Characterization of Homoleptic and Heteroleptic Cyclometalated Iridium(Iii) Complexes for Photocatalysis. *J. Organomet. Chem.* **2015**, *776*, 51-59.
(882) Day, J. I.; Teegardin, K.; Weaver, J.; Chan, J. Advances in Photocatalysis: A Microreview of Visible Light Mediated Ruthenium and Iridium Catalyzed Organic Transformations. *Org. Process Res. Dev.* **2016**, *20*, 1156-1163.
(883) Hari, D. P.; Konig, B. Synthetic Applications of Eosin Y in Photoredox Catalysis. *Chem. Commun.* **2014**, *50*, 6688-6699.
(884) Seret, A.; Gandin, E.; Van De Vorst, A. Flash Photolysis of Eosin in Aqueous Micellar Dispersions. *Chem. Phys. Lett.* **1987**, *135*, 427-431.
(885) Arbeloa, E. M.; Previtali, C. M.; Bertolotti, S. G. A Comparative Study on the Photophysics and Photochemistry of Xanthene Dyes in the Presence of Polyamidoamine (Pamam) Dendrimers. *ChemPhysChem* **2018**, *19*, 934-942.
(886) Penzkofer, A.; Beidoun, A.; Daiber, M. Intersystem-Crossing and Excited-State Absorption in Eosin Y Solutions Determined by Picosecond Double Pulse Transient Absorption Measurements. *J. Lumin.* **1992**, *51*, 297-314.
(887) Shen, T.; Zhao, Z.-G.; Yu, Q.; Xu, H.-J. Photosensitized Reduction of Benzil by Heteroatom-Containing Anthracene Dyes. *J. Photochem. Photobiol., A* **1989**, *47*, 203-212.
(888) Zhang, X.-F. Z.; Zhang, I.; Liu, L. Photophysics of Halogenated Fluoresceins: Involvement of Both Intramolecular Electron Transfer and Heavy Atom Effect in the Deactivation of Excited States. *Photochem. Photobiol.* **2010**, *86*, 492-498.

(889) Luo, J.; Zhang, J. Donor–Acceptor Fluorophores for Visible-Light-Promoted Organic Synthesis: Photoredox/Ni Dual Catalytic C(Sp3)–C(Sp2) Cross-Coupling. *ACS Catal.* **2016**, *6*, 873-877.
(890) Ishimatsu, R.; Matsunami, S.; Shizu, K.; Adachi, C.; Nakano, K.; Imato, T. Solvent Effect on Thermally Activated Delayed Fluorescence by 1,2,3,5-Tetrakis(Carbazol-9-Yl)-4,6-Dicyanobenzene. *J. Phys. Chem. A* **2013**, *117*, 5607-5612.
(891) Ishimatsu, R.; Matsunami, S.; Kasahara, T.; Mizuno, J.; Edura, T.; Adachi, C.; Nakano, K.; Imato, T. Electrogenerated Chemiluminescence of Donor-Acceptor Molecules with Thermally Activated Delayed Fluorescence. *Angew. Chem., Int. Ed.* **2014**, *53*, 6993-6996.
(892) Speckmeier, E.; Fischer, T. G.; Zeitler, K. A Toolbox Approach to Construct Broadly Applicable Metal-Free Catalysts for Photoredox Chemistry: Deliberate Tuning of Redox Potentials and Importance of Halogens in Donor-Acceptor Cyanoarenes. *J. Am. Chem. Soc.* **2018**, *140*, 15353-15365.
